# Sandcastle: software for revealing latent information in multiple experimental ChIP-chip datasets via a novel normalisation procedure

**DOI:** 10.1038/srep13395

**Published:** 2015-08-26

**Authors:** Mark Bennett, Katie Ellen Evans, Shirong Yu, Yumin Teng, Richard M. Webster, James Powell, Raymond Waters, Simon H. Reed

**Affiliations:** 1 Cancer and Genetics Building, Cardiff University, School of Medicine, Heath Park, Cardiff, CF14 4XN, UK

## Abstract

ChIP-chip is a microarray based technology for determining the genomic locations of chromatin bound factors of interest, such as proteins. Standard ChIP-chip analyses employ peak detection methodologies to generate lists of genomic binding sites. No previously published method exists to enable comparative analyses of enrichment levels derived from datasets examining different experimental conditions. This restricts the use of the technology to binary comparisons of presence or absence of features between datasets. Here we present the R package Sandcastle — Software for the Analysis and Normalisation of Data from ChIP-chip AssayS of Two or more Linked Experiments — which allows for comparative analyses of data from multiple experiments by normalising all datasets to a common background. Relative changes in binding levels between experimental datasets can thus be determined, enabling the extraction of latent information from ChIP-chip experiments. Novel enrichment detection and peak calling algorithms are also presented, with a range of graphical tools, which facilitate these analyses. The software and documentation are available for download from http://reedlab.cardiff.ac.uk/sandcastle.

Chromatin immunoprecipitation (ChIP) on microarray chips (ChIP-chip) is a technology that was originally developed to identify binding sites of chromatin-binding proteins throughout whole genomes^1^. It has since been adapted to analyse a number of other factors, including epigenetic modifications^2^,^3^, nucleosome positioning^4^, and DNA damage^5^. The procedure has been described and discussed elsewhere^6^ and is outlined in Fig. 1. Briefly, cellular chromatin (Fig. 1a) is extracted and fragmented. Fragments bound to the protein or other factor of interest are extracted by immunoprecipitation (IP; Fig. 1b), and DNA purified from these fragments. This DNA, along with total input DNA, is amplified, differentially fluorescently labelled (Fig. 1c), and applied to a microarray slide (Fig. 1d) containing discrete DNA probes covering a genome of interest, or section thereof. The slide is optically scanned and the fluorescence intensities are converted to numerical values (Fig. 1e), which are analysed to determine the genomic locations at which the factor of interest is present.

Prior to Sandcastle, common analysis procedures of ChIP-chip datasets used a peak detection algorithm to determine binding sites^7^, reducing a dataset to a list of genomic locations at which the factor of interest is deemed to be present. When datasets are generated from multiple different experimental conditions, where key parameters have been changed, analyses are limited to comparing these lists between different datasets to determine whether or not the factor is present at the same locations^8^. These procedures do not permit detection of changes in the level of binding at any location where the presence of the factor has not changed, but the level of binding has. The results of such an analysis would suggest that there has been no change between the experimental conditions, because binding is present at the same site in both, when in fact there has, because the level of that binding is different, which may represent an important biological event. There are methods to analyse multiple ChIP-chip datasets together^9^,^10^,^11^, and differences in patterns of data from different experimental conditions have previously been examined, such as histone positions^3^, and DNA methylation^12^,^13^, but not in ways that allow comparative analyses of relative binding levels at the same genomic locations. Other software packages for the analysis of ChIP-chip data^14^,^15^,^16^ include a range of facilities for data processing, but are still only able to analyse datasets from one single experimental condition at a time.

ChIP-chip datasets are replete with information beyond the binary ‘presence’ or ‘absence’ of the factor of interest at different genomic locations, meaning there is the potential for more advanced analyses than only peak detection to be carried out. Fluorescent signal intensities from the microarrays are related to the DNA amounts hybridised, thereby representing binding levels of the factor of interest at each genomic location. The Sandcastle normalisation procedure presented here is intended to allow the extraction of this information to compare relative binding levels between datasets derived from different experimental conditions. This was not previously possible because there existed no suitable normalisation procedure. Normalisation, in the context of microarray data analysis, is the processing of datasets to reduce inherent technical variations that exist between them, whilst capturing any biological variations, allowing these to be analysed free from the confounding effects of technical variation. The need for data normalisation is well understood in the field of gene expression microarray analysis^17^, where the nature of investigations requires comparisons to be made between expression levels from different experimental conditions. Several normalisation methods have been developed for this purpose. However, these methods are not necessarily suited to ChIP-chip data^18^ and may have the effect of removing many genuine biological differences^19^. Cross comparison of ChIP-chip experiments has previously been highlighted as a significant problem that needs to be addressed^9^.

To address these problems we have developed a novel normalisation procedure for ChIP-chip data, which allows any number of datasets from linked experiments to be normalised in a way that allows relative comparisons to be made between them, allowing additional information to be extracted. Consequently, analyses can be taken beyond simple binding site identifications, to include relative comparisons across different datasets. This allows a wealth of latent information to be retrieved from datasets, enabling more detailed biological conclusions to be drawn. This method has been written into an R (R Development Core Team. *R: A Language and Environment for Statistical Computing*, R Foundation for Statistical Computing, Vienna, 2011) package named Sandcastle (**S**oftware for the **A**nalysis and **N**ormalisation of **D**ata from **C**hIP-chip **A**ssay**S** of **T**wo or more **L**inked **E**xperiments), together with additional tools for ChIP-chip analysis, including an enriched region detection algorithm, a peak detection procedure and methods to display data, to facilitate the discovery of new biological information.

We show how the normalisation works, validate the results by independent methods, and demonstrate how the procedure enhances the analysis of ChIP-chip data.

## Sandcastle

The tools described in this paper are presented as the package Sandcastle, written in the R statistical programming language. The package contains a novel normalisation procedure, an enrichment detection procedure, and multiple graphical tools. The novel normalisation procedure allows for relative comparisons of enrichment levels to be made between datasets from different experimental conditions, which until now has not been possible. The enrichment detection procedure allows regions of enrichment to be identified from datasets. The graphical tools allow the display of the various results that can be generated in Sandcastle.

The software, full documentation, example plots and an example work flow are available at http://reedlab.cardiff.ac.uk/sandcastle.

### Data

ChIP-chip data files can be loaded into Sandcastle from two formats. Data from Agilent microarrays can be loaded directly from files created with Agilent’s Feature Extraction software. Data from other sources can be loaded from tab-delimited files of the format described in the Sandcastle documentation. Data can also be written to tab-delimited files from Sandcastle, for loading into other software or transferring to different hardware, for example. Data are loaded as arrayData objects, which contain log_2_ IP:input ratios along with other essential information such as genome coordinates and probe annotations.

### Normalisation

**Figure 2:**
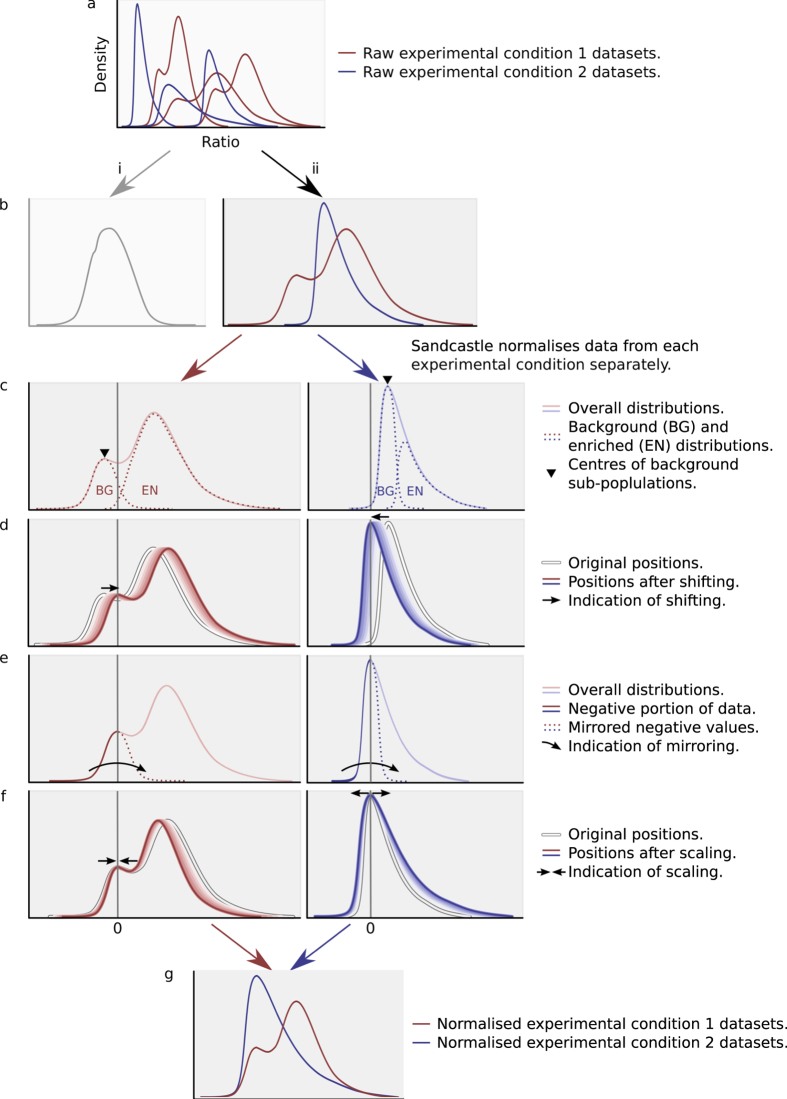
Representation of the normalisation procedure. (**a**) Raw density profiles of datasets from two experimental conditions (red and blue), each with three replicates. Differences in the shapes of the profiles indicate experimentally induced biologically relevant changes, but these cannot be compared in their raw state. (**b**) Quantile normalising all datasets together (**i**) removes much of the experimentally induced, biologically relevant differences between them. This is not desirable, as these differences cannot then be investigated. Sandcastle quantile normalises the datasets from each experimental condition separately, to maintain these biological differences. Quantile normalisation makes each of the datasets follow the same distribution, meaning all density profiles from each experimental condition overlap each other (**ii**). This reduces intra-condition – but not inter-condition – technical variations. (**c**) Each dataset consists of two overlapping sub-populations (dashed lines), background (BG) and enriched (EN). These cannot be fully discerned in the data and only the overall population (solid lines) is known. Sandcastle performs inter-condition normalisation based on estimated background sub-populations. This requires the central (modal) point of the background sub-populations to be identifiable (marked with triangles). If this central point cannot be discerned (for example, if the background sub-population is too small) then the Sandcastle normalisation cannot be applied. (**d**) Data are first shifted to centre the modal point of the estimated background sub-population on zero (indicated by arrows). (**e**) To estimate the properties of the whole background sub-population all negative values (the left-hand side of the estimated background sub-population following the shift step) are mirrored into the positive (indicated by arrow; dashed lines show mirrored data). This allows the standard deviation of the estimated background sub-population to be calculated. (**f**) Data are scaled to the make the calculated standard deviation of the estimated background sub-population 1 (indicated by arrows). (**g**) The resulting fully normalised datasets have estimated background sub-populations with the same mean (0) and standard deviation (1). Comparisons of data between conditions can now be made relative to this common background. For clarity axis labels are only shown in (**a**) - all other *x*- and *y*-axes are ratio and density values respectively. Vertical grey lines indicate 0, which are only labelled in (**f**).

The Sandcastle normalisation process is divided into four stages, detailed in the following sections. Figure 2 shows the process with representations of six datasets from two experimental conditions (red and blue density profiles), starting with the data in their raw state, where biologically relevant experimentally induced changes, as well as technical variations, are evident in the different shapes of the profiles (Fig. 2A). The procedure is only applicable to data where the modal point of the background sub-population is discernible in the density profile of the dataset, as detailed below. A function is included in Sandcastle to assist in determining whether or not the normalisation procedure has worked on a set of data, which is fully described in the Sandcastle documentation.

#### Stage 1: pre-processing

Superfluous data, such as from probes present on the microarray but corresponding to sites deleted from the genome being examined, are removed, to prevent their confounding influence. For example, in the case of the *Saccharomyces cerevisiae* laboratory strain BY4742 used in the datasets analysed here, four genes are deleted. There are probes on the Agilent microarrays used that correspond to these regions, but because there is no corresponding genomic material they produce only random values, many at the extremes of the data distribution. Removing the values of these probes means they cannot be accidentally identified as biologically relevant in any downstream analyses. For any probes with missing values in a dataset, for example as a result of attempting to take the logarithm of a negative starting value, the values for those same probes are removed from all other datasets. This means across all datasets each probe has a full complement of values. A large number of such missing values may indicate problems with one or more datasets and so the number of probes removed is displayed in R during the normalisation procedure. This step should only remove a small number of probes and leave the datasets very close to their initial, raw state.

#### Stage 2: quantile normalisation

Quantile normalisation^20^ aims to make the distributions of all datasets the same. Applying quantile normalisation to ChIP-chip datasets from more than one experimental condition will therefore remove much of any biologically relevant differences between datasets induced by the change in experimental conditions (Fig. 2Bi). This is therefore not a viable option for the intra-condition normalisation of ChIP-chip data where changes between the conditions are to be examined. The Sandcastle normalisation procedure overcomes this by performing intra- and inter-condition normalisation separately (Fig. 2Bii). The intra-condition normalisation applies the standard quantile normalisation procedure, using the method implemented in the R preprocessCore package (version 1.30.0), to groups of datasets from each experimental condition. This results in the reduction of technical variations between datasets from the same experimental condition, but has no influence on technical variations between datasets from different experimental conditions. This is the function of the next stages of the Sandcastle normalisation procedure.

#### Background theory to the Sandcastle normalisation

Sandcastle achieves inter-condition normalisation by mimicking the quantile normalisation procedure on a subset of each dataset. ChIP-chip datasets form skewed or bi-modal distributions^6^, represented in the density profiles in Fig. 2A, which in their raw states have incomparable, essentially arbitrary values, and as such cannot be reliably compared. The datasets are made up of two overlapping distributions: ‘background’ signals (equal ratios of IP and input), representing background noise inherent at sites with no enrichment, and ‘enriched’ signals (higher levels of IP than input), representing sites of enrichment for the feature of interest (Fig. 2C). The background sub-population, which consists of random noise, will approximate a normal distribution (ND), which has been pointed out previously^21^. The Sandcastle normalisation procedure works to make the distributions of all of the background sub-populations, from all of the different experimental conditions being normalised, follow the same statistical properties, thereby acting as an internal constant between the datasets, against which relative changes in the enriched sub-populations can be identified between the different conditions.

The theory behind this is as follows. If multiple ChIP-chip experiments were carried out where no material was enriched, the distributions of all datasets would approximate NDs. As there would be no biological differences between the datasets they could be quantile normalised as one group, the result of which would be each dataset following the same ND. An alternative approach, and the principle behind the novel stages of the Sandcastle normalisation method presented here, would be to transform each of the datasets to a common ND, by adjusting them to the same mode and standard deviation (SD) by ‘shifting’ and ‘scaling’ them. This would make the datasets comparable, in the same way that they would be if quantile normalised. In Sandcastle this theory is applied to the background sub-populations of ChIP-chip datasets, which are transformed so that they follow the same distribution, and thus become comparable between the datasets from different experimental conditions. The enriched sub-populations are transformed simultaneously, meaning that these portions of the datasets also become comparable between conditions. This procedure is detailed in the following sections.

As the background and enriched sub-populations are overlapping in a dataset, neither can be fully distinguished (Fig. 2C). The Sandcastle normalisation procedure therefore uses an estimate of the background sub-populations’ properties. A kernel density estimation (KDE; generated with the R density function) of the dataset is used to estimate the point of the peak of the background sub-population as a pseudo-mode (marked with triangles in Fig. 2C). Sandcastle takes this to be the left-most maxima of the KDE. This relies on the centre of the background sub-population being discernible in the overall population. If this point cannot be identified, for example if the background sub-population is too small, then the Sandcastle normalisation procedure cannot be applied. This is described in detail in the Sandcastle documentation. In our experience the large majority of datasets do have a discernible background sub-population modal point and are therefore suitable for Sandcastle normalisation.

#### Stage 3: shifting

The first step in estimating the background sub-population is to make the identified modal-points of the background sub-populations lie on the same point. This is achieved by ‘shifting’ each dataset, by the subtraction of a number *m* to every value of the dataset, where *m* is the *x*-axis value of the modal point of the background sub-population. This results in the centre of each estimated background sub-population lying on zero (Fig. 2D).

This gives each background sub-population the same central point (that is, mean) but does not bring them to the same properties as they may have different spreads (that is, SDs). To calculate the SD of a set of data requires all values to be known. As this is not the case with ChIP-chip data, the total background sub-population has to be estimated. This is achieved by ‘mirroring’ all negative values (all values to the left of the centre of the shifted background sub-population) into the positive (represented in Fig. 2E) to create a symmetrical dataset approximating that of the true background sub-population. From this a SD can be calculated, which represents an estimate of the SD of the true background sub-population.

#### Stage 4: scaling

In order to make the estimated background sub-populations follow the same properties, datasets are ‘scaled’ by the multiplication of a factor *s* to every value of each dataset (Fig. 2F), where *s* is the reciprocal of the SD of the estimated background sub-population. This results in the estimated background sub-population having a SD of 1. This transforms the background sub-populations to the standard normal distribution (SND). In this way every dataset contains a theoretically consistent background sub-population, acting as a reference standard between datasets, against which relative changes in the enriched sub-populations can be measured (Fig. 2G).

### Enrichment detection

Following Sandcastle normalisation datasets consist of a SND sub-population, containing background probes, and a second sub-population of higher values, containing enriched probes. Any enriched probes represent the presence of the IPed factor of interest and so need to be identified in order to investigate the factor. The two sub-populations may partially overlap in datasets and so a method of separating them is required. The Sandcastle enrichment detection method (EDM) achieves this by using the defined properties of the SND to identify probes which are statistically likely to be from the SND, and therefore background, or not, and therefore enriched. The procedure works under the following assumptions.

- An IPed factor will show enrichment at the same genomic location(s) across all replicate datasets.
- An IPed factor will create a region of enrichment at least as large as the average chromatin shear size.

The first assumption accounts for the fact that spurious peaks may occur in datasets for reasons other than enrichment of the factor of interest, whereas true enrichment should always occur at the same sites. Even averaging replicate datasets may retain such spurious peaks in the final data. Therefore Sandcastle analyses all replicate datasets, which allows for spurious peaks in individual datasets to be eliminated while still identifying true enrichment appearing in all datasets.

The second assumption accounts for an advantage of the high resolution of ChIP-chip, which is that sonicated DNA fragments applied to the microarray have the potential to bind to more than one probe. This means that enrichment information may be ‘shared’ between multiple adjacent probes and that sites of enrichment will result in all such related probes showing some level of enrichment, not just the probe closest to the enriched site. This phenomena is used to identify enrichment by analysing multiple adjacent probes within a ‘window’, the size of which should be set to the average sonicated chromatin fragment size.

**Figure 3:**
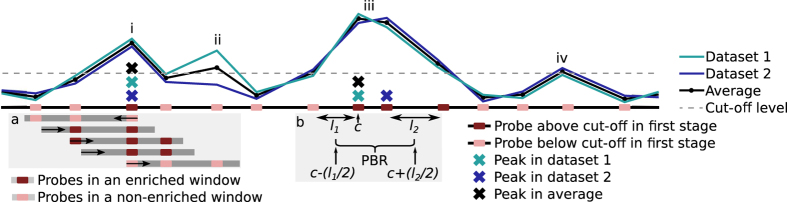
Enrichment and peak detection processes. Two replicate datasets (coloured lines) and their average (black line) are represented along with the probe positions (coloured boxes). The cut-off value calculated for the particular dataset is shown (dashed line). All probes with all values above this cut-off are identified in the first stage of the enrichment detection procedure (highlighted probes). Windows around these probes are analysed (demonstrated with grey boxes for one probe in box (**a**)) to determine which, if any, windows contain probes deemed to be enriched over the whole window region. The first window extends upstream from the probe being analysed (as indicated by the arrow). The next window extends upwards from the furthest probe in this window, but not including it (as indicated by the arrow), and this process is repeated for all probes until the initial identified probe is reached. In this way all possible windows are identified for analysis. If the initial probe is found to be in any enriched window it is returned as an enriched probe by the software, whereas if it is not found to be in any enriched window it is not. If peak detection is required, averaged data are used to identify all maxima within the enriched probes (black crosses), each of which is returned as one peak. Maxima within individual datasets are also identified (coloured crosses) which are used to calculate potential binding regions of each peak (PBRs; demonstrated for one peak in box (**b**)). The PBR represents the region most likely to contain the binding site of the factor of interest and is defined as half the distance from the maxima to the next probe, up- and downstream of the maxima, unless this is distance is greater than the average chromatin shear size, in which case the distance is set to the average chromatin shear size. Peaks where all maxima fall at the same probe (as ‘i’) will therefore have narrower PBRs than those where they fall at adjacent probes (as ‘iii’). If all probe values are not above the cut-off (as ‘ii’ and ‘iv’) they are not identified as being enriched.

Sandcastle requires probes meet both of these assumptions for them to be called as enriched. The first assumption requires the analysis of replicate values from each probe, which determines any probes over a defined cut-off value (described in the following sections) at the same locations across all replicate datasets (represented in Fig. 3, referred to here as ‘seed’ probes). The second assumption requires the analysis of windows around these seed probes to determine which are within genome sections where all probes are above a defined cut-off value (described in the following sections) across a region at least as large as the average chromatin shear size. For each seed probe every possible window, that is, every possible combination of probes within a window containing the seed probe, is calculated by Sandcastle for analysis. This is achieved by first identifying the furthest probe upstream from the seed probe that can fall within the same window (represented in Fig. 3 box ‘a’, with the window with the arrow pointing to the left of the seed probe at peak ‘i’). All probes between and including this probe and the seed probe represent the first window for analysis. Subsequent windows are created from each of the probes present in this first window, beginning at their start coordinate, thus including them, and their end coordinate, thus not including them, extending downstream towards the seed probe, and stopping when this seed probe becomes the first probe in the window (represented in Fig. 3 box ‘a’, with the four windows with arrows pointing to the right). This means that, unlike a sliding window approach, no potential probe combinations in a window can be missed. This process is repeated for each of the seed probes identified in the first enrichment detection stage.

Probes that meet both of these criteria are called as enriched, the result of which is a list showing whether or not each probe in the dataset is enriched (TRUE) or not (FALSE). This format of results is useful for factors which exhibit extended regions of enrichment, such as histone modifications. An optional additional peak detection procedure may then be run, which aims to identify peaks within these identified enriched probes. This is useful for factors which exhibit distinct, localised binding sites.

To determine which probes meet the two assumptions of the procedure requires the analysis of groups of one or more probes at a time, enrichment being determined by comparison of the probe values to pre-calculated cut-offs. These cut-offs are calculated using the known statistical properties of the SND, and define the point over which a given set of values are considered to be statistically unlikely to be from the SND. Probes with values over the cut-off value are considered statistically unlikely to be from the background distribution.

Using cut-off values in this way is similar to calculating the probability that a set of values are from the SND and comparing this to a predefined significance level (for example, 5% or 1% are commonly used). An advantage of using cut-offs is that they can be computed for a given set of data in advance, and so a probability value does not need to be calculated for every set of values being tested. The Sandcastle cut-offs are calculated at a level that takes into account the familywise error rate, meaning that the cut-offs are set at a level that seeks to find no false positive results. Accounting for this at the start of the procedure means that an additional multiple testing correction is not required on the final results.

The familywise error rate is accounted for in the cut-off values by the setting of a user defined value of the number of false positive results to ‘find’, described as follows. If calculating the probability that a given set of values are from the SND, all results with probabilities less than a threshold defined by the significance level of the test would be considered to contain probes from the enriched sub-population. This approach can result in a number of false positives (Type I errors), the expected number of which can be calculated as:


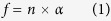


where *f* is the number of false positives, *n* is the size of the dataset and *α* is the significance level. For example, at the 5% significance level, 2,200 false positive probes would be expected in a dataset of 44,000 probes (the approximate size of the datasets analysed here) by chance alone. Because of this generation of false positive results this type of approach requires the application of a multiple testing correction^22^, which aims to remove these false positive results while maintaining any true positives.

The Sandcastle EDM takes into account at the outset the familywise error rate (the likelihood of finding false positive results in the dataset being analysed), and adjusts the significance level accordingly, in an attempt to find no false positives. This is achieved as follows. The equation (1) above can be rearranged to find the significance level associated with a particular number of false positives:


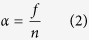


Thus if *f* is defined by the user (via the FP argument in the Sandcastle enrichmentDetection function), *α* can be calculated for a dataset of size *n*. By defining *f* as a value less than one, that is, seeking to ‘find’ less than one false positive results from a dataset, the corresponding significance level can be calculated. For example, defining *f* as 0.9 requires the significance level of 0.00205% in a dataset of 44,000 probes. These are the significance levels that would result in 0.9 (that is, on average less than one) false positive results from the dataset.

These significance levels are used to calculate cut-off values using the qnorm function of R, which takes a probability value and returns the corresponding z-score of the SND. For example, for the dataset of 44,000 probes the cut-off value would be 4.102284. Any probe above this cut-off value in this dataset would meet the first assumption of the EDM, in the same way their calculated probability values would define them based on the calculated significance level. This means that any values over the cut-off will, by definition, have statistically significant probability values and so can be defined as not being from the SND.

The Sandcastle EDM is designed to analyse multiple probes from replicate datasets and within windows at the same time, which means lower cut-off values can be used. In the same way that independent probability values can be multiplied together to give an overall probability value lower than the individual values, the cut-off values are reduced with increasing numbers of probes, which maintains the same overall probability value. This means that if datasets were analysed individually probes may be statistically considered to be from the background sub-population, but when analysed together the lower probability may mean they are statistically considered to be from the enriched sub-population. The probability *p* for cut-off calculations is calculated as follows:


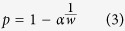


where *α* is the significance level calculated in Equation 2 and *w* is the number of probes in the window. This gives a probability value that, when raised to the power of the number of probes *w* being analysed, gives the original starting probability and thus maintains the starting significance level. The value *p* is used to determine the associated z-score in R via the qnorm function, which is used as the cut-off value.

As shown above, with a dataset of 44,000 probes, one probe has a cut-off value of 4.102284 with an *f* value defined at 0.9. The probability of this occurring in the SND by chance is 2.045455 × 10^−5^. Two probes from the same dataset would have a calculated cut-off value of 2.610336. Individually a probe of this value has a probability of occurring by chance of 4.522667 × 10^−3^. The probability of two such probes occurring together is 4.522667 × 10^−3^ × 4.522667 × 10^−3^ = 2.045455 × 10^−5^, that is, the cut-off value for a group of two probes corresponds to the same significance level as the group containing one probe. This trend continues for larger numbers of probes, with the lower cut-off values meaning that smaller enriched values can be detected with more replicate datasets. This means the ability to detect the same sized peaks is less when analysing individual datasets compared to multiple datasets, but when ChIP-chip experiments are carried out in replicate this will not present a problem. The method uses a single cut-off per group of probes, and all values must be over that cut-off in order to be called as enriched. One single value below the cut-off will prevent enrichment being called and so consistent data between replicate datasets is required for the method to function optimally.

These calculations give cut-offs based on the assumption of independence between all values. This will be the case between separate, biological replicate datasets, as when investigating the first assumption of the EDM. This tests the null hypothesis that all probe values being analysed are from the SND. When one or more probe values are below the cut-off this hypothesis is accepted and probes are not labelled as being enriched. Alternatively, when all probe values are over the cut-off value the alternative hypothesis of all probe values being from an enriched sub-population can be accepted. Investigating the second assumption of the EDM requires the analysis of adjacent probes from the same dataset, where the assumption of independence between all values being tested may not hold. Depending on the nature of the dataset, there may be some level of relationship between adjacent probes even from the background SND, which may give rise to false positives. This is why the second test is used only to validate the results of the first assumption, not to directly identify any new regions of enrichment. At least one of the probes within the windows being tested in this situation is known to be enriched, based on the results of investigating the first assumption of the EDM. The second assumption of the EDM states that adjacent probes should also show a level of enrichment, thereby invalidating the assumption of independence between probes. Therefore the results of the tests around these sites are not used to directly identify enrichment based on the same hypothesis stated above, for investigating the first assumption. Instead this tests the null hypothesis that probe values being analysed are not from the SND. If all probe values are above the cut-off this hypothesis is accepted and no changes to the statuses are made. If any of the probe values are below the cut-off the alternative hypothesis of all probes being from the SND is accepted and all probes are labelled as not being enriched. A probe found to be enriched based on the first assumption needs to be found to be within any one enriched window in order for it to maintain its enriched status, regardless of any other windows that may find it not to be enriched.

### Peak Detection

The EDM detailed above is useful for datasets with extended regions of enrichment, such as epigenetic modifications, where the feature of interest may extend over several hundreds or thousands of bases. For datasets where the feature of interest is located more precisely, such as a protein that binds to distinct loci, these regions of enrichment may be less informative. For this situation Sandcastle has an additional peak detection procedure, to identify peak centres within the detected enriched regions. The procedure for this is detailed below.

Probes identified as being from the enriched sub-population in the EDM are analysed to find maxima in the averaged data from all replicates. A maximum is any probe where the probes immediately either side have lower values, except for probes at the starts and ends of regions, where the probe on the one available side has a lower value. Using the averaged data in this way ensures that identified maxima are present in the majority or all of the individual datasets, and that spurious peaks in individual datasets will not influence the result.

A score is calculated for each of the maxima. The score shows the proportion of individual datasets that have a maxima at the same site as that identified from the averaged data. For example, a score of 1 indicates that all replicate datasets have a peak at the site found from the averaged data (as in Fig. 3 peak ‘i’), and a score of 0.5 indicates that half of the replicate datasets have a peak at the site found from the averaged data (as in Fig. 3 peak ‘iii’). A potential binding region (PBR) is also calculated, which provides an estimate of the most likely genome region containing the true binding site of the factor of interest, as shown in Fig. 3 box ‘b’. For peaks with a score of 1 this is calculated up- and downstream from the peak probe as the smaller of the average chromatin shear size or half the distance to the adjacent probes. For peaks with a score of less than one the same distances are used, but taken from maxima in the individual datasets (as in Fig. 3 box ‘b’, where the PBR is calculated as being from halfway along length *l*_1_ to halfway along length *l*_2_, where those lengths represent the distance between the probes at peak sites to their next probe up-/downstream). Thus PBRs are wider for peaks with lower scores, representing the fact that the greater variability between datasets means less certainty in estimating the binding site.

### Genome annotations

The biomaRt package^23^ is used to download genome annotation data for the organism being investigated. This information can be added to genome plots, to show ORF positions, and incorporated into analyses of, for example, binding site locations.

### Graphics

A selection of graphical outputs can be created from arrayData objects including genomePlot, which plots data along the genome, which can be incorporated with genome annotation data, profilePlot, which overlays multiple sections of data (from genic, intergenic, peak or user defined regions) along with trend lines, and positionsPlot, which combines genome annotation data with the results of the peak detection procedure. Examples of these plots are shown on the Sandcastle website along with a full demonstration script.

### Dataset simulation

A method to predict ChIP-chip binding profiles is included, which can be used to simulate the results of assays where the expected binding site locations are known. This has been used to generate profiles of DNA damage created with UV radiation^5^ to validate the result of this procedure.

## Results

### Validation of the Sandcastle Normalisation Procedure

**Figure 4:**
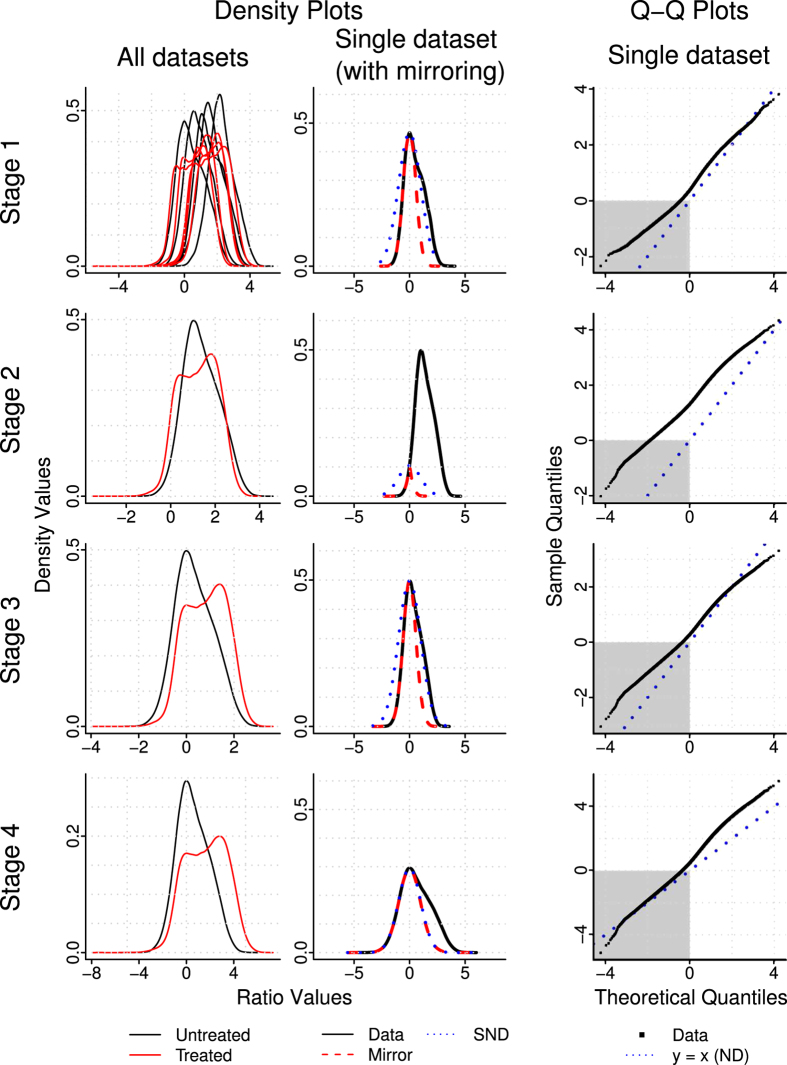
Examples of how the normalisation procedure affects real ChIP-chip datasets. Data following each stage of the procedure are shown on each line (raw data, quantile normalisation applied, pseudo-modal shift applied, scaling applied). The first column shows all untreated (black line) and UV treated (red line) H3Ac binding datasets. Following the quantile normalisation step the replicate datasets follow the same overlapping distributions, hence only two visible lines. The second column shows a selected single untreated H3Ac dataset (black line) along with data mirrored about the zero point (red dashed line) and the SND over this same range (blue dotted line). The third column shows the same data as Q-Q plots, along with the position of the SND (blue dotted line), with data points below zero highlighted (grey box). These graphs show the estimated background region of the fully normalised data closely match the SND. All density plot *x*- and *y*-axes show ratio and density values respectively. All Q-Q plot *x*- and *y*-axes show theoretical and sample quantiles respectively.

The Sandcastle normalisation procedure described here was applied to two sets of *S. cerevisiae* ChIP-chip data: five repeats each of histone H3 acetylation (H3Ac) before and after UV treatment and three repeats each of Gcn5p binding before and at two time points after UV treatment. Both of these factors are present in the yeast genome at basal levels, and UV irradiation is known to induce changes^24^,^25^. We therefore investigated whether or not ChIP-chip data, normalised with Sandcastle, could be used to identify these same changes at high resolution throughout the yeast genome. The effects of normalisation on H3Ac data is represented in Fig. 4. The first column shows density curves of all datasets (pre-UV black and post-UV red) as they move through the different stages of the procedure. The different shapes of the densities of the two conditions demonstrate that ChIP-chip is capable of reflecting induced biological changes. However, as previously discussed, those changes cannot be examined in the data in its raw state. This is reflected in plots of these datasets along a short section of the genome (Supplementary Figs 1–4), which show that although the enriched regions are identifiable there is no consistency between the repeats of the different conditions. The second column of Fig. 4 shows the estimation of the background sub-population (red dashed line) in a single dataset (black solid line), with a good match between this and the SND (blue dotted line) after the final normalisation stage. This is also shown in normal Q-Q plots of the same data in the third column. Plots for all datasets are shown in Supplementary Figs 5–8.

There is no other normalisation procedure available for ChIP-chip data which enables comparative analyses of different datasets, and so the method presented here cannot be evaluated relative to any existing method. To validate its efficacy the results were instead compared with an alternative, quantitative technology — Q-PCR — to independently measure enrichment levels at a number of sites throughout the genome. Sites were selected from the normalised microarray data to display varying levels of initial enrichment and change in enrichment between experimental conditions (Supplementary Figs 9–10). Six sites were selected from the H3Ac datasets (H1–H6), representing varying levels of acetylation before treatment, with varying changes in response to treatment. Five sites were selected from the Gcn5p binding datasets (G1–G5), representing large, small and non-peak sites before treatment, with varying changes in response to treatment. For each set of Q-PCR data n ≥ 3.

**Figure 5:**
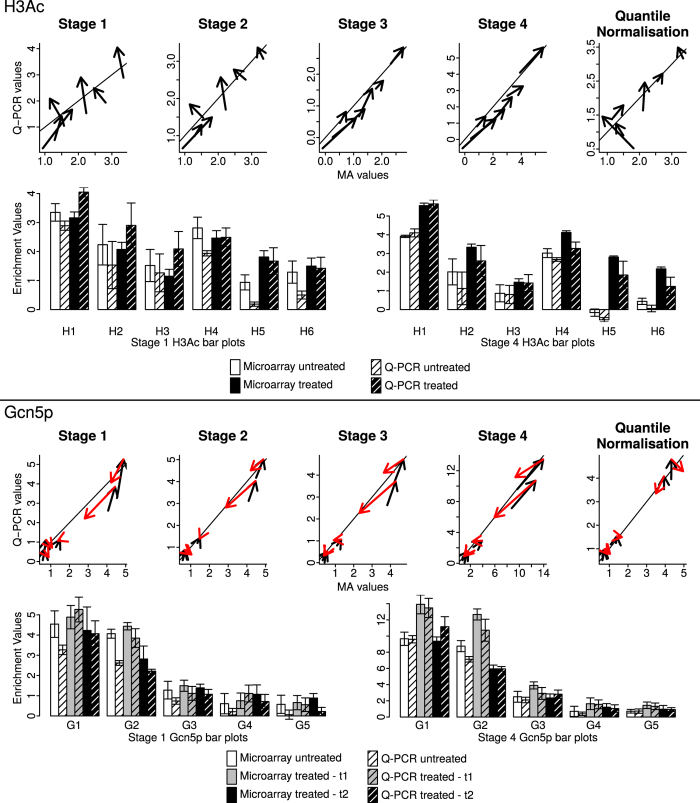
Validating the microarray normalisation procedure with Q-PCR. 6 H3Ac (top) and 5 Gcn5p (bottom) sites were examined with Q-PCR. For each normalisation stage (Stage 1 — Stage 4) a comparison between the microarray and Q-PCR data was made. Additionally all datasets were quantile normalised as one (Quantile Normalised). For each analysis Q-PCR data values were scaled to the microarray data values. Arrows represent 2 data points, from 2 experimental conditions, with the head of the arrow marking the second condition. The Gcn5p data shows arrows for untreated to time point 1 (t1; black) and time point 1 to time point 2 (t2; red). The angle of the arrows relative to the line *y* = *x* shows the similarity of the change between experimental conditions recorded by the two technologies, with angles close to this angle representing similar changes in both technologies. The distance of the points from the line *y* = *x* represents the similarity of values between the two technologies, with points closer to the line having more similar values. Bar charts show Q-PCR (shaded) and microarray (unshaded) values in preprocessed (Stage 1) and fully normalised (Stage 4) datasets for untreated (white) and treated (black, H3Ac; grey and black, Gcn5p) datasets. Error bars show standard errors.

Raw and normalised microarray and Q-PCR data for histone acetylation and Gcn5p binding are shown in Fig. 5. For each stage of the Sandcastle procedure a plot is shown representing the relationship between the microarray and Q-PCR data, with data from the same loci at the different experimental conditions linked by arrows. In each case the Q-PCR data are scaled to the microarray values (see Methods). The graphs reveal how the relationship between microarray and Q-PCR data change through the procedure, from very little similarity in the raw data to a high degree of similarity in the fully normalised data. For each stage of the procedure three metrics were calculated to analyse the relationship between the two sets of values, shown in Table 1. Spearman’s rank correlation *ρ* values were calculated between the microarray and Q-PCR values for all sites to give an overall measure of the concordance between values. The distance *d* of the points away from the line *y* = *x* represents the similarity of the microarray and Q-PCR values: the more similar the values the smaller the distance between the points and the line. The angle *a* of the arrows relative to the line *y* = *x* represents the similarity of the change in values between conditions: the more similar the change the closer to zero the angle becomes. This important measure shows how any experimentally induced changes are reflected in the microarray data, the revelation of which is the primary aim of the normalisation procedure. The Gcn5p binding data show increasing *ρ* values and decreasing averaged *d* and *a* values through the normalisation procedure. The H3Ac data show similar results, with a slight increase and decrease in the *ρ* and *d* values respectively at the last step.

Additionally, for each type of data, quantile normalisation alone was applied to all datasets together, as the method is intended to be used with gene expression microarray data. The resulting *ρ*, *d* and *a* values show that the Sandcastle normalisation procedure outperforms quantile normalisation, whether quantile normalisation is applied to all datasets together (Fig. 5 and Table 1 ‘Quantile Normalisation’) or to groups of replicate datasets (Fig. 5 and Table 1 ‘Stage 2’).

Barplots of the stage 1 and stage 4 data are also displayed, showing the increased similarity between microarray and Q-PCR data after the full normalisation procedure, along with error bars representing standard errors. Barplots of all data throughout the procedure are shown in Supplementary Figs 11–12. Together these results show that the raw microarray data give a poor representation of the biological differences between datasets from different experimental conditions. The association between microarray and Q-PCR values increases throughout the normalisation procedure, with the closest matches occurring after the application of the full procedure.

The robustness of the procedure was tested by performing the normalisation procedure on subsets of the H3Ac replicates. The data were split into all possible combinations of two groups of datasets (one of two datasets and one of three; 10 pairs total) and each group normalised independently. For each pair of datasets the resulting normalised data were averaged and Spearman’s rank correlation *ρ* values between them calculated. In addition, Spearman’s rank correlation *ρ* values were calculated between each of the normalised subsets and the average of the result of the normalisation performed on all five datasets, as it is intended to be performed. Comparing the pairs of datasets the median *ρ* value was 0.986 (range 0.972–0.991) and comparing subsets to the full complement the median *ρ* value was 0.996 (range 0.990–0.998). These values show that the normalisation method is robust.

These results demonstrate that the novel normalisation procedure presented here is capable of transforming ChIP-chip from a binary technology, permitting only the identification of enrichment, to one capable of allowing relative comparisons to be made between the values of enrichment between different experimental conditions, to determine how a factor of interest changes at a high resolution over a whole genome.

UV irradiation is known to induce genome-wide changes to the basal levels of H3Ac found in unirradiated cells^24^. These changes are reflected in the Q-PCR results (Fig. 5 top panel) but not the raw microarray data. Following Sandcastle normalisation the increases are reflected in the microarray data. Examining the whole dataset (Supplementary Table 2) shows that the increase is not uniform, but that the extents of the increases vary across the genome. UV irradiation is also known to induce an increase in Gcn5 binding levels, followed by a reduction soon afterwards^25^. Once again these changes are reflected in the Q-PCR results but only in the microarray data after Sandcastle normalisation (Fig. 5 bottom panel). Examining the data across the whole genome (Supplementary Table 2) again shows the extents of these changes vary at different sites. Analysing these changes throughout the genome by, for example, calculating the fold change between conditions, allows regions of different properties to be identified, which may correspond to, for example, different classes of binding sites in the case of the Gcn5p binding data, or different cellular responses to UV damage, in the case of the H3Ac data.

### Validating Sandcastle Peak Detection

Testing the performance of ChIP-chip EDMs poses a problem, because the nature of the investigations means that true positives and negatives are not known without full validation, which is impractical. To test the specificity of the Sandcastle method, four ‘background’ datasets were analysed, each comprising 41,452 probes. These datasets were generated from assays using a myc antibody against cells with no myc-tagged proteins, meaning that no material was IPed. The number of enriched probes found from these datasets is 5 (0.012% of the total) when analysing all datasets together, and an average of 24 (0.058% of the total) when analysing the four datasets individually, showing the method is capable of correctly identifying background probes with a very low false positive rate. This confirms that the assumptions of the EDM hold in real data.

**Figure 6:**
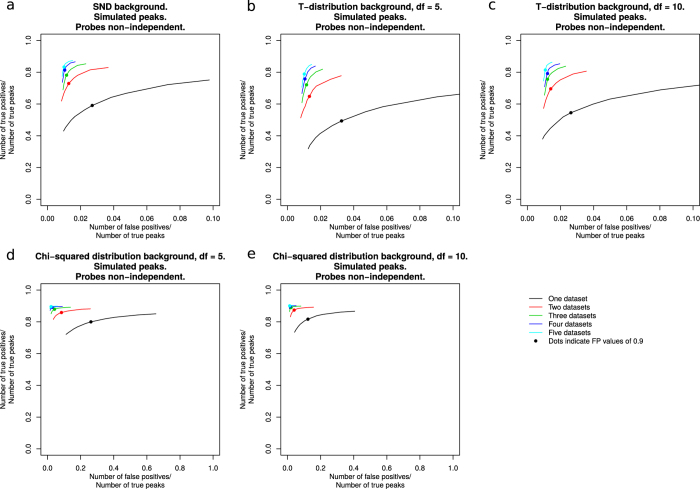
Performance of the Sandcastle EDM on simulated datasets. ROC-like curves showing the performance of the Sandcastle EDM at detecting simulated peaks in datasets with varying background distributions (data from a normal distribution, (**a**) T-distributions, (**b**,**c**) chi-squared distributions, (**d**,**e**)). *y*-axes show the proportion of true positives correctly identified and *x*-axes show the proportion of false positive results as a proportion of the number of true positives, such that the best possible results would lie in the top-left corners of the plots. Full calculation details are shown in the Methods section ‘ROC-like curves’. Coloured lines show the analysis of different numbers of datasets with results from varying FP values, each being the average of 50 simulations. Dots show the default FP value of 0.9. Increased performance is achieved when analysing multiple replicate datasets together than by analysing them individually. Even when simulating background distributions that violate the assumptions of the EDM the performance is still high. Results shown here are from data simulated with a degree of dependence between probe values in the same region, as may be expected in real data. Further plots are shown in Supplementary Figures S13–15.

To test the detection of enrichment, artificial datasets were created, to simulate binding sites and extended regions of enrichment (full details in Methods). The sizes of ‘peaks’ and enriched regions were randomised, to test the sensitivity of the method, and taken from a range defined by the simulated background distribution. The simulated enriched values were in the range of, and in many cases smaller, than values seen in our real ChIP-chip data, so as to make the simulations as realistic as possible. The distributions of the background sub-populations were varied to simulate data that meet and violate the assumptions of the method. The SND was used to simulate data meeting the assumptions of the method, the T-distribution was used to simulate data with a distribution with extended tails, and the chi-squared distribution was used to simulate data with skewed distributions. Background data were left as random (independent) values, or processed with the R density function to simulate relationships between probes a set distance apart (non-independence, as may be the case in real data). All datasets were normalised with Sandcastle normalisation prior to analysis and were analysed individually and in groups of 2, 3, 4 and 5, to investigate the performance of the EDM with varying numbers of datasets. Each set of simulations were repeated 50 times and enrichment detection performed with a range of FP values to create ROC-like curves (see Methods section ‘ROC-like curves’ for description) of the results (Fig. 6 and Supplementary Figs 13–15).

In all cases, whether investigating peaks or regions, and whether or not datasets are simulated with independent background probes, results are consistently best for datasets analysed in groups of 5, and become as fewer datasets are analysed. All results in Fig. 6, from all distributions, show maximum *y*-axis values of over 0.80 (over 80% of true positives correctly identified), with corresponding *x*-axis values of less than 0.02 (the number of false positive results is less than 2% of the number of true positives). These results show that the Sandcastle EDM performs very well, and even if the properties of the background distributions of datasets violate the assumptions of the software it is able to find a large proportion of the true peaks or enriched regions with a small proportion of false positive results. The resulting values from the default FP value of 0.9 is highlighted in each plot, which can be seen to lie at or close to the point of optimal performance in all cases. This provides good evidence that 0.9 is a suitable default value for the algorithm.

Combined with the Q-PCR validation of a selection of sites found with the EDM on real ChIP-chip datasets, this provides good evidence that the EDM can reliably detect enrichment in ChIP-chip datasets. We successfully compared our method with a previously published method, ChIPOTle^21^ (see next section). Other methods tested failed to run (see Supplementary Table 1).

### Comparison with ChIPOTle

**Figure 7:**
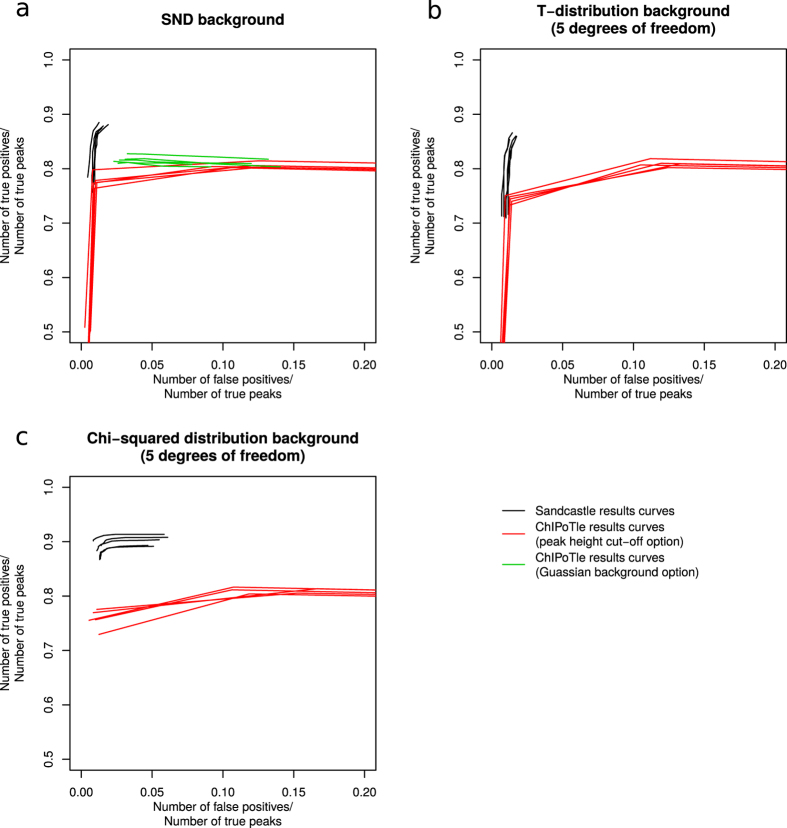
Comparison of Sandcastle with ChIPOTle. ROC-like curves showing the results of Sandcastle peak detection (black lines) compared with ChIPOTle peak detection (green and red lines) in data with background sub-populations simulated with data from normal (**a**), T- (5 degrees of freedom; (**b**)) and chi-squared (5 degrees of freedom; (**c**)) distributions. *y*-axes show the proportion of true positives correctly identified and *x*-axes show the proportion of false positive results as a proportion of the number of true positives, such that the best possible results would lie in the top-left corners of the plots. Full calculation details are shown in the Methods section ‘ROC-like curves’. For the normally distributed datasets (**a**) ChIPOTle was run with options assuming a Gaussian distribution (green lines) and the default option of using a peak height cut-off (red lines). The other distributions (**b**,**c**) were run using only the peak height cut-off option (red lines). For all tests it can be seen that Sandcastle outperforms ChIPOTle, as the results lie closer to the top-left corners of the plots.

Simulated datasets were used to compare the performance of the Sandcastle EDM with ChIPOTle^21^, an existing ChIP-chip peak detection software tool (see Methods). Five sets of simulated datasets for each of the normal, T (5 degrees of freedom) and chi-squared (5 degrees of freedom) background distributions were tested (see Methods) and ROC-like plots created of the results (see Methods section ‘ROC-like curves’ for description), along with the results of the analyses of the same datasets in Sandcastle. Each of the curves is plotted separately in Fig. 7, and it can be seen that Sandcastle (black lines) outperforms ChIPOTle (red lines; results from varying the peak height cut-off option, and green lines; results from varying the P-value cut-off option, where background data are assumed to be Gaussian) in all cases, being as they are, closer to the top-left of the plots. Comparing all results at the *x*-axis point of 0.05 (the proportion of false positive results is 5% of the total number of true peaks) shows corresponding *y*-axis values (the proportion of true peaks correctly identified) in the approximate range of 0.76–0.79 for the ChIPOTle peak height cut-off results (red lines), 0.81–0.82 for the ChIPOTle P-value cut-off results (green lines) and ≥0.86 for the Sandcastle results (black lines), showing that Sandcastle is capable of finding more true positive results than ChIPOTle for the same number of false positive results generated.

All results generated from the ‘Assume Gaussian Background’ option and some results generated from the default ‘Peak Height Cut-off’ option show the unusual trend of decreasing *y*-axis values with increasing *x*-axis values. This means that with the increasing P-value and decreasing peak height cut-offs used to generate these results the software stops detecting some peaks that it was previously able to detect, for unknown reasons relating to the functioning of ChIPOTle.

A significant disadvantage of the ChIPOTle software is that it does not have the functionality to determine regions of enrichment, so this element of the simulation could not be compared.

## Discussion

We have presented Sandcastle, an R software package containing tools for advanced, novel analyses of ChIP-chip data. It contains a new normalisation procedure, enabling the extraction of previously irretrievable information from datasets, an enriched region detection algorithm, which removes the need for the application of a multiple testing correction, and a suite of tools for analysing the resulting data. This enables a new dimension of ChIP-chip analyses to be undertaken which may be applied to legacy, as well as new, datasets. The normalisation procedure has been validated with Q-PCR at a number of sites across the yeast genome and the EDM has been tested with a number of simulated datasets and compared to ChIPOTle, an existing peak detection method.

The normalisation procedure allows relative comparisons of enrichment levels from different experimental conditions, to facilitate the discovery of biologically relevant changes beyond the current presence or absence calling. No other current method can achieve this. It allows changes in levels of protein binding, epigenetic modifications or other biological factors to be detected between conditions, where previously only the presence or absence of enrichment could be detected in each condition. This represents a significant advance in the analysis of this type of data. Coupled with the enriched region detection algorithm, Sandcastle provides a complete platform for these analyses. The procedure normalises data to the SND, thus treating datasets from different conditions independently. This means that datasets from one condition are not influenced by any unusual features in another, and that datasets from additional experimental conditions can be added, normalised and analysed alongside the original data without having to re-normalise.

We validated our method by comparing the results of the normalised data to 27 sets of Q-PCR data. We calculated three metrics to determine how well the two match. Spearman’s rank correlation *ρ* values, where higher values show a higher overall correlation between the two sets of data, are higher in the normalised data than the raw data. The distances *d* of the points to the line *y* = *x*, where lower values show more similarity between the two sets of values, are lower in the normalised data than the raw data. The angle *a* of the arrows relative to the line *y* = *x*, where lower values show more similarity of the induced changes detected by the two technologies, are lower in the normalised data than the raw data. All of these results show that the normalised microarray data provide a good match to the Q-PCR data, revealing patterns not present in the raw data, showing the significance and power of the procedure. Coupled with the fact that the normalisation procedure transforms the microarray data to be able to represent, on a genome wide scale, known global biological phenomena that cannot be seen in the raw data — that is, UV-induced changes in H3Ac levels and Gcn5p binding — these results show that the normalisation procedure transforms ChIP-chip into a technology that allows relative comparisons to be made between datasets throughout genomes.

There are some limitations to the procedure. For example, the method relies on the assumption that the background sub-population of data approximately follow a ND. In our experience, and that of others^21^, this assumption holds for the vast majority of datasets. A clear maxima is required in the density of the background sub-population, at which the software assigns the pseudo-mode. If this criterion is not met then the normalisation cannot be applied. In this situation data from spike-in probes may be used in place of the estimated background.

The shifting of the data may introduce variations, as it is based on an estimated pseudo-mode. There are several other sources of variation in microarray experiments, which limit the accuracy of results, and additional small variations thus introduced are unlikely to adversely affect any conclusions which may be drawn. This should be borne in mind and taken into account when performing analyses. Microarray data can be used to generate hypotheses, which can be further tested and validated by other, independent methodologies.

The method has been developed to allow comparisons of differences in a single parameter due to changes in experimental conditions. It cannot compare between datasets that examine different parameters. For example, datasets generated with different antibodies will potentially have variations due to differing antibody performances.

The EDM has been designed to take account of the familywise error rate from the outset, thus removing the need to apply an additional multiple testing correction to final results. This removes the additional source of variation that can be introduced by differing conservation levels of different multiple testing corrections. All regions of enrichment are initially identified, and a peak identification processes subsequently invoked if required. This allows relevant data to be analysed for any given dataset. All replicate datasets are analysed at once, which improves the power of detection. Good reproducibility between datasets is therefore required. With several repeats of a dataset being analysed together, smaller binding peaks can be detected than is the case when analysing all the datasets separately and combining the results. Therefore on high resolution microarrays with several repeats it should be possible to detect small binding sites with a high level of confidence. Simulated datasets were generated to analyse with the EDM, the results of which showed that the method is able to correctly identify a large proportion of true positives with only a small proportion of false positives, even when data were simulated to not meet the assumptions of normality in the background sub-population. The Sandcastle results were compared with the results of analysing the same simulated data with ChIPOTle, which showed that Sandcastle is better able to correctly identify more true positives whilst identifying fewer false positives, especially where the background data are not normally distributed.

Comparisons of peaks or enriched areas from different arrays can be made by statistical or graphical methods, using the Sandcastle tools. The graphical plotting functions provided in the package enable results to be simply and clearly displayed for ease of data analysis. Results are provided in easily accessible formats for users to plot as they wish.

## Conclusions

Overall, this package represents an important advance in the processing of ChIP-chip data. Comparisons of data from different arrays add a new dimension to analyses and can be used to facilitate the discovery of new biological phenomena. The datasets analysed here, and with other, unpublished data, have been shown to match results generated by an independent analysis method, namely Q-PCR, following the normalisation procedure. This confirms the validity of the technique. Specialist computing equipment is not required; a normal desktop computer with a recent version of R can load, normalise and detect enrichment in a batch of data in minutes. The software and documentation are available for download from http://reedlab.cardiff.ac.uk/sandcastle.

## Methods

### Calculating the values in Table 1

Spearman’s rank correlation *ρ* values were calculated using the R function cor.test with the method ‘spearman’.

Distance values *d* were calculated for each point using the formula:


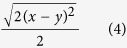


and scaled to the largest microarray value of the plot.

Angle values *a* were calculated using the R script: abs((round(atan((y2 − y1)/(x2 − x1)) * (180/pi), 2)) − 45) Where any values over 90 were reduced by 180, such that the maximum angle returned was 90 (perpendicular to the line *y* = *x*) and the minimum was 0 (parallel to the line *y* = *x*).

### Comparing Q-PCR and microarray data

To create Fig. 5 individual Q-PCR replicate data values were scaled to the microarray data values for each plot (such that the largest Q-PCR value is equal to the largest microarray value and the smallest Q-PCR value is equal to the smallest microarray value) using the following formula, where *m* = the minimum microarray value, *M* = the maximum microarray value, *q* = the minimum Q-PCR value, *Q* = the maximum Q-PCR value and *v* = the Q-PCR value to be scaled:


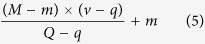


### Dataset simulation for enrichment detection testing

The performance of the enrichment detection process was tested using simulated datasets. These datasets were generated by the following process:

- Simulated 2000 random peak sites or 500 random enriched region sites. Lengths of enriched regions were randomly varied in length from 5 to 41 probes.
- Generated random data for the background distributions. Data from the standard normal distribution was used to represent data expected under the assumptions of Sandcastle. Data from T-distributions with 5 and 10 degrees of freedom were used to simulate background distributions with longer tails than expected in real data. Data from chi-squared distributions with 5 and 10 degrees of freedom were used to simulate skewed data, with an increased number of values on the right-hand-side of the distribution.
- Simulated random enrichment levels. Enrichment levels were based on the properties of the simulated background dataset such that they were consistent between all simulations. After Sandcastle normalisation the enrichment values ranged from approximately 2 to 10, with a median around 5, for all simulated datasets. These values are lower than what we see in many of our real ChIP-chip datasets.
- Simulated spurious peaks. Random values from the same distribution as that used to create the simulated enrichment levels were added to 200 random probes in the peak simulations and 50 random probes in the regions simulations, outside of the simulated enrichment sites. These sites varied randomly between datasets and are intended to mimic inherent variations in microarrays, beyond simple background noise.
- The R density function was used to simulate enrichment, resulting in a region of enrichment around each site, as would be expected in real data. Additionally, half of the datasets had the density function applied to the simulated background data as well, to simulate non-independence between all probes on the array. Final data were created by the addition of the simulated enriched data and the simulated background data. Therefore the randomness of the simulated background data was maintained in the simulated enriched data. A chromatin shear size of 600 bp was simulated.
- This process was repeated 5 times to generate 5 datasets with the same simulated peak/enriched regions sites, but different random background values and enrichment levels for each simulation. This is intended to mimic five replicate datasets of the same experimental condition.
- Peak or enrichment detection was performed on the simulated peak and enriched region datasets respectively, with the first dataset analysed alone, the first and second together, the first three together, the first four together and all five together, with FP values of 0.004, 0.009, 0.04, 0.09, 0.4, 0.9 (the default), 4, 9, 40, and 90.
- Each set of simulations was repeated 50 times for each test and ROC-like plots of the results created, with five separate lines showing the results from each of the five combinations of datasets, averaged over the 50 simulations.
- This generated a total of 5 (different background distributions) × 2 (peaks/enriched regions) × 2 (independent/non-independent background data) × 5 (simulated datasets per test) × 50 (simulations) = 5000 simulated datasets.

Results are plotted as ROC-like curves, as described in the following section.

### ROC-like curves

To compare the performances of the methods, ROC-like curves were generated from results as described in^7^. Here the *y*-axes show (the number of true positive results obtained)/(the total number of actual peaks), and the *x*-axes show (the number of false positive results obtained)/(the total number of actual peaks). This method is used because the very large number of true negatives can “result in a large absolute number of false positives even at extremely low false-positive rates”^7^. The best possible result appears at the top-left of these plots, representing results containing the correct identification of all true positives (*y*-axis value of 1, representing 100% sensitivity) and no false positives (*x*-axis value of 0, representing 100% specificity).

Enriched region plots use a modified version of this, where the *y*-axes show (the number of simulated regions overlapping a detected region by 80% or more)/(the total number of simulated regions) and the *x*-axes show (the number of detected regions not overlapping an enriched region by 80% or more)/(the total number of simulated regions), to account for the fact that regions of enrichment, rather than peaks at specific sites, are being analysed.

### Running ChIPOTle

For each of the simulations with normal, T (5 degrees of freedom), and chi-squared (5 degree of freedom) distribution background sub-populations, 5 sets of 5 simulated datasets (see ‘Dataset simulation for enrichment detection testing’ section) were written into text files from R and then loaded into ChIPOTle in Excel for peak detection. For each set of data the 5 repeats were averaged (as suggested by the documentation) and analysed in 2 subsets (chromosomes 1–9 and 10–17), as ChIPOTle could not process the datasets in their entirety. The ‘Window Size’ was set to 600 (the simulated size) and the ‘Step Size’ to 150 (one quarter of the window size, as suggested by the documentation). All other settings were left as their defaults and results generated with ‘Peak Height Cut-off’ values of 0.25, 0.5, 1 (the default), 2 and 4, to create a range of results from which to calculate the values for the ROC-like curves. In addition the ‘Assume Gaussian’ option was selected for the datasets generated with normally distributed background values, as these data should meet the assumptions as detailed in the documentation, and results generated with ‘P Value Cut-off’ values of 0.00001, 0.0001, 0.001 (the default), 0.01 and 0.1. Detected probe IDs were loaded back into R and compared to the probe IDs at the simulated peak sites using the calculations detailed in the previous section.

### Quantitative PCR

Quantitative real-time PCR was performed using the iQTM SYBRgreen supermix (Bio-Rad) and the iCycler MyiQTM real-time PCR detection system (Bio-Rad). Primers were designed around the corresponding microarray probes and are available on request. The reaction was performed using the following PCR program: 1. 95 °C for 3 minutes, 2. 95 °C for 15 seconds, 3. 55 °C for 20 seconds -followed by plate reading. Steps 2–3 were repeated 44 times. In addition a melt curve was undertaken to ensure a single PCR product and all samples were amplified a minimum of three times.

## Additional Information

**How to cite this article**: Bennett, M. *et al.* Sandcastle: software for revealing latent information in multiple experimental ChIP-chip datasets via a novel normalisation procedure. *Sci. Rep.* **5**, 13395; doi: 10.1038/srep13395 (2015).

## Supplementary Material

Supplementary Information

## Figures and Tables

**Figure 1:**
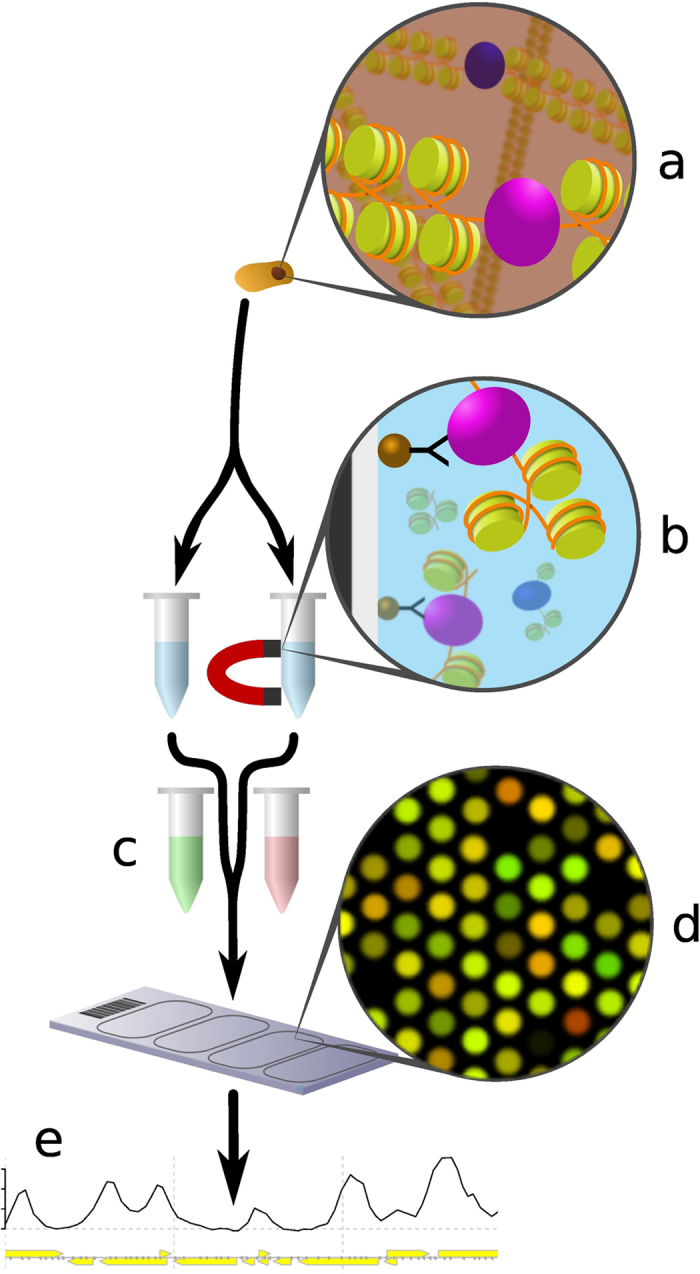
Representation of the ChIP-chip procedure. Proteins are crosslinked to chromatin (**a**) which is extracted, sonicated and split into two samples. IP is carried out on one sample to separate out the chromatin bound to the factor of interest (**b**). Both samples are purified to DNA, amplified by PCR and differentially labelled (**c**). They are allowed to hybridise to the microarray probes and the resulting intensity values from the scanned image (**d**) are converted to numerical values which can be plotted (**e**) and processed as required by the investigation. Figure created and drawn by Mark Bennett.

**Table 1:** Associations between microarray and Q-PCR data.

**Dataset**	***ρ***	***d***	***a***
H3Ac Stage 1	0.720	0.098	49.843
H3Ac Stage 2	0.846	0.070	51.720
H3Ac Stage 3	0.979	0.059	8.438
H3Ac Stage 4	0.965	0.061	6.597
H3Ac Quantile normalisation	0.804	0.074	49.027
Gcn5 Stage 1	0.943	0.072	34.994
Gcn5 Stage 2	0.954	0.035	29.779
Gcn5 Stage 3	0.975	0.040	15.207
Gcn5 Stage 4	0.983	0.029	11.257
Gcn5 Quantile normalisation	0.961	0.034	50.083

Values calculated from each of the normalisation stages shown in Fig. 5. *ρ* shows Spearman’s rank correlation coefficients for all data points. *d* shows the mean distance values to the line *y* = *x* for all data points. *a* shows the mean angle for all arrows.
