# Supplementary material for: Sandcastle: software for revealing latent information in multiple experimental ChIP-chip datasets via a novel normalisation procedure
Source: Sci Rep. 2015 Aug 26;5:13395. doi: 10.1038/srep13395 (PMC4549617; doi:10.1038/srep13395)
Supplement: Supplementary Information [file srep13395-s1.pdf]

Sandcastle: software for revealing latent information in multiple experimental ChIP-chip datasets via a novel normalisation procedure

Mark Bennett, Katie Ellen Evans, Shirong Yu,
Yumin Teng, Richard M. Webster, James Powell,
Raymond Waters and Simon H. Reed

Cancer Genetics Building,
Cardiff University,
School of Medicine,
Heath Park,
Cardiff,
CF14 4XN,
UK.

Supplementary Information

Method	Program Publicly Available	Comments
SAEM (Ren et al, 2000)	No	-
MDScan (Liu et al, 2002)	No	-
Median percentile rank (Buck and Lieb, 2004)	No	-
Peakfinder (Glynn et al, 2004)	Yes	Error message prevents running the program.
No name (Cawley et al, 2004)	No	-
HMM (Li et al, 2005)	No	-
Chipper (Gibbons et al, 2005)	Yes	Not suitable for our microarray resolution.
No name (Kim et al, 2005)	No	-
TileMap (Ji and Wong, 2005)	Yes	Runs via Cisgenome. Required files could not be generated by following the instructions.
JBD (Qi et al, 2006)	No	-
TAMALPAIS (Bieda et al, 2006)	Yes	Not suitable for our microarray resolution.
MAT (Johnspn et al, 2006)	Yes	Only suitable for Affymetrix data.
Permuta (Lucas et al, 2007)	No	-
MA2C (Song et al, 2007)	Yes	Only suitable for Nimblegen data.
Mpeak (Zheng et al, 2007)	Yes	Error message prevents running the program.
Tilescope (Zhang et al, 2007)	No	-
Poisson approximation (Zhang, 2008)	No	-
Splitter (Shulha et al)	Yes	Not documented.
CoCAS (Benoukraf et al, 2009)	Yes	Requires unavailable additional software.
JAMIE (Wu and Ji, 2010)	Yes	Requires unavailable additional software.
DECODE (Barrett et al, 2011)	No	-
Wavelet (Karpikov, 2011)	No	-

Supplementary Table S1: Table showing published ChIP-chip peak detection tools.

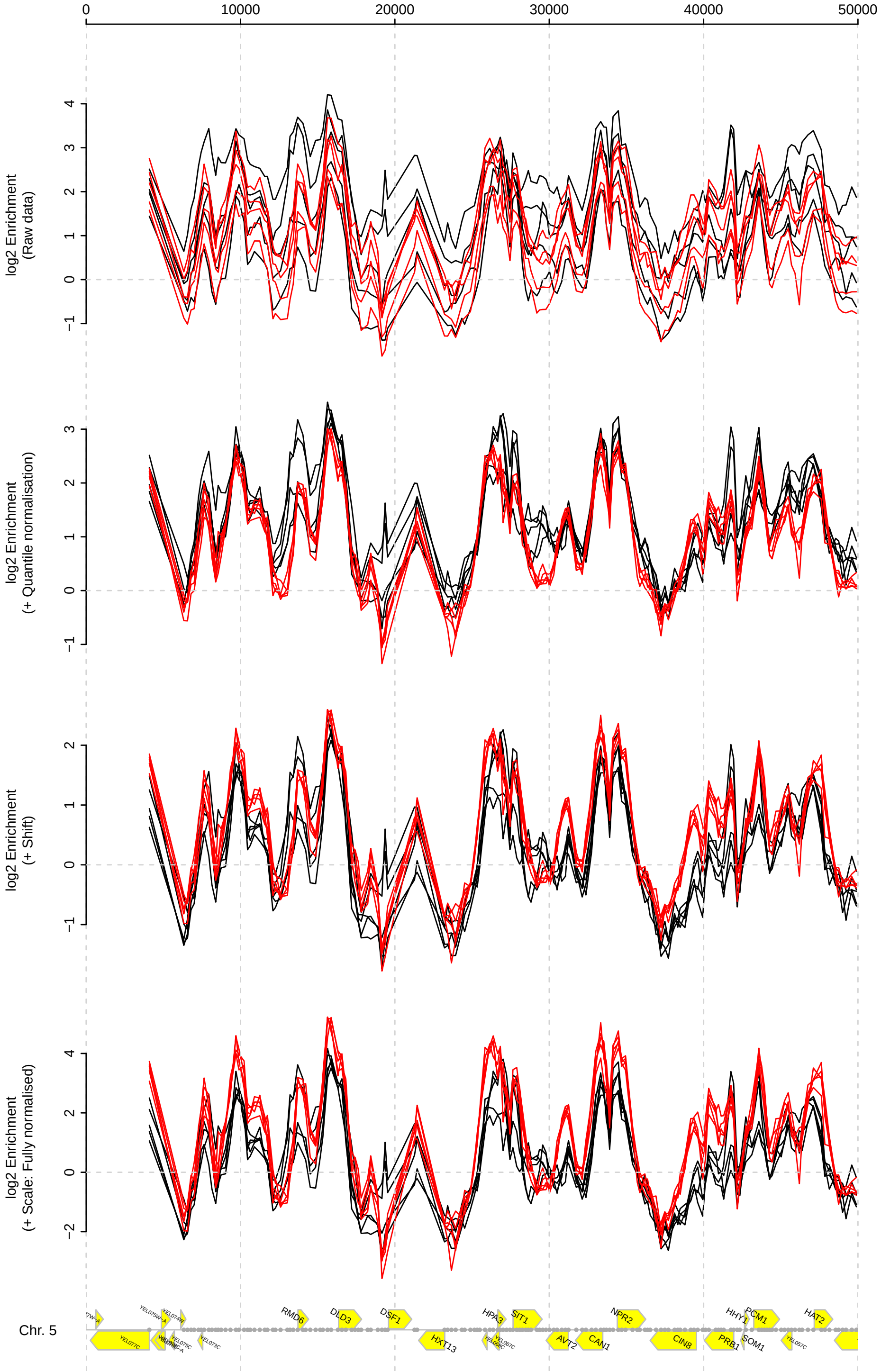

Supplementary Figure S1: Yeast genome section showing histone H3 acetylation in all replicate datasets after each of the four stages of the Sandcastle normalisation procedure. Black: untreated. Red: UV treated.

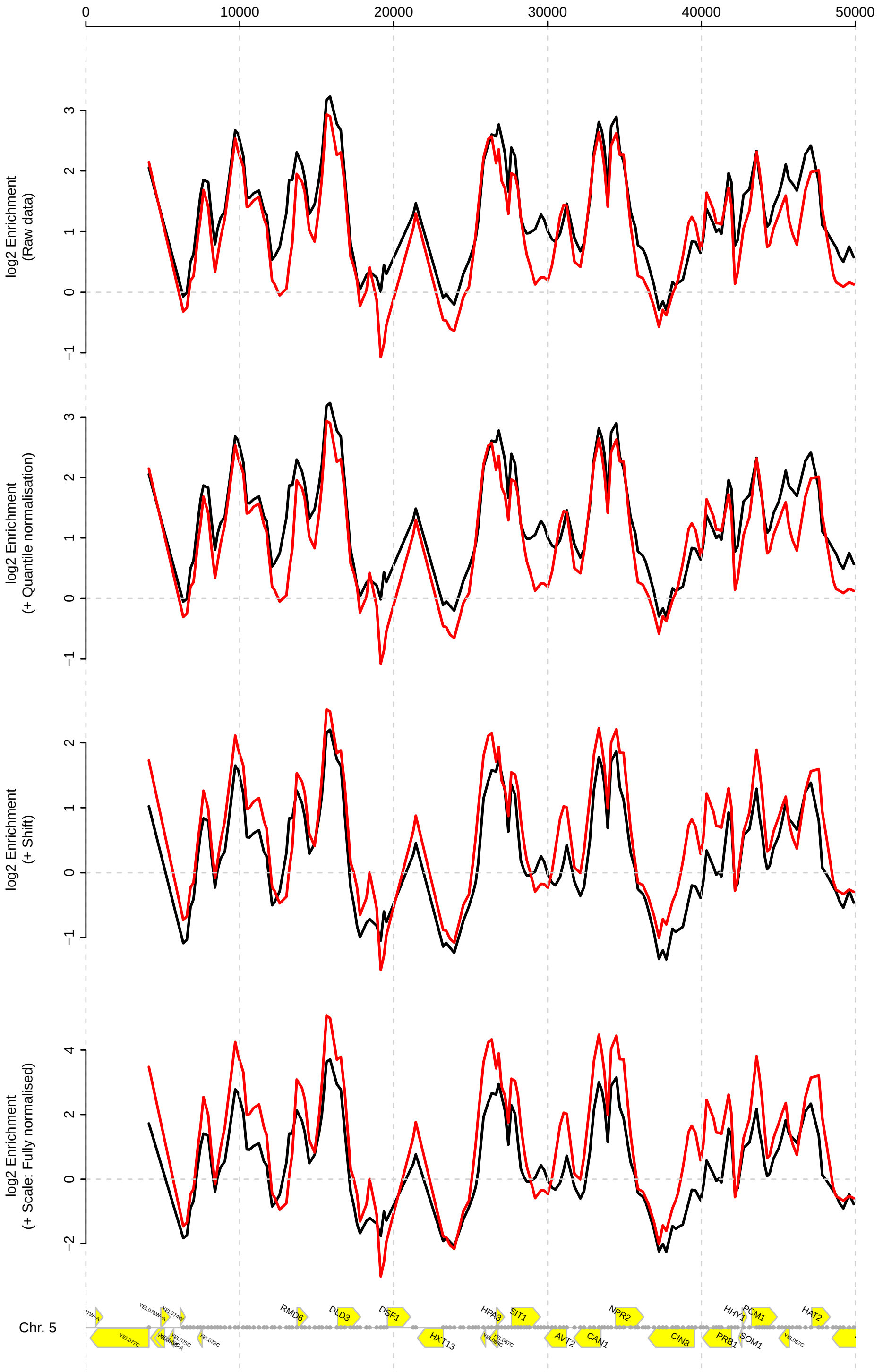

Supplementary Figure S2: Yeast genome section showing histone H3 acetylation in averaged datasets after each of the four stages of the Sandcastle normalisation procedure. Black: untreated. Red: UV treated.

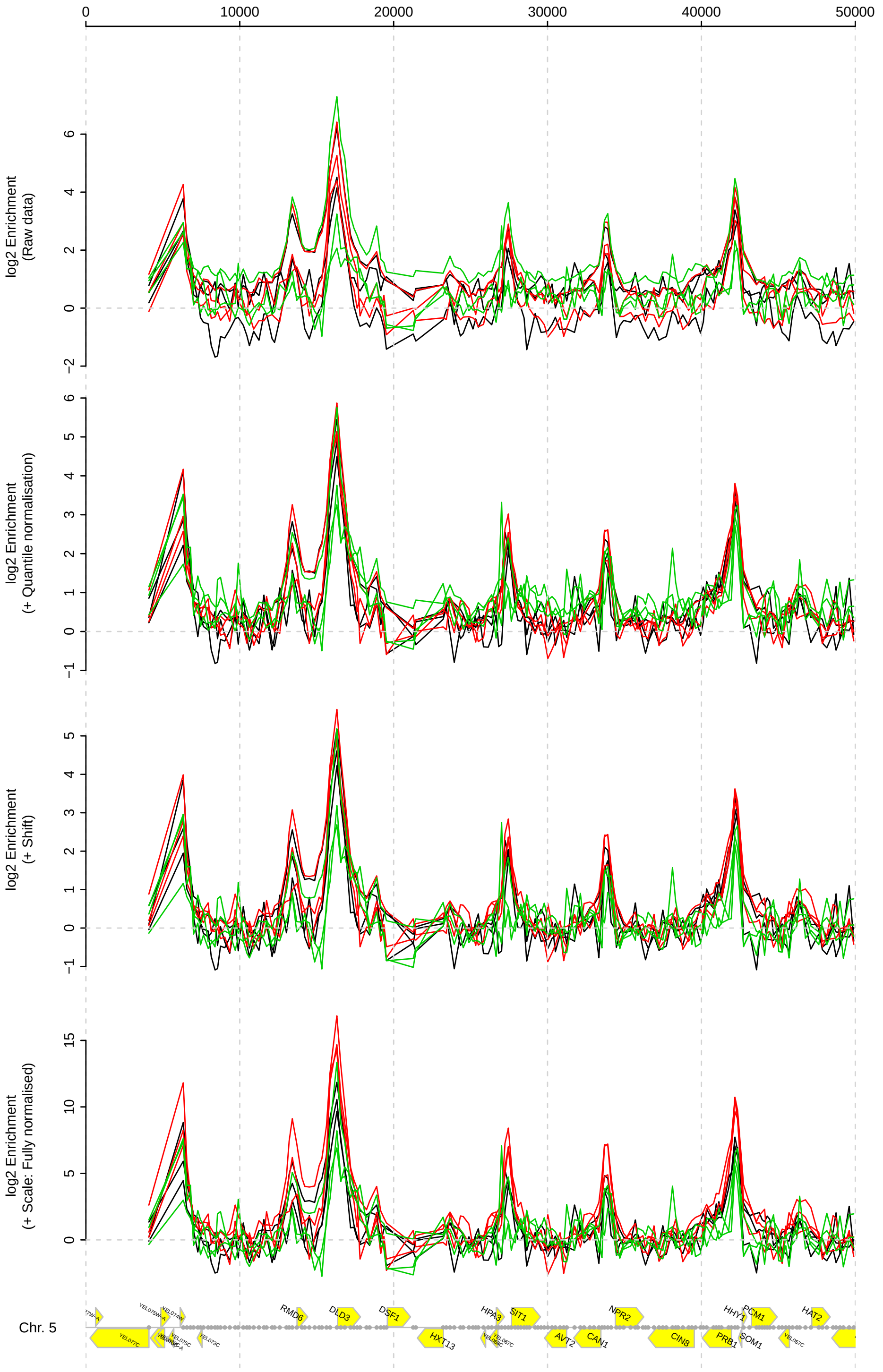

Supplementary Figure S3: Yeast genome section showing Gcn5p binding in all replicate datasets after each of the four stages of the Sandcastle normalisation procedure. Black: untreated. Red: UV treated t1. Green: UV treated t2.

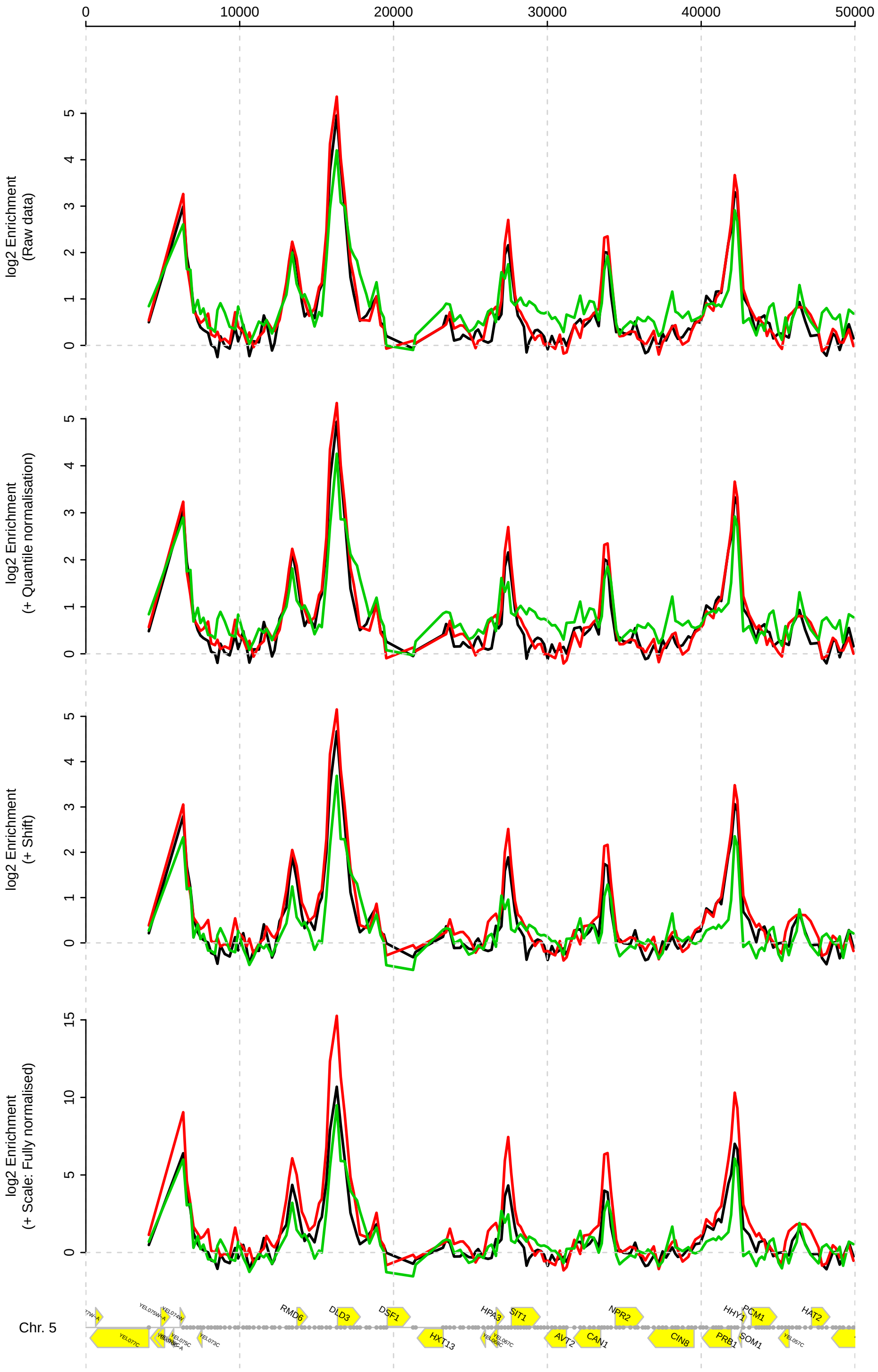

Supplementary Figure S4: Yeast genome section showing Gcn5p binding in averaged datasets after each of the four stages of the Sandcastle normalisation procedure. Black: untreated. Red: UV treated t1. Green: UV treated t2.

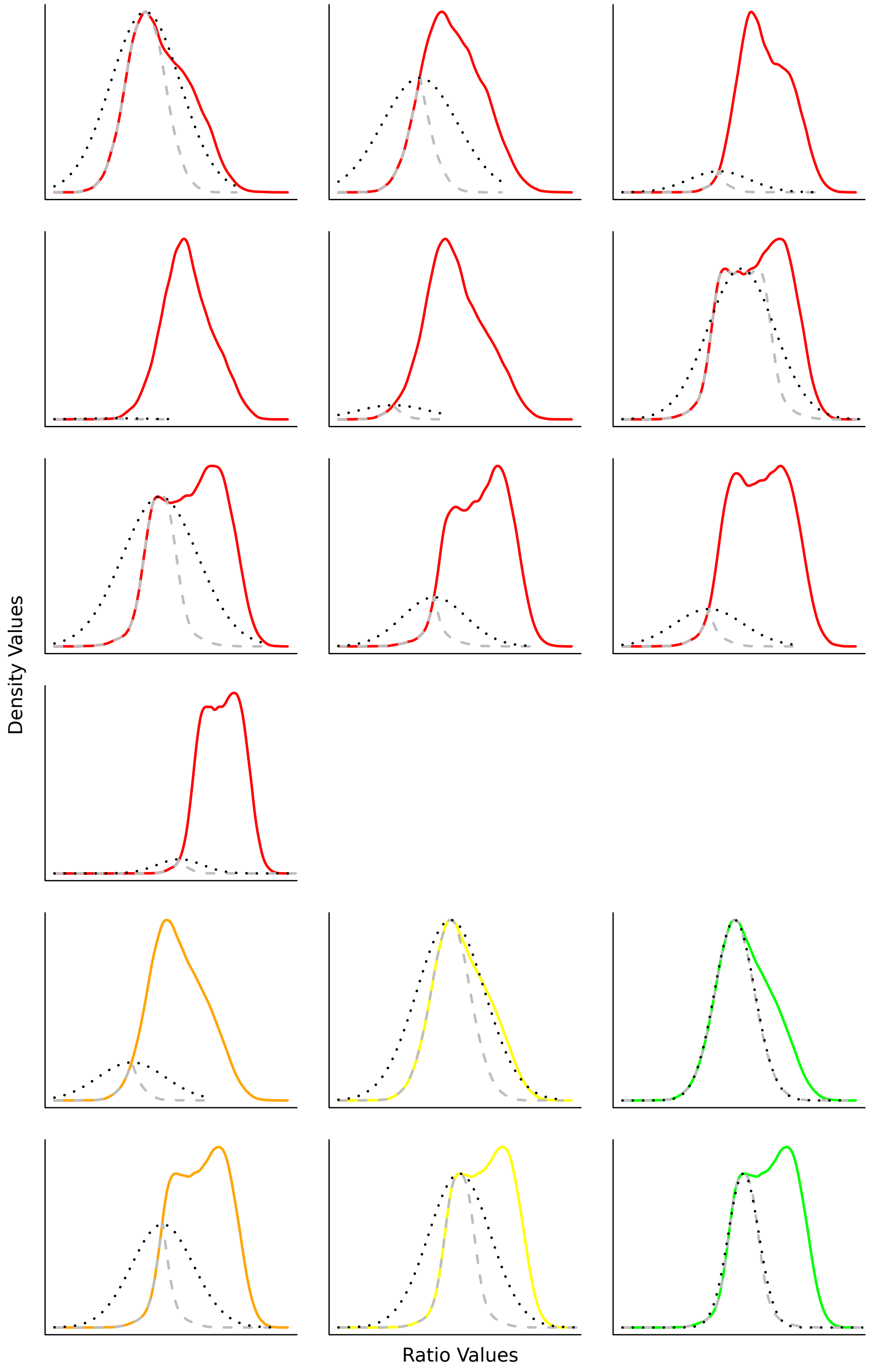

Supplementary Figure S5: Density histograms of all H3Ac datasets through the normalisation procedure. Red: Raw datasets. Orange: Data following quantile normalisation (quantile normalisation means all datasets now follow the same distribution and so only one from each experimental conditions is shown). Yellow: data following pseudo-modal shift. Green: fully normalised data. Dashed grey lines show negative data mirrored about zero. Dotted black lines show the standard normal distribution over the same range.

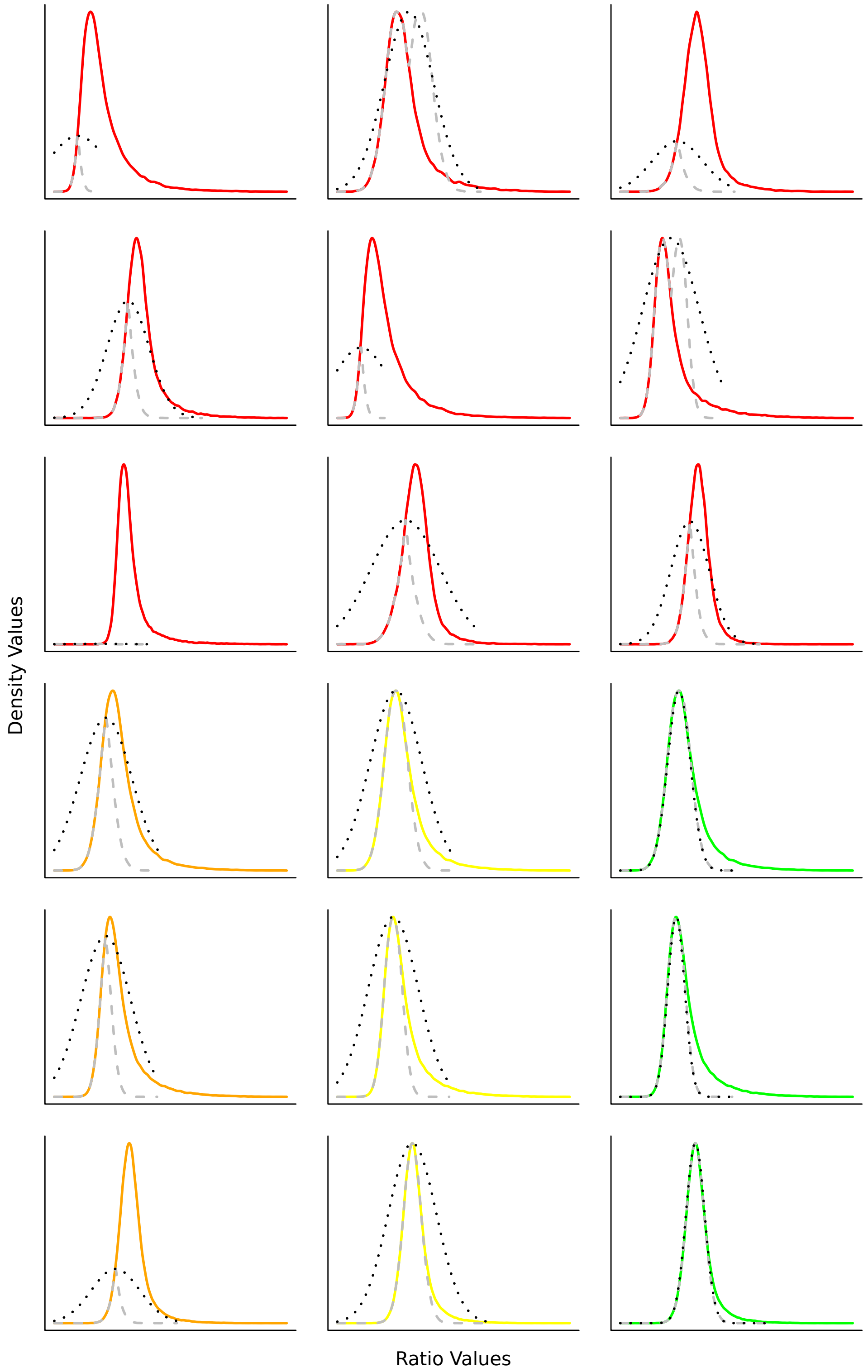

Supplementary Figure S6: Density histograms of all Gcn5p datasets through the normalisation procedure. Red: Raw datasets. Orange: Data following quantile normalisation (quantile normalisation means all datasets now follow the same distribution and so only one from each experimental conditions is shown). Yellow: data following pseudo-modal shift. Green: fully normalised data. Dashed grey lines show negative data mirrored about zero. Dotted black lines show the standard normal distribution over the same range. All x-axes are ratios. All y-axes are densities.

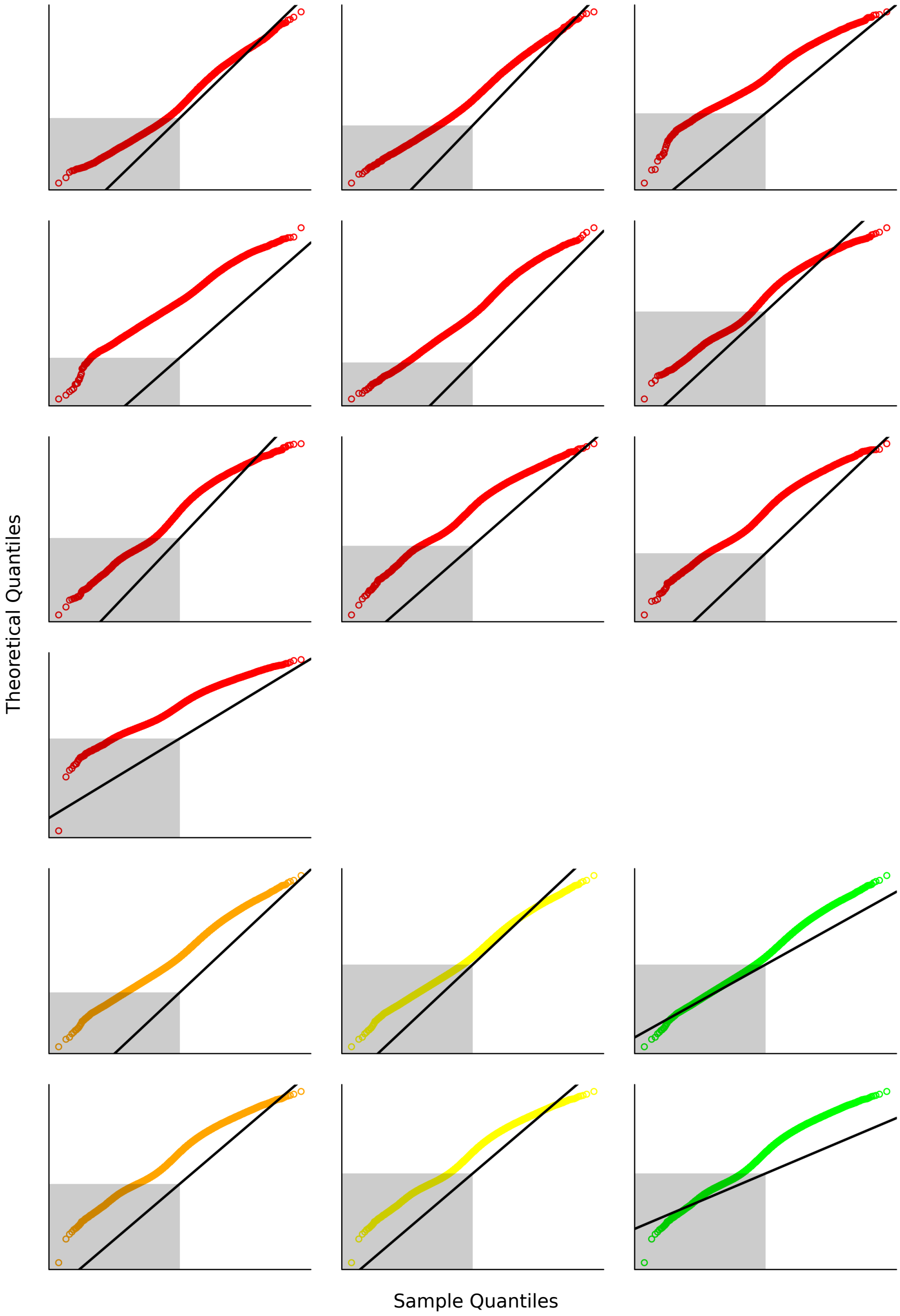

Supplementary Figure S7: Q-Q plots of all H3Ac datasets through the normalisation procedure. Red: Raw datasets. Orange: Data following quantile normalisation (quantile normalisation means all datasets now follow the same distribution and so only one from each experimental conditions is shown). Yellow: data following pseudo-modal shift. Green: fully normalised data. Black lines show the line $y = x$ representing the standard normal distribution. Grey boxes show negative data which should match the standard normal distribution following normalisation. All x-axes are theoretical quantiles. All y-axes are sample quantiles.

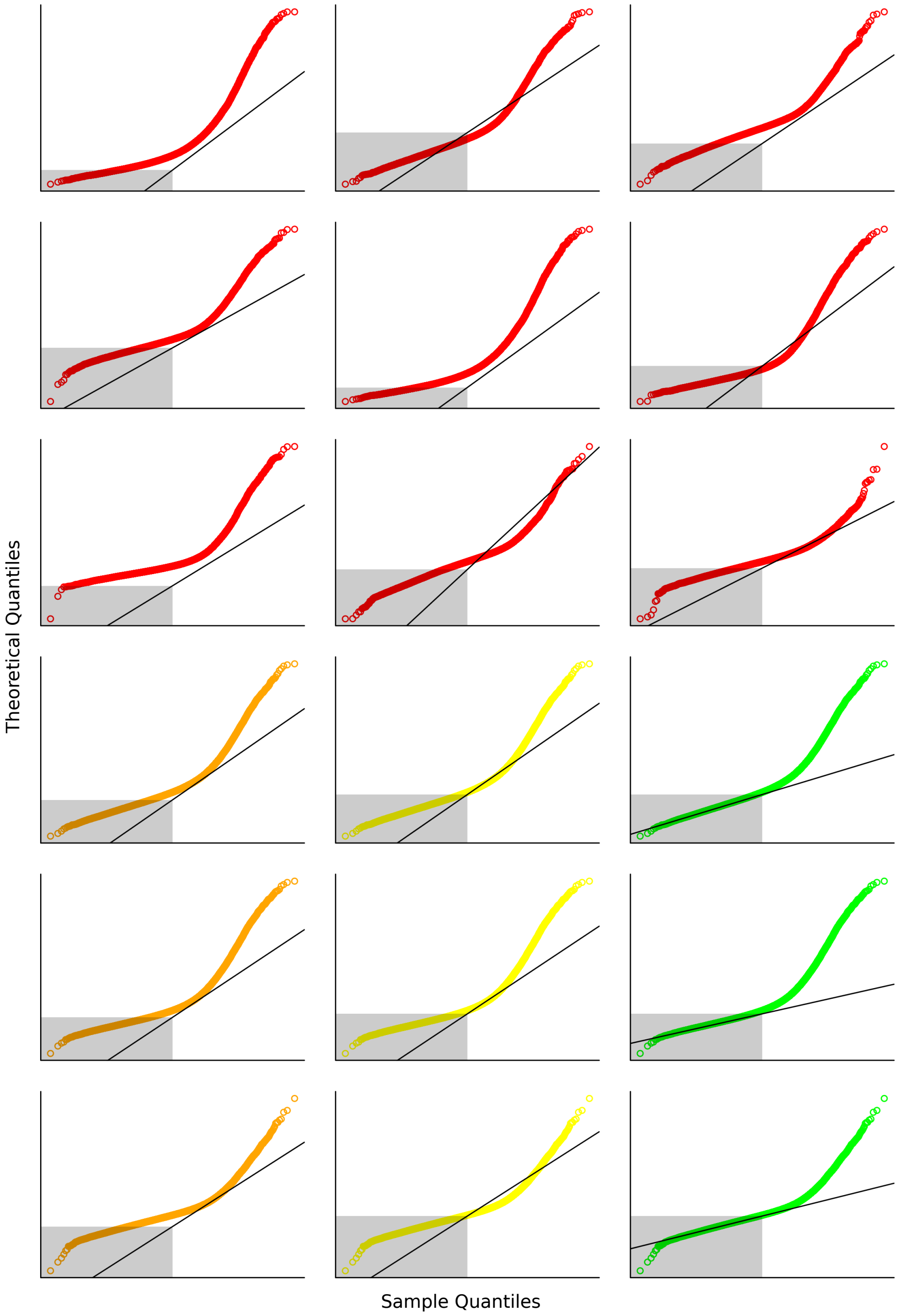

Supplementary Figure S8: Q-Q plots of all Gcn5p datasets through the normalisation procedure. Red: Raw datasets. Orange: Data following quantile normalisation (quantile normalisation means all datasets now follow the same distribution and so only one from each experimental conditions is shown). Yellow: data following pseudo-modal shift. Green: fully normalised data. Black lines show the line $y = x$ representing the standard normal distribution. Grey boxes show negative data which should match the standard normal distribution following normalisation. All x-axes are theoretical quantiles. All y-axes are sample quantiles.

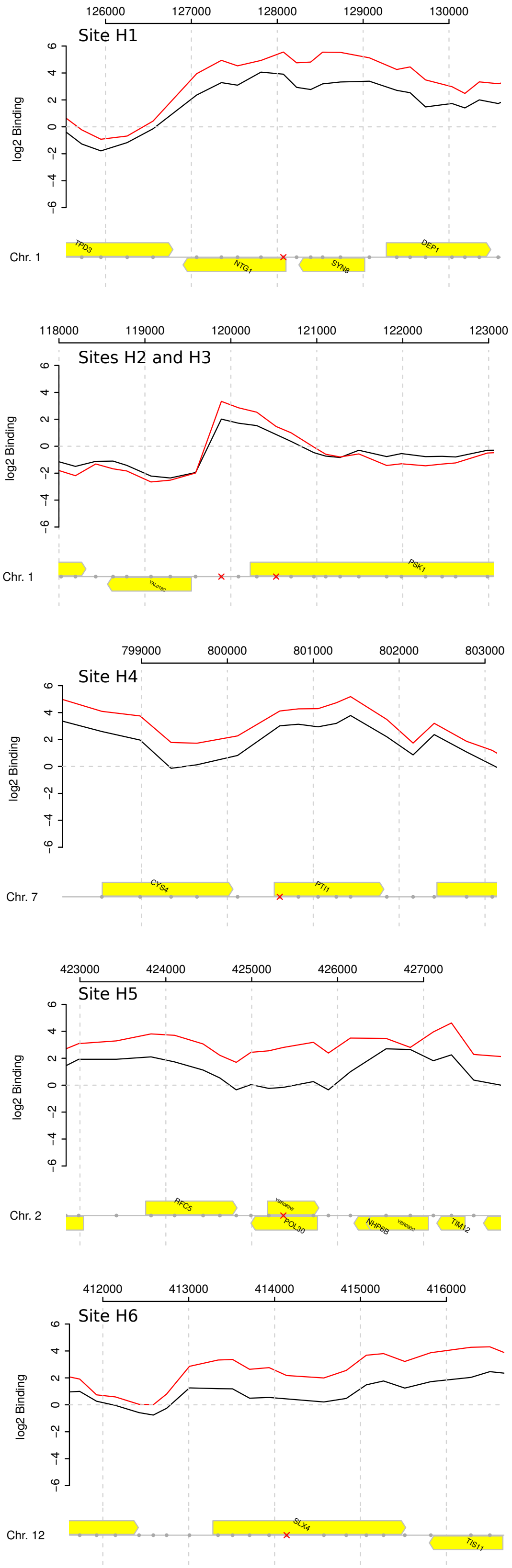

Supplementary Figure S9: Regions of H3Ac data selected for Q-PCR analysis. Replicate datasets are shown as different coloured lines. Red crosses indicate probes selected for analysis.

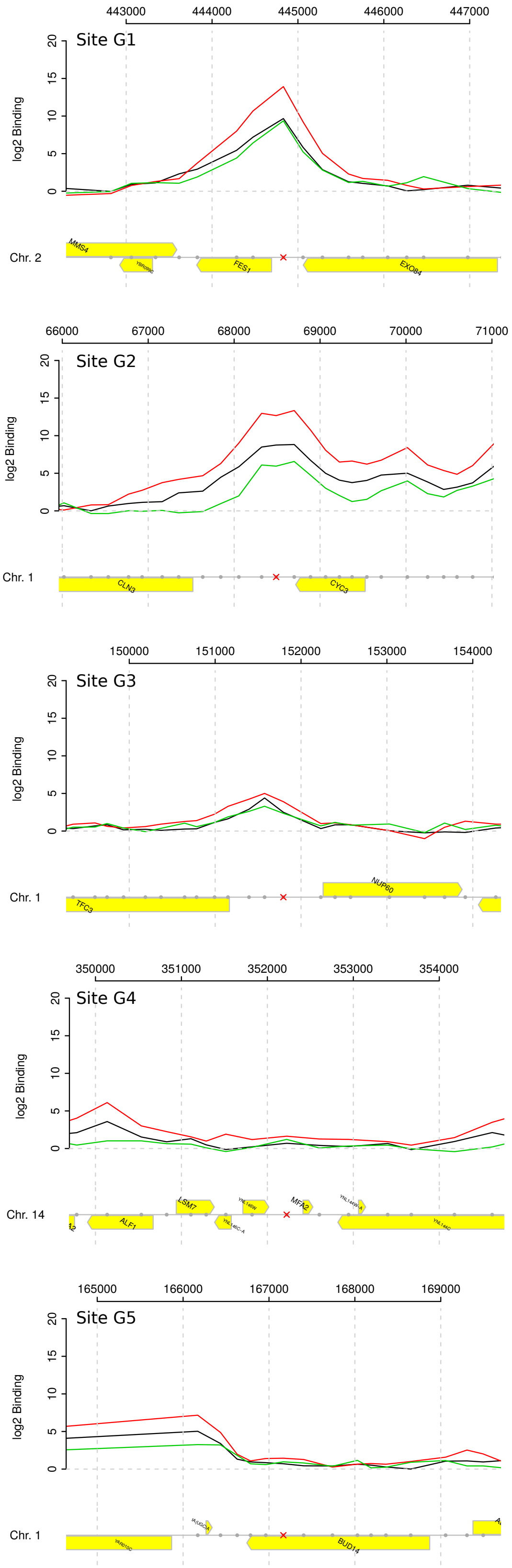

Supplementary Figure S10: Regions of Gcn5p binding data selected for Q-PCR analysis. Replicate datasets are shown as different coloured lines. Red crosses indicate probes selected for analysis.

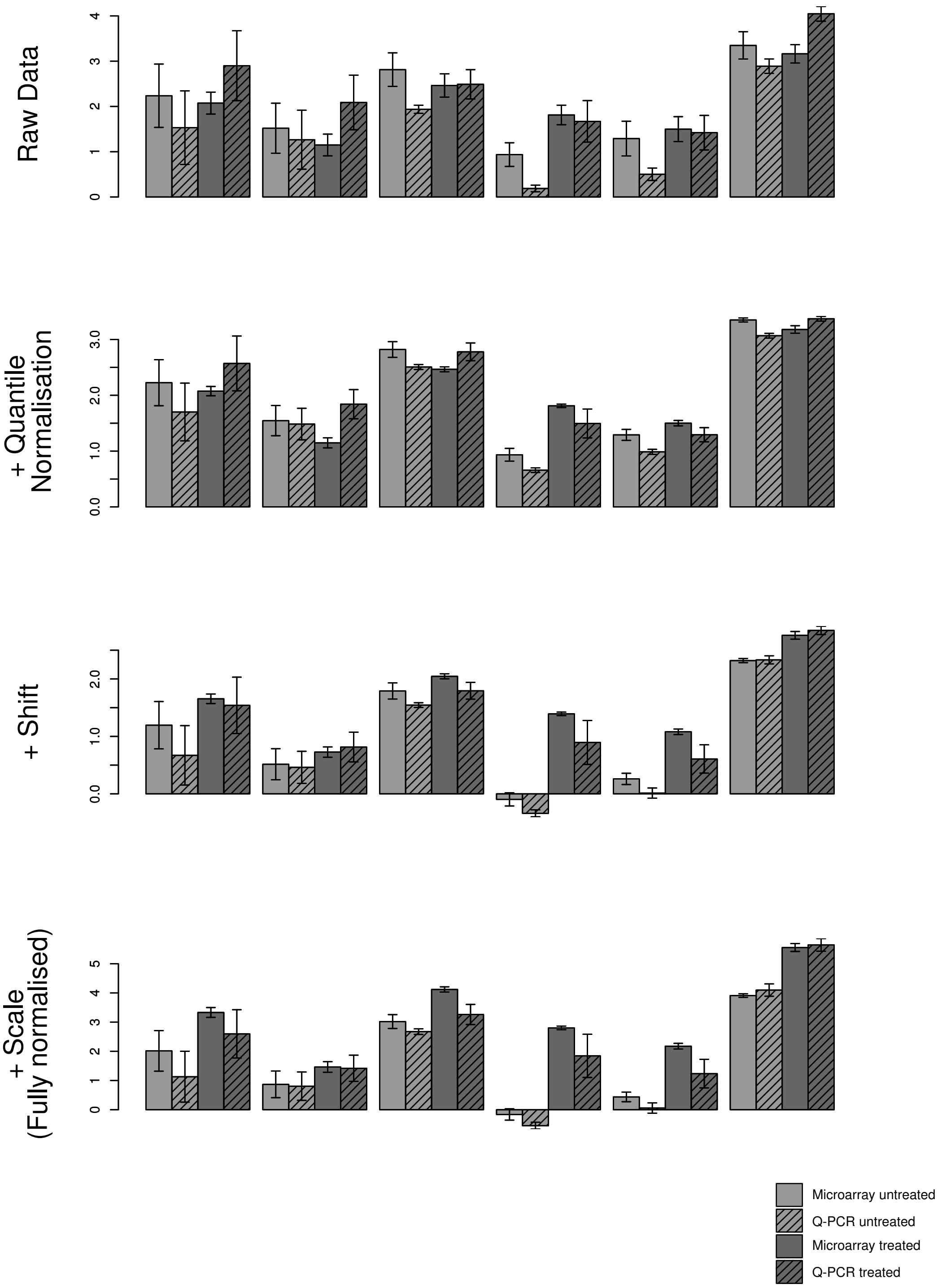

Supplementary Figure S11: Bar charts showing microarray and Q-PCR data at selected sites. H3Ac microarray (plain) and Q-PCR (shaded) data undergoing normalisation in untreated (light) and UV treated (dark) samples. Error bars show standard errors.

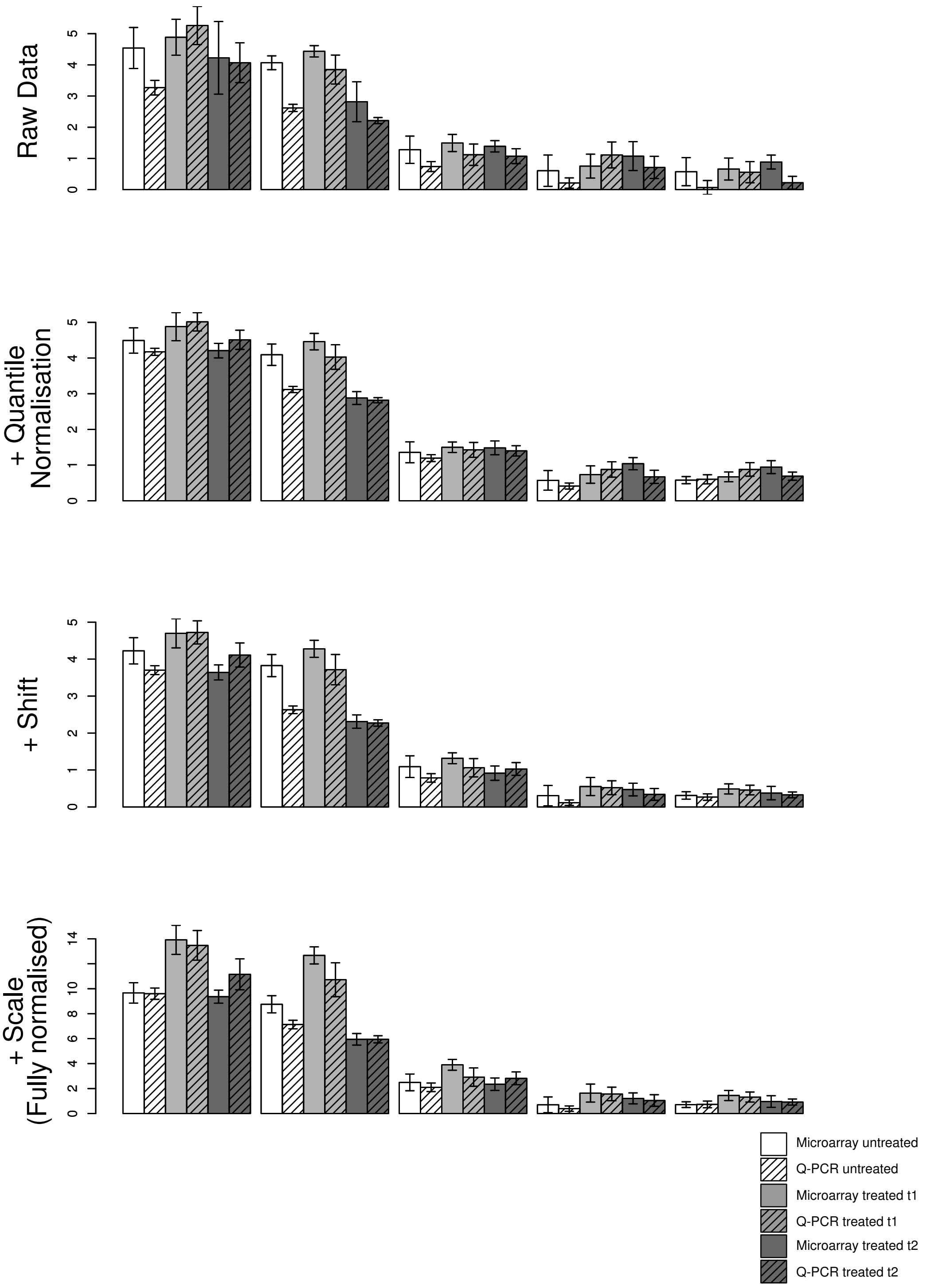

Supplementary Figure S12: textbfBar charts showing microarray and Q-PCR data at selected sites. Gcn5p binding microarray (plain) and Q-PCR (shaded) data undergoing normalisation in untreated (white) and UV treated t1 (light) t2 (dark) samples. Error bars show standard errors.

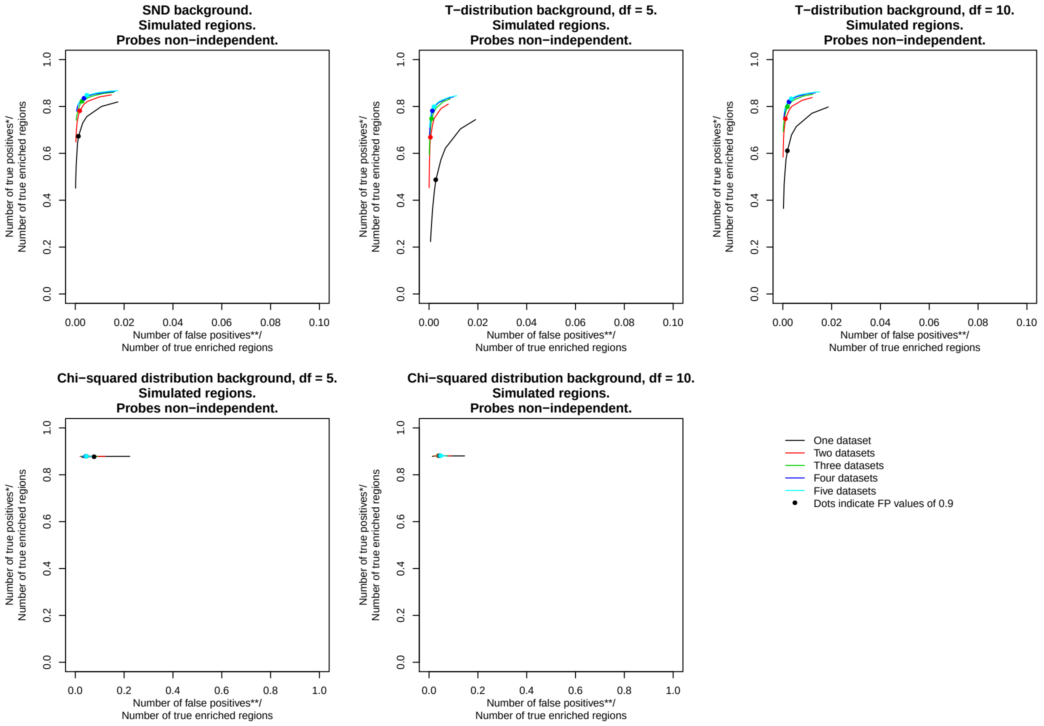

Supplementary Figure S13: Sensitivity and specificity of the Sandcastle EDM on simulated datasets. ROC-like curves showing the performance of the Sandcastle EDM at detecting simulated regions in datasets with varying background distributions. Coloured lines show the analysis of different numbers of datasets with results from varying FP values, each being the average of 50 simulations. Dots show the default FP value of 0.9. Higher specificity/sensitivity values are created when analysing multiple replicate datasets together than by analysing them individually. Even when simulating background distributions that violate the assumptions of the EDM high sensitivity and specificity values are generated. Results shown here are from data simulated with a degree of dependence between probe values in the same region, as may be expected in real data. *True positives are calculated as the number of simulated regions overlapping a detected region by 80% or more. **False positives are calculated as the number of detected regions not overlapping an enriched region by 80% or more.

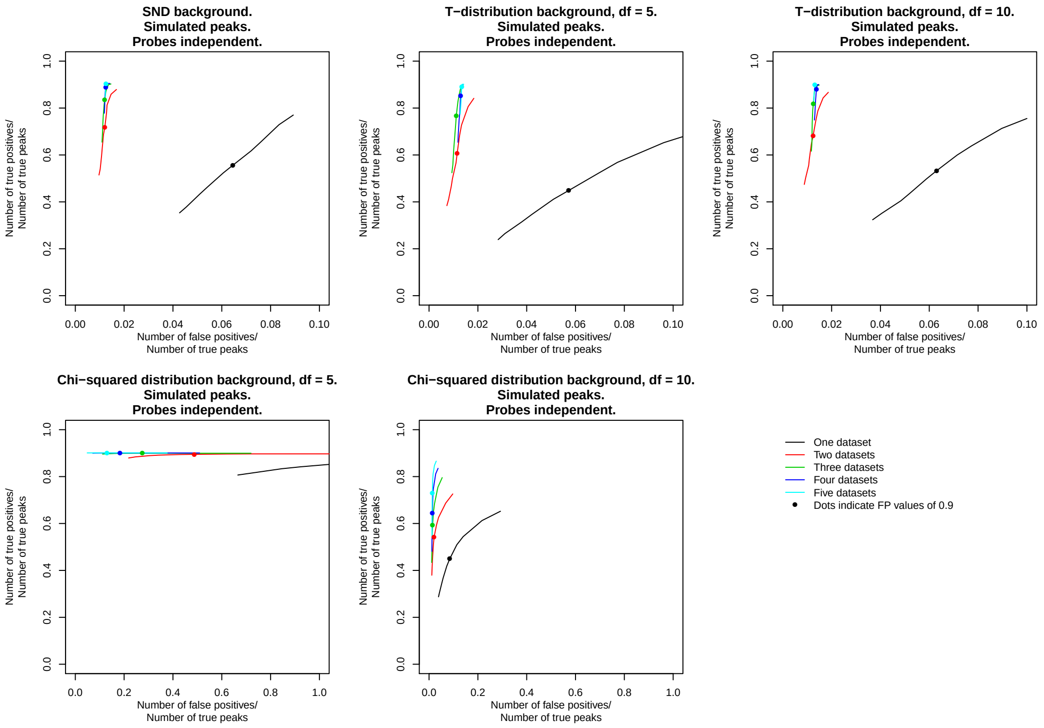

Supplementary Figure S14: Sensitivity and specificity of the Sandcastle EDM on simulated datasets. ROC-like curves showing the performance of the Sandcastle EDM at detecting simulated peaks in datasets with varying background distributions. Coloured lines show the analysis of different numbers of datasets with results from varying FP values, each being the average of 50 simulations. Dots show the default FP value of 0.9. Higher specificity/sensitivity values are created when analysing multiple replicate datasets together than by analysing them individually. Even when simulating background distributions that violate the assumptions of the EDM high sensitivity and specificity values are generated. Results shown here are from data simulated with no degree of dependence between probe values, which may not be expected in real data.

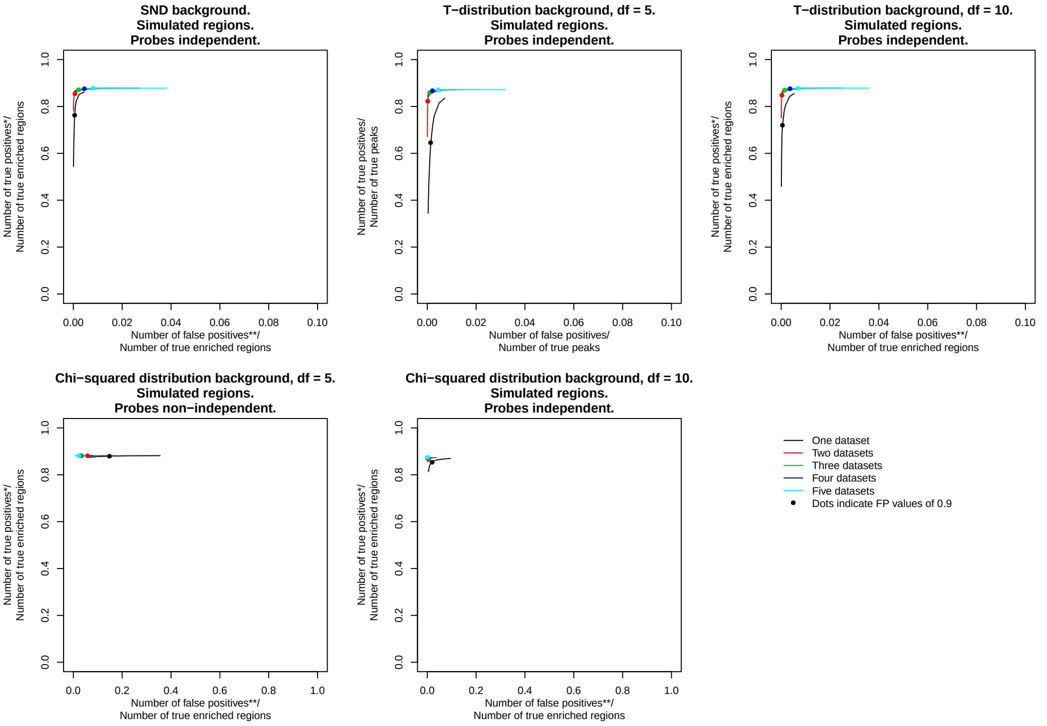

Supplementary Figure S15: Sensitivity and specificity of the Sandcastle EDM on simulated datasets. ROC-like curves showing the performance of the Sandcastle EDM at detecting simulated regions in datasets with varying background distributions. Coloured lines show the analysis of different numbers of datasets with results from varying FP values, each being the average of 50 simulations. Dots show the default FP value of 0.9. Higher specificity/sensitivity values are created when analysing multiple replicate datasets together than by analysing them individually. Even when simulating background distributions that violate the assumptions of the EDM high sensitivity and specificity values are generated. Results shown here are from data simulated with no degree of dependence between probe values, which may not be expected in real data. *True positives are calculated as the number of simulated regions overlapping a detected region by 80% or more. **False positives are calculated as the number of detected regions not overlapping an enriched region by 80% or more.

Dataset	Description
Gcn5 U	Gcn5 enrichment over the whole genome in cells not treated with UV. The three line colours show three replicate datasets. The red crosses show probes detected as enriched.
Gcn5 T1	Gcn5 enrichment over the whole genome in cells treated with UV, time point 1. The three line colours show three replicate datasets. The red crosses show probes detected as enriched.
Gcn5 T2	Gcn5 enrichment over the whole genome in cells treated with UV, time point 2. The three line colours show three replicate datasets. The red crosses show probes detected as enriched.
Gcn5 comparison	Gcn5 enrichment over the whole genome. Black line shows average of untreated datasets. Red line shows average of T1 datasets. Green line shows average of T2 datasets.
H3Ac U	H3Ac enrichment over the whole genome in cells not treated with UV. The five line colours show five replicate datasets. The red crosses show probes detected as enriched.
H3Ac T	H3Ac enrichment over the whole genome in cells treated with UV. The five line colours show five replicate datasets. The red crosses show probes detected as enriched.
H3Ac comparison	H3Ac enrichment over the whole genome. Black line shows average of untreated datasets. Red line shows average of treated datasets.
Abf1 U	Abf1 enrichment over the whole genome in cells not treated with UV. The three line colours show three replicate datasets. The red crosses show probes detected as enriched.

Supplementary Table S2: Key for additional supplementary figures, shown in the following pages.

The following plots show Gcn5 enrichment over the whole genome in cells not treated with UV. The three line colours show three replicate datasets. The red crosses show probes detected as enriched.

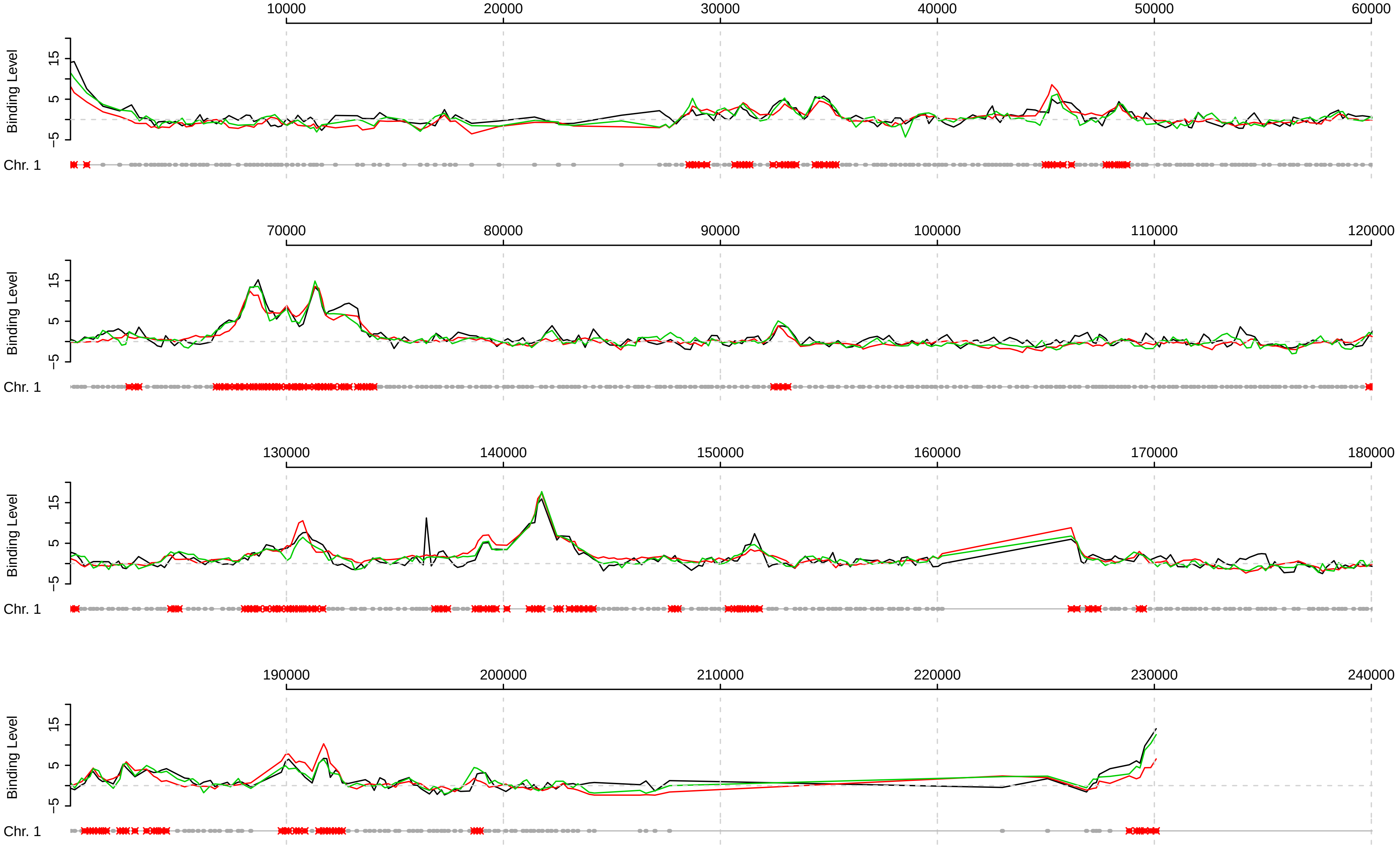

10000
20000
30000
40000
50000
60000
70000
80000
90000
100000
110000
120000
130000
140000
150000
160000
170000
180000
190000
200000
210000
220000
230000
240000
Binding Level
−5
5
15
Chr. 1

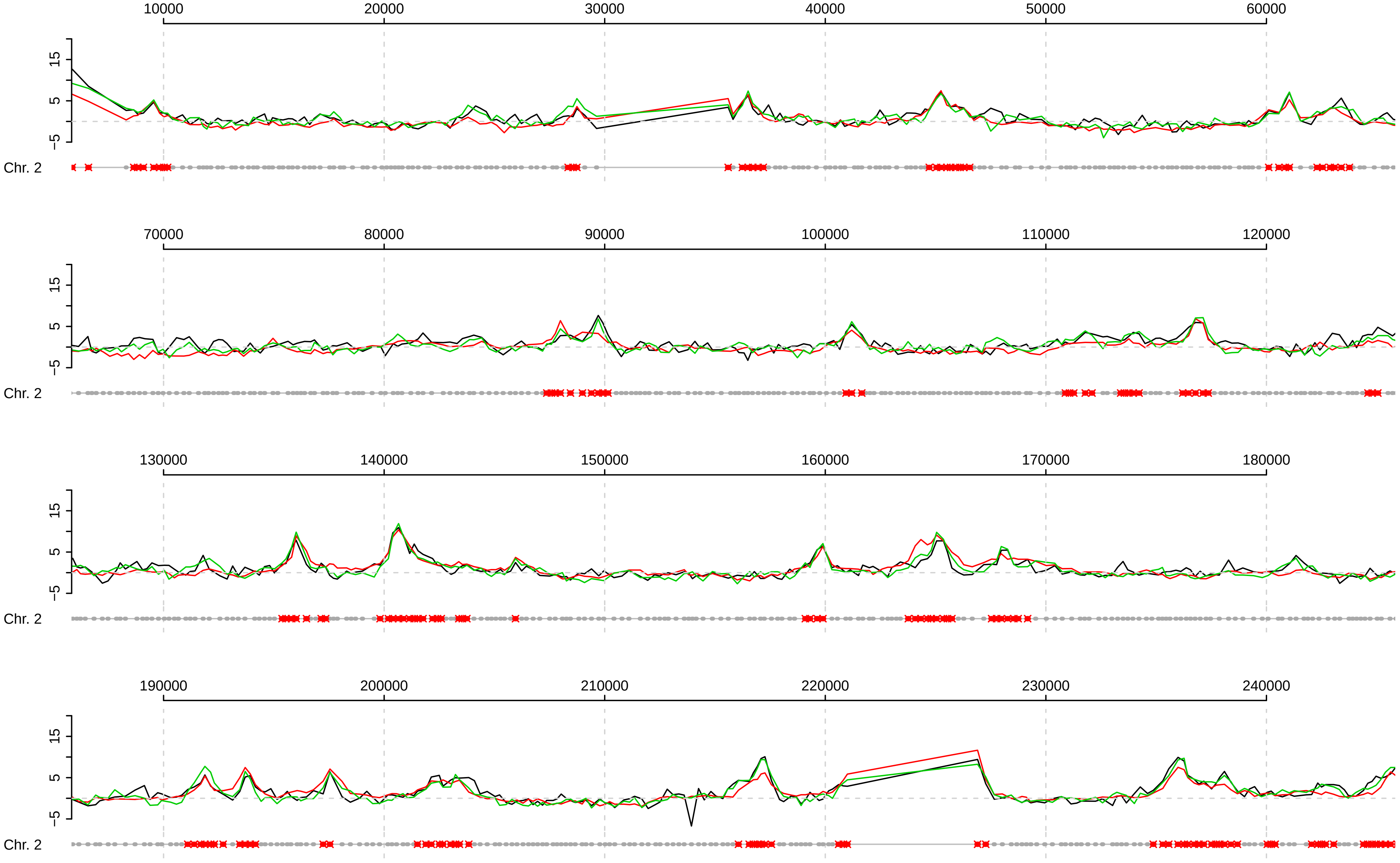

10000
20000
30000
40000
50000
60000
70000
80000
90000
100000
110000
120000
130000
140000
150000
160000
170000
180000
190000
200000
210000
220000
230000
240000
−5
5
15
Chr. 2

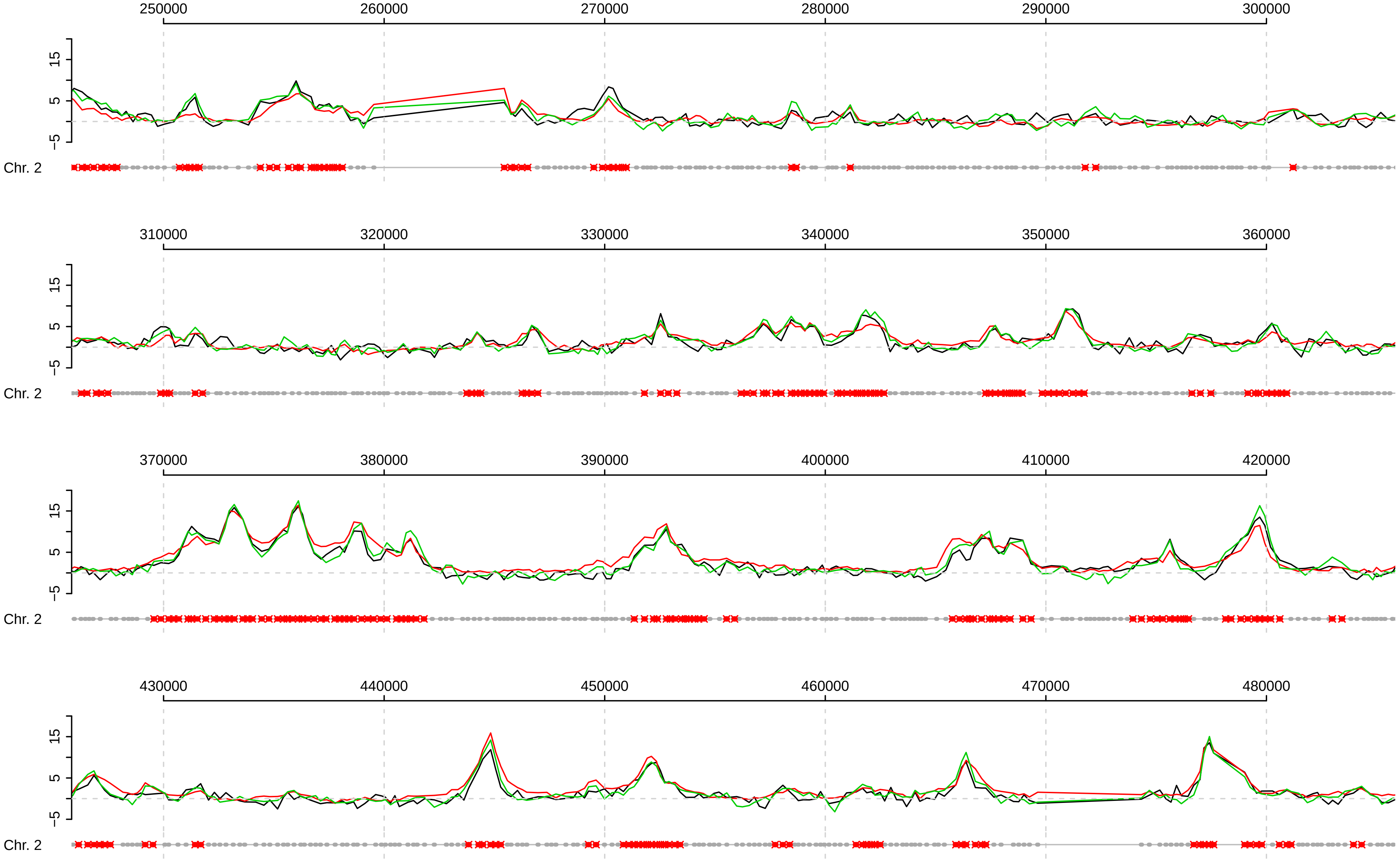

250000
260000
270000
280000
290000
300000
15
5
−5
Chr. 2
310000
320000
330000
340000
350000
360000
15
5
−5
Chr. 2
370000
380000
390000
400000
410000
420000
15
5
−5
Chr. 2
430000
440000
450000
460000
470000
480000
15
5
−5
Chr. 2

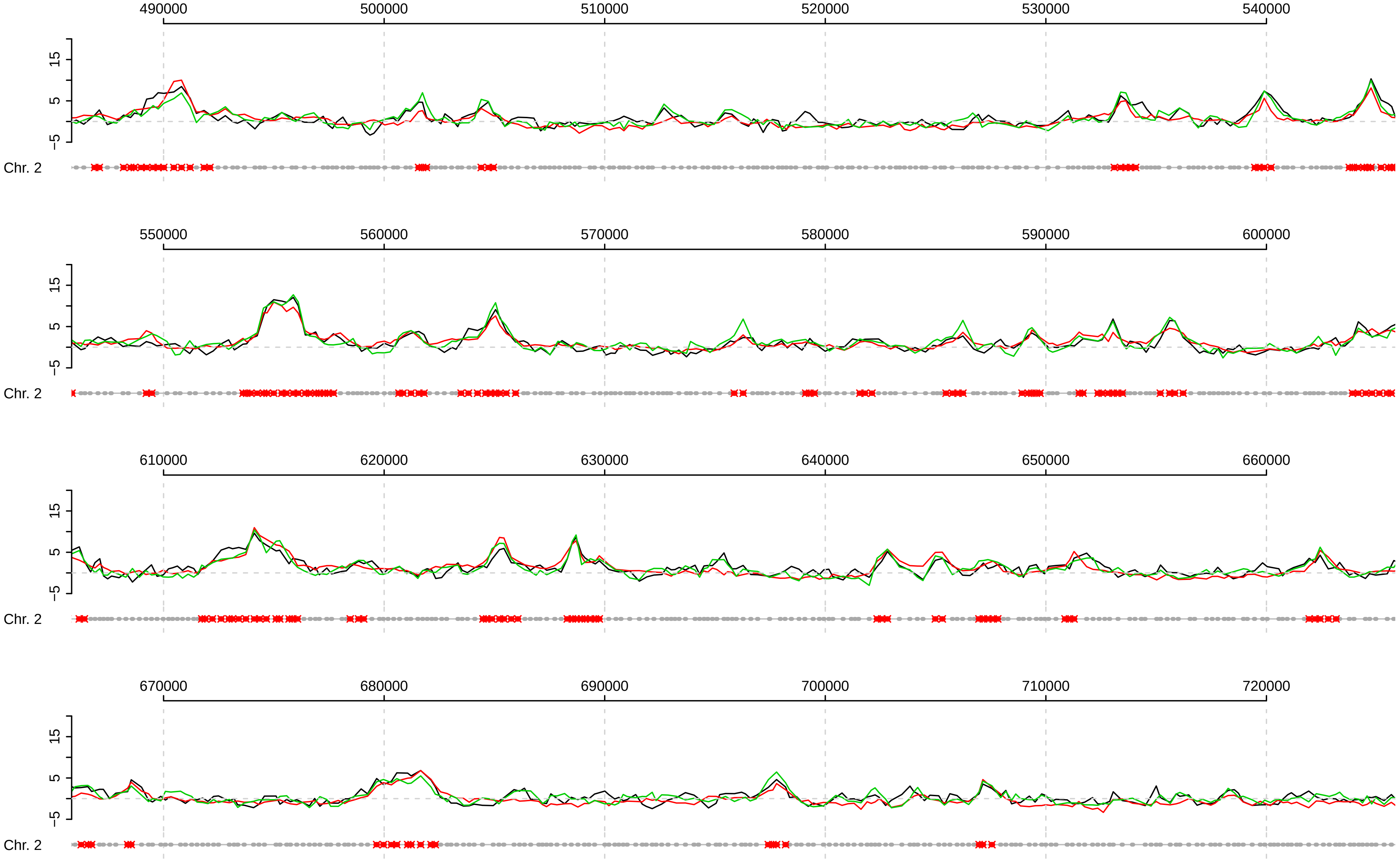

490000
500000
510000
520000
530000
540000
15
5
−5
Chr. 2
550000
560000
570000
580000
590000
600000
15
5
−5
Chr. 2
610000
620000
630000
640000
650000
660000
15
5
−5
Chr. 2
670000
680000
690000
700000
710000
720000
15
5
−5
Chr. 2

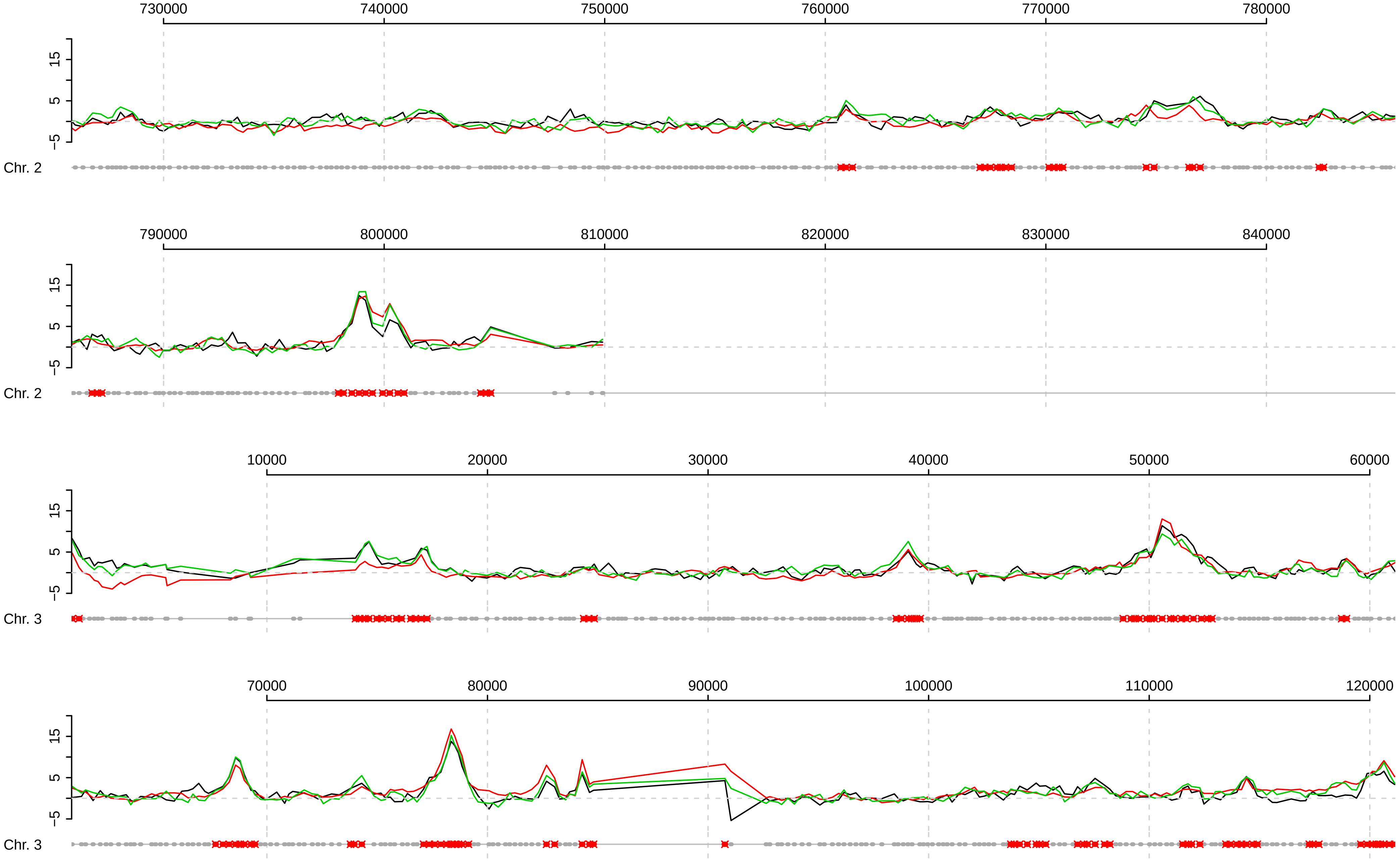

730000
740000
750000
760000
770000
780000
15
5
−5
Chr. 2
790000
800000
810000
820000
830000
840000
15
5
−5
Chr. 2
10000
20000
30000
40000
50000
60000
15
5
−5
Chr. 3
70000
80000
90000
100000
110000
120000
15
5
−5
Chr. 3

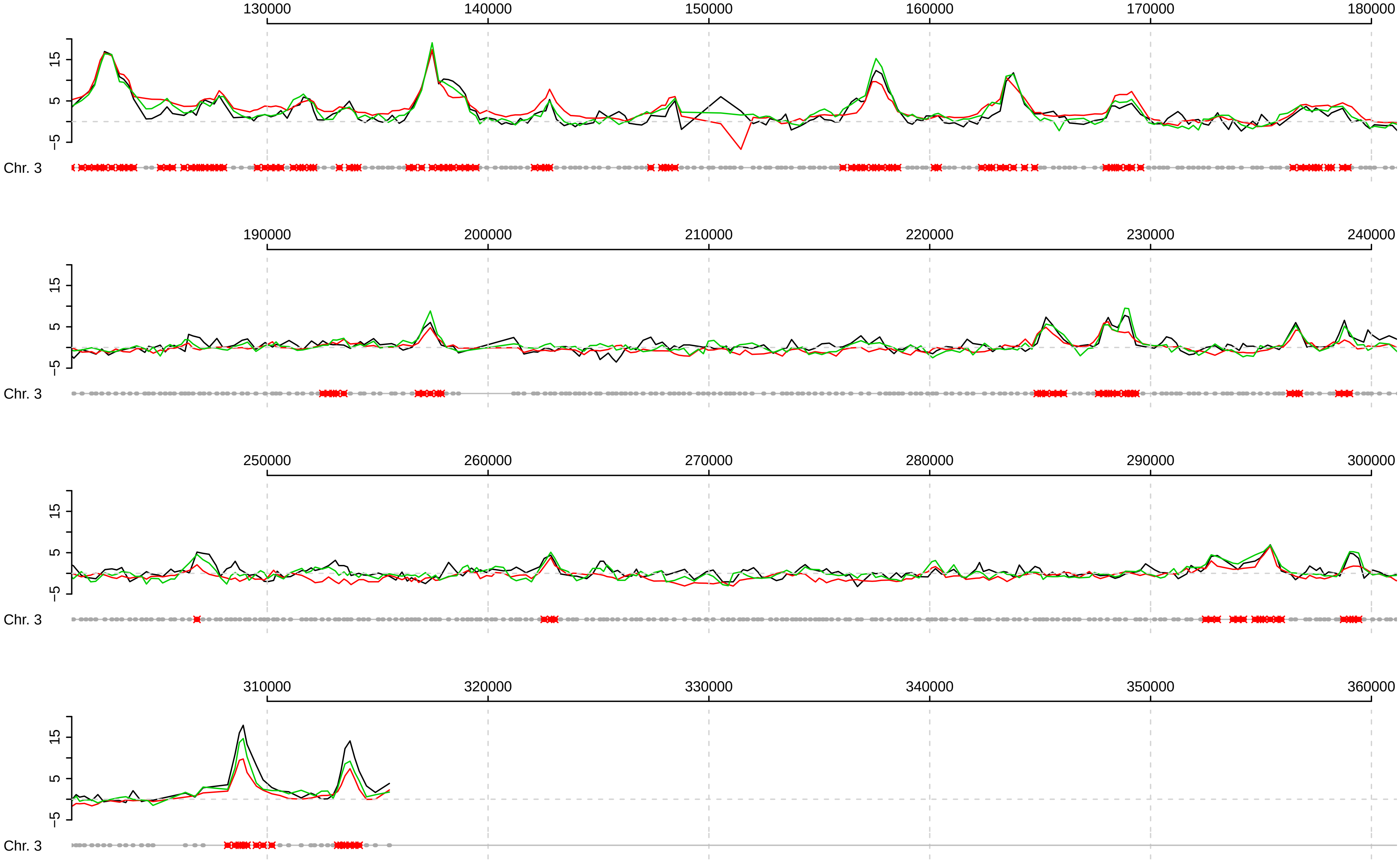

130000
140000
150000
160000
170000
180000
15
5
−5
Chr. 3
190000
200000
210000
220000
230000
240000
15
5
−5
Chr. 3
250000
260000
270000
280000
290000
300000
15
5
−5
Chr. 3
310000
320000
330000
340000
350000
360000
15
5
−5
Chr. 3

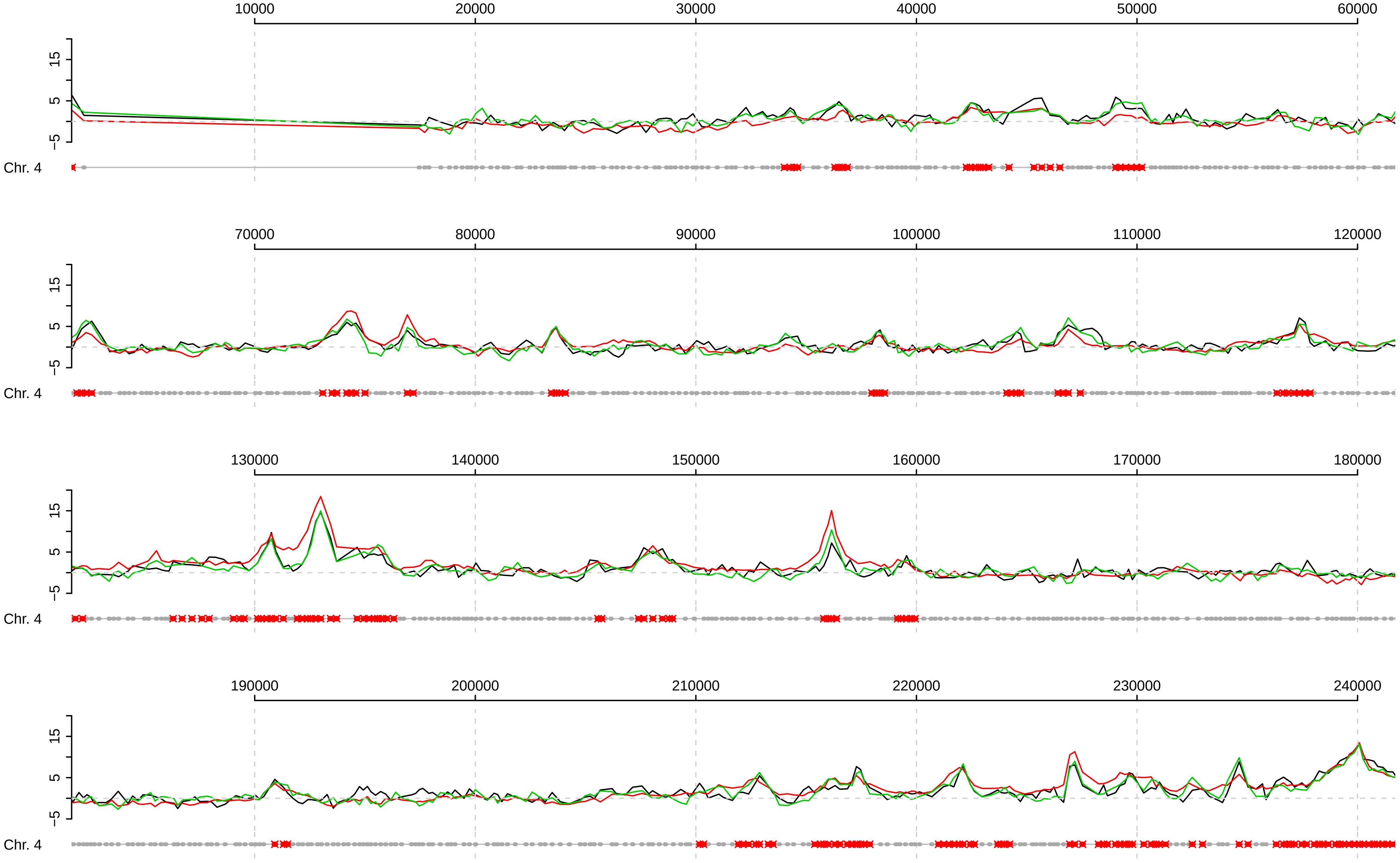
10000
20000
30000
40000
50000
60000
70000
80000
90000
100000
110000
120000
130000
140000
150000
160000
170000
180000
190000
200000
210000
220000
230000
240000
−5
5
15
Chr. 4

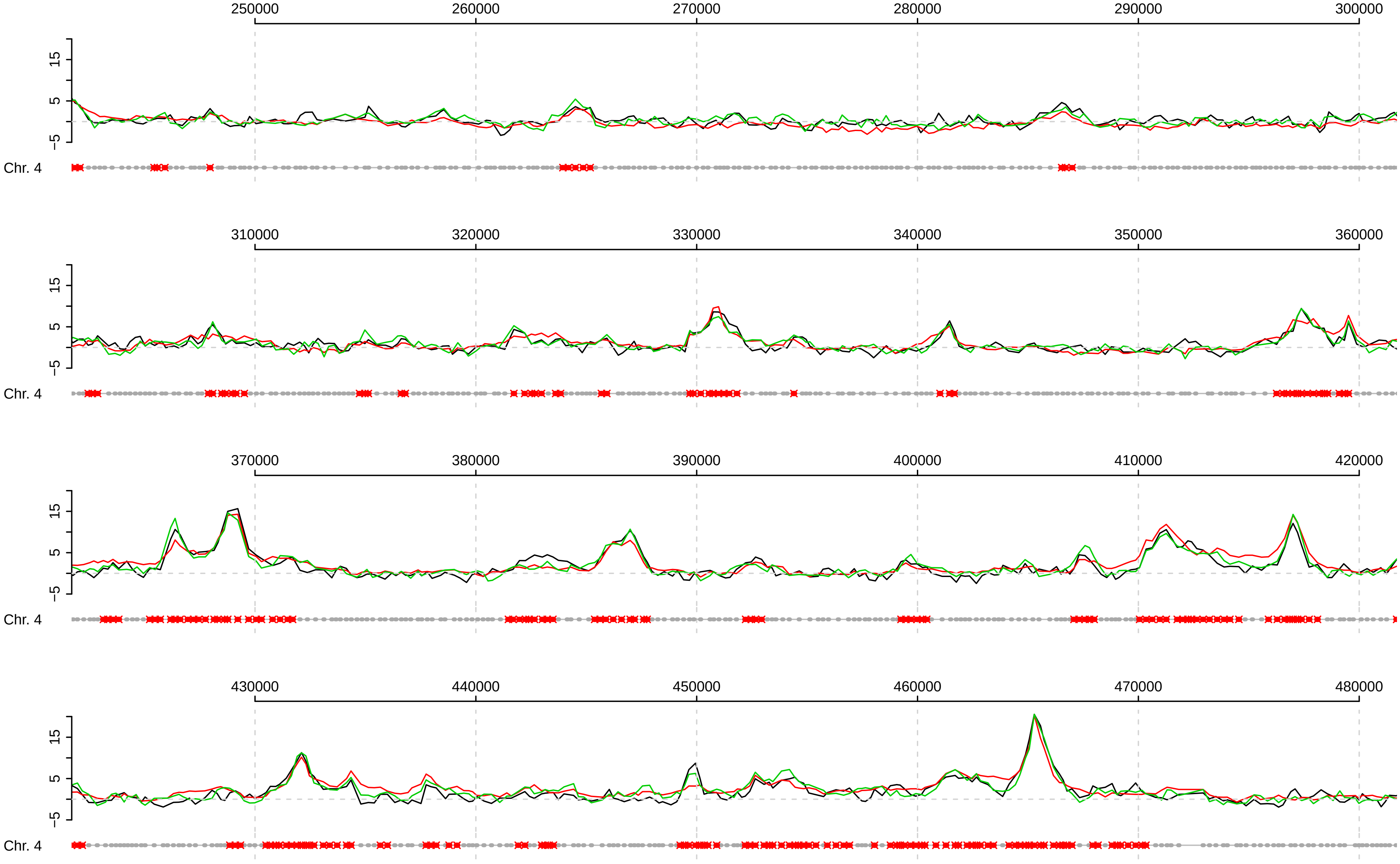

250000
260000
270000
280000
290000
300000
15
5
−5
Chr. 4
310000
320000
330000
340000
350000
360000
15
5
−5
Chr. 4
370000
380000
390000
400000
410000
420000
15
5
−5
Chr. 4
430000
440000
450000
460000
470000
480000
15
5
−5
Chr. 4

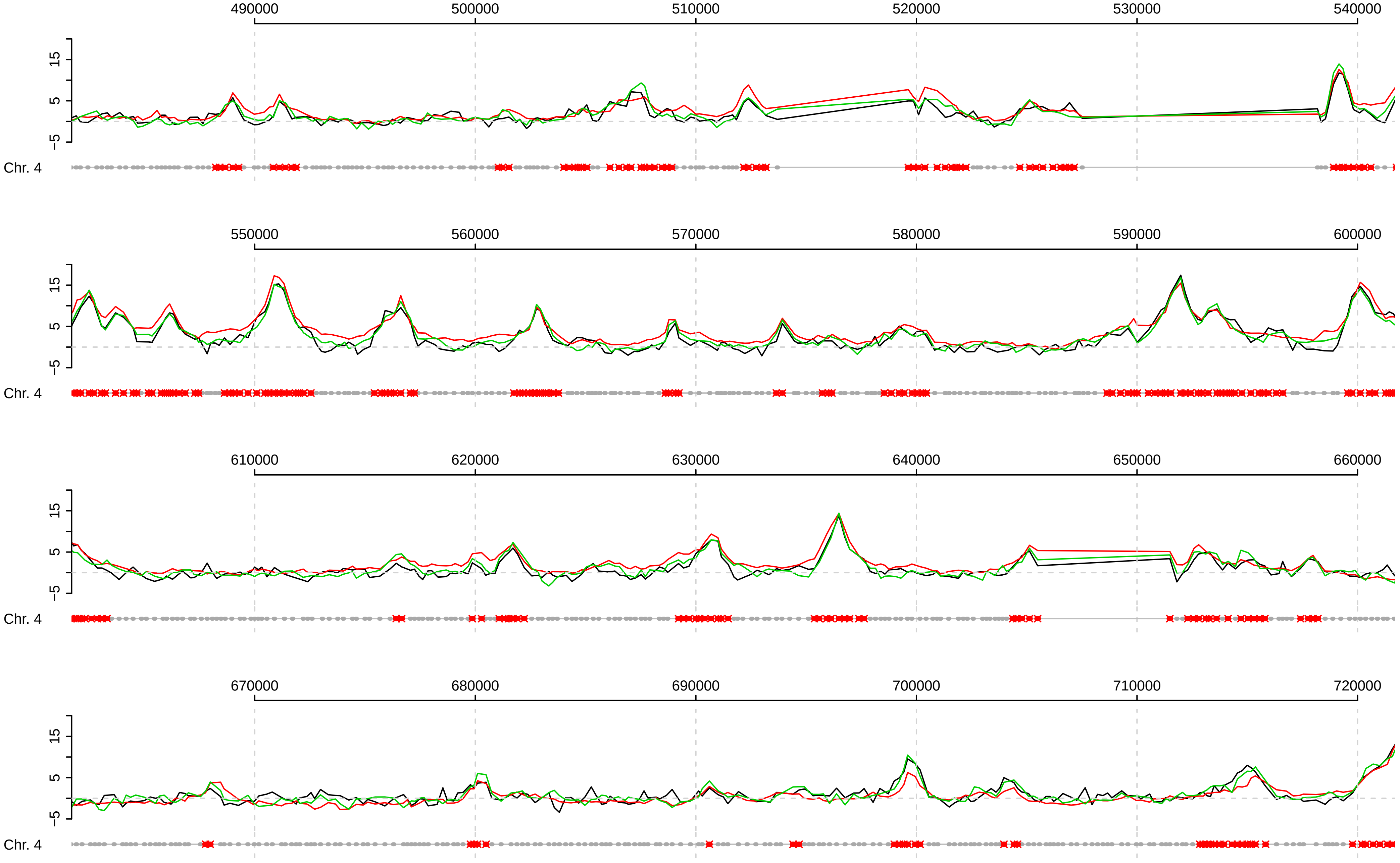
490000
500000
510000
520000
530000
540000
15
5
−5
Chr. 4
550000
560000
570000
580000
590000
600000
15
5
−5
Chr. 4
610000
620000
630000
640000
650000
660000
15
5
−5
Chr. 4
670000
680000
690000
700000
710000
720000
15
5
−5
Chr. 4

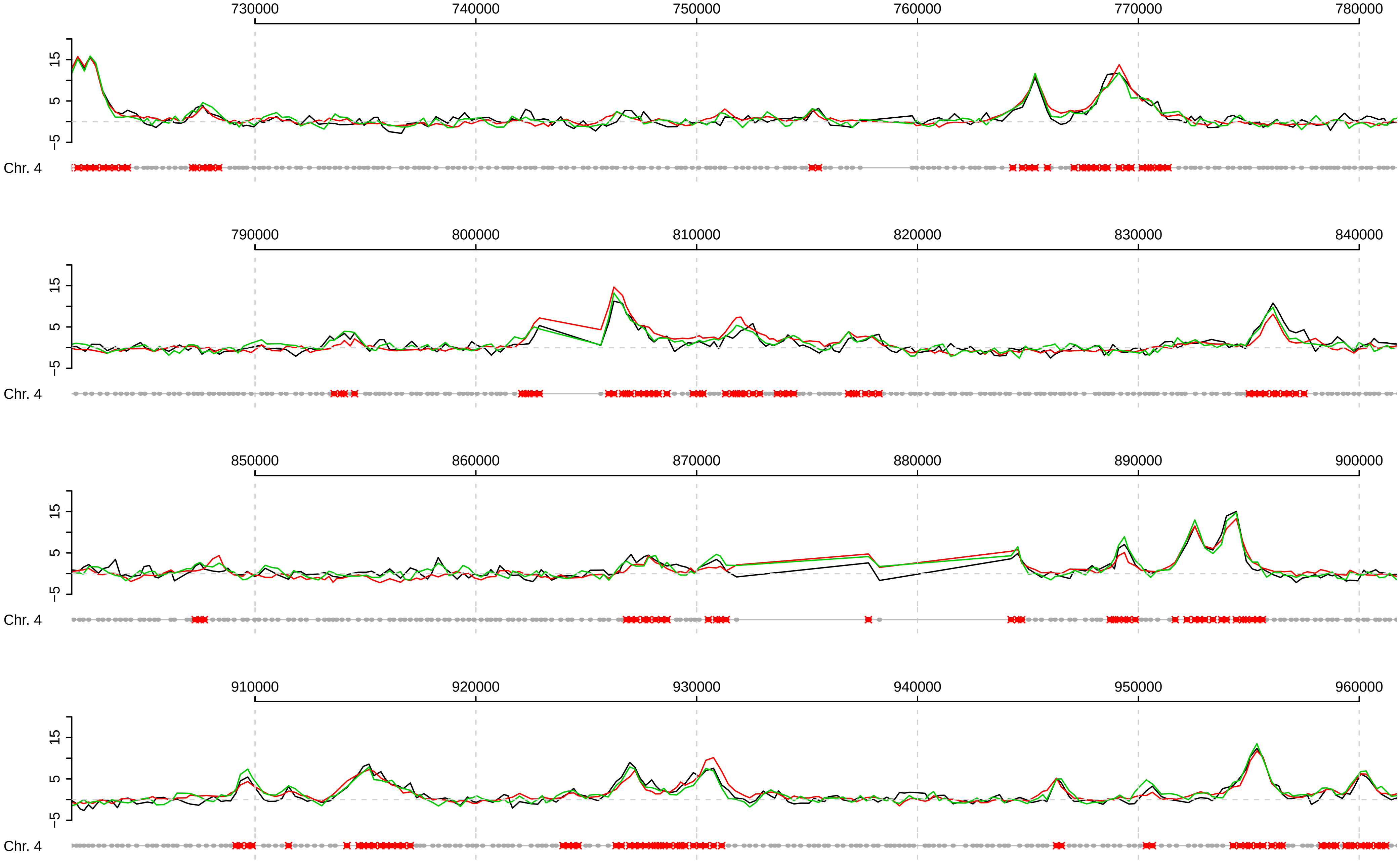

730000
740000
750000
760000
770000
780000
15
5
−5
Chr. 4
790000
800000
810000
820000
830000
840000
15
5
−5
Chr. 4
850000
860000
870000
880000
890000
900000
15
5
−5
Chr. 4
910000
920000
930000
940000
950000
960000
15
5
−5
Chr. 4

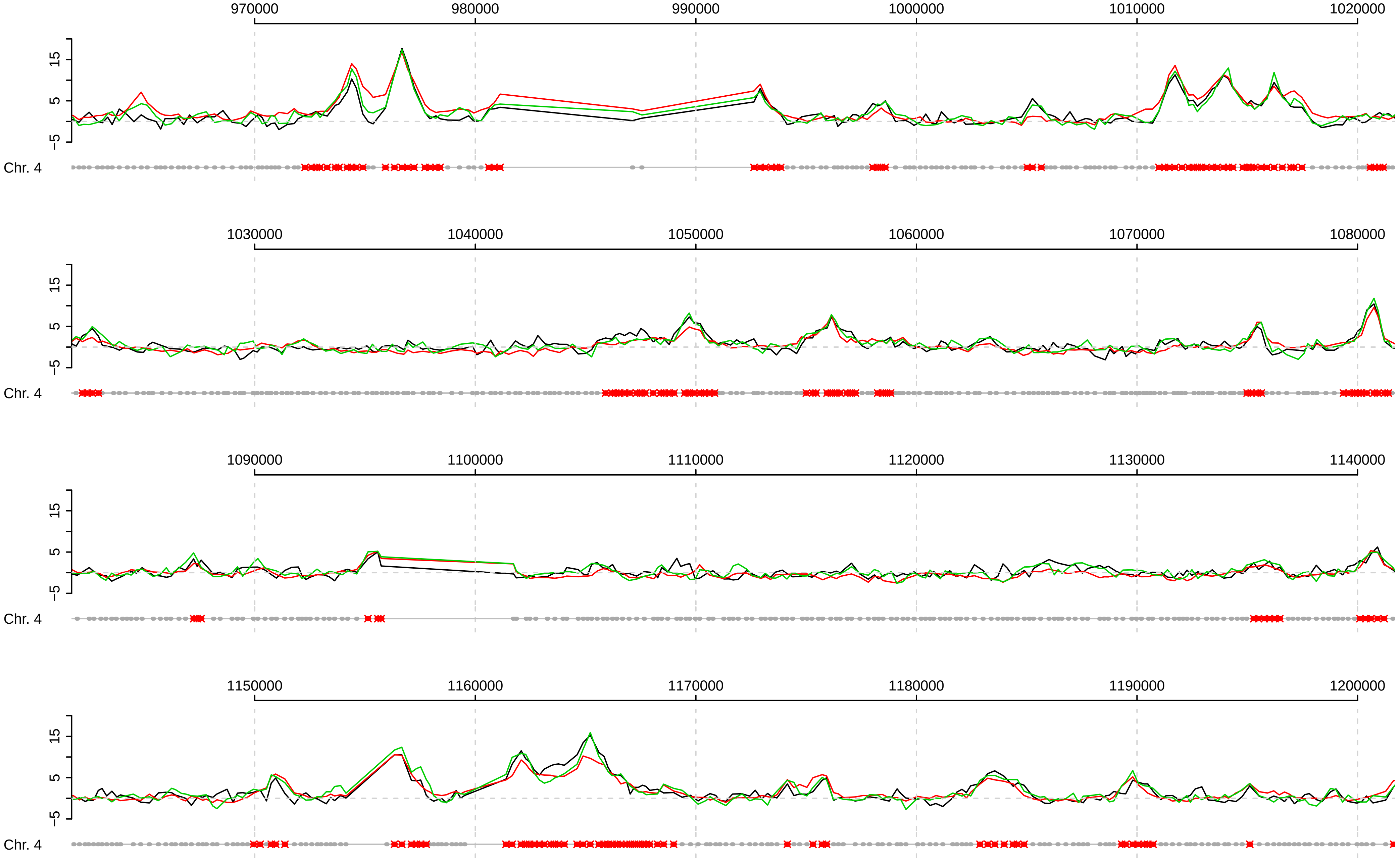
970000
980000
990000
1000000
1010000
1020000
1030000
1040000
1050000
1060000
1070000
1080000
1090000
1100000
1110000
1120000
1130000
1140000
1150000
1160000
1170000
1180000
1190000
1200000
15
5
−5
Chr. 4

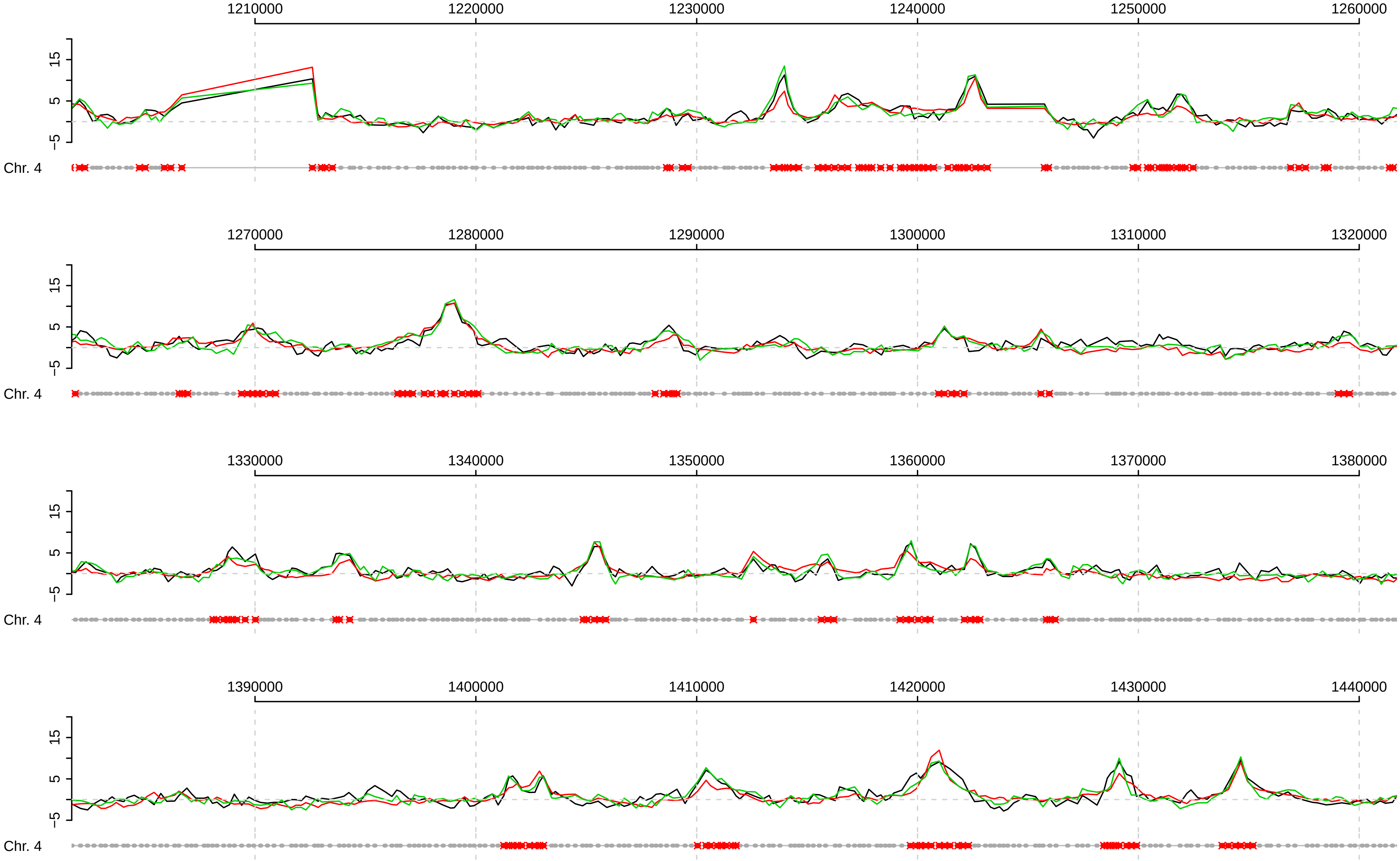

1210000
1220000
1230000
1240000
1250000
1260000
15
5
−5
Chr. 4
1270000
1280000
1290000
1300000
1310000
1320000
15
5
−5
Chr. 4
1330000
1340000
1350000
1360000
1370000
1380000
15
5
−5
Chr. 4
1390000
1400000
1410000
1420000
1430000
1440000
15
5
−5
Chr. 4

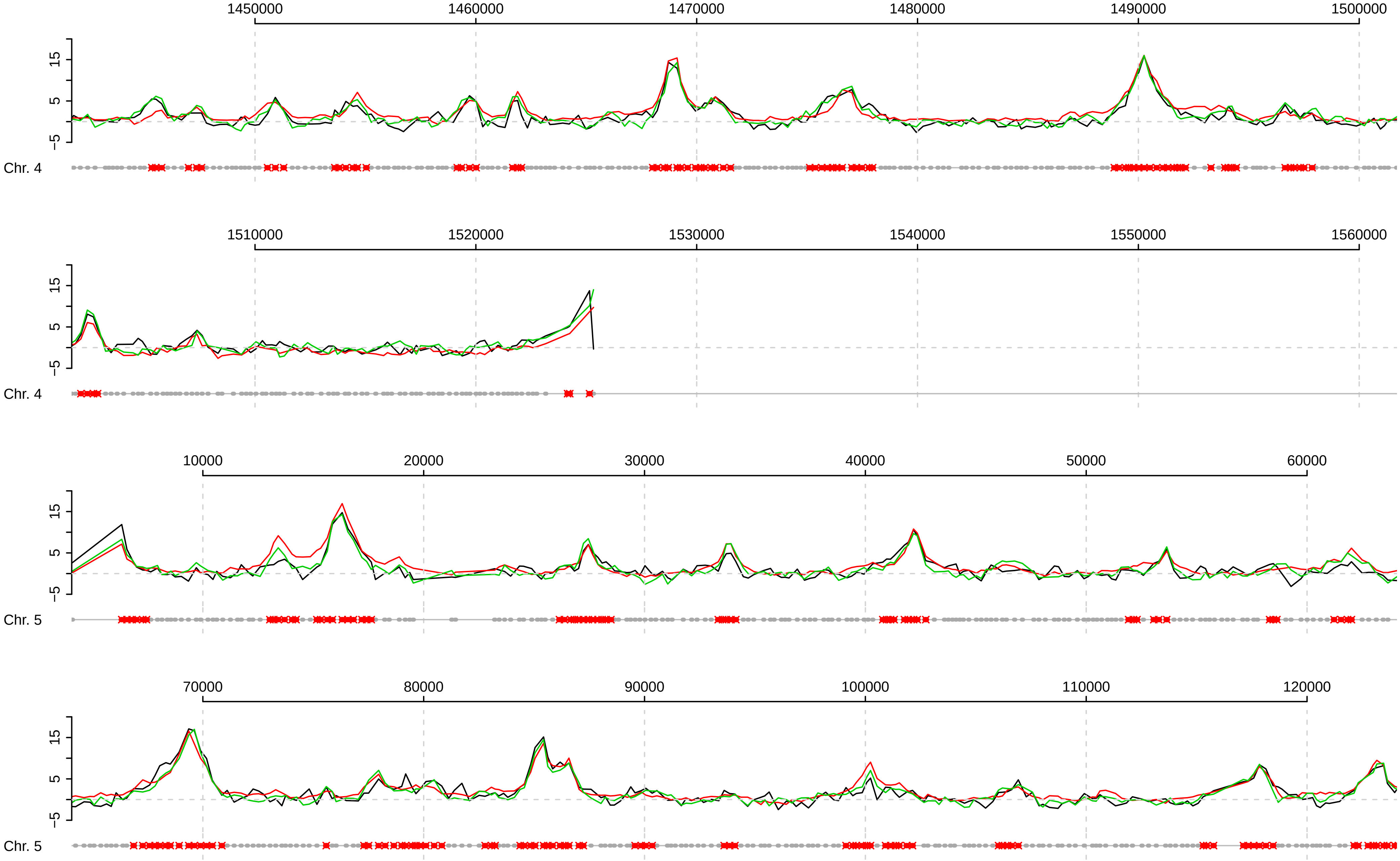
1450000
1460000
1470000
1480000
1490000
1500000
15
5
−5
Chr. 4
1510000
1520000
1530000
1540000
1550000
1560000
15
5
−5
Chr. 4
10000
20000
30000
40000
50000
60000
15
5
−5
Chr. 5
70000
80000
90000
100000
110000
120000
15
5
−5
Chr. 5

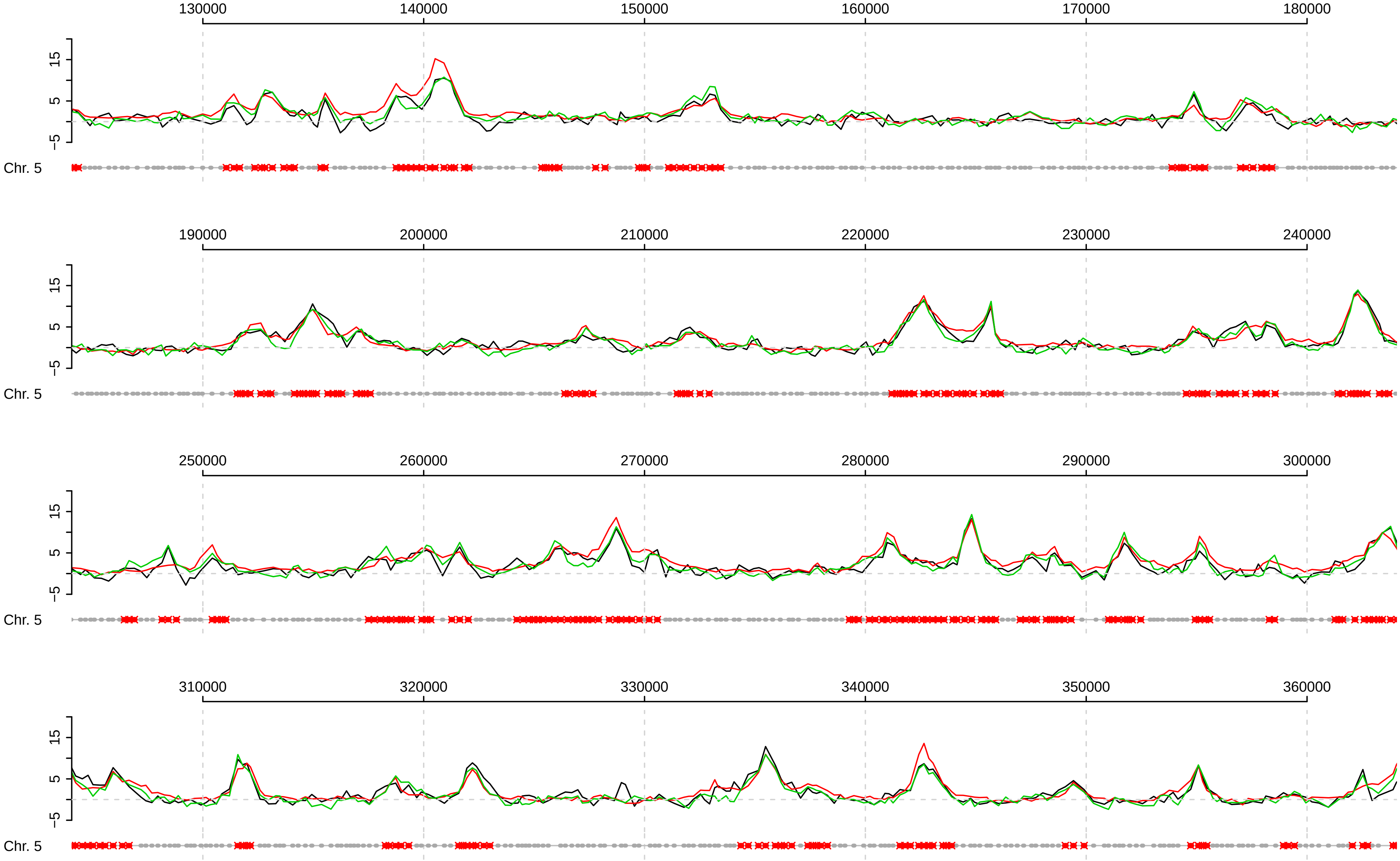

130000
140000
150000
160000
170000
180000
15
5
−5
Chr. 5
190000
200000
210000
220000
230000
240000
15
5
−5
Chr. 5
250000
260000
270000
280000
290000
300000
15
5
−5
Chr. 5
310000
320000
330000
340000
350000
360000
15
5
−5
Chr. 5

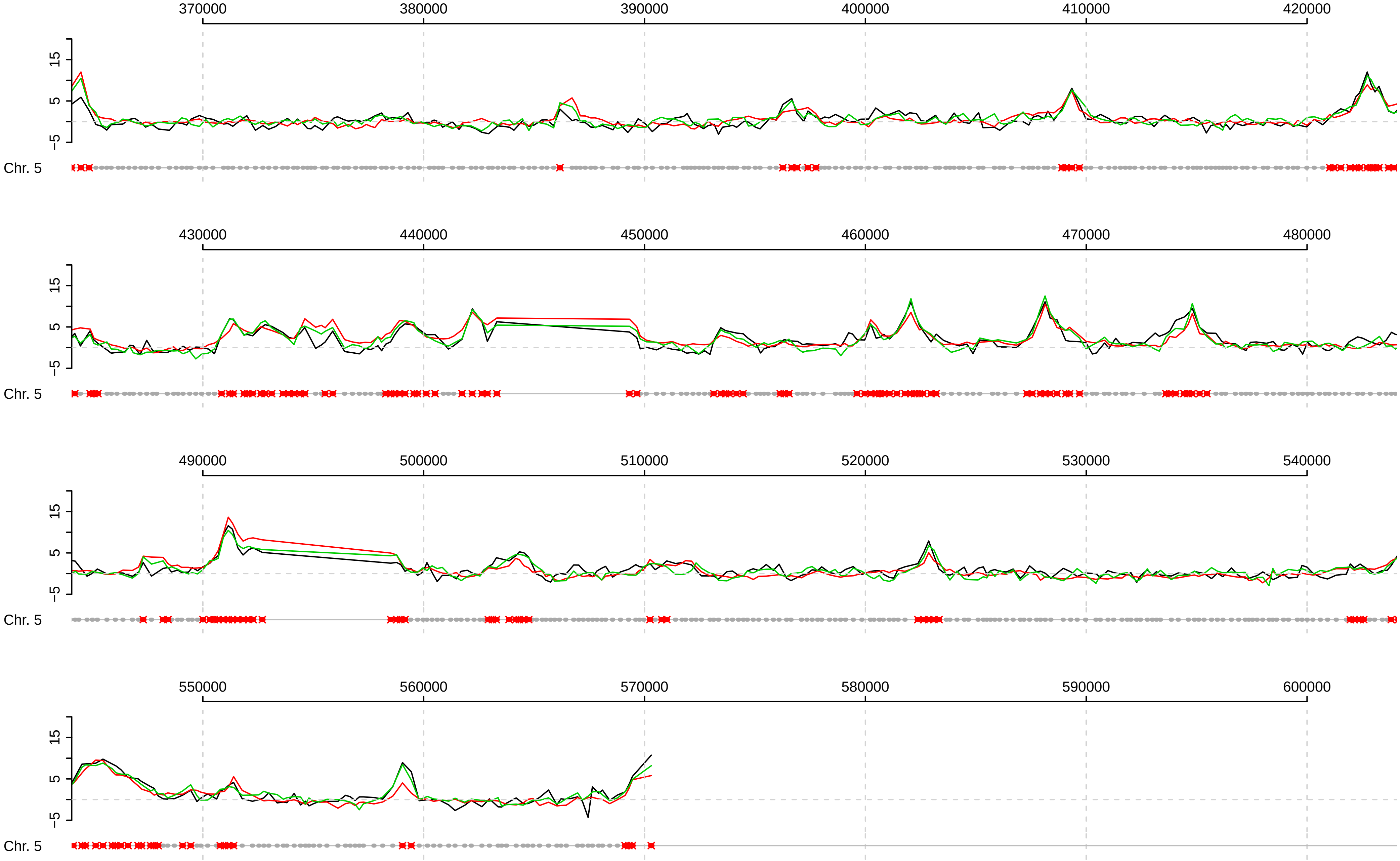
370000
380000
390000
400000
410000
420000
15
5
−5
Chr. 5
430000
440000
450000
460000
470000
480000
15
5
−5
Chr. 5
490000
500000
510000
520000
530000
540000
15
5
−5
Chr. 5
550000
560000
570000
580000
590000
600000
15
5
−5
Chr. 5

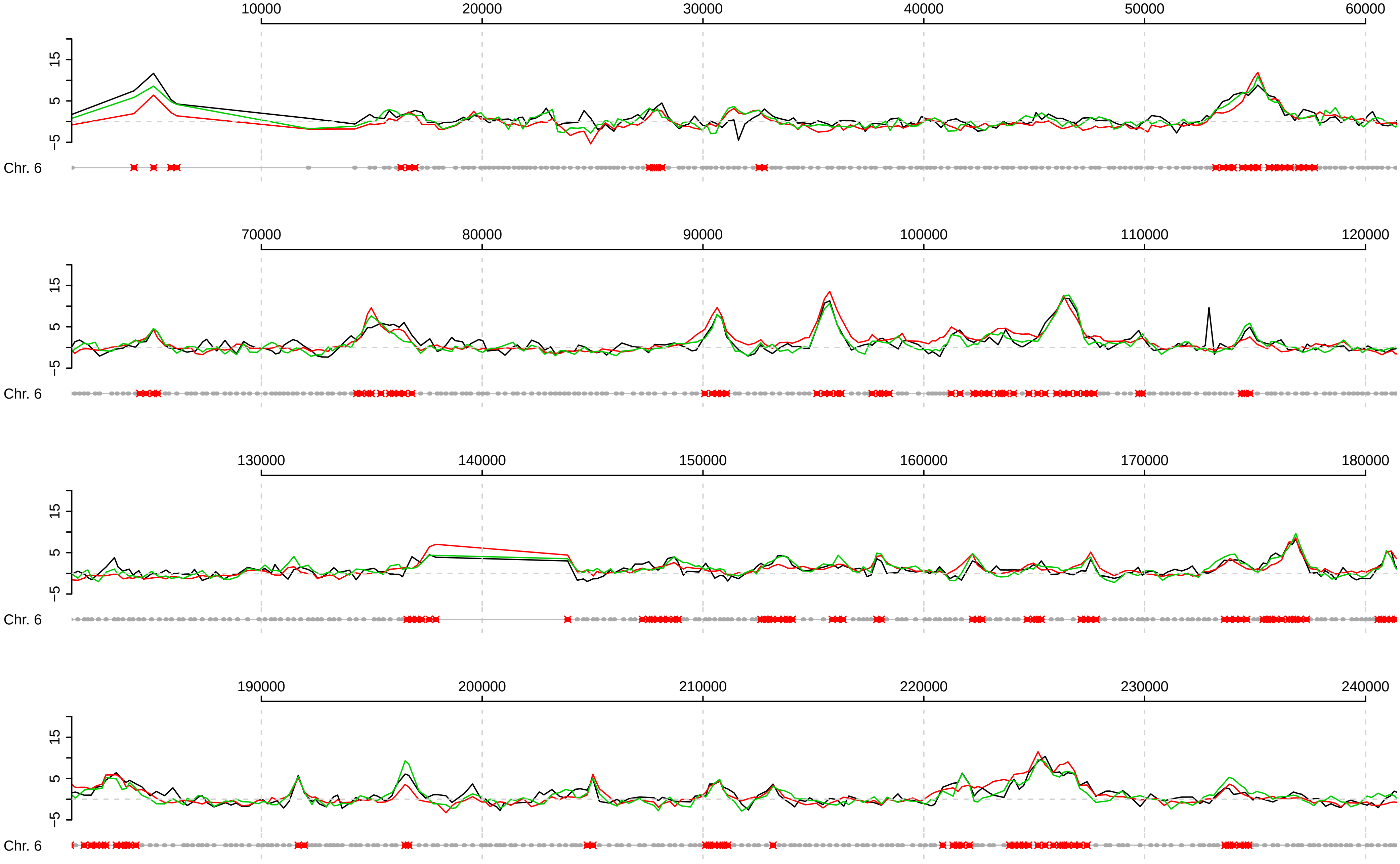

10000
20000
30000
40000
50000
60000
15
5
−5
Chr. 6
70000
80000
90000
100000
110000
120000
Chr. 6
130000
140000
150000
160000
170000
180000
Chr. 6
190000
200000
210000
220000
230000
240000
Chr. 6

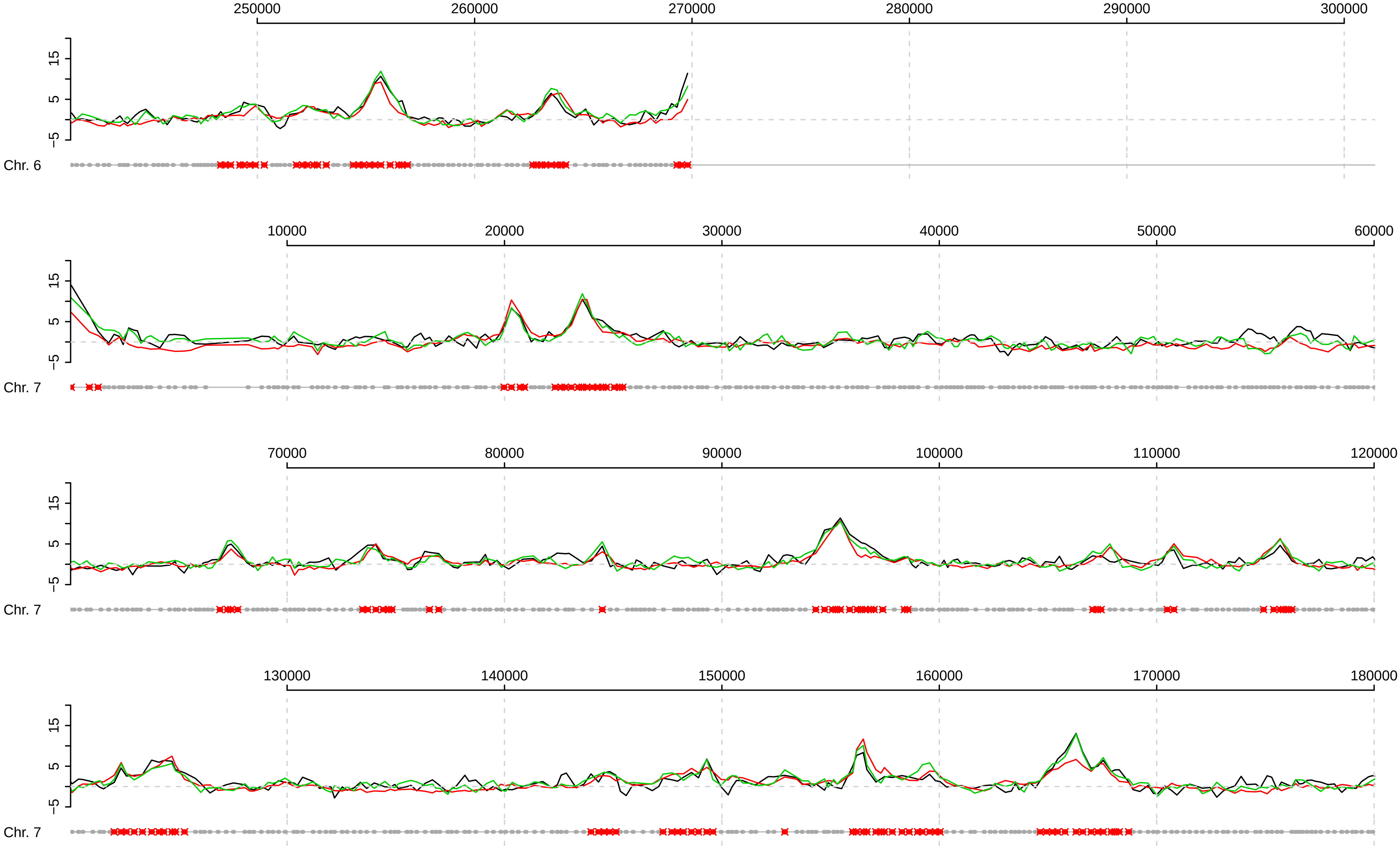

250000
260000
270000
280000
290000
300000
Chr. 6
10000
20000
30000
40000
50000
60000
Chr. 7
70000
80000
90000
100000
110000
120000
Chr. 7
130000
140000
150000
160000
170000
180000
Chr. 7
15
5
−5

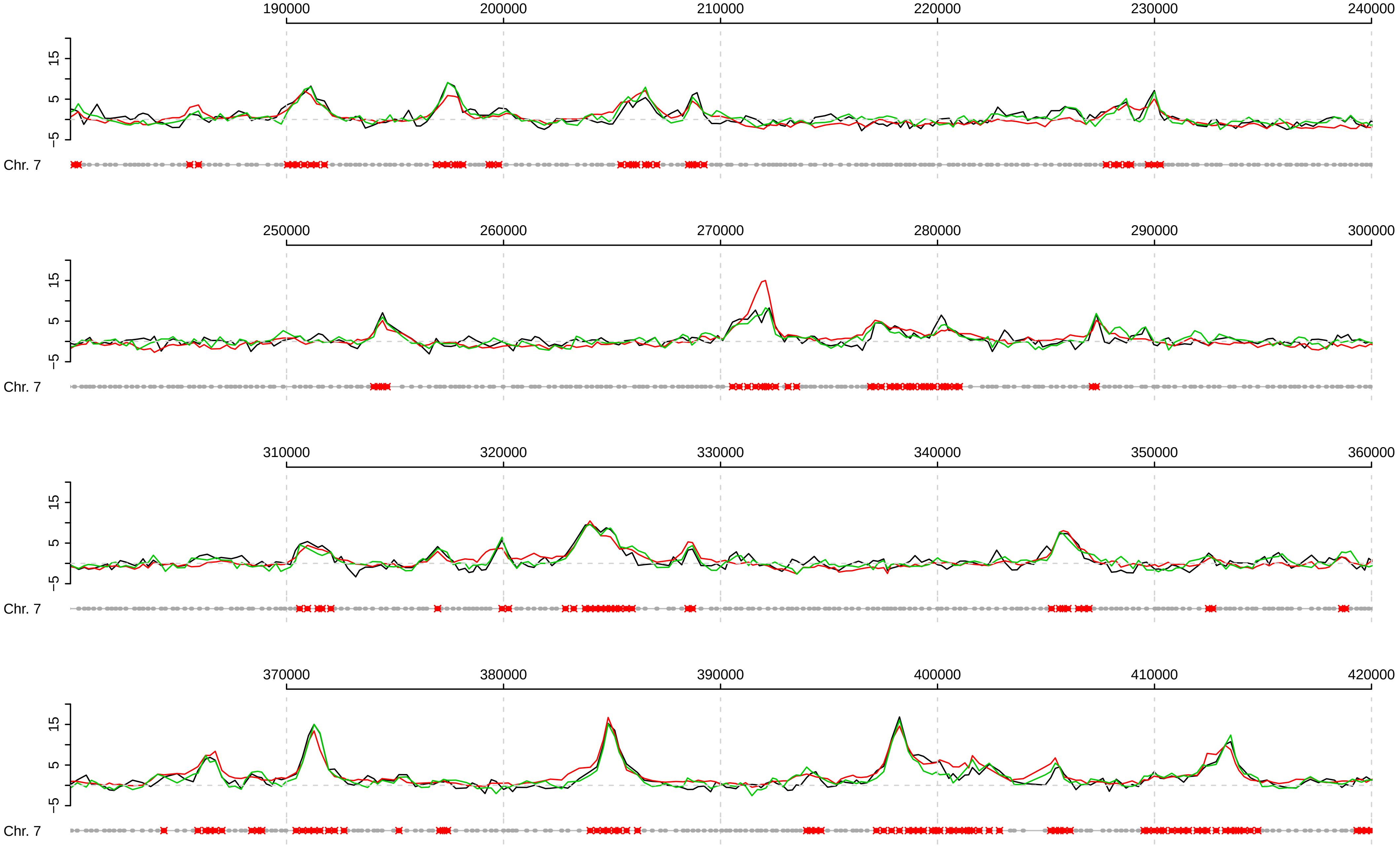
190000
200000
210000
220000
230000
240000
15
5
−5
Chr. 7
250000
260000
270000
280000
290000
300000
15
5
−5
Chr. 7
310000
320000
330000
340000
350000
360000
15
5
−5
Chr. 7
370000
380000
390000
400000
410000
420000
15
5
−5
Chr. 7

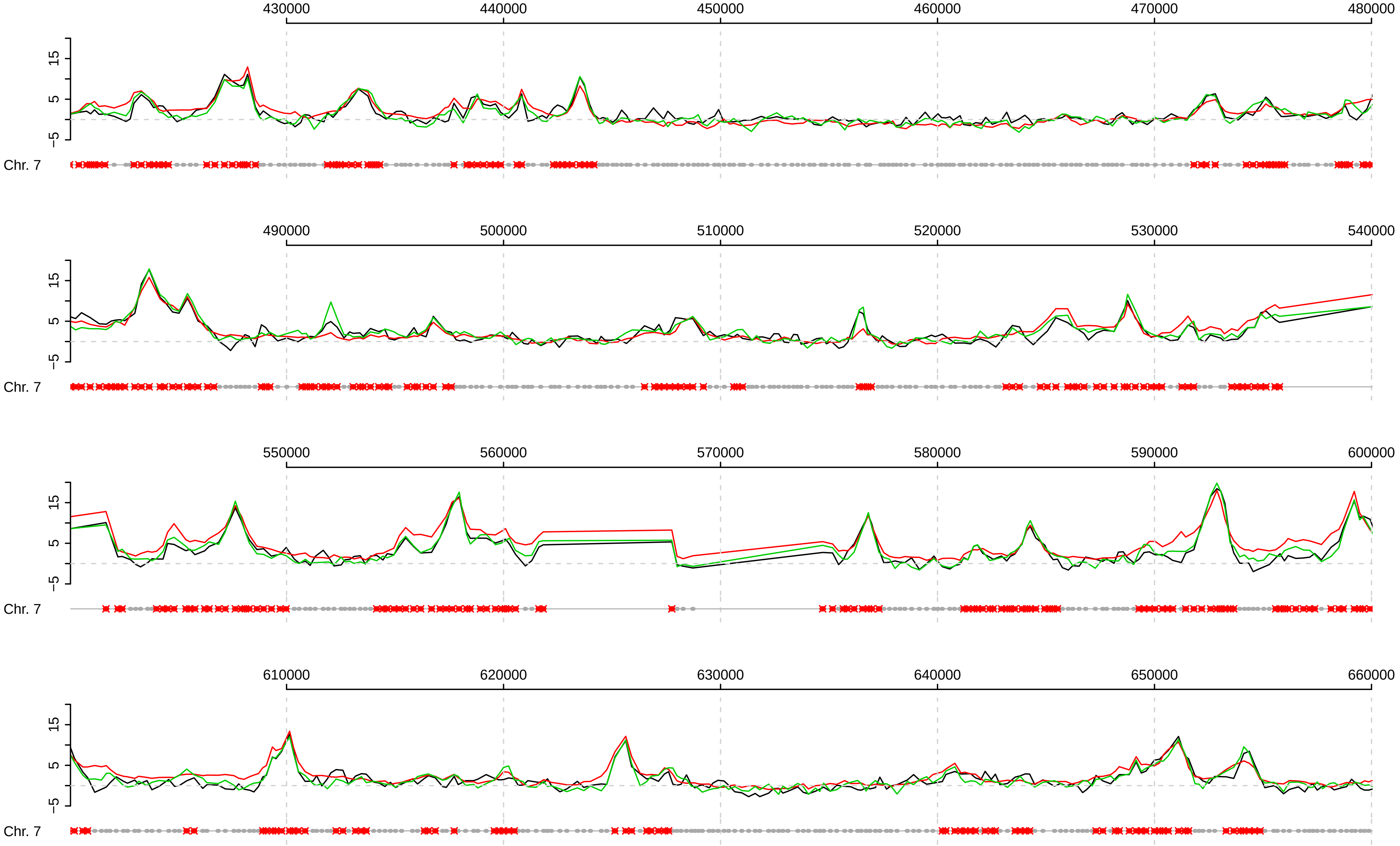
430000
440000
450000
460000
470000
480000
15
5
−5
Chr. 7
490000
500000
510000
520000
530000
540000
15
5
−5
Chr. 7
550000
560000
570000
580000
590000
600000
15
5
−5
Chr. 7
610000
620000
630000
640000
650000
660000
15
5
−5
Chr. 7

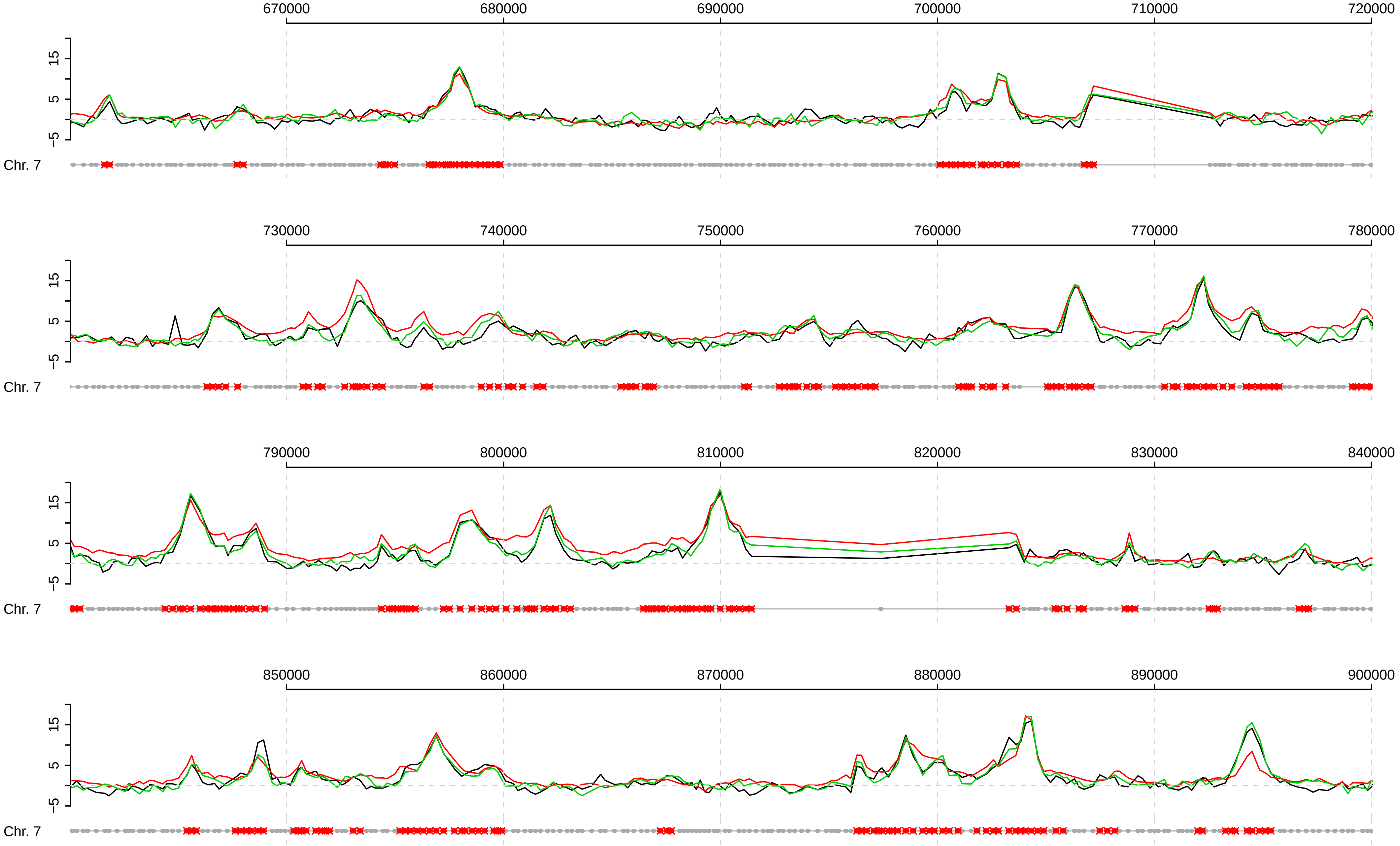

670000
680000
690000
700000
710000
720000
15
5
−5
Chr. 7
730000
740000
750000
760000
770000
780000
15
5
−5
Chr. 7
790000
800000
810000
820000
830000
840000
15
5
−5
Chr. 7
850000
860000
870000
880000
890000
900000
15
5
−5
Chr. 7

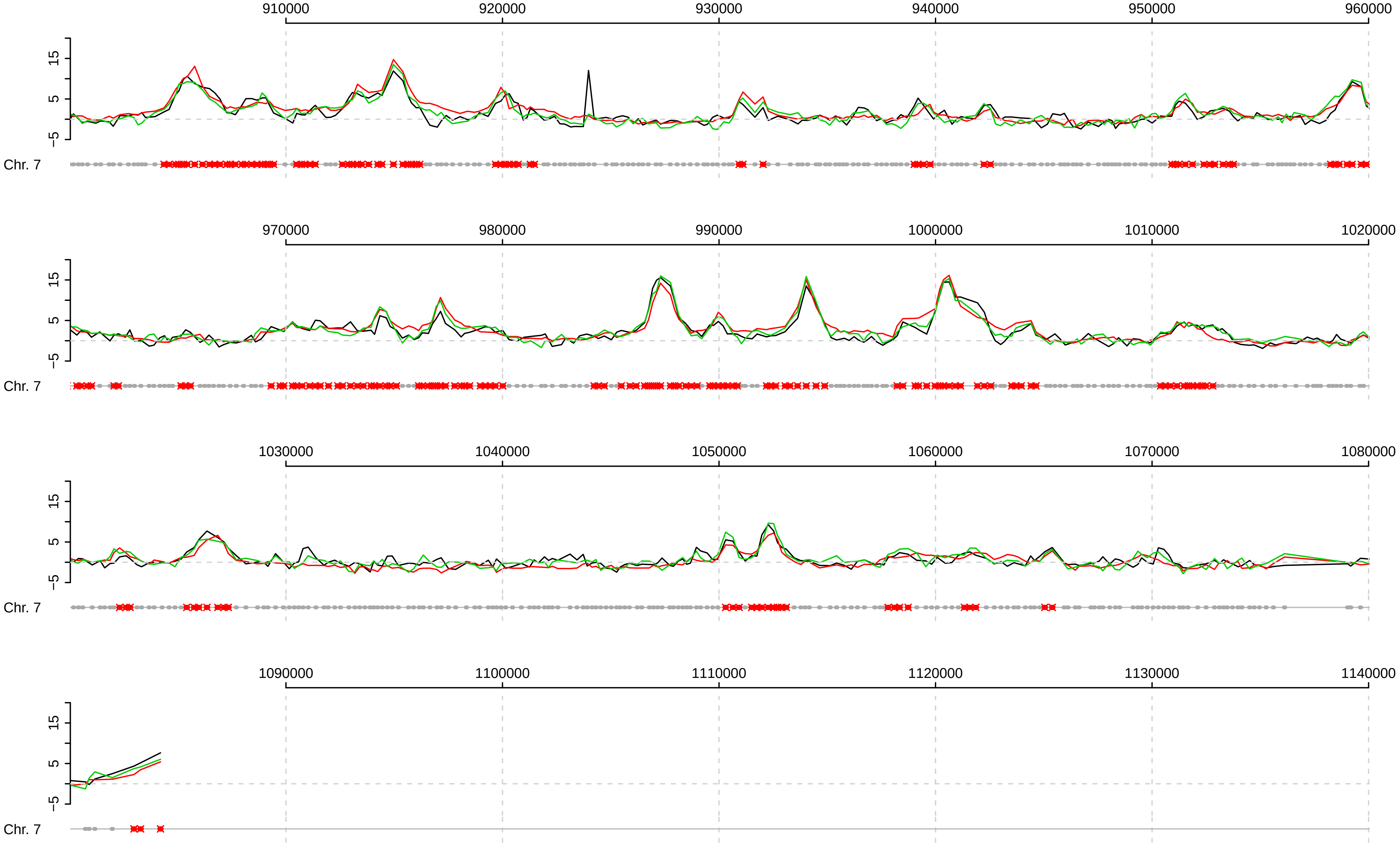
910000
920000
930000
940000
950000
960000
−5
5
15
Chr. 7
970000
980000
990000
1000000
1010000
1020000
−5
5
15
Chr. 7
1030000
1040000
1050000
1060000
1070000
1080000
−5
5
15
Chr. 7
1090000
1100000
1110000
1120000
1130000
1140000
−5
5
15
Chr. 7

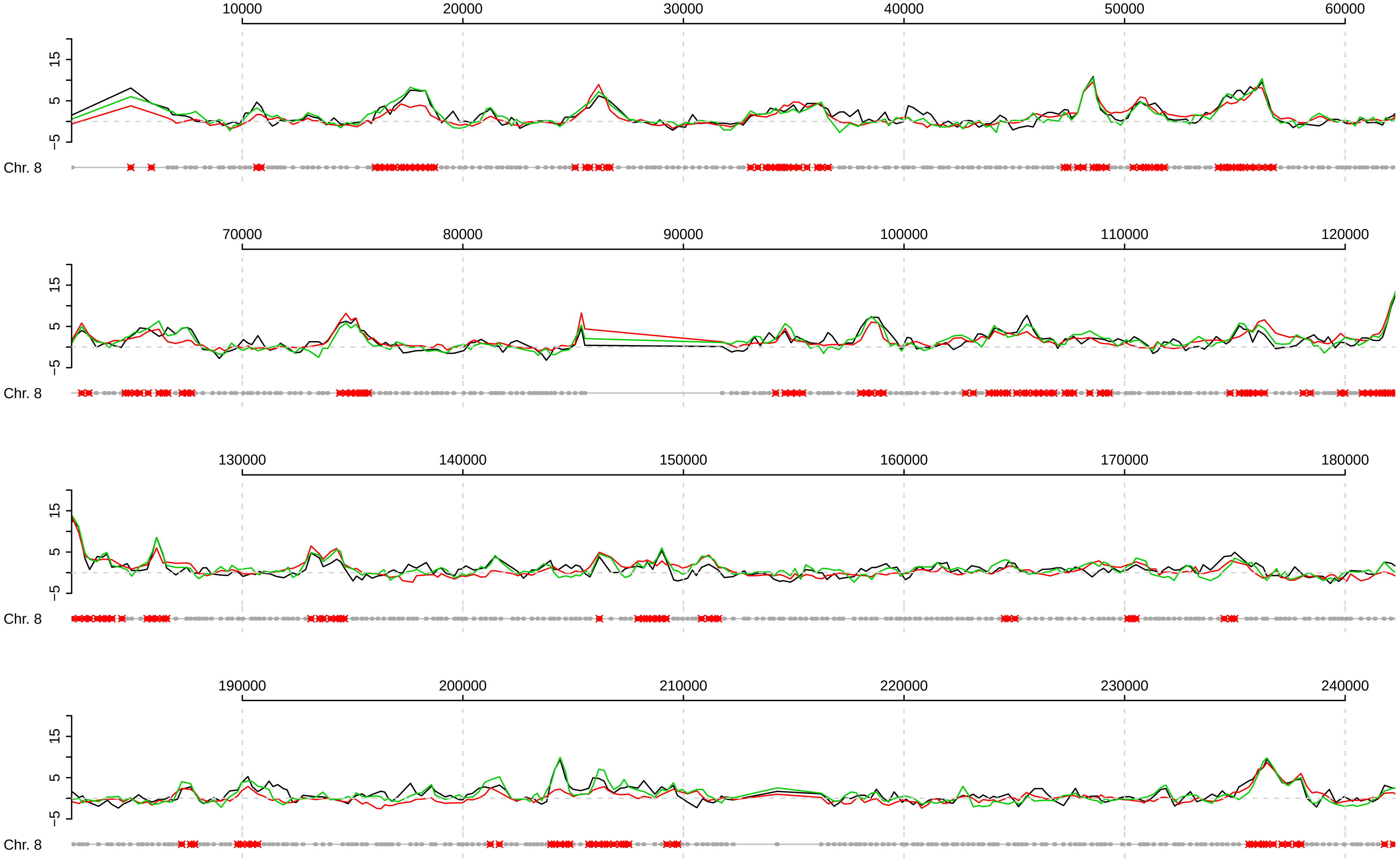
10000
20000
30000
40000
50000
60000
15
5
−5
Chr. 8
70000
80000
90000
100000
110000
120000
15
5
−5
Chr. 8
130000
140000
150000
160000
170000
180000
15
5
−5
Chr. 8
190000
200000
210000
220000
230000
240000
15
5
−5
Chr. 8

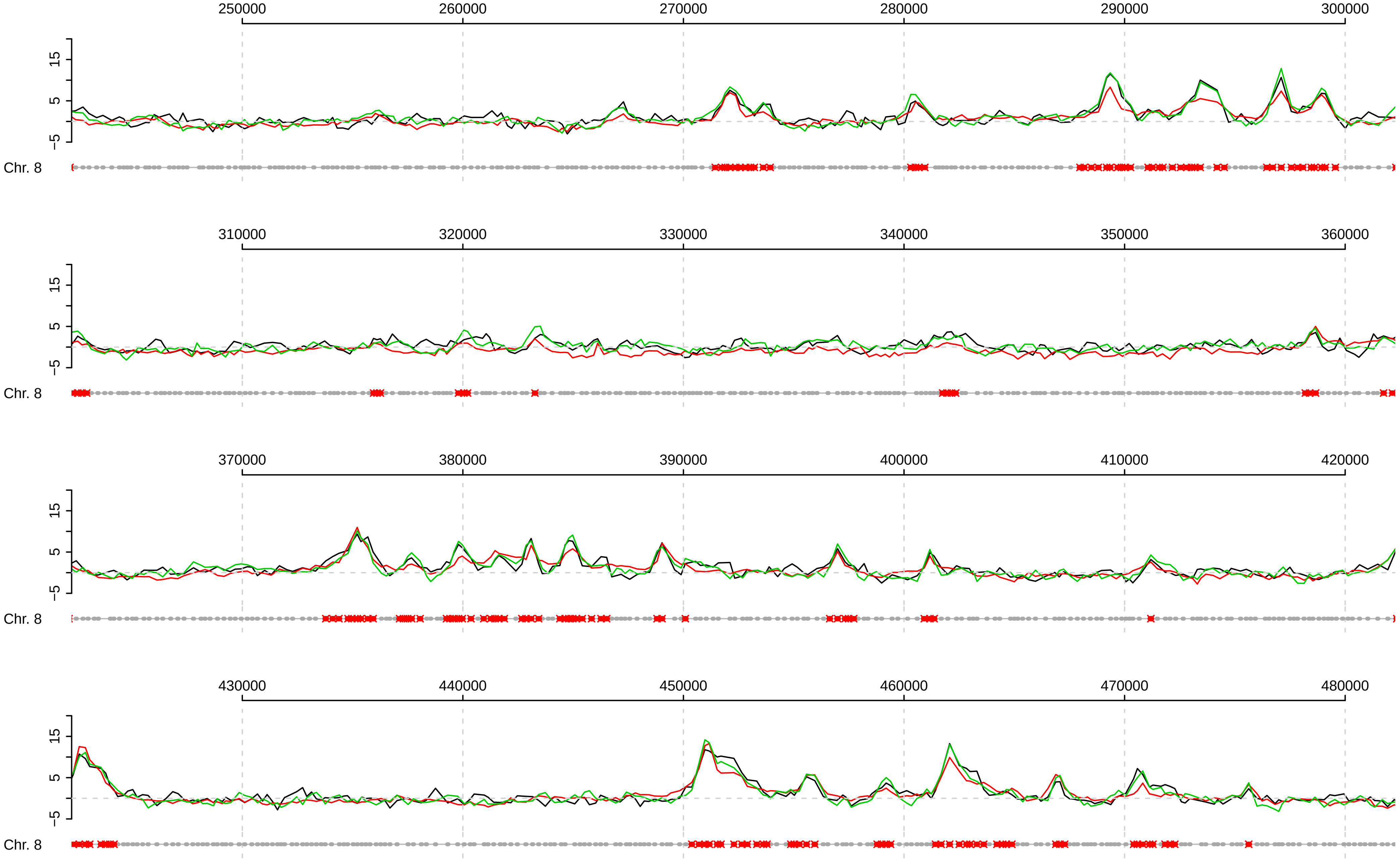

250000
260000
270000
280000
290000
300000
15
5
−5
Chr. 8
310000
320000
330000
340000
350000
360000
15
5
−5
Chr. 8
370000
380000
390000
400000
410000
420000
15
5
−5
Chr. 8
430000
440000
450000
460000
470000
480000
15
5
−5
Chr. 8

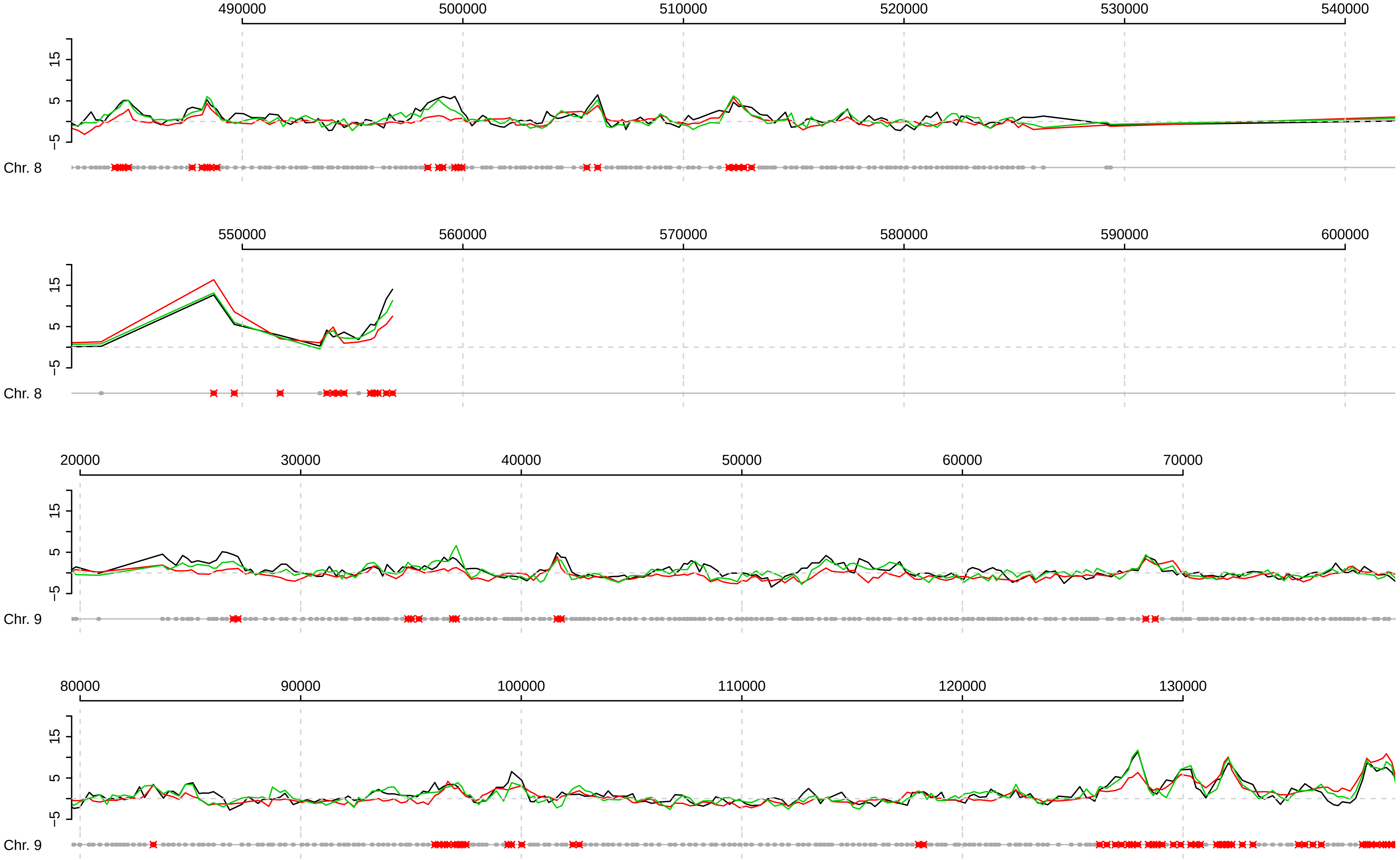
490000
500000
510000
520000
530000
540000
15
5
−5
Chr. 8
550000
560000
570000
580000
590000
600000
15
5
−5
Chr. 8
20000
30000
40000
50000
60000
70000
15
5
−5
Chr. 9
80000
90000
100000
110000
120000
130000
15
5
−5
Chr. 9

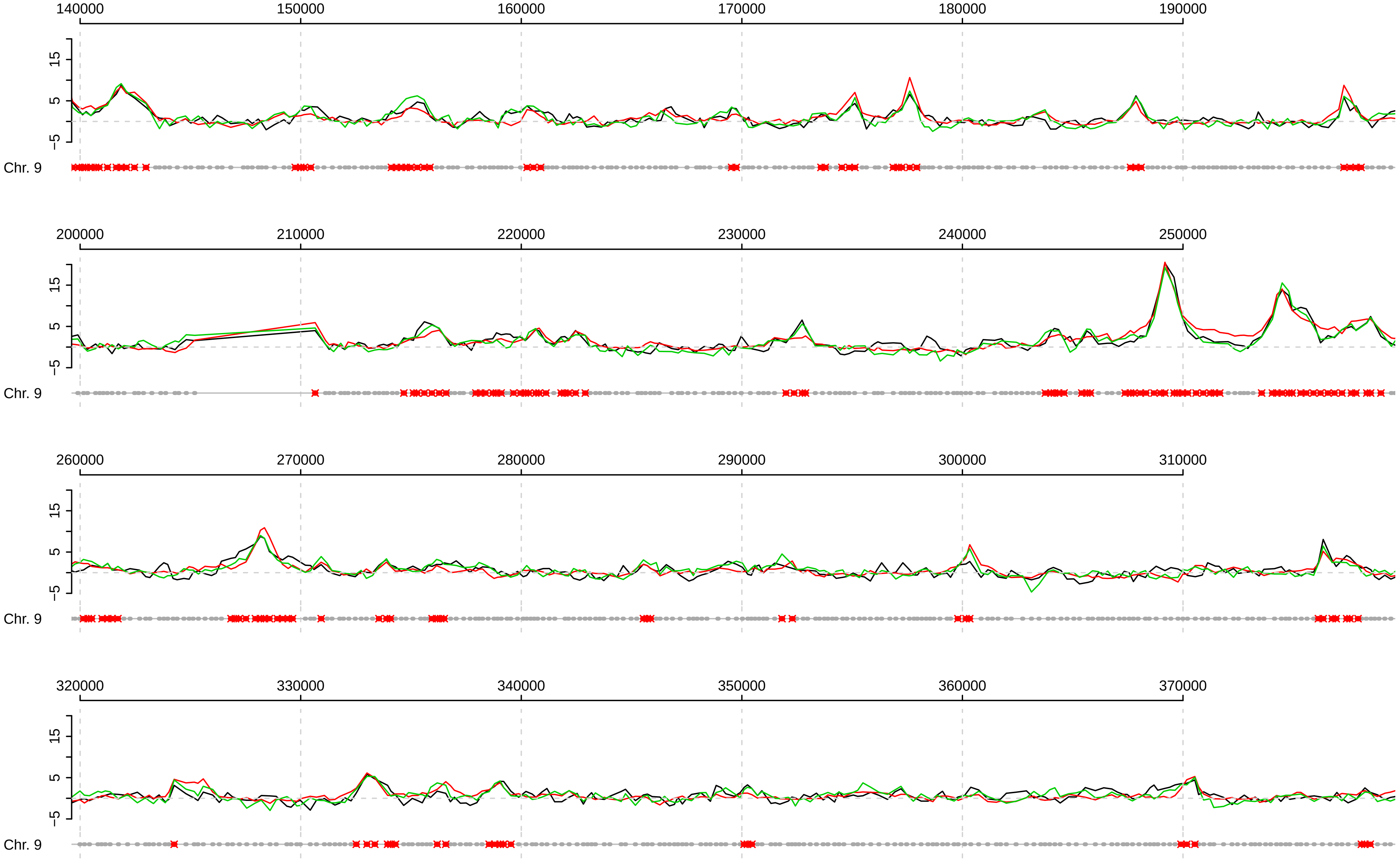

140000
150000
160000
170000
180000
190000
15
5
−5
Chr. 9
200000
210000
220000
230000
240000
250000
15
5
−5
Chr. 9
260000
270000
280000
290000
300000
310000
15
5
−5
Chr. 9
320000
330000
340000
350000
360000
370000
15
5
−5
Chr. 9

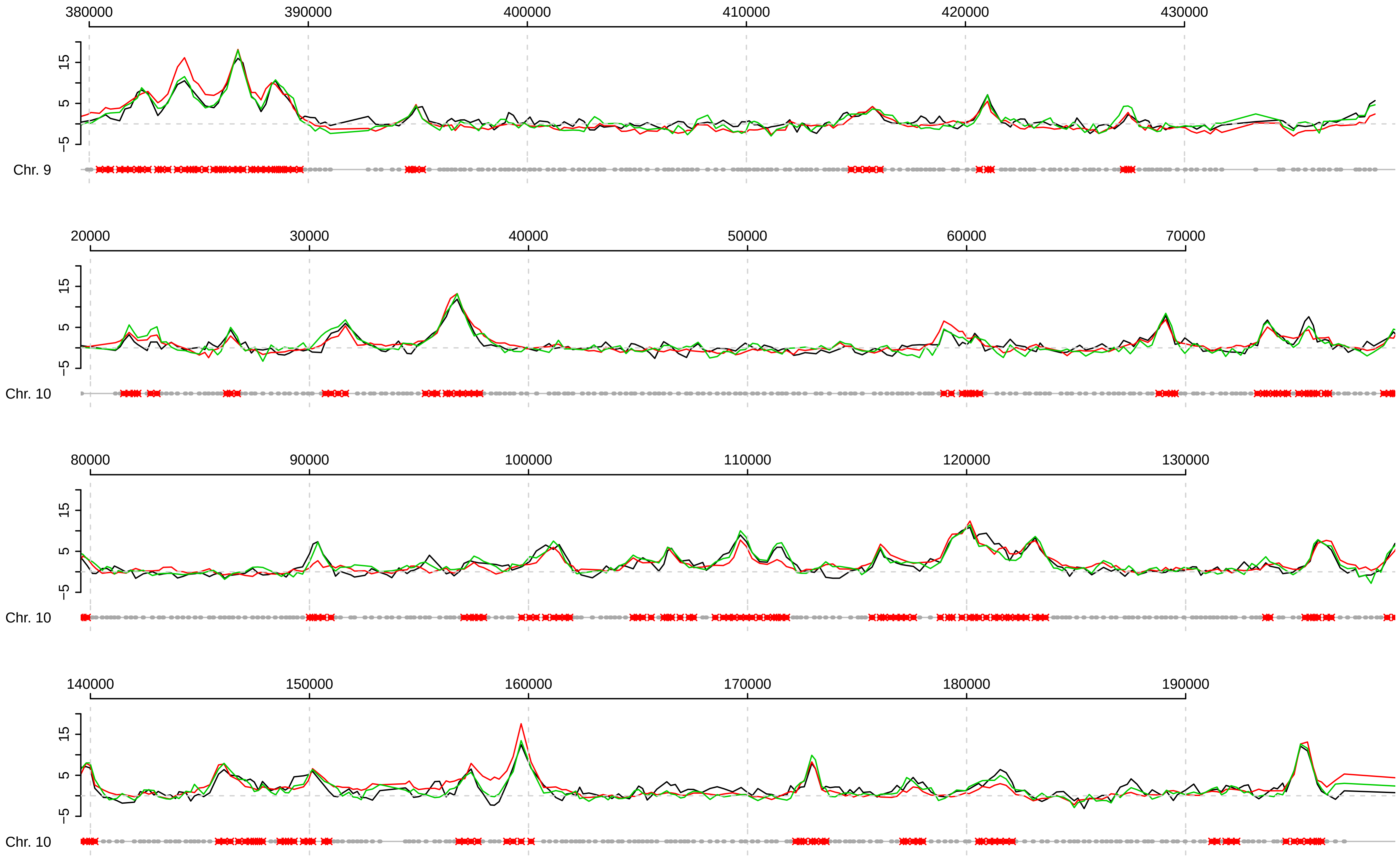

380000
390000
400000
410000
420000
430000
15
5
−5
Chr. 9
20000
30000
40000
50000
60000
70000
15
5
−5
Chr. 10
80000
90000
100000
110000
120000
130000
15
5
−5
Chr. 10
140000
150000
160000
170000
180000
190000
15
5
−5
Chr. 10

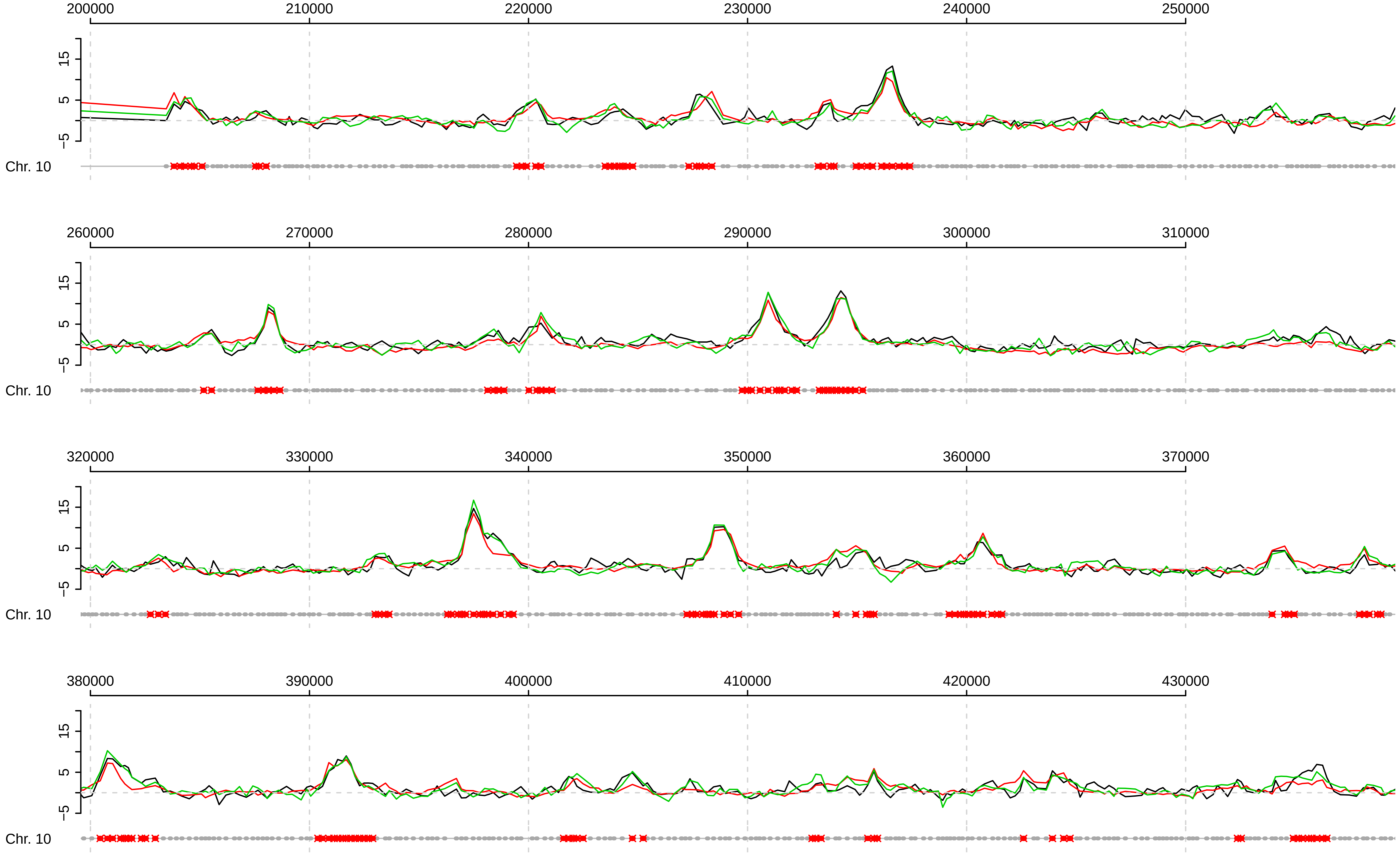

200000
210000
220000
230000
240000
250000
15
5
−5
Chr. 10
260000
270000
280000
290000
300000
310000
15
5
−5
Chr. 10
320000
330000
340000
350000
360000
370000
15
5
−5
Chr. 10
380000
390000
400000
410000
420000
430000
15
5
−5
Chr. 10

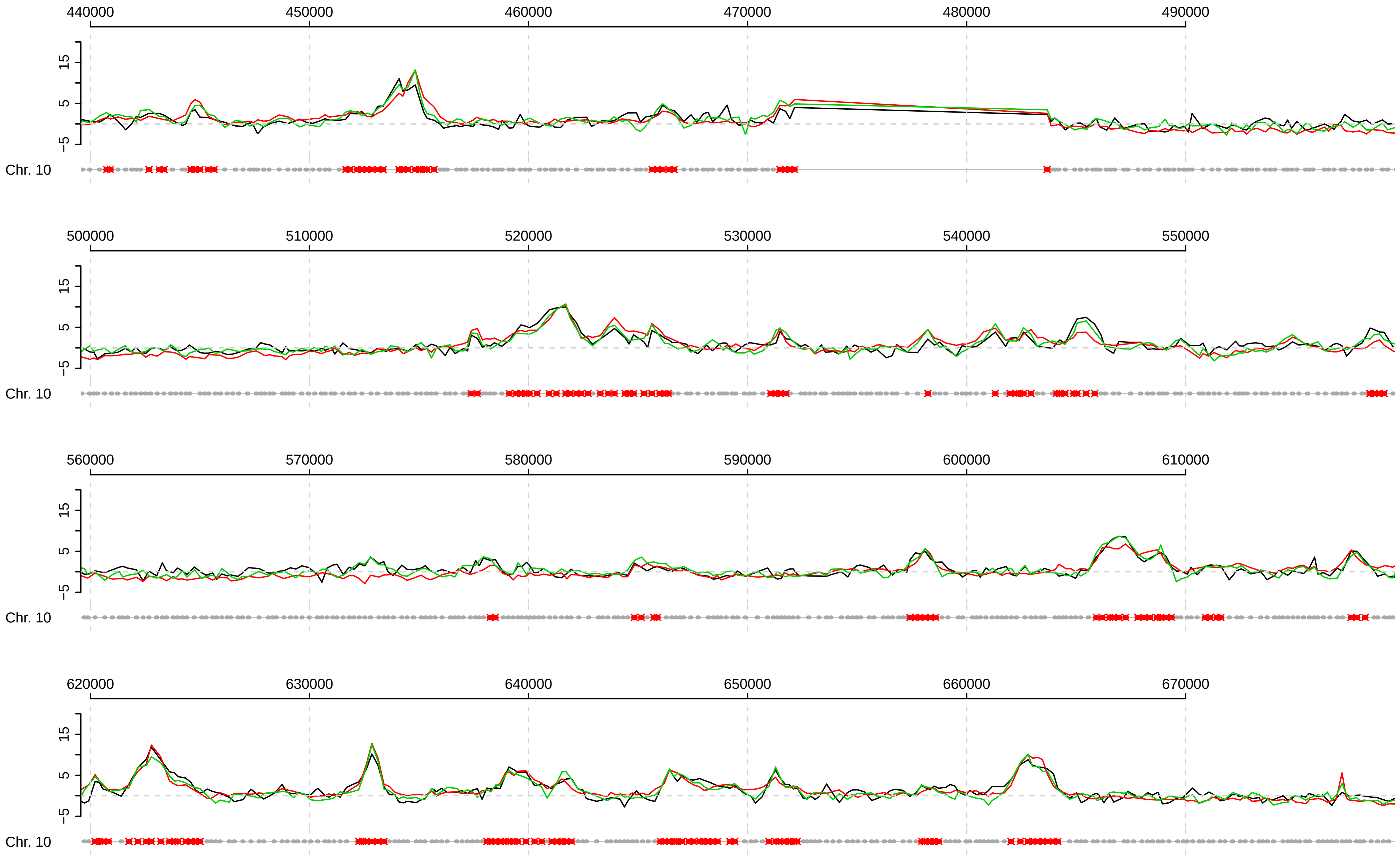

440000
450000
460000
470000
480000
490000
15
5
−5
Chr. 10
500000
510000
520000
530000
540000
550000
15
5
−5
Chr. 10
560000
570000
580000
590000
600000
610000
15
5
−5
Chr. 10
620000
630000
640000
650000
660000
670000
15
5
−5
Chr. 10

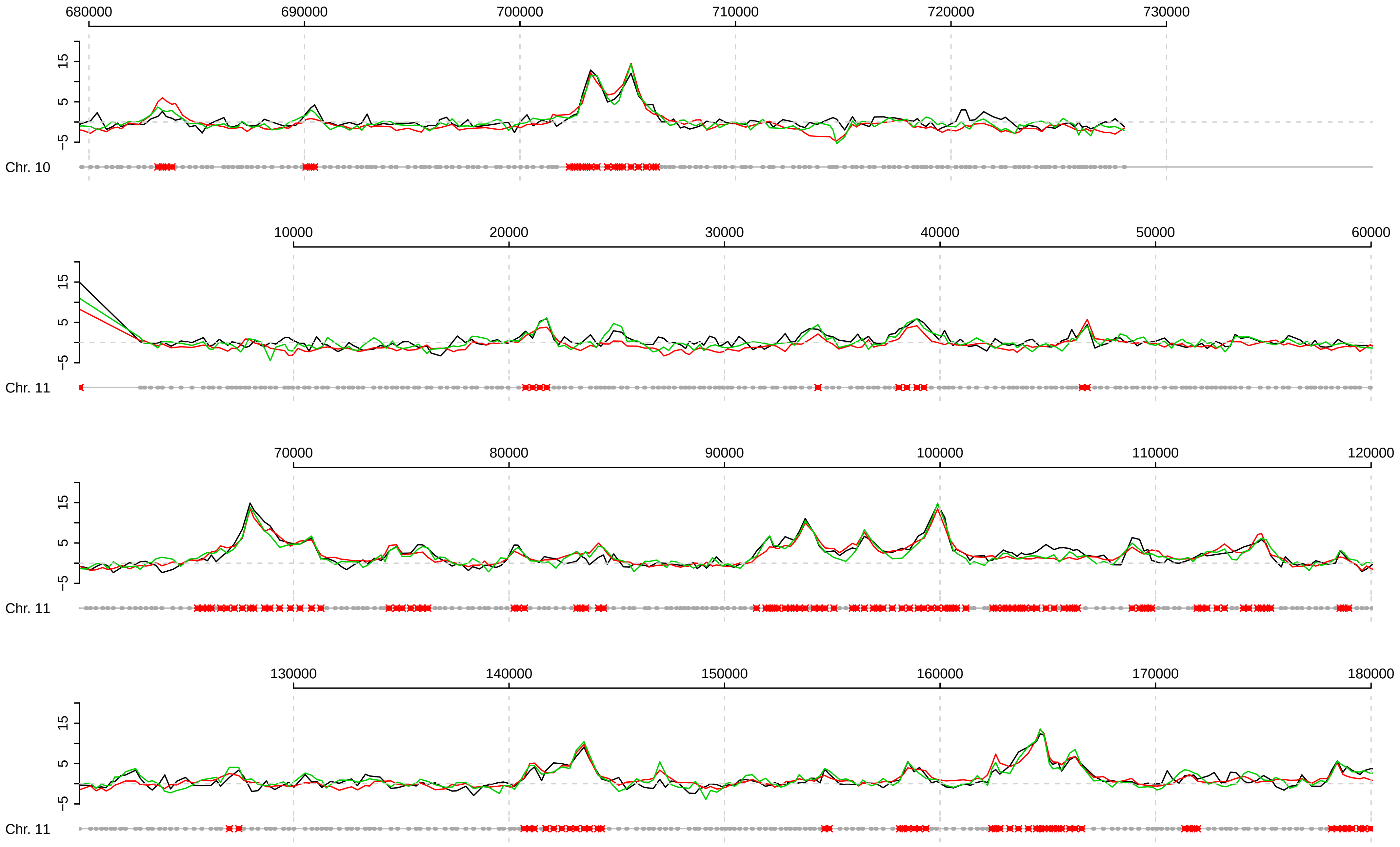

680000
690000
700000
710000
720000
730000
15
5
−5
Chr. 10
10000
20000
30000
40000
50000
60000
15
5
−5
Chr. 11
70000
80000
90000
100000
110000
120000
15
5
−5
Chr. 11
130000
140000
150000
160000
170000
180000
15
5
−5
Chr. 11

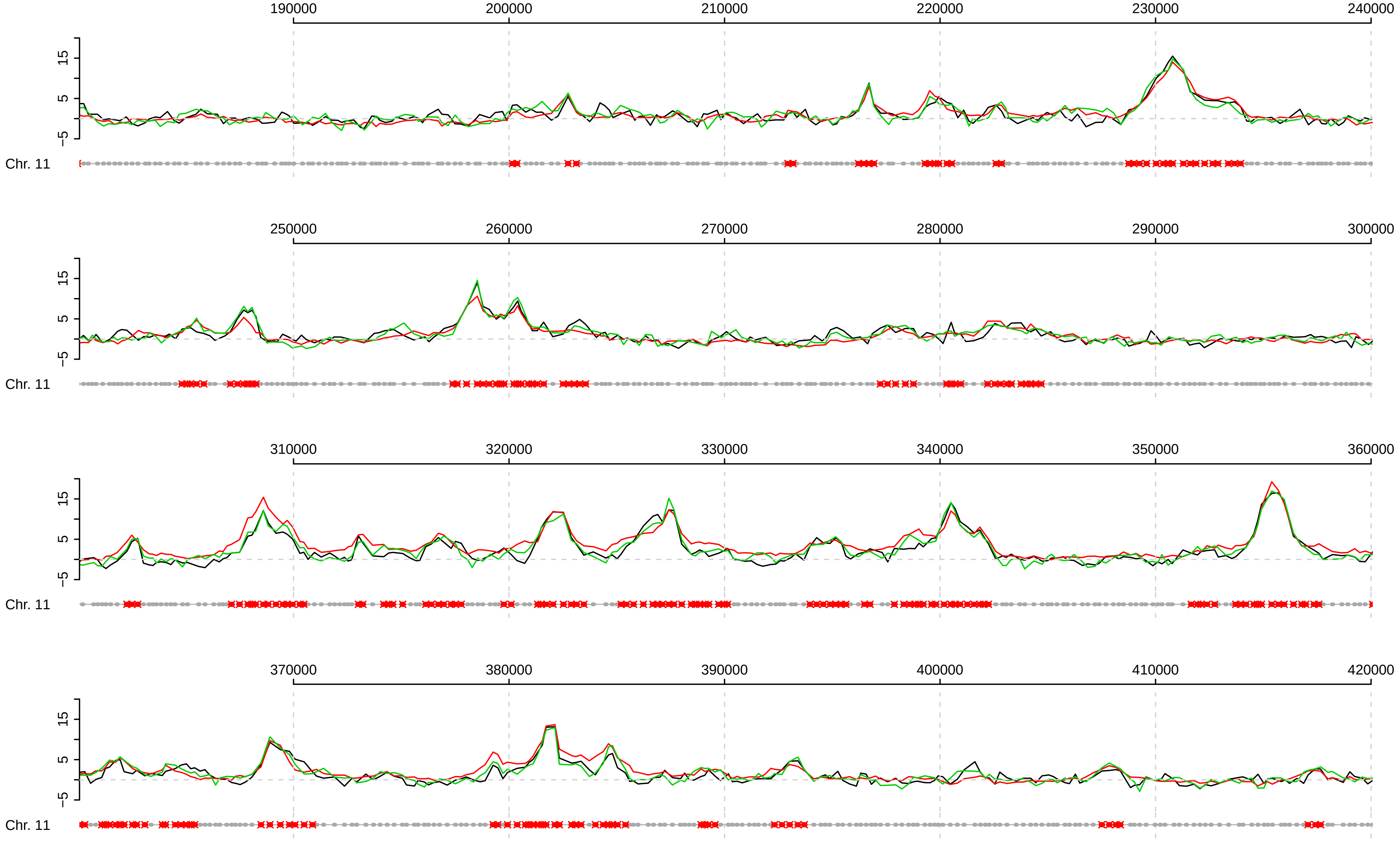

190000
200000
210000
220000
230000
240000
15
5
−5
Chr. 11
250000
260000
270000
280000
290000
300000
15
5
−5
Chr. 11
310000
320000
330000
340000
350000
360000
15
5
−5
Chr. 11
370000
380000
390000
400000
410000
420000
15
5
−5
Chr. 11

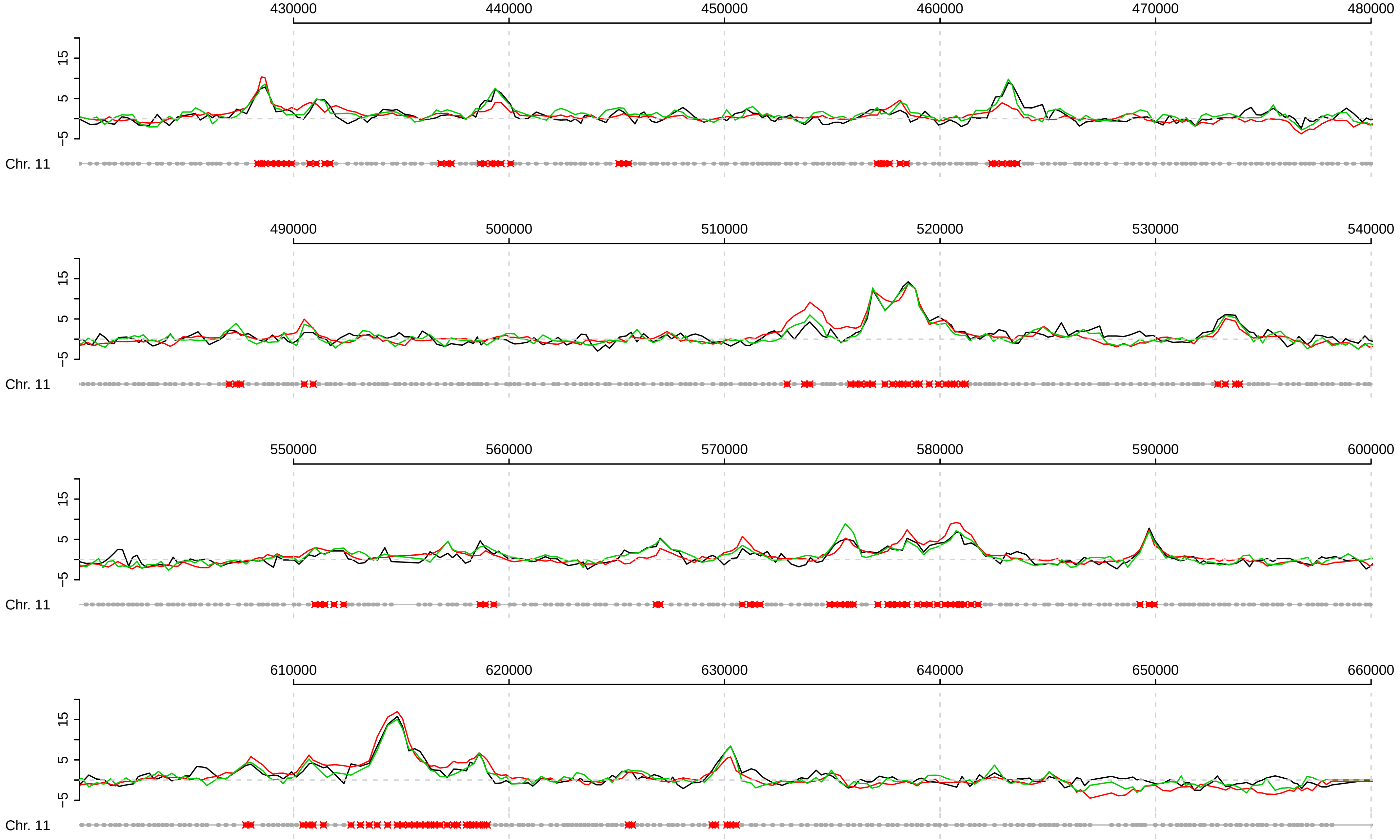
430000
440000
450000
460000
470000
480000
15
5
−5
Chr. 11
490000
500000
510000
520000
530000
540000
15
5
−5
Chr. 11
550000
560000
570000
580000
590000
600000
15
5
−5
Chr. 11
610000
620000
630000
640000
650000
660000
15
5
−5
Chr. 11

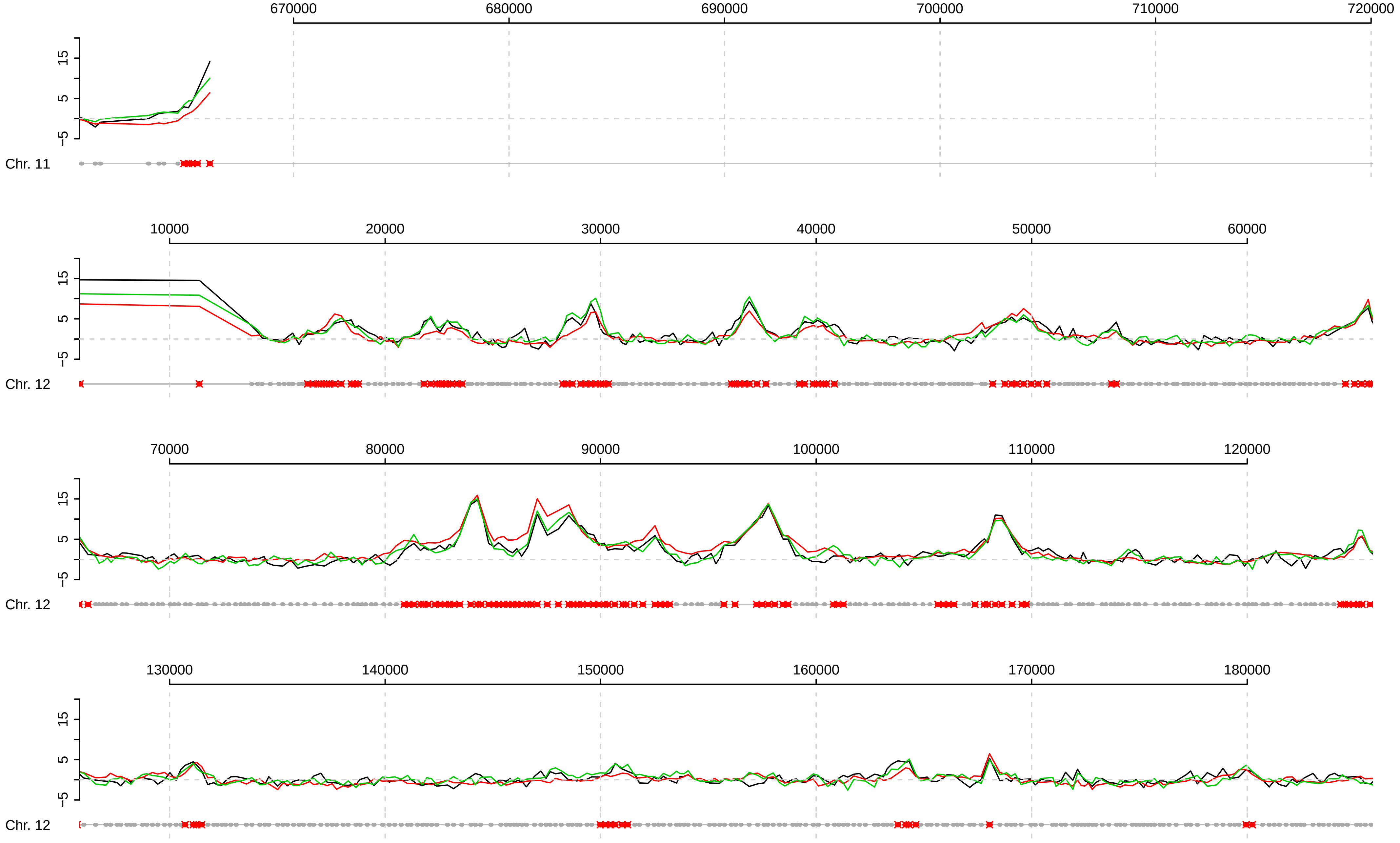
670000
680000
690000
700000
710000
720000
15
5
−5
Chr. 11
10000
20000
30000
40000
50000
60000
Chr. 12
70000
80000
90000
100000
110000
120000
Chr. 12
130000
140000
150000
160000
170000
180000
Chr. 12

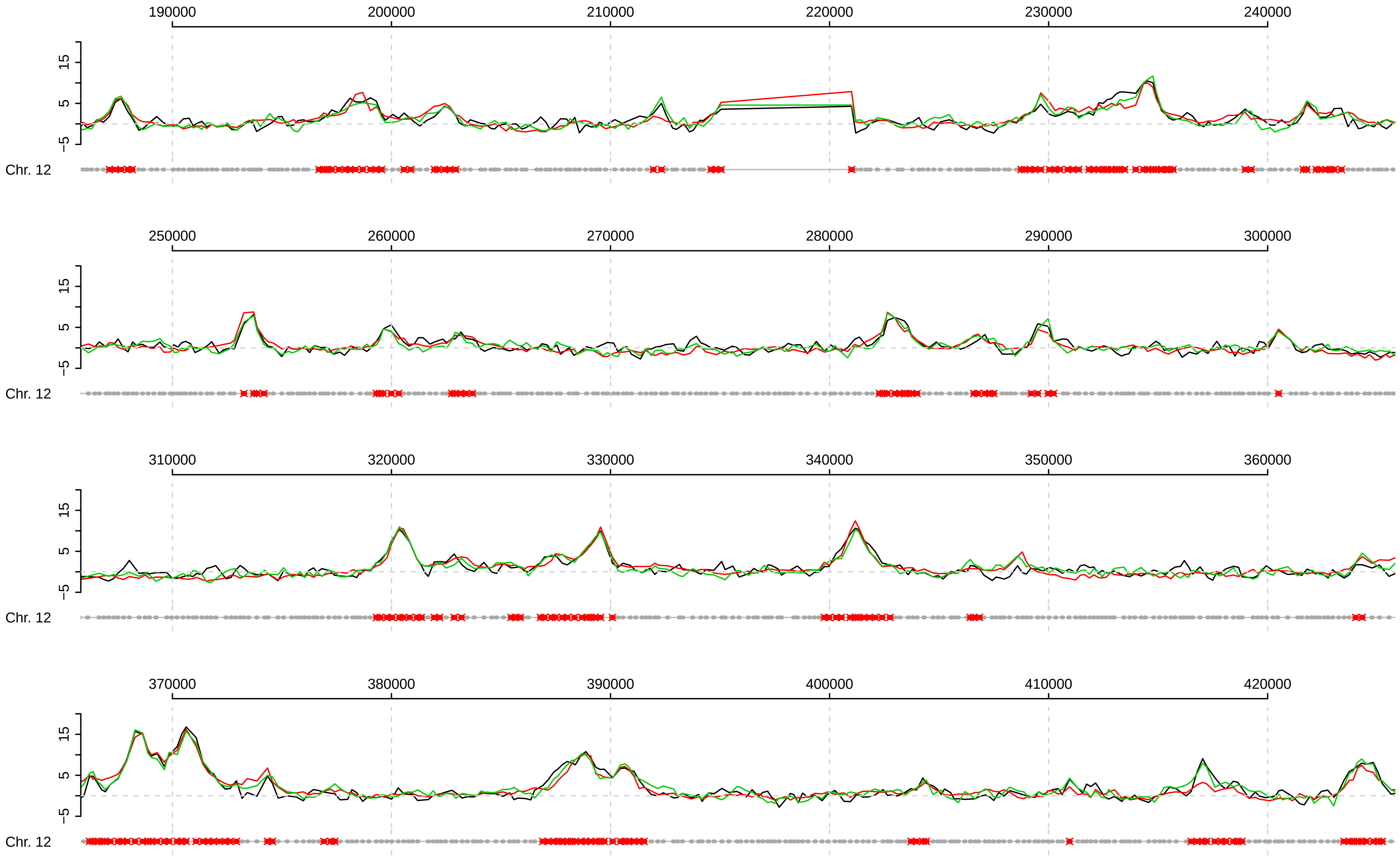

190000
200000
210000
220000
230000
240000
15
5
−5
Chr. 12
250000
260000
270000
280000
290000
300000
15
5
−5
Chr. 12
310000
320000
330000
340000
350000
360000
15
5
−5
Chr. 12
370000
380000
390000
400000
410000
420000
15
5
−5
Chr. 12

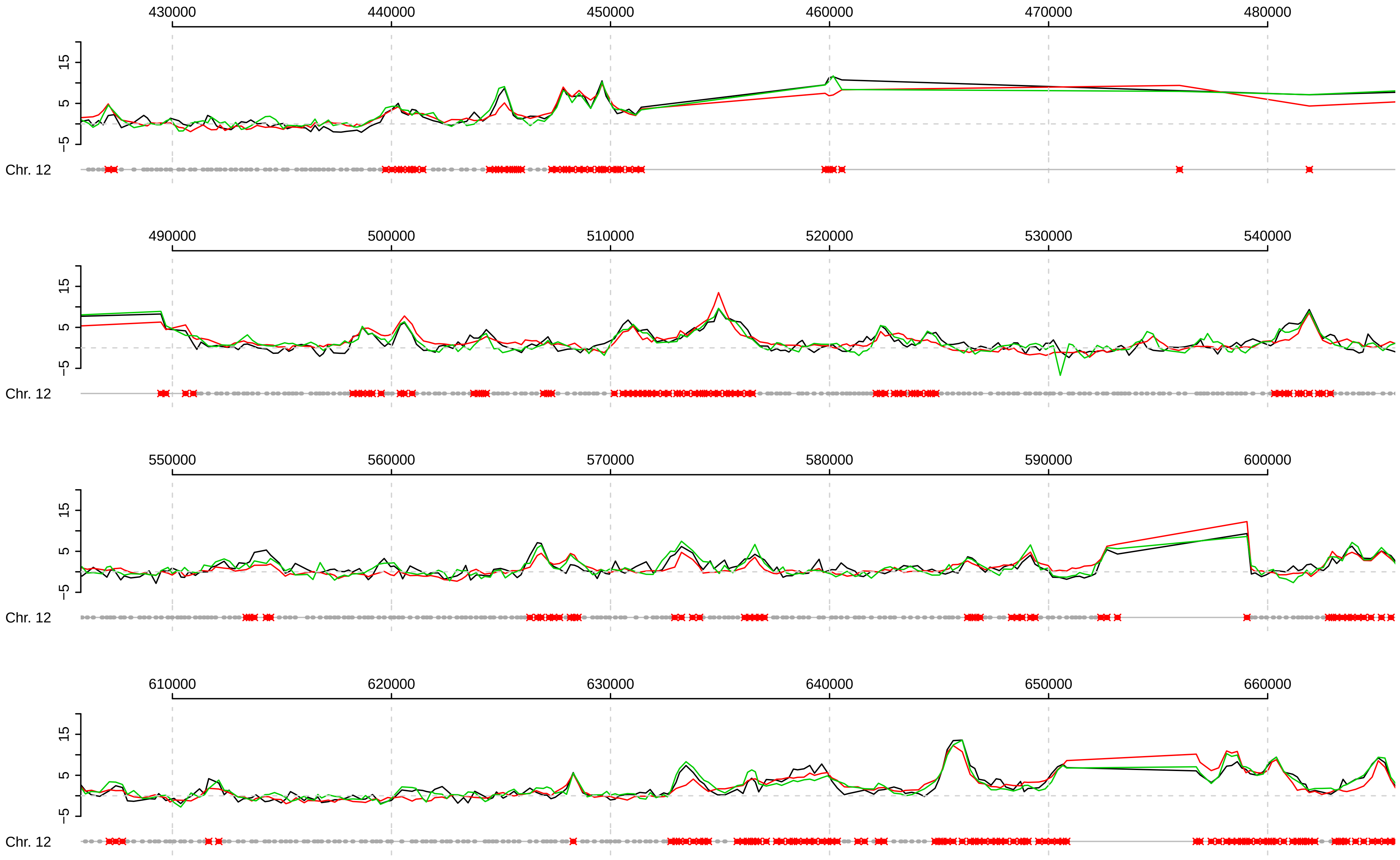

430000
440000
450000
460000
470000
480000
15
5
−5
Chr. 12
490000
500000
510000
520000
530000
540000
15
5
−5
Chr. 12
550000
560000
570000
580000
590000
600000
15
5
−5
Chr. 12
610000
620000
630000
640000
650000
660000
15
5
−5
Chr. 12

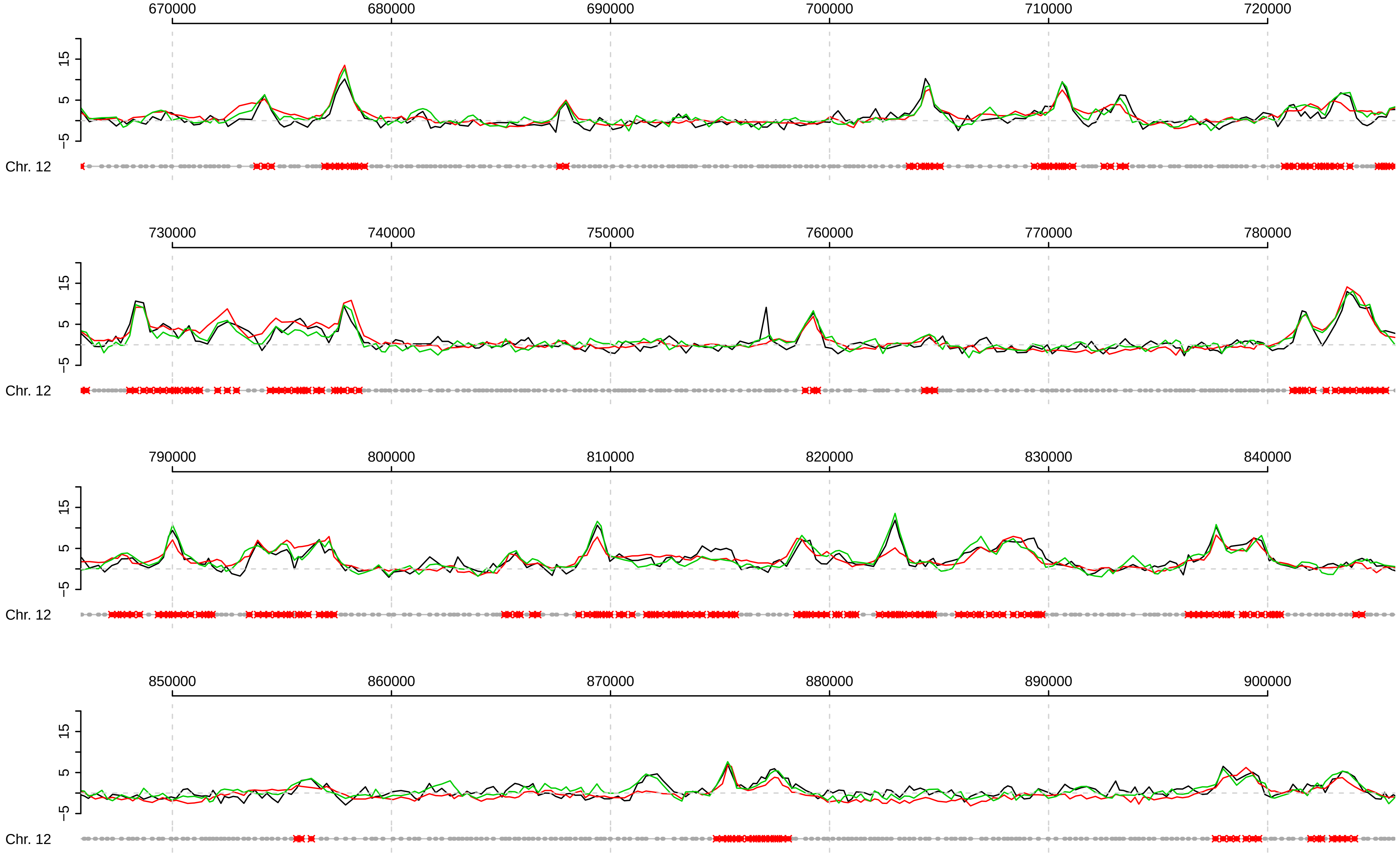

670000
680000
690000
700000
710000
720000
15
5
−5
Chr. 12
730000
740000
750000
760000
770000
780000
15
5
−5
Chr. 12
790000
800000
810000
820000
830000
840000
15
5
−5
Chr. 12
850000
860000
870000
880000
890000
900000
15
5
−5
Chr. 12

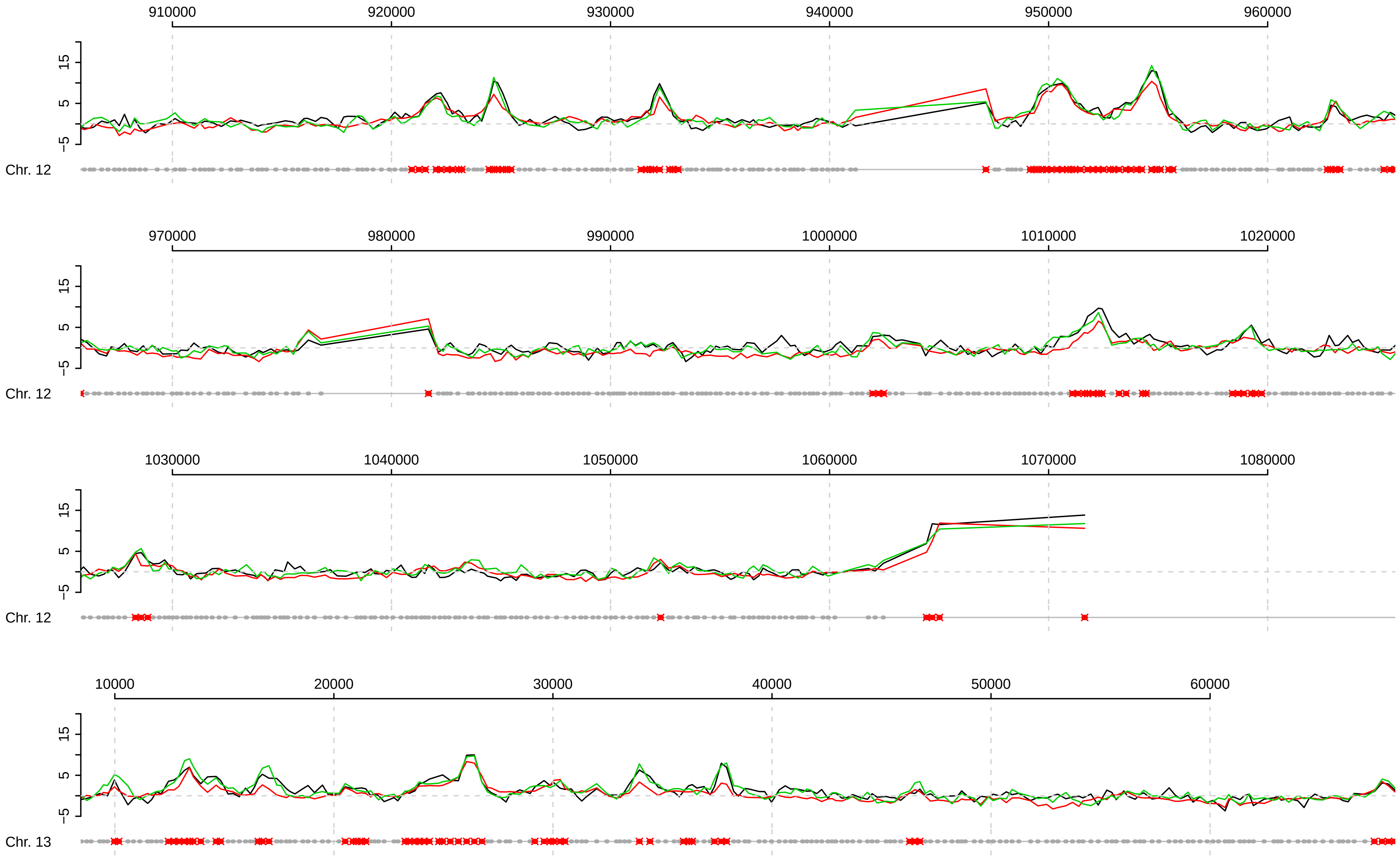

910000
920000
930000
940000
950000
960000
15
5
−5
Chr. 12
970000
980000
990000
1000000
1010000
1020000
15
5
−5
Chr. 12
1030000
1040000
1050000
1060000
1070000
1080000
15
5
−5
Chr. 12
10000
20000
30000
40000
50000
60000
15
5
−5
Chr. 13

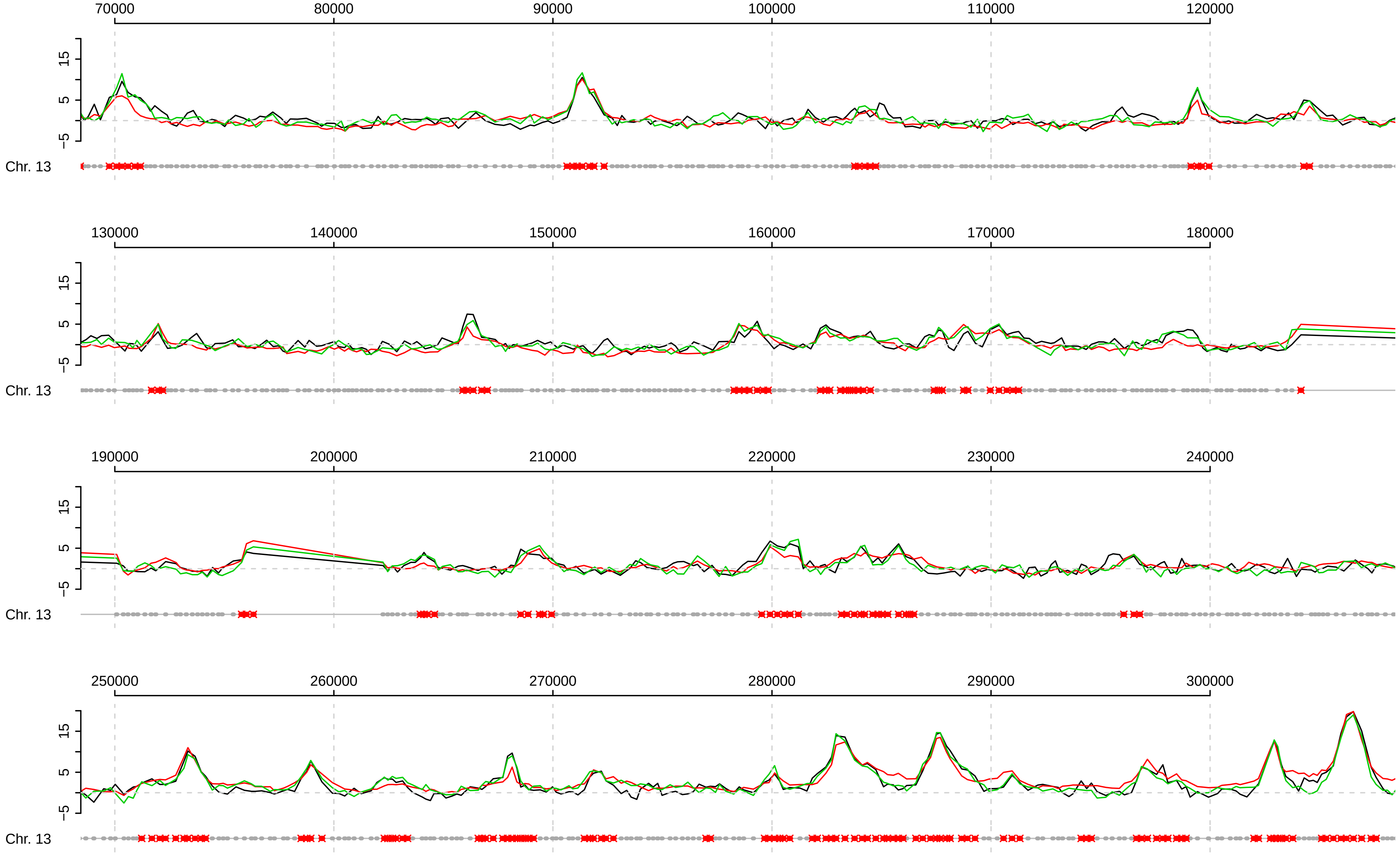

70000
80000
90000
100000
110000
120000
130000
140000
150000
160000
170000
180000
190000
200000
210000
220000
230000
240000
250000
260000
270000
280000
290000
300000
15
5
−5
Chr. 13

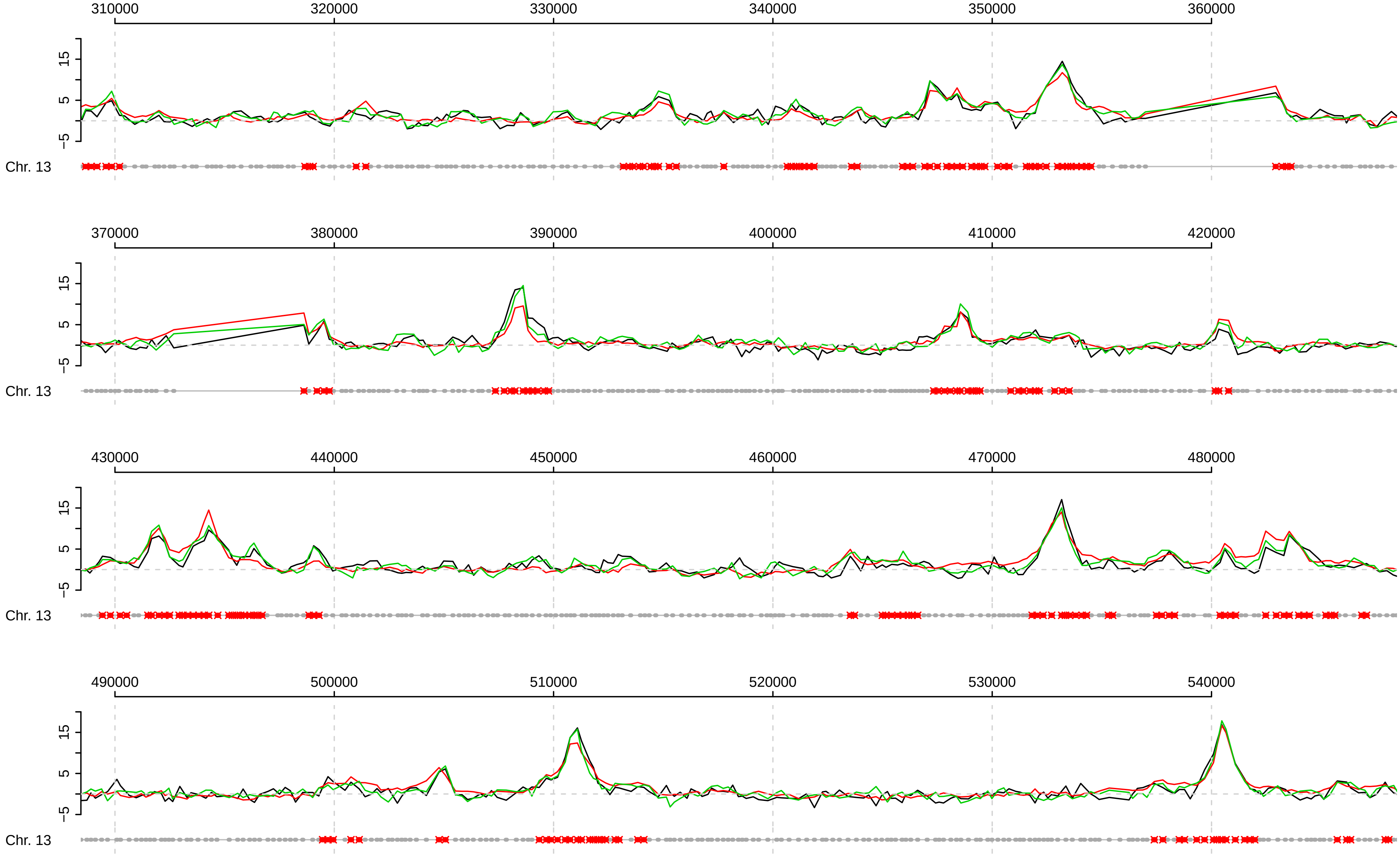

310000
320000
330000
340000
350000
360000
15
5
−5
Chr. 13
370000
380000
390000
400000
410000
420000
15
5
−5
Chr. 13
430000
440000
450000
460000
470000
480000
15
5
−5
Chr. 13
490000
500000
510000
520000
530000
540000
15
5
−5
Chr. 13

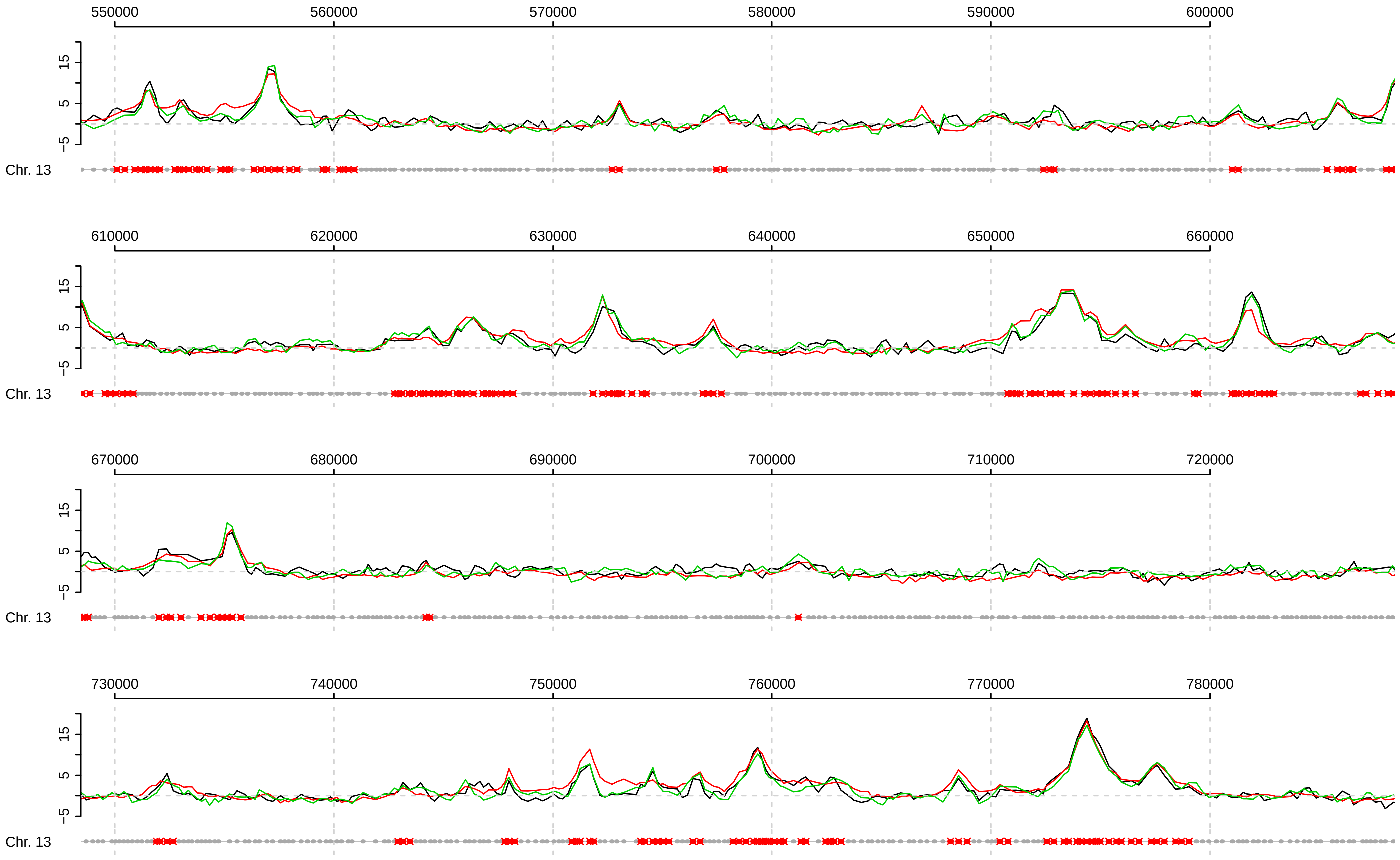
550000
560000
570000
580000
590000
600000
−5
5
15
Chr. 13
610000
620000
630000
640000
650000
660000
−5
5
15
Chr. 13
670000
680000
690000
700000
710000
720000
−5
5
15
Chr. 13
730000
740000
750000
760000
770000
780000
−5
5
15
Chr. 13

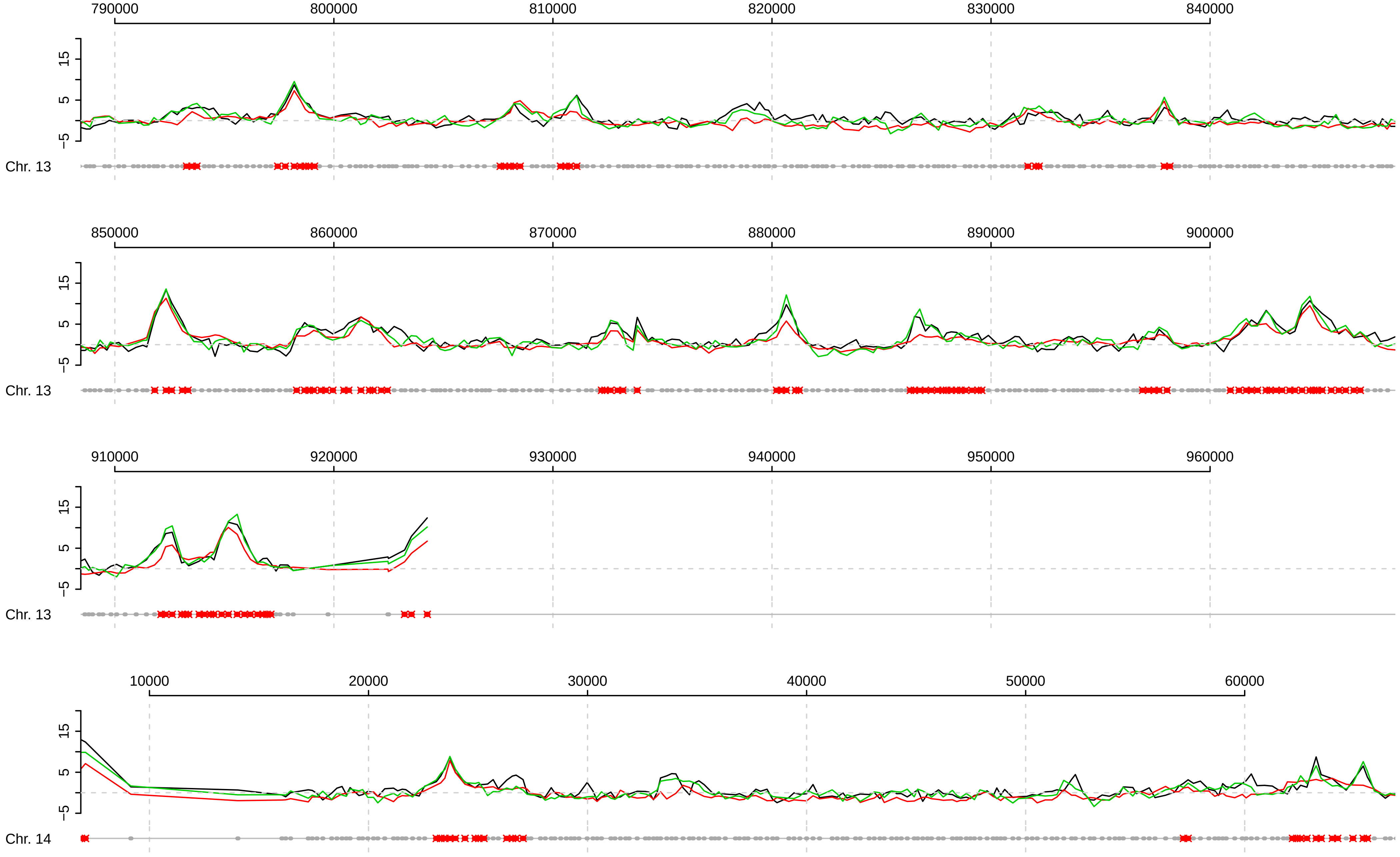

790000
800000
810000
820000
830000
840000
15
5
−5
Chr. 13
850000
860000
870000
880000
890000
900000
Chr. 13
910000
920000
930000
940000
950000
960000
Chr. 13
10000
20000
30000
40000
50000
60000
Chr. 14

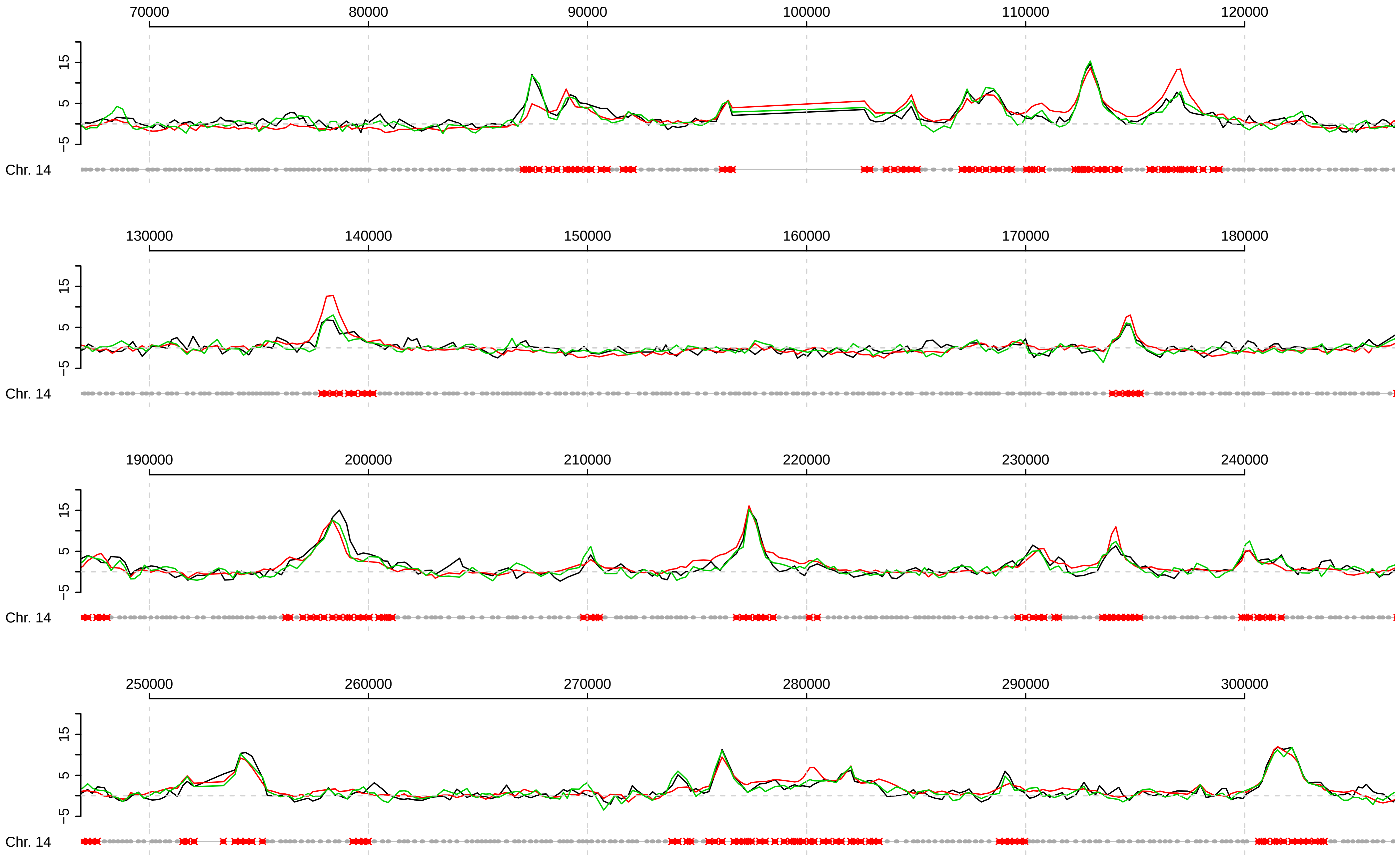
70000
80000
90000
100000
110000
120000
15
5
−5
Chr. 14
130000
140000
150000
160000
170000
180000
15
5
−5
Chr. 14
190000
200000
210000
220000
230000
240000
15
5
−5
Chr. 14
250000
260000
270000
280000
290000
300000
15
5
−5
Chr. 14

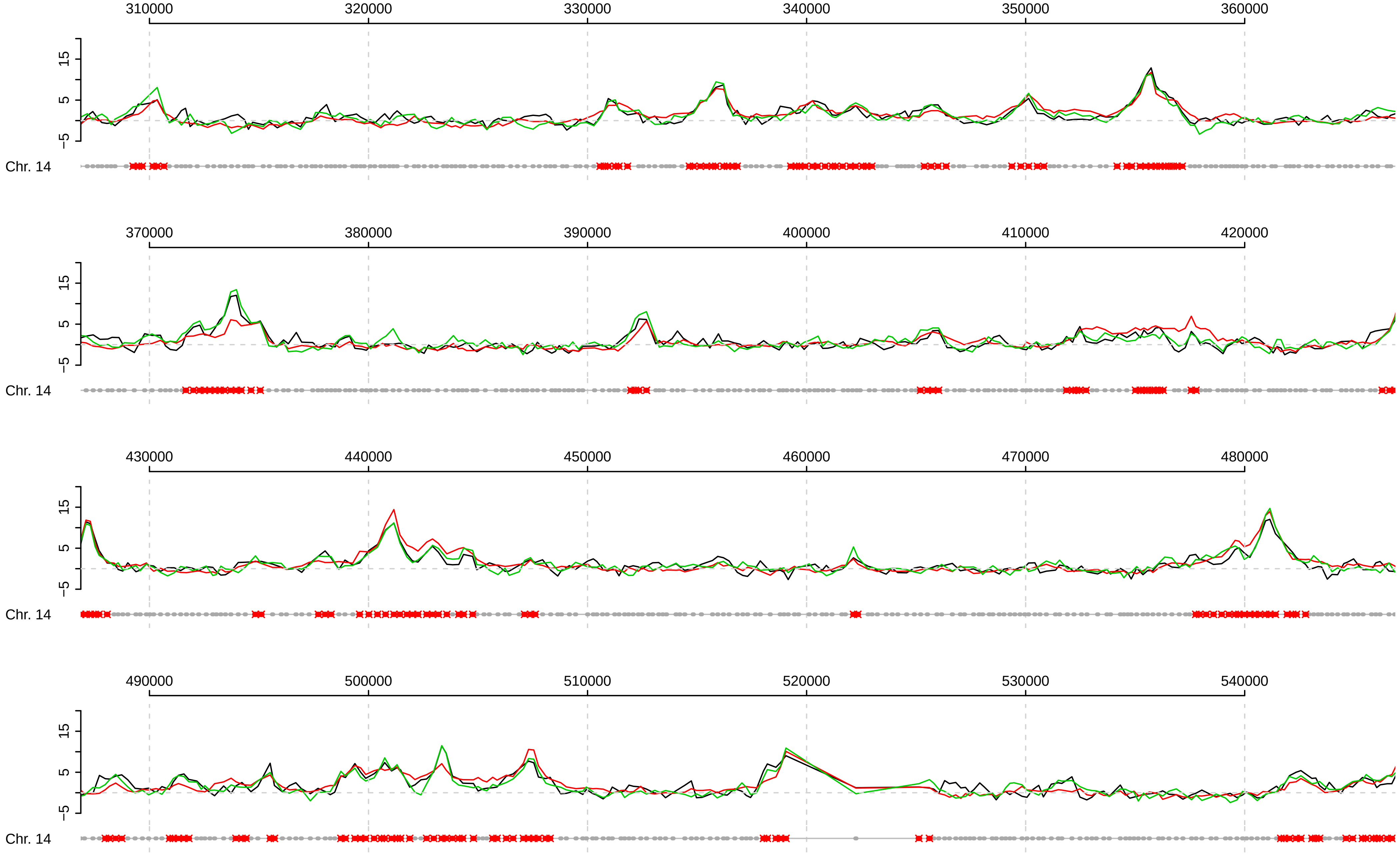
310000
320000
330000
340000
350000
360000
15
5
−5
Chr. 14
370000
380000
390000
400000
410000
420000
15
5
−5
Chr. 14
430000
440000
450000
460000
470000
480000
15
5
−5
Chr. 14
490000
500000
510000
520000
530000
540000
15
5
−5
Chr. 14

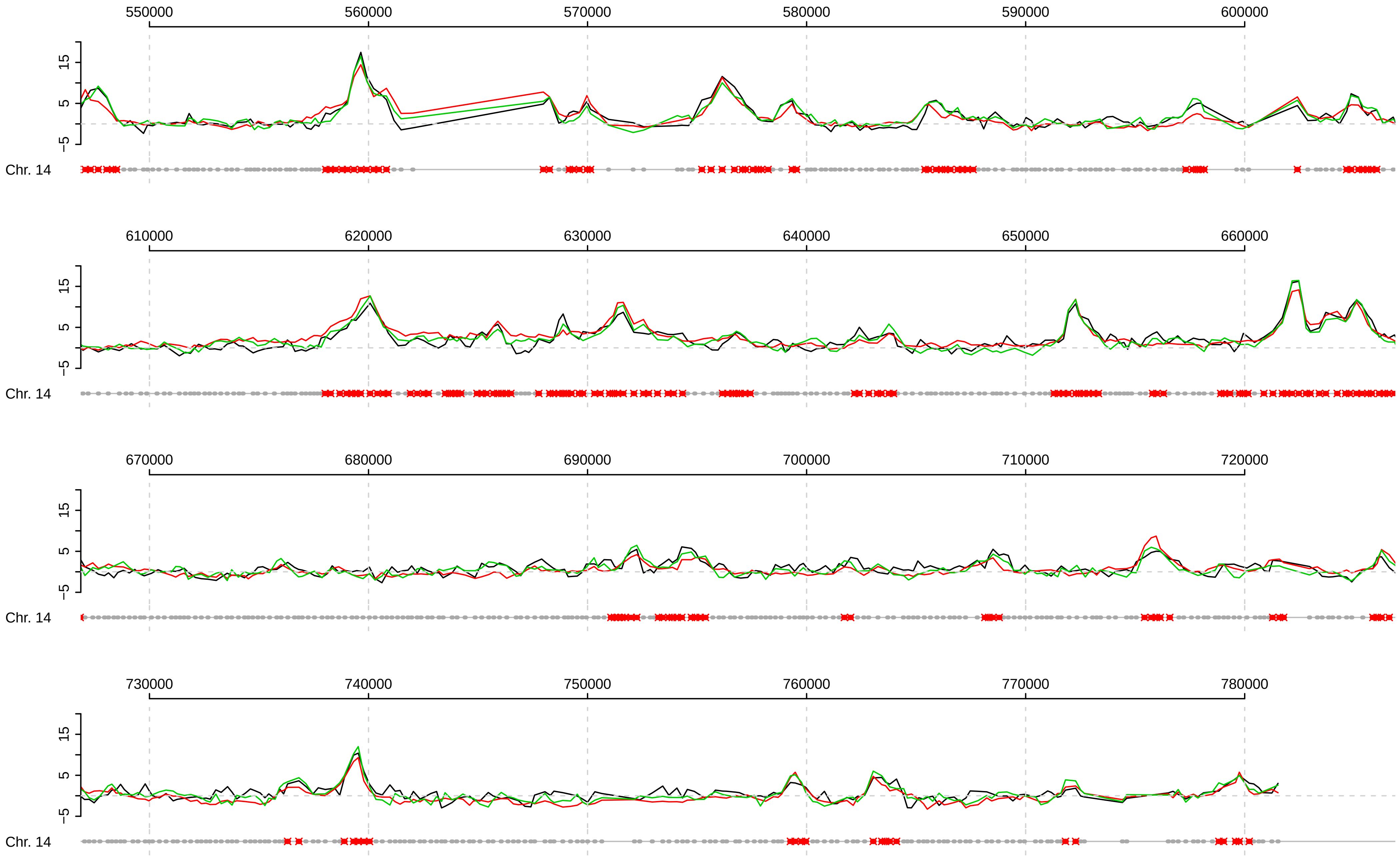
550000
560000
570000
580000
590000
600000
15
5
−5
Chr. 14
610000
620000
630000
640000
650000
660000
Chr. 14
670000
680000
690000
700000
710000
720000
Chr. 14
730000
740000
750000
760000
770000
780000
Chr. 14

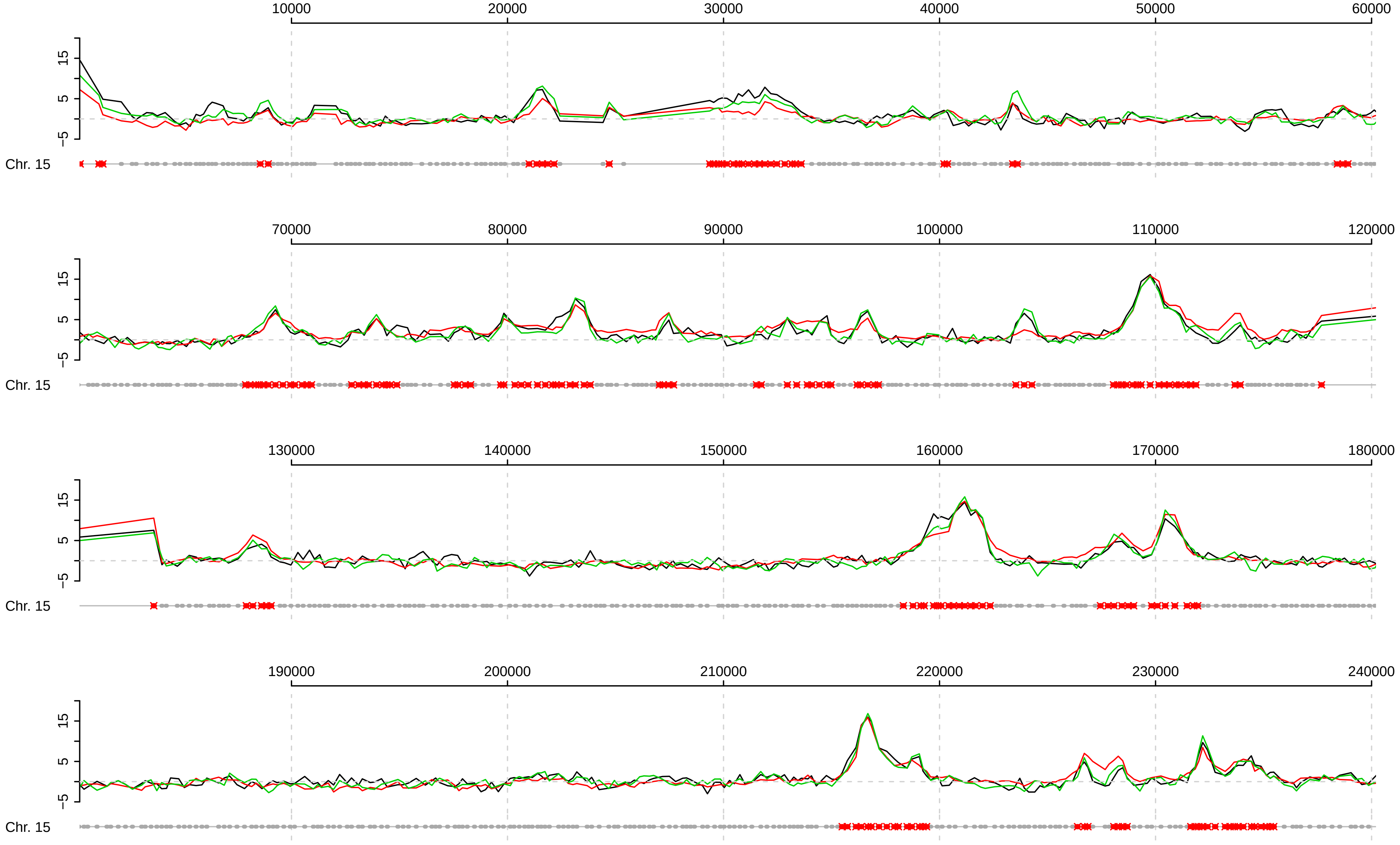

10000
20000
30000
40000
50000
60000
15
5
−5
Chr. 15
70000
80000
90000
100000
110000
120000
15
5
−5
Chr. 15
130000
140000
150000
160000
170000
180000
15
5
−5
Chr. 15
190000
200000
210000
220000
230000
240000
15
5
−5
Chr. 15

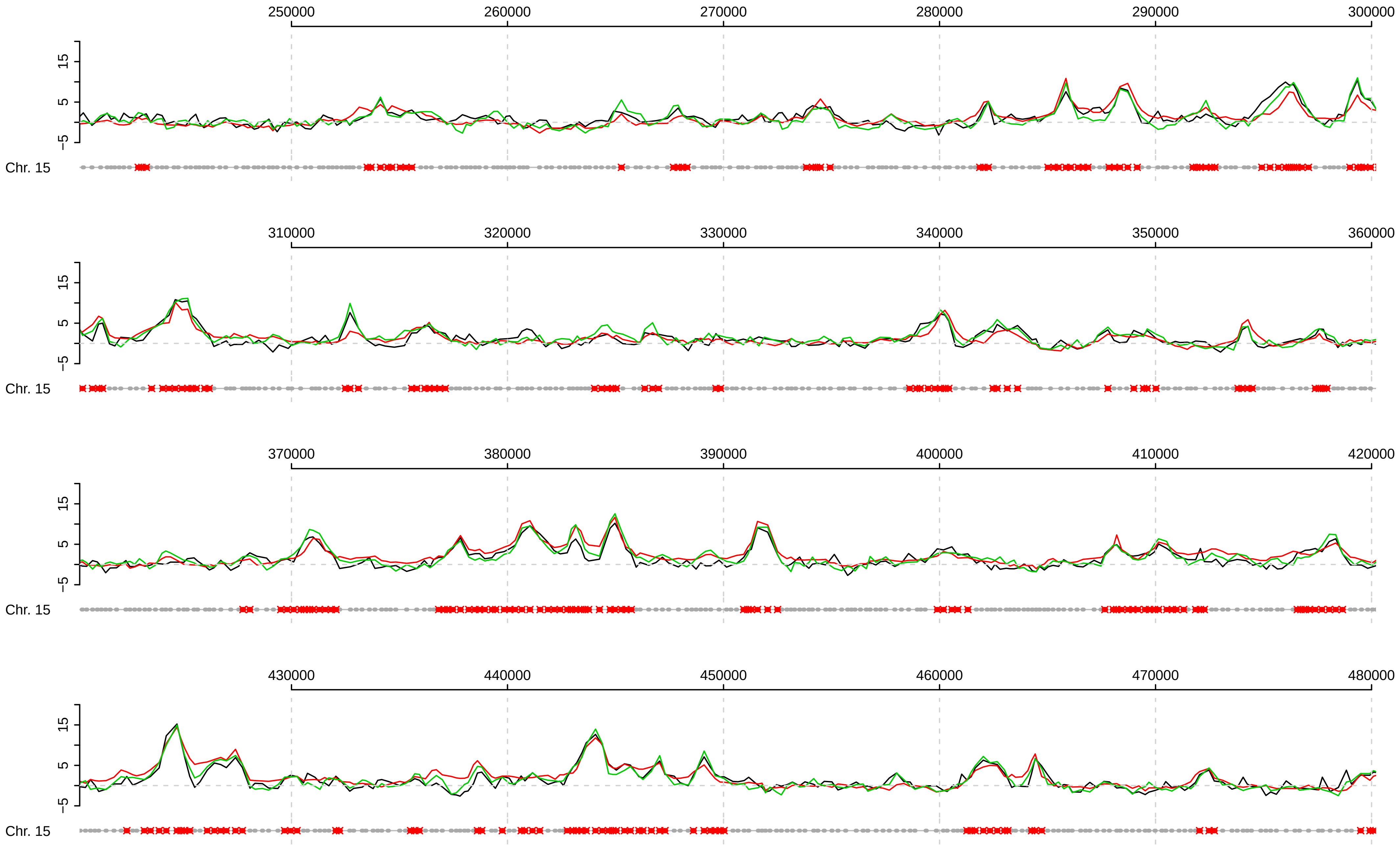
250000
260000
270000
280000
290000
300000
15
5
−5
Chr. 15
310000
320000
330000
340000
350000
360000
15
5
−5
Chr. 15
370000
380000
390000
400000
410000
420000
15
5
−5
Chr. 15
430000
440000
450000
460000
470000
480000
15
5
−5
Chr. 15

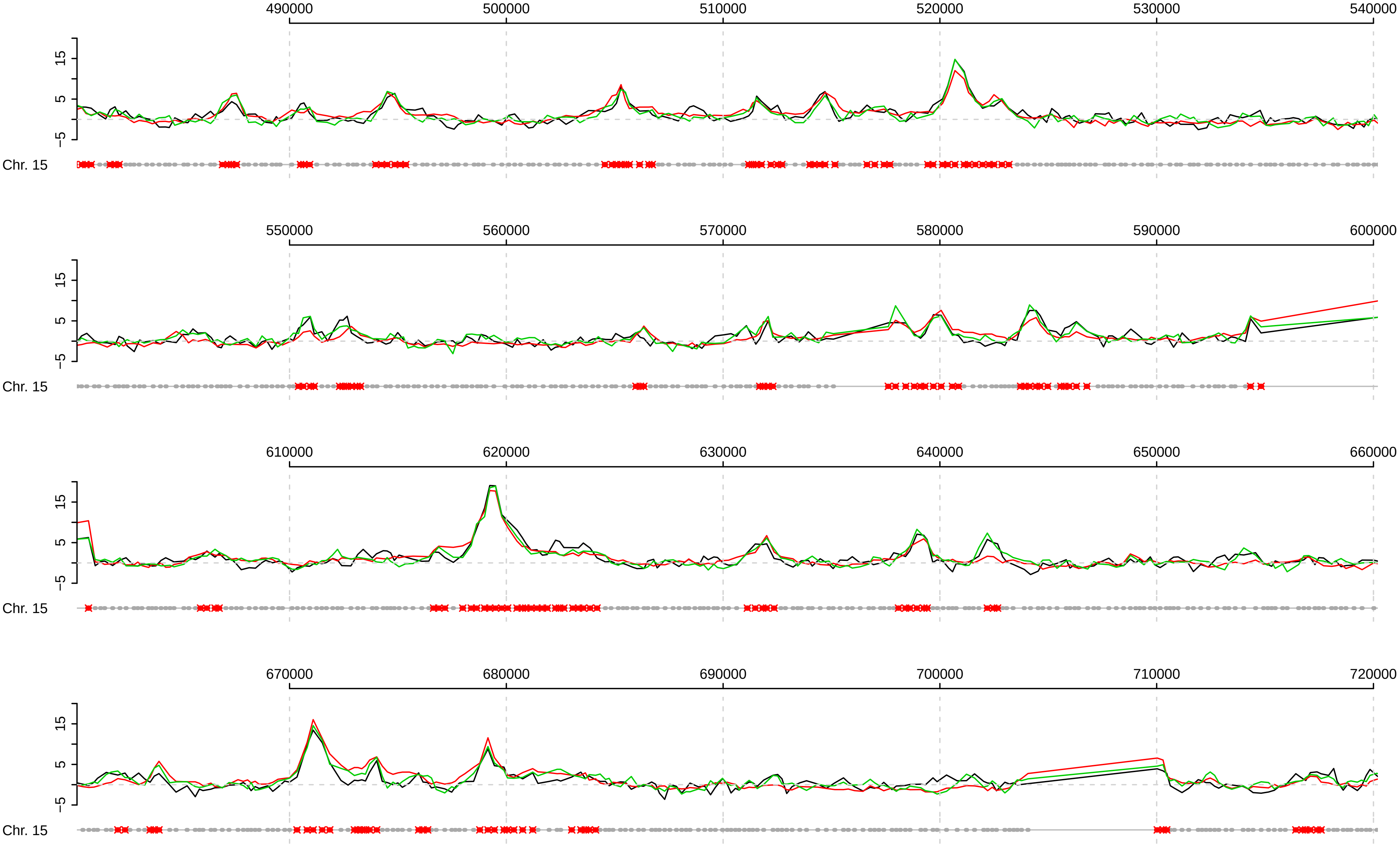
490000
500000
510000
520000
530000
540000
550000
560000
570000
580000
590000
600000
610000
620000
630000
640000
650000
660000
670000
680000
690000
700000
710000
720000
15
5
−5
Chr. 15

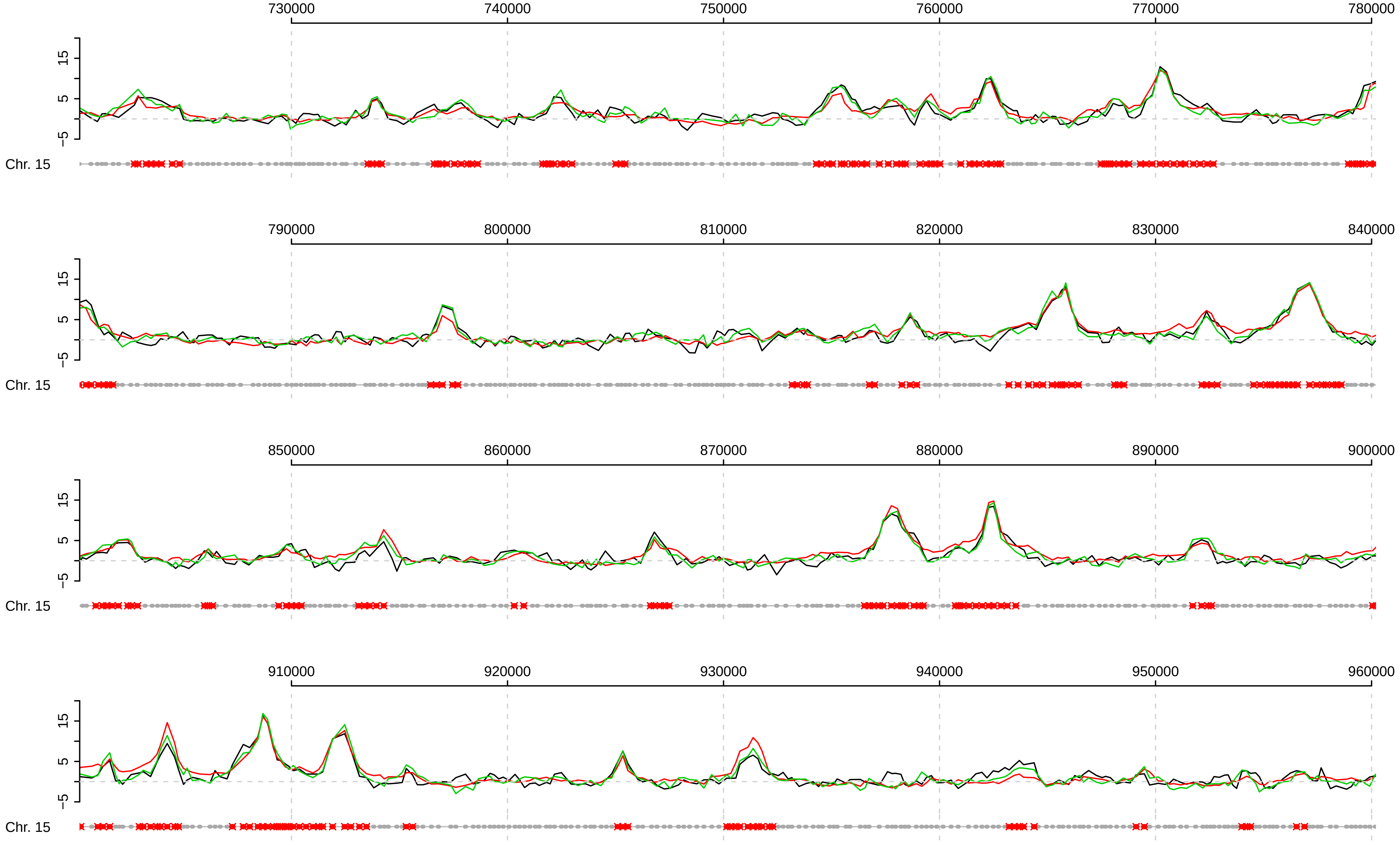

730000
740000
750000
760000
770000
780000
15
5
−5
Chr. 15
790000
800000
810000
820000
830000
840000
15
5
−5
Chr. 15
850000
860000
870000
880000
890000
900000
15
5
−5
Chr. 15
910000
920000
930000
940000
950000
960000
15
5
−5
Chr. 15

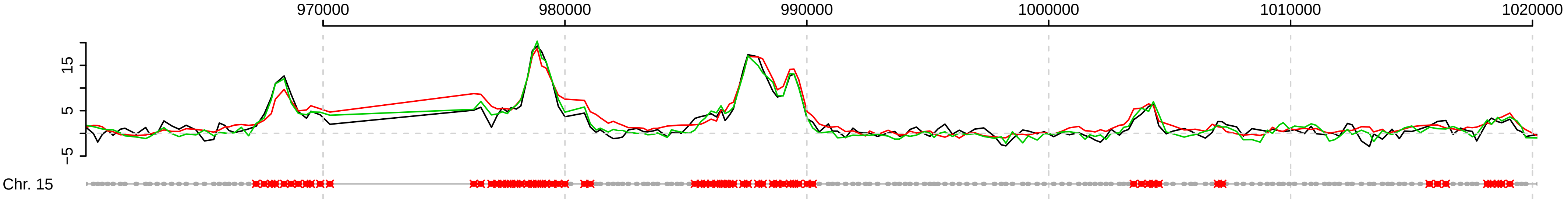

970000
980000
990000
1000000
1010000
1020000
15
5
−5
Chr. 15

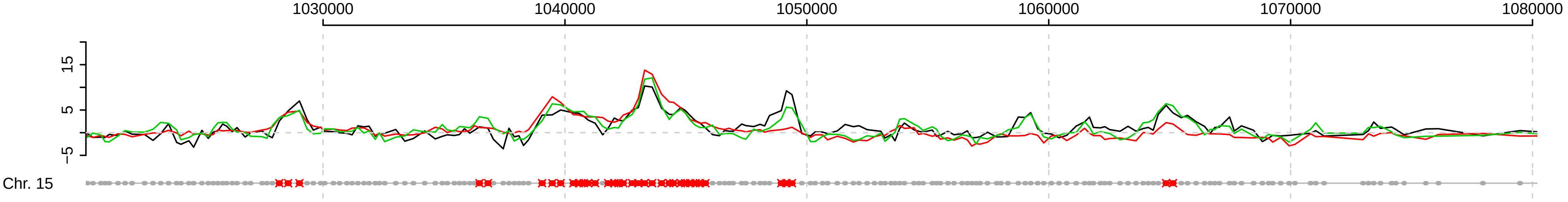

1030000
1040000
1050000
1060000
1070000
1080000
15
5
−5
Chr. 15

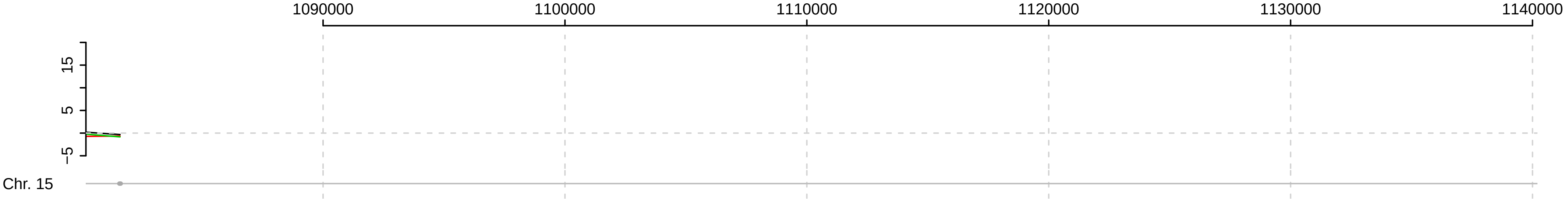

1090000
1100000
1110000
1120000
1130000
1140000
15
5
−5
Chr. 15

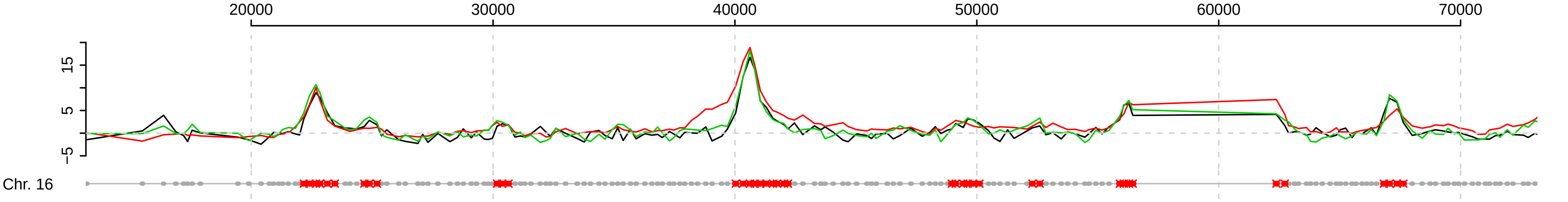

20000
30000
40000
50000
60000
70000
15
5
−5
Chr. 16

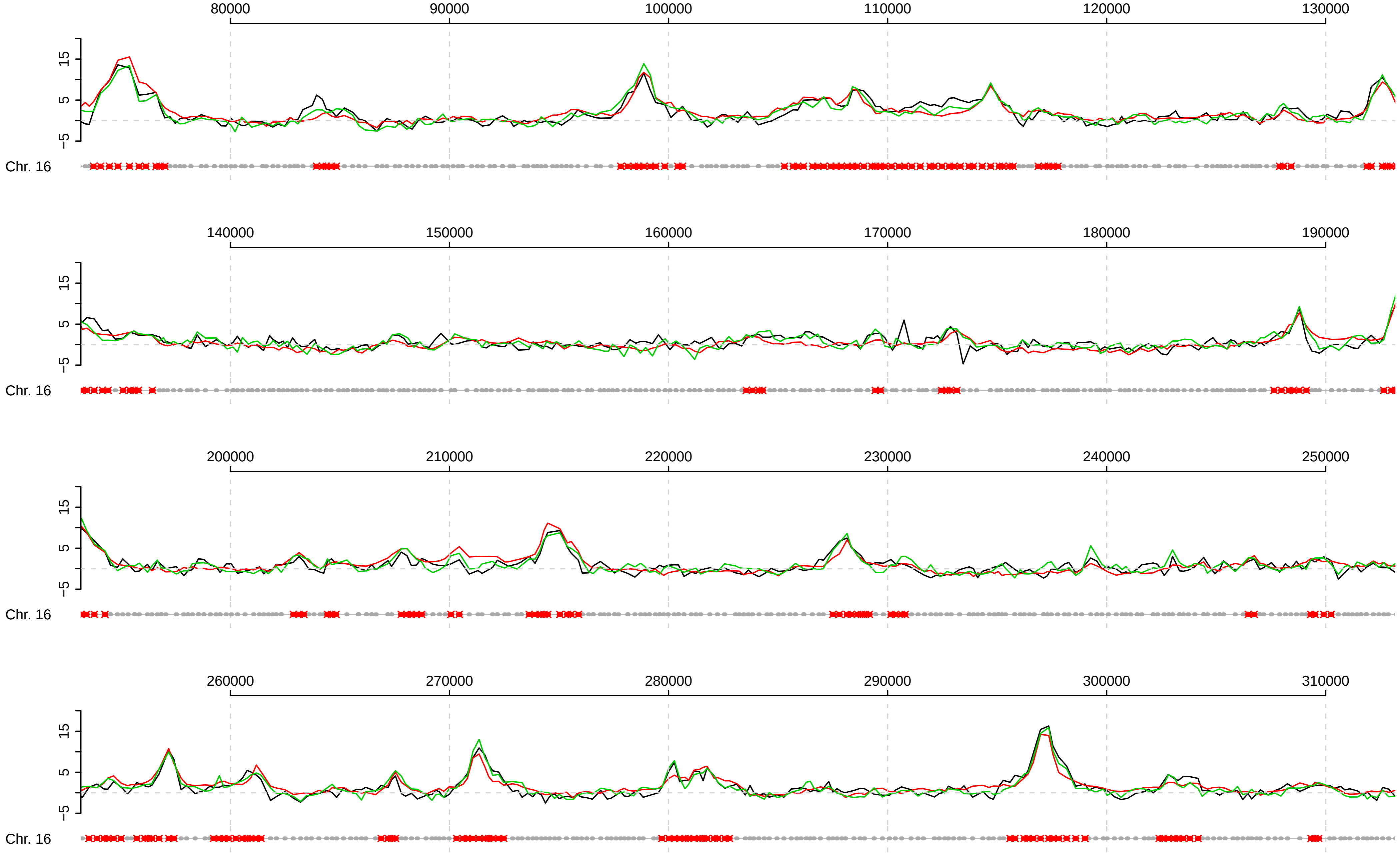

80000
90000
100000
110000
120000
130000
15
5
−5
Chr. 16
140000
150000
160000
170000
180000
190000
15
5
−5
Chr. 16
200000
210000
220000
230000
240000
250000
15
5
−5
Chr. 16
260000
270000
280000
290000
300000
310000
15
5
−5
Chr. 16

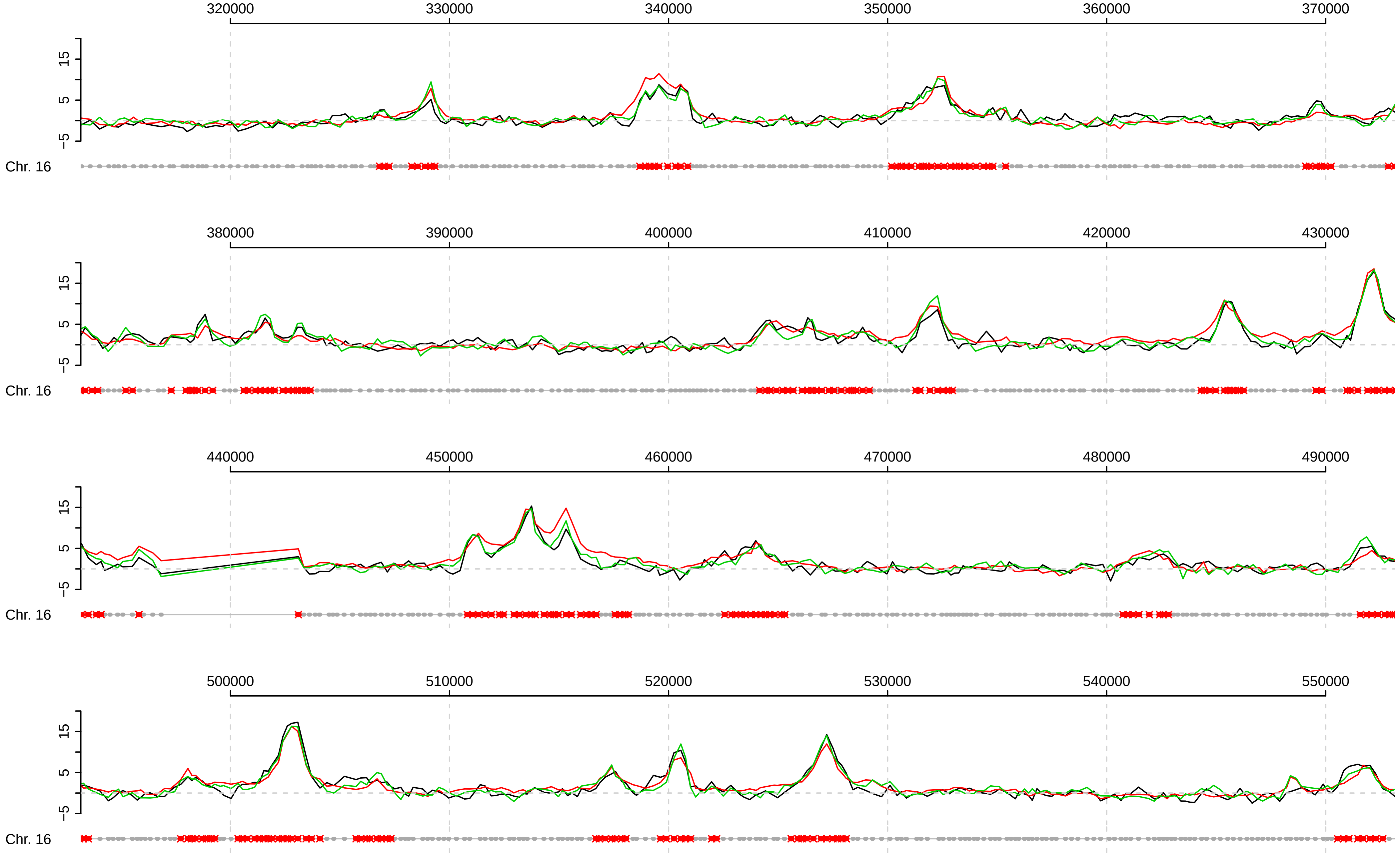

320000
330000
340000
350000
360000
370000
15
5
−5
Chr. 16
380000
390000
400000
410000
420000
430000
15
5
−5
Chr. 16
440000
450000
460000
470000
480000
490000
15
5
−5
Chr. 16
500000
510000
520000
530000
540000
550000
15
5
−5
Chr. 16

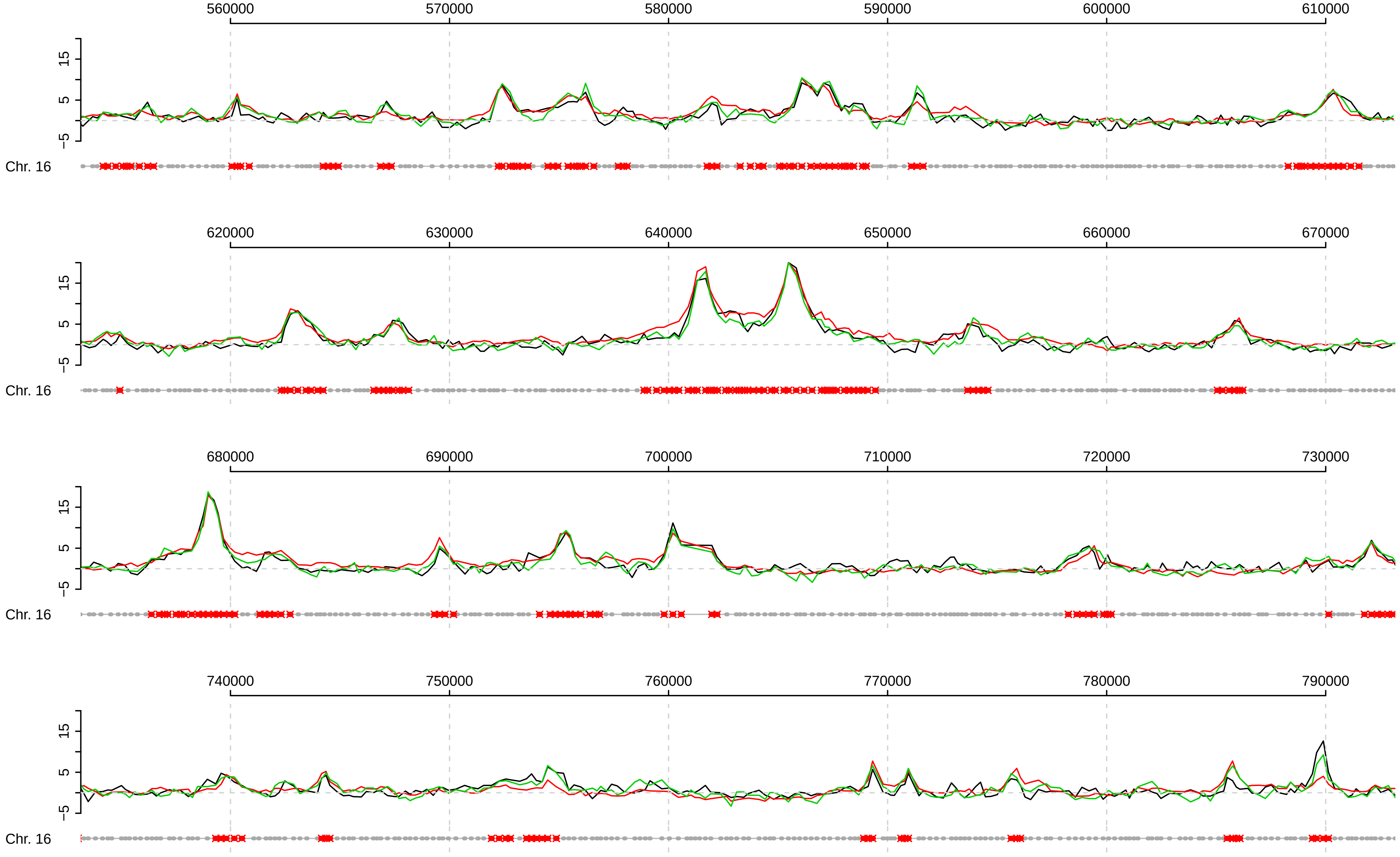

560000
570000
580000
590000
600000
610000
15
5
−5
Chr. 16
620000
630000
640000
650000
660000
670000
15
5
−5
Chr. 16
680000
690000
700000
710000
720000
730000
15
5
−5
Chr. 16
740000
750000
760000
770000
780000
790000
15
5
−5
Chr. 16

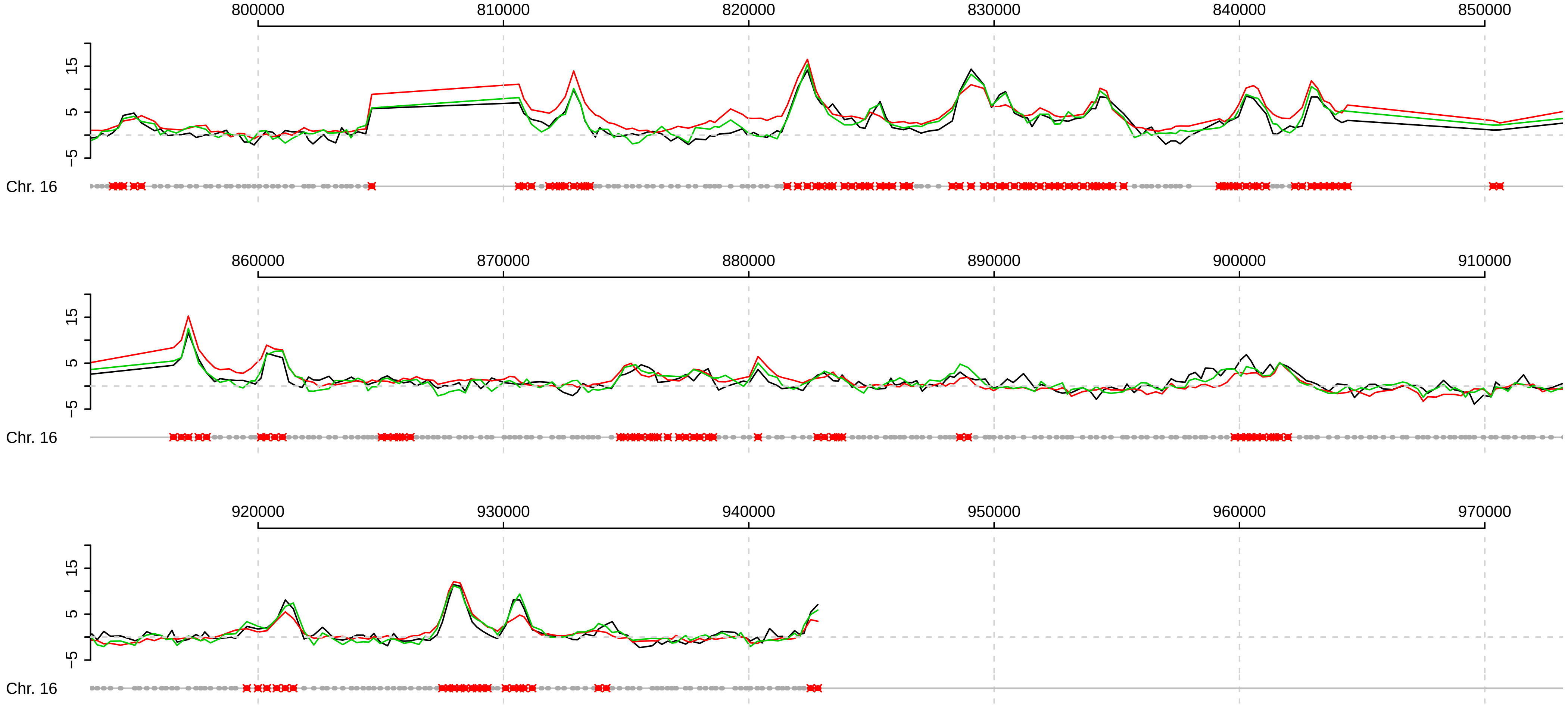
800000
810000
820000
830000
840000
850000
15
5
−5
Chr. 16
860000
870000
880000
890000
900000
910000
15
5
−5
Chr. 16
920000
930000
940000
950000
960000
970000
15
5
−5
Chr. 16

The following plots show Gcn5 enrichment over the whole genome in cells treated with UV, time point 1 (T1). The three line colours show three replicate datasets. The red crosses show probes detected as enriched.

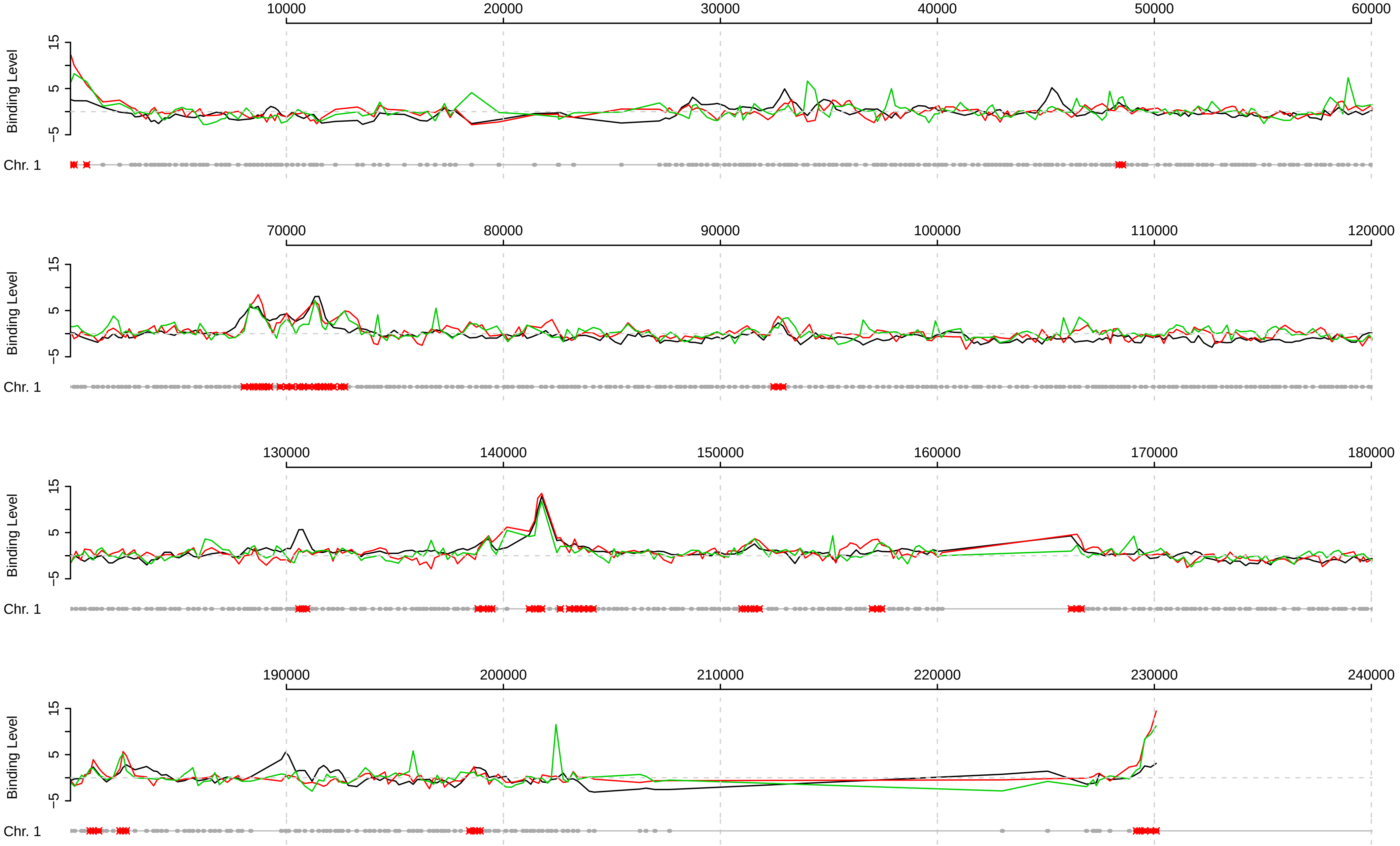

10000
20000
30000
40000
50000
60000
Binding Level
15
5
−5
Chr. 1
70000
80000
90000
100000
110000
120000
Binding Level
15
5
−5
Chr. 1
130000
140000
150000
160000
170000
180000
Binding Level
15
5
−5
Chr. 1
190000
200000
210000
220000
230000
240000
Binding Level
15
5
−5
Chr. 1

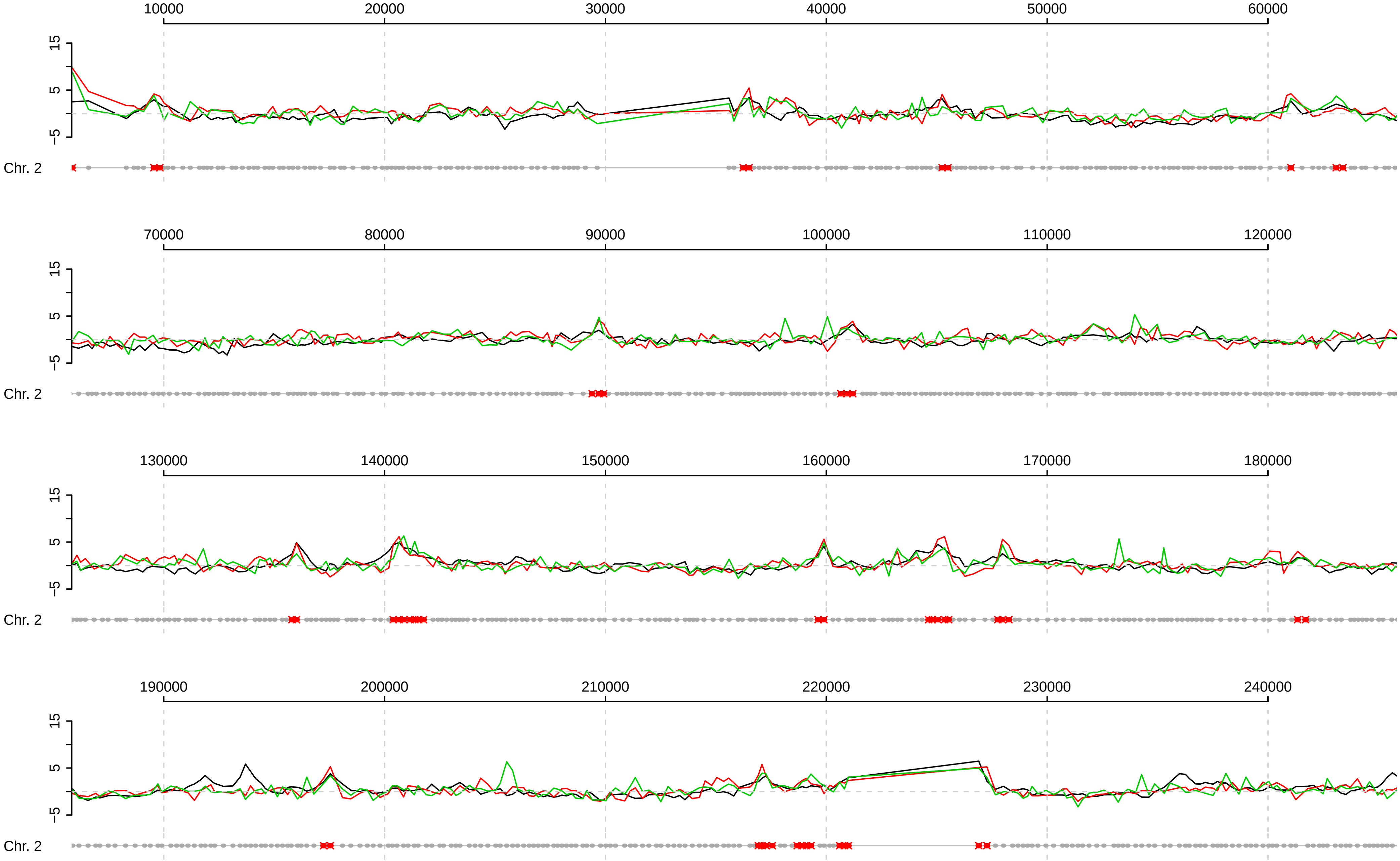

10000
20000
30000
40000
50000
60000
15
5
−5
Chr. 2
70000
80000
90000
100000
110000
120000
15
5
−5
Chr. 2
130000
140000
150000
160000
170000
180000
15
5
−5
Chr. 2
190000
200000
210000
220000
230000
240000
15
5
−5
Chr. 2

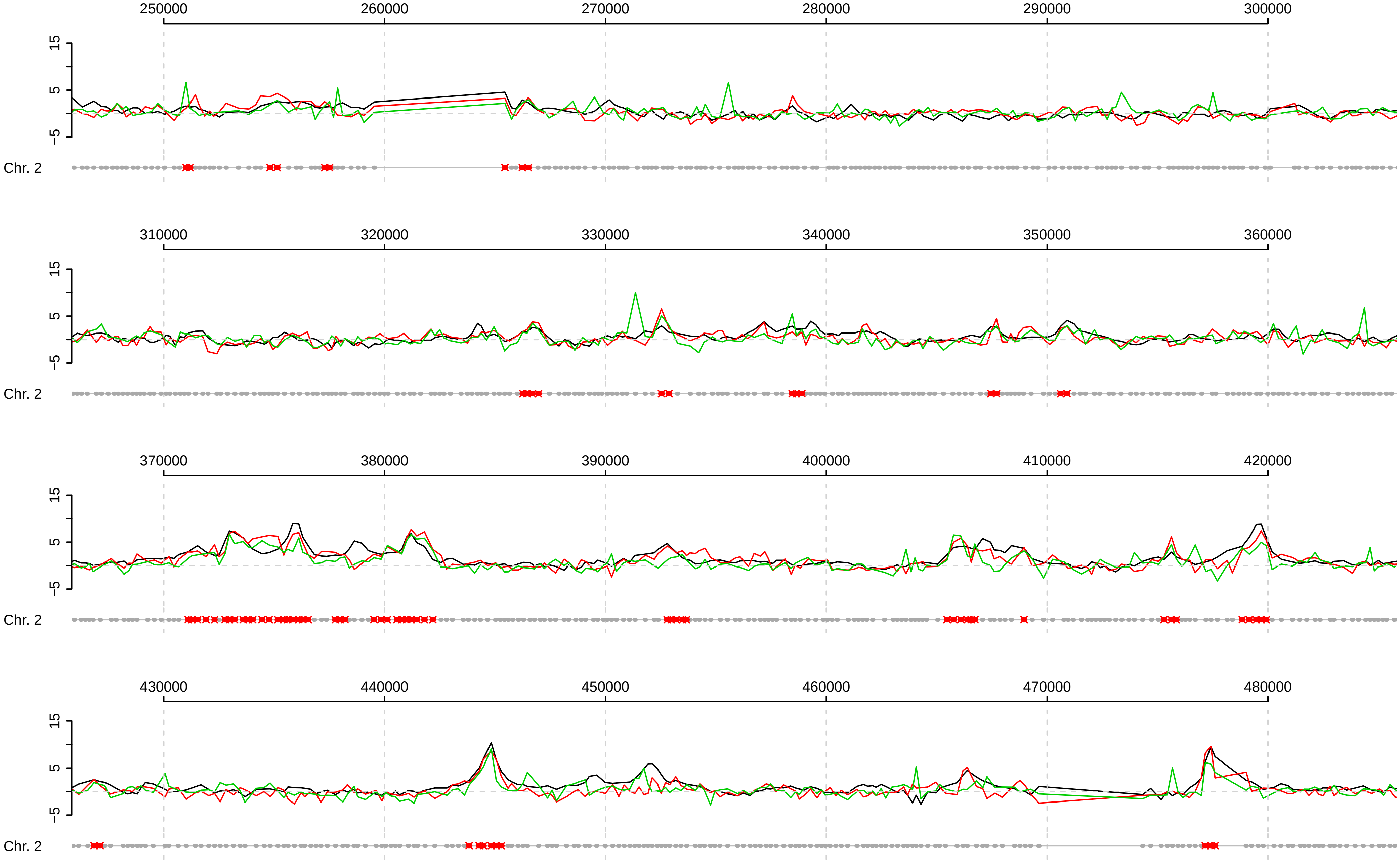
250000
260000
270000
280000
290000
300000
15
5
−5
Chr. 2
310000
320000
330000
340000
350000
360000
15
5
−5
Chr. 2
370000
380000
390000
400000
410000
420000
15
5
−5
Chr. 2
430000
440000
450000
460000
470000
480000
15
5
−5
Chr. 2

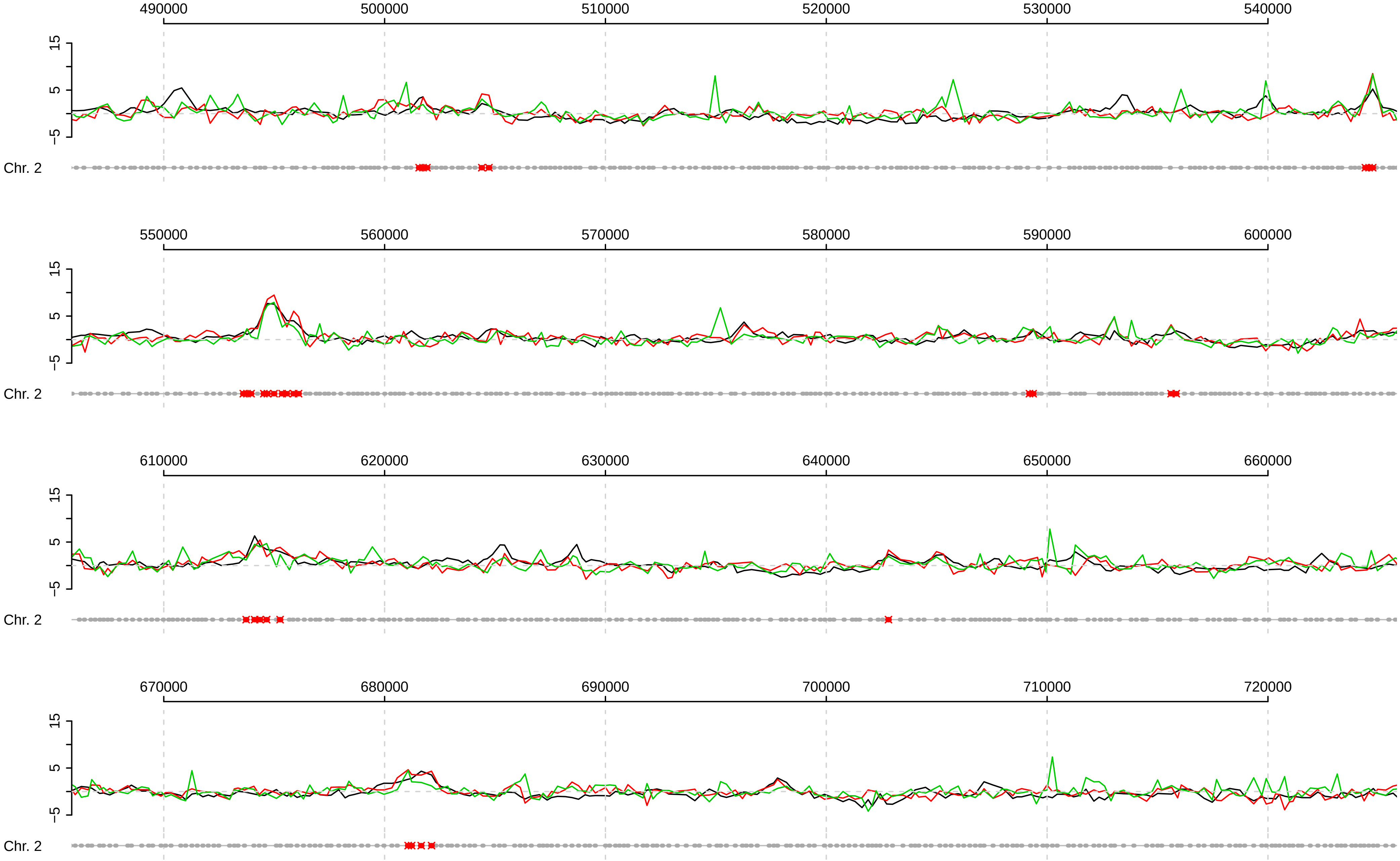

490000
500000
510000
520000
530000
540000
15
5
−5
Chr. 2
550000
560000
570000
580000
590000
600000
15
5
−5
Chr. 2
610000
620000
630000
640000
650000
660000
15
5
−5
Chr. 2
670000
680000
690000
700000
710000
720000
15
5
−5
Chr. 2

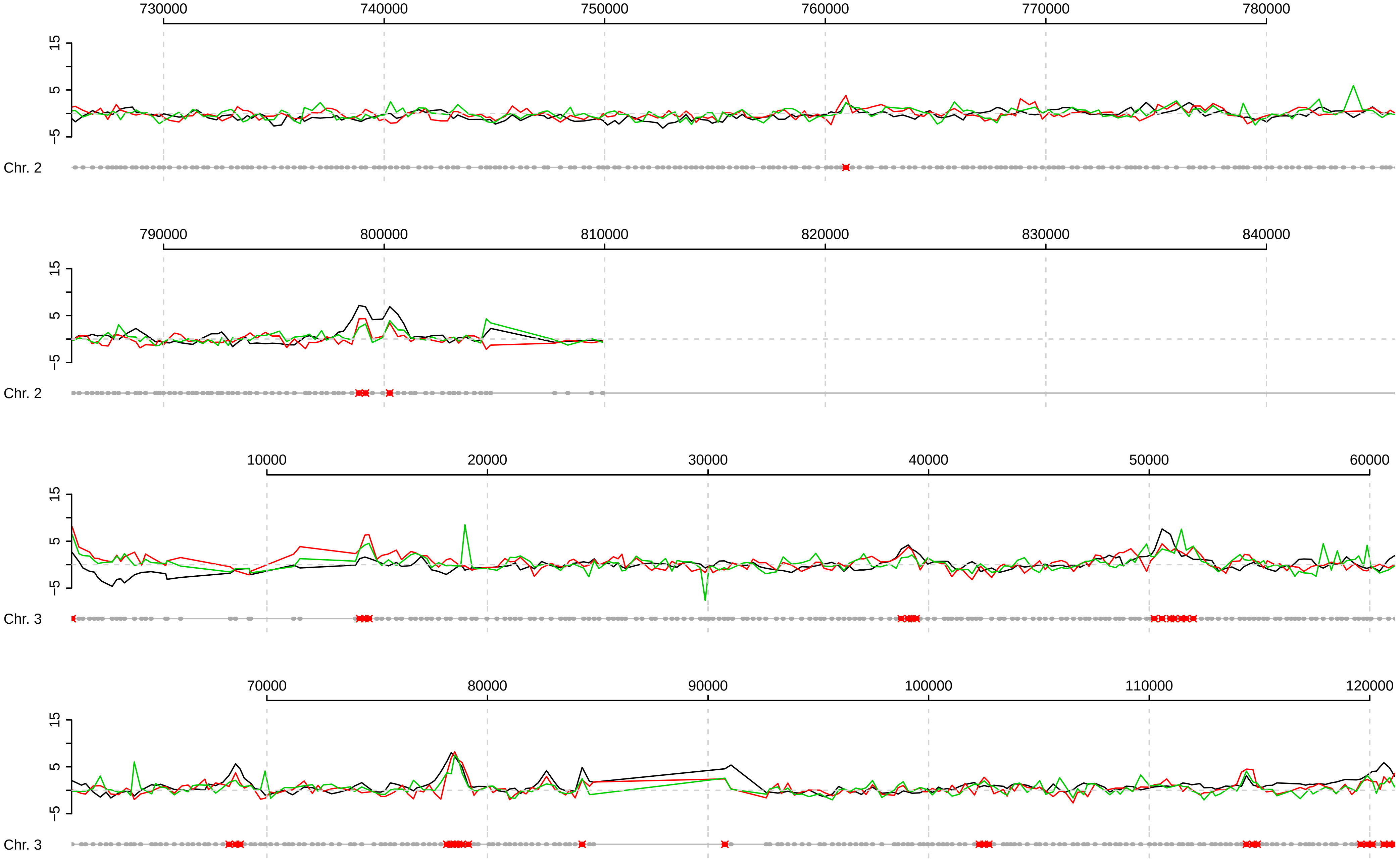

730000
740000
750000
760000
770000
780000
15
5
−5
Chr. 2
790000
800000
810000
820000
830000
840000
15
5
−5
Chr. 2
10000
20000
30000
40000
50000
60000
15
5
−5
Chr. 3
70000
80000
90000
100000
110000
120000
15
5
−5
Chr. 3

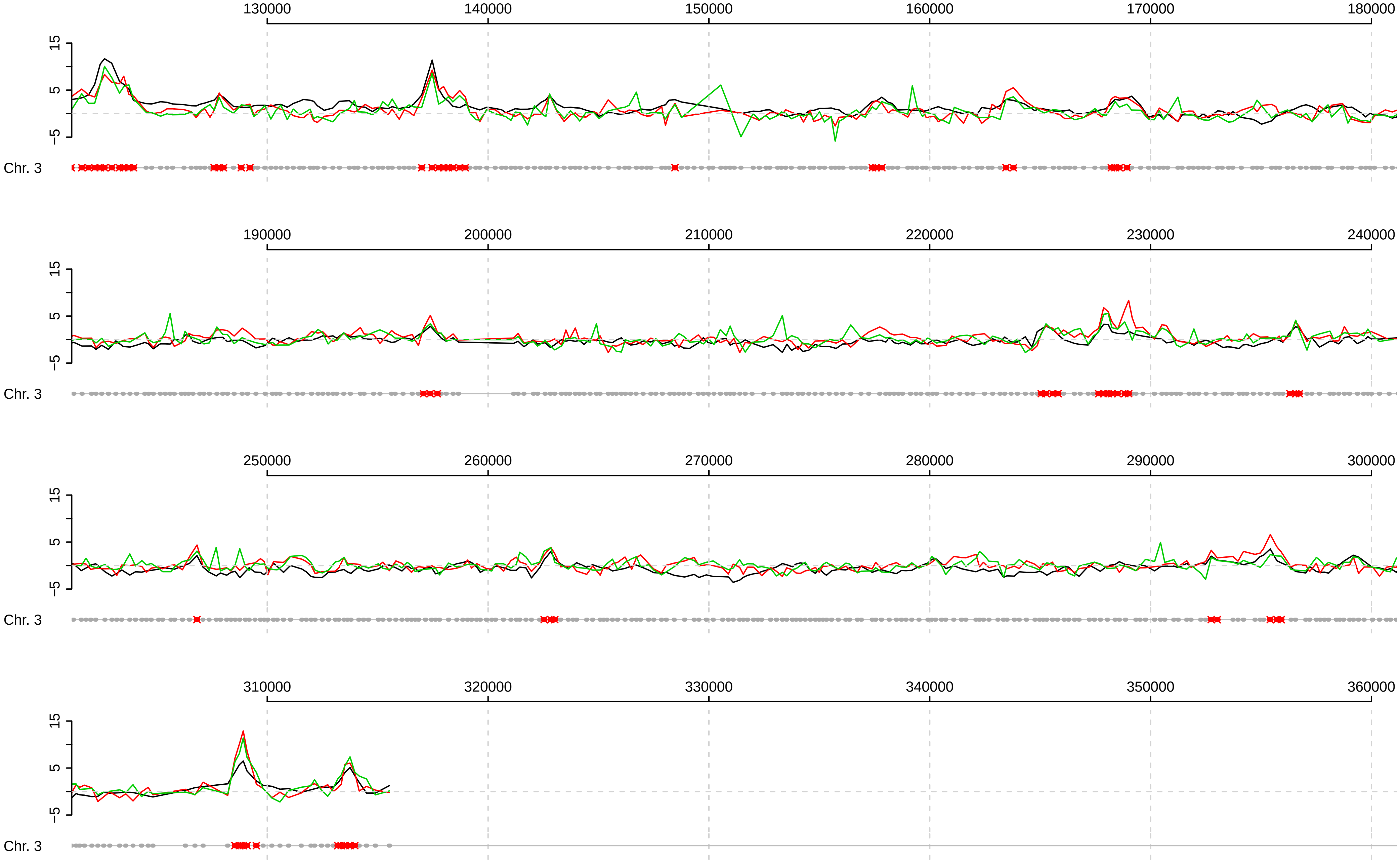
130000
140000
150000
160000
170000
180000
15
5
−5
Chr. 3
190000
200000
210000
220000
230000
240000
15
5
−5
Chr. 3
250000
260000
270000
280000
290000
300000
15
5
−5
Chr. 3
310000
320000
330000
340000
350000
360000
15
5
−5
Chr. 3

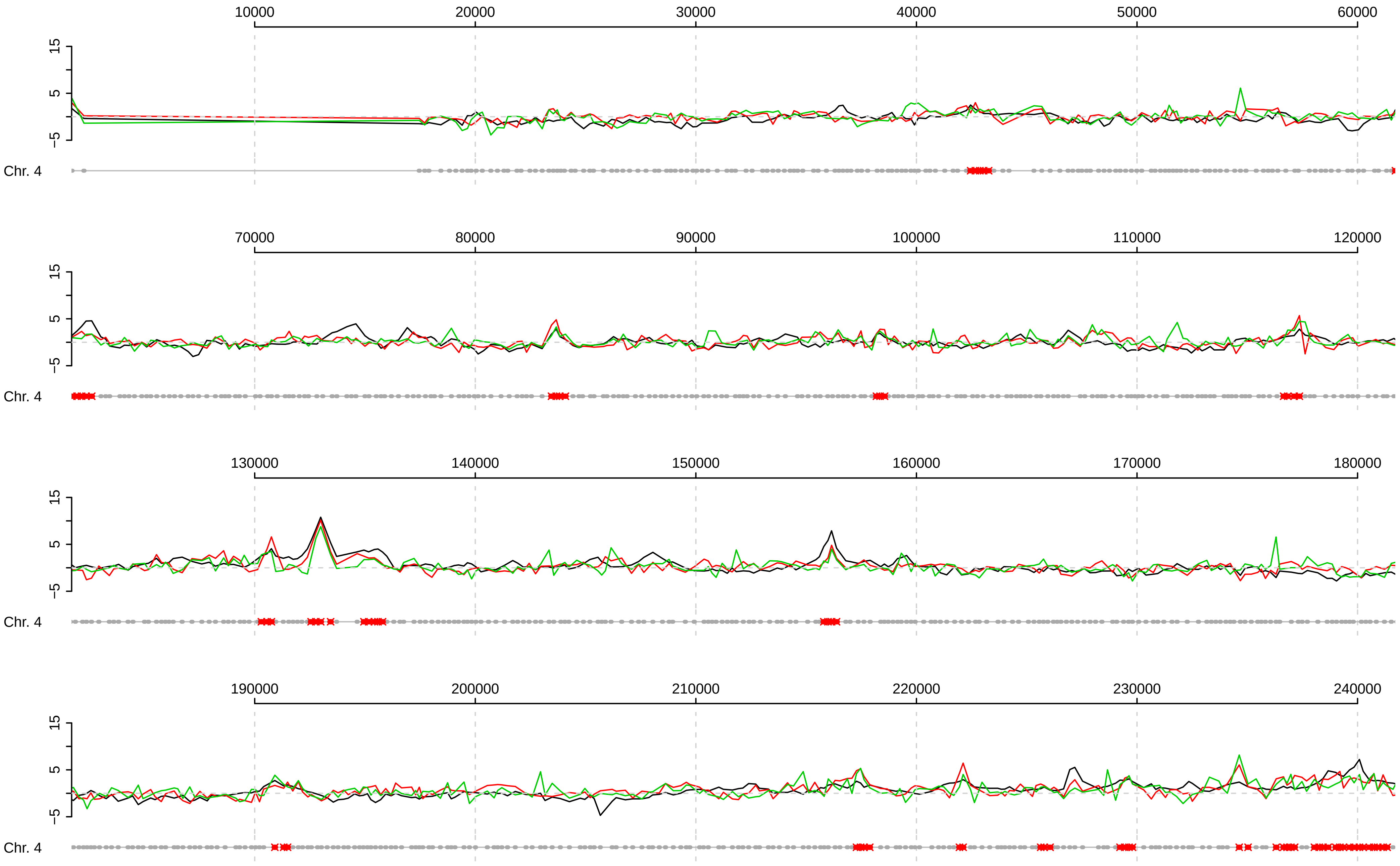

10000
20000
30000
40000
50000
60000
15
5
−5
Chr. 4
70000
80000
90000
100000
110000
120000
15
5
−5
Chr. 4
130000
140000
150000
160000
170000
180000
15
5
−5
Chr. 4
190000
200000
210000
220000
230000
240000
15
5
−5
Chr. 4

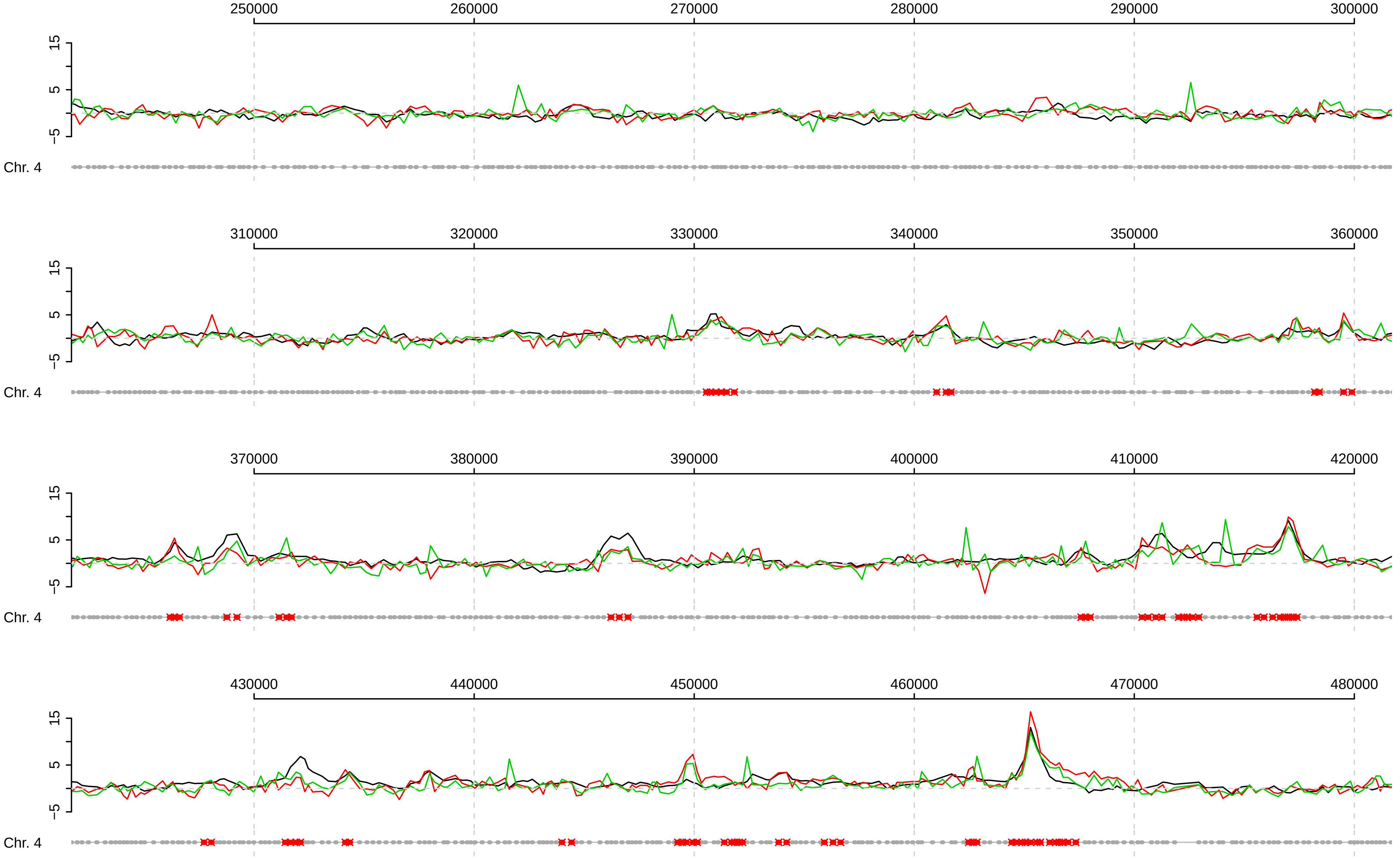

250000
260000
270000
280000
290000
300000
15
5
−5
Chr. 4
310000
320000
330000
340000
350000
360000
370000
380000
390000
400000
410000
420000
430000
440000
450000
460000
470000
480000

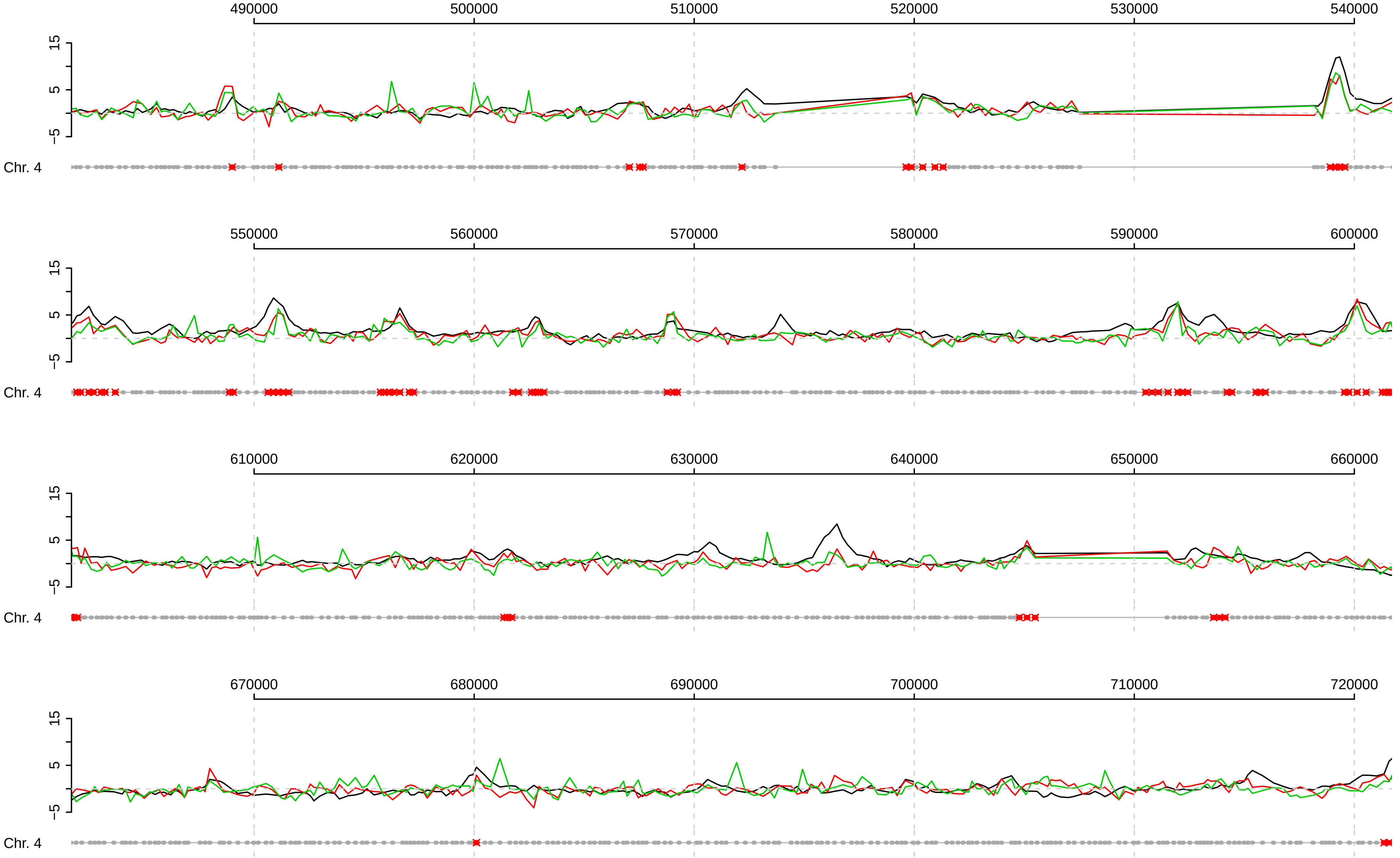
490000
500000
510000
520000
530000
540000
15
5
−5
Chr. 4
550000
560000
570000
580000
590000
600000
15
5
−5
Chr. 4
610000
620000
630000
640000
650000
660000
15
5
−5
Chr. 4
670000
680000
690000
700000
710000
720000
15
5
−5
Chr. 4

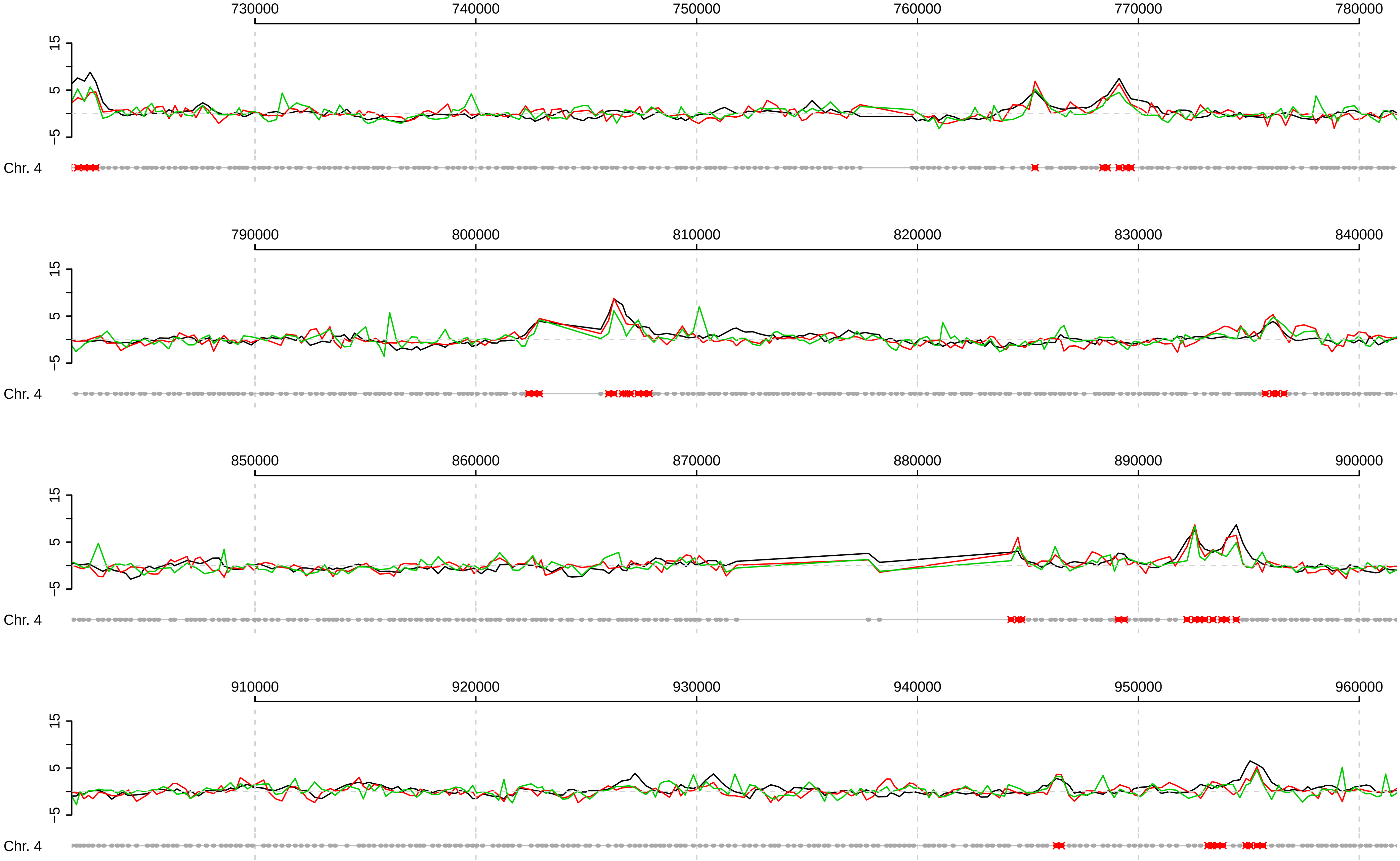

730000
740000
750000
760000
770000
780000
15
5
−5
Chr. 4
790000
800000
810000
820000
830000
840000
15
5
−5
Chr. 4
850000
860000
870000
880000
890000
900000
15
5
−5
Chr. 4
910000
920000
930000
940000
950000
960000
15
5
−5
Chr. 4

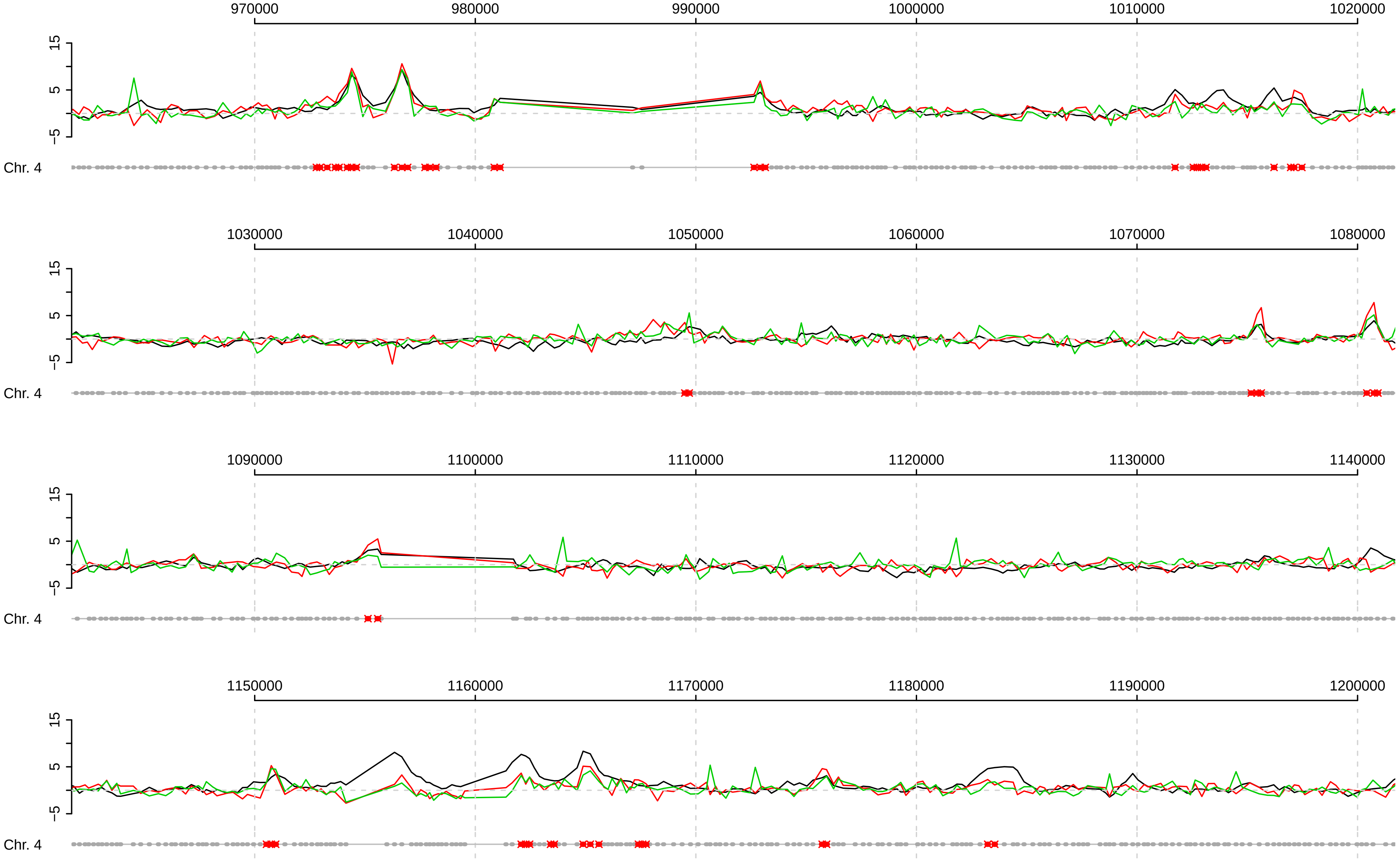
970000
980000
990000
1000000
1010000
1020000
15
5
−5
Chr. 4
1030000
1040000
1050000
1060000
1070000
1080000
15
5
−5
Chr. 4
1090000
1100000
1110000
1120000
1130000
1140000
15
5
−5
Chr. 4
1150000
1160000
1170000
1180000
1190000
1200000
15
5
−5
Chr. 4

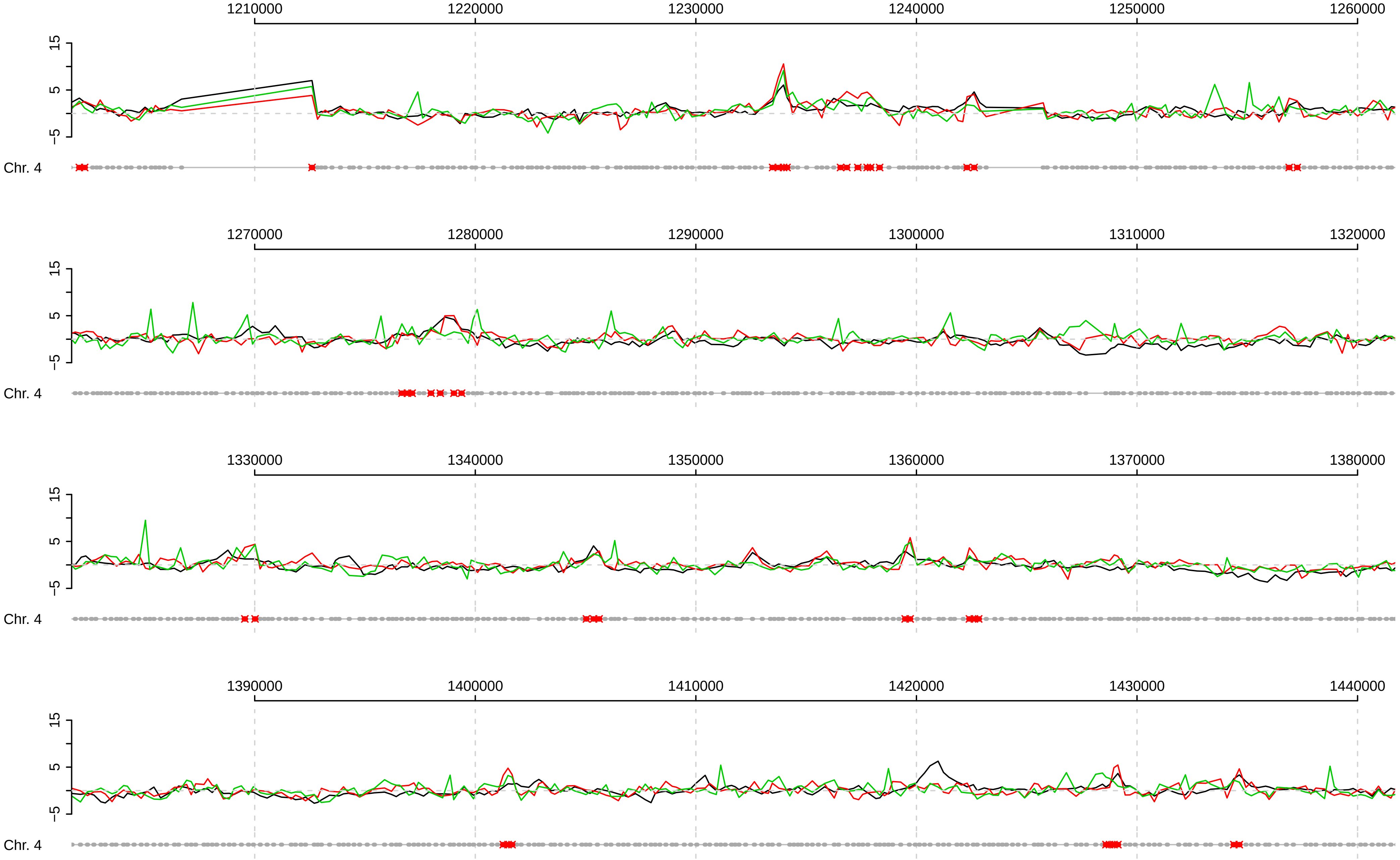
1210000
1220000
1230000
1240000
1250000
1260000
15
5
−5
Chr. 4
1270000
1280000
1290000
1300000
1310000
1320000
15
5
−5
Chr. 4
1330000
1340000
1350000
1360000
1370000
1380000
15
5
−5
Chr. 4
1390000
1400000
1410000
1420000
1430000
1440000
15
5
−5
Chr. 4

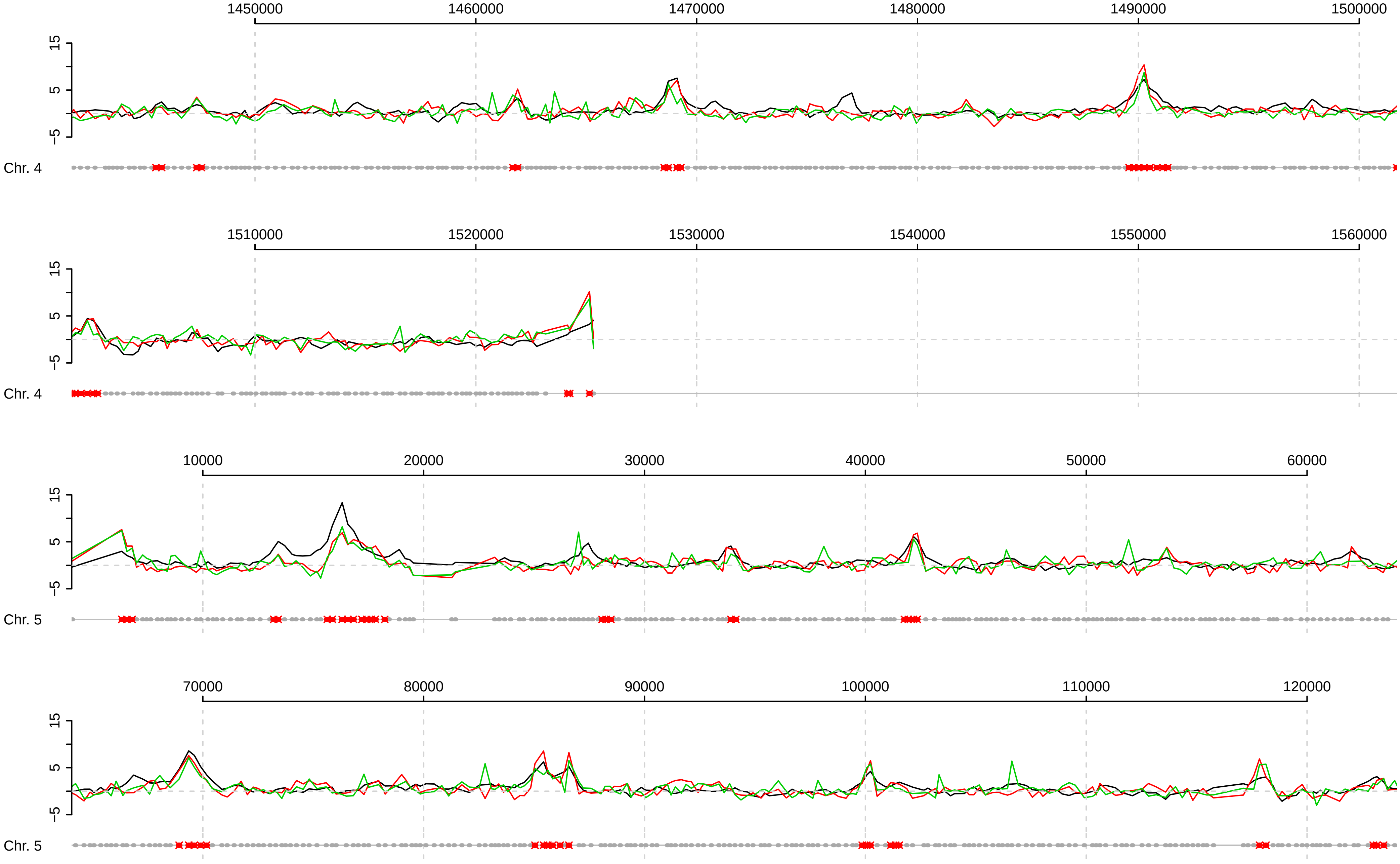

1450000
1460000
1470000
1480000
1490000
1500000
15
5
−5
Chr. 4
1510000
1520000
1530000
1540000
1550000
1560000
15
5
−5
Chr. 4
10000
20000
30000
40000
50000
60000
15
5
−5
Chr. 5
70000
80000
90000
100000
110000
120000
15
5
−5
Chr. 5

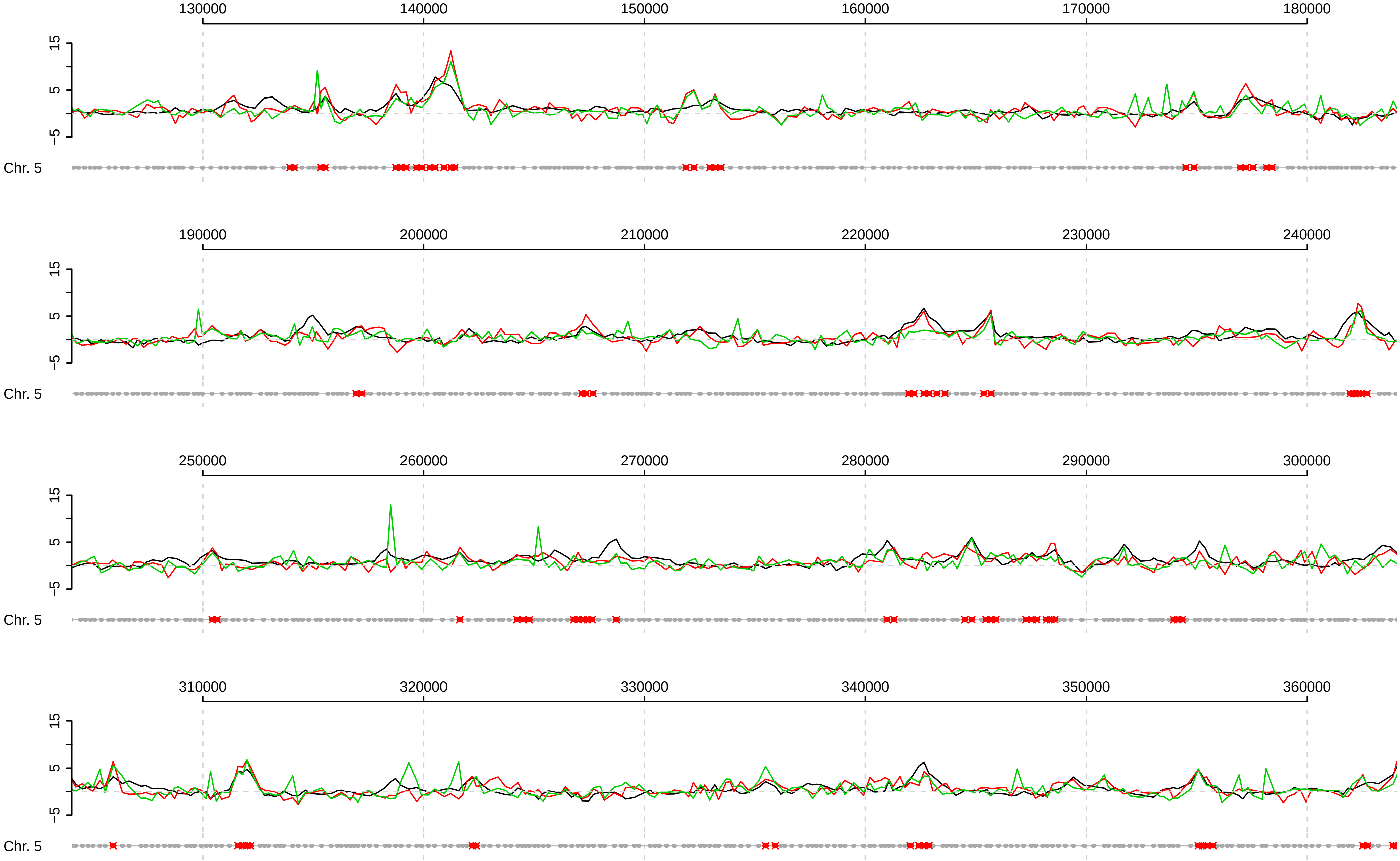

130000
140000
150000
160000
170000
180000
15
5
−5
Chr. 5
190000
200000
210000
220000
230000
240000
15
5
−5
Chr. 5
250000
260000
270000
280000
290000
300000
15
5
−5
Chr. 5
310000
320000
330000
340000
350000
360000
15
5
−5
Chr. 5

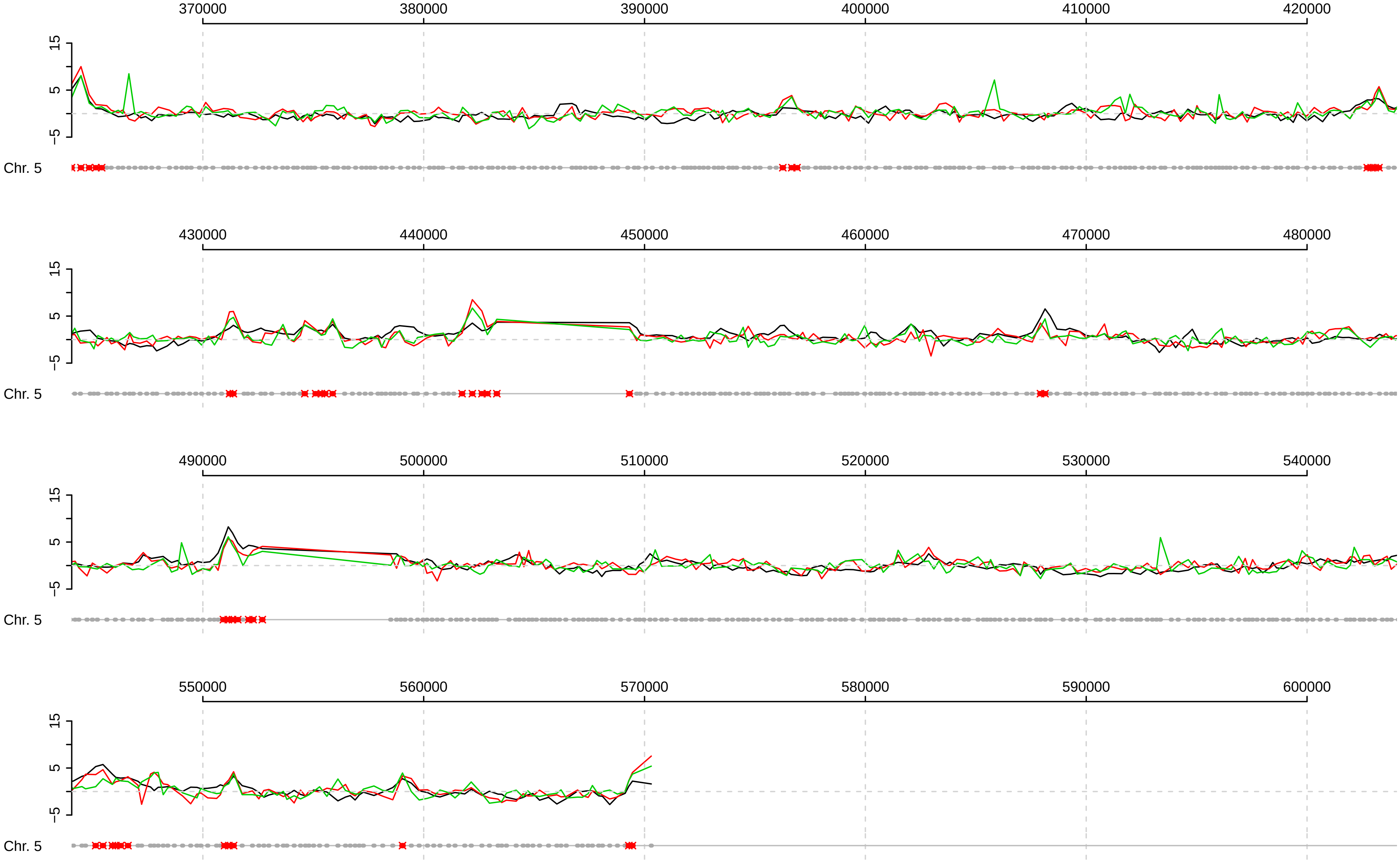
370000
380000
390000
400000
410000
420000
15
5
−5
Chr. 5
430000
440000
450000
460000
470000
480000
15
5
−5
Chr. 5
490000
500000
510000
520000
530000
540000
15
5
−5
Chr. 5
550000
560000
570000
580000
590000
600000
15
5
−5
Chr. 5

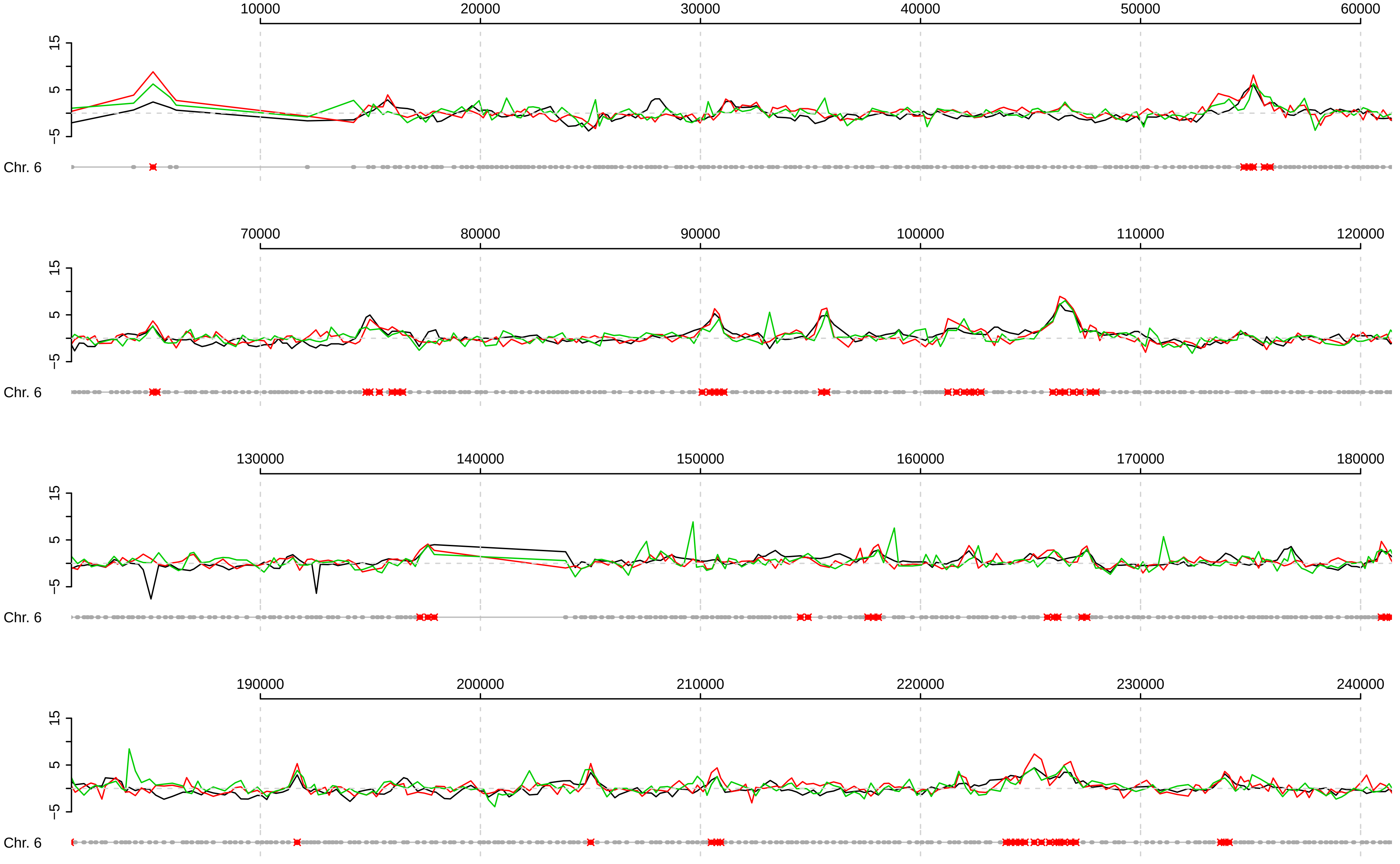

10000
20000
30000
40000
50000
60000
15
5
−5
Chr. 6
70000
80000
90000
100000
110000
120000
15
5
−5
Chr. 6
130000
140000
150000
160000
170000
180000
15
5
−5
Chr. 6
190000
200000
210000
220000
230000
240000
15
5
−5
Chr. 6

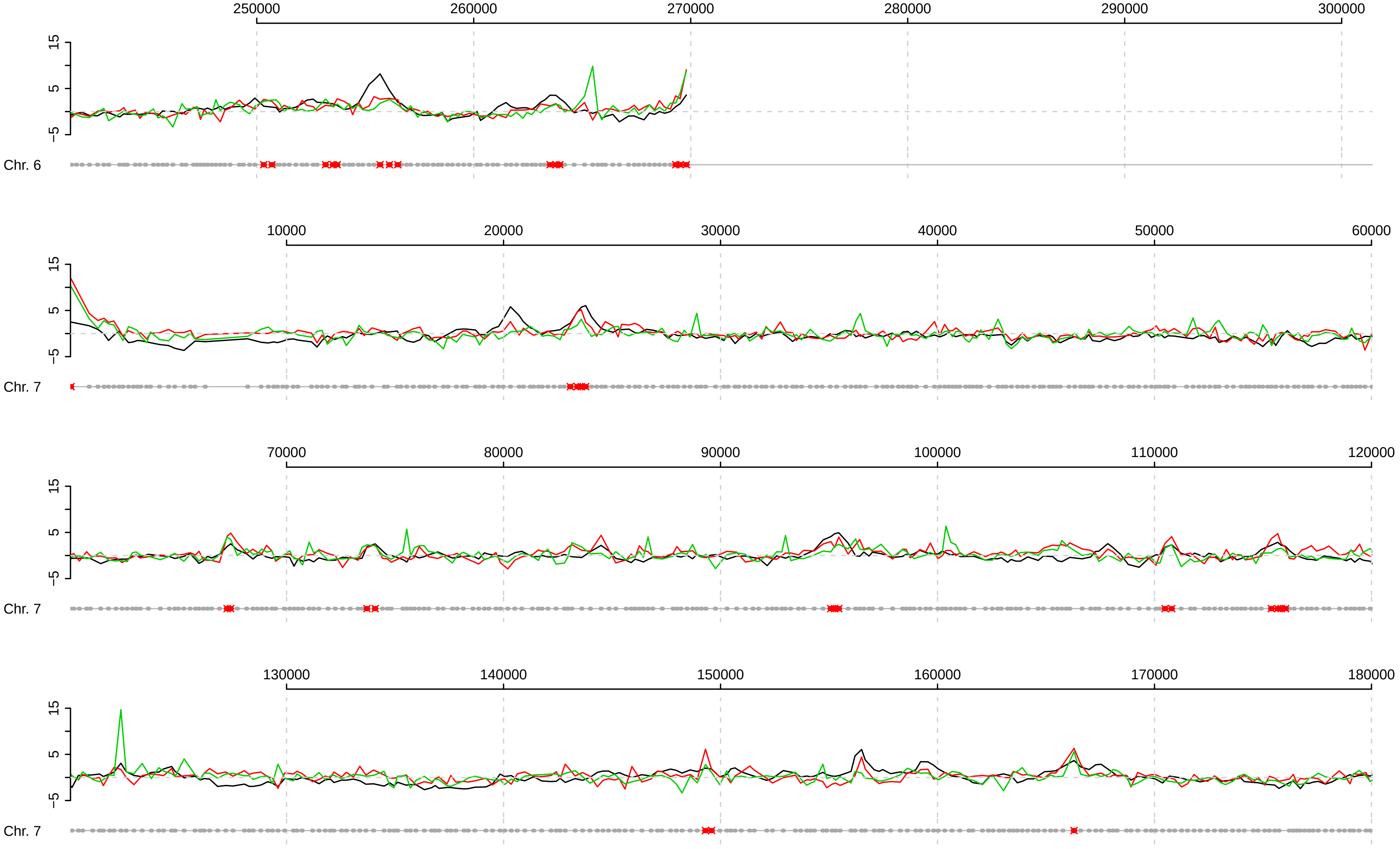

250000
260000
270000
280000
290000
300000
15
5
−5
Chr. 6
10000
20000
30000
40000
50000
60000
15
5
−5
Chr. 7
70000
80000
90000
100000
110000
120000
15
5
−5
Chr. 7
130000
140000
150000
160000
170000
180000
15
5
−5
Chr. 7

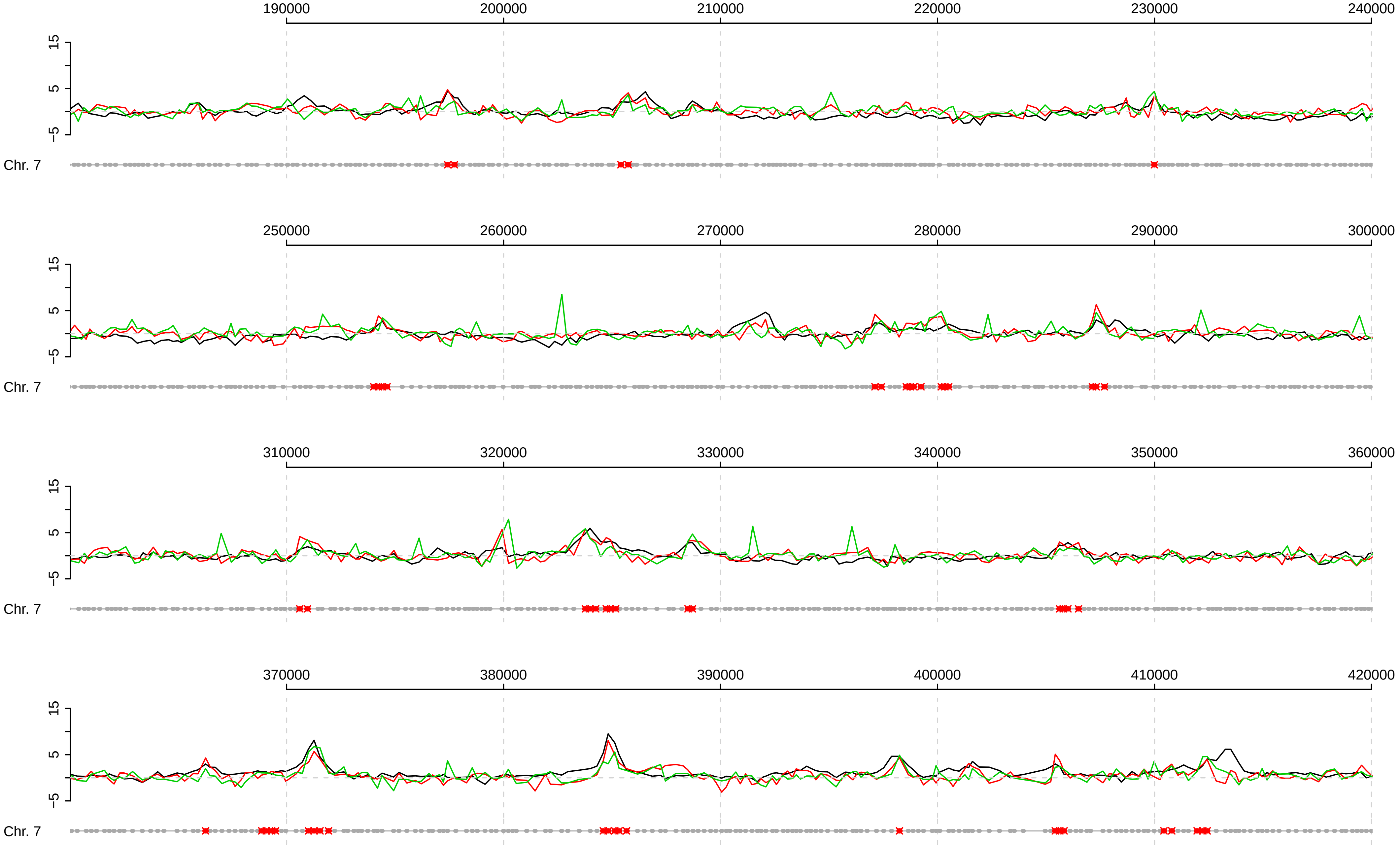

190000
200000
210000
220000
230000
240000
15
5
−5
Chr. 7
250000
260000
270000
280000
290000
300000
310000
320000
330000
340000
350000
360000
370000
380000
390000
400000
410000
420000

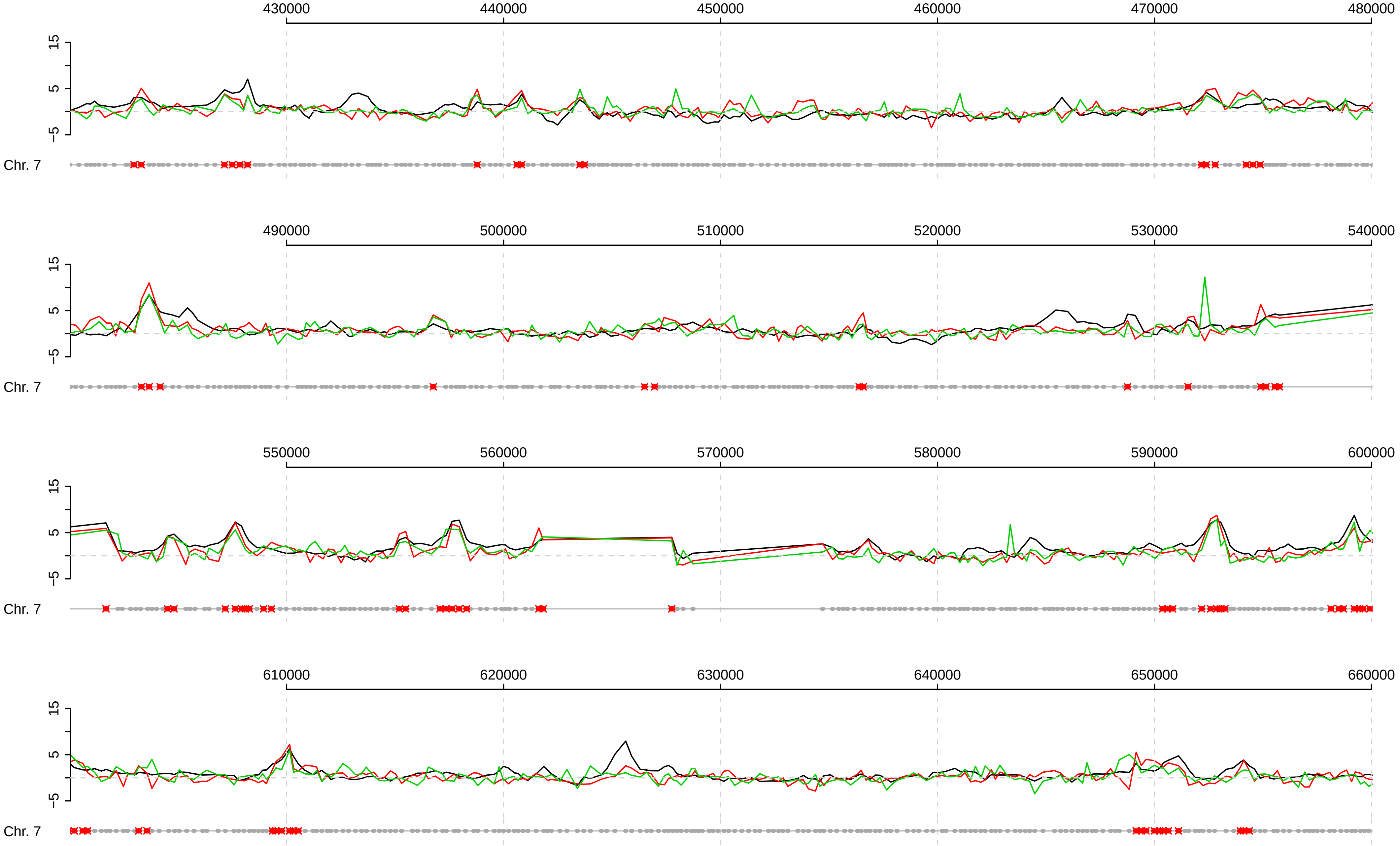

430000
440000
450000
460000
470000
480000
15
5
−5
Chr. 7
490000
500000
510000
520000
530000
540000
15
5
−5
Chr. 7
550000
560000
570000
580000
590000
600000
15
5
−5
Chr. 7
610000
620000
630000
640000
650000
660000
15
5
−5
Chr. 7

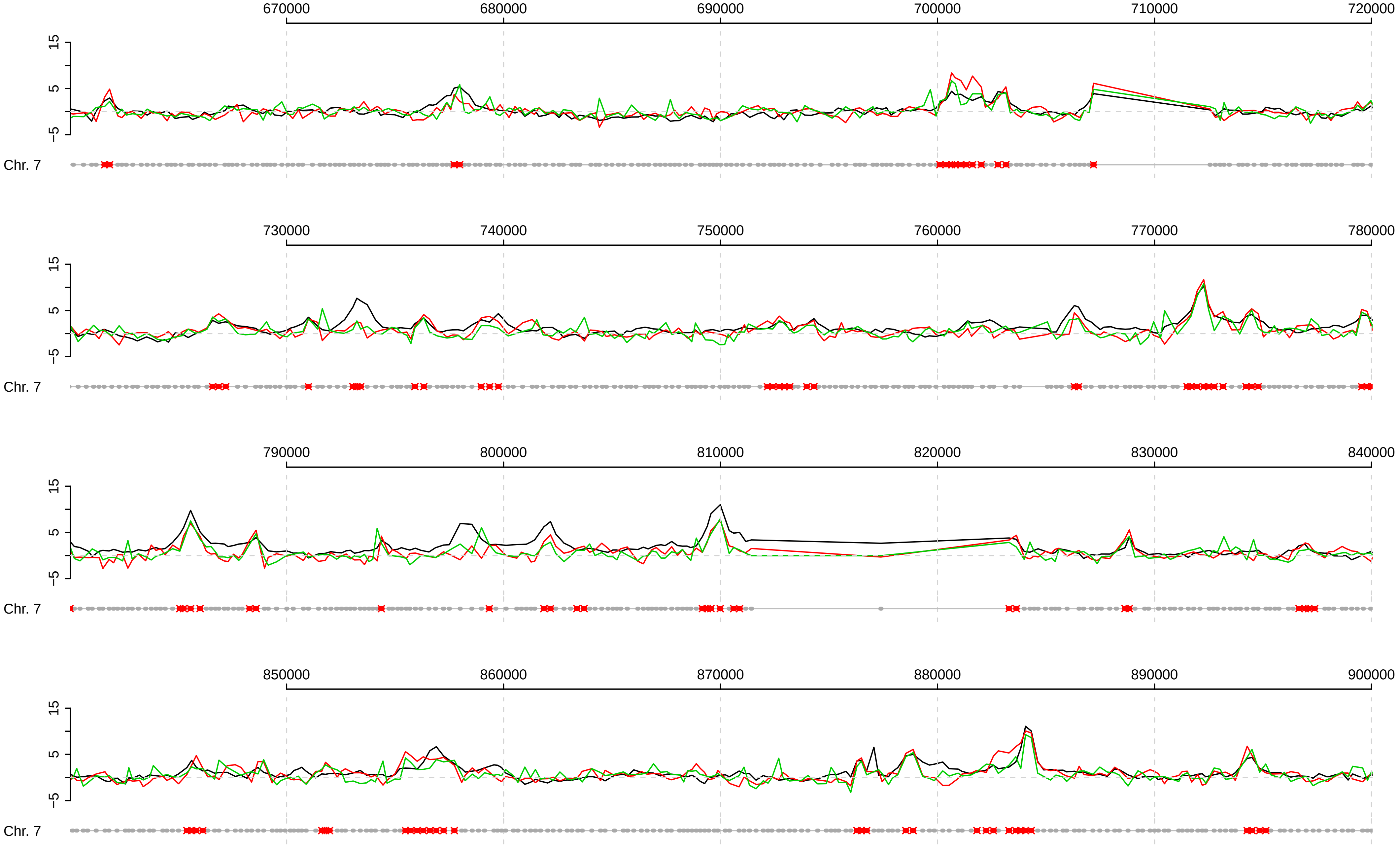

670000
680000
690000
700000
710000
720000
15
5
−5
Chr. 7
730000
740000
750000
760000
770000
780000
15
5
−5
Chr. 7
790000
800000
810000
820000
830000
840000
15
5
−5
Chr. 7
850000
860000
870000
880000
890000
900000
15
5
−5
Chr. 7

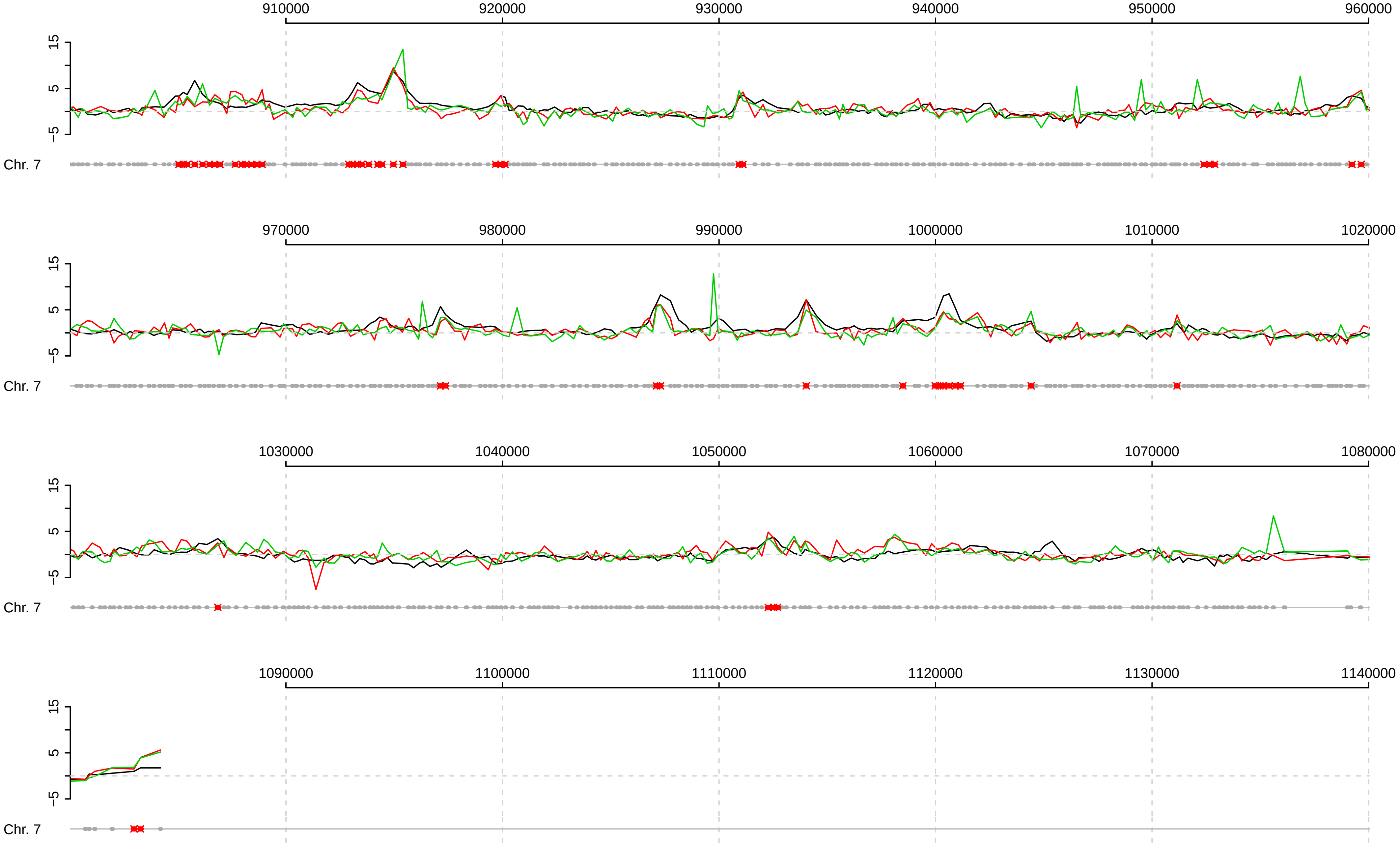

910000
920000
930000
940000
950000
960000
15
5
−5
Chr. 7
970000
980000
990000
1000000
1010000
1020000
15
5
−5
Chr. 7
1030000
1040000
1050000
1060000
1070000
1080000
15
5
−5
Chr. 7
1090000
1100000
1110000
1120000
1130000
1140000
15
5
−5
Chr. 7

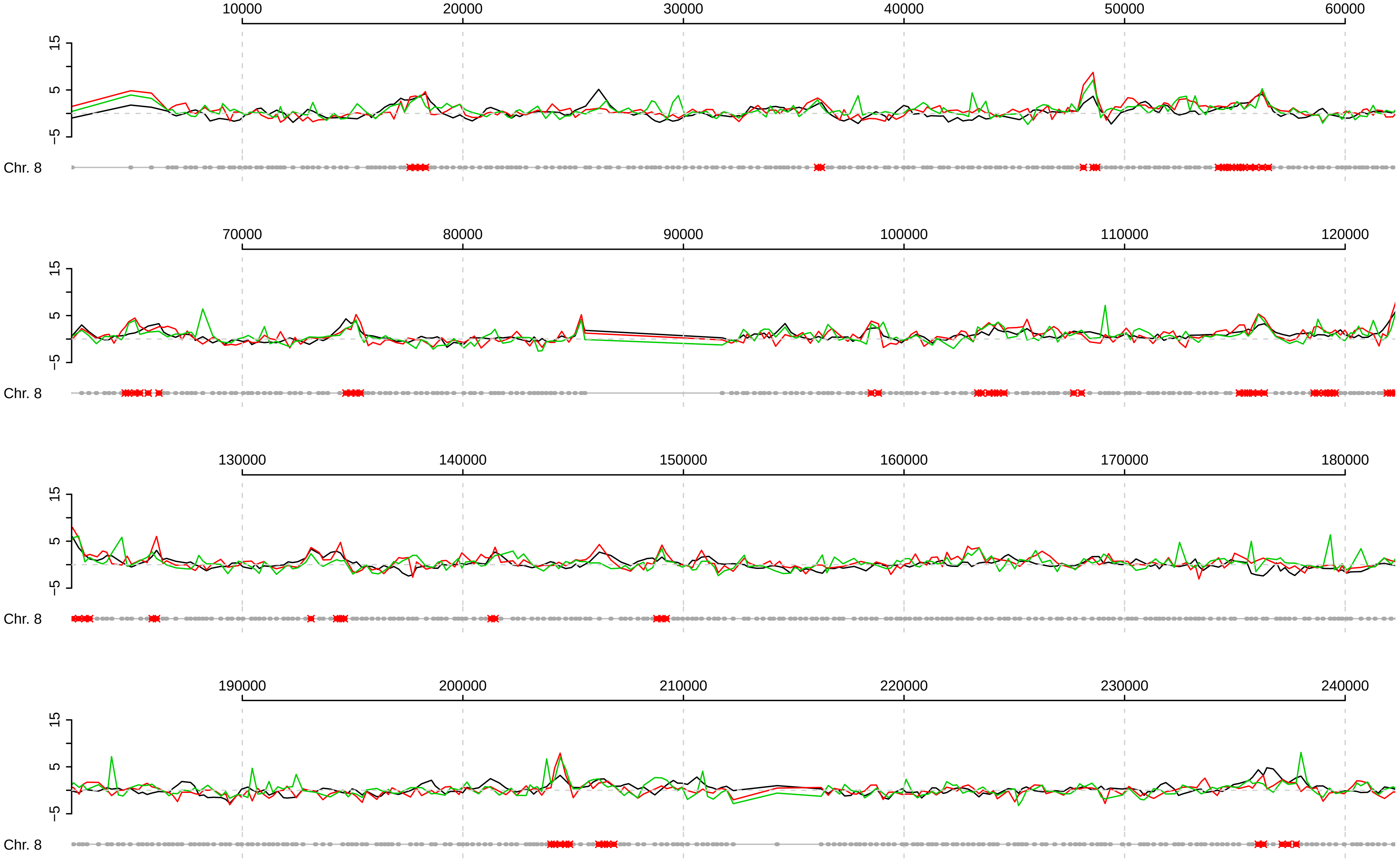

10000
20000
30000
40000
50000
60000
15
5
−5
Chr. 8
70000
80000
90000
100000
110000
120000
15
5
−5
Chr. 8
130000
140000
150000
160000
170000
180000
15
5
−5
Chr. 8
190000
200000
210000
220000
230000
240000
15
5
−5
Chr. 8

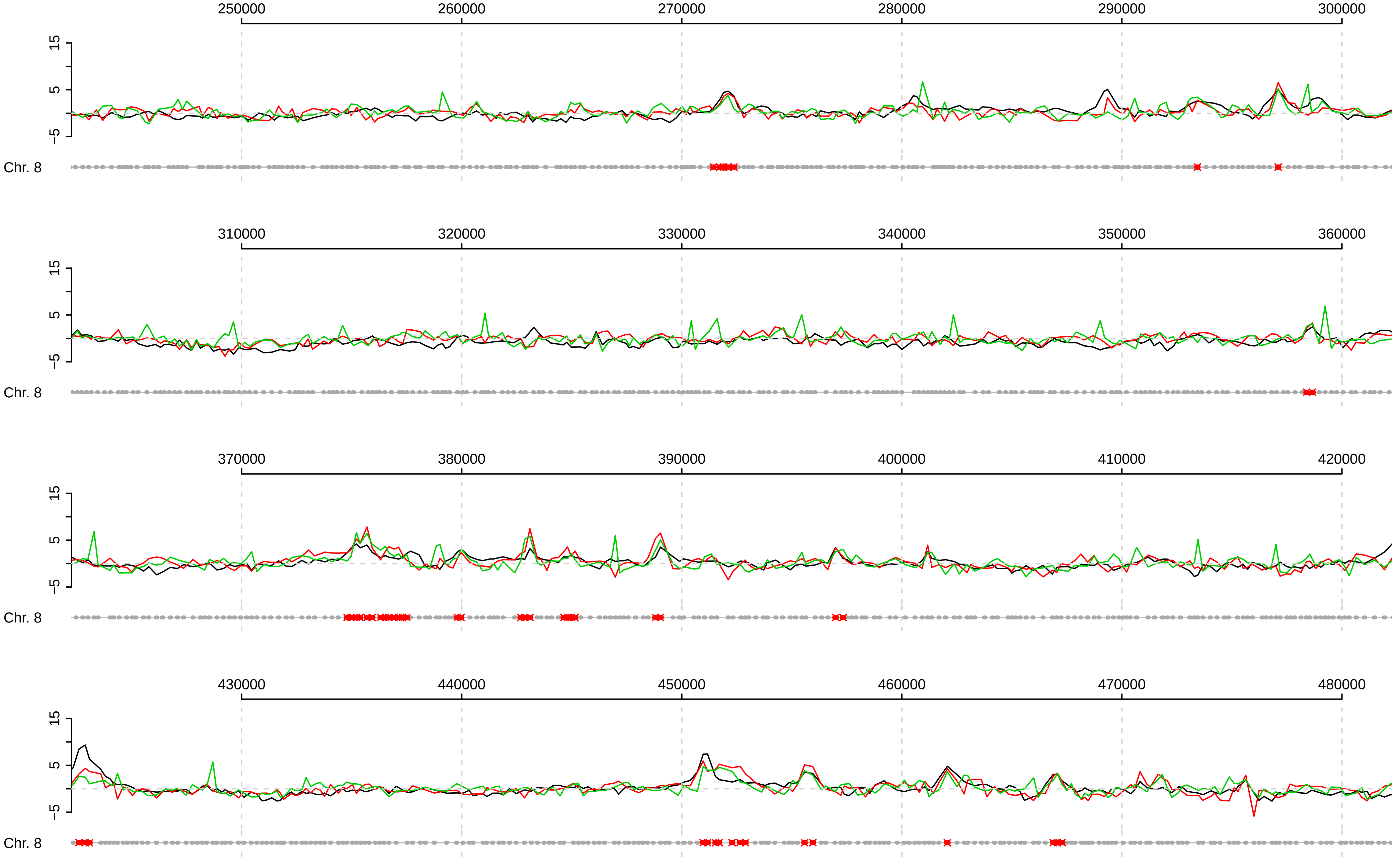

250000
260000
270000
280000
290000
300000
15
5
−5
Chr. 8
310000
320000
330000
340000
350000
360000
15
5
−5
Chr. 8
370000
380000
390000
400000
410000
420000
15
5
−5
Chr. 8
430000
440000
450000
460000
470000
480000
15
5
−5
Chr. 8

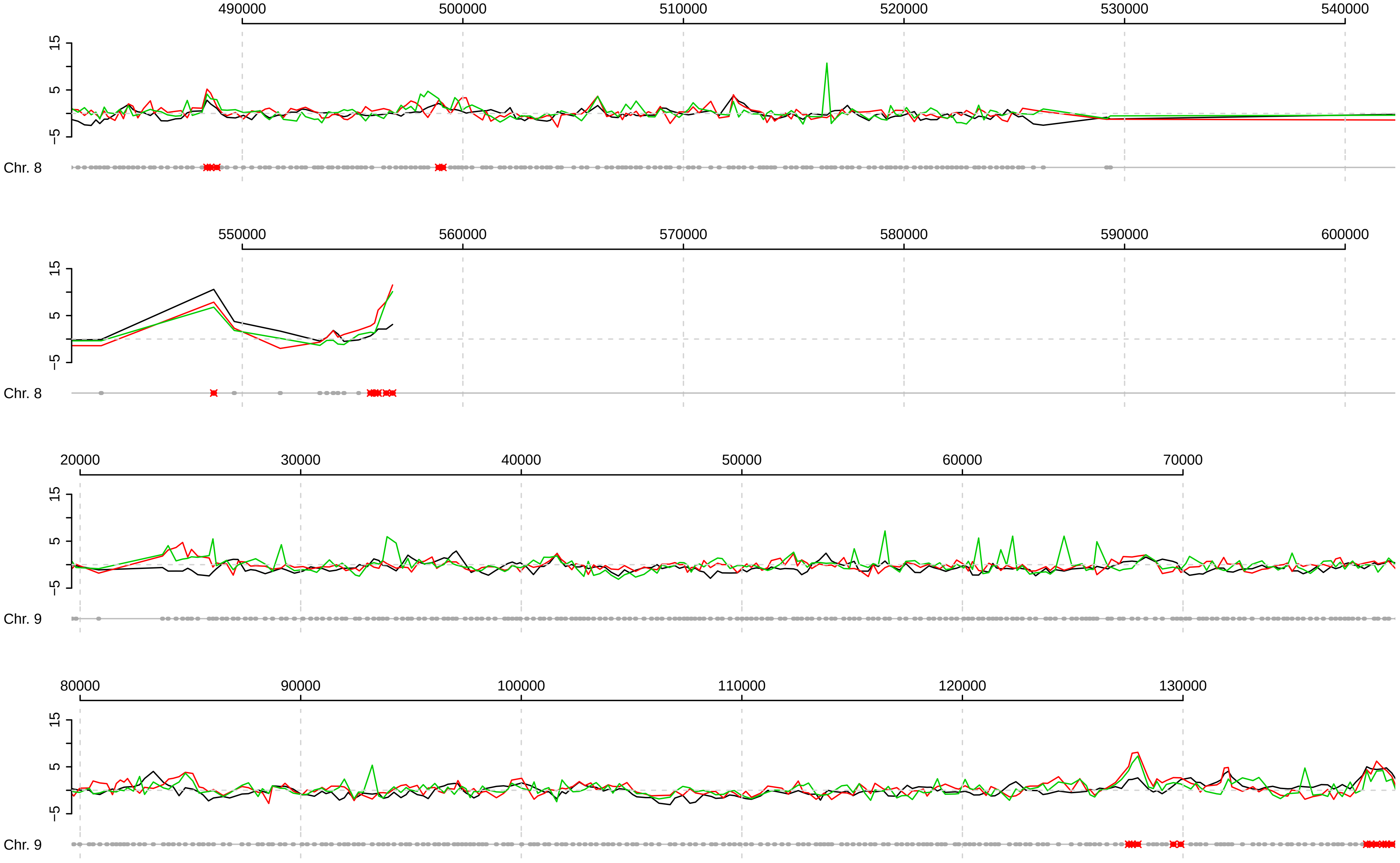

490000
500000
510000
520000
530000
540000
15
5
−5
Chr. 8
550000
560000
570000
580000
590000
600000
15
5
−5
Chr. 8
20000
30000
40000
50000
60000
70000
15
5
−5
Chr. 9
80000
90000
100000
110000
120000
130000
15
5
−5
Chr. 9

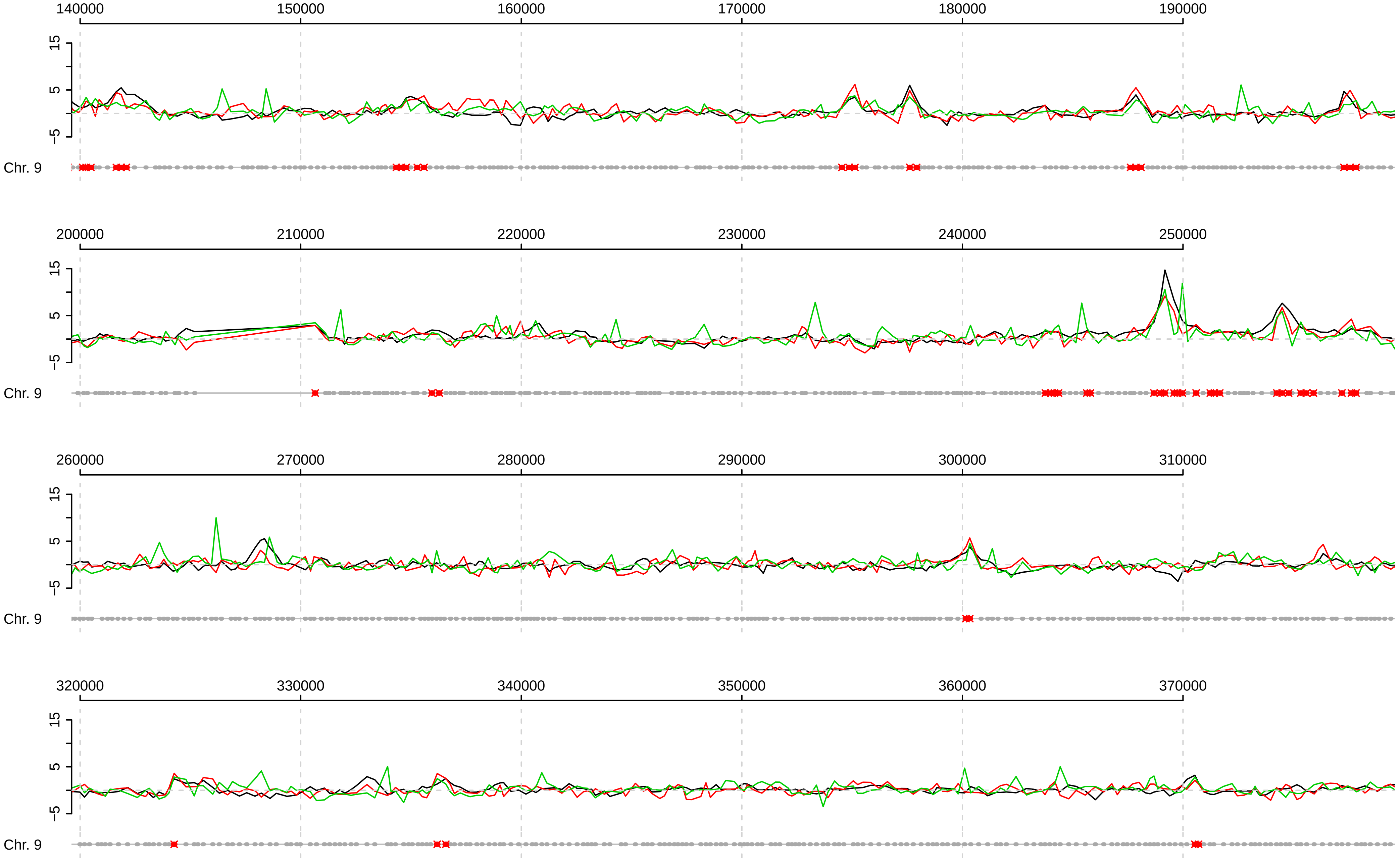
140000
150000
160000
170000
180000
190000
15
5
−5
Chr. 9
200000
210000
220000
230000
240000
250000
15
5
−5
Chr. 9
260000
270000
280000
290000
300000
310000
15
5
−5
Chr. 9
320000
330000
340000
350000
360000
370000
15
5
−5
Chr. 9

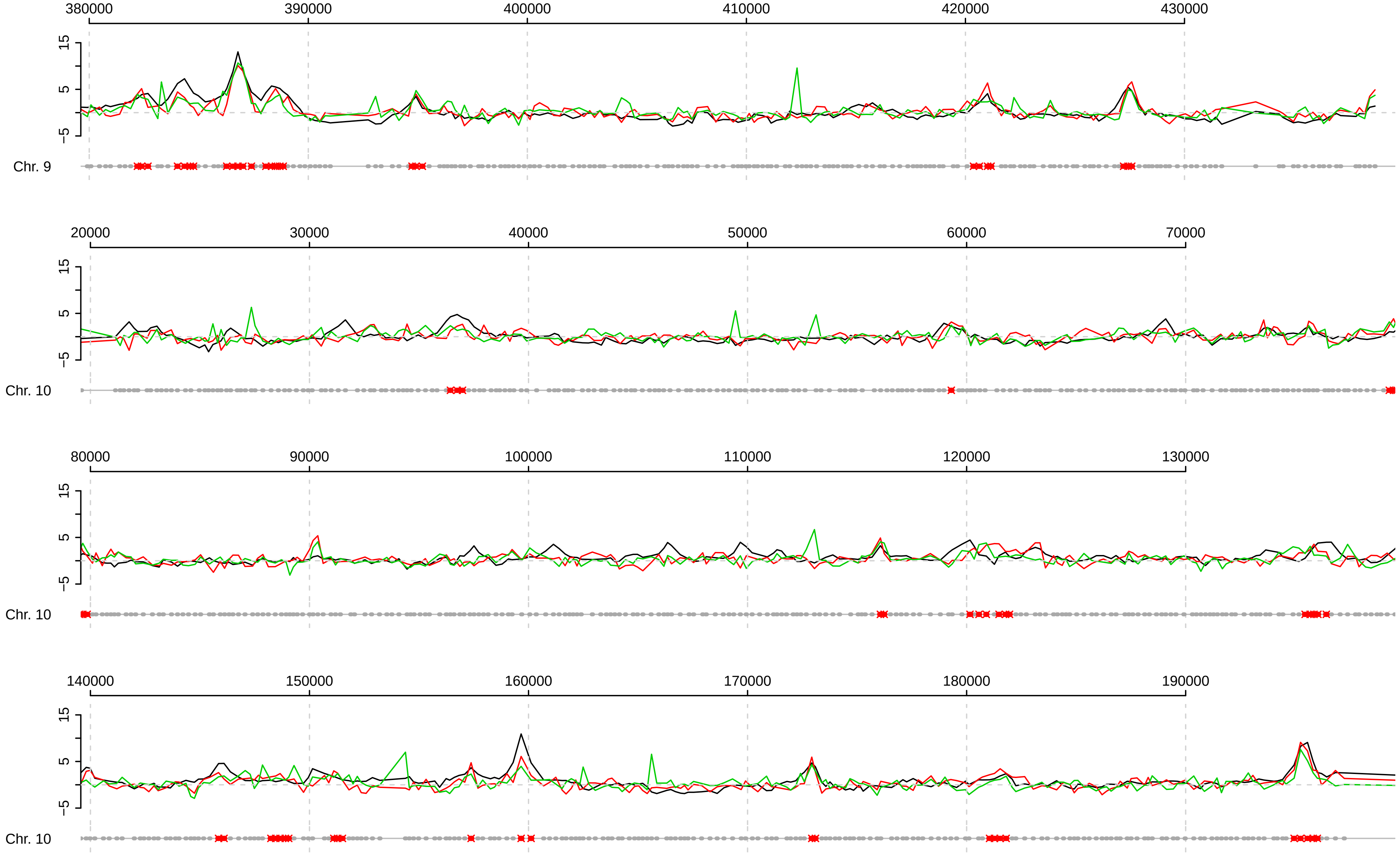

380000
390000
400000
410000
420000
430000
15
5
−5
Chr. 9
20000
30000
40000
50000
60000
70000
15
5
−5
Chr. 10
80000
90000
100000
110000
120000
130000
15
5
−5
Chr. 10
140000
150000
160000
170000
180000
190000
15
5
−5
Chr. 10

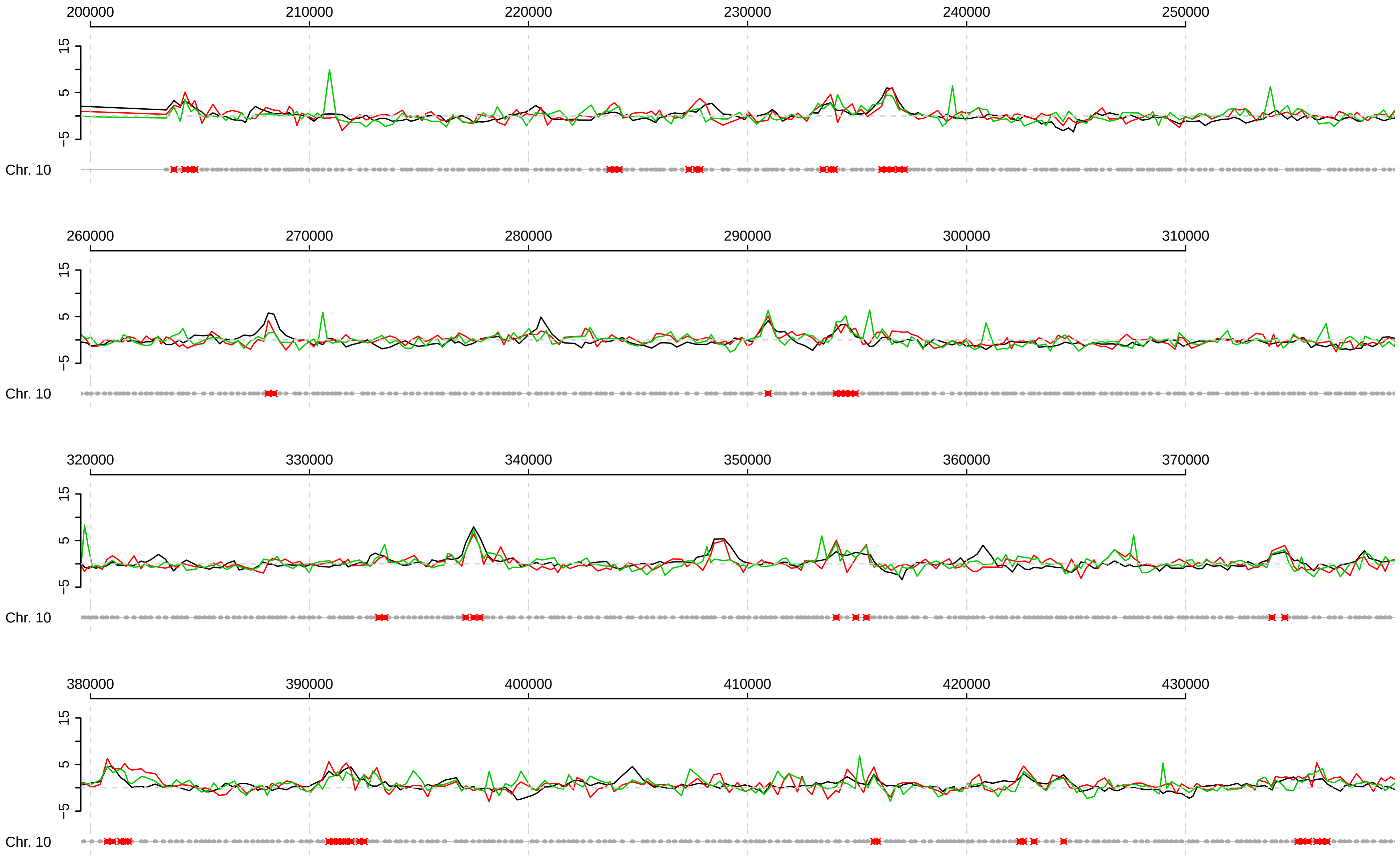

200000
210000
220000
230000
240000
250000
15
5
−5
Chr. 10
260000
270000
280000
290000
300000
310000
15
5
−5
Chr. 10
320000
330000
340000
350000
360000
370000
15
5
−5
Chr. 10
380000
390000
400000
410000
420000
430000
15
5
−5
Chr. 10

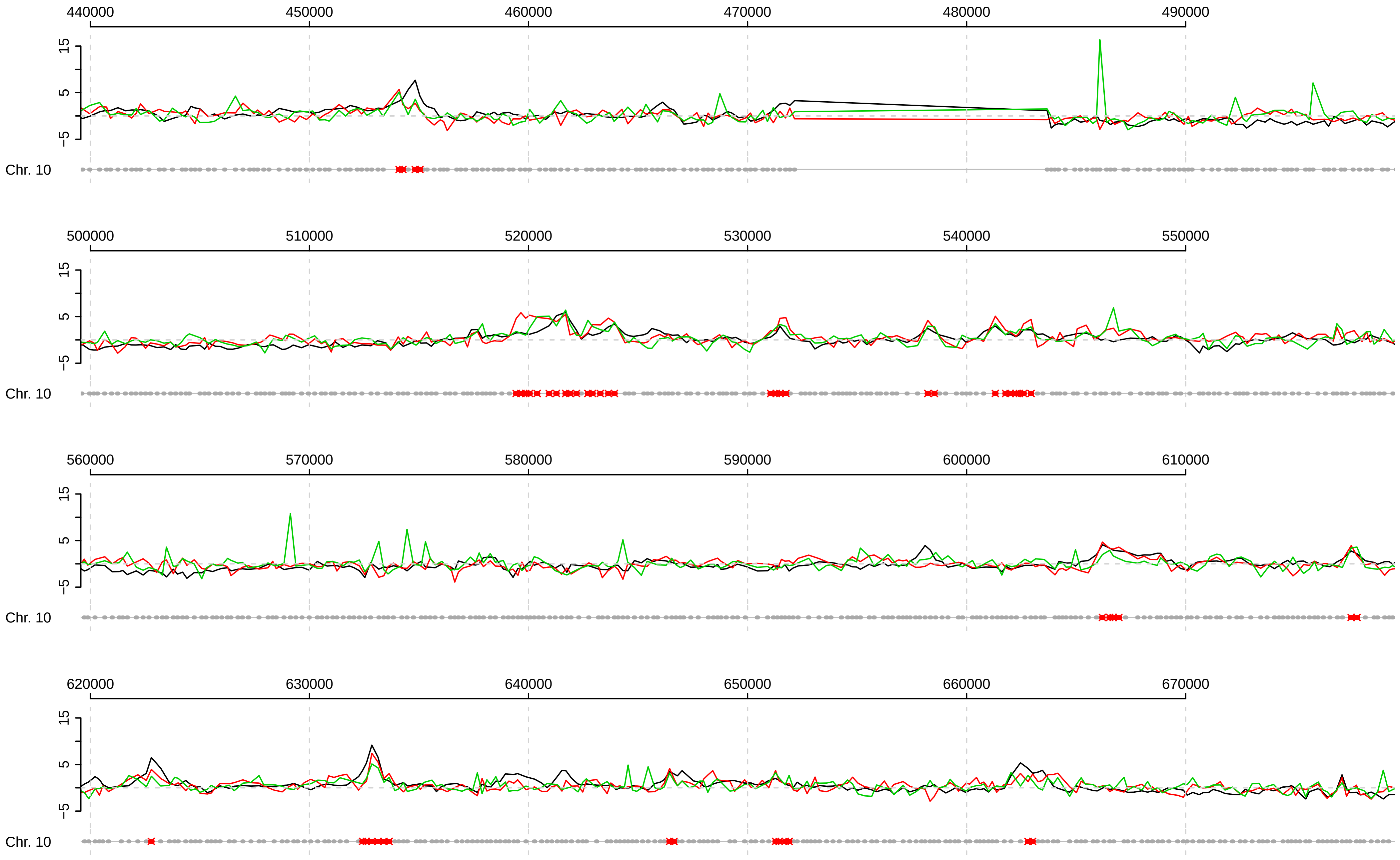

440000
450000
460000
470000
480000
490000
15
5
−5
Chr. 10
500000
510000
520000
530000
540000
550000
15
5
−5
Chr. 10
560000
570000
580000
590000
600000
610000
15
5
−5
Chr. 10
620000
630000
640000
650000
660000
670000
15
5
−5
Chr. 10

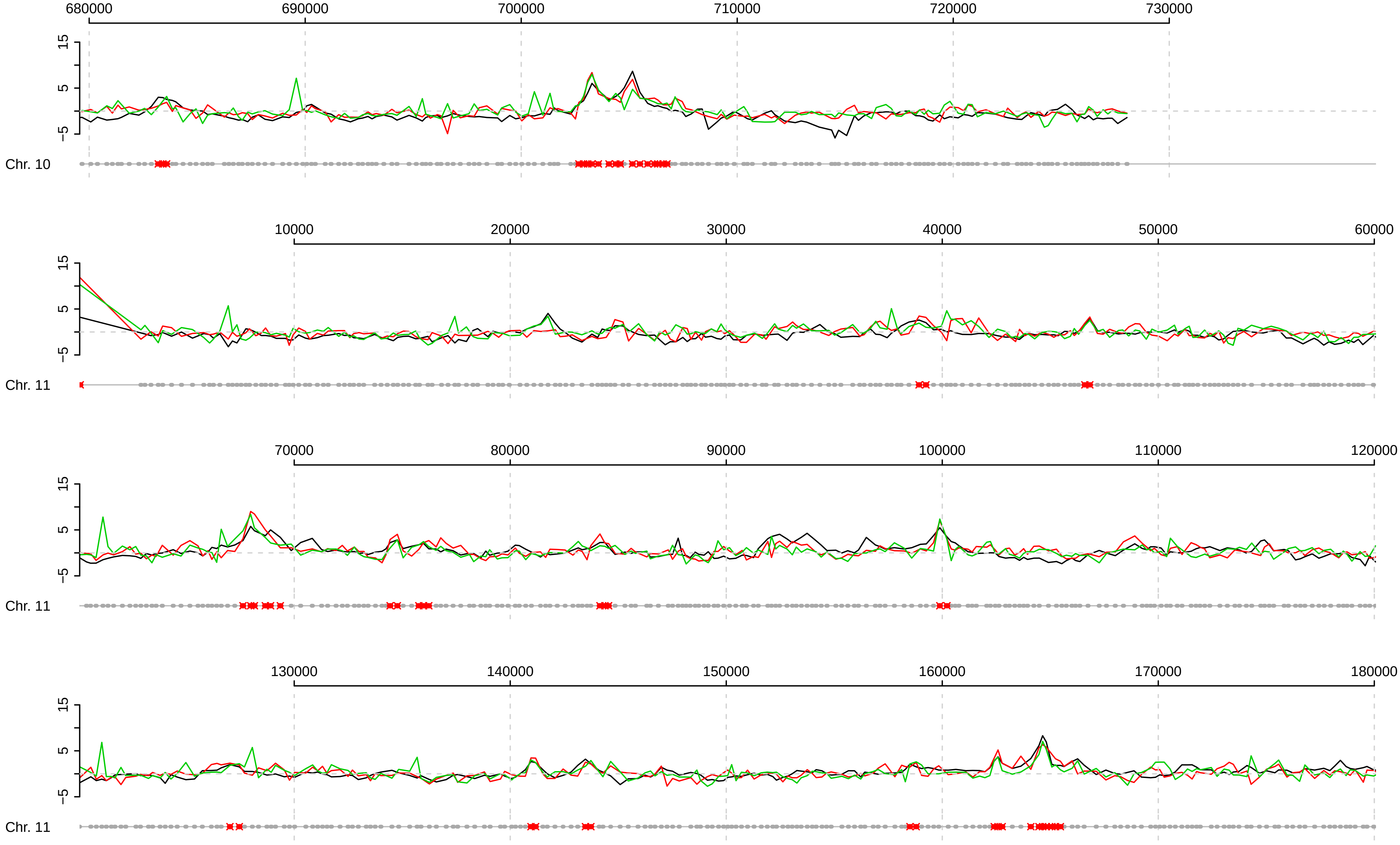
680000
690000
700000
710000
720000
730000
15
5
−5
Chr. 10
10000
20000
30000
40000
50000
60000
Chr. 11
70000
80000
90000
100000
110000
120000
Chr. 11
130000
140000
150000
160000
170000
180000
Chr. 11

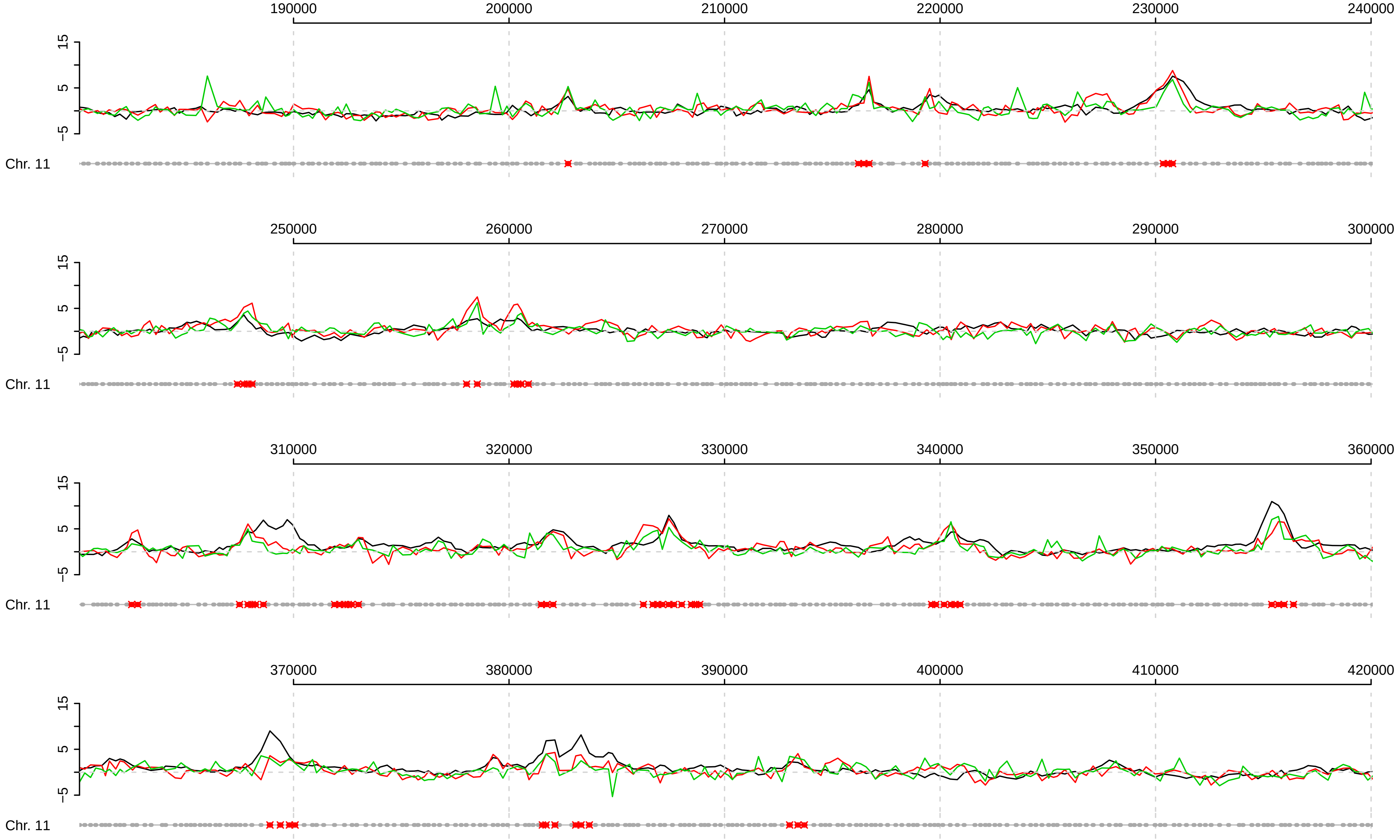

190000
200000
210000
220000
230000
240000
15
5
−5
Chr. 11
250000
260000
270000
280000
290000
300000
15
5
−5
Chr. 11
310000
320000
330000
340000
350000
360000
15
5
−5
Chr. 11
370000
380000
390000
400000
410000
420000
15
5
−5
Chr. 11

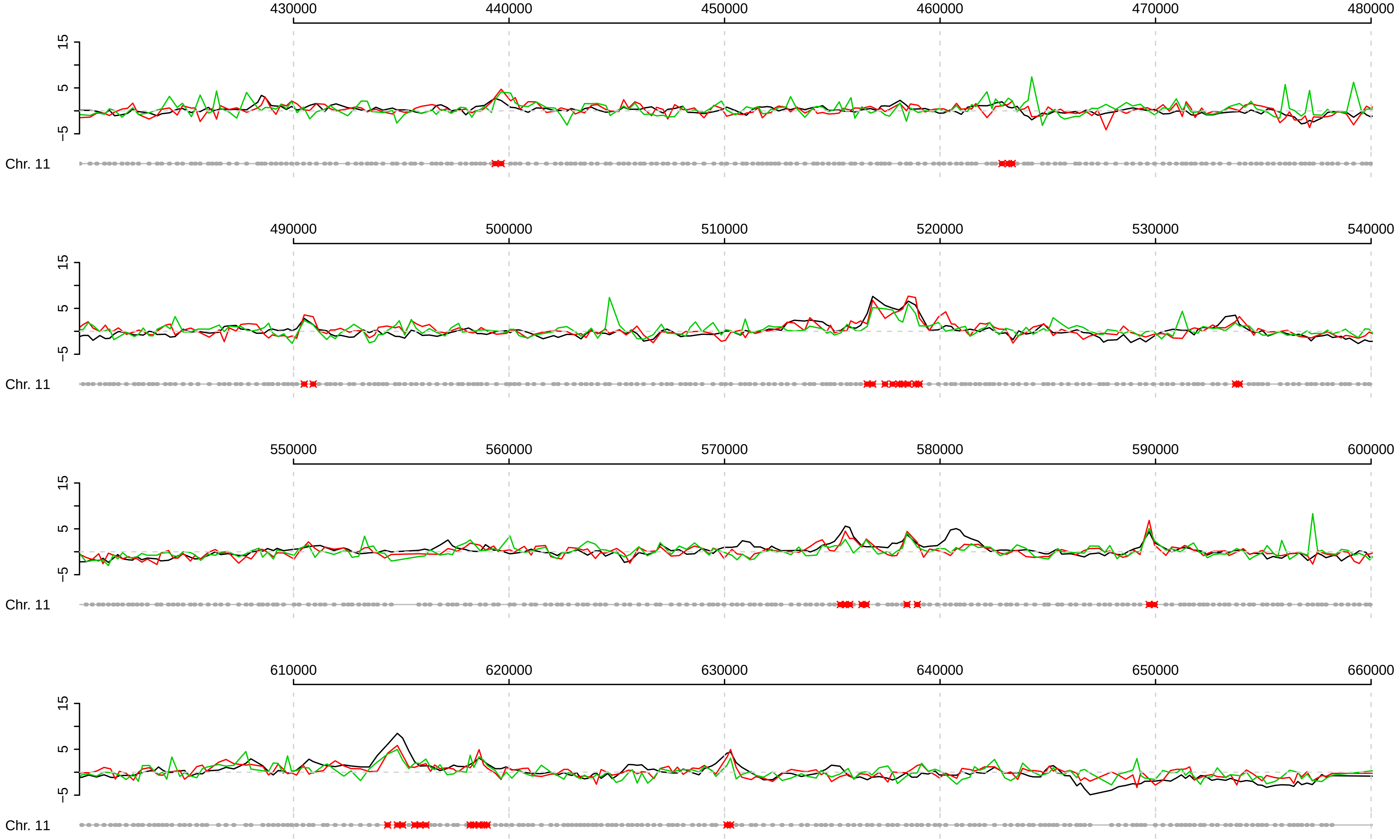
430000
440000
450000
460000
470000
480000
15
5
−5
Chr. 11
490000
500000
510000
520000
530000
540000
15
5
−5
Chr. 11
550000
560000
570000
580000
590000
600000
15
5
−5
Chr. 11
610000
620000
630000
640000
650000
660000
15
5
−5
Chr. 11

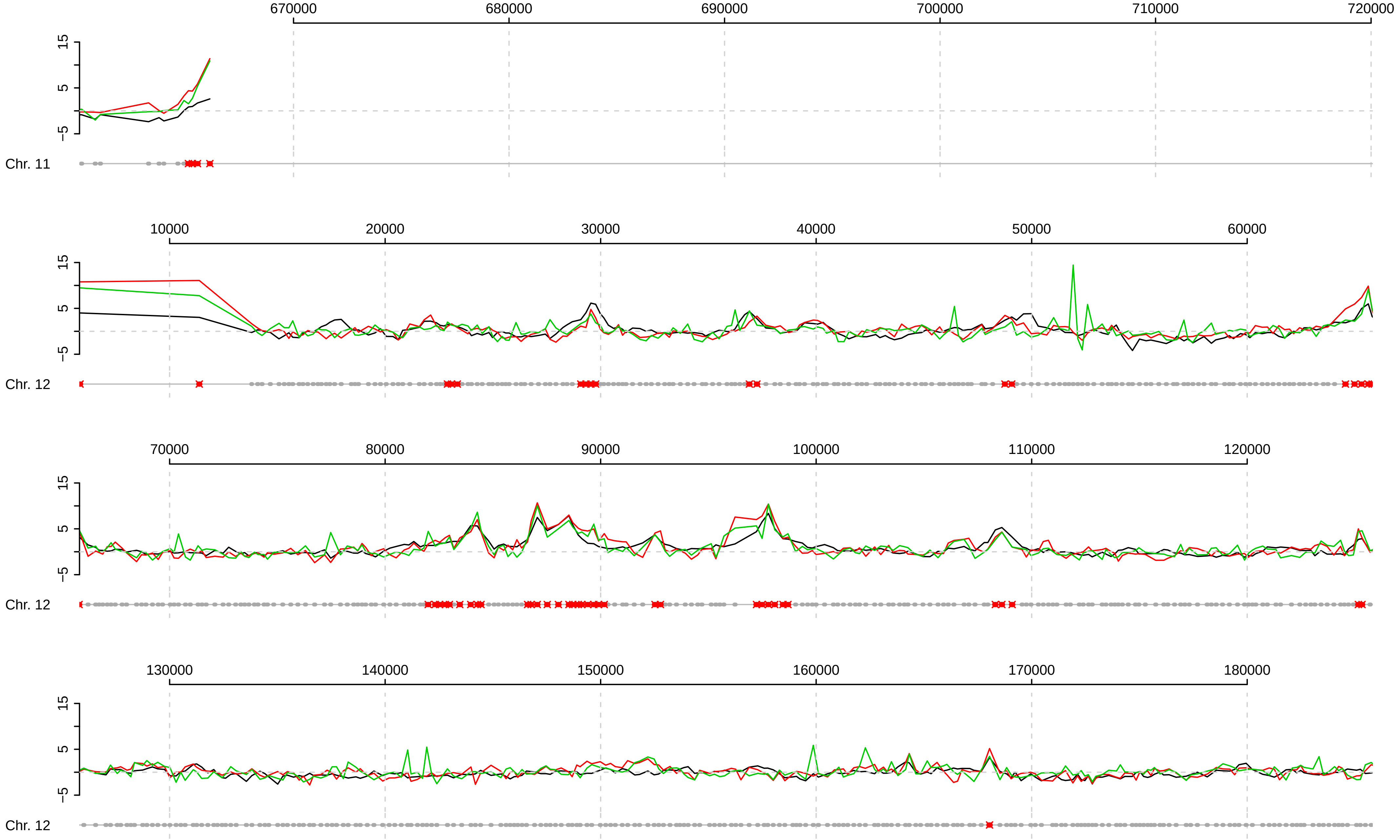
670000
680000
690000
700000
710000
720000
15
5
−5
Chr. 11
10000
20000
30000
40000
50000
60000
Chr. 12
70000
80000
90000
100000
110000
120000
Chr. 12
130000
140000
150000
160000
170000
180000
Chr. 12

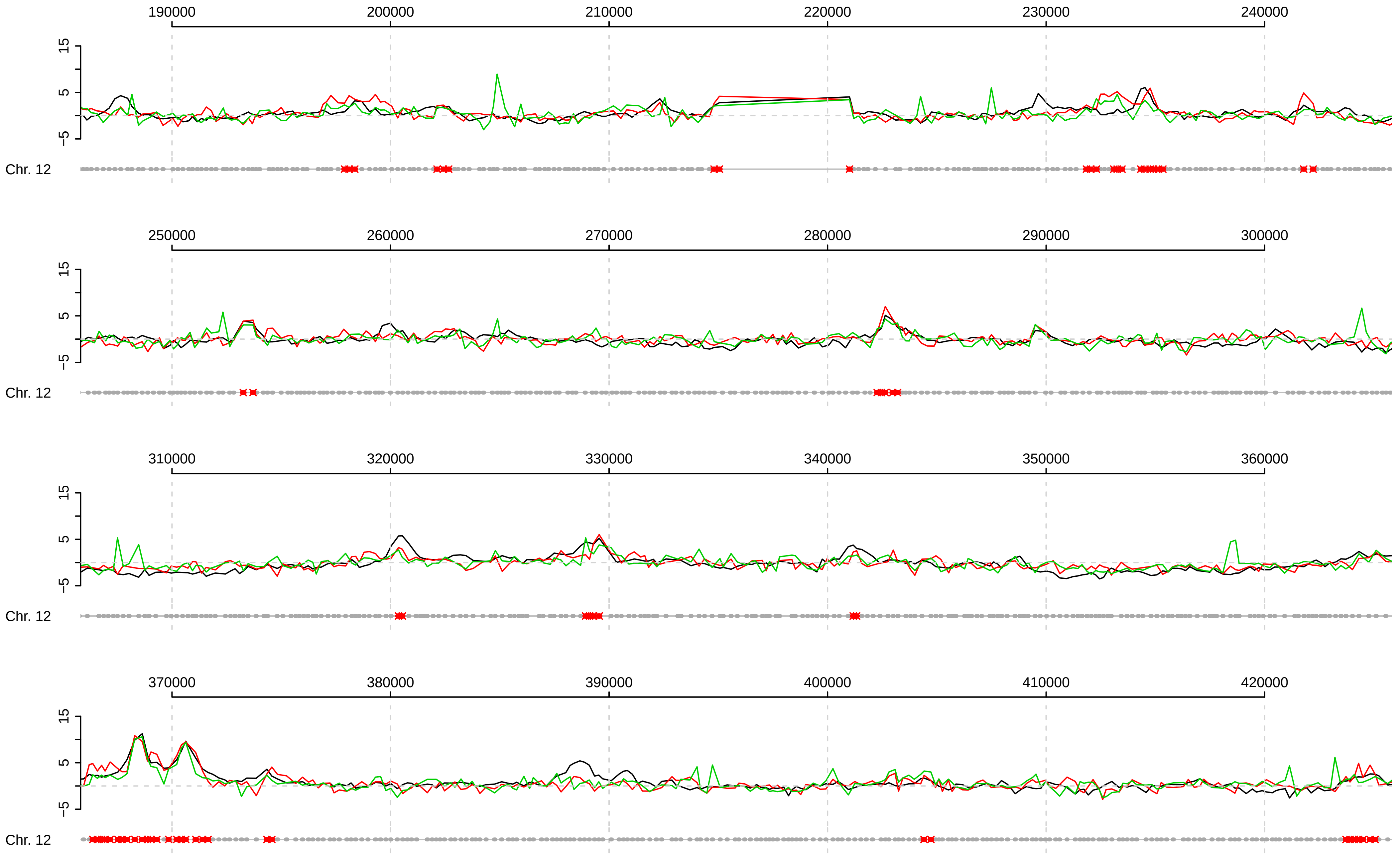

190000
200000
210000
220000
230000
240000
15
5
−5
Chr. 12
250000
260000
270000
280000
290000
300000
15
5
−5
Chr. 12
310000
320000
330000
340000
350000
360000
15
5
−5
Chr. 12
370000
380000
390000
400000
410000
420000
15
5
−5
Chr. 12

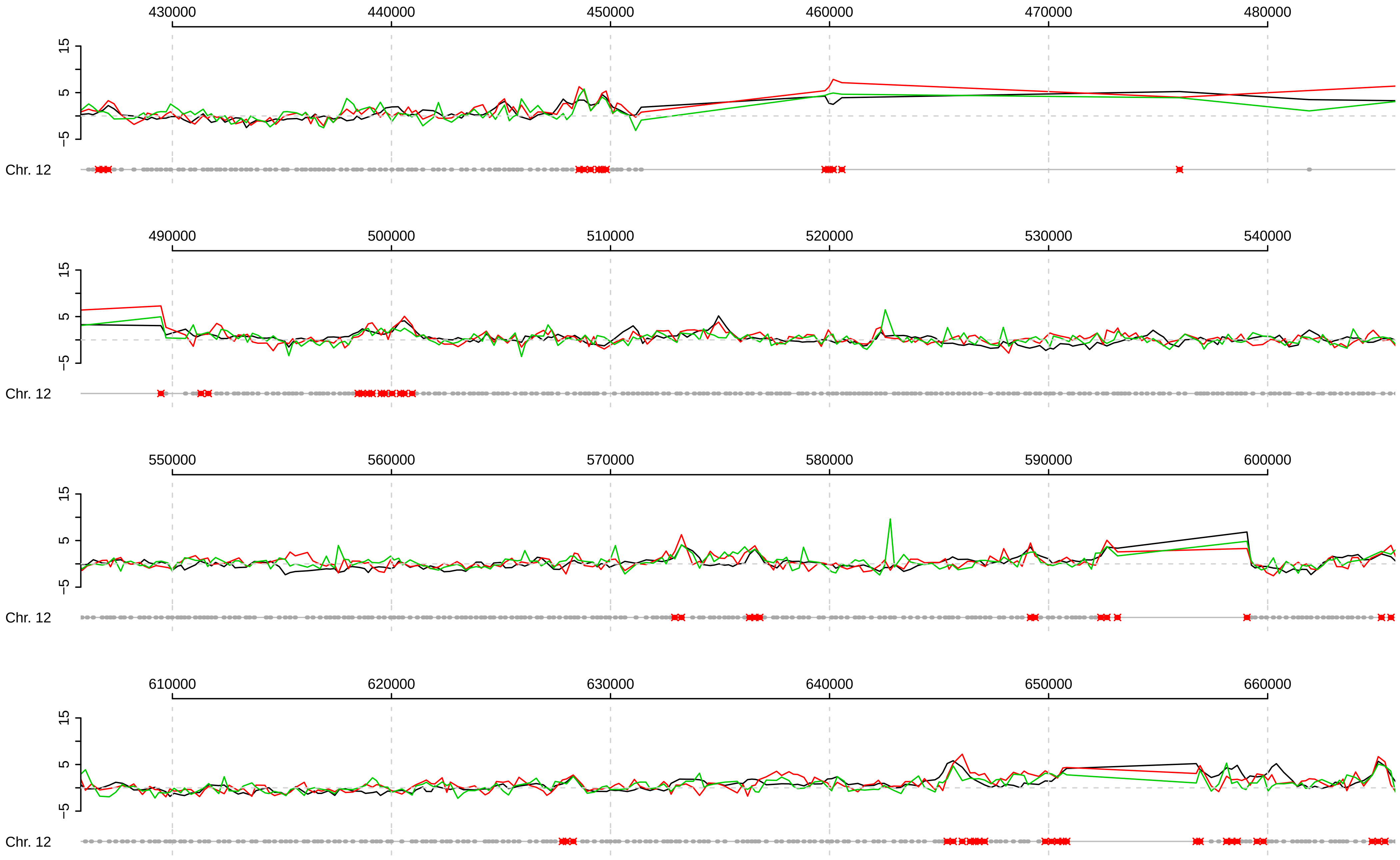

430000
440000
450000
460000
470000
480000
15
5
−5
Chr. 12
490000
500000
510000
520000
530000
540000
15
5
−5
Chr. 12
550000
560000
570000
580000
590000
600000
15
5
−5
Chr. 12
610000
620000
630000
640000
650000
660000
15
5
−5
Chr. 12

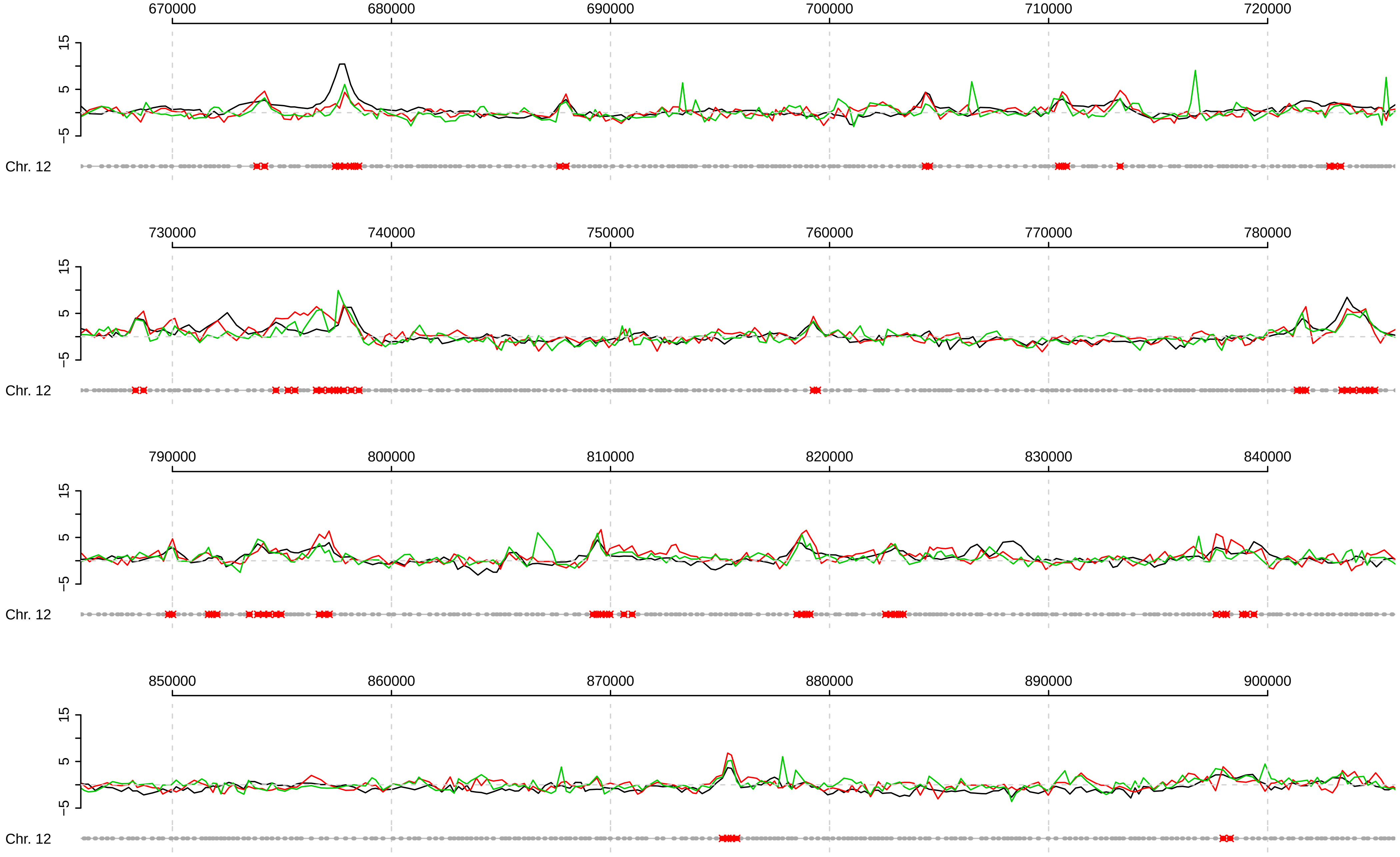
670000
680000
690000
700000
710000
720000
15
5
−5
Chr. 12
730000
740000
750000
760000
770000
780000
15
5
−5
Chr. 12
790000
800000
810000
820000
830000
840000
15
5
−5
Chr. 12
850000
860000
870000
880000
890000
900000
15
5
−5
Chr. 12

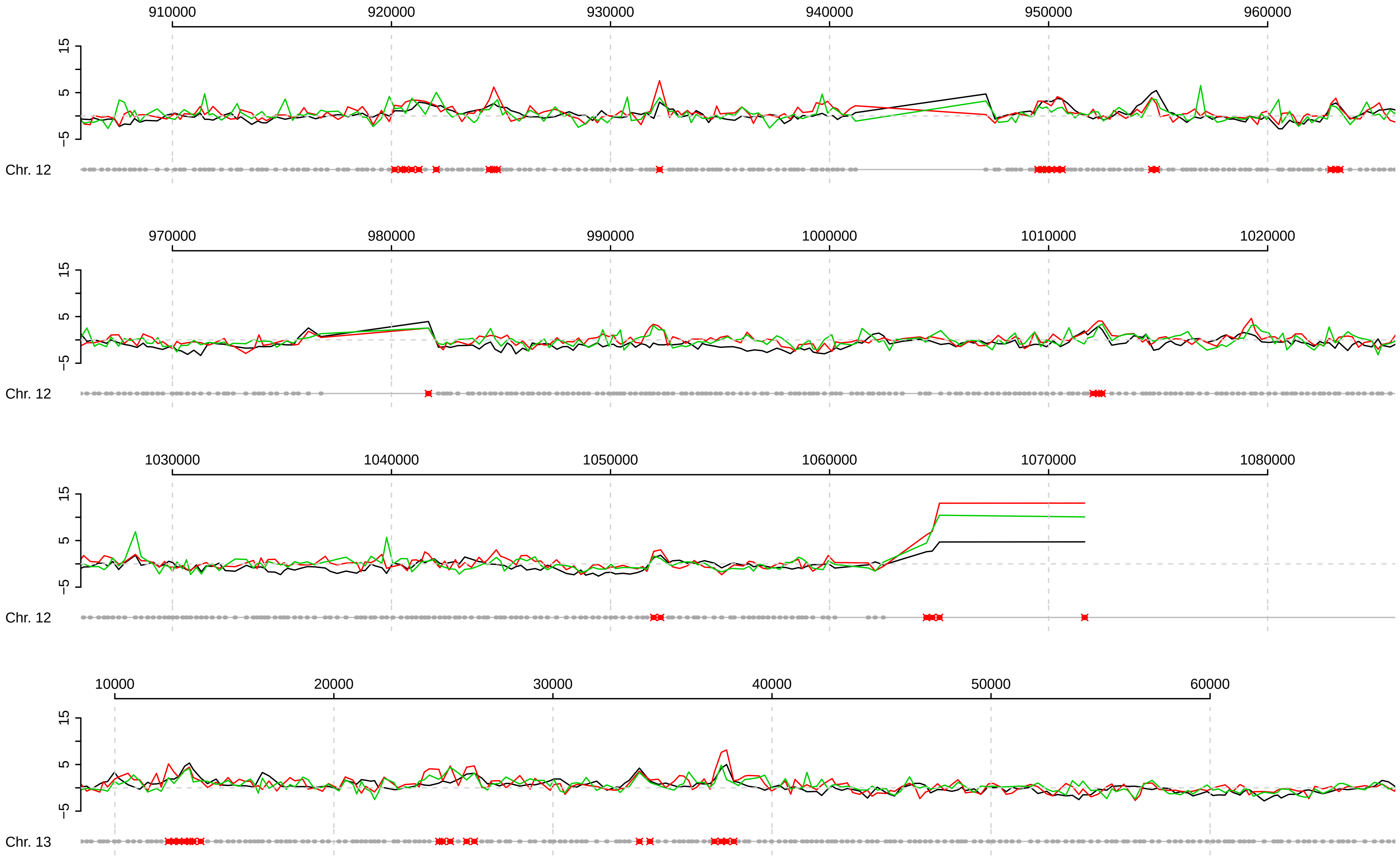
910000
920000
930000
940000
950000
960000
15
5
−5
Chr. 12
970000
980000
990000
1000000
1010000
1020000
15
5
−5
Chr. 12
1030000
1040000
1050000
1060000
1070000
1080000
15
5
−5
Chr. 12
10000
20000
30000
40000
50000
60000
15
5
−5
Chr. 13

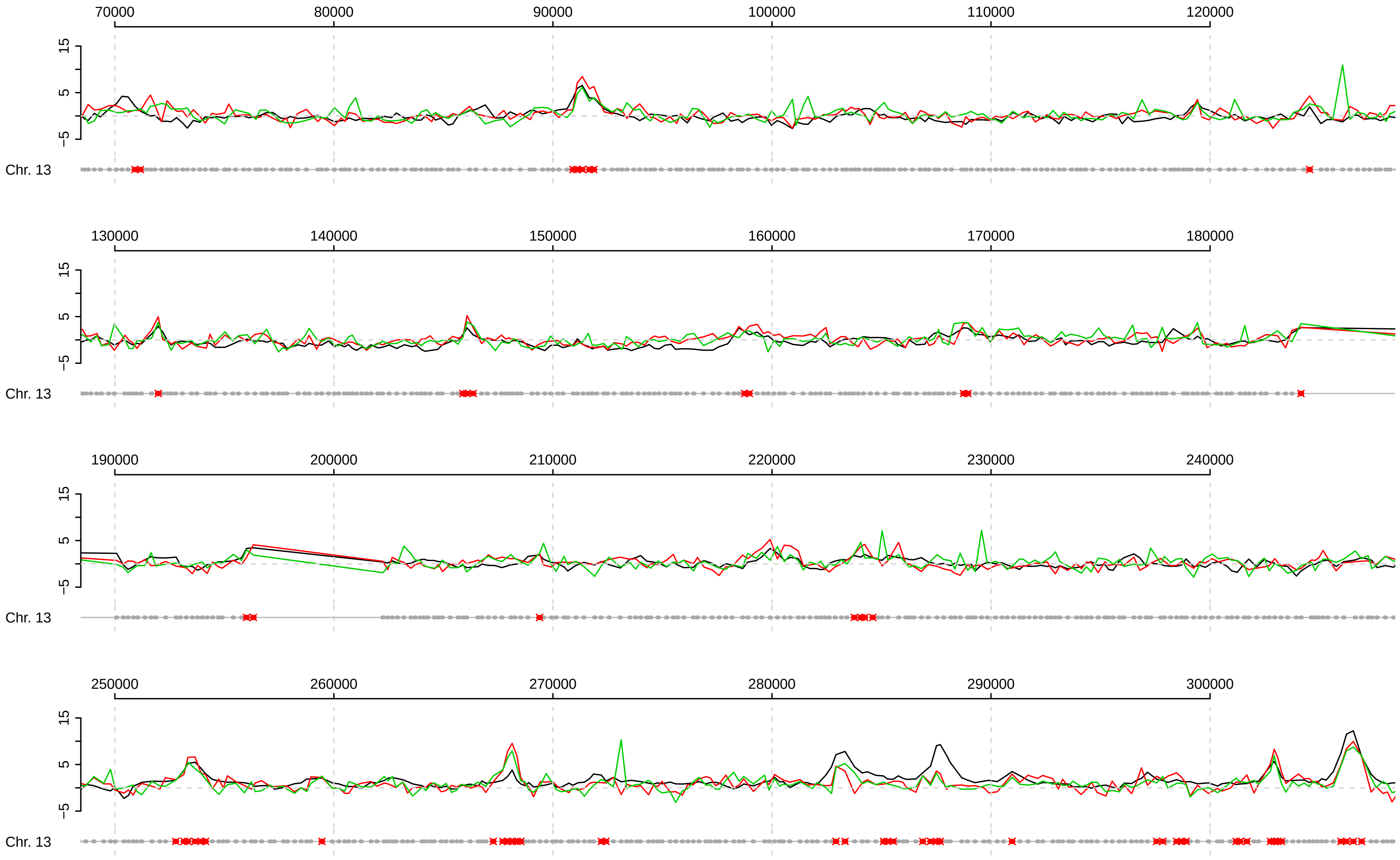

70000
80000
90000
100000
110000
120000
15
5
−5
Chr. 13
130000
140000
150000
160000
170000
180000
15
5
−5
Chr. 13
190000
200000
210000
220000
230000
240000
15
5
−5
Chr. 13
250000
260000
270000
280000
290000
300000
15
5
−5
Chr. 13

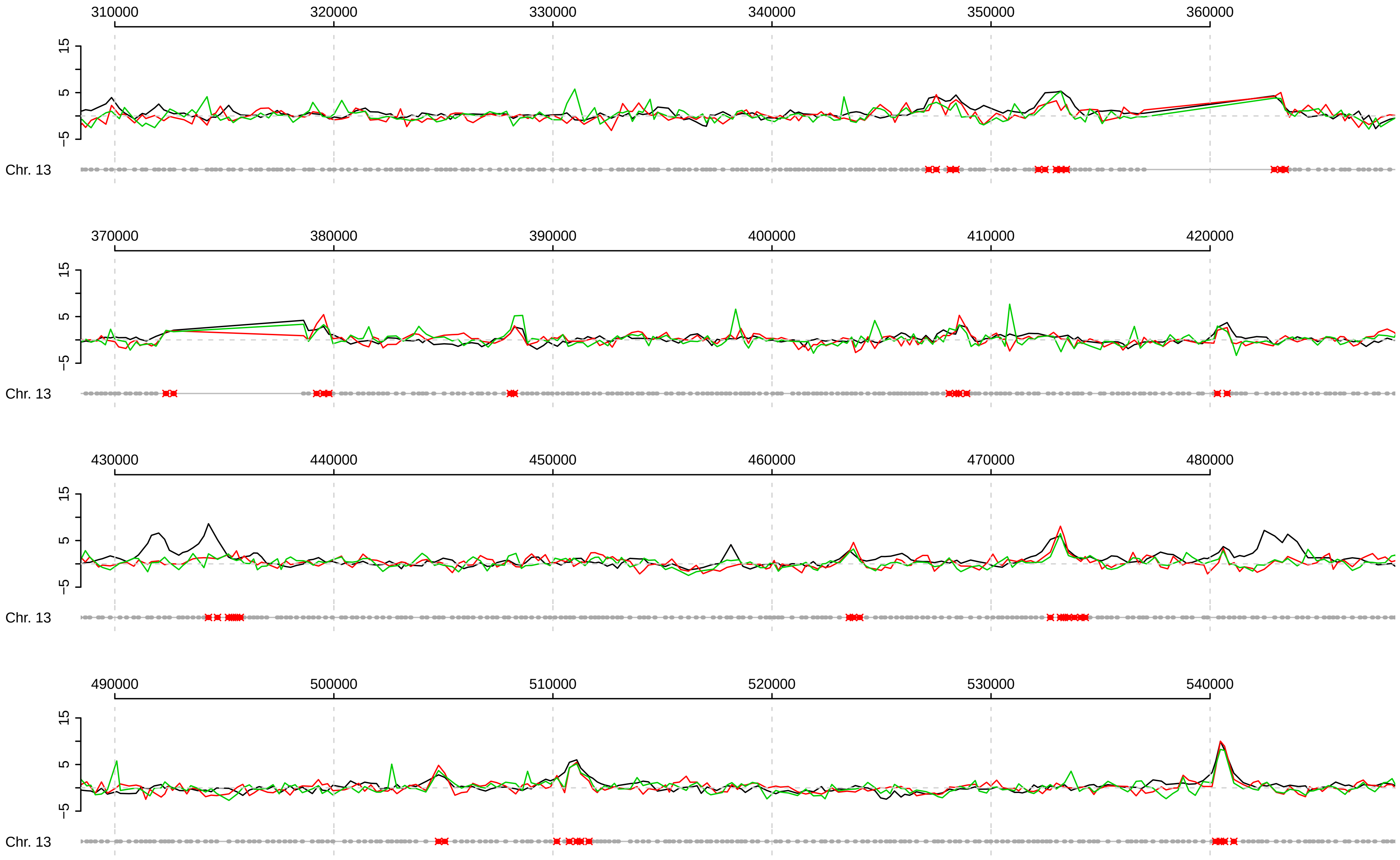

310000
320000
330000
340000
350000
360000
15
5
−5
Chr. 13
370000
380000
390000
400000
410000
420000
15
5
−5
Chr. 13
430000
440000
450000
460000
470000
480000
15
5
−5
Chr. 13
490000
500000
510000
520000
530000
540000
15
5
−5
Chr. 13

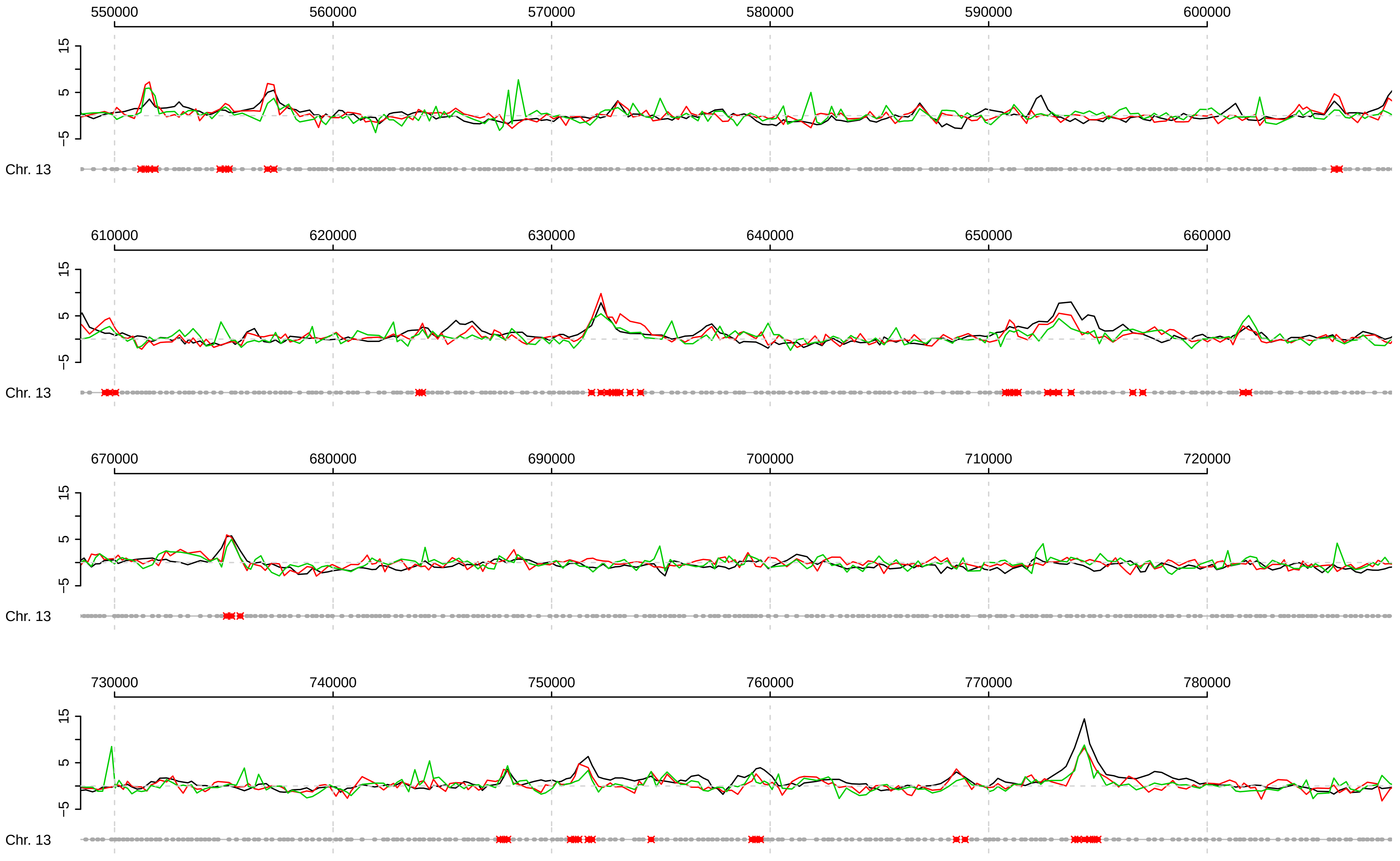
550000
560000
570000
580000
590000
600000
15
5
−5
Chr. 13
610000
620000
630000
640000
650000
660000
15
5
−5
Chr. 13
670000
680000
690000
700000
710000
720000
15
5
−5
Chr. 13
730000
740000
750000
760000
770000
780000
15
5
−5
Chr. 13

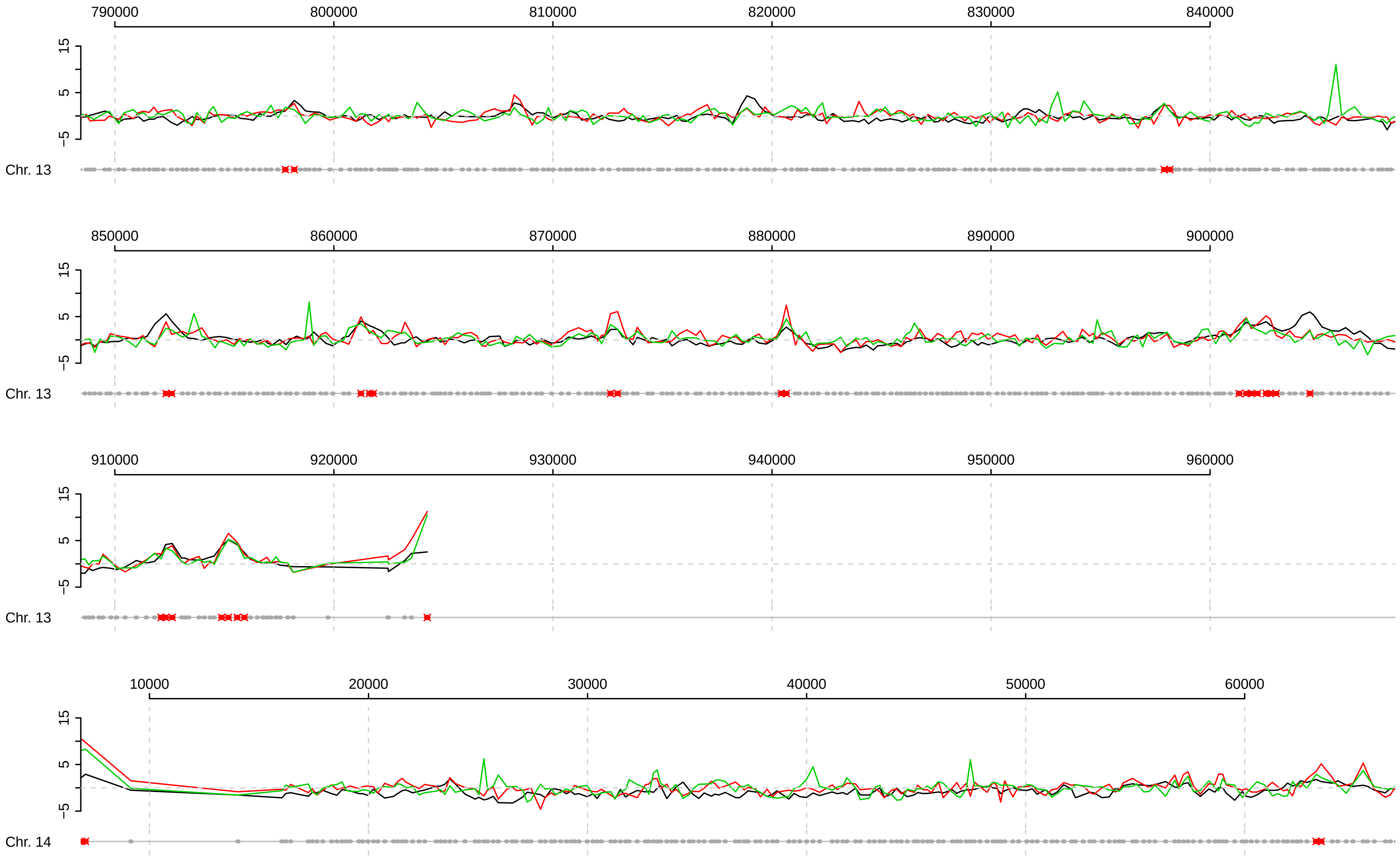

790000
800000
810000
820000
830000
840000
15
5
−5
Chr. 13
850000
860000
870000
880000
890000
900000
15
5
−5
Chr. 13
910000
920000
930000
940000
950000
960000
15
5
−5
Chr. 13
10000
20000
30000
40000
50000
60000
15
5
−5
Chr. 14

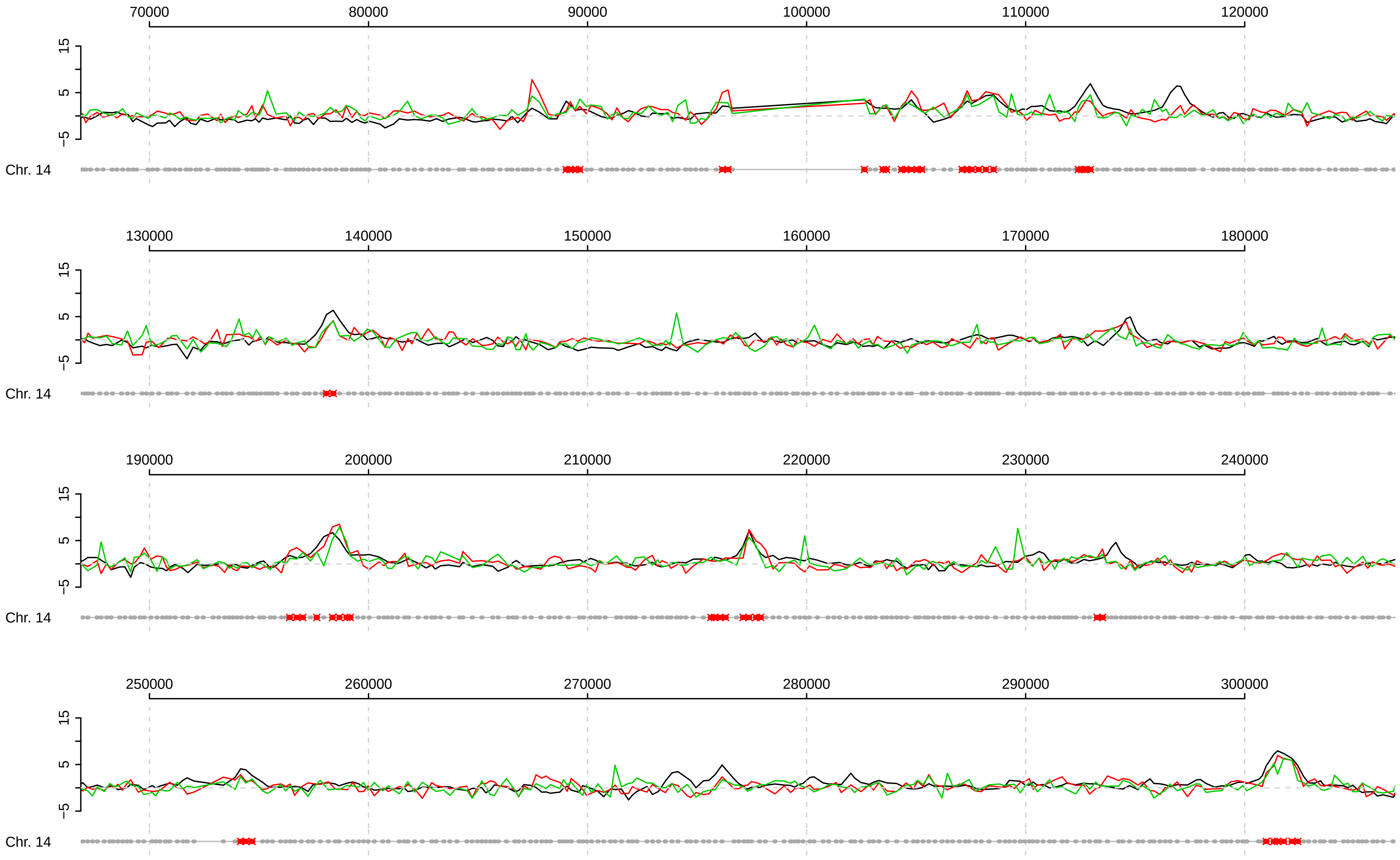

70000
80000
90000
100000
110000
120000
15
5
−5
Chr. 14
130000
140000
150000
160000
170000
180000
15
5
−5
Chr. 14
190000
200000
210000
220000
230000
240000
15
5
−5
Chr. 14
250000
260000
270000
280000
290000
300000
15
5
−5
Chr. 14

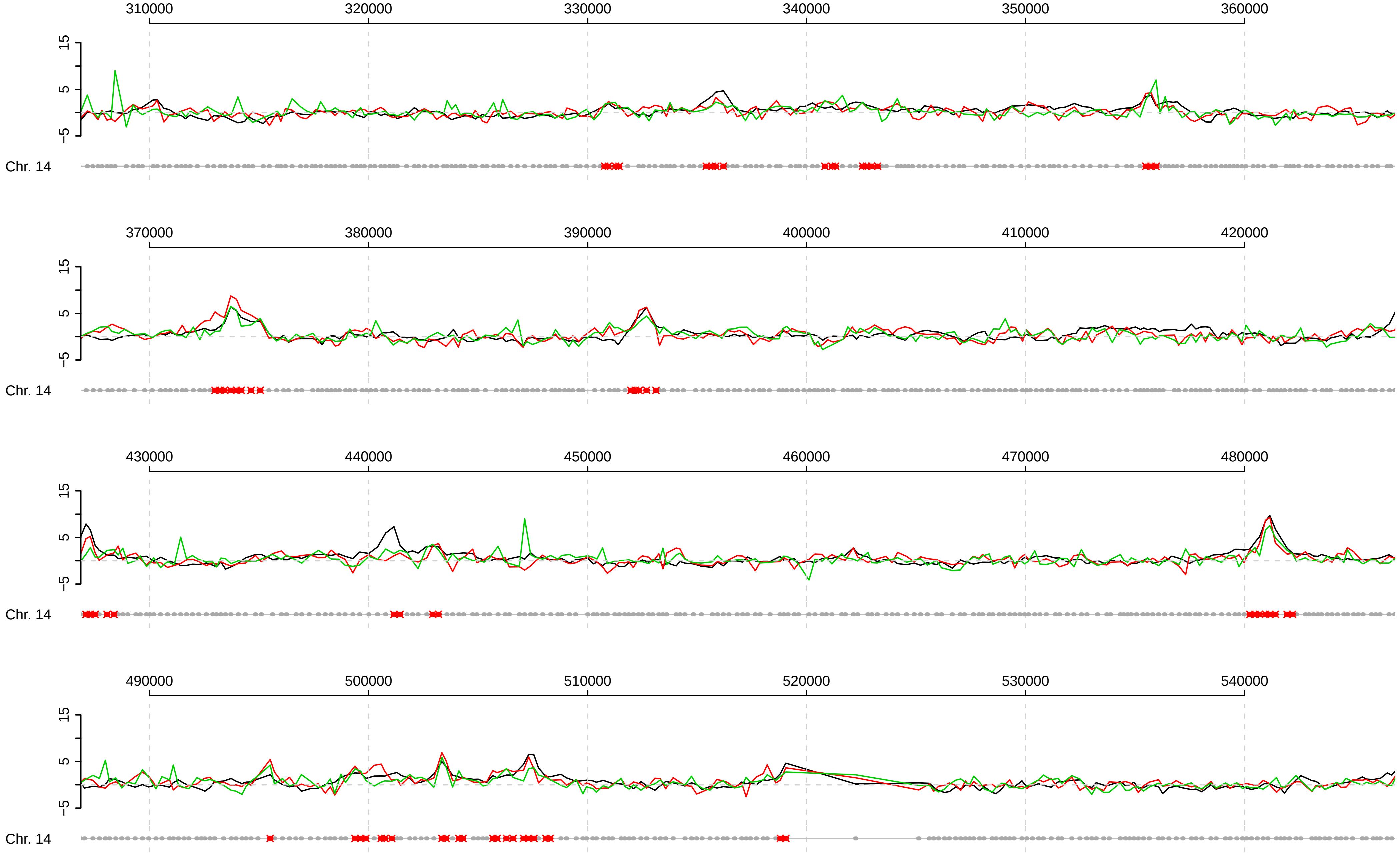
310000
320000
330000
340000
350000
360000
15
5
−5
Chr. 14
370000
380000
390000
400000
410000
420000
15
5
−5
Chr. 14
430000
440000
450000
460000
470000
480000
15
5
−5
Chr. 14
490000
500000
510000
520000
530000
540000
15
5
−5
Chr. 14

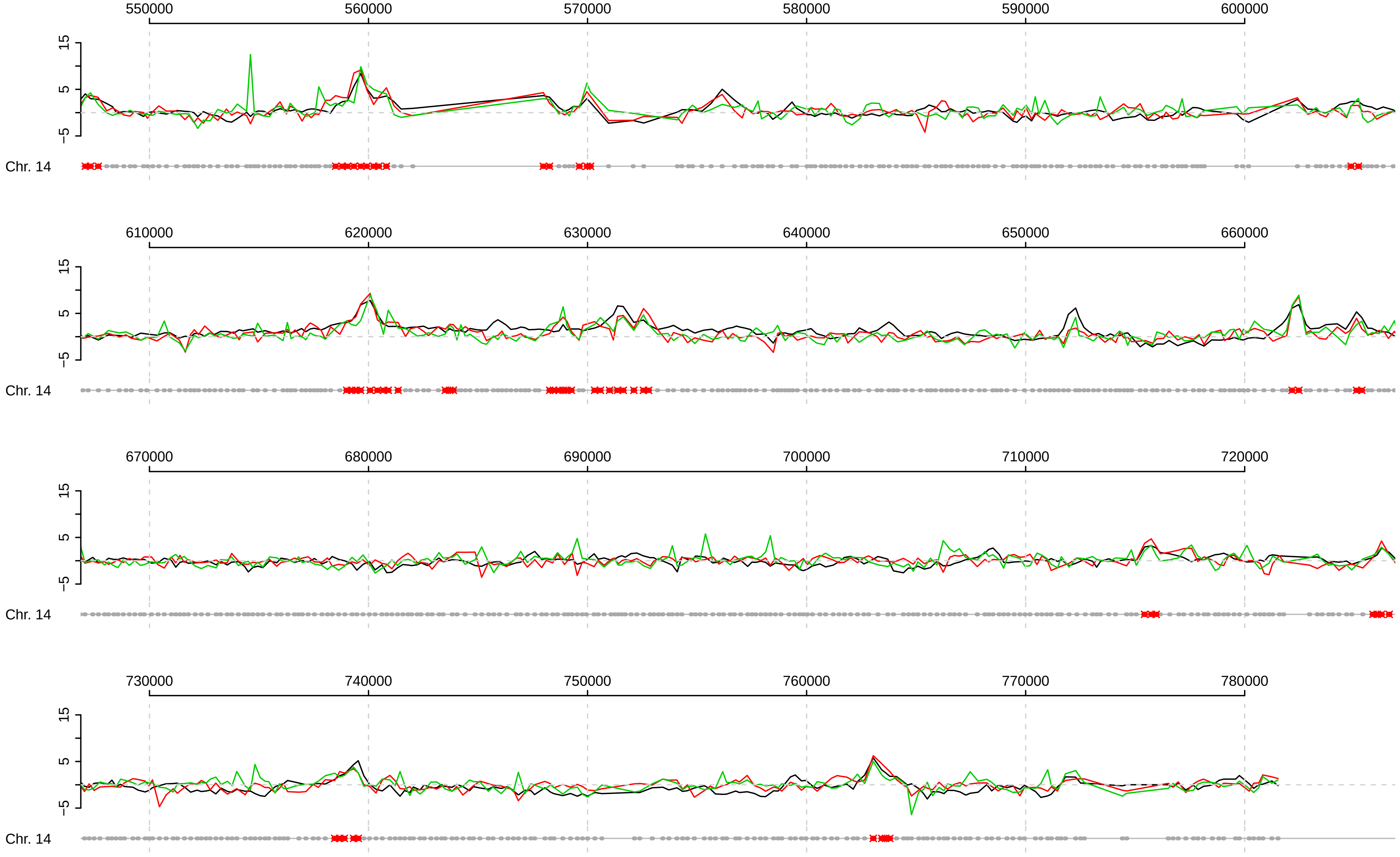
550000
560000
570000
580000
590000
600000
15
5
−5
Chr. 14
610000
620000
630000
640000
650000
660000
15
5
−5
Chr. 14
670000
680000
690000
700000
710000
720000
15
5
−5
Chr. 14
730000
740000
750000
760000
770000
780000
15
5
−5
Chr. 14

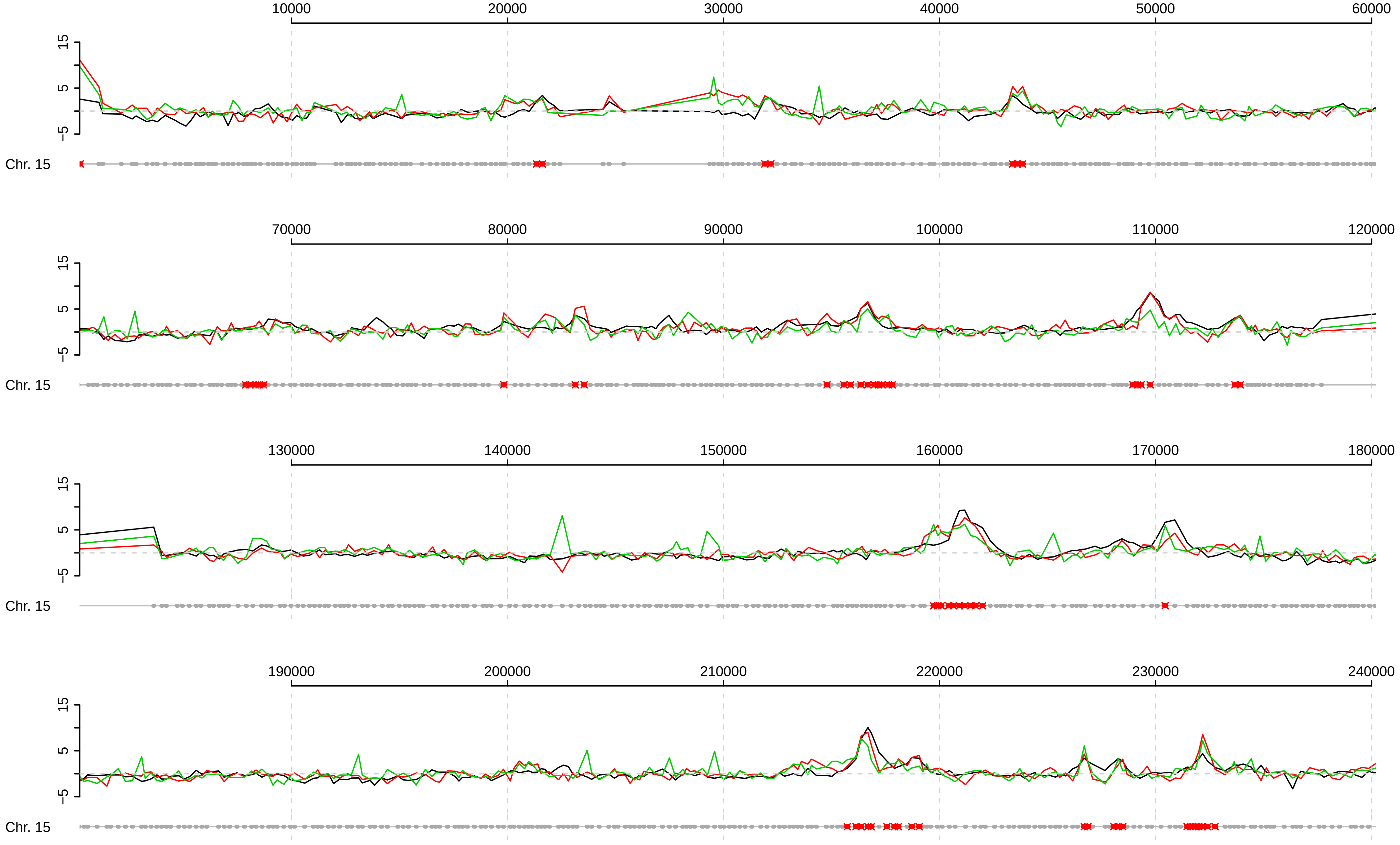

10000
20000
30000
40000
50000
60000
15
5
−5
Chr. 15
70000
80000
90000
100000
110000
120000
15
5
−5
Chr. 15
130000
140000
150000
160000
170000
180000
15
5
−5
Chr. 15
190000
200000
210000
220000
230000
240000
15
5
−5
Chr. 15

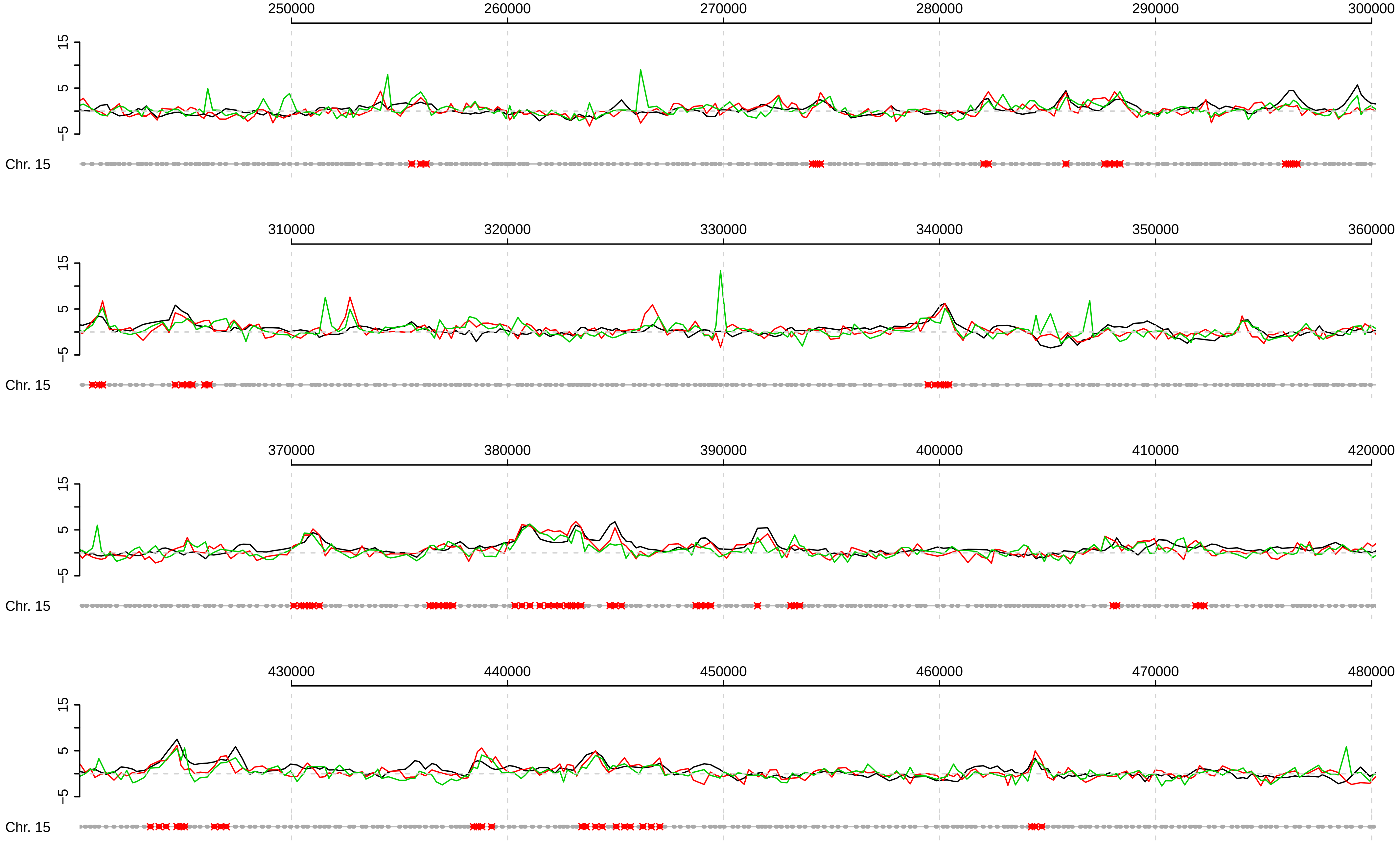

250000
260000
270000
280000
290000
300000
15
5
−5
Chr. 15
310000
320000
330000
340000
350000
360000
15
5
−5
Chr. 15
370000
380000
390000
400000
410000
420000
15
5
−5
Chr. 15
430000
440000
450000
460000
470000
480000
15
5
−5
Chr. 15

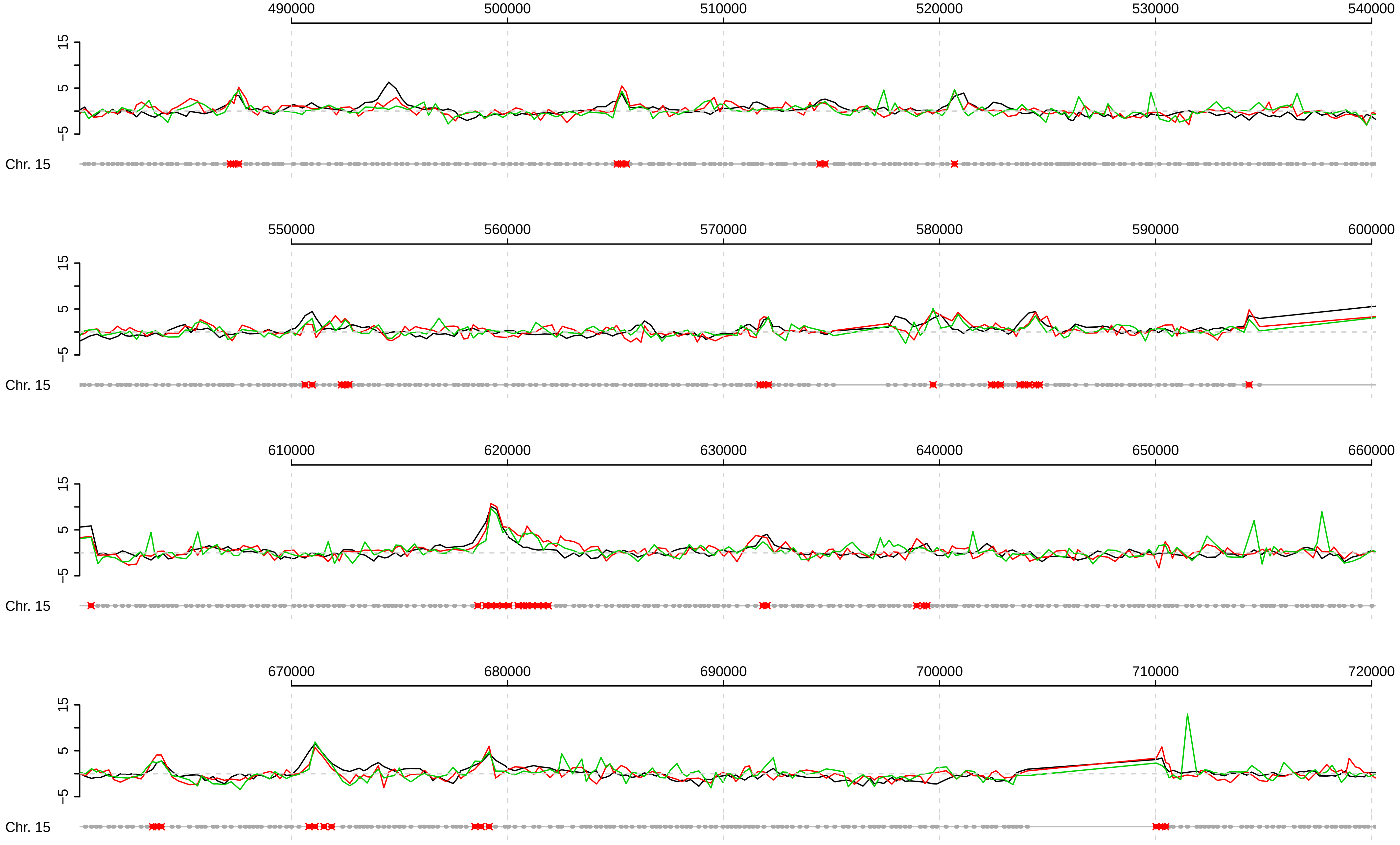
490000
500000
510000
520000
530000
540000
15
5
−5
Chr. 15
550000
560000
570000
580000
590000
600000
15
5
−5
Chr. 15
610000
620000
630000
640000
650000
660000
15
5
−5
Chr. 15
670000
680000
690000
700000
710000
720000
15
5
−5
Chr. 15

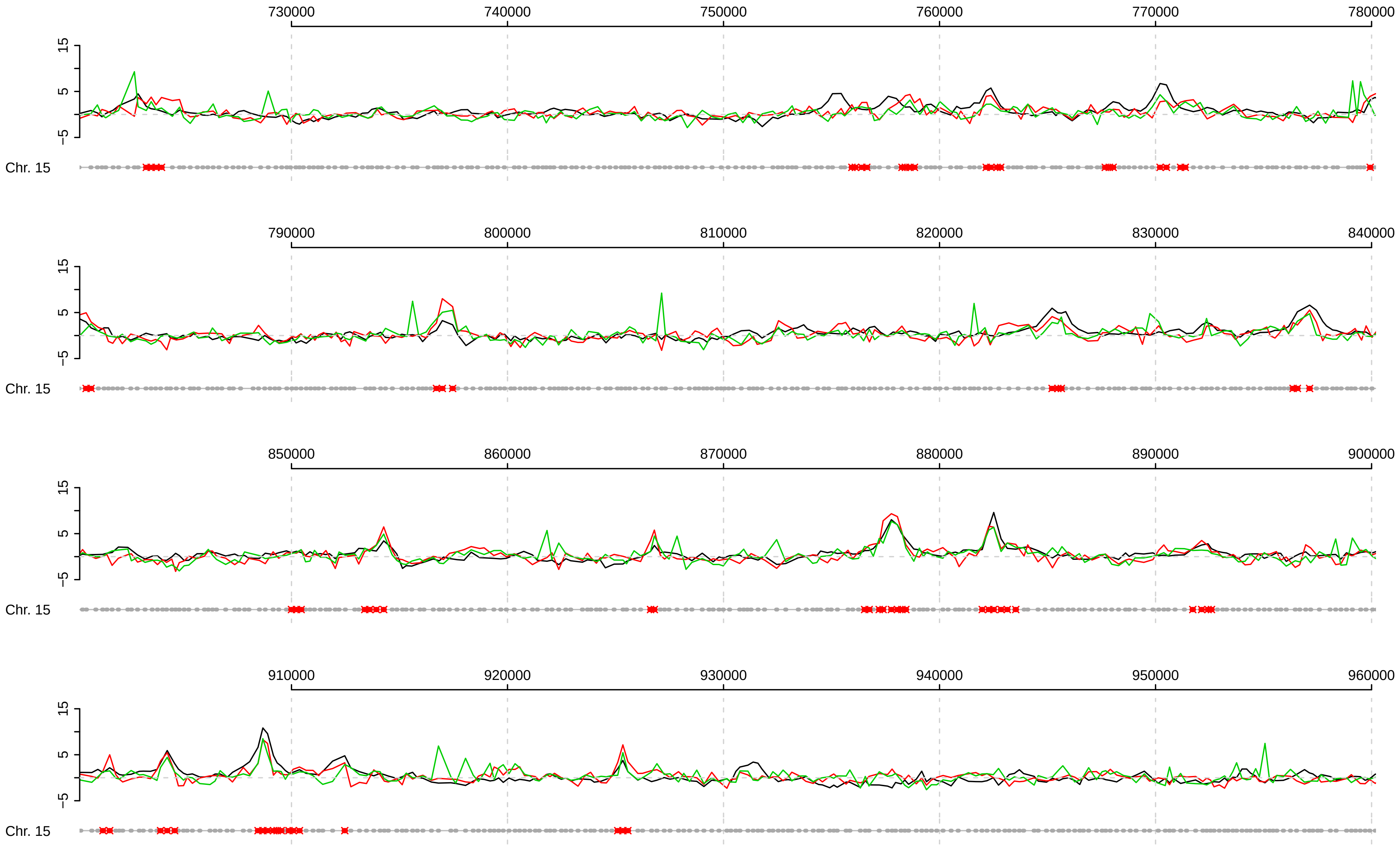

730000
740000
750000
760000
770000
780000
15
5
−5
Chr. 15
790000
800000
810000
820000
830000
840000
15
5
−5
Chr. 15
850000
860000
870000
880000
890000
900000
15
5
−5
Chr. 15
910000
920000
930000
940000
950000
960000
15
5
−5
Chr. 15

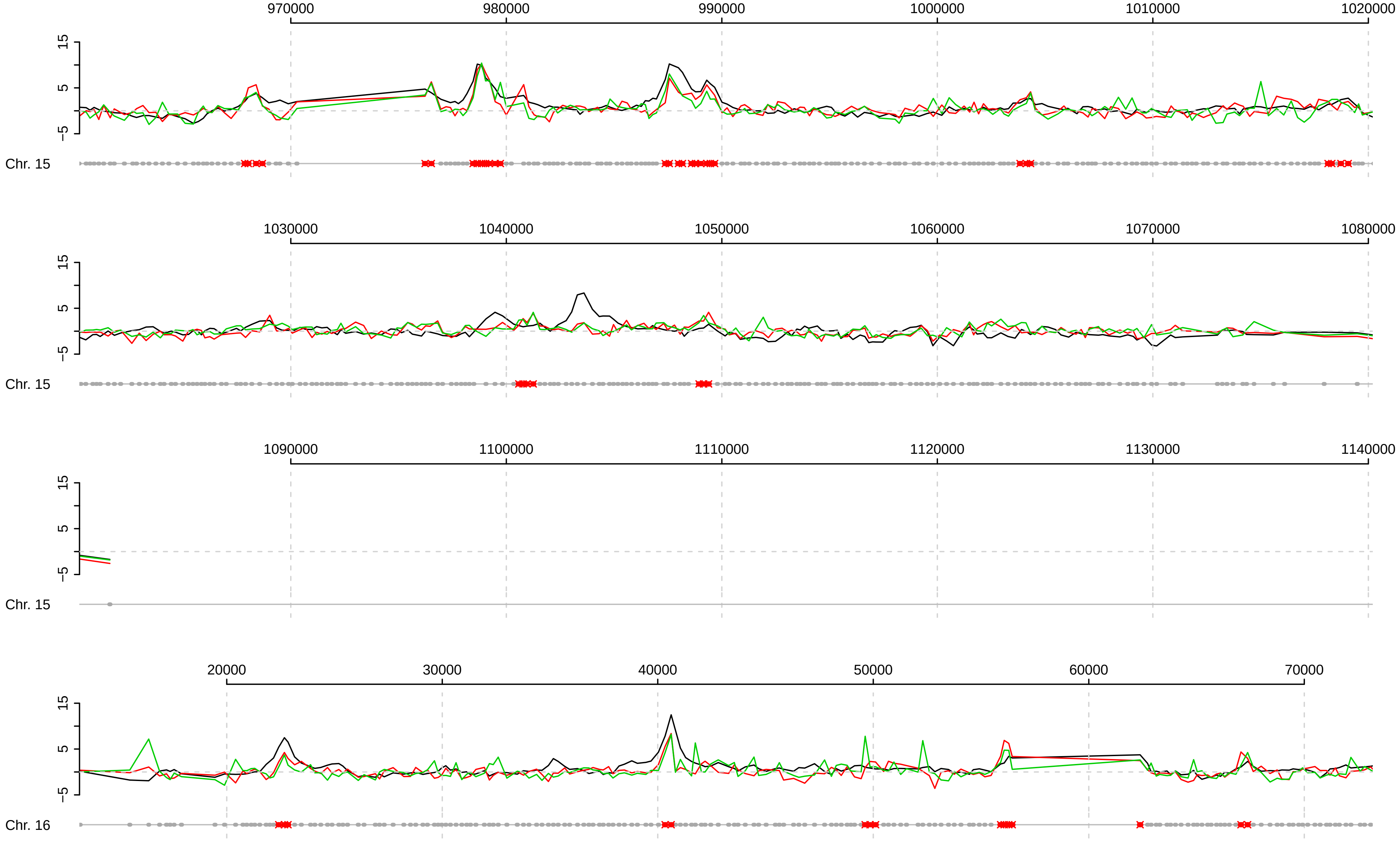
970000
980000
990000
1000000
1010000
1020000
15
5
−5
Chr. 15
1030000
1040000
1050000
1060000
1070000
1080000
15
5
−5
Chr. 15
1090000
1100000
1110000
1120000
1130000
1140000
15
5
−5
Chr. 15
20000
30000
40000
50000
60000
70000
15
5
−5
Chr. 16

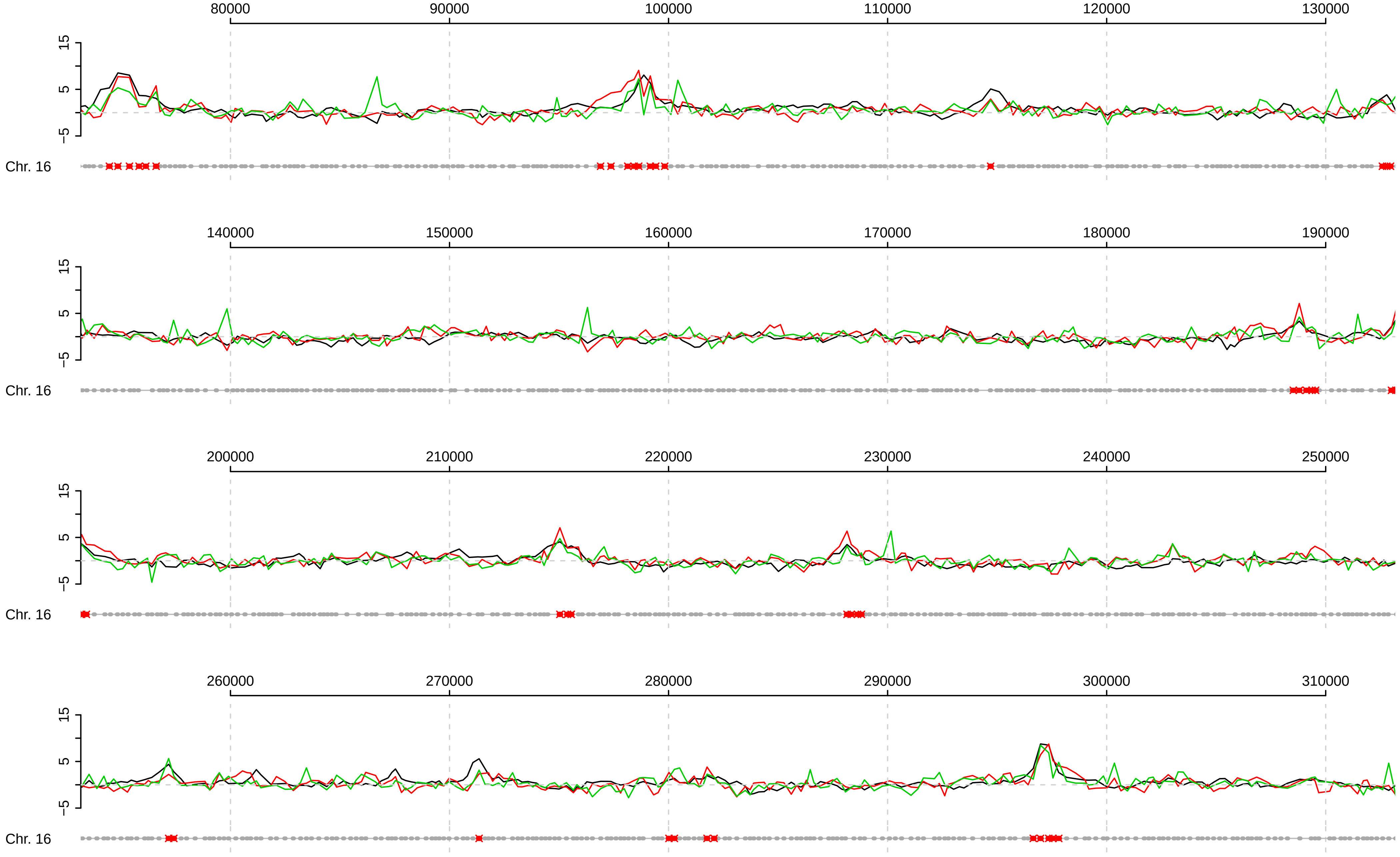
80000
90000
100000
110000
120000
130000
15
5
−5
Chr. 16
140000
150000
160000
170000
180000
190000
15
5
−5
Chr. 16
200000
210000
220000
230000
240000
250000
15
5
−5
Chr. 16
260000
270000
280000
290000
300000
310000
15
5
−5
Chr. 16

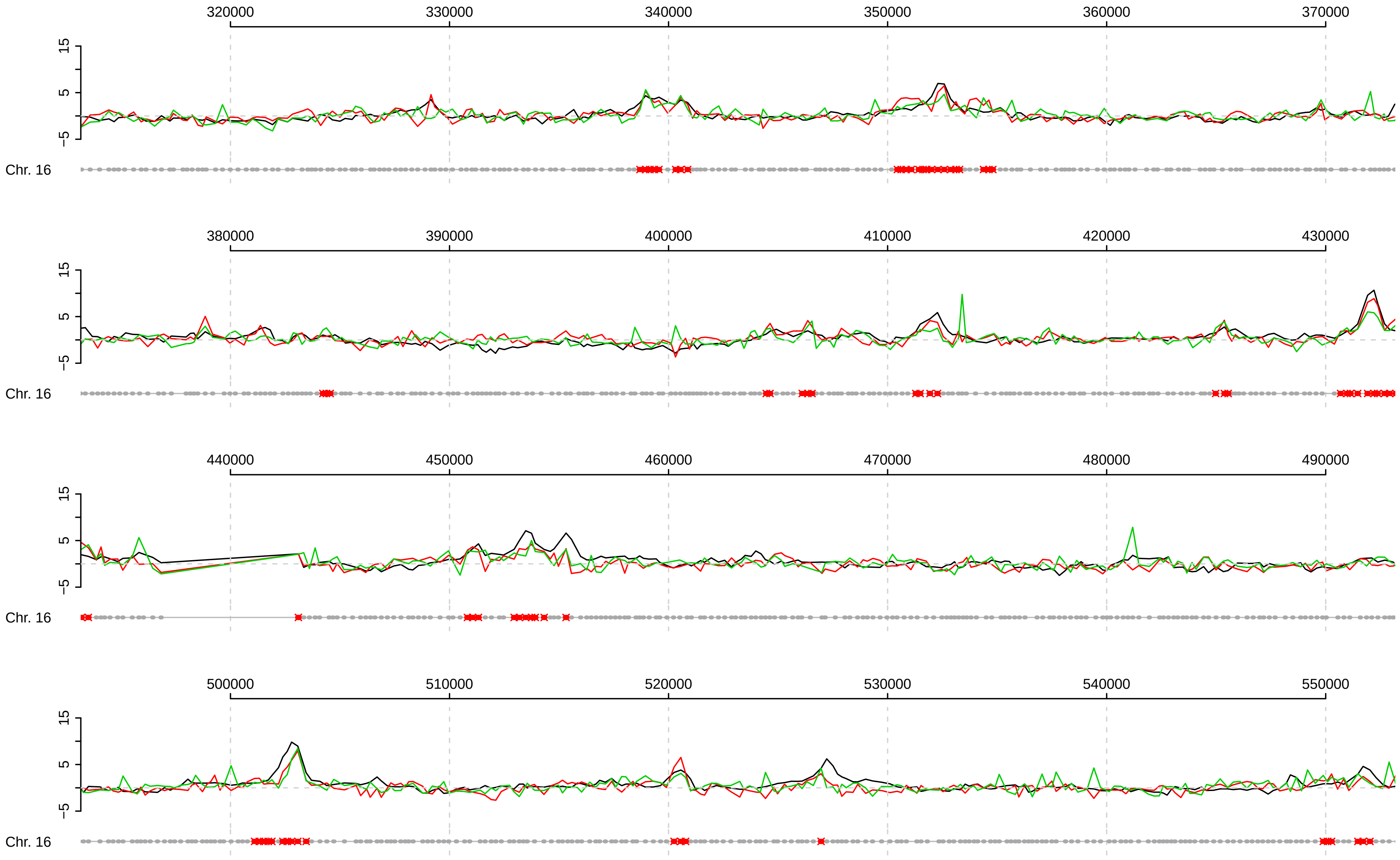
320000
330000
340000
350000
360000
370000
15
5
−5
Chr. 16
380000
390000
400000
410000
420000
430000
15
5
−5
Chr. 16
440000
450000
460000
470000
480000
490000
15
5
−5
Chr. 16
500000
510000
520000
530000
540000
550000
15
5
−5
Chr. 16

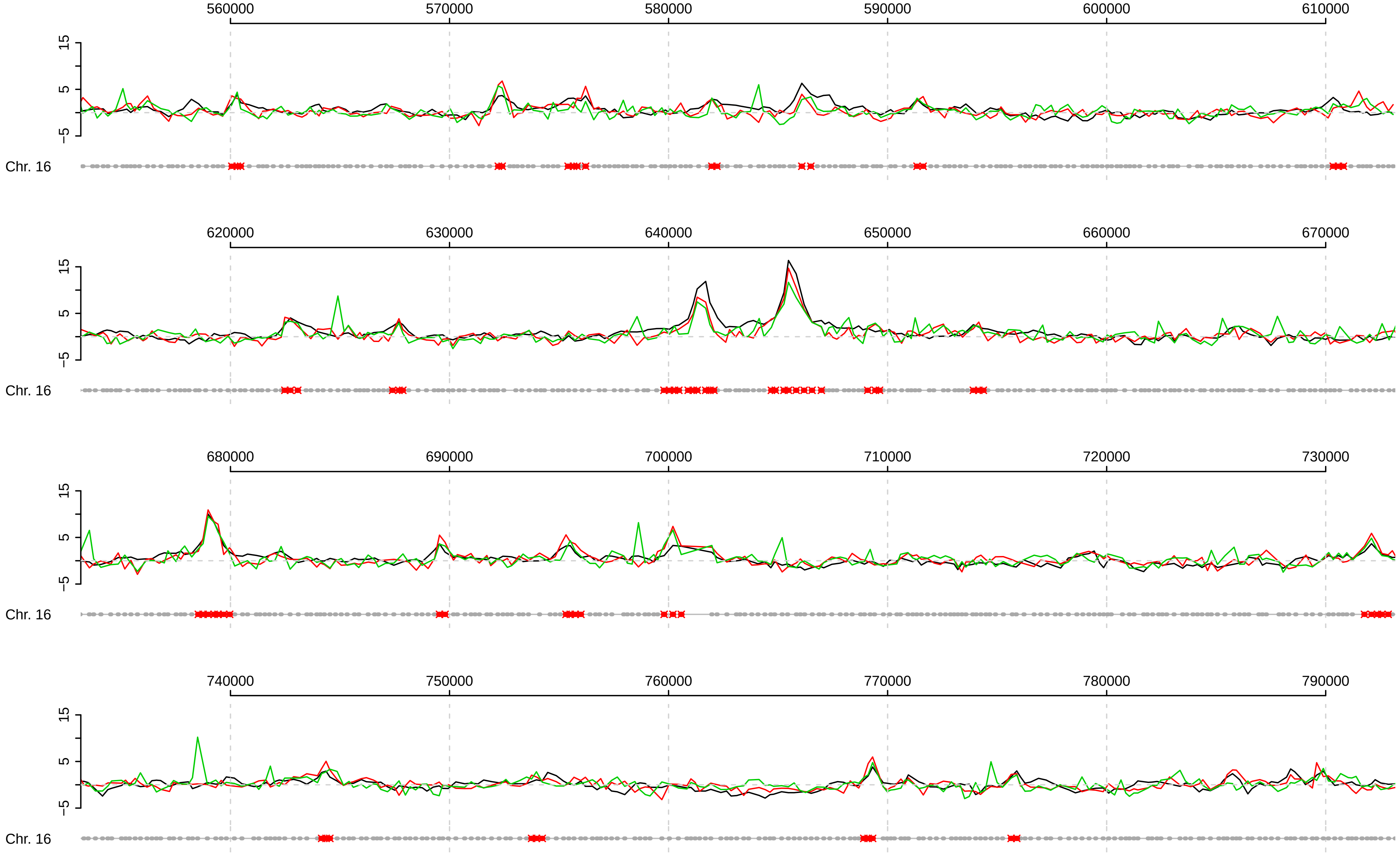
560000
570000
580000
590000
600000
610000
15
5
−5
Chr. 16
620000
630000
640000
650000
660000
670000
15
5
−5
Chr. 16
680000
690000
700000
710000
720000
730000
15
5
−5
Chr. 16
740000
750000
760000
770000
780000
790000
15
5
−5
Chr. 16

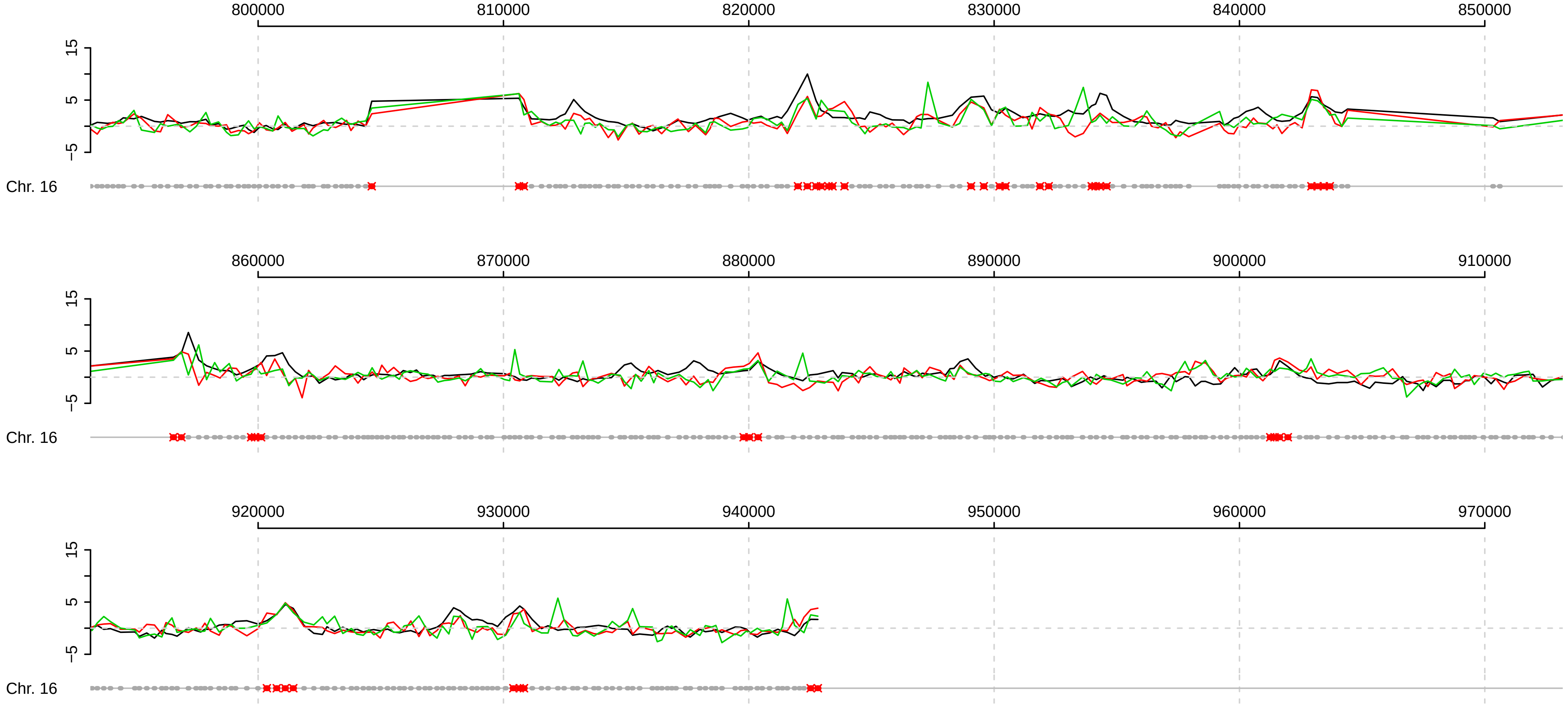
800000
810000
820000
830000
840000
850000
15
5
−5
Chr. 16
860000
870000
880000
890000
900000
910000
15
5
−5
Chr. 16
920000
930000
940000
950000
960000
970000
15
5
−5
Chr. 16

The following plots show Gcn5 enrichment over the whole genome in cells treated with UV, time point 2 (T2). The three line colours show three replicate datasets. The red crosses show probes detected as enriched.

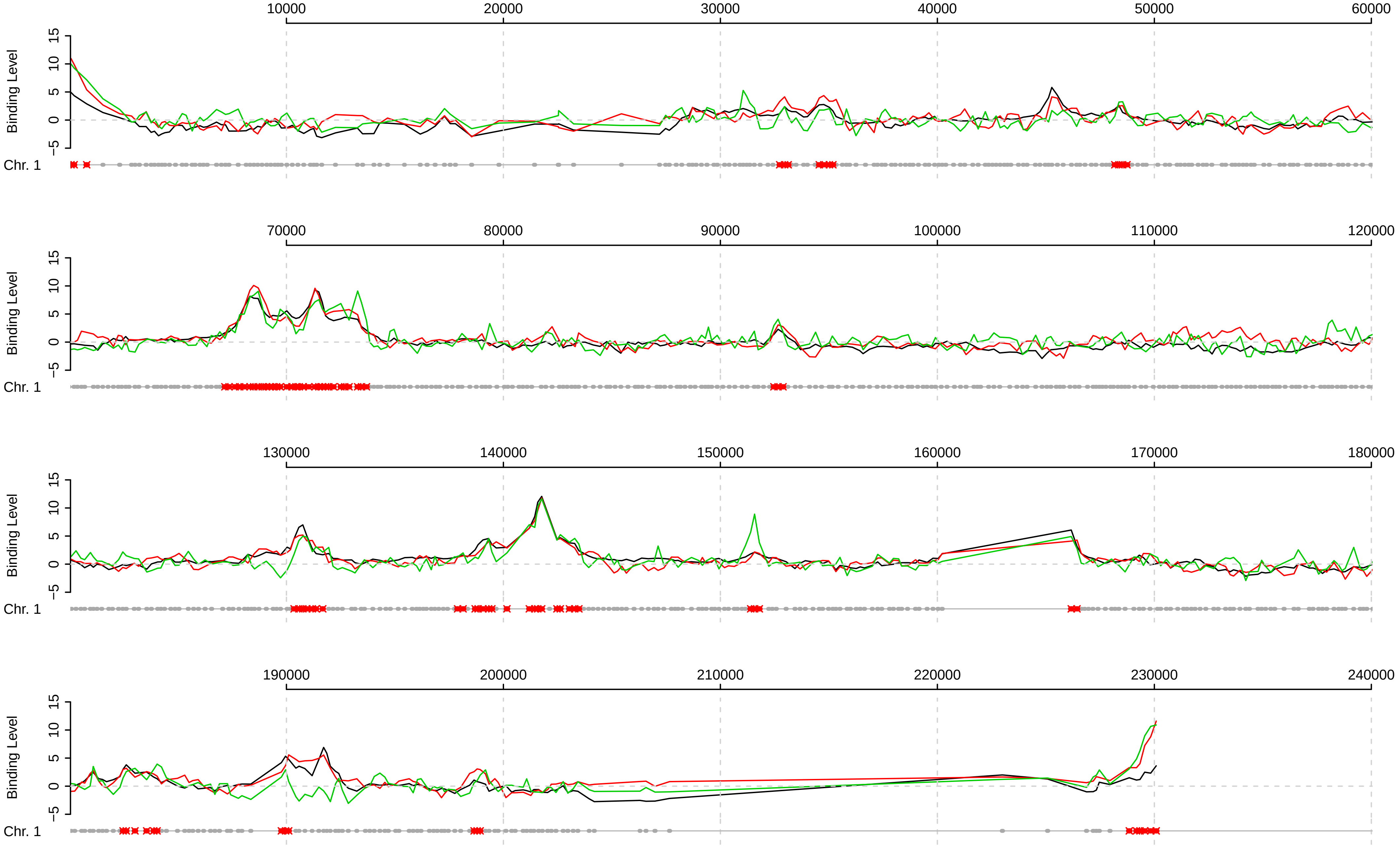

10000
20000
30000
40000
50000
60000
Binding Level
15
10
5
0
−5
Chr. 1
70000
80000
90000
100000
110000
120000
Binding Level
Chr. 1
130000
140000
150000
160000
170000
180000
Binding Level
Chr. 1
190000
200000
210000
220000
230000
240000
Binding Level
Chr. 1

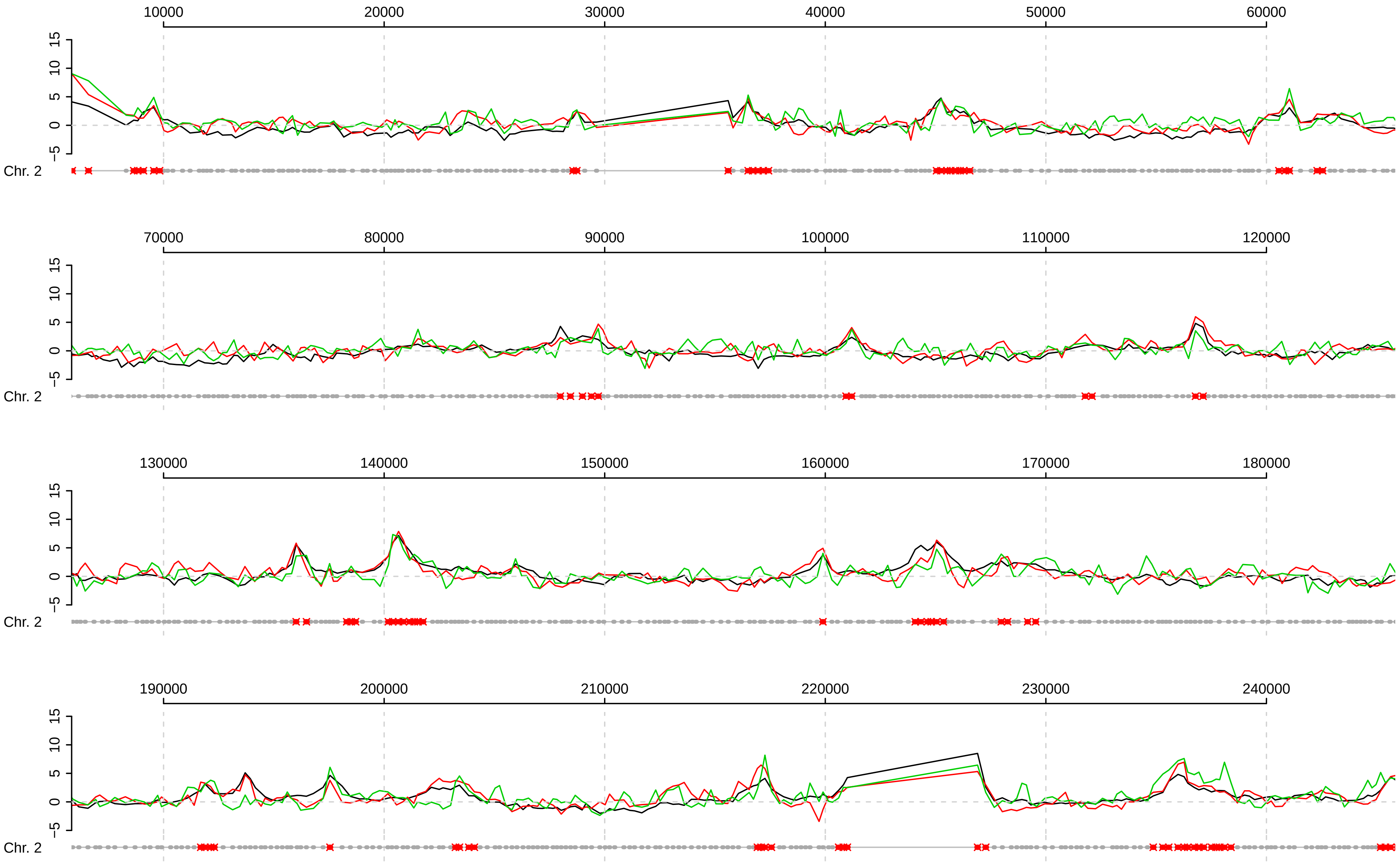

10000
20000
30000
40000
50000
60000
−5
0
5
10
15
Chr. 2
70000
80000
90000
100000
110000
120000
−5
0
5
10
15
Chr. 2
130000
140000
150000
160000
170000
180000
−5
0
5
10
15
Chr. 2
190000
200000
210000
220000
230000
240000
−5
0
5
10
15
Chr. 2

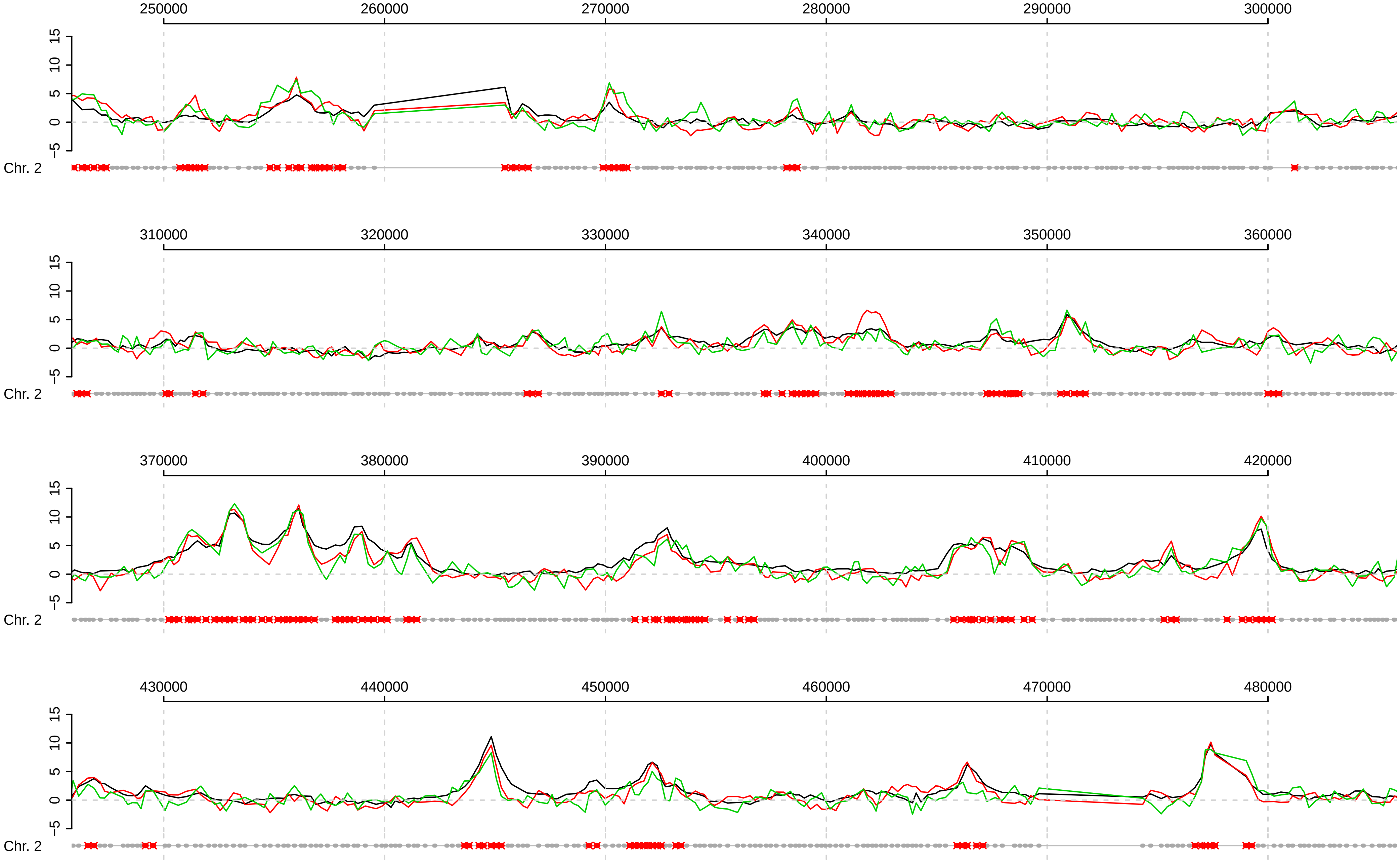
250000
260000
270000
280000
290000
300000
310000
320000
330000
340000
350000
360000
370000
380000
390000
400000
410000
420000
430000
440000
450000
460000
470000
480000
−5
0
5
10
15
Chr. 2

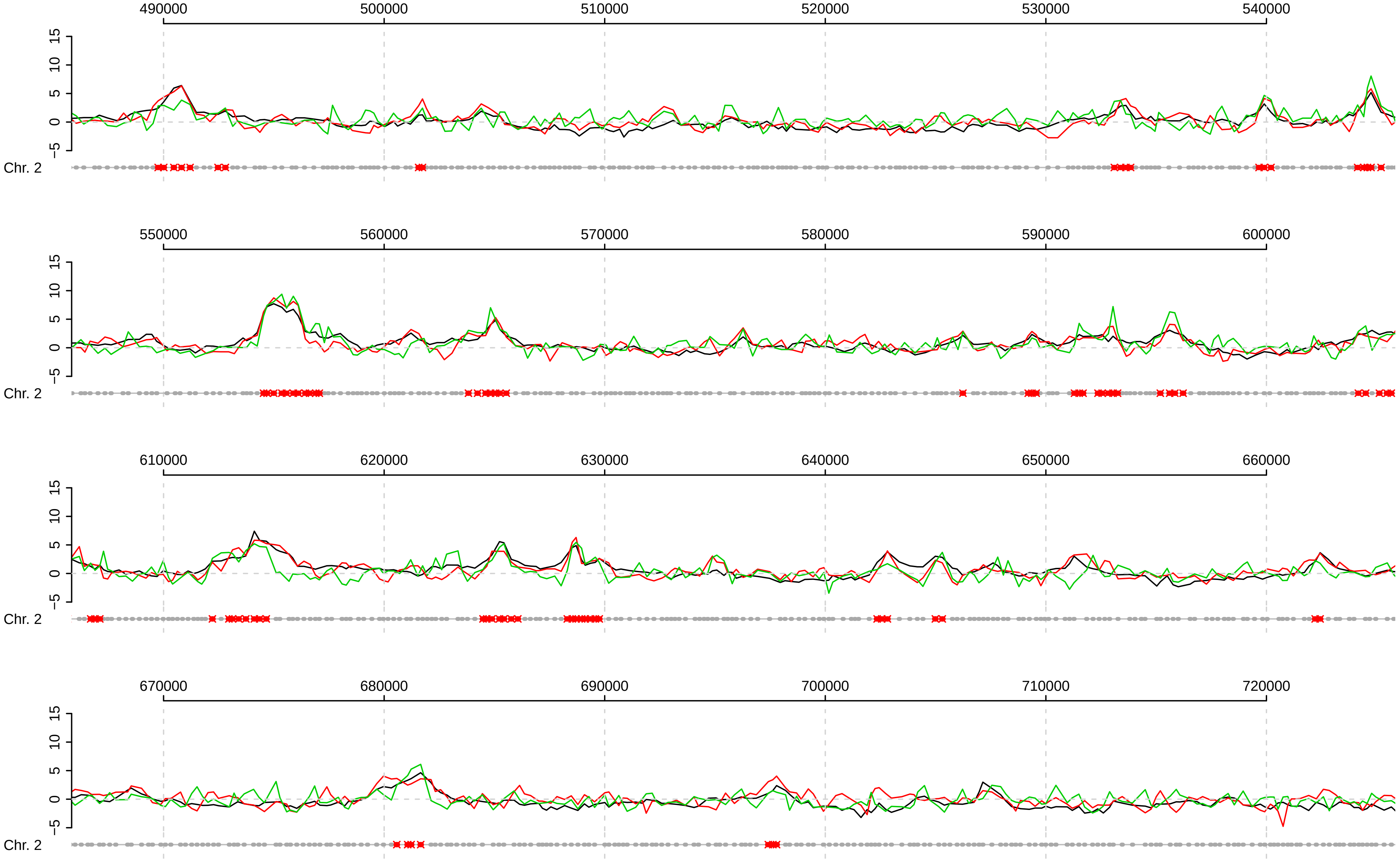
490000
500000
510000
520000
530000
540000
550000
560000
570000
580000
590000
600000
610000
620000
630000
640000
650000
660000
670000
680000
690000
700000
710000
720000
−5
0
5
10
15
Chr. 2

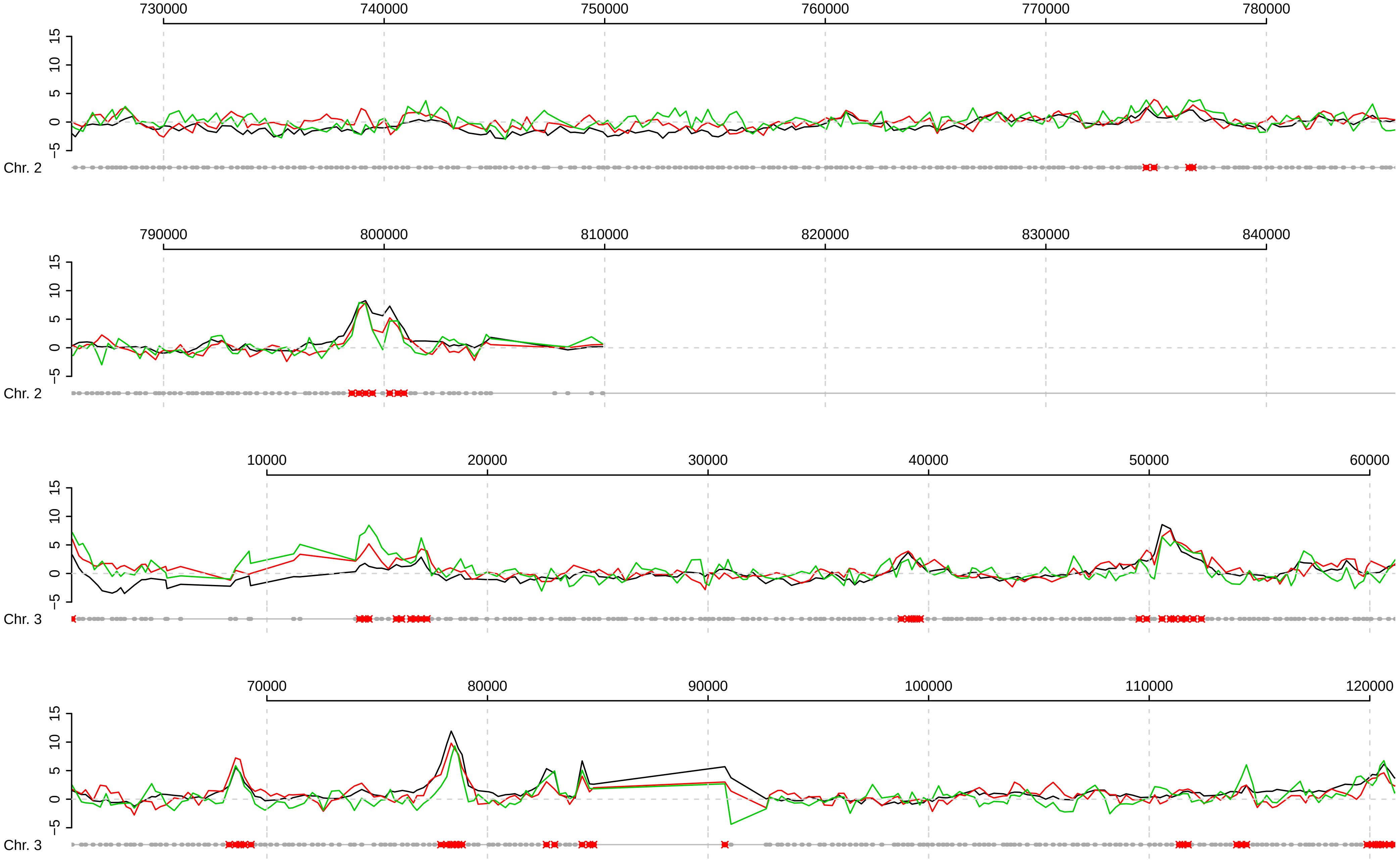

730000
740000
750000
760000
770000
780000
15
10
5
0
−5
Chr. 2
790000
800000
810000
820000
830000
840000
15
10
5
0
−5
Chr. 2
10000
20000
30000
40000
50000
60000
15
10
5
0
−5
Chr. 3
70000
80000
90000
100000
110000
120000
15
10
5
0
−5
Chr. 3

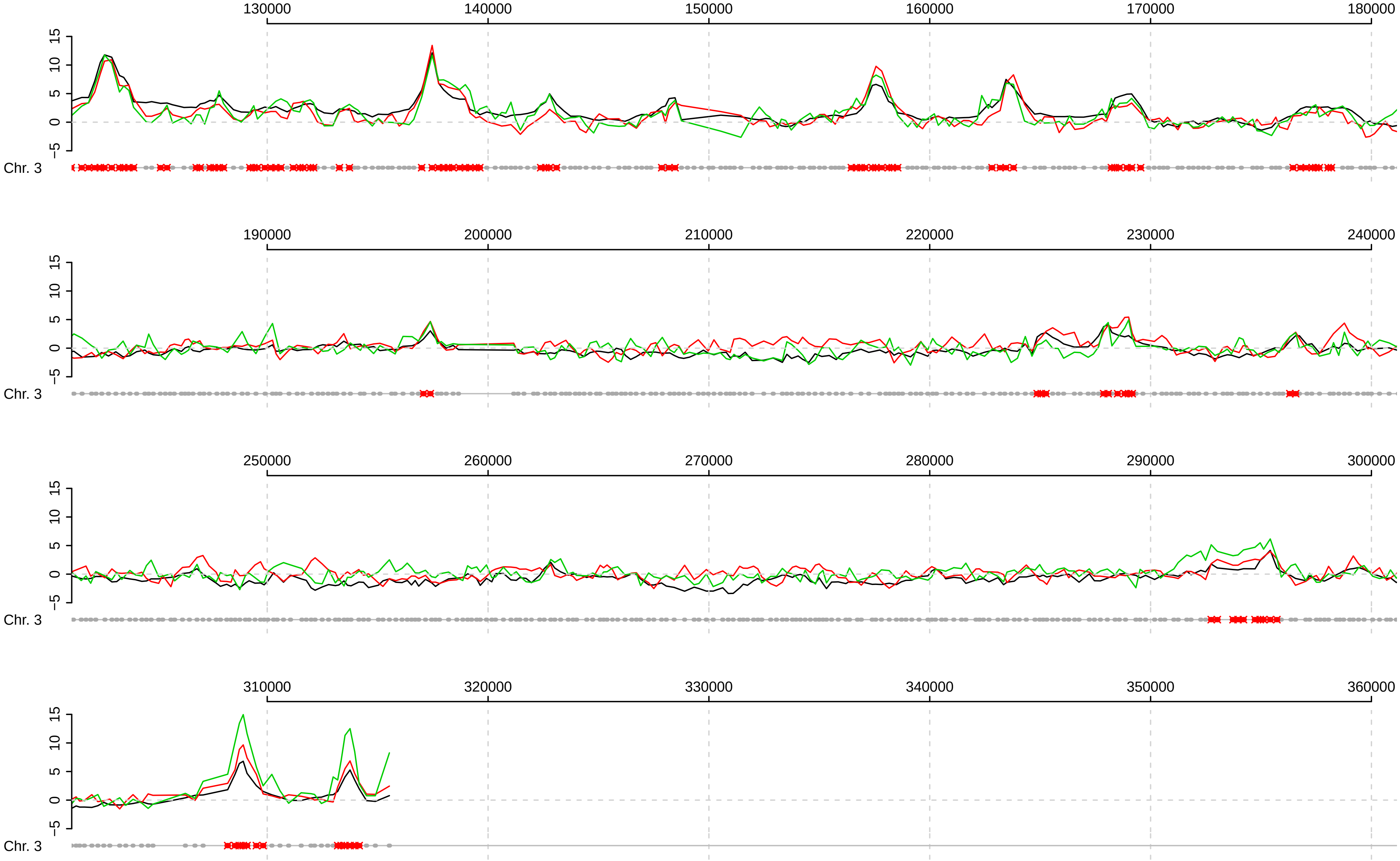

130000
140000
150000
160000
170000
180000
190000
200000
210000
220000
230000
240000
250000
260000
270000
280000
290000
300000
310000
320000
330000
340000
350000
360000
−5
0
5
10
15
Chr. 3

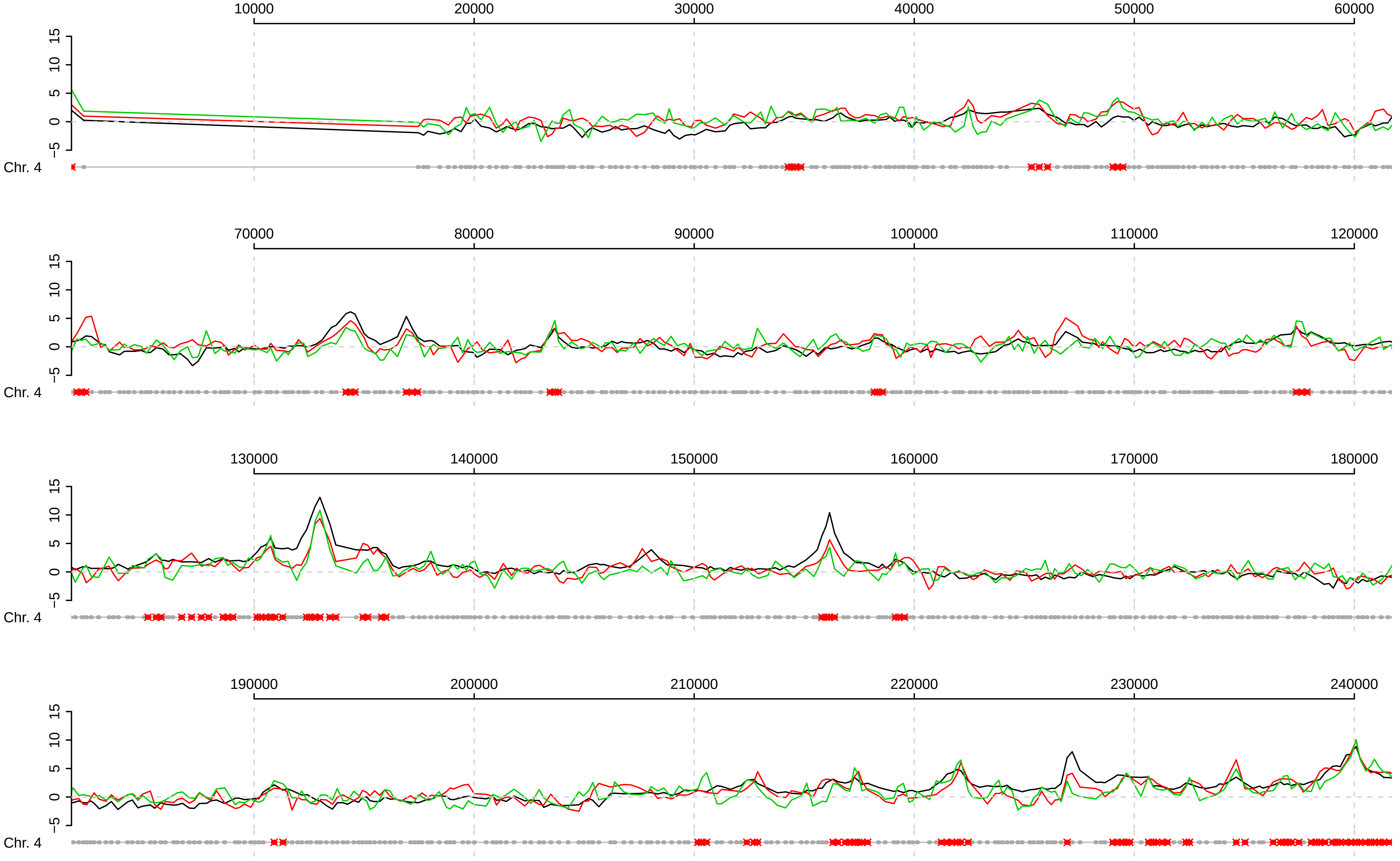

10000
20000
30000
40000
50000
60000
15
10
5
0
−5
Chr. 4
70000
80000
90000
100000
110000
120000
15
10
5
0
−5
Chr. 4
130000
140000
150000
160000
170000
180000
15
10
5
0
−5
Chr. 4
190000
200000
210000
220000
230000
240000
15
10
5
0
−5
Chr. 4

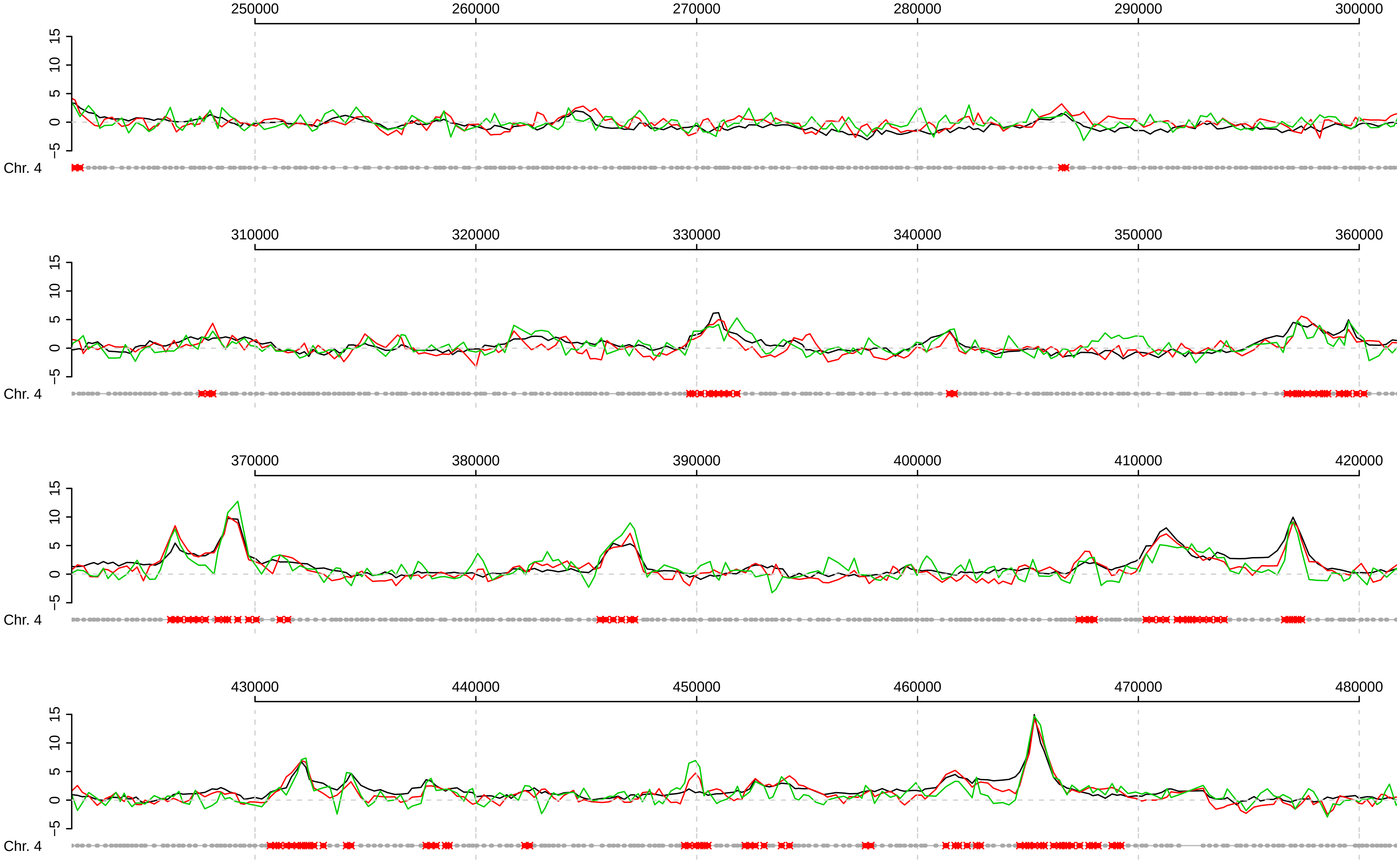
250000
260000
270000
280000
290000
300000
310000
320000
330000
340000
350000
360000
370000
380000
390000
400000
410000
420000
430000
440000
450000
460000
470000
480000
−5
0
5
10
15
Chr. 4

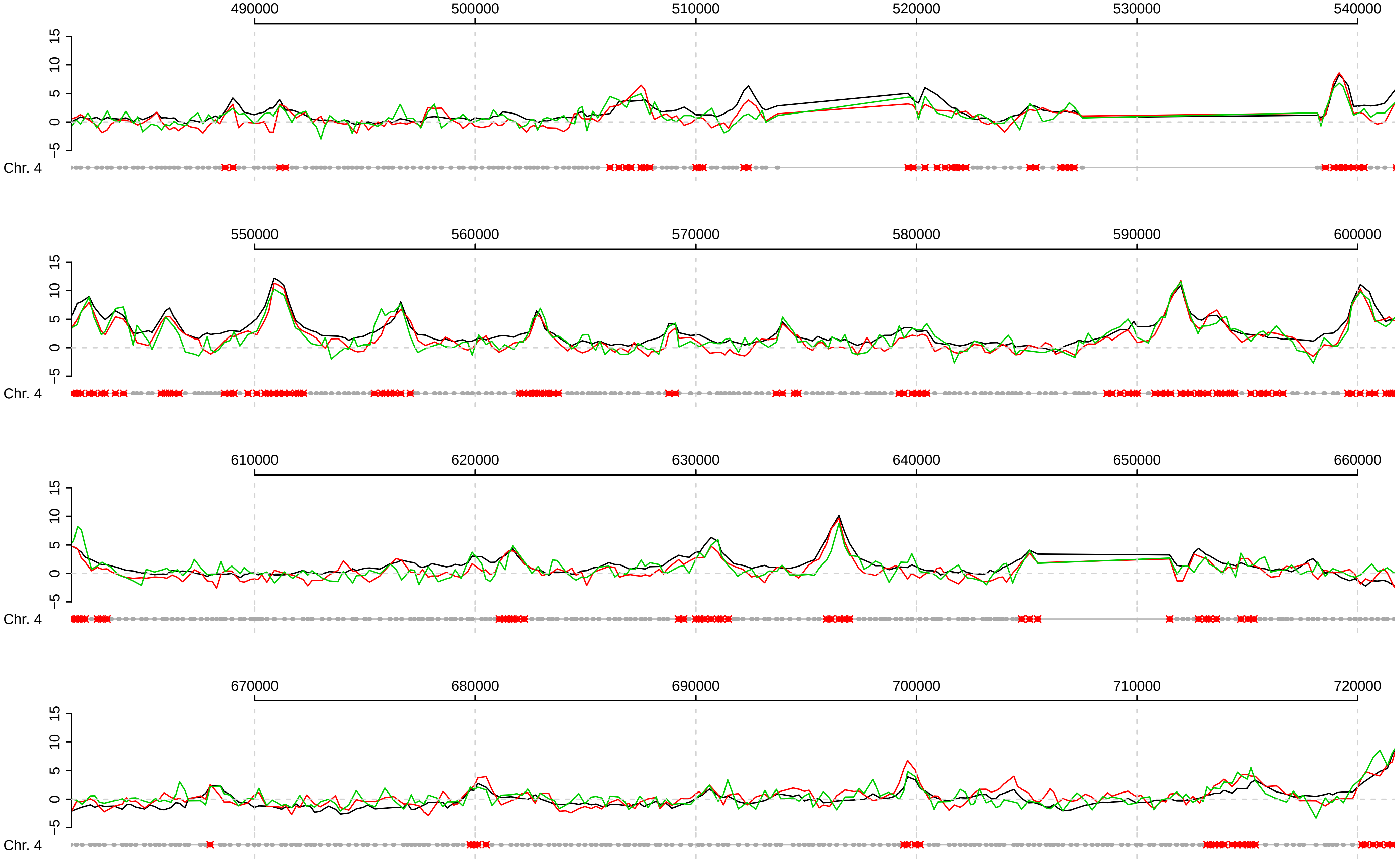
490000
500000
510000
520000
530000
540000
550000
560000
570000
580000
590000
600000
610000
620000
630000
640000
650000
660000
670000
680000
690000
700000
710000
720000
15
10
5
0
−5
Chr. 4

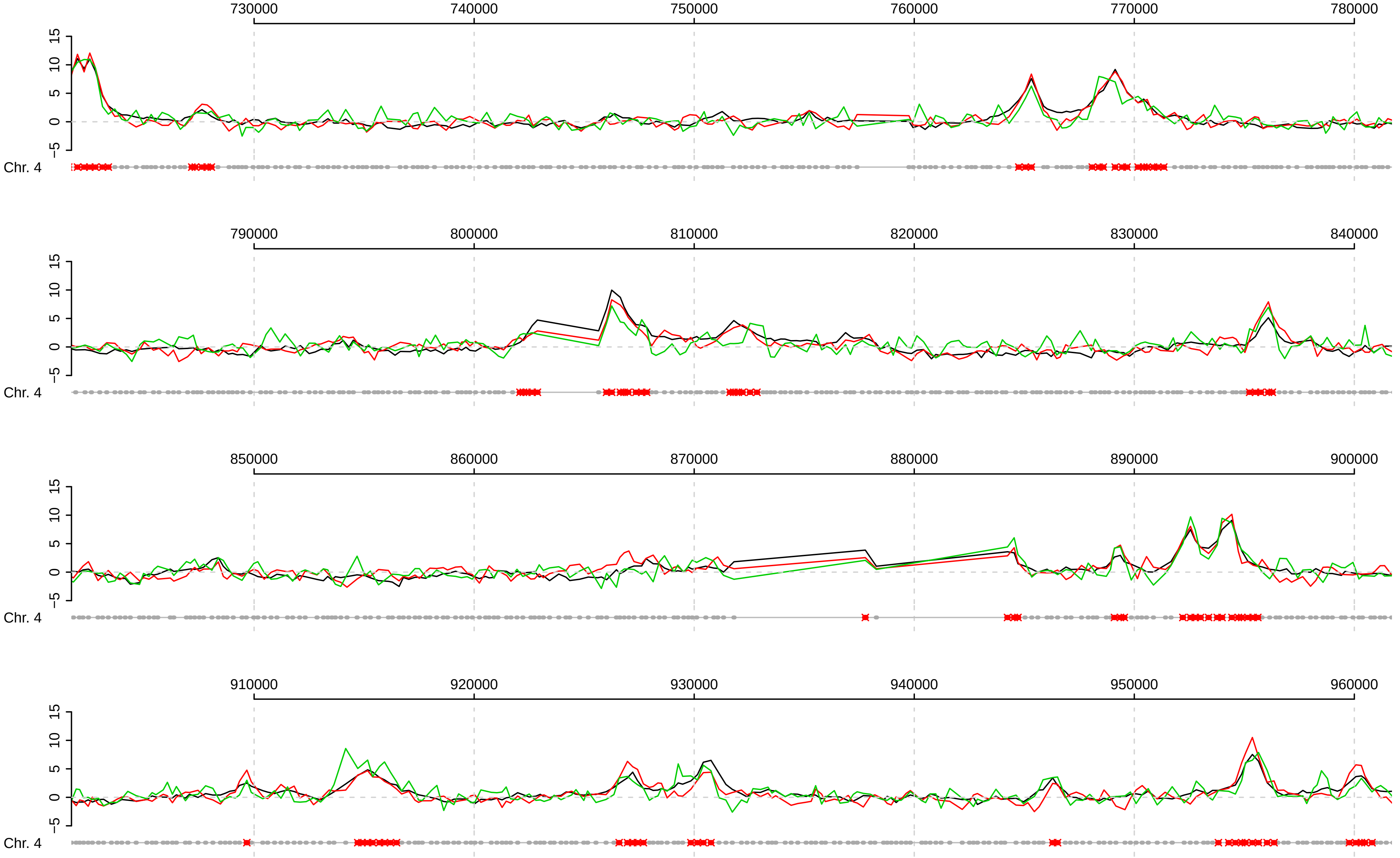
730000
740000
750000
760000
770000
780000
Chr. 4
790000
800000
810000
820000
830000
840000
Chr. 4
850000
860000
870000
880000
890000
900000
Chr. 4
910000
920000
930000
940000
950000
960000
Chr. 4

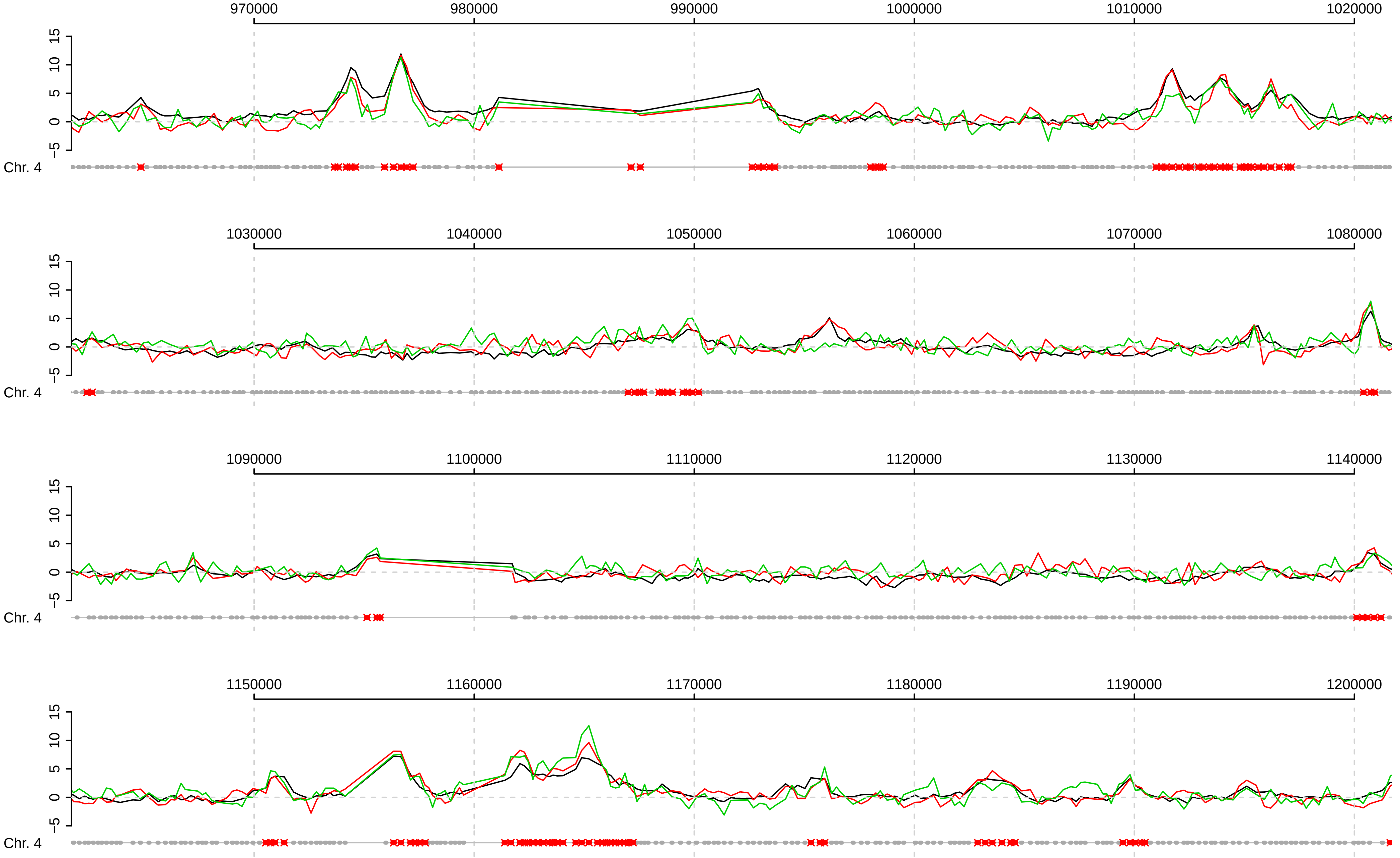

970000
980000
990000
1000000
1010000
1020000
15
10
5
0
−5
Chr. 4
1030000
1040000
1050000
1060000
1070000
1080000
15
10
5
0
−5
Chr. 4
1090000
1100000
1110000
1120000
1130000
1140000
15
10
5
0
−5
Chr. 4
1150000
1160000
1170000
1180000
1190000
1200000
15
10
5
0
−5
Chr. 4

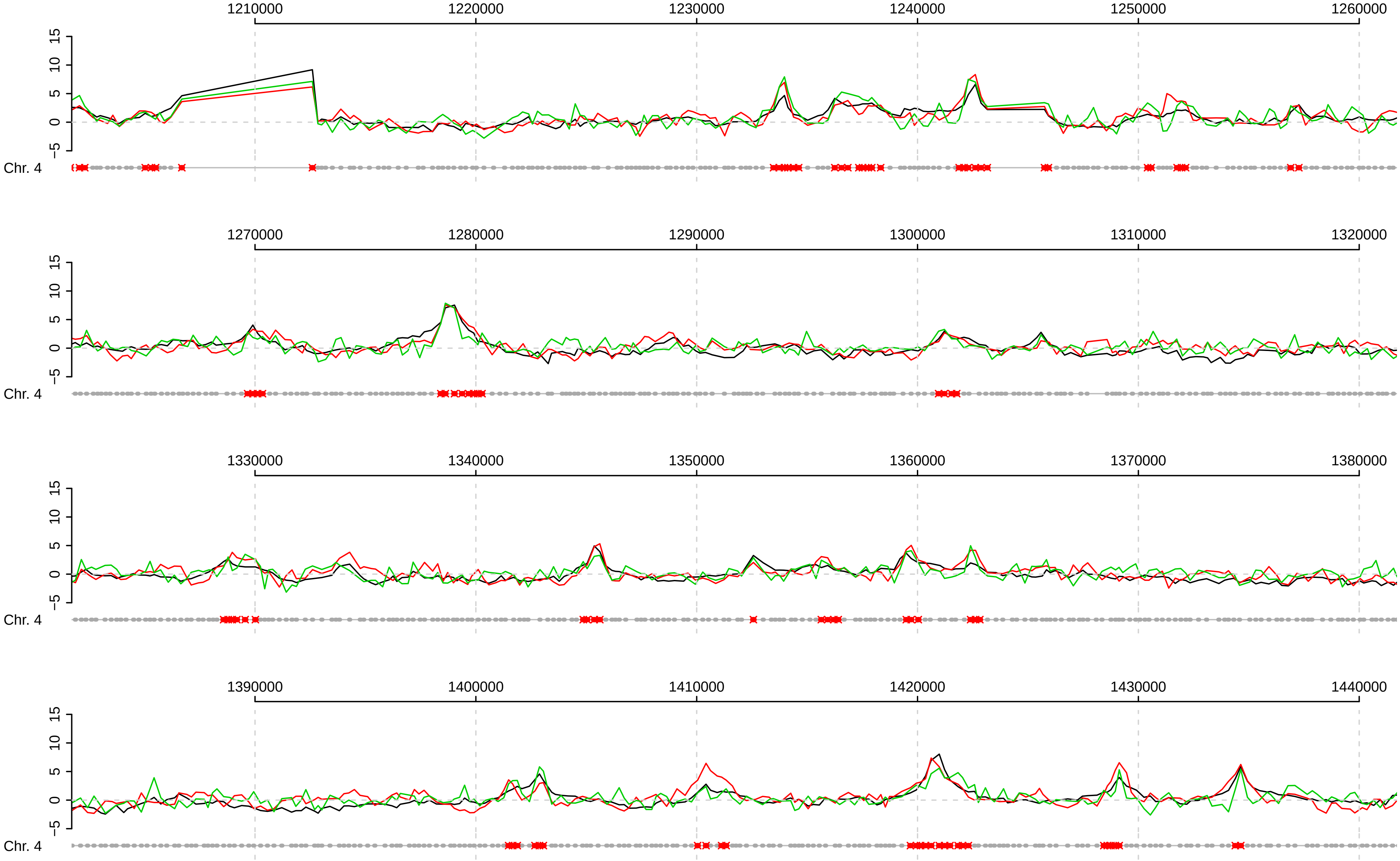

1210000
1220000
1230000
1240000
1250000
1260000
Chr. 4
1270000
1280000
1290000
1300000
1310000
1320000
Chr. 4
1330000
1340000
1350000
1360000
1370000
1380000
Chr. 4
1390000
1400000
1410000
1420000
1430000
1440000
Chr. 4
−5
0
5
10
15

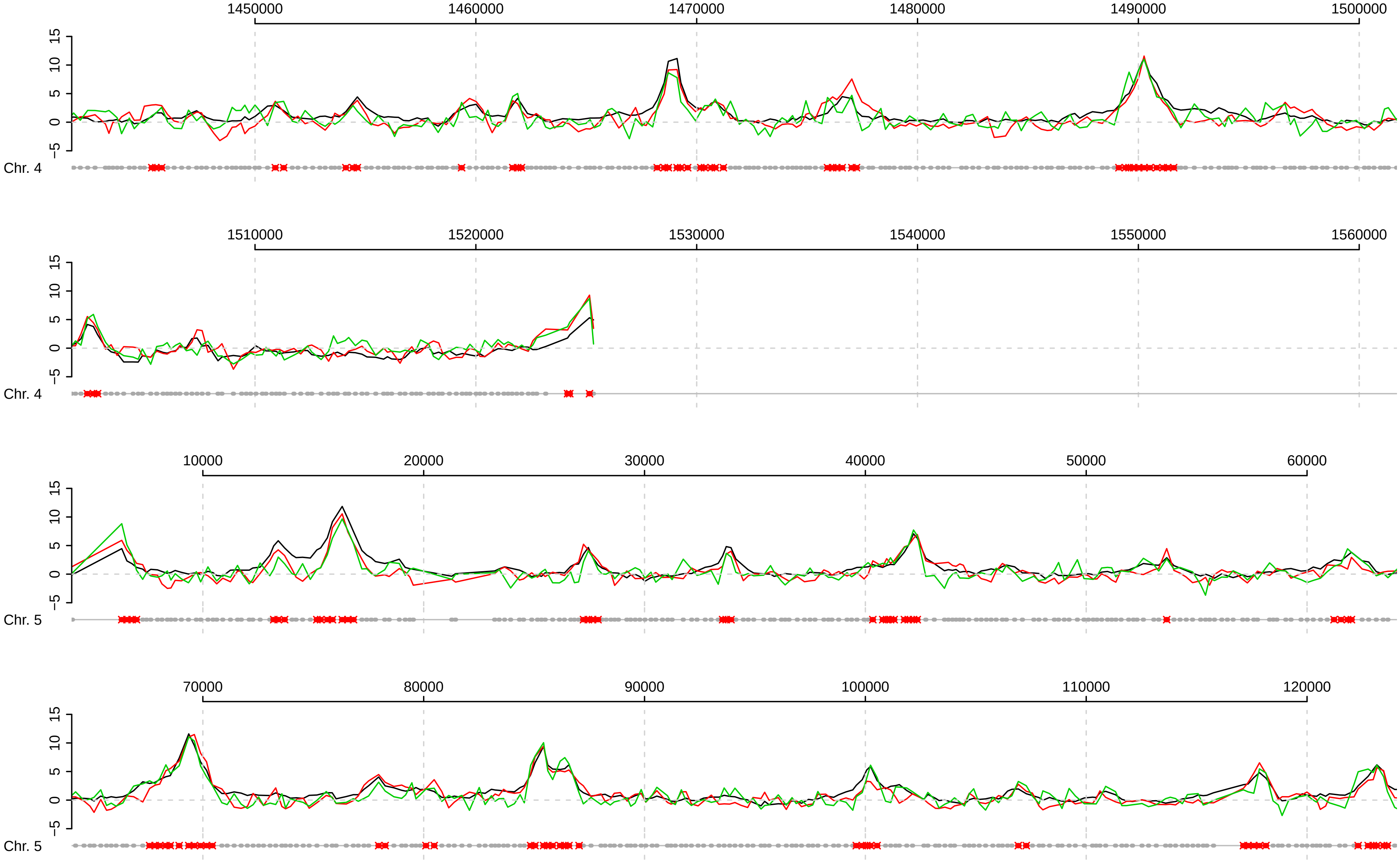
1450000
1460000
1470000
1480000
1490000
1500000
15
10
5
0
−5
Chr. 4
1510000
1520000
1530000
1540000
1550000
1560000
15
10
5
0
−5
Chr. 4
10000
20000
30000
40000
50000
60000
15
10
5
0
−5
Chr. 5
70000
80000
90000
100000
110000
120000
15
10
5
0
−5
Chr. 5

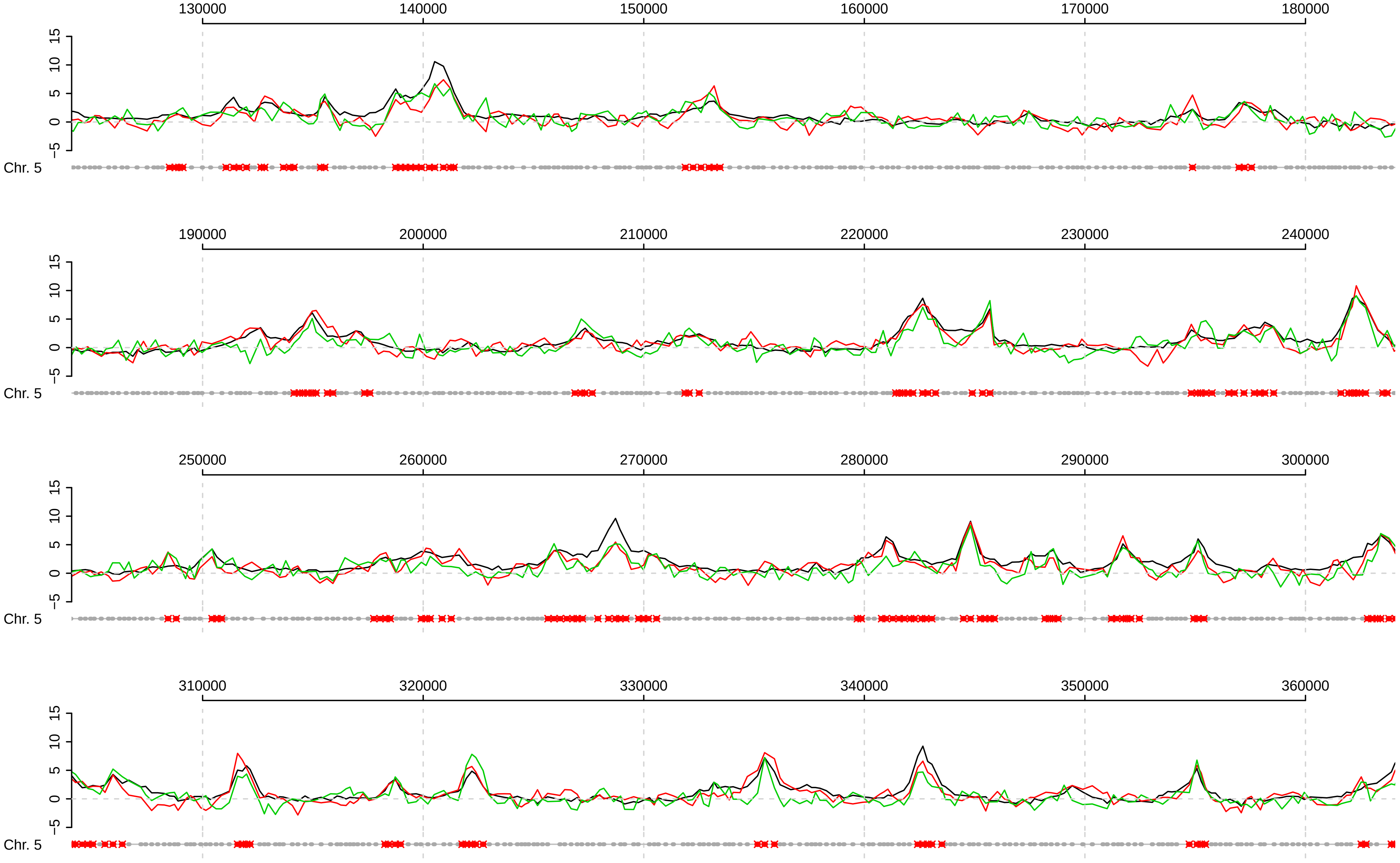

130000
140000
150000
160000
170000
180000
190000
200000
210000
220000
230000
240000
250000
260000
270000
280000
290000
300000
310000
320000
330000
340000
350000
360000
−5
0
5
10
15
Chr. 5

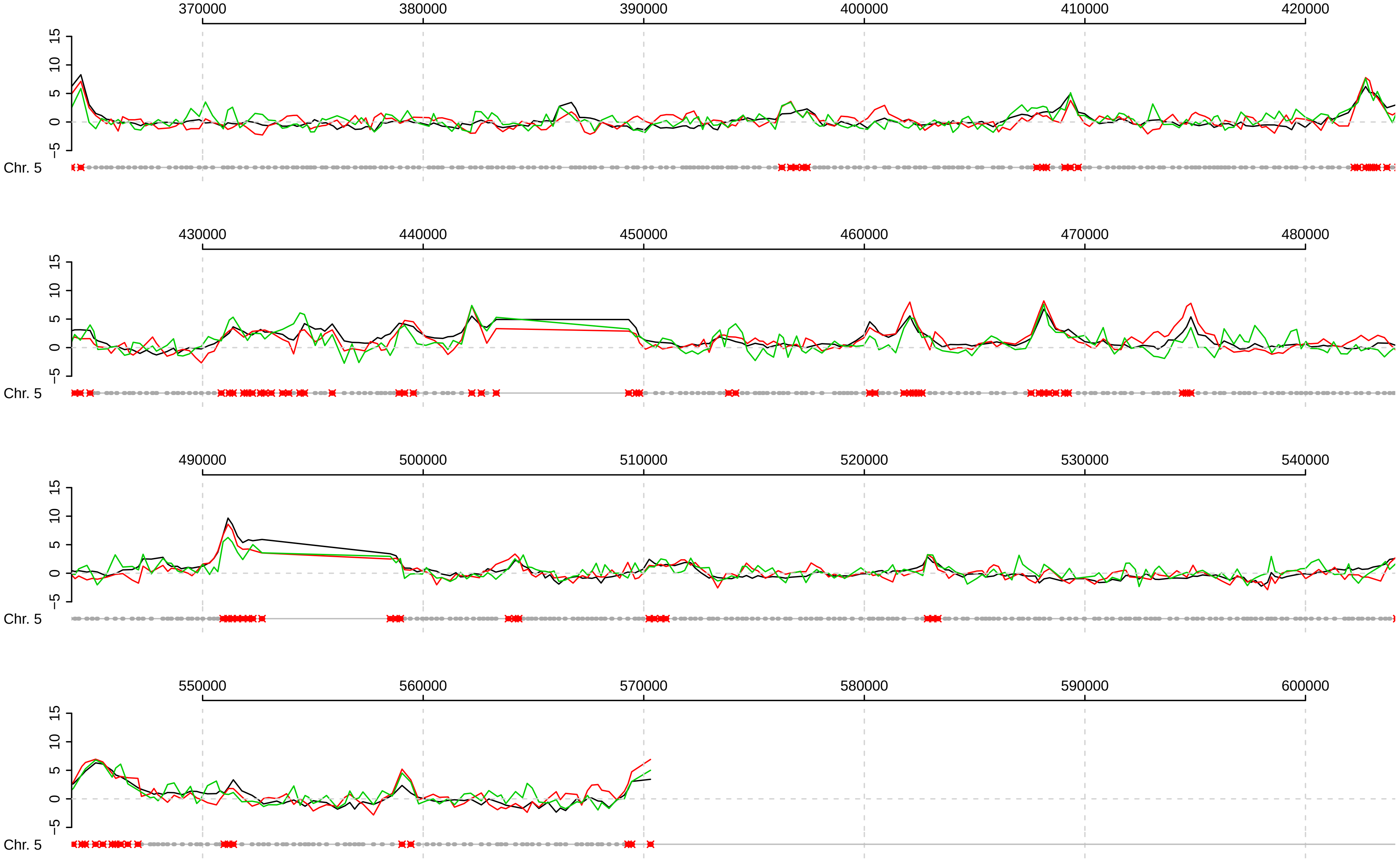
370000
380000
390000
400000
410000
420000
15
10
5
0
−5
Chr. 5
430000
440000
450000
460000
470000
480000
Chr. 5
490000
500000
510000
520000
530000
540000
Chr. 5
550000
560000
570000
580000
590000
600000
Chr. 5

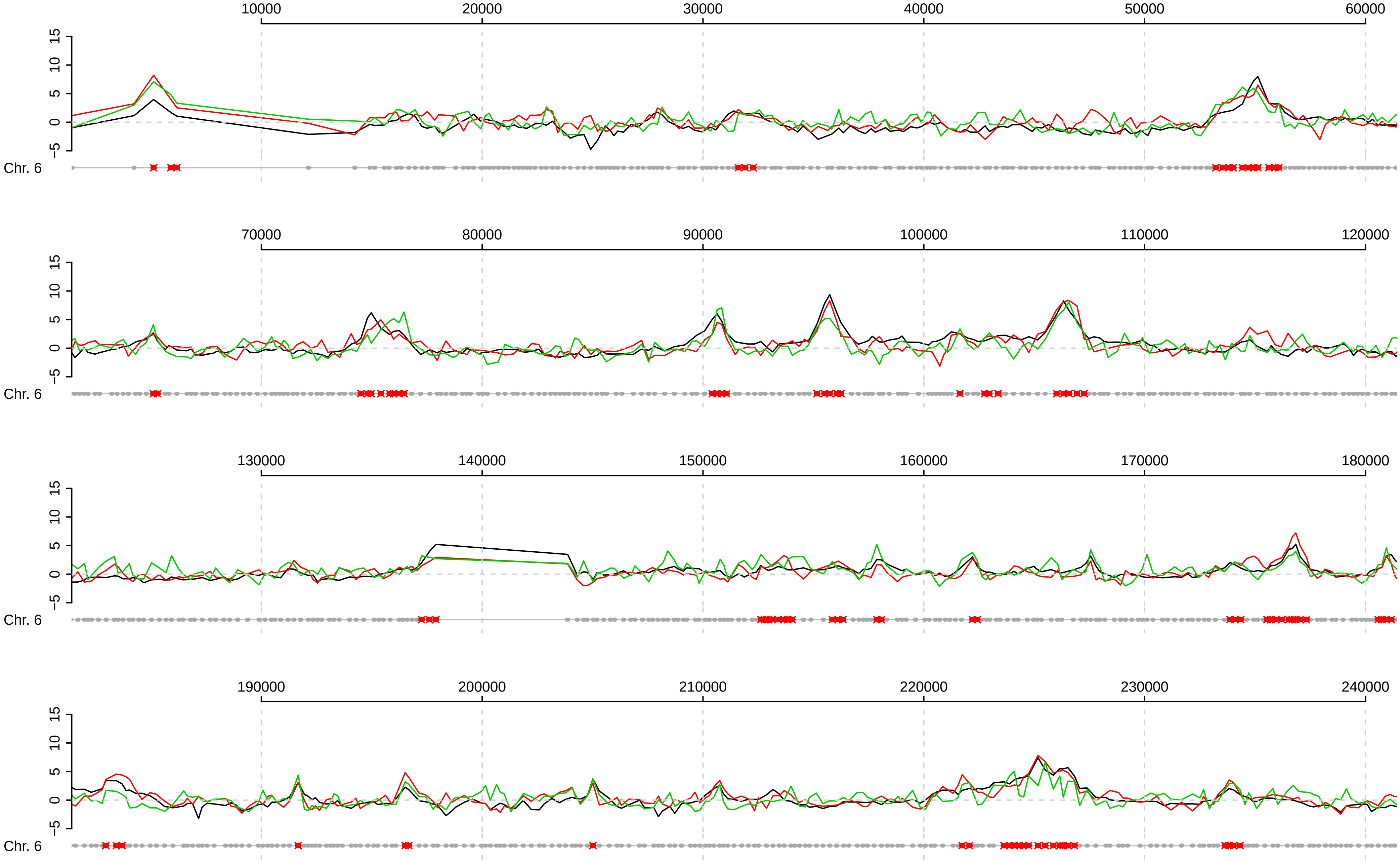
10000
20000
30000
40000
50000
60000
Chr. 6
70000
80000
90000
100000
110000
120000
Chr. 6
130000
140000
150000
160000
170000
180000
Chr. 6
190000
200000
210000
220000
230000
240000
Chr. 6

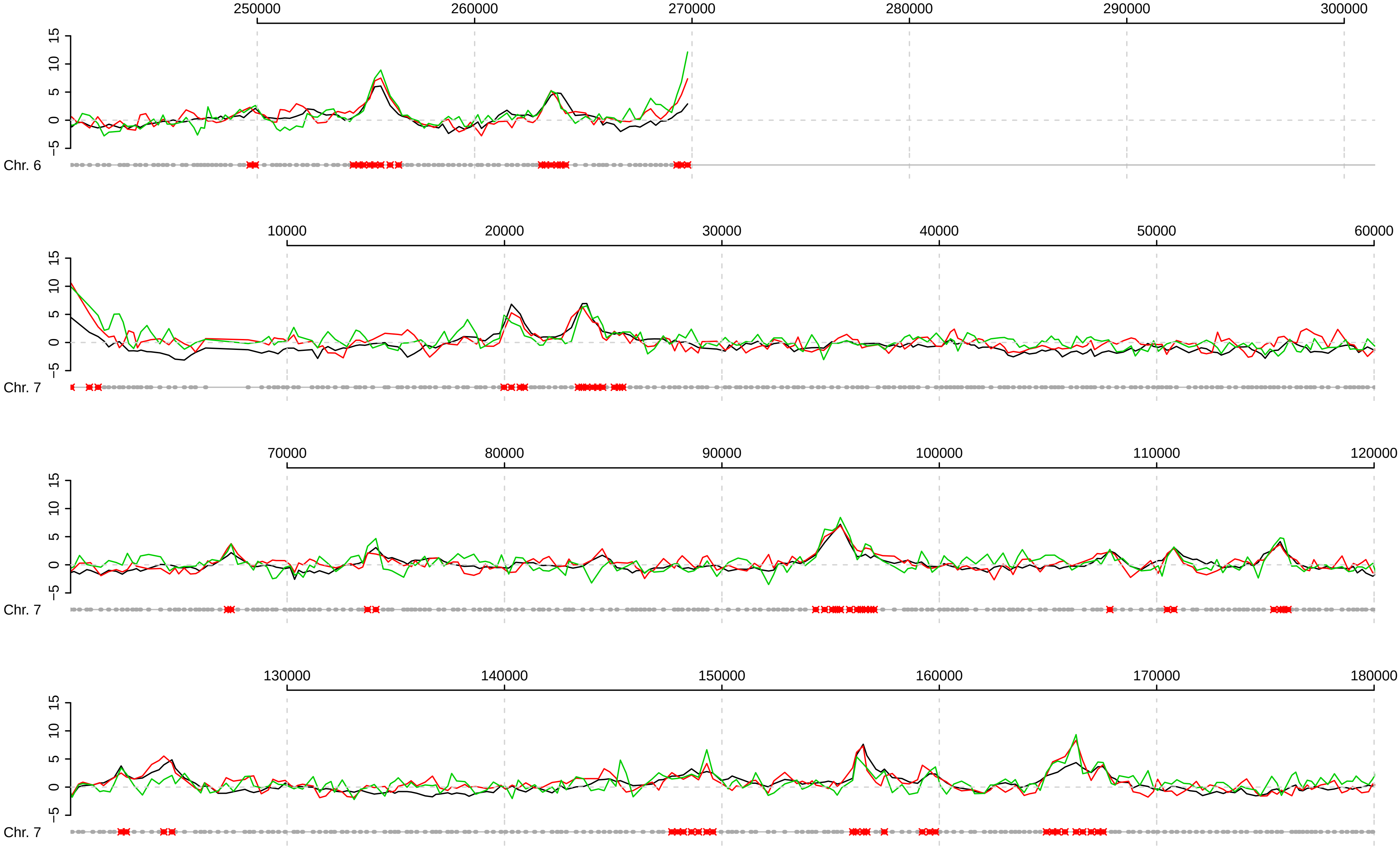
250000
260000
270000
280000
290000
300000
15
10
5
0
−5
Chr. 6
10000
20000
30000
40000
50000
60000
15
10
5
0
−5
Chr. 7
70000
80000
90000
100000
110000
120000
15
10
5
0
−5
Chr. 7
130000
140000
150000
160000
170000
180000
15
10
5
0
−5
Chr. 7

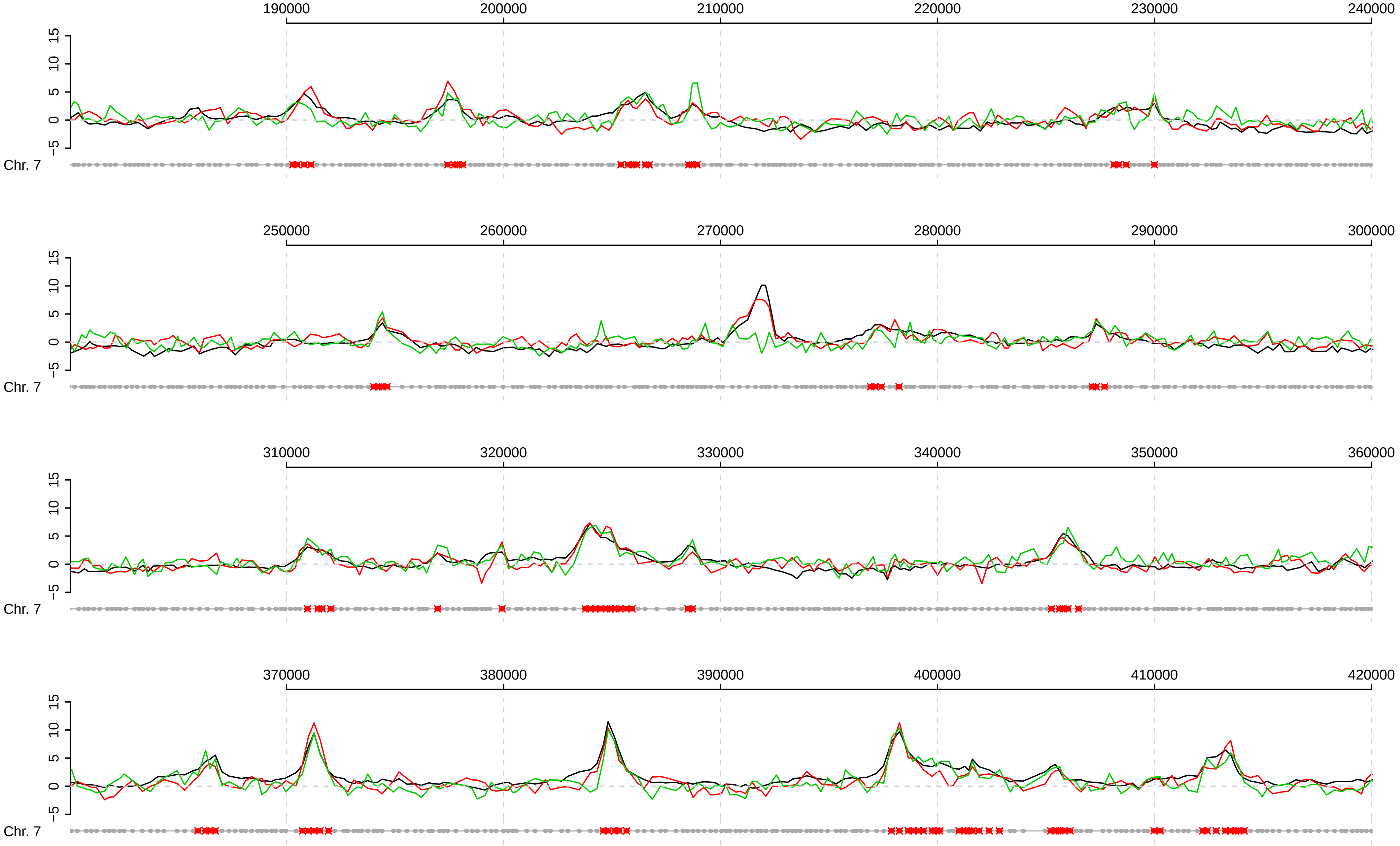

190000
200000
210000
220000
230000
240000
250000
260000
270000
280000
290000
300000
310000
320000
330000
340000
350000
360000
370000
380000
390000
400000
410000
420000
−5
0
5
10
15
Chr. 7

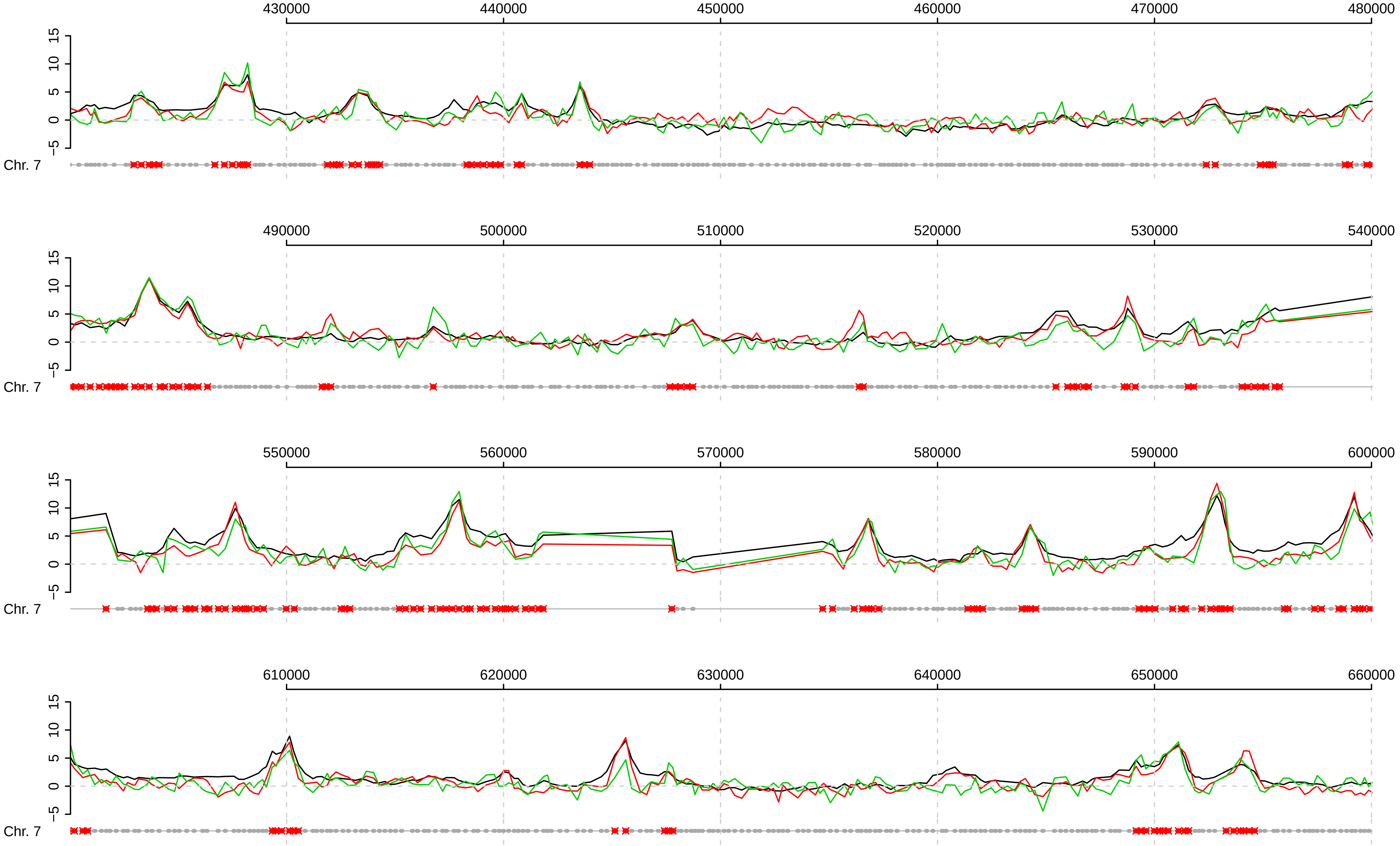

430000
440000
450000
460000
470000
480000
490000
500000
510000
520000
530000
540000
550000
560000
570000
580000
590000
600000
610000
620000
630000
640000
650000
660000
−5
0
5
10
15
Chr. 7

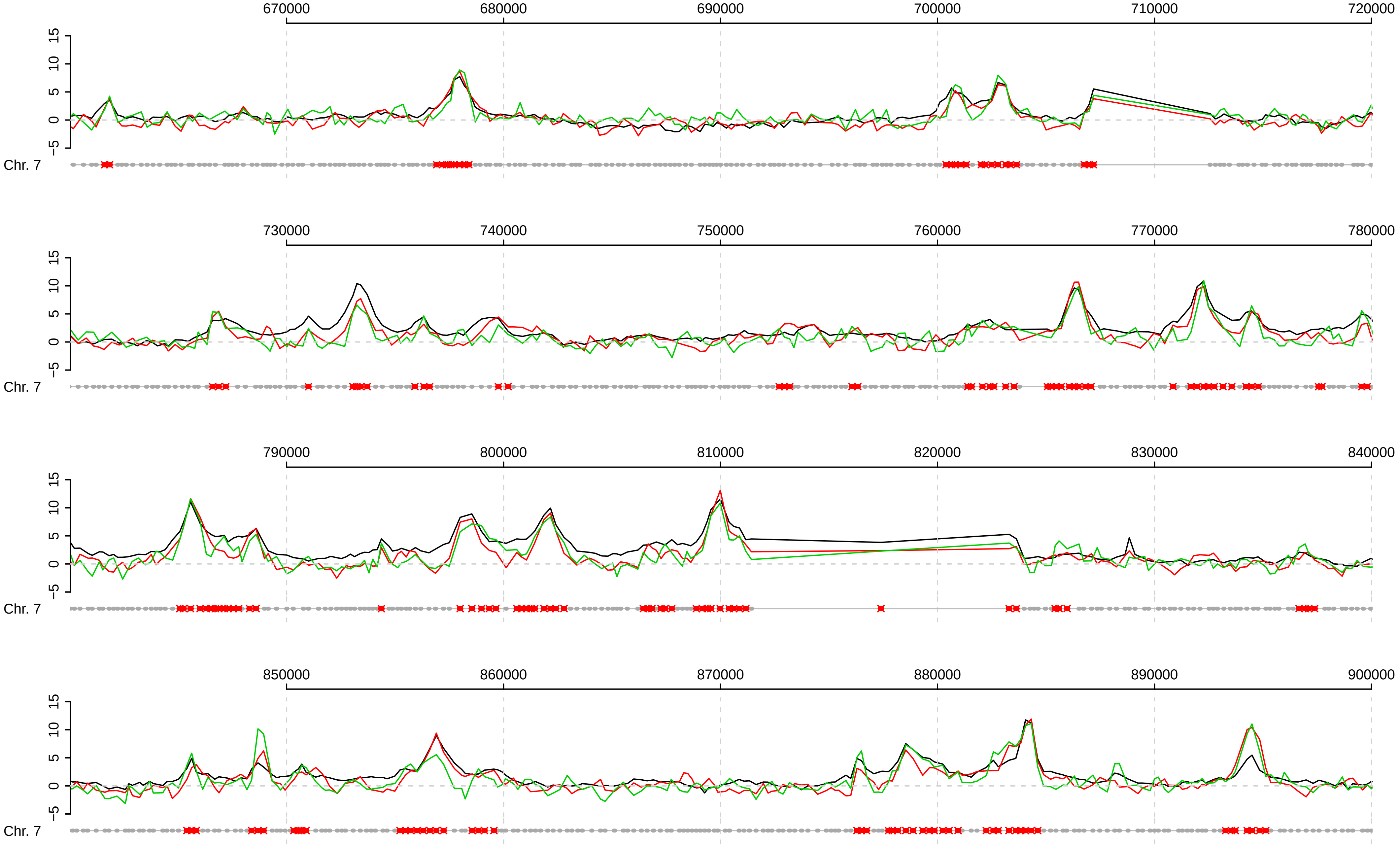
670000
680000
690000
700000
710000
720000
15
10
5
0
−5
Chr. 7
730000
740000
750000
760000
770000
780000
15
10
5
0
−5
Chr. 7
790000
800000
810000
820000
830000
840000
15
10
5
0
−5
Chr. 7
850000
860000
870000
880000
890000
900000
15
10
5
0
−5
Chr. 7

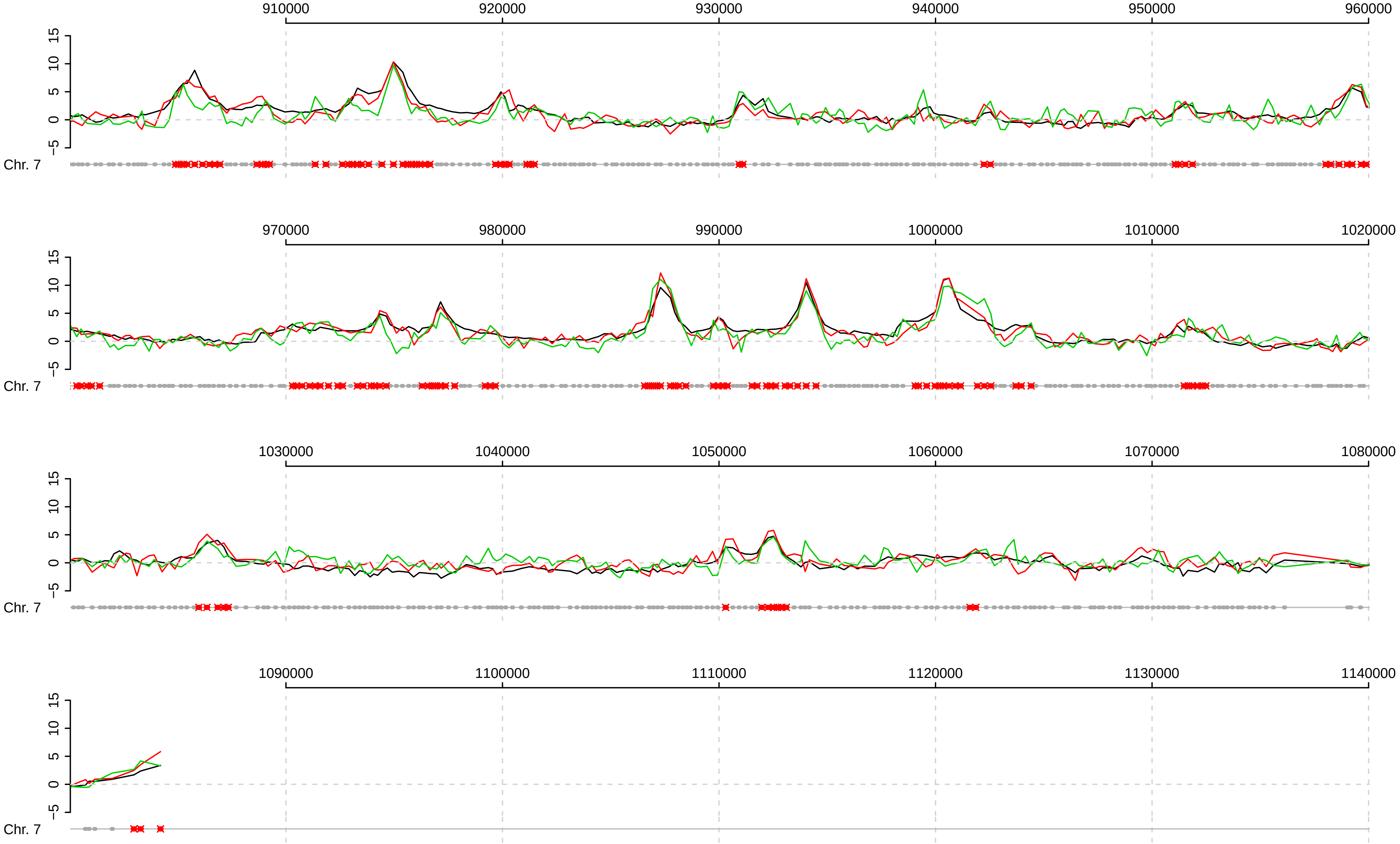

910000
920000
930000
940000
950000
960000
970000
980000
990000
1000000
1010000
1020000
1030000
1040000
1050000
1060000
1070000
1080000
1090000
1100000
1110000
1120000
1130000
1140000
−5
0
5
10
15
Chr. 7

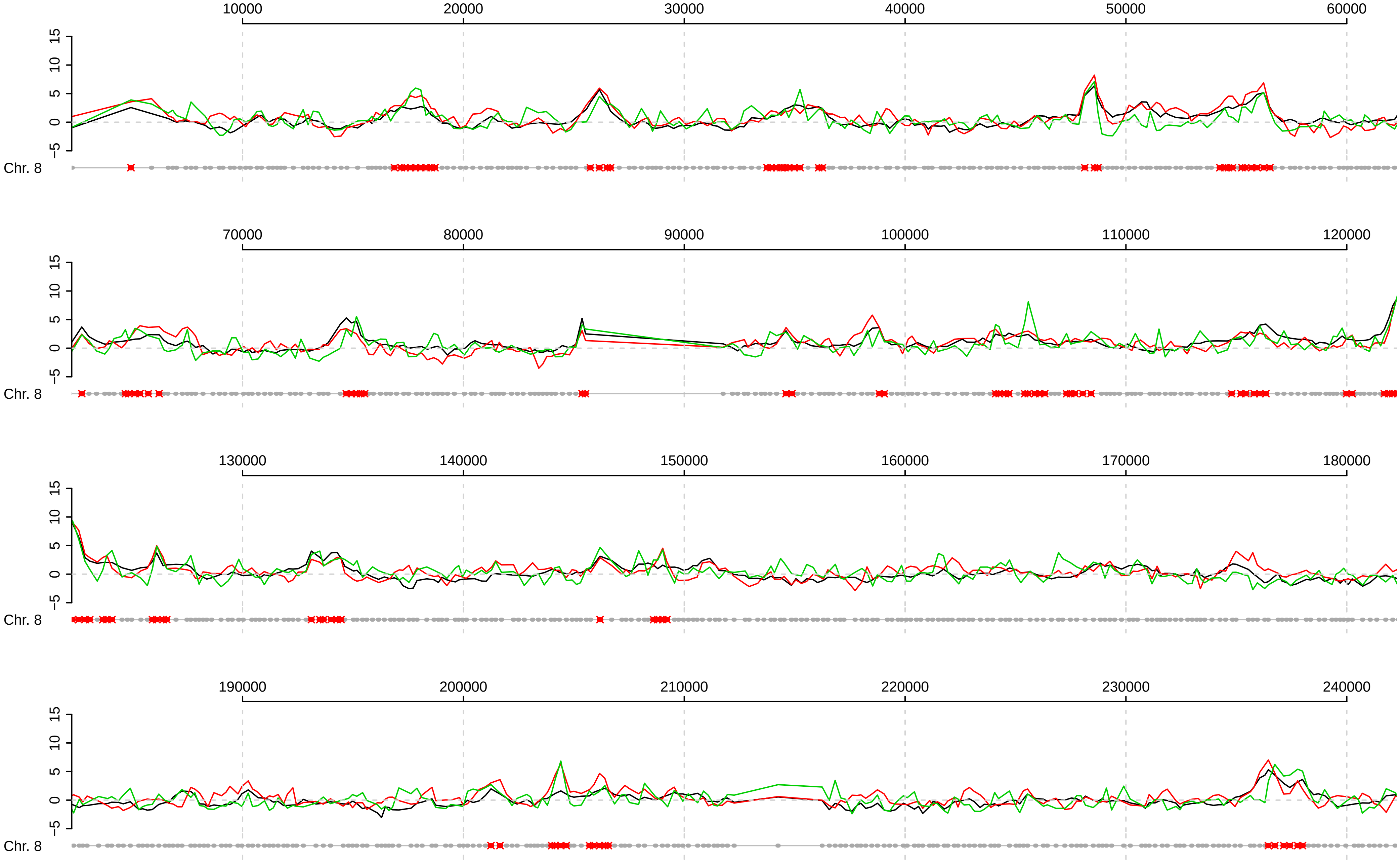

10000
20000
30000
40000
50000
60000
70000
80000
90000
100000
110000
120000
130000
140000
150000
160000
170000
180000
190000
200000
210000
220000
230000
240000
−5
0
5
10
15
Chr. 8

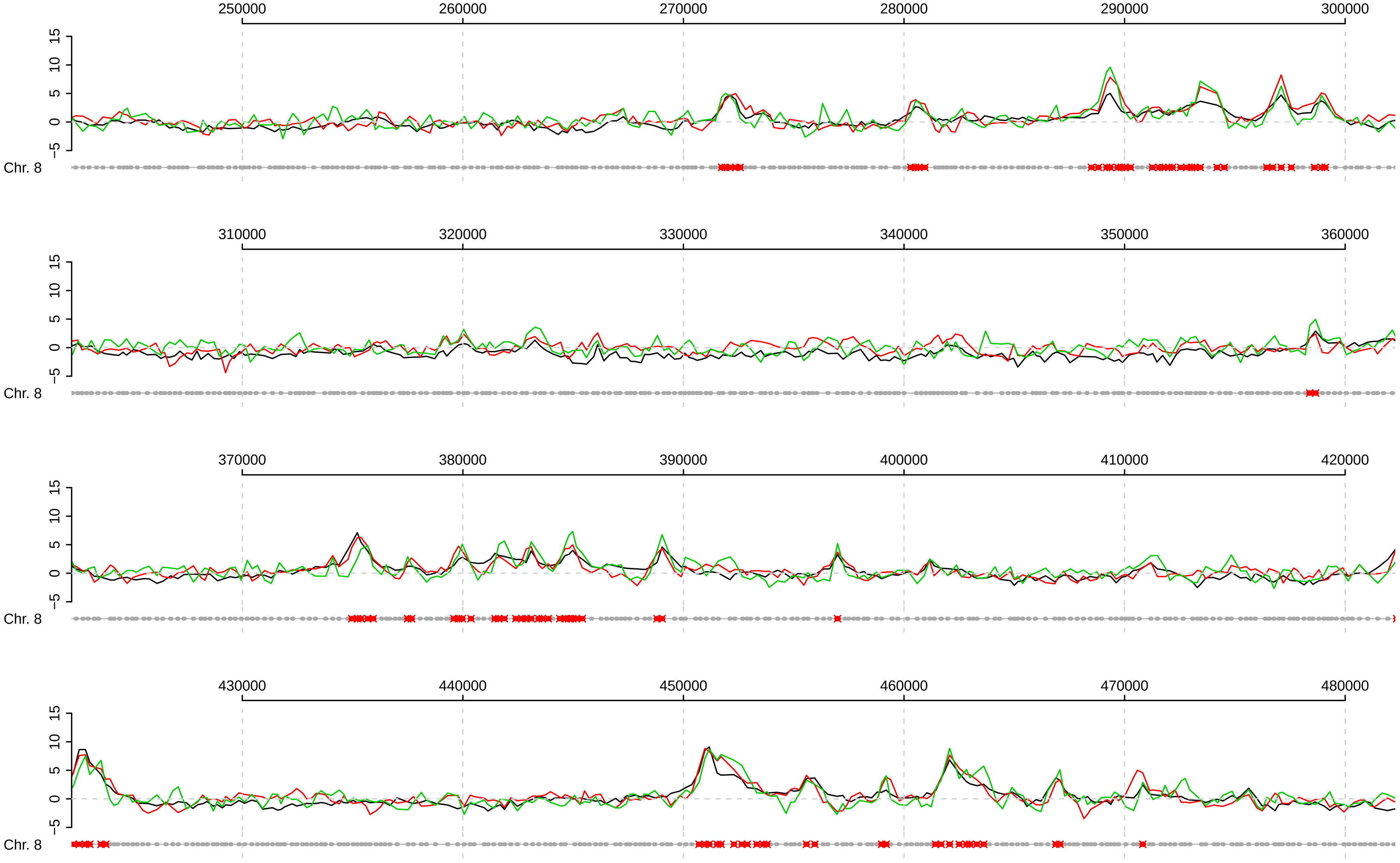

250000
260000
270000
280000
290000
300000
310000
320000
330000
340000
350000
360000
370000
380000
390000
400000
410000
420000
430000
440000
450000
460000
470000
480000
15
10
5
0
−5
Chr. 8

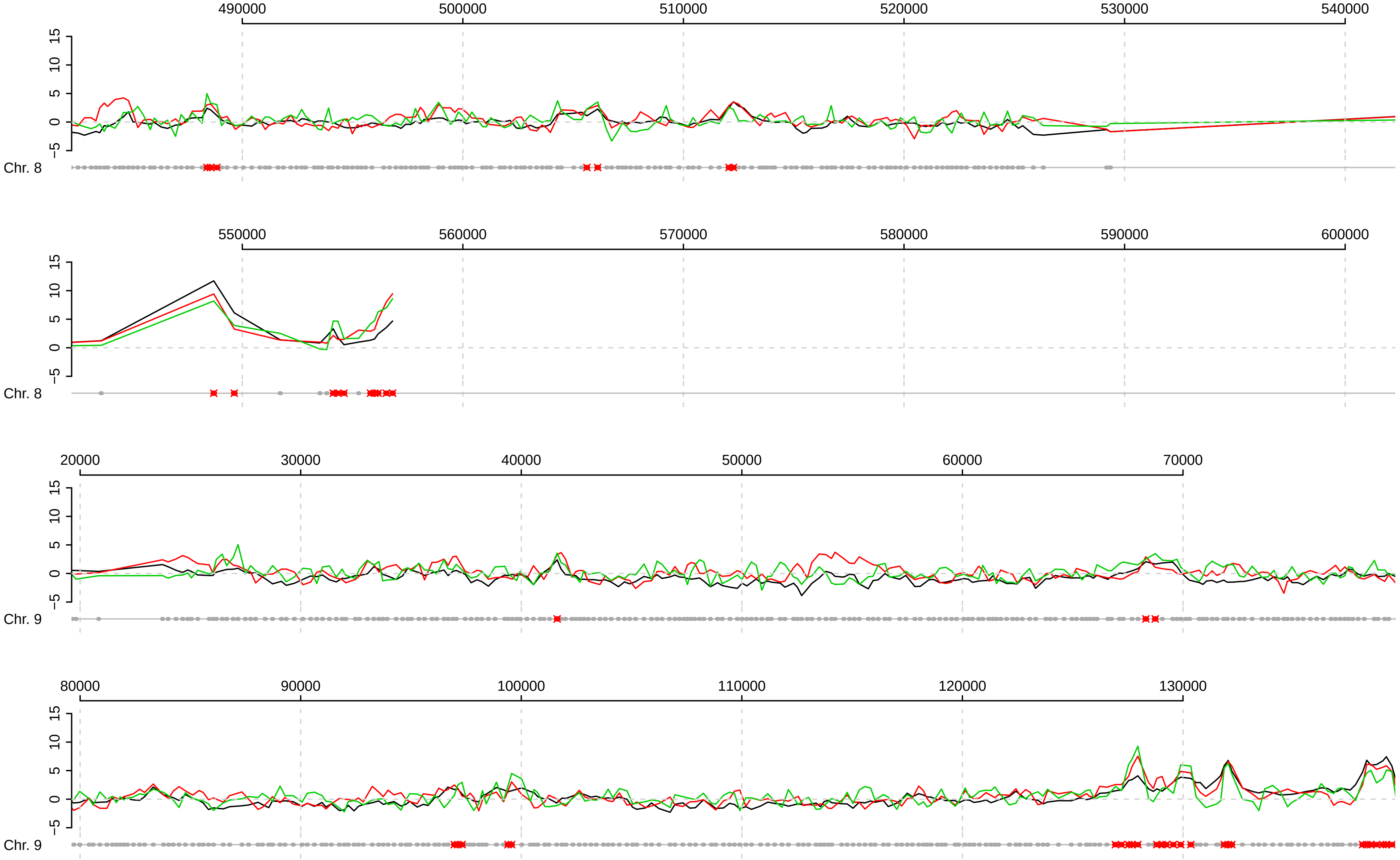
490000
500000
510000
520000
530000
540000
15
10
5
0
−5
Chr. 8
550000
560000
570000
580000
590000
600000
15
10
5
0
−5
Chr. 8
20000
30000
40000
50000
60000
70000
15
10
5
0
−5
Chr. 9
80000
90000
100000
110000
120000
130000
15
10
5
0
−5
Chr. 9

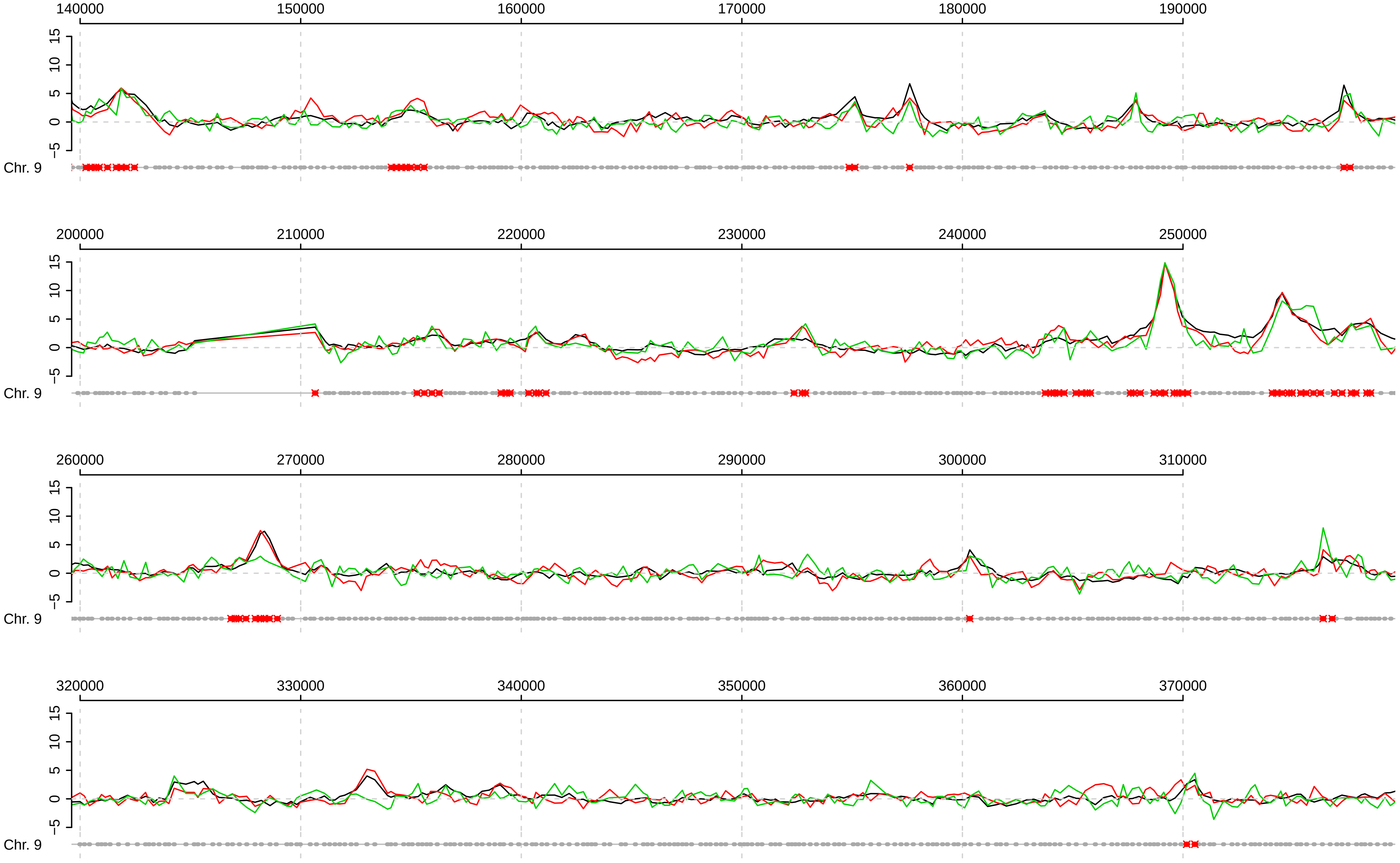
140000
150000
160000
170000
180000
190000
Chr. 9
200000
210000
220000
230000
240000
250000
Chr. 9
260000
270000
280000
290000
300000
310000
Chr. 9
320000
330000
340000
350000
360000
370000
Chr. 9
−5
0
5
10
15

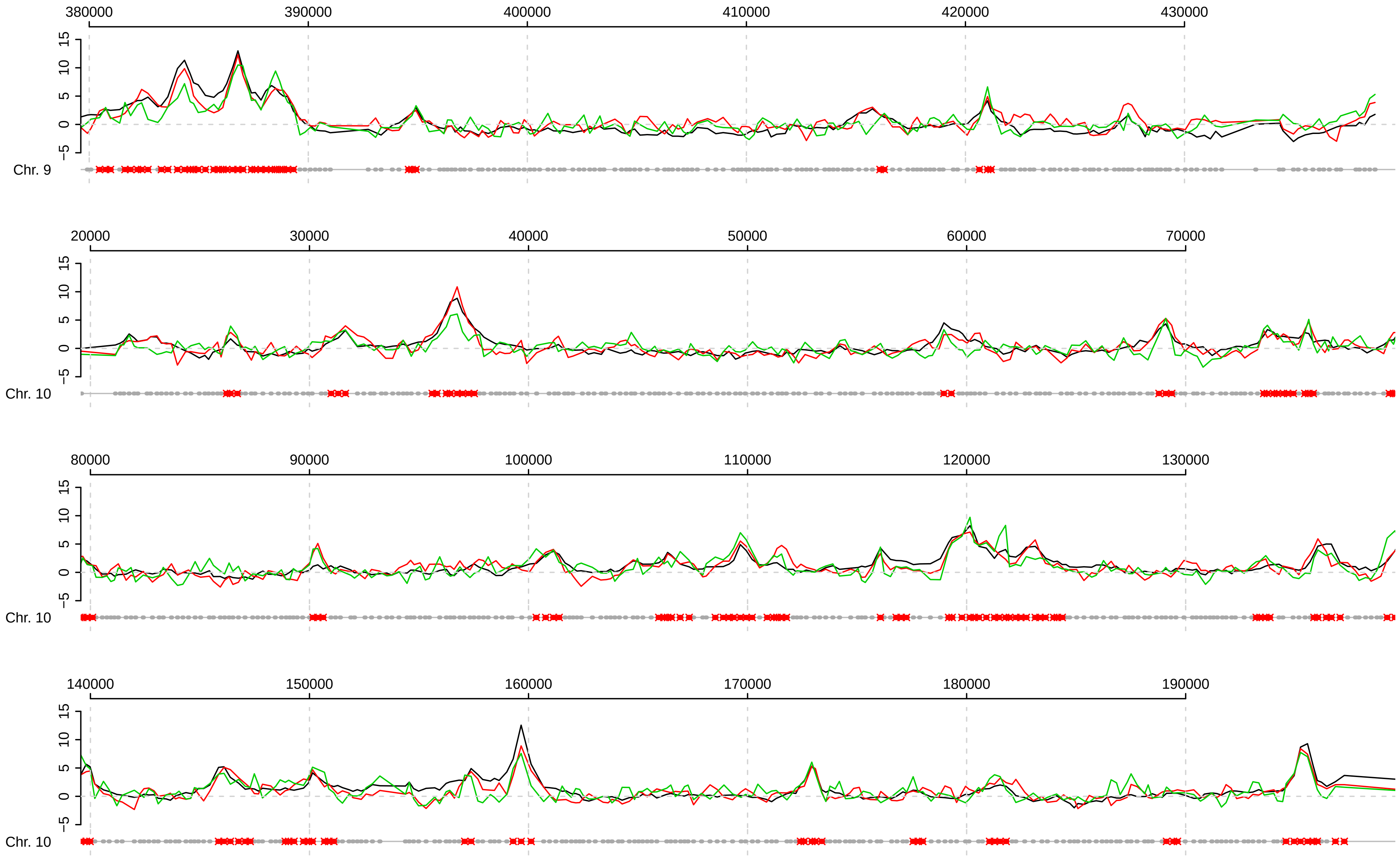
380000
390000
400000
410000
420000
430000
Chr. 9
20000
30000
40000
50000
60000
70000
Chr. 10
80000
90000
100000
110000
120000
130000
Chr. 10
140000
150000
160000
170000
180000
190000
Chr. 10
−5
0
5
10
15

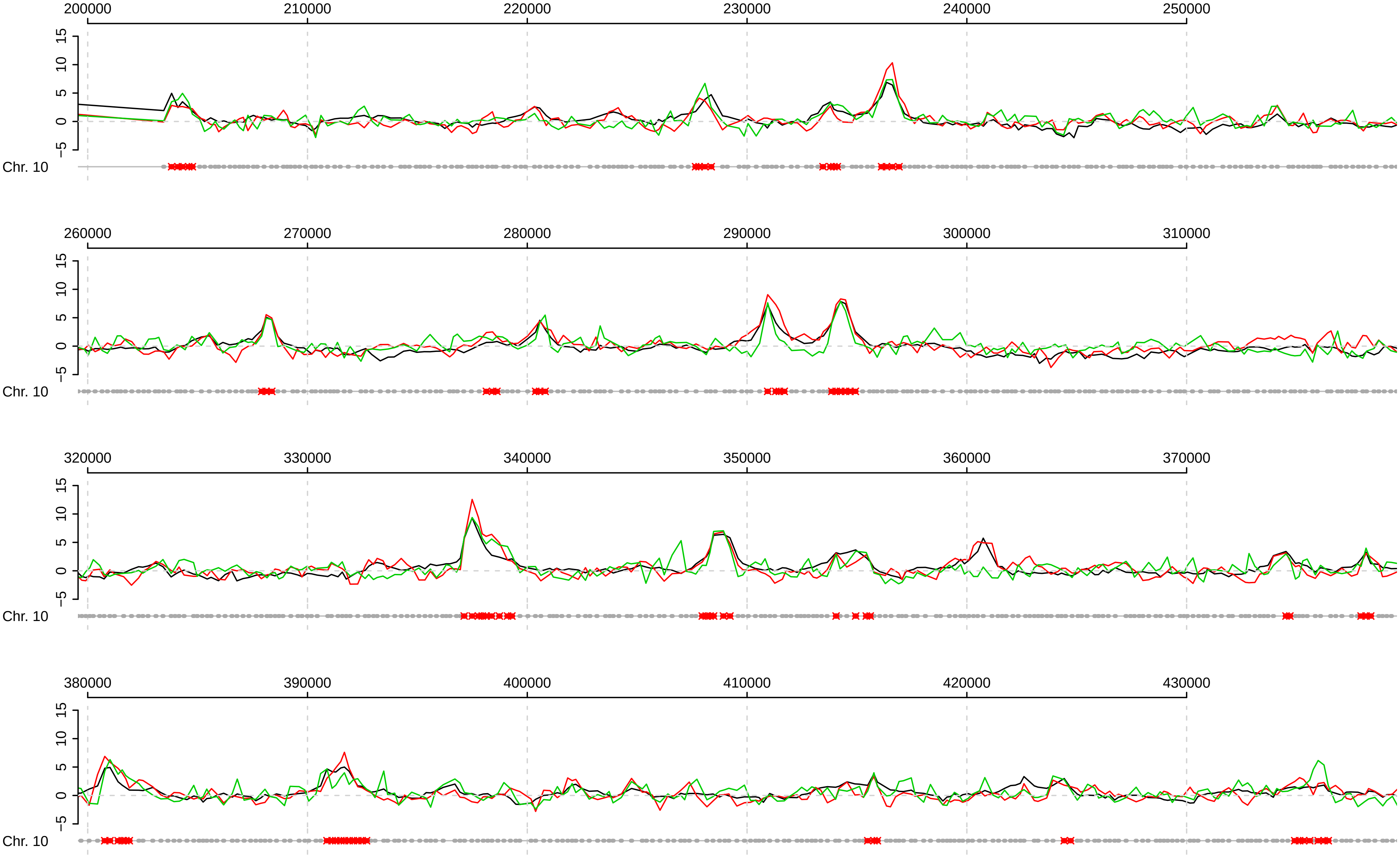
200000
210000
220000
230000
240000
250000
Chr. 10
260000
270000
280000
290000
300000
310000
Chr. 10
320000
330000
340000
350000
360000
370000
Chr. 10
380000
390000
400000
410000
420000
430000
Chr. 10
−5
0
5
10
15

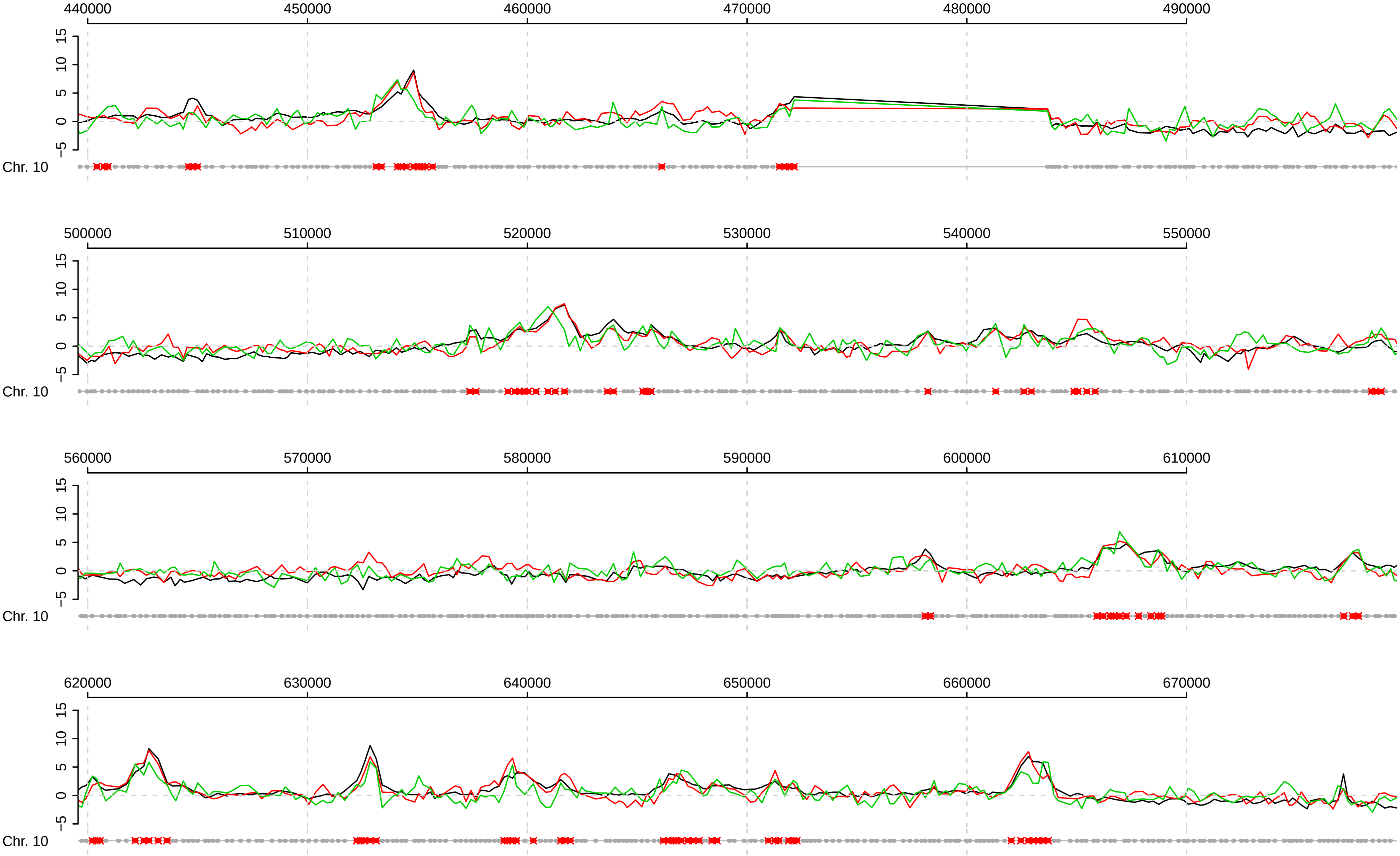
440000
450000
460000
470000
480000
490000
Chr. 10
500000
510000
520000
530000
540000
550000
Chr. 10
560000
570000
580000
590000
600000
610000
Chr. 10
620000
630000
640000
650000
660000
670000
Chr. 10
15
10
5
0
−5

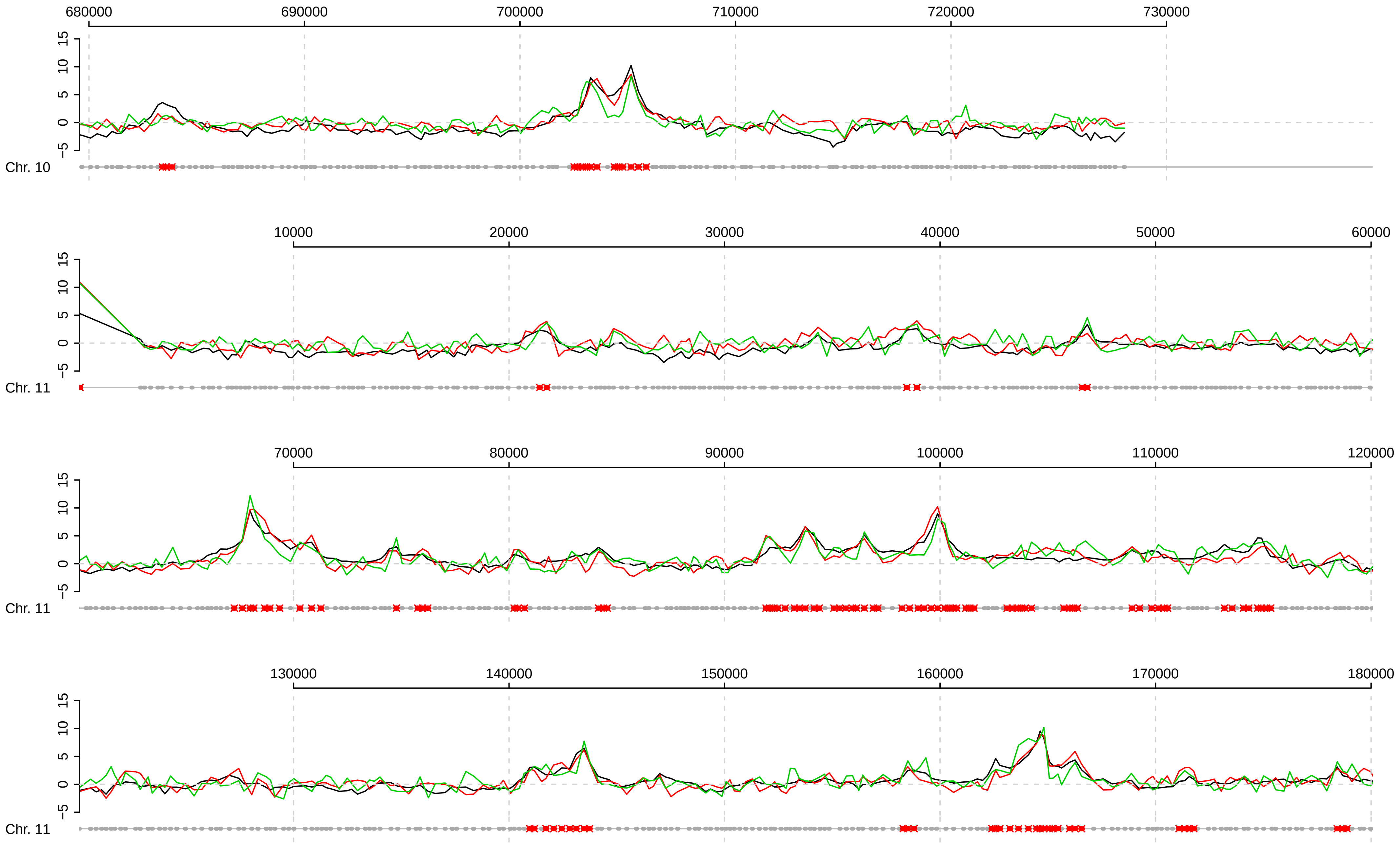

680000
690000
700000
710000
720000
730000
Chr. 10
10000
20000
30000
40000
50000
60000
Chr. 11
70000
80000
90000
100000
110000
120000
Chr. 11
130000
140000
150000
160000
170000
180000
Chr. 11
−5
0
5
10
15

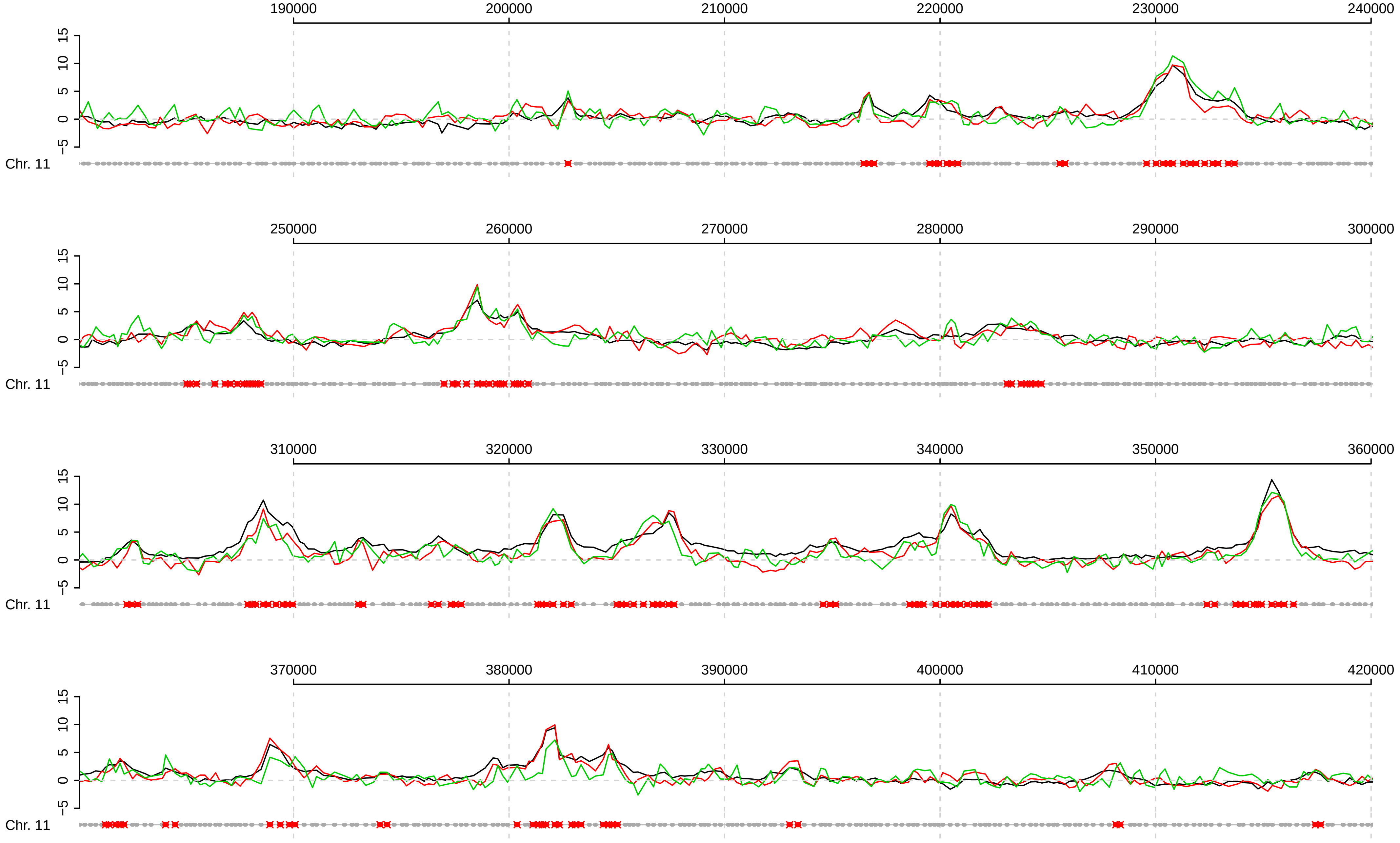

190000
200000
210000
220000
230000
240000
Chr. 11
250000
260000
270000
280000
290000
300000
Chr. 11
310000
320000
330000
340000
350000
360000
Chr. 11
370000
380000
390000
400000
410000
420000
Chr. 11
15
10
5
0
−5

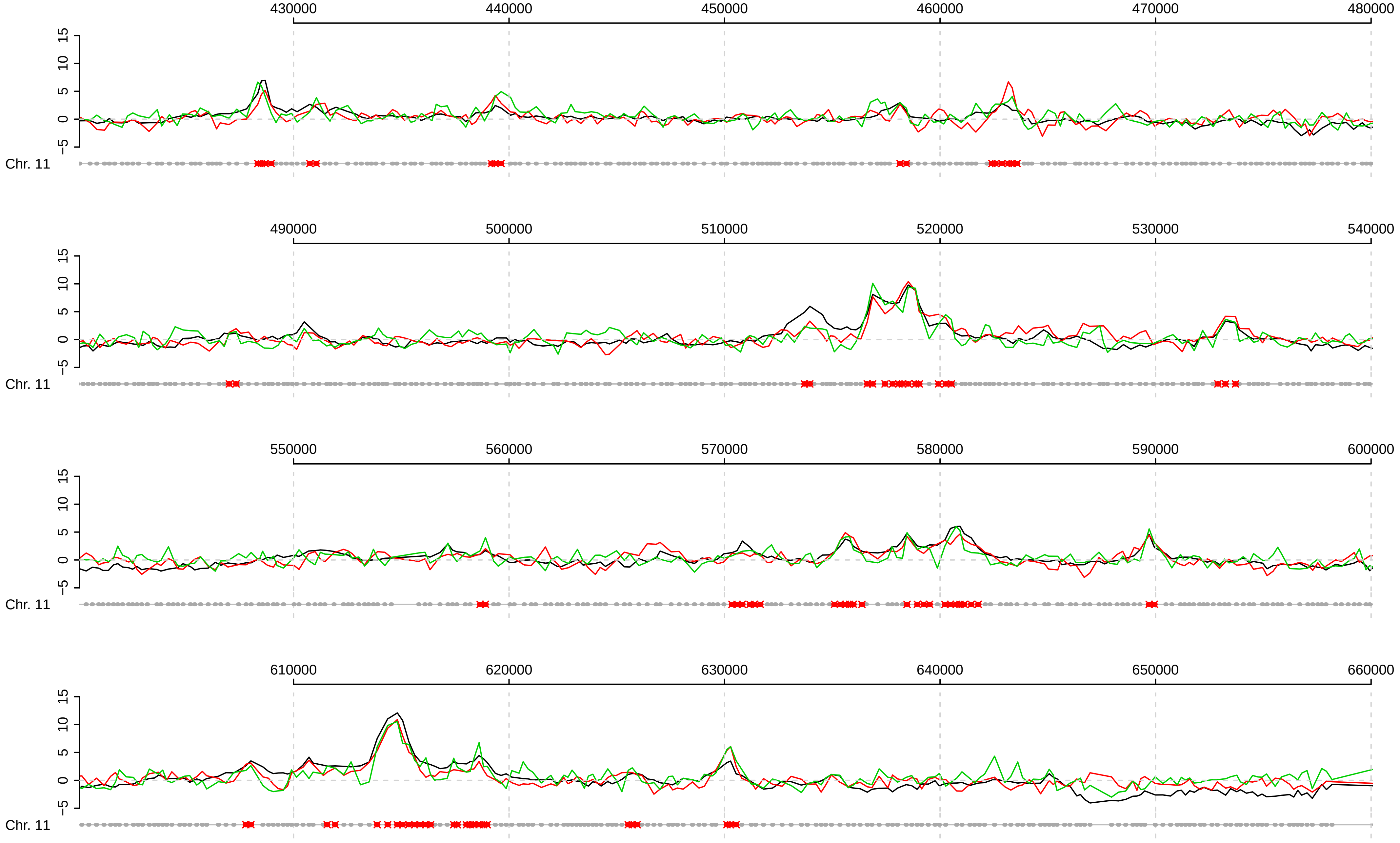

430000
440000
450000
460000
470000
480000
15
10
5
0
−5
Chr. 11
490000
500000
510000
520000
530000
540000
Chr. 11
550000
560000
570000
580000
590000
600000
Chr. 11
610000
620000
630000
640000
650000
660000
Chr. 11

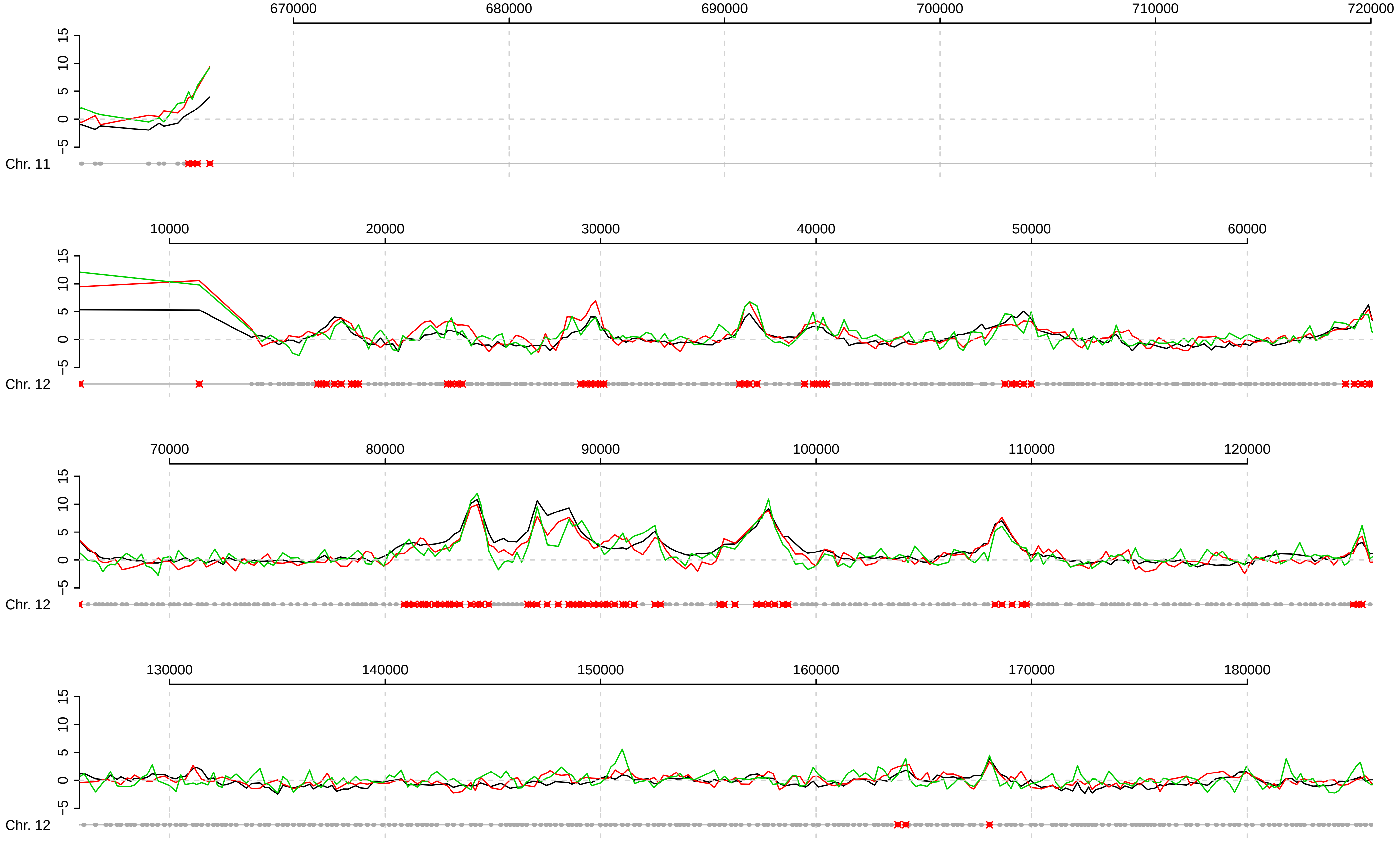
670000
680000
690000
700000
710000
720000
−5
0
5
10
15
Chr. 11
10000
20000
30000
40000
50000
60000
Chr. 12
70000
80000
90000
100000
110000
120000
Chr. 12
130000
140000
150000
160000
170000
180000
Chr. 12

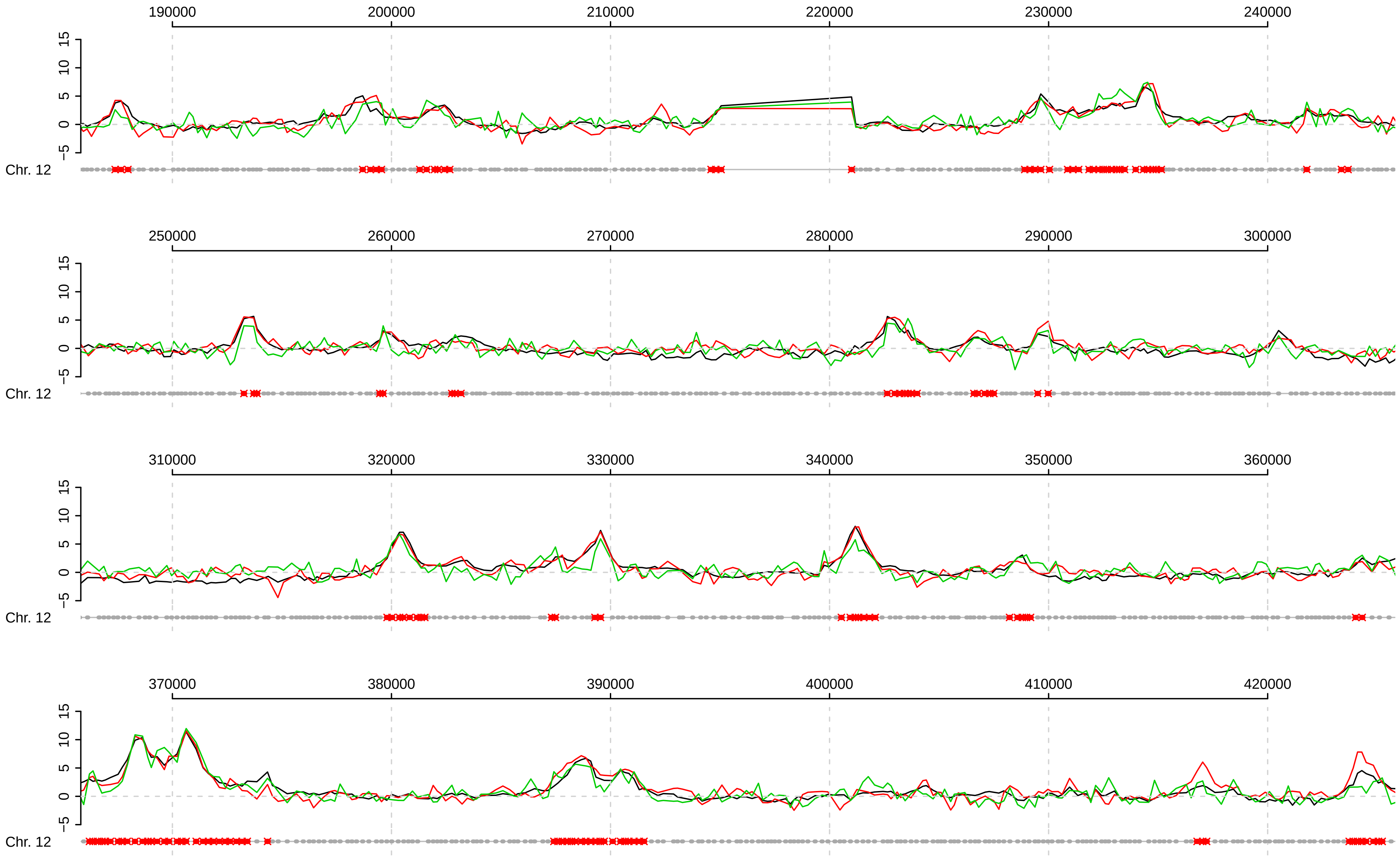
190000
200000
210000
220000
230000
240000
15
10
5
0
−5
Chr. 12
250000
260000
270000
280000
290000
300000
Chr. 12
310000
320000
330000
340000
350000
360000
Chr. 12
370000
380000
390000
400000
410000
420000
Chr. 12

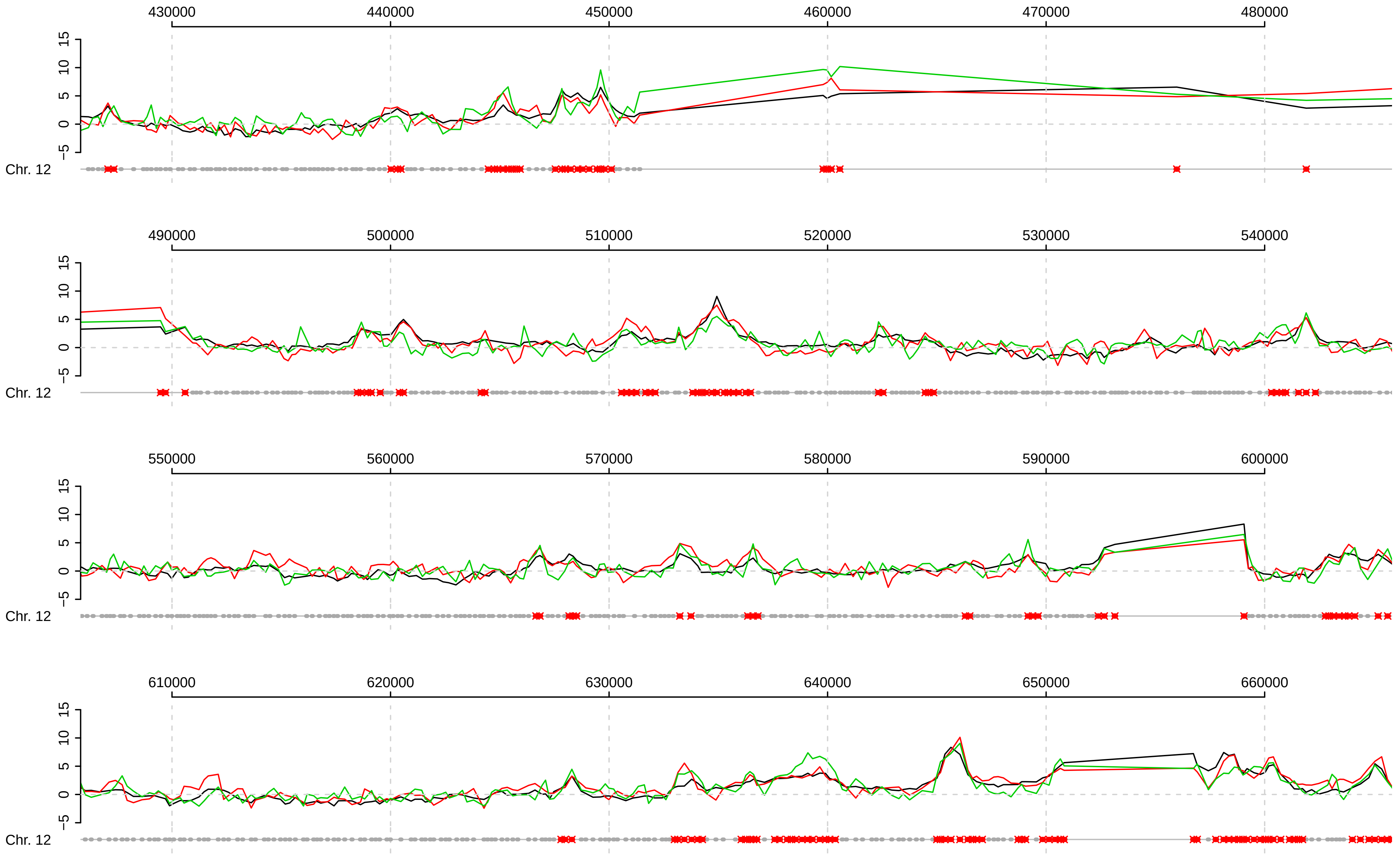
430000
440000
450000
460000
470000
480000
490000
500000
510000
520000
530000
540000
550000
560000
570000
580000
590000
600000
610000
620000
630000
640000
650000
660000
15
10
5
0
−5
Chr. 12

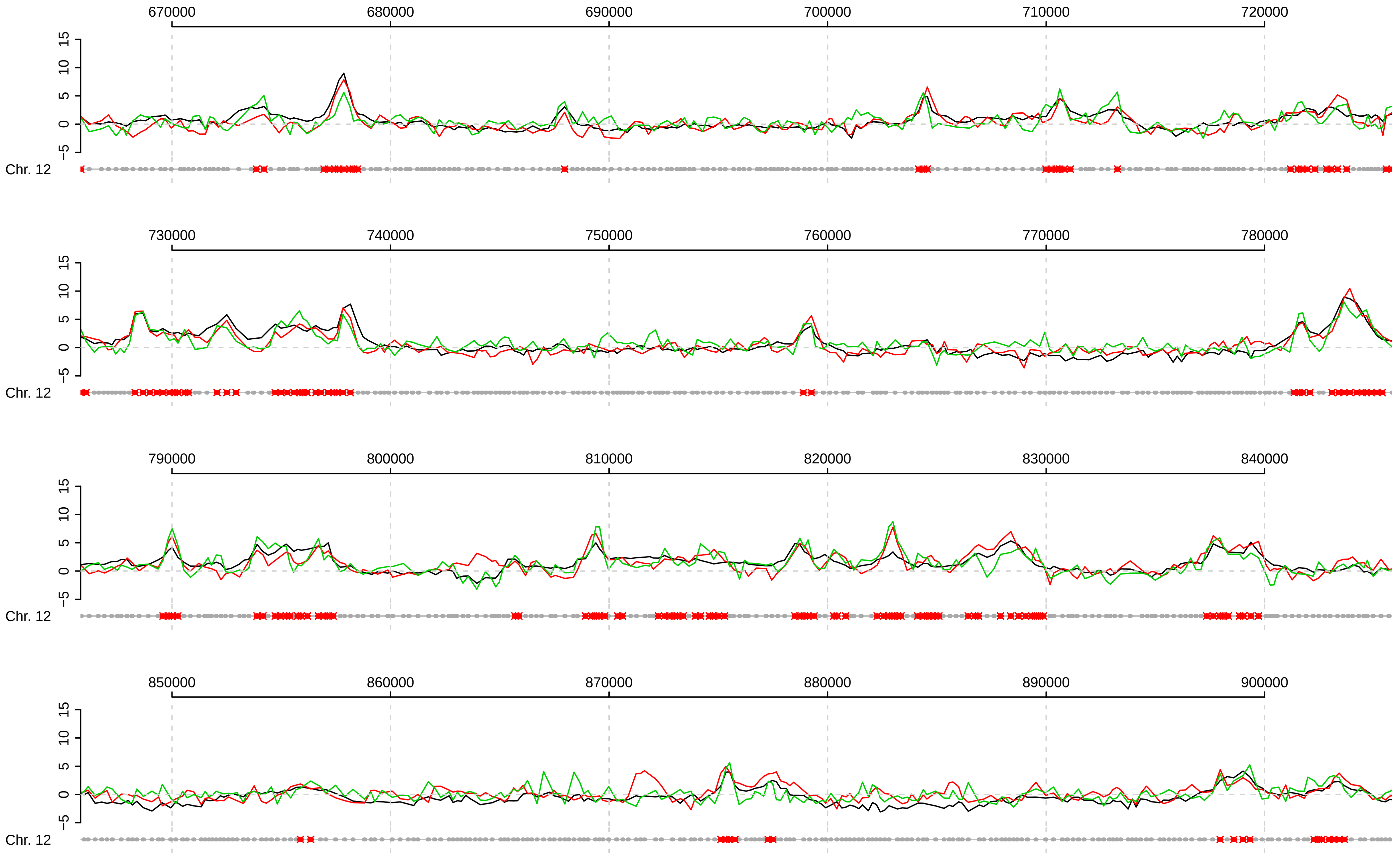

670000
680000
690000
700000
710000
720000
15
10
5
0
−5
Chr. 12
730000
740000
750000
760000
770000
780000
Chr. 12
790000
800000
810000
820000
830000
840000
Chr. 12
850000
860000
870000
880000
890000
900000
Chr. 12

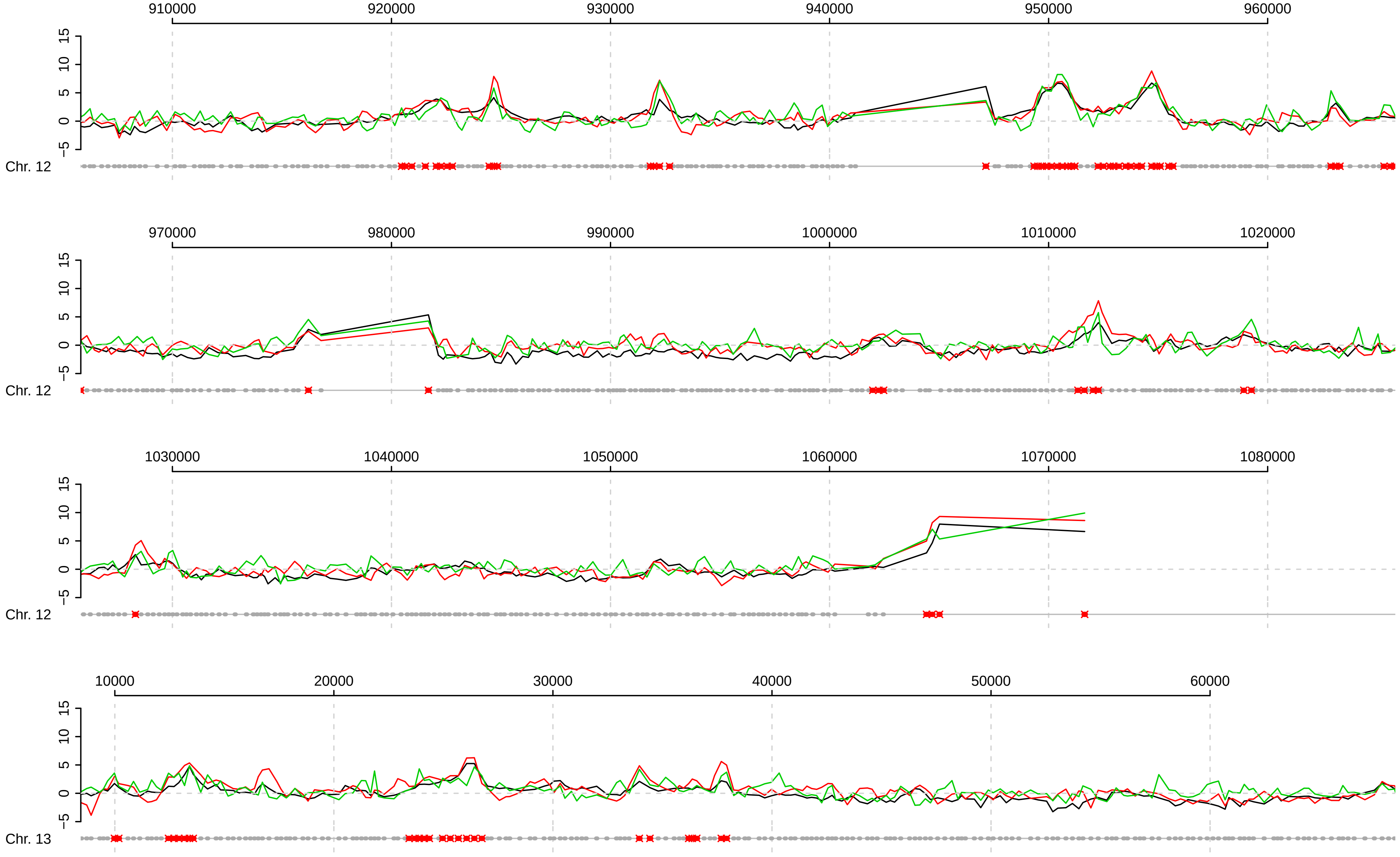

910000
920000
930000
940000
950000
960000
15
10
5
0
−5
Chr. 12
970000
980000
990000
1000000
1010000
1020000
15
10
5
0
−5
Chr. 12
1030000
1040000
1050000
1060000
1070000
1080000
15
10
5
0
−5
Chr. 12
10000
20000
30000
40000
50000
60000
15
10
5
0
−5
Chr. 13

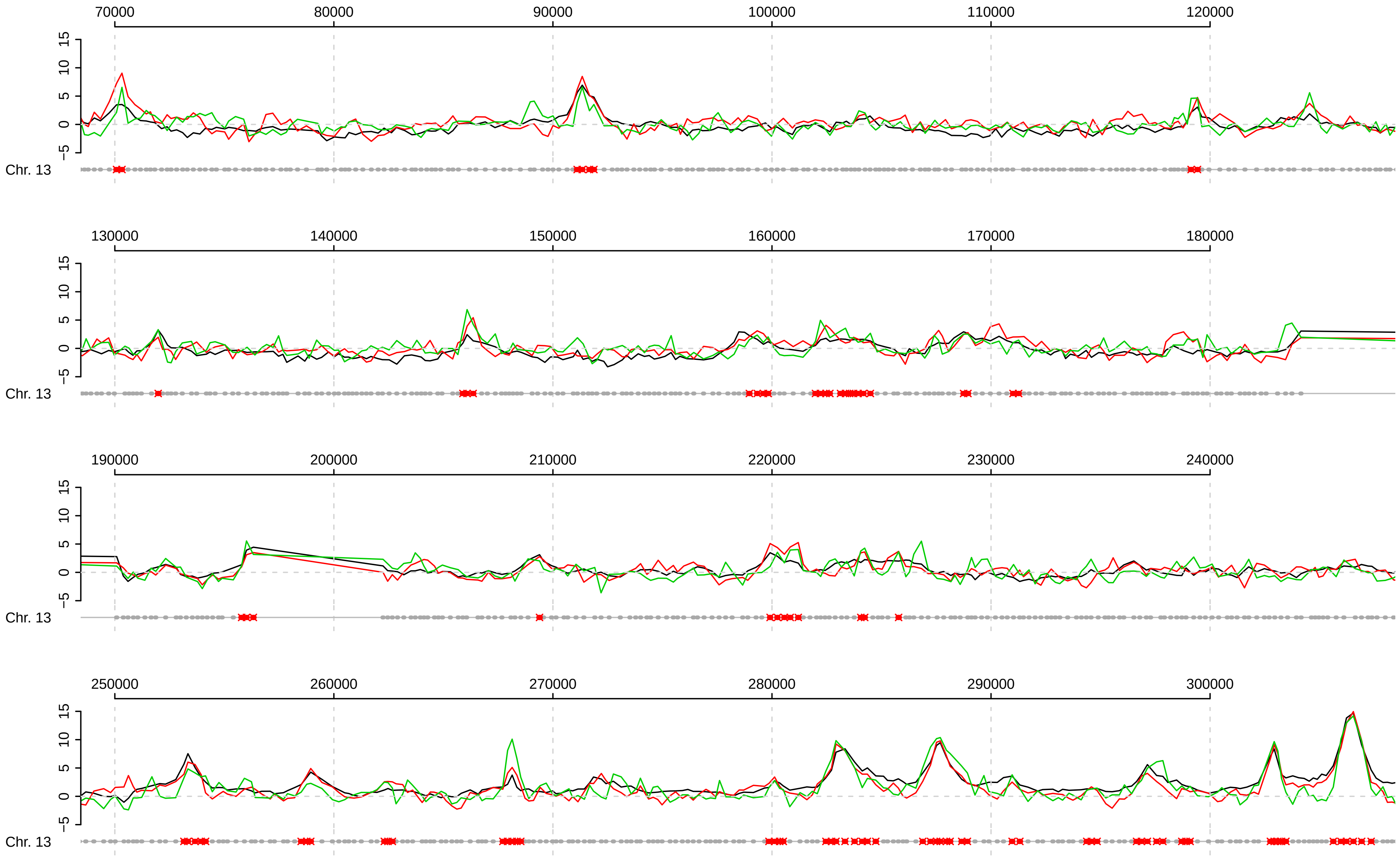
70000
80000
90000
100000
110000
120000
Chr. 13
130000
140000
150000
160000
170000
180000
Chr. 13
190000
200000
210000
220000
230000
240000
Chr. 13
250000
260000
270000
280000
290000
300000
Chr. 13
15
10
5
0
−5

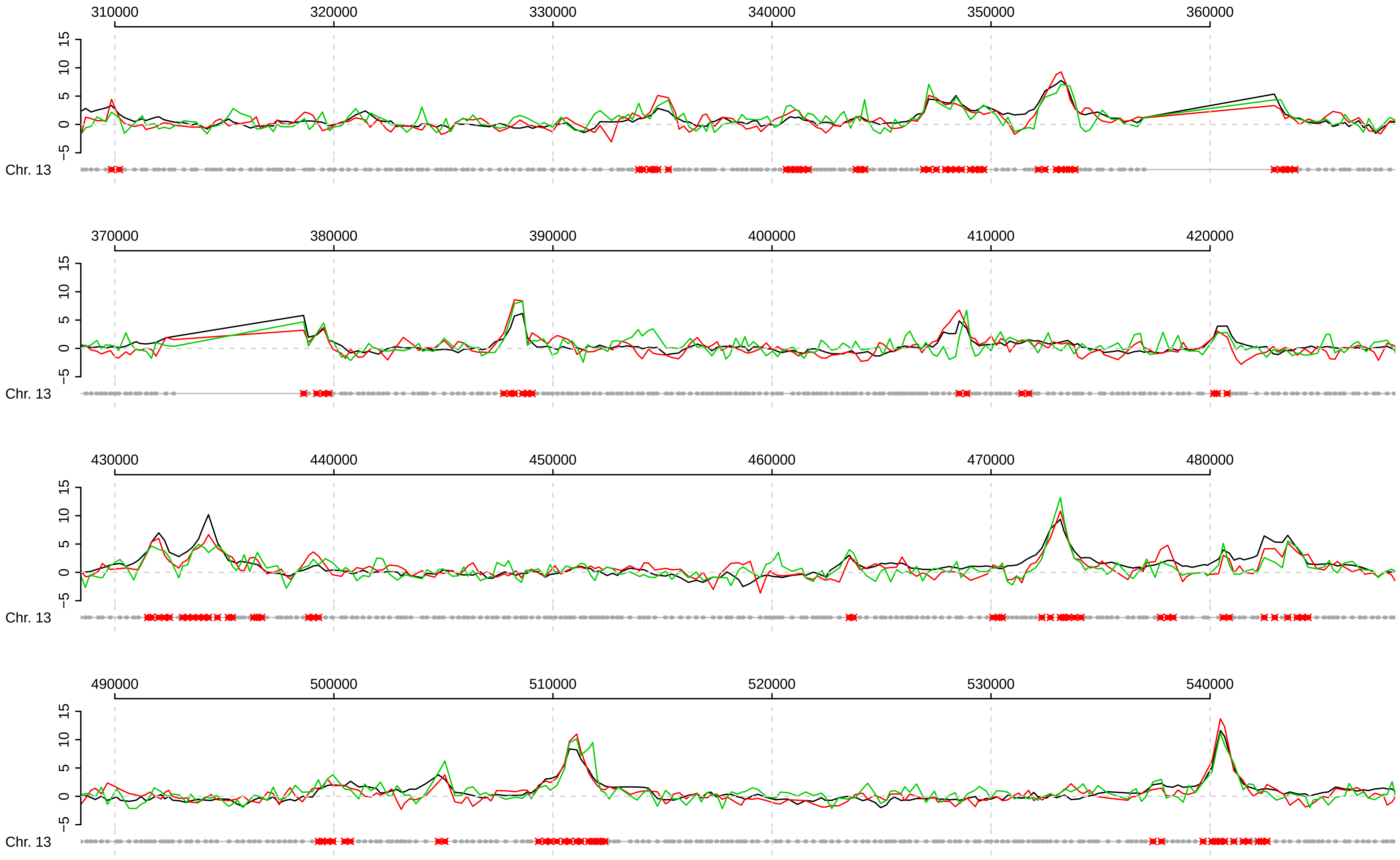

310000
320000
330000
340000
350000
360000
370000
380000
390000
400000
410000
420000
430000
440000
450000
460000
470000
480000
490000
500000
510000
520000
530000
540000
−5
0
5
10
15
Chr. 13

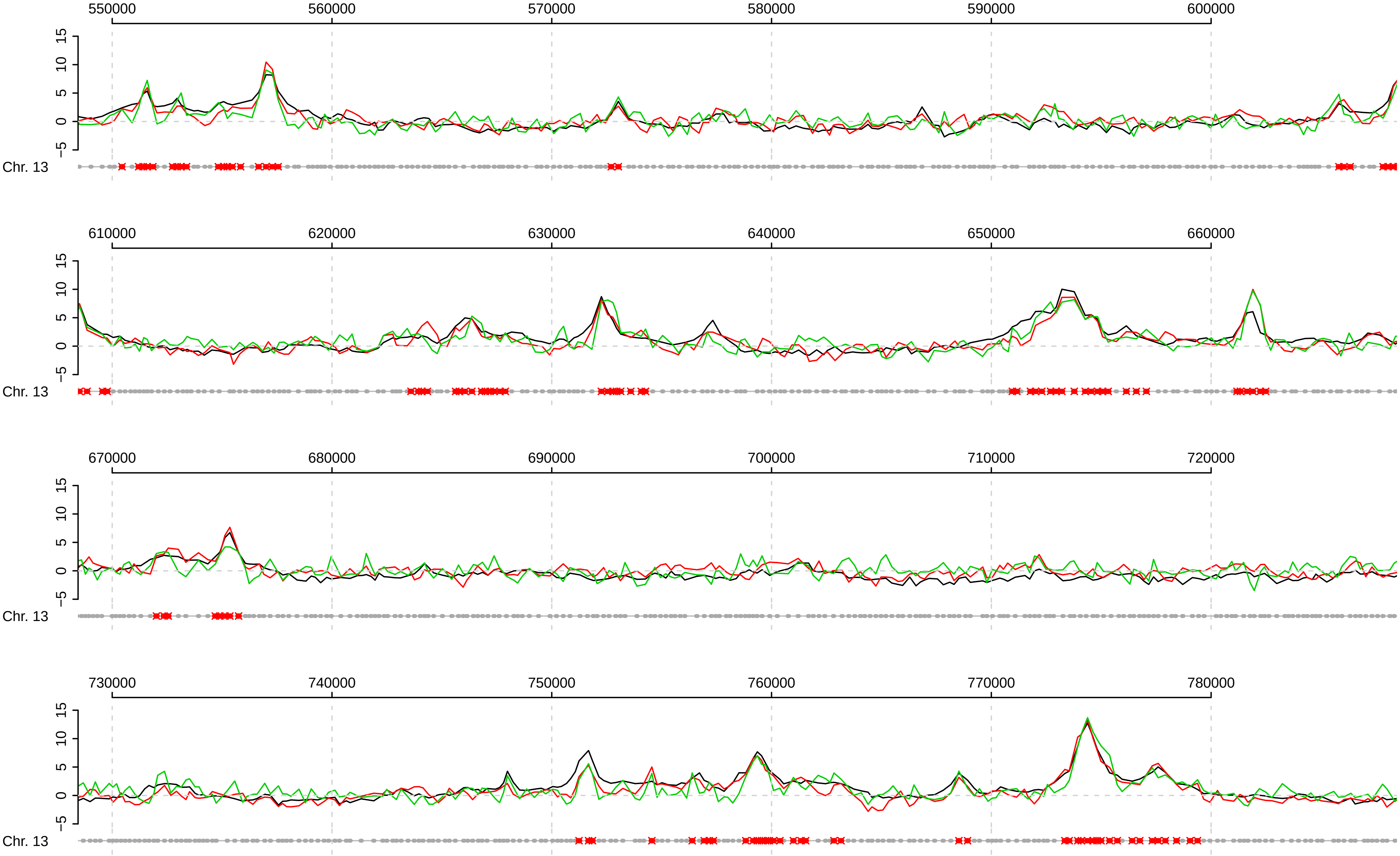

550000
560000
570000
580000
590000
600000
15
10
5
0
−5
Chr. 13
610000
620000
630000
640000
650000
660000
15
10
5
0
−5
Chr. 13
670000
680000
690000
700000
710000
720000
15
10
5
0
−5
Chr. 13
730000
740000
750000
760000
770000
780000
15
10
5
0
−5
Chr. 13

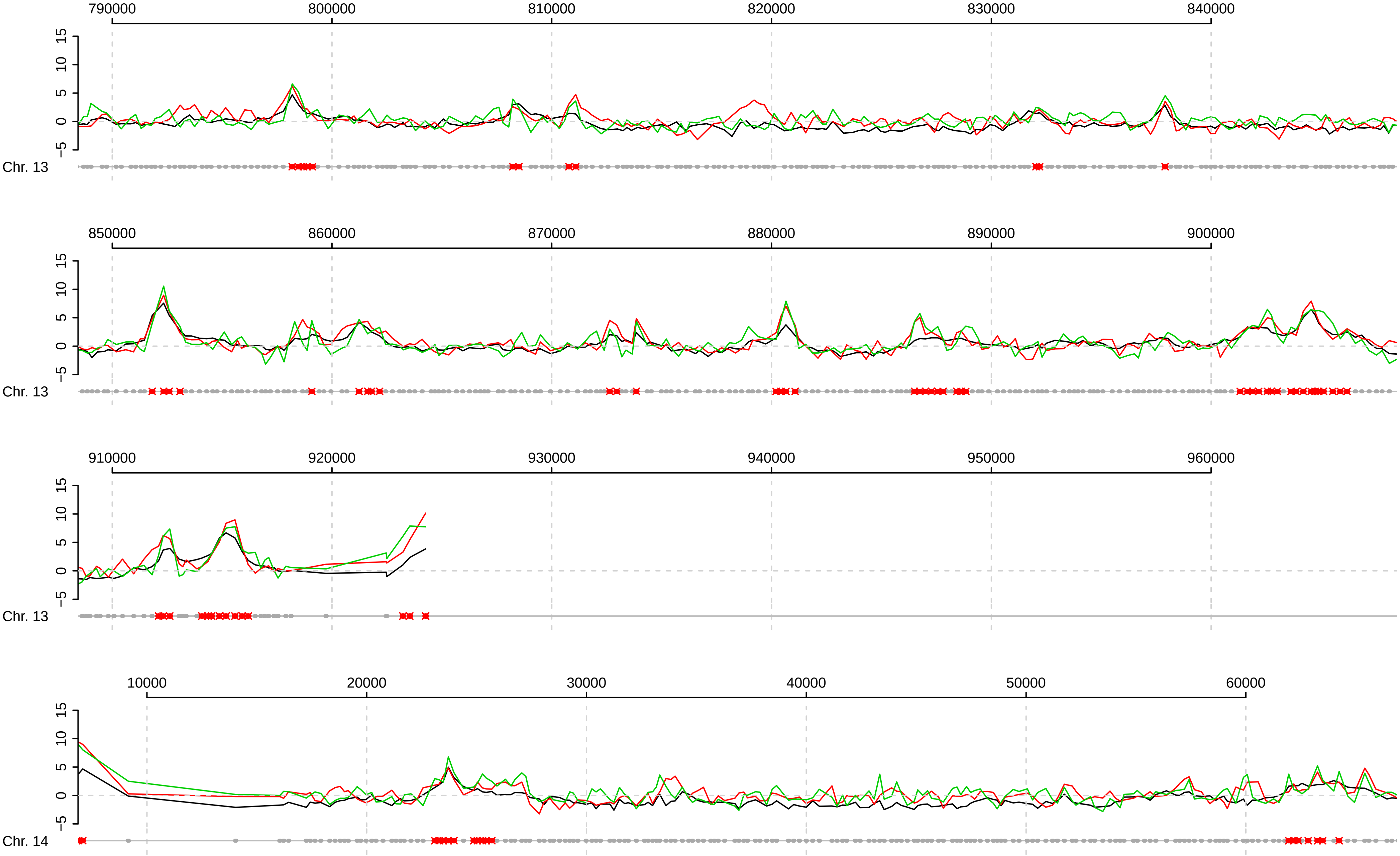

790000
800000
810000
820000
830000
840000
Chr. 13
850000
860000
870000
880000
890000
900000
Chr. 13
910000
920000
930000
940000
950000
960000
Chr. 13
10000
20000
30000
40000
50000
60000
Chr. 14
−5
0
5
10
15

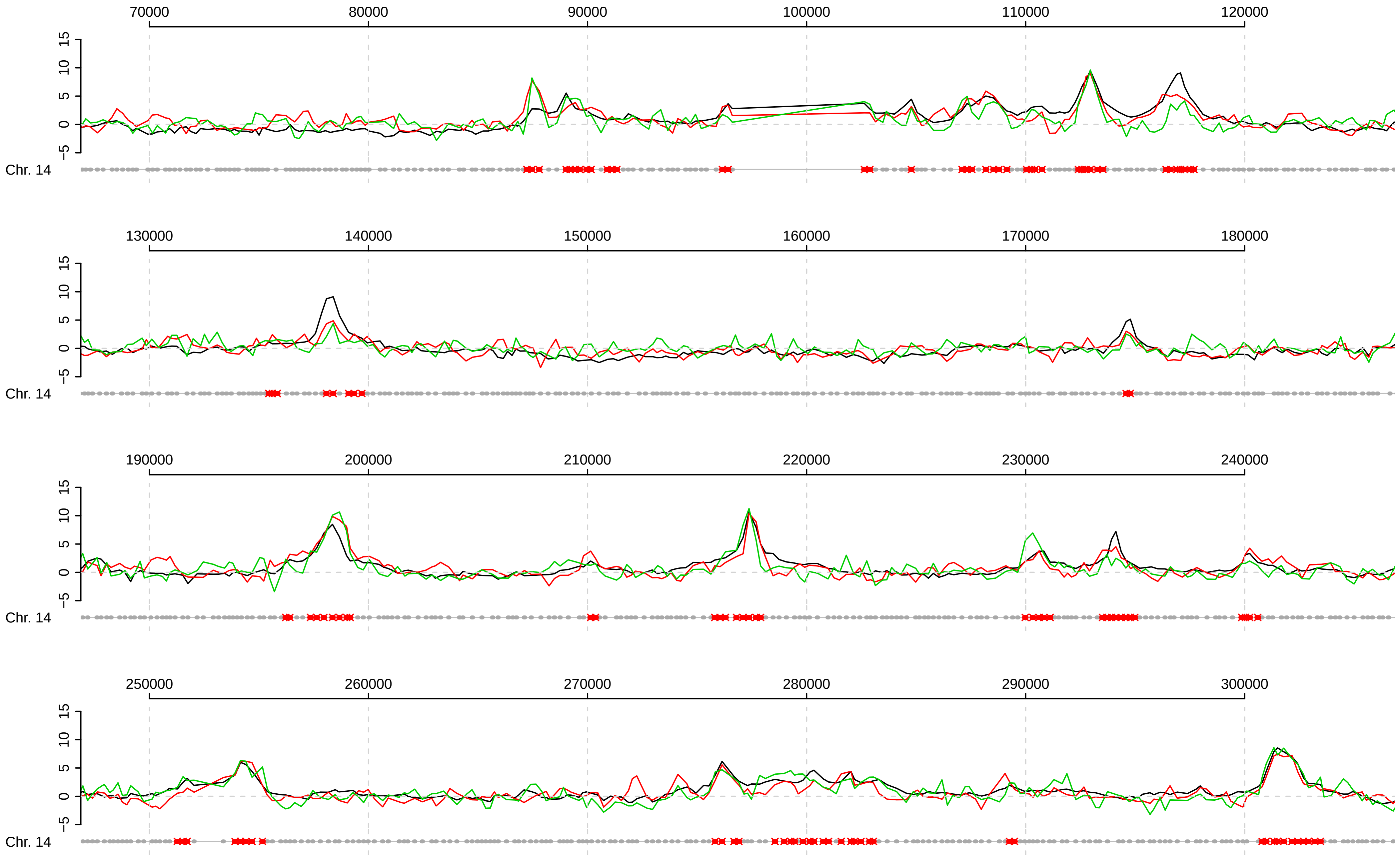

70000
80000
90000
100000
110000
120000
130000
140000
150000
160000
170000
180000
190000
200000
210000
220000
230000
240000
250000
260000
270000
280000
290000
300000
−5
0
5
10
15
Chr. 14

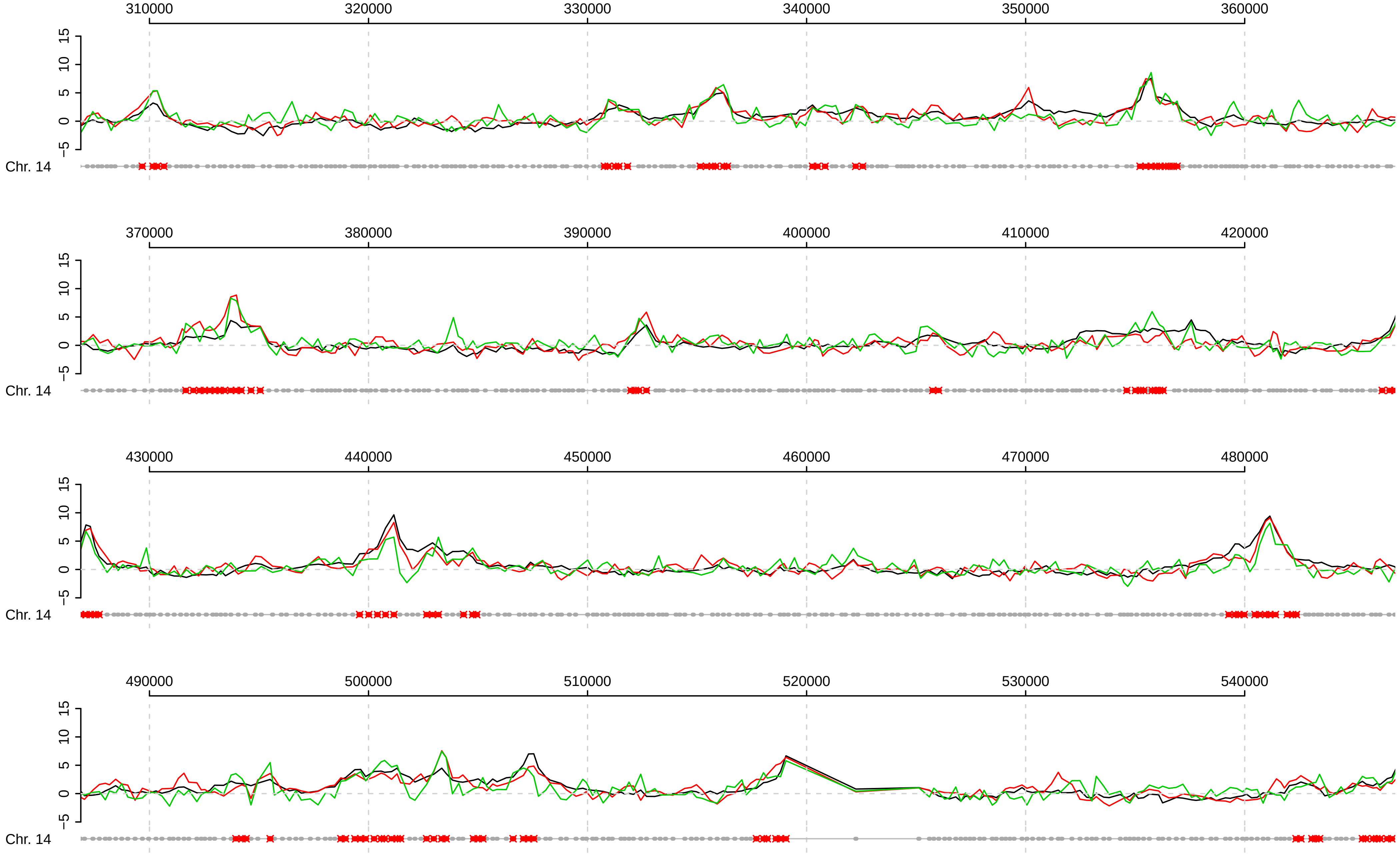
310000
320000
330000
340000
350000
360000
370000
380000
390000
400000
410000
420000
430000
440000
450000
460000
470000
480000
490000
500000
510000
520000
530000
540000
15
10
5
0
−5
Chr. 14

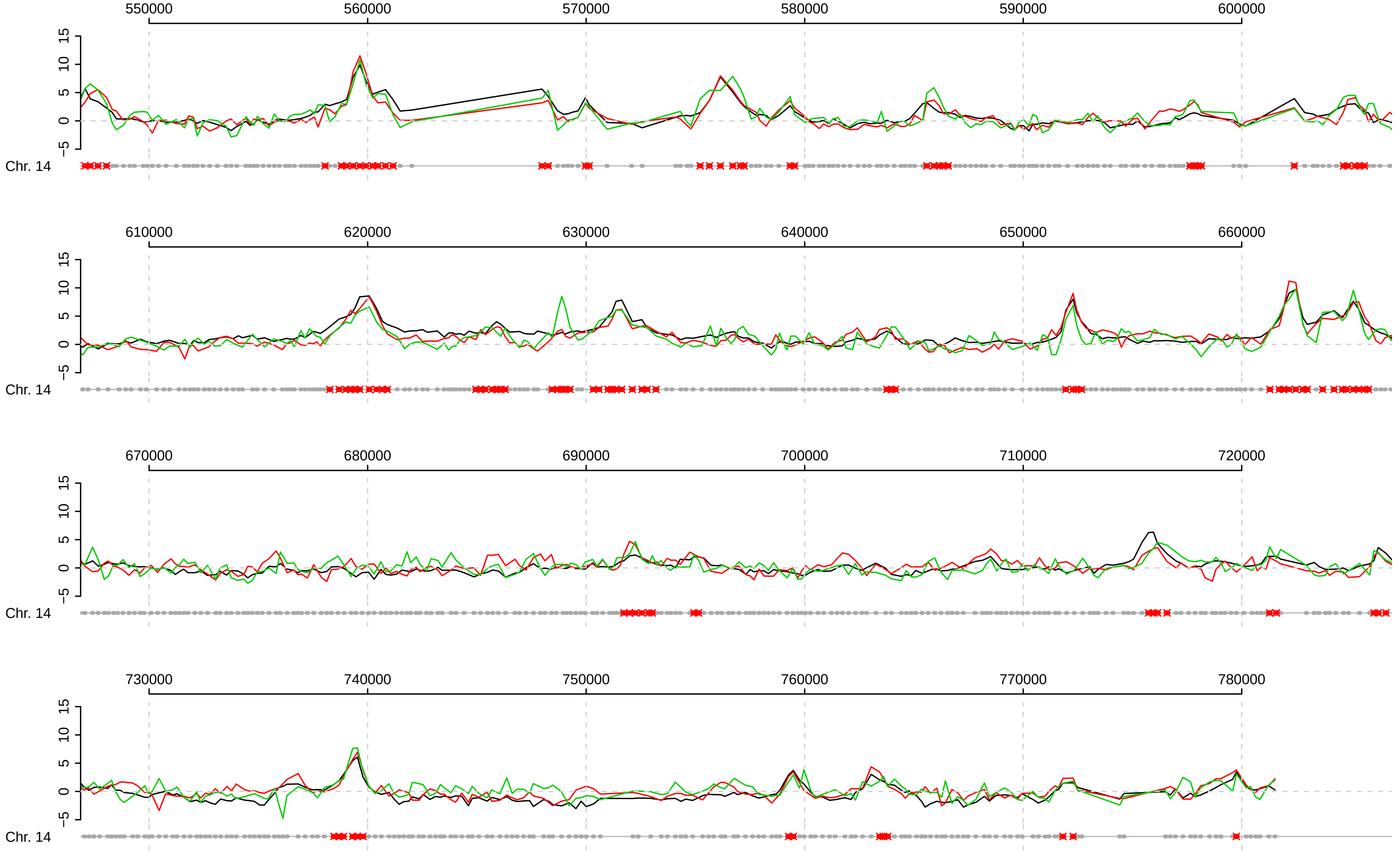

550000
560000
570000
580000
590000
600000
15
10
5
0
−5
Chr. 14
610000
620000
630000
640000
650000
660000
15
10
5
0
−5
Chr. 14
670000
680000
690000
700000
710000
720000
15
10
5
0
−5
Chr. 14
730000
740000
750000
760000
770000
780000
15
10
5
0
−5
Chr. 14

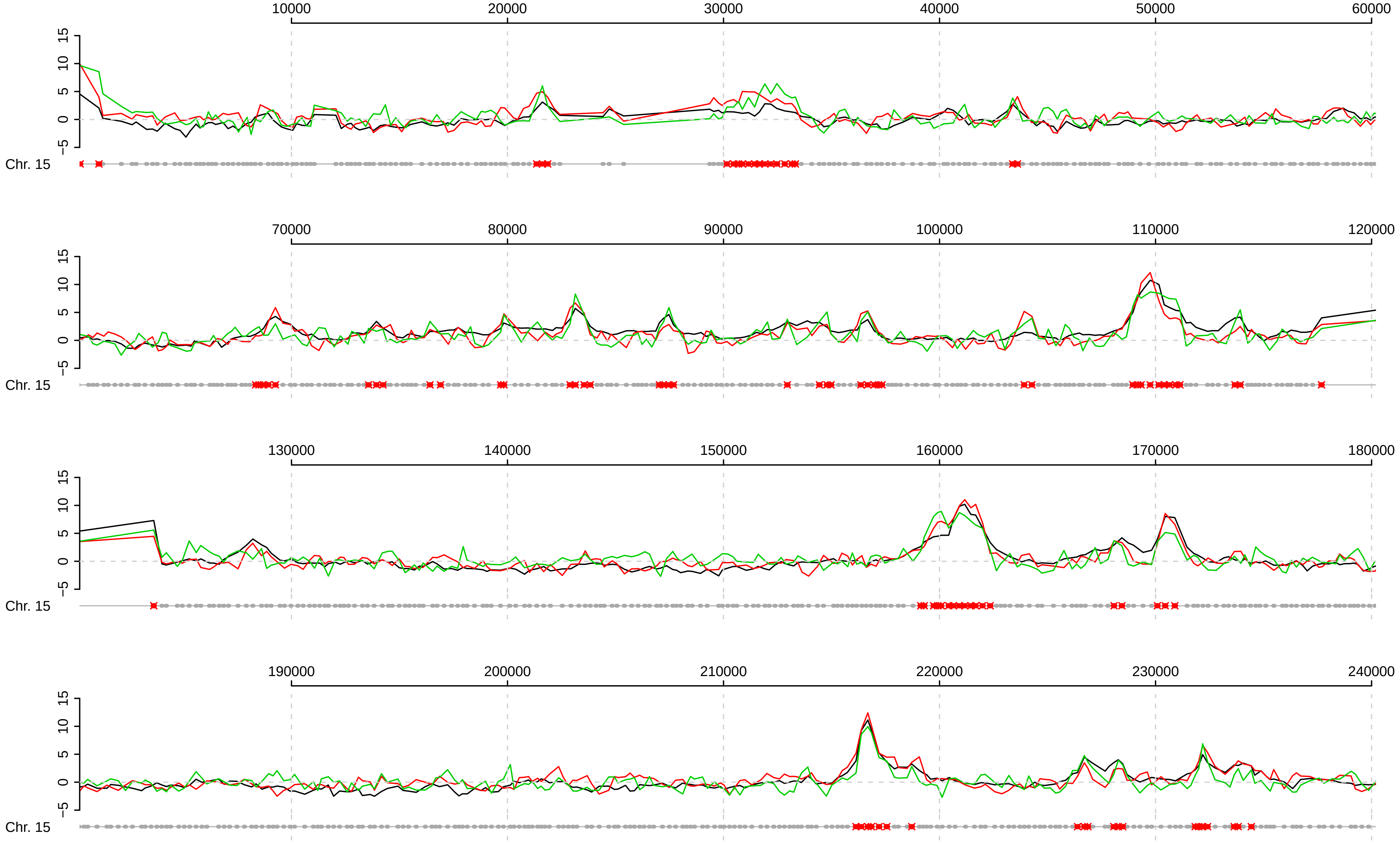

10000
20000
30000
40000
50000
60000
70000
80000
90000
100000
110000
120000
130000
140000
150000
160000
170000
180000
190000
200000
210000
220000
230000
240000
15
10
5
0
−5
Chr. 15

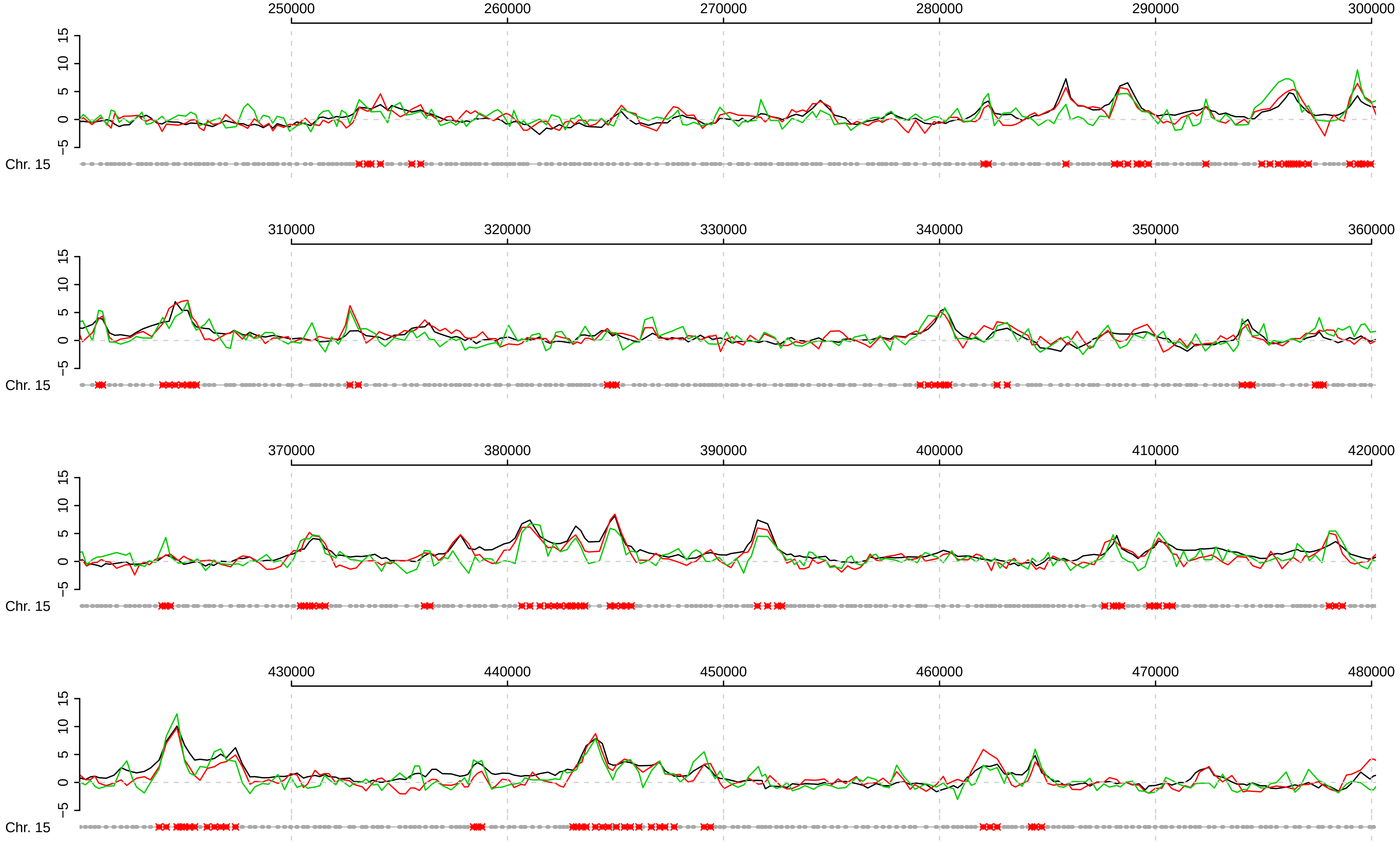
250000
260000
270000
280000
290000
300000
15
10
5
0
−5
Chr. 15
310000
320000
330000
340000
350000
360000
15
10
5
0
−5
Chr. 15
370000
380000
390000
400000
410000
420000
15
10
5
0
−5
Chr. 15
430000
440000
450000
460000
470000
480000
15
10
5
0
−5
Chr. 15

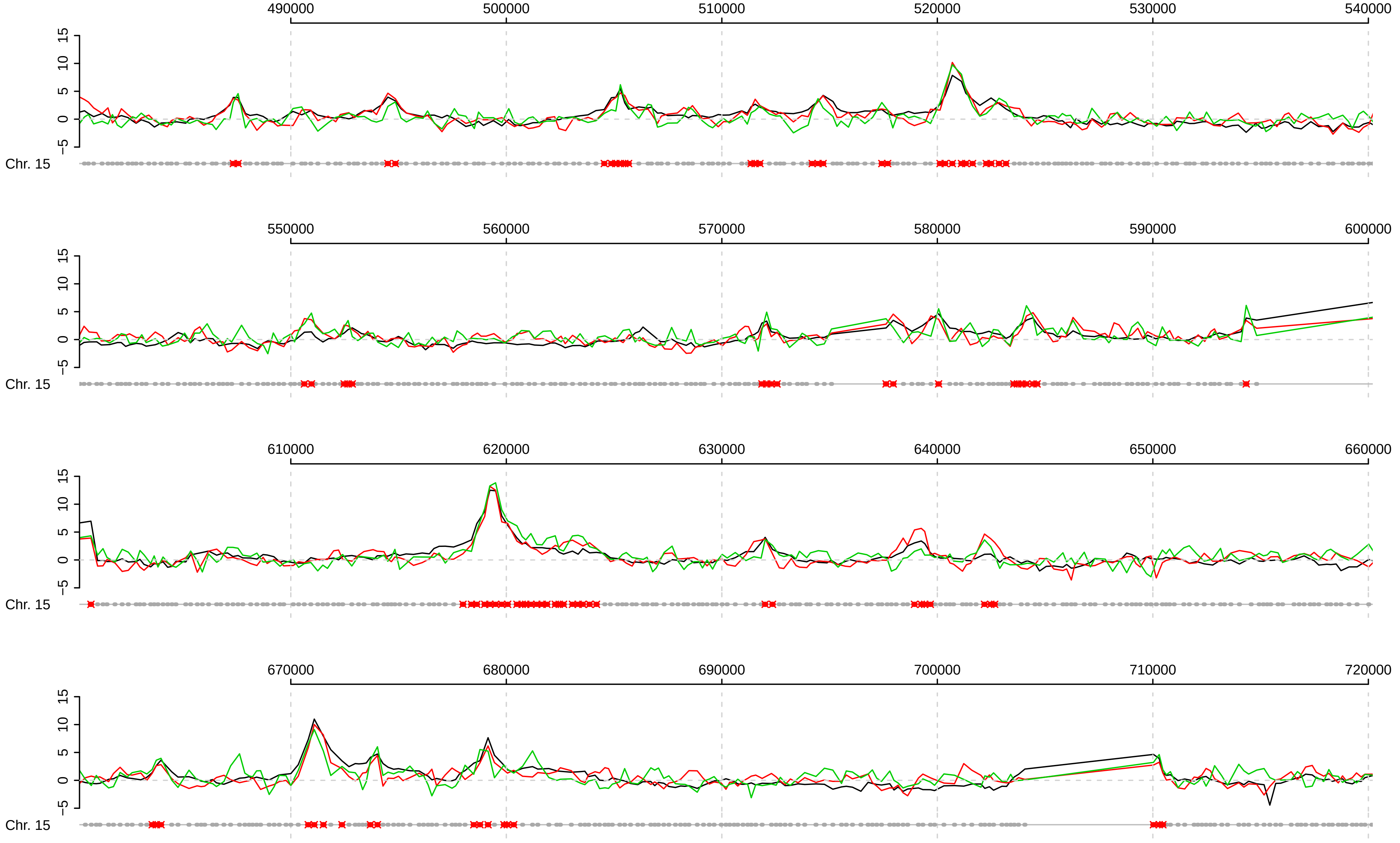

490000
500000
510000
520000
530000
540000
15
10
5
0
−5
Chr. 15
550000
560000
570000
580000
590000
600000
15
10
5
0
−5
Chr. 15
610000
620000
630000
640000
650000
660000
15
10
5
0
−5
Chr. 15
670000
680000
690000
700000
710000
720000
15
10
5
0
−5
Chr. 15

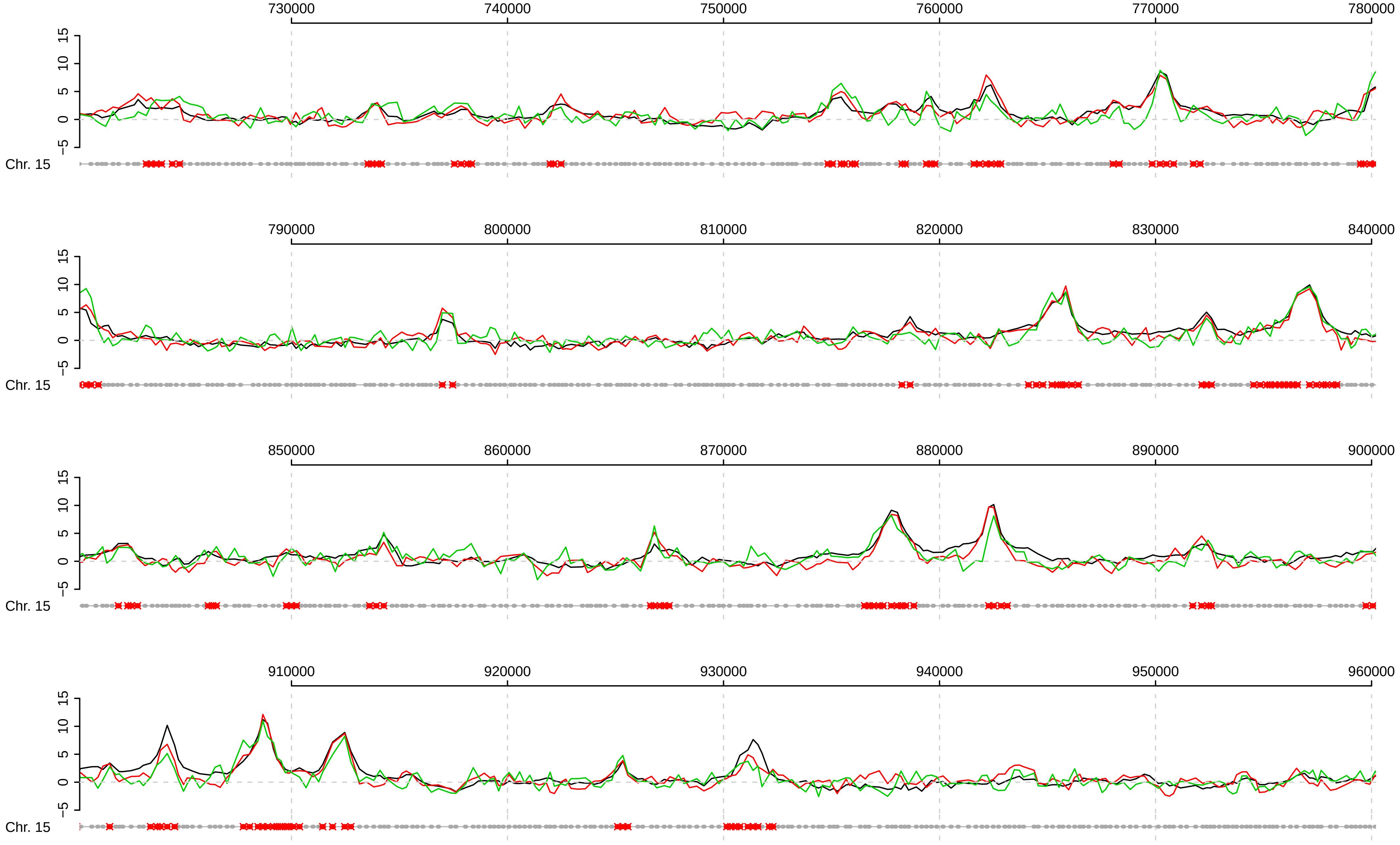

730000
740000
750000
760000
770000
780000
15
10
5
0
−5
Chr. 15
790000
800000
810000
820000
830000
840000
Chr. 15
850000
860000
870000
880000
890000
900000
Chr. 15
910000
920000
930000
940000
950000
960000
Chr. 15

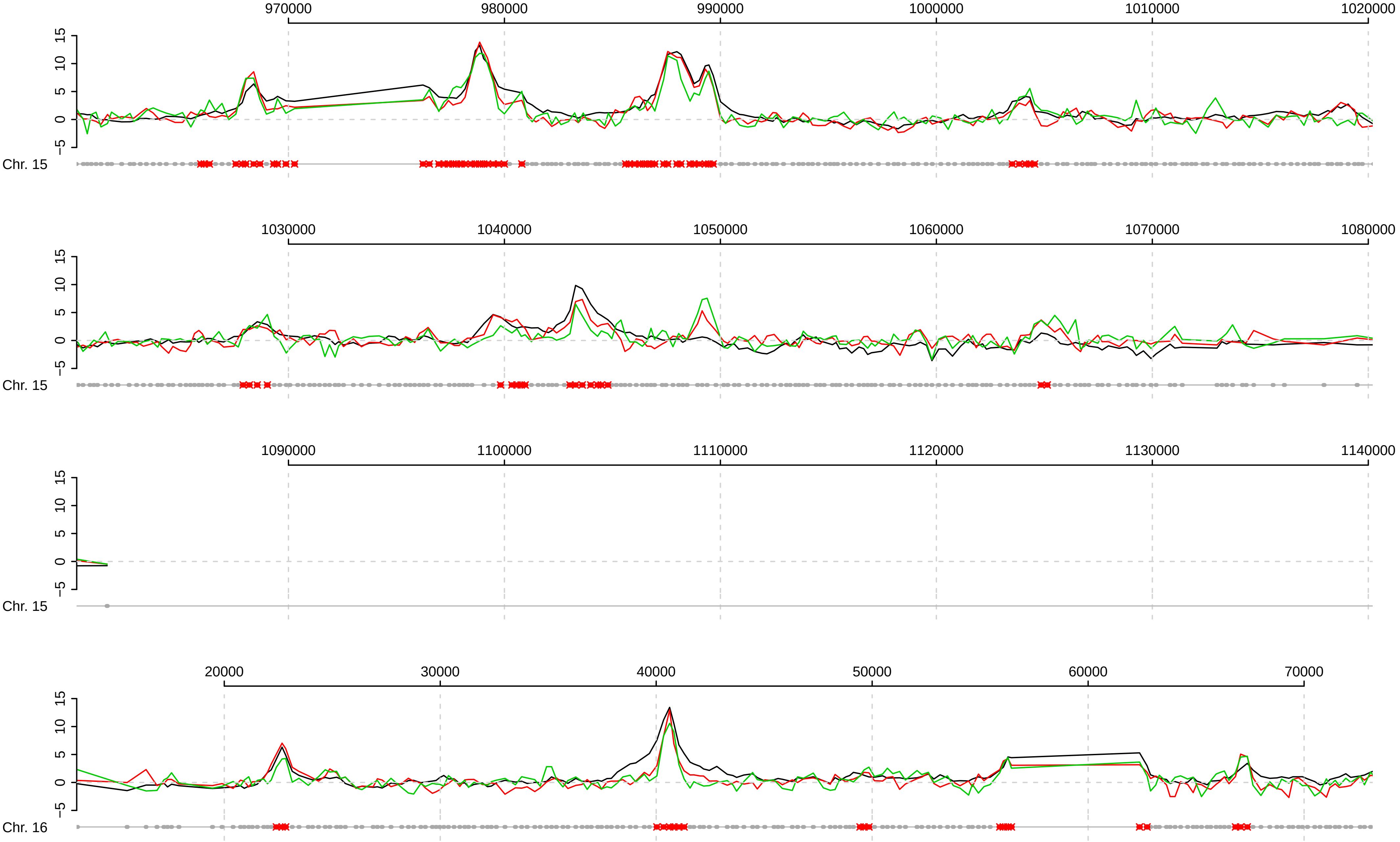
970000
980000
990000
1000000
1010000
1020000
15
10
5
0
−5
Chr. 15
1030000
1040000
1050000
1060000
1070000
1080000
15
10
5
0
−5
Chr. 15
1090000
1100000
1110000
1120000
1130000
1140000
15
10
5
0
−5
Chr. 15
20000
30000
40000
50000
60000
70000
15
10
5
0
−5
Chr. 16

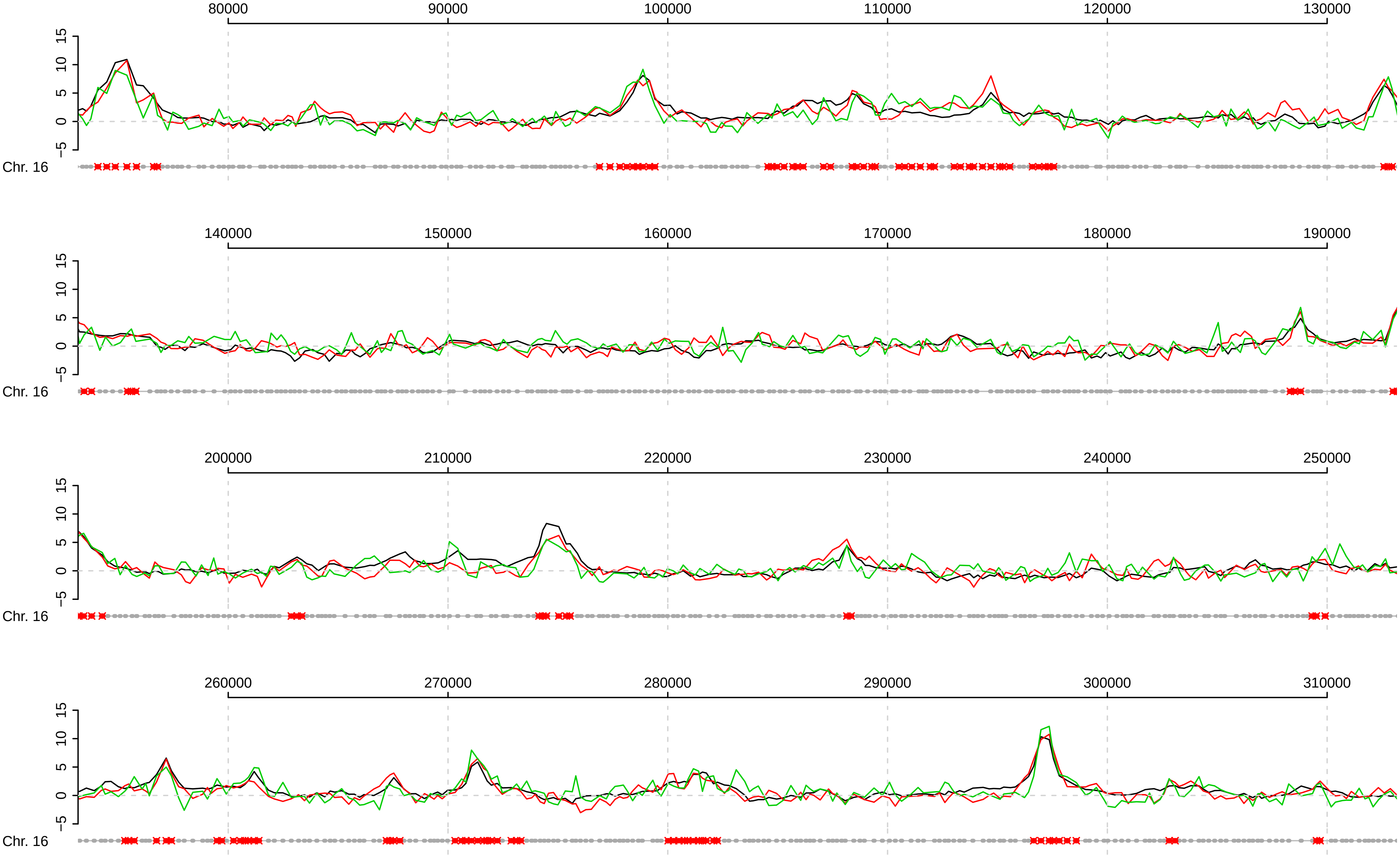

80000
90000
100000
110000
120000
130000
15
10
5
0
−5
Chr. 16
140000
150000
160000
170000
180000
190000
15
10
5
0
−5
Chr. 16
200000
210000
220000
230000
240000
250000
15
10
5
0
−5
Chr. 16
260000
270000
280000
290000
300000
310000
15
10
5
0
−5
Chr. 16

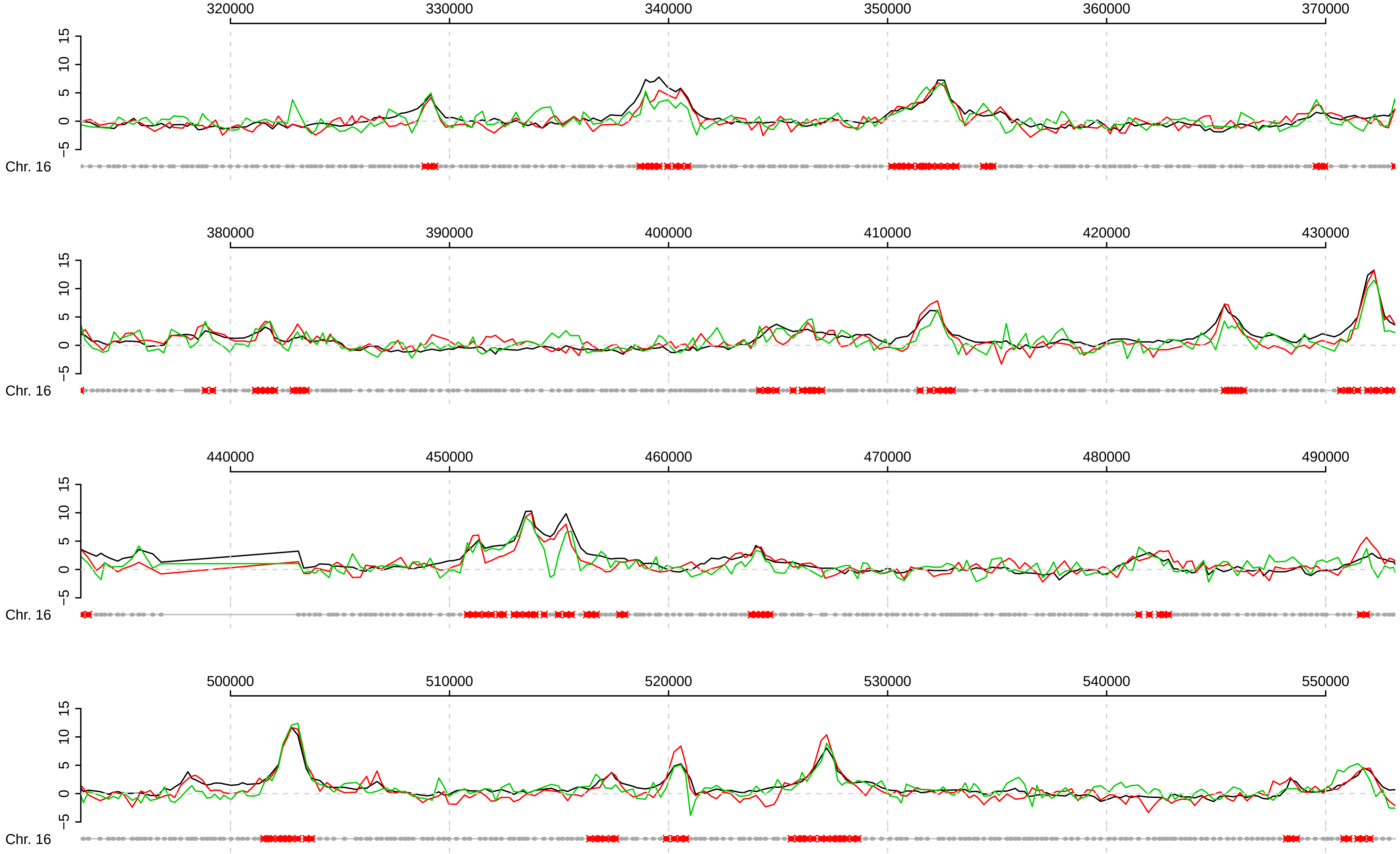
320000
330000
340000
350000
360000
370000
380000
390000
400000
410000
420000
430000
440000
450000
460000
470000
480000
490000
500000
510000
520000
530000
540000
550000
−5
0
5
10
15
Chr. 16

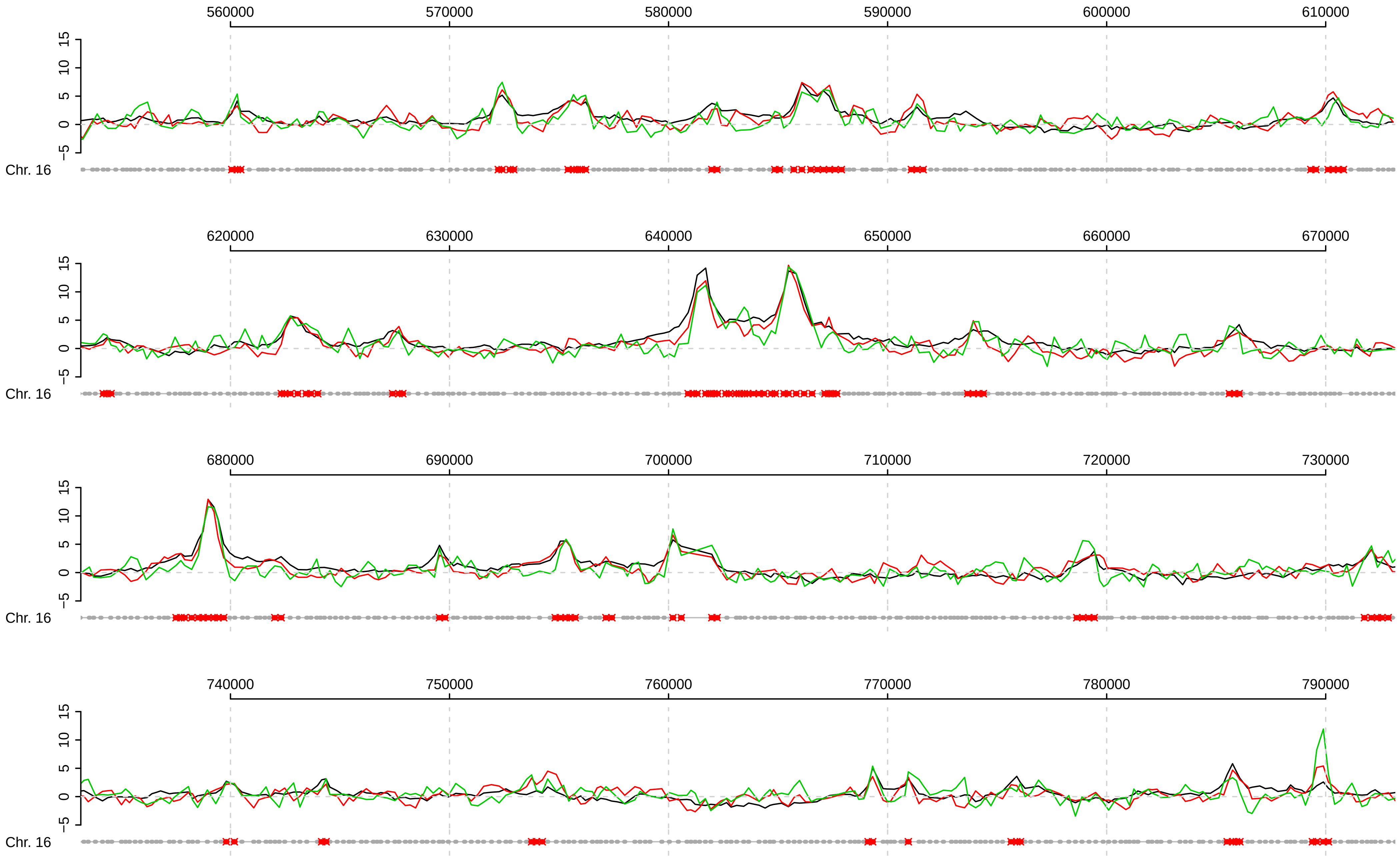

560000
570000
580000
590000
600000
610000
620000
630000
640000
650000
660000
670000
680000
690000
700000
710000
720000
730000
740000
750000
760000
770000
780000
790000
−5
0
5
10
15
Chr. 16

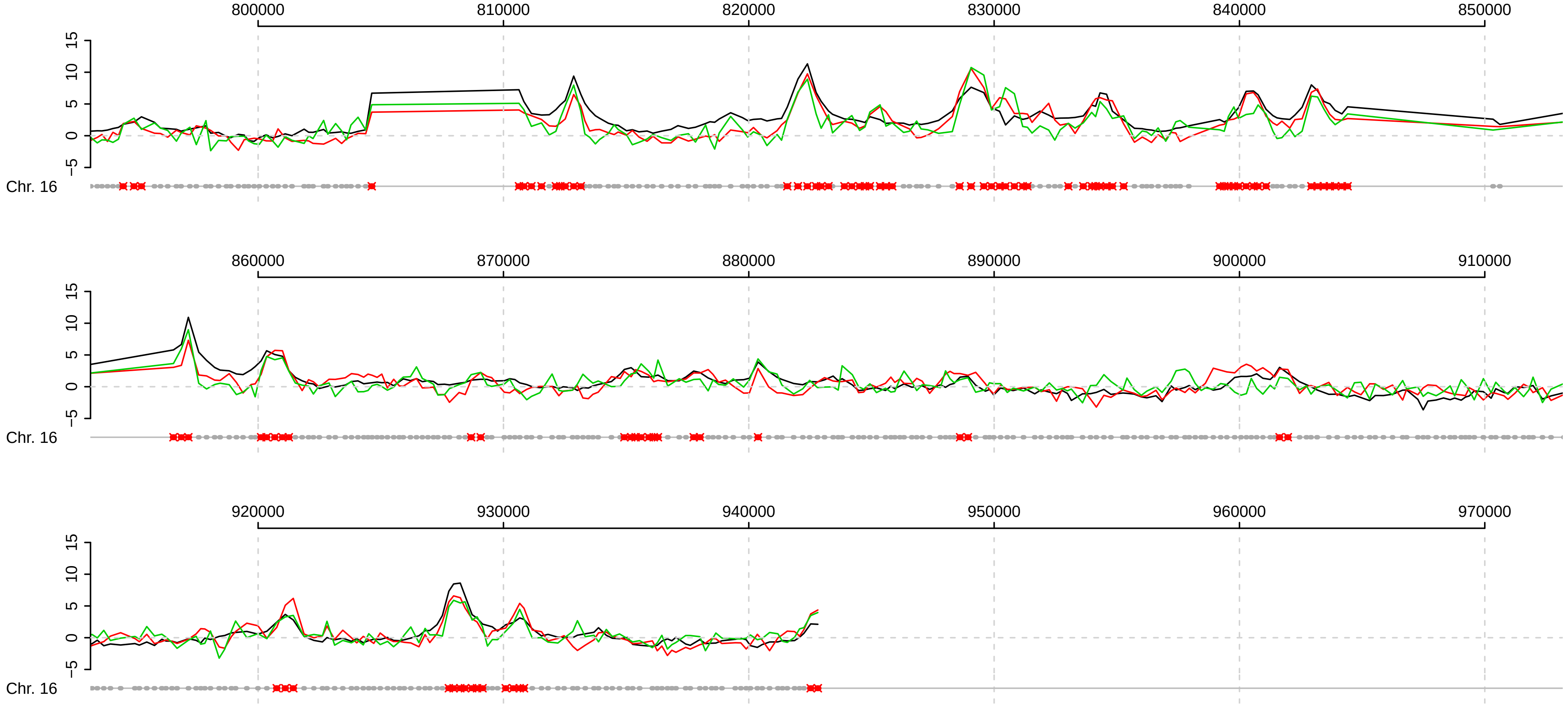
800000
810000
820000
830000
840000
850000
15
10
5
0
−5
Chr. 16
860000
870000
880000
890000
900000
910000
15
10
5
0
−5
Chr. 16
920000
930000
940000
950000
960000
970000
15
10
5
0
−5
Chr. 16

The following plots show Gcn5 enrichment over the whole genome. Black line shows average of untreated datasets. Red line shows average of T1 datasets. Green line shows average of T2 datasets.

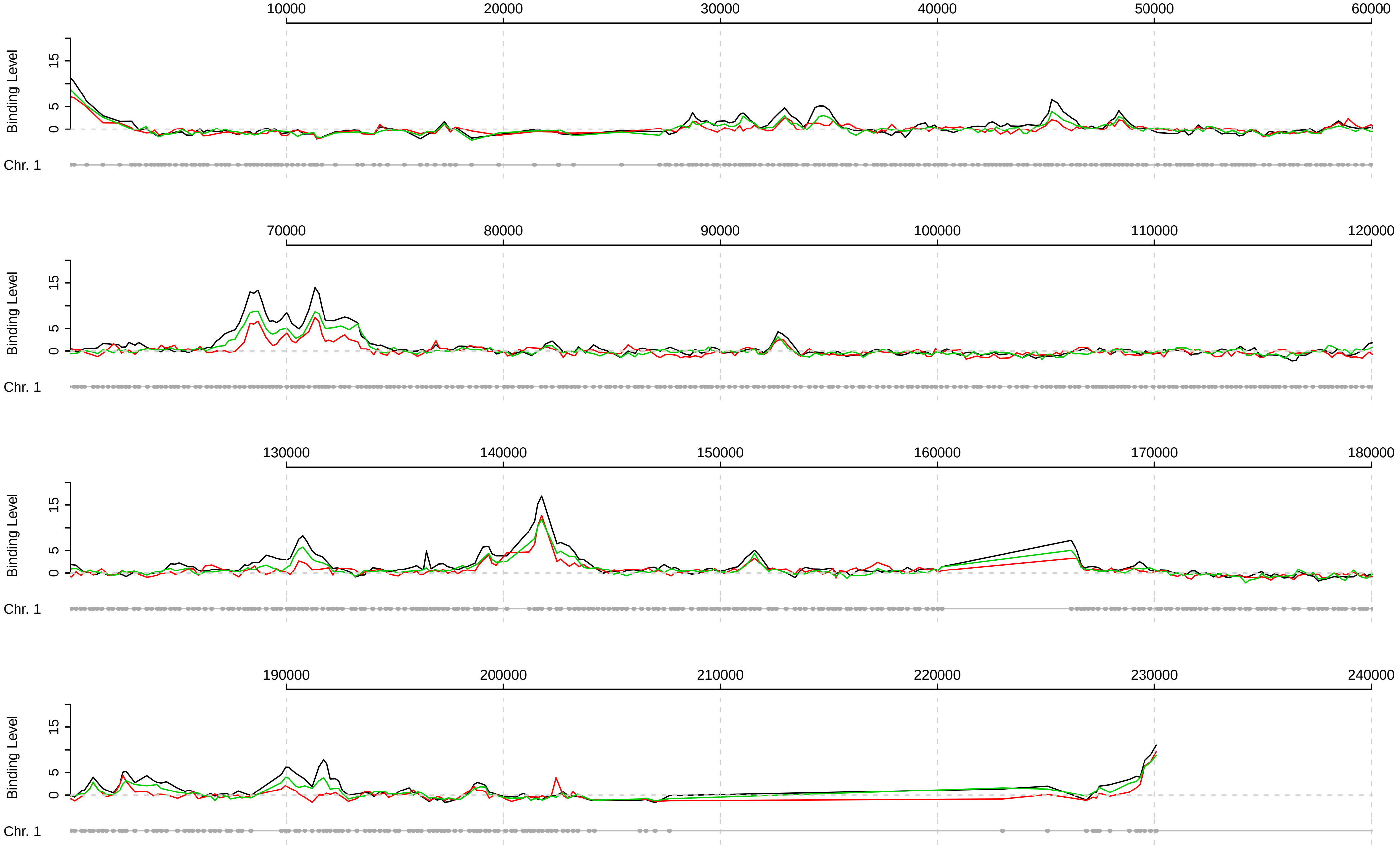

10000
20000
30000
40000
50000
60000
Binding Level
0
5
15
Chr. 1
70000
80000
90000
100000
110000
120000
Binding Level
0
5
15
Chr. 1
130000
140000
150000
160000
170000
180000
Binding Level
0
5
15
Chr. 1
190000
200000
210000
220000
230000
240000
Binding Level
0
5
15
Chr. 1

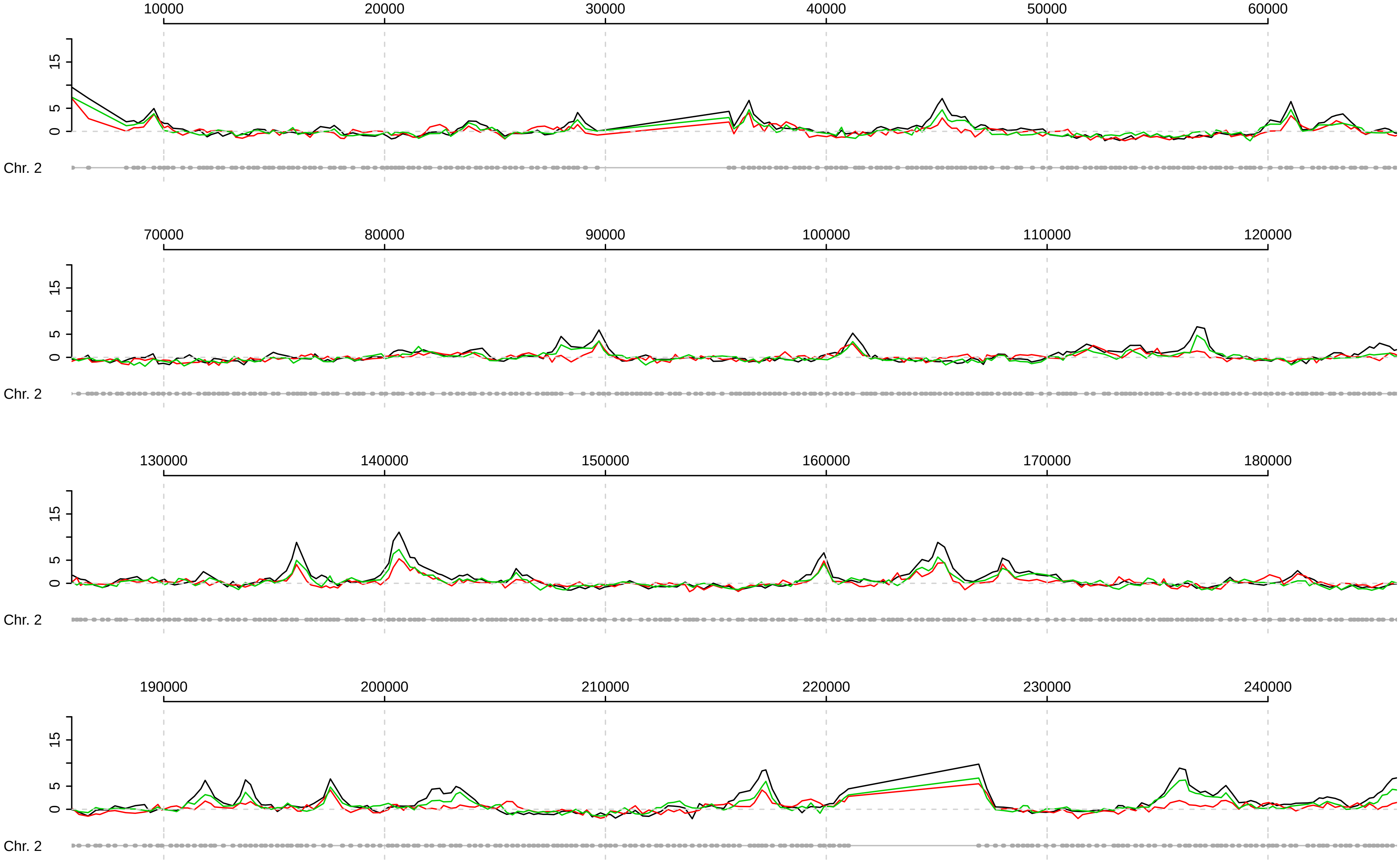
10000
20000
30000
40000
50000
60000
0
5
15
Chr. 2
70000
80000
90000
100000
110000
120000
0
5
15
Chr. 2
130000
140000
150000
160000
170000
180000
0
5
15
Chr. 2
190000
200000
210000
220000
230000
240000
0
5
15
Chr. 2

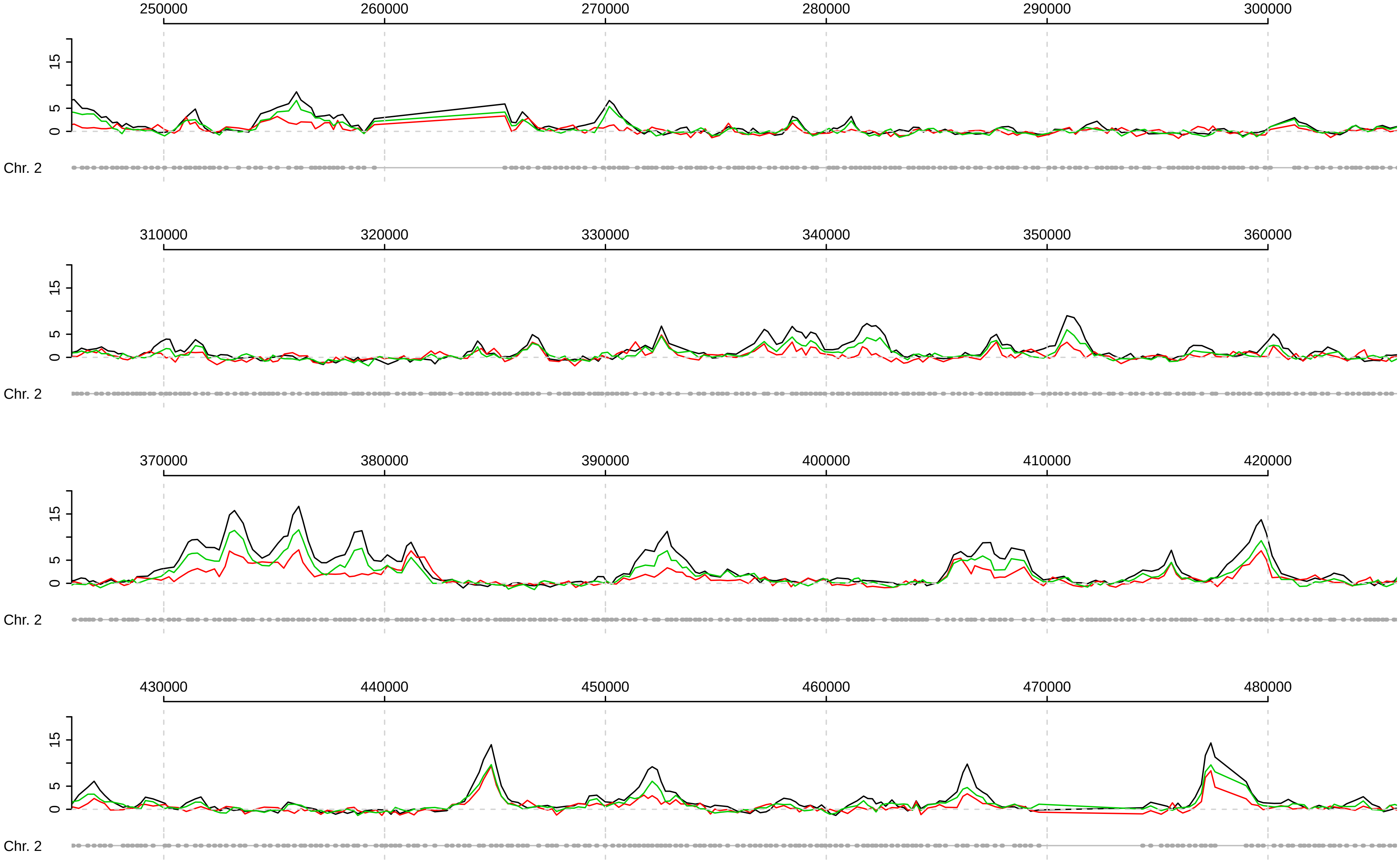

250000
260000
270000
280000
290000
300000
0
5
15
Chr. 2
310000
320000
330000
340000
350000
360000
0
5
15
Chr. 2
370000
380000
390000
400000
410000
420000
0
5
15
Chr. 2
430000
440000
450000
460000
470000
480000
0
5
15
Chr. 2

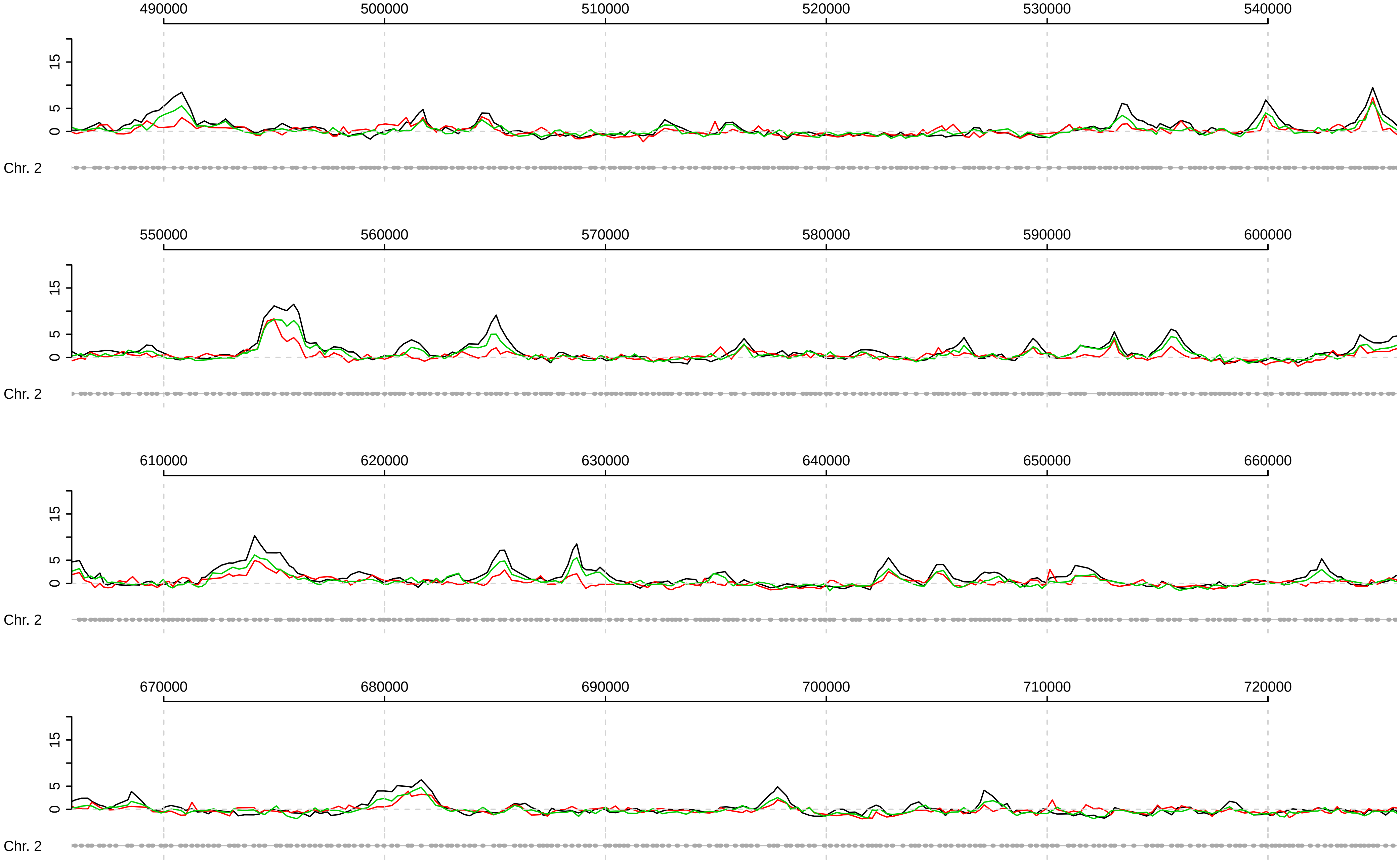
490000
500000
510000
520000
530000
540000
15
5
0
Chr. 2
550000
560000
570000
580000
590000
600000
15
5
0
Chr. 2
610000
620000
630000
640000
650000
660000
15
5
0
Chr. 2
670000
680000
690000
700000
710000
720000
15
5
0
Chr. 2

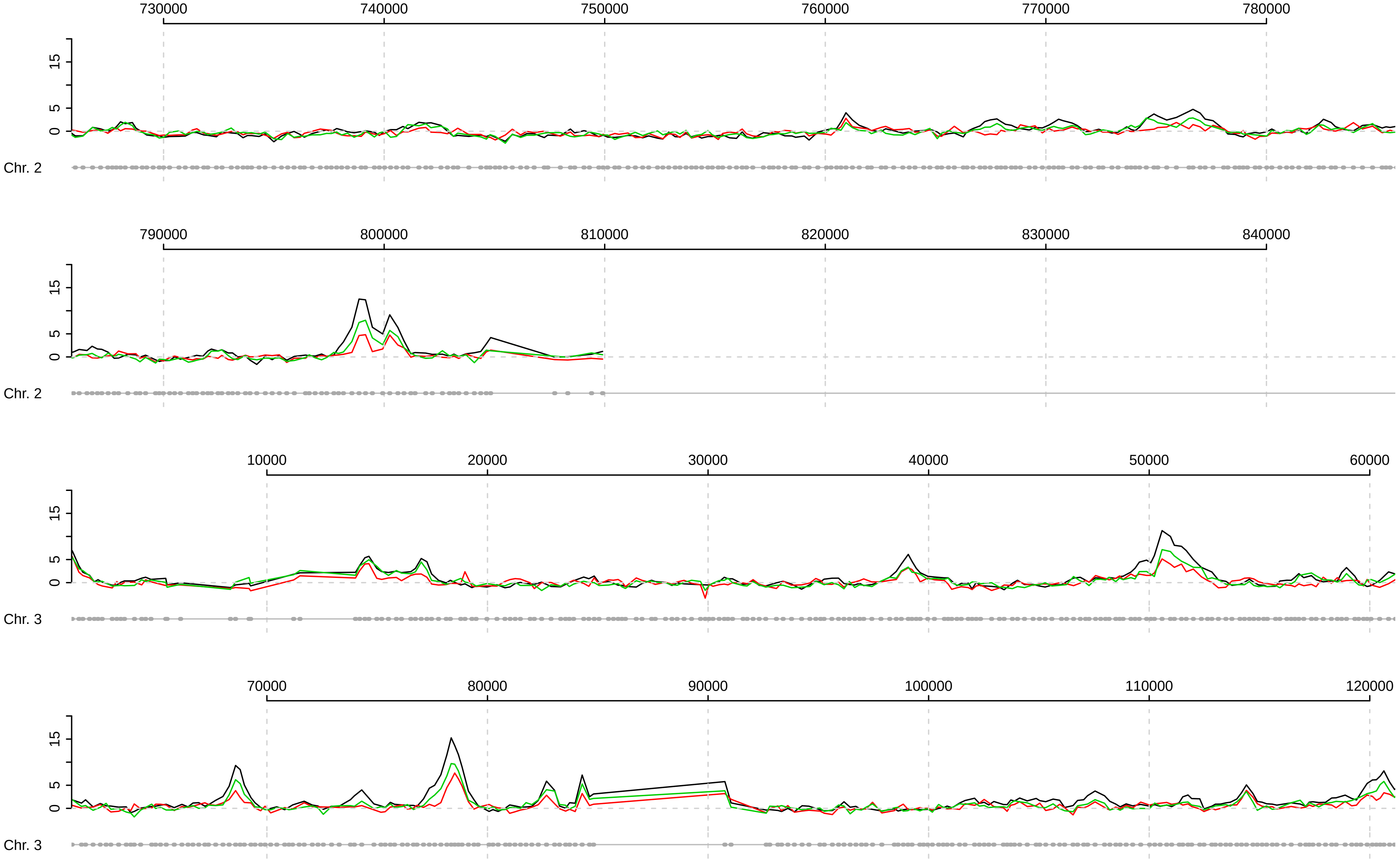

730000
740000
750000
760000
770000
780000
0
5
15
Chr. 2
790000
800000
810000
820000
830000
840000
0
5
15
Chr. 2
10000
20000
30000
40000
50000
60000
0
5
15
Chr. 3
70000
80000
90000
100000
110000
120000
0
5
15
Chr. 3

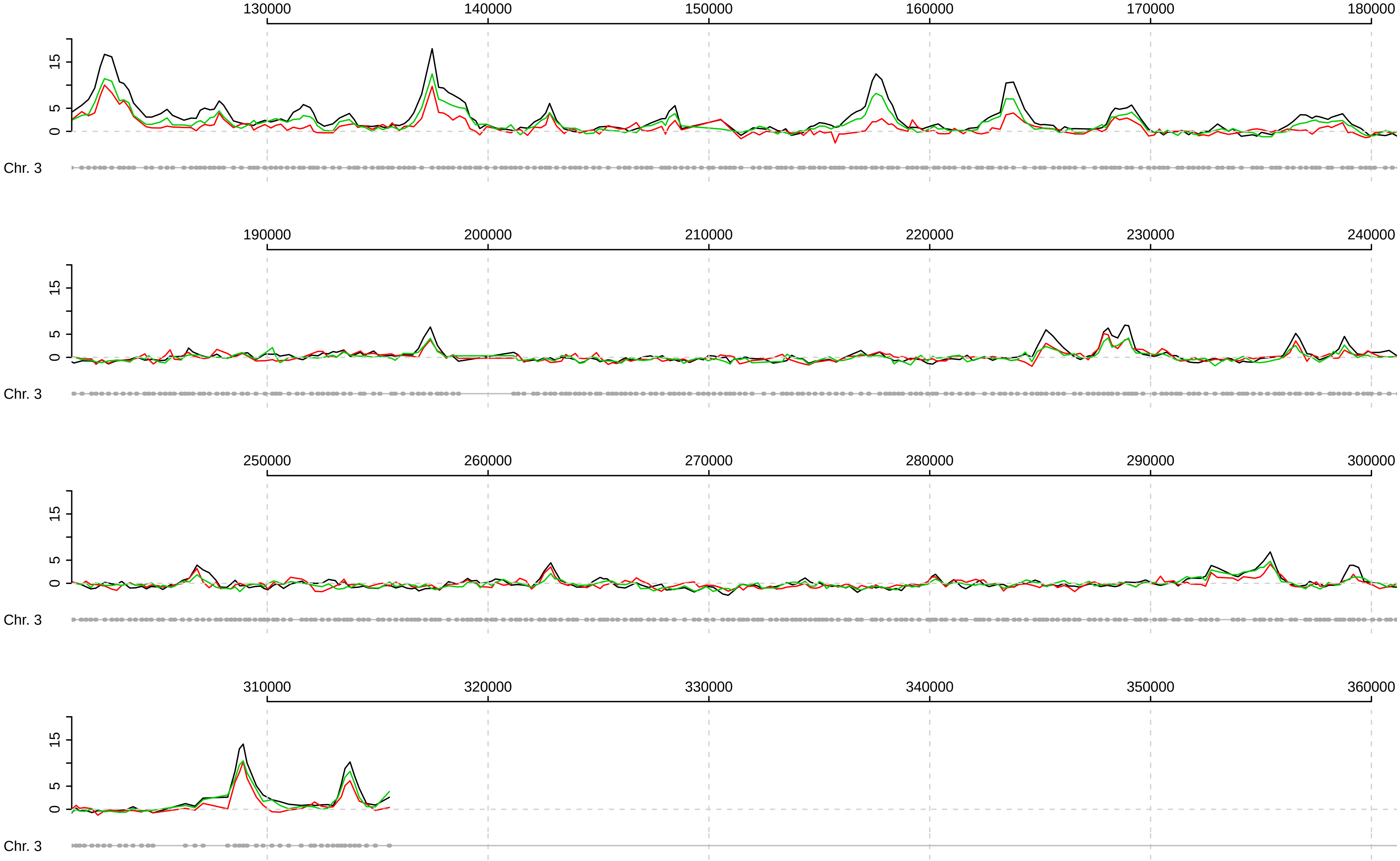

130000
140000
150000
160000
170000
180000
15
5
0
Chr. 3
190000
200000
210000
220000
230000
240000
15
5
0
Chr. 3
250000
260000
270000
280000
290000
300000
15
5
0
Chr. 3
310000
320000
330000
340000
350000
360000
15
5
0
Chr. 3

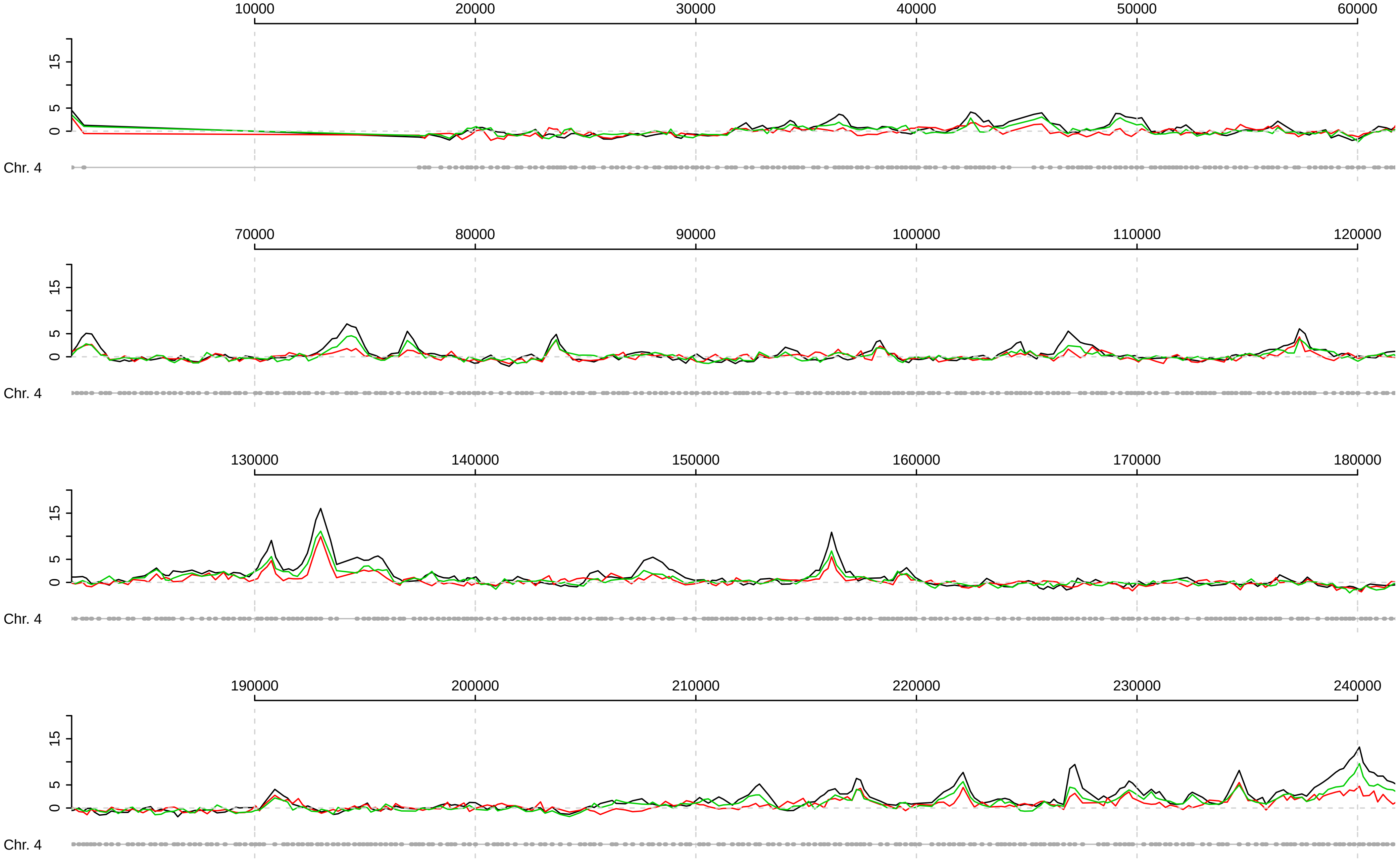
10000
20000
30000
40000
50000
60000
15
5
0
Chr. 4
70000
80000
90000
100000
110000
120000
15
5
0
Chr. 4
130000
140000
150000
160000
170000
180000
15
5
0
Chr. 4
190000
200000
210000
220000
230000
240000
15
5
0
Chr. 4

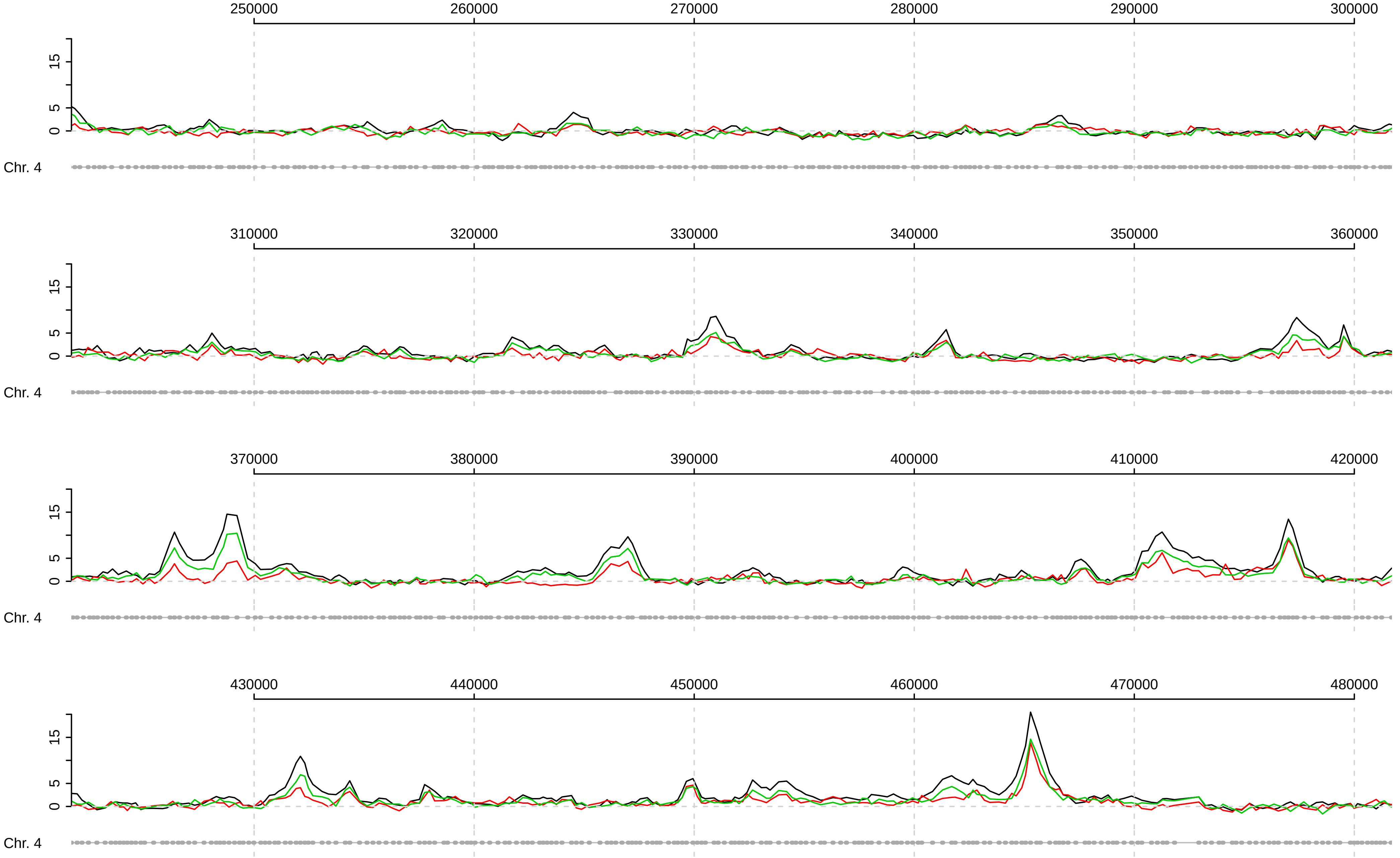

250000
260000
270000
280000
290000
300000
0
5
15
Chr. 4
310000
320000
330000
340000
350000
360000
0
5
15
Chr. 4
370000
380000
390000
400000
410000
420000
0
5
15
Chr. 4
430000
440000
450000
460000
470000
480000
0
5
15
Chr. 4

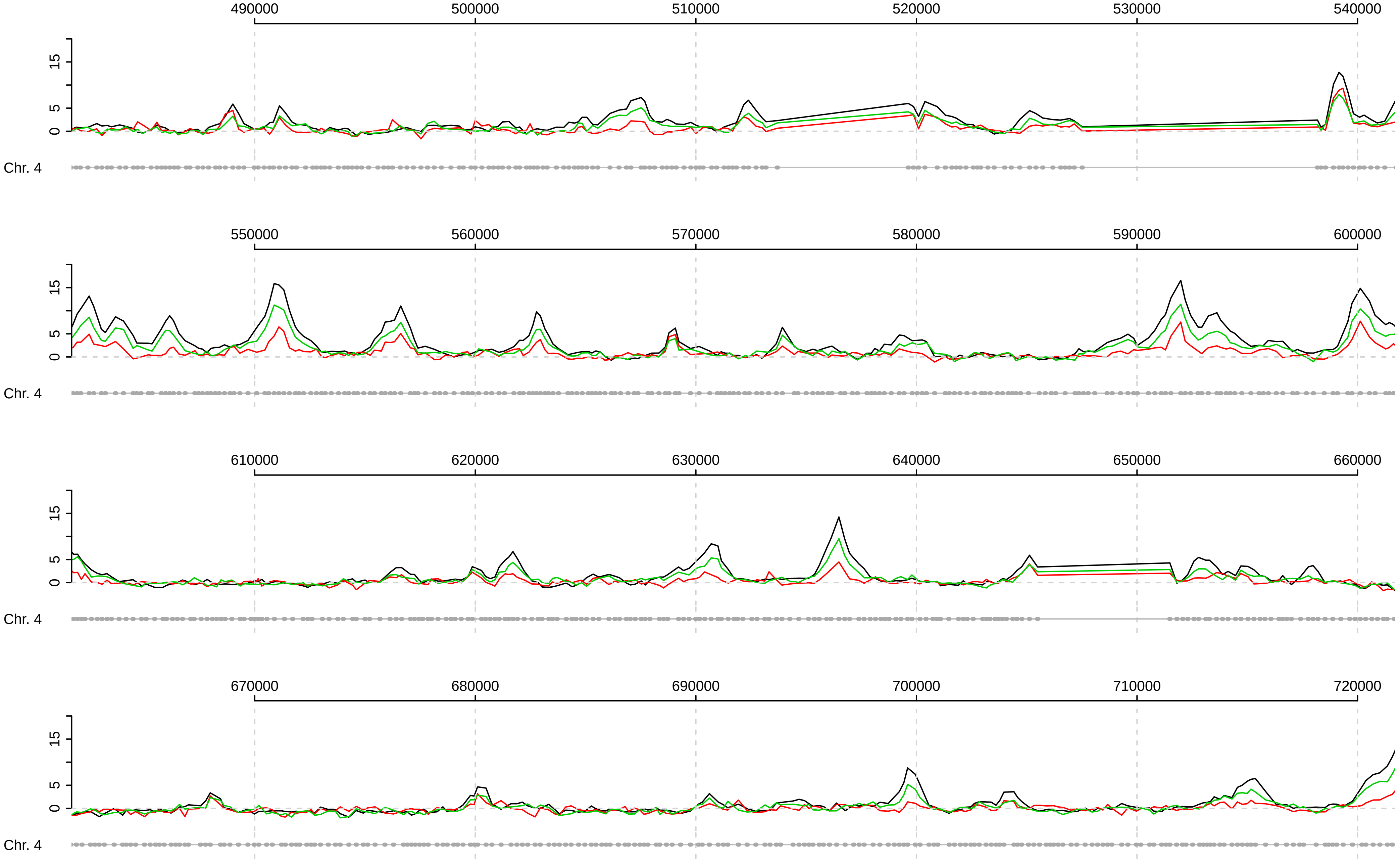
490000
500000
510000
520000
530000
540000
0
5
15
Chr. 4
550000
560000
570000
580000
590000
600000
0
5
15
Chr. 4
610000
620000
630000
640000
650000
660000
0
5
15
Chr. 4
670000
680000
690000
700000
710000
720000
0
5
15
Chr. 4

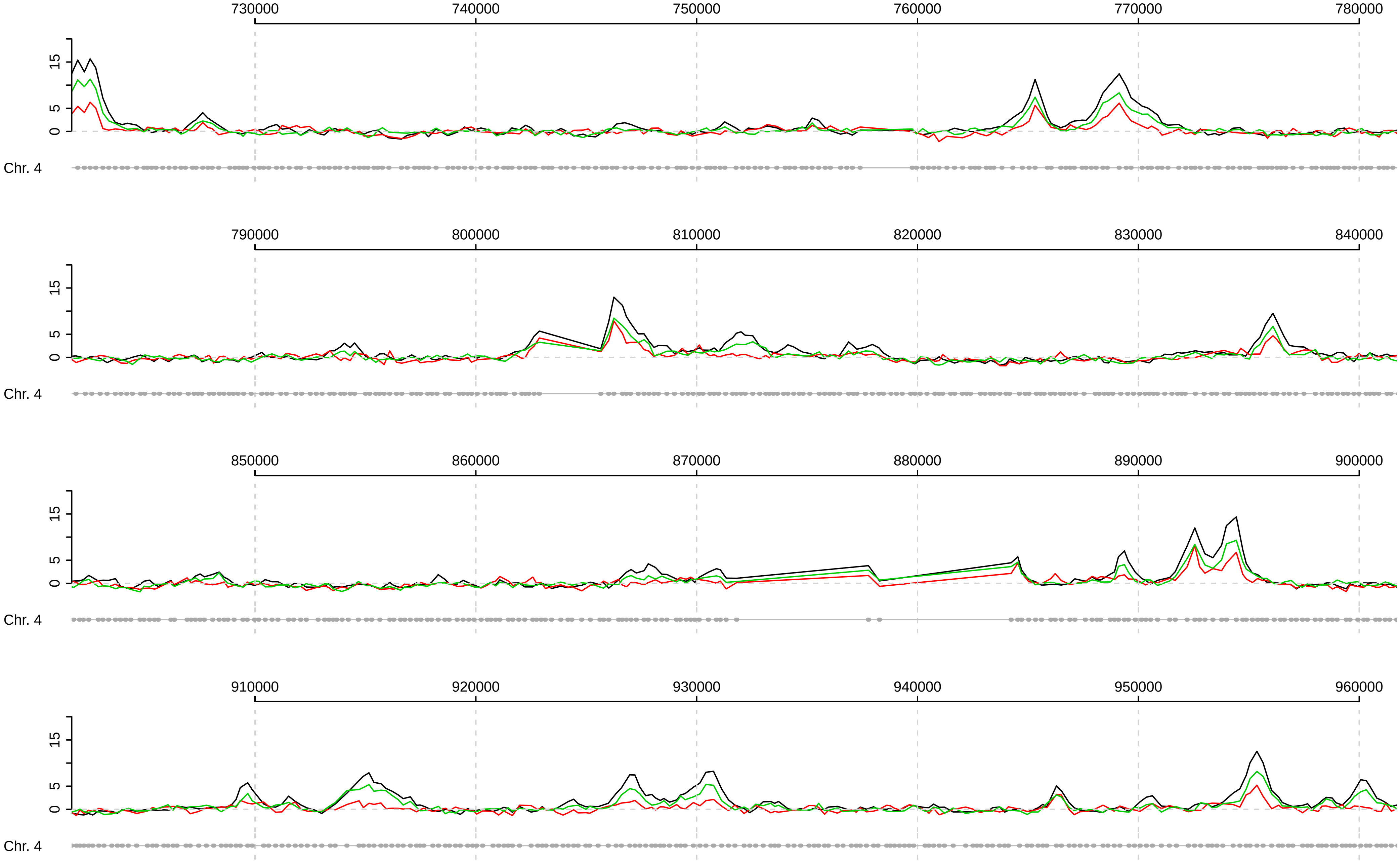

730000 740000 750000 760000 770000 780000
0 5 15
Chr. 4
790000 800000 810000 820000 830000 840000
0 5 15
Chr. 4
850000 860000 870000 880000 890000 900000
0 5 15
Chr. 4
910000 920000 930000 940000 950000 960000
0 5 15
Chr. 4

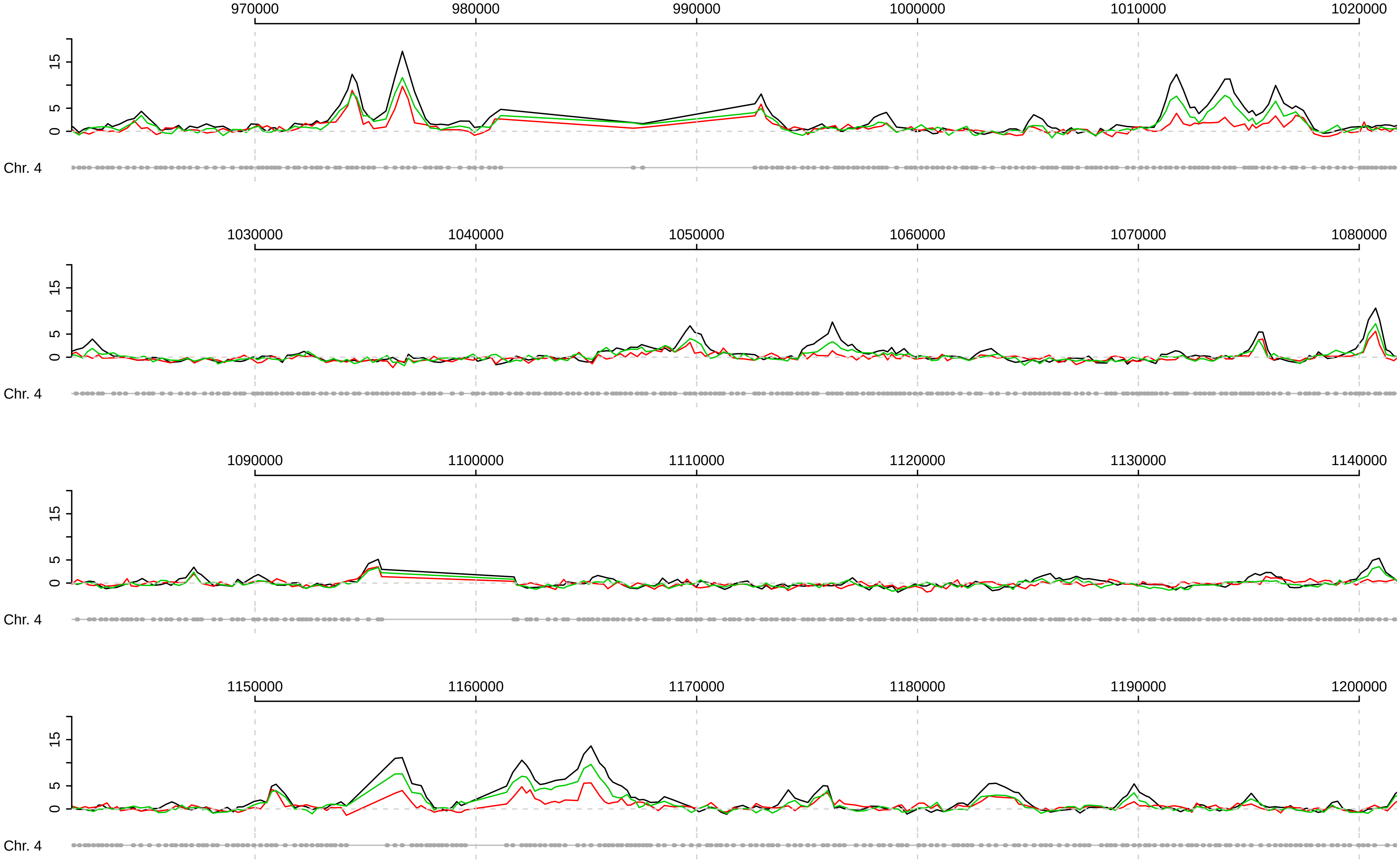

970000
980000
990000
1000000
1010000
1020000
15
5
0
Chr. 4
1030000
1040000
1050000
1060000
1070000
1080000
15
5
0
Chr. 4
1090000
1100000
1110000
1120000
1130000
1140000
15
5
0
Chr. 4
1150000
1160000
1170000
1180000
1190000
1200000
15
5
0
Chr. 4

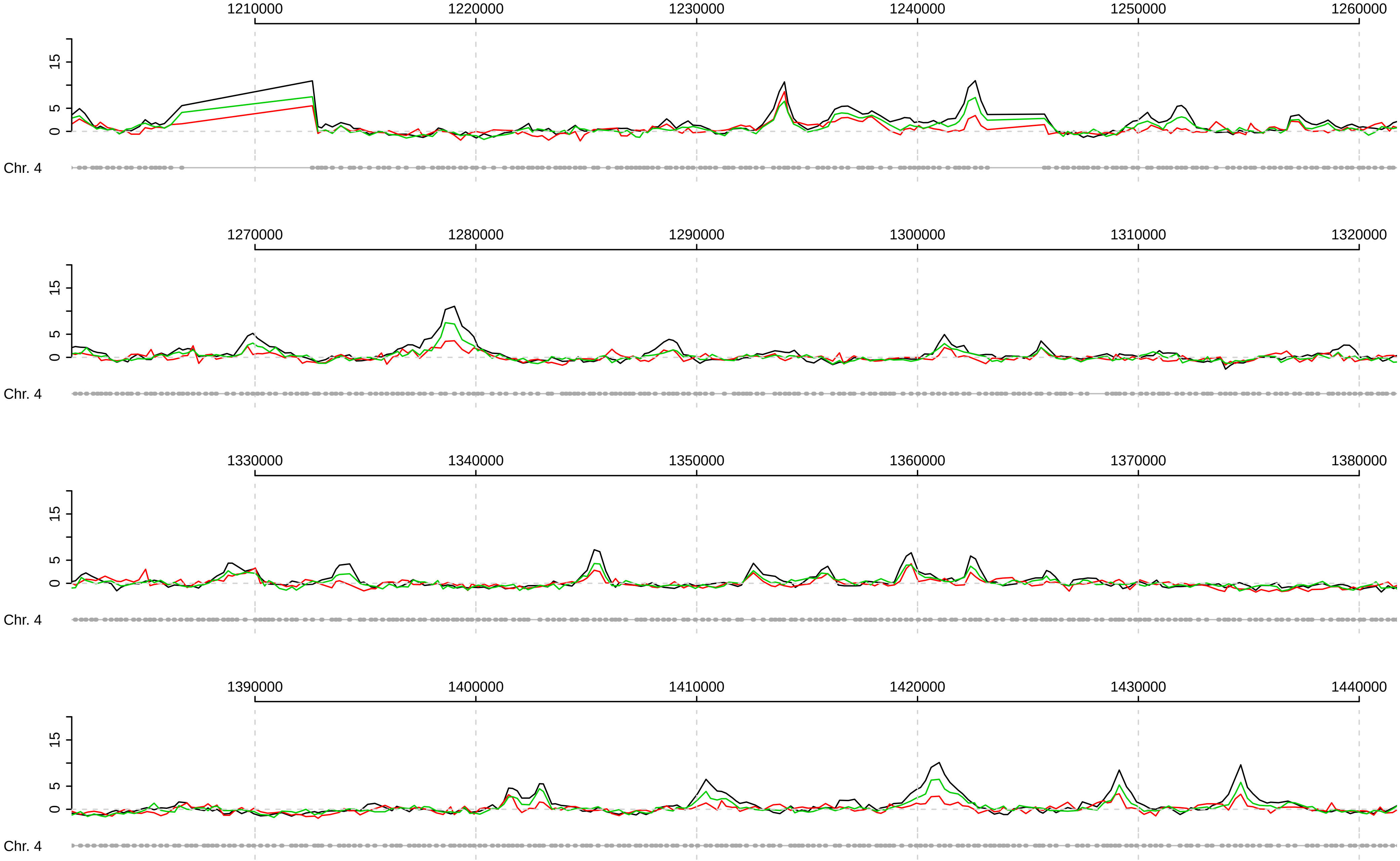
1210000
1220000
1230000
1240000
1250000
1260000
0
5
15
Chr. 4
1270000
1280000
1290000
1300000
1310000
1320000
0
5
15
Chr. 4
1330000
1340000
1350000
1360000
1370000
1380000
0
5
15
Chr. 4
1390000
1400000
1410000
1420000
1430000
1440000
0
5
15
Chr. 4

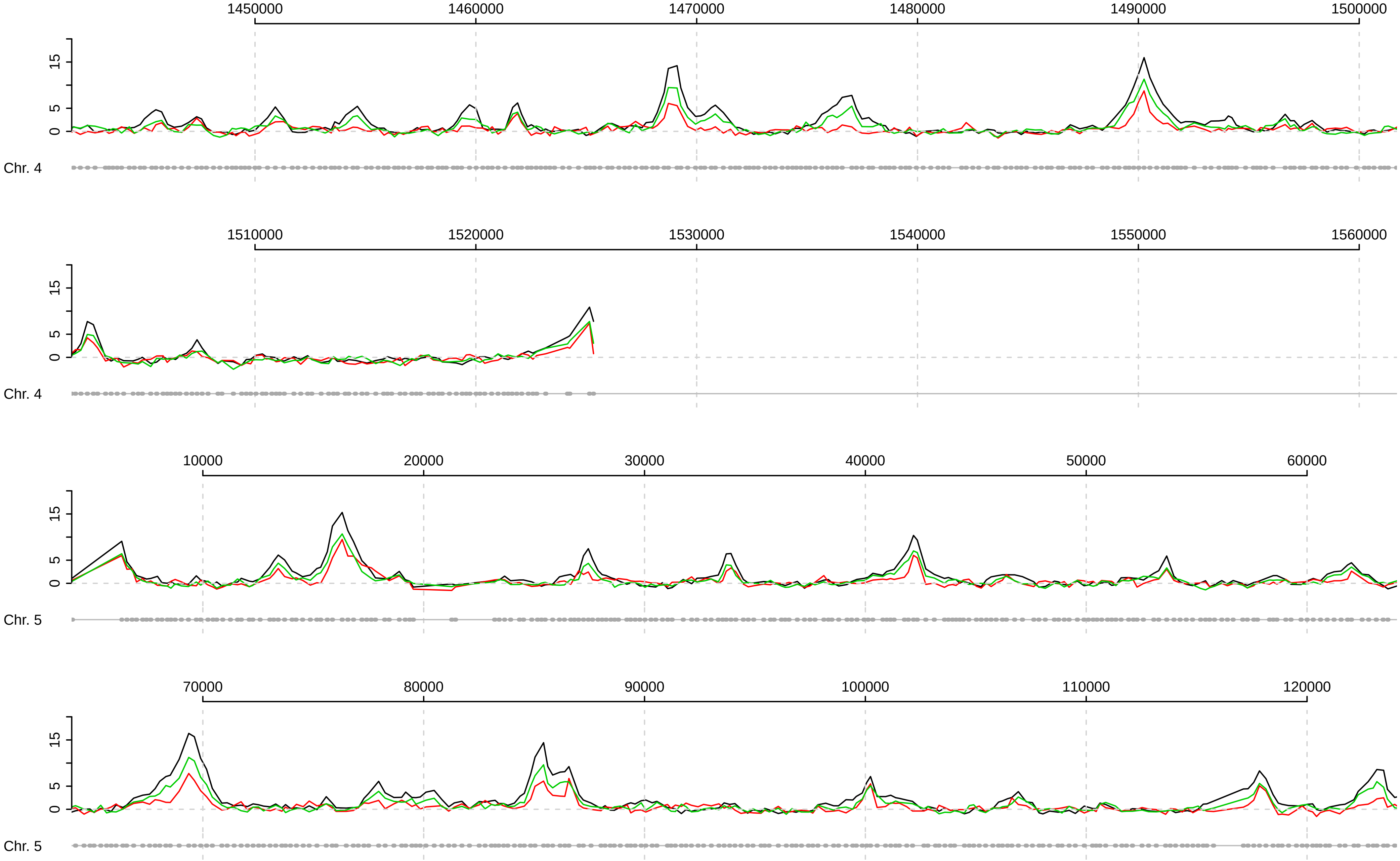
1450000
1460000
1470000
1480000
1490000
1500000
15
5
0
Chr. 4
1510000
1520000
1530000
1540000
1550000
1560000
15
5
0
Chr. 4
10000
20000
30000
40000
50000
60000
15
5
0
Chr. 5
70000
80000
90000
100000
110000
120000
15
5
0
Chr. 5

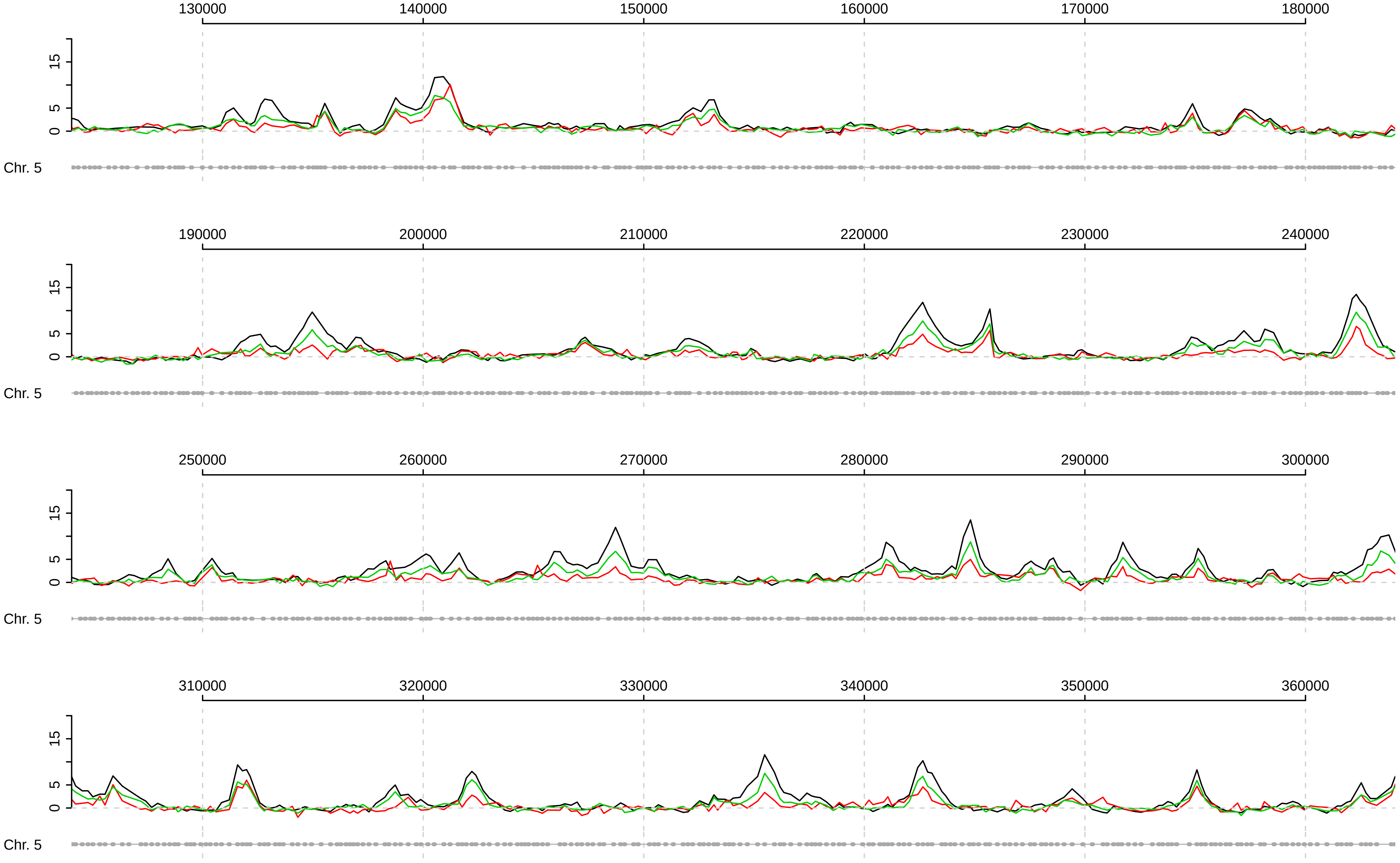

130000
140000
150000
160000
170000
180000
190000
200000
210000
220000
230000
240000
250000
260000
270000
280000
290000
300000
310000
320000
330000
340000
350000
360000
0
5
15
Chr. 5

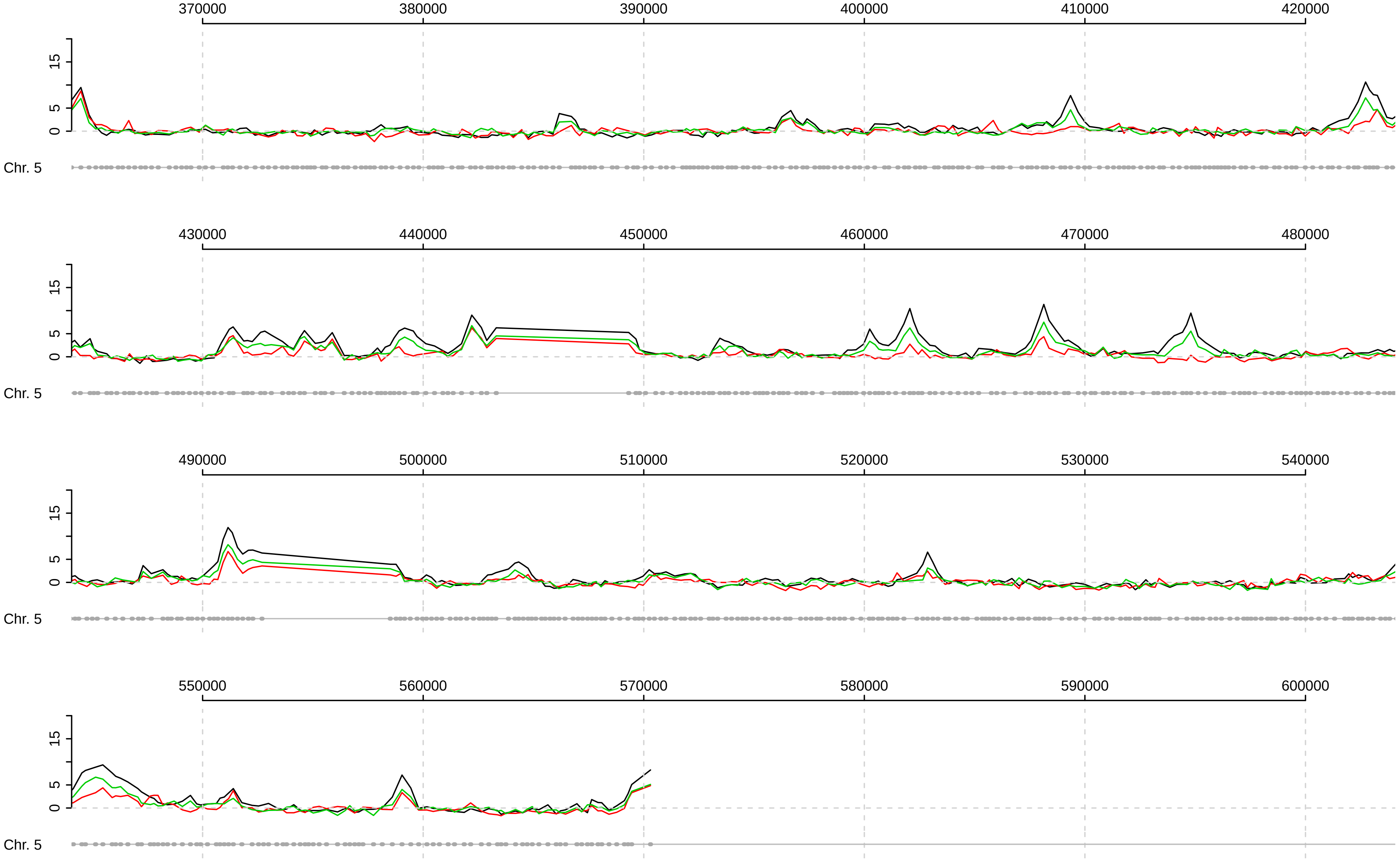

370000
380000
390000
400000
410000
420000
430000
440000
450000
460000
470000
480000
490000
500000
510000
520000
530000
540000
550000
560000
570000
580000
590000
600000
0
5
15
Chr. 5

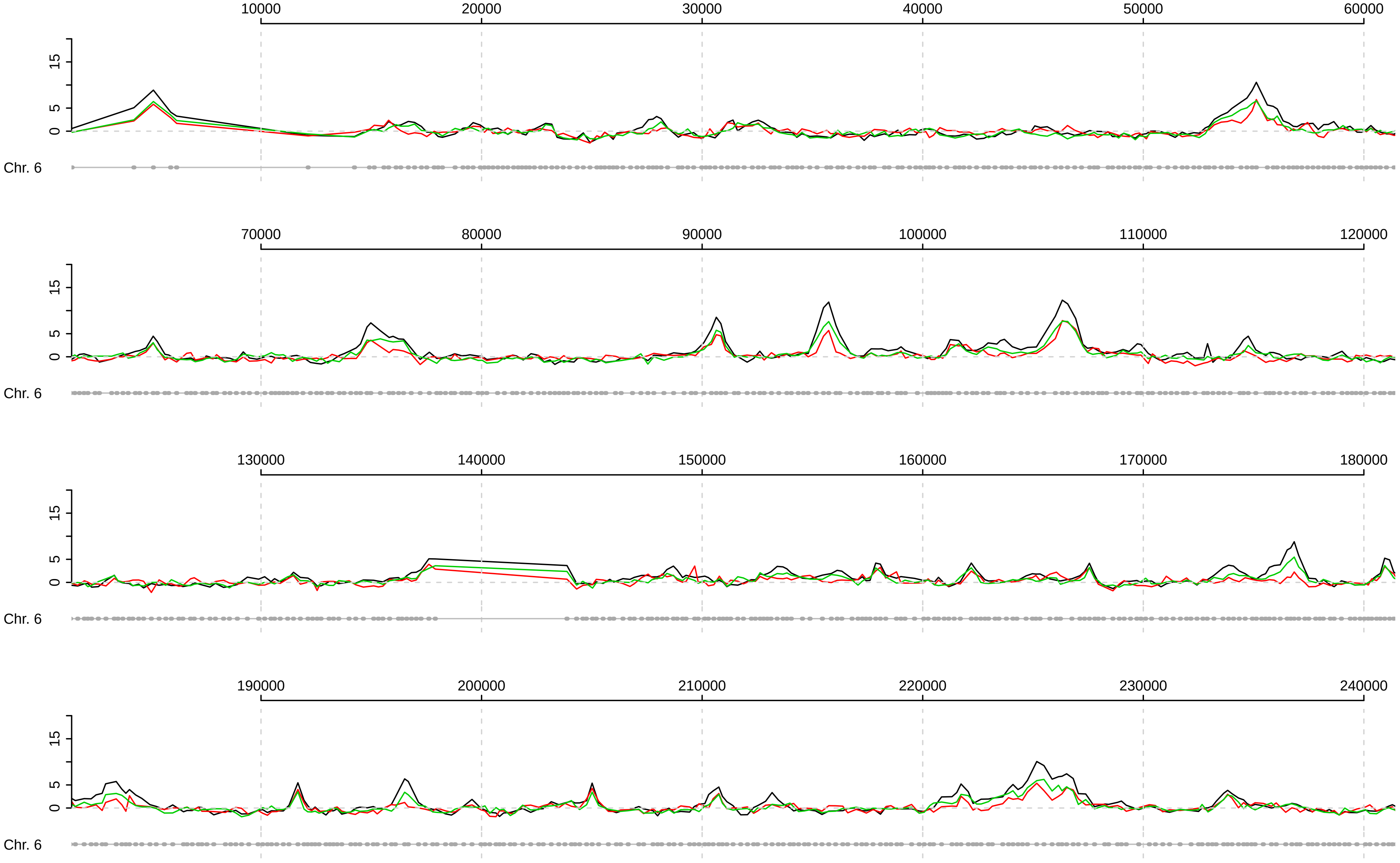

10000
20000
30000
40000
50000
60000
15
5
0
Chr. 6
70000
80000
90000
100000
110000
120000
15
5
0
Chr. 6
130000
140000
150000
160000
170000
180000
15
5
0
Chr. 6
190000
200000
210000
220000
230000
240000
15
5
0
Chr. 6

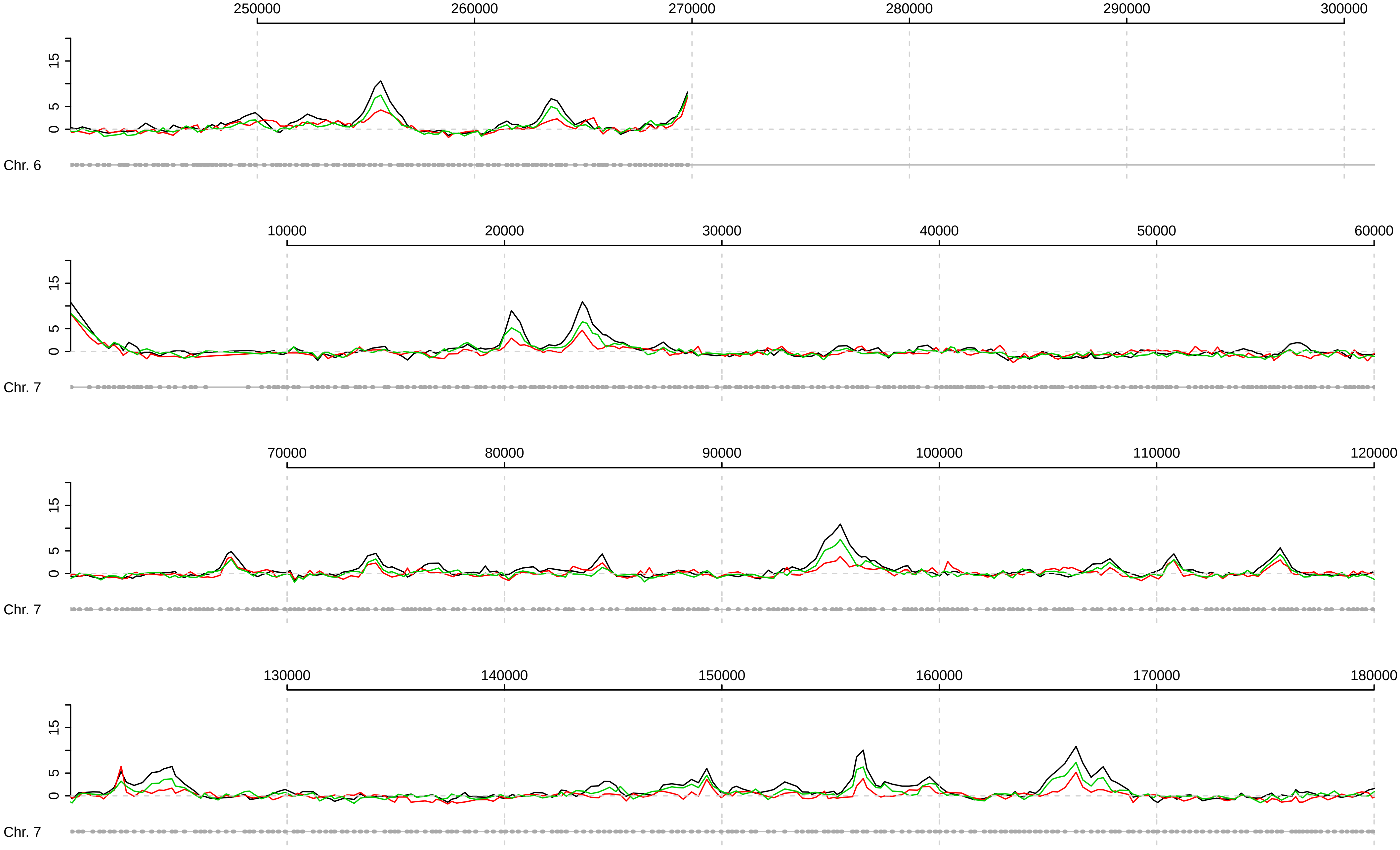
250000
260000
270000
280000
290000
300000
0
5
15
Chr. 6
10000
20000
30000
40000
50000
60000
0
5
15
Chr. 7
70000
80000
90000
100000
110000
120000
0
5
15
Chr. 7
130000
140000
150000
160000
170000
180000
0
5
15
Chr. 7

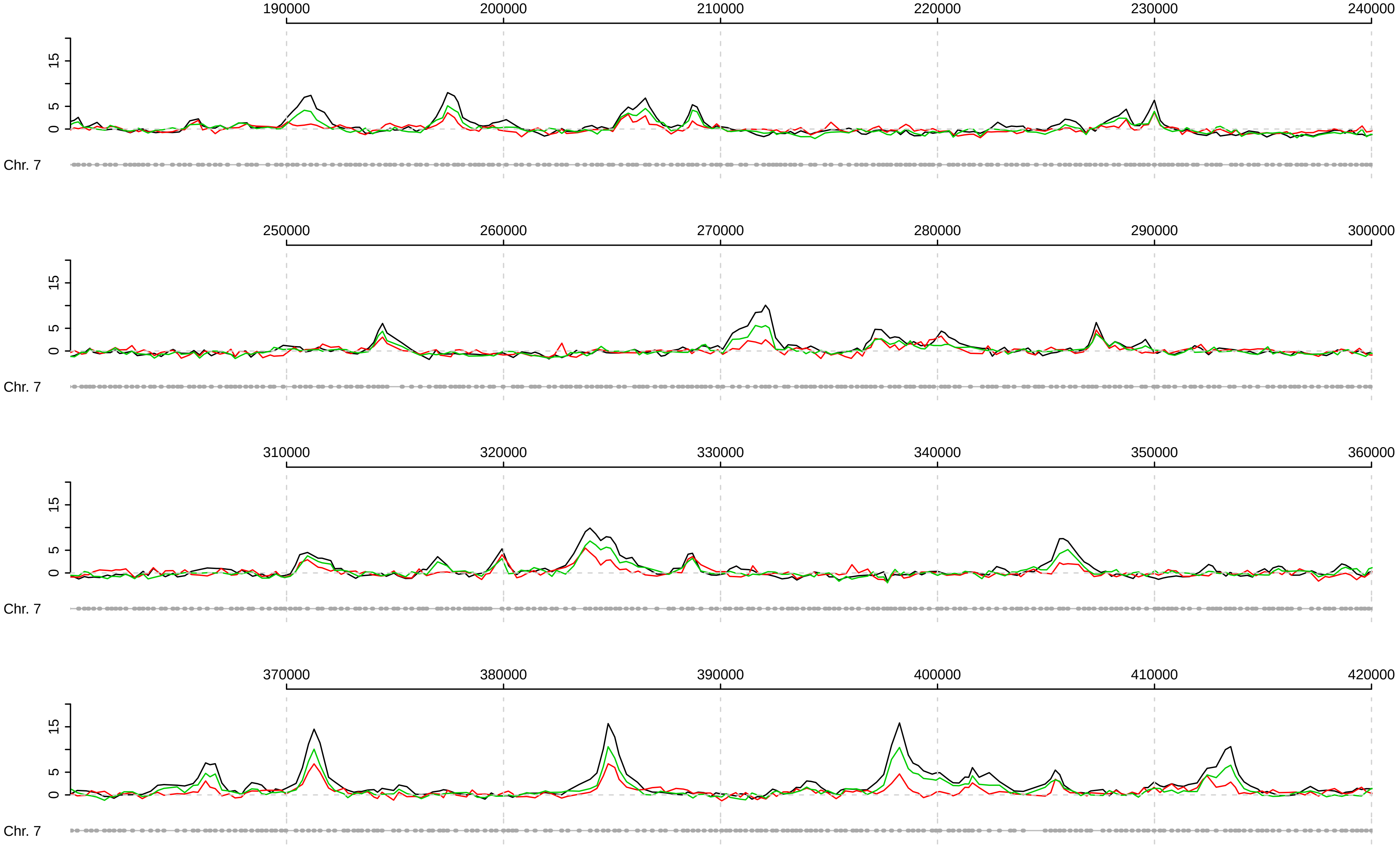
190000
200000
210000
220000
230000
240000
15
5
0
Chr. 7
250000
260000
270000
280000
290000
300000
15
5
0
Chr. 7
310000
320000
330000
340000
350000
360000
15
5
0
Chr. 7
370000
380000
390000
400000
410000
420000
15
5
0
Chr. 7

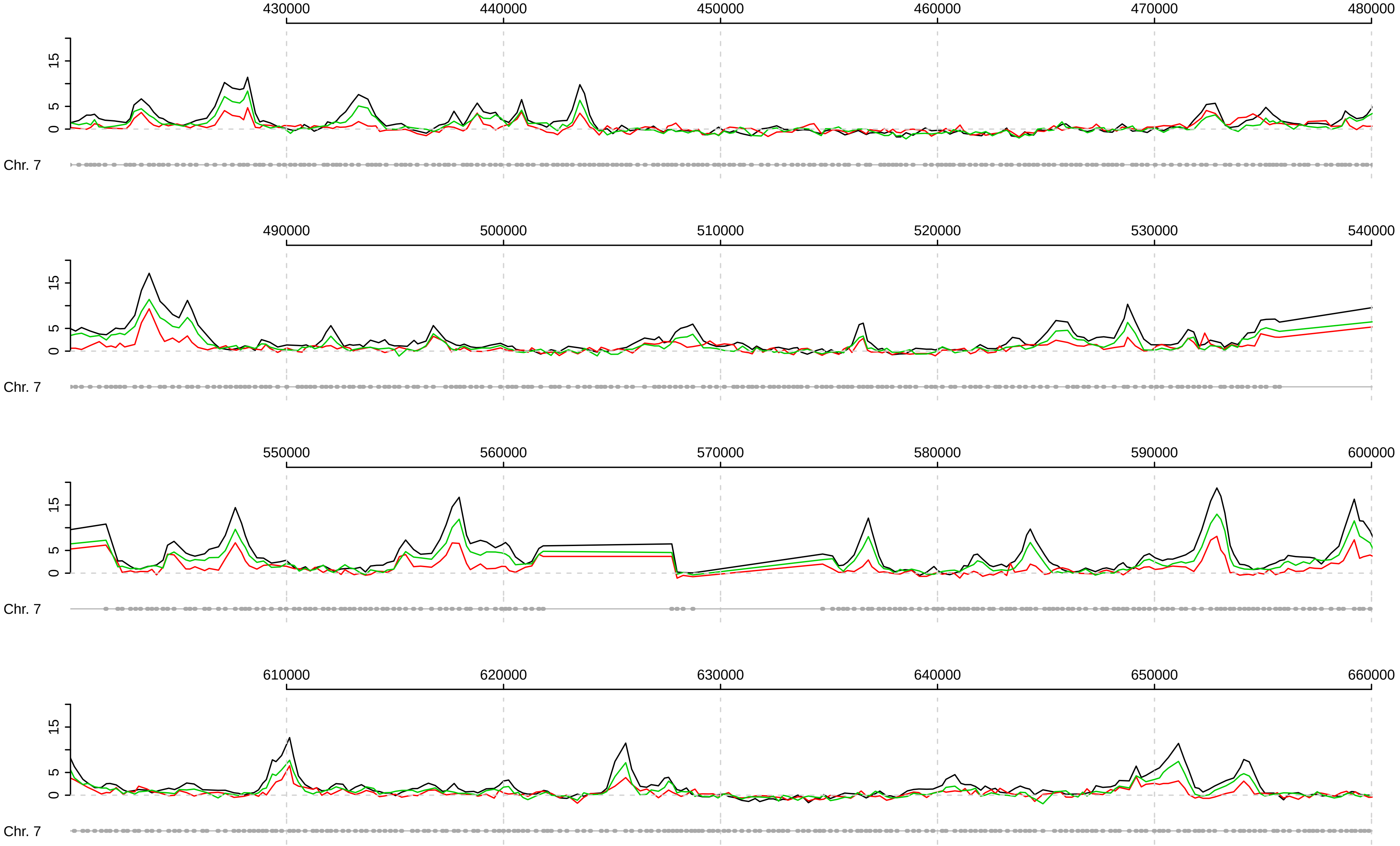

430000
440000
450000
460000
470000
480000
0
5
15
Chr. 7
490000
500000
510000
520000
530000
540000
0
5
15
Chr. 7
550000
560000
570000
580000
590000
600000
0
5
15
Chr. 7
610000
620000
630000
640000
650000
660000
0
5
15
Chr. 7

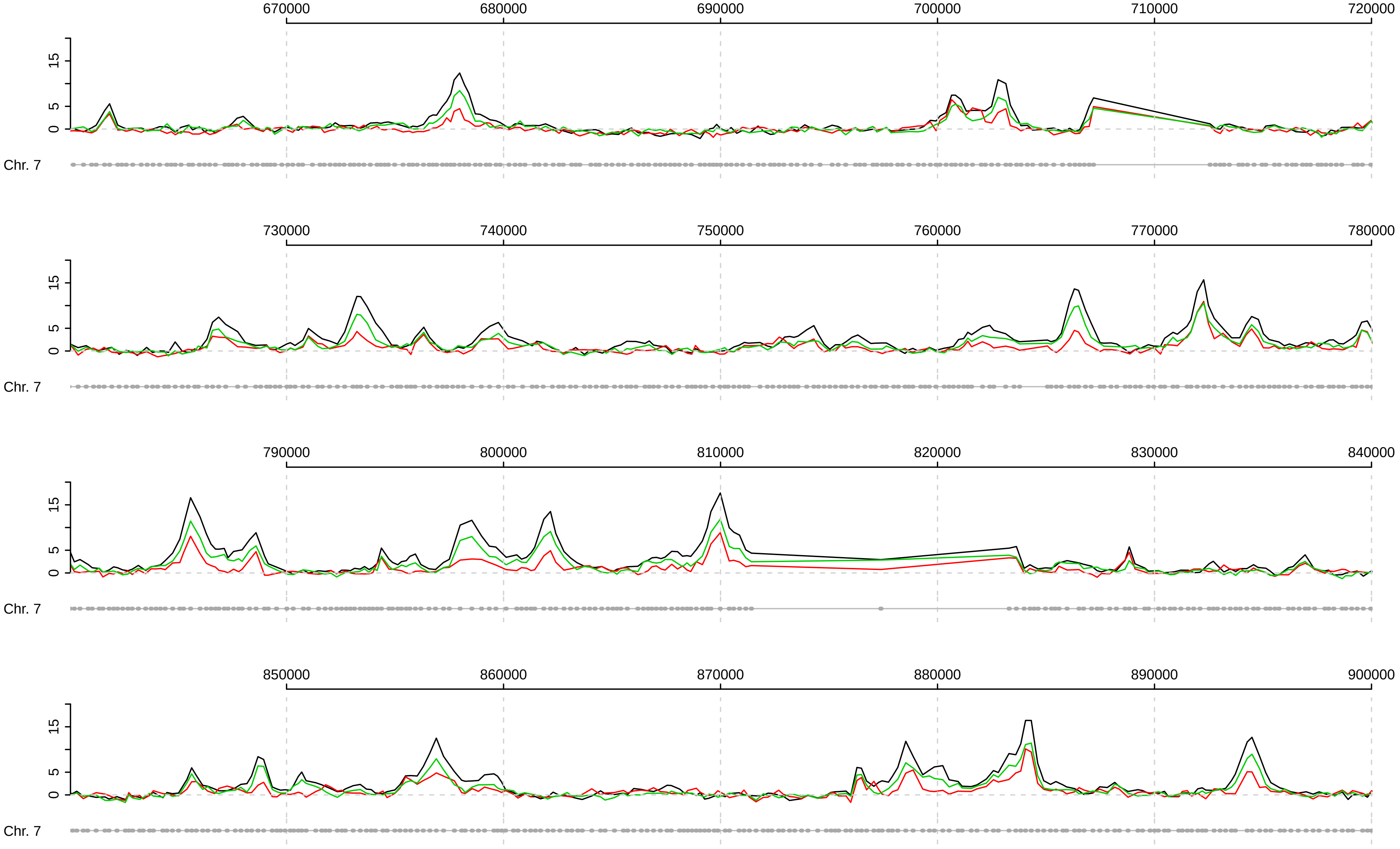

670000
680000
690000
700000
710000
720000
0
5
15
Chr. 7
730000
740000
750000
760000
770000
780000
0
5
15
Chr. 7
790000
800000
810000
820000
830000
840000
0
5
15
Chr. 7
850000
860000
870000
880000
890000
900000
0
5
15
Chr. 7

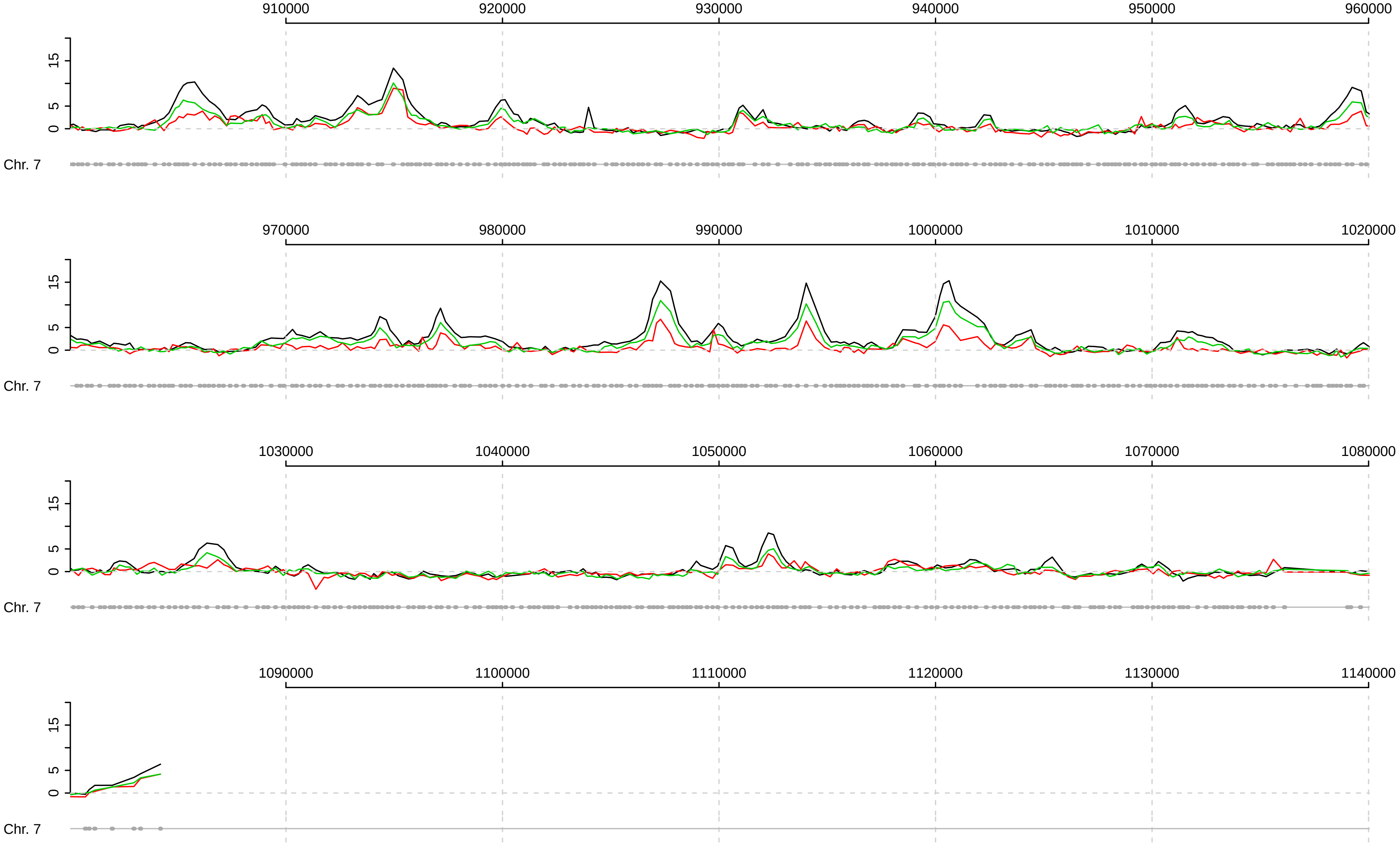

910000
920000
930000
940000
950000
960000
Chr. 7
970000
980000
990000
1000000
1010000
1020000
Chr. 7
1030000
1040000
1050000
1060000
1070000
1080000
Chr. 7
1090000
1100000
1110000
1120000
1130000
1140000
Chr. 7
0
5
15

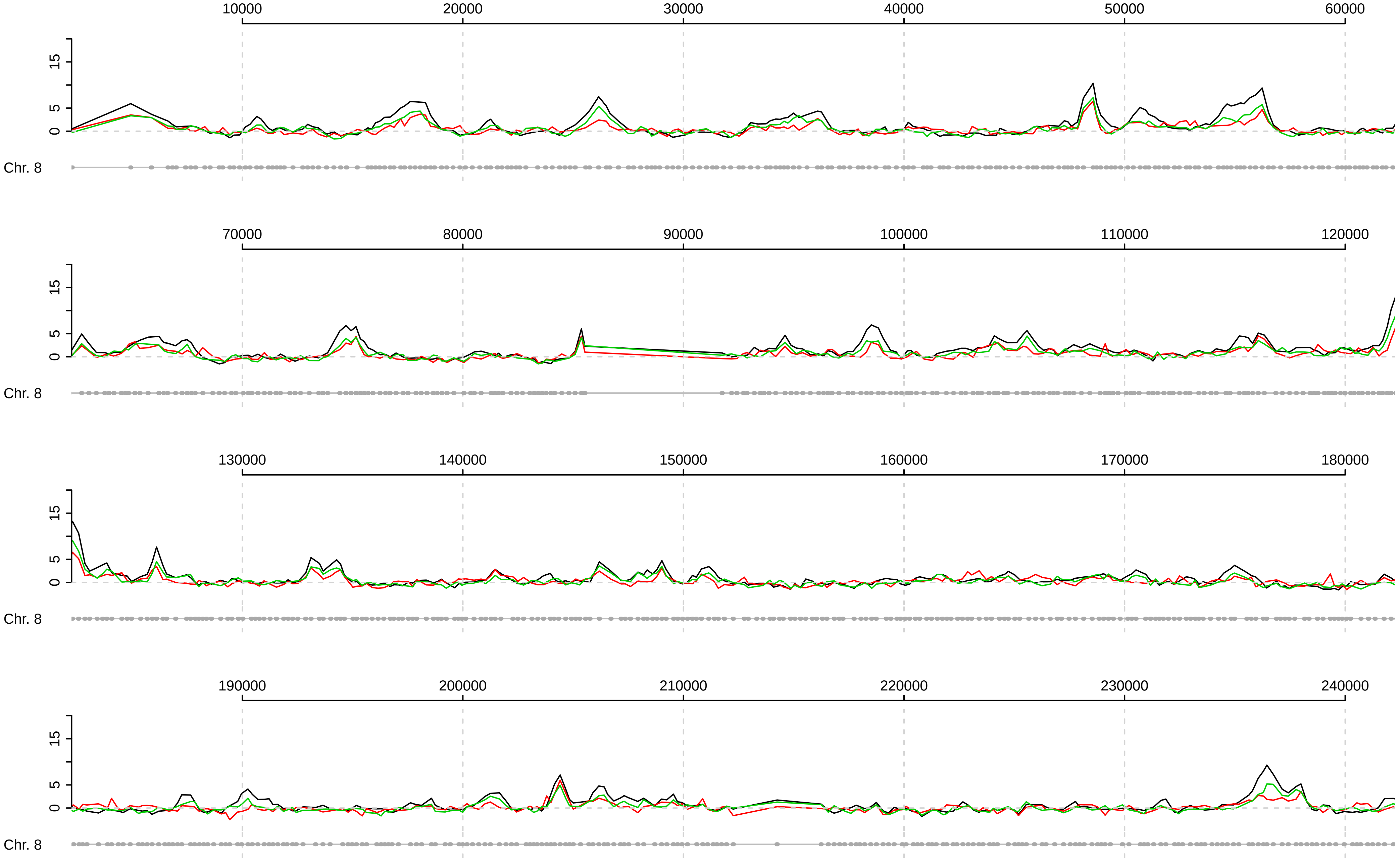

10000 20000 30000 40000 50000 60000
0 5 15
Chr. 8
70000 80000 90000 100000 110000 120000
0 5 15
Chr. 8
130000 140000 150000 160000 170000 180000
0 5 15
Chr. 8
190000 200000 210000 220000 230000 240000
0 5 15
Chr. 8

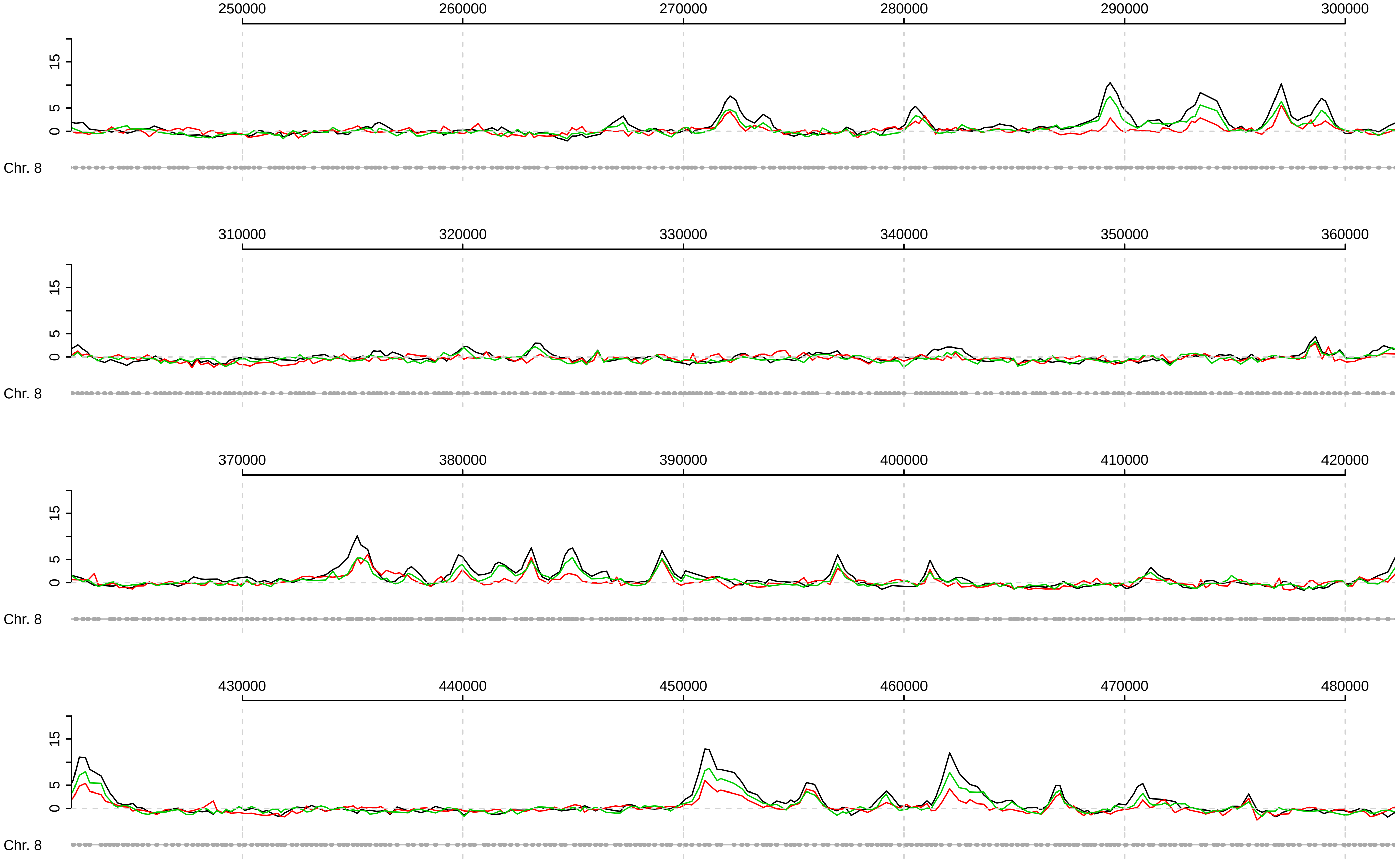
250000
260000
270000
280000
290000
300000
0
5
15
Chr. 8
310000
320000
330000
340000
350000
360000
0
5
15
Chr. 8
370000
380000
390000
400000
410000
420000
0
5
15
Chr. 8
430000
440000
450000
460000
470000
480000
0
5
15
Chr. 8

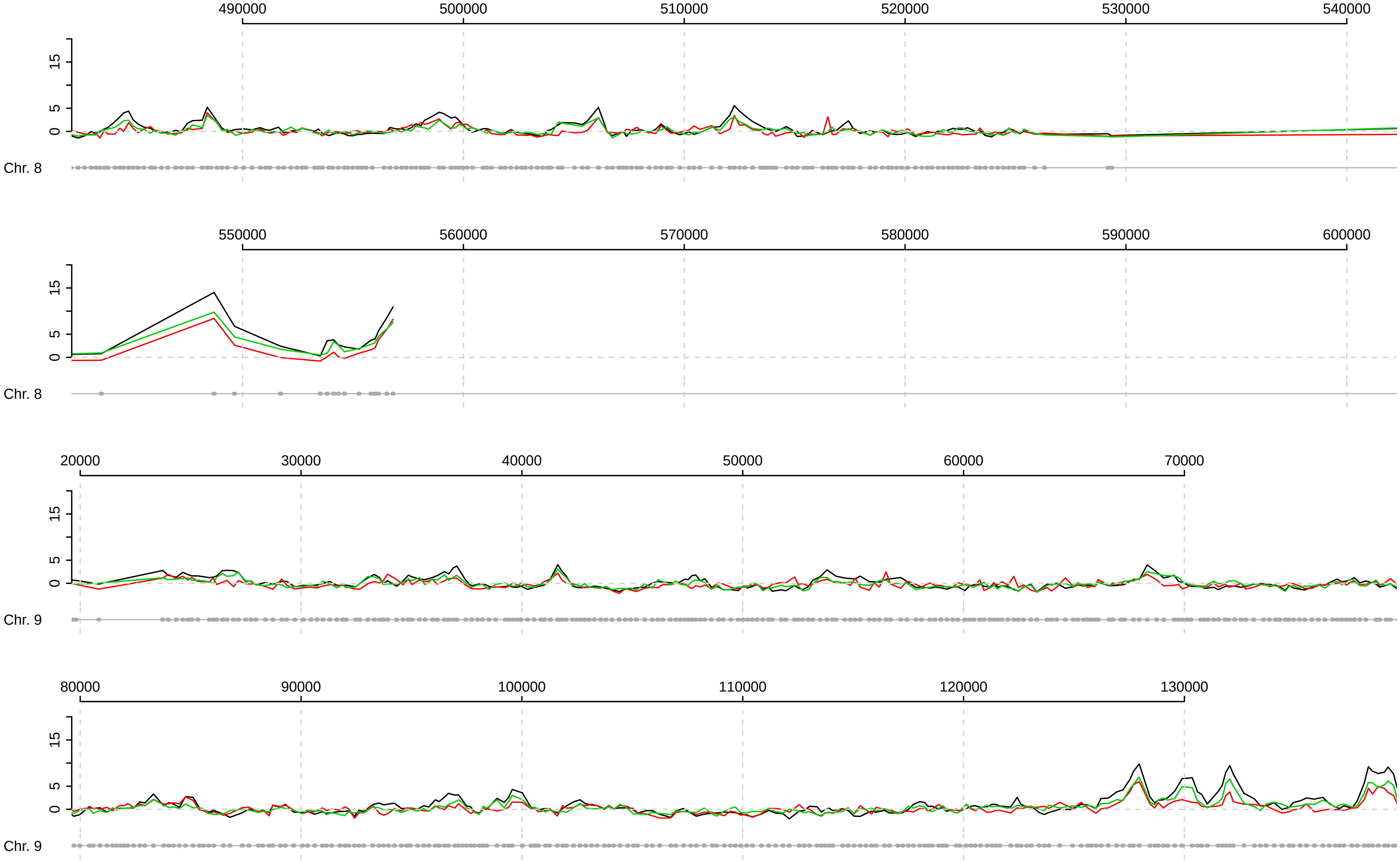
490000
500000
510000
520000
530000
540000
15
5
0
Chr. 8
550000
560000
570000
580000
590000
600000
15
5
0
Chr. 8
20000
30000
40000
50000
60000
70000
15
5
0
Chr. 9
80000
90000
100000
110000
120000
130000
15
5
0
Chr. 9

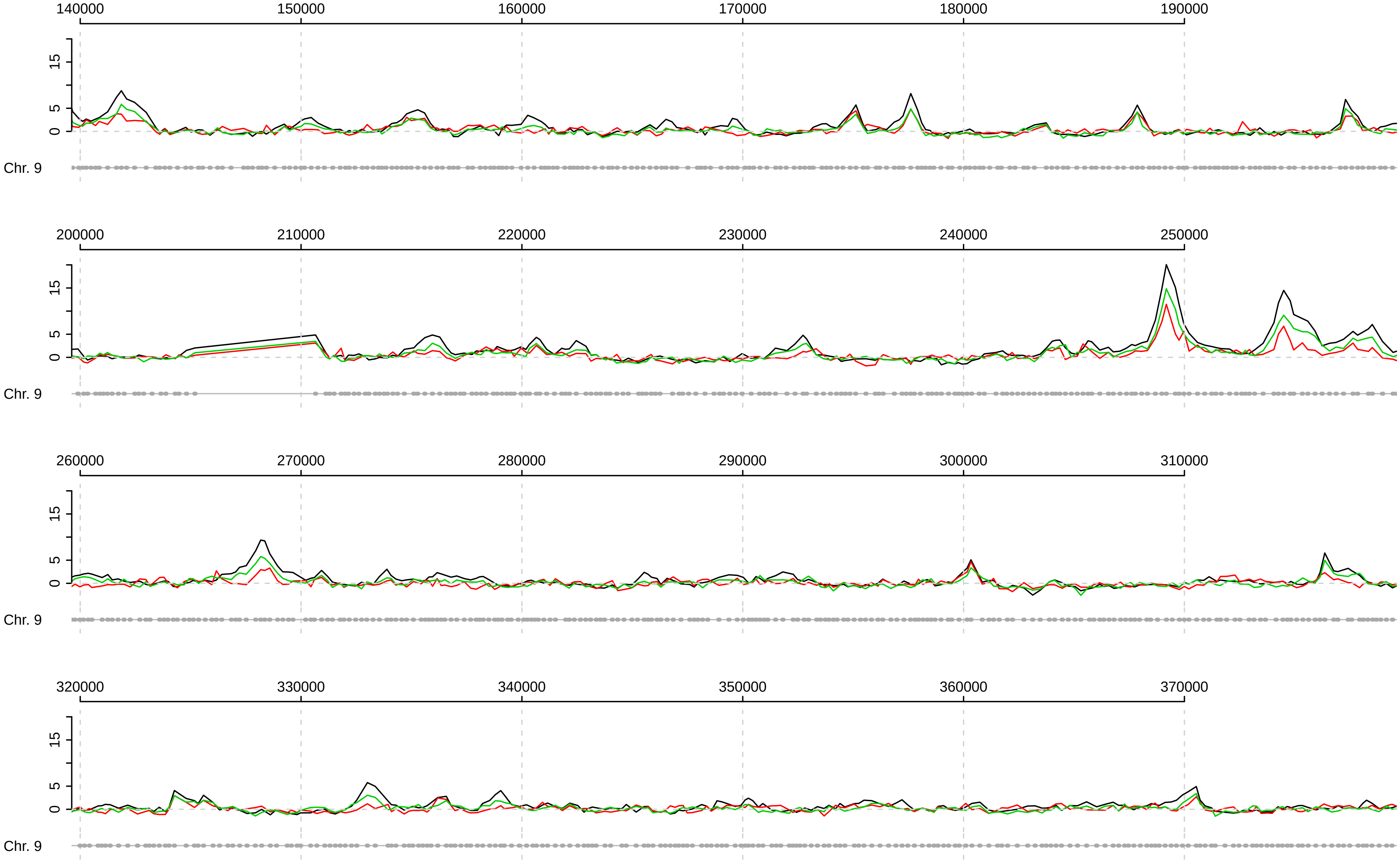

140000
150000
160000
170000
180000
190000
0
5
15
Chr. 9
200000
210000
220000
230000
240000
250000
0
5
15
Chr. 9
260000
270000
280000
290000
300000
310000
0
5
15
Chr. 9
320000
330000
340000
350000
360000
370000
0
5
15
Chr. 9

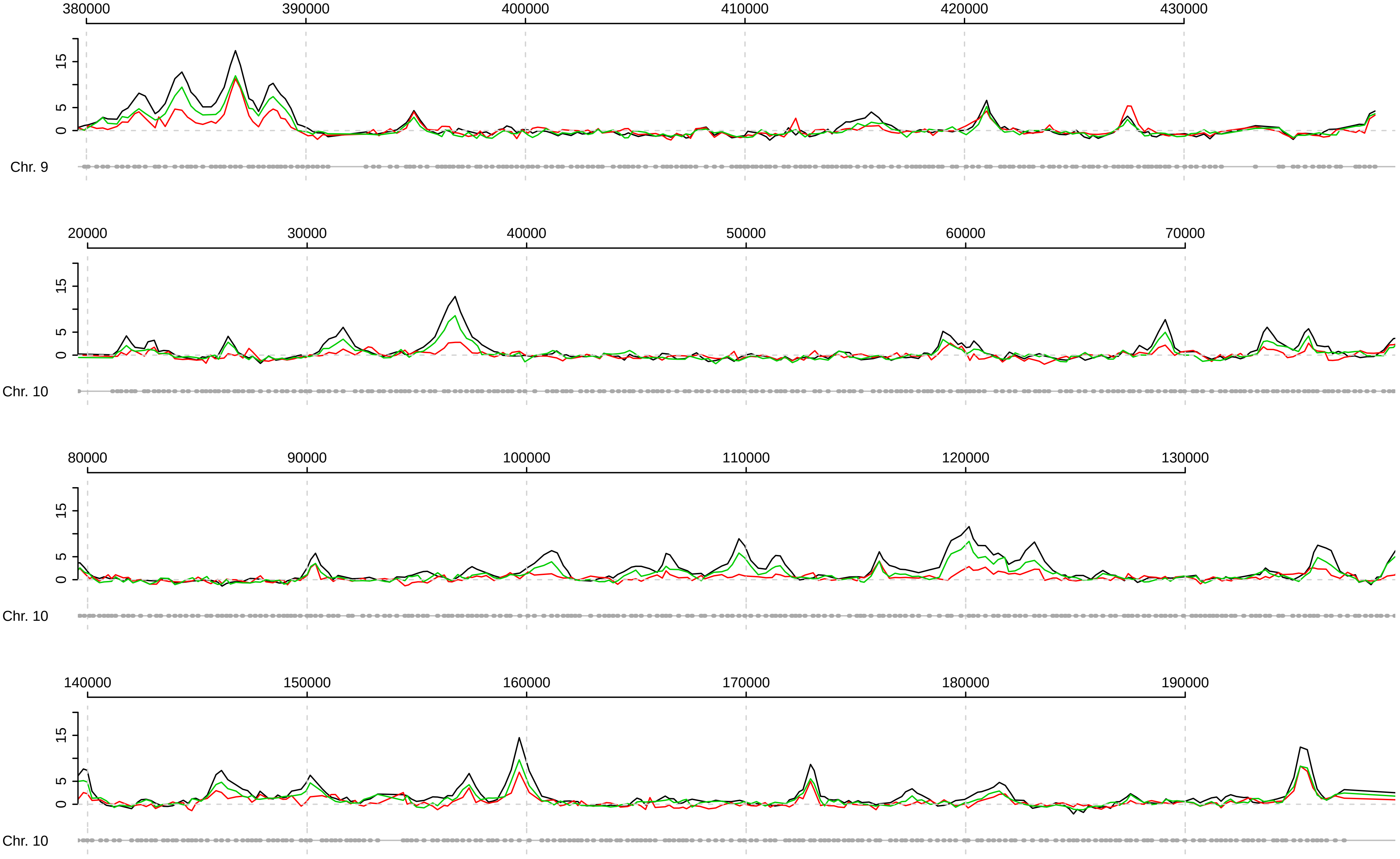
380000
390000
400000
410000
420000
430000
0
5
15
Chr. 9
20000
30000
40000
50000
60000
70000
0
5
15
Chr. 10
80000
90000
100000
110000
120000
130000
0
5
15
Chr. 10
140000
150000
160000
170000
180000
190000
0
5
15
Chr. 10

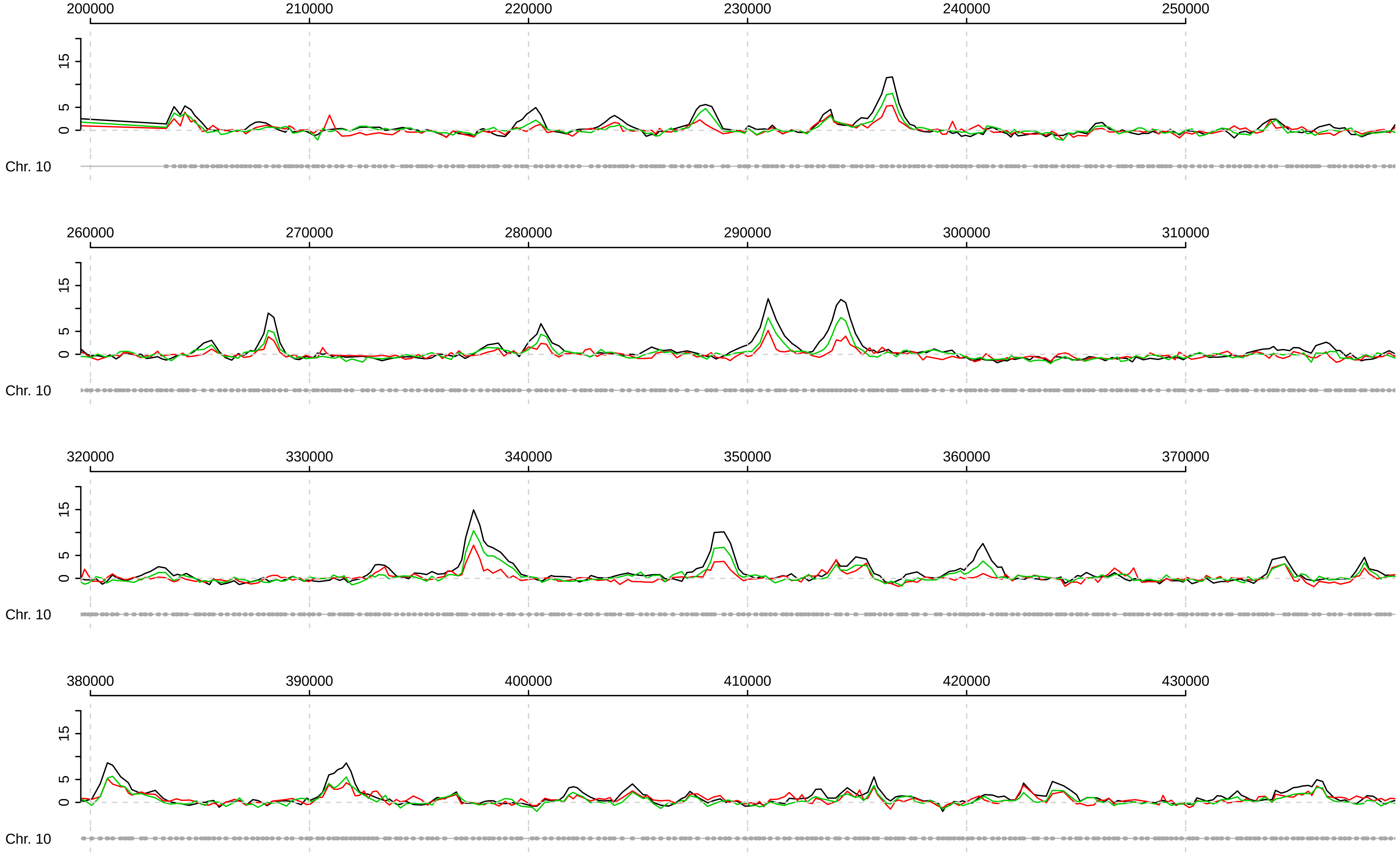
200000
210000
220000
230000
240000
250000
15
5
0
Chr. 10
260000
270000
280000
290000
300000
310000
15
5
0
Chr. 10
320000
330000
340000
350000
360000
370000
15
5
0
Chr. 10
380000
390000
400000
410000
420000
430000
15
5
0
Chr. 10

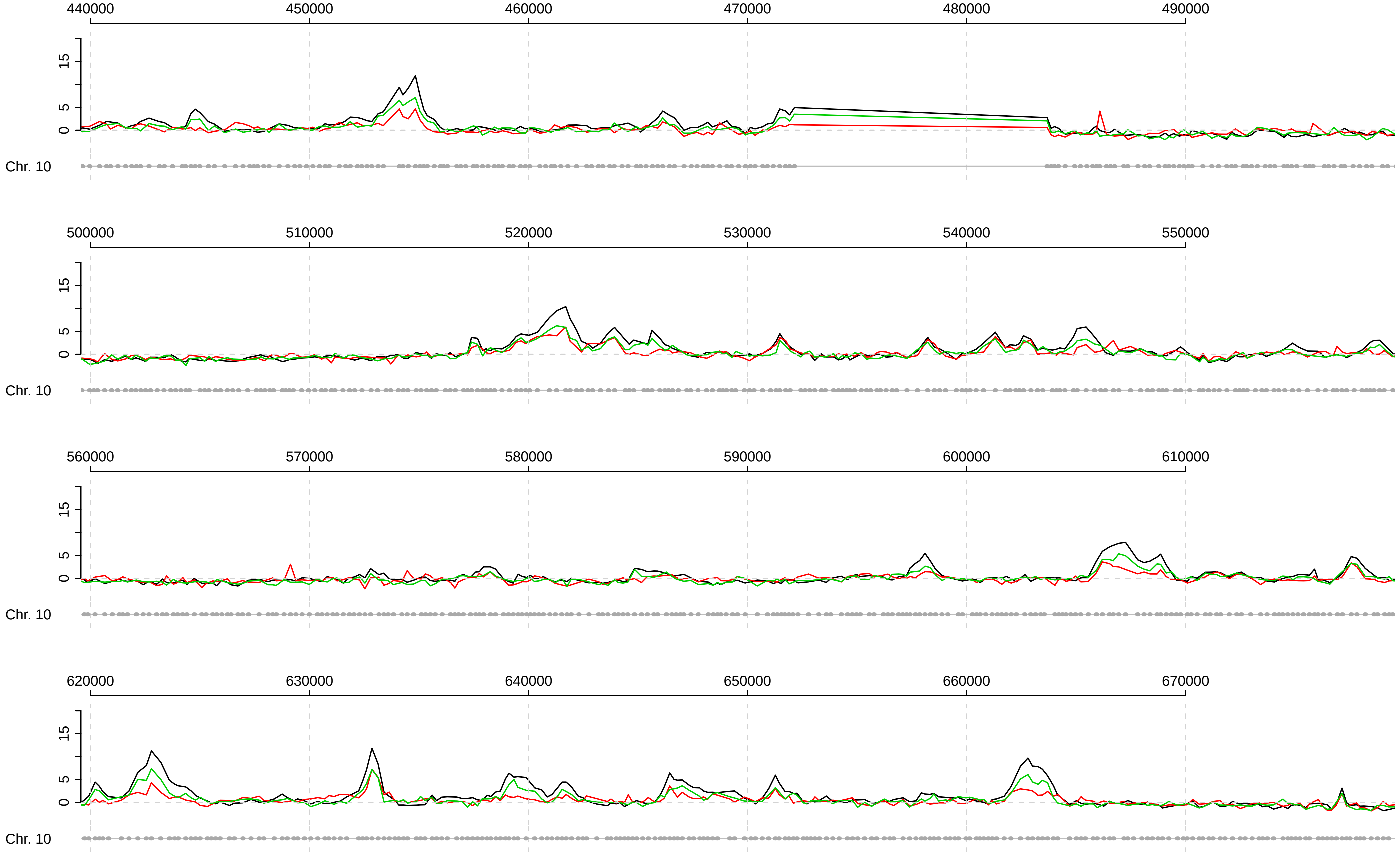

440000
450000
460000
470000
480000
490000
0
5
15
Chr. 10
500000
510000
520000
530000
540000
550000
0
5
15
Chr. 10
560000
570000
580000
590000
600000
610000
0
5
15
Chr. 10
620000
630000
640000
650000
660000
670000
0
5
15
Chr. 10

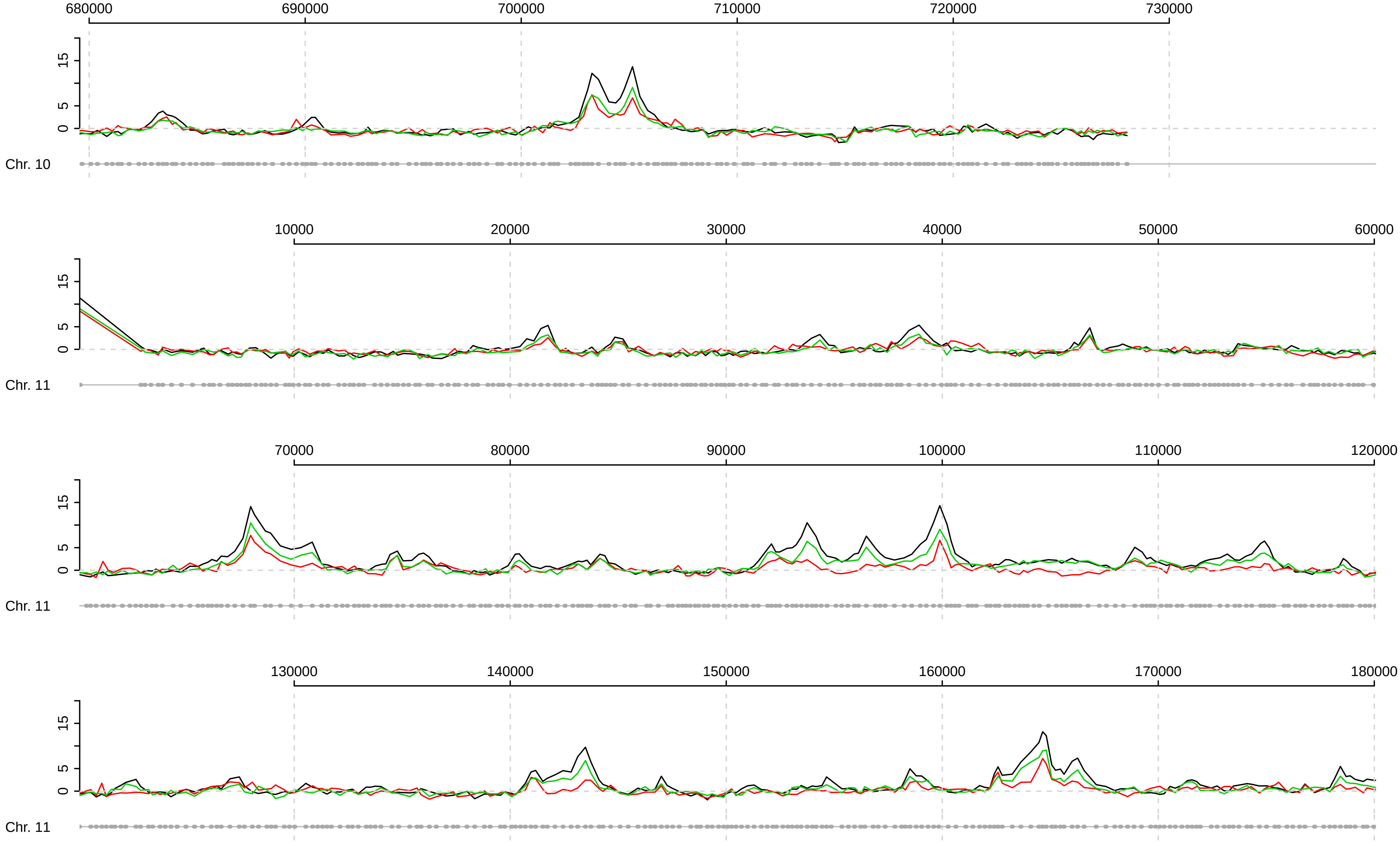

680000
690000
700000
710000
720000
730000
15
5
0
Chr. 10
10000
20000
30000
40000
50000
60000
15
5
0
Chr. 11
70000
80000
90000
100000
110000
120000
15
5
0
Chr. 11
130000
140000
150000
160000
170000
180000
15
5
0
Chr. 11

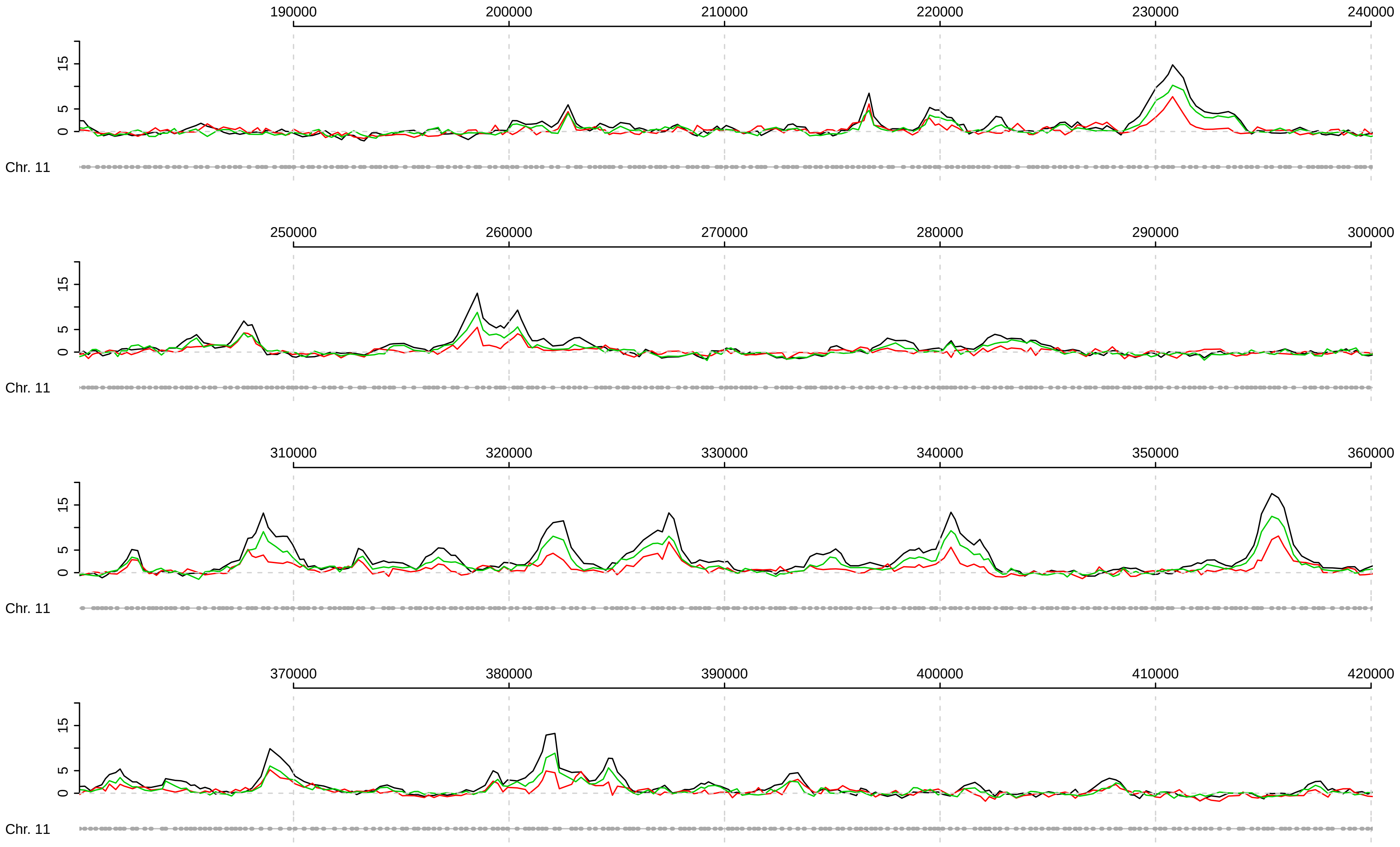
190000
200000
210000
220000
230000
240000
15
5
0
Chr. 11
250000
260000
270000
280000
290000
300000
15
5
0
Chr. 11
310000
320000
330000
340000
350000
360000
15
5
0
Chr. 11
370000
380000
390000
400000
410000
420000
15
5
0
Chr. 11

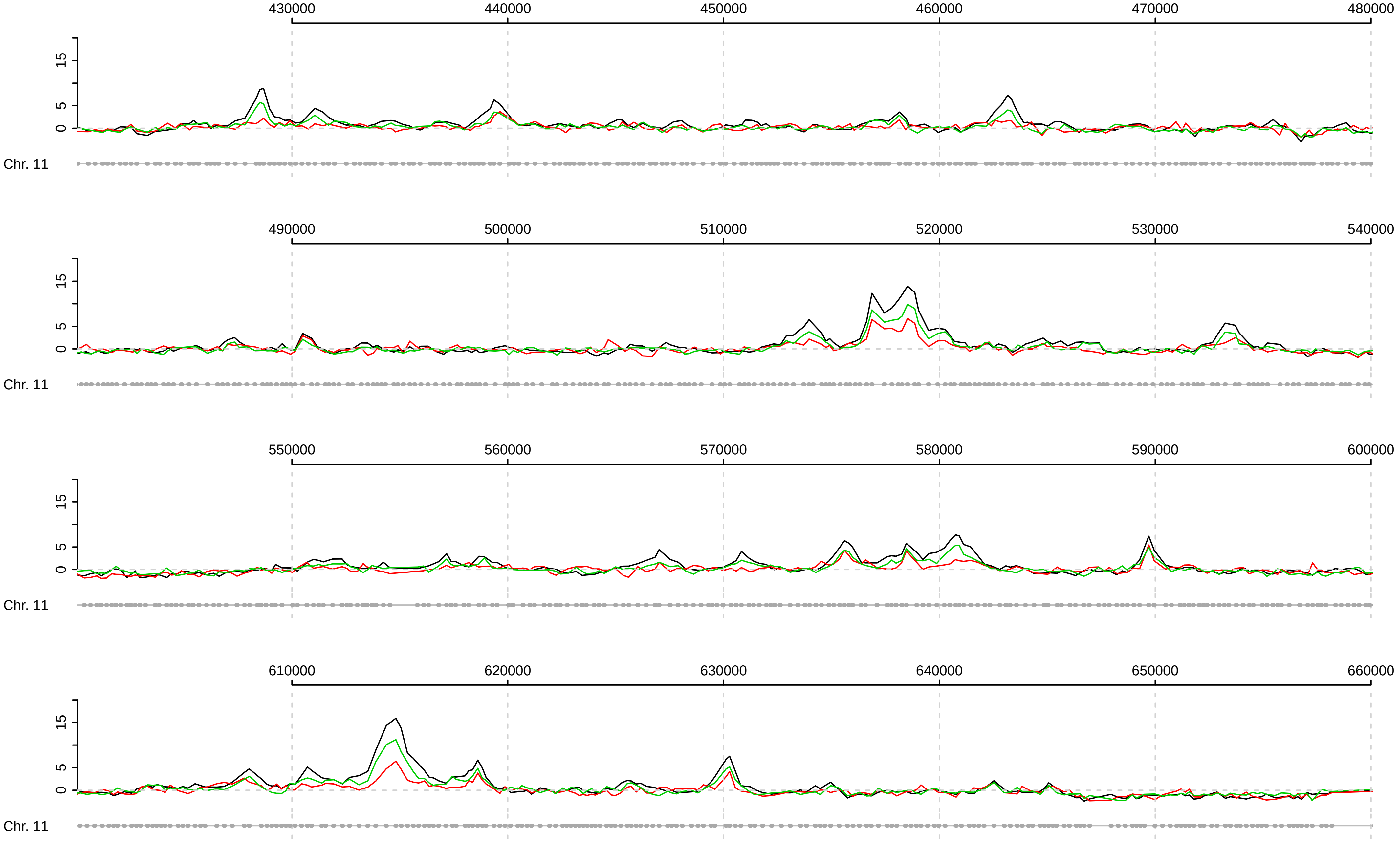
430000
440000
450000
460000
470000
480000
15
5
0
Chr. 11
490000
500000
510000
520000
530000
540000
15
5
0
Chr. 11
550000
560000
570000
580000
590000
600000
15
5
0
Chr. 11
610000
620000
630000
640000
650000
660000
15
5
0
Chr. 11

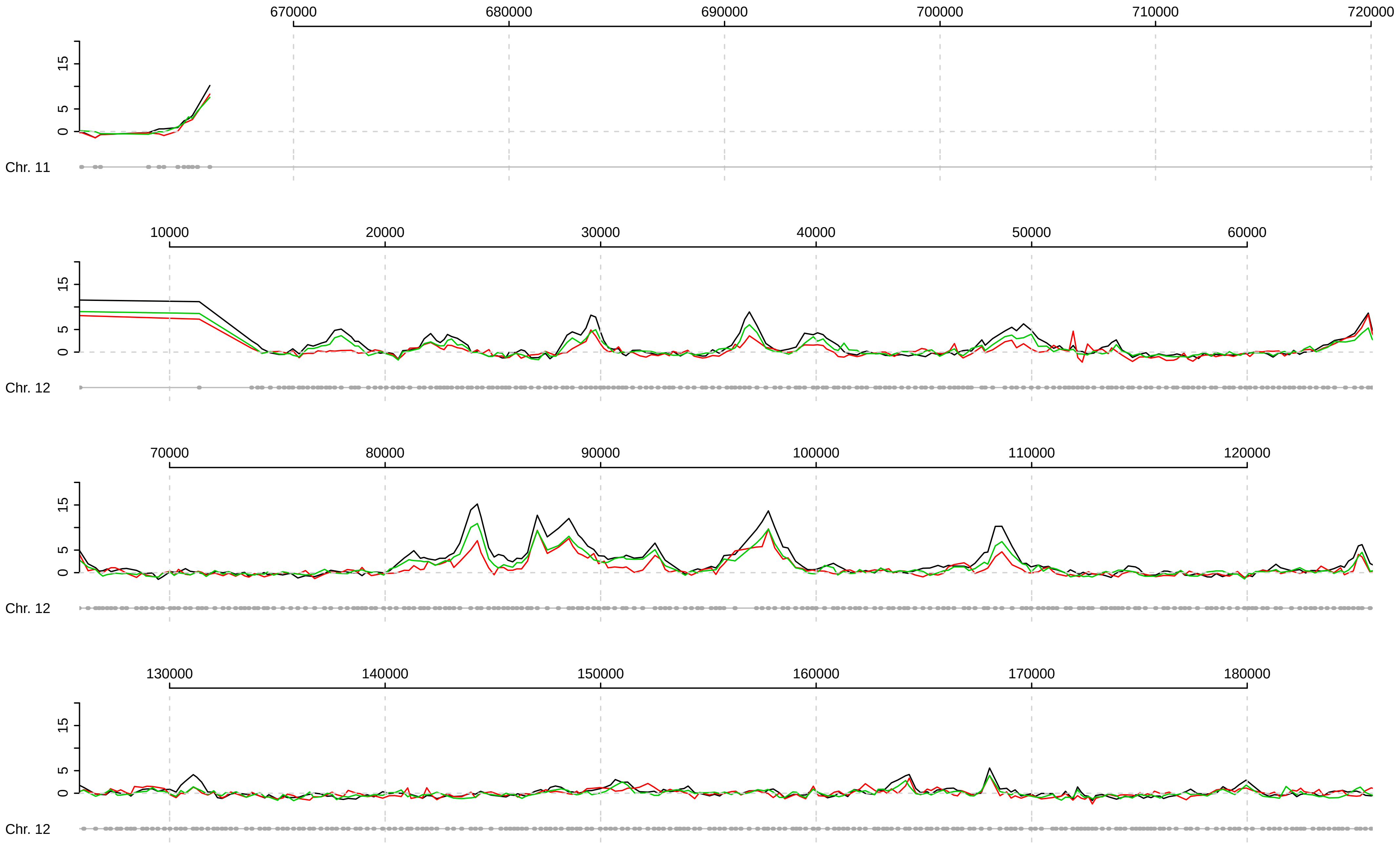
670000
680000
690000
700000
710000
720000
0
5
15
Chr. 11
10000
20000
30000
40000
50000
60000
Chr. 12
70000
80000
90000
100000
110000
120000
Chr. 12
130000
140000
150000
160000
170000
180000
Chr. 12

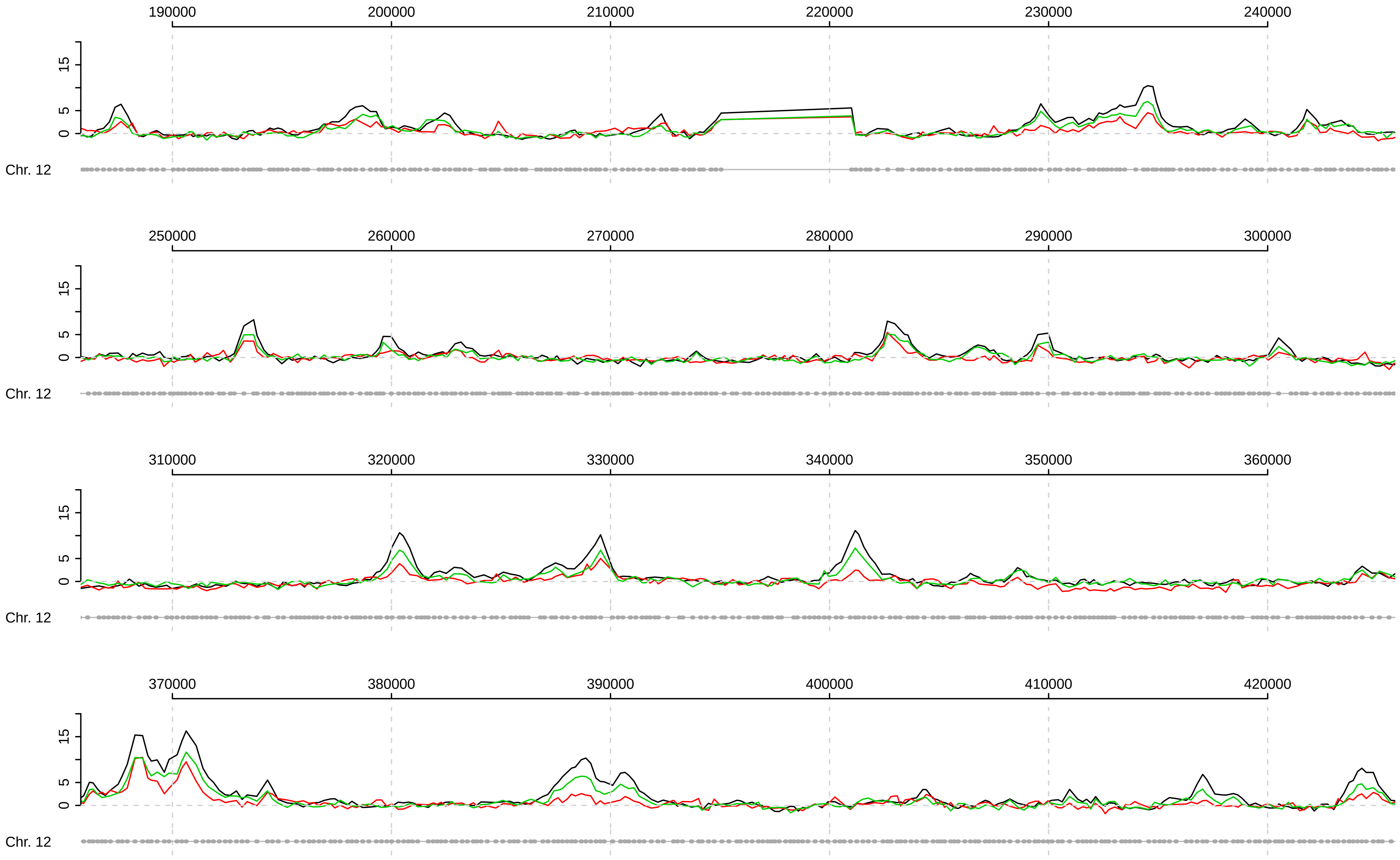

190000
200000
210000
220000
230000
240000
0
5
15
Chr. 12
250000
260000
270000
280000
290000
300000
0
5
15
Chr. 12
310000
320000
330000
340000
350000
360000
0
5
15
Chr. 12
370000
380000
390000
400000
410000
420000
0
5
15
Chr. 12

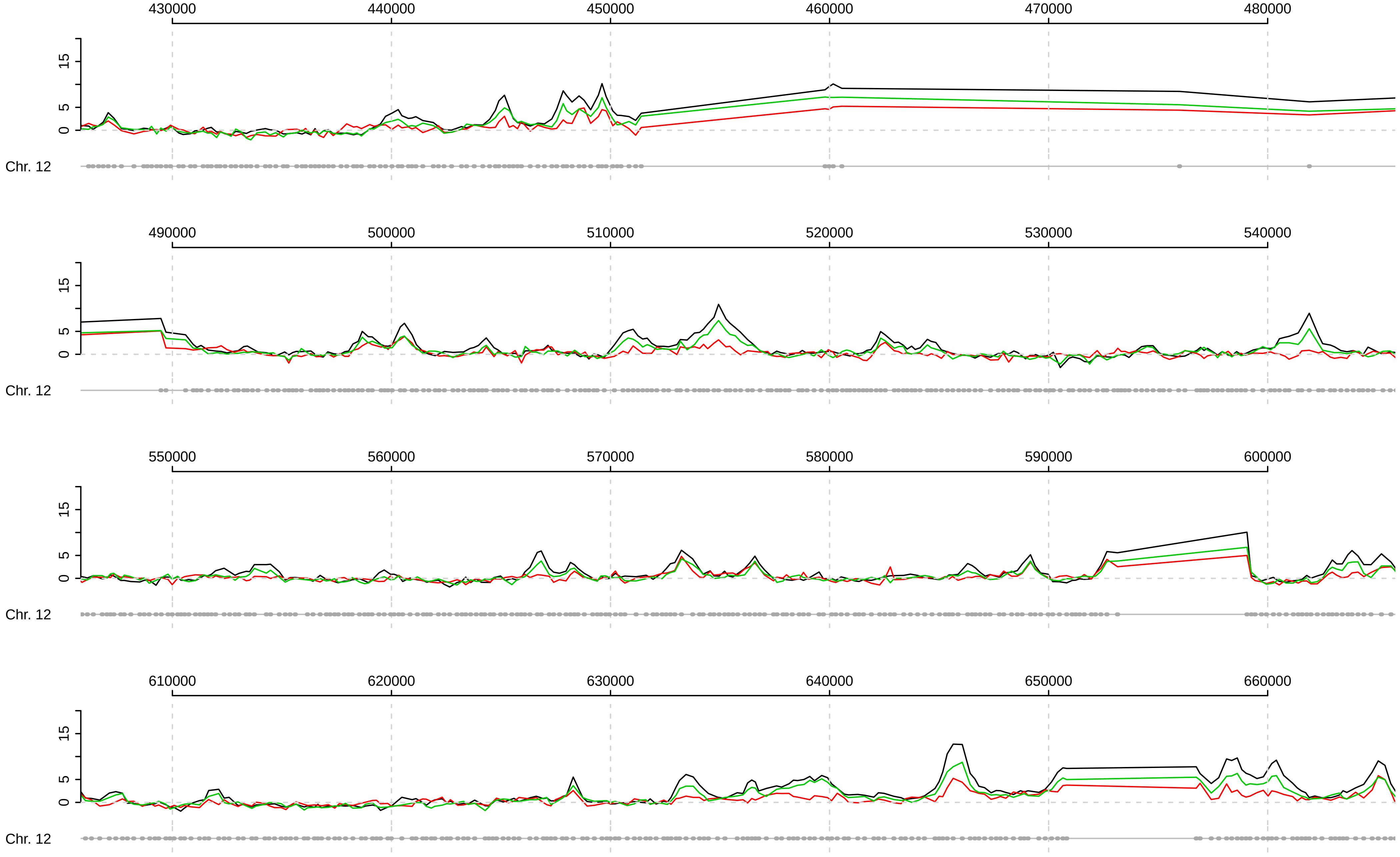

430000
440000
450000
460000
470000
480000
0
5
15
Chr. 12
490000
500000
510000
520000
530000
540000
0
5
15
Chr. 12
550000
560000
570000
580000
590000
600000
0
5
15
Chr. 12
610000
620000
630000
640000
650000
660000
0
5
15
Chr. 12

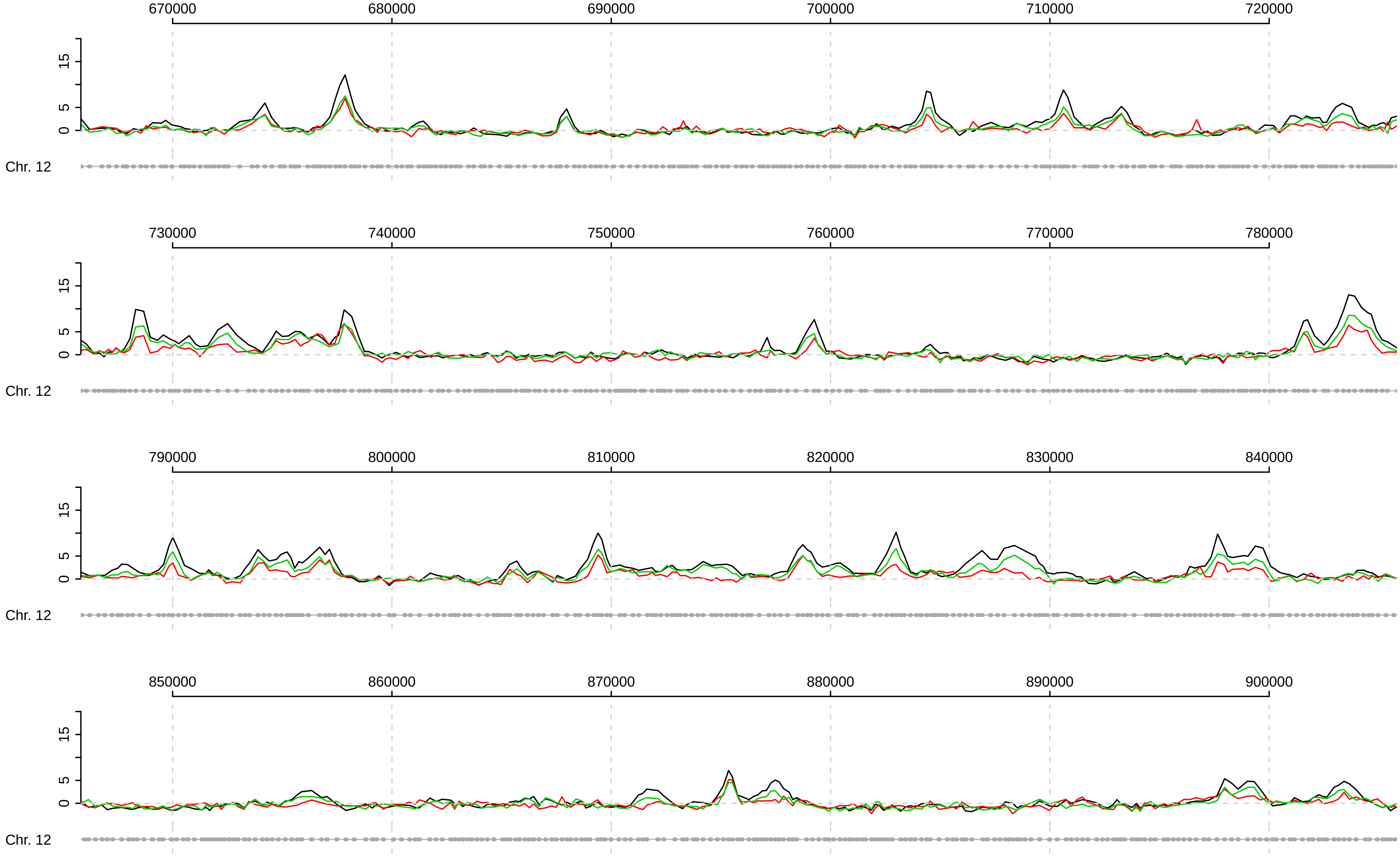
670000
680000
690000
700000
710000
720000
15
5
0
Chr. 12
730000
740000
750000
760000
770000
780000
15
5
0
Chr. 12
790000
800000
810000
820000
830000
840000
15
5
0
Chr. 12
850000
860000
870000
880000
890000
900000
15
5
0
Chr. 12

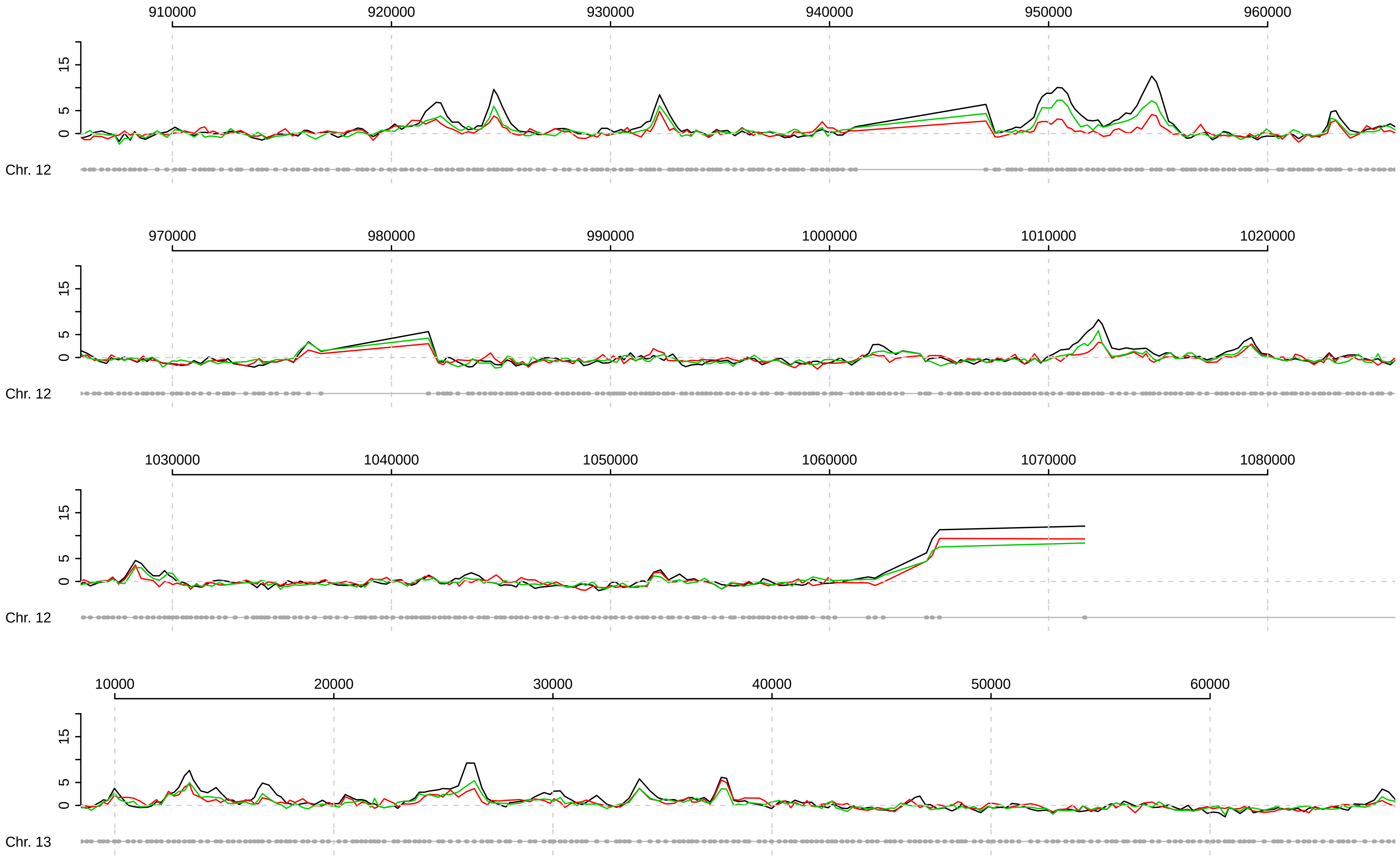
910000
920000
930000
940000
950000
960000
15
5
0
Chr. 12
970000
980000
990000
1000000
1010000
1020000
15
5
0
Chr. 12
1030000
1040000
1050000
1060000
1070000
1080000
15
5
0
Chr. 12
10000
20000
30000
40000
50000
60000
15
5
0
Chr. 13

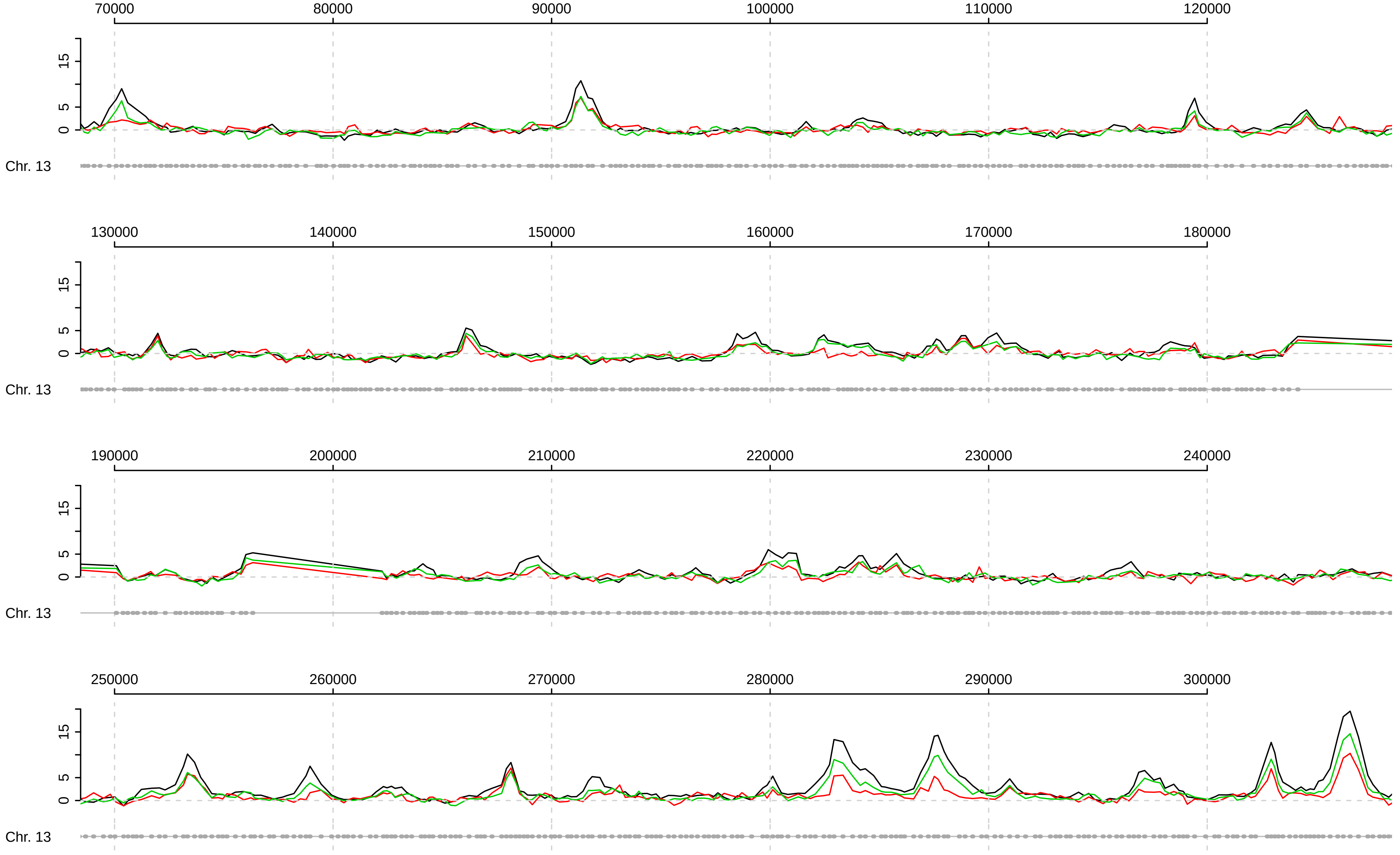

70000
80000
90000
100000
110000
120000
0
5
15
Chr. 13
130000
140000
150000
160000
170000
180000
0
5
15
Chr. 13
190000
200000
210000
220000
230000
240000
0
5
15
Chr. 13
250000
260000
270000
280000
290000
300000
0
5
15
Chr. 13

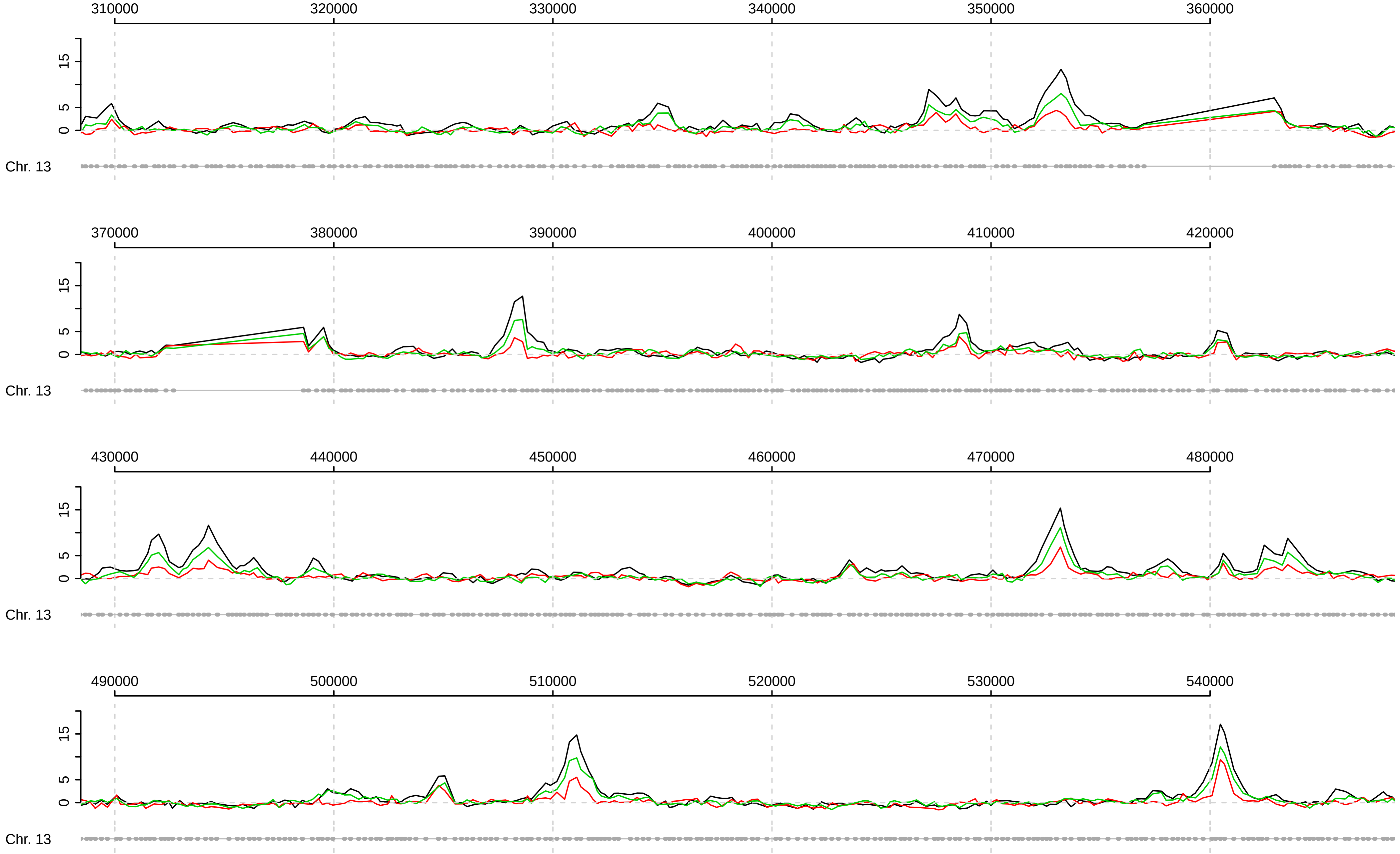
310000
320000
330000
340000
350000
360000
15
5
0
Chr. 13
370000
380000
390000
400000
410000
420000
15
5
0
Chr. 13
430000
440000
450000
460000
470000
480000
15
5
0
Chr. 13
490000
500000
510000
520000
530000
540000
15
5
0
Chr. 13

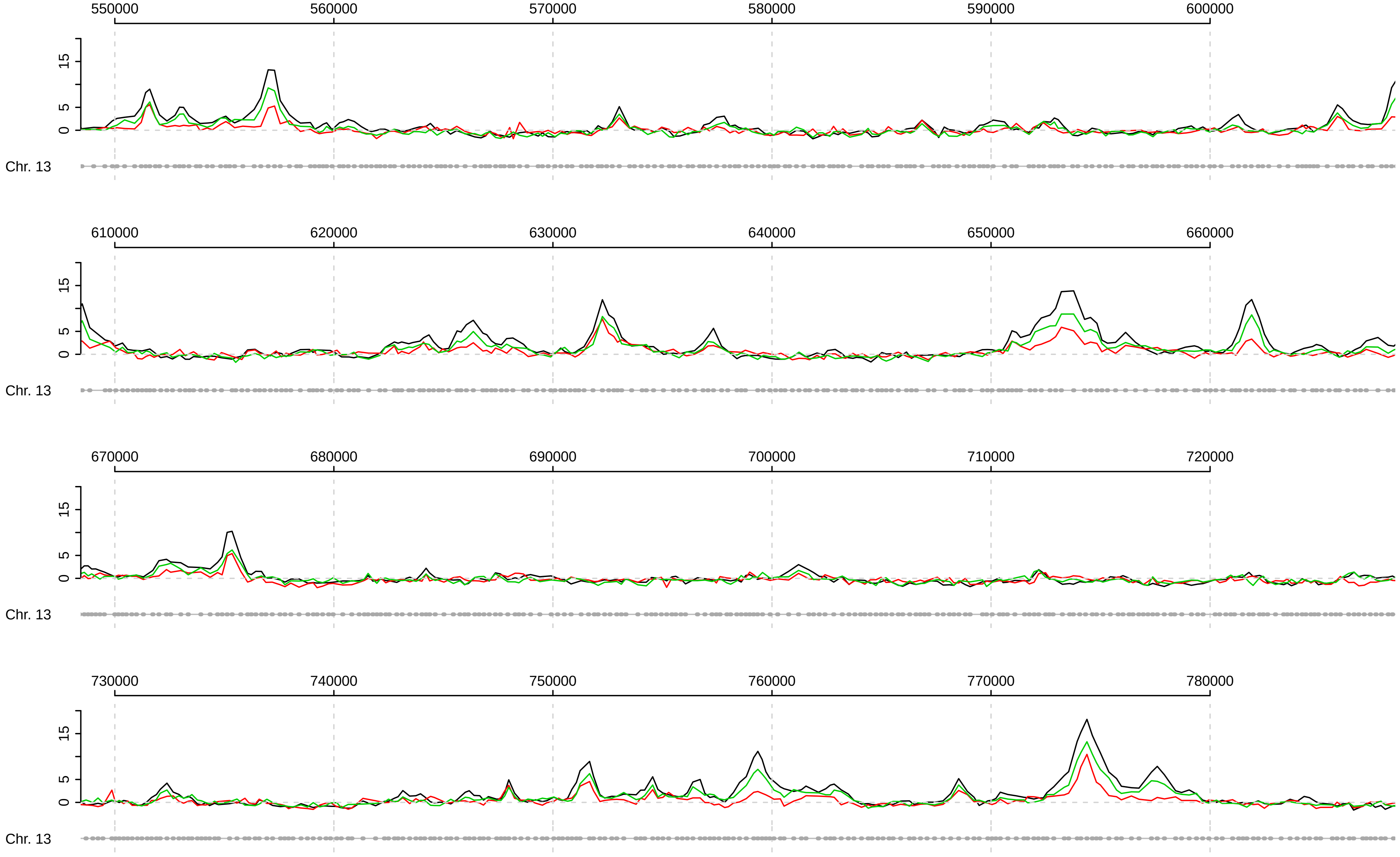

550000
560000
570000
580000
590000
600000
0
5
15
Chr. 13
610000
620000
630000
640000
650000
660000
0
5
15
Chr. 13
670000
680000
690000
700000
710000
720000
0
5
15
Chr. 13
730000
740000
750000
760000
770000
780000
0
5
15
Chr. 13

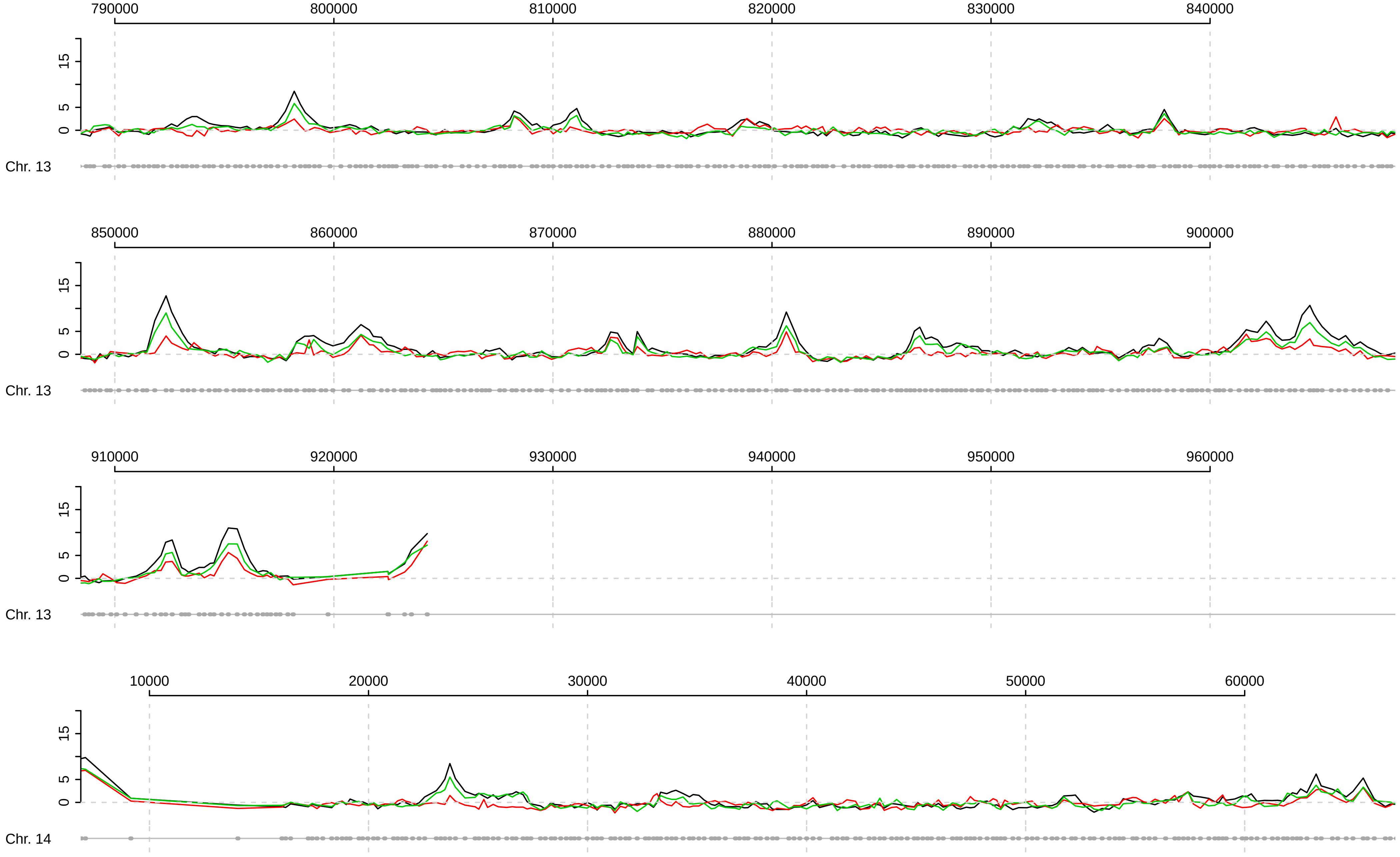
790000
800000
810000
820000
830000
840000
15
5
0
Chr. 13
850000
860000
870000
880000
890000
900000
15
5
0
Chr. 13
910000
920000
930000
940000
950000
960000
15
5
0
Chr. 13
10000
20000
30000
40000
50000
60000
15
5
0
Chr. 14

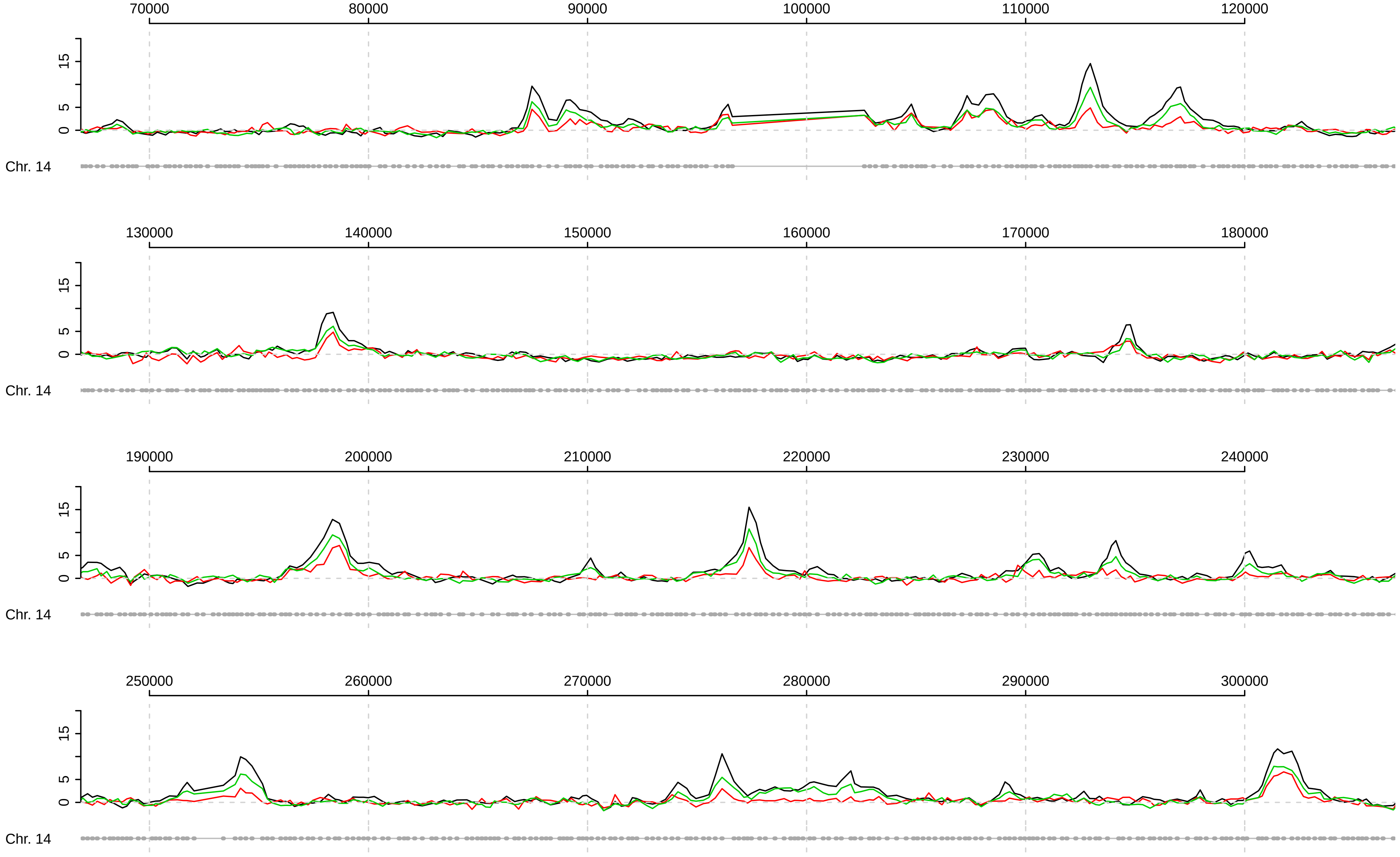

70000
80000
90000
100000
110000
120000
0
5
15
Chr. 14
130000
140000
150000
160000
170000
180000
0
5
15
Chr. 14
190000
200000
210000
220000
230000
240000
0
5
15
Chr. 14
250000
260000
270000
280000
290000
300000
0
5
15
Chr. 14

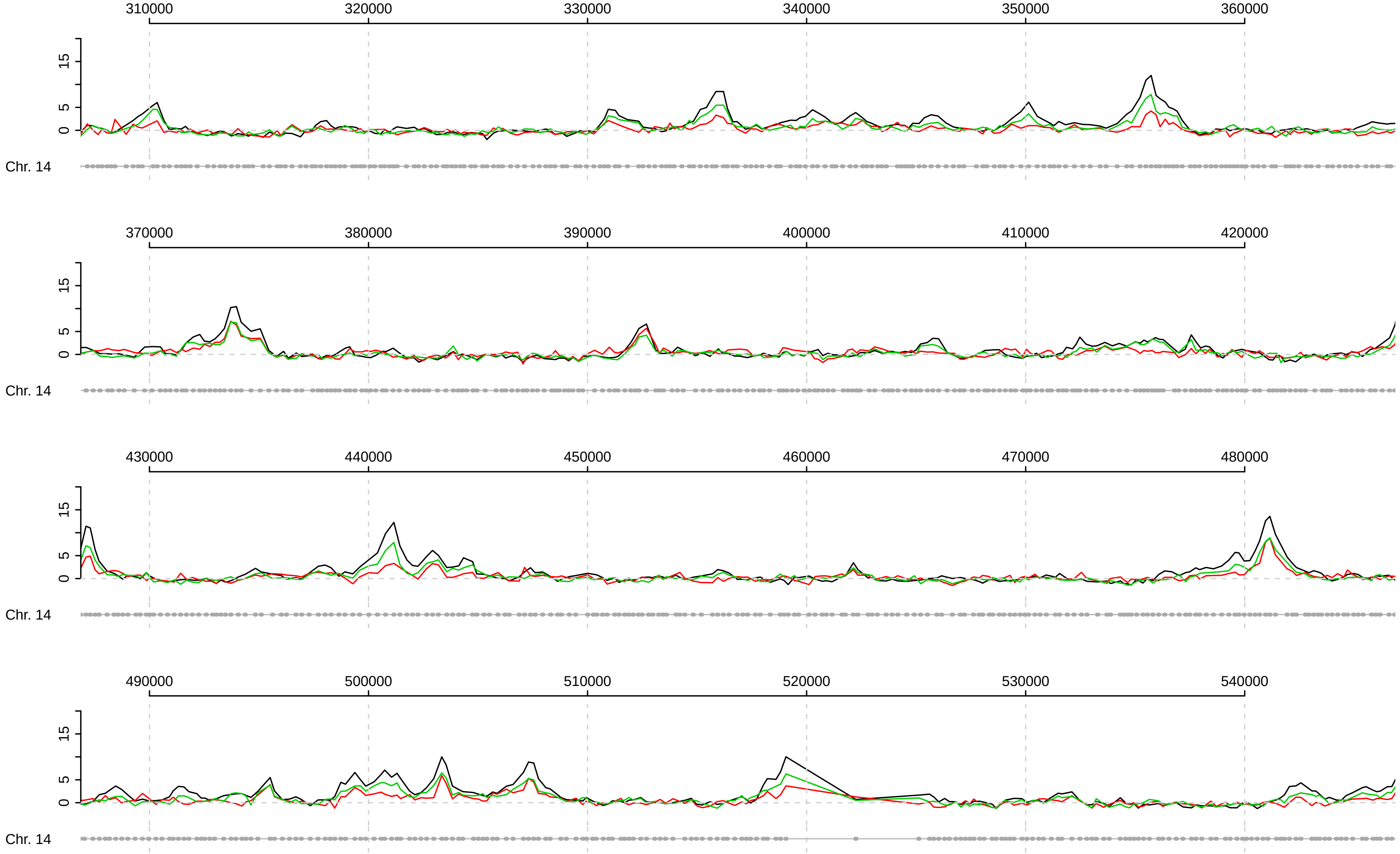
310000
320000
330000
340000
350000
360000
0
5
15
Chr. 14
370000
380000
390000
400000
410000
420000
0
5
15
Chr. 14
430000
440000
450000
460000
470000
480000
0
5
15
Chr. 14
490000
500000
510000
520000
530000
540000
0
5
15
Chr. 14

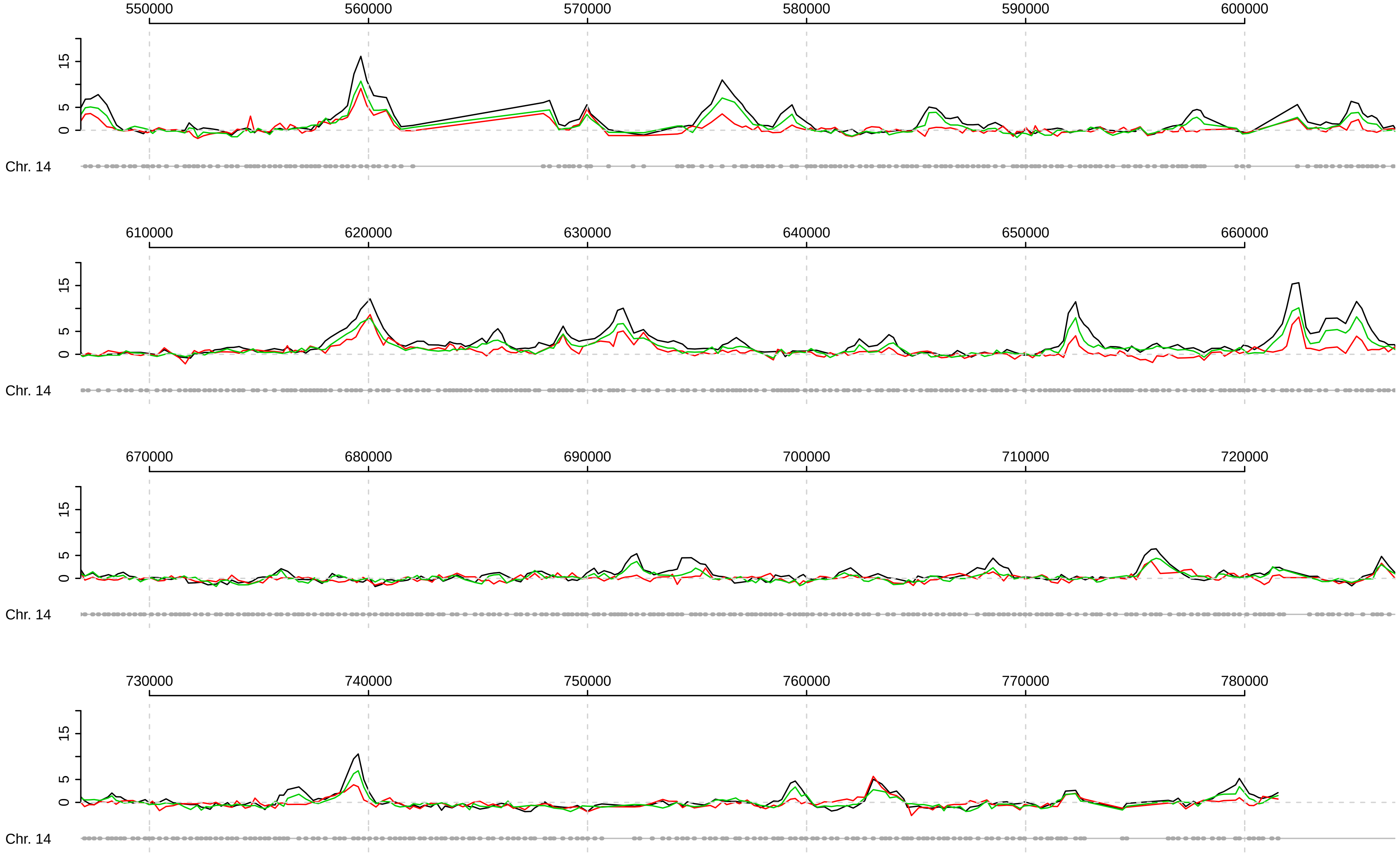

550000
560000
570000
580000
590000
600000
0
5
15
Chr. 14
610000
620000
630000
640000
650000
660000
0
5
15
Chr. 14
670000
680000
690000
700000
710000
720000
0
5
15
Chr. 14
730000
740000
750000
760000
770000
780000
0
5
15
Chr. 14

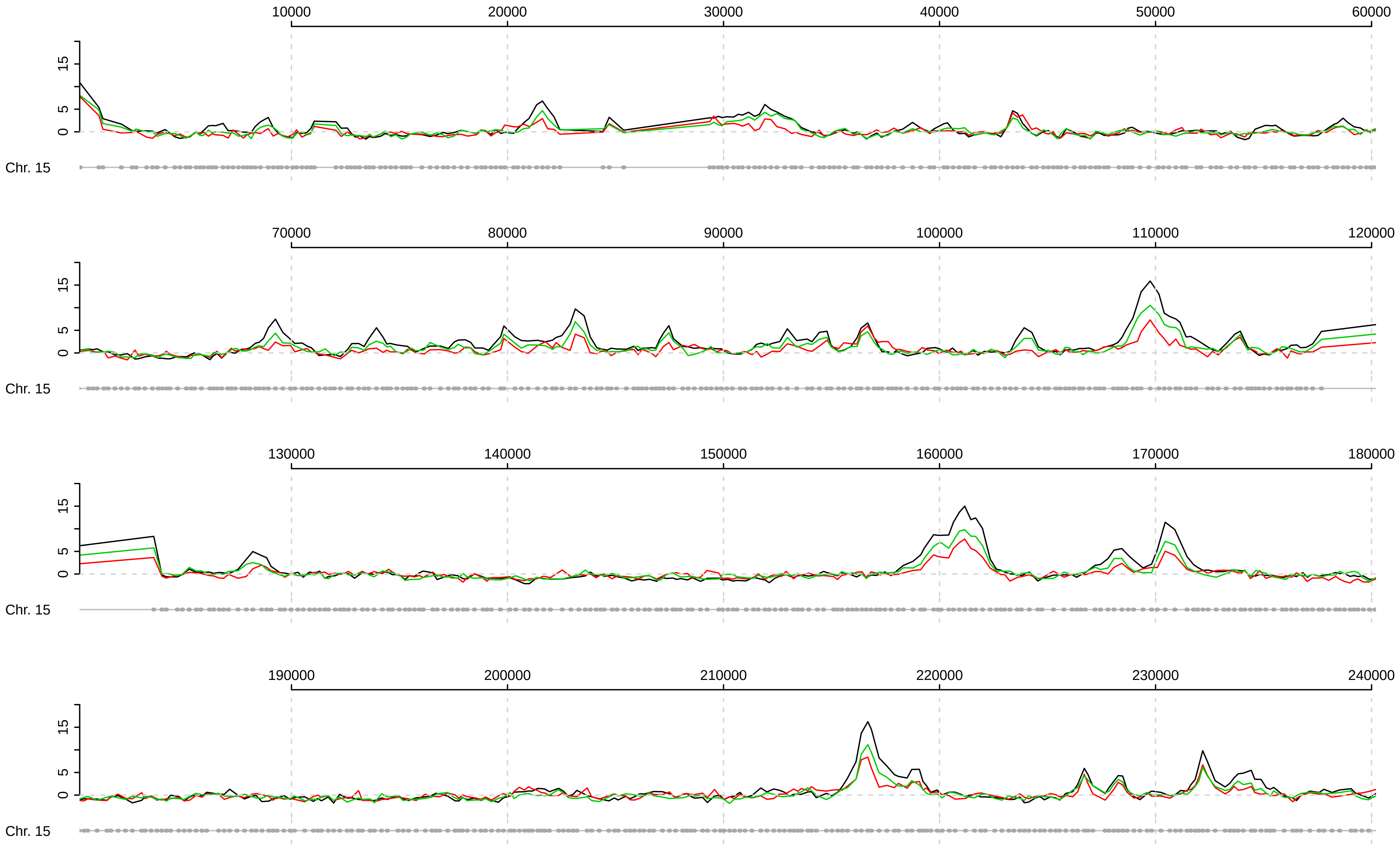

10000
20000
30000
40000
50000
60000
0
5
15
Chr. 15
70000
80000
90000
100000
110000
120000
0
5
15
Chr. 15
130000
140000
150000
160000
170000
180000
0
5
15
Chr. 15
190000
200000
210000
220000
230000
240000
0
5
15
Chr. 15

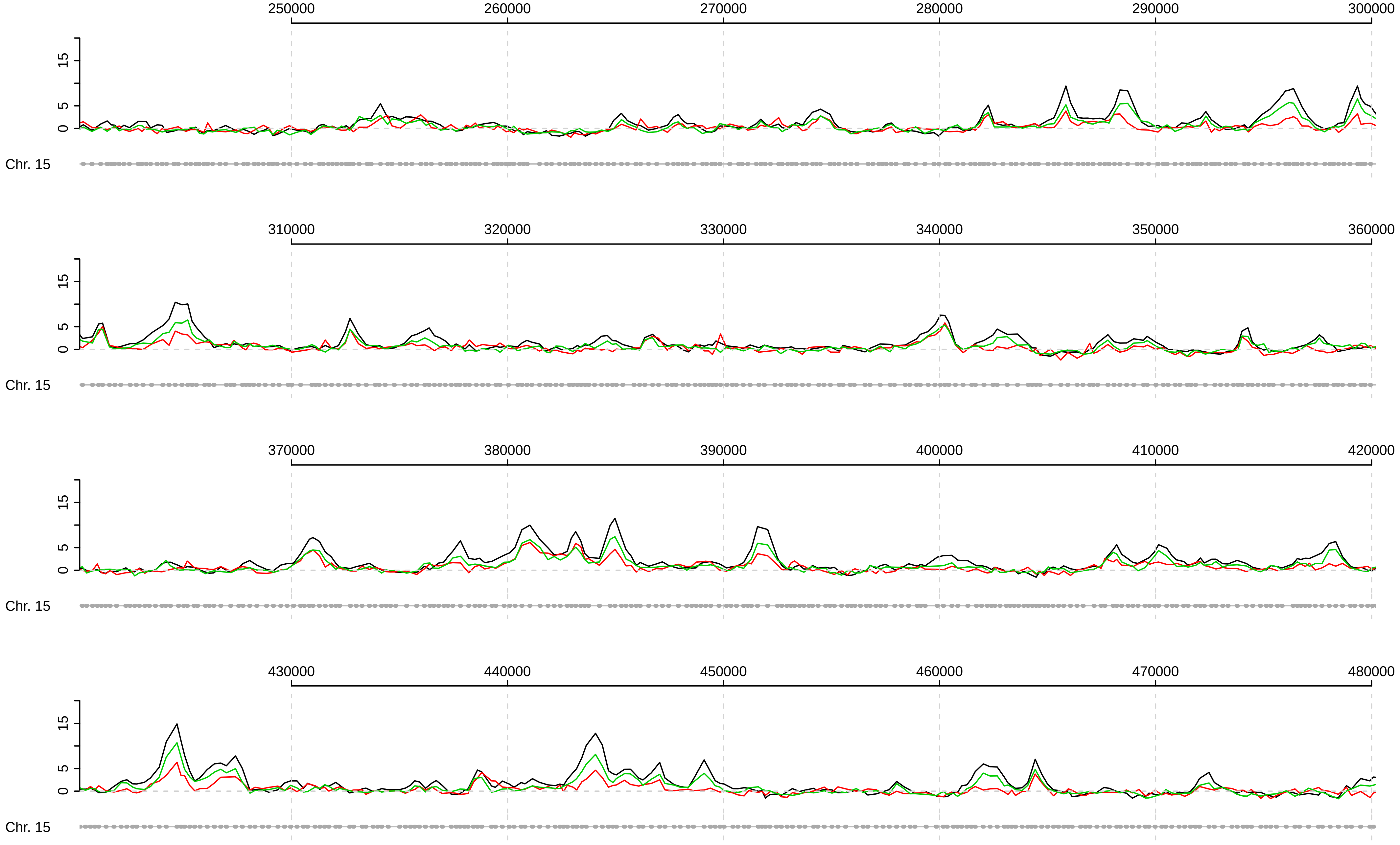

250000
260000
270000
280000
290000
300000
Chr. 15
310000
320000
330000
340000
350000
360000
Chr. 15
370000
380000
390000
400000
410000
420000
Chr. 15
430000
440000
450000
460000
470000
480000
Chr. 15
0
5
15

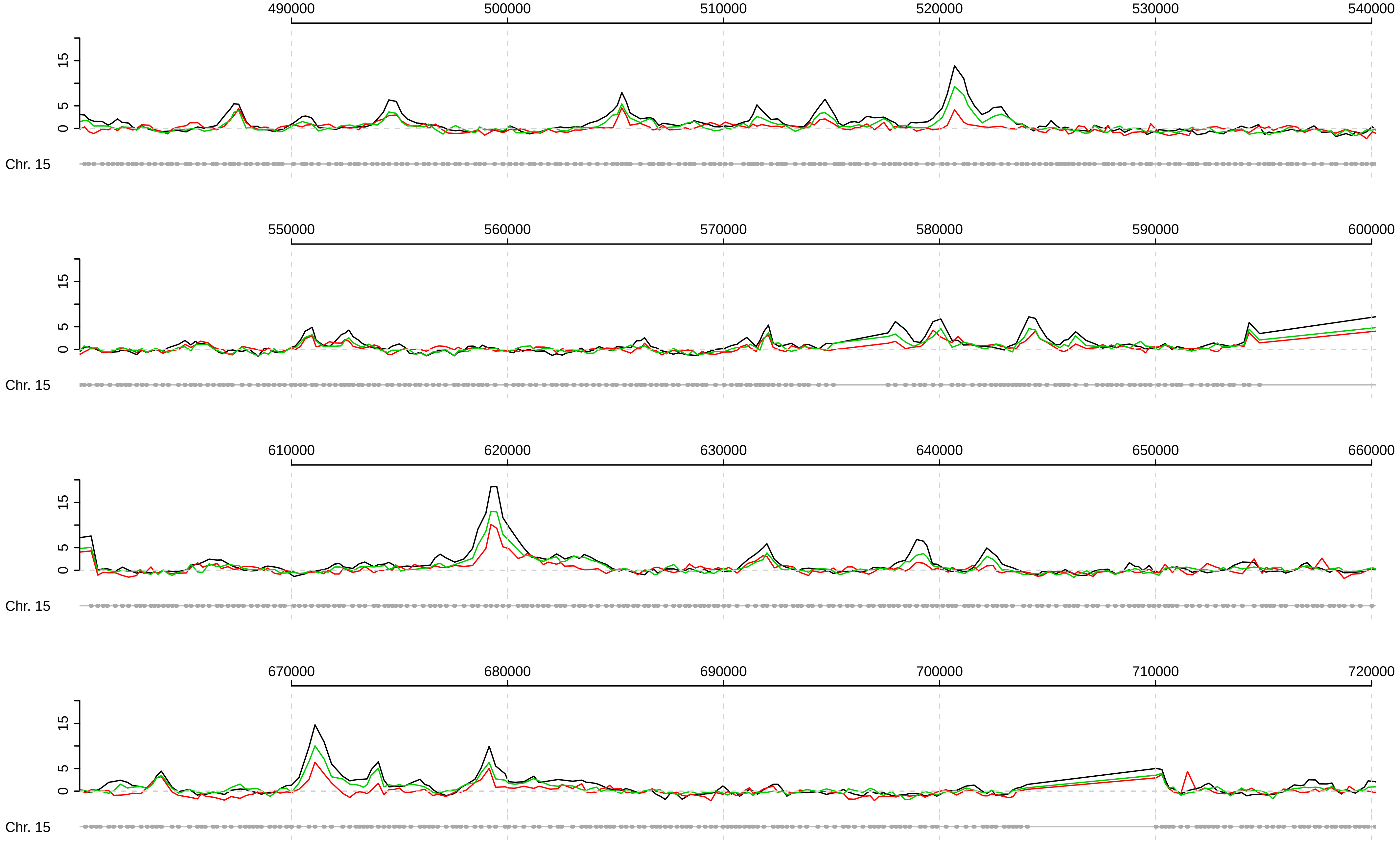

490000 500000 510000 520000 530000 540000
0 5 15
Chr. 15
550000 560000 570000 580000 590000 600000
0 5 15
Chr. 15
610000 620000 630000 640000 650000 660000
0 5 15
Chr. 15
670000 680000 690000 700000 710000 720000
0 5 15
Chr. 15

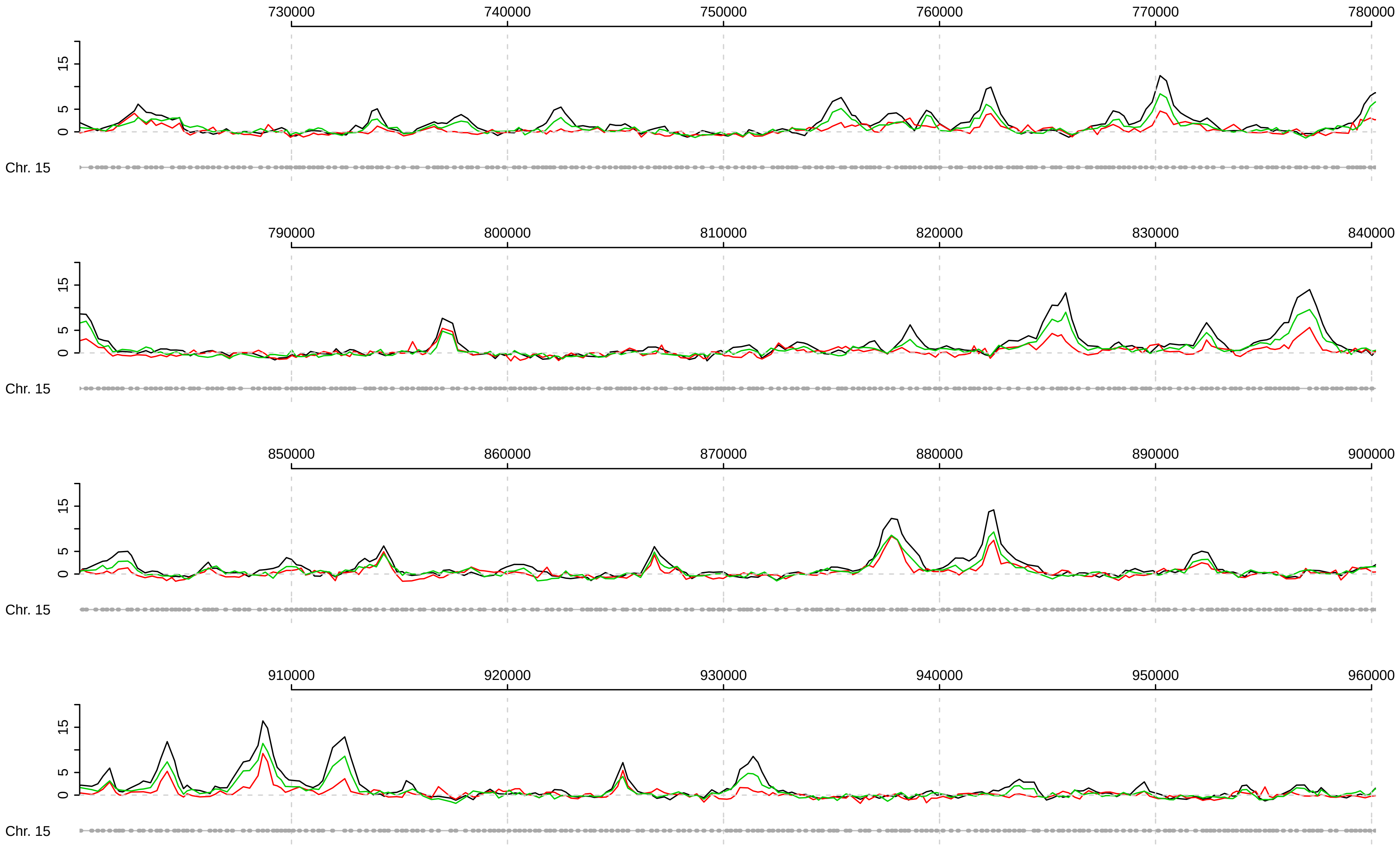

730000
740000
750000
760000
770000
780000
0
5
15
Chr. 15
790000
800000
810000
820000
830000
840000
0
5
15
Chr. 15
850000
860000
870000
880000
890000
900000
0
5
15
Chr. 15
910000
920000
930000
940000
950000
960000
0
5
15
Chr. 15

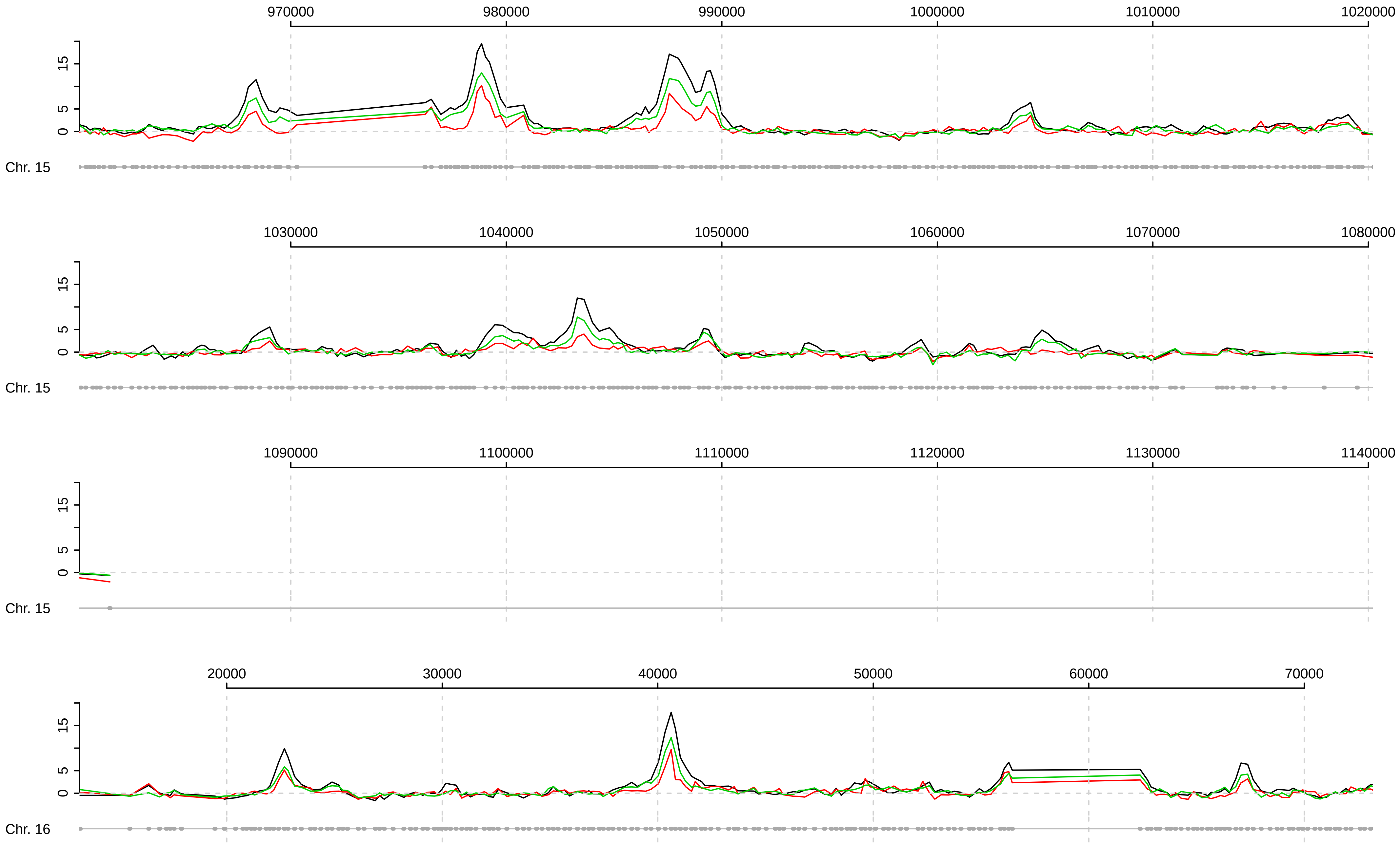

970000
980000
990000
1000000
1010000
1020000
0
5
15
Chr. 15
1030000
1040000
1050000
1060000
1070000
1080000
0
5
15
Chr. 15
1090000
1100000
1110000
1120000
1130000
1140000
0
5
15
Chr. 15
20000
30000
40000
50000
60000
70000
0
5
15
Chr. 16

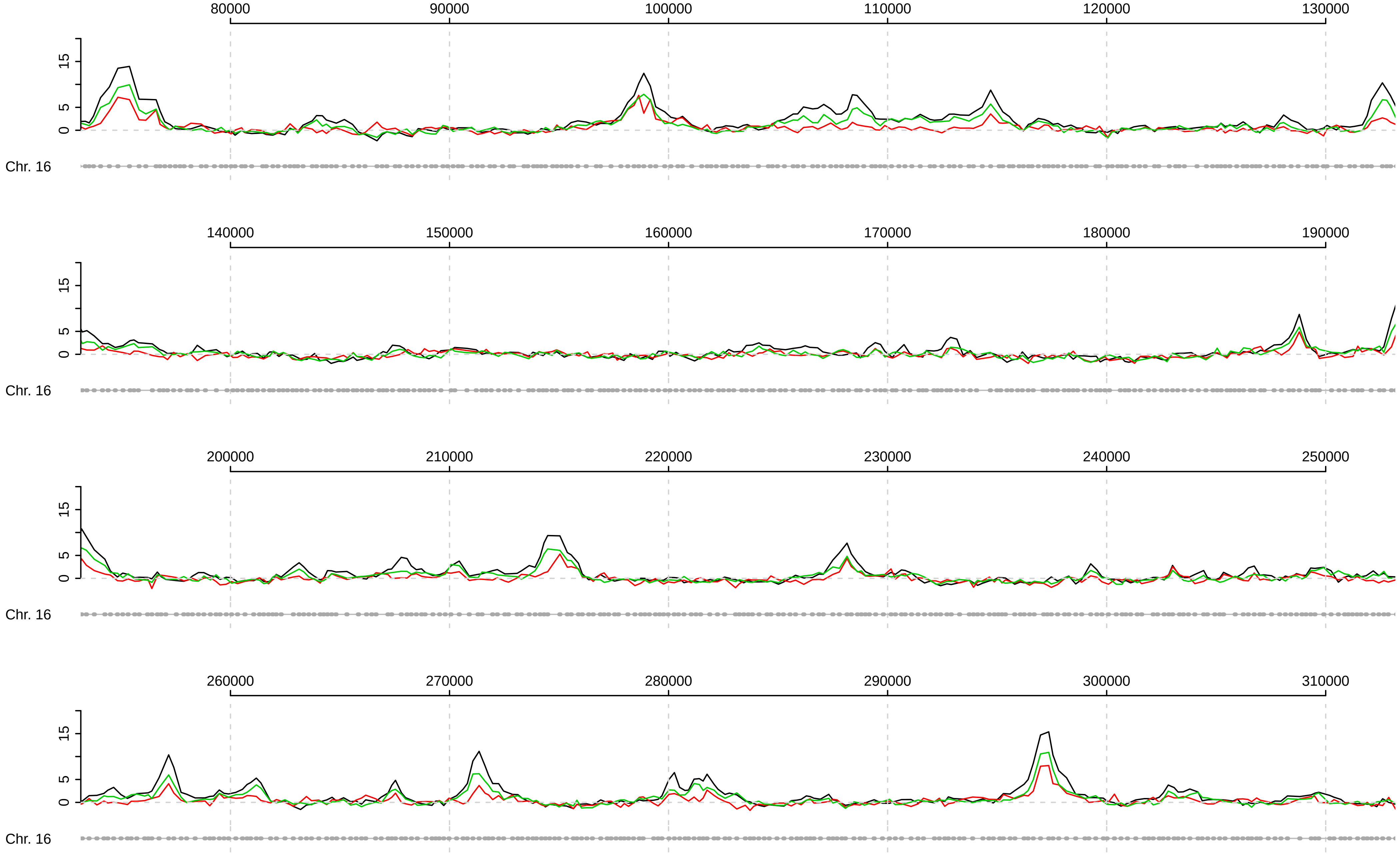

80000
90000
100000
110000
120000
130000
15
5
0
Chr. 16
140000
150000
160000
170000
180000
190000
15
5
0
Chr. 16
200000
210000
220000
230000
240000
250000
15
5
0
Chr. 16
260000
270000
280000
290000
300000
310000
15
5
0
Chr. 16

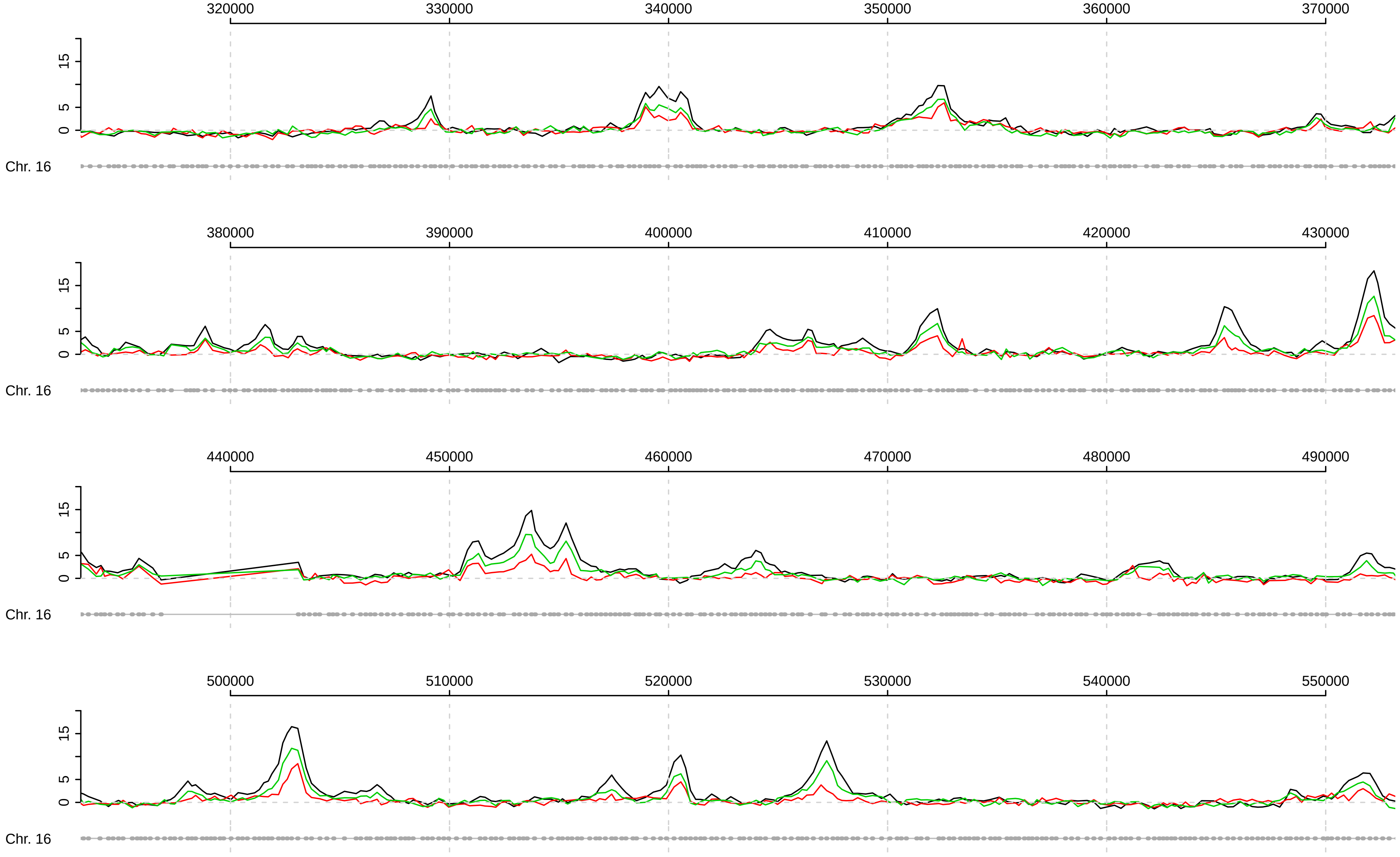

320000
330000
340000
350000
360000
370000
0
5
15
Chr. 16
380000
390000
400000
410000
420000
430000
0
5
15
Chr. 16
440000
450000
460000
470000
480000
490000
0
5
15
Chr. 16
500000
510000
520000
530000
540000
550000
0
5
15
Chr. 16

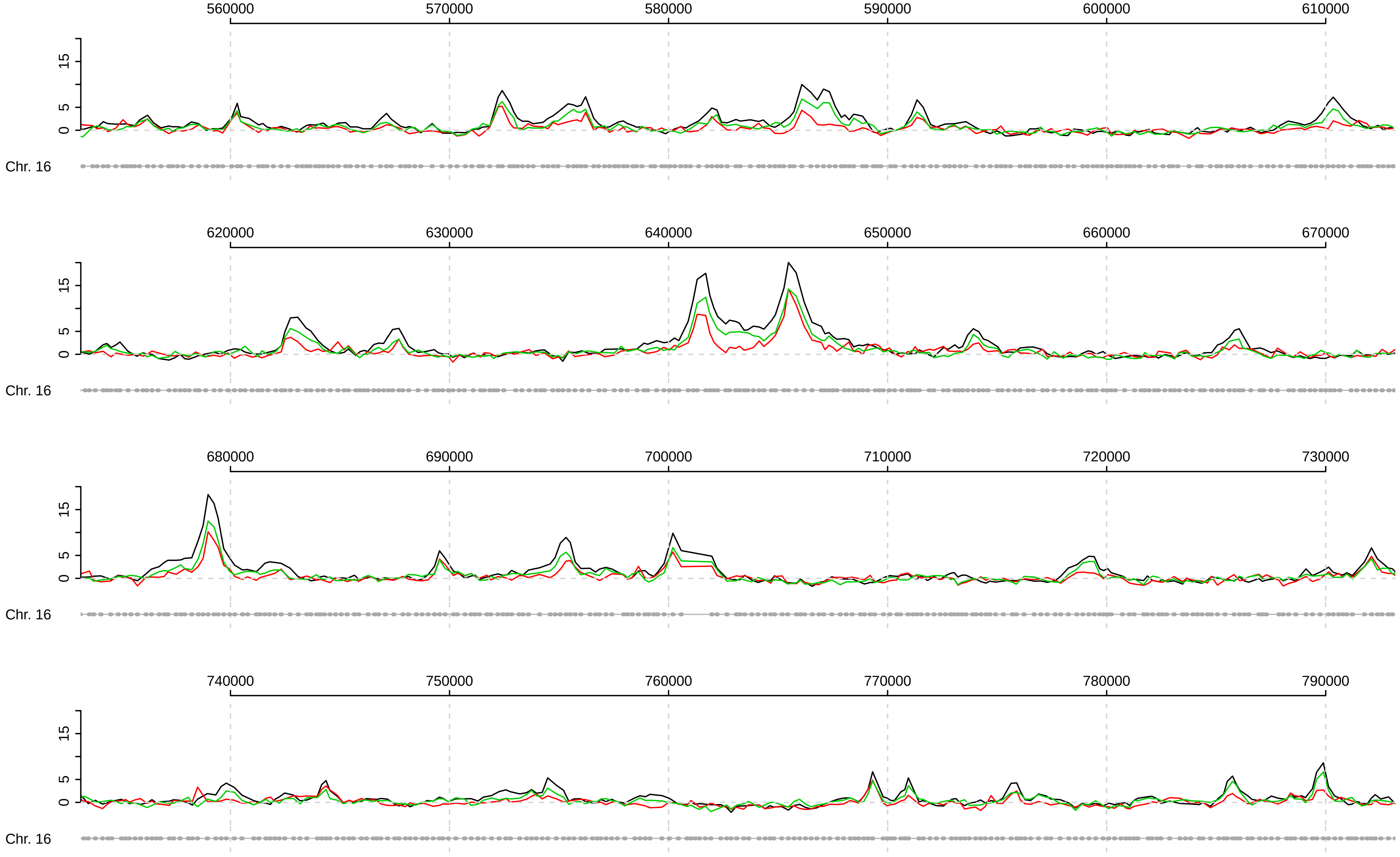

560000
570000
580000
590000
600000
610000
0
5
15
Chr. 16
620000
630000
640000
650000
660000
670000
0
5
15
Chr. 16
680000
690000
700000
710000
720000
730000
0
5
15
Chr. 16
740000
750000
760000
770000
780000
790000
0
5
15
Chr. 16

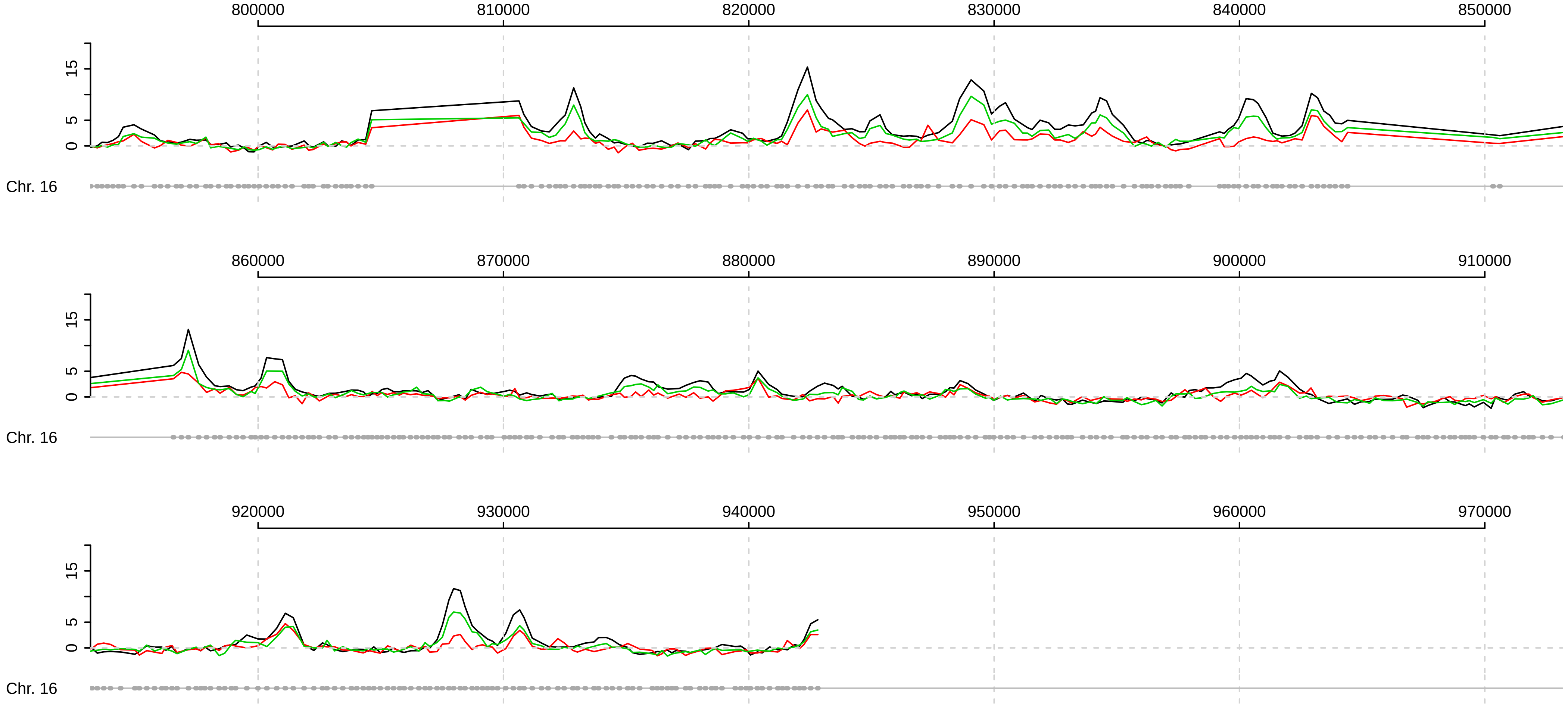

800000
810000
820000
830000
840000
850000
15
5
0
Chr. 16
860000
870000
880000
890000
900000
910000
15
5
0
Chr. 16
920000
930000
940000
950000
960000
970000
15
5
0
Chr. 16

The following plots show H3Ac enrichment over the whole genome in cells not treated with UV. The five line colours show five replicate datasets. The red crosses show probes detected as enriched.

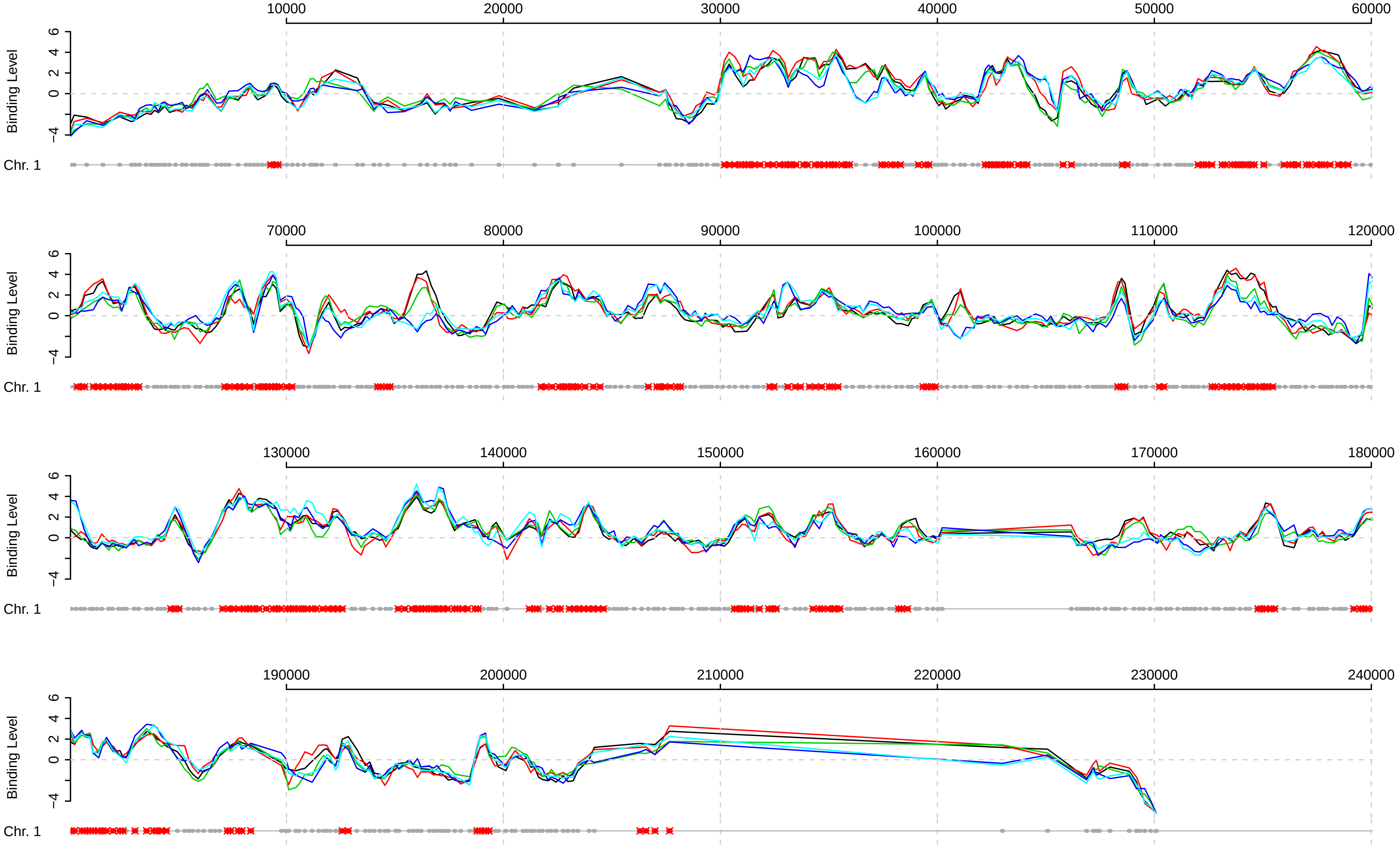
10000
20000
30000
40000
50000
60000
Binding Level
Chr. 1
70000
80000
90000
100000
110000
120000
130000
140000
150000
160000
170000
180000
190000
200000
210000
220000
230000
240000

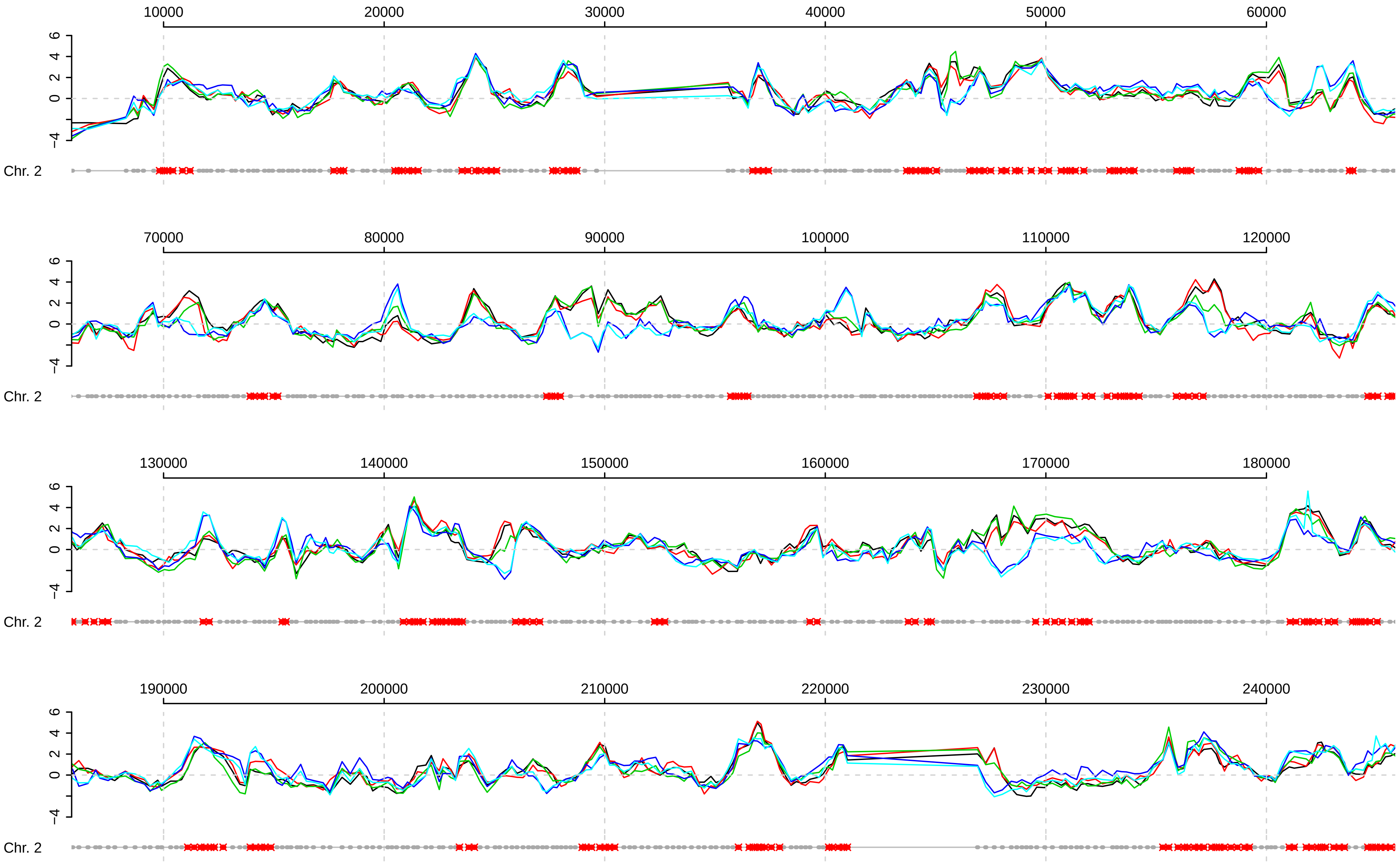
10000
20000
30000
40000
50000
60000
70000
80000
90000
100000
110000
120000
130000
140000
150000
160000
170000
180000
190000
200000
210000
220000
230000
240000
Chr. 2
Chr. 2
Chr. 2
Chr. 2

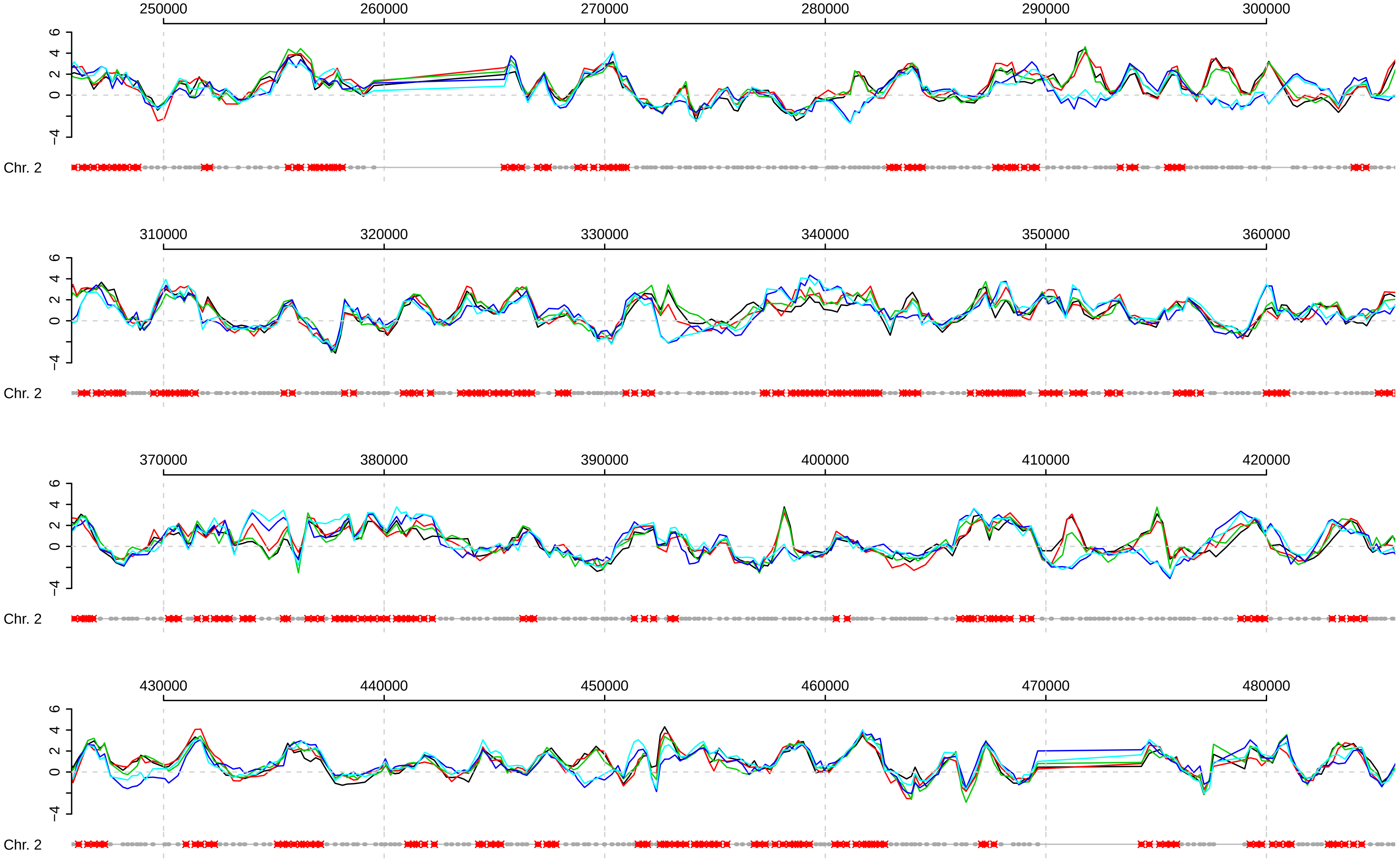

250000
260000
270000
280000
290000
300000
6
4
2
0
−4
Chr. 2
310000
320000
330000
340000
350000
360000
6
4
2
0
−4
Chr. 2
370000
380000
390000
400000
410000
420000
6
4
2
0
−4
Chr. 2
430000
440000
450000
460000
470000
480000
6
4
2
0
−4
Chr. 2

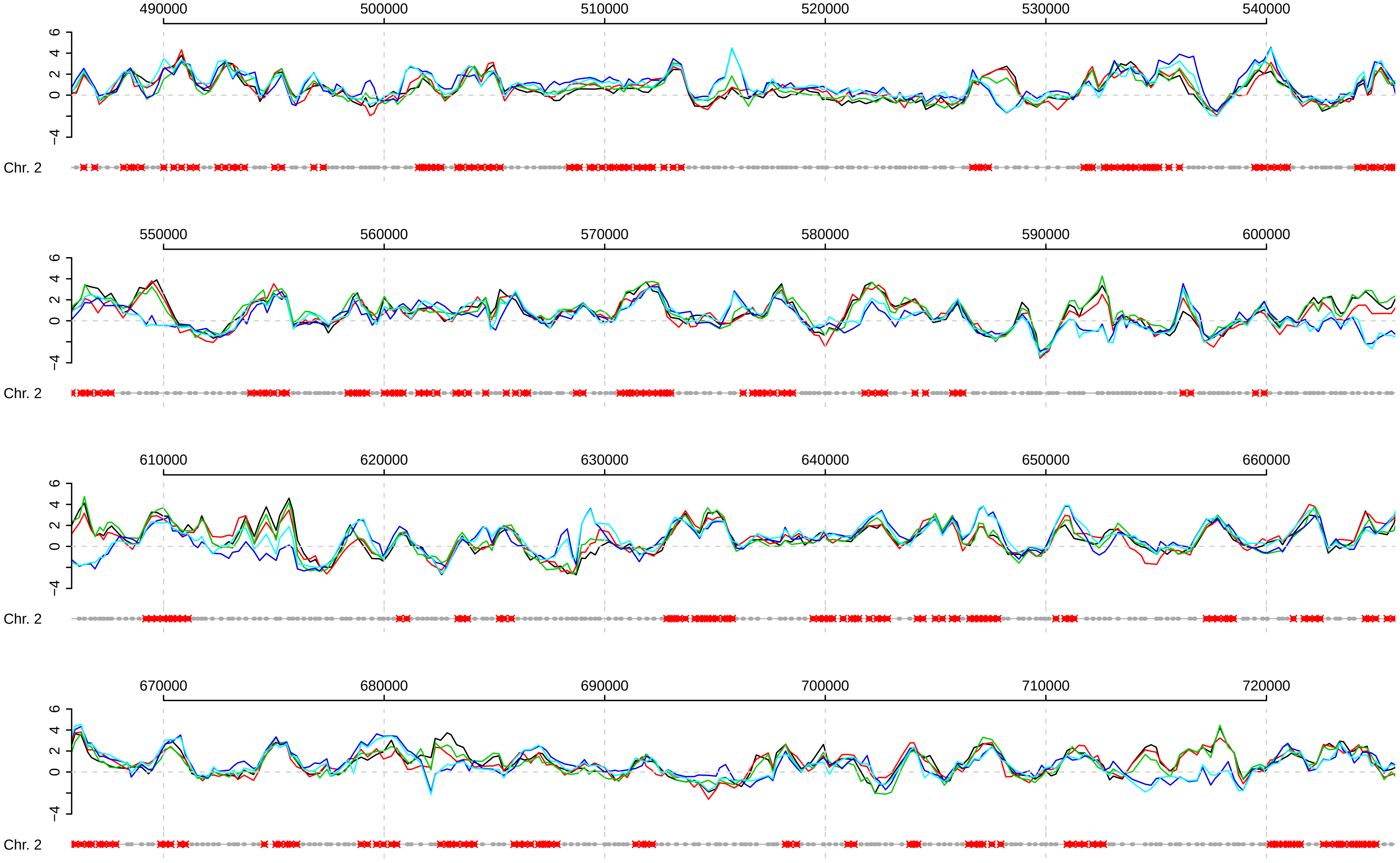

490000
500000
510000
520000
530000
540000
6
4
2
0
−4
Chr. 2
550000
560000
570000
580000
590000
600000
6
4
2
0
−4
Chr. 2
610000
620000
630000
640000
650000
660000
6
4
2
0
−4
Chr. 2
670000
680000
690000
700000
710000
720000
6
4
2
0
−4
Chr. 2

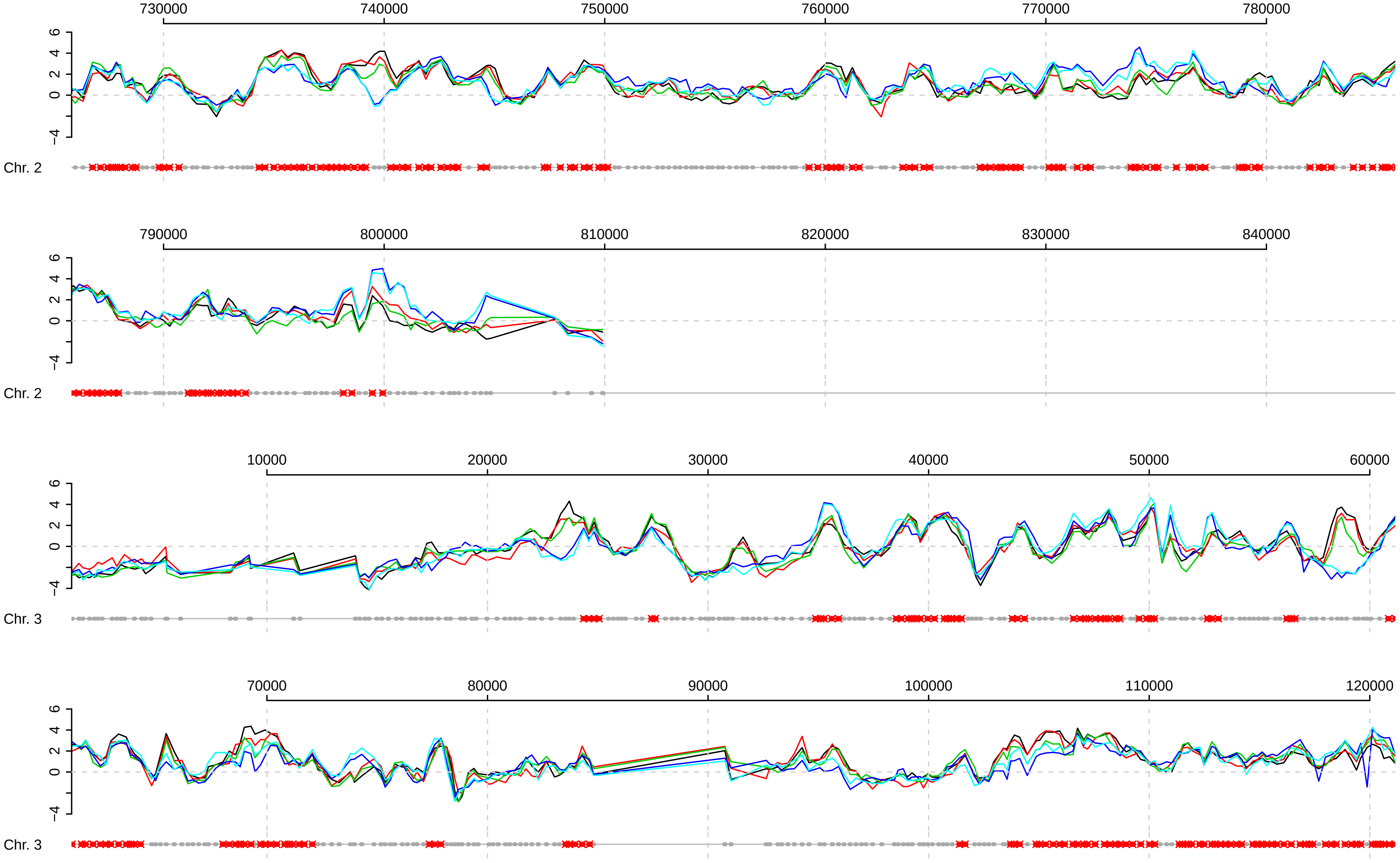

730000
740000
750000
760000
770000
780000
Chr. 2
790000
800000
810000
820000
830000
840000
Chr. 2
10000
20000
30000
40000
50000
60000
Chr. 3
70000
80000
90000
100000
110000
120000
Chr. 3

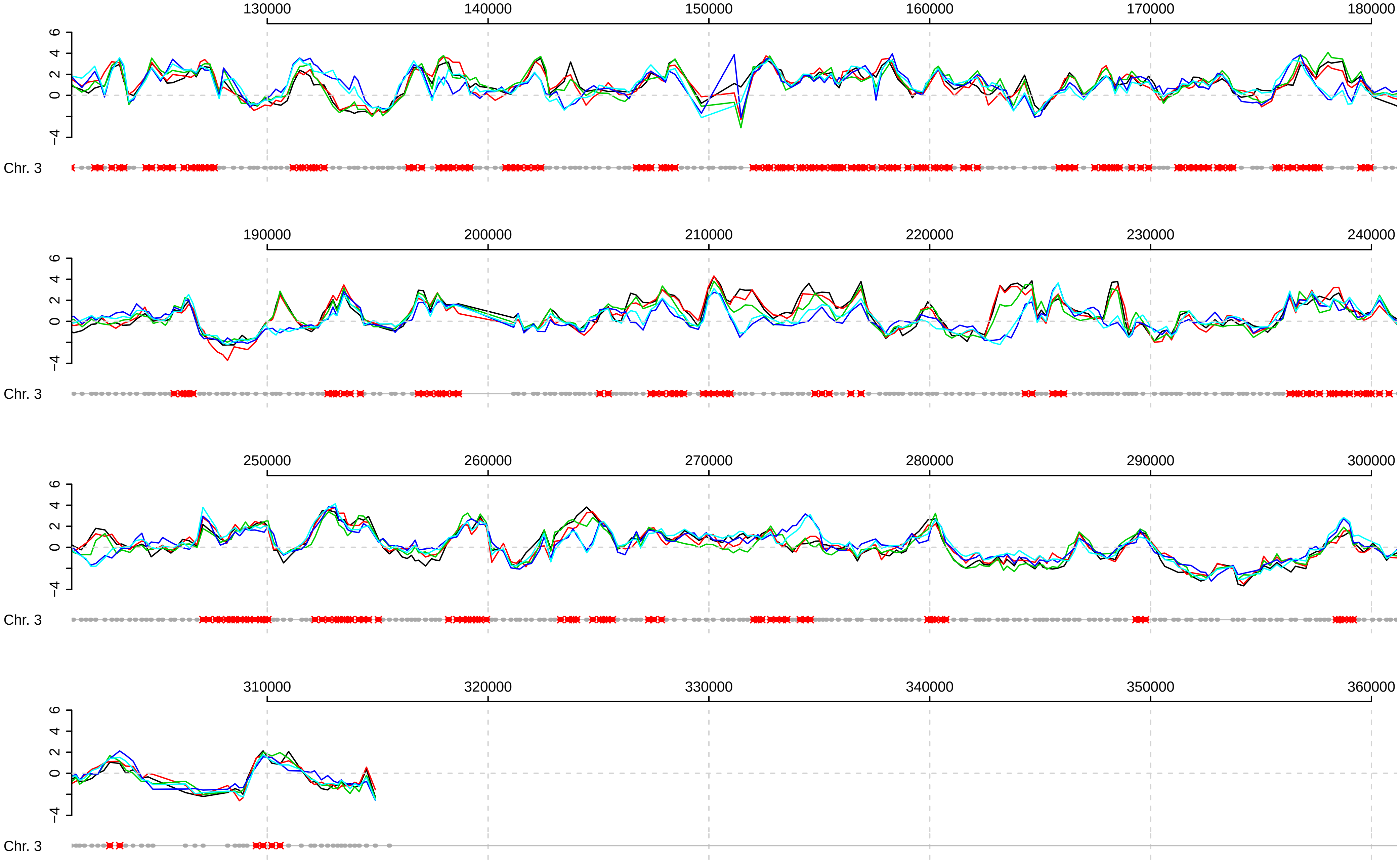
130000
140000
150000
160000
170000
180000
6
4
2
0
−4
Chr. 3
190000
200000
210000
220000
230000
240000
Chr. 3
250000
260000
270000
280000
290000
300000
Chr. 3
310000
320000
330000
340000
350000
360000
Chr. 3

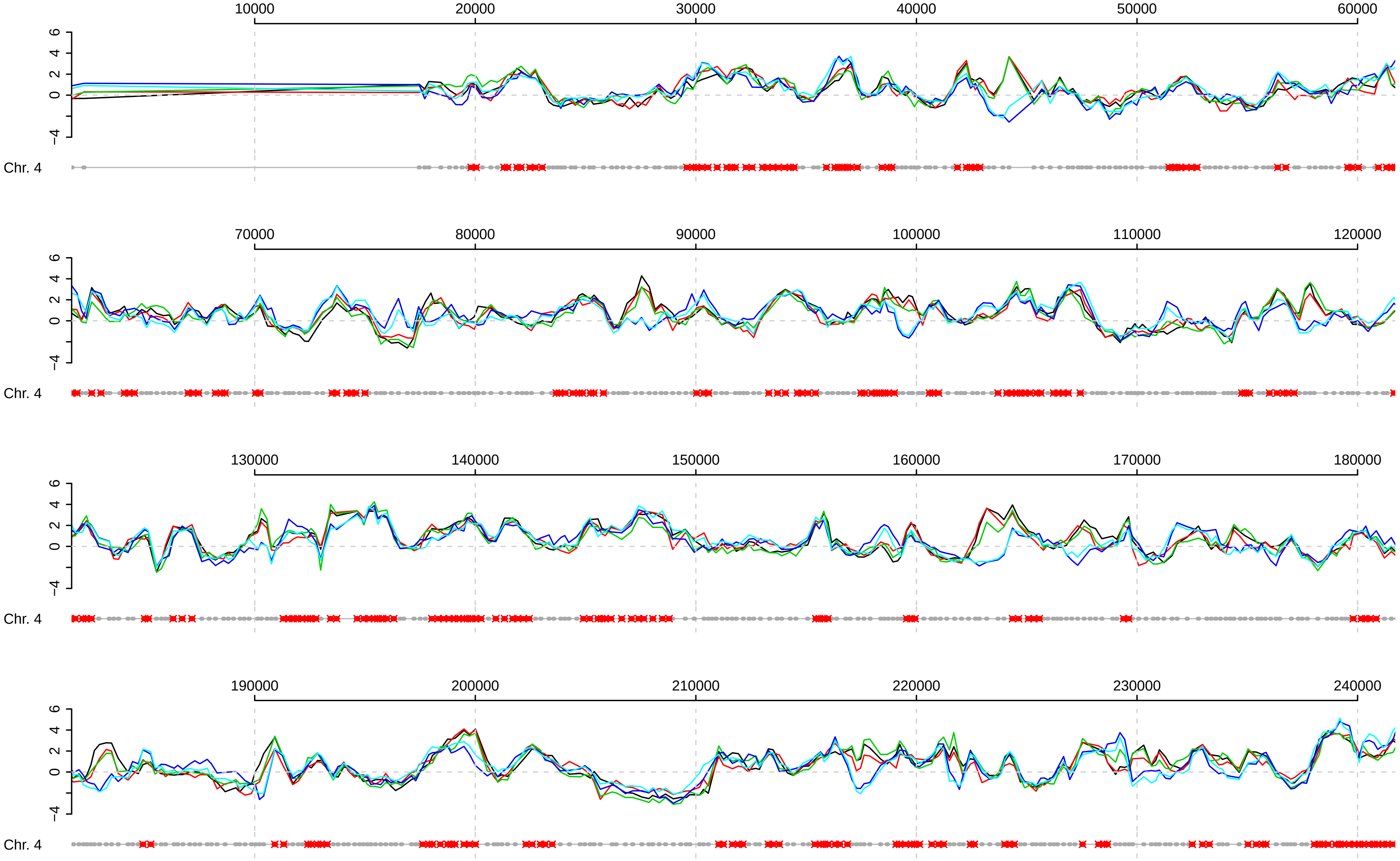

10000
20000
30000
40000
50000
60000
70000
80000
90000
100000
110000
120000
130000
140000
150000
160000
170000
180000
190000
200000
210000
220000
230000
240000
6
4
2
0
−4
Chr. 4

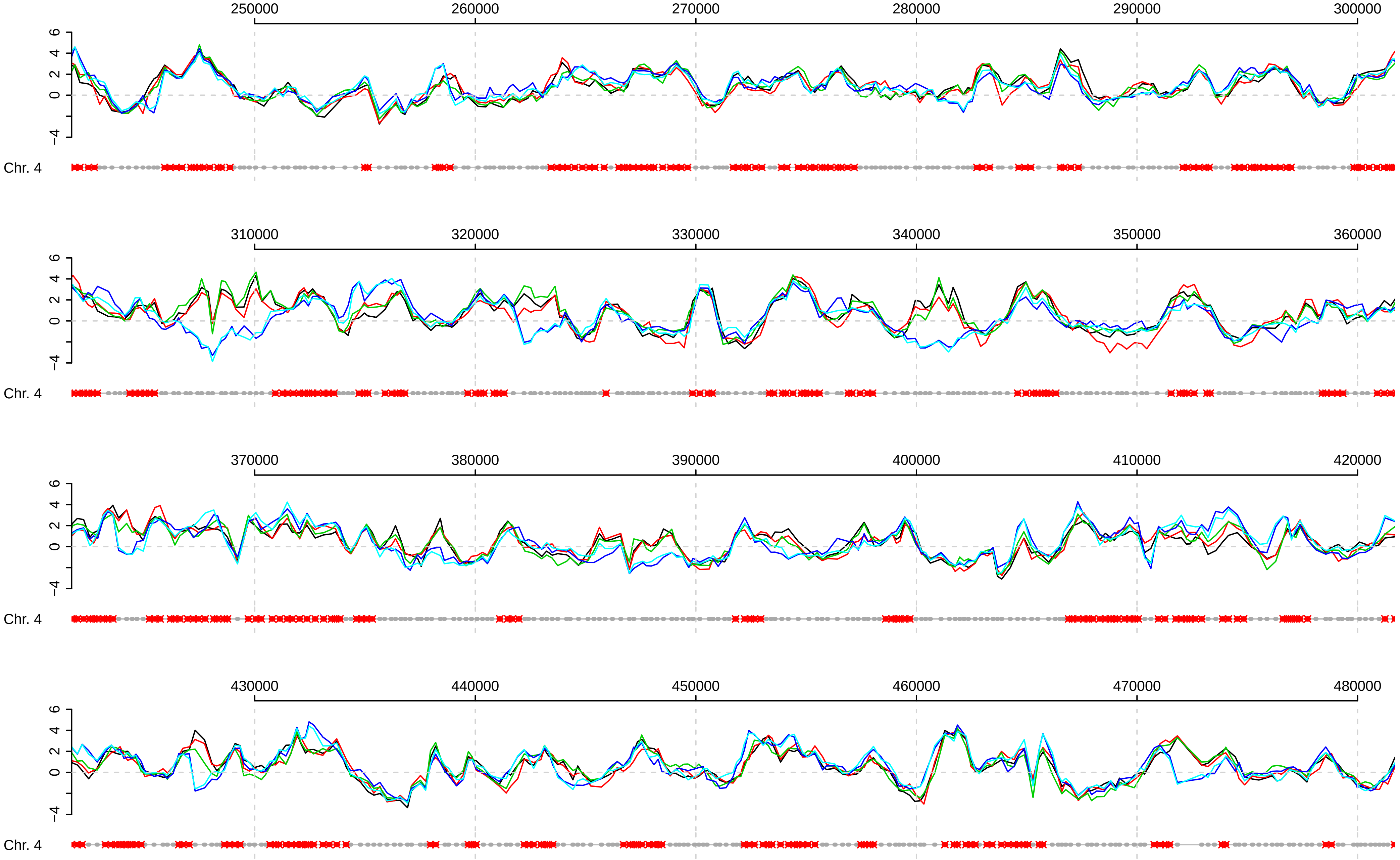

250000
260000
270000
280000
290000
300000
6
4
2
0
−4
Chr. 4
310000
320000
330000
340000
350000
360000
6
4
2
0
−4
Chr. 4
370000
380000
390000
400000
410000
420000
6
4
2
0
−4
Chr. 4
430000
440000
450000
460000
470000
480000
6
4
2
0
−4
Chr. 4

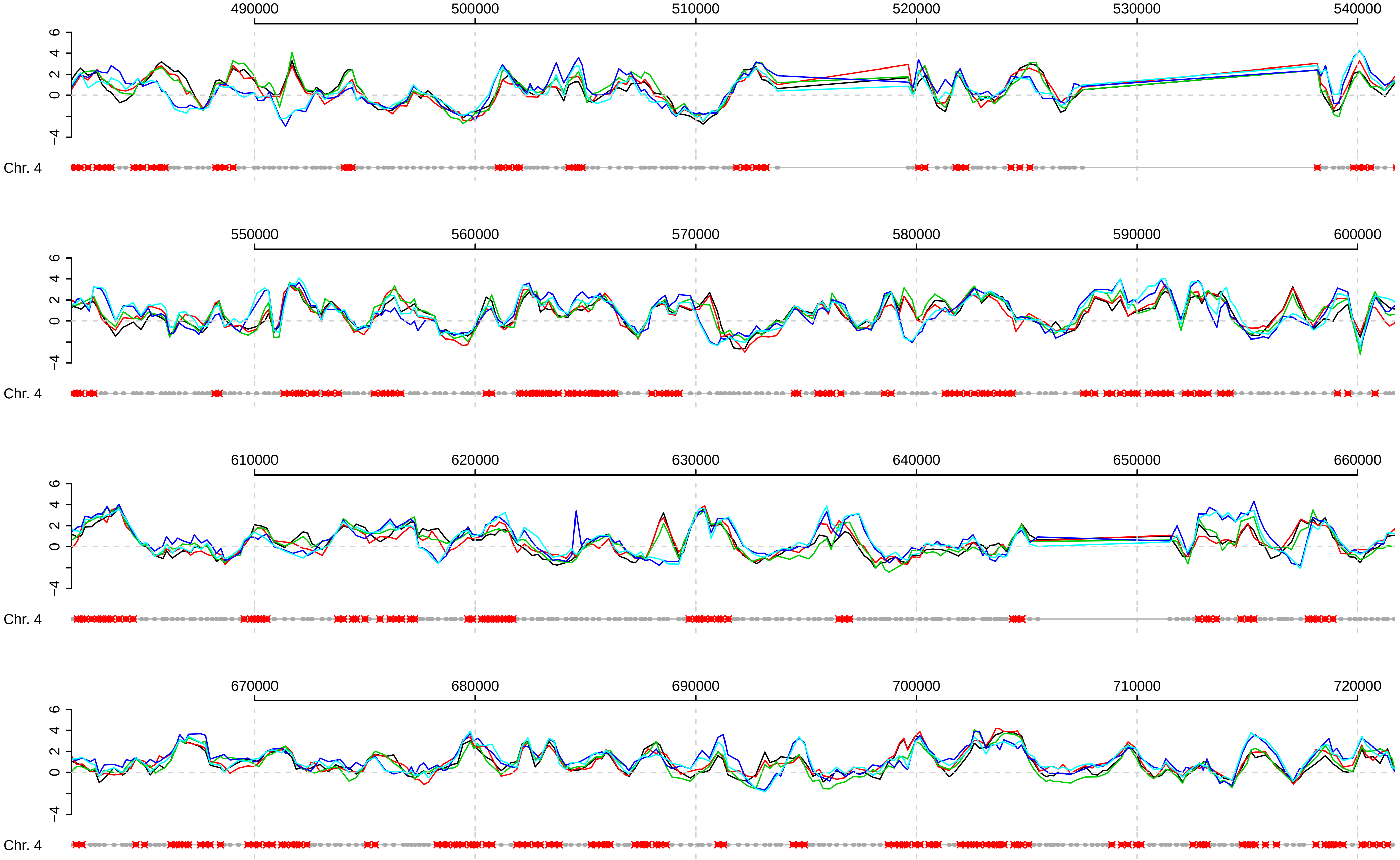
490000
500000
510000
520000
530000
540000
6
4
2
0
−4
Chr. 4
550000
560000
570000
580000
590000
600000
Chr. 4
610000
620000
630000
640000
650000
660000
Chr. 4
670000
680000
690000
700000
710000
720000
Chr. 4

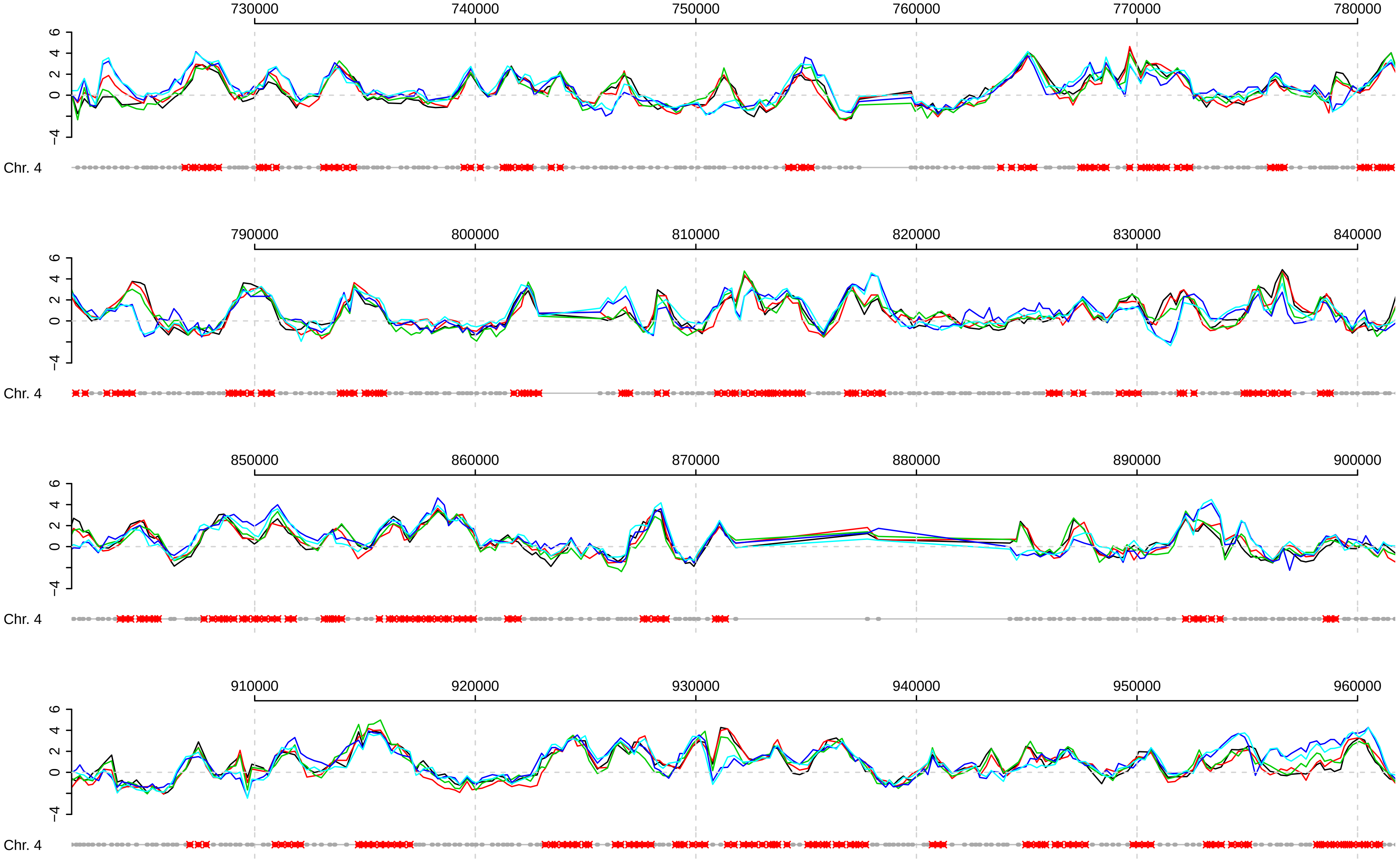

730000
740000
750000
760000
770000
780000
6
4
2
0
−4
Chr. 4
790000
800000
810000
820000
830000
840000
Chr. 4
850000
860000
870000
880000
890000
900000
Chr. 4
910000
920000
930000
940000
950000
960000
Chr. 4

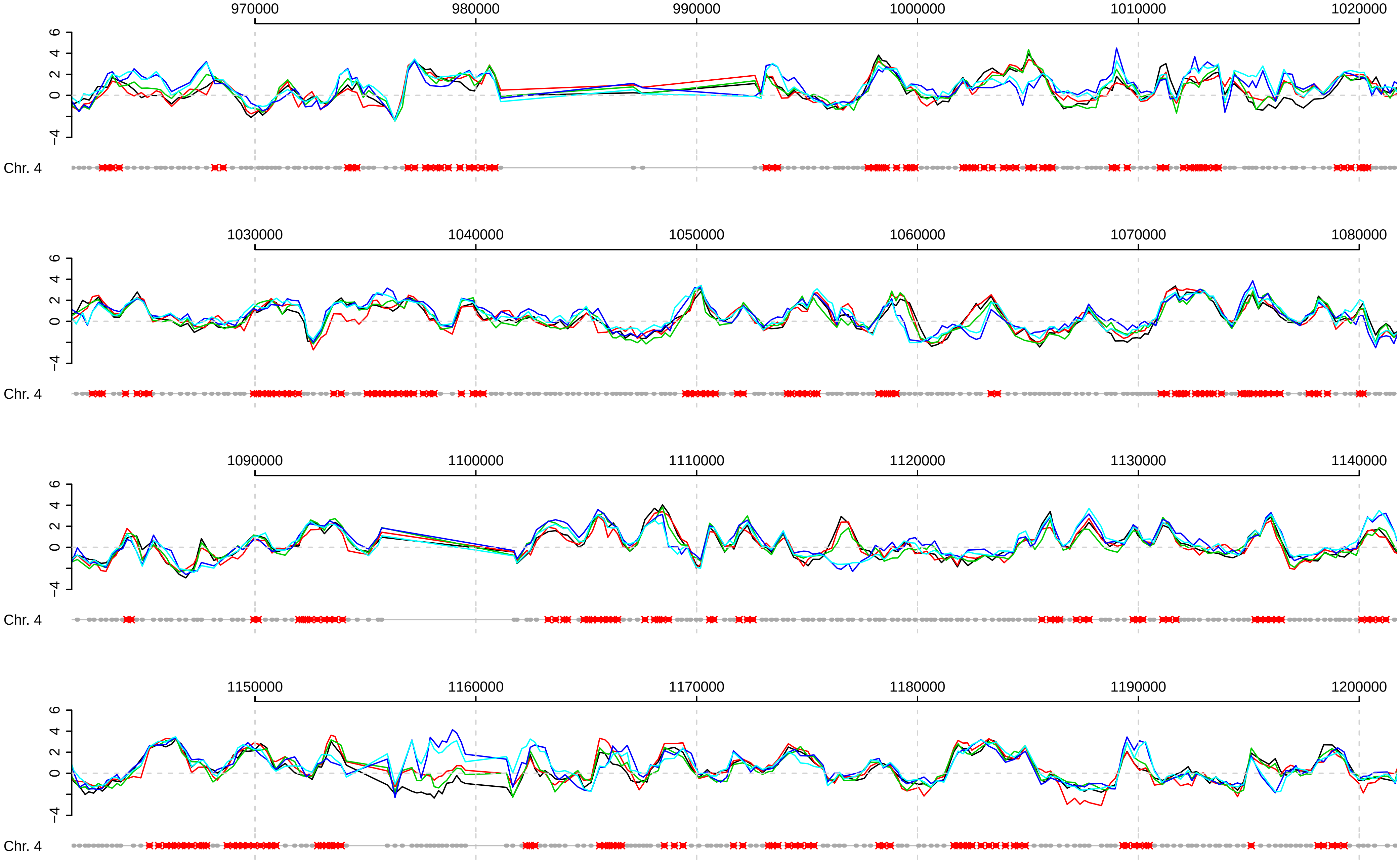

970000
980000
990000
1000000
1010000
1020000
1030000
1040000
1050000
1060000
1070000
1080000
1090000
1100000
1110000
1120000
1130000
1140000
1150000
1160000
1170000
1180000
1190000
1200000
6
4
2
0
−4
Chr. 4

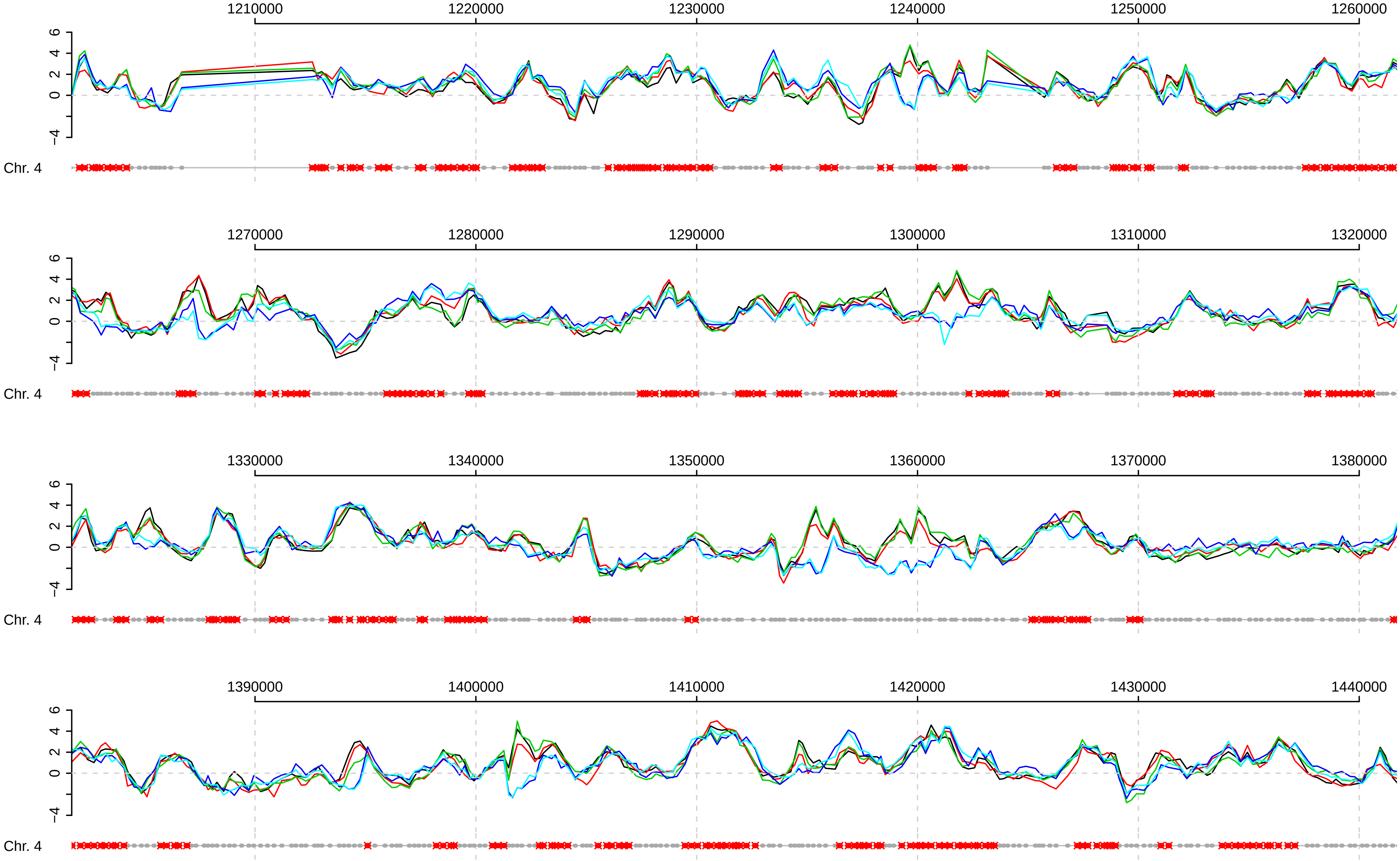

1210000
1220000
1230000
1240000
1250000
1260000
6
4
2
0
−4
Chr. 4
1270000
1280000
1290000
1300000
1310000
1320000
6
4
2
0
−4
Chr. 4
1330000
1340000
1350000
1360000
1370000
1380000
6
4
2
0
−4
Chr. 4
1390000
1400000
1410000
1420000
1430000
1440000
6
4
2
0
−4
Chr. 4

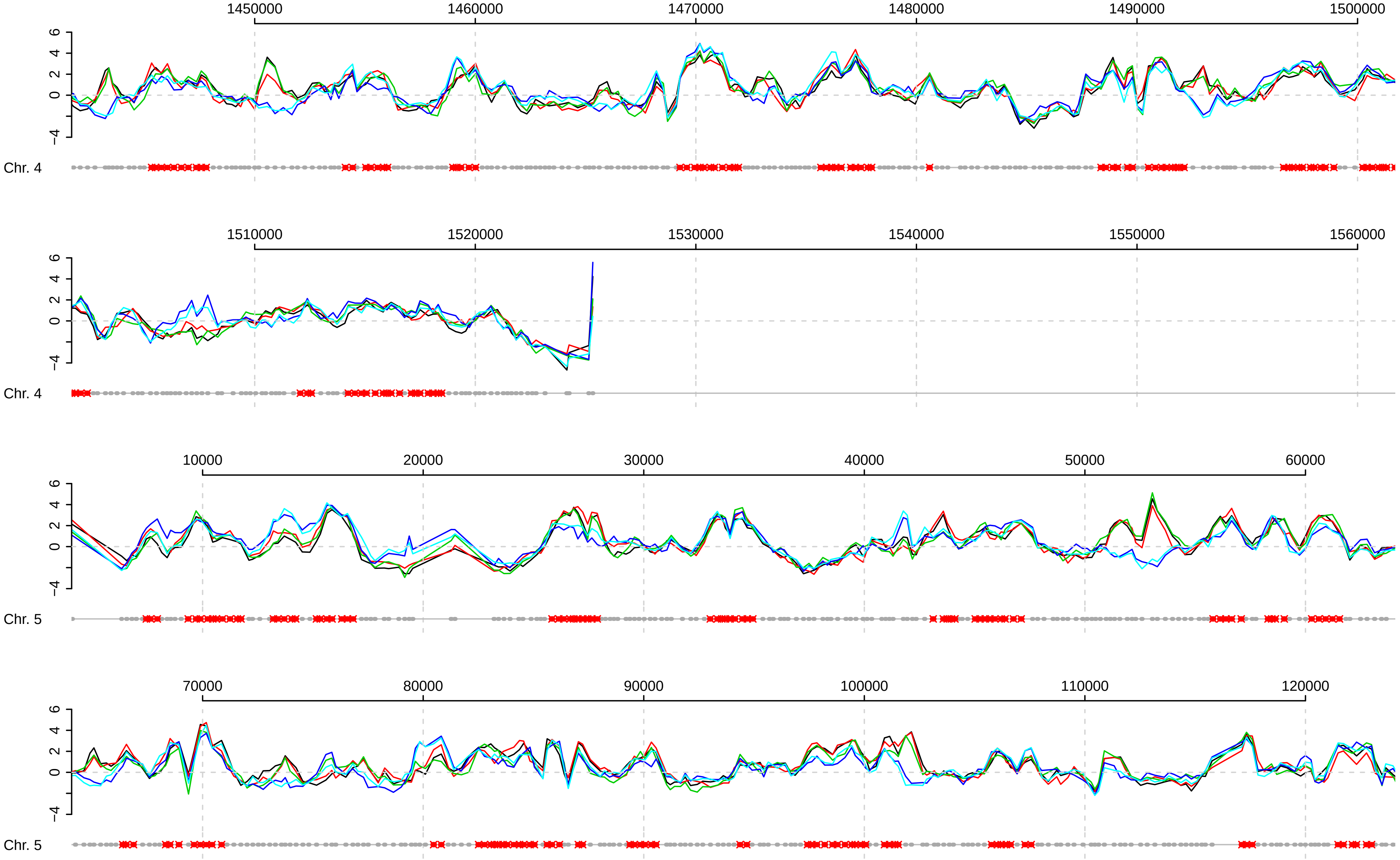
1450000
1460000
1470000
1480000
1490000
1500000
Chr. 4
1510000
1520000
1530000
1540000
1550000
1560000
Chr. 4
10000
20000
30000
40000
50000
60000
Chr. 5
70000
80000
90000
100000
110000
120000
Chr. 5
6
4
2
0
−4

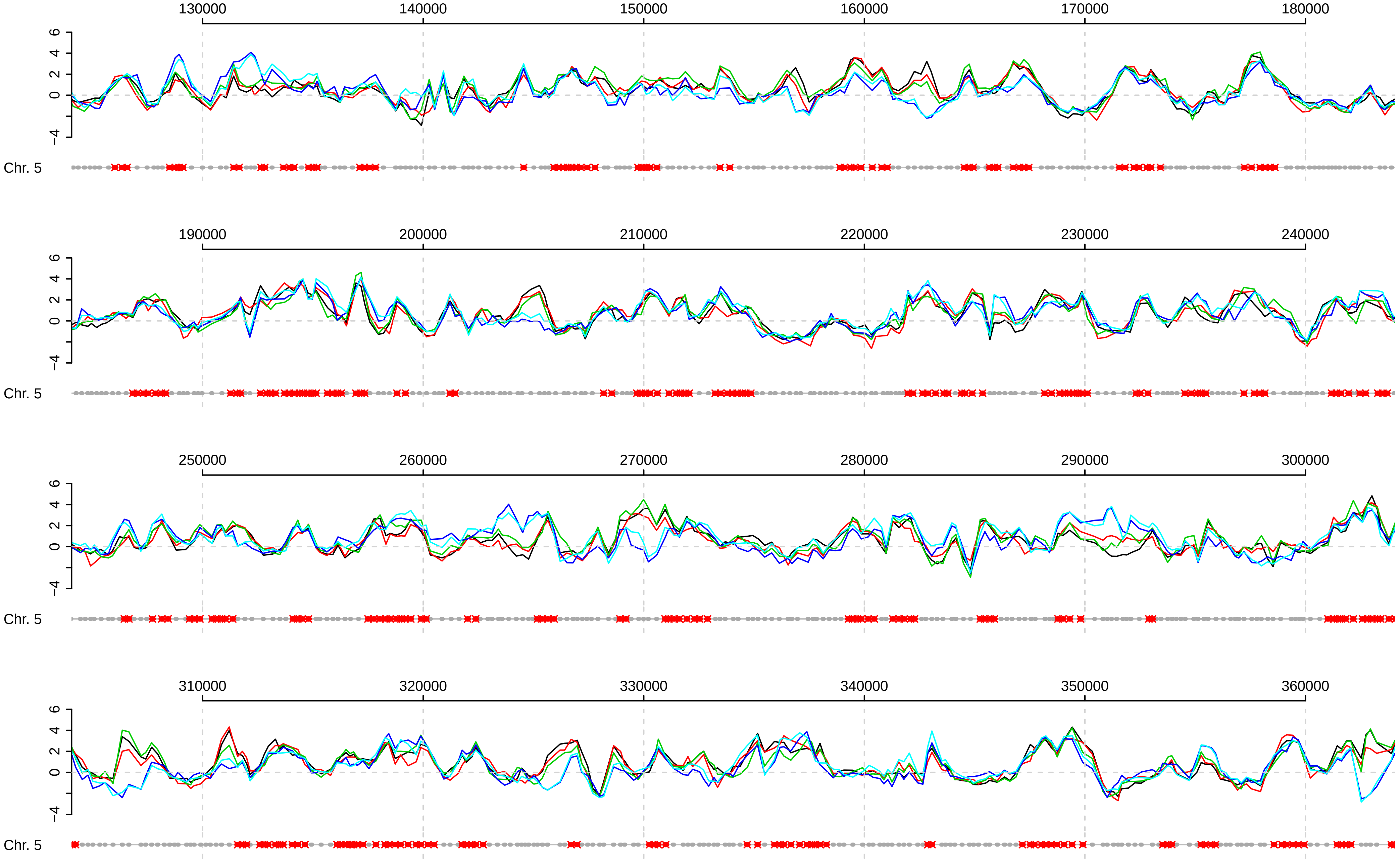

130000
140000
150000
160000
170000
180000
6
4
2
0
−4
Chr. 5
190000
200000
210000
220000
230000
240000
6
4
2
0
−4
Chr. 5
250000
260000
270000
280000
290000
300000
6
4
2
0
−4
Chr. 5
310000
320000
330000
340000
350000
360000
6
4
2
0
−4
Chr. 5

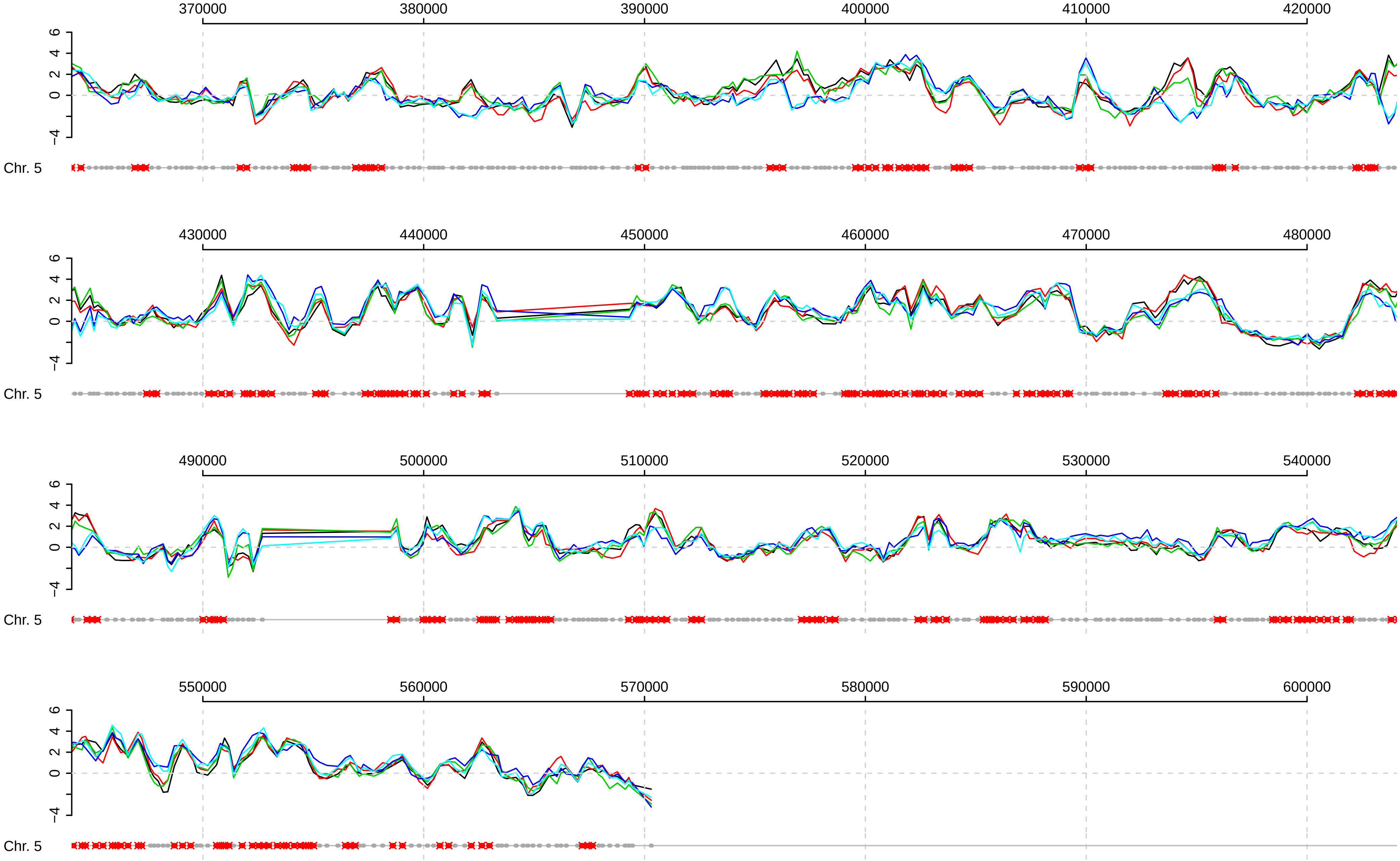

370000
380000
390000
400000
410000
420000
6
4
2
0
−4
Chr. 5
430000
440000
450000
460000
470000
480000
Chr. 5
490000
500000
510000
520000
530000
540000
Chr. 5
550000
560000
570000
580000
590000
600000
Chr. 5

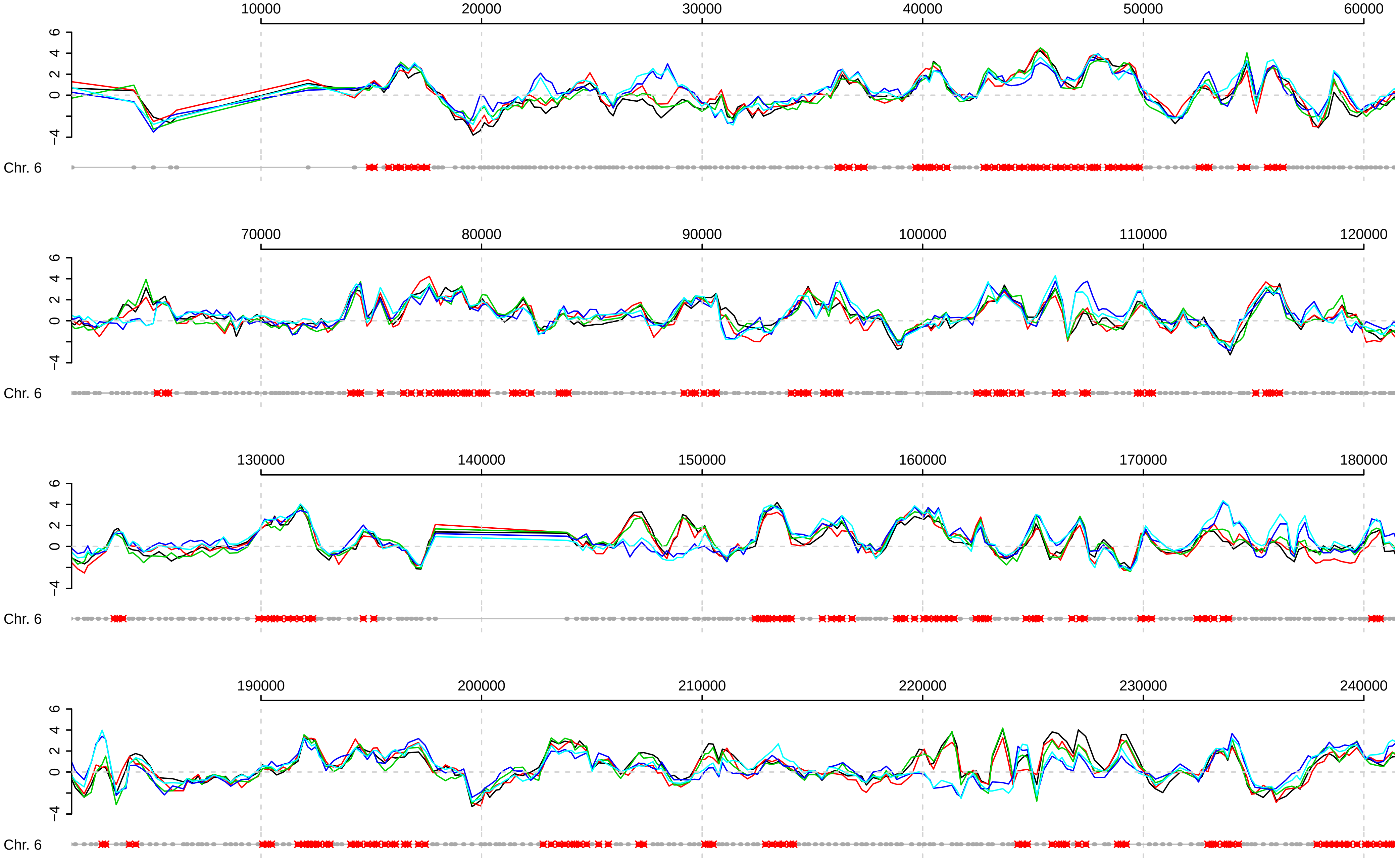
10000
20000
30000
40000
50000
60000
6
4
2
0
−4
Chr. 6
70000
80000
90000
100000
110000
120000
Chr. 6
130000
140000
150000
160000
170000
180000
Chr. 6
190000
200000
210000
220000
230000
240000
Chr. 6

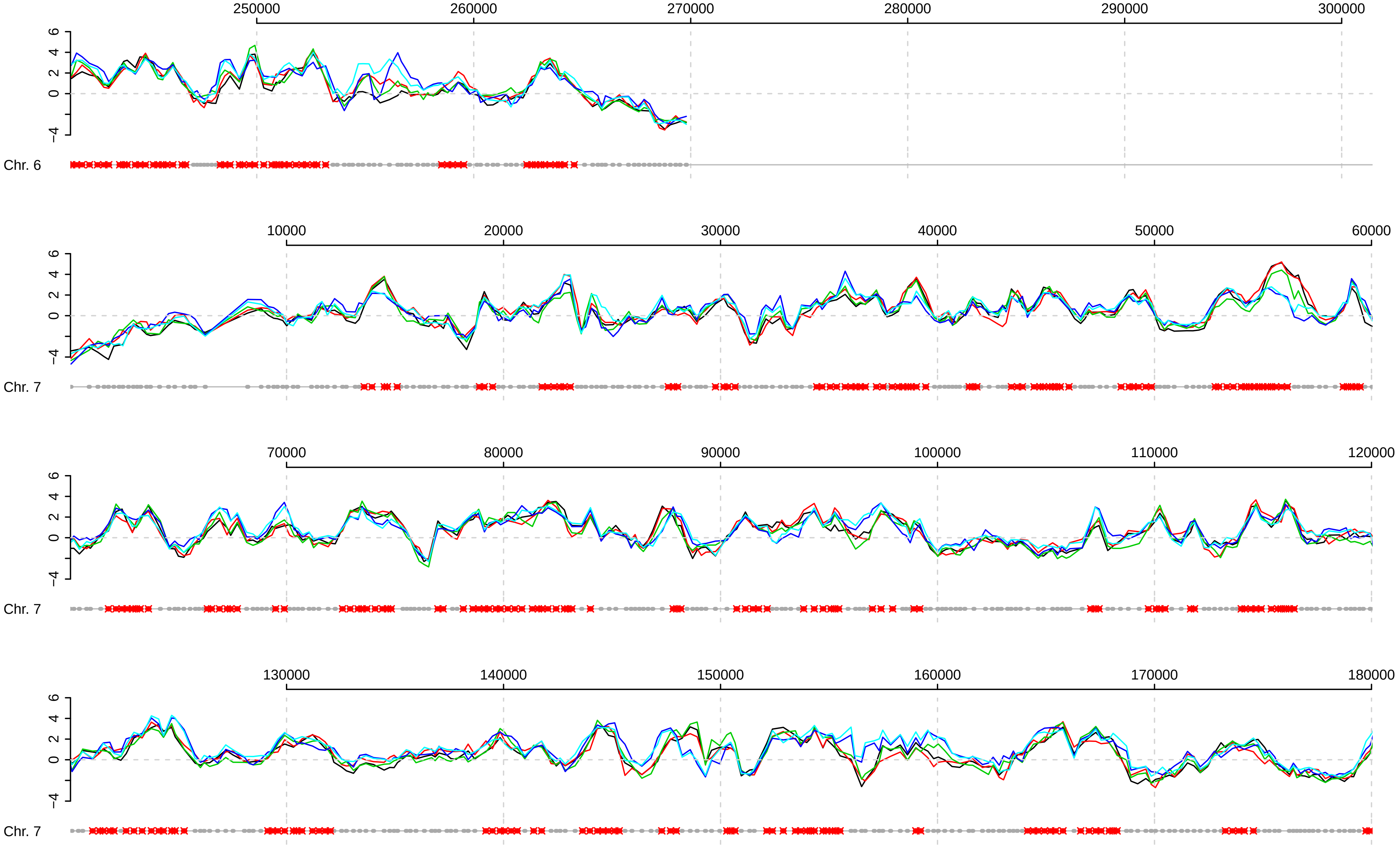
250000
260000
270000
280000
290000
300000
6
4
2
0
−4
Chr. 6
10000
20000
30000
40000
50000
60000
Chr. 7
70000
80000
90000
100000
110000
120000
Chr. 7
130000
140000
150000
160000
170000
180000
Chr. 7

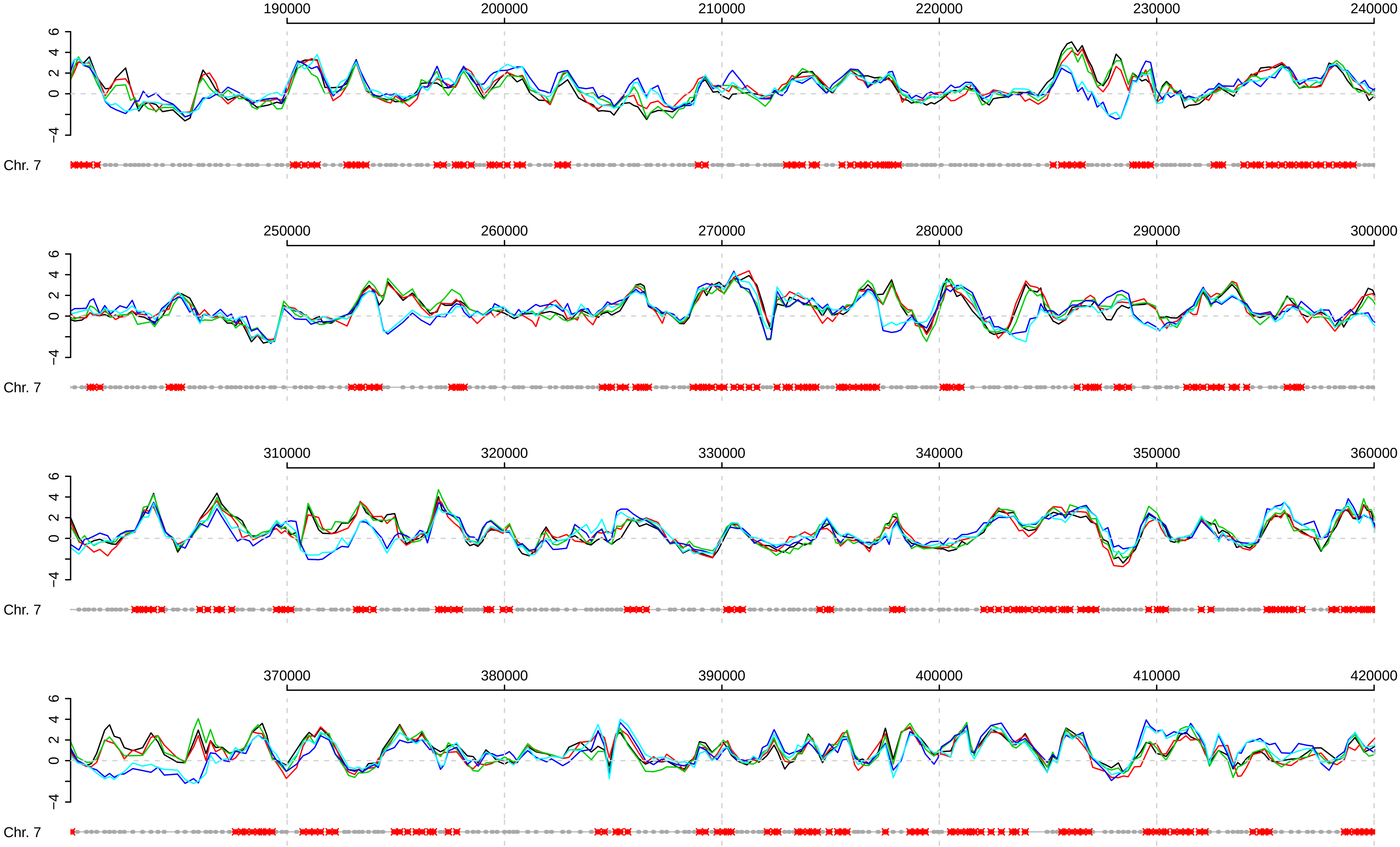

190000
200000
210000
220000
230000
240000
6
4
2
0
−4
Chr. 7
250000
260000
270000
280000
290000
300000
6
4
2
0
−4
Chr. 7
310000
320000
330000
340000
350000
360000
6
4
2
0
−4
Chr. 7
370000
380000
390000
400000
410000
420000
6
4
2
0
−4
Chr. 7

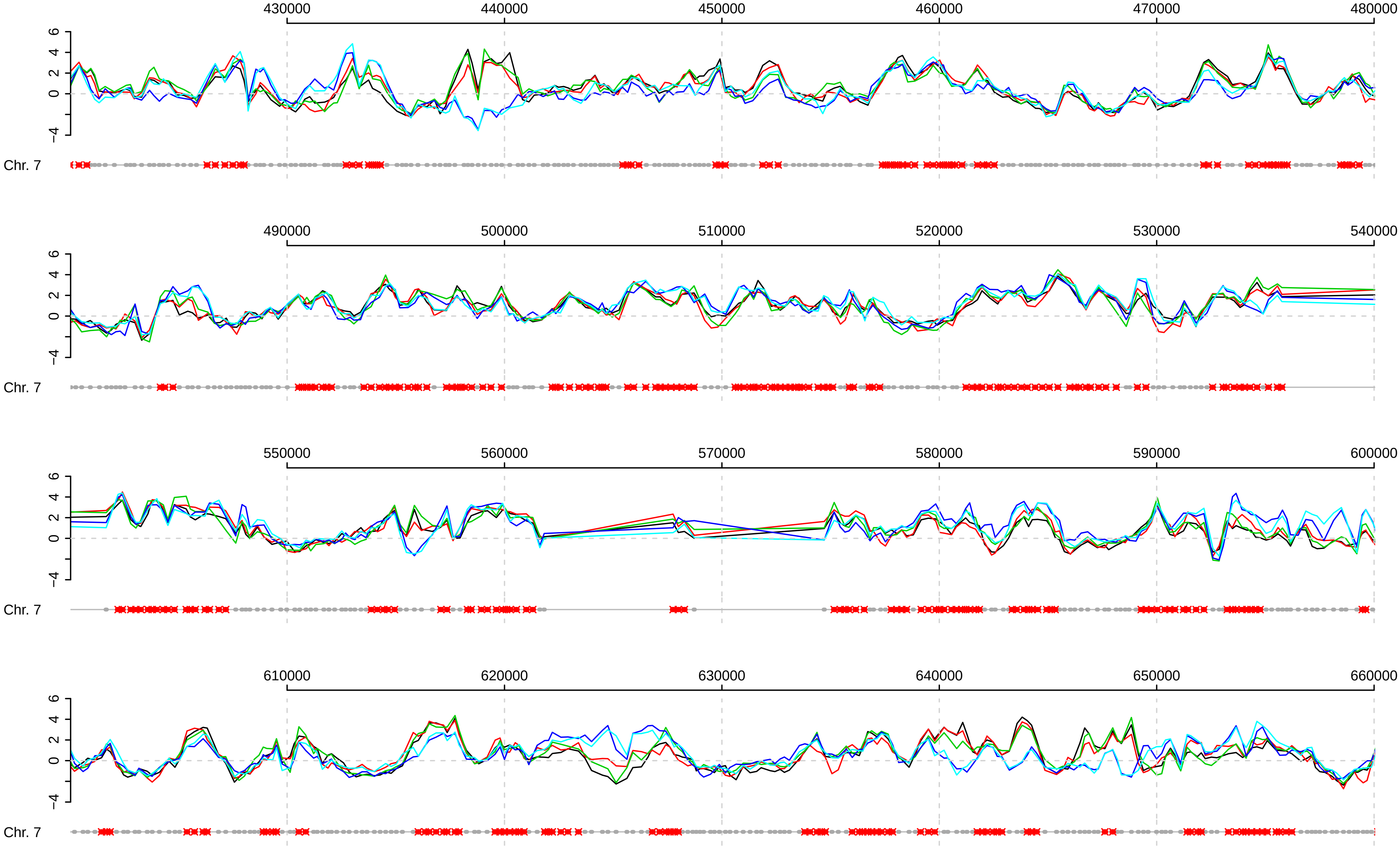

430000
440000
450000
460000
470000
480000
6
4
2
0
−4
Chr. 7
490000
500000
510000
520000
530000
540000
6
4
2
0
−4
Chr. 7
550000
560000
570000
580000
590000
600000
6
4
2
0
−4
Chr. 7
610000
620000
630000
640000
650000
660000
6
4
2
0
−4
Chr. 7

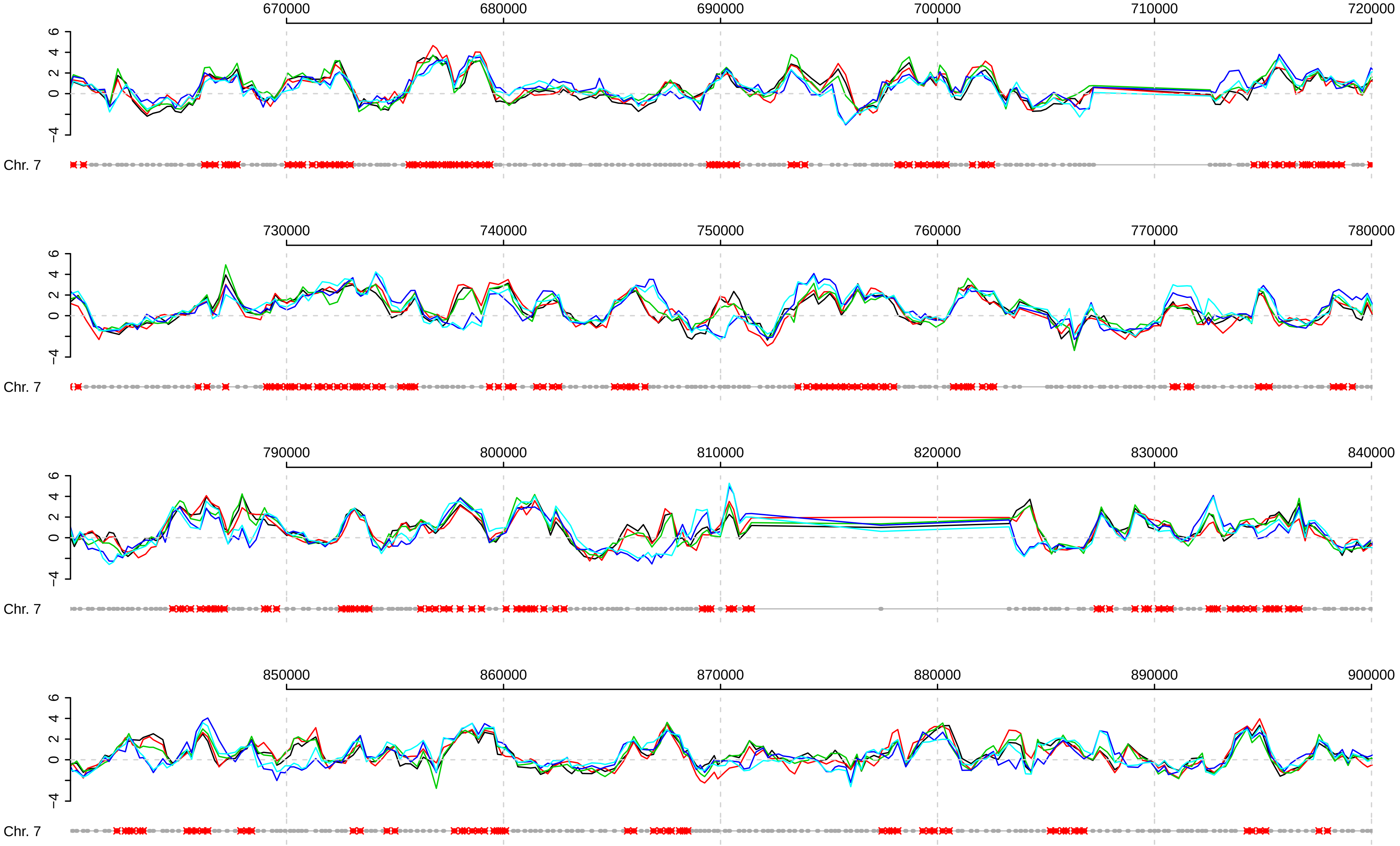
670000
680000
690000
700000
710000
720000
6
4
2
0
−4
Chr. 7
730000
740000
750000
760000
770000
780000
6
4
2
0
−4
Chr. 7
790000
800000
810000
820000
830000
840000
6
4
2
0
−4
Chr. 7
850000
860000
870000
880000
890000
900000
6
4
2
0
−4
Chr. 7

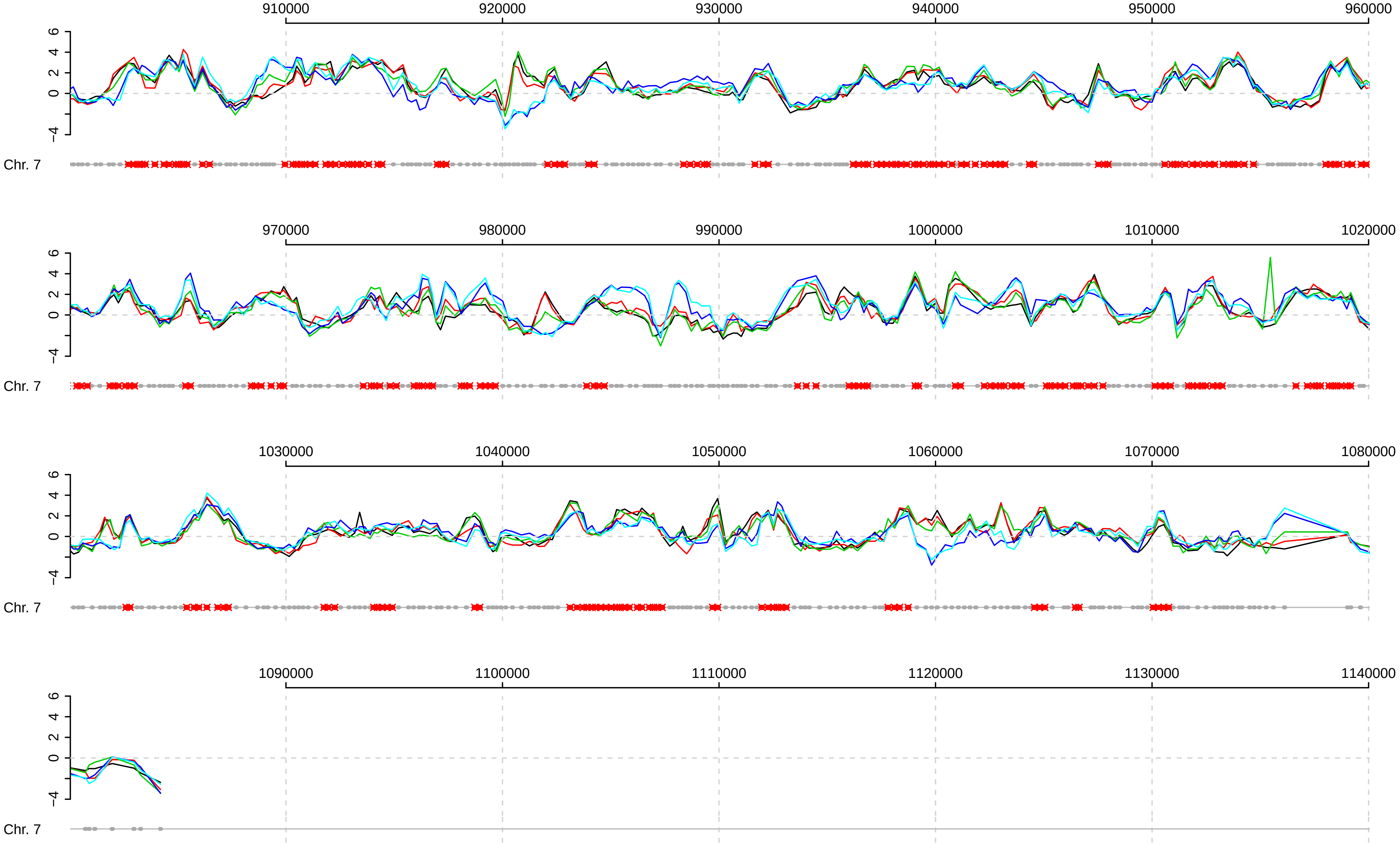

910000
920000
930000
940000
950000
960000
6
4
2
0
−4
Chr. 7
970000
980000
990000
1000000
1010000
1020000
Chr. 7
1030000
1040000
1050000
1060000
1070000
1080000
Chr. 7
1090000
1100000
1110000
1120000
1130000
1140000
Chr. 7

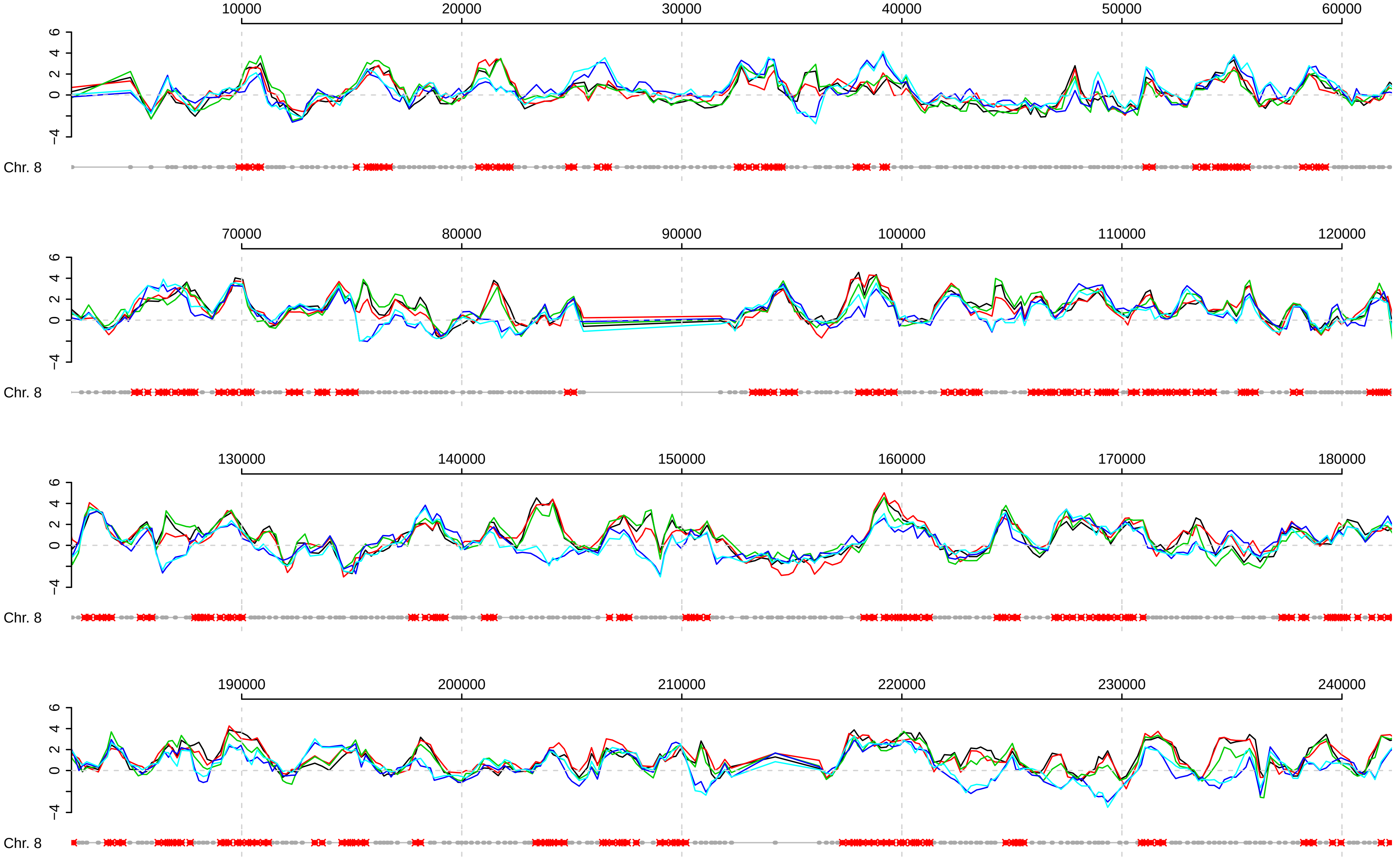

10000
20000
30000
40000
50000
60000
6
4
2
0
−4
Chr. 8
70000
80000
90000
100000
110000
120000
Chr. 8
130000
140000
150000
160000
170000
180000
Chr. 8
190000
200000
210000
220000
230000
240000
Chr. 8

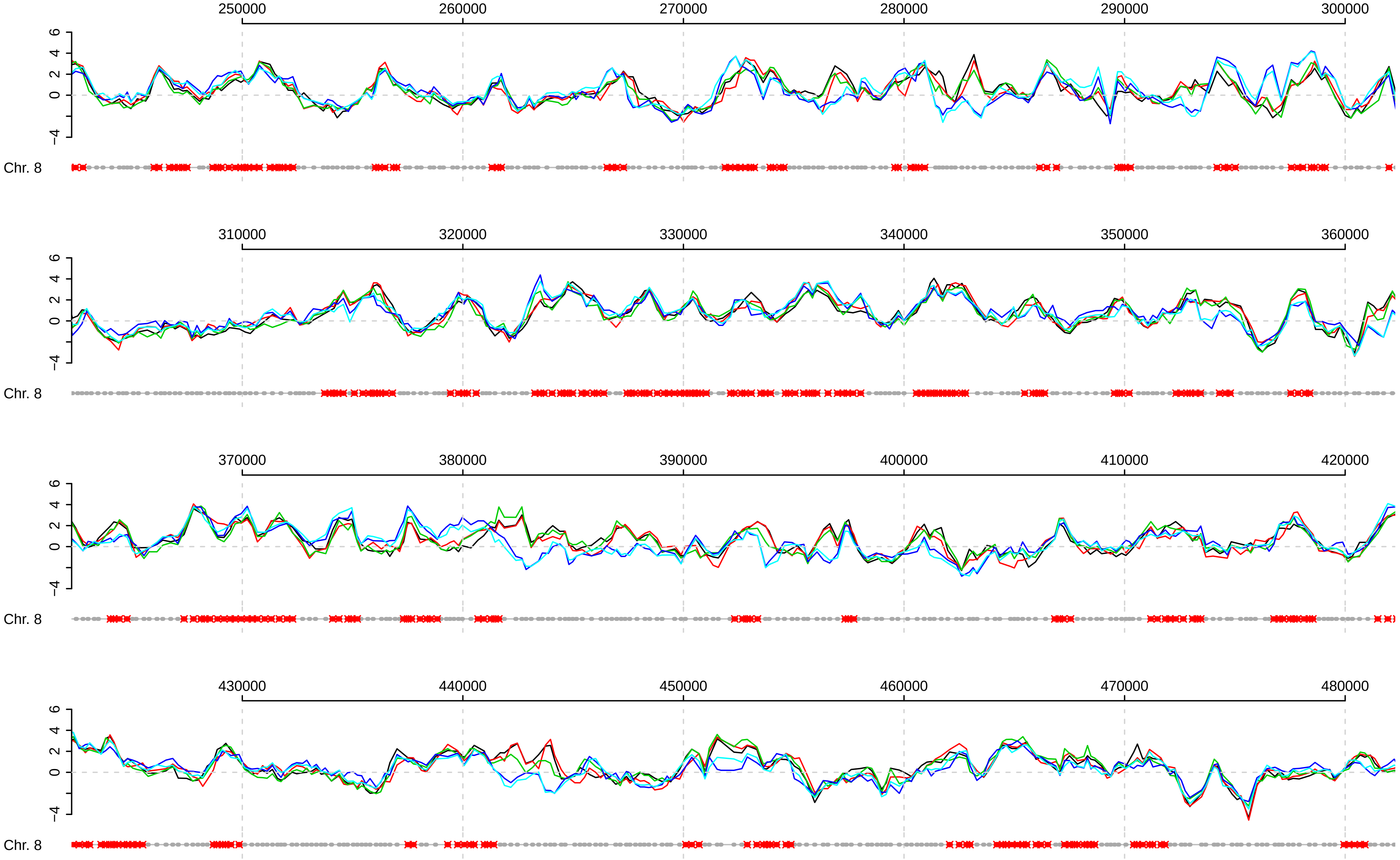

250000
260000
270000
280000
290000
300000
6
4
2
0
−4
Chr. 8
310000
320000
330000
340000
350000
360000
Chr. 8
370000
380000
390000
400000
410000
420000
Chr. 8
430000
440000
450000
460000
470000
480000
Chr. 8

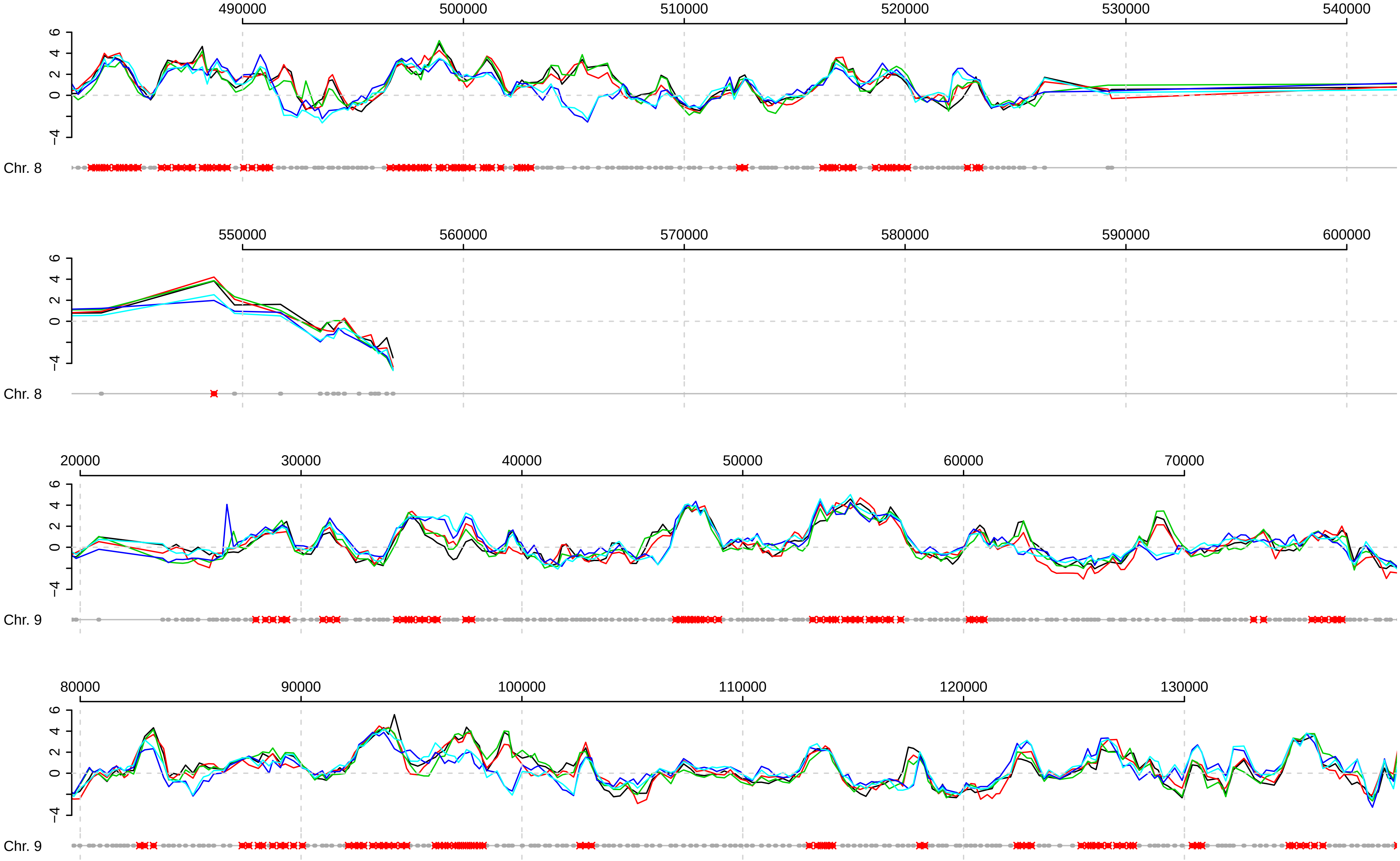

490000
500000
510000
520000
530000
540000
6
4
2
0
−4
Chr. 8
550000
560000
570000
580000
590000
600000
6
4
2
0
−4
Chr. 8
20000
30000
40000
50000
60000
70000
6
4
2
0
−4
Chr. 9
80000
90000
100000
110000
120000
130000
6
4
2
0
−4
Chr. 9

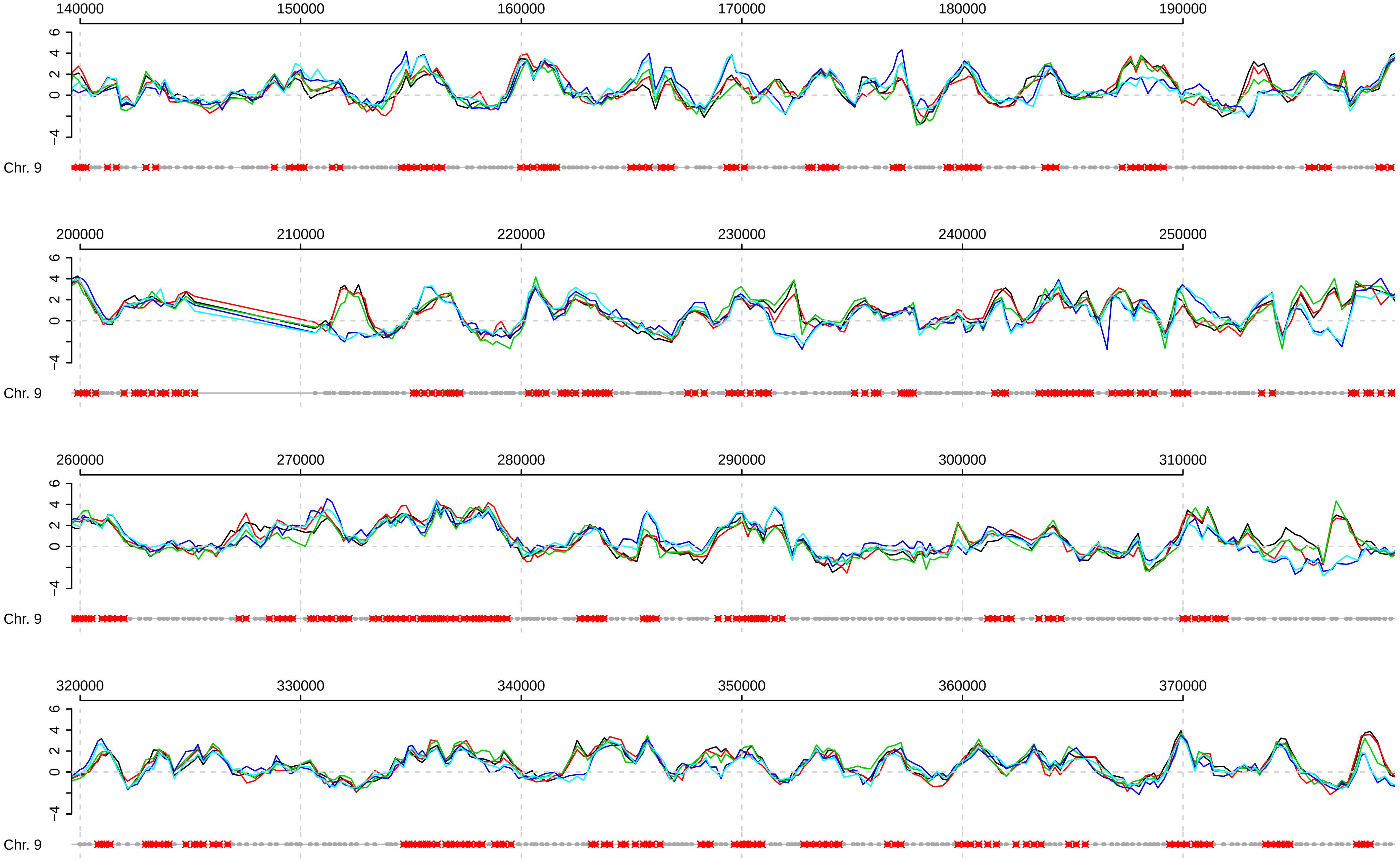
140000
150000
160000
170000
180000
190000
6
4
2
0
−4
Chr. 9
200000
210000
220000
230000
240000
250000
6
4
2
0
−4
Chr. 9
260000
270000
280000
290000
300000
310000
6
4
2
0
−4
Chr. 9
320000
330000
340000
350000
360000
370000
6
4
2
0
−4
Chr. 9

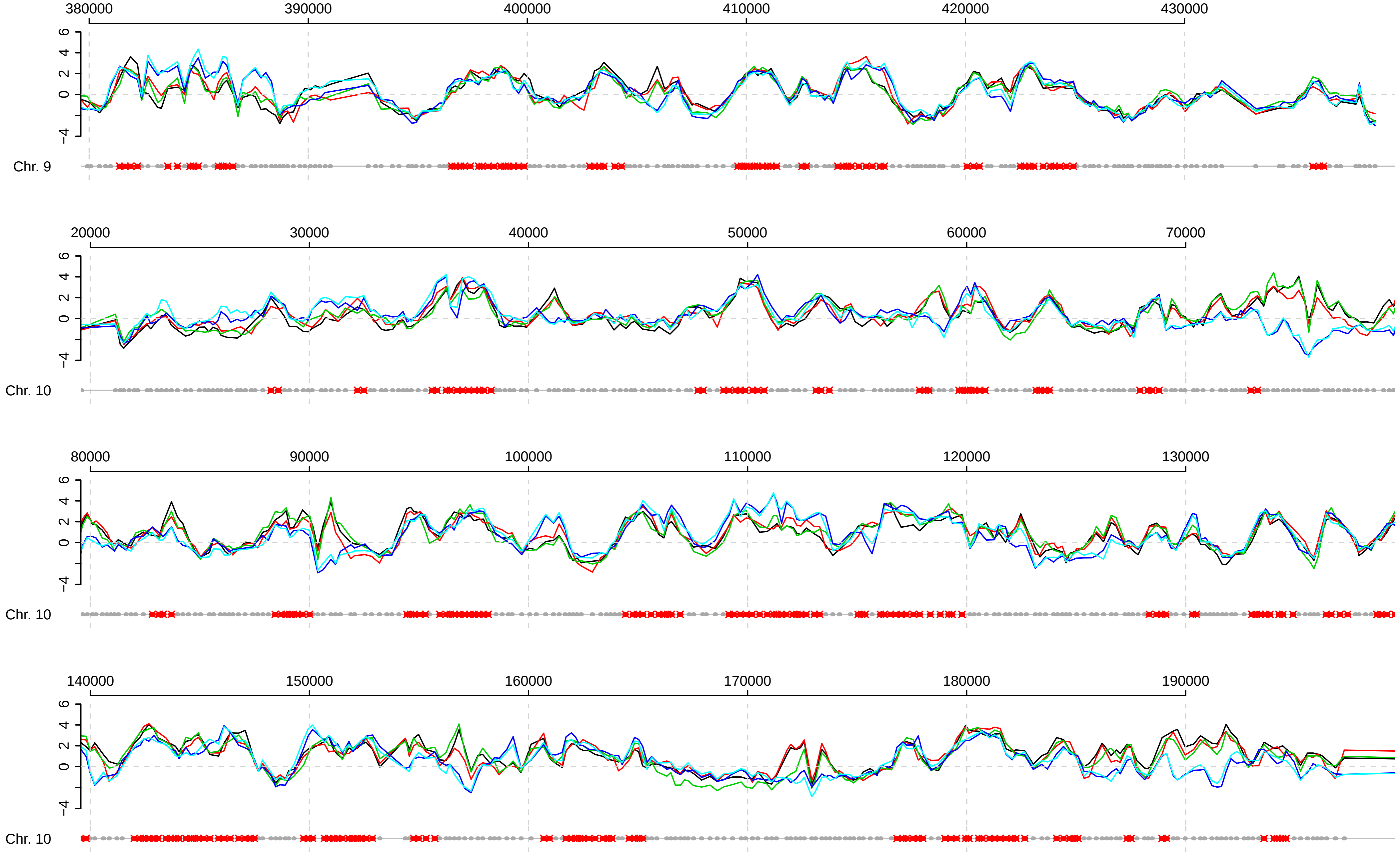
380000
390000
400000
410000
420000
430000
6
4
2
0
−4
Chr. 9
20000
30000
40000
50000
60000
70000
6
4
2
0
−4
Chr. 10
80000
90000
100000
110000
120000
130000
6
4
2
0
−4
Chr. 10
140000
150000
160000
170000
180000
190000
6
4
2
0
−4
Chr. 10

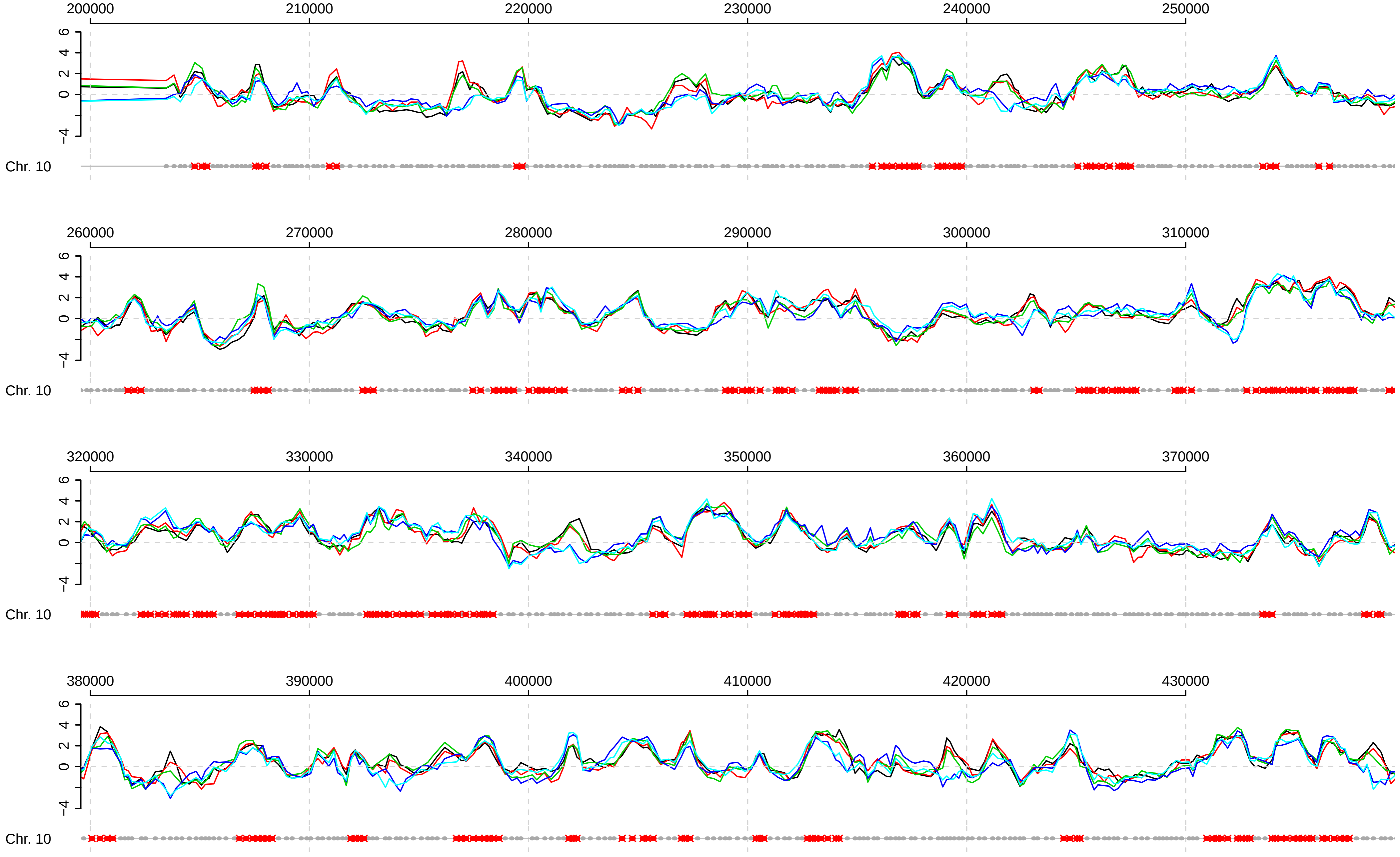

200000
210000
220000
230000
240000
250000
Chr. 10
260000
270000
280000
290000
300000
310000
Chr. 10
320000
330000
340000
350000
360000
370000
Chr. 10
380000
390000
400000
410000
420000
430000
Chr. 10

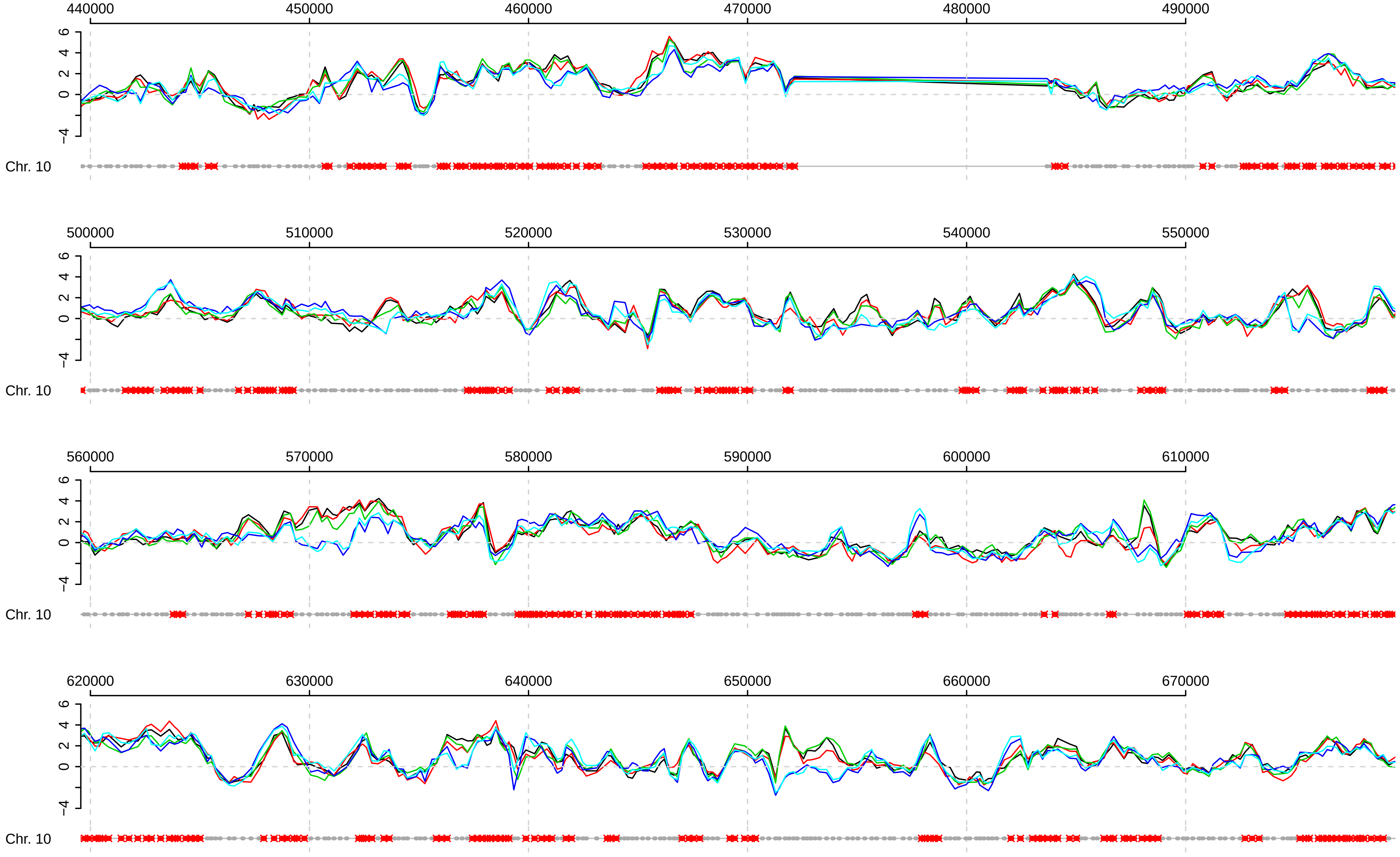

440000
450000
460000
470000
480000
490000
6
4
2
0
−4
Chr. 10
500000
510000
520000
530000
540000
550000
6
4
2
0
−4
Chr. 10
560000
570000
580000
590000
600000
610000
6
4
2
0
−4
Chr. 10
620000
630000
640000
650000
660000
670000
6
4
2
0
−4
Chr. 10

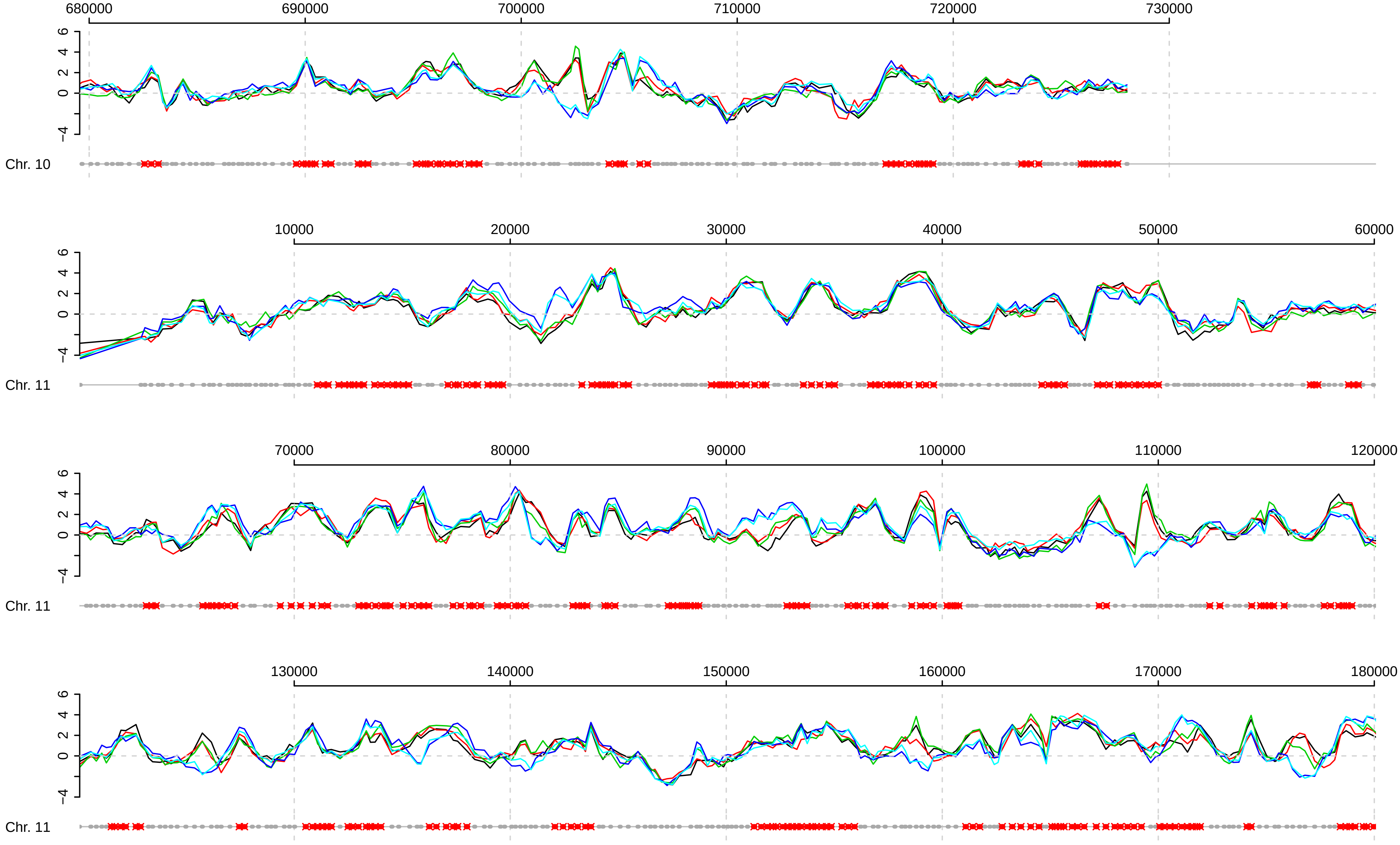

680000
690000
700000
710000
720000
730000
Chr. 10
10000
20000
30000
40000
50000
60000
Chr. 11
70000
80000
90000
100000
110000
120000
Chr. 11
130000
140000
150000
160000
170000
180000
Chr. 11

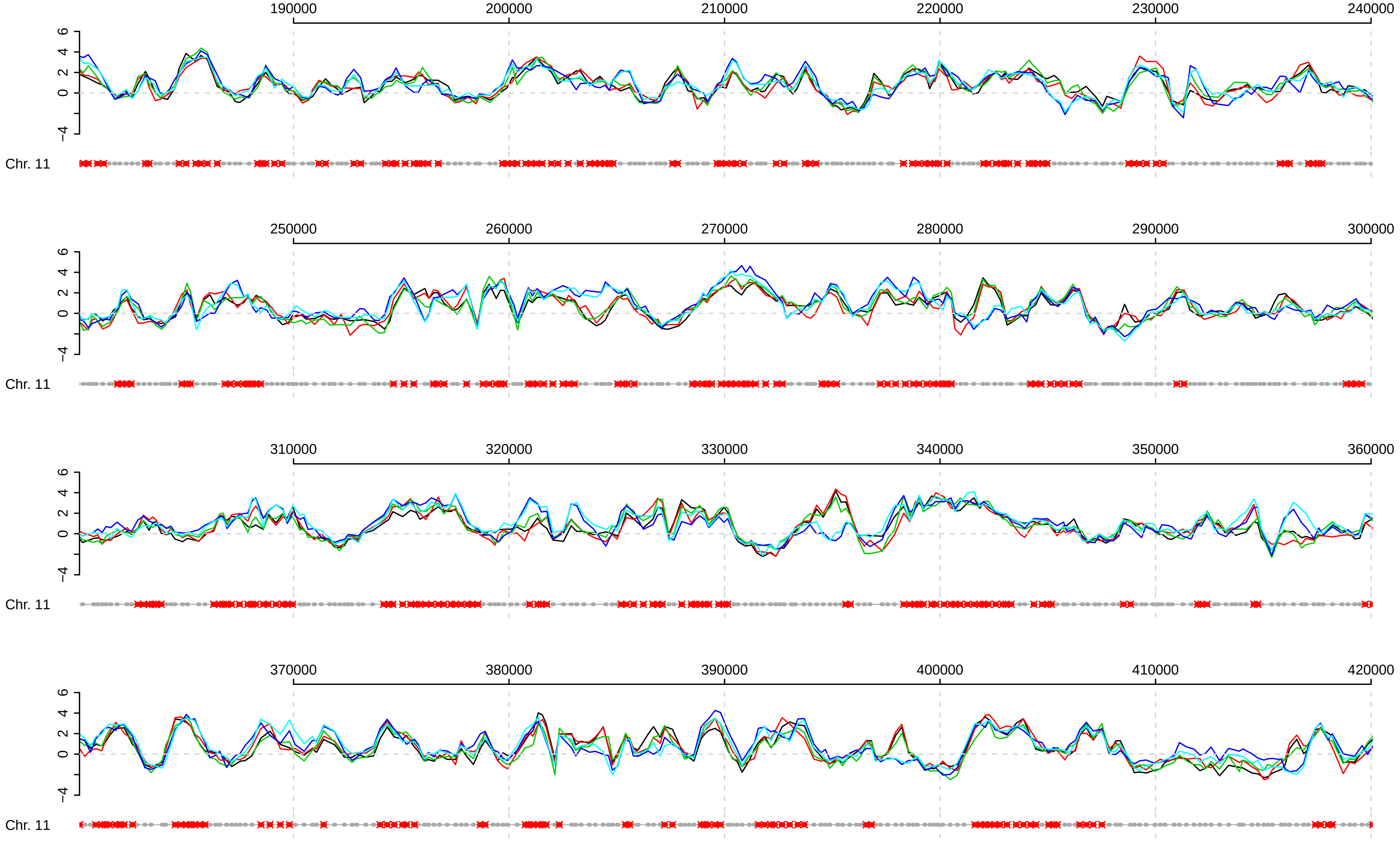
190000
200000
210000
220000
230000
240000
6
4
2
0
−4
Chr. 11
250000
260000
270000
280000
290000
300000
6
4
2
0
−4
Chr. 11
310000
320000
330000
340000
350000
360000
6
4
2
0
−4
Chr. 11
370000
380000
390000
400000
410000
420000
6
4
2
0
−4
Chr. 11

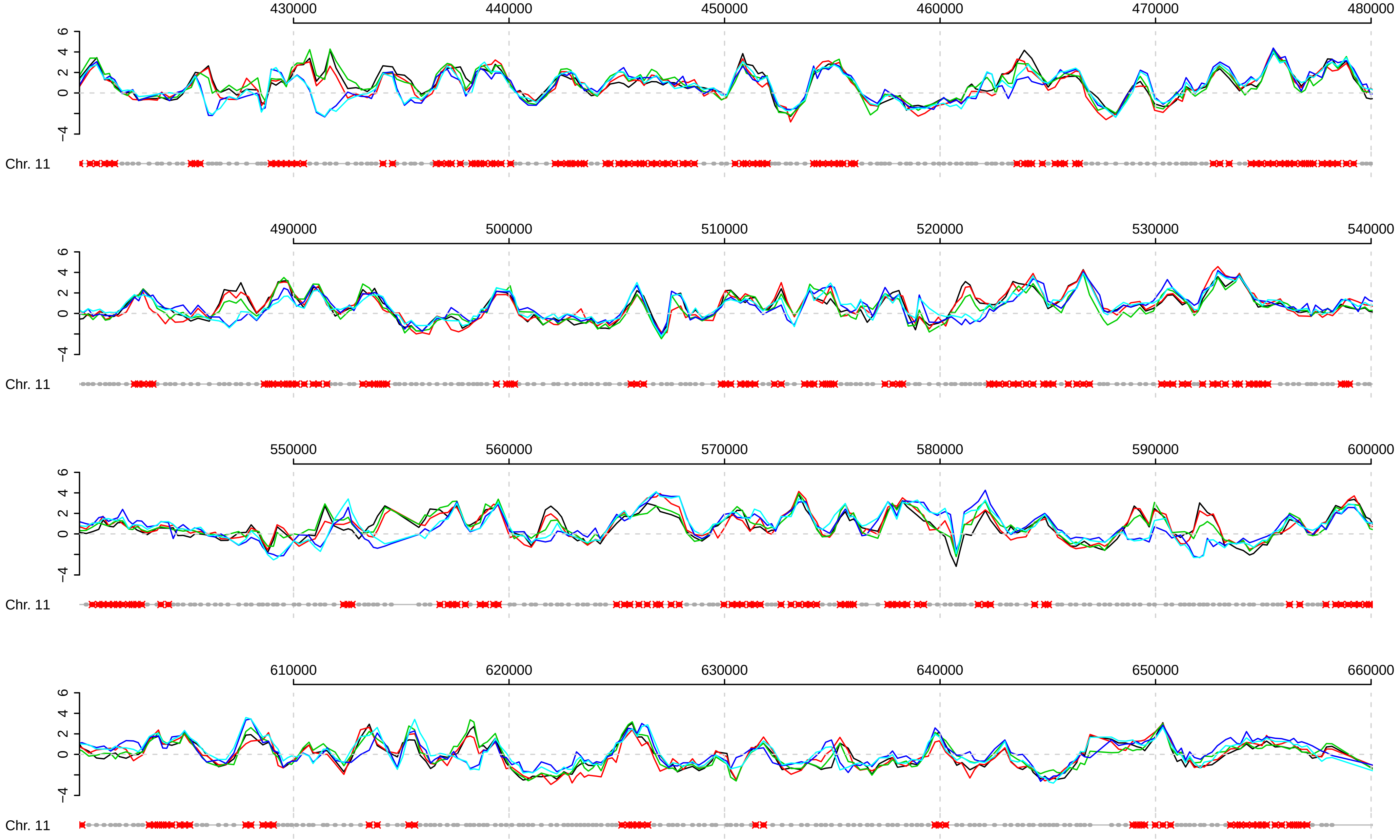

430000
440000
450000
460000
470000
480000
Chr. 11
490000
500000
510000
520000
530000
540000
Chr. 11
550000
560000
570000
580000
590000
600000
Chr. 11
610000
620000
630000
640000
650000
660000
Chr. 11

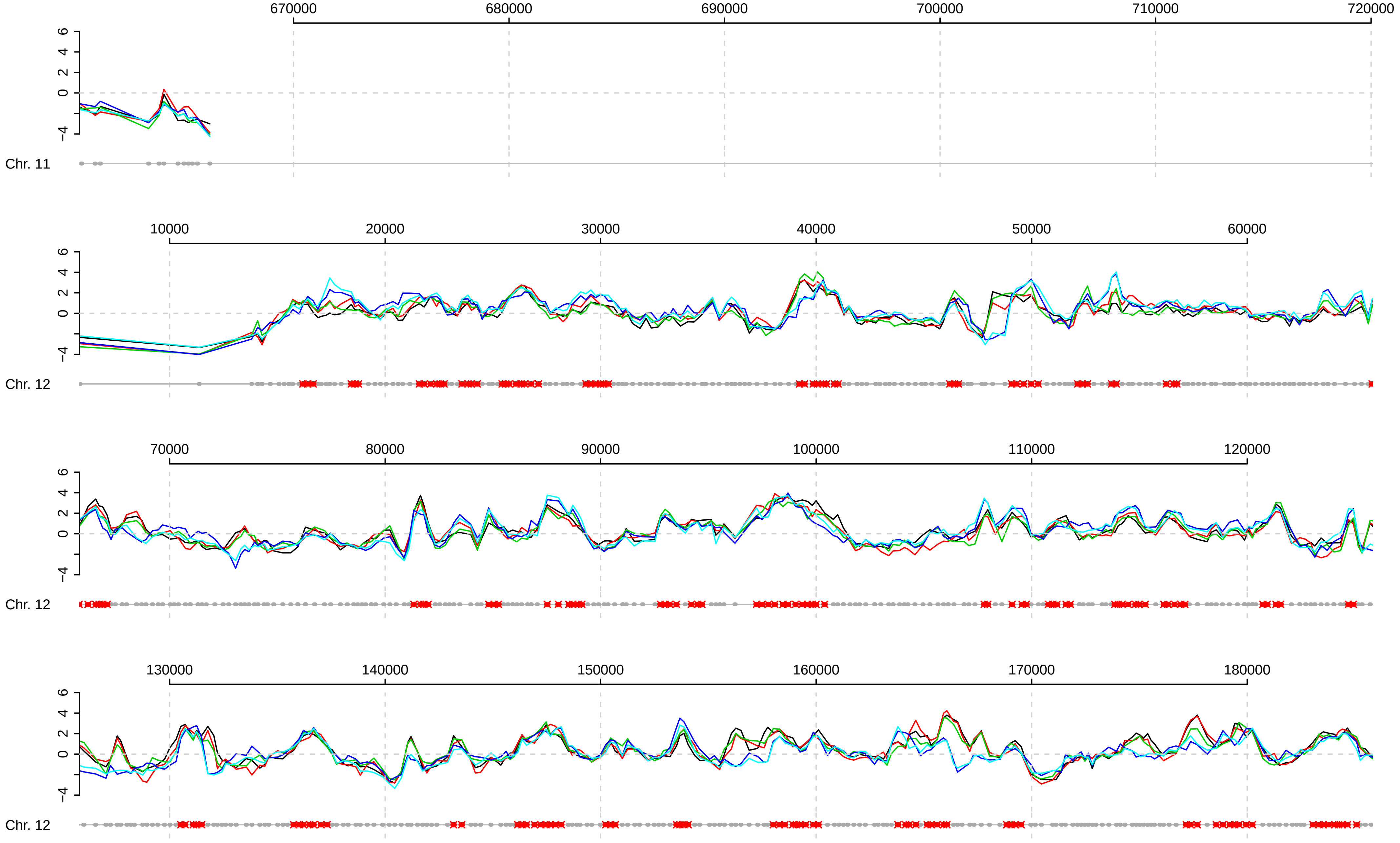

670000
680000
690000
700000
710000
720000
6
4
2
0
−4
Chr. 11
10000
20000
30000
40000
50000
60000
Chr. 12
70000
80000
90000
100000
110000
120000
Chr. 12
130000
140000
150000
160000
170000
180000
Chr. 12

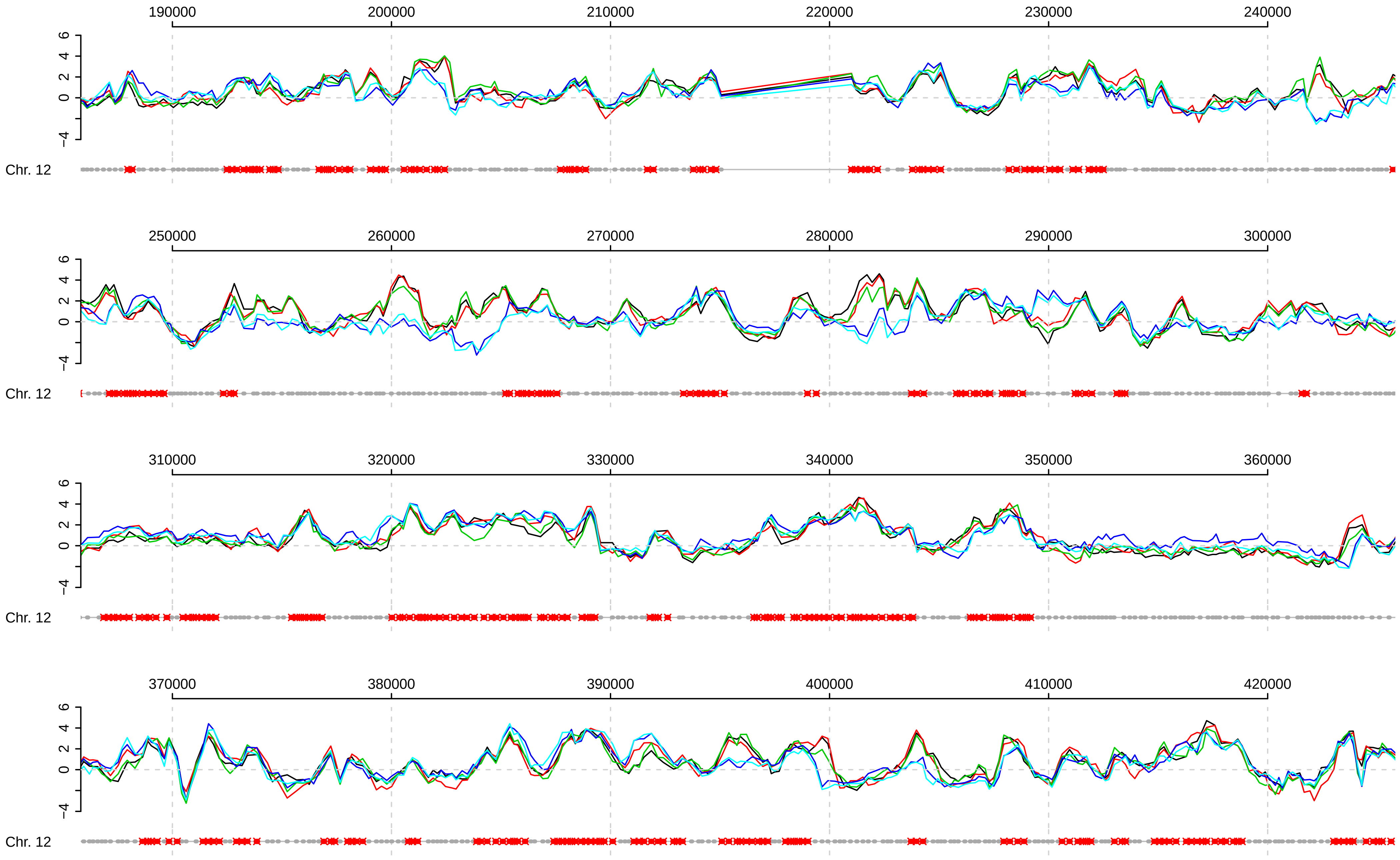

190000
200000
210000
220000
230000
240000
6
4
2
0
−4
Chr. 12
250000
260000
270000
280000
290000
300000
Chr. 12
310000
320000
330000
340000
350000
360000
Chr. 12
370000
380000
390000
400000
410000
420000
Chr. 12

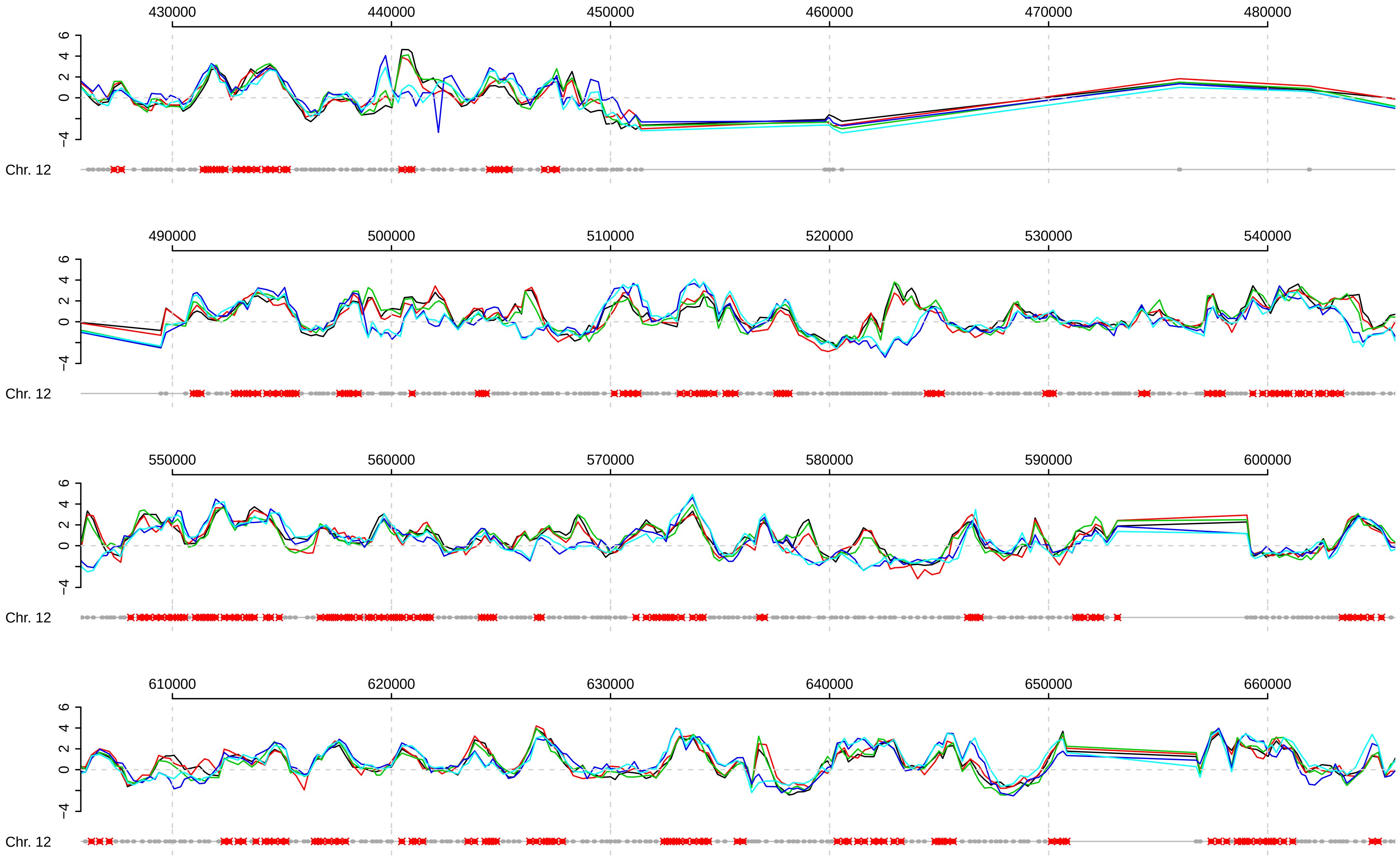

430000
440000
450000
460000
470000
480000
6
4
2
0
−4
Chr. 12
490000
500000
510000
520000
530000
540000
6
4
2
0
−4
Chr. 12
550000
560000
570000
580000
590000
600000
6
4
2
0
−4
Chr. 12
610000
620000
630000
640000
650000
660000
6
4
2
0
−4
Chr. 12

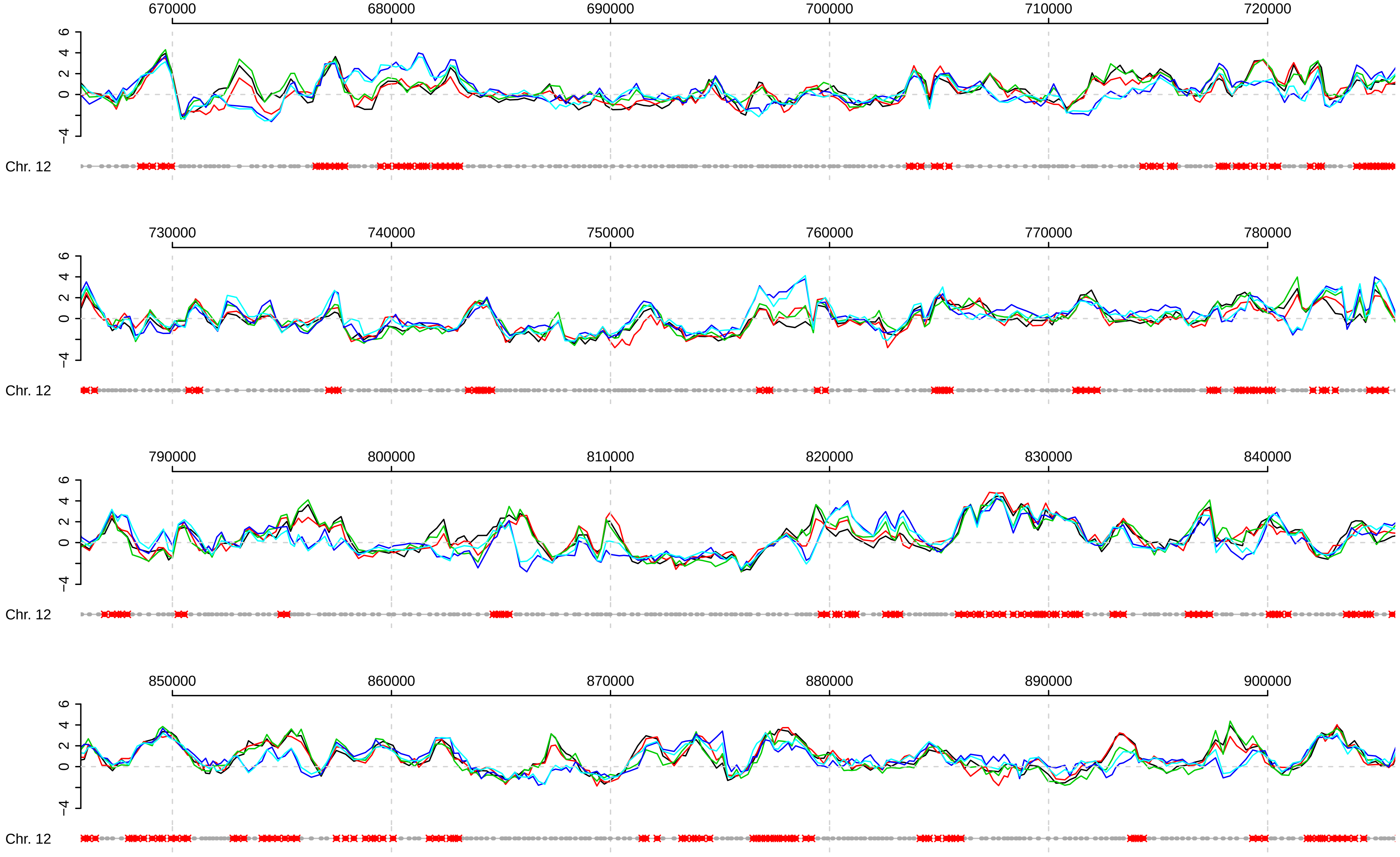
670000
680000
690000
700000
710000
720000
730000
740000
750000
760000
770000
780000
790000
800000
810000
820000
830000
840000
850000
860000
870000
880000
890000
900000
−4
0
2
4
6
Chr. 12

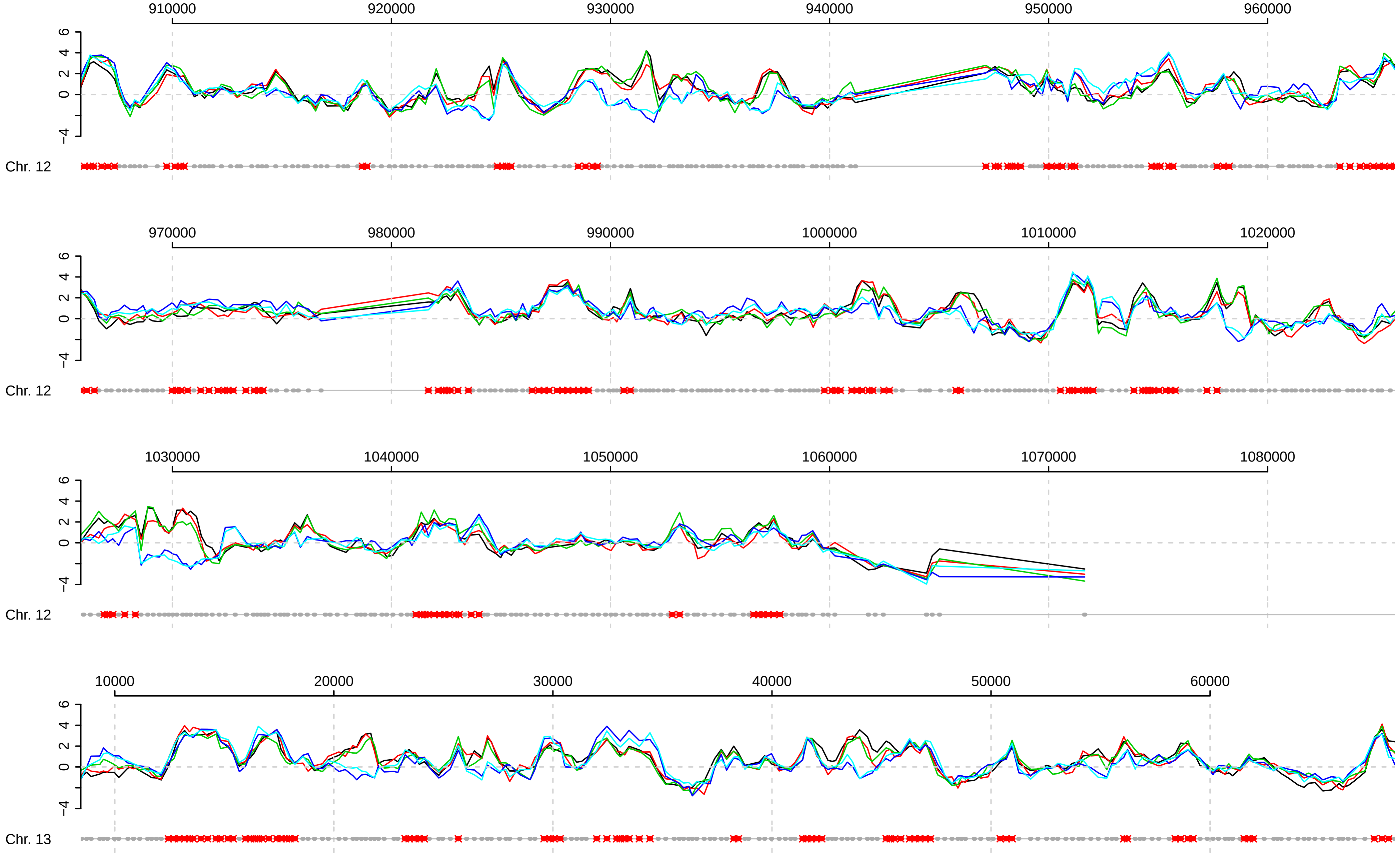
910000
920000
930000
940000
950000
960000
6
4
2
0
−4
Chr. 12
970000
980000
990000
1000000
1010000
1020000
6
4
2
0
−4
Chr. 12
1030000
1040000
1050000
1060000
1070000
1080000
6
4
2
0
−4
Chr. 12
10000
20000
30000
40000
50000
60000
6
4
2
0
−4
Chr. 13

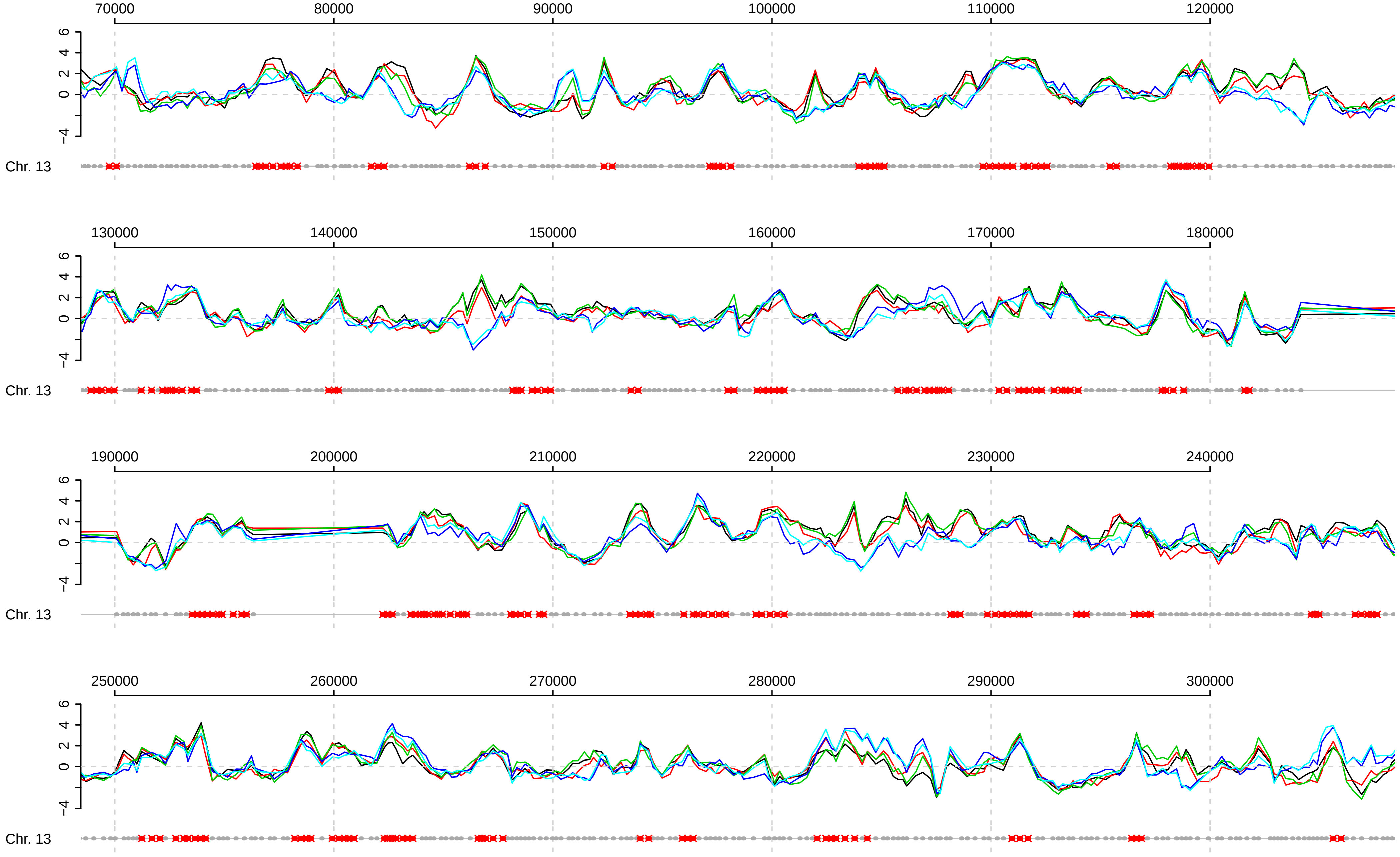

70000
80000
90000
100000
110000
120000
Chr. 13
130000
140000
150000
160000
170000
180000
Chr. 13
190000
200000
210000
220000
230000
240000
Chr. 13
250000
260000
270000
280000
290000
300000
Chr. 13

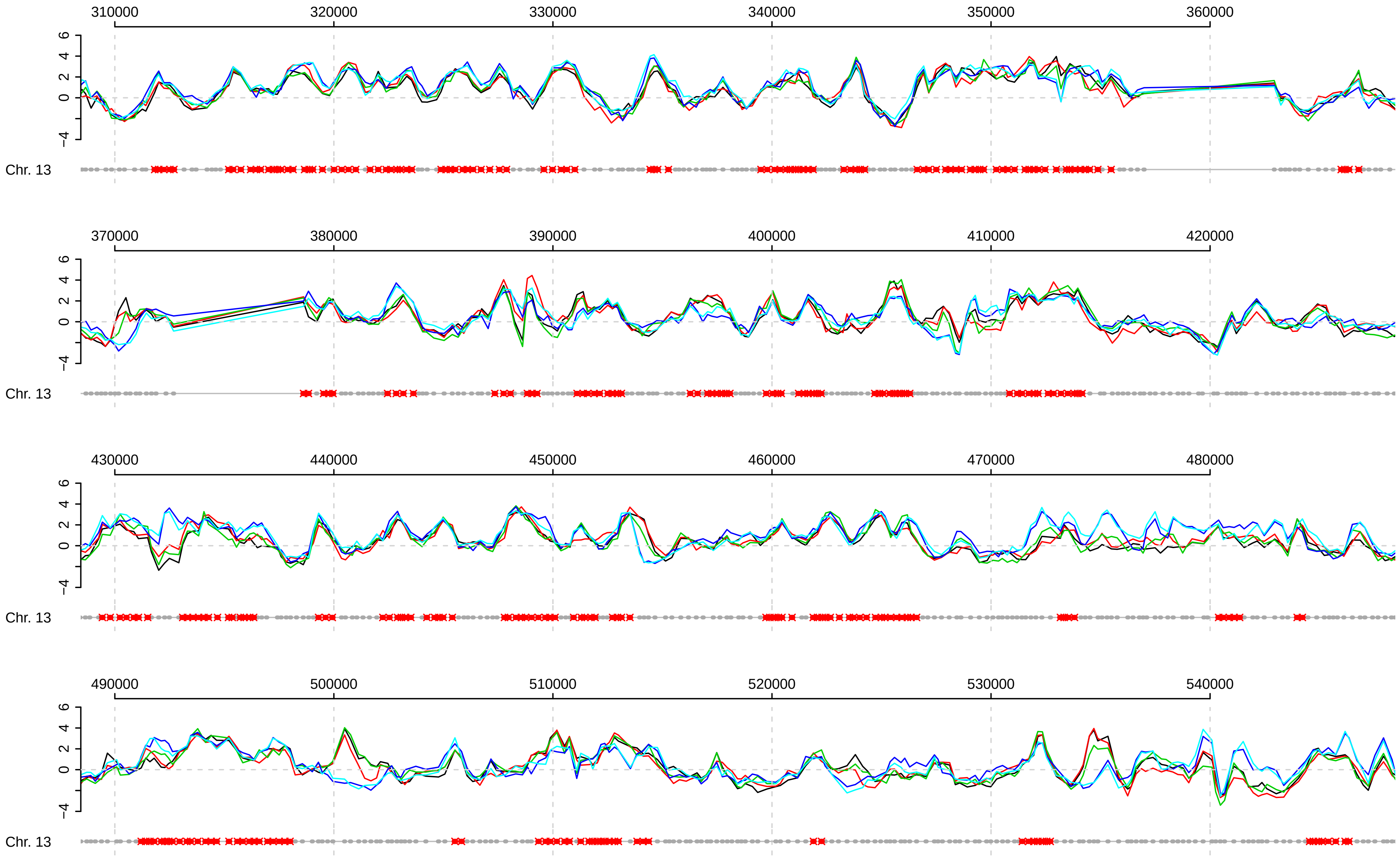

310000
320000
330000
340000
350000
360000
6
4
2
0
−4
Chr. 13
370000
380000
390000
400000
410000
420000
Chr. 13
430000
440000
450000
460000
470000
480000
Chr. 13
490000
500000
510000
520000
530000
540000
Chr. 13

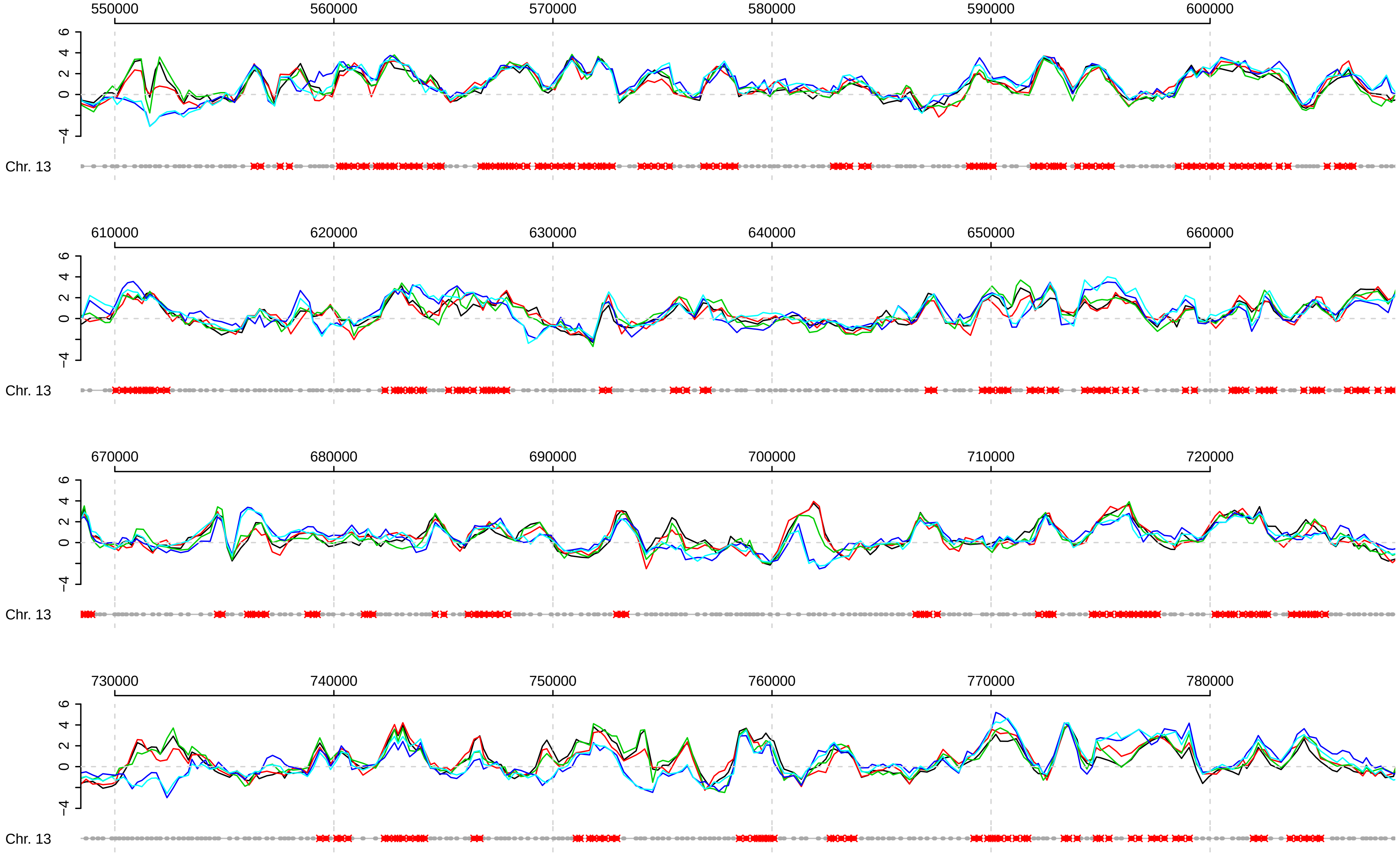
550000
560000
570000
580000
590000
600000
Chr. 13
610000
620000
630000
640000
650000
660000
Chr. 13
670000
680000
690000
700000
710000
720000
Chr. 13
730000
740000
750000
760000
770000
780000
Chr. 13

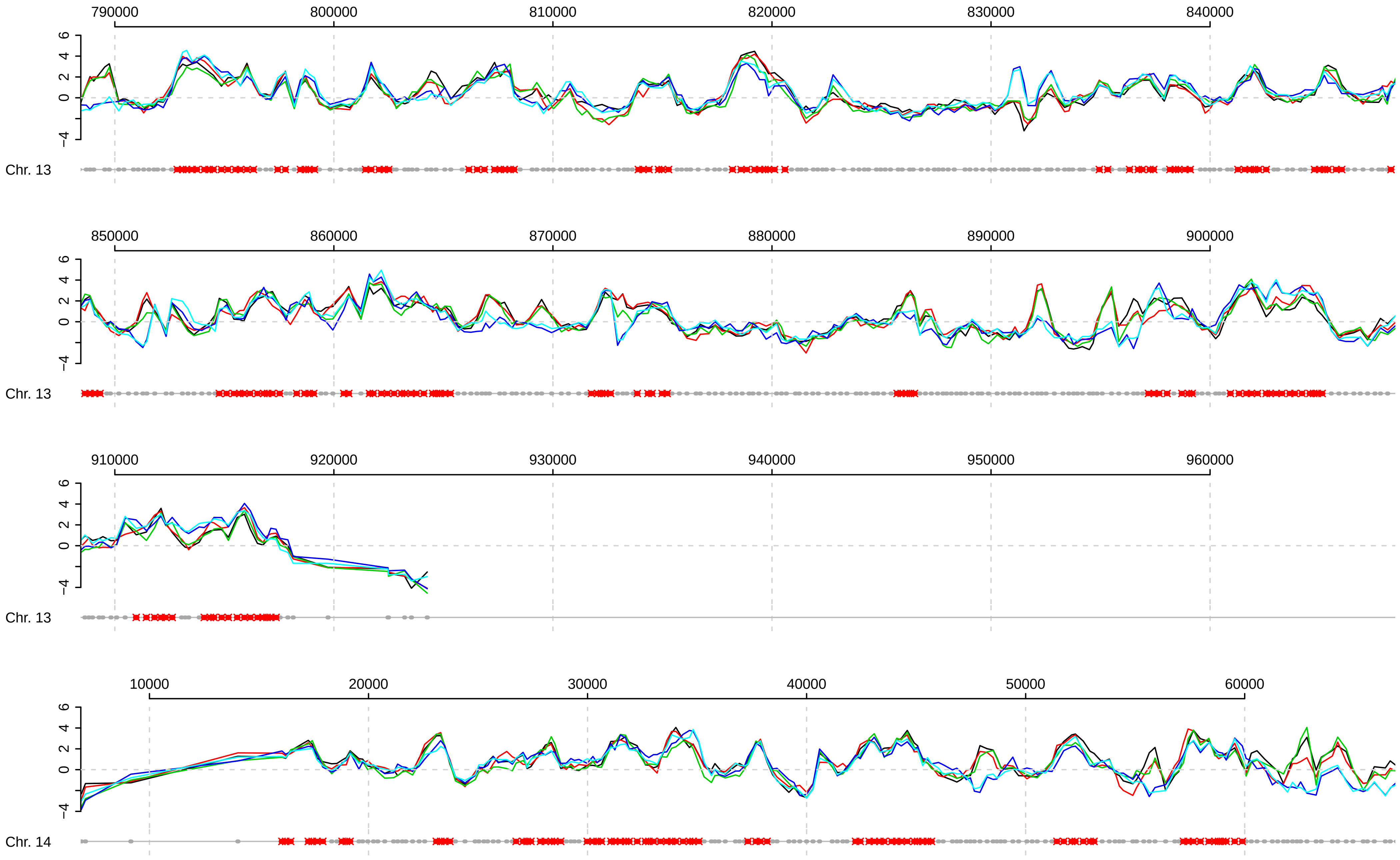

790000
800000
810000
820000
830000
840000
Chr. 13
850000
860000
870000
880000
890000
900000
Chr. 13
910000
920000
930000
940000
950000
960000
Chr. 13
10000
20000
30000
40000
50000
60000
Chr. 14

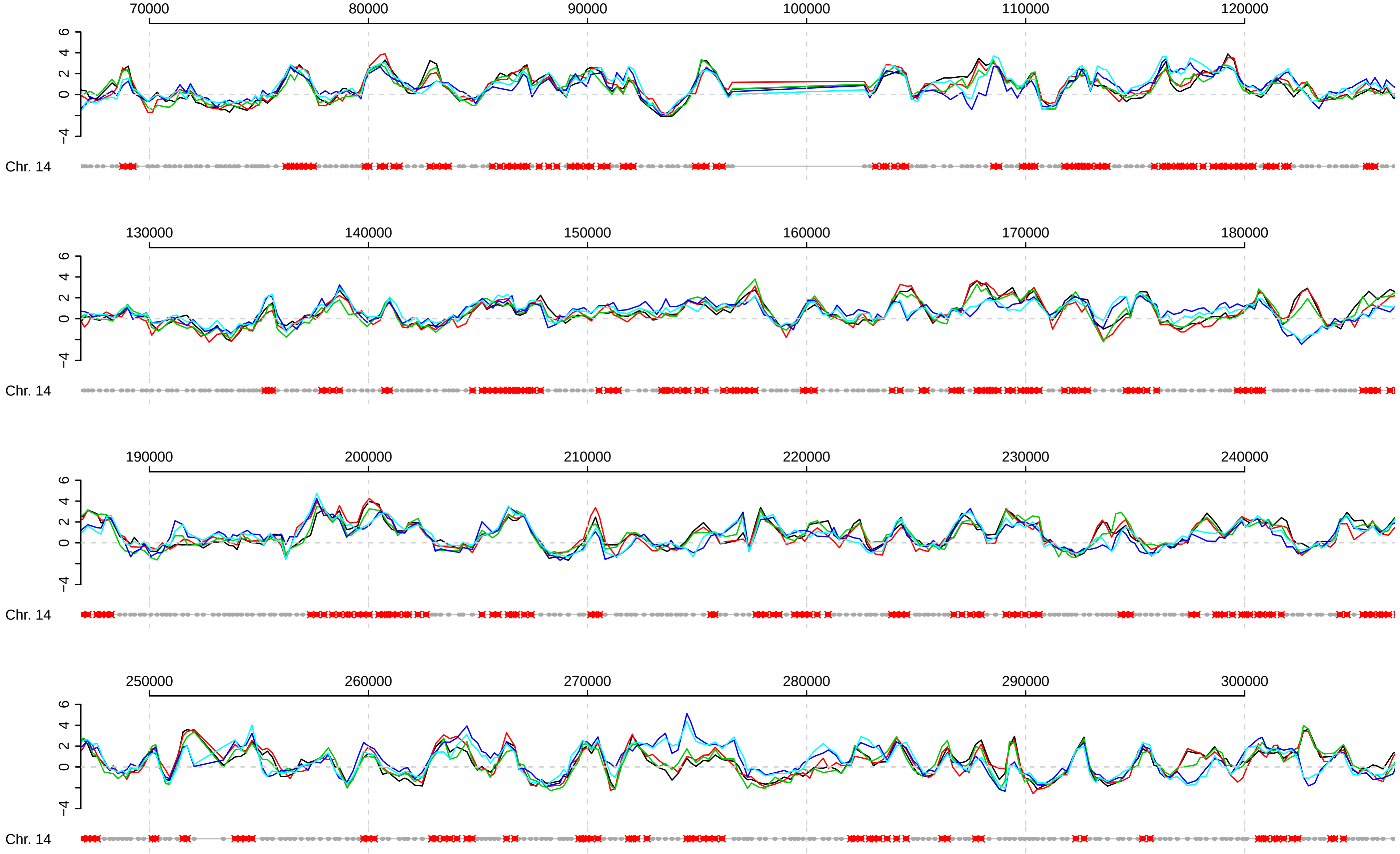

70000
80000
90000
100000
110000
120000
6
4
2
0
−4
Chr. 14
130000
140000
150000
160000
170000
180000
6
4
2
0
−4
Chr. 14
190000
200000
210000
220000
230000
240000
6
4
2
0
−4
Chr. 14
250000
260000
270000
280000
290000
300000
6
4
2
0
−4
Chr. 14

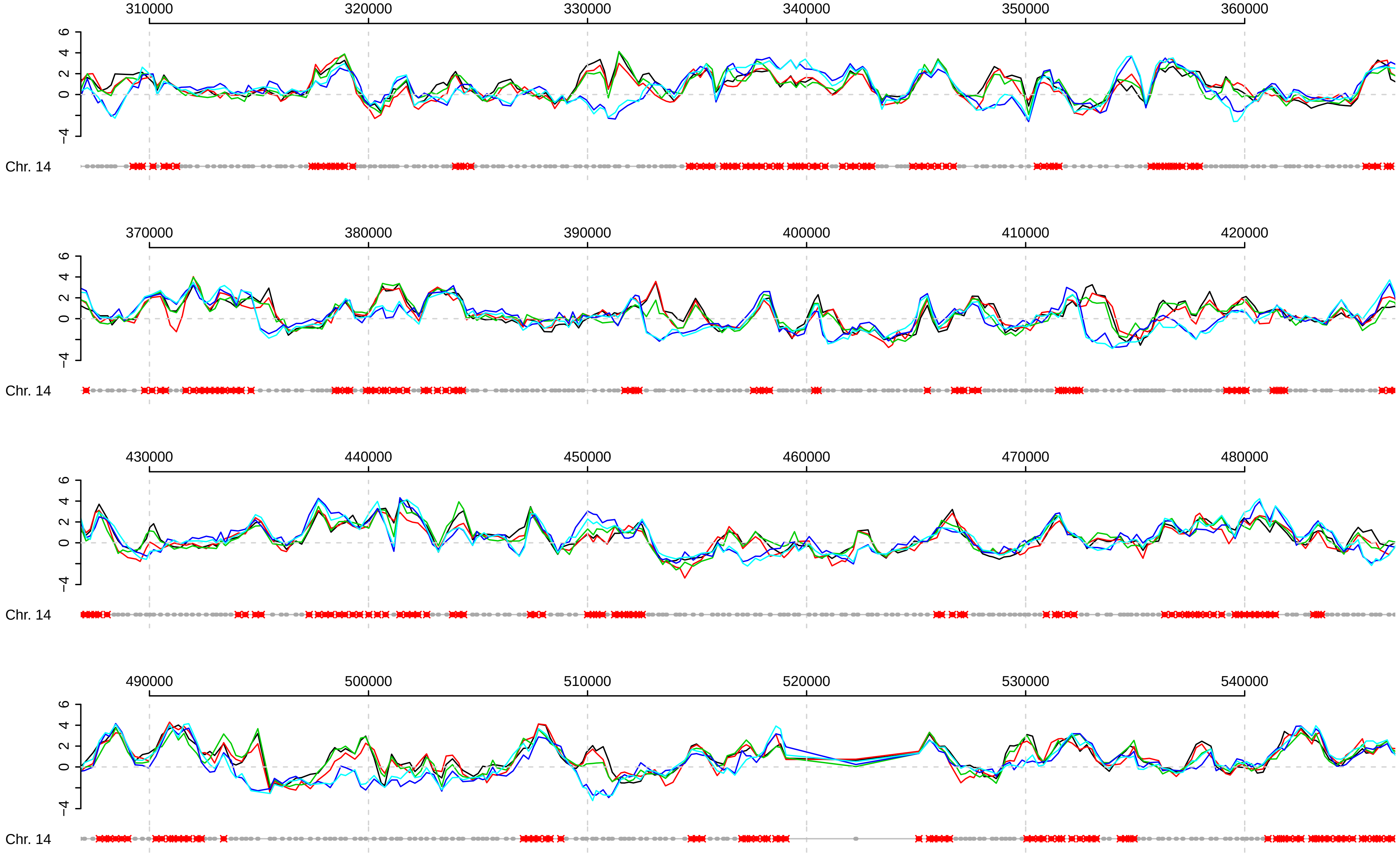

310000
320000
330000
340000
350000
360000
Chr. 14
370000
380000
390000
400000
410000
420000
Chr. 14
430000
440000
450000
460000
470000
480000
Chr. 14
490000
500000
510000
520000
530000
540000
Chr. 14

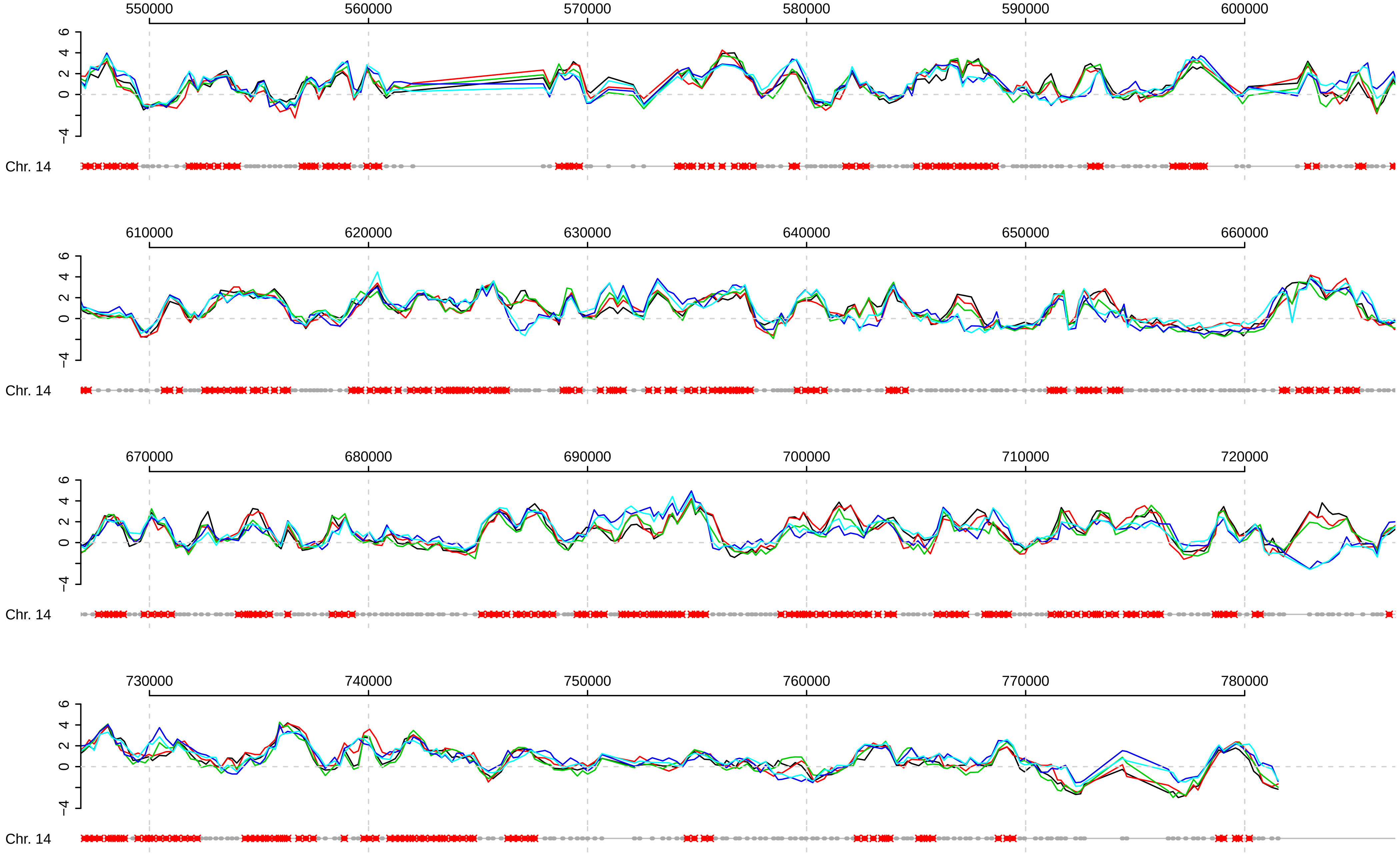

550000
560000
570000
580000
590000
600000
6
4
2
0
−4
Chr. 14
610000
620000
630000
640000
650000
660000
Chr. 14
670000
680000
690000
700000
710000
720000
Chr. 14
730000
740000
750000
760000
770000
780000
Chr. 14

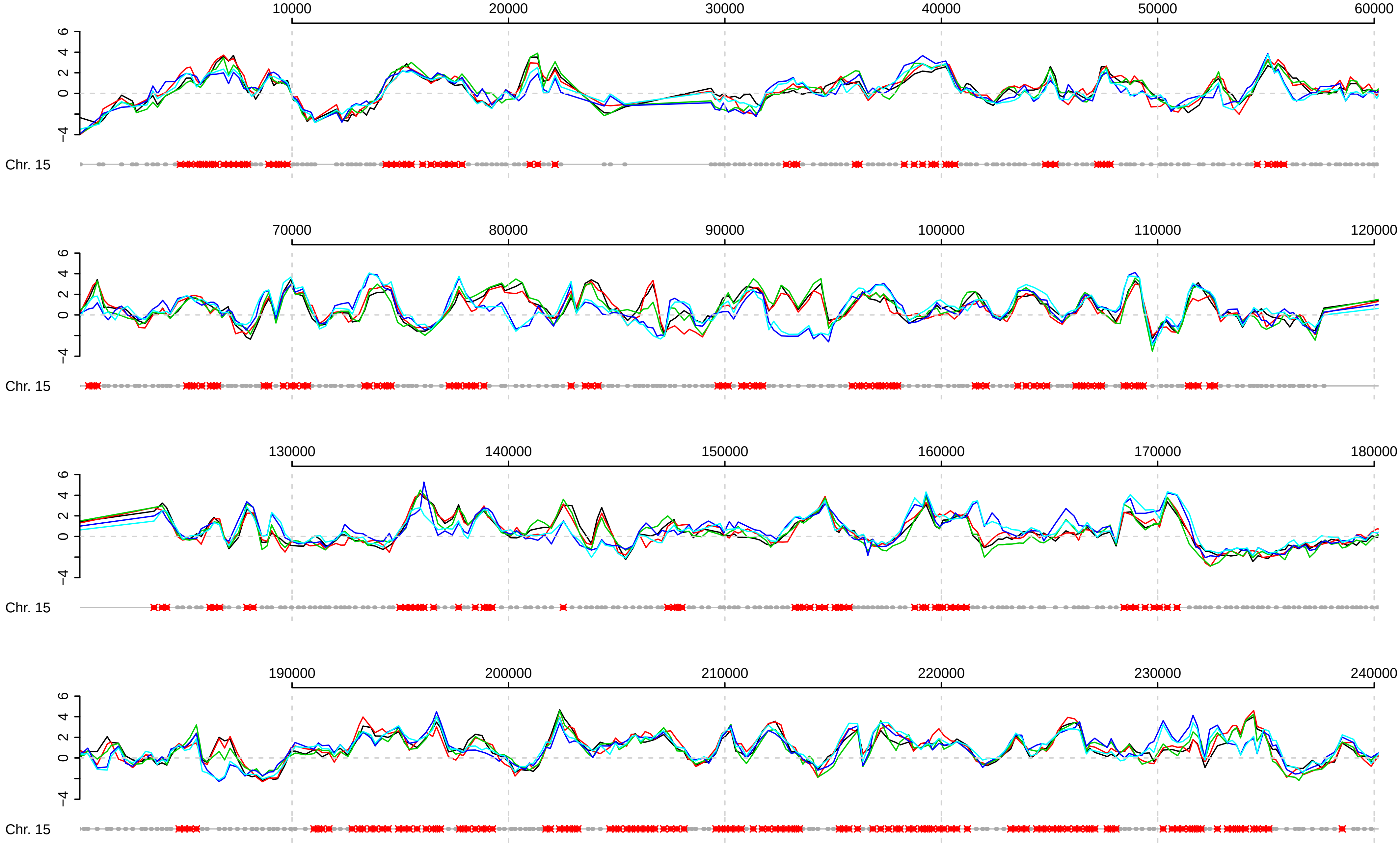

10000
20000
30000
40000
50000
60000
6
4
2
0
−4
Chr. 15
70000
80000
90000
100000
110000
120000
Chr. 15
130000
140000
150000
160000
170000
180000
Chr. 15
190000
200000
210000
220000
230000
240000
Chr. 15

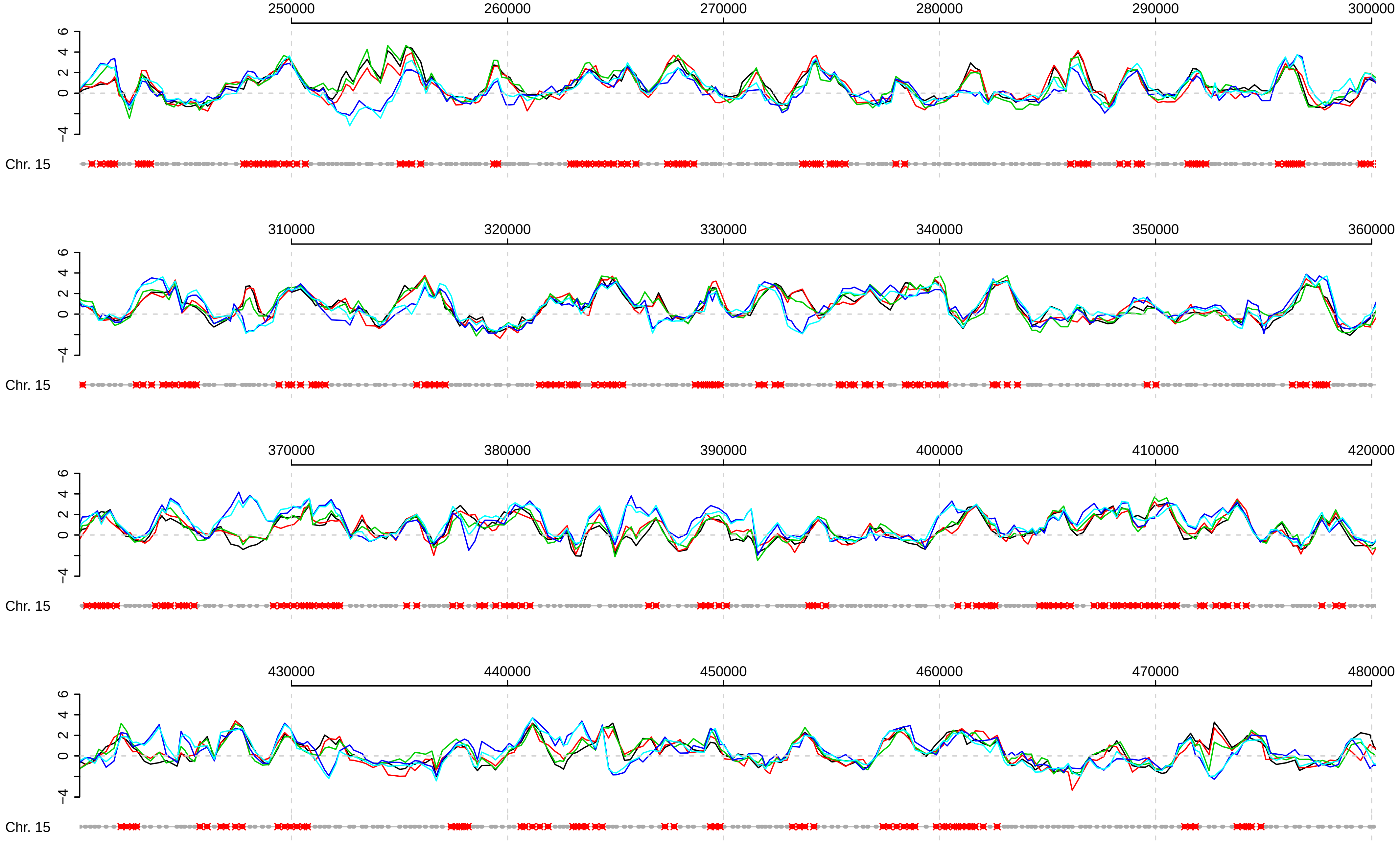

250000
260000
270000
280000
290000
300000
6
4
2
0
−4
Chr. 15
310000
320000
330000
340000
350000
360000
6
4
2
0
−4
Chr. 15
370000
380000
390000
400000
410000
420000
6
4
2
0
−4
Chr. 15
430000
440000
450000
460000
470000
480000
6
4
2
0
−4
Chr. 15

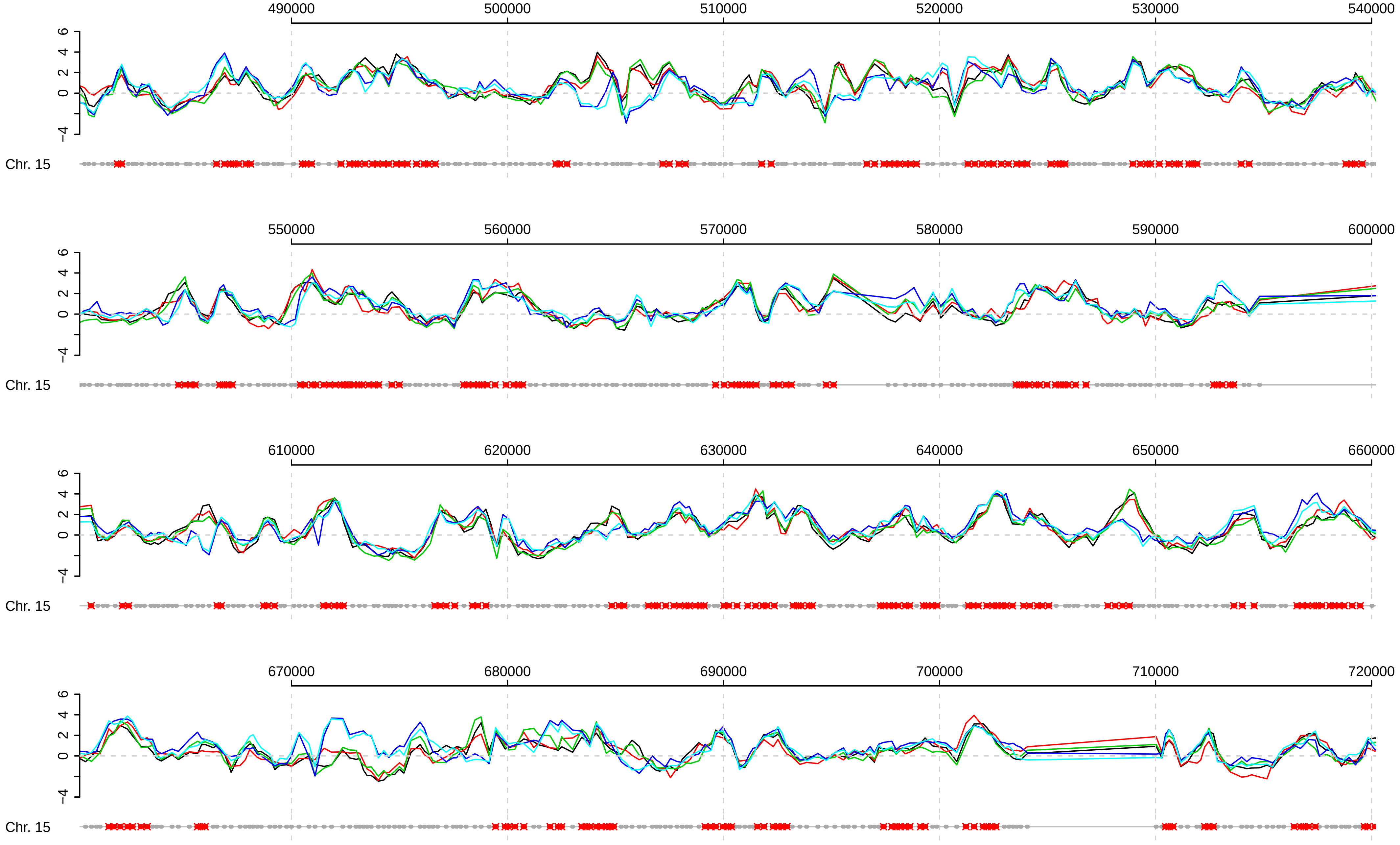
490000
500000
510000
520000
530000
540000
6
4
2
0
−4
Chr. 15
550000
560000
570000
580000
590000
600000
6
4
2
0
−4
Chr. 15
610000
620000
630000
640000
650000
660000
6
4
2
0
−4
Chr. 15
670000
680000
690000
700000
710000
720000
6
4
2
0
−4
Chr. 15

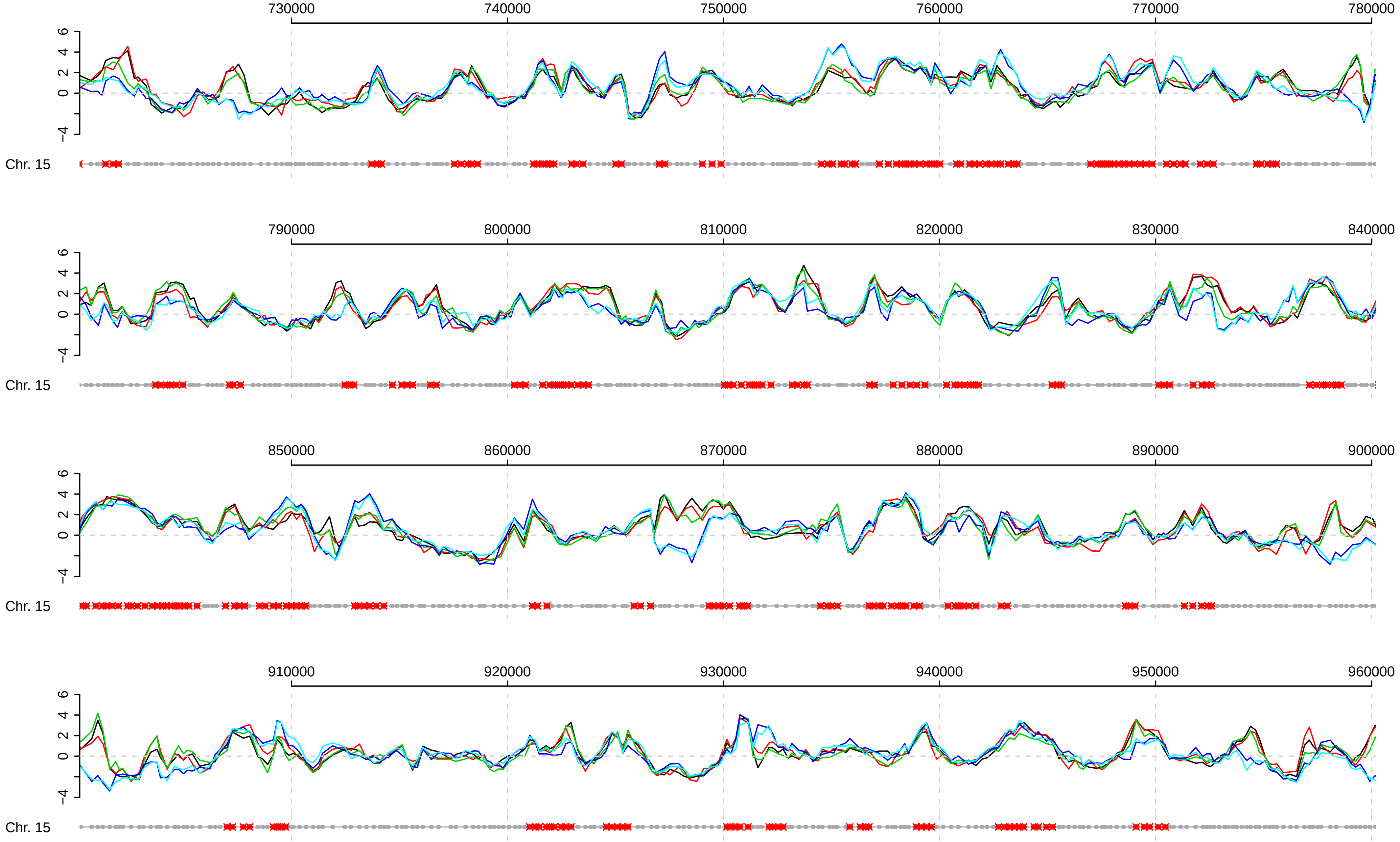

730000
740000
750000
760000
770000
780000
6
4
2
0
−4
Chr. 15
790000
800000
810000
820000
830000
840000
6
4
2
0
−4
Chr. 15
850000
860000
870000
880000
890000
900000
6
4
2
0
−4
Chr. 15
910000
920000
930000
940000
950000
960000
6
4
2
0
−4
Chr. 15

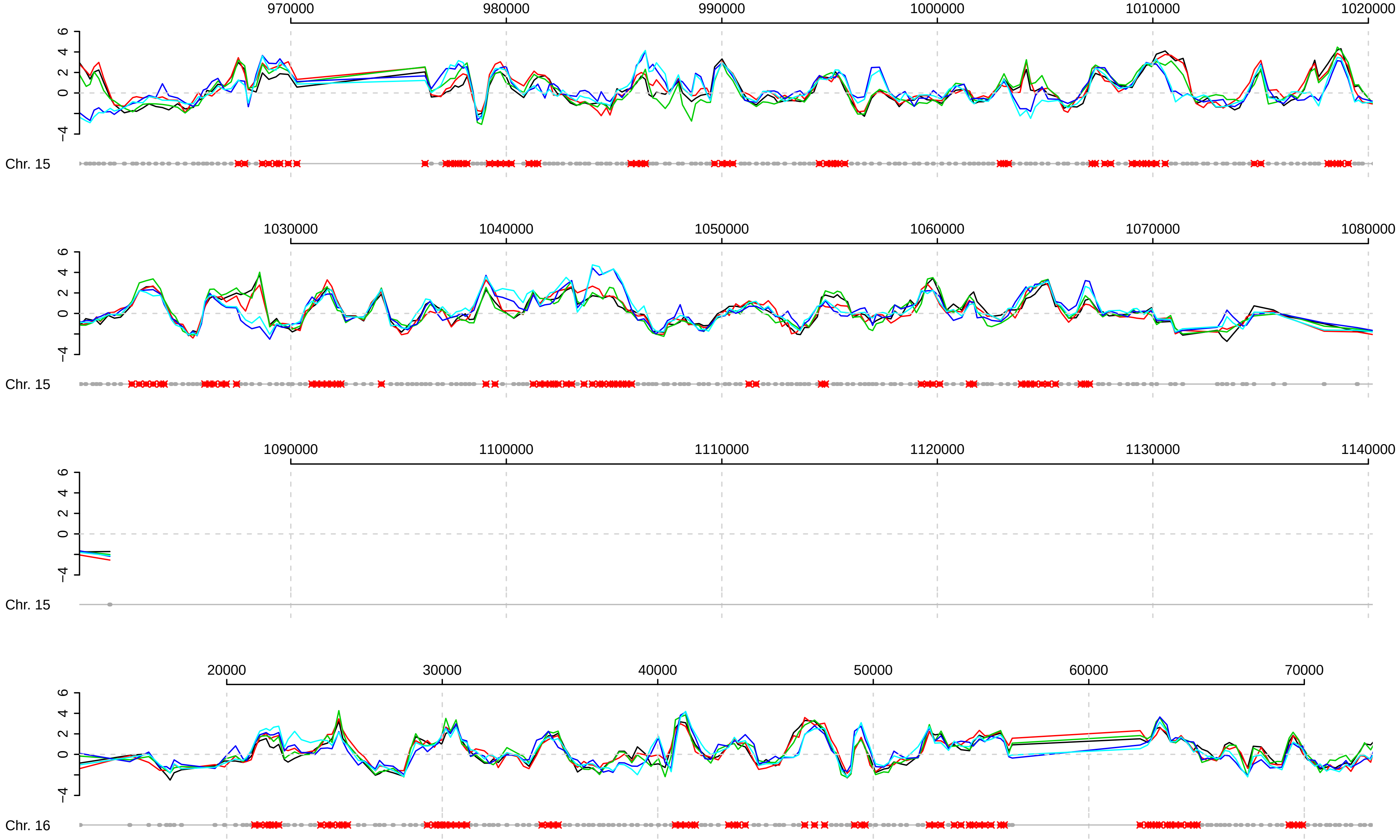

970000
980000
990000
1000000
1010000
1020000
6
4
2
0
−4
Chr. 15
1030000
1040000
1050000
1060000
1070000
1080000
6
4
2
0
−4
Chr. 15
1090000
1100000
1110000
1120000
1130000
1140000
6
4
2
0
−4
Chr. 15
20000
30000
40000
50000
60000
70000
6
4
2
0
−4
Chr. 16

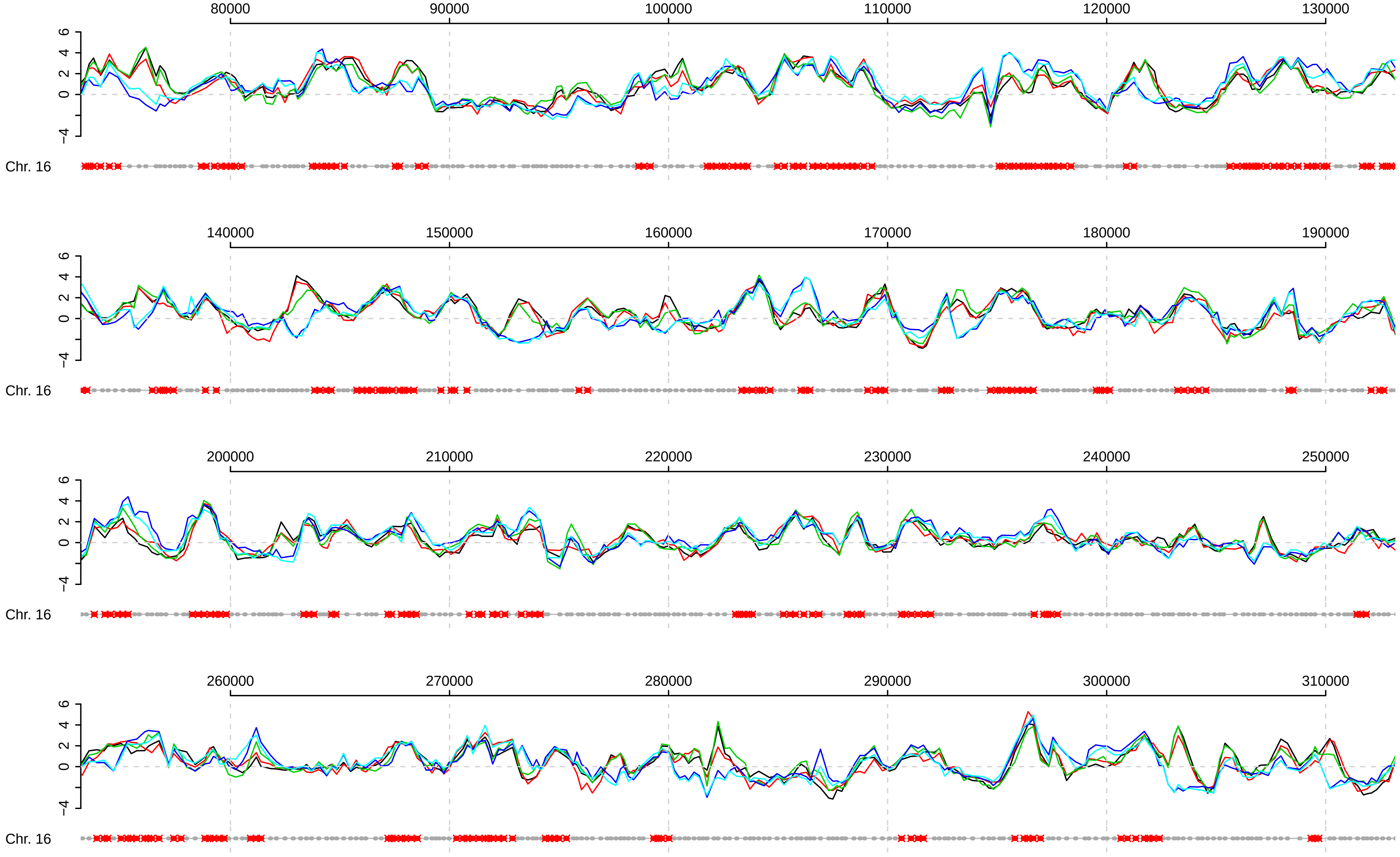

80000
90000
100000
110000
120000
130000
6
4
2
0
−4
Chr. 16
140000
150000
160000
170000
180000
190000
Chr. 16
200000
210000
220000
230000
240000
250000
Chr. 16
260000
270000
280000
290000
300000
310000
Chr. 16

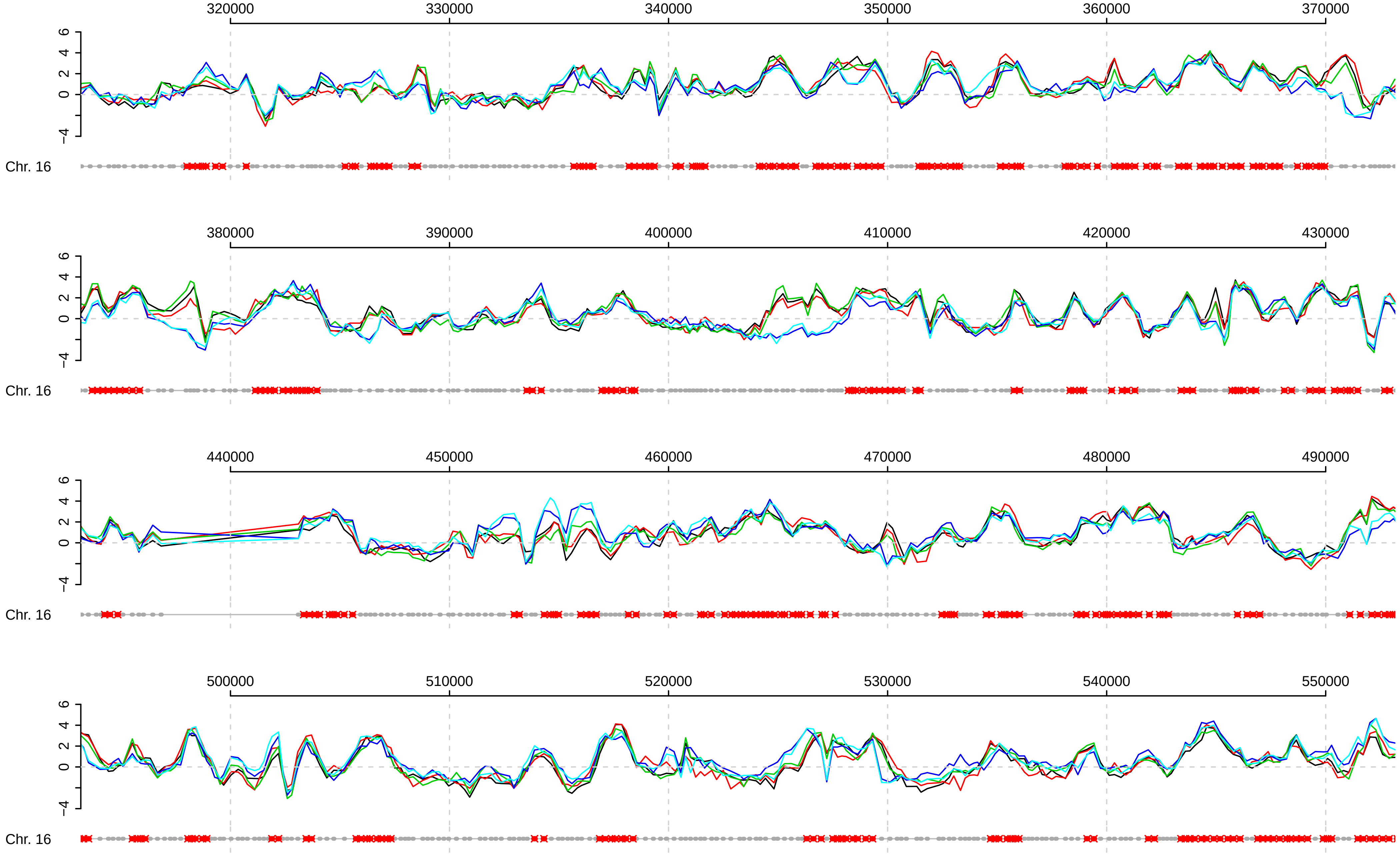

320000
330000
340000
350000
360000
370000
Chr. 16
380000
390000
400000
410000
420000
430000
Chr. 16
440000
450000
460000
470000
480000
490000
Chr. 16
500000
510000
520000
530000
540000
550000
Chr. 16

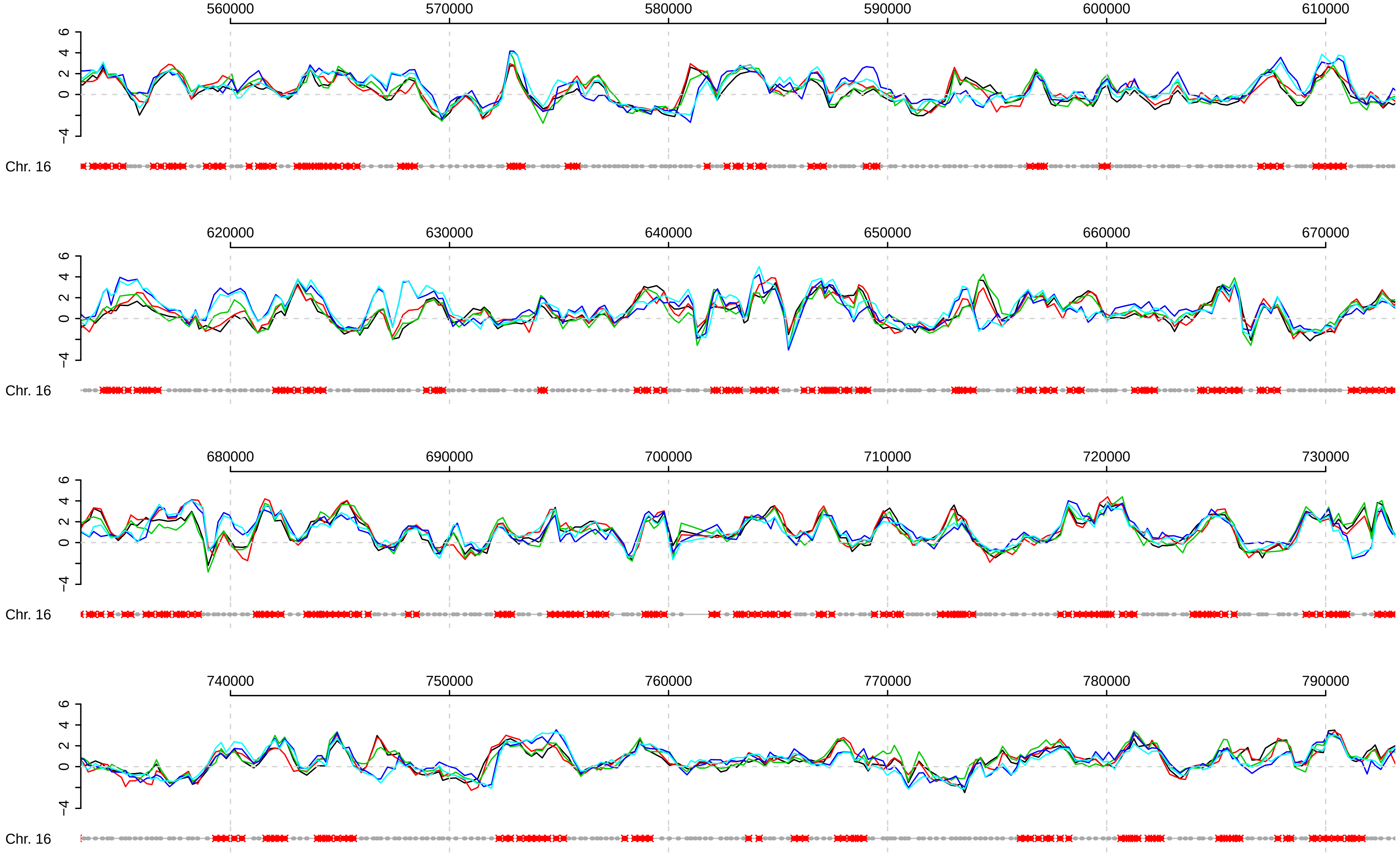

560000 570000 580000 590000 600000 610000
620000 630000 640000 650000 660000 670000
680000 690000 700000 710000 720000 730000
740000 750000 760000 770000 780000 790000
6 4 2 0 −4
Chr. 16

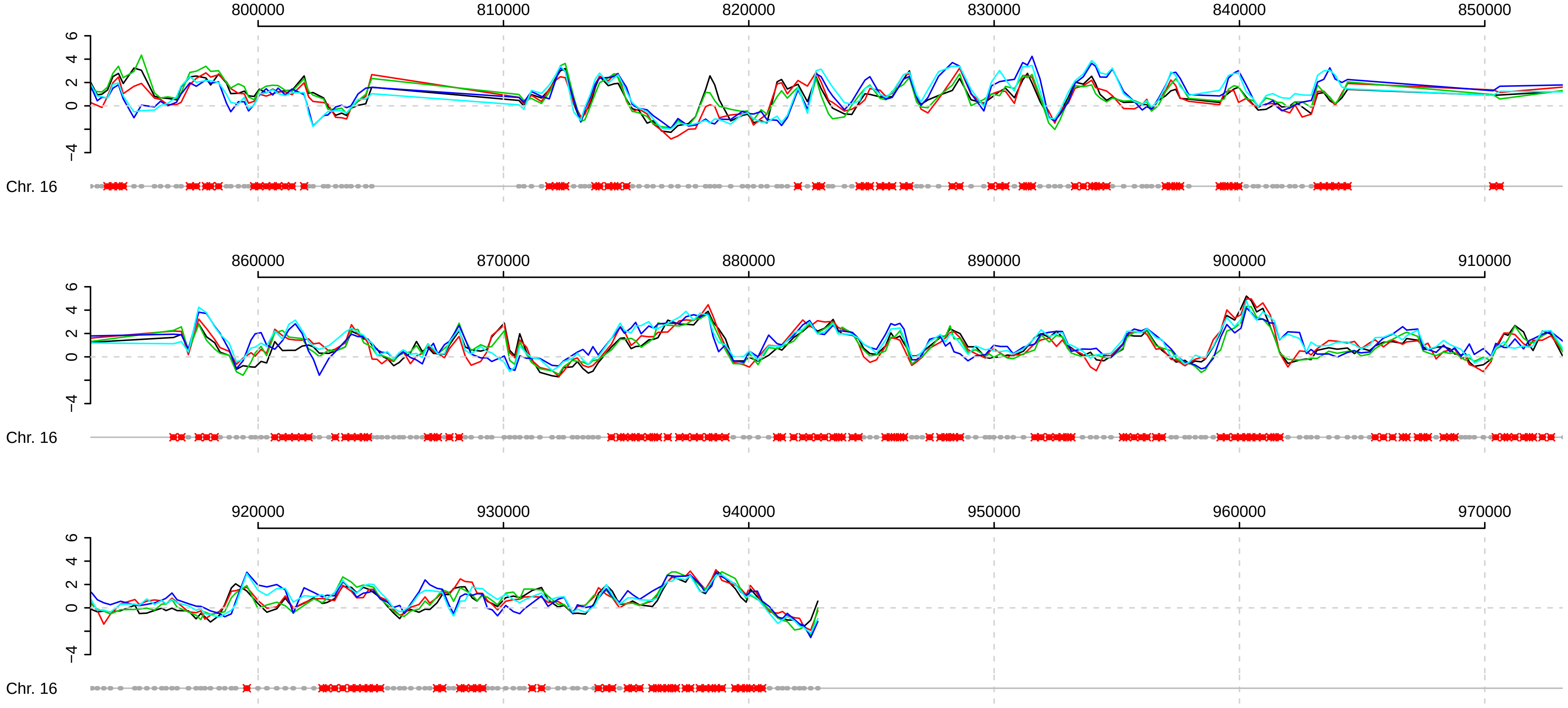
800000
810000
820000
830000
840000
850000
6
4
2
0
−4
Chr. 16
860000
870000
880000
890000
900000
910000
Chr. 16
920000
930000
940000
950000
960000
970000
Chr. 16

The following plots show H3Ac enrichment over the whole genome in cells treated with UV. The five line colours show five replicate datasets. The red crosses show probes detected as enriched.

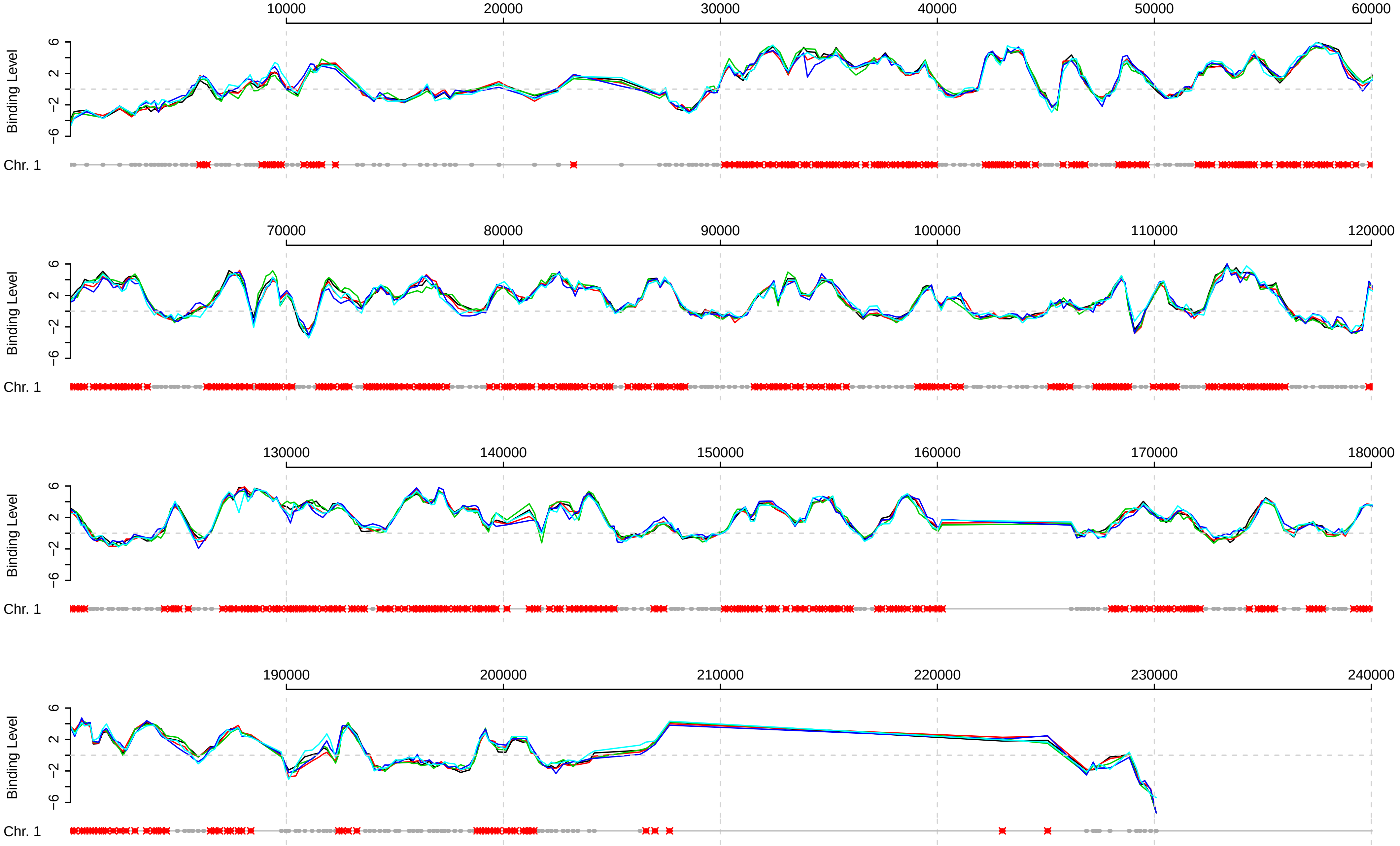

10000 20000 30000 40000 50000 60000
70000 80000 90000 100000 110000 120000
130000 140000 150000 160000 170000 180000
190000 200000 210000 220000 230000 240000
Binding Level
6
2
−2
−6
Chr. 1

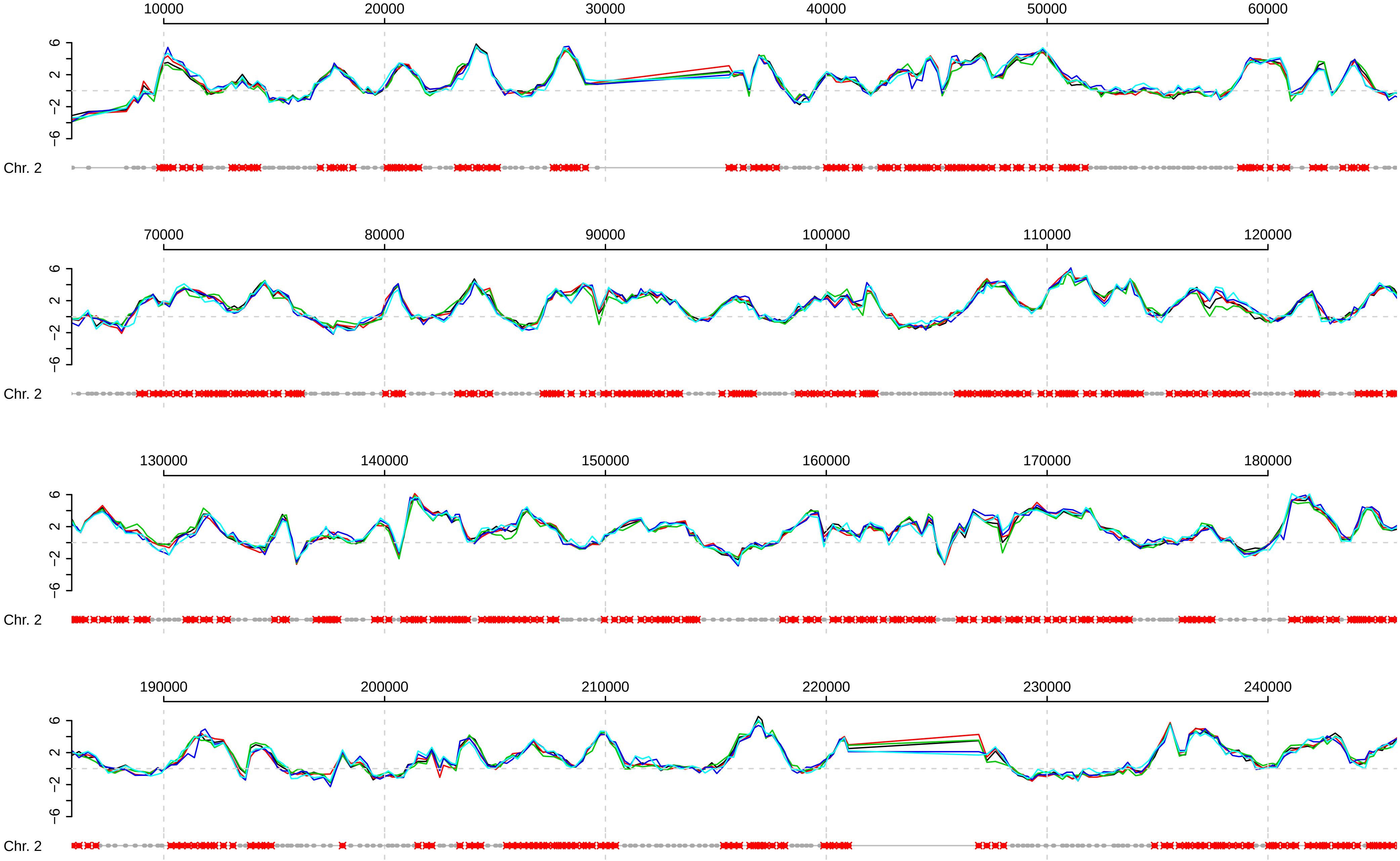
10000
20000
30000
40000
50000
60000
6
2
−2
−6
Chr. 2
70000
80000
90000
100000
110000
120000
6
2
−2
−6
Chr. 2
130000
140000
150000
160000
170000
180000
6
2
−2
−6
Chr. 2
190000
200000
210000
220000
230000
240000
6
2
−2
−6
Chr. 2

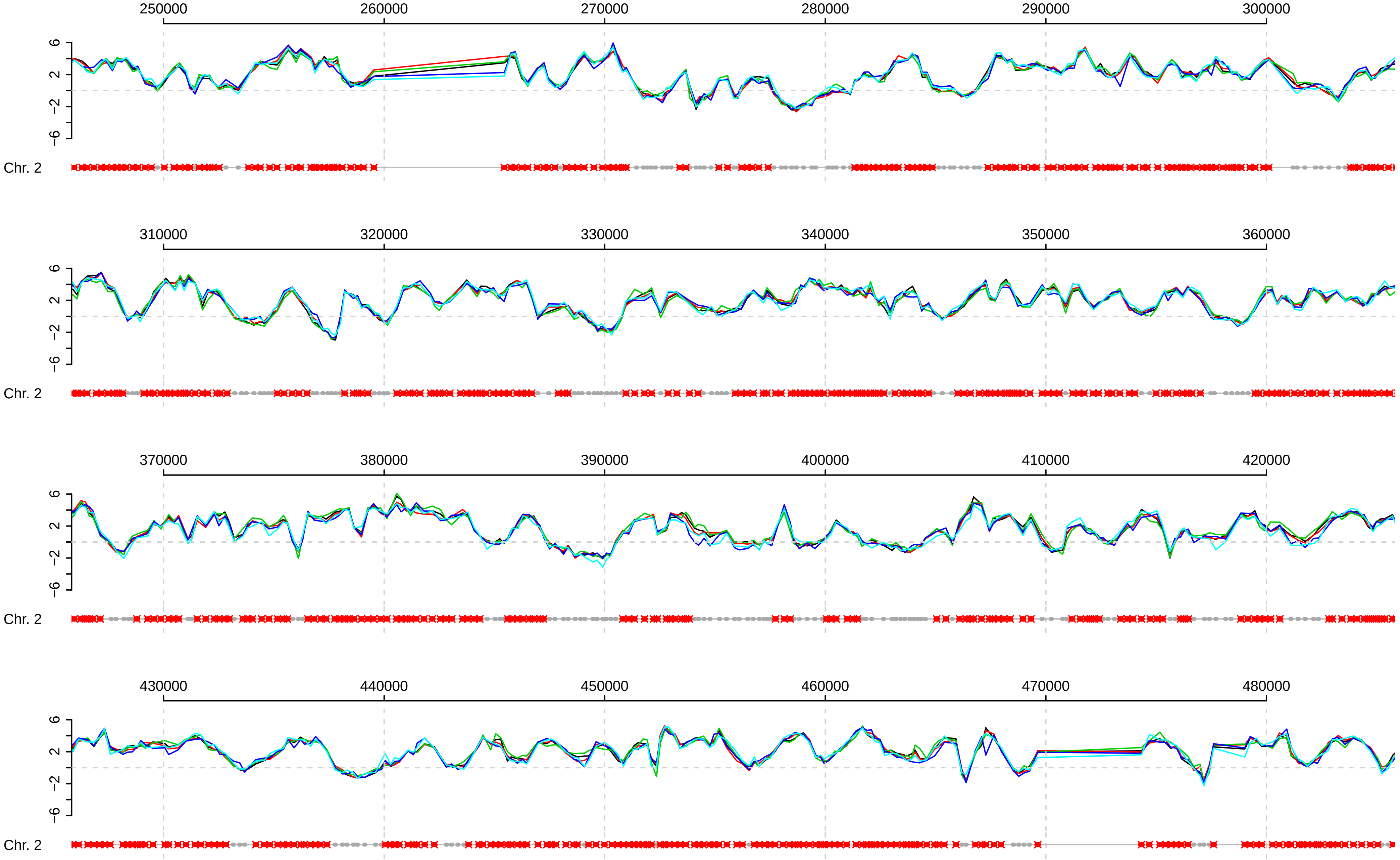

250000
260000
270000
280000
290000
300000
6
2
−2
−6
Chr. 2
310000
320000
330000
340000
350000
360000
6
2
−2
−6
Chr. 2
370000
380000
390000
400000
410000
420000
6
2
−2
−6
Chr. 2
430000
440000
450000
460000
470000
480000
6
2
−2
−6
Chr. 2

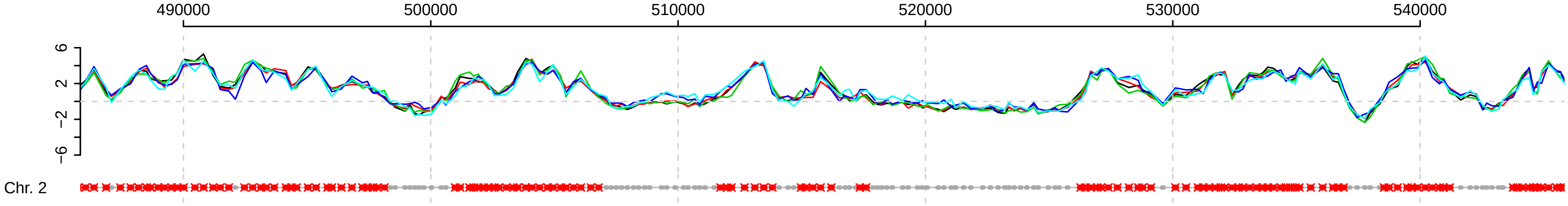

490000
500000
510000
520000
530000
540000
6
2
−2
−6
Chr. 2

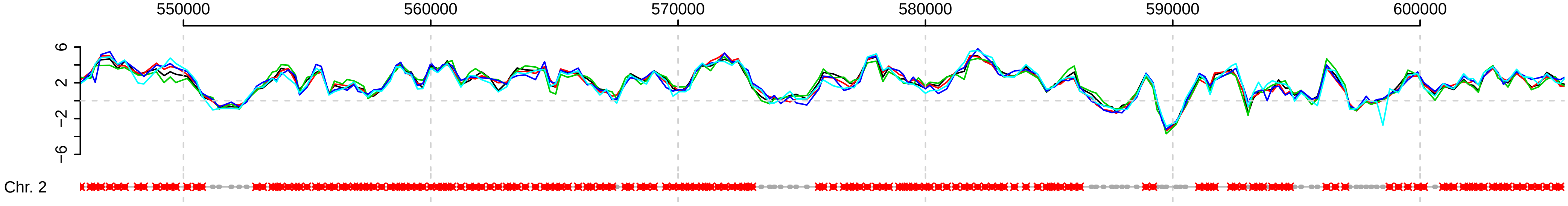

550000
560000
570000
580000
590000
600000
6
2
−2
−6
Chr. 2

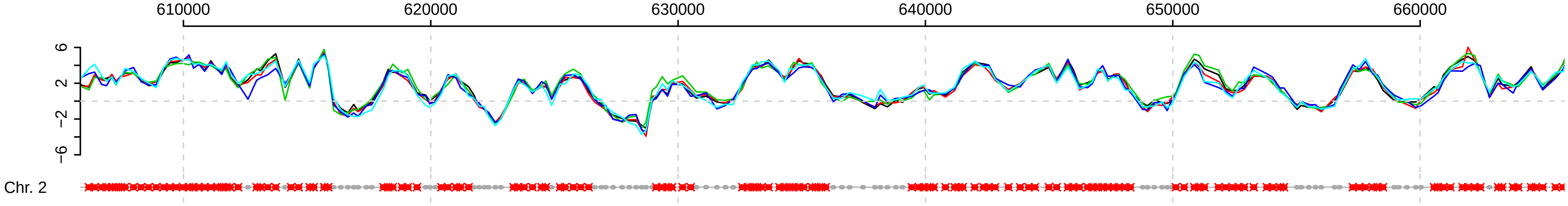

610000
620000
630000
640000
650000
660000
6
2
−2
−6
Chr. 2

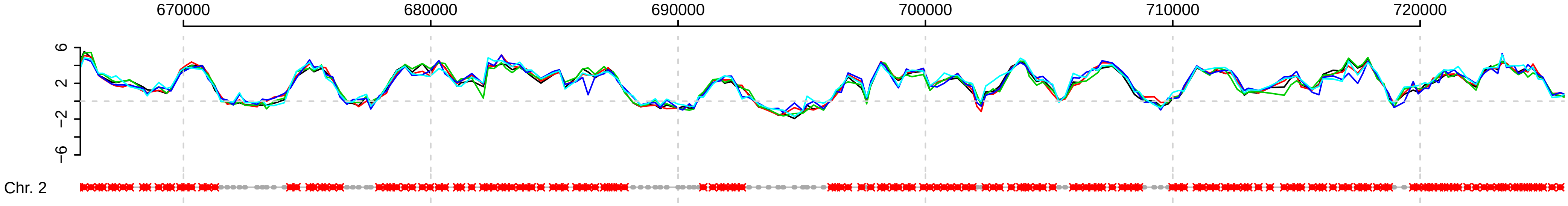

670000
680000
690000
700000
710000
720000
6
2
−2
−6
Chr. 2

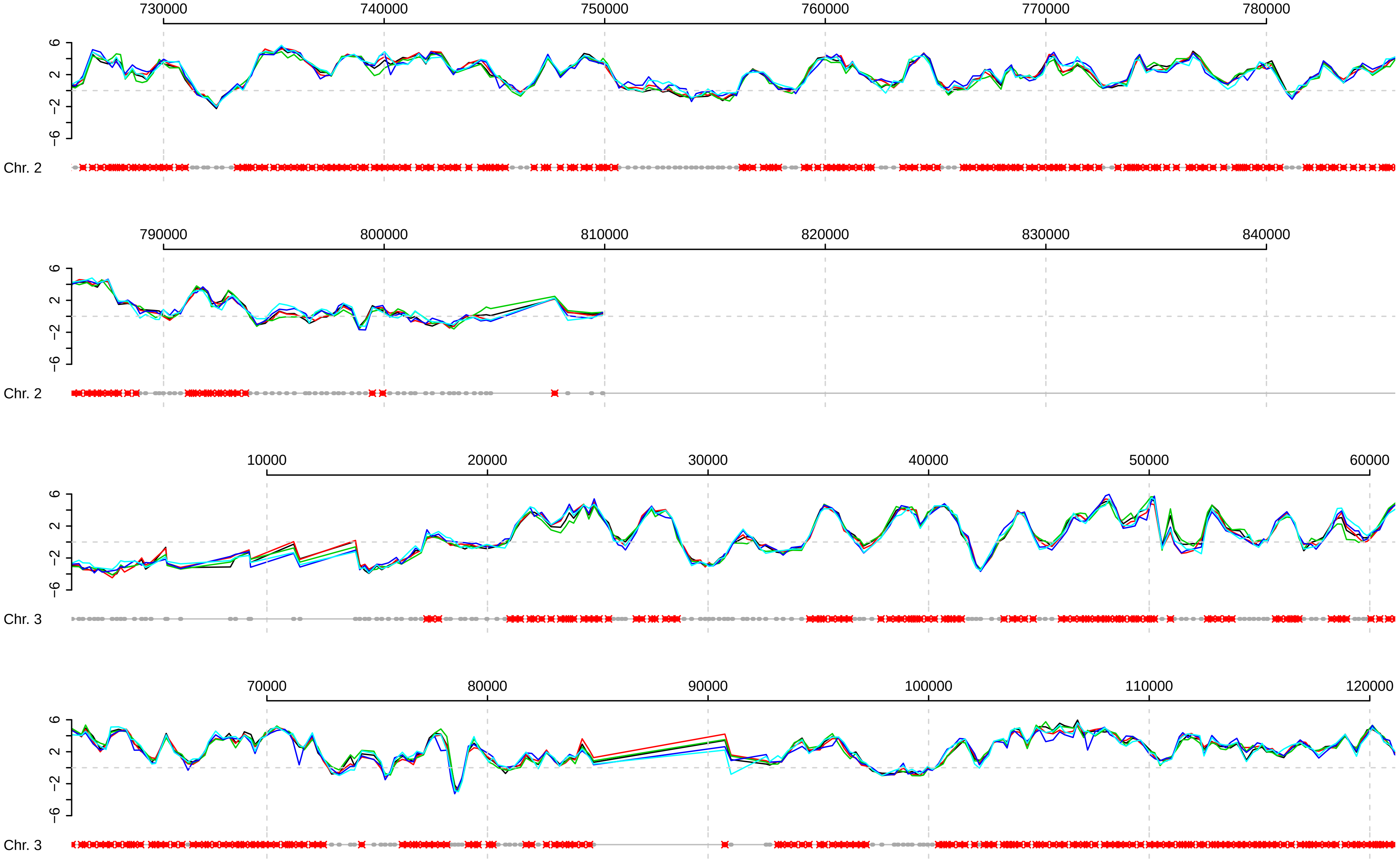
730000
740000
750000
760000
770000
780000
6
2
−2
−6
Chr. 2
790000
800000
810000
820000
830000
840000
6
2
−2
−6
Chr. 2
10000
20000
30000
40000
50000
60000
6
2
−2
−6
Chr. 3
70000
80000
90000
100000
110000
120000
6
2
−2
−6
Chr. 3

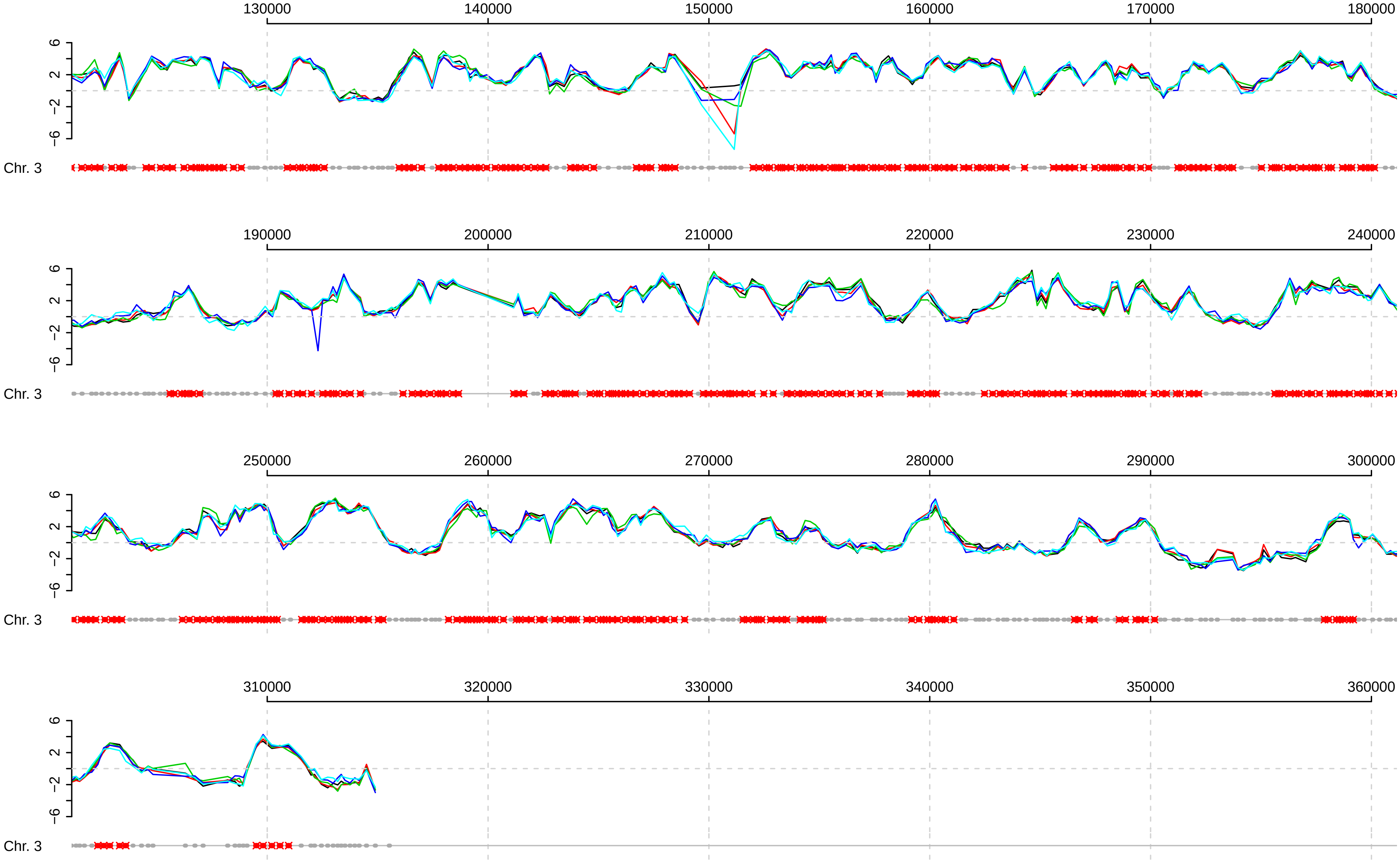
130000
140000
150000
160000
170000
180000
6
2
−2
−6
Chr. 3
190000
200000
210000
220000
230000
240000
6
2
−2
−6
Chr. 3
250000
260000
270000
280000
290000
300000
6
2
−2
−6
Chr. 3
310000
320000
330000
340000
350000
360000
6
2
−2
−6
Chr. 3

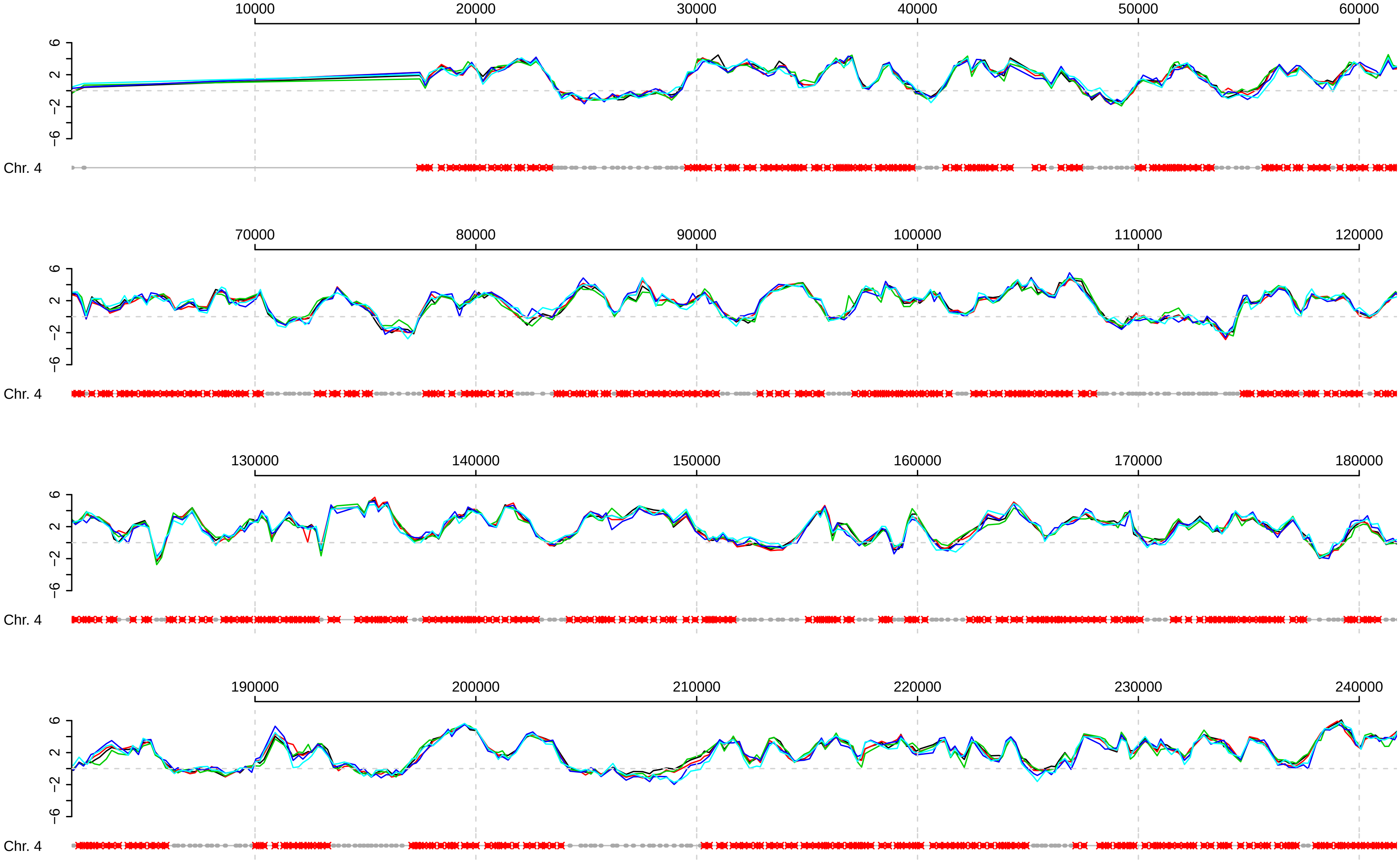

10000
20000
30000
40000
50000
60000
6
2
−2
−6
Chr. 4
70000
80000
90000
100000
110000
120000
6
2
−2
−6
Chr. 4
130000
140000
150000
160000
170000
180000
6
2
−2
−6
Chr. 4
190000
200000
210000
220000
230000
240000
6
2
−2
−6
Chr. 4

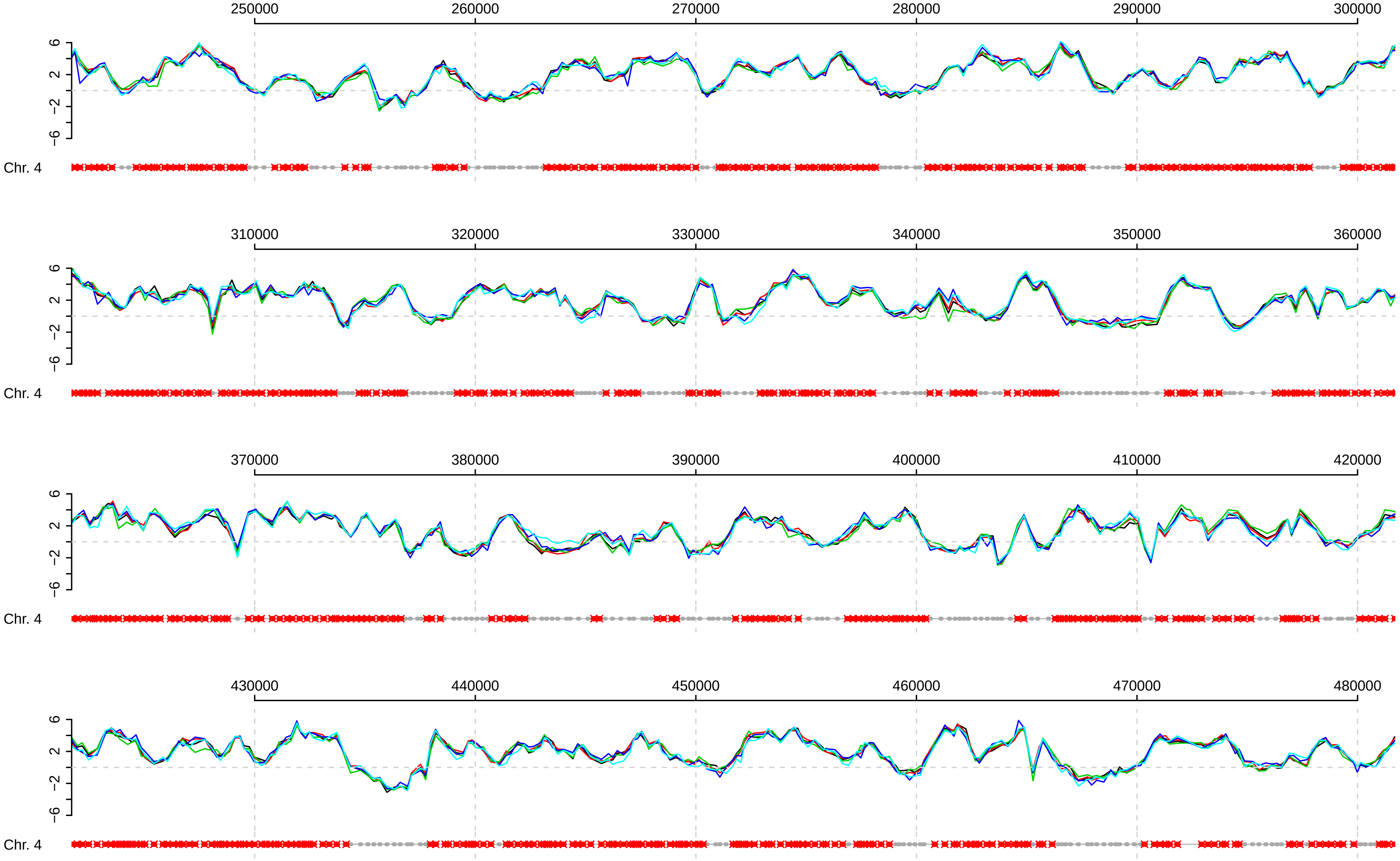
250000
260000
270000
280000
290000
300000
6
2
−2
−6
Chr. 4
310000
320000
330000
340000
350000
360000
6
2
−2
−6
Chr. 4
370000
380000
390000
400000
410000
420000
6
2
−2
−6
Chr. 4
430000
440000
450000
460000
470000
480000
6
2
−2
−6
Chr. 4

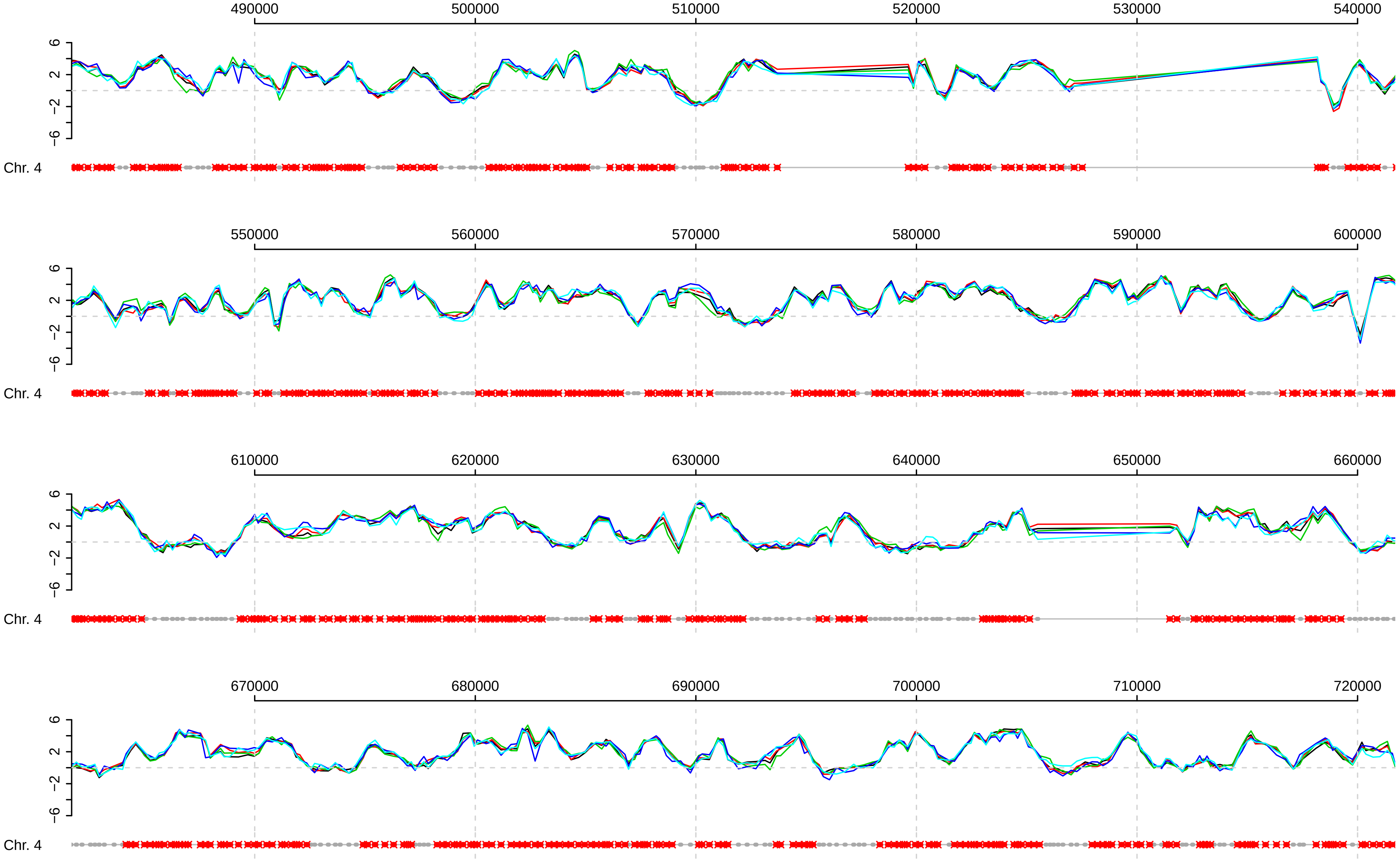

490000
500000
510000
520000
530000
540000
6
2
−2
−6
Chr. 4
550000
560000
570000
580000
590000
600000
6
2
−2
−6
Chr. 4
610000
620000
630000
640000
650000
660000
6
2
−2
−6
Chr. 4
670000
680000
690000
700000
710000
720000
6
2
−2
−6
Chr. 4

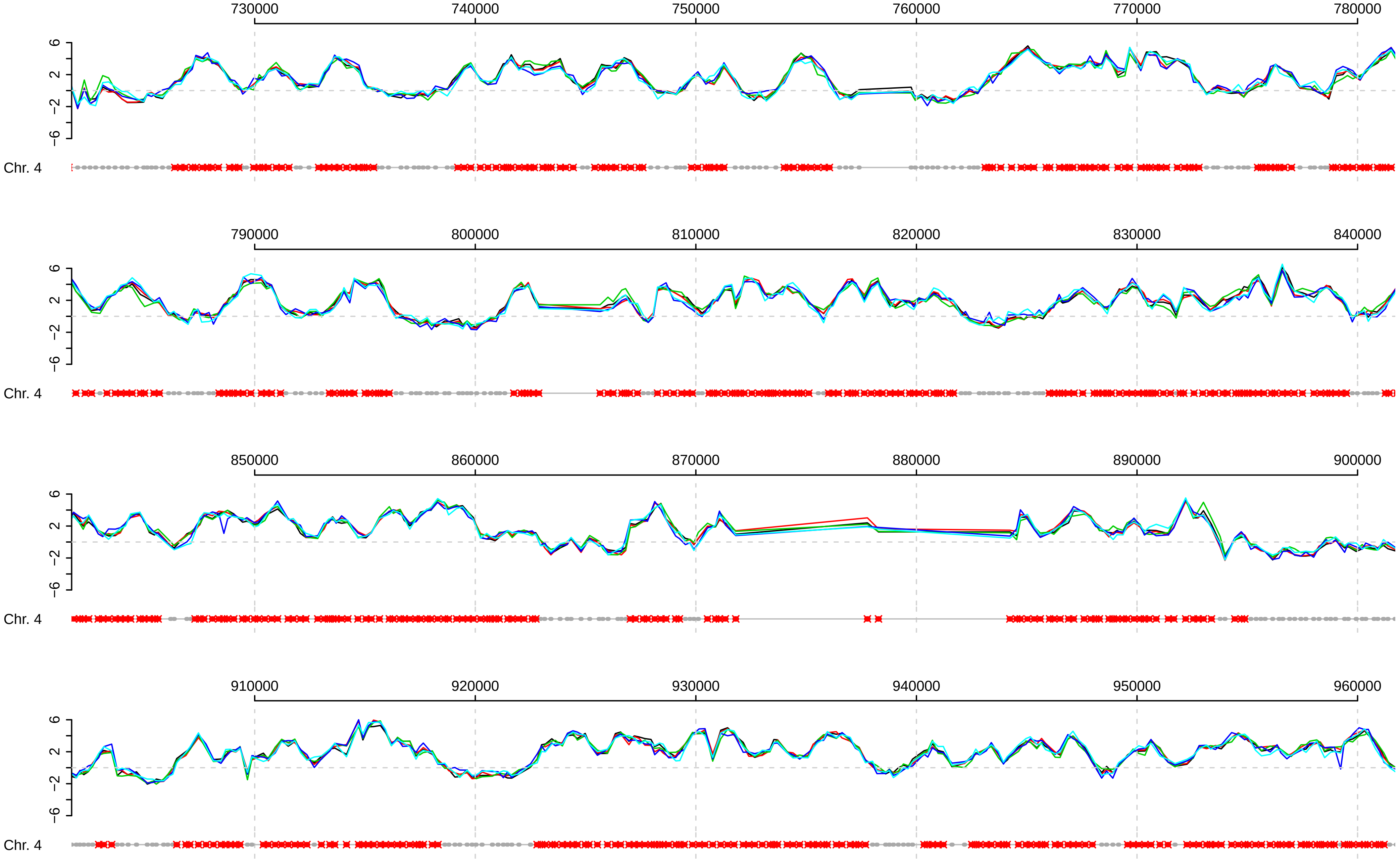

730000
740000
750000
760000
770000
780000
6
2
−2
−6
Chr. 4
790000
800000
810000
820000
830000
840000
6
2
−2
−6
Chr. 4
850000
860000
870000
880000
890000
900000
6
2
−2
−6
Chr. 4
910000
920000
930000
940000
950000
960000
6
2
−2
−6
Chr. 4

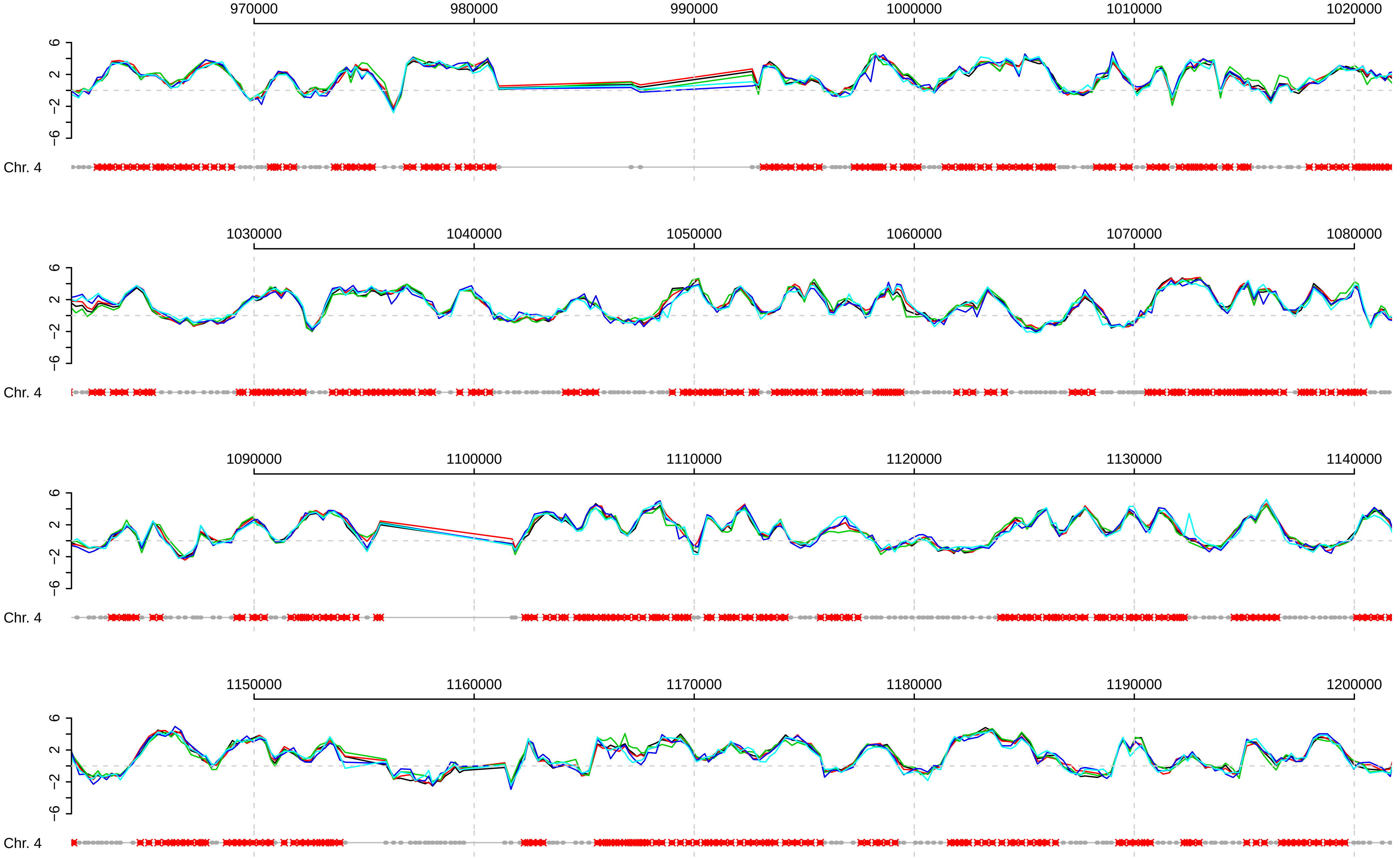
970000
980000
990000
1000000
1010000
1020000
6
2
−2
−6
Chr. 4
1030000
1040000
1050000
1060000
1070000
1080000
6
2
−2
−6
Chr. 4
1090000
1100000
1110000
1120000
1130000
1140000
6
2
−2
−6
Chr. 4
1150000
1160000
1170000
1180000
1190000
1200000
6
2
−2
−6
Chr. 4

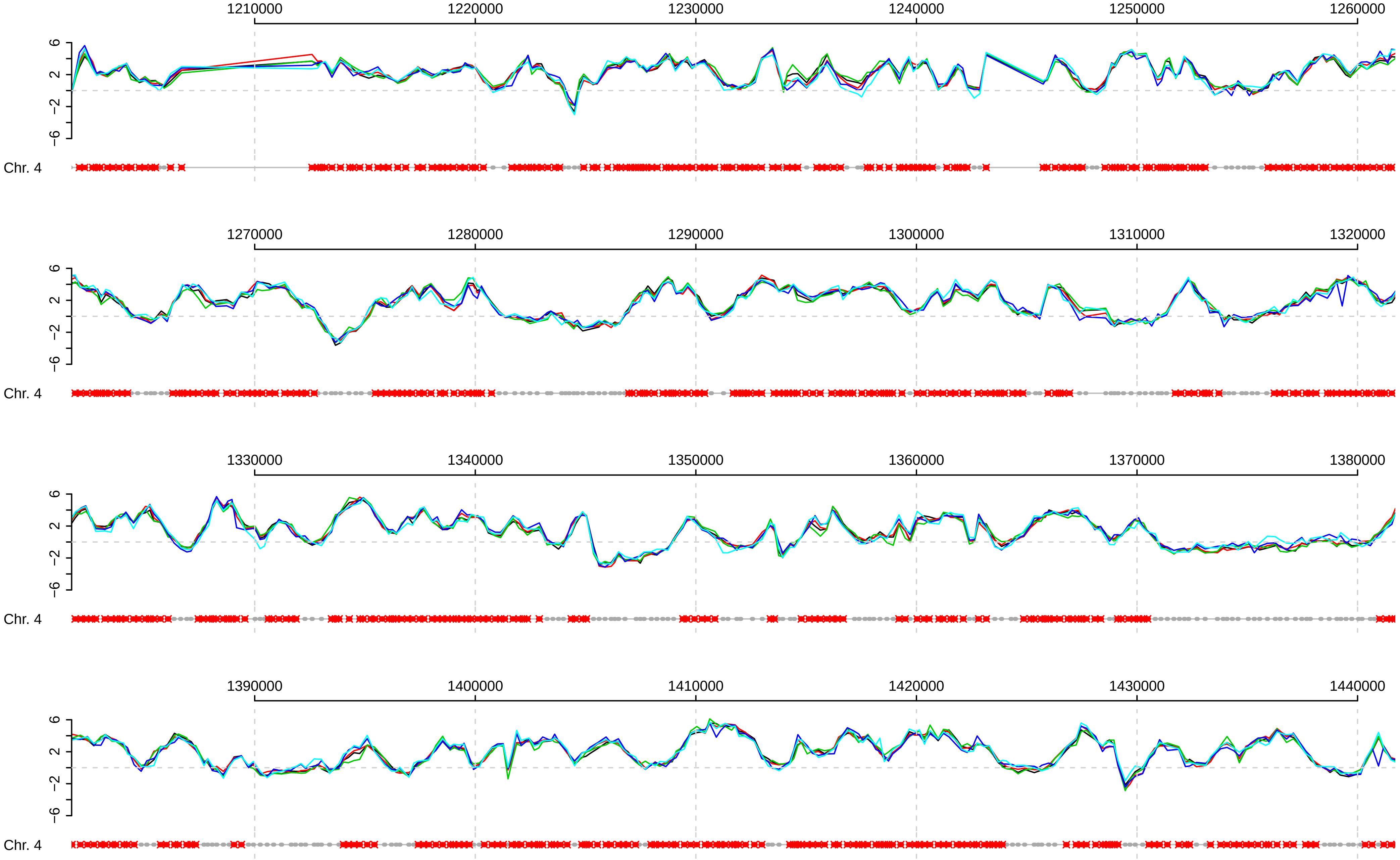

1210000
1220000
1230000
1240000
1250000
1260000
6
2
−2
−6
Chr. 4
1270000
1280000
1290000
1300000
1310000
1320000
6
2
−2
−6
Chr. 4
1330000
1340000
1350000
1360000
1370000
1380000
6
2
−2
−6
Chr. 4
1390000
1400000
1410000
1420000
1430000
1440000
6
2
−2
−6
Chr. 4

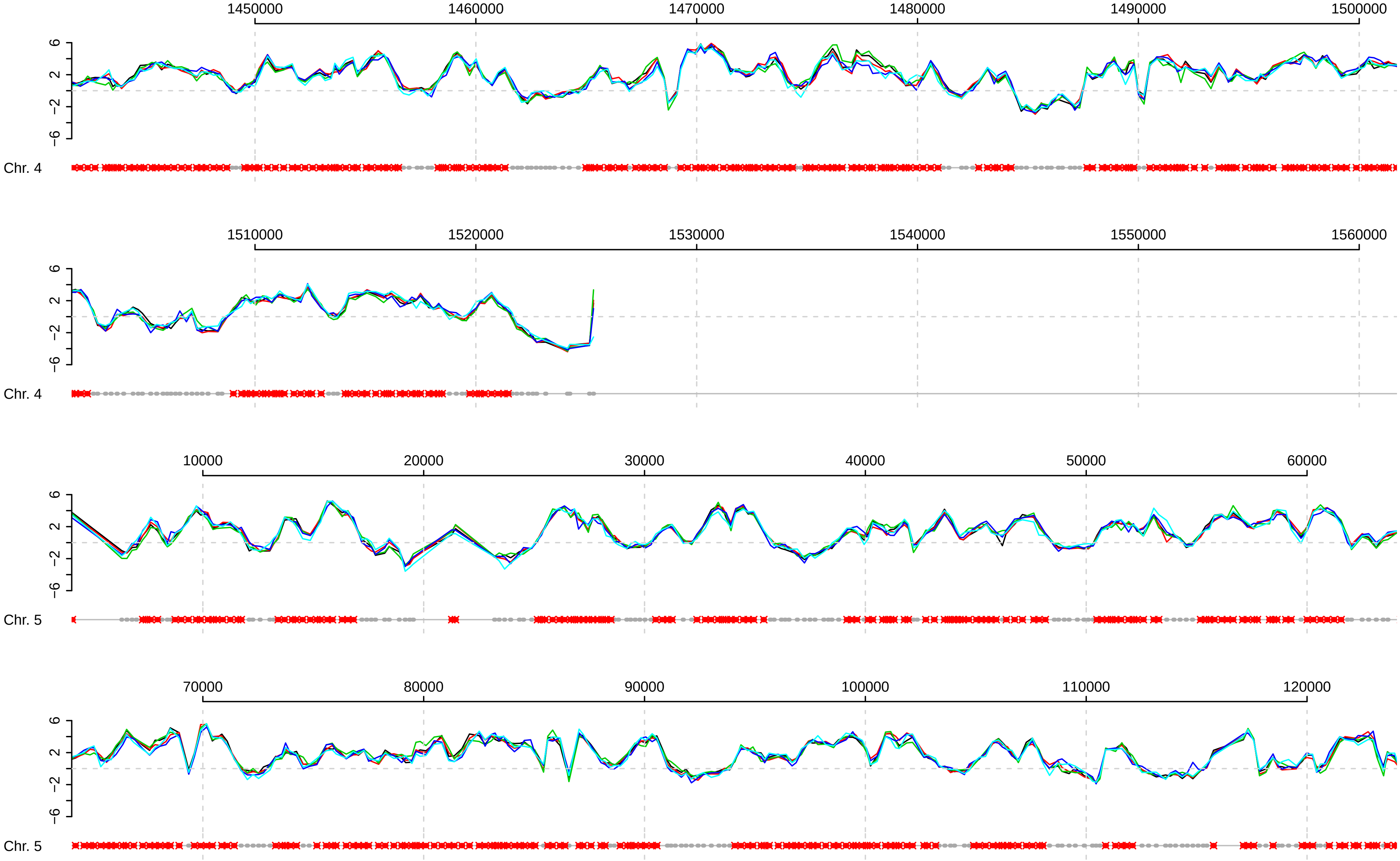
1450000
1460000
1470000
1480000
1490000
1500000
6
2
−2
−6
Chr. 4
1510000
1520000
1530000
1540000
1550000
1560000
6
2
−2
−6
Chr. 4
10000
20000
30000
40000
50000
60000
6
2
−2
−6
Chr. 5
70000
80000
90000
100000
110000
120000
6
2
−2
−6
Chr. 5

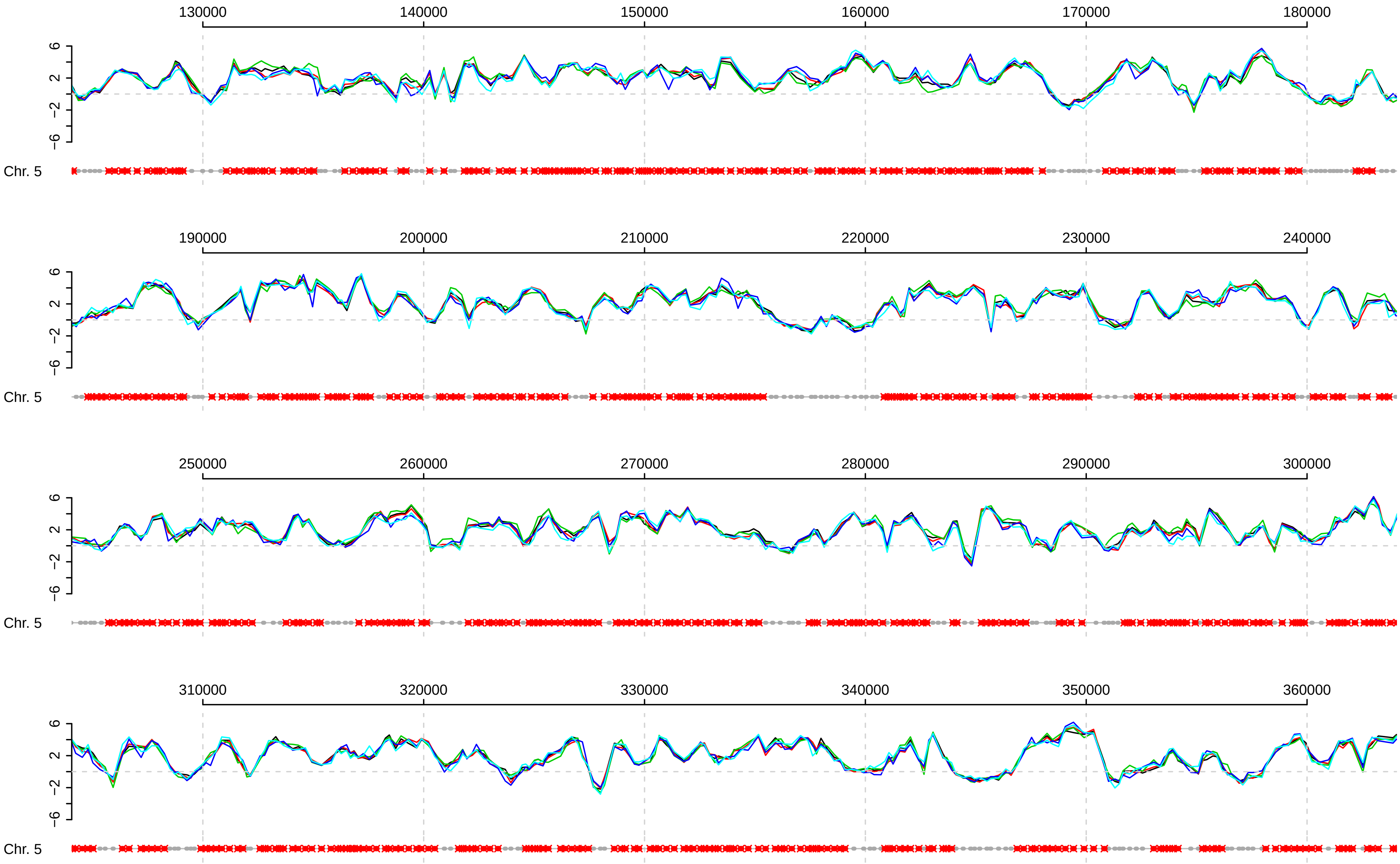
130000
140000
150000
160000
170000
180000
6
2
−2
−6
Chr. 5
190000
200000
210000
220000
230000
240000
6
2
−2
−6
Chr. 5
250000
260000
270000
280000
290000
300000
6
2
−2
−6
Chr. 5
310000
320000
330000
340000
350000
360000
6
2
−2
−6
Chr. 5

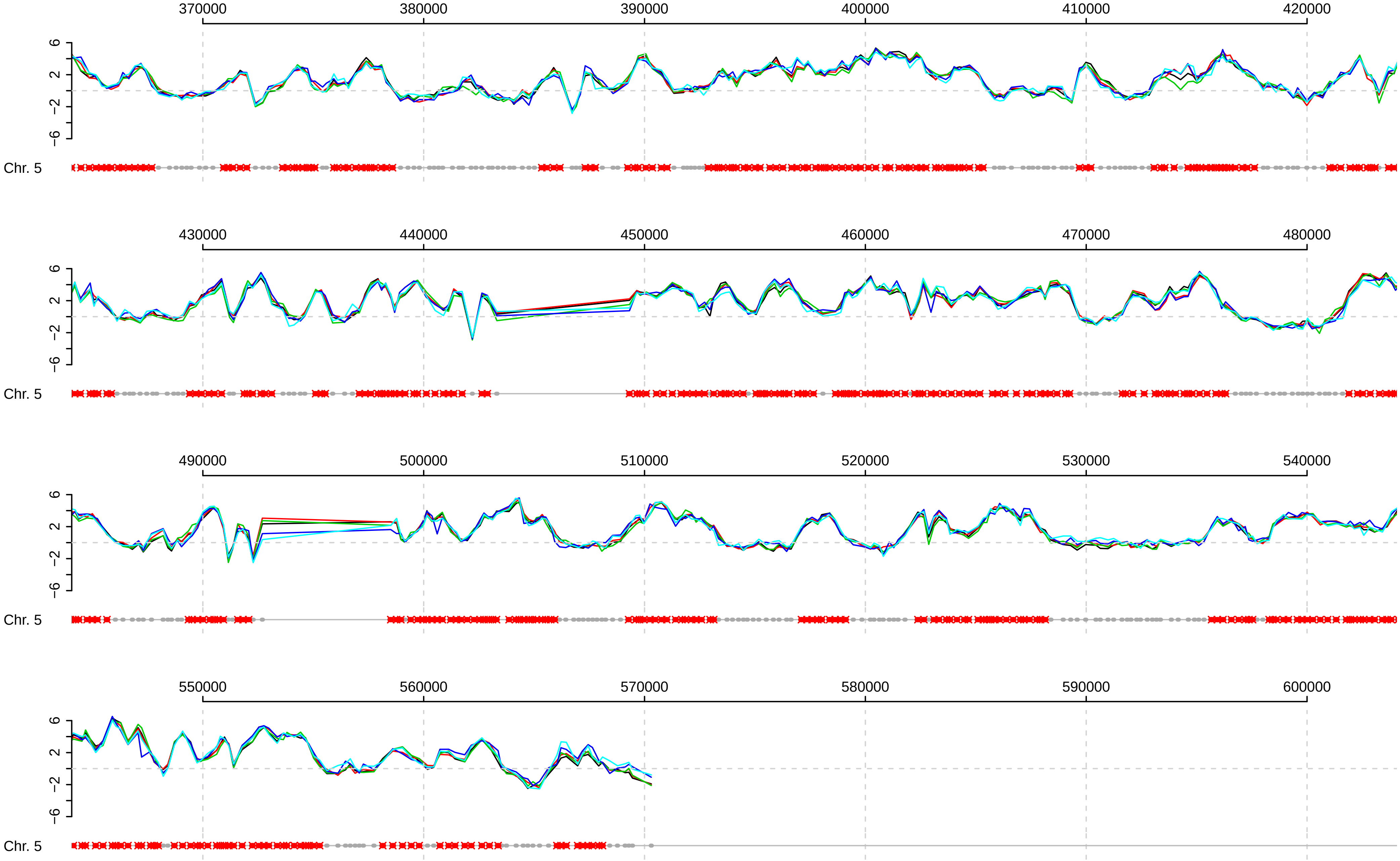
370000
380000
390000
400000
410000
420000
6
2
−2
−6
Chr. 5
430000
440000
450000
460000
470000
480000
6
2
−2
−6
Chr. 5
490000
500000
510000
520000
530000
540000
6
2
−2
−6
Chr. 5
550000
560000
570000
580000
590000
600000
6
2
−2
−6
Chr. 5

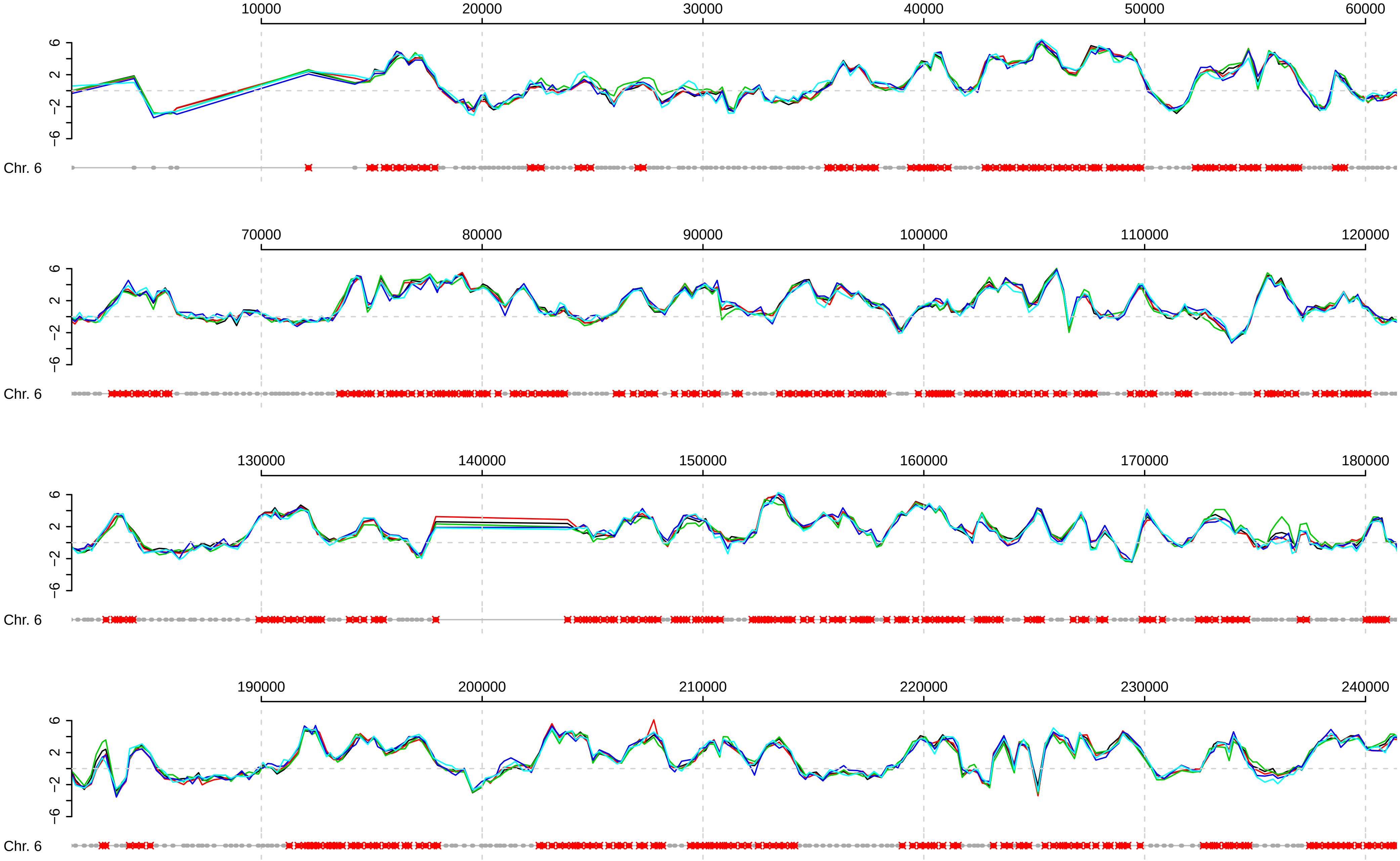

10000
20000
30000
40000
50000
60000
70000
80000
90000
100000
110000
120000
130000
140000
150000
160000
170000
180000
190000
200000
210000
220000
230000
240000
6
2
−2
−6
Chr. 6

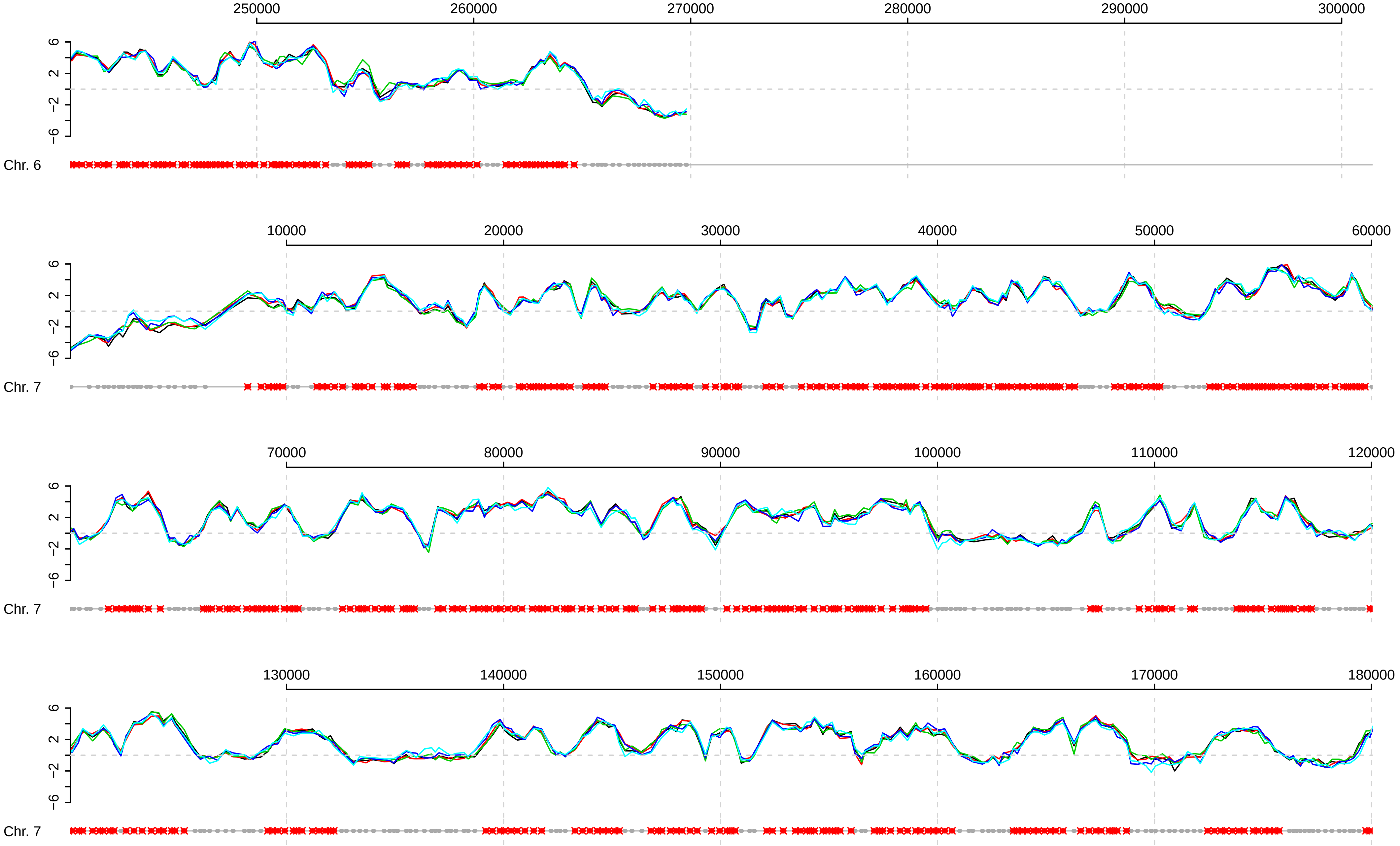
250000
260000
270000
280000
290000
300000
6
2
−2
−6
Chr. 6
10000
20000
30000
40000
50000
60000
6
2
−2
−6
Chr. 7
70000
80000
90000
100000
110000
120000
6
2
−2
−6
Chr. 7
130000
140000
150000
160000
170000
180000
6
2
−2
−6
Chr. 7

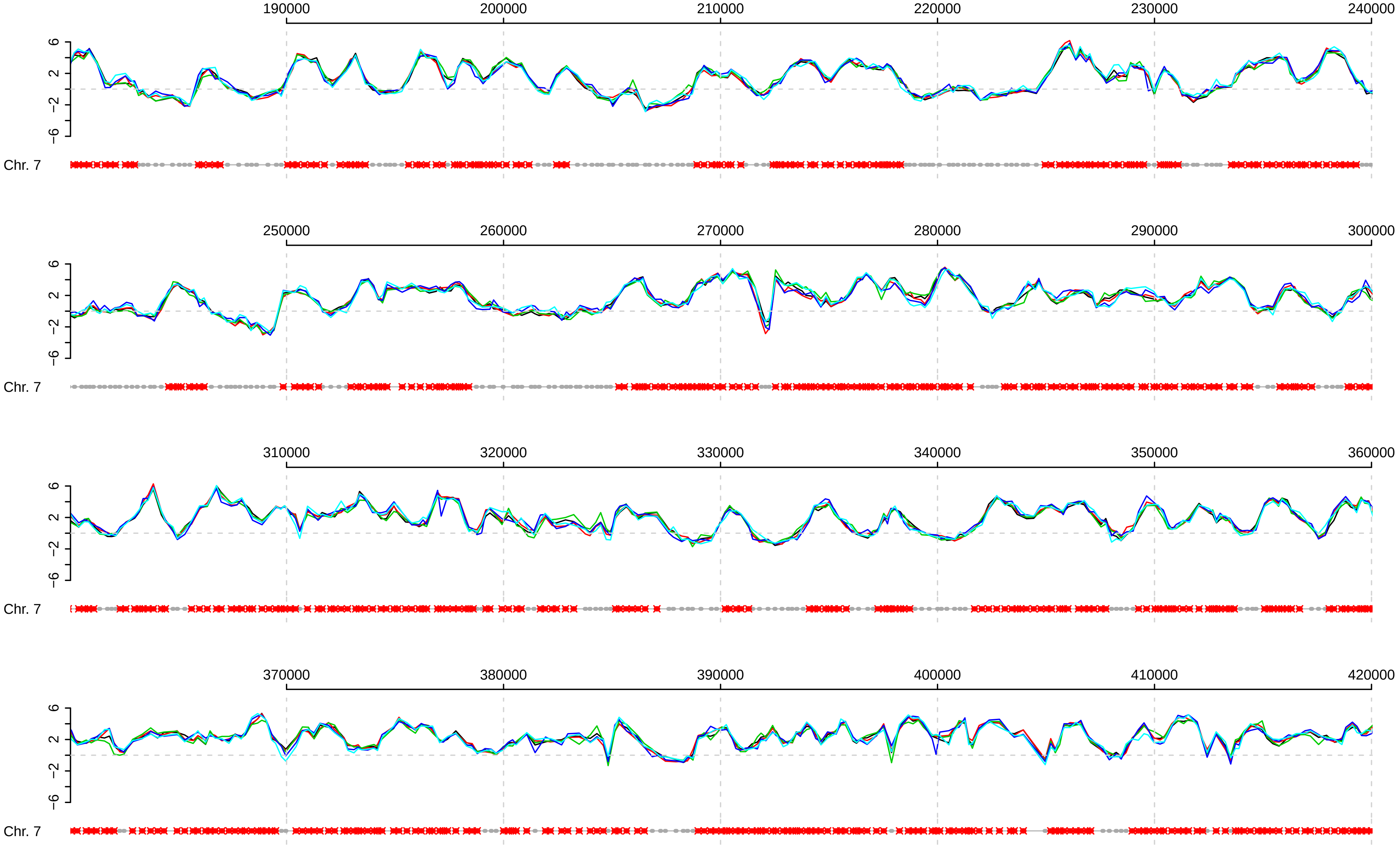

190000
200000
210000
220000
230000
240000
250000
260000
270000
280000
290000
300000
310000
320000
330000
340000
350000
360000
370000
380000
390000
400000
410000
420000
6
2
−2
−6
Chr. 7

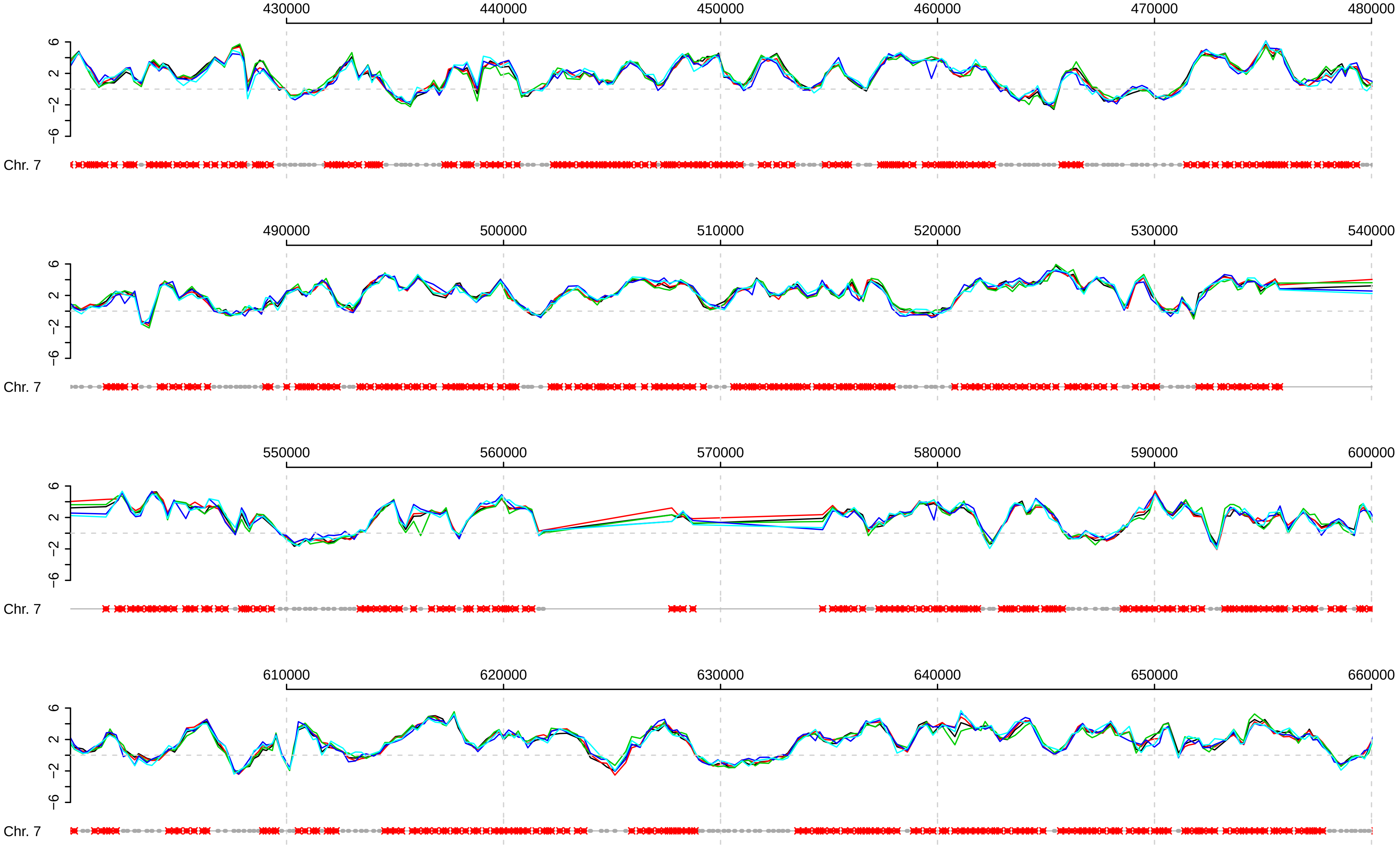
430000
440000
450000
460000
470000
480000
6
2
−2
−6
Chr. 7
490000
500000
510000
520000
530000
540000
6
2
−2
−6
Chr. 7
550000
560000
570000
580000
590000
600000
6
2
−2
−6
Chr. 7
610000
620000
630000
640000
650000
660000
6
2
−2
−6
Chr. 7

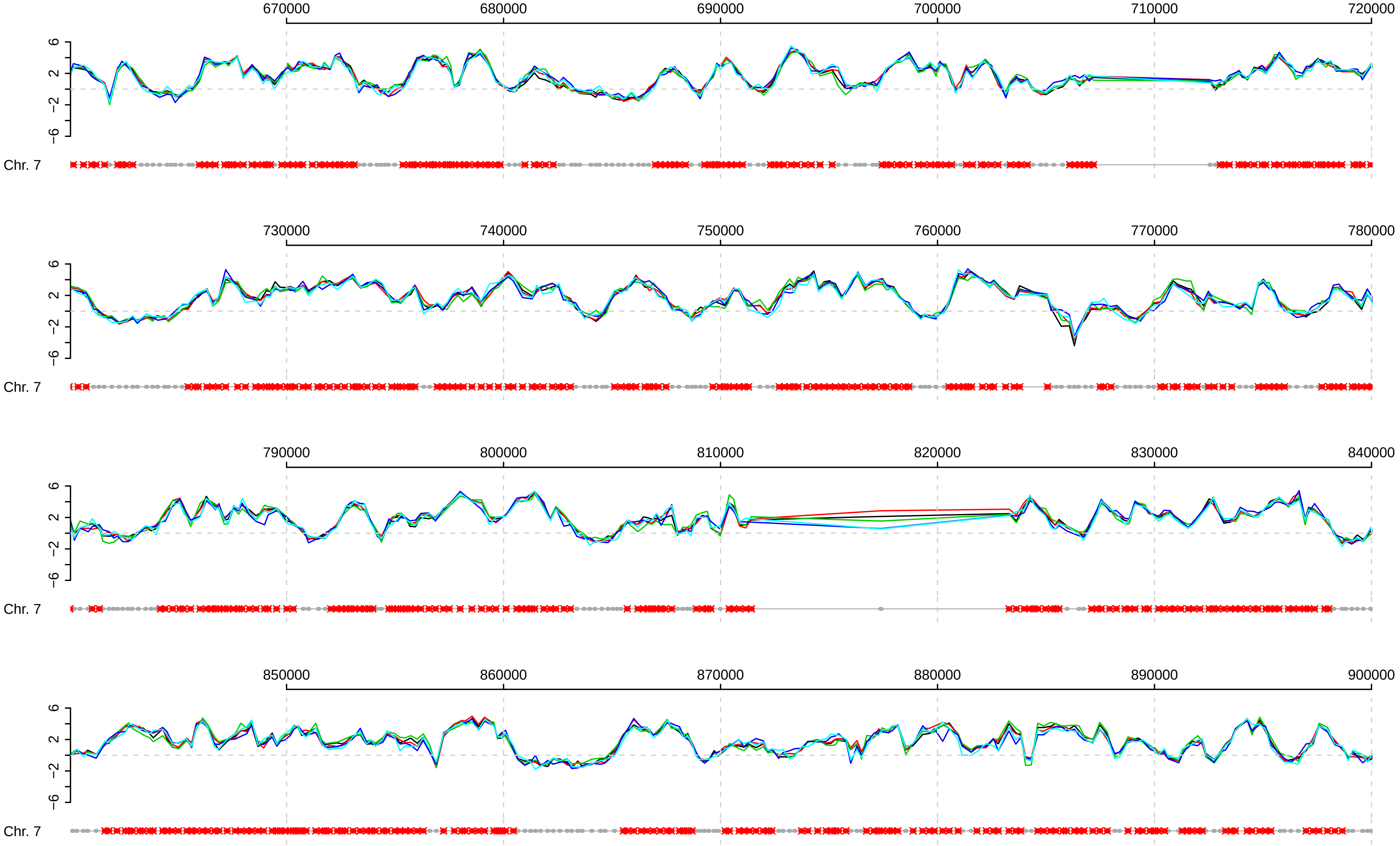
670000
680000
690000
700000
710000
720000
6
2
−2
−6
Chr. 7
730000
740000
750000
760000
770000
780000
6
2
−2
−6
Chr. 7
790000
800000
810000
820000
830000
840000
6
2
−2
−6
Chr. 7
850000
860000
870000
880000
890000
900000
6
2
−2
−6
Chr. 7

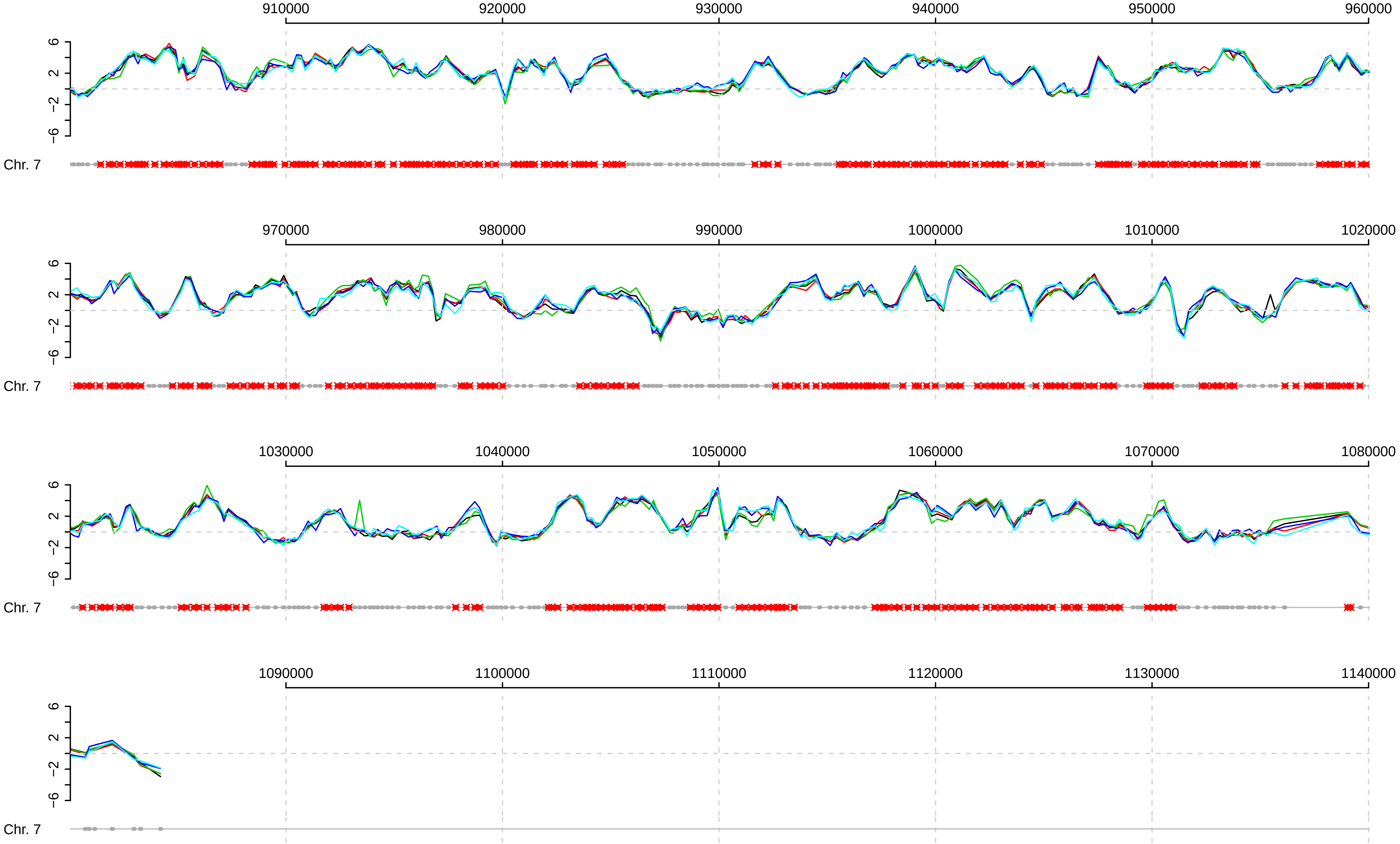

910000
920000
930000
940000
950000
960000
6
2
−2
−6
Chr. 7
970000
980000
990000
1000000
1010000
1020000
6
2
−2
−6
Chr. 7
1030000
1040000
1050000
1060000
1070000
1080000
6
2
−2
−6
Chr. 7
1090000
1100000
1110000
1120000
1130000
1140000
6
2
−2
−6
Chr. 7

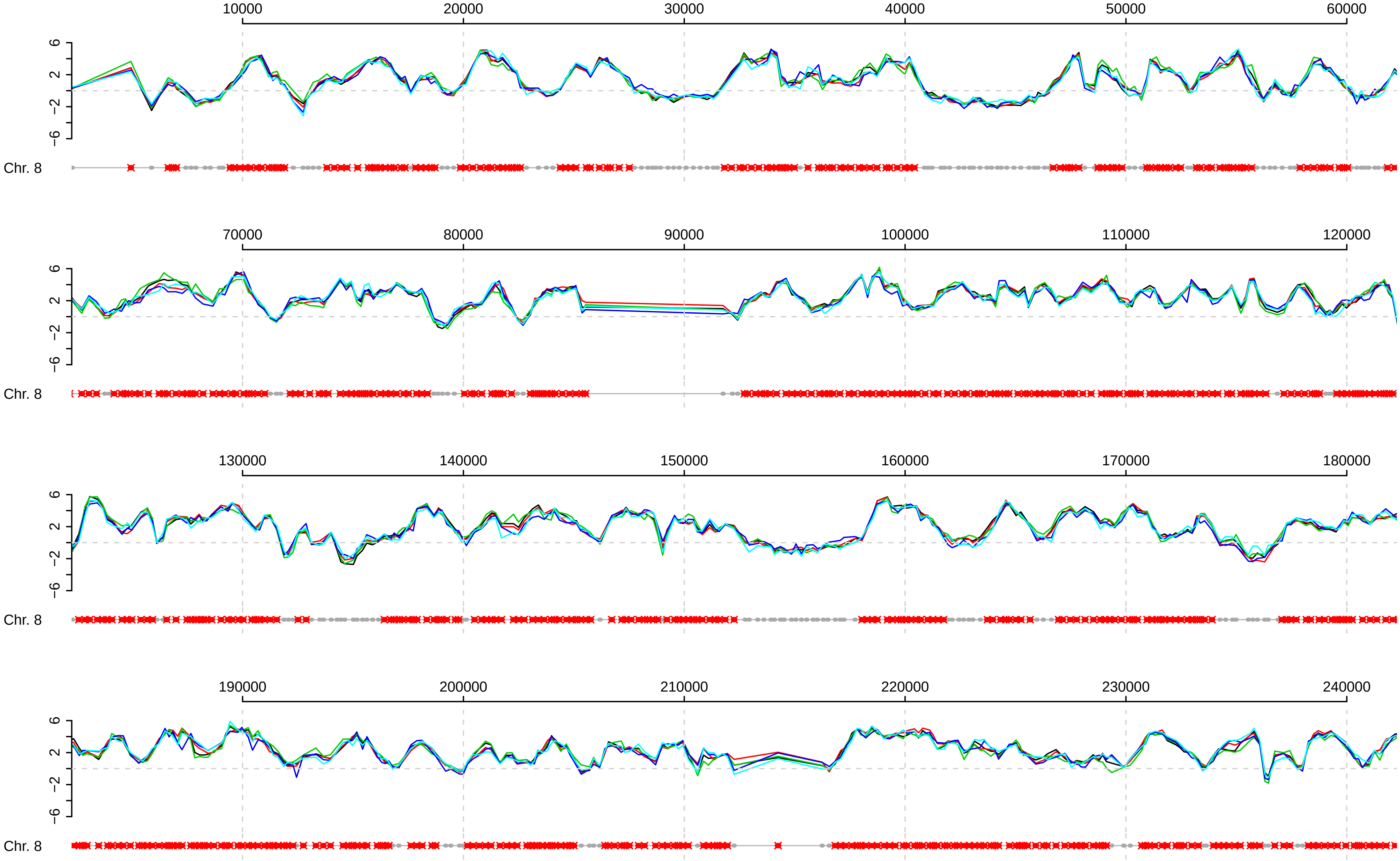

10000
20000
30000
40000
50000
60000
6
2
−2
−6
Chr. 8
70000
80000
90000
100000
110000
120000
6
2
−2
−6
Chr. 8
130000
140000
150000
160000
170000
180000
6
2
−2
−6
Chr. 8
190000
200000
210000
220000
230000
240000
6
2
−2
−6
Chr. 8

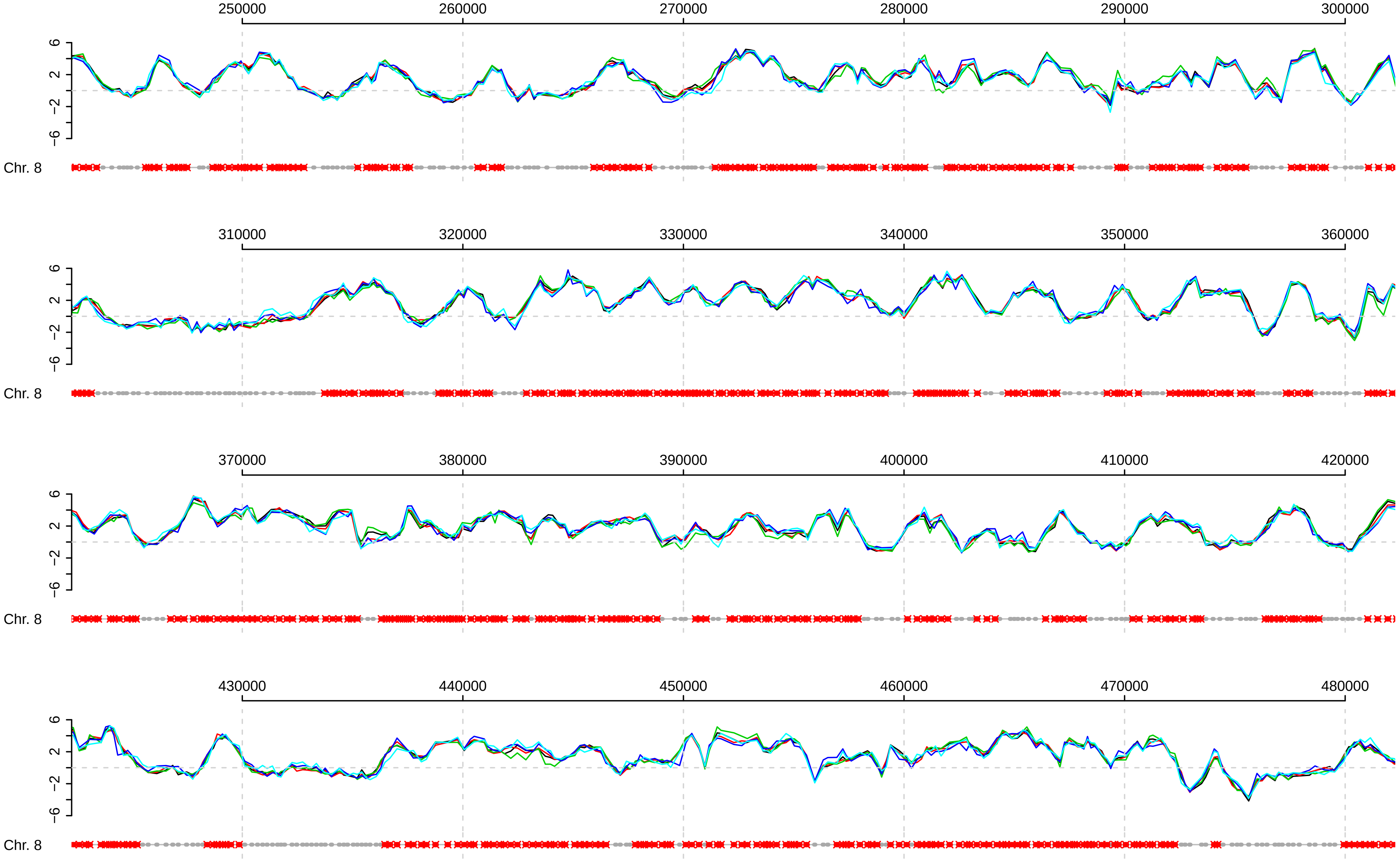
250000
260000
270000
280000
290000
300000
6
2
−2
−6
Chr. 8
310000
320000
330000
340000
350000
360000
6
2
−2
−6
Chr. 8
370000
380000
390000
400000
410000
420000
6
2
−2
−6
Chr. 8
430000
440000
450000
460000
470000
480000
6
2
−2
−6
Chr. 8

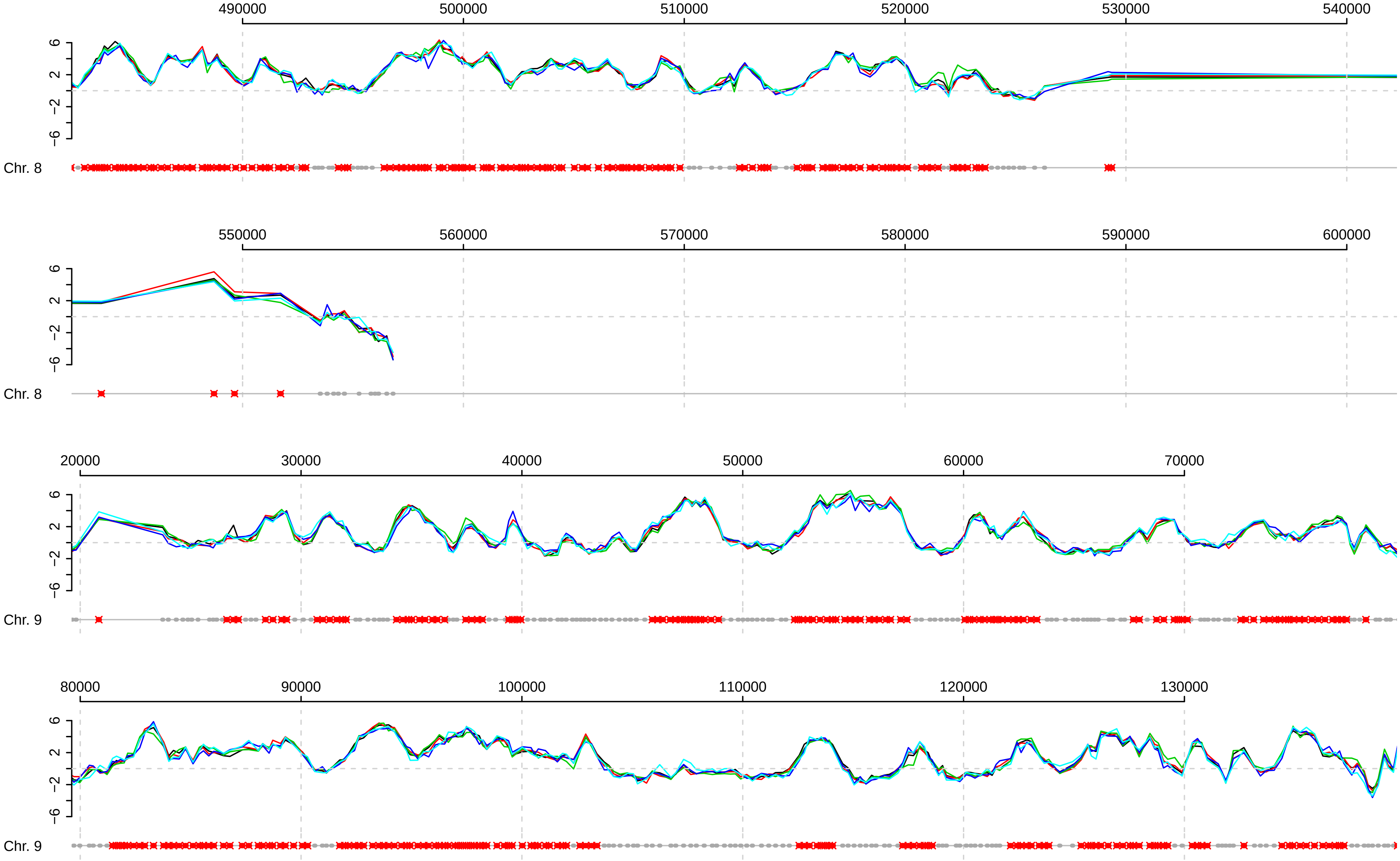
490000
500000
510000
520000
530000
540000
6
2
−2
−6
Chr. 8
550000
560000
570000
580000
590000
600000
6
2
−2
−6
Chr. 8
20000
30000
40000
50000
60000
70000
6
2
−2
−6
Chr. 9
80000
90000
100000
110000
120000
130000
6
2
−2
−6
Chr. 9

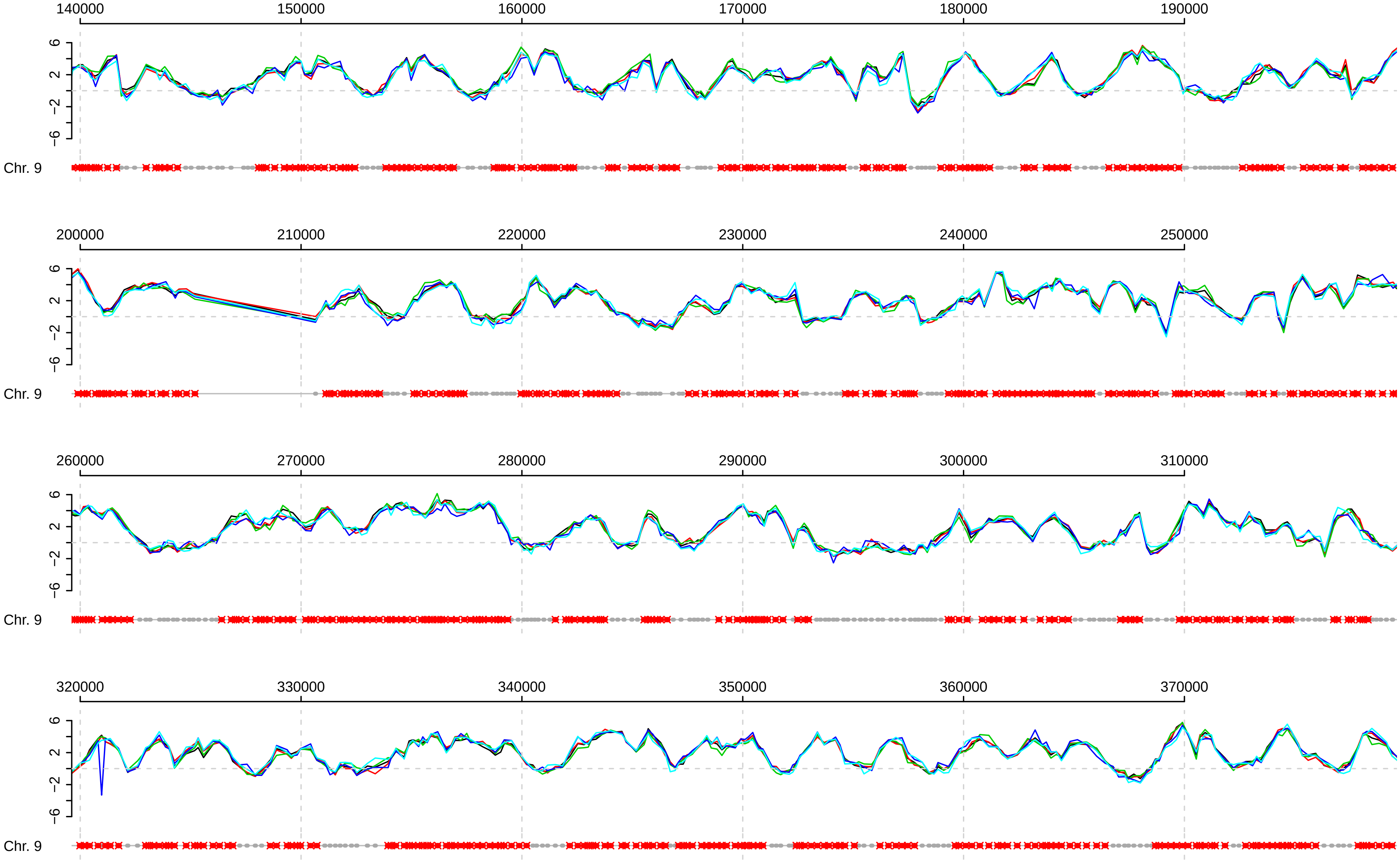
140000
150000
160000
170000
180000
190000
6
2
−2
−6
Chr. 9
200000
210000
220000
230000
240000
250000
6
2
−2
−6
Chr. 9
260000
270000
280000
290000
300000
310000
6
2
−2
−6
Chr. 9
320000
330000
340000
350000
360000
370000
6
2
−2
−6
Chr. 9

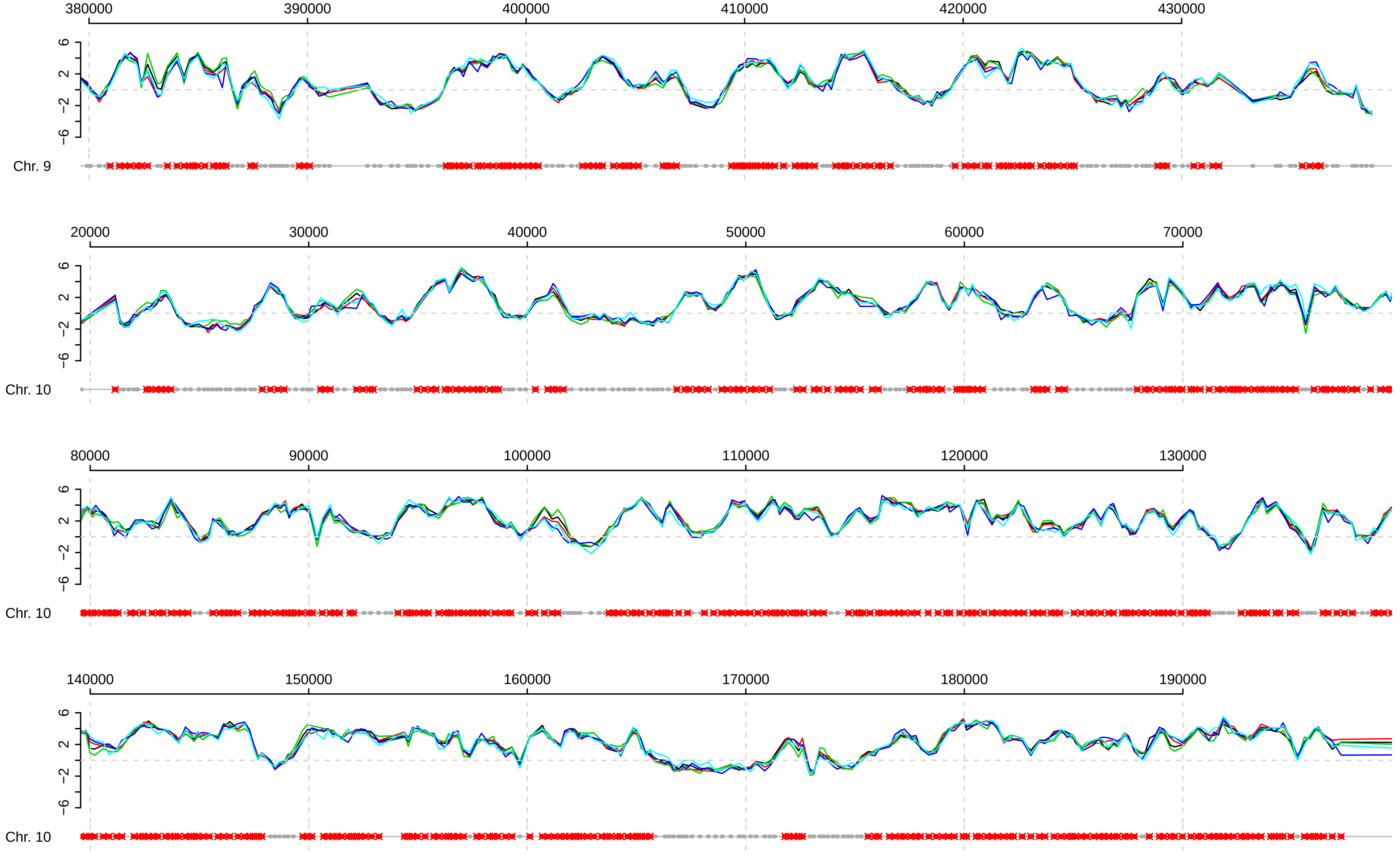
380000
390000
400000
410000
420000
430000
6
2
−2
−6
Chr. 9
20000
30000
40000
50000
60000
70000
Chr. 10
80000
90000
100000
110000
120000
130000
Chr. 10
140000
150000
160000
170000
180000
190000
Chr. 10

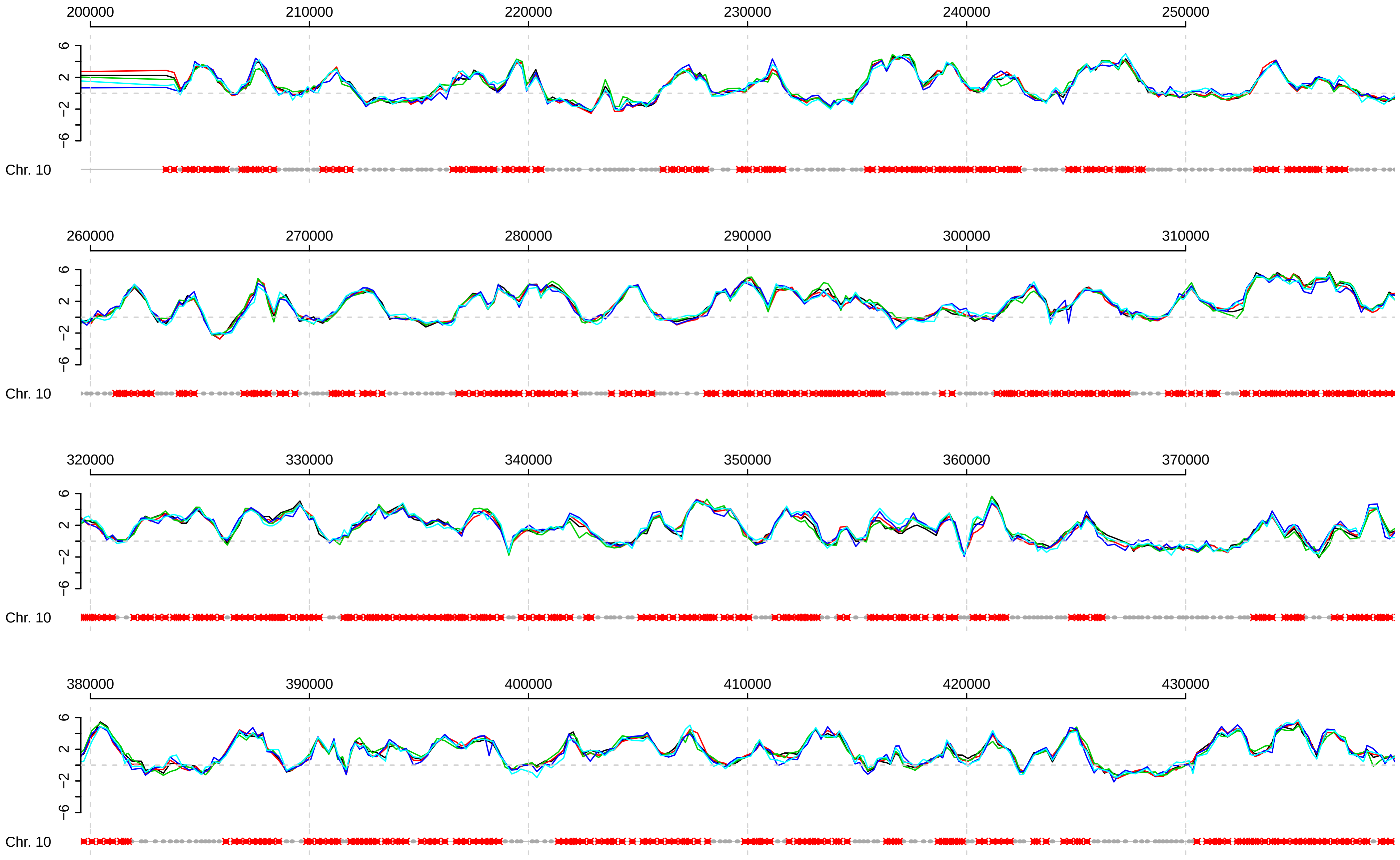

200000
210000
220000
230000
240000
250000
6
2
−2
−6
Chr. 10
260000
270000
280000
290000
300000
310000
6
2
−2
−6
Chr. 10
320000
330000
340000
350000
360000
370000
6
2
−2
−6
Chr. 10
380000
390000
400000
410000
420000
430000
6
2
−2
−6
Chr. 10

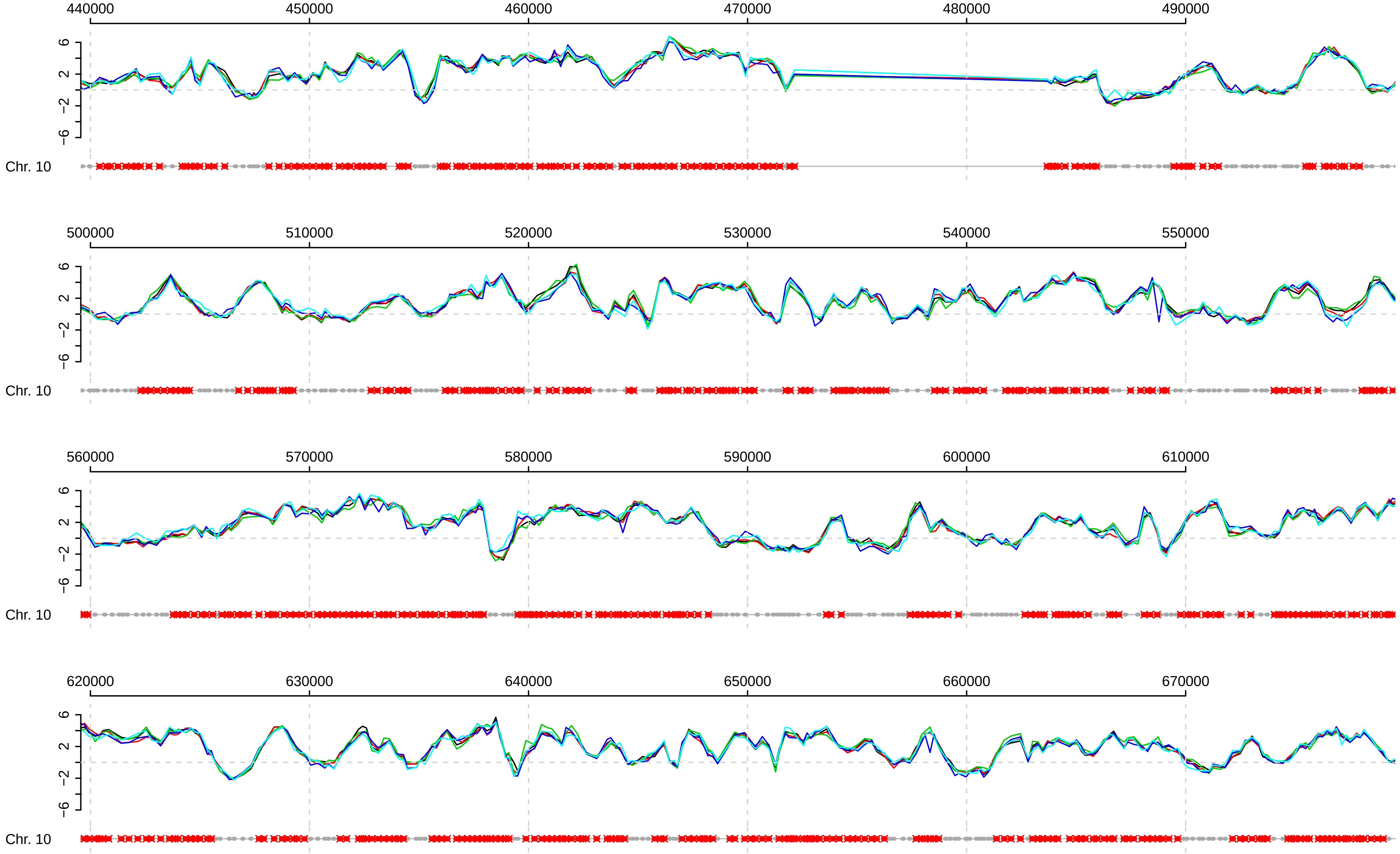

440000
450000
460000
470000
480000
490000
6
2
−2
−6
Chr. 10
500000
510000
520000
530000
540000
550000
6
2
−2
−6
Chr. 10
560000
570000
580000
590000
600000
610000
6
2
−2
−6
Chr. 10
620000
630000
640000
650000
660000
670000
6
2
−2
−6
Chr. 10

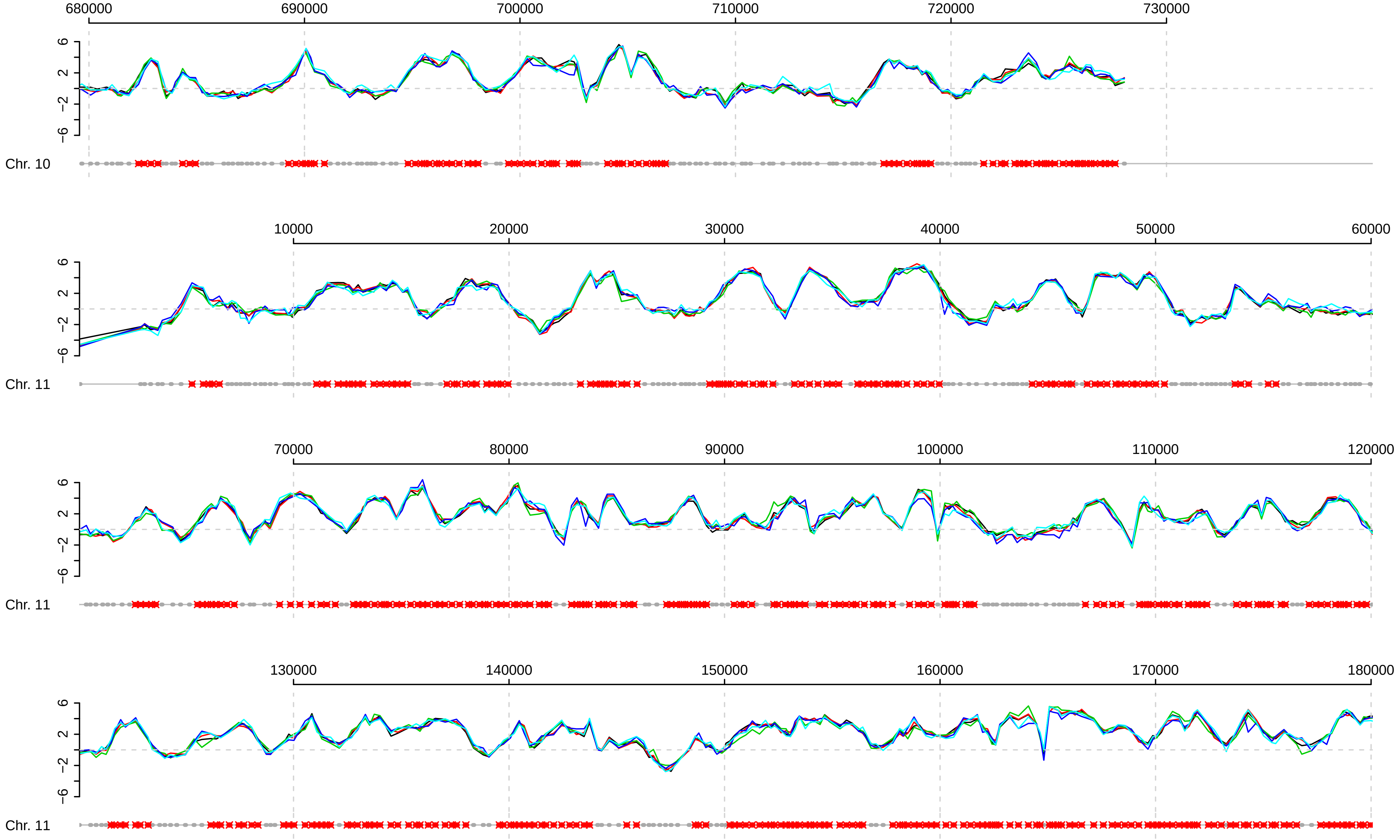
680000
690000
700000
710000
720000
730000
6
2
−2
−6
Chr. 10
10000
20000
30000
40000
50000
60000
Chr. 11
70000
80000
90000
100000
110000
120000
Chr. 11
130000
140000
150000
160000
170000
180000
Chr. 11

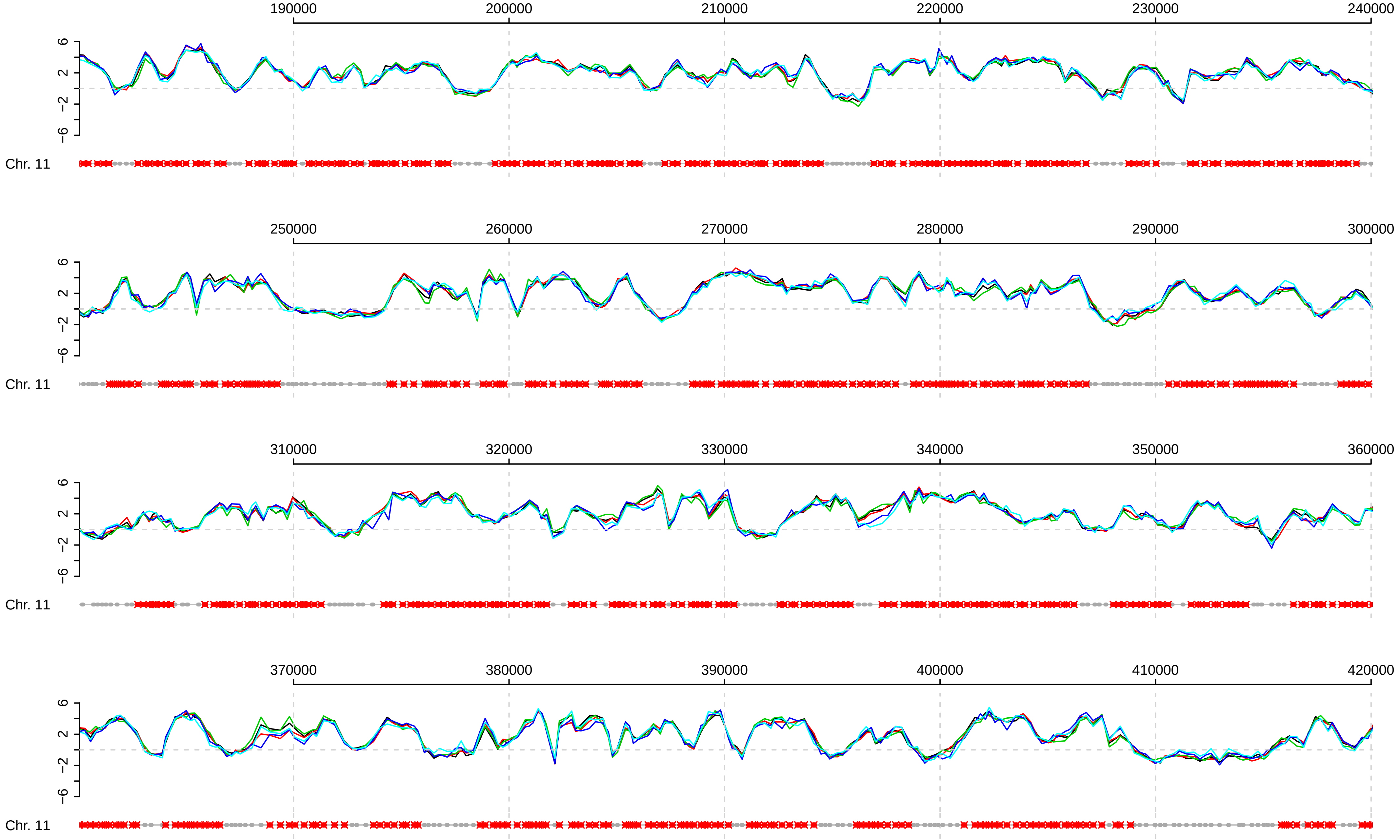

190000
200000
210000
220000
230000
240000
6
2
−2
−6
Chr. 11
250000
260000
270000
280000
290000
300000
6
2
−2
−6
Chr. 11
310000
320000
330000
340000
350000
360000
6
2
−2
−6
Chr. 11
370000
380000
390000
400000
410000
420000
6
2
−2
−6
Chr. 11

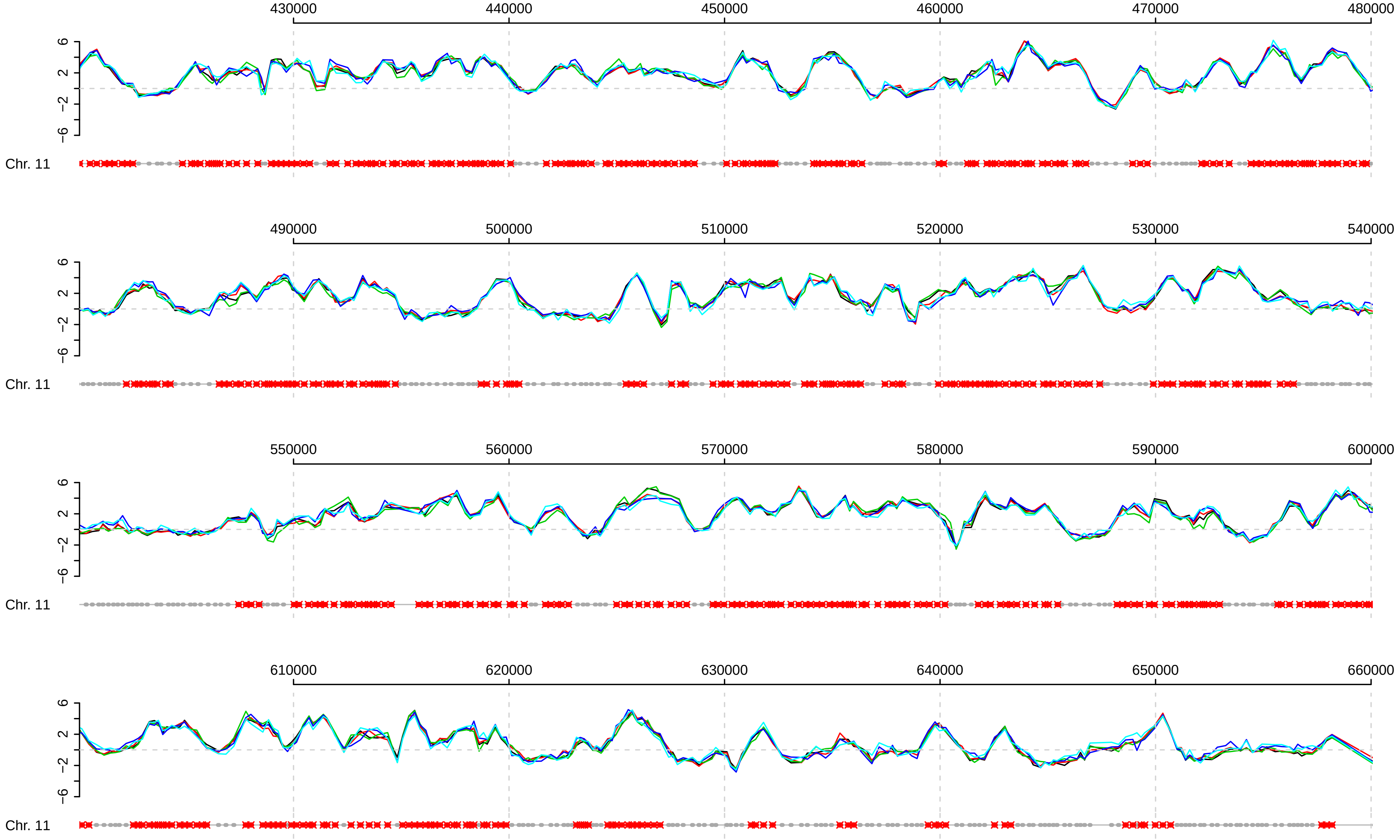

430000
440000
450000
460000
470000
480000
490000
500000
510000
520000
530000
540000
550000
560000
570000
580000
590000
600000
610000
620000
630000
640000
650000
660000
6
2
−2
−6
Chr. 11

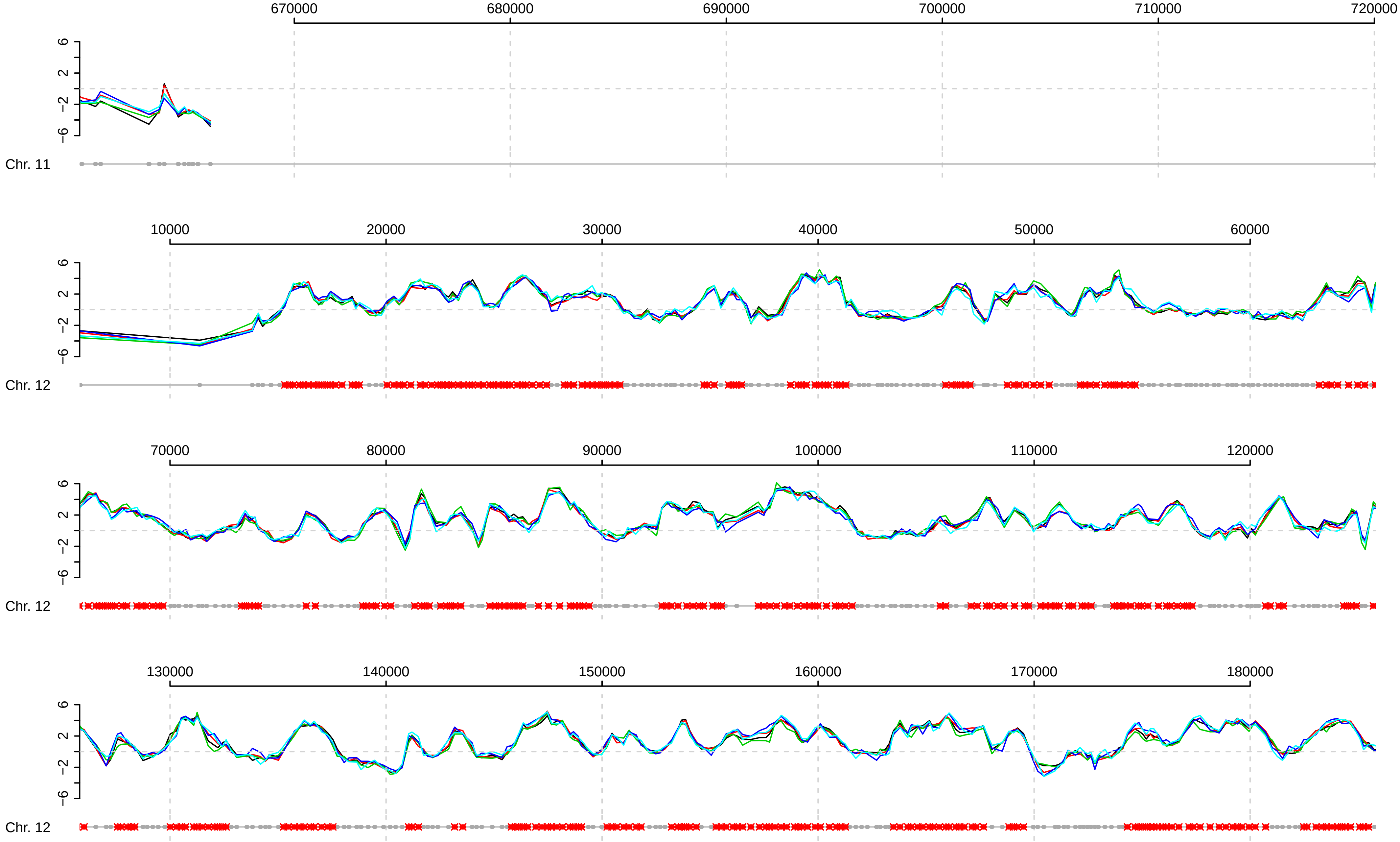

670000
680000
690000
700000
710000
720000
6
2
−2
−6
Chr. 11
10000
20000
30000
40000
50000
60000
6
2
−2
−6
Chr. 12
70000
80000
90000
100000
110000
120000
6
2
−2
−6
Chr. 12
130000
140000
150000
160000
170000
180000
6
2
−2
−6
Chr. 12

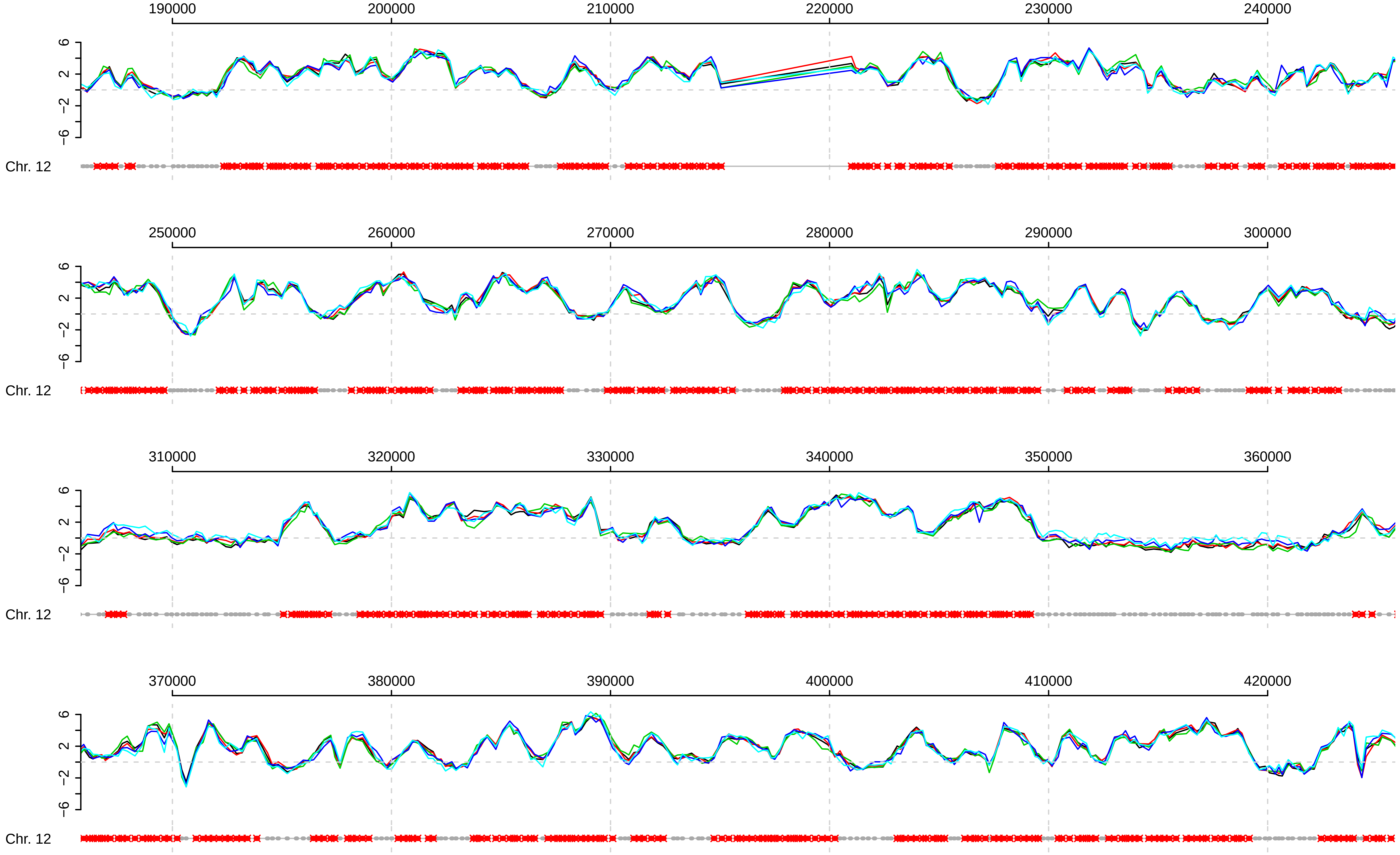

190000
200000
210000
220000
230000
240000
6
2
−2
−6
Chr. 12
250000
260000
270000
280000
290000
300000
6
2
−2
−6
Chr. 12
310000
320000
330000
340000
350000
360000
6
2
−2
−6
Chr. 12
370000
380000
390000
400000
410000
420000
6
2
−2
−6
Chr. 12

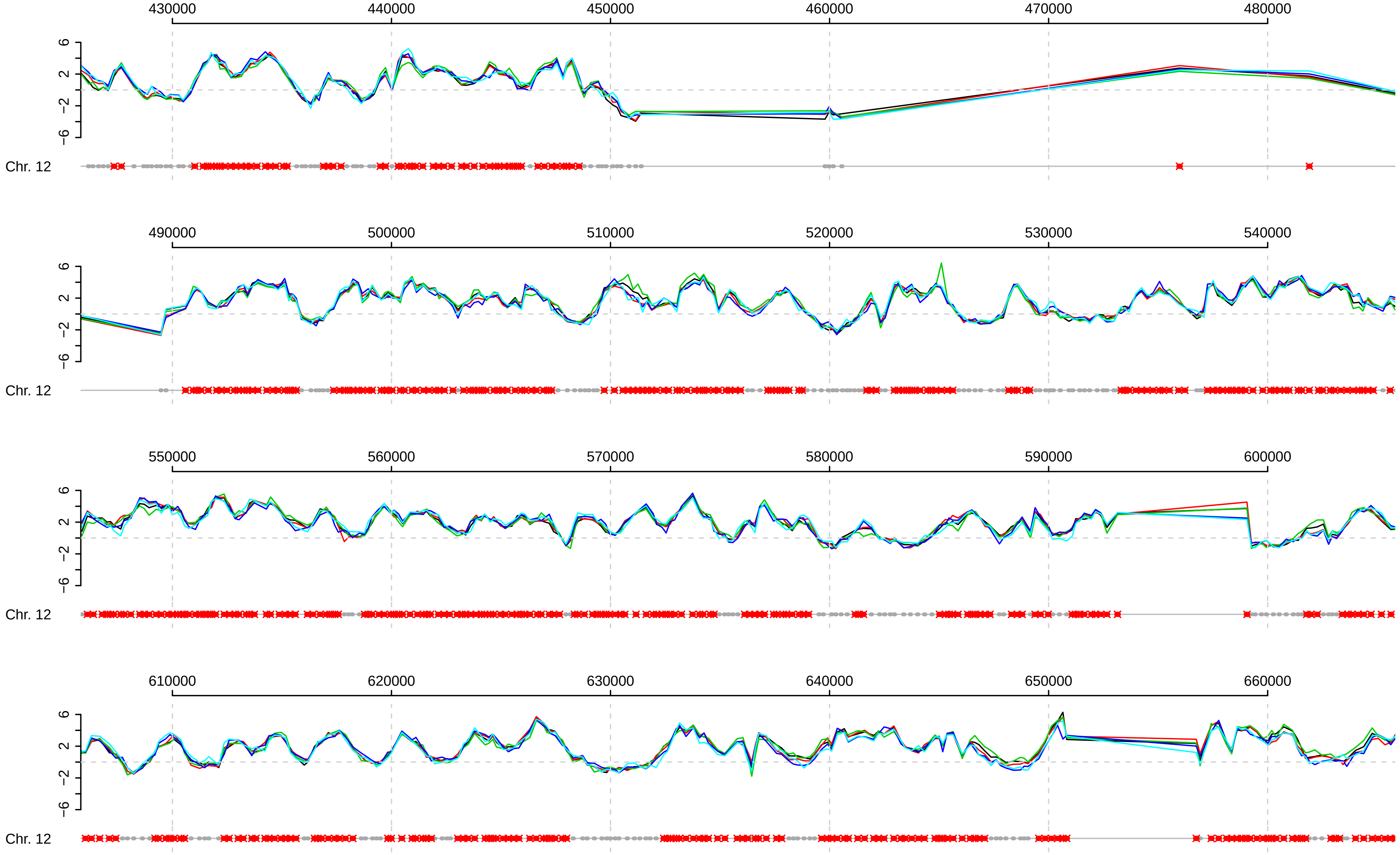

430000
440000
450000
460000
470000
480000
6
2
−2
−6
Chr. 12
490000
500000
510000
520000
530000
540000
6
2
−2
−6
Chr. 12
550000
560000
570000
580000
590000
600000
6
2
−2
−6
Chr. 12
610000
620000
630000
640000
650000
660000
6
2
−2
−6
Chr. 12

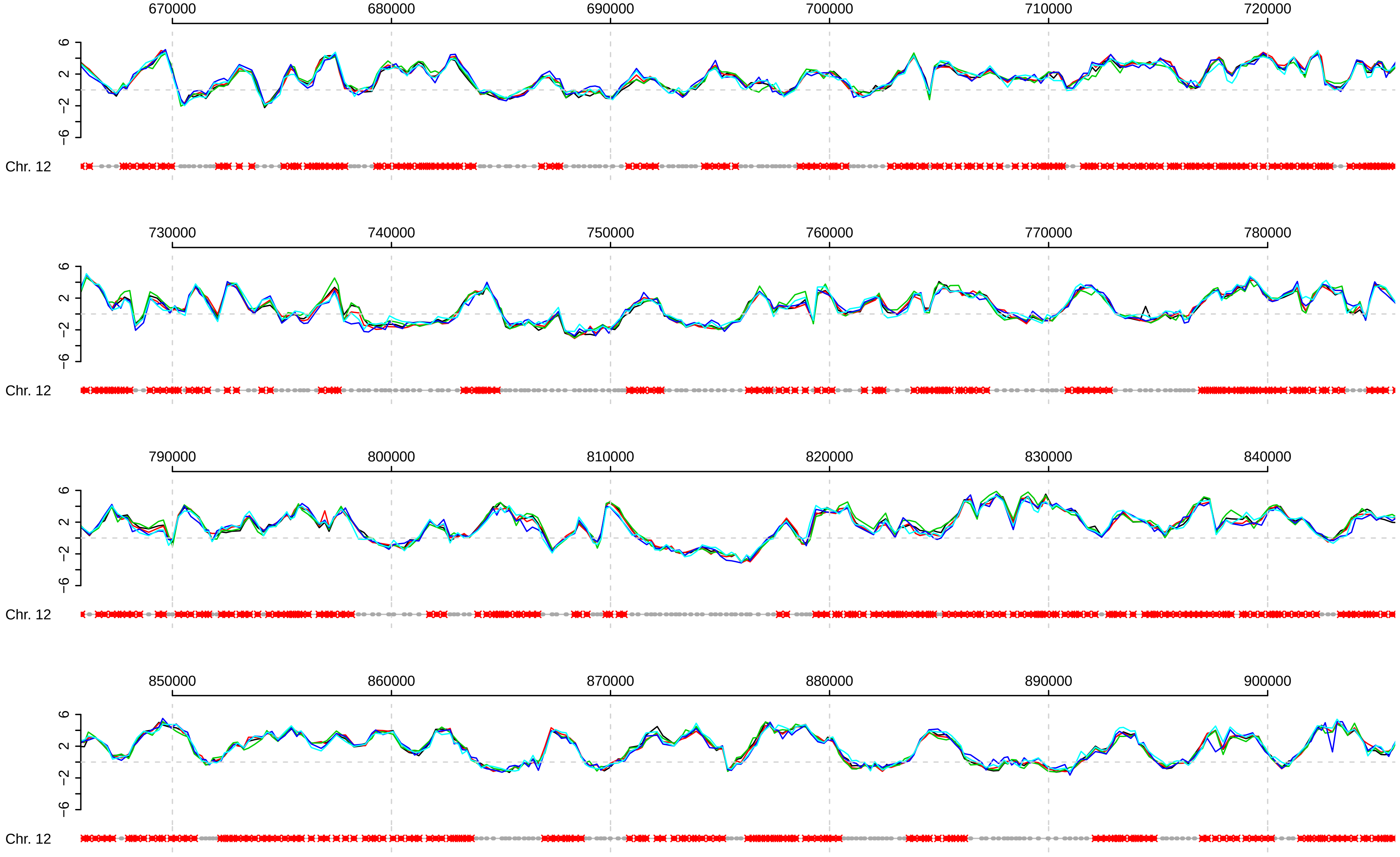

670000
680000
690000
700000
710000
720000
6
2
−2
−6
Chr. 12
730000
740000
750000
760000
770000
780000
6
2
−2
−6
Chr. 12
790000
800000
810000
820000
830000
840000
6
2
−2
−6
Chr. 12
850000
860000
870000
880000
890000
900000
6
2
−2
−6
Chr. 12

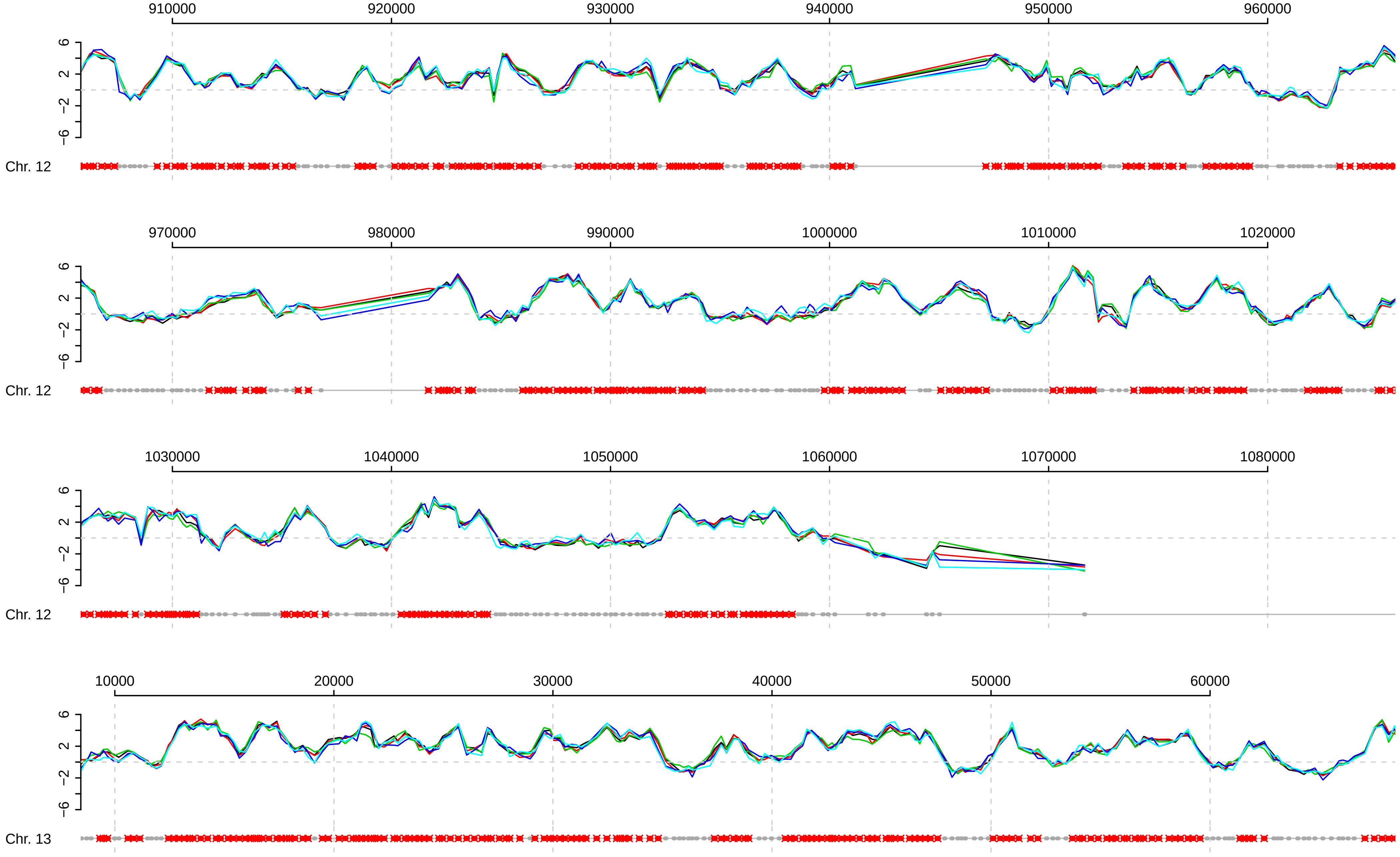

910000
920000
930000
940000
950000
960000
6
2
−2
−6
Chr. 12
970000
980000
990000
1000000
1010000
1020000
6
2
−2
−6
Chr. 12
1030000
1040000
1050000
1060000
1070000
1080000
6
2
−2
−6
Chr. 12
10000
20000
30000
40000
50000
60000
6
2
−2
−6
Chr. 13

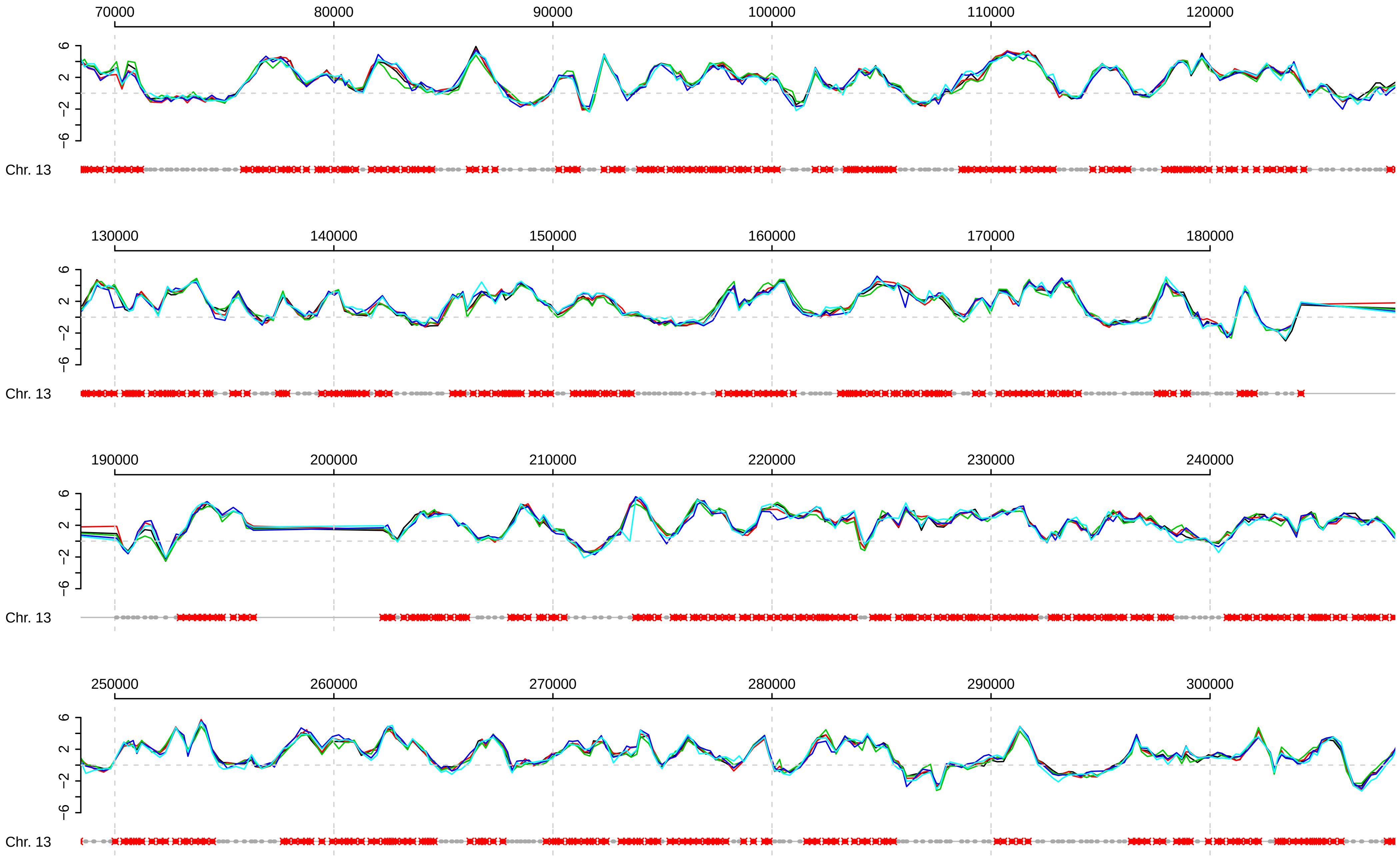
70000
80000
90000
100000
110000
120000
6
2
−2
−6
Chr. 13
130000
140000
150000
160000
170000
180000
6
2
−2
−6
Chr. 13
190000
200000
210000
220000
230000
240000
6
2
−2
−6
Chr. 13
250000
260000
270000
280000
290000
300000
6
2
−2
−6
Chr. 13

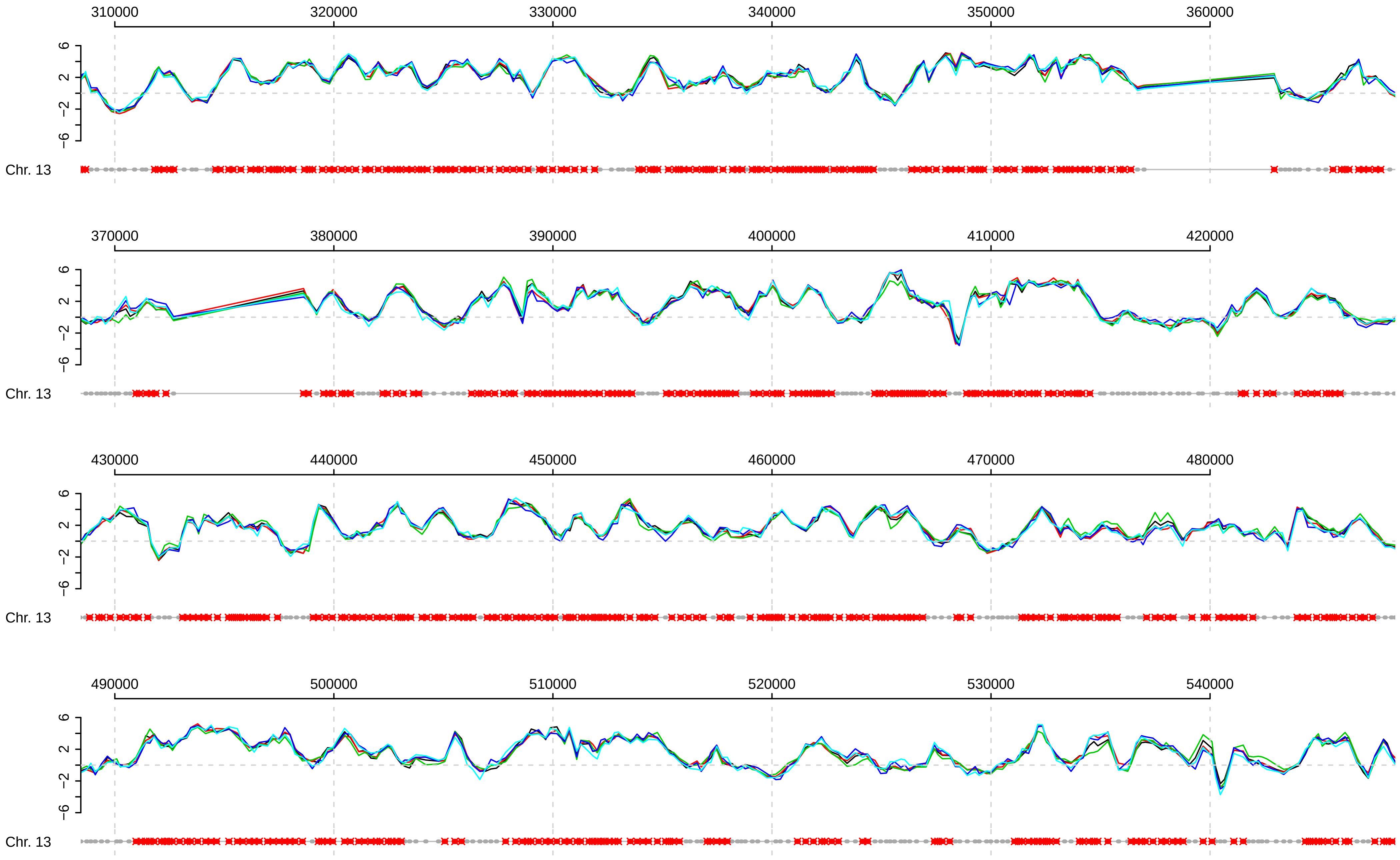
310000
320000
330000
340000
350000
360000
6
2
−2
−6
Chr. 13
370000
380000
390000
400000
410000
420000
6
2
−2
−6
Chr. 13
430000
440000
450000
460000
470000
480000
6
2
−2
−6
Chr. 13
490000
500000
510000
520000
530000
540000
6
2
−2
−6
Chr. 13

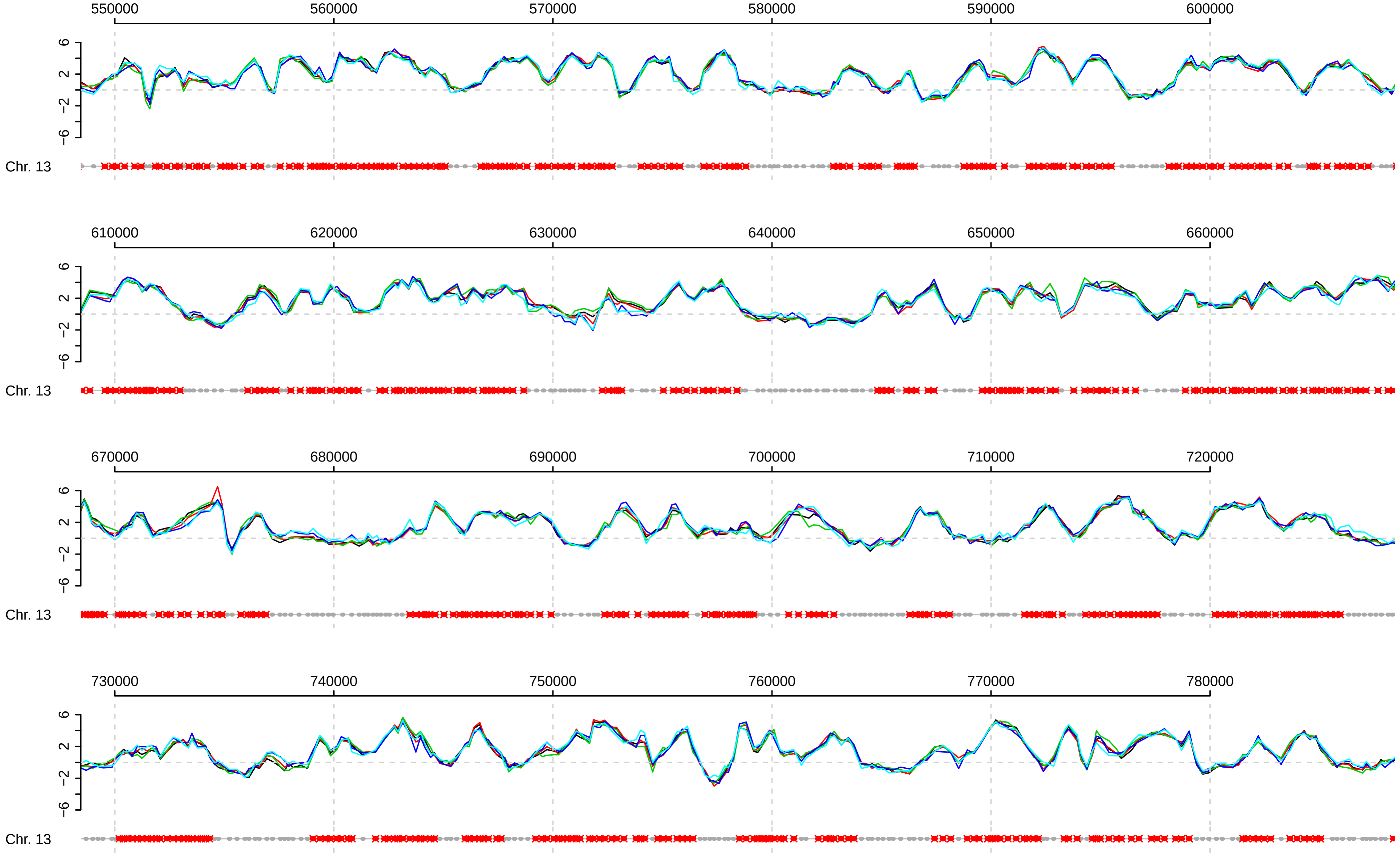
550000
560000
570000
580000
590000
600000
6
2
−2
−6
Chr. 13
610000
620000
630000
640000
650000
660000
6
2
−2
−6
Chr. 13
670000
680000
690000
700000
710000
720000
6
2
−2
−6
Chr. 13
730000
740000
750000
760000
770000
780000
6
2
−2
−6
Chr. 13

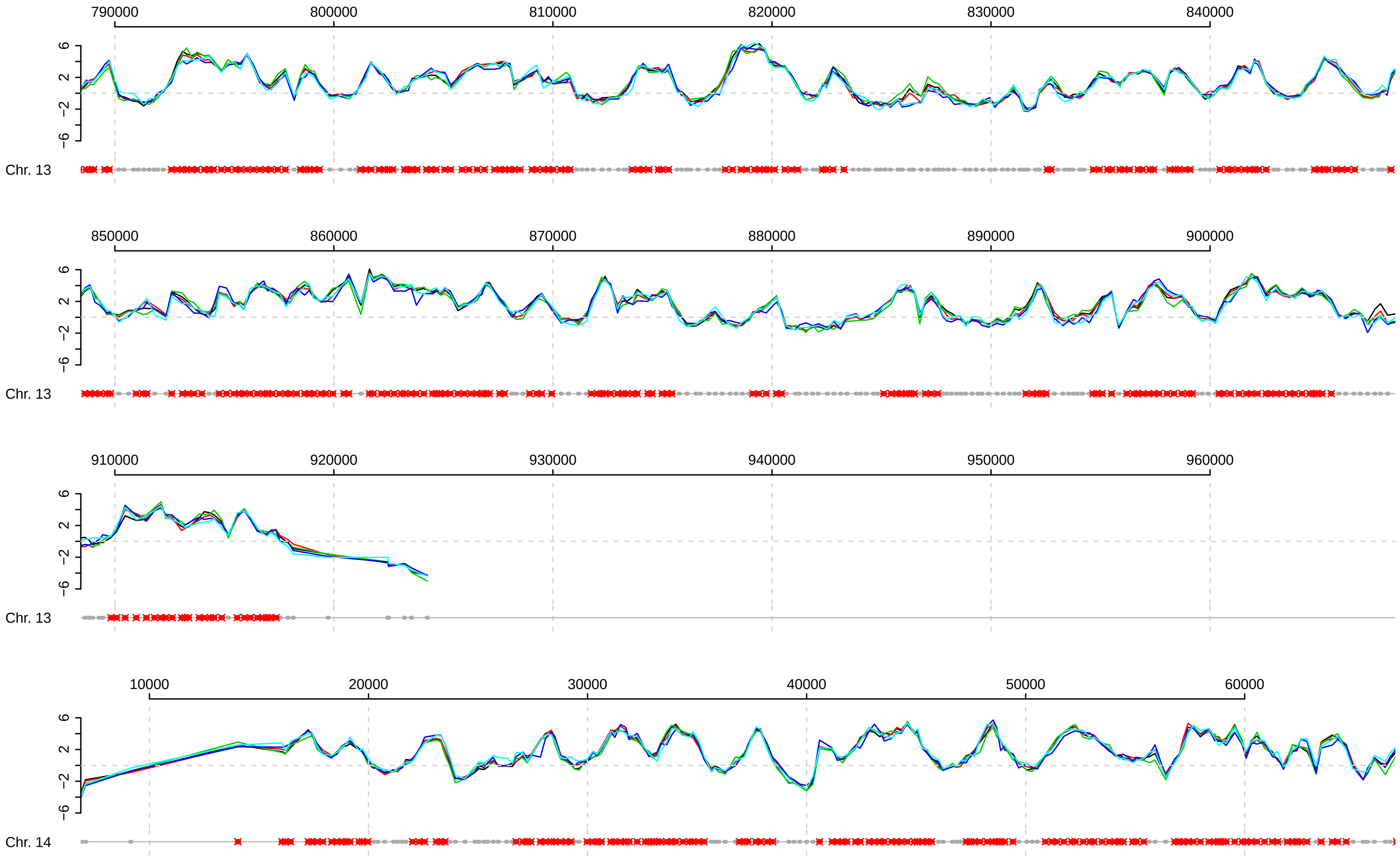

790000
800000
810000
820000
830000
840000
6
2
−2
−6
Chr. 13
850000
860000
870000
880000
890000
900000
6
2
−2
−6
Chr. 13
910000
920000
930000
940000
950000
960000
6
2
−2
−6
Chr. 13
10000
20000
30000
40000
50000
60000
6
2
−2
−6
Chr. 14

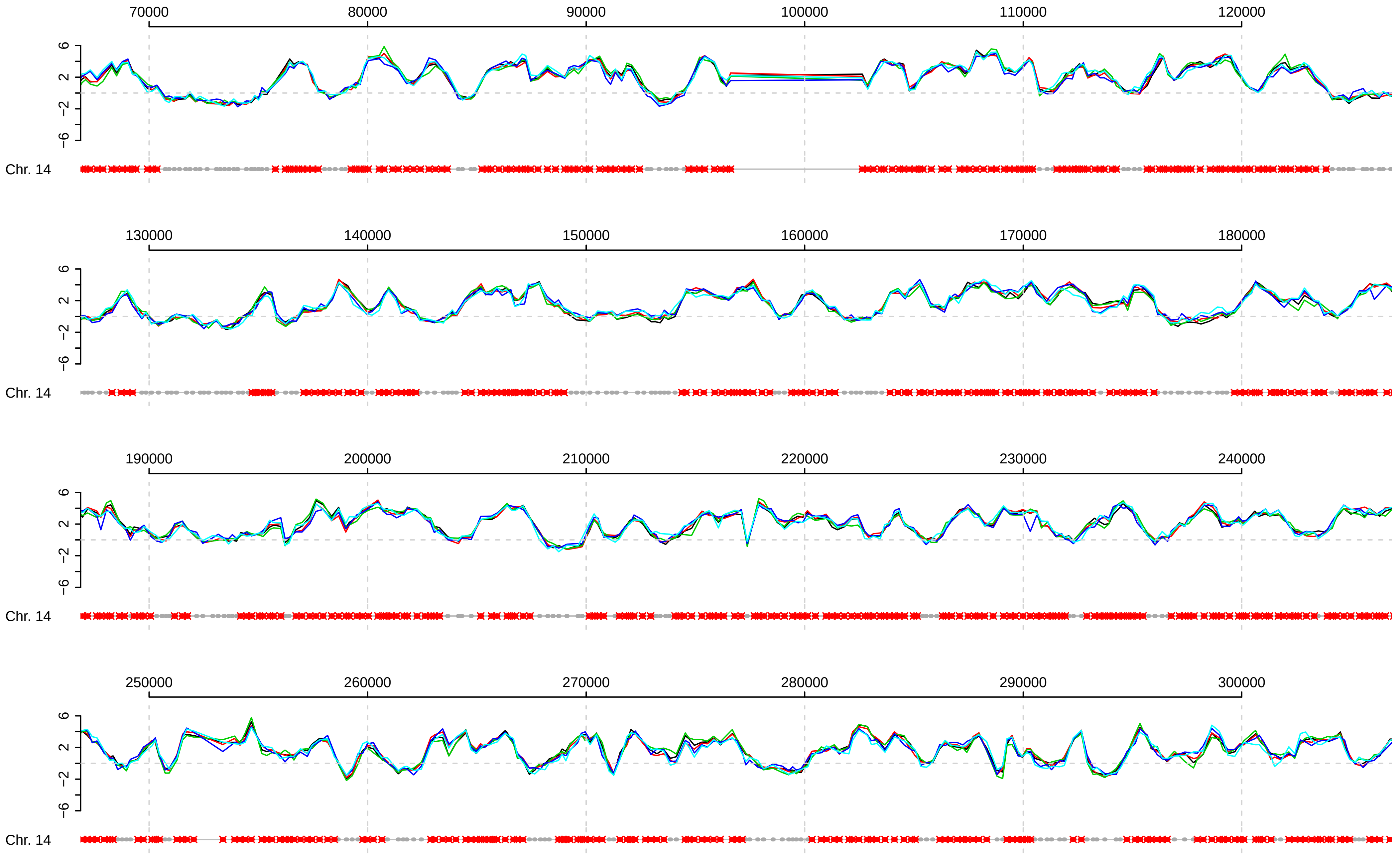

70000
80000
90000
100000
110000
120000
6
2
−2
−6
Chr. 14
130000
140000
150000
160000
170000
180000
6
2
−2
−6
Chr. 14
190000
200000
210000
220000
230000
240000
6
2
−2
−6
Chr. 14
250000
260000
270000
280000
290000
300000
6
2
−2
−6
Chr. 14

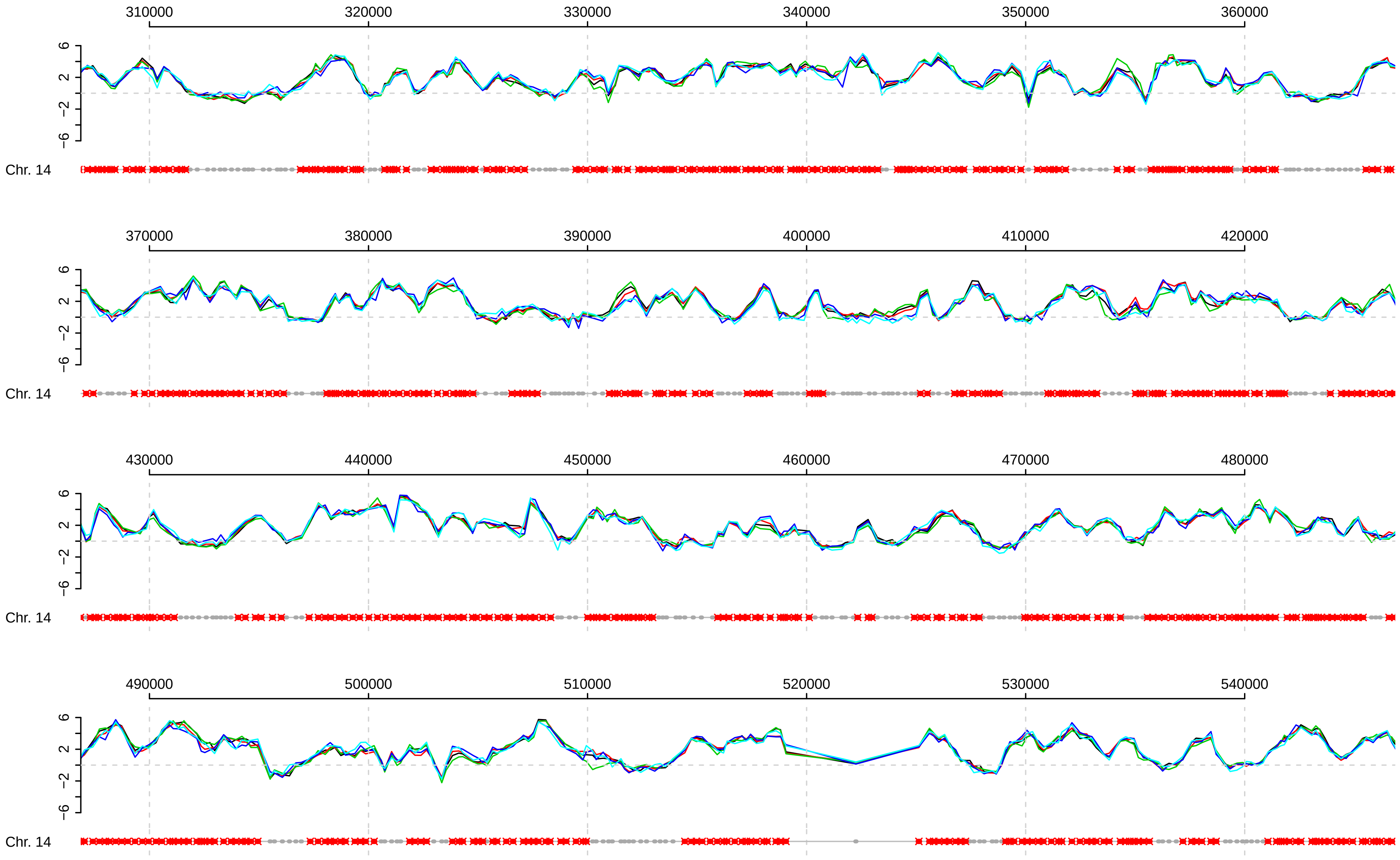

310000
320000
330000
340000
350000
360000
6
2
−2
−6
Chr. 14
370000
380000
390000
400000
410000
420000
6
2
−2
−6
Chr. 14
430000
440000
450000
460000
470000
480000
6
2
−2
−6
Chr. 14
490000
500000
510000
520000
530000
540000
6
2
−2
−6
Chr. 14

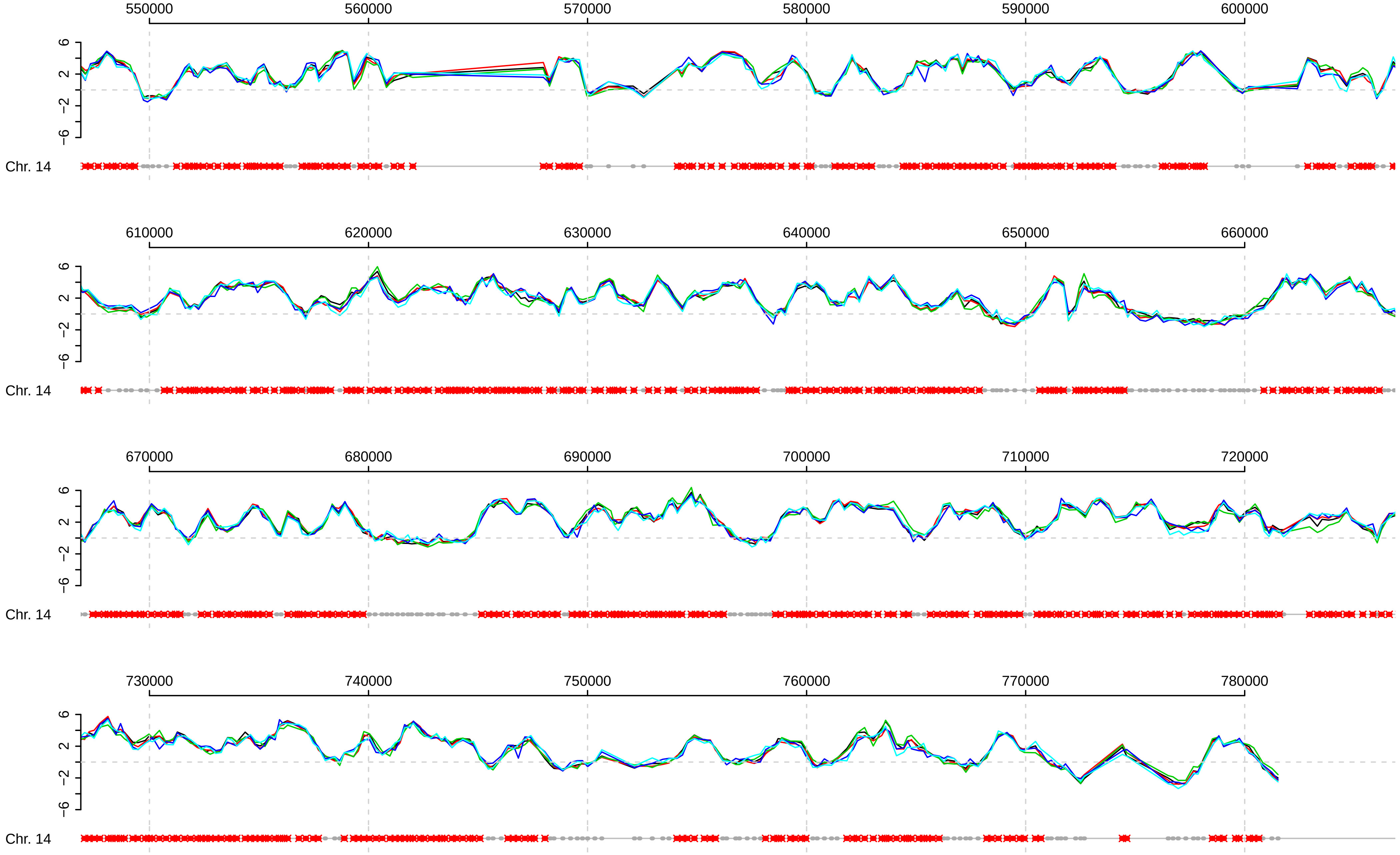
550000
560000
570000
580000
590000
600000
6
2
−2
−6
Chr. 14
610000
620000
630000
640000
650000
660000
6
2
−2
−6
Chr. 14
670000
680000
690000
700000
710000
720000
6
2
−2
−6
Chr. 14
730000
740000
750000
760000
770000
780000
6
2
−2
−6
Chr. 14

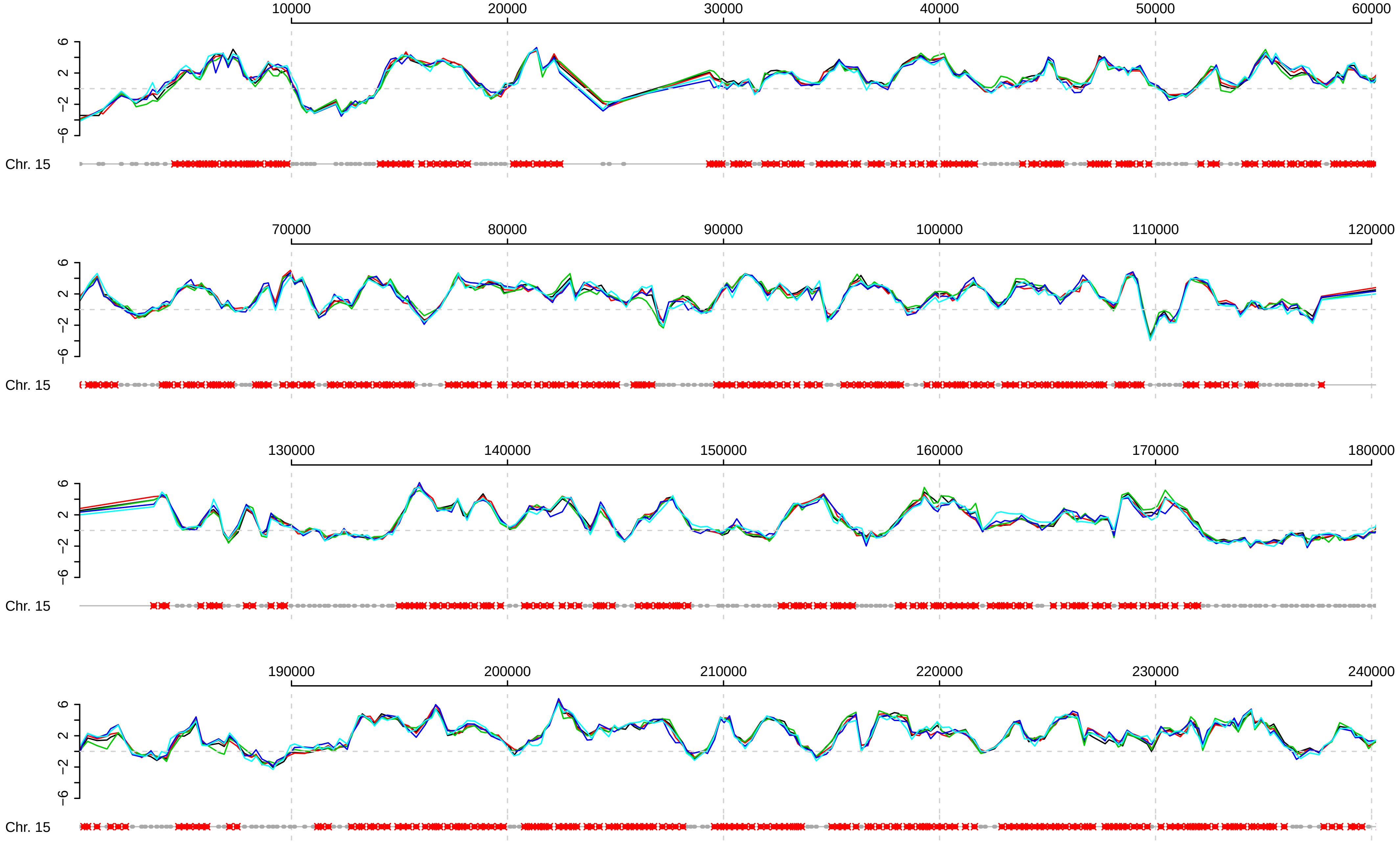

10000
20000
30000
40000
50000
60000
70000
80000
90000
100000
110000
120000
130000
140000
150000
160000
170000
180000
190000
200000
210000
220000
230000
240000
Chr. 15

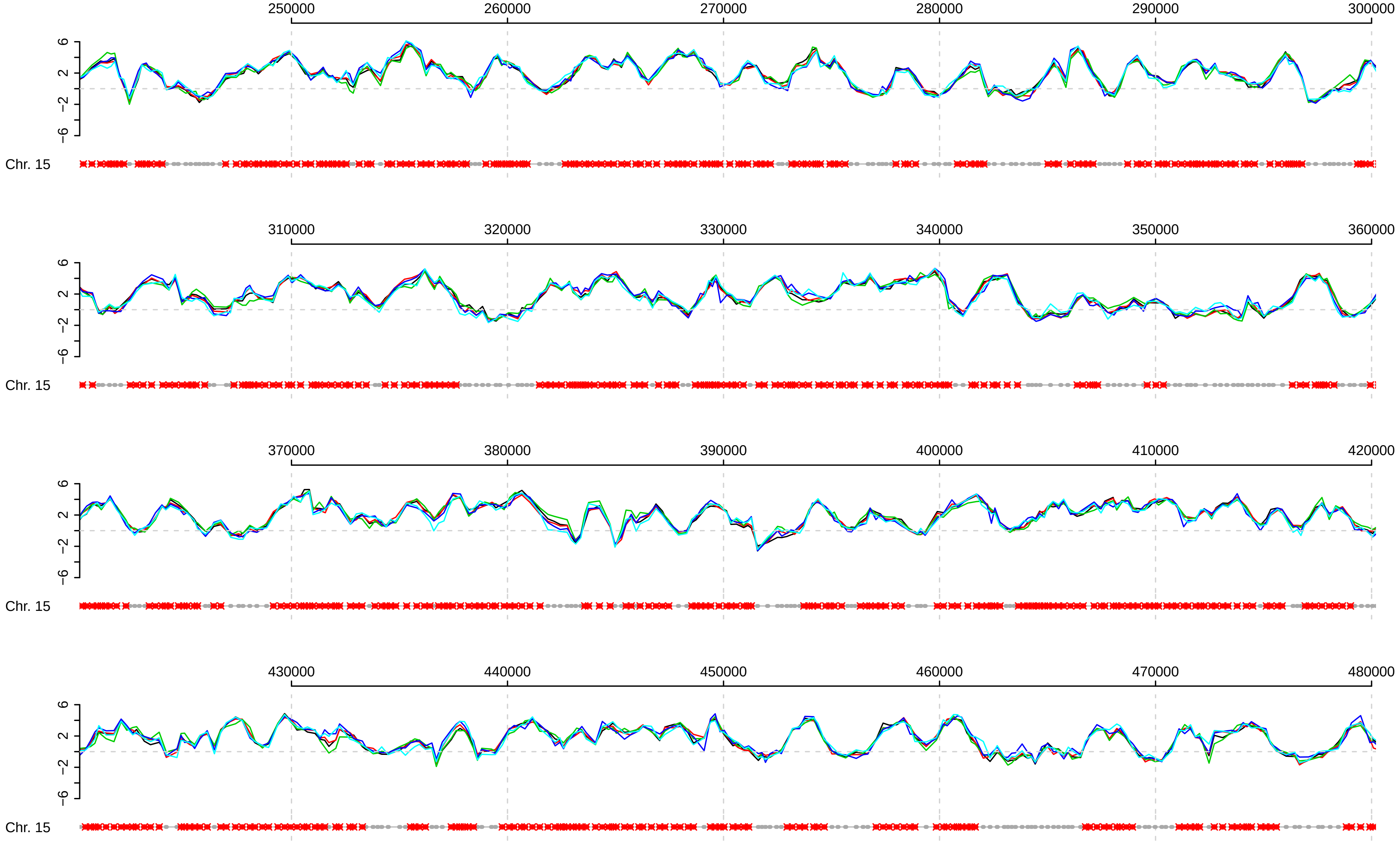
250000
260000
270000
280000
290000
300000
6
2
−2
−6
Chr. 15
310000
320000
330000
340000
350000
360000
6
2
−2
−6
Chr. 15
370000
380000
390000
400000
410000
420000
6
2
−2
−6
Chr. 15
430000
440000
450000
460000
470000
480000
6
2
−2
−6
Chr. 15

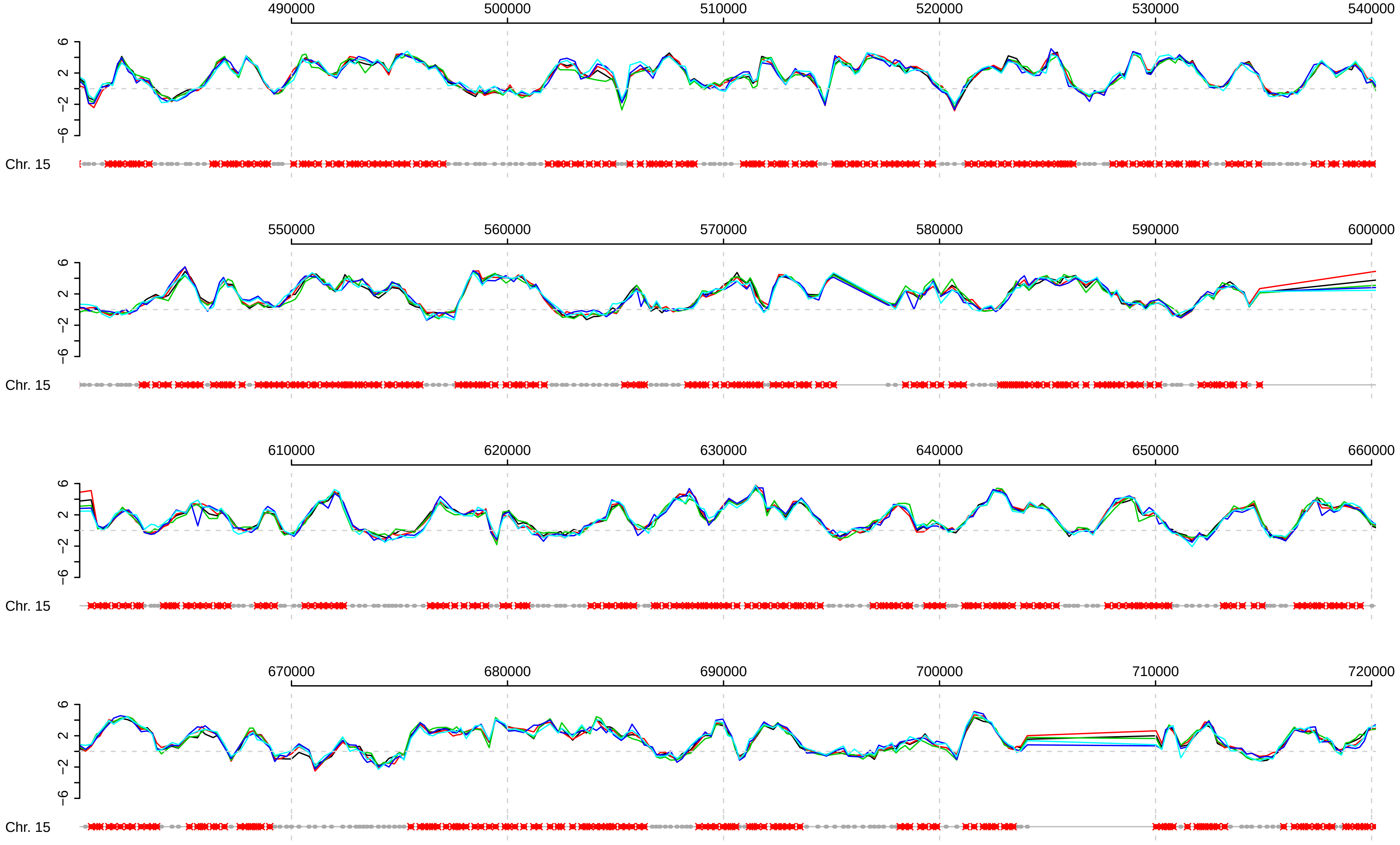
490000
500000
510000
520000
530000
540000
6
2
−2
−6
Chr. 15
550000
560000
570000
580000
590000
600000
6
2
−2
−6
Chr. 15
610000
620000
630000
640000
650000
660000
6
2
−2
−6
Chr. 15
670000
680000
690000
700000
710000
720000
6
2
−2
−6
Chr. 15

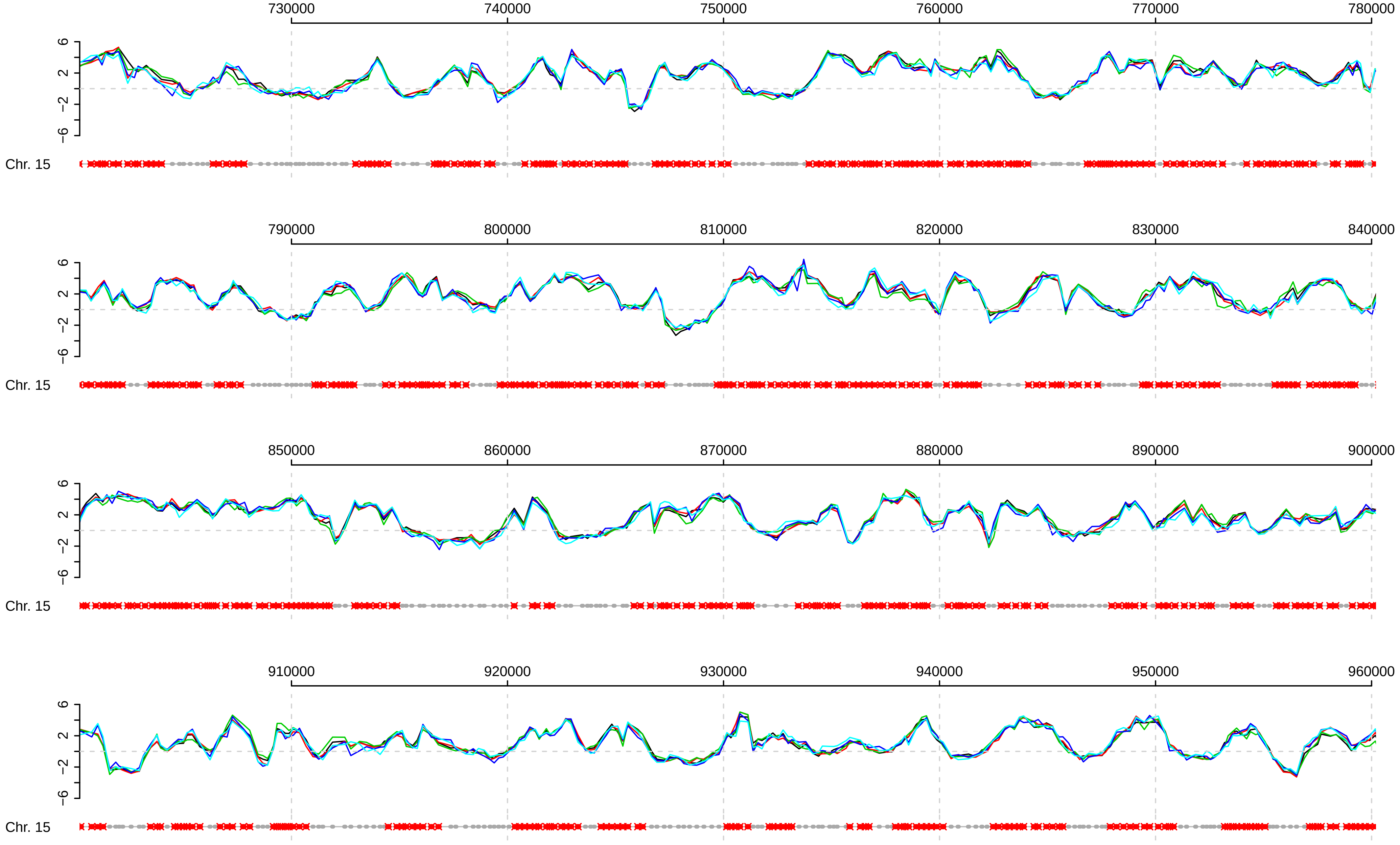

730000
740000
750000
760000
770000
780000
6
2
−2
−6
Chr. 15
790000
800000
810000
820000
830000
840000
6
2
−2
−6
Chr. 15
850000
860000
870000
880000
890000
900000
6
2
−2
−6
Chr. 15
910000
920000
930000
940000
950000
960000
6
2
−2
−6
Chr. 15

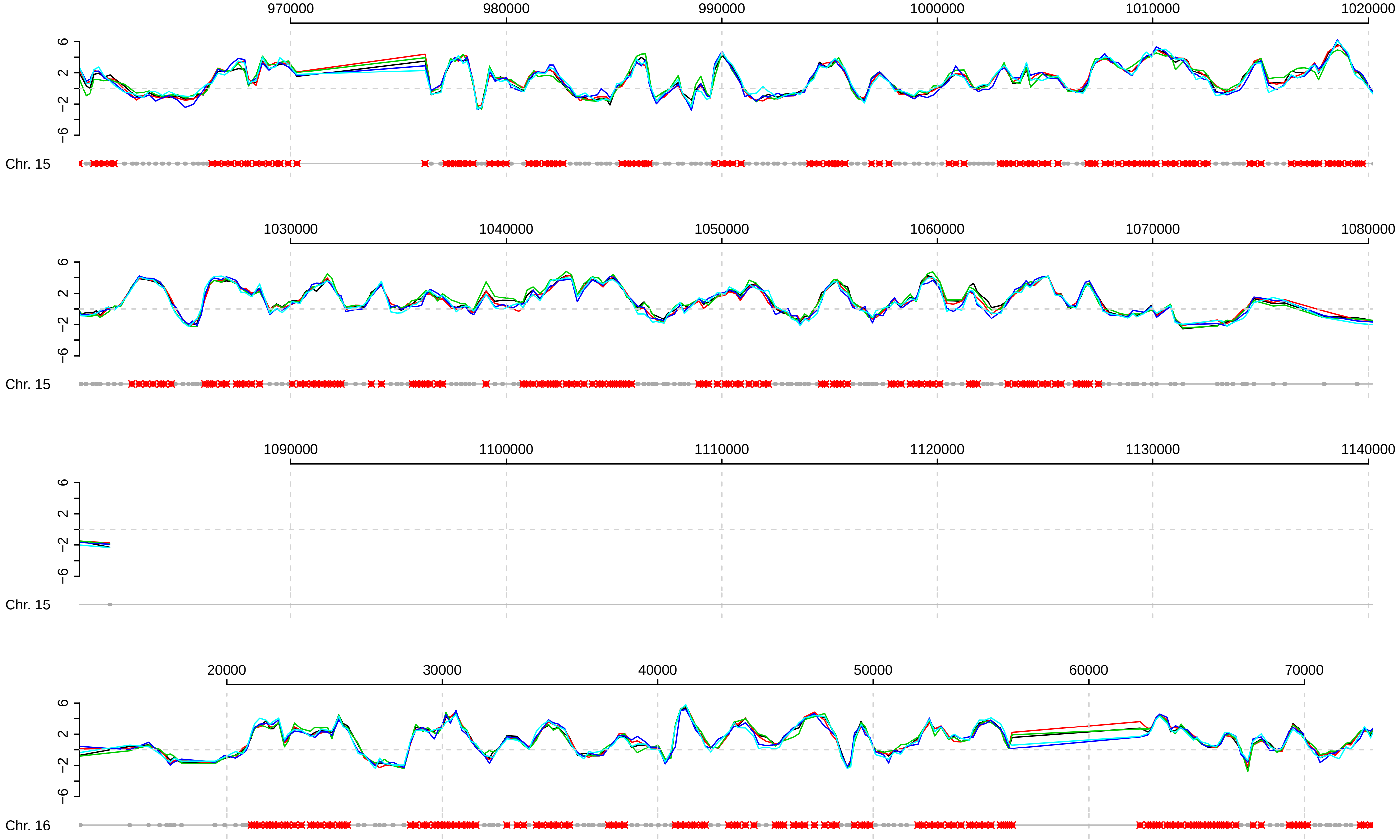

970000
980000
990000
1000000
1010000
1020000
6
2
−2
−6
Chr. 15
1030000
1040000
1050000
1060000
1070000
1080000
6
2
−2
−6
Chr. 15
1090000
1100000
1110000
1120000
1130000
1140000
6
2
−2
−6
Chr. 15
20000
30000
40000
50000
60000
70000
6
2
−2
−6
Chr. 16

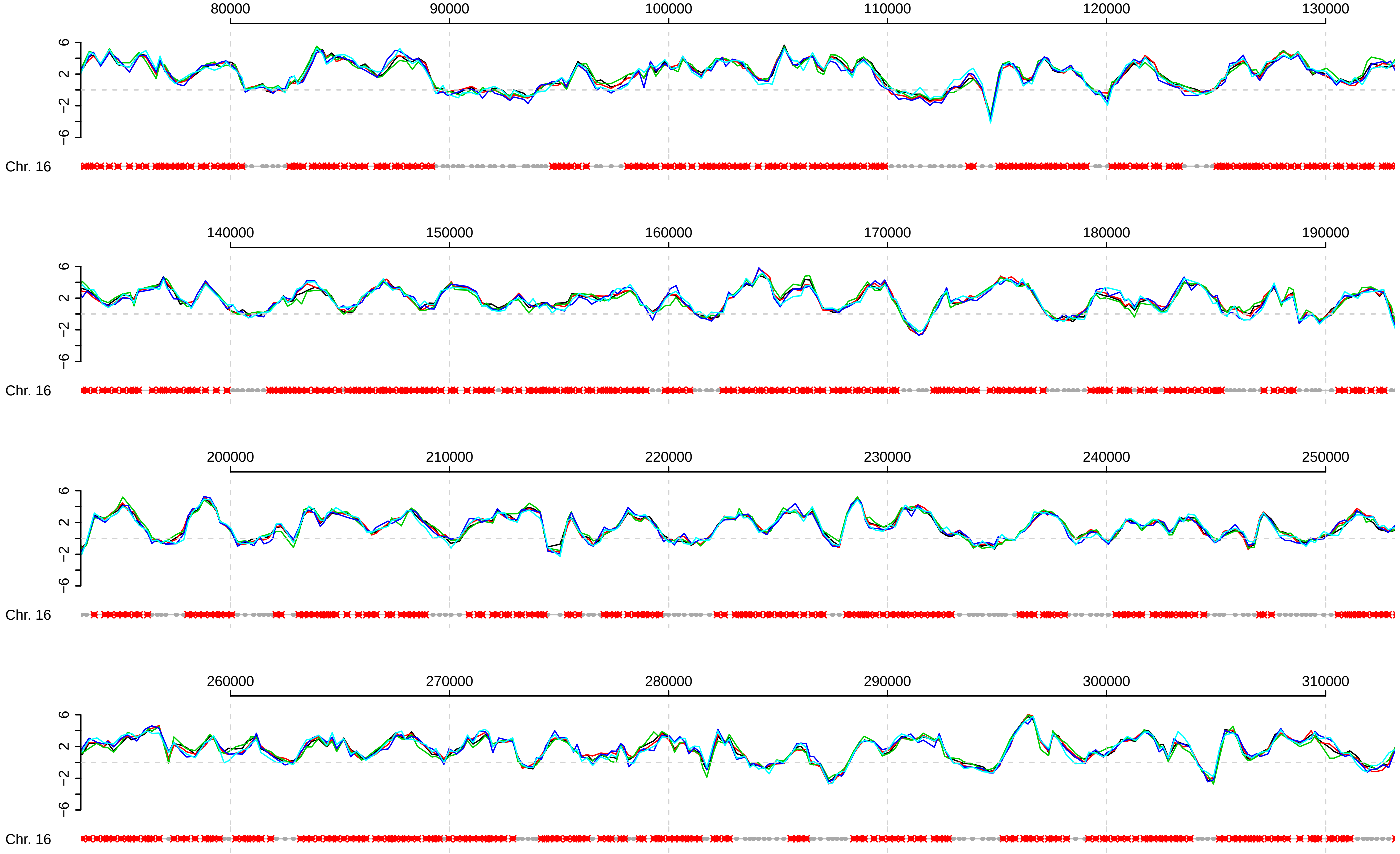
80000
90000
100000
110000
120000
130000
6
2
−2
−6
Chr. 16
140000
150000
160000
170000
180000
190000
6
2
−2
−6
Chr. 16
200000
210000
220000
230000
240000
250000
6
2
−2
−6
Chr. 16
260000
270000
280000
290000
300000
310000
6
2
−2
−6
Chr. 16

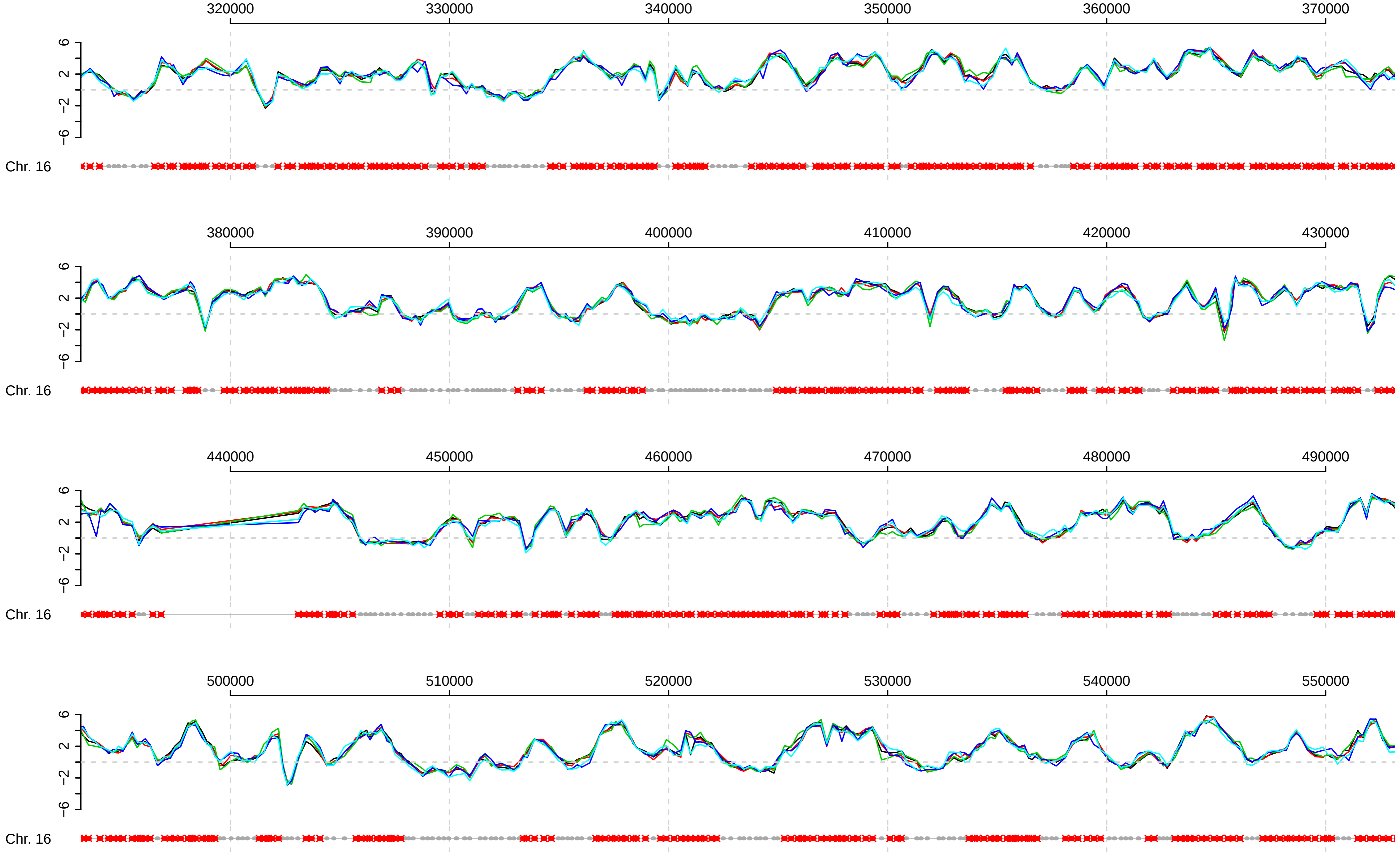

320000
330000
340000
350000
360000
370000
6
2
−2
−6
Chr. 16
380000
390000
400000
410000
420000
430000
6
2
−2
−6
Chr. 16
440000
450000
460000
470000
480000
490000
6
2
−2
−6
Chr. 16
500000
510000
520000
530000
540000
550000
6
2
−2
−6
Chr. 16

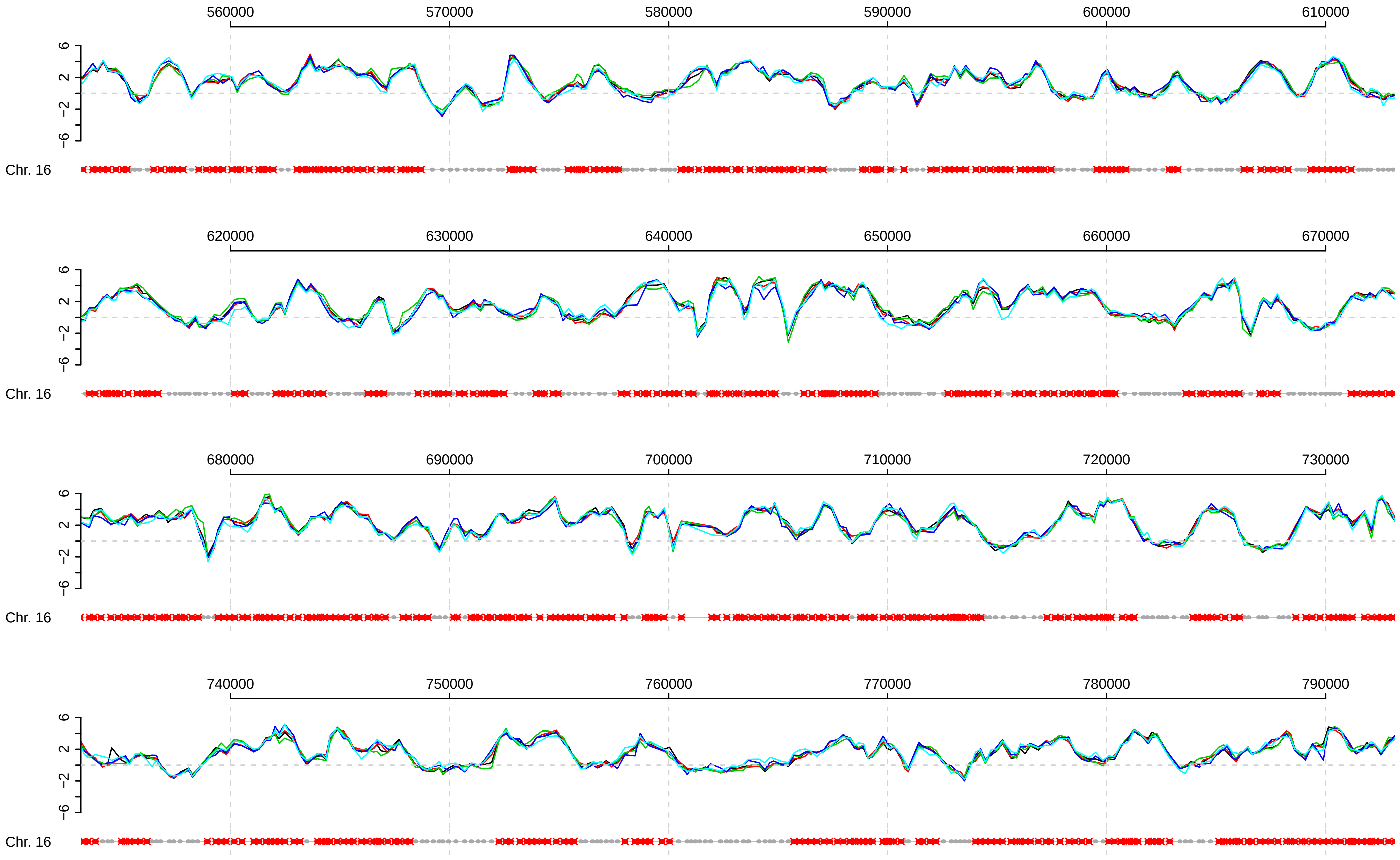

560000
570000
580000
590000
600000
610000
6
2
−2
−6
Chr. 16
620000
630000
640000
650000
660000
670000
6
2
−2
−6
Chr. 16
680000
690000
700000
710000
720000
730000
6
2
−2
−6
Chr. 16
740000
750000
760000
770000
780000
790000
6
2
−2
−6
Chr. 16

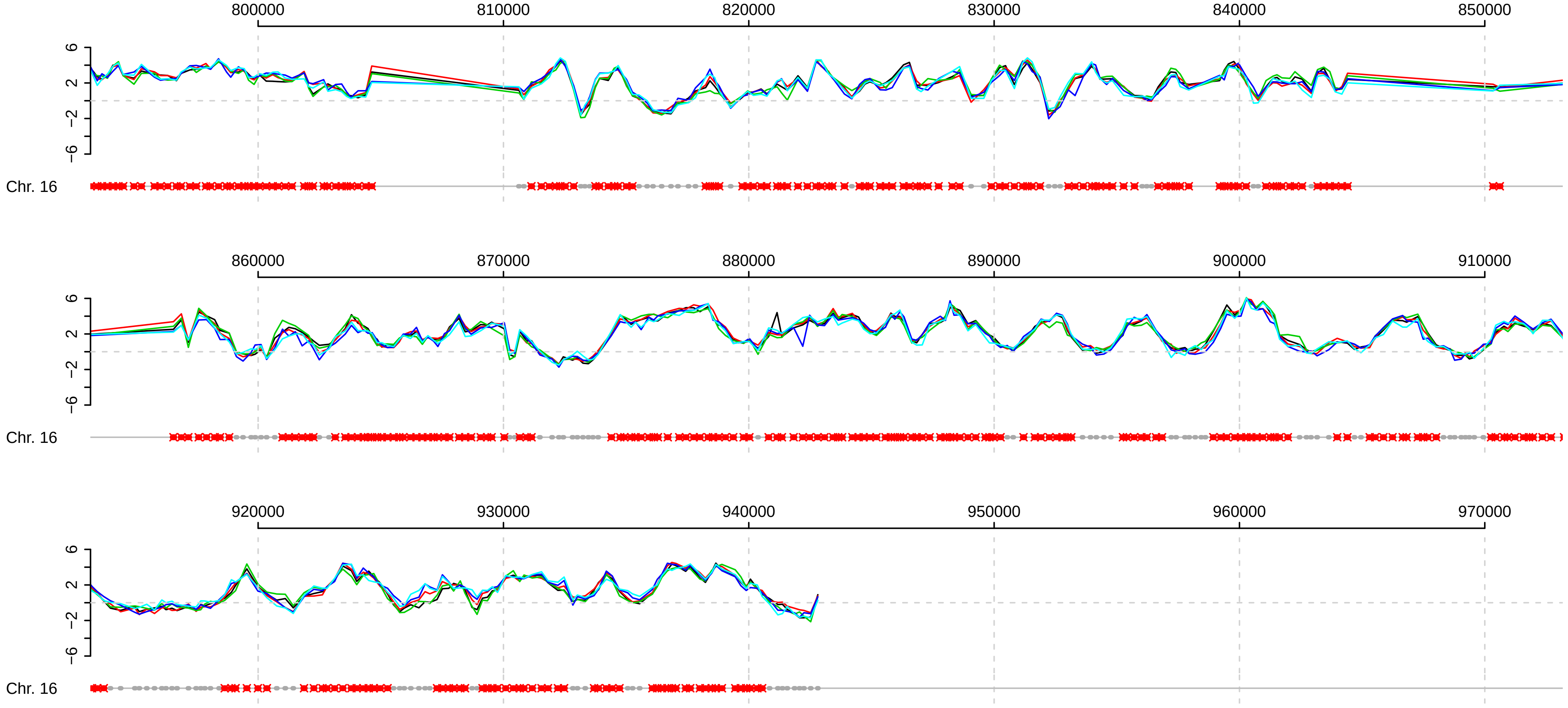
800000
810000
820000
830000
840000
850000
6
2
−2
−6
Chr. 16
860000
870000
880000
890000
900000
910000
6
2
−2
−6
Chr. 16
920000
930000
940000
950000
960000
970000
6
2
−2
−6
Chr. 16

The following plots show H3Ac enrichment over the whole genome. Black line shows average of untreated datasets. Red line shows average of treated datasets.

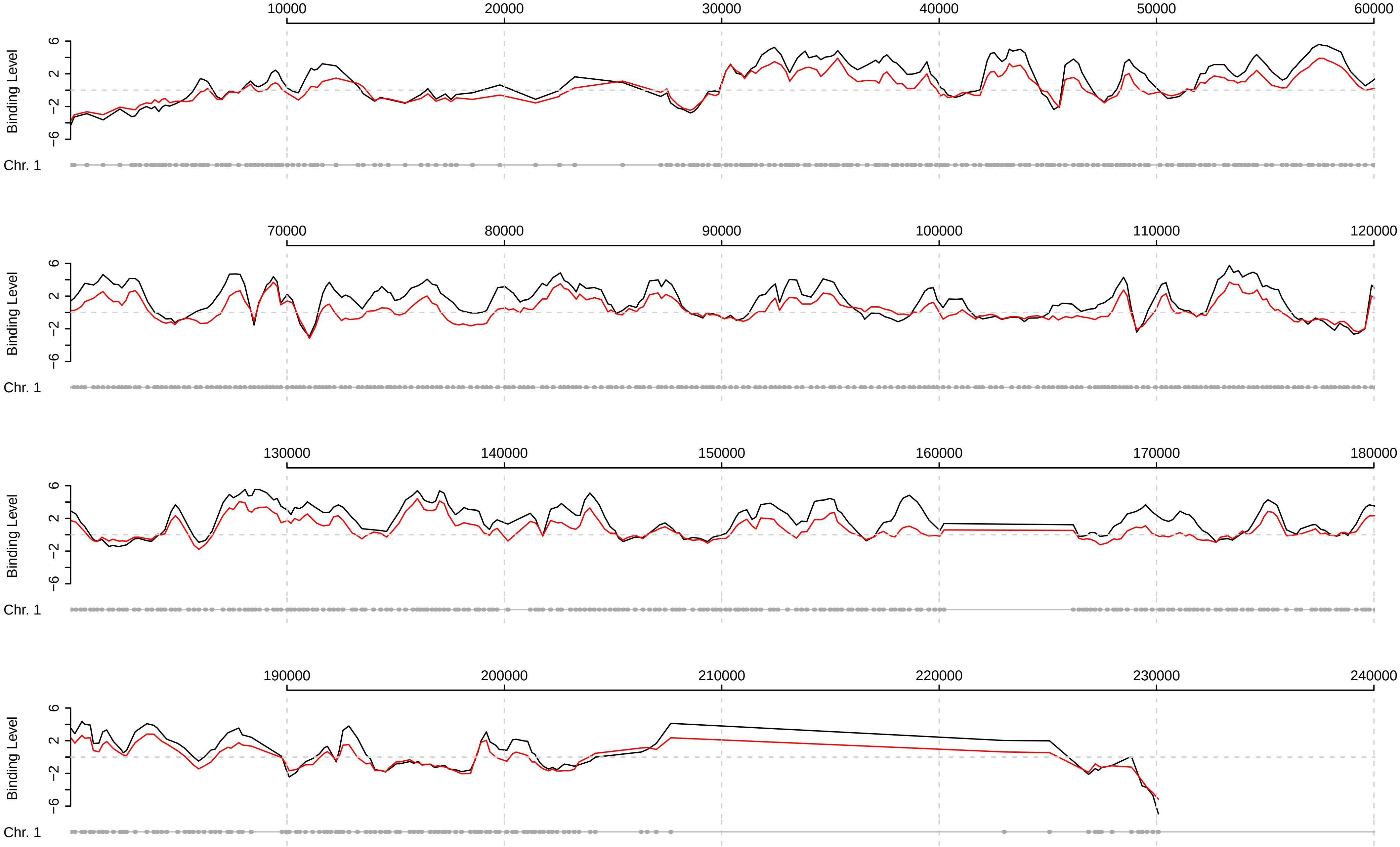

10000
20000
30000
40000
50000
60000
70000
80000
90000
100000
110000
120000
130000
140000
150000
160000
170000
180000
190000
200000
210000
220000
230000
240000
Binding Level
6
2
−2
−6
Chr. 1

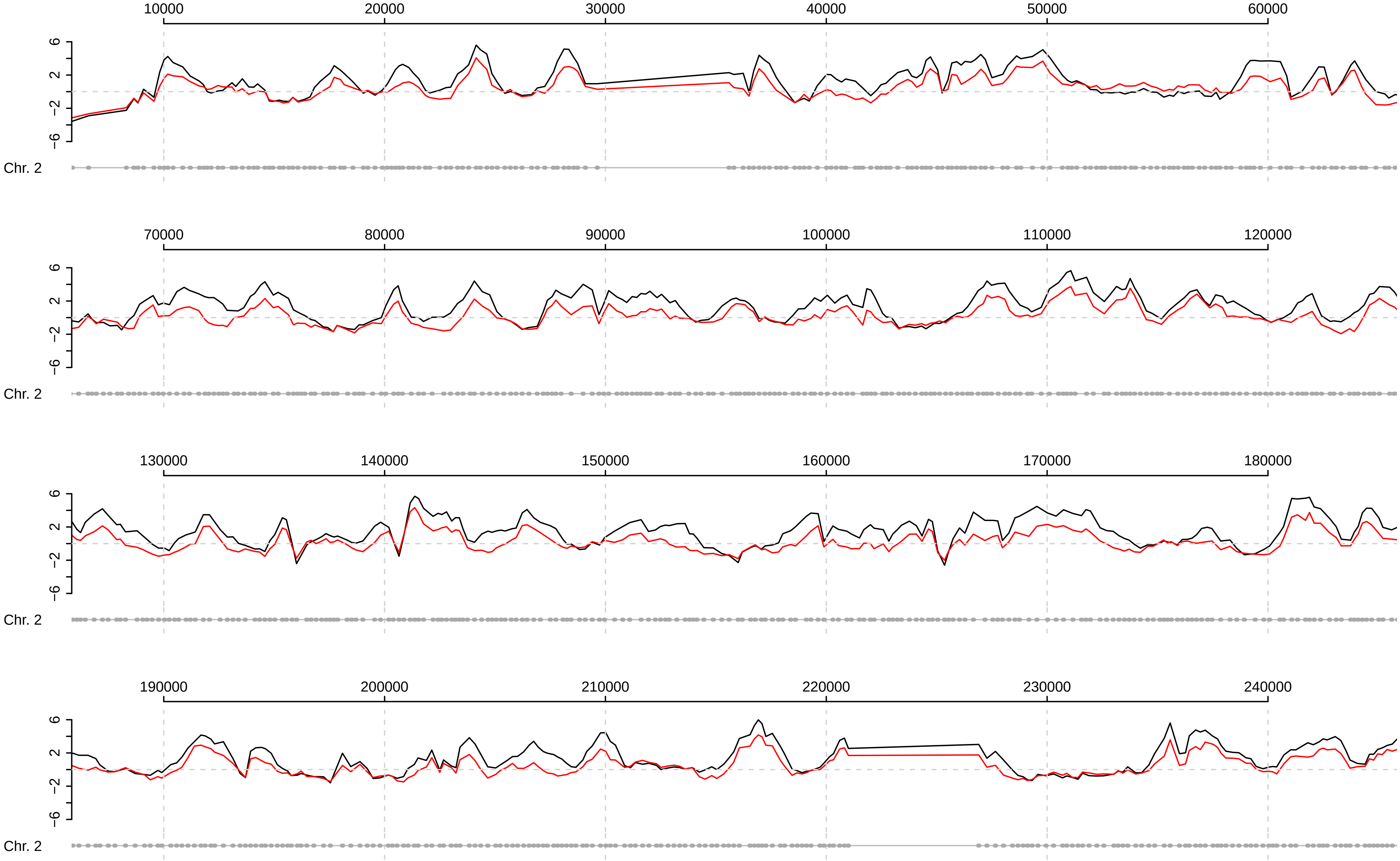

10000
20000
30000
40000
50000
60000
6
2
−2
−6
Chr. 2
70000
80000
90000
100000
110000
120000
6
2
−2
−6
Chr. 2
130000
140000
150000
160000
170000
180000
6
2
−2
−6
Chr. 2
190000
200000
210000
220000
230000
240000
6
2
−2
−6
Chr. 2

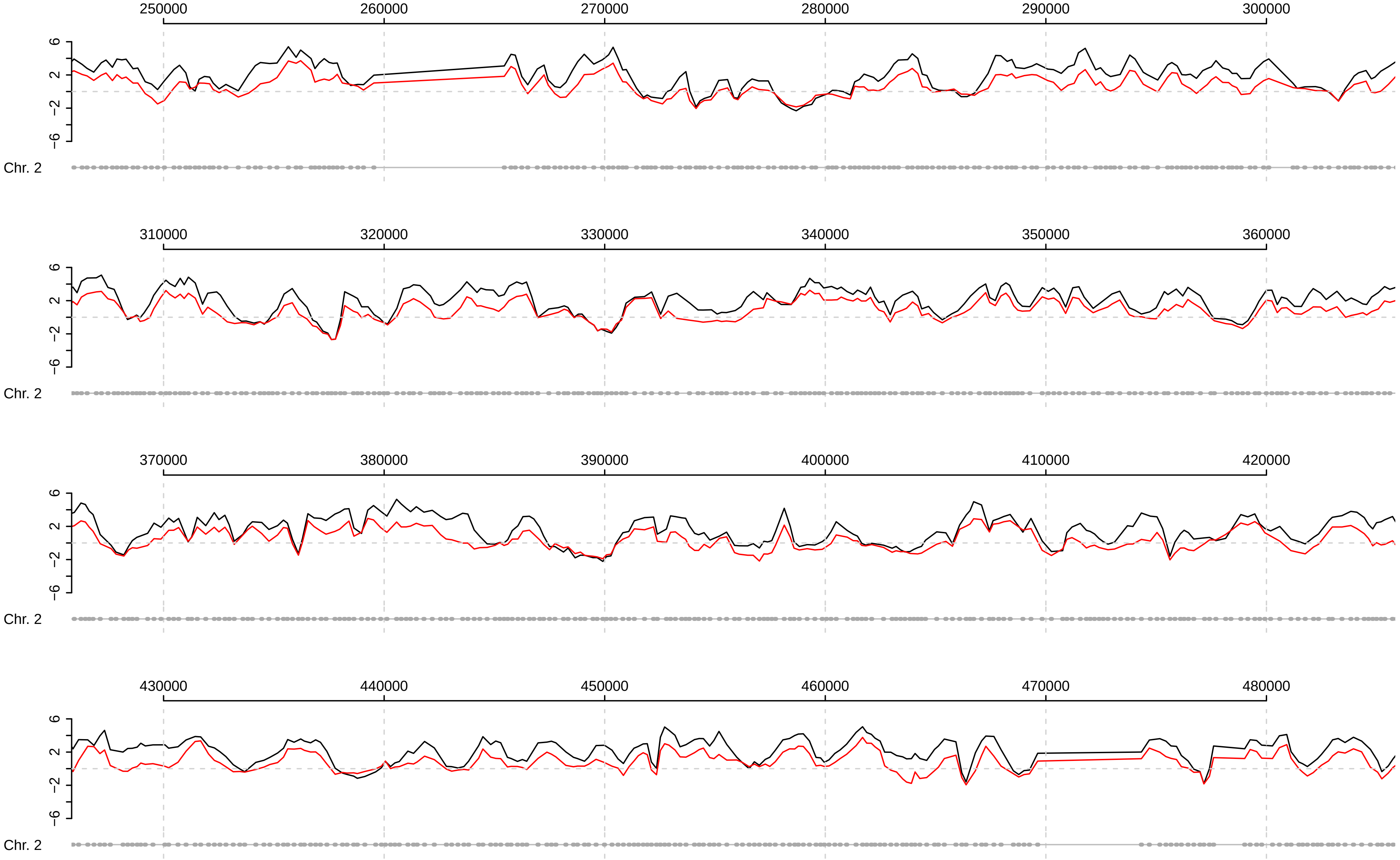
250000
260000
270000
280000
290000
300000
6
2
−2
−6
Chr. 2
310000
320000
330000
340000
350000
360000
6
2
−2
−6
Chr. 2
370000
380000
390000
400000
410000
420000
6
2
−2
−6
Chr. 2
430000
440000
450000
460000
470000
480000
6
2
−2
−6
Chr. 2

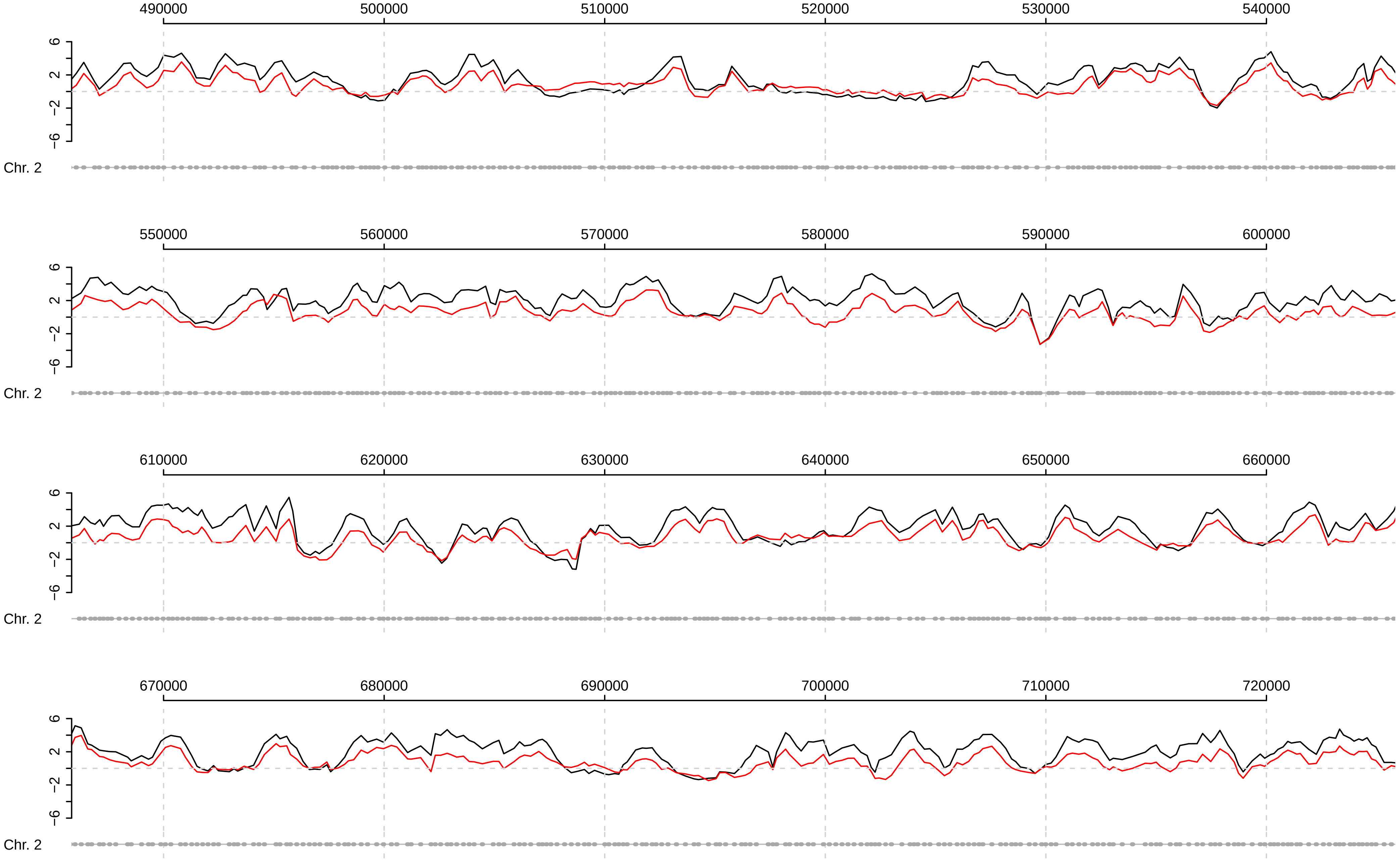

490000
500000
510000
520000
530000
540000
6
2
−2
−6
Chr. 2
550000
560000
570000
580000
590000
600000
6
2
−2
−6
Chr. 2
610000
620000
630000
640000
650000
660000
6
2
−2
−6
Chr. 2
670000
680000
690000
700000
710000
720000
6
2
−2
−6
Chr. 2

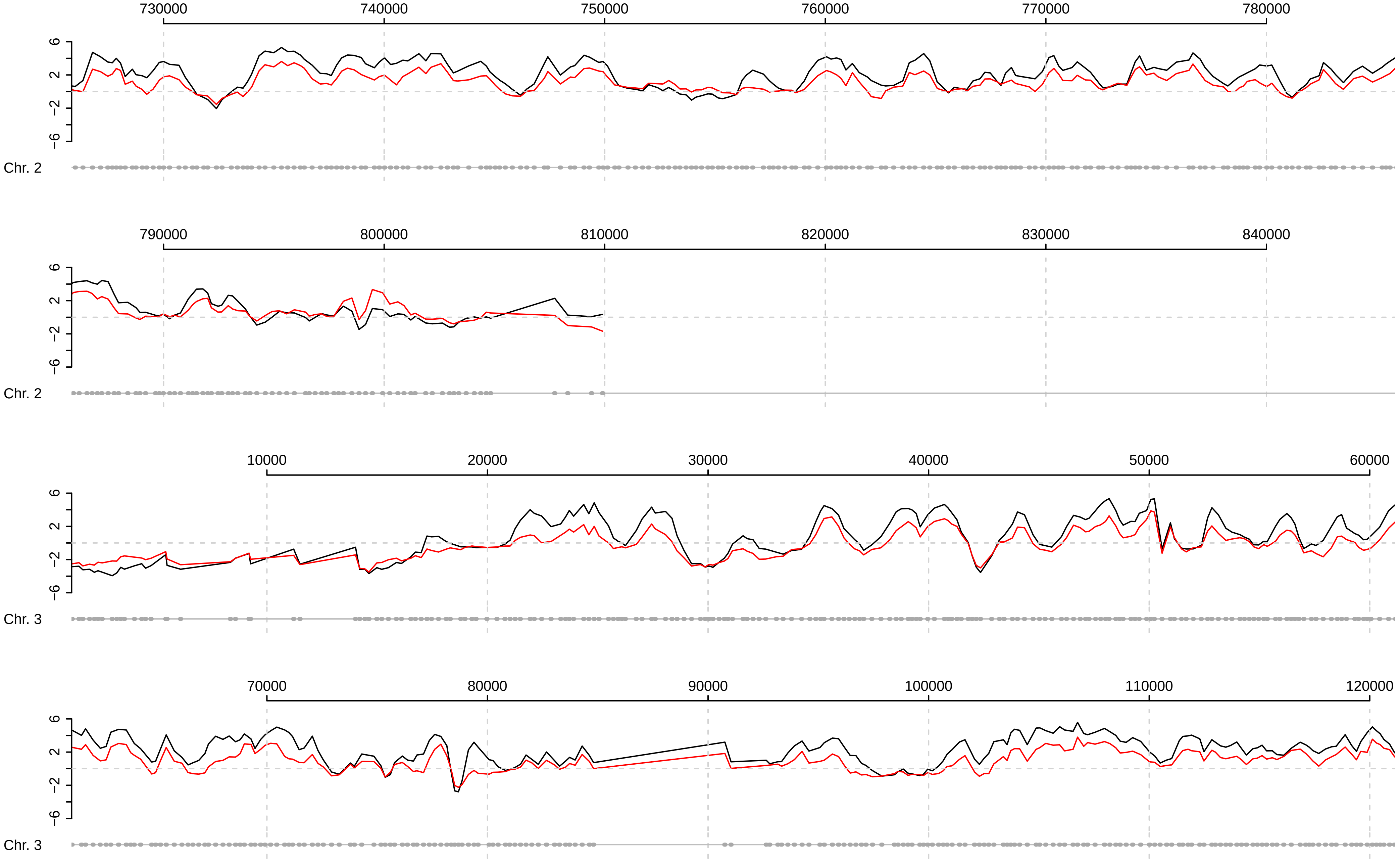

730000
740000
750000
760000
770000
780000
Chr. 2
790000
800000
810000
820000
830000
840000
Chr. 2
10000
20000
30000
40000
50000
60000
Chr. 3
70000
80000
90000
100000
110000
120000
Chr. 3
6
2
−2
−6

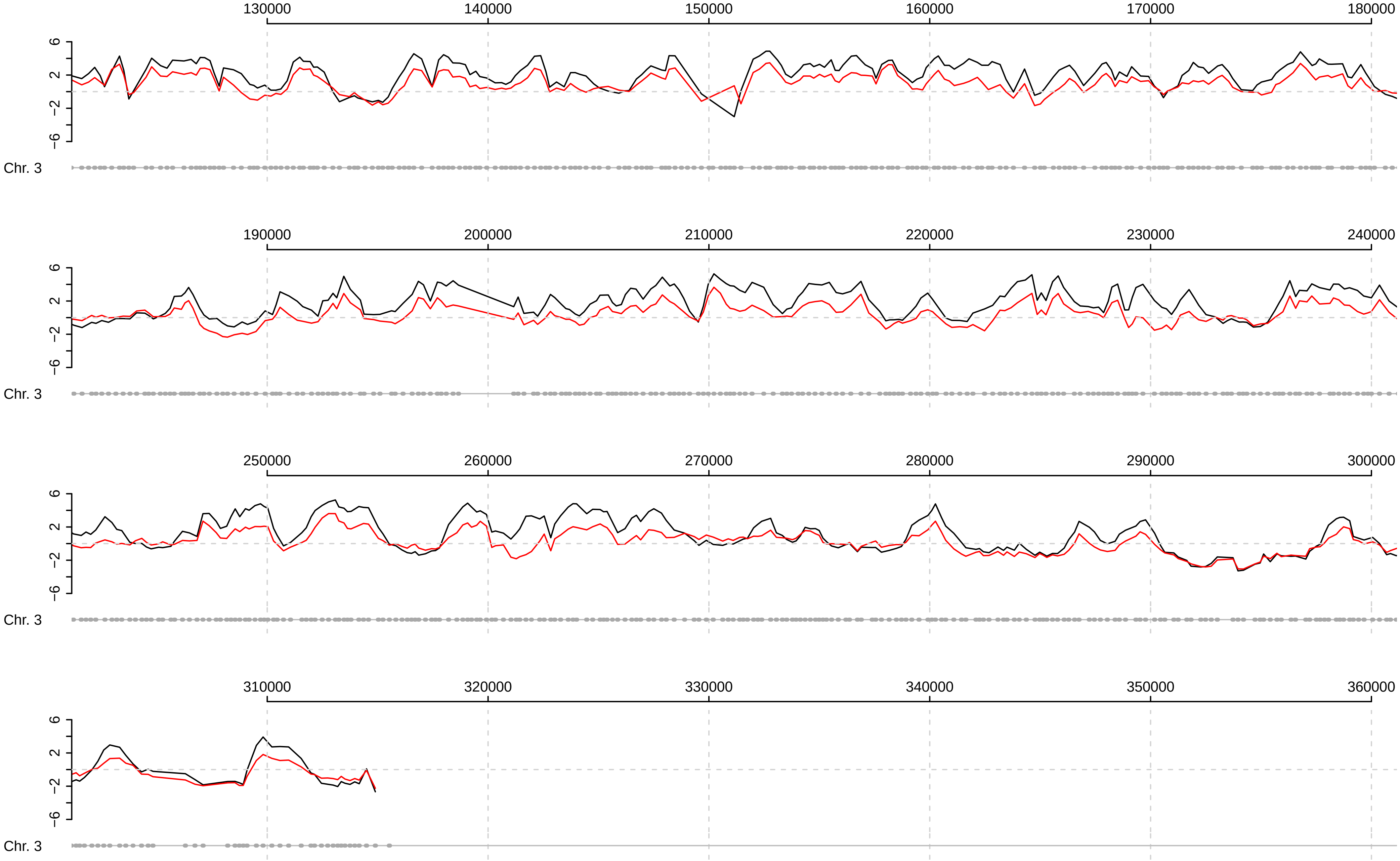

130000
140000
150000
160000
170000
180000
6
2
−2
−6
Chr. 3
190000
200000
210000
220000
230000
240000
6
2
−2
−6
Chr. 3
250000
260000
270000
280000
290000
300000
6
2
−2
−6
Chr. 3
310000
320000
330000
340000
350000
360000
6
2
−2
−6
Chr. 3

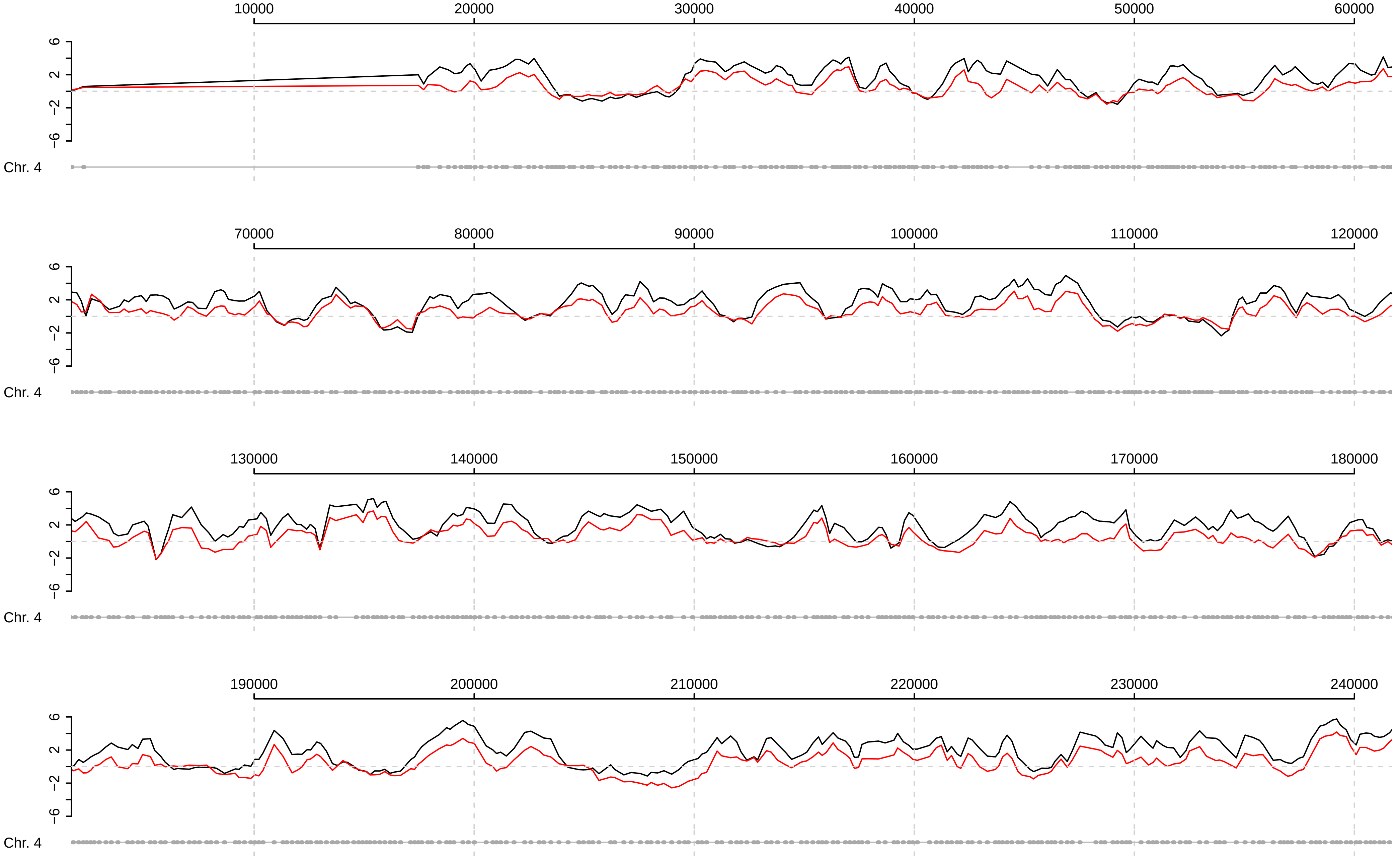
10000
20000
30000
40000
50000
60000
Chr. 4
70000
80000
90000
100000
110000
120000
Chr. 4
130000
140000
150000
160000
170000
180000
Chr. 4
190000
200000
210000
220000
230000
240000
Chr. 4
6
2
−2
−6

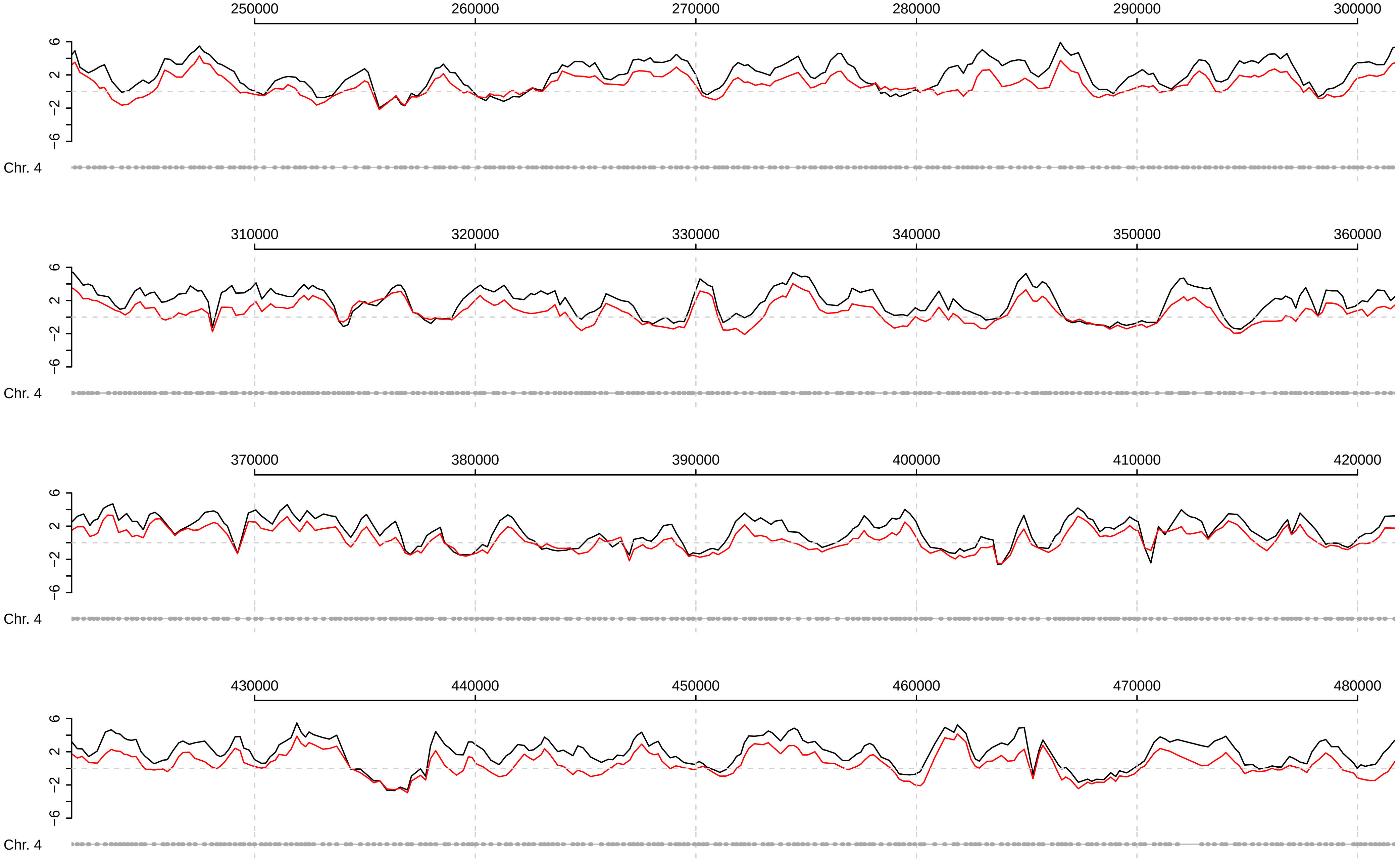

250000
260000
270000
280000
290000
300000
6
2
−2
−6
Chr. 4
310000
320000
330000
340000
350000
360000
6
2
−2
−6
Chr. 4
370000
380000
390000
400000
410000
420000
6
2
−2
−6
Chr. 4
430000
440000
450000
460000
470000
480000
6
2
−2
−6
Chr. 4

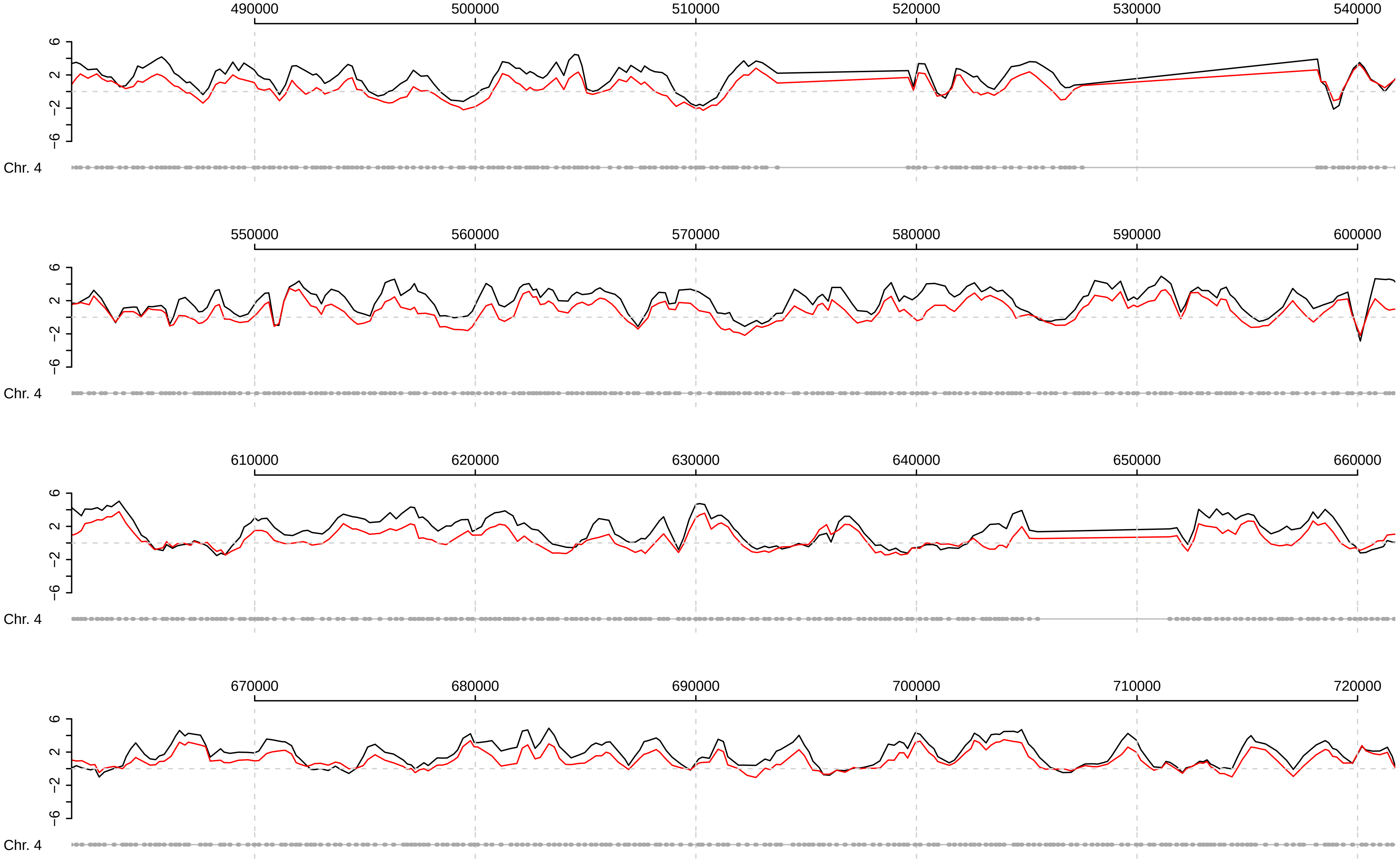

490000
500000
510000
520000
530000
540000
550000
560000
570000
580000
590000
600000
610000
620000
630000
640000
650000
660000
670000
680000
690000
700000
710000
720000
6
2
−2
−6
Chr. 4

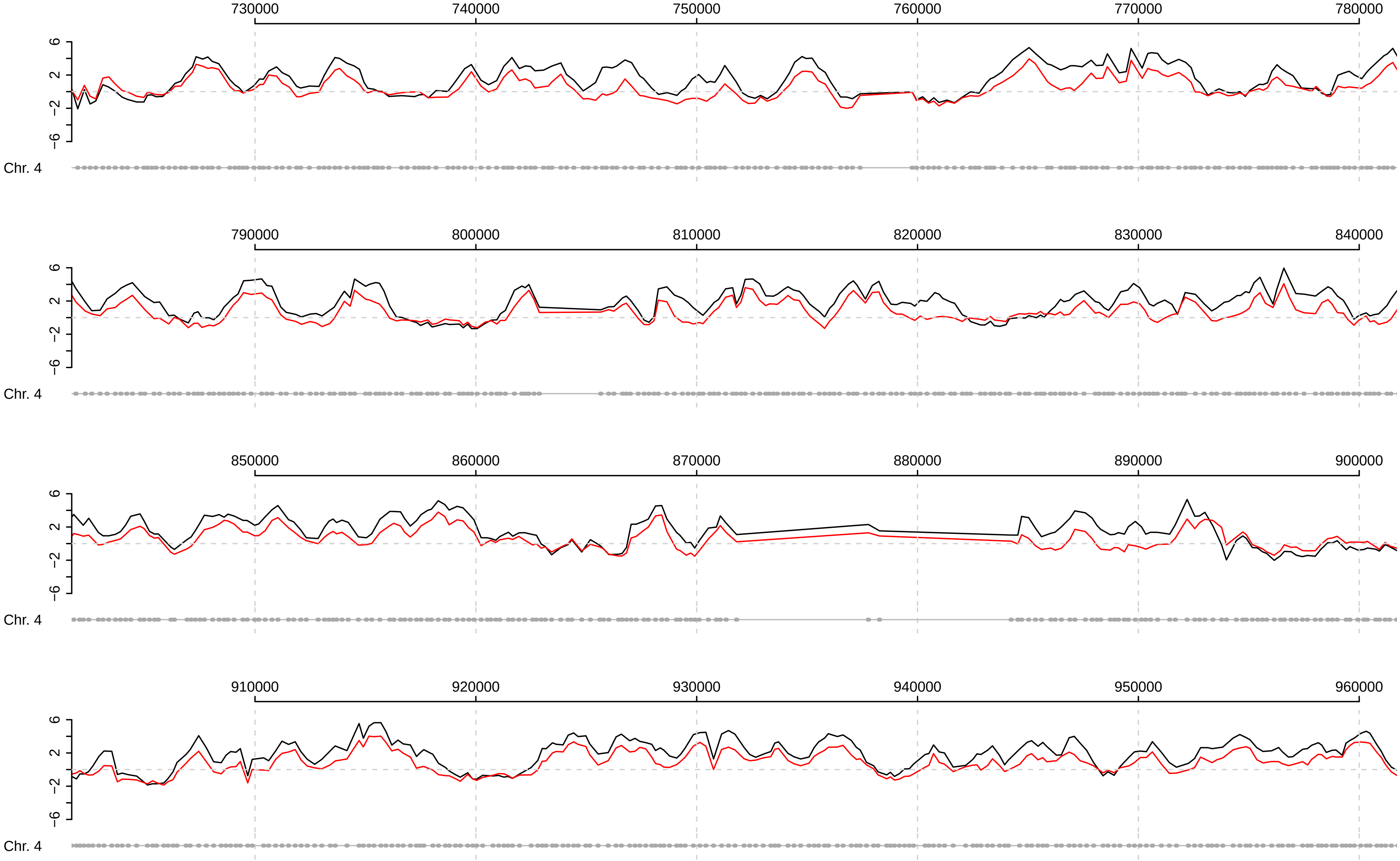
730000
740000
750000
760000
770000
780000
6
2
−2
−6
Chr. 4
790000
800000
810000
820000
830000
840000
6
2
−2
−6
Chr. 4
850000
860000
870000
880000
890000
900000
6
2
−2
−6
Chr. 4
910000
920000
930000
940000
950000
960000
6
2
−2
−6
Chr. 4

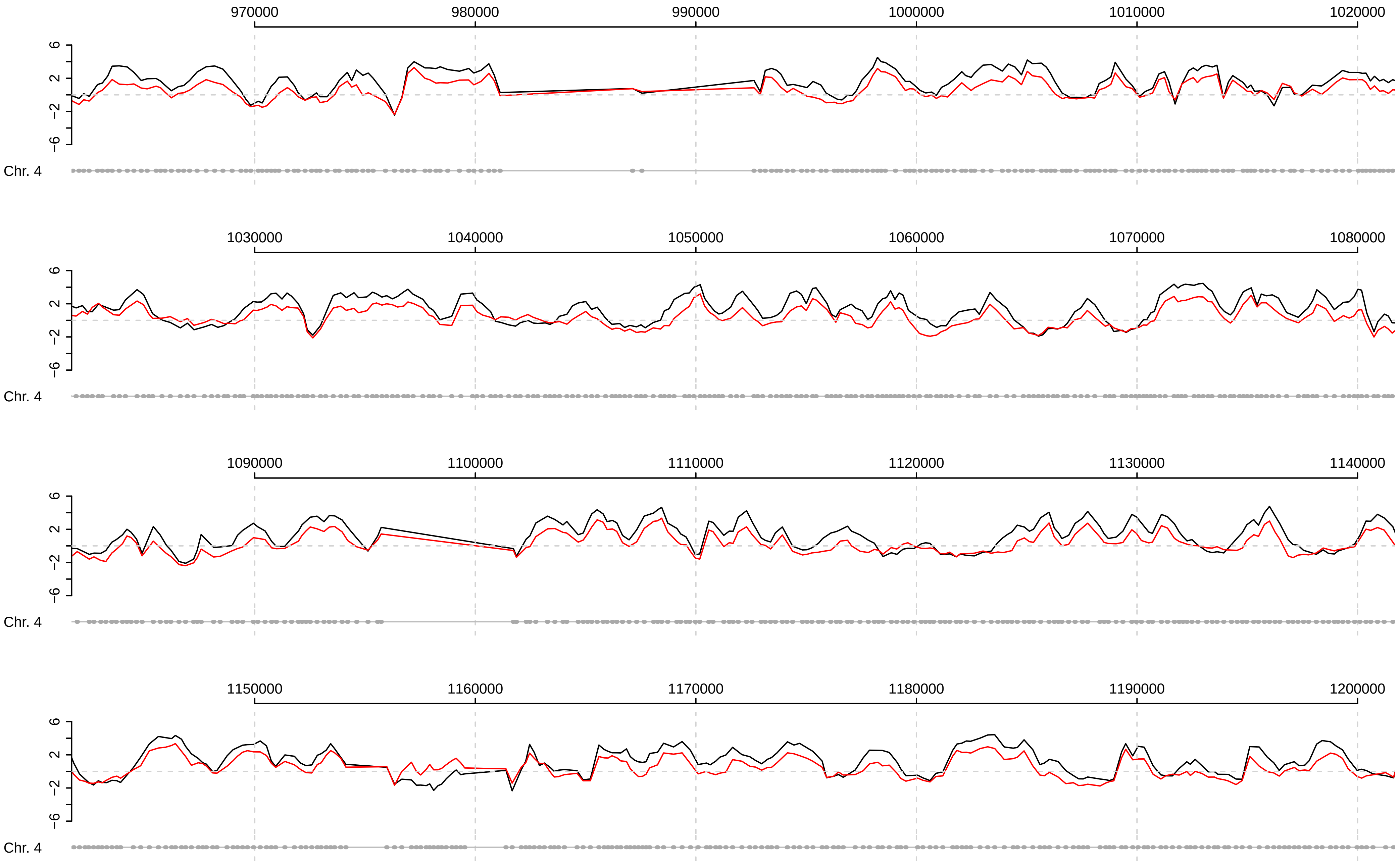
970000
980000
990000
1000000
1010000
1020000
6
2
−2
−6
Chr. 4
1030000
1040000
1050000
1060000
1070000
1080000
6
2
−2
−6
Chr. 4
1090000
1100000
1110000
1120000
1130000
1140000
6
2
−2
−6
Chr. 4
1150000
1160000
1170000
1180000
1190000
1200000
6
2
−2
−6
Chr. 4

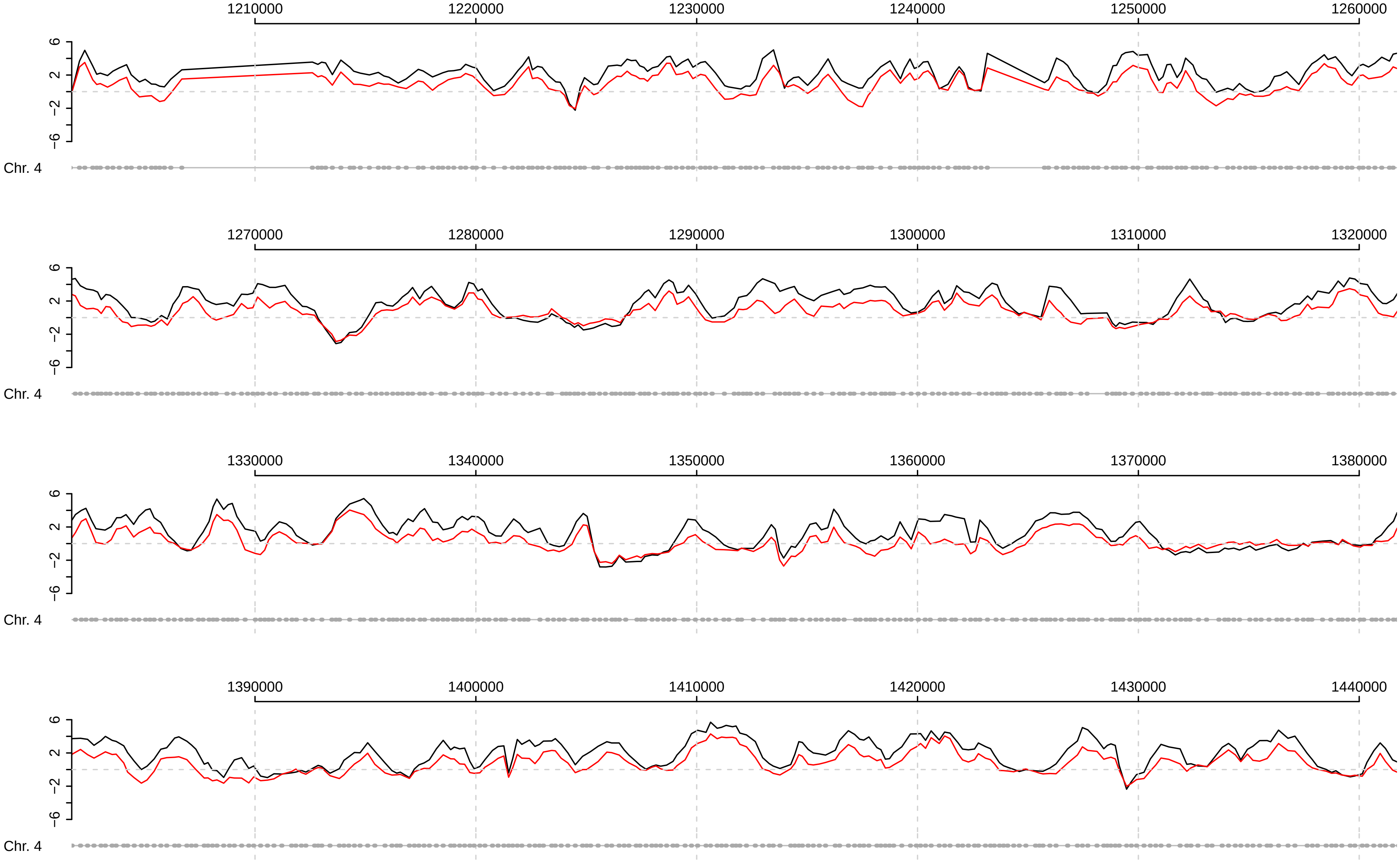

1210000
1220000
1230000
1240000
1250000
1260000
6
2
−2
−6
Chr. 4
1270000
1280000
1290000
1300000
1310000
1320000
6
2
−2
−6
Chr. 4
1330000
1340000
1350000
1360000
1370000
1380000
6
2
−2
−6
Chr. 4
1390000
1400000
1410000
1420000
1430000
1440000
6
2
−2
−6
Chr. 4

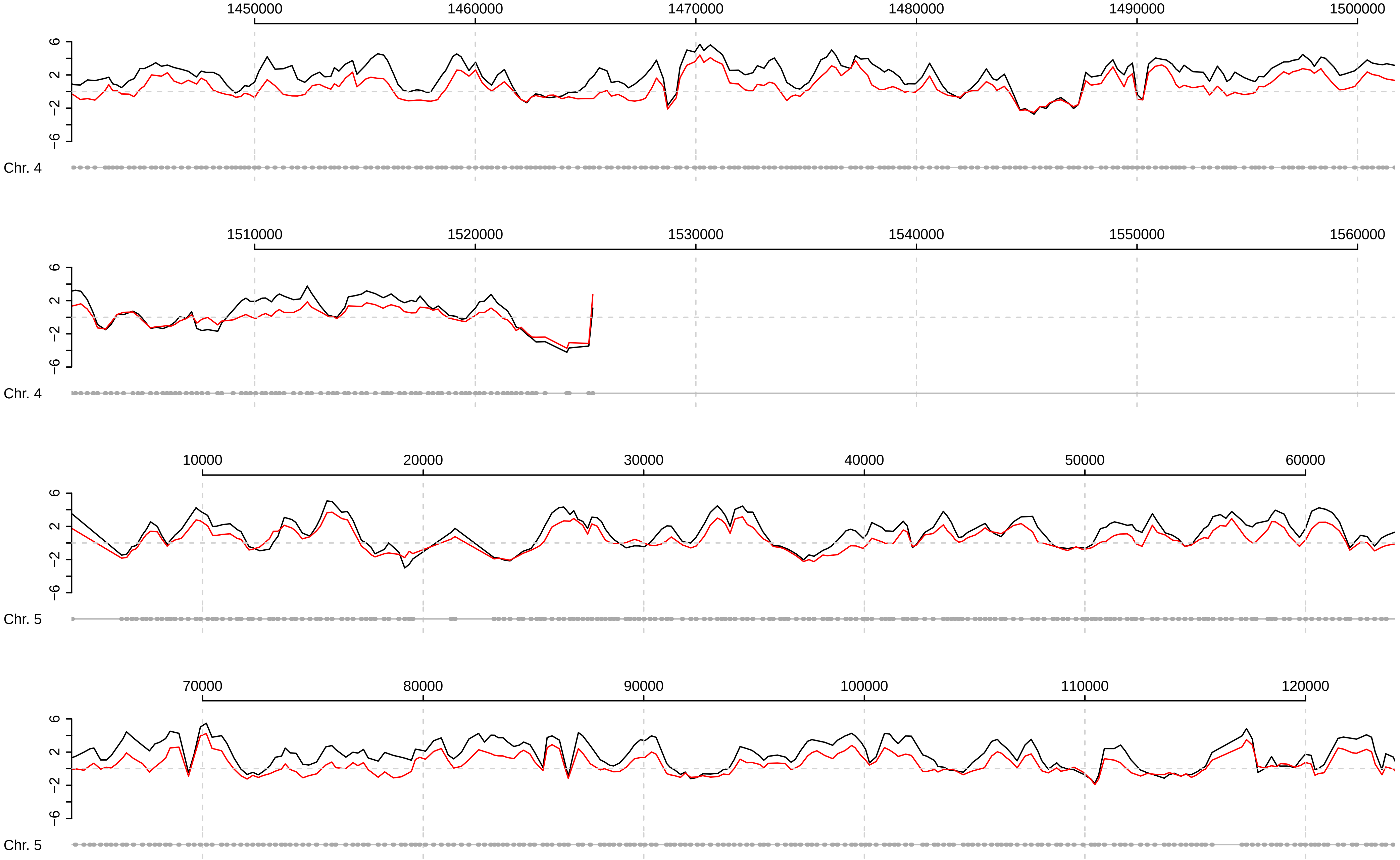
1450000
1460000
1470000
1480000
1490000
1500000
6
2
−2
−6
Chr. 4
1510000
1520000
1530000
1540000
1550000
1560000
6
2
−2
−6
Chr. 4
10000
20000
30000
40000
50000
60000
6
2
−2
−6
Chr. 5
70000
80000
90000
100000
110000
120000
6
2
−2
−6
Chr. 5

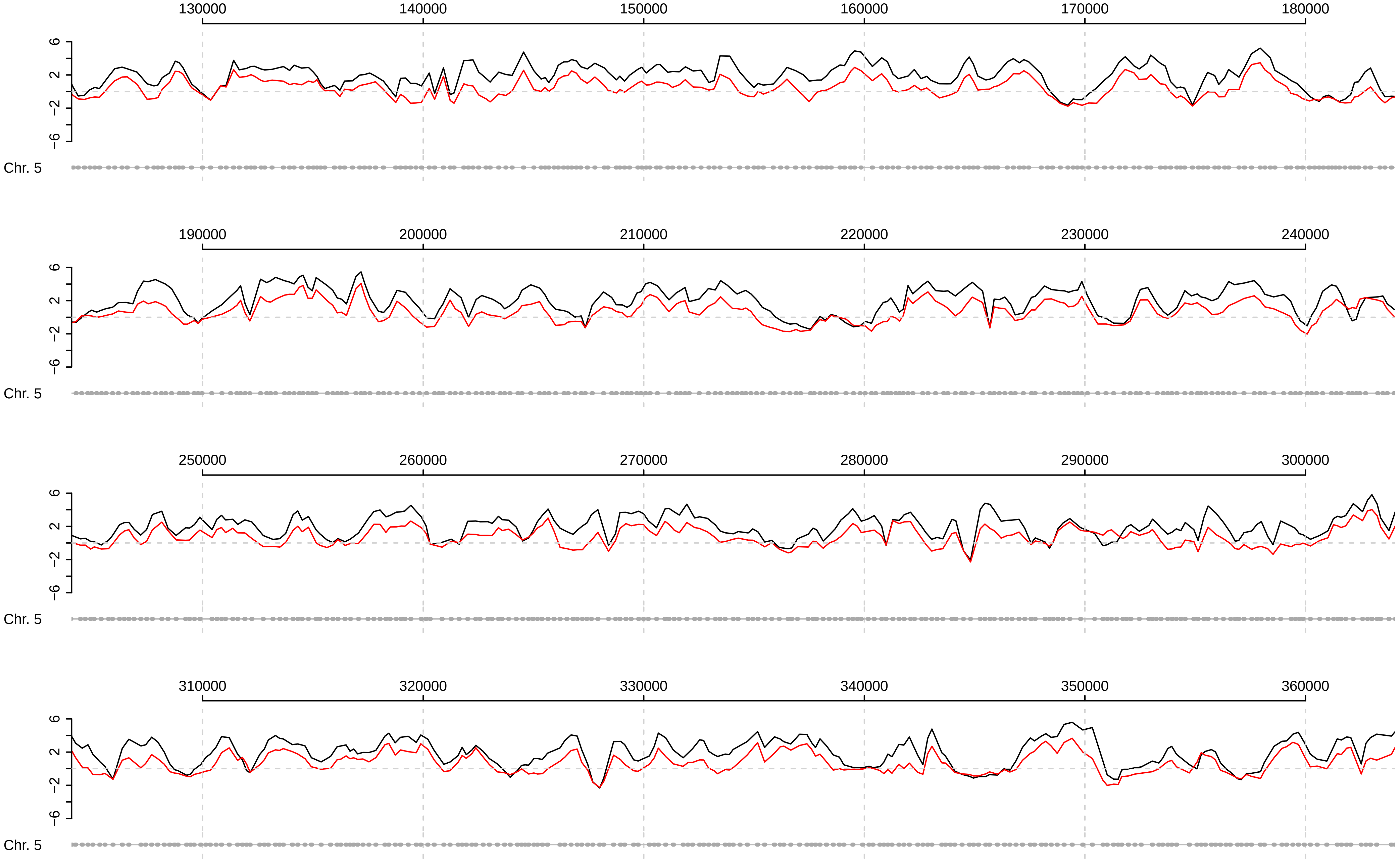
130000
140000
150000
160000
170000
180000
6
2
−2
−6
Chr. 5
190000
200000
210000
220000
230000
240000
6
2
−2
−6
Chr. 5
250000
260000
270000
280000
290000
300000
6
2
−2
−6
Chr. 5
310000
320000
330000
340000
350000
360000
6
2
−2
−6
Chr. 5

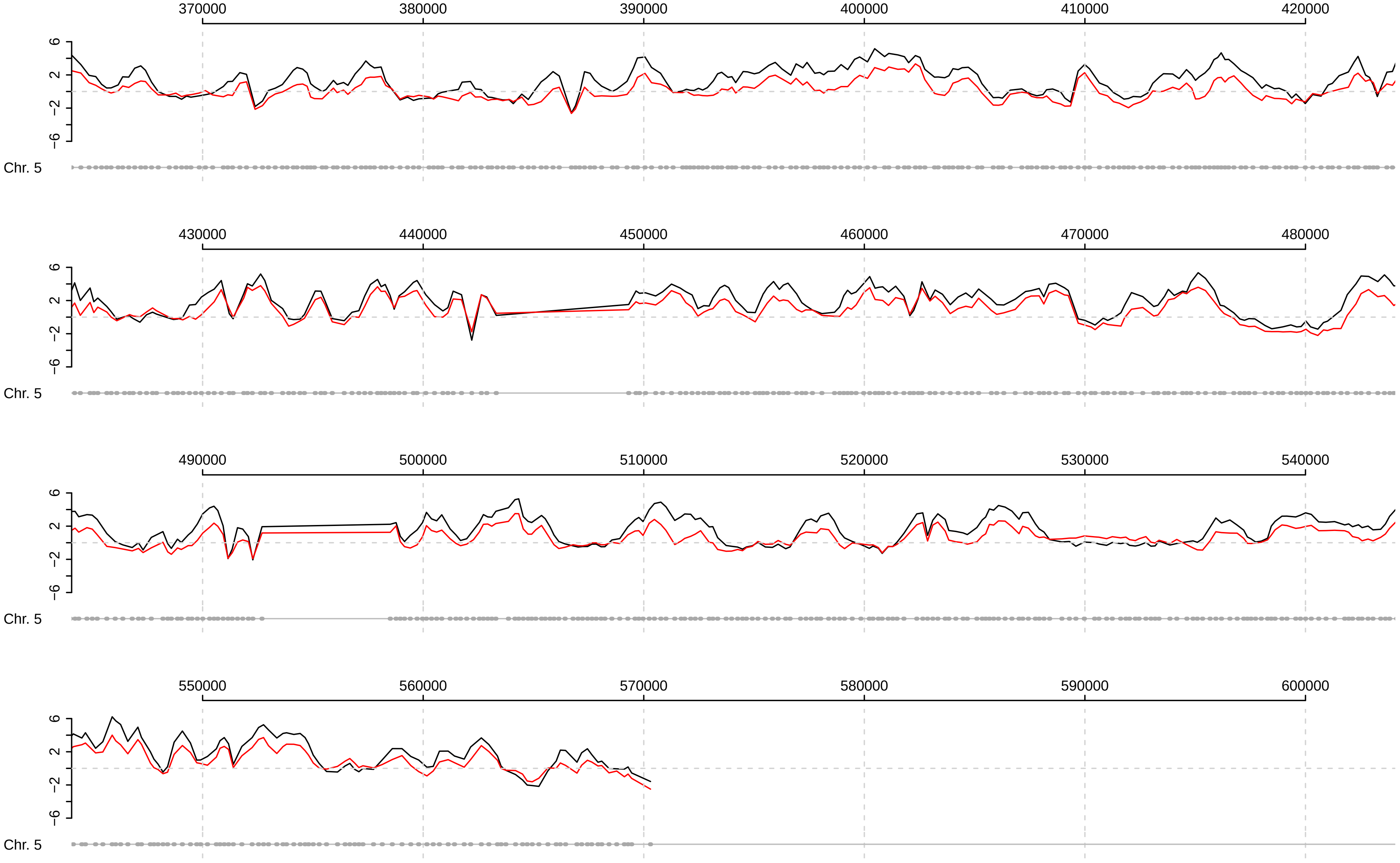
370000
380000
390000
400000
410000
420000
6
2
−2
−6
Chr. 5
430000
440000
450000
460000
470000
480000
6
2
−2
−6
Chr. 5
490000
500000
510000
520000
530000
540000
6
2
−2
−6
Chr. 5
550000
560000
570000
580000
590000
600000
6
2
−2
−6
Chr. 5

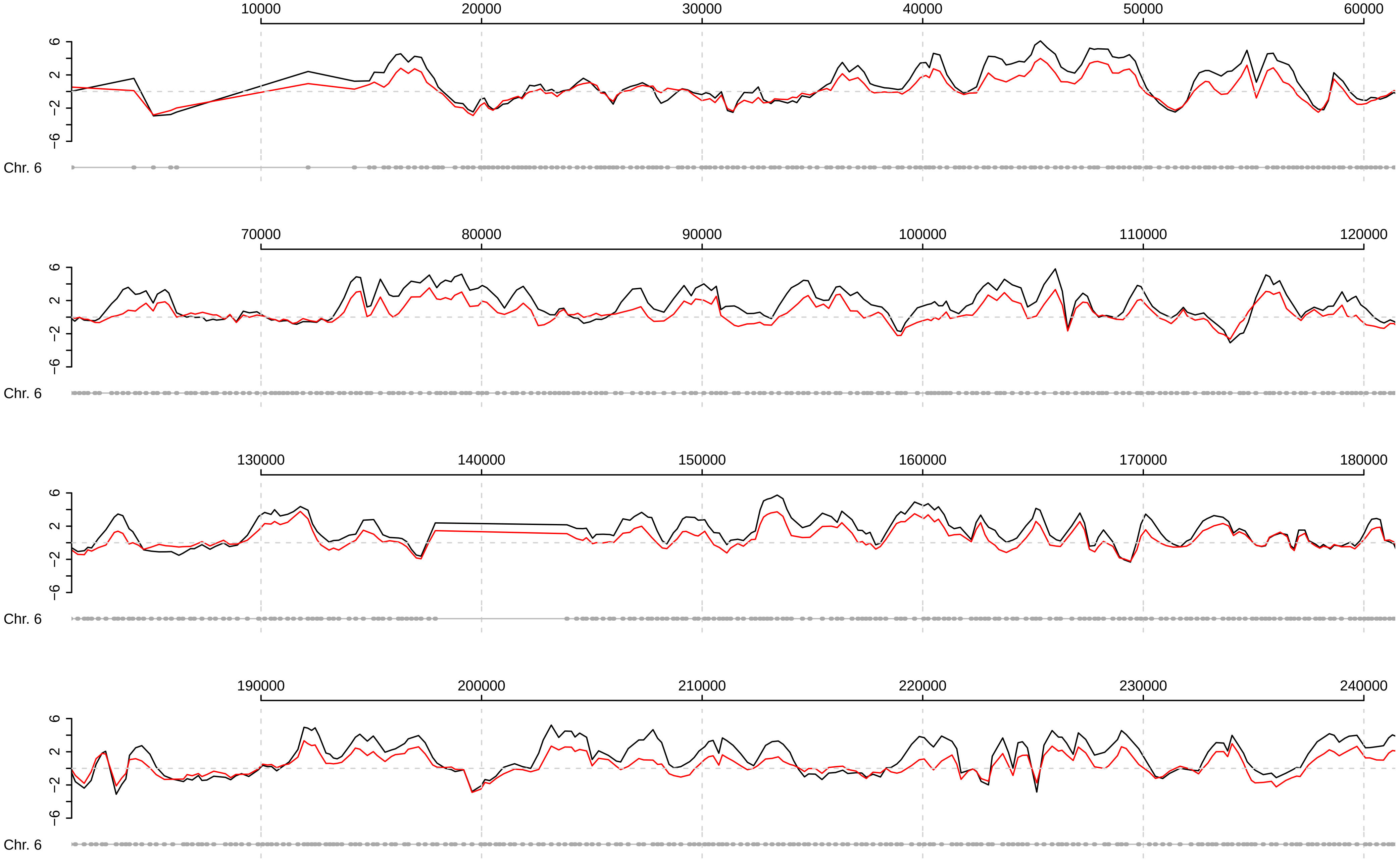

10000
20000
30000
40000
50000
60000
6
2
−2
−6
Chr. 6
70000
80000
90000
100000
110000
120000
6
2
−2
−6
Chr. 6
130000
140000
150000
160000
170000
180000
6
2
−2
−6
Chr. 6
190000
200000
210000
220000
230000
240000
6
2
−2
−6
Chr. 6

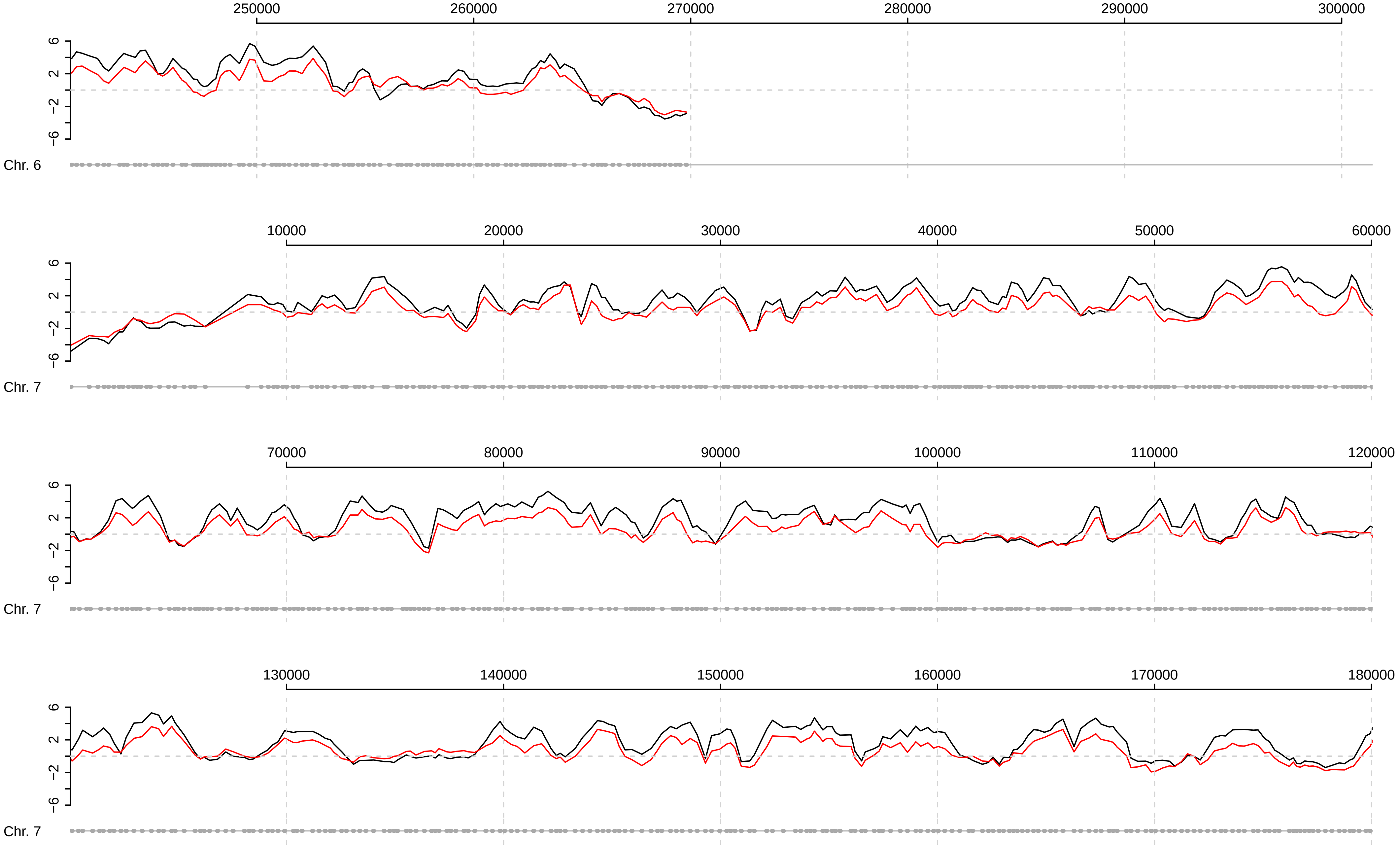

250000
260000
270000
280000
290000
300000
6
2
−2
−6
Chr. 6
10000
20000
30000
40000
50000
60000
Chr. 7
70000
80000
90000
100000
110000
120000
Chr. 7
130000
140000
150000
160000
170000
180000
Chr. 7

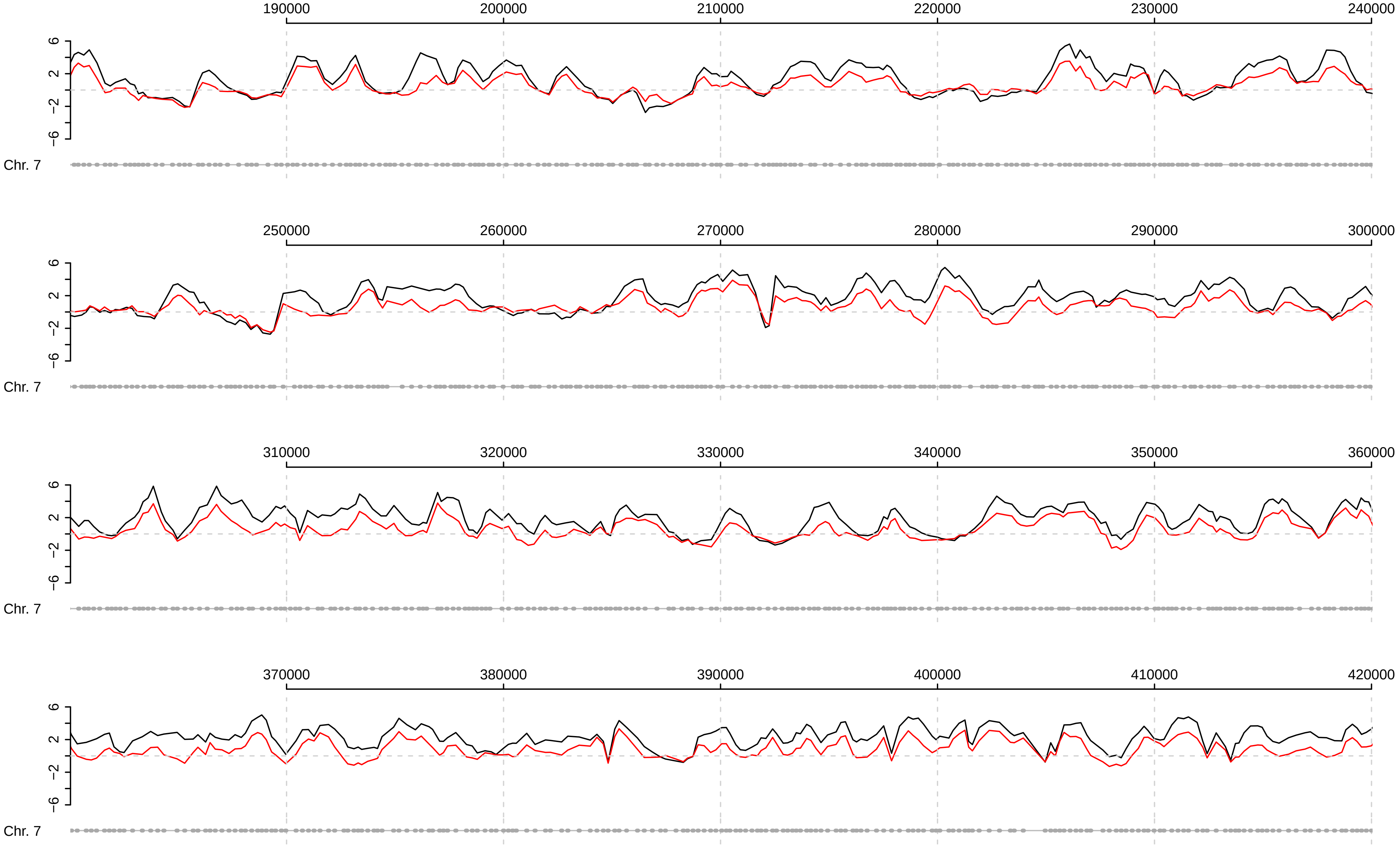

190000
200000
210000
220000
230000
240000
6
2
−2
−6
Chr. 7
250000
260000
270000
280000
290000
300000
6
2
−2
−6
Chr. 7
310000
320000
330000
340000
350000
360000
6
2
−2
−6
Chr. 7
370000
380000
390000
400000
410000
420000
6
2
−2
−6
Chr. 7

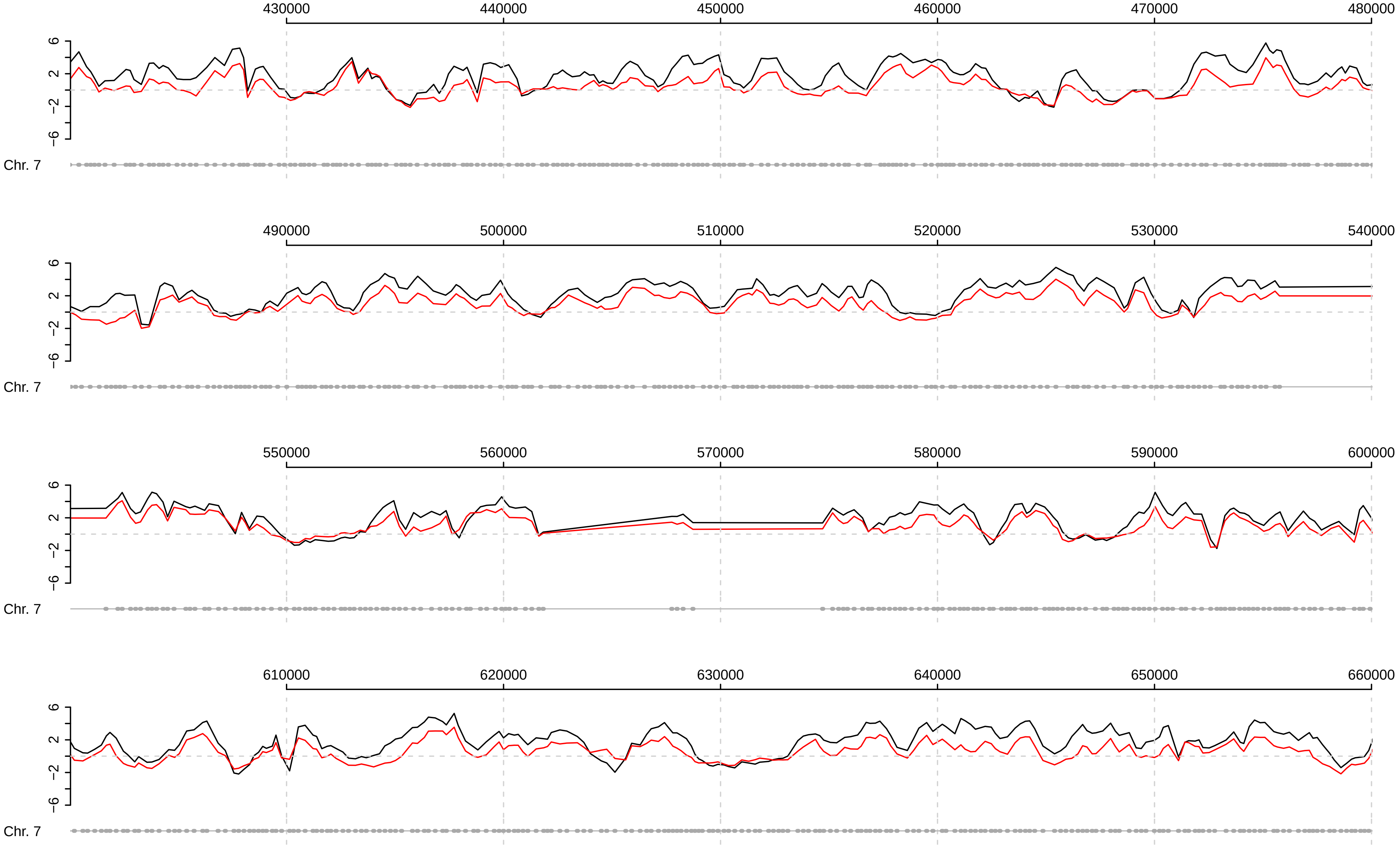

430000
440000
450000
460000
470000
480000
6
2
−2
−6
Chr. 7
490000
500000
510000
520000
530000
540000
6
2
−2
−6
Chr. 7
550000
560000
570000
580000
590000
600000
6
2
−2
−6
Chr. 7
610000
620000
630000
640000
650000
660000
6
2
−2
−6
Chr. 7

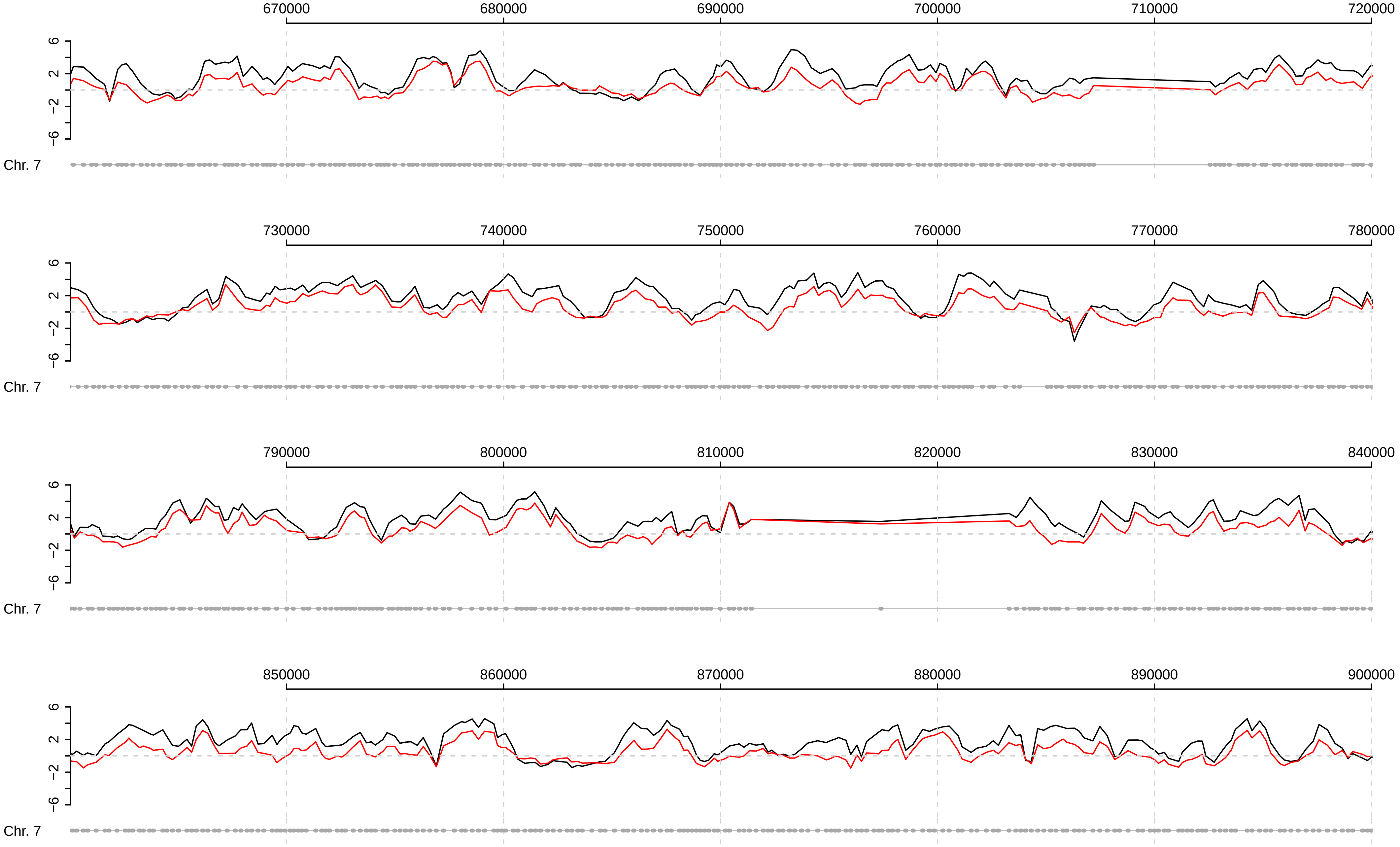
670000
680000
690000
700000
710000
720000
6
2
−2
−6
Chr. 7
730000
740000
750000
760000
770000
780000
6
2
−2
−6
Chr. 7
790000
800000
810000
820000
830000
840000
6
2
−2
−6
Chr. 7
850000
860000
870000
880000
890000
900000
6
2
−2
−6
Chr. 7

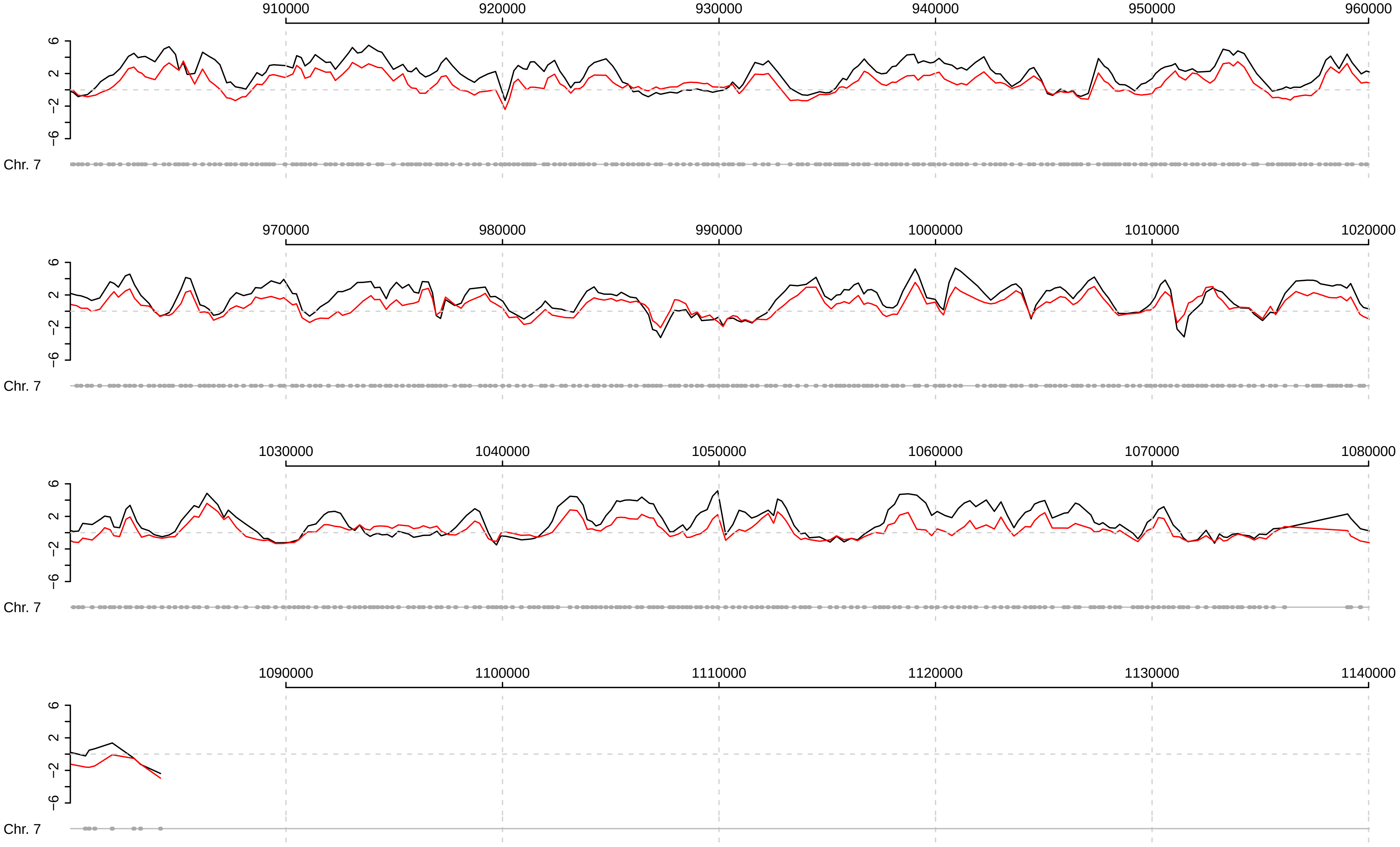

910000
920000
930000
940000
950000
960000
6
2
−2
−6
Chr. 7
970000
980000
990000
1000000
1010000
1020000
Chr. 7
1030000
1040000
1050000
1060000
1070000
1080000
Chr. 7
1090000
1100000
1110000
1120000
1130000
1140000
Chr. 7

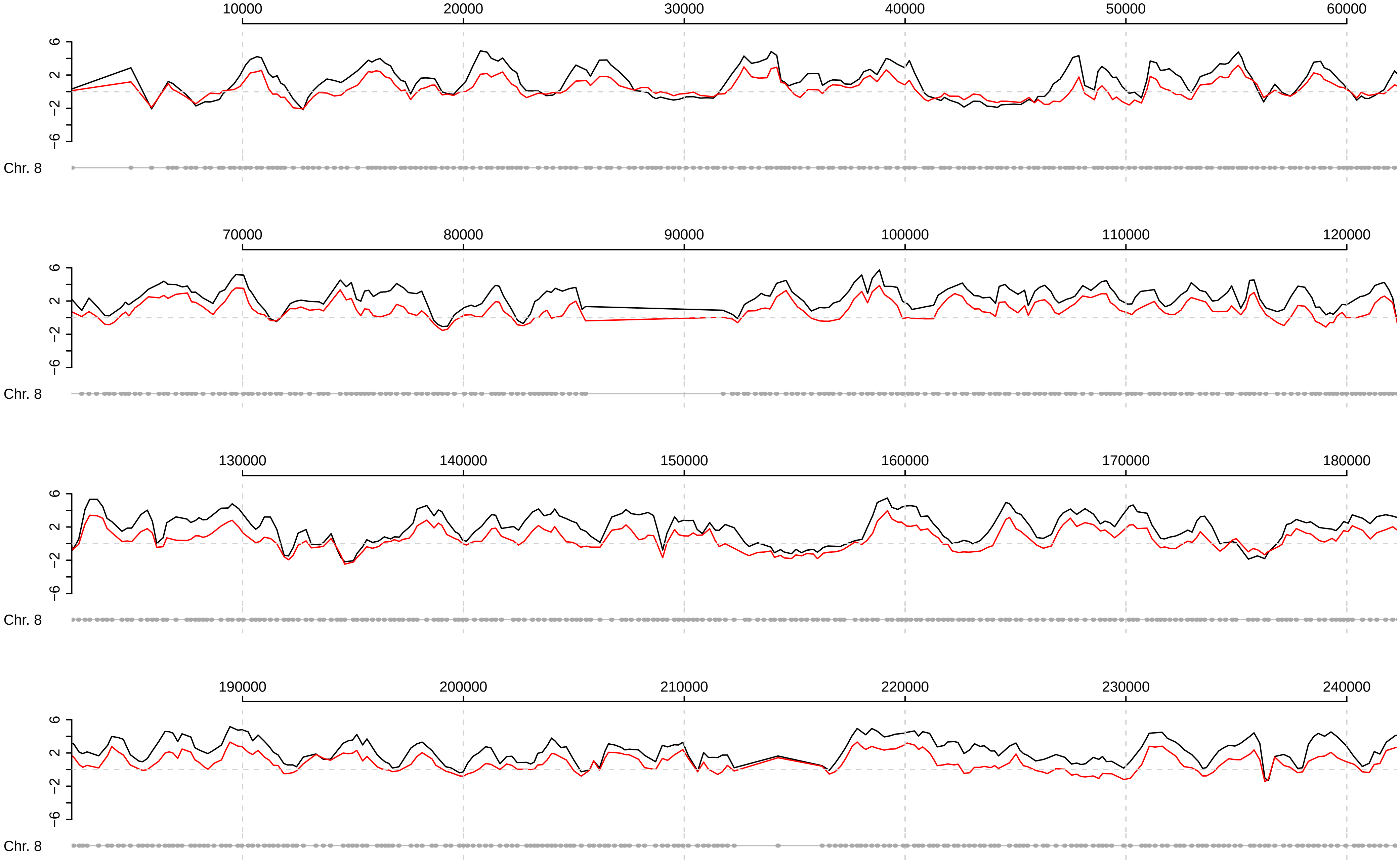

10000
20000
30000
40000
50000
60000
6
2
−2
−6
Chr. 8
70000
80000
90000
100000
110000
120000
6
2
−2
−6
Chr. 8
130000
140000
150000
160000
170000
180000
6
2
−2
−6
Chr. 8
190000
200000
210000
220000
230000
240000
6
2
−2
−6
Chr. 8

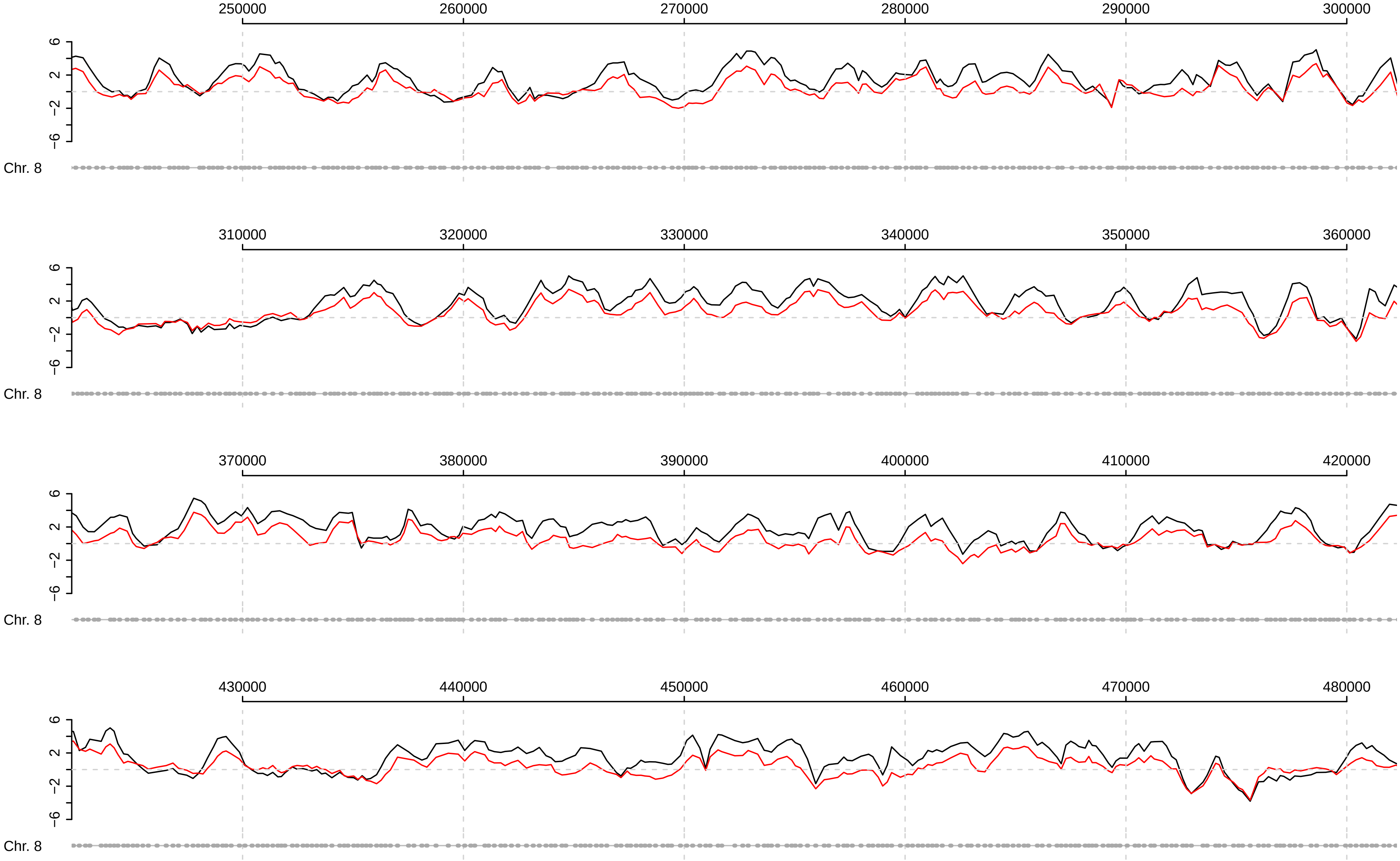

250000
260000
270000
280000
290000
300000
6
2
−2
−6
Chr. 8
310000
320000
330000
340000
350000
360000
6
2
−2
−6
Chr. 8
370000
380000
390000
400000
410000
420000
6
2
−2
−6
Chr. 8
430000
440000
450000
460000
470000
480000
6
2
−2
−6
Chr. 8

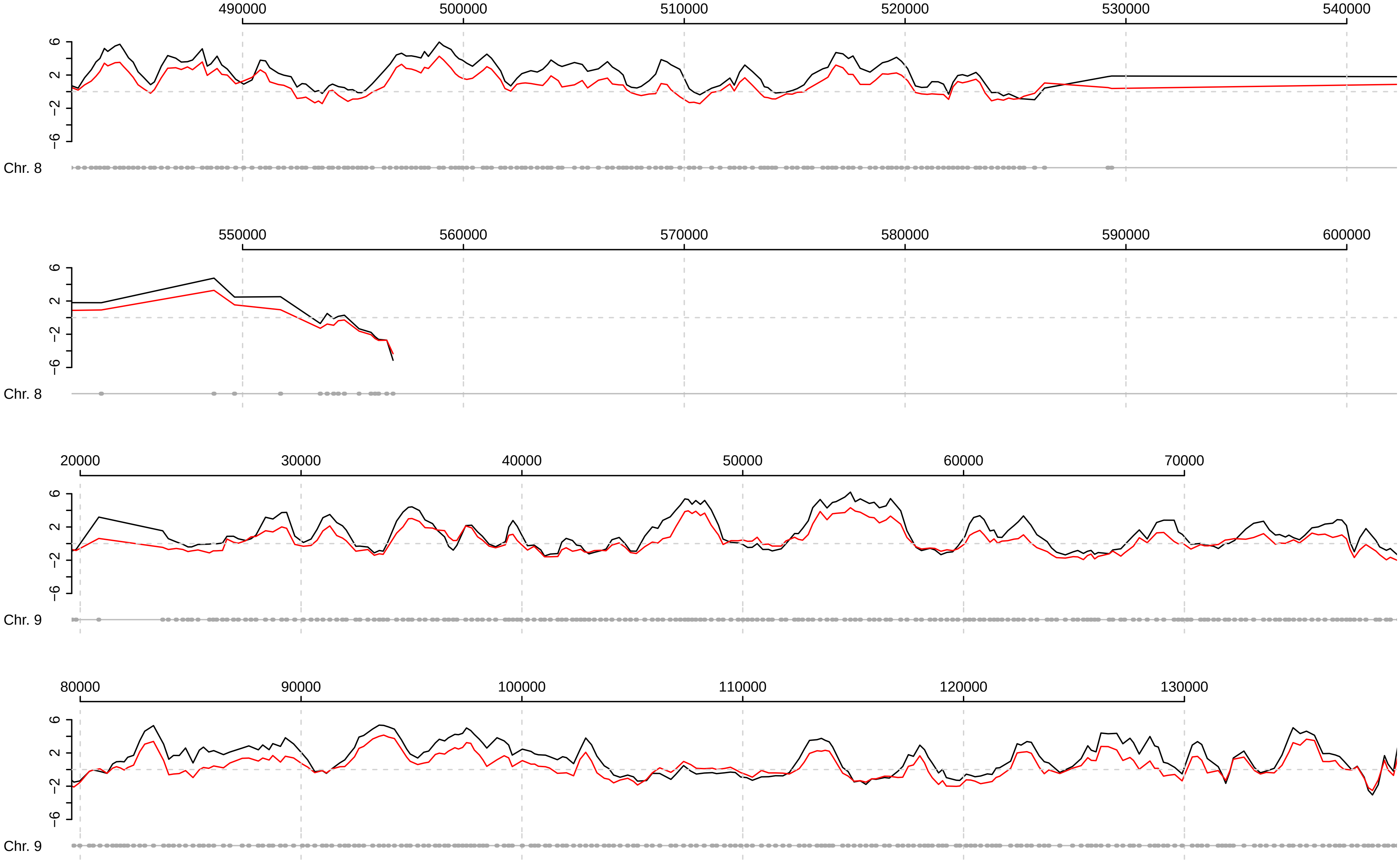

490000
500000
510000
520000
530000
540000
6
2
−2
−6
Chr. 8
550000
560000
570000
580000
590000
600000
6
2
−2
−6
Chr. 8
20000
30000
40000
50000
60000
70000
6
2
−2
−6
Chr. 9
80000
90000
100000
110000
120000
130000
6
2
−2
−6
Chr. 9

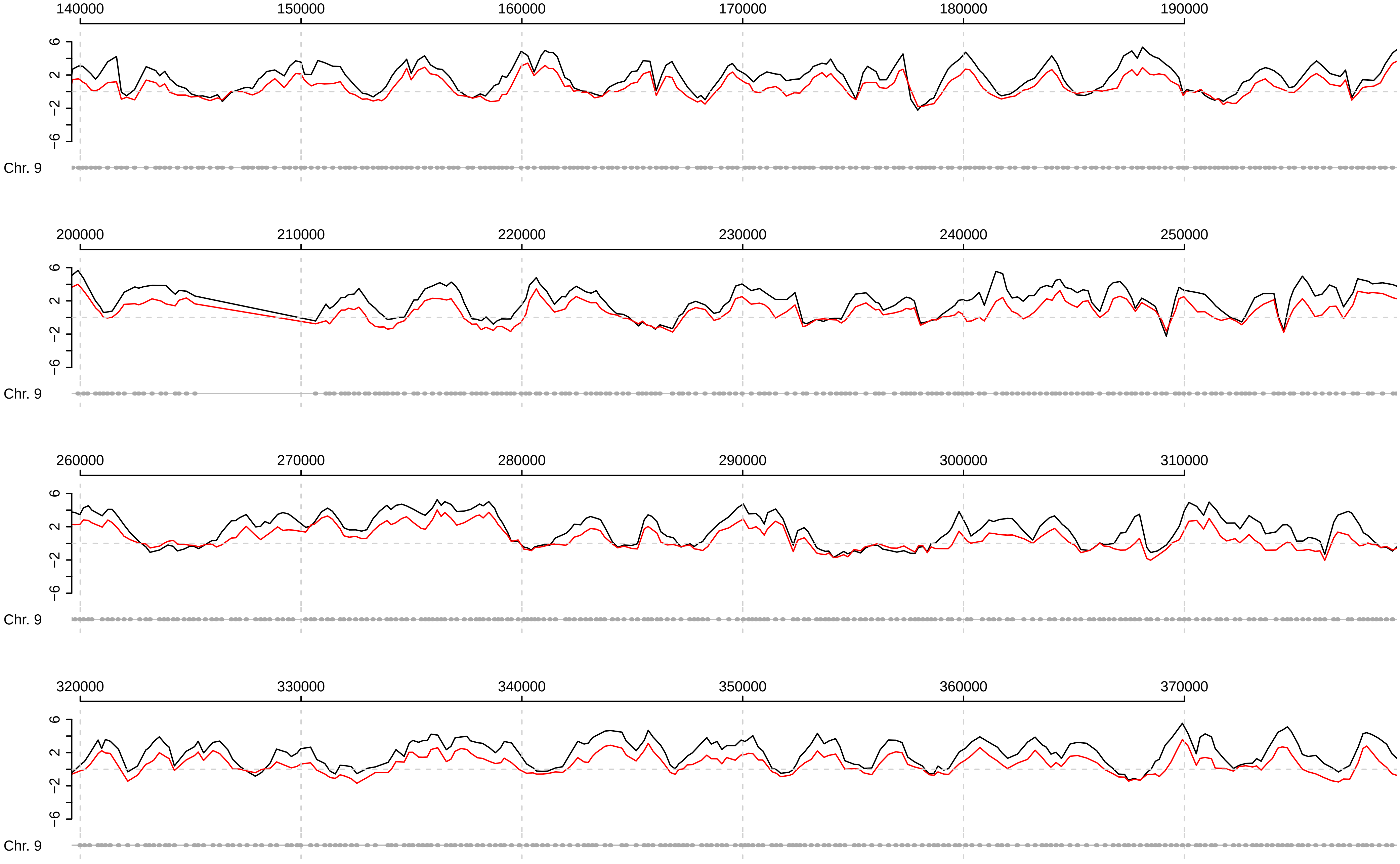

140000
150000
160000
170000
180000
190000
6
2
−2
−6
Chr. 9
200000
210000
220000
230000
240000
250000
6
2
−2
−6
Chr. 9
260000
270000
280000
290000
300000
310000
6
2
−2
−6
Chr. 9
320000
330000
340000
350000
360000
370000
6
2
−2
−6
Chr. 9

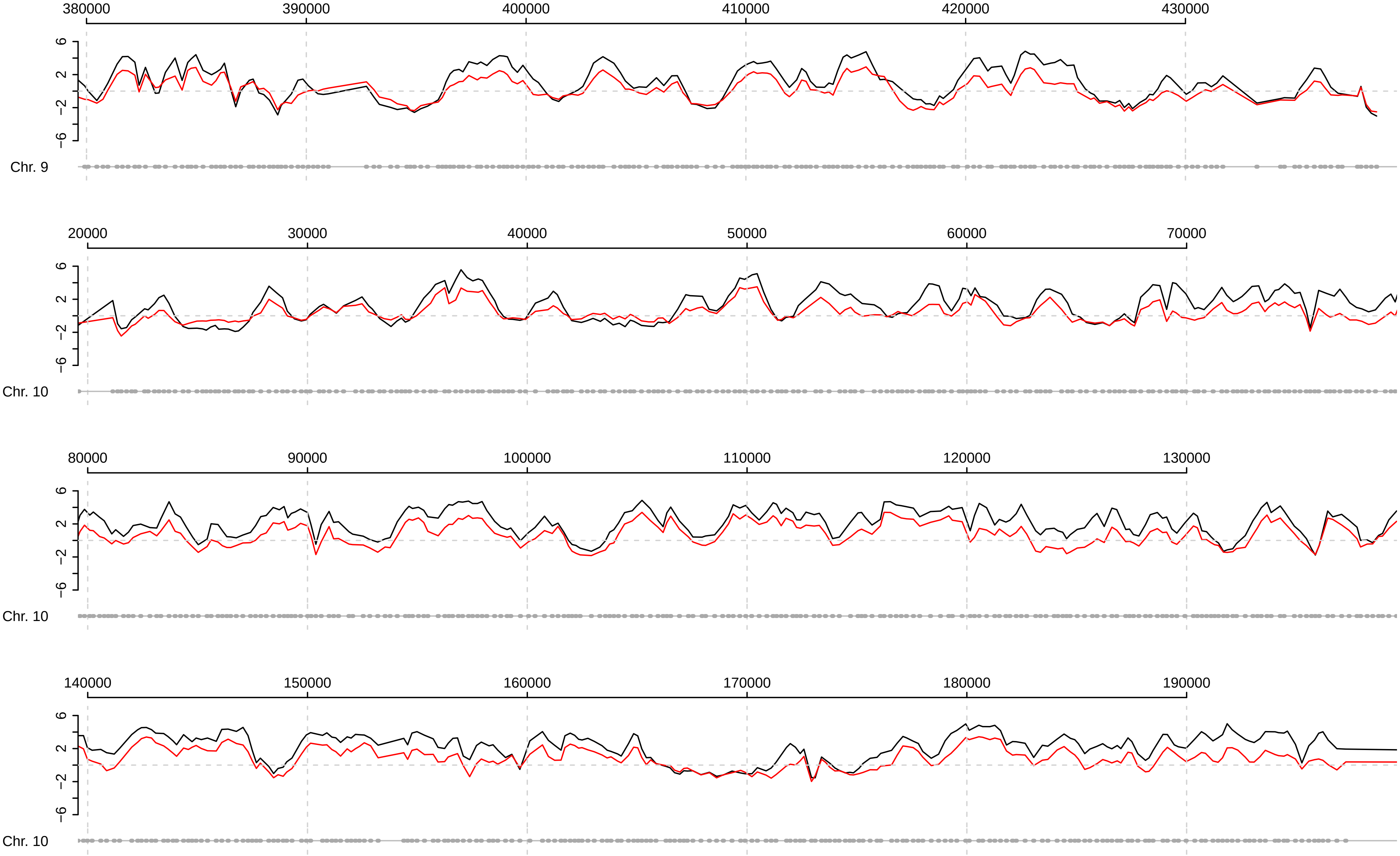

380000
390000
400000
410000
420000
430000
6
2
−2
−6
Chr. 9
20000
30000
40000
50000
60000
70000
6
2
−2
−6
Chr. 10
80000
90000
100000
110000
120000
130000
6
2
−2
−6
Chr. 10
140000
150000
160000
170000
180000
190000
6
2
−2
−6
Chr. 10

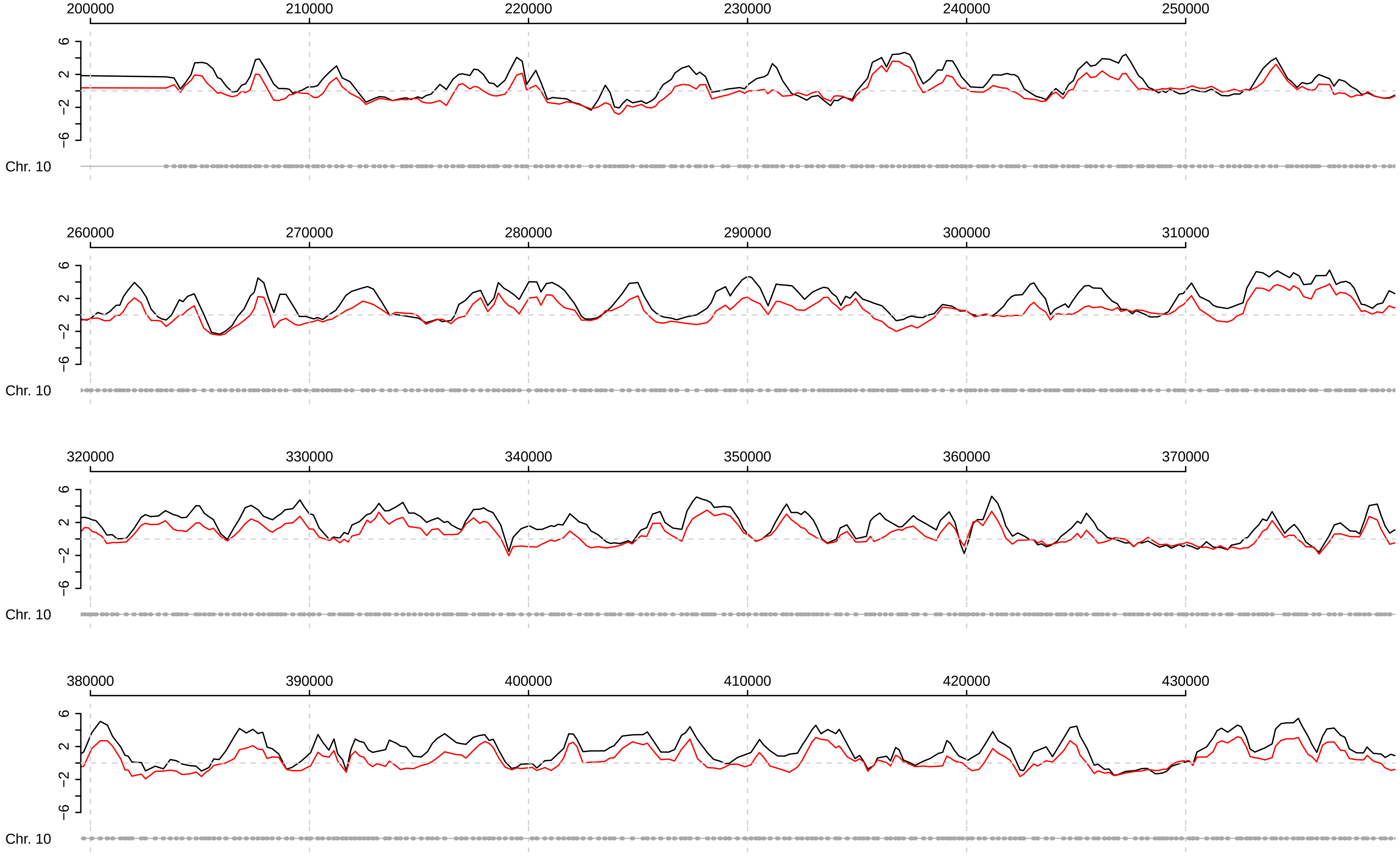
200000
210000
220000
230000
240000
250000
6
2
−2
−6
Chr. 10
260000
270000
280000
290000
300000
310000
6
2
−2
−6
Chr. 10
320000
330000
340000
350000
360000
370000
6
2
−2
−6
Chr. 10
380000
390000
400000
410000
420000
430000
6
2
−2
−6
Chr. 10

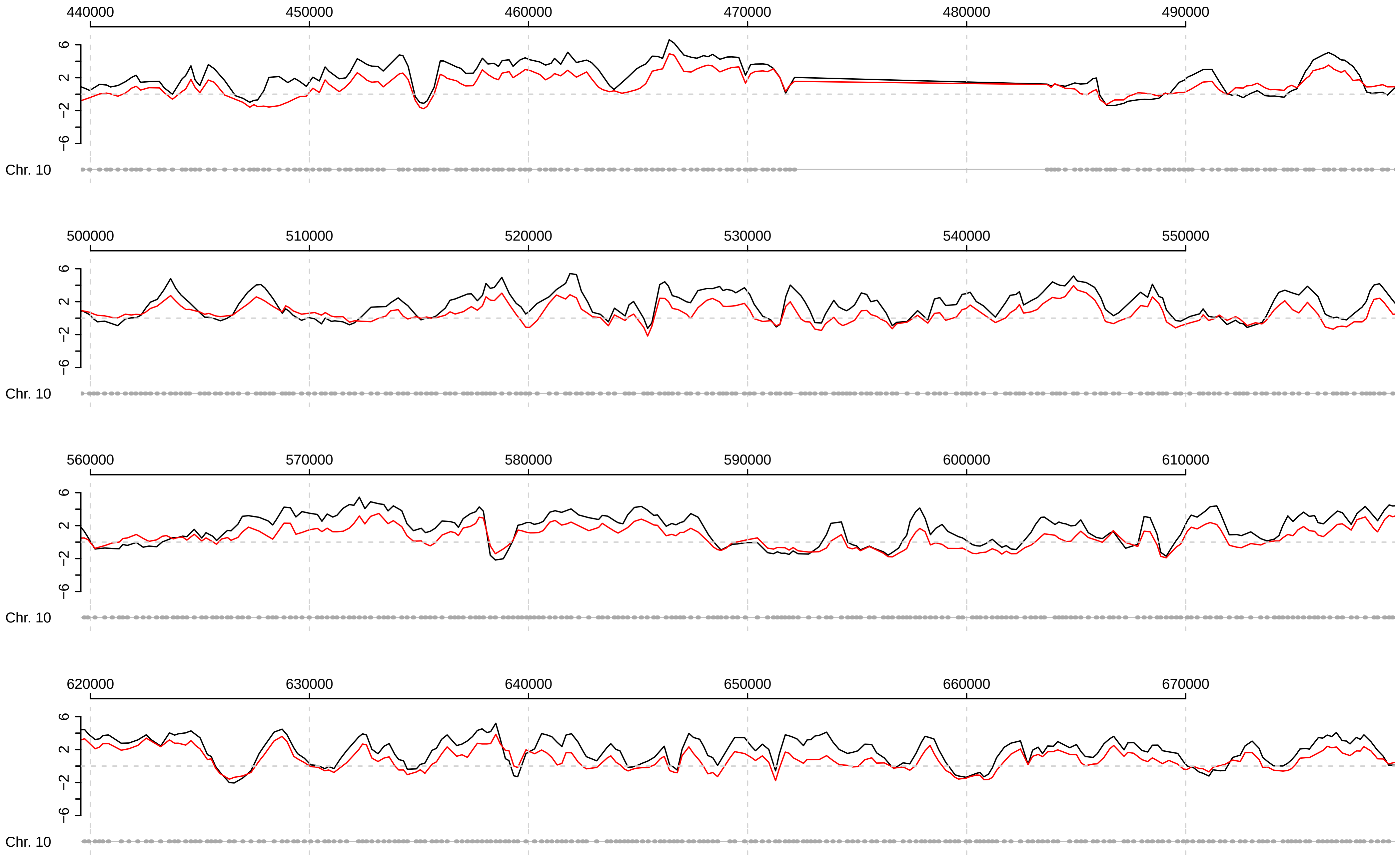

440000
450000
460000
470000
480000
490000
6
2
−2
−6
Chr. 10
500000
510000
520000
530000
540000
550000
6
2
−2
−6
Chr. 10
560000
570000
580000
590000
600000
610000
6
2
−2
−6
Chr. 10
620000
630000
640000
650000
660000
670000
6
2
−2
−6
Chr. 10

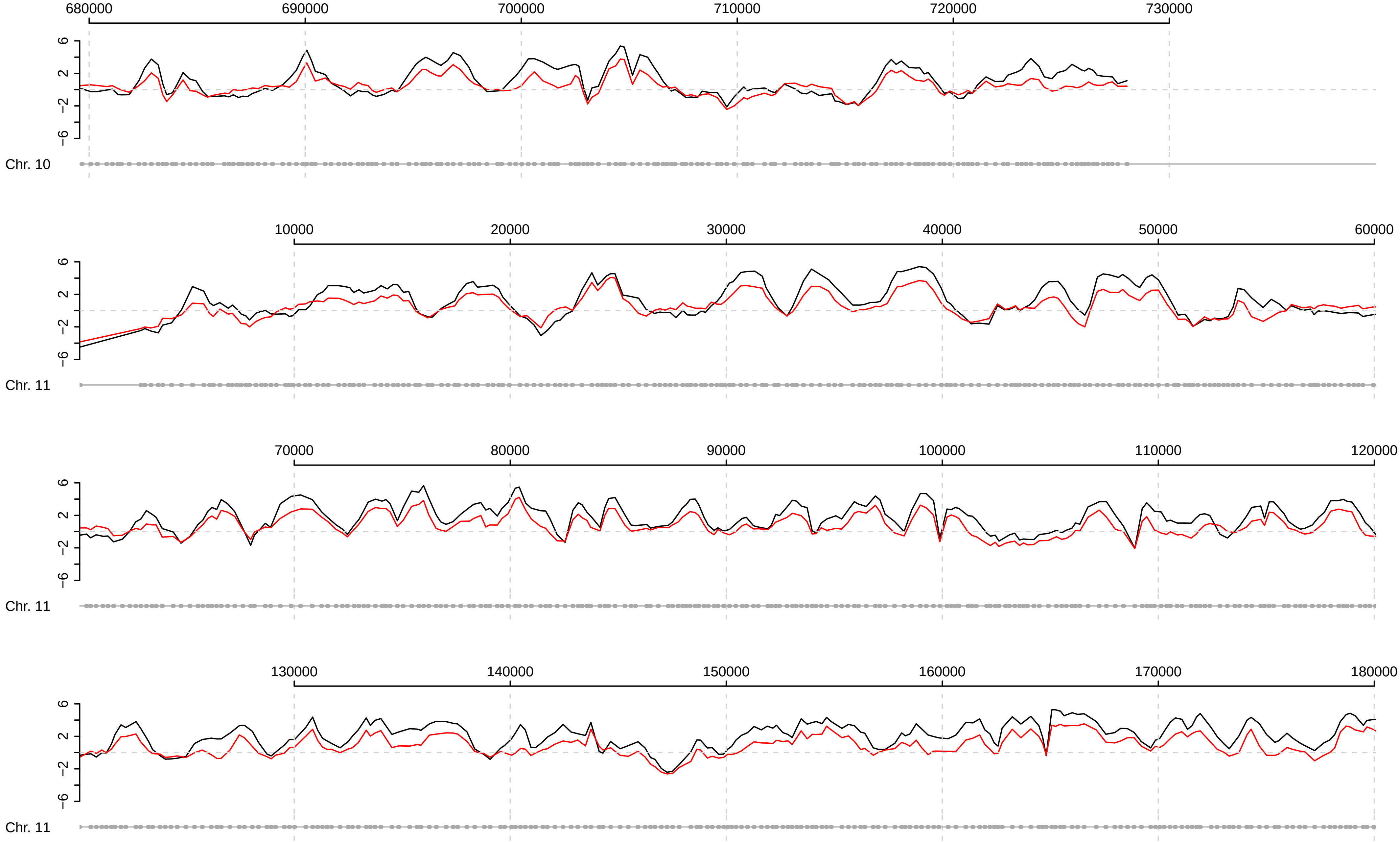

680000
690000
700000
710000
720000
730000
6
2
−2
−6
Chr. 10
10000
20000
30000
40000
50000
60000
Chr. 11
70000
80000
90000
100000
110000
120000
Chr. 11
130000
140000
150000
160000
170000
180000
Chr. 11

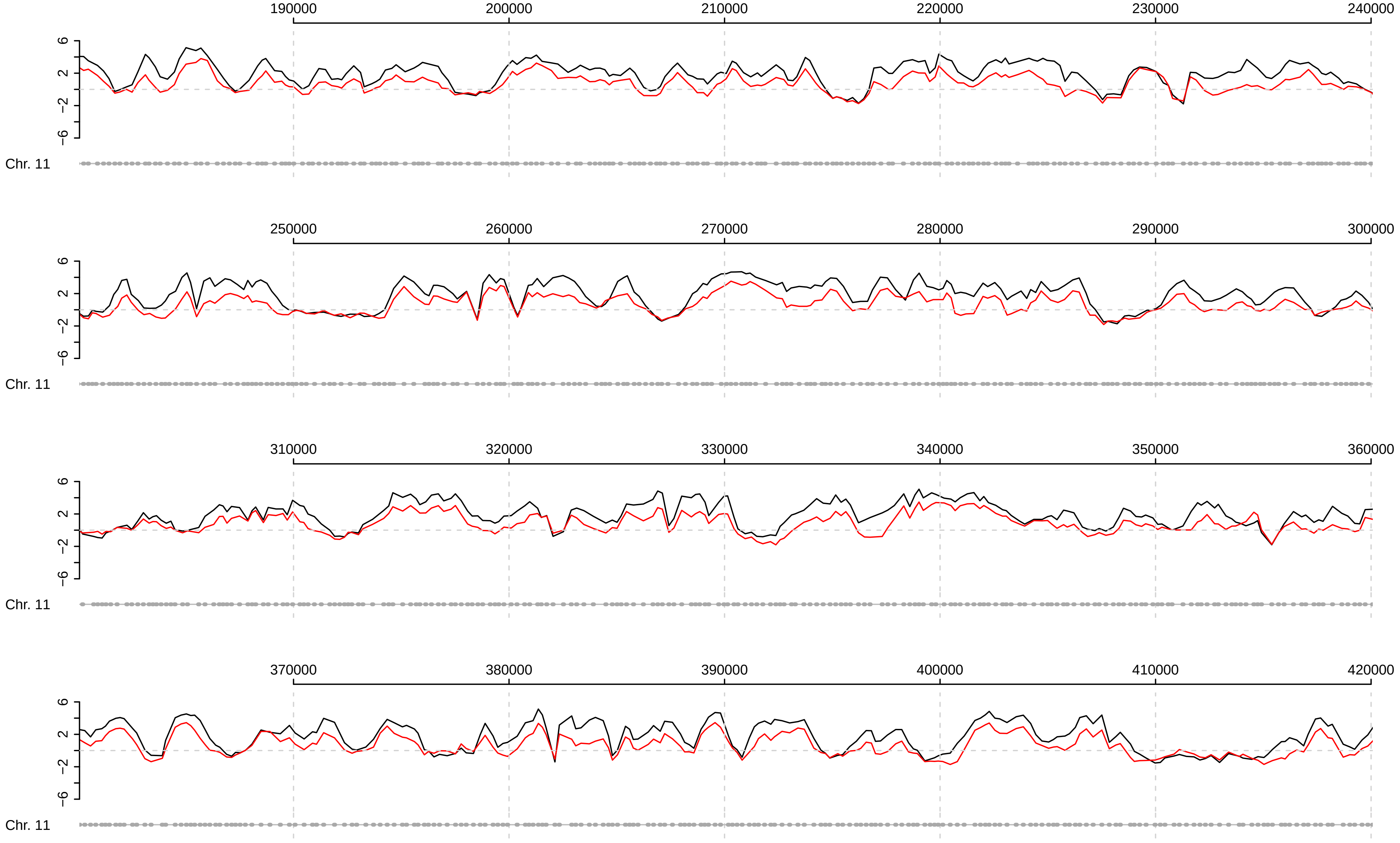

190000
200000
210000
220000
230000
240000
6
2
−2
−6
Chr. 11
250000
260000
270000
280000
290000
300000
Chr. 11
310000
320000
330000
340000
350000
360000
Chr. 11
370000
380000
390000
400000
410000
420000
Chr. 11

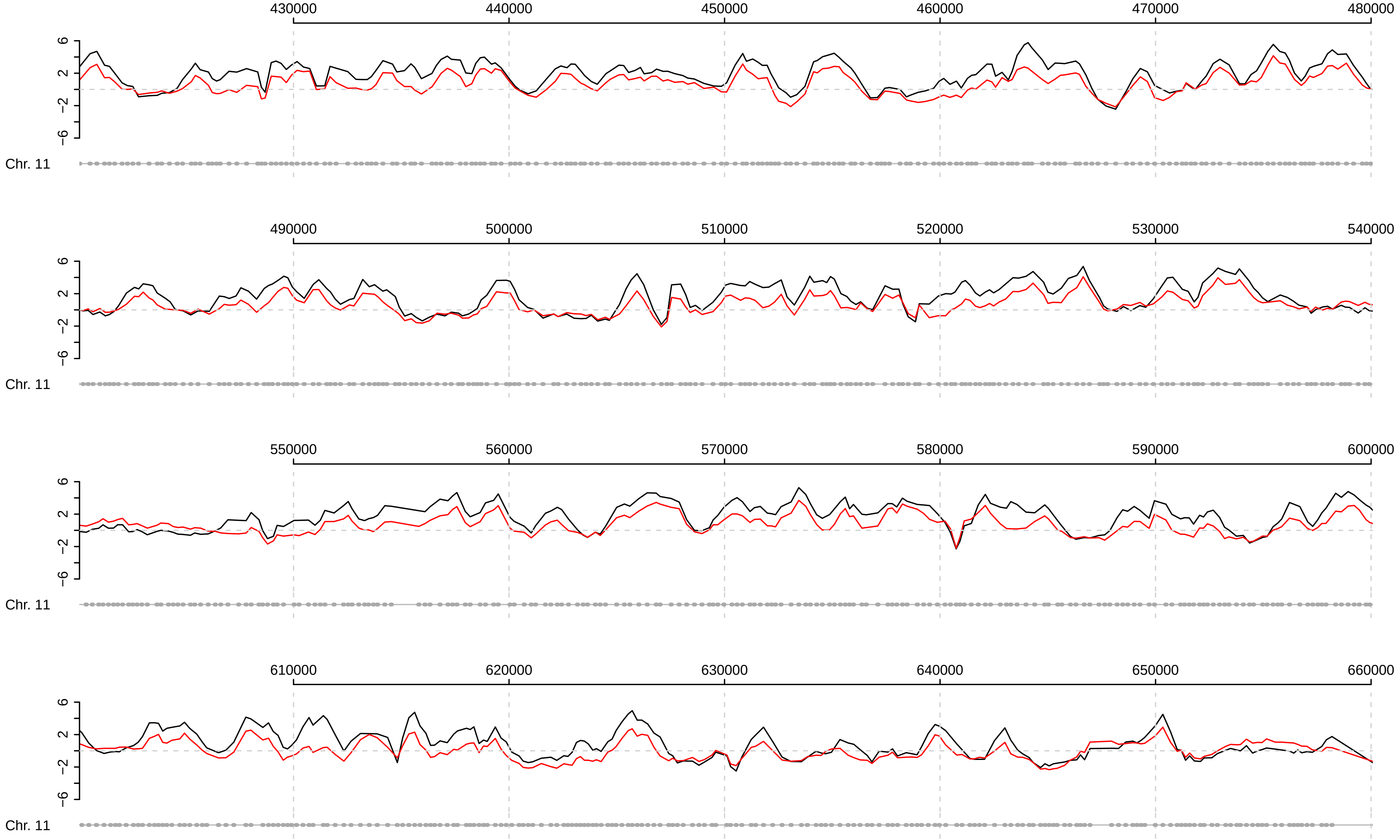

430000
440000
450000
460000
470000
480000
6
2
−2
−6
Chr. 11
490000
500000
510000
520000
530000
540000
6
2
−2
−6
Chr. 11
550000
560000
570000
580000
590000
600000
6
2
−2
−6
Chr. 11
610000
620000
630000
640000
650000
660000
6
2
−2
−6
Chr. 11

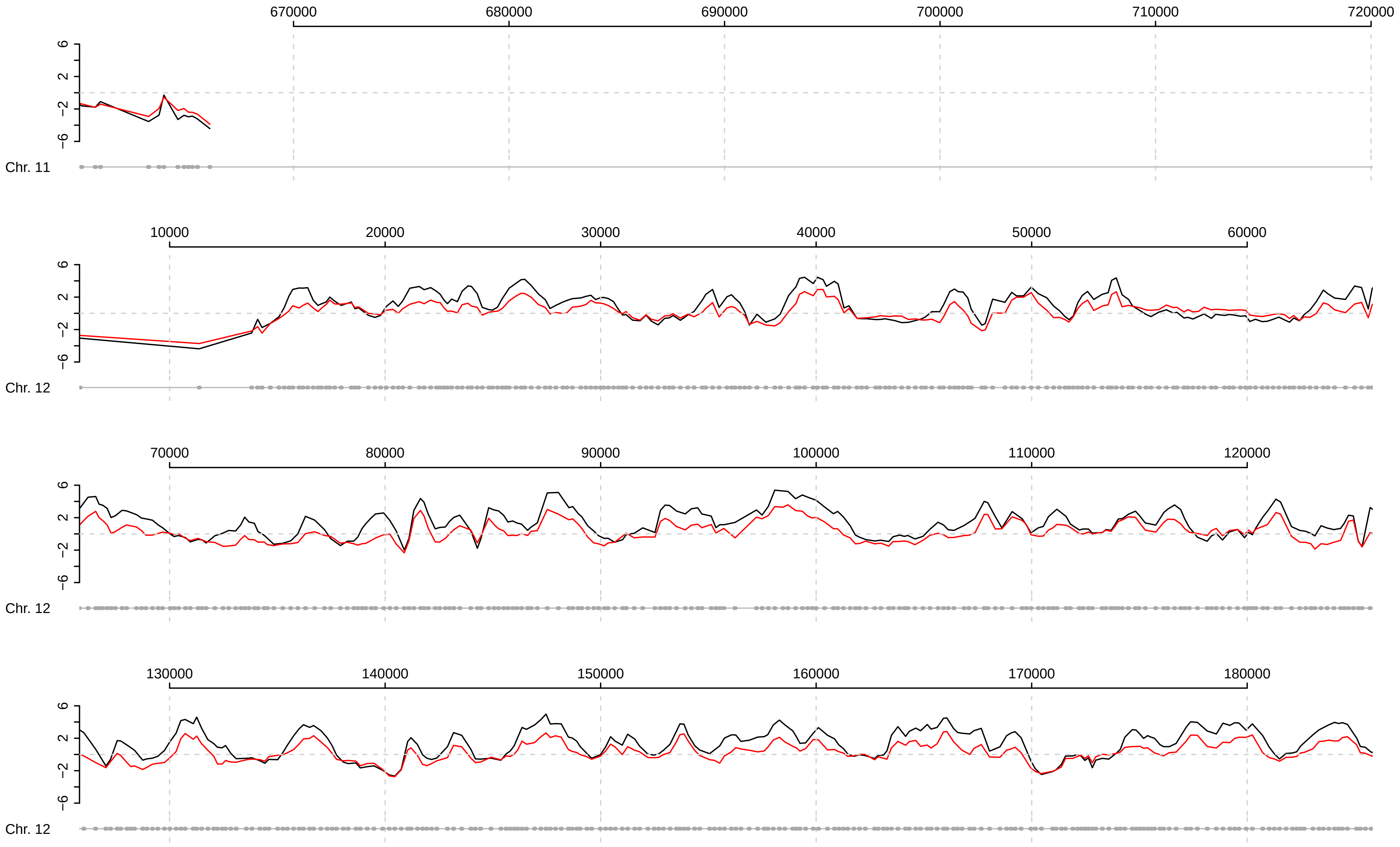

670000
680000
690000
700000
710000
720000
6
2
−2
−6
Chr. 11
10000
20000
30000
40000
50000
60000
Chr. 12
70000
80000
90000
100000
110000
120000
Chr. 12
130000
140000
150000
160000
170000
180000
Chr. 12

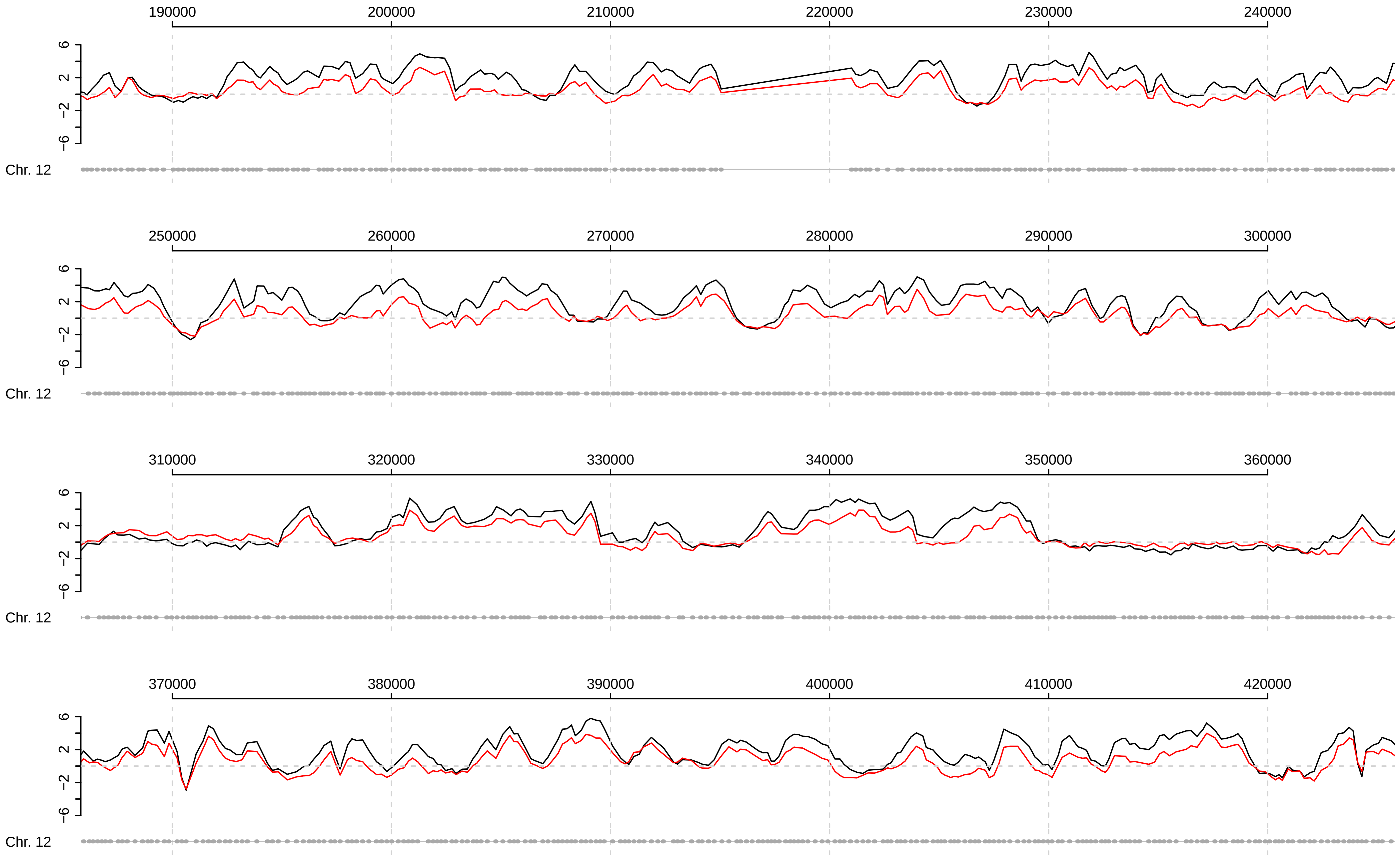

190000
200000
210000
220000
230000
240000
6
2
−2
−6
Chr. 12
250000
260000
270000
280000
290000
300000
310000
320000
330000
340000
350000
360000
370000
380000
390000
400000
410000
420000

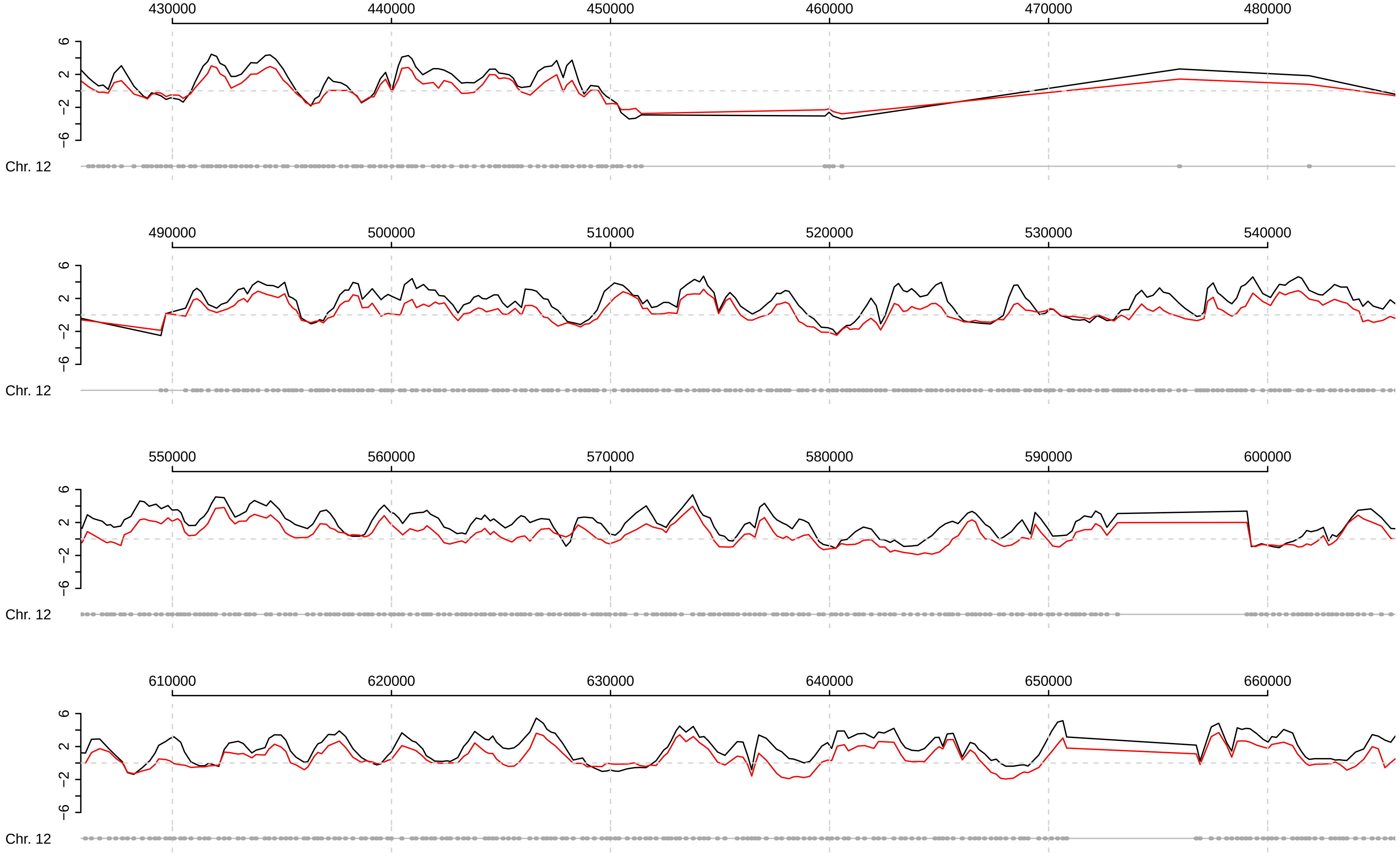

430000
440000
450000
460000
470000
480000
6
2
−2
−6
Chr. 12
490000
500000
510000
520000
530000
540000
6
2
−2
−6
Chr. 12
550000
560000
570000
580000
590000
600000
6
2
−2
−6
Chr. 12
610000
620000
630000
640000
650000
660000
6
2
−2
−6
Chr. 12

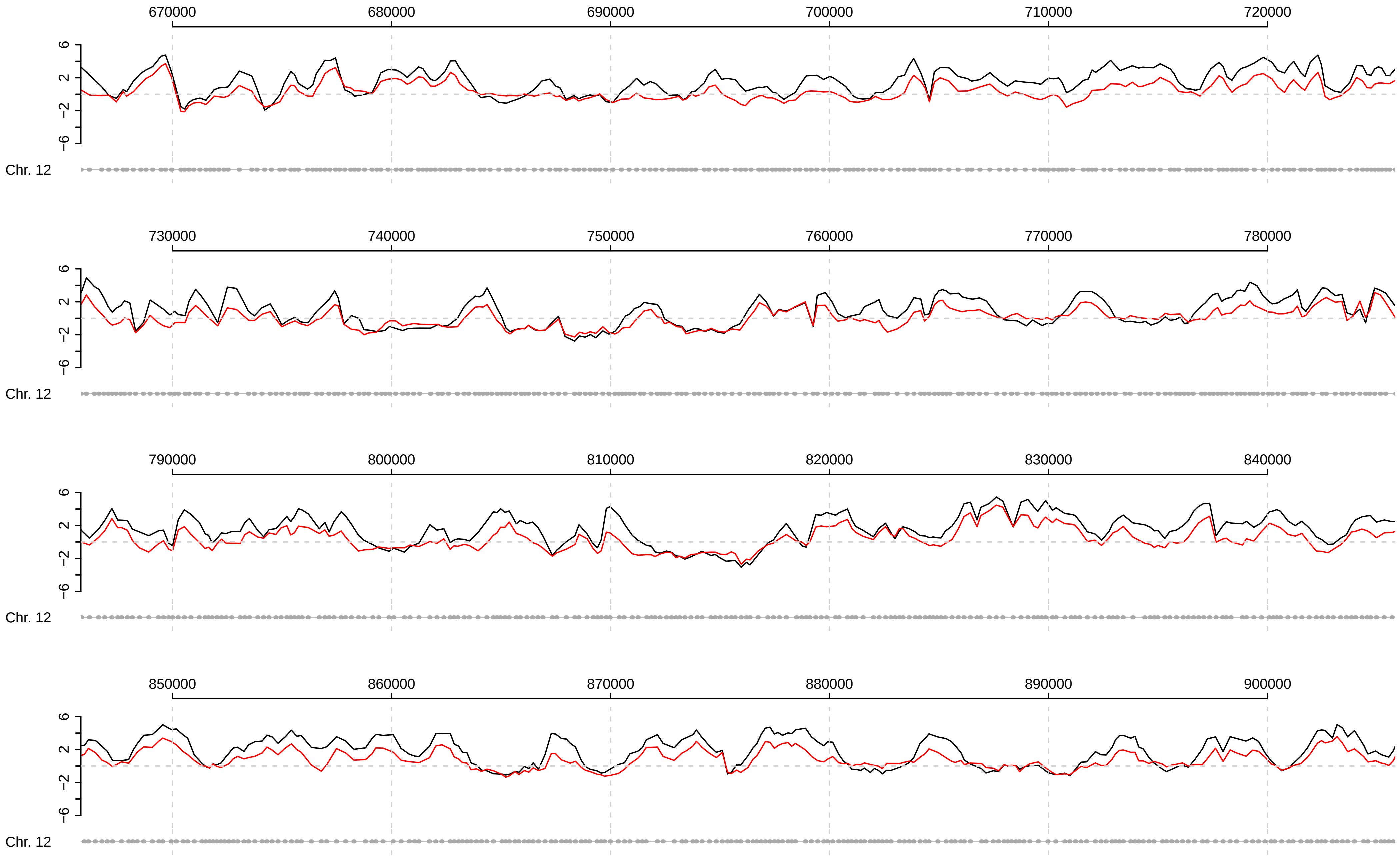

670000
680000
690000
700000
710000
720000
6
2
−2
−6
Chr. 12
730000
740000
750000
760000
770000
780000
6
2
−2
−6
Chr. 12
790000
800000
810000
820000
830000
840000
6
2
−2
−6
Chr. 12
850000
860000
870000
880000
890000
900000
6
2
−2
−6
Chr. 12

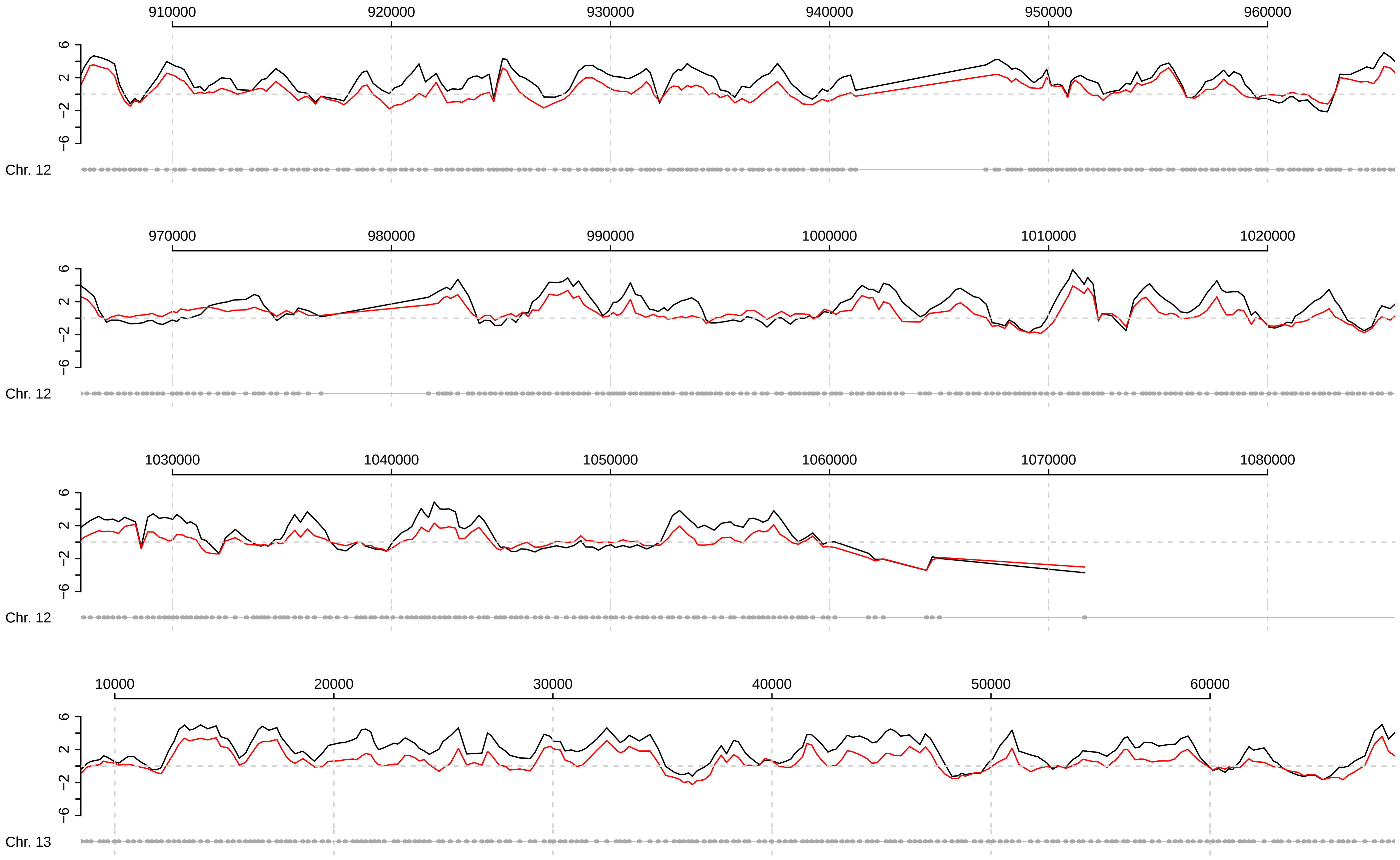
910000
920000
930000
940000
950000
960000
6
2
−2
−6
Chr. 12
970000
980000
990000
1000000
1010000
1020000
6
2
−2
−6
Chr. 12
1030000
1040000
1050000
1060000
1070000
1080000
6
2
−2
−6
Chr. 12
10000
20000
30000
40000
50000
60000
6
2
−2
−6
Chr. 13

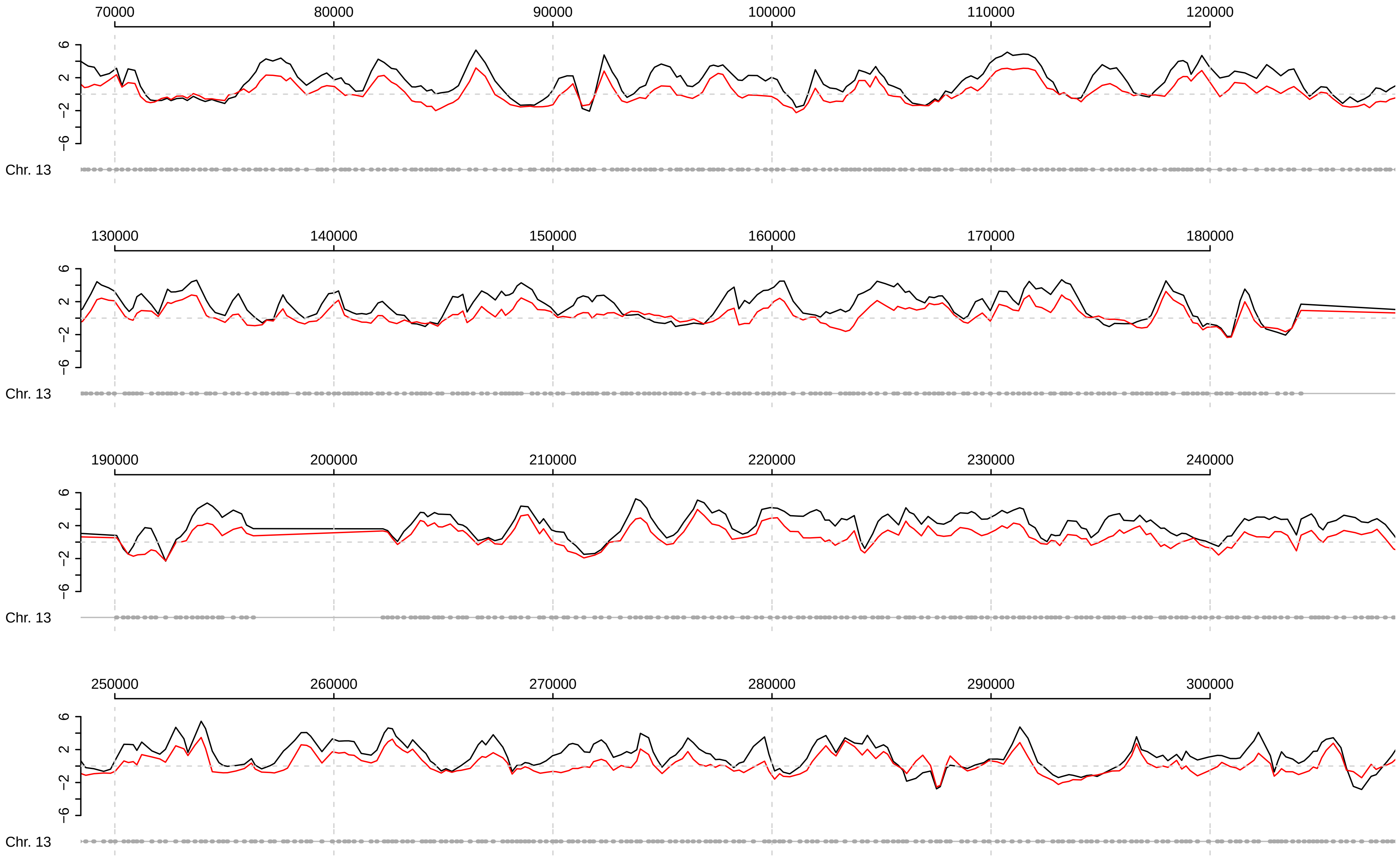
70000
80000
90000
100000
110000
120000
6
2
−2
−6
Chr. 13
130000
140000
150000
160000
170000
180000
6
2
−2
−6
Chr. 13
190000
200000
210000
220000
230000
240000
6
2
−2
−6
Chr. 13
250000
260000
270000
280000
290000
300000
6
2
−2
−6
Chr. 13

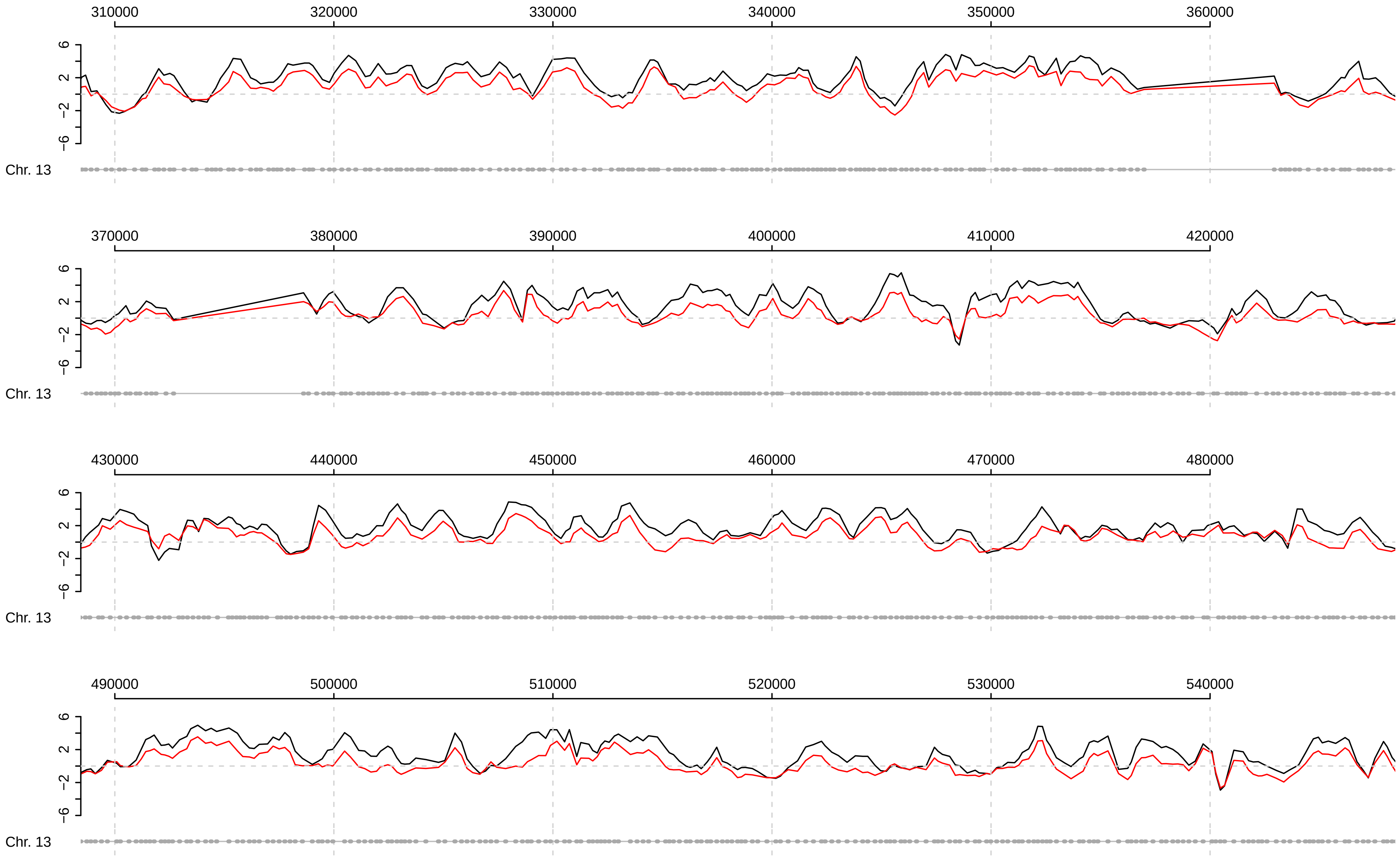

310000
320000
330000
340000
350000
360000
6
2
−2
−6
Chr. 13
370000
380000
390000
400000
410000
420000
6
2
−2
−6
Chr. 13
430000
440000
450000
460000
470000
480000
6
2
−2
−6
Chr. 13
490000
500000
510000
520000
530000
540000
6
2
−2
−6
Chr. 13

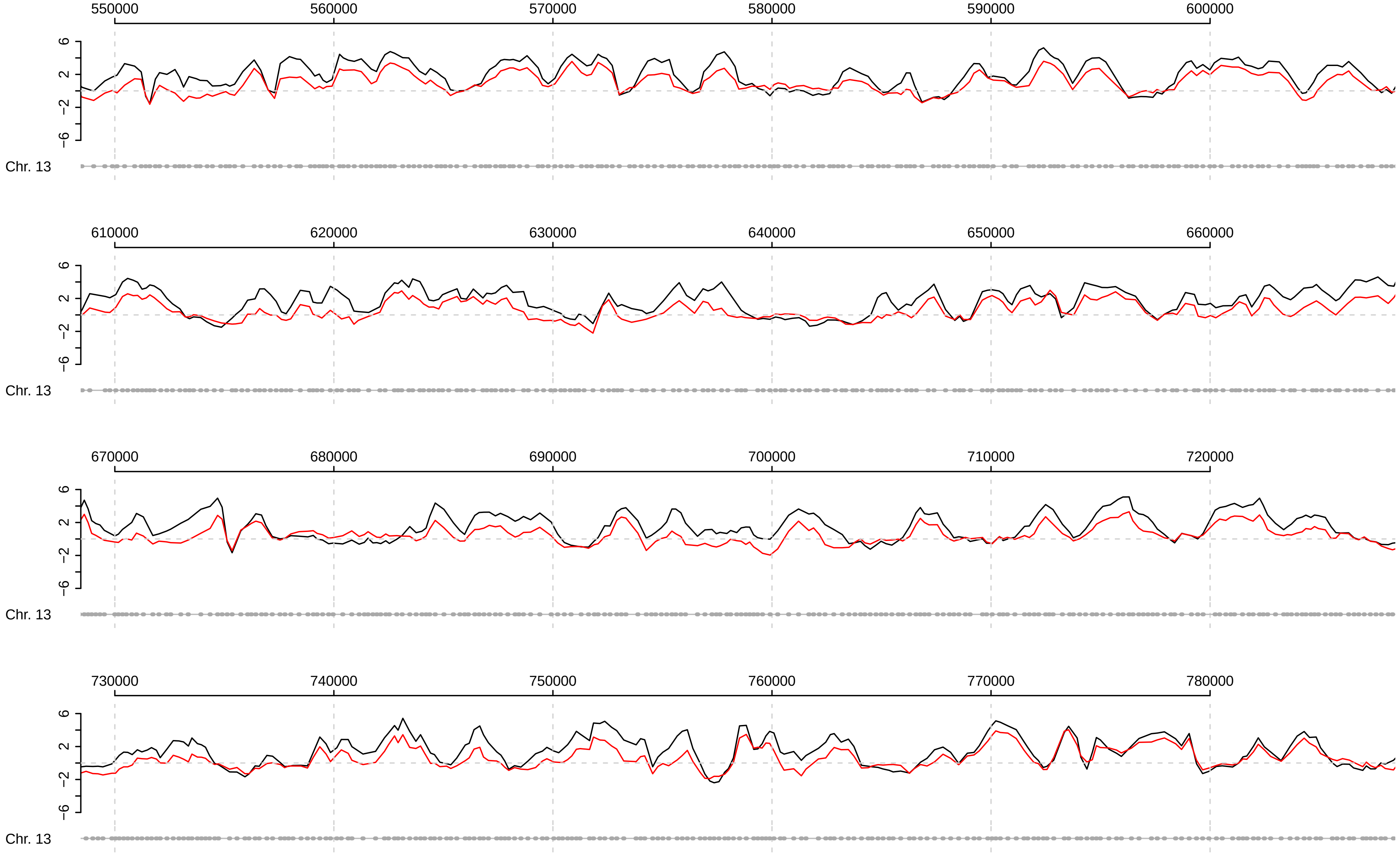

550000
560000
570000
580000
590000
600000
6
2
−2
−6
Chr. 13
610000
620000
630000
640000
650000
660000
6
2
−2
−6
Chr. 13
670000
680000
690000
700000
710000
720000
6
2
−2
−6
Chr. 13
730000
740000
750000
760000
770000
780000
6
2
−2
−6
Chr. 13

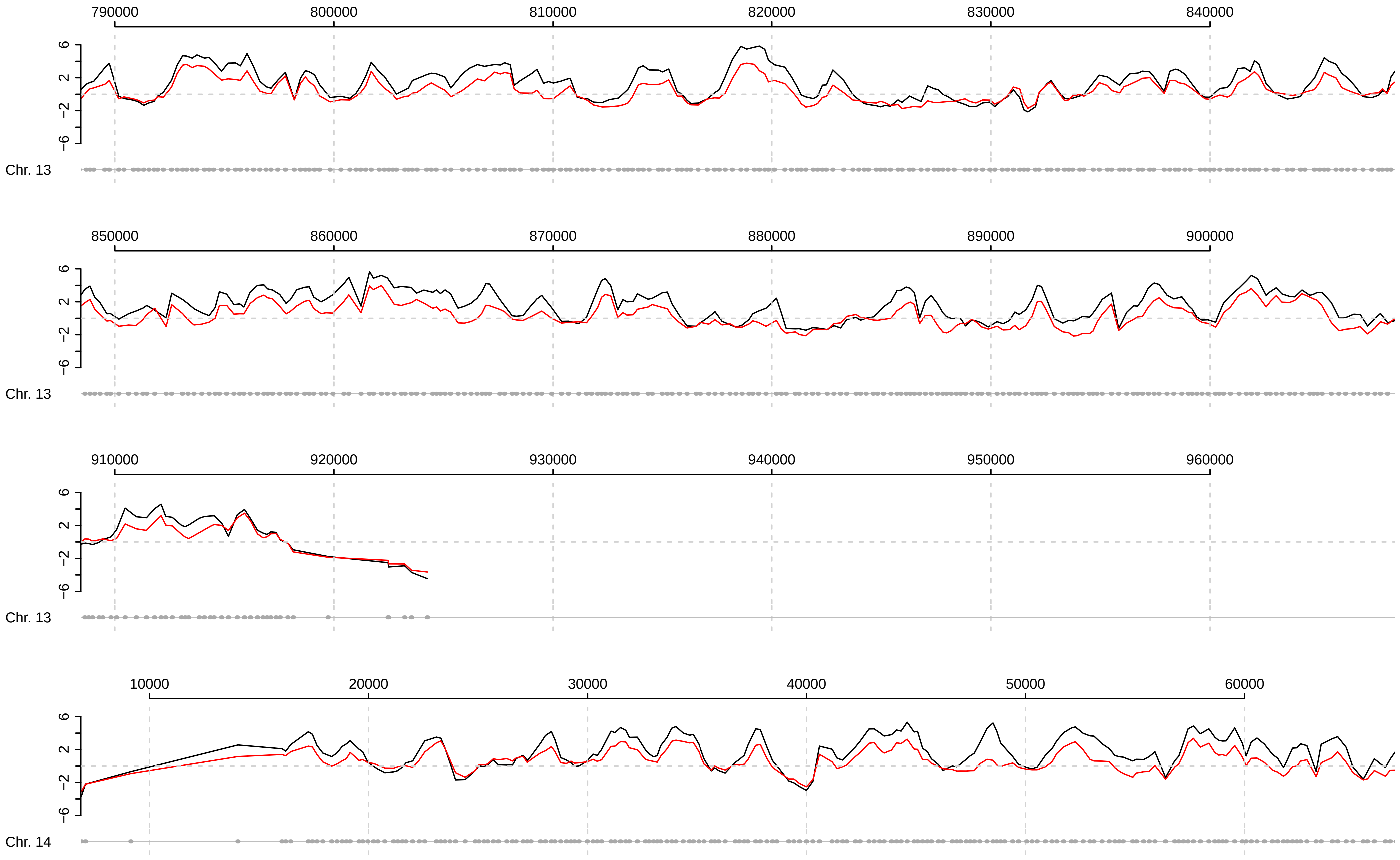

790000
800000
810000
820000
830000
840000
6
2
−2
−6
Chr. 13
850000
860000
870000
880000
890000
900000
Chr. 13
910000
920000
930000
940000
950000
960000
Chr. 13
10000
20000
30000
40000
50000
60000
Chr. 14

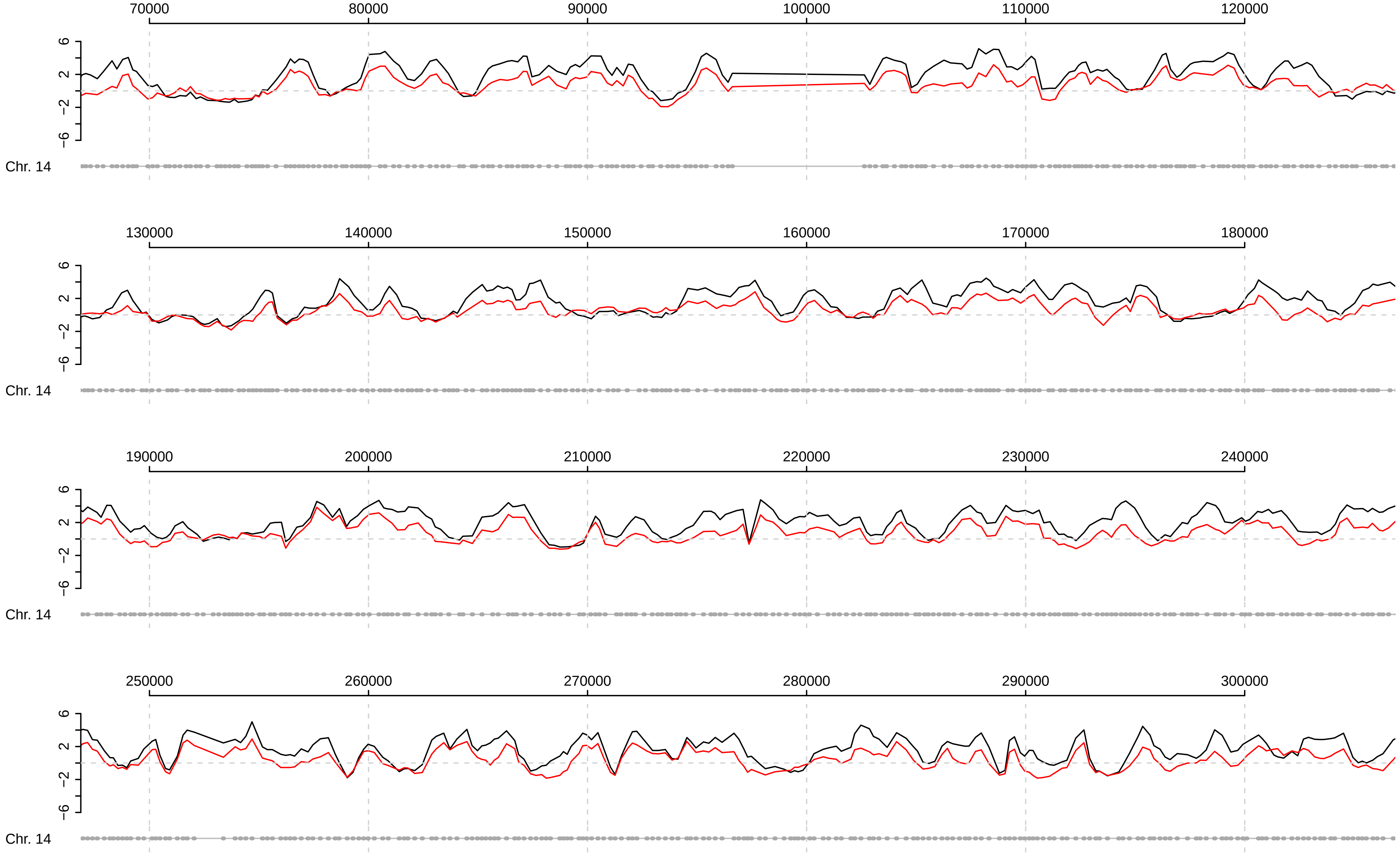
70000
80000
90000
100000
110000
120000
6
2
−2
−6
Chr. 14
130000
140000
150000
160000
170000
180000
6
2
−2
−6
Chr. 14
190000
200000
210000
220000
230000
240000
6
2
−2
−6
Chr. 14
250000
260000
270000
280000
290000
300000
6
2
−2
−6
Chr. 14

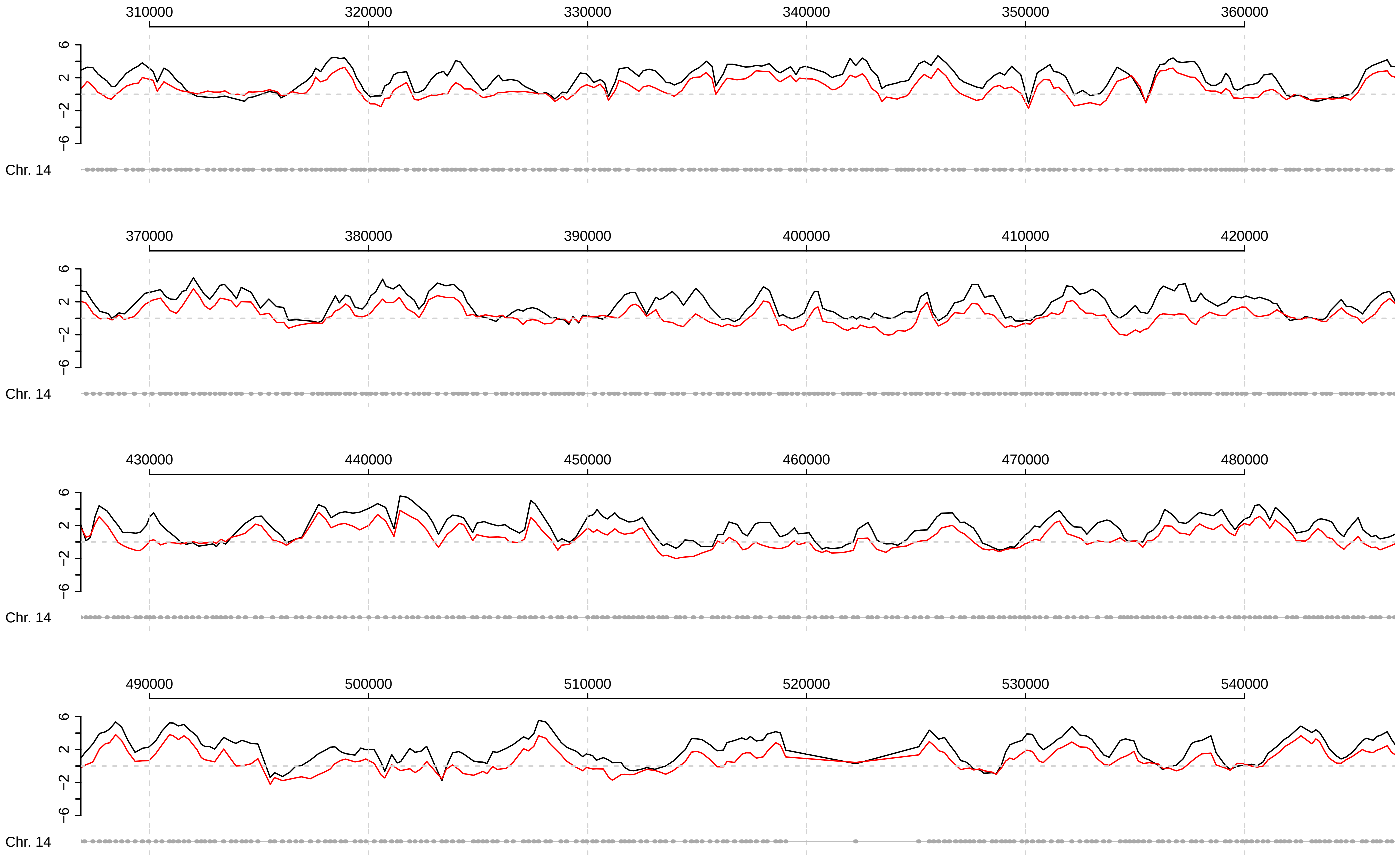
310000
320000
330000
340000
350000
360000
6
2
−2
−6
Chr. 14
370000
380000
390000
400000
410000
420000
6
2
−2
−6
Chr. 14
430000
440000
450000
460000
470000
480000
6
2
−2
−6
Chr. 14
490000
500000
510000
520000
530000
540000
6
2
−2
−6
Chr. 14

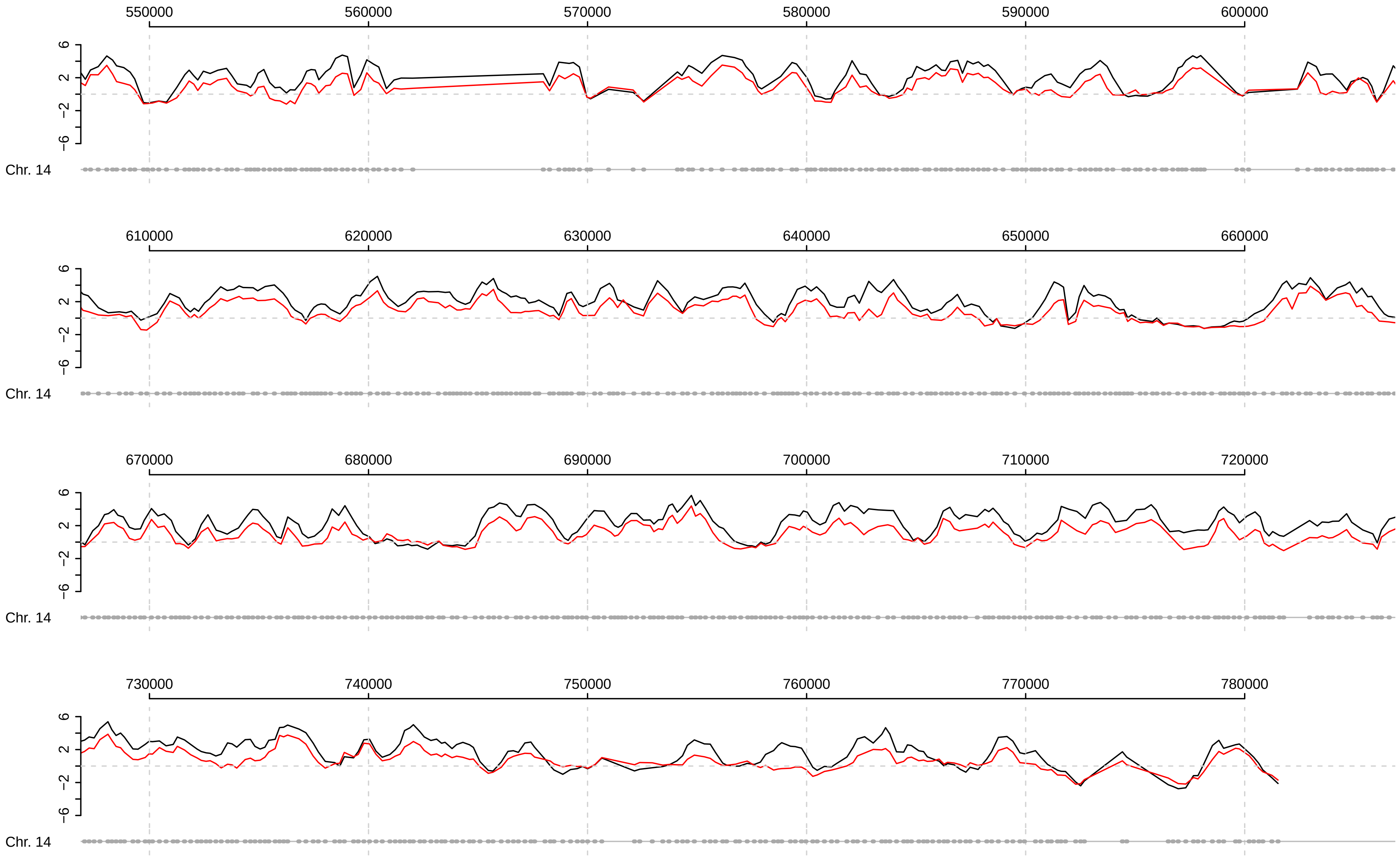
550000
560000
570000
580000
590000
600000
6
2
−2
−6
Chr. 14
610000
620000
630000
640000
650000
660000
6
2
−2
−6
Chr. 14
670000
680000
690000
700000
710000
720000
6
2
−2
−6
Chr. 14
730000
740000
750000
760000
770000
780000
6
2
−2
−6
Chr. 14

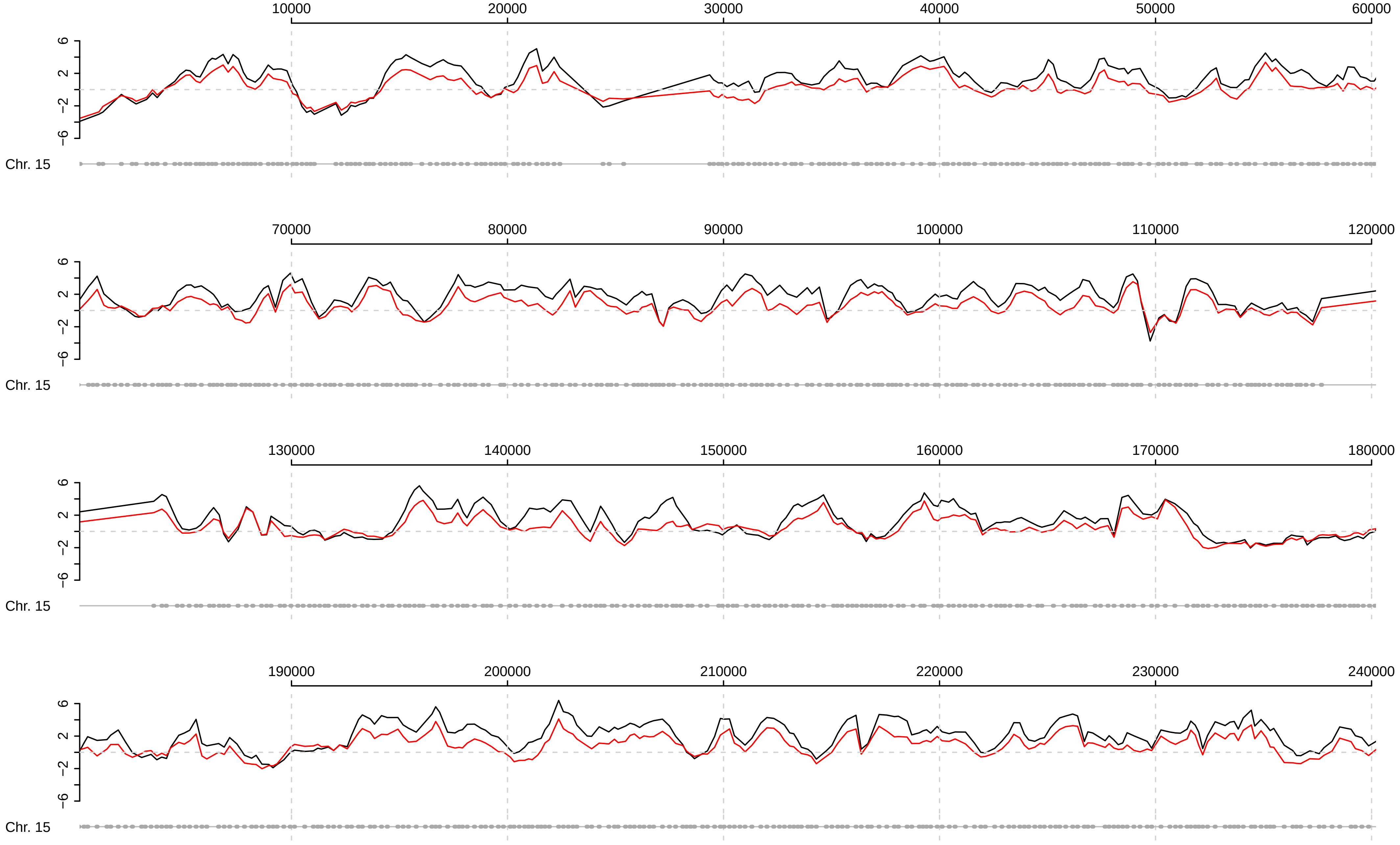
10000
20000
30000
40000
50000
60000
70000
80000
90000
100000
110000
120000
130000
140000
150000
160000
170000
180000
190000
200000
210000
220000
230000
240000
6
2
−2
−6
Chr. 15

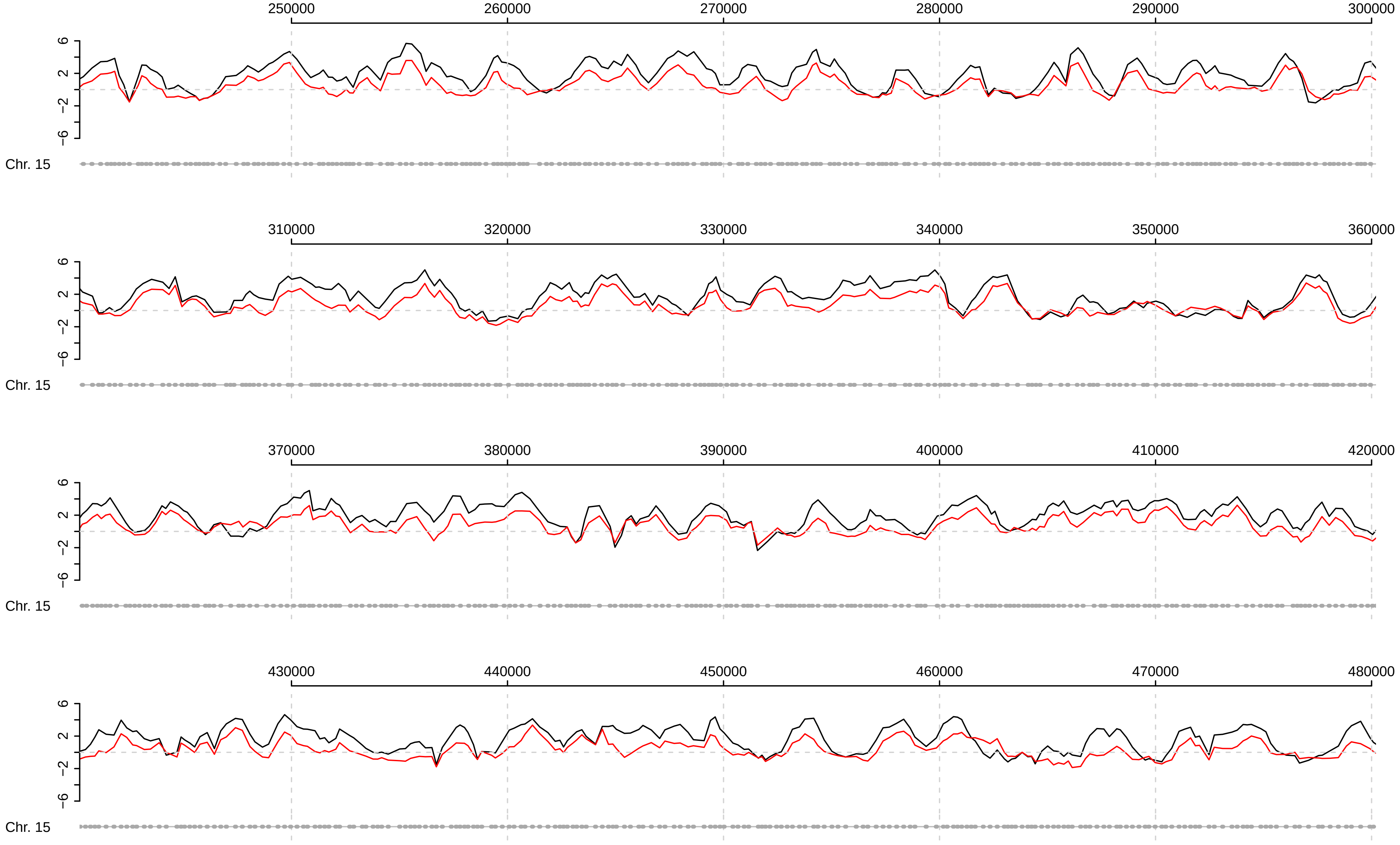
250000
260000
270000
280000
290000
300000
6
2
−2
−6
Chr. 15
310000
320000
330000
340000
350000
360000
6
2
−2
−6
Chr. 15
370000
380000
390000
400000
410000
420000
6
2
−2
−6
Chr. 15
430000
440000
450000
460000
470000
480000
6
2
−2
−6
Chr. 15

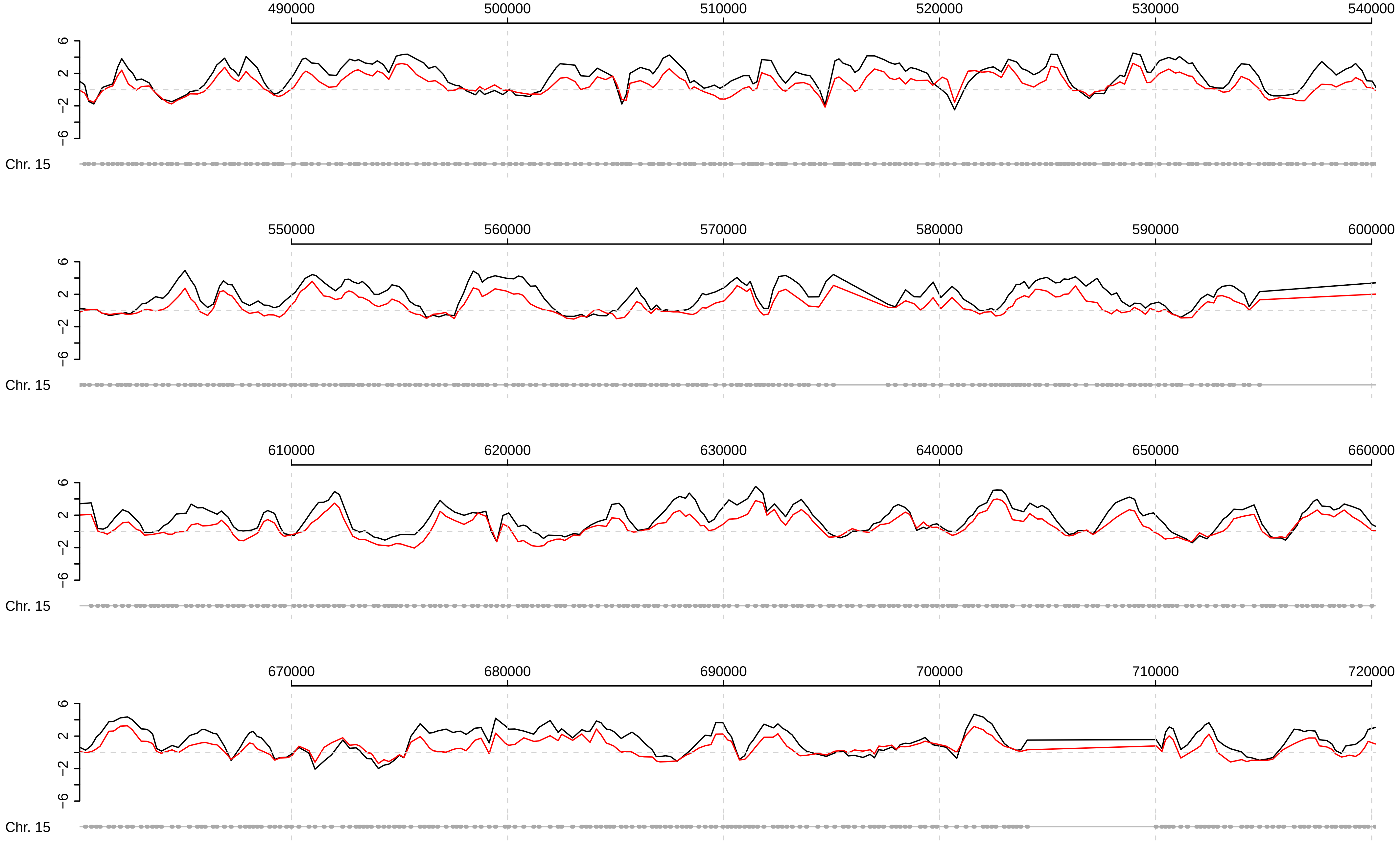
490000
500000
510000
520000
530000
540000
6
2
−2
−6
Chr. 15
550000
560000
570000
580000
590000
600000
6
2
−2
−6
Chr. 15
610000
620000
630000
640000
650000
660000
6
2
−2
−6
Chr. 15
670000
680000
690000
700000
710000
720000
6
2
−2
−6
Chr. 15

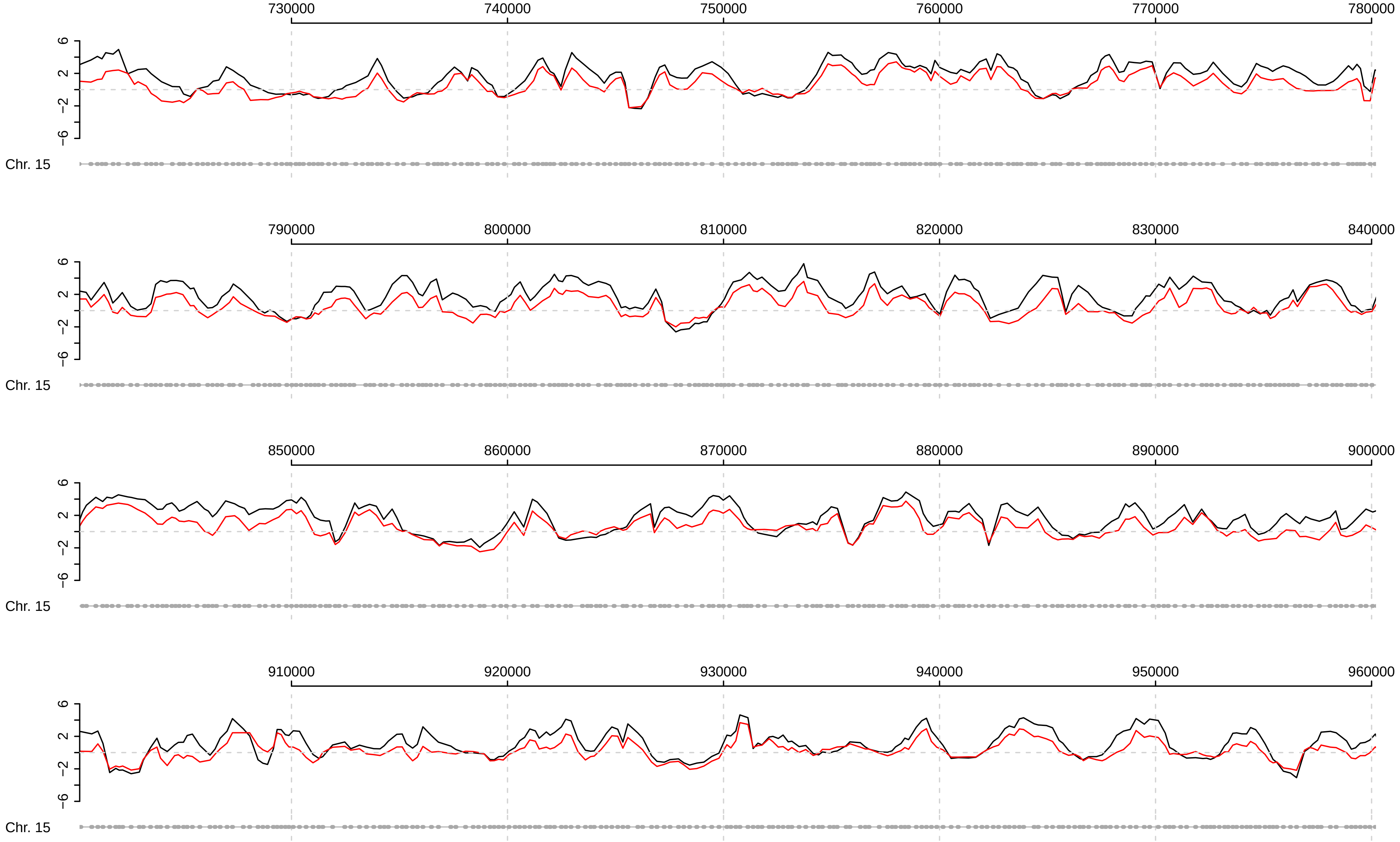
730000 740000 750000 760000 770000 780000
6 2 −2 −6
Chr. 15
790000 800000 810000 820000 830000 840000
6 2 −2 −6
Chr. 15
850000 860000 870000 880000 890000 900000
6 2 −2 −6
Chr. 15
910000 920000 930000 940000 950000 960000
6 2 −2 −6
Chr. 15

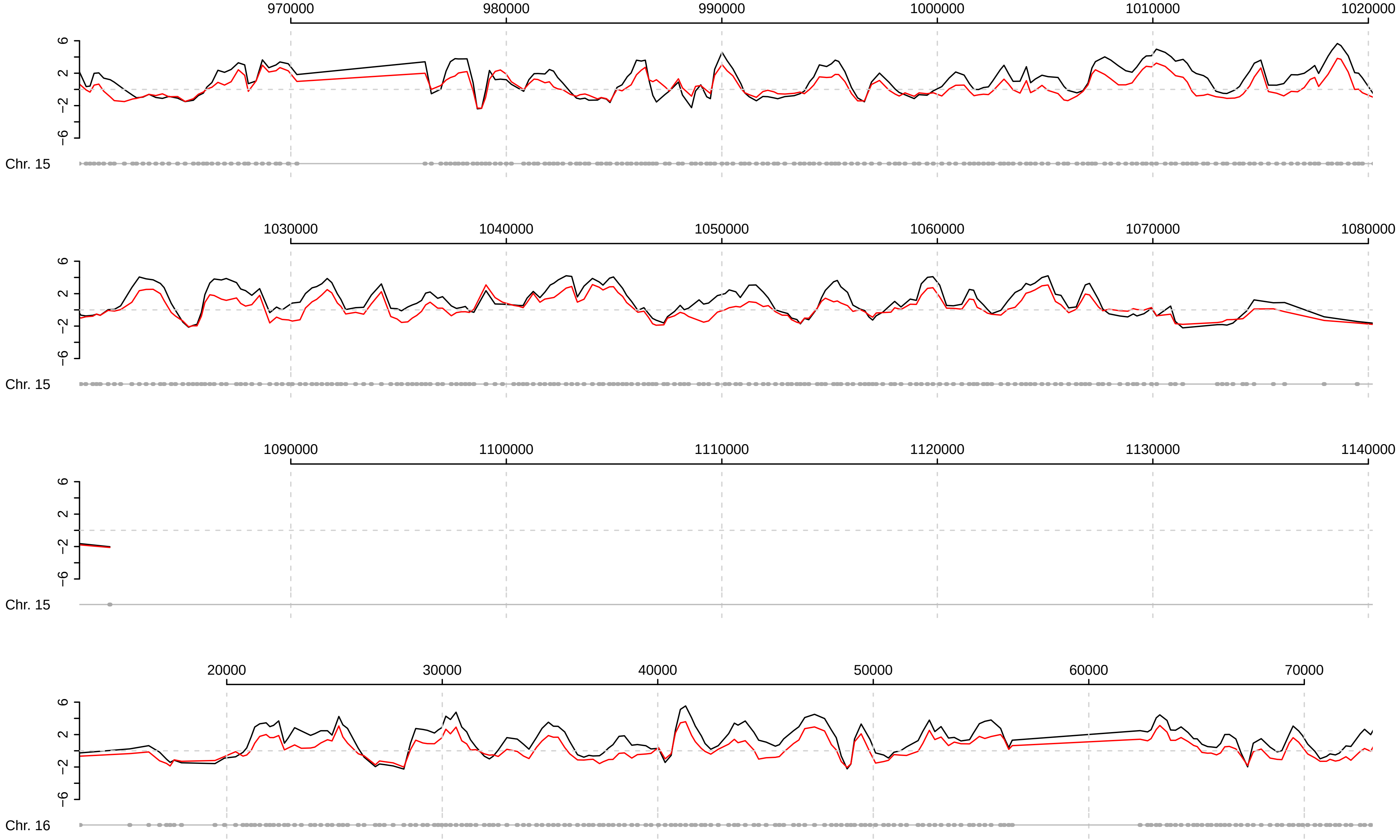
970000
980000
990000
1000000
1010000
1020000
6
2
−2
−6
Chr. 15
1030000
1040000
1050000
1060000
1070000
1080000
6
2
−2
−6
Chr. 15
1090000
1100000
1110000
1120000
1130000
1140000
6
2
−2
−6
Chr. 15
20000
30000
40000
50000
60000
70000
6
2
−2
−6
Chr. 16

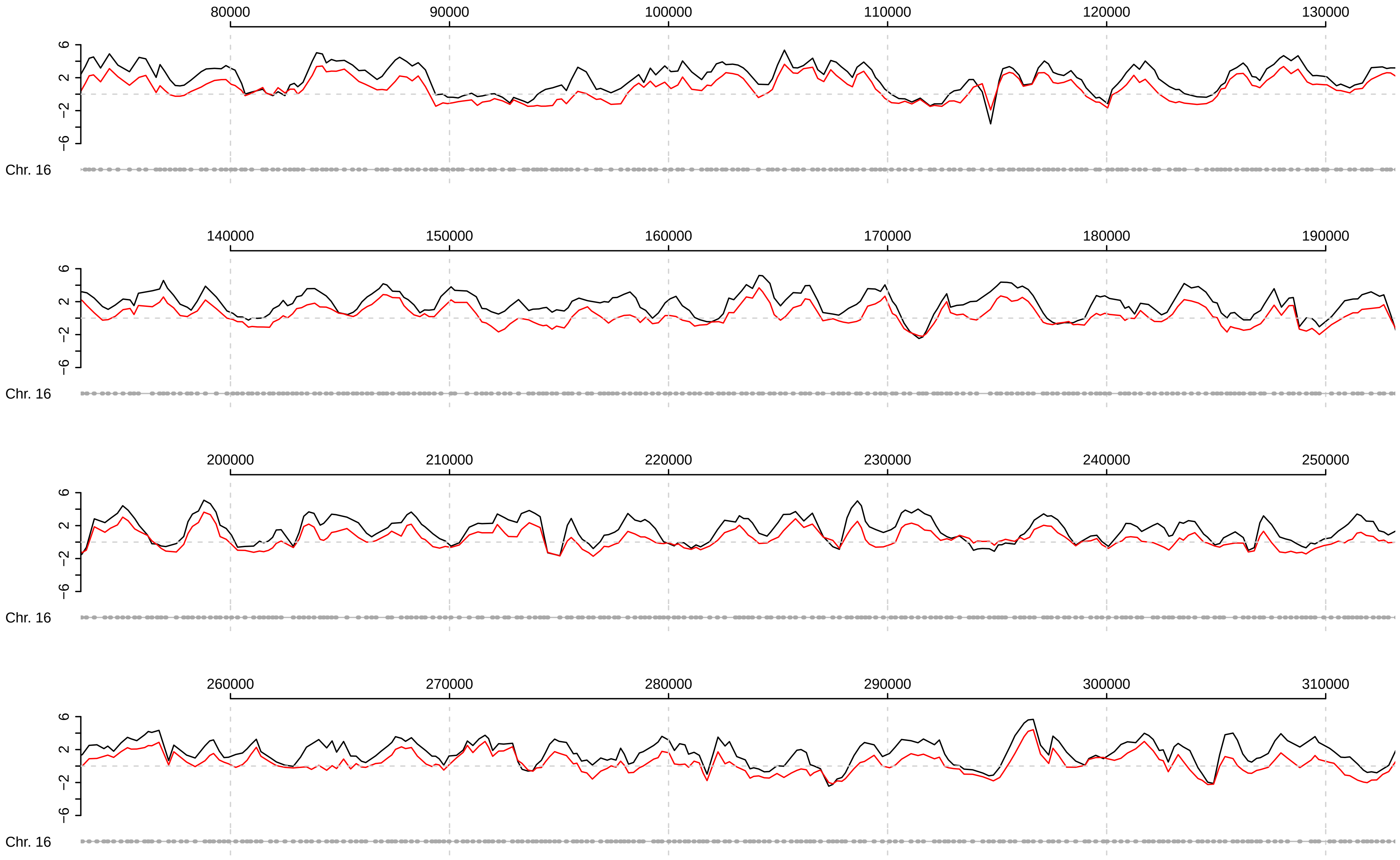

80000
90000
100000
110000
120000
130000
6
2
−2
−6
Chr. 16
140000
150000
160000
170000
180000
190000
6
2
−2
−6
Chr. 16
200000
210000
220000
230000
240000
250000
6
2
−2
−6
Chr. 16
260000
270000
280000
290000
300000
310000
6
2
−2
−6
Chr. 16

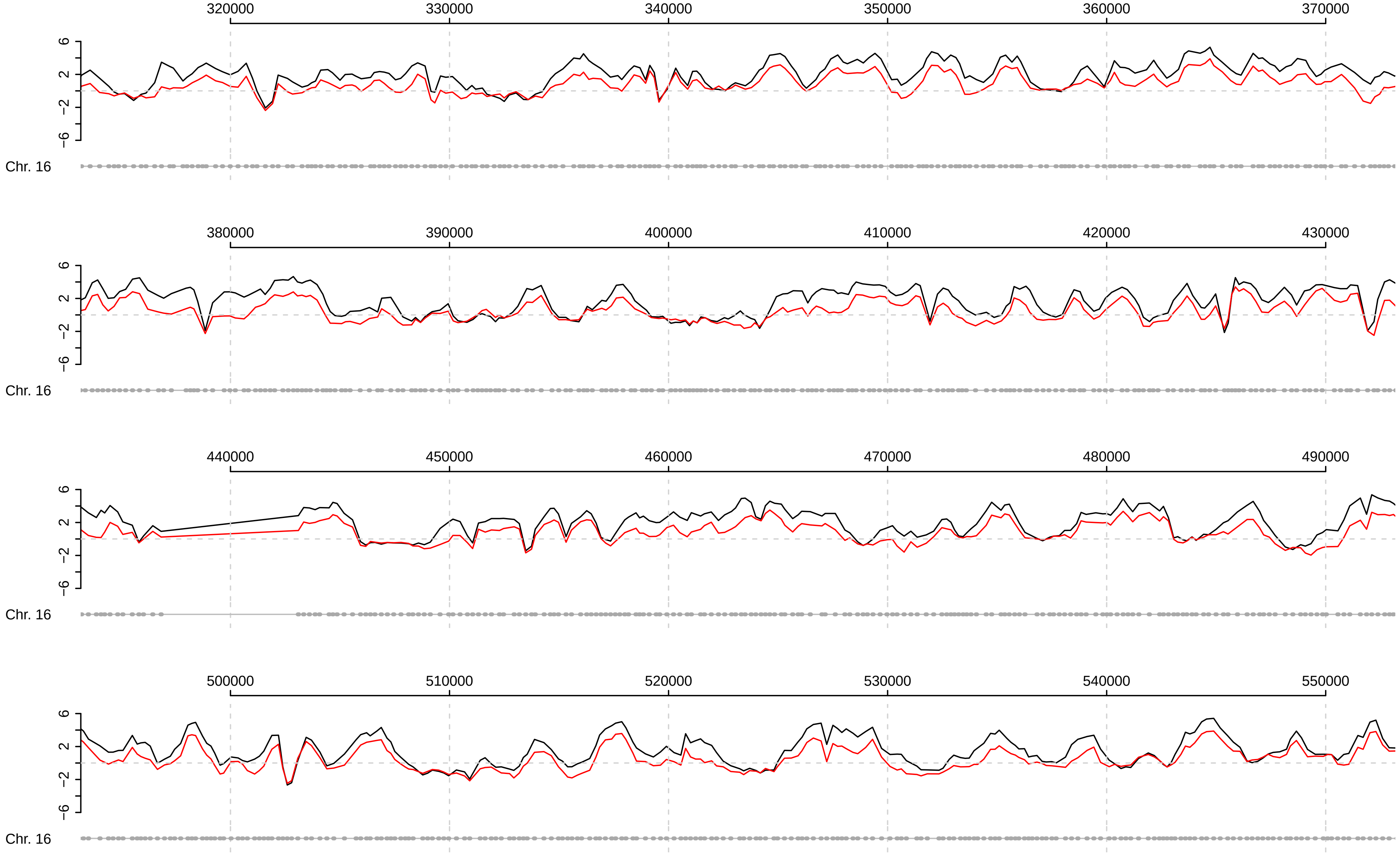

320000
330000
340000
350000
360000
370000
380000
390000
400000
410000
420000
430000
440000
450000
460000
470000
480000
490000
500000
510000
520000
530000
540000
550000
6
2
−2
−6
Chr. 16

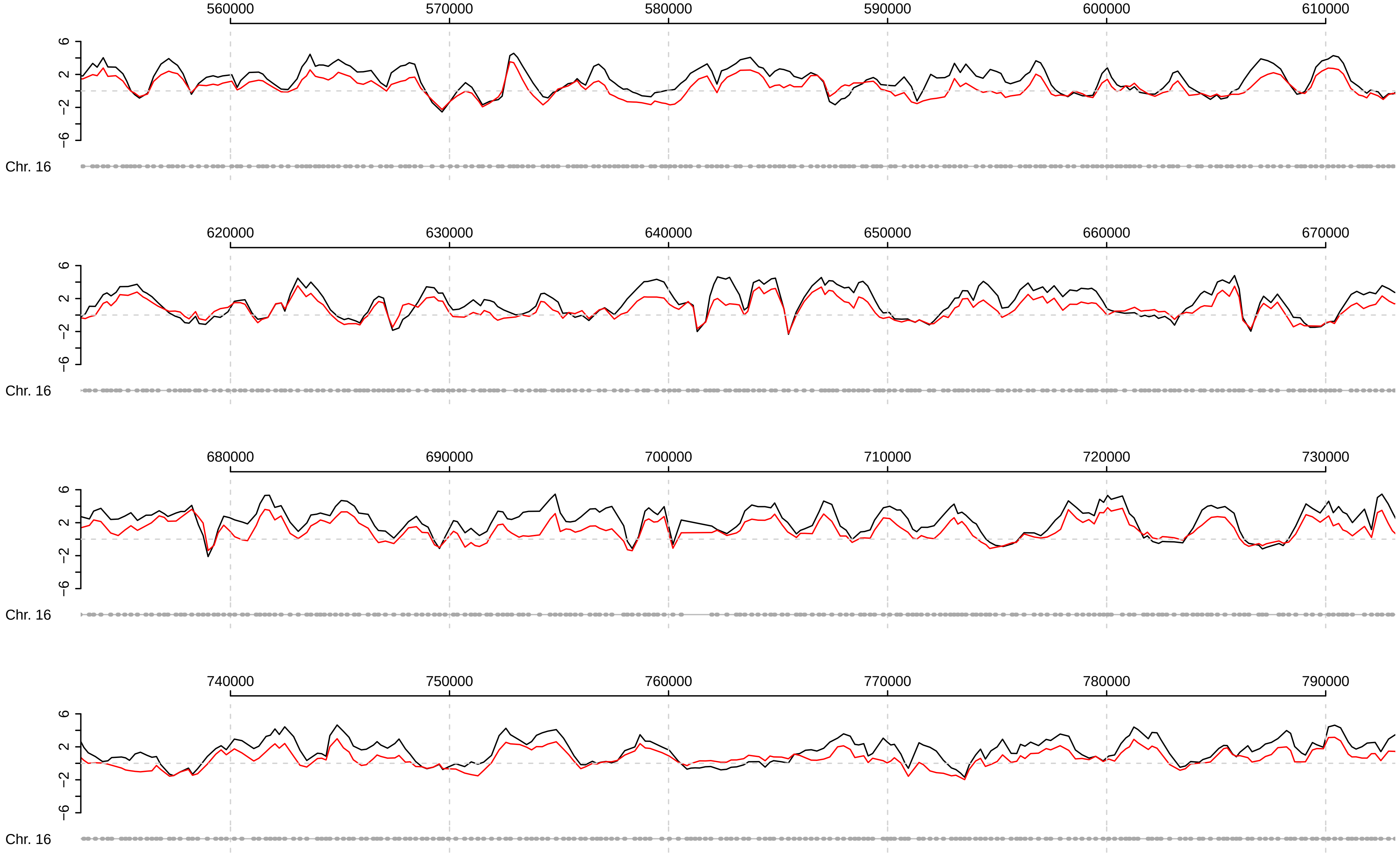
560000
570000
580000
590000
600000
610000
6
2
−2
−6
Chr. 16
620000
630000
640000
650000
660000
670000
6
2
−2
−6
Chr. 16
680000
690000
700000
710000
720000
730000
6
2
−2
−6
Chr. 16
740000
750000
760000
770000
780000
790000
6
2
−2
−6
Chr. 16

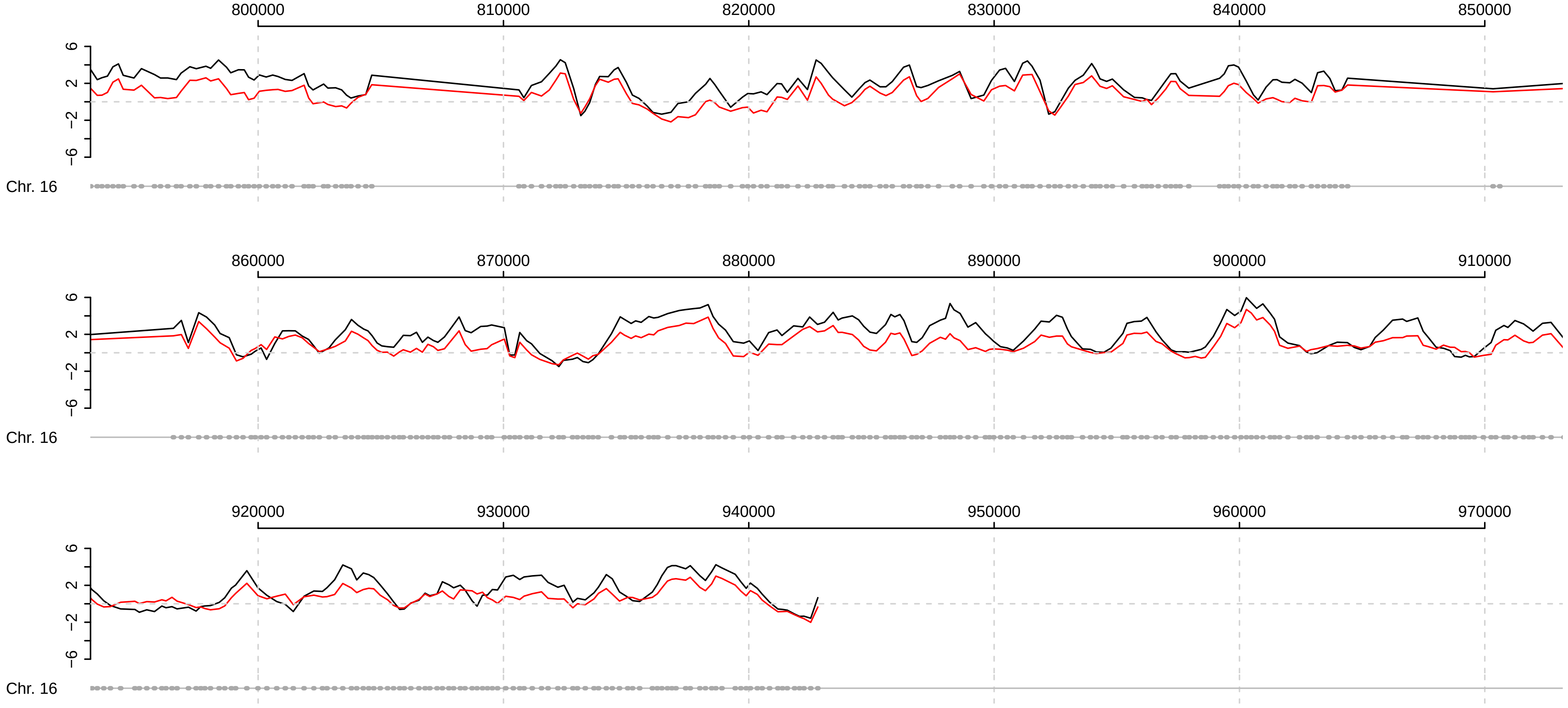
800000
810000
820000
830000
840000
850000
860000
870000
880000
890000
900000
910000
920000
930000
940000
950000
960000
970000
6
2
−2
−6
Chr. 16

The following plots show Abf1 enrichment over the whole genome in cells not treated with UV. The three line colours show three replicate datasets. The red crosses show probes detected as enriched.

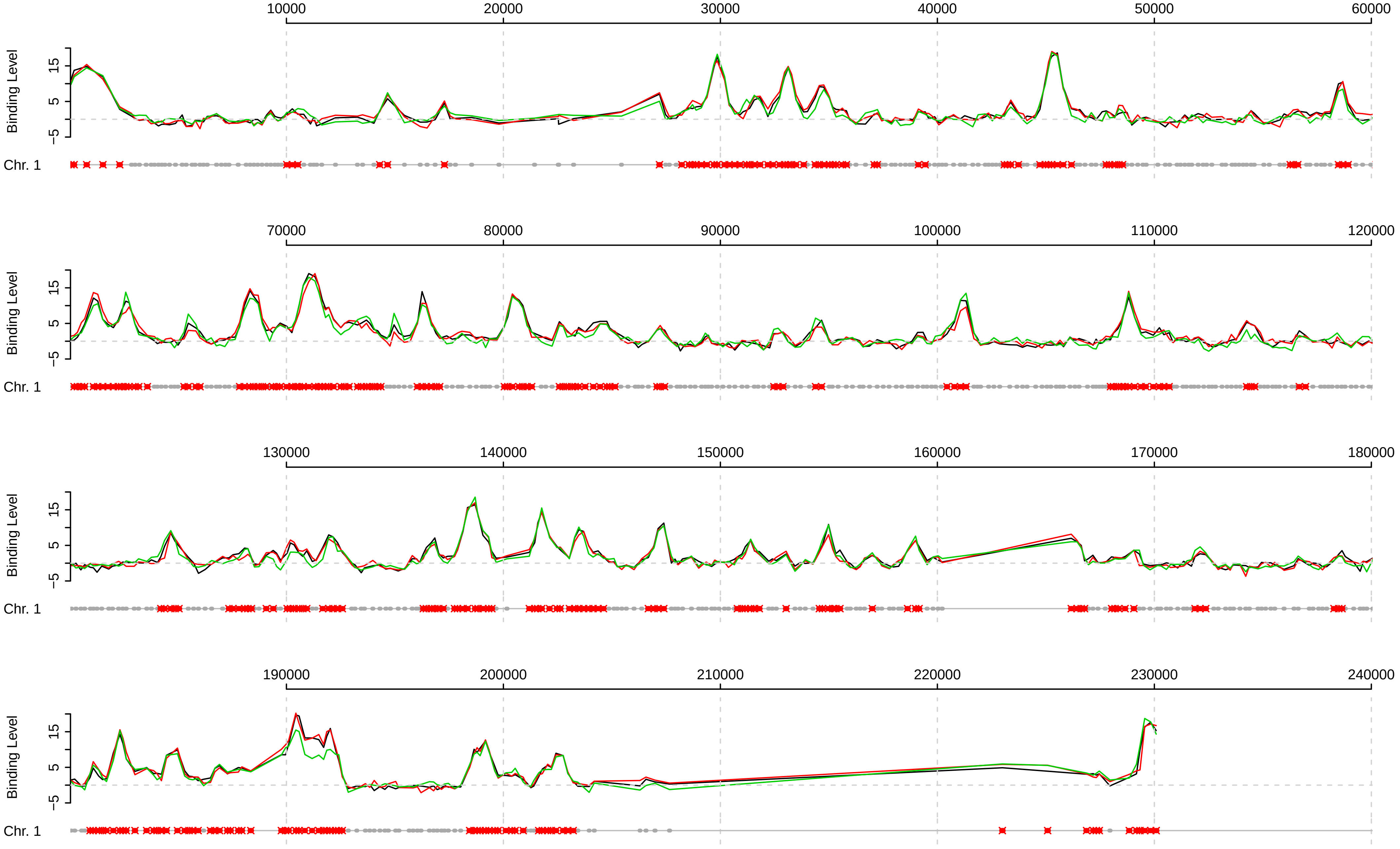

10000
20000
30000
40000
50000
60000
Binding Level
15
5
−5
Chr. 1
70000
80000
90000
100000
110000
120000
Binding Level
15
5
−5
Chr. 1
130000
140000
150000
160000
170000
180000
Binding Level
15
5
−5
Chr. 1
190000
200000
210000
220000
230000
240000
Binding Level
15
5
−5
Chr. 1

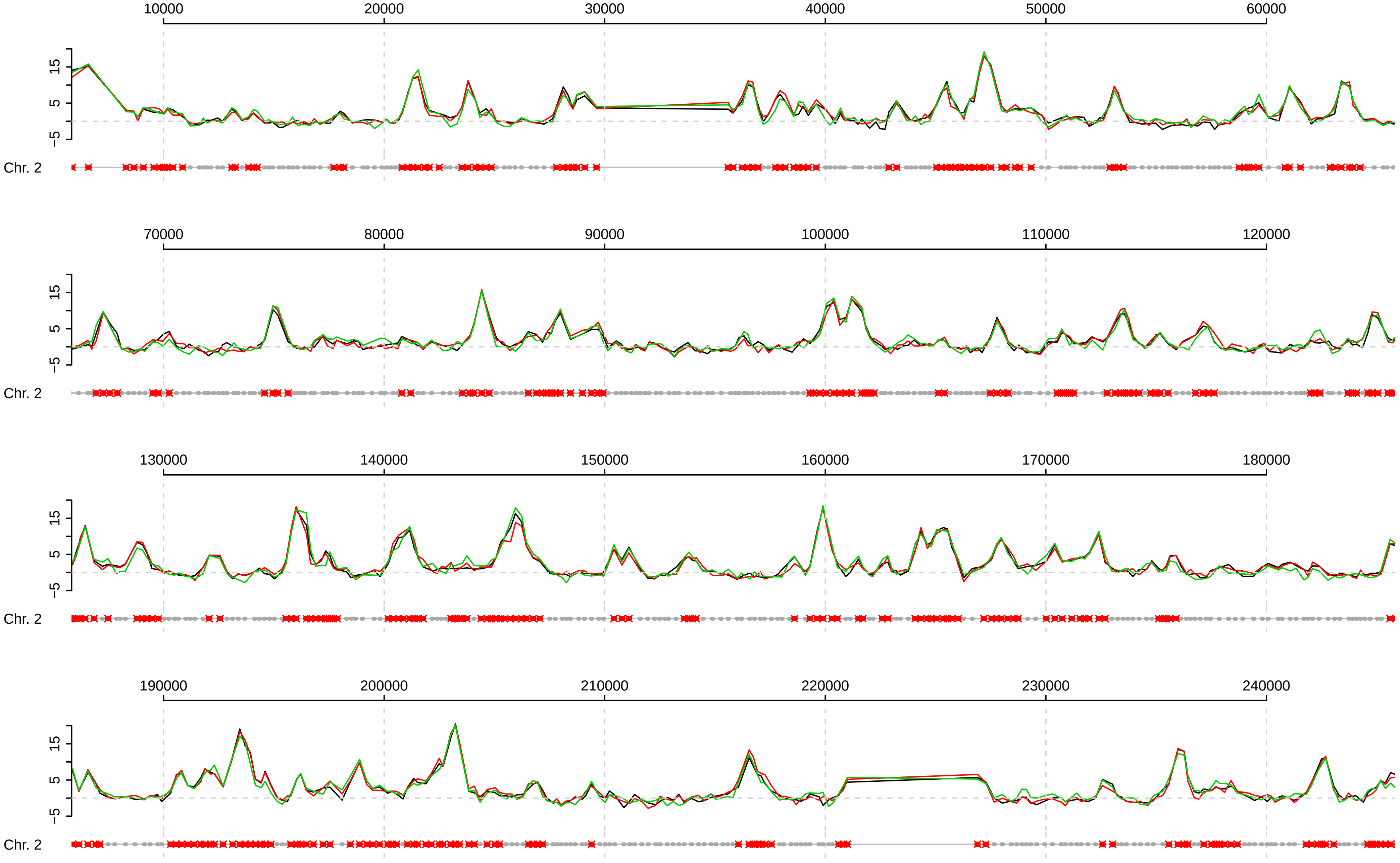
10000
20000
30000
40000
50000
60000
15
5
−5
Chr. 2
70000
80000
90000
100000
110000
120000
15
5
−5
Chr. 2
130000
140000
150000
160000
170000
180000
15
5
−5
Chr. 2
190000
200000
210000
220000
230000
240000
15
5
−5
Chr. 2

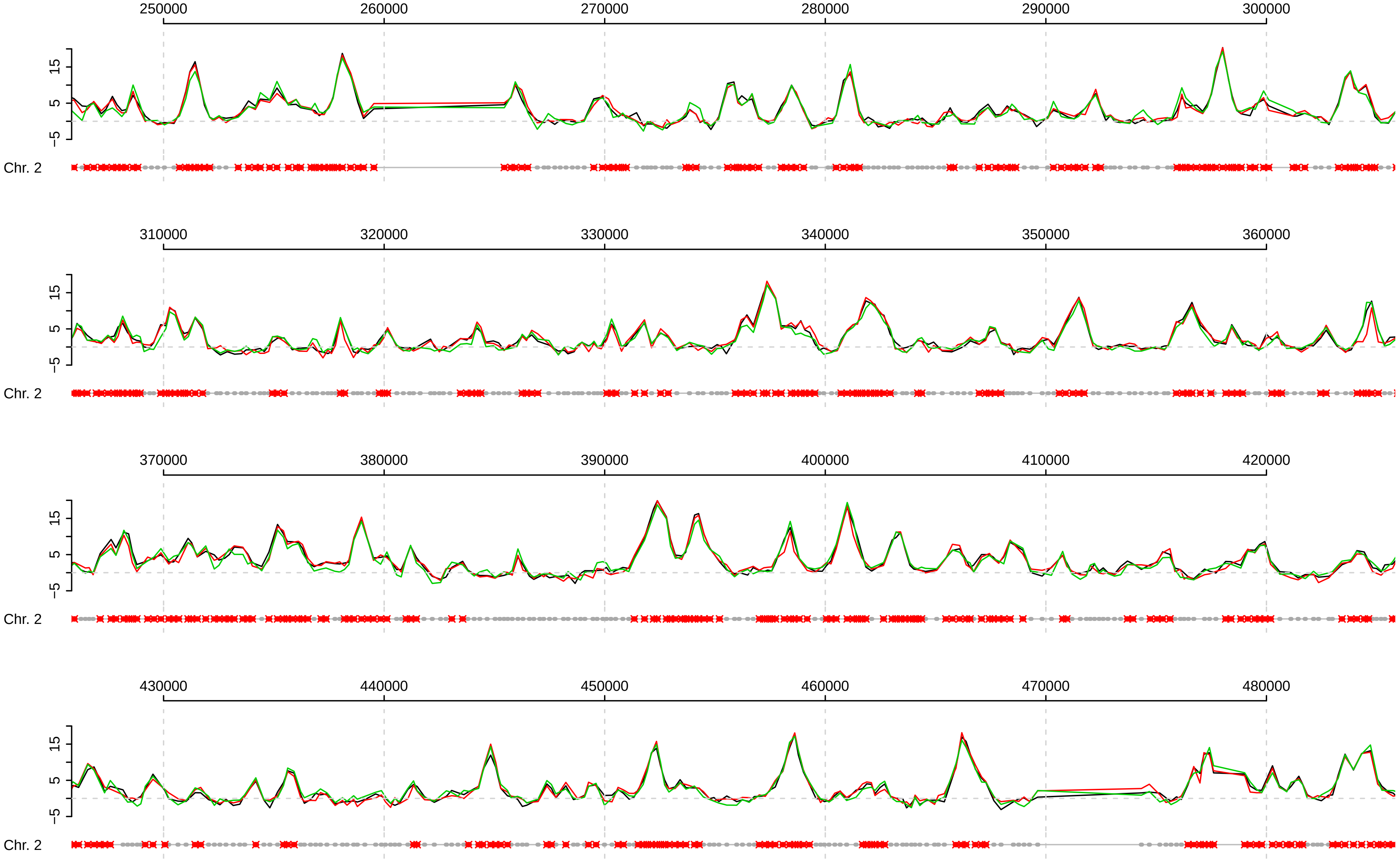
250000
260000
270000
280000
290000
300000
15
5
−5
Chr. 2
310000
320000
330000
340000
350000
360000
15
5
−5
Chr. 2
370000
380000
390000
400000
410000
420000
15
5
−5
Chr. 2
430000
440000
450000
460000
470000
480000
15
5
−5
Chr. 2

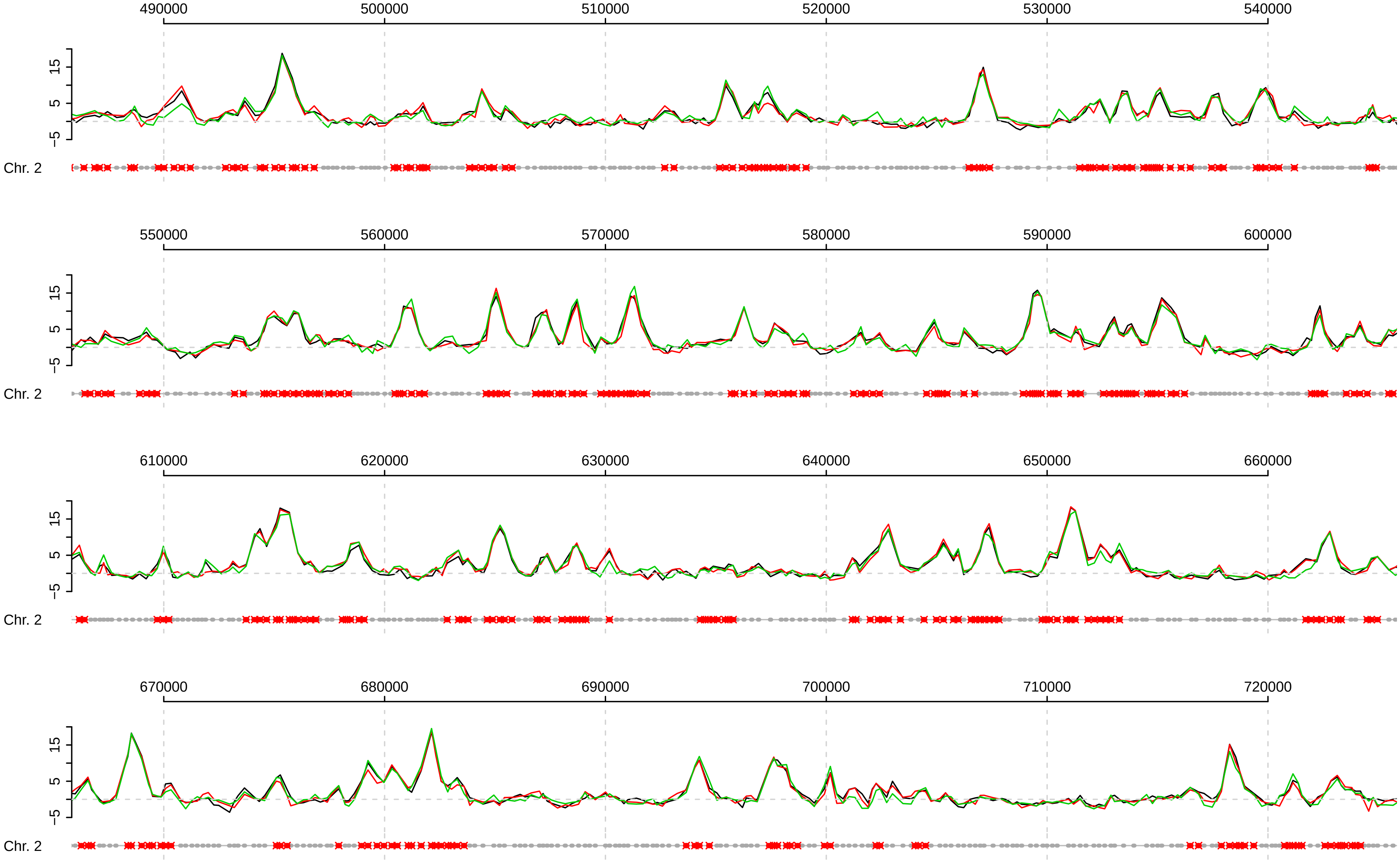
490000
500000
510000
520000
530000
540000
15
5
−5
Chr. 2
550000
560000
570000
580000
590000
600000
15
5
−5
Chr. 2
610000
620000
630000
640000
650000
660000
15
5
−5
Chr. 2
670000
680000
690000
700000
710000
720000
15
5
−5
Chr. 2

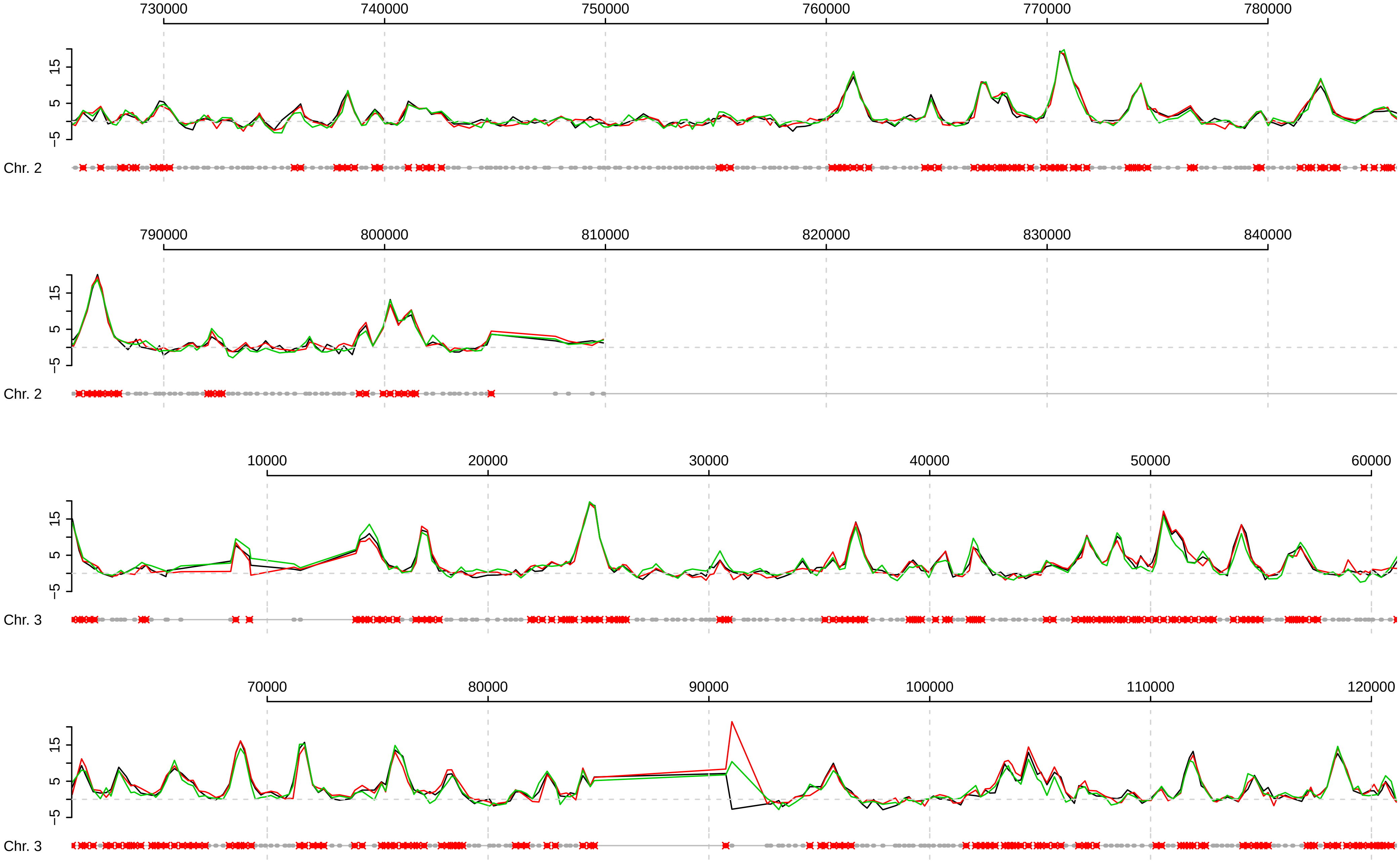

730000
740000
750000
760000
770000
780000
15
5
−5
Chr. 2
790000
800000
810000
820000
830000
840000
15
5
−5
Chr. 2
10000
20000
30000
40000
50000
60000
15
5
−5
Chr. 3
70000
80000
90000
100000
110000
120000
15
5
−5
Chr. 3

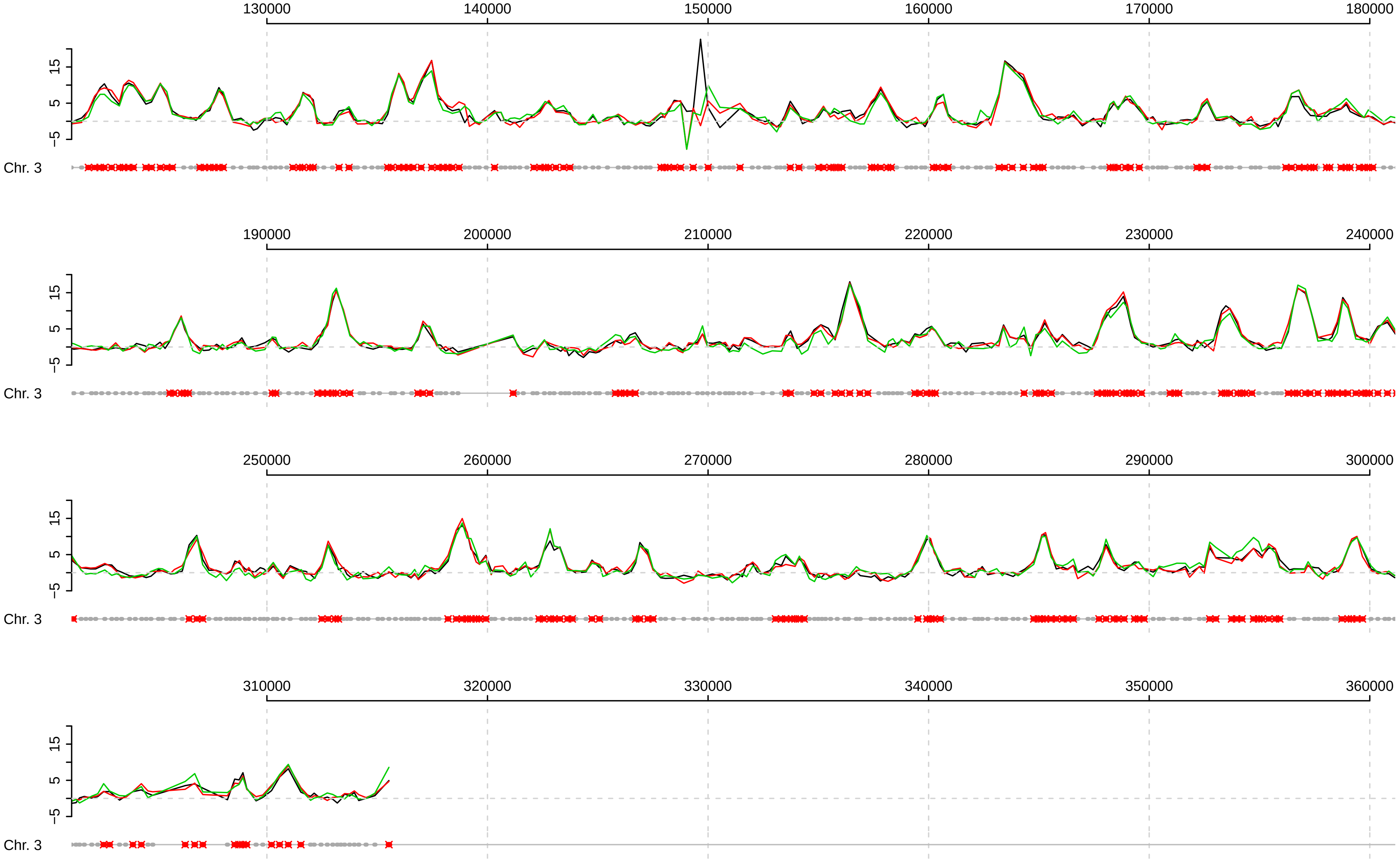
130000
140000
150000
160000
170000
180000
15
5
−5
Chr. 3
190000
200000
210000
220000
230000
240000
15
5
−5
Chr. 3
250000
260000
270000
280000
290000
300000
15
5
−5
Chr. 3
310000
320000
330000
340000
350000
360000
15
5
−5
Chr. 3

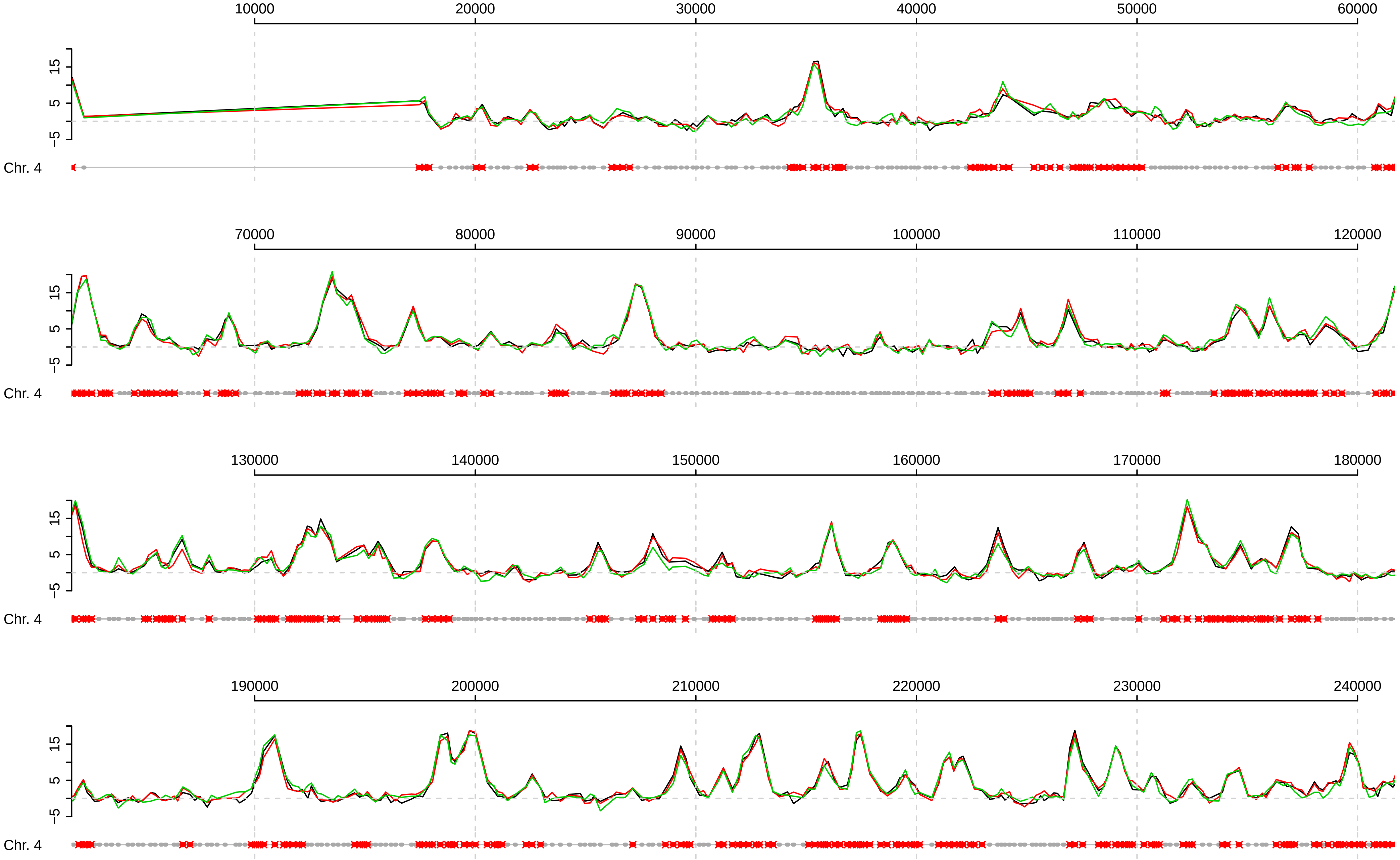
10000
20000
30000
40000
50000
60000
15
5
−5
Chr. 4
70000
80000
90000
100000
110000
120000
15
5
−5
Chr. 4
130000
140000
150000
160000
170000
180000
15
5
−5
Chr. 4
190000
200000
210000
220000
230000
240000
15
5
−5
Chr. 4

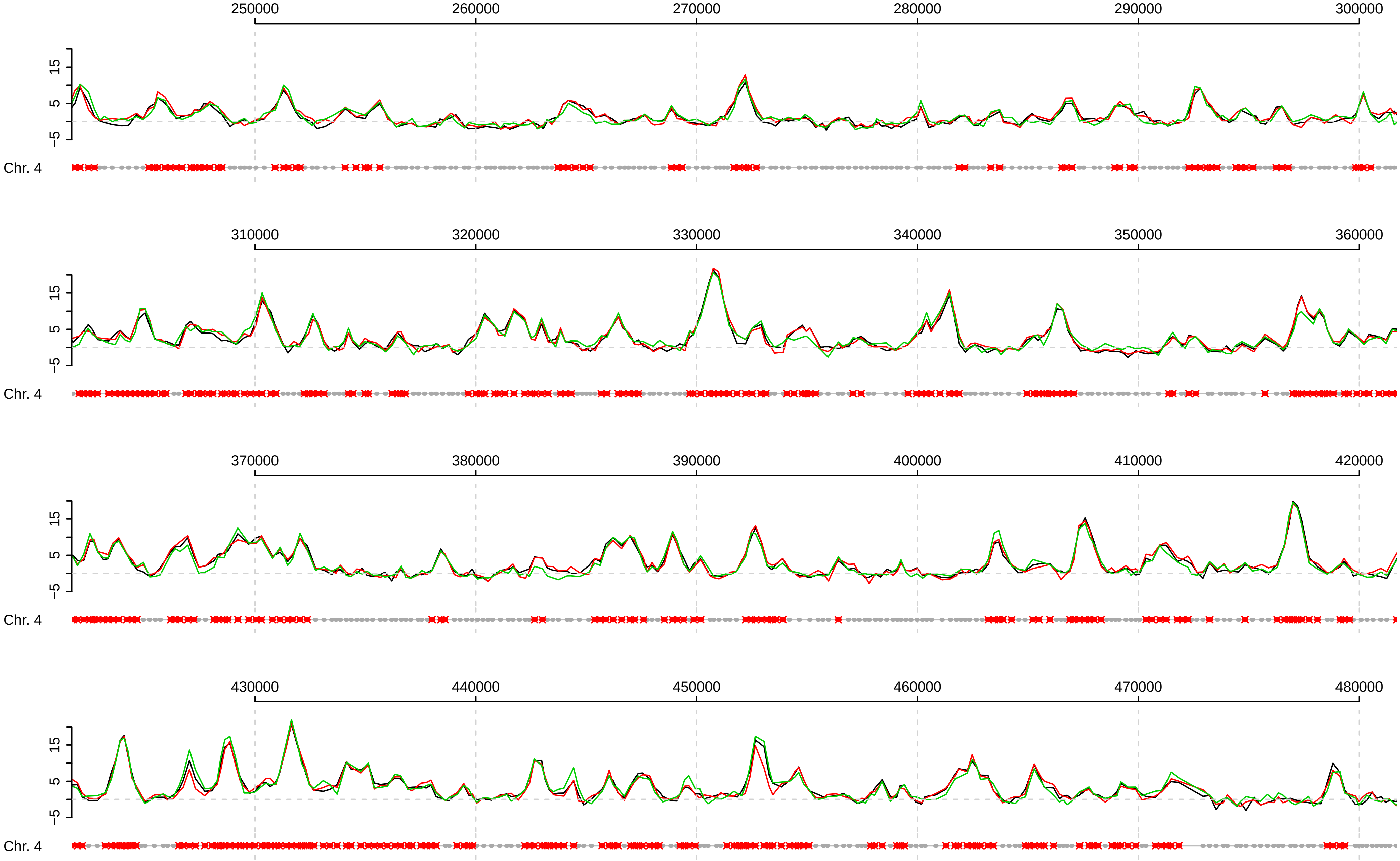
250000
260000
270000
280000
290000
300000
15
5
−5
Chr. 4
310000
320000
330000
340000
350000
360000
15
5
−5
Chr. 4
370000
380000
390000
400000
410000
420000
15
5
−5
Chr. 4
430000
440000
450000
460000
470000
480000
15
5
−5
Chr. 4

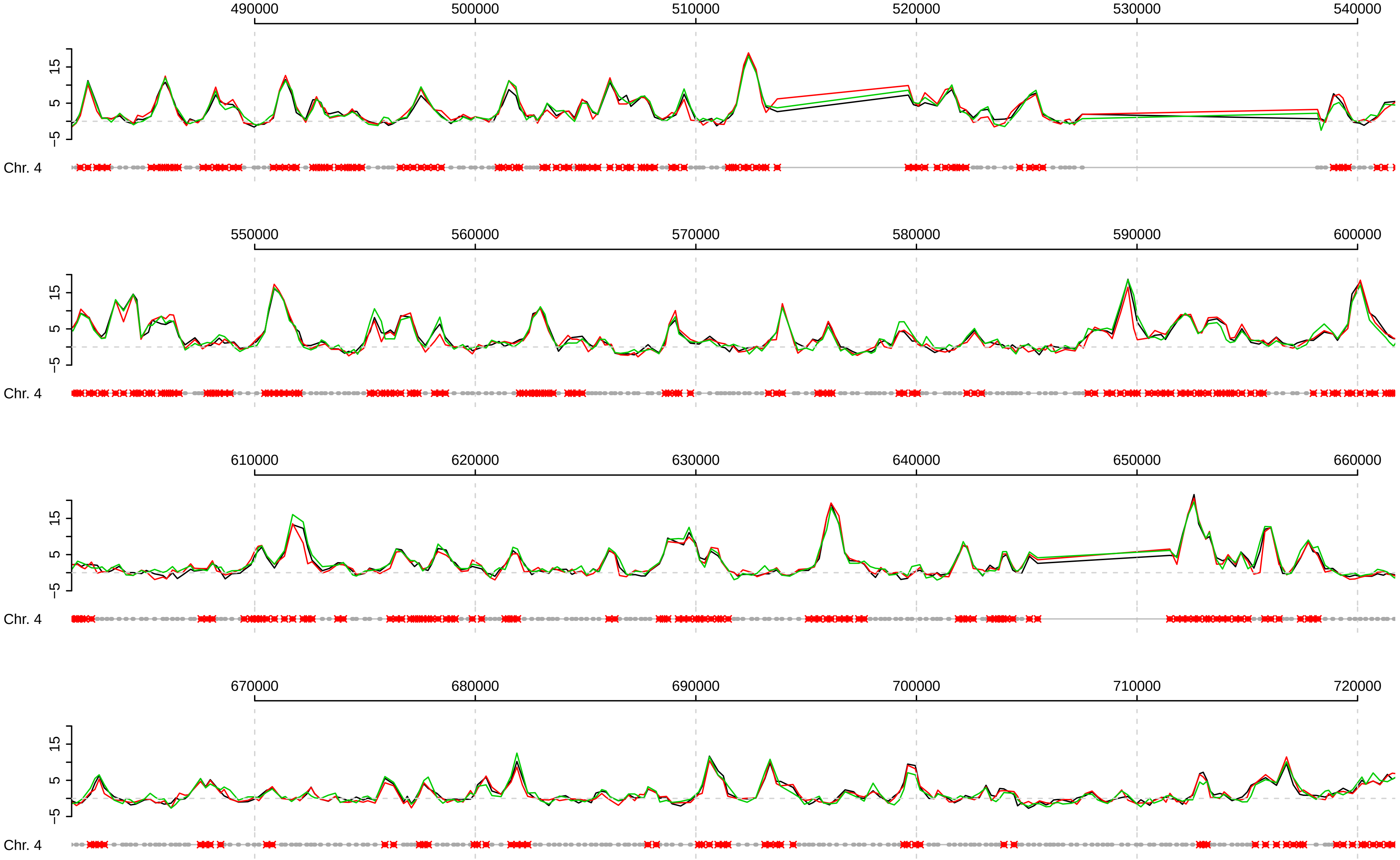

490000
500000
510000
520000
530000
540000
15
5
−5
Chr. 4
550000
560000
570000
580000
590000
600000
15
5
−5
Chr. 4
610000
620000
630000
640000
650000
660000
15
5
−5
Chr. 4
670000
680000
690000
700000
710000
720000
15
5
−5
Chr. 4

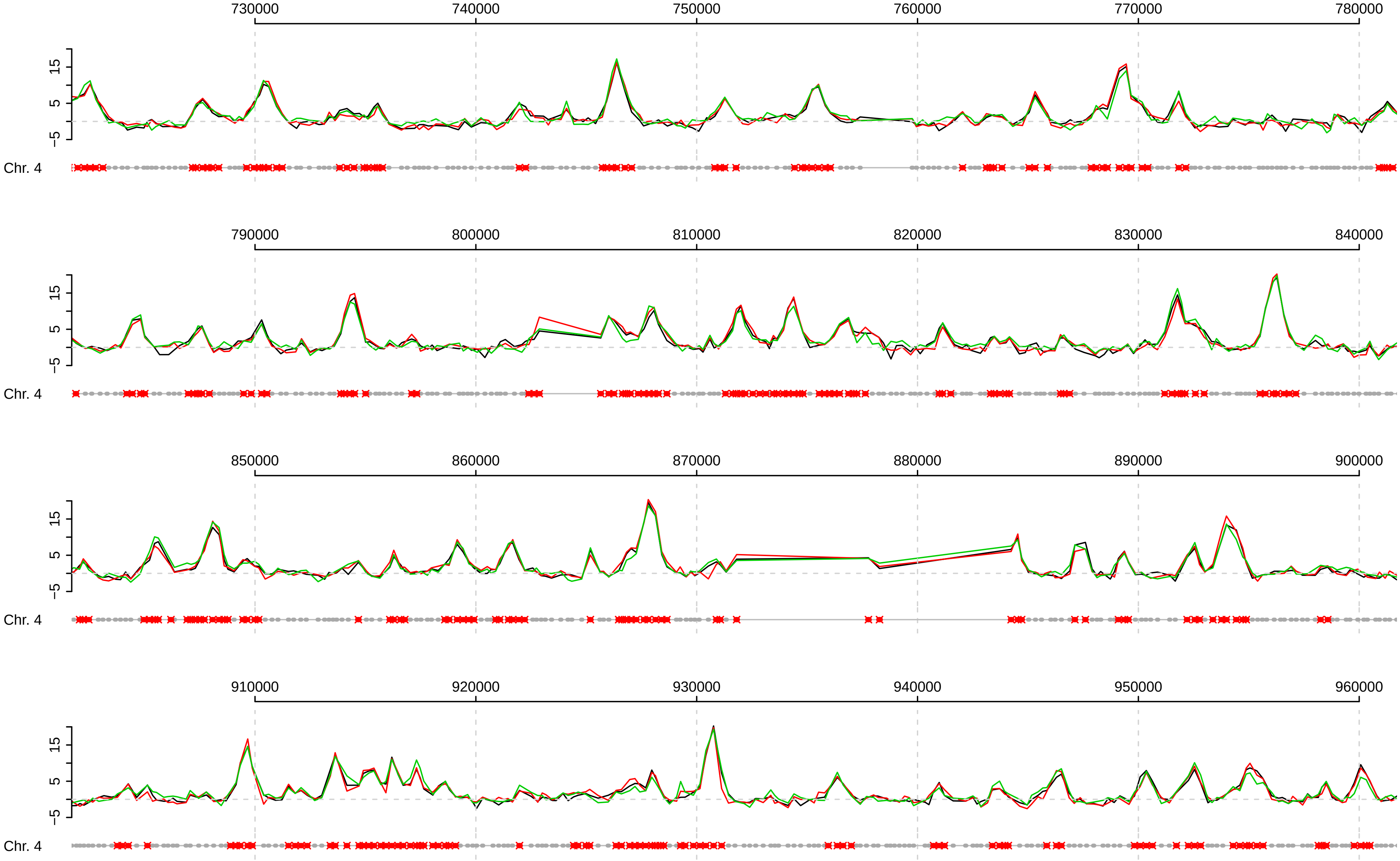

730000
740000
750000
760000
770000
780000
15
5
−5
Chr. 4
790000
800000
810000
820000
830000
840000
15
5
−5
Chr. 4
850000
860000
870000
880000
890000
900000
15
5
−5
Chr. 4
910000
920000
930000
940000
950000
960000
15
5
−5
Chr. 4

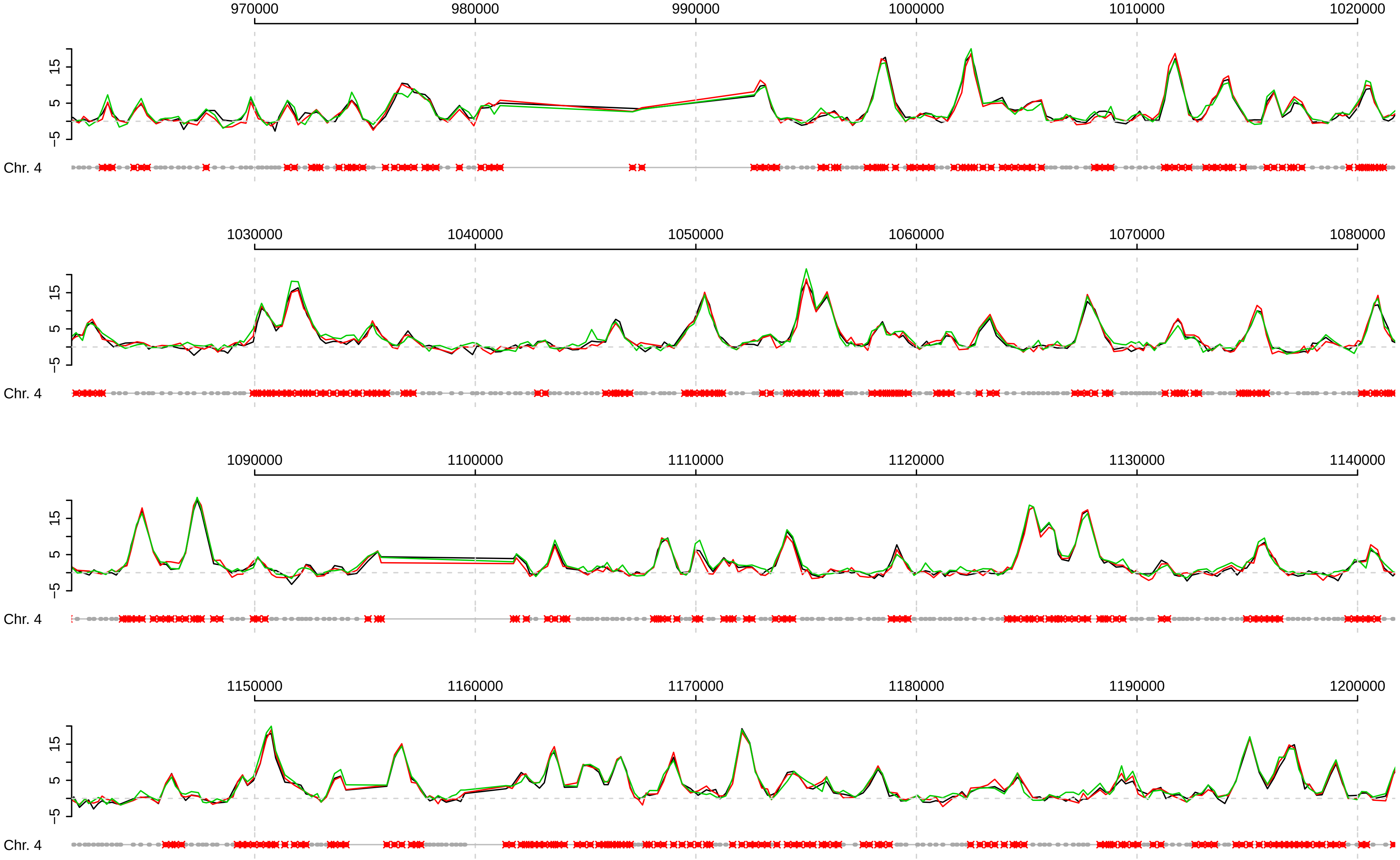

970000
980000
990000
1000000
1010000
1020000
15
5
−5
Chr. 4
1030000
1040000
1050000
1060000
1070000
1080000
15
5
−5
Chr. 4
1090000
1100000
1110000
1120000
1130000
1140000
15
5
−5
Chr. 4
1150000
1160000
1170000
1180000
1190000
1200000
15
5
−5
Chr. 4

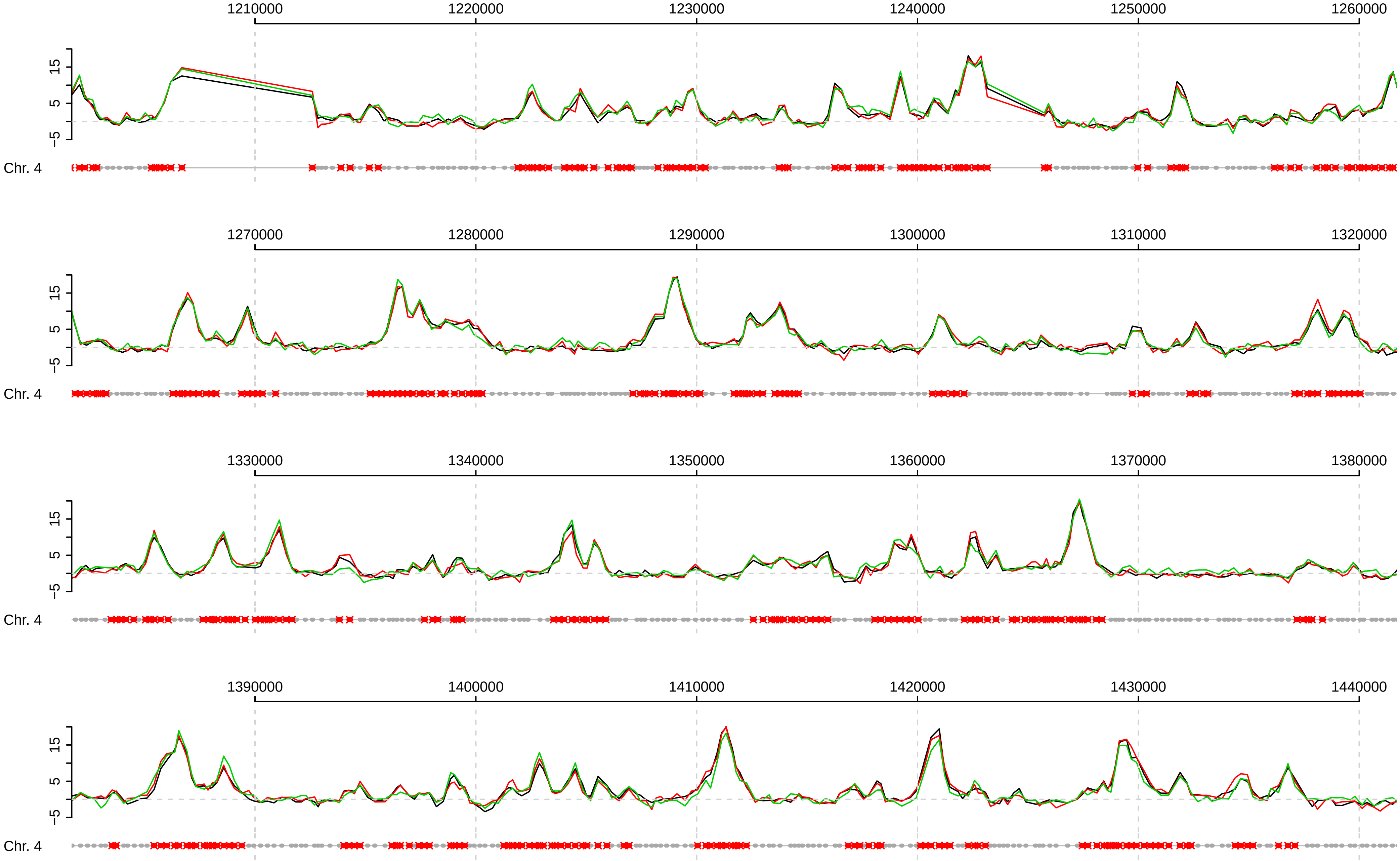

1210000
1220000
1230000
1240000
1250000
1260000
15
5
−5
Chr. 4
1270000
1280000
1290000
1300000
1310000
1320000
15
5
−5
Chr. 4
1330000
1340000
1350000
1360000
1370000
1380000
15
5
−5
Chr. 4
1390000
1400000
1410000
1420000
1430000
1440000
15
5
−5
Chr. 4

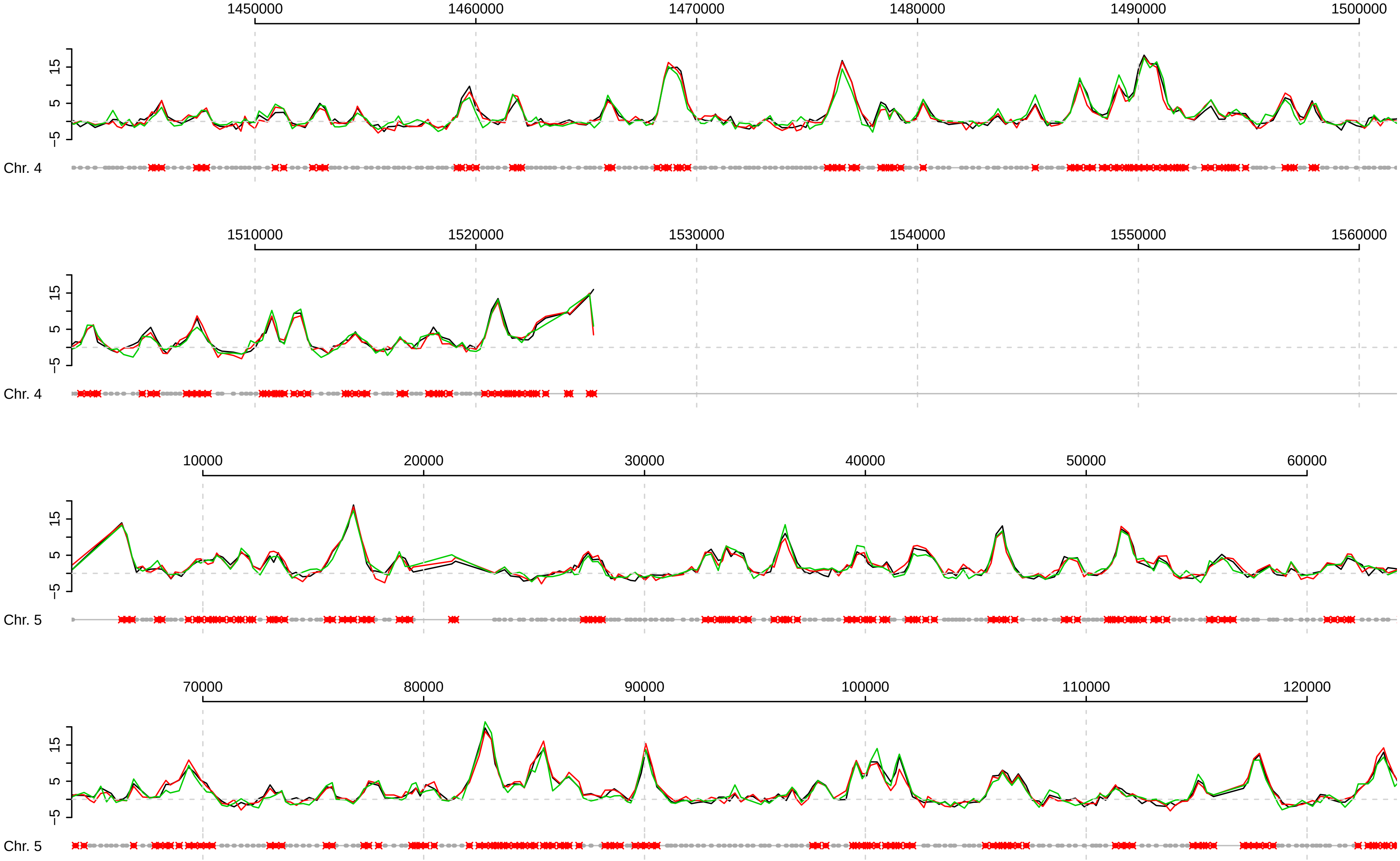

1450000
1460000
1470000
1480000
1490000
1500000
15
5
−5
Chr. 4
1510000
1520000
1530000
1540000
1550000
1560000
15
5
−5
Chr. 4
10000
20000
30000
40000
50000
60000
15
5
−5
Chr. 5
70000
80000
90000
100000
110000
120000
15
5
−5
Chr. 5

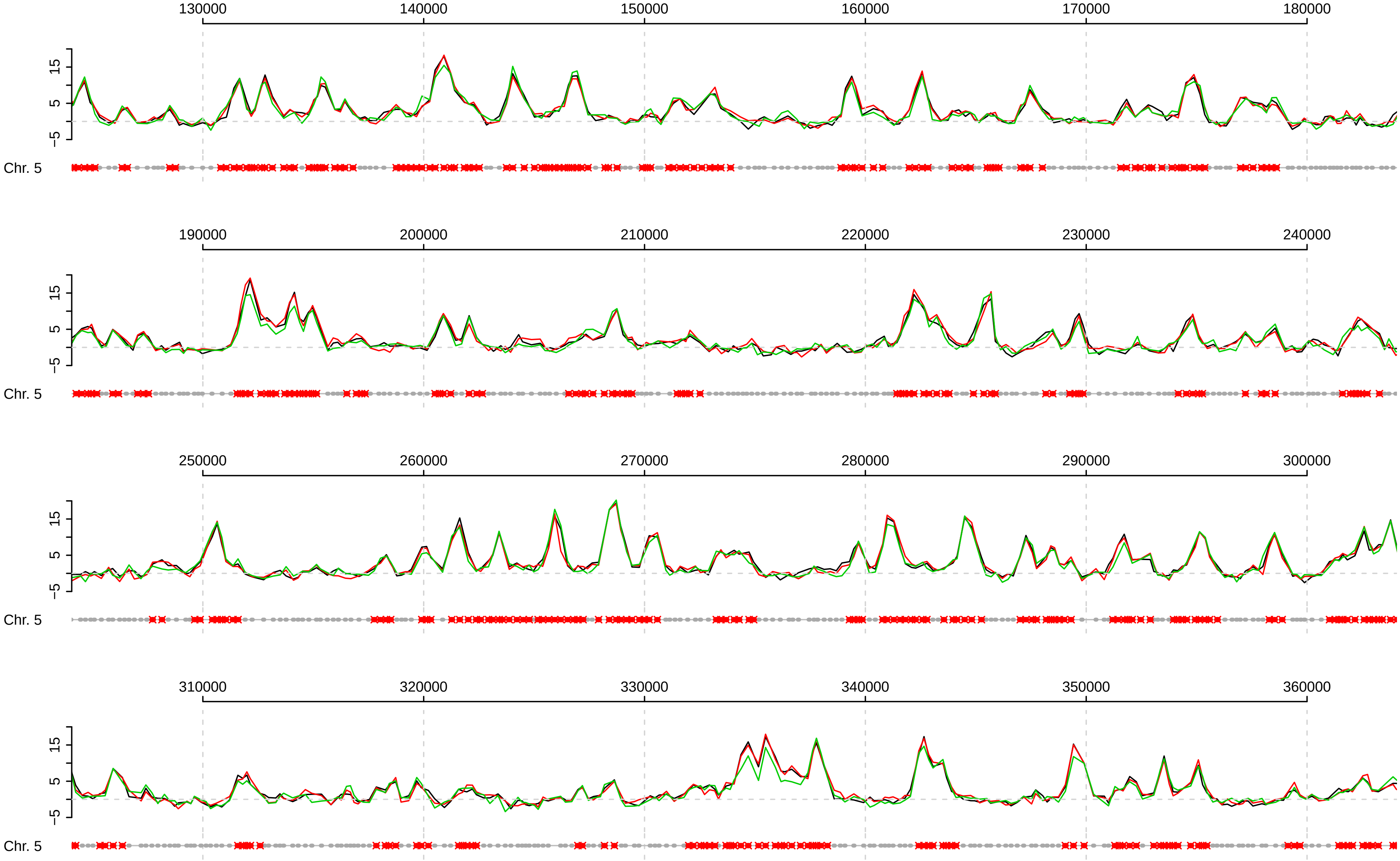

130000
140000
150000
160000
170000
180000
15
5
−5
Chr. 5
190000
200000
210000
220000
230000
240000
15
5
−5
Chr. 5
250000
260000
270000
280000
290000
300000
15
5
−5
Chr. 5
310000
320000
330000
340000
350000
360000
15
5
−5
Chr. 5

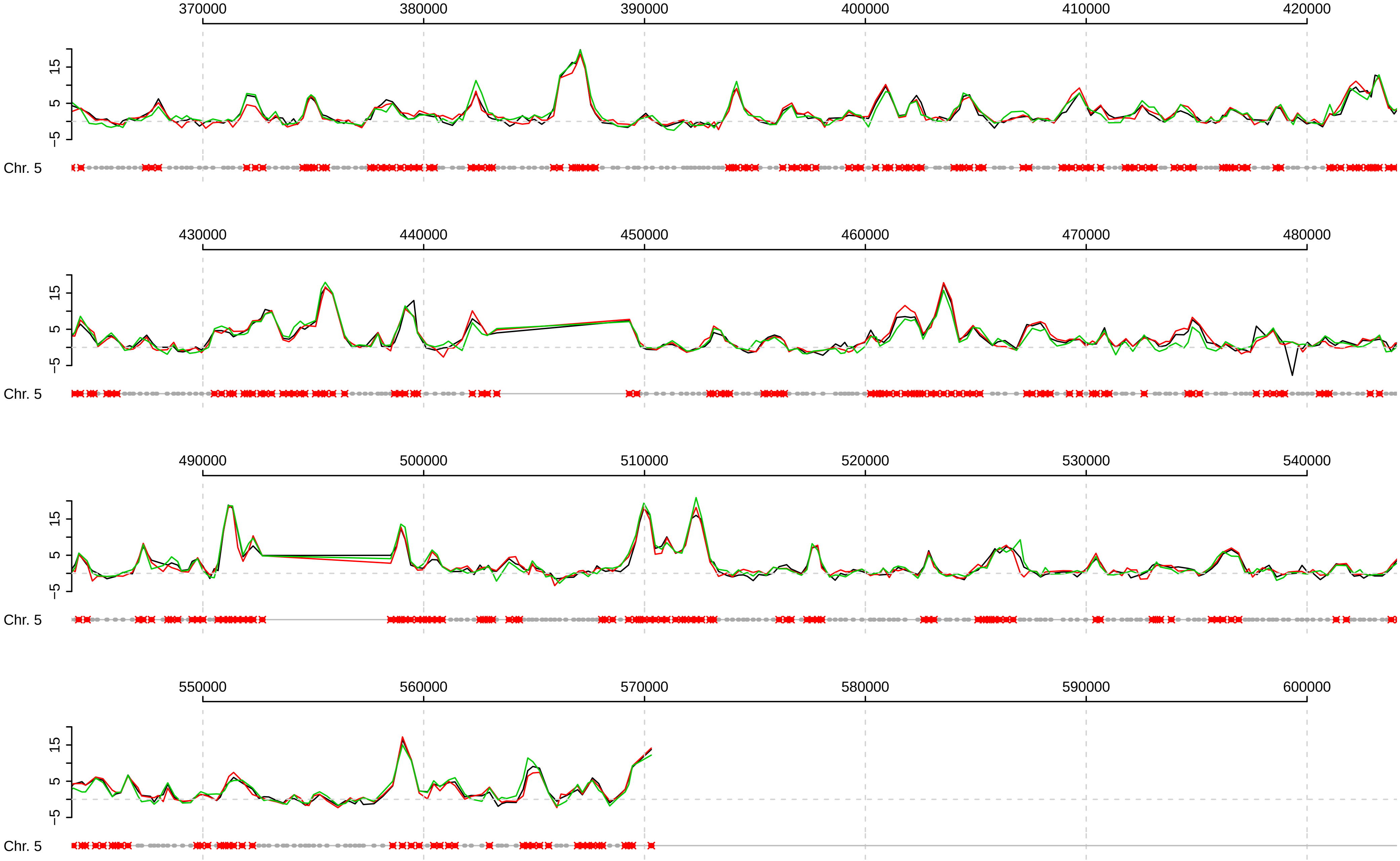
370000
380000
390000
400000
410000
420000
15
5
−5
Chr. 5
430000
440000
450000
460000
470000
480000
15
5
−5
Chr. 5
490000
500000
510000
520000
530000
540000
15
5
−5
Chr. 5
550000
560000
570000
580000
590000
600000
15
5
−5
Chr. 5

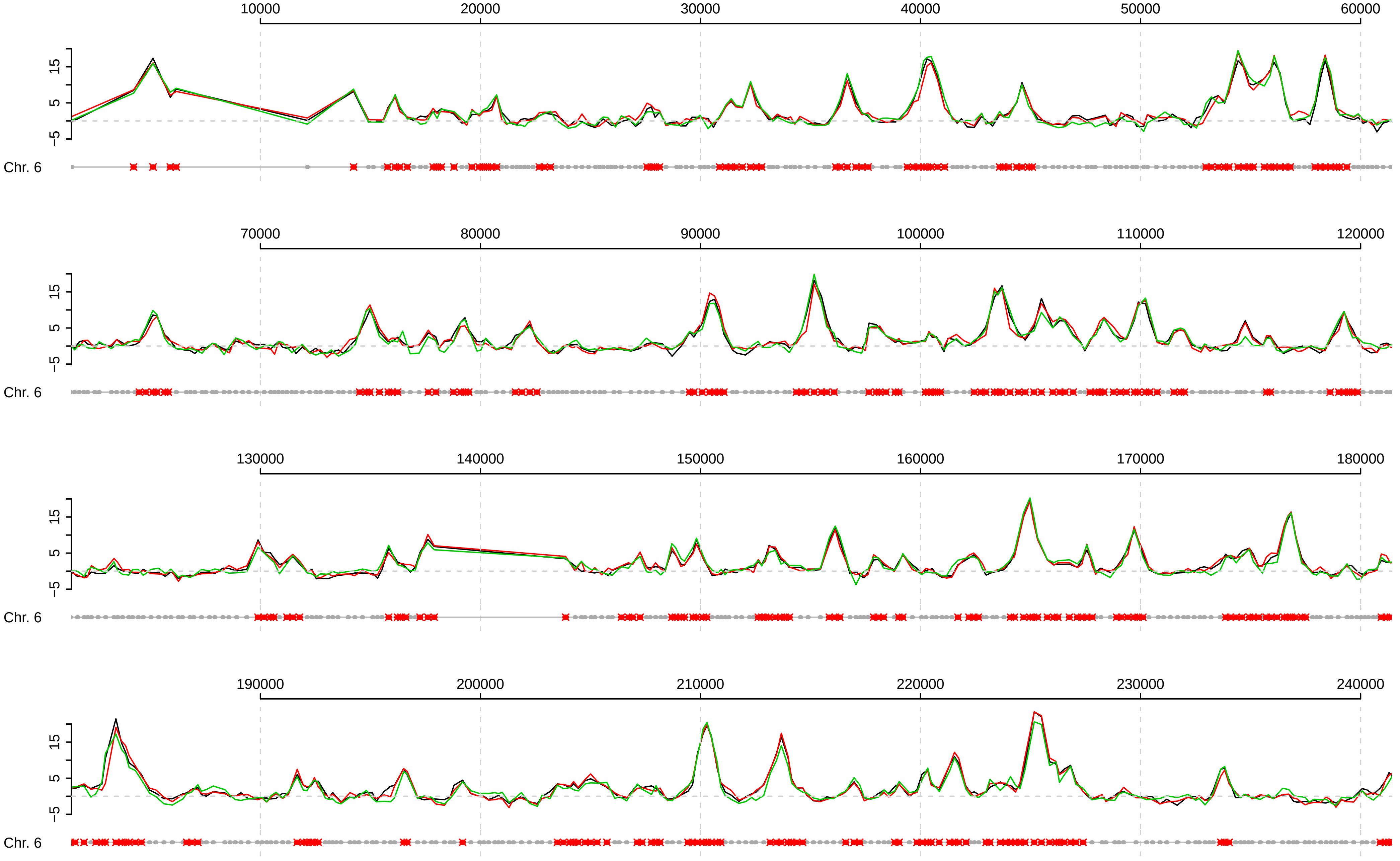

10000
20000
30000
40000
50000
60000
70000
80000
90000
100000
110000
120000
130000
140000
150000
160000
170000
180000
190000
200000
210000
220000
230000
240000
−5
5
15
Chr. 6

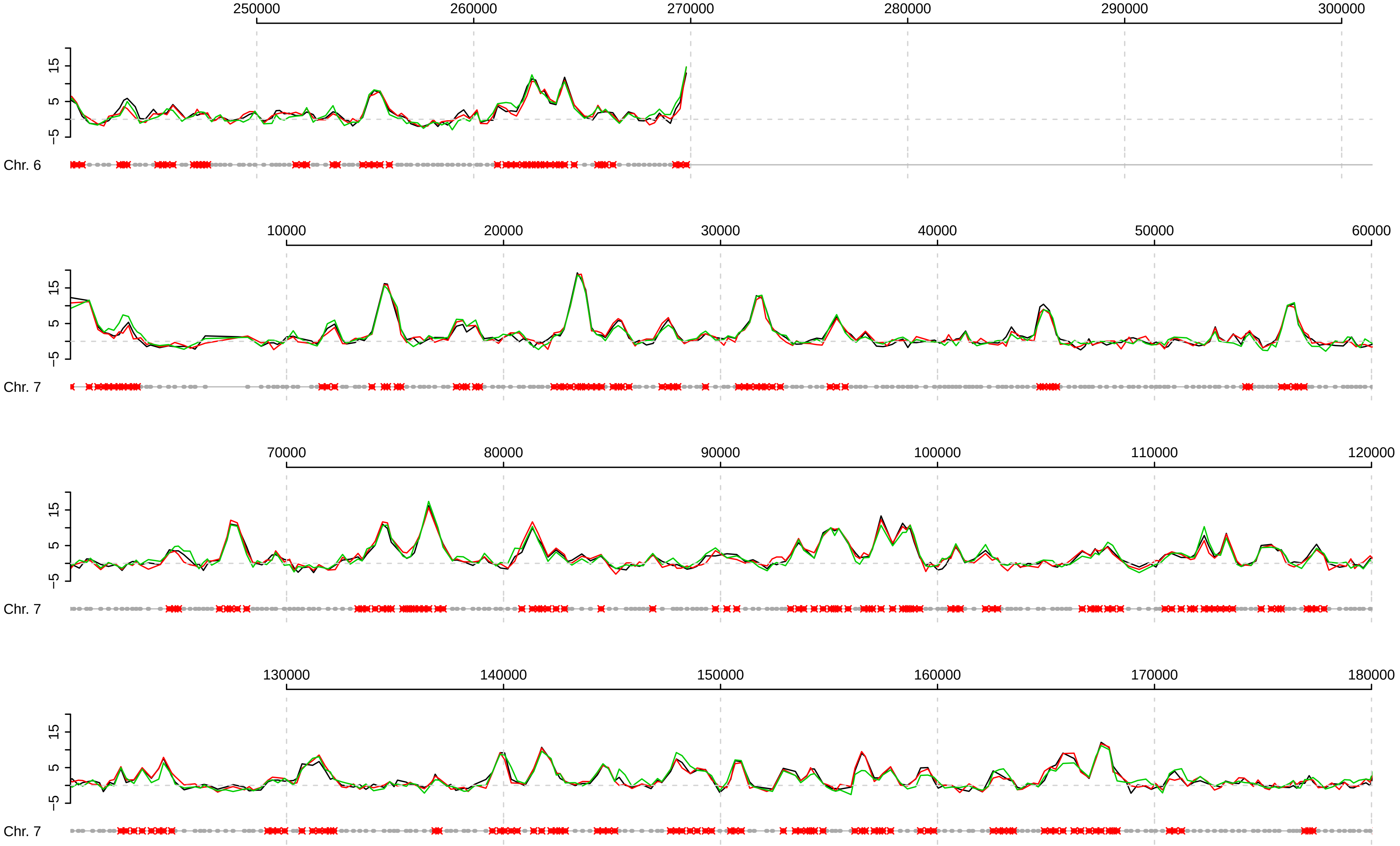

250000
260000
270000
280000
290000
300000
15
5
−5
Chr. 6
10000
20000
30000
40000
50000
60000
15
5
−5
Chr. 7
70000
80000
90000
100000
110000
120000
15
5
−5
Chr. 7
130000
140000
150000
160000
170000
180000
15
5
−5
Chr. 7

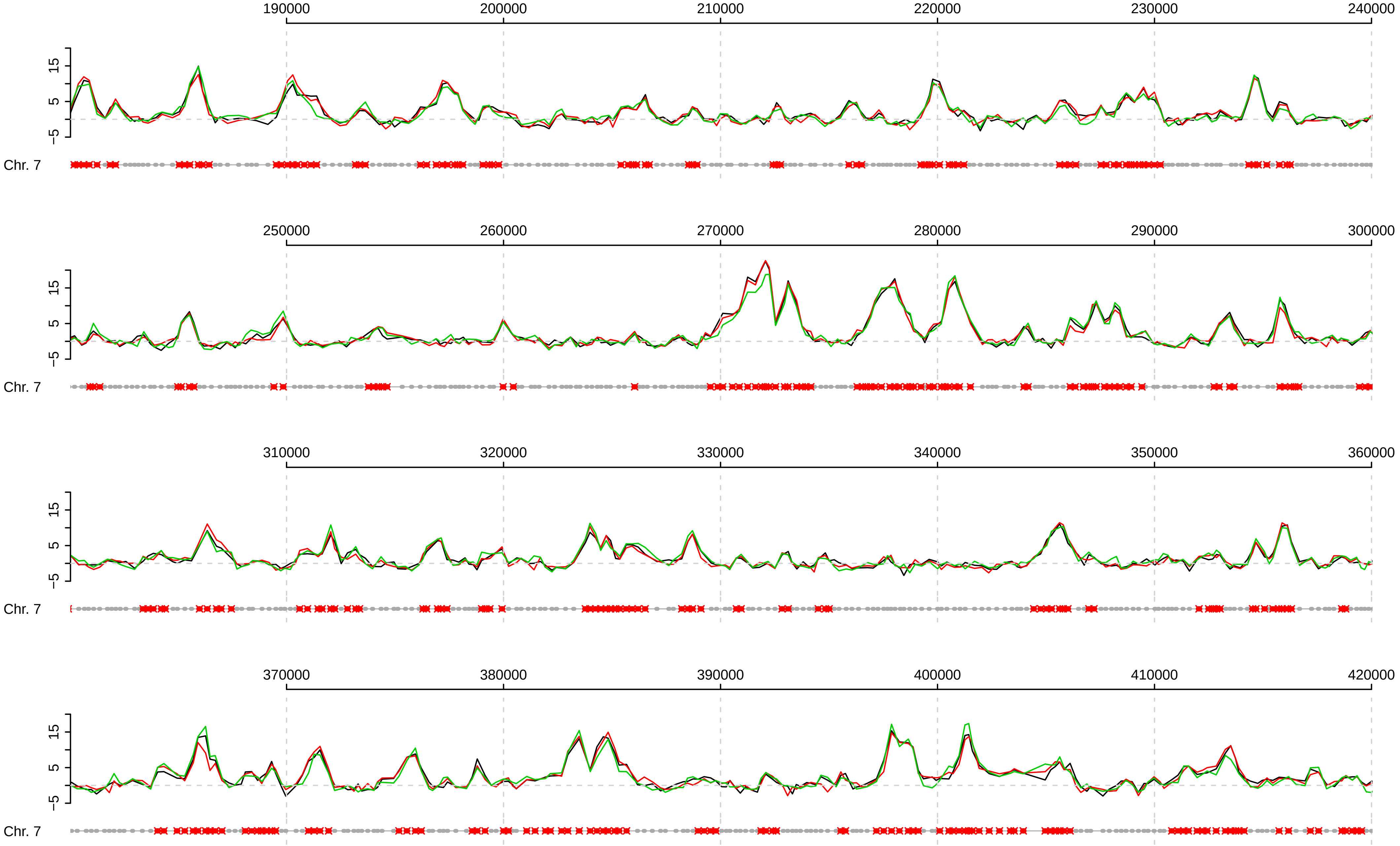
190000
200000
210000
220000
230000
240000
15
5
−5
Chr. 7
250000
260000
270000
280000
290000
300000
15
5
−5
Chr. 7
310000
320000
330000
340000
350000
360000
15
5
−5
Chr. 7
370000
380000
390000
400000
410000
420000
15
5
−5
Chr. 7

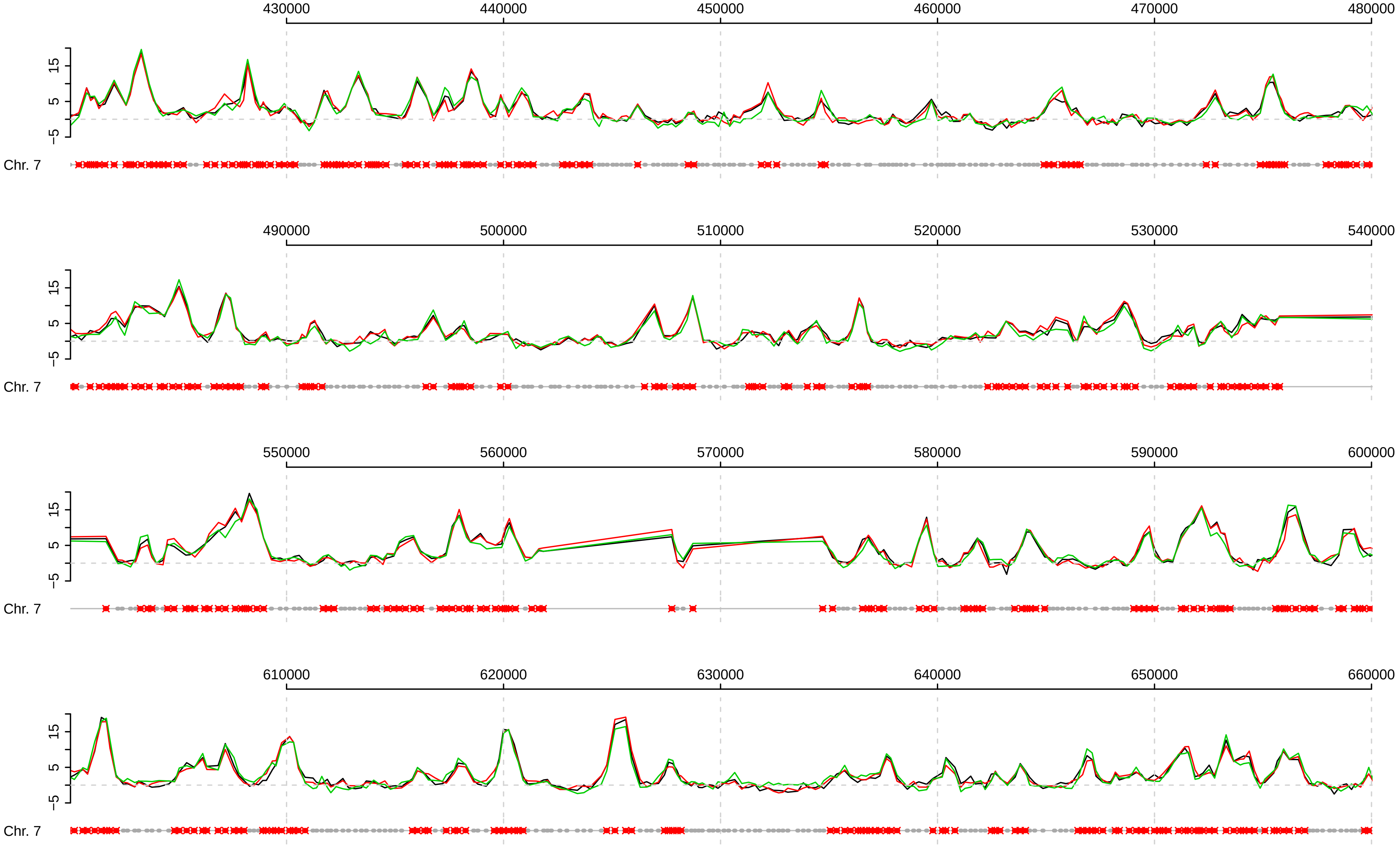

430000
440000
450000
460000
470000
480000
15
5
−5
Chr. 7
490000
500000
510000
520000
530000
540000
15
5
−5
Chr. 7
550000
560000
570000
580000
590000
600000
15
5
−5
Chr. 7
610000
620000
630000
640000
650000
660000
15
5
−5
Chr. 7

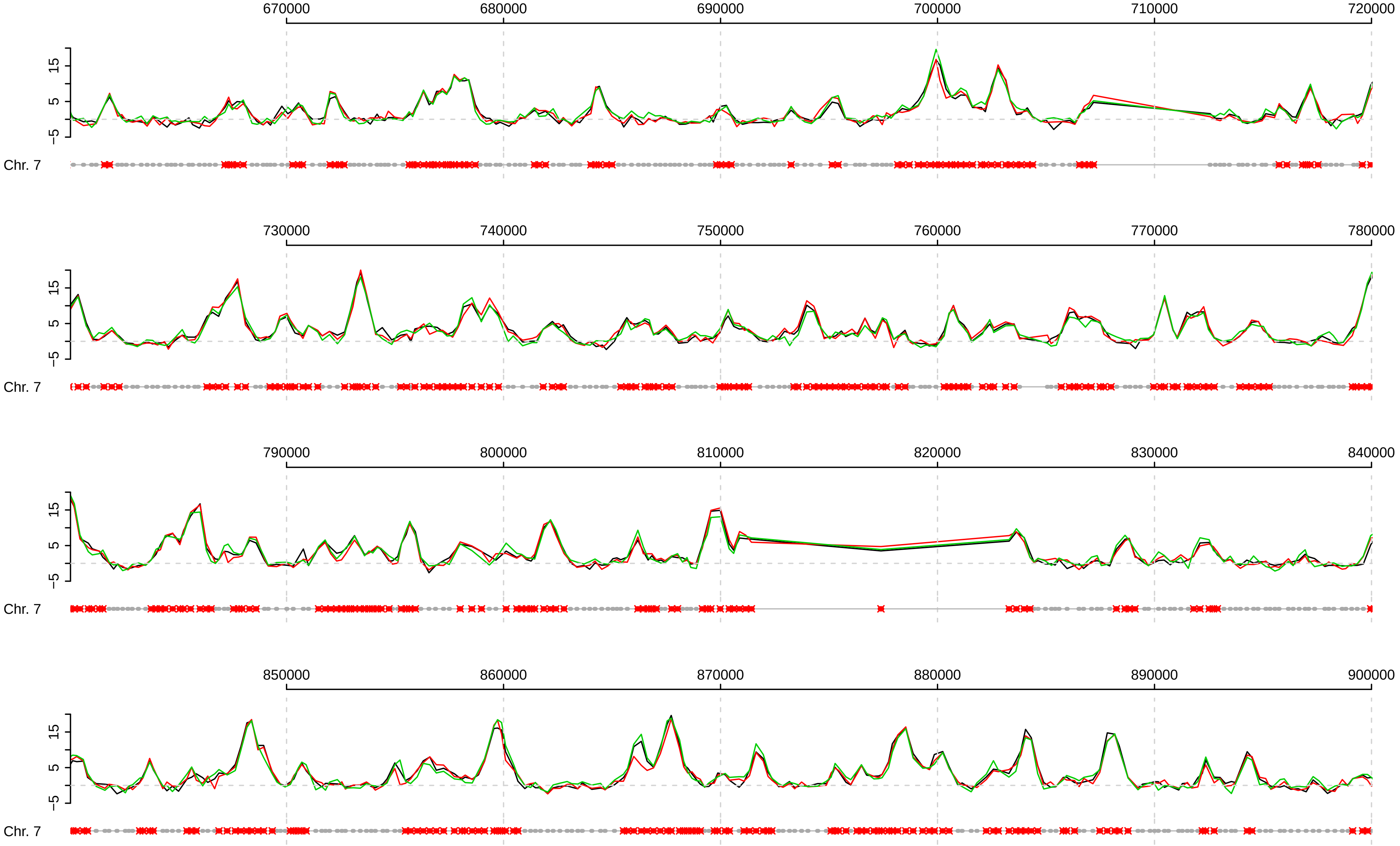
670000
680000
690000
700000
710000
720000
15
5
−5
Chr. 7
730000
740000
750000
760000
770000
780000
15
5
−5
Chr. 7
790000
800000
810000
820000
830000
840000
15
5
−5
Chr. 7
850000
860000
870000
880000
890000
900000
15
5
−5
Chr. 7

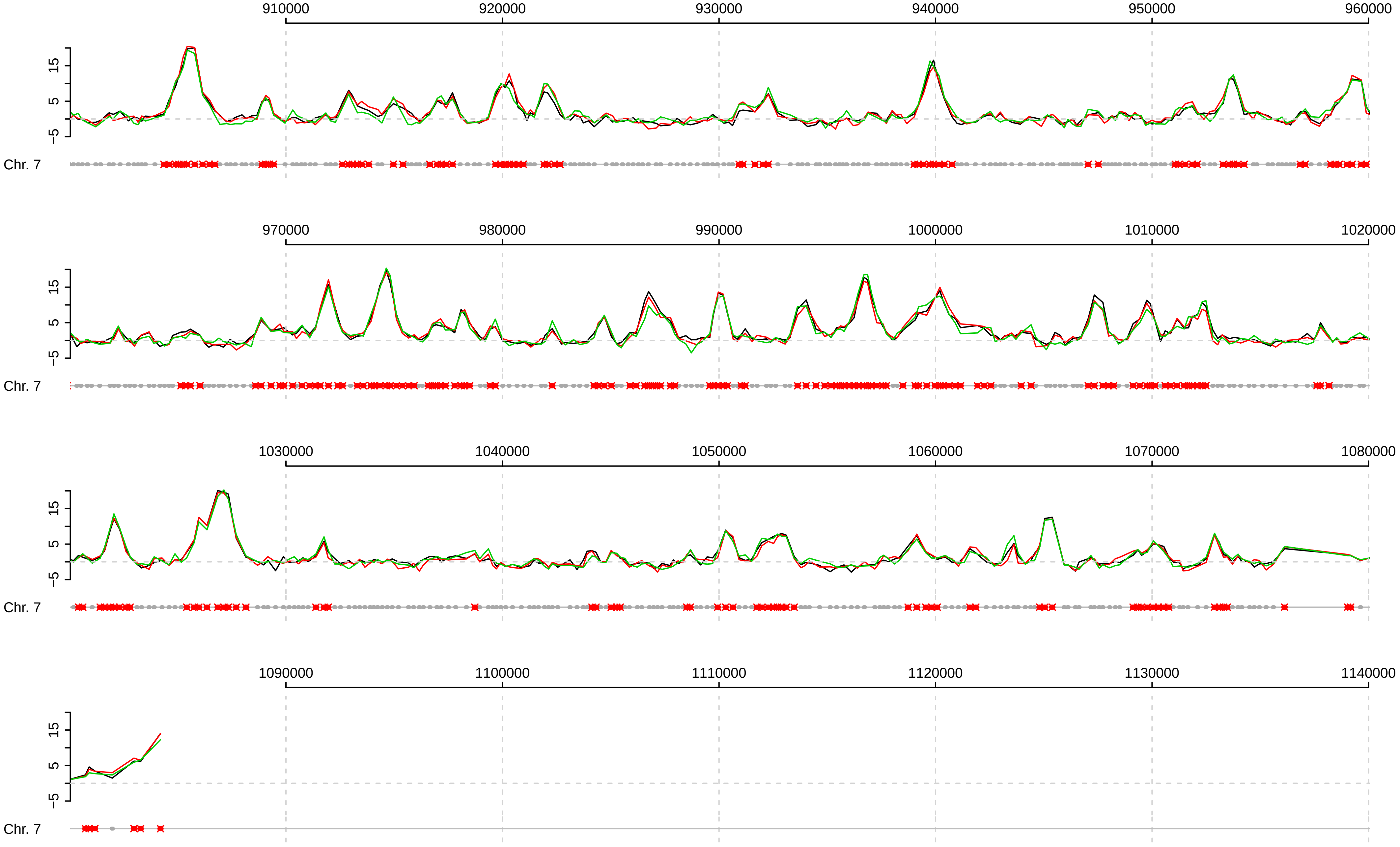

910000
920000
930000
940000
950000
960000
15
5
−5
Chr. 7
970000
980000
990000
1000000
1010000
1020000
15
5
−5
Chr. 7
1030000
1040000
1050000
1060000
1070000
1080000
15
5
−5
Chr. 7
1090000
1100000
1110000
1120000
1130000
1140000
15
5
−5
Chr. 7

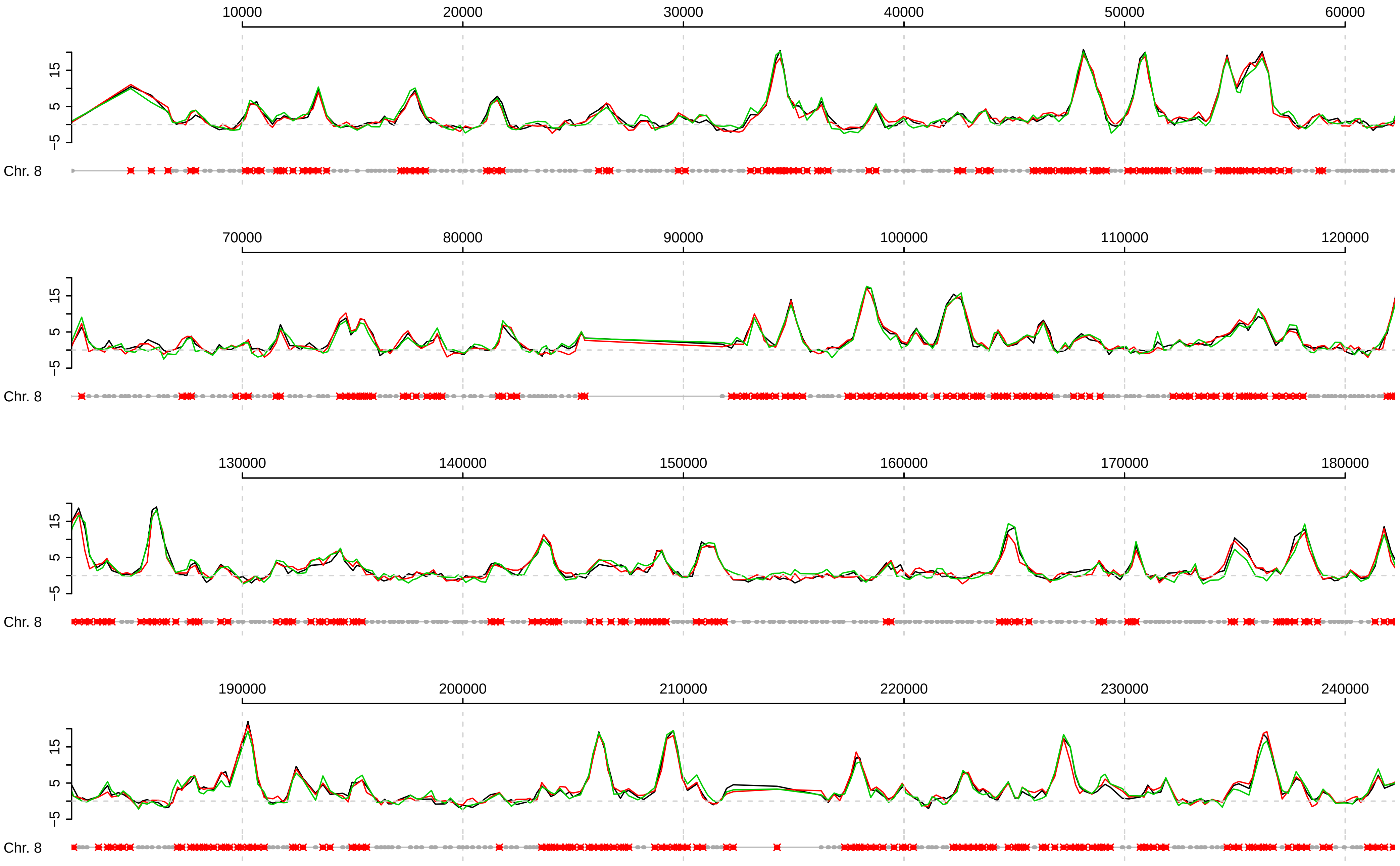

10000
20000
30000
40000
50000
60000
15
5
−5
Chr. 8
70000
80000
90000
100000
110000
120000
15
5
−5
Chr. 8
130000
140000
150000
160000
170000
180000
15
5
−5
Chr. 8
190000
200000
210000
220000
230000
240000
15
5
−5
Chr. 8

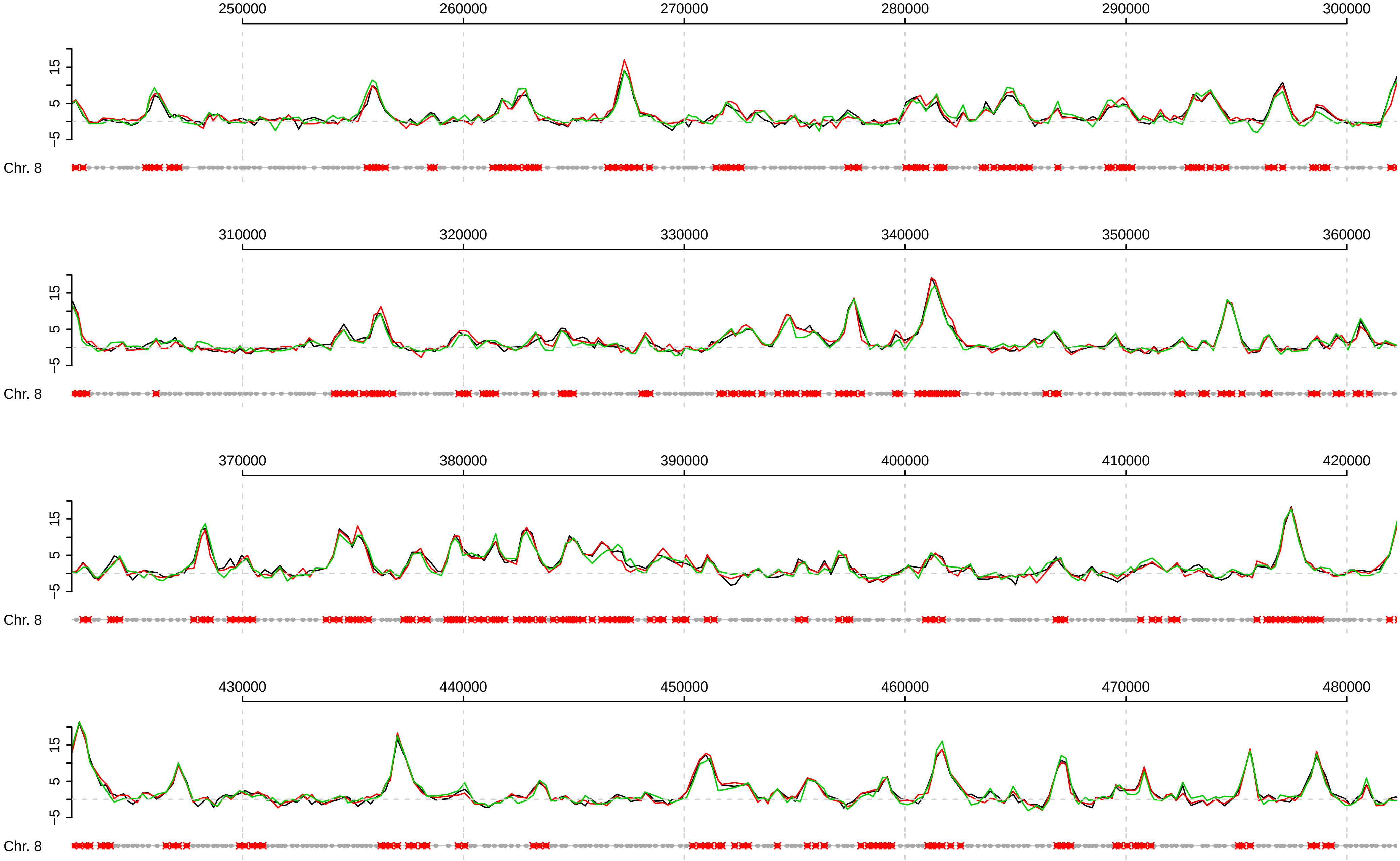

250000
260000
270000
280000
290000
300000
15
5
−5
Chr. 8
310000
320000
330000
340000
350000
360000
15
5
−5
Chr. 8
370000
380000
390000
400000
410000
420000
15
5
−5
Chr. 8
430000
440000
450000
460000
470000
480000
15
5
−5
Chr. 8

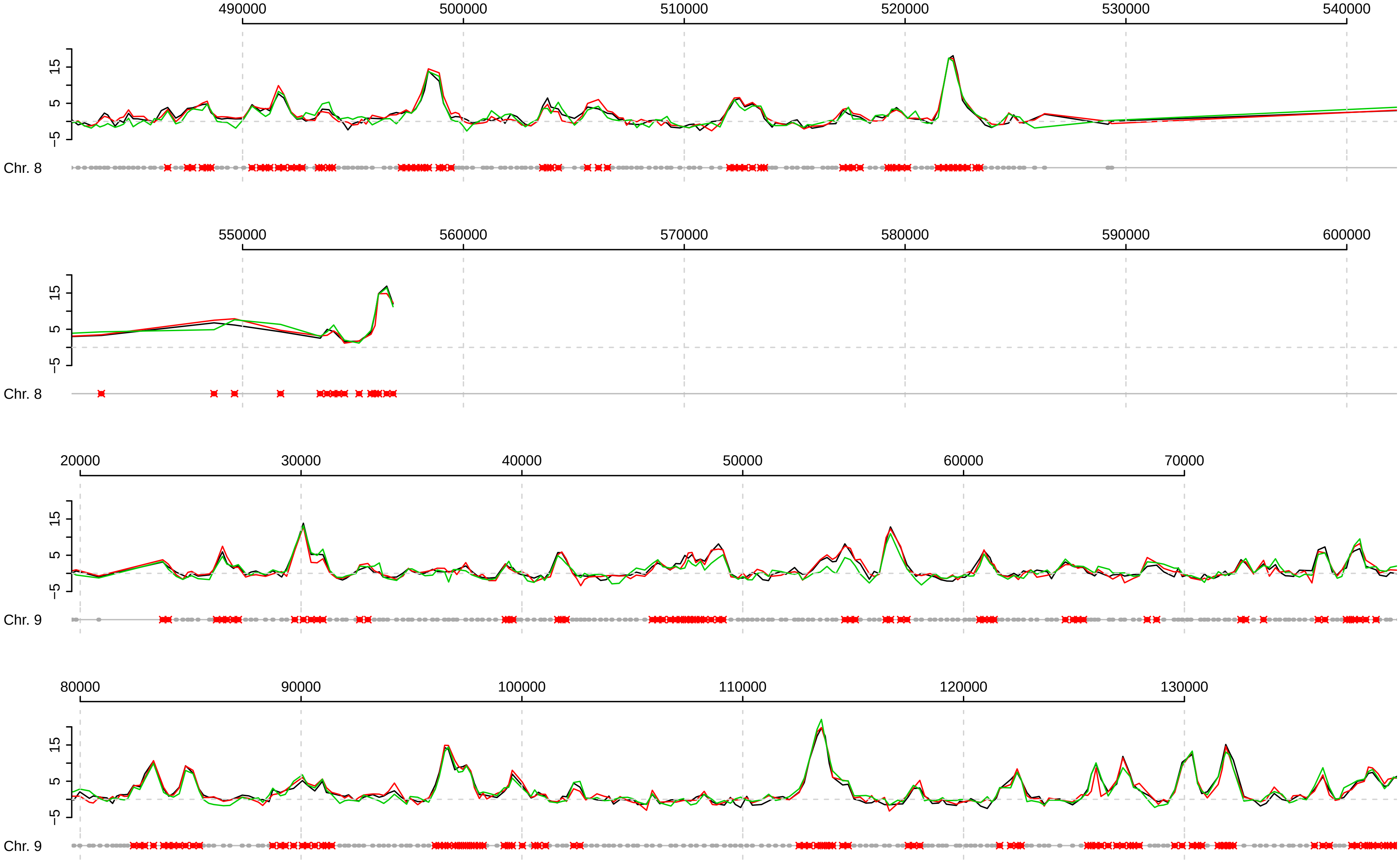
490000
500000
510000
520000
530000
540000
15
5
−5
Chr. 8
550000
560000
570000
580000
590000
600000
Chr. 8
20000
30000
40000
50000
60000
70000
Chr. 9
80000
90000
100000
110000
120000
130000
Chr. 9

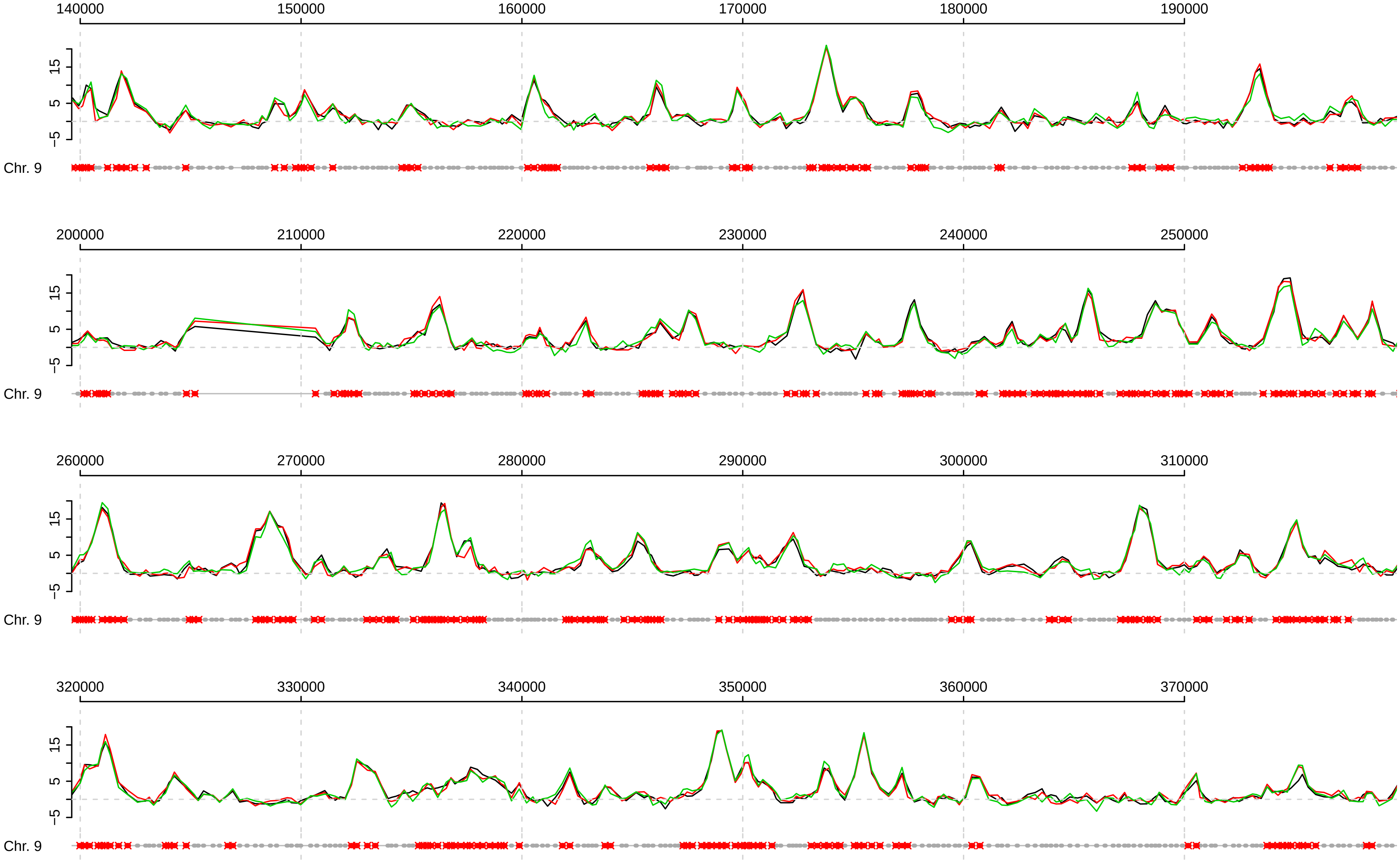

140000
150000
160000
170000
180000
190000
15
5
−5
Chr. 9
200000
210000
220000
230000
240000
250000
260000
270000
280000
290000
300000
310000
320000
330000
340000
350000
360000
370000

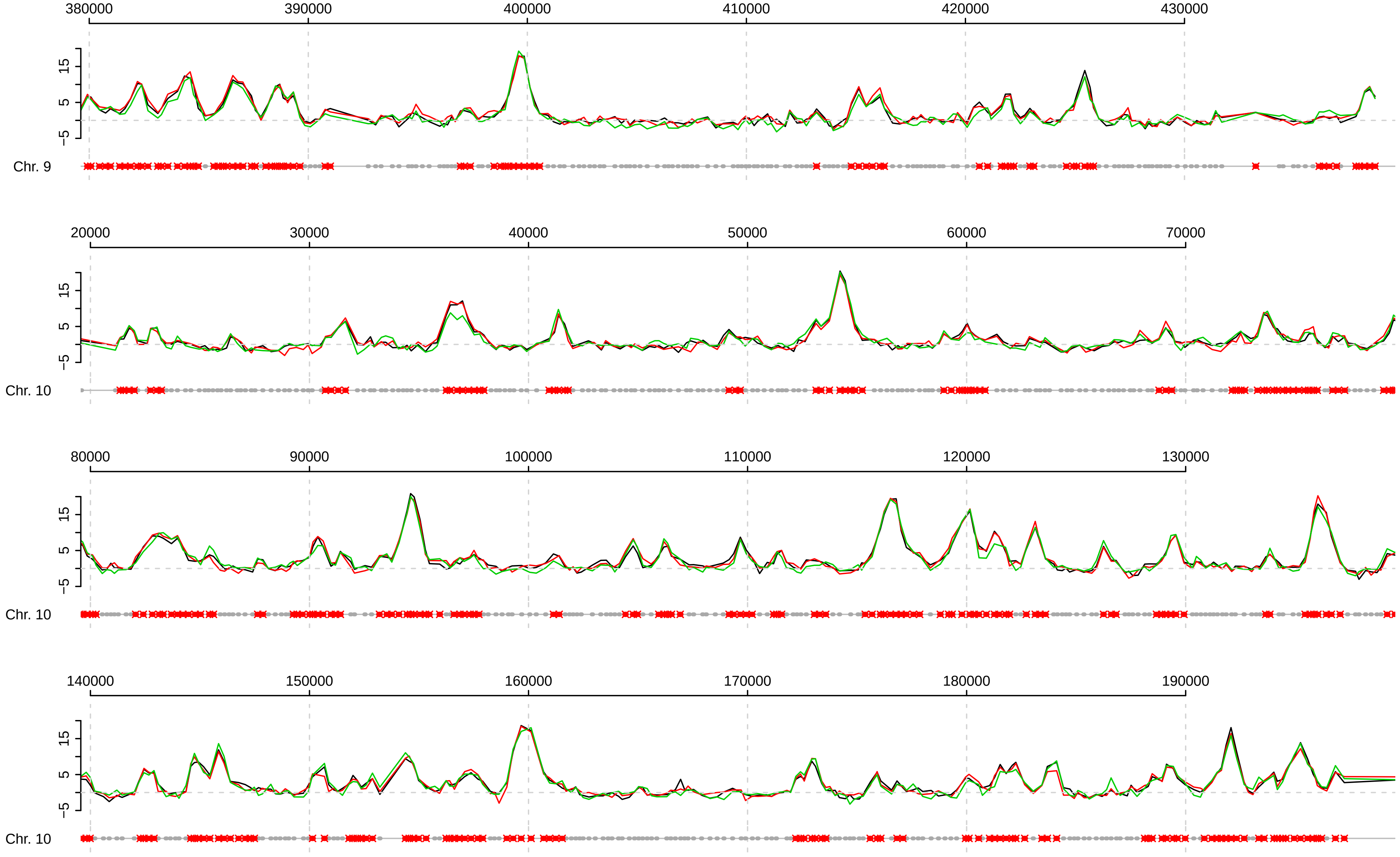
380000
390000
400000
410000
420000
430000
15
5
−5
Chr. 9
20000
30000
40000
50000
60000
70000
Chr. 10
80000
90000
100000
110000
120000
130000
Chr. 10
140000
150000
160000
170000
180000
190000
Chr. 10

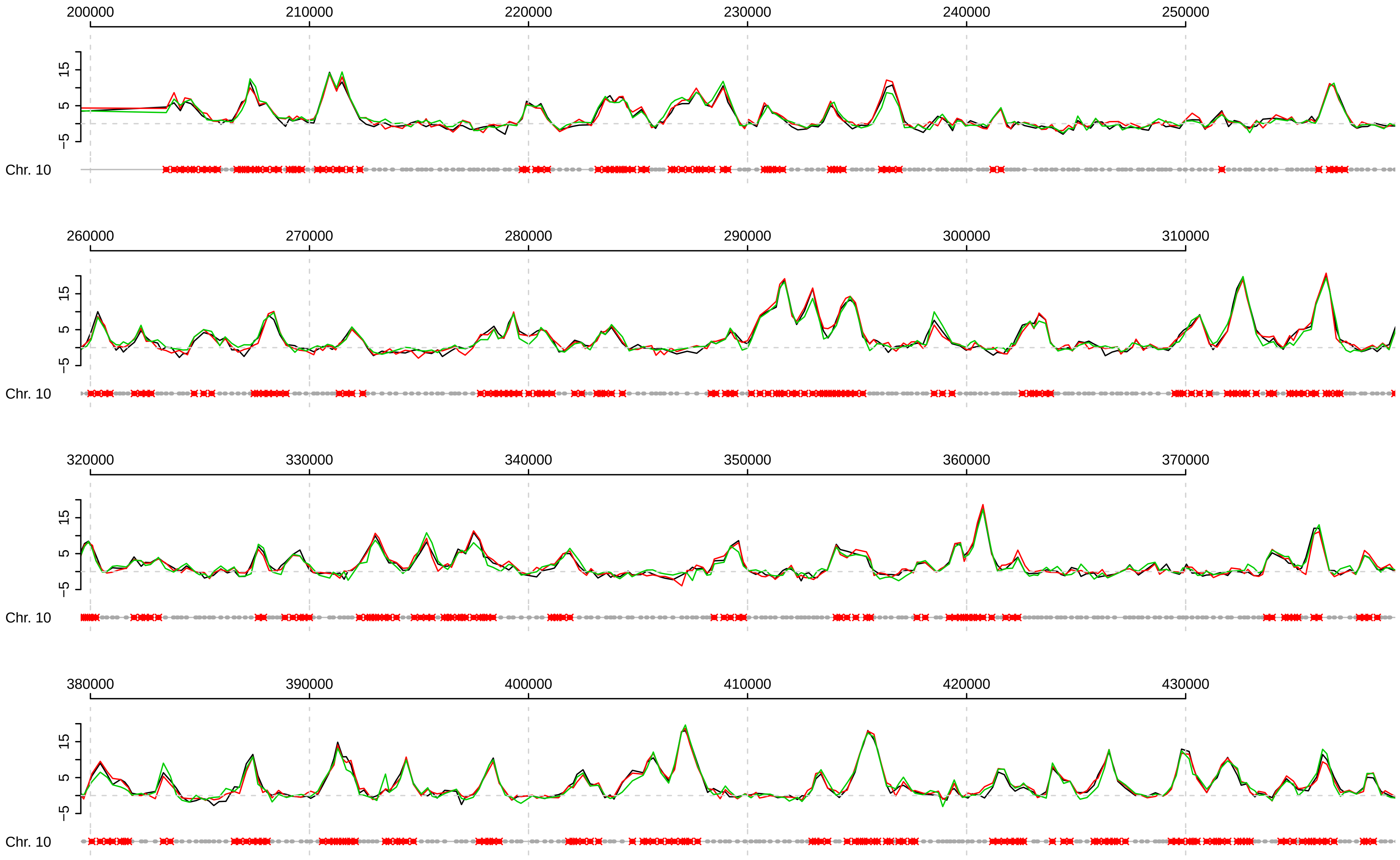

200000
210000
220000
230000
240000
250000
15
5
−5
Chr. 10
260000
270000
280000
290000
300000
310000
15
5
−5
Chr. 10
320000
330000
340000
350000
360000
370000
15
5
−5
Chr. 10
380000
390000
400000
410000
420000
430000
15
5
−5
Chr. 10

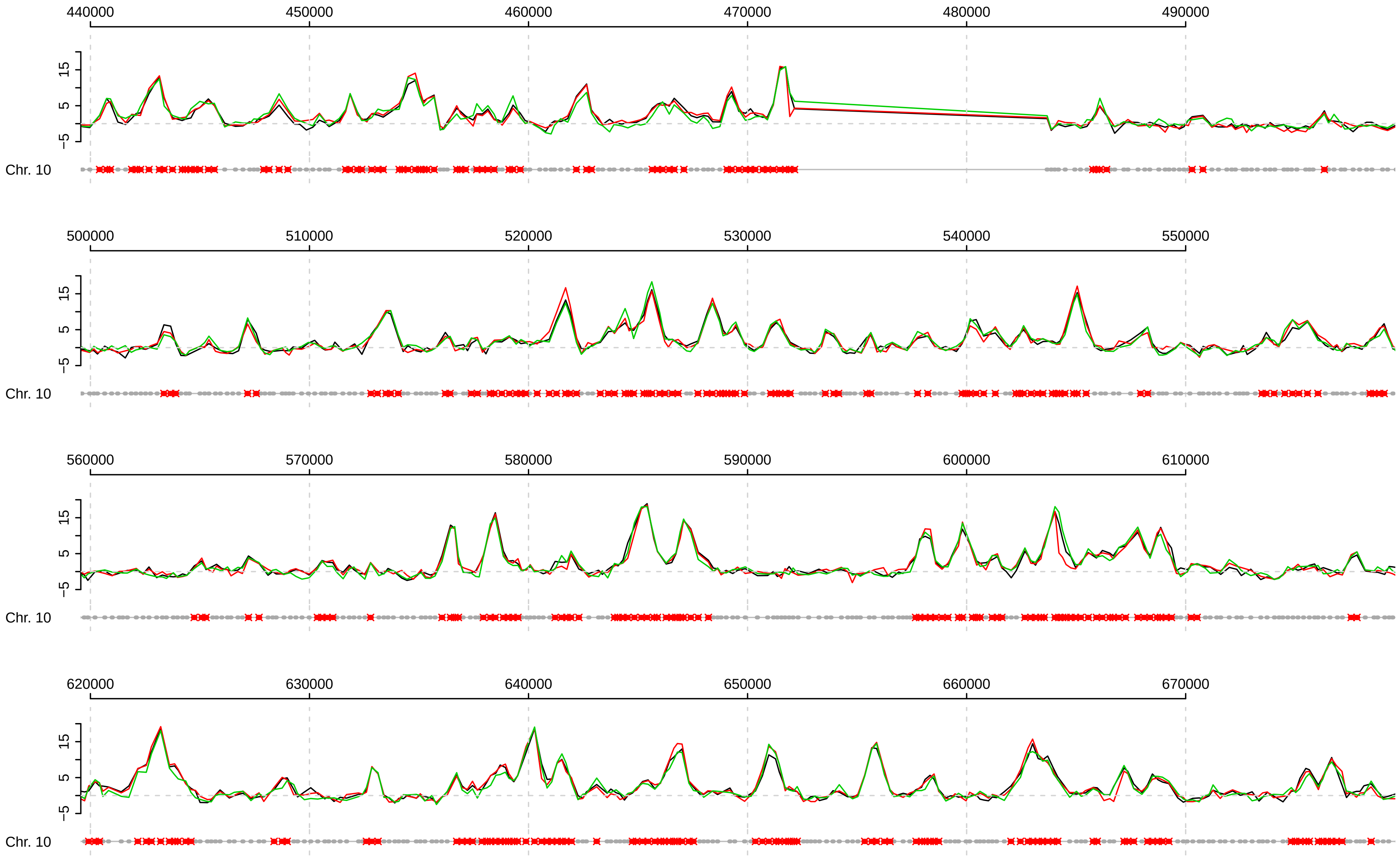

440000
450000
460000
470000
480000
490000
15
5
−5
Chr. 10
500000
510000
520000
530000
540000
550000
15
5
−5
Chr. 10
560000
570000
580000
590000
600000
610000
15
5
−5
Chr. 10
620000
630000
640000
650000
660000
670000
15
5
−5
Chr. 10

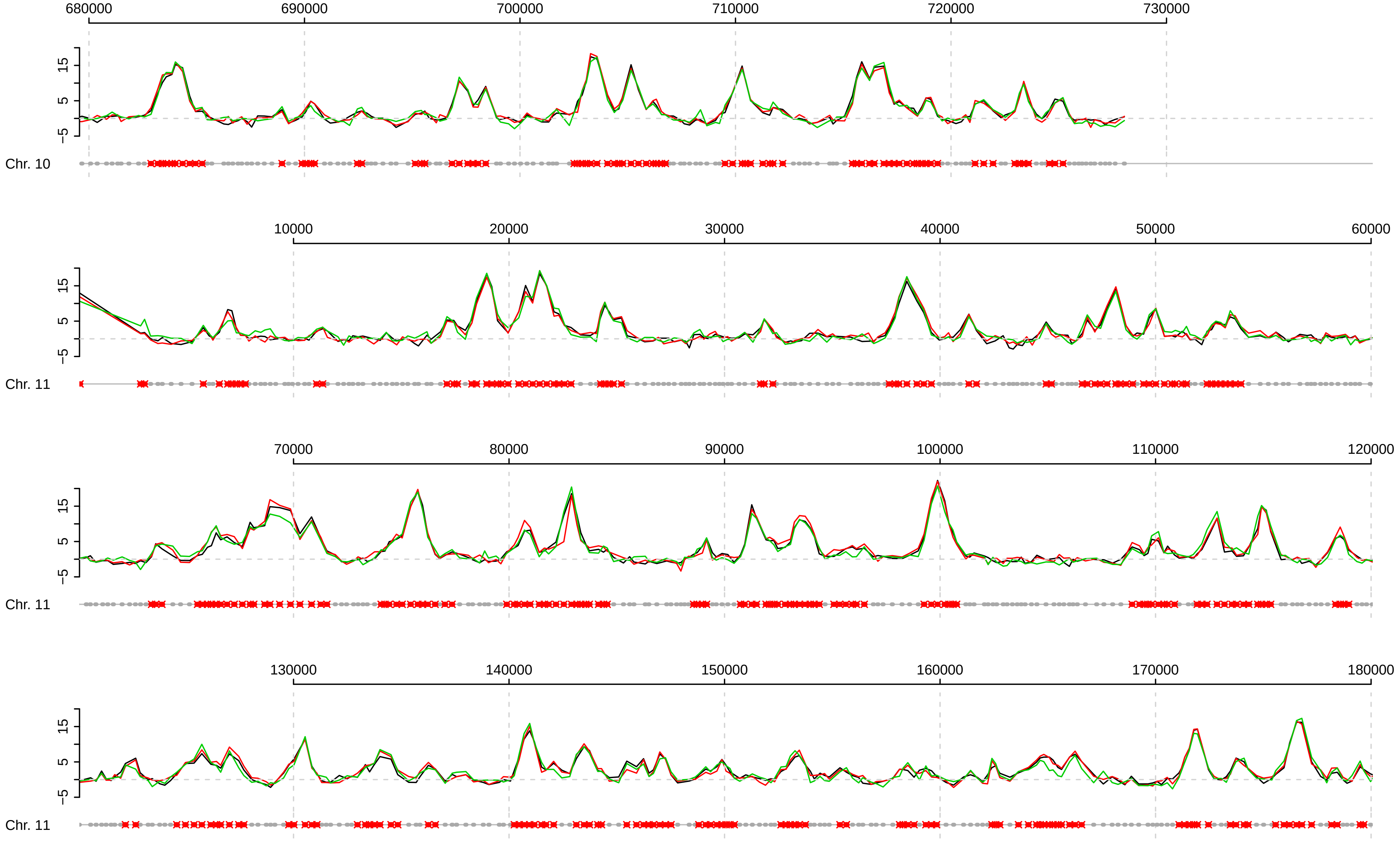

680000
690000
700000
710000
720000
730000
15
5
−5
Chr. 10
10000
20000
30000
40000
50000
60000
Chr. 11
70000
80000
90000
100000
110000
120000
Chr. 11
130000
140000
150000
160000
170000
180000
Chr. 11

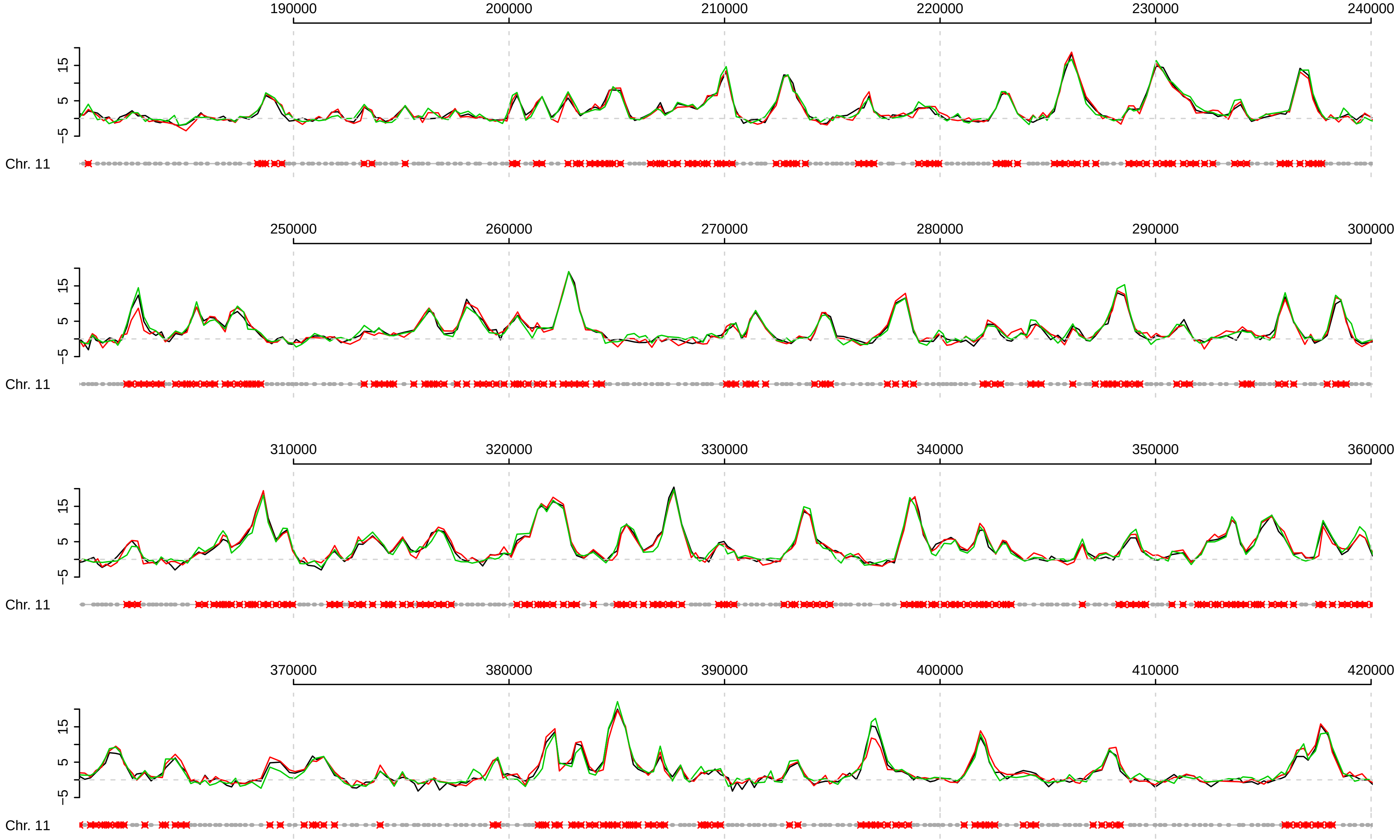

190000
200000
210000
220000
230000
240000
15
5
−5
Chr. 11
250000
260000
270000
280000
290000
300000
15
5
−5
Chr. 11
310000
320000
330000
340000
350000
360000
15
5
−5
Chr. 11
370000
380000
390000
400000
410000
420000
15
5
−5
Chr. 11

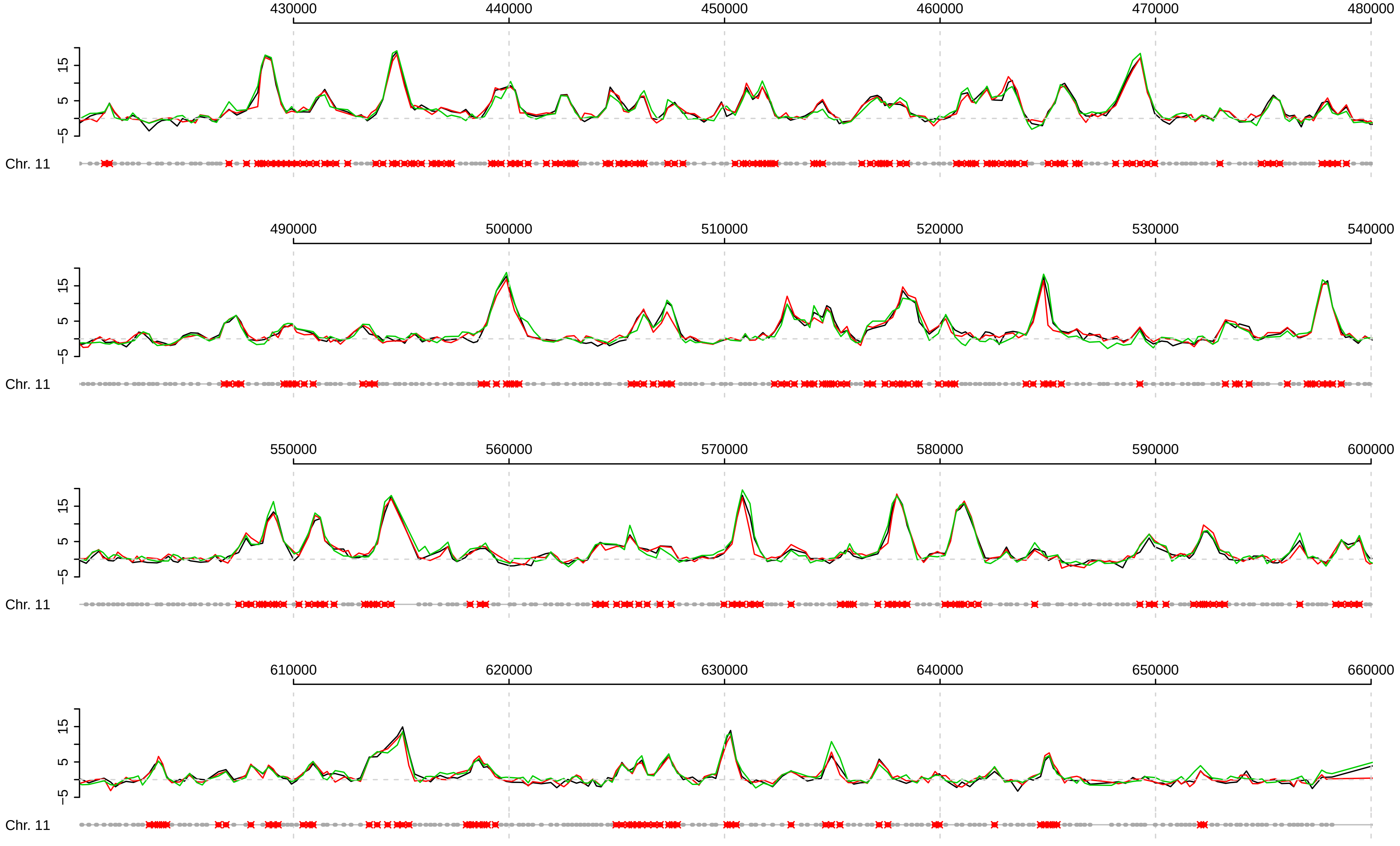
430000
440000
450000
460000
470000
480000
15
5
−5
Chr. 11
490000
500000
510000
520000
530000
540000
15
5
−5
Chr. 11
550000
560000
570000
580000
590000
600000
15
5
−5
Chr. 11
610000
620000
630000
640000
650000
660000
15
5
−5
Chr. 11

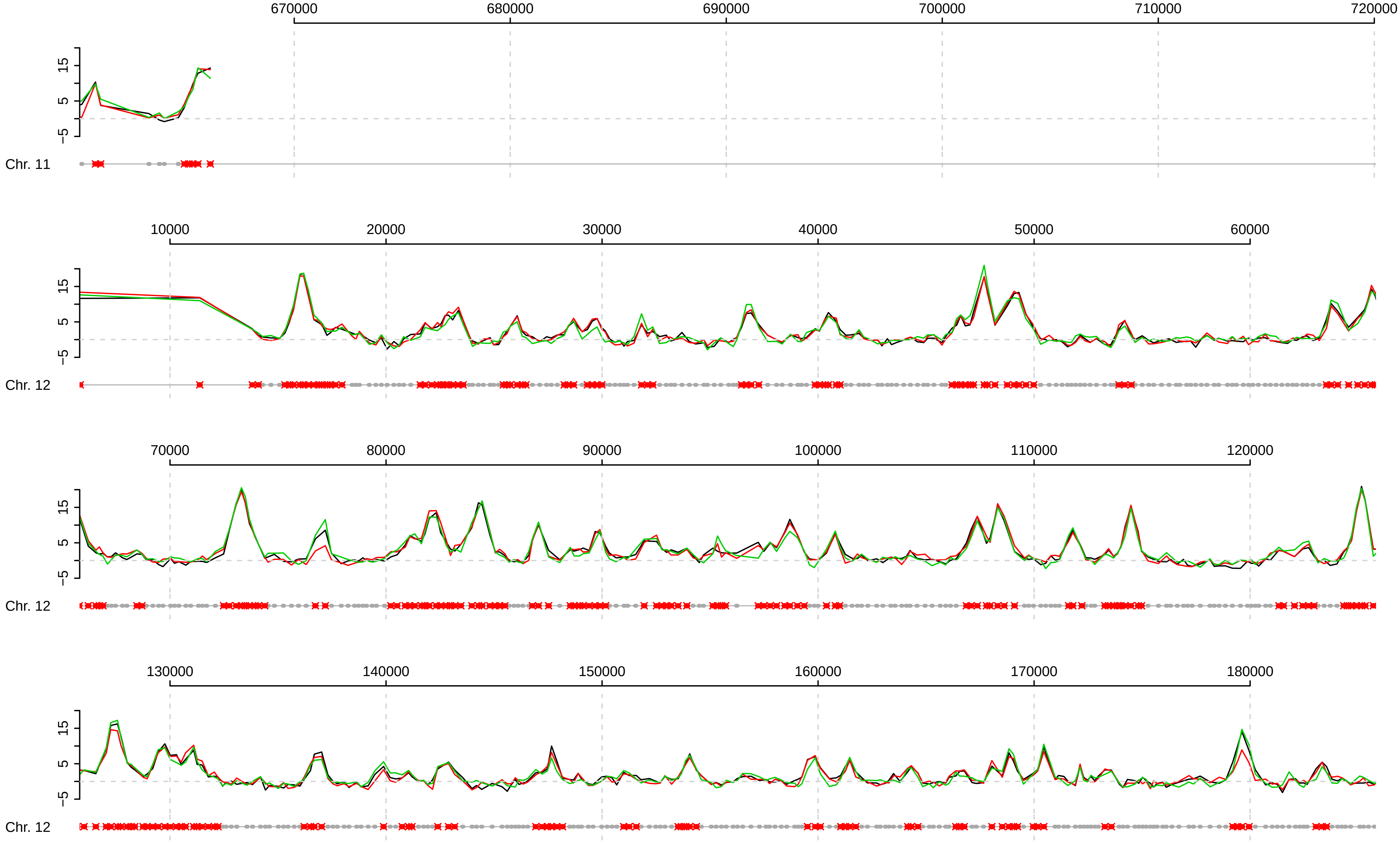

670000
680000
690000
700000
710000
720000
15
5
−5
Chr. 11
10000
20000
30000
40000
50000
60000
15
5
−5
Chr. 12
70000
80000
90000
100000
110000
120000
15
5
−5
Chr. 12
130000
140000
150000
160000
170000
180000
15
5
−5
Chr. 12

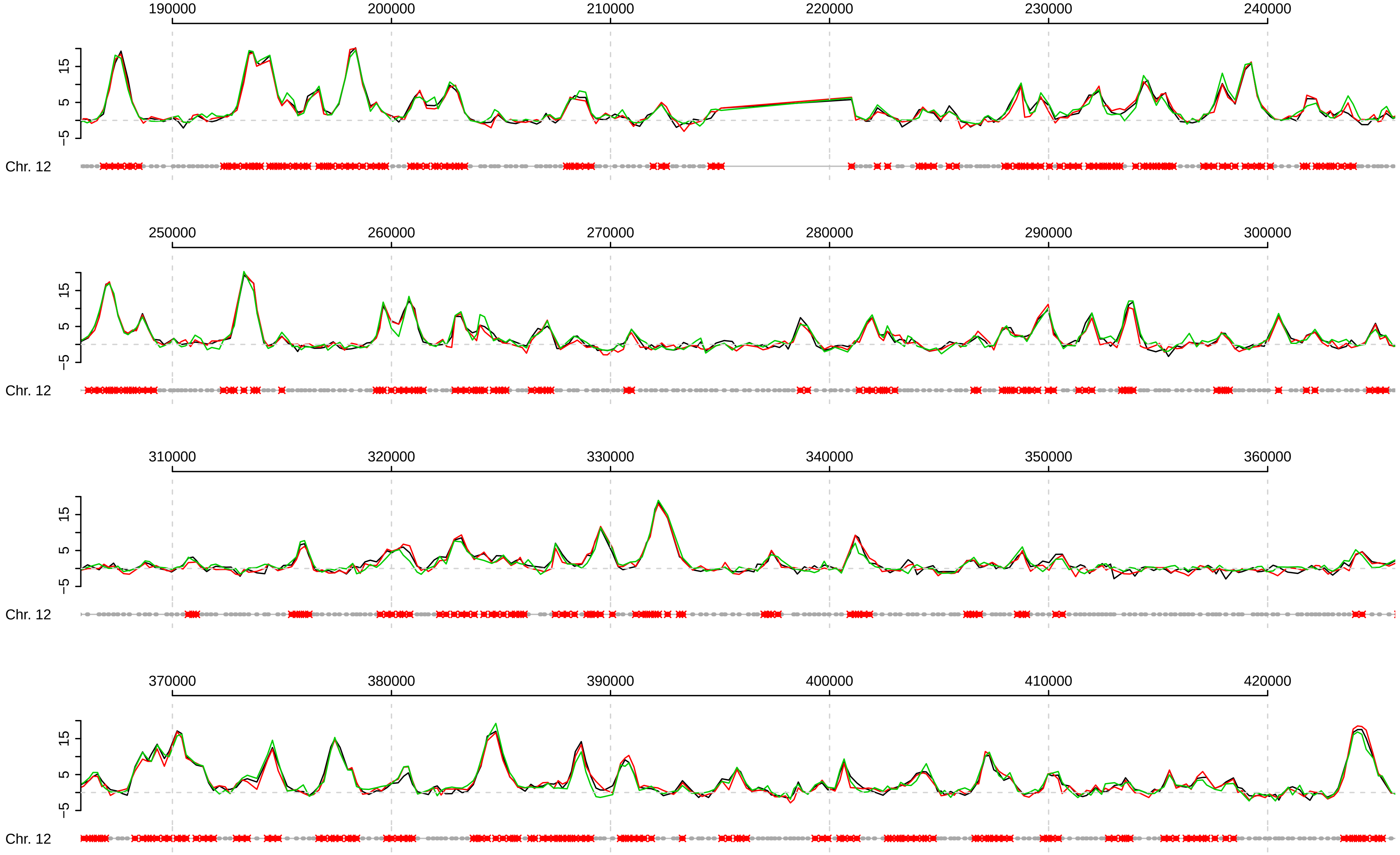
190000
200000
210000
220000
230000
240000
15
5
−5
Chr. 12
250000
260000
270000
280000
290000
300000
15
5
−5
Chr. 12
310000
320000
330000
340000
350000
360000
15
5
−5
Chr. 12
370000
380000
390000
400000
410000
420000
15
5
−5
Chr. 12

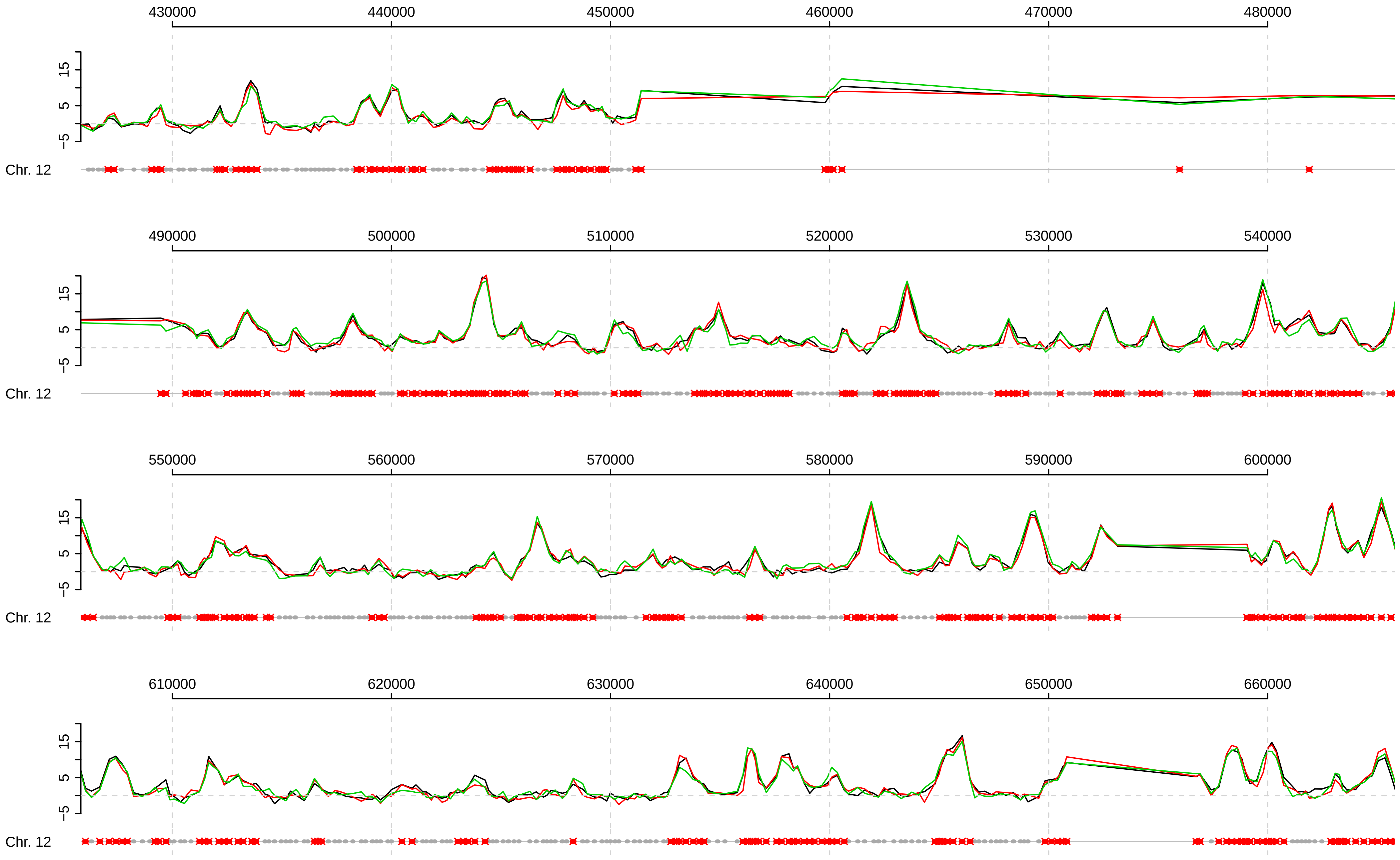

430000
440000
450000
460000
470000
480000
15
5
−5
Chr. 12
490000
500000
510000
520000
530000
540000
15
5
−5
Chr. 12
550000
560000
570000
580000
590000
600000
15
5
−5
Chr. 12
610000
620000
630000
640000
650000
660000
15
5
−5
Chr. 12

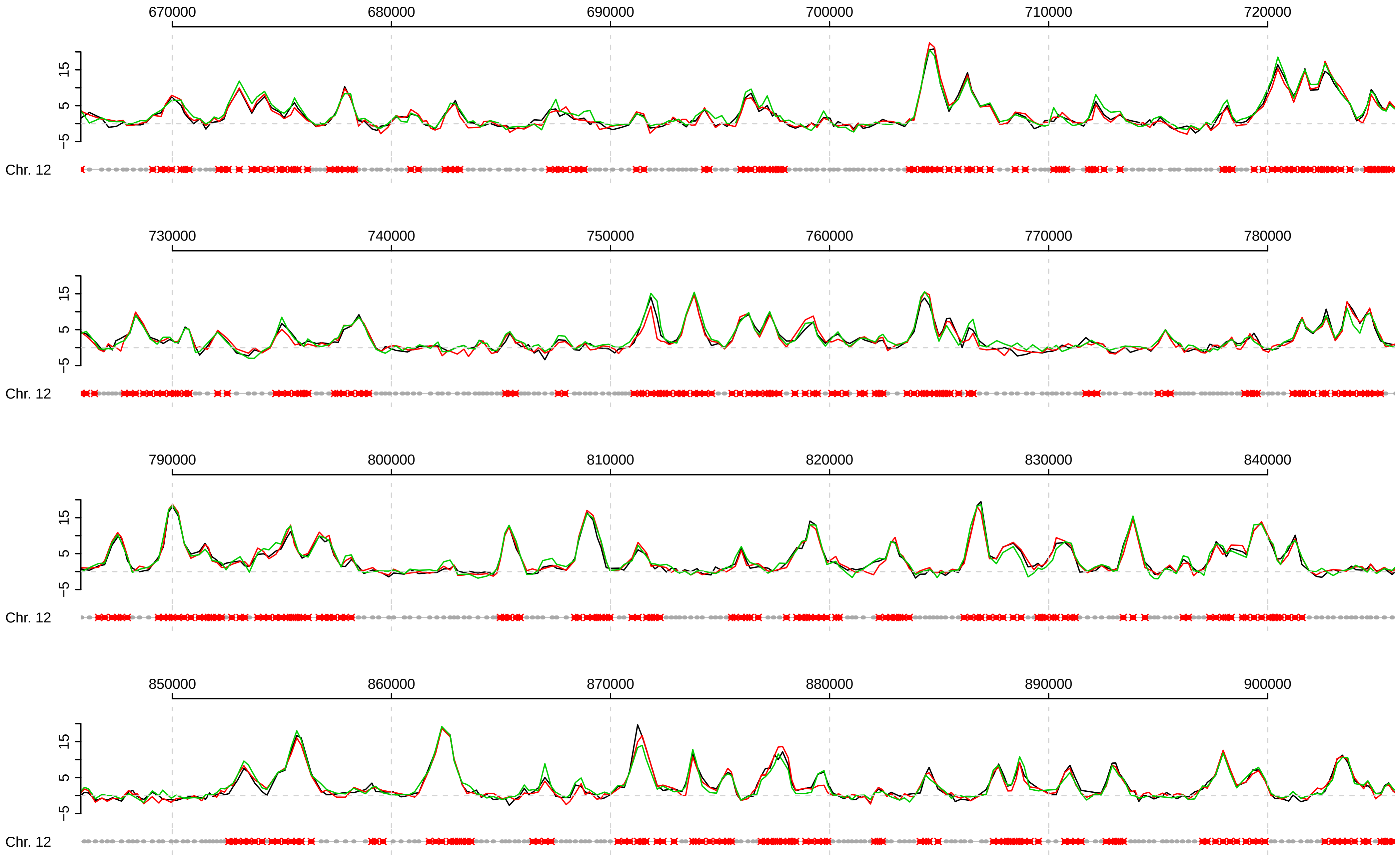

670000
680000
690000
700000
710000
720000
15
5
−5
Chr. 12
730000
740000
750000
760000
770000
780000
15
5
−5
Chr. 12
790000
800000
810000
820000
830000
840000
15
5
−5
Chr. 12
850000
860000
870000
880000
890000
900000
15
5
−5
Chr. 12

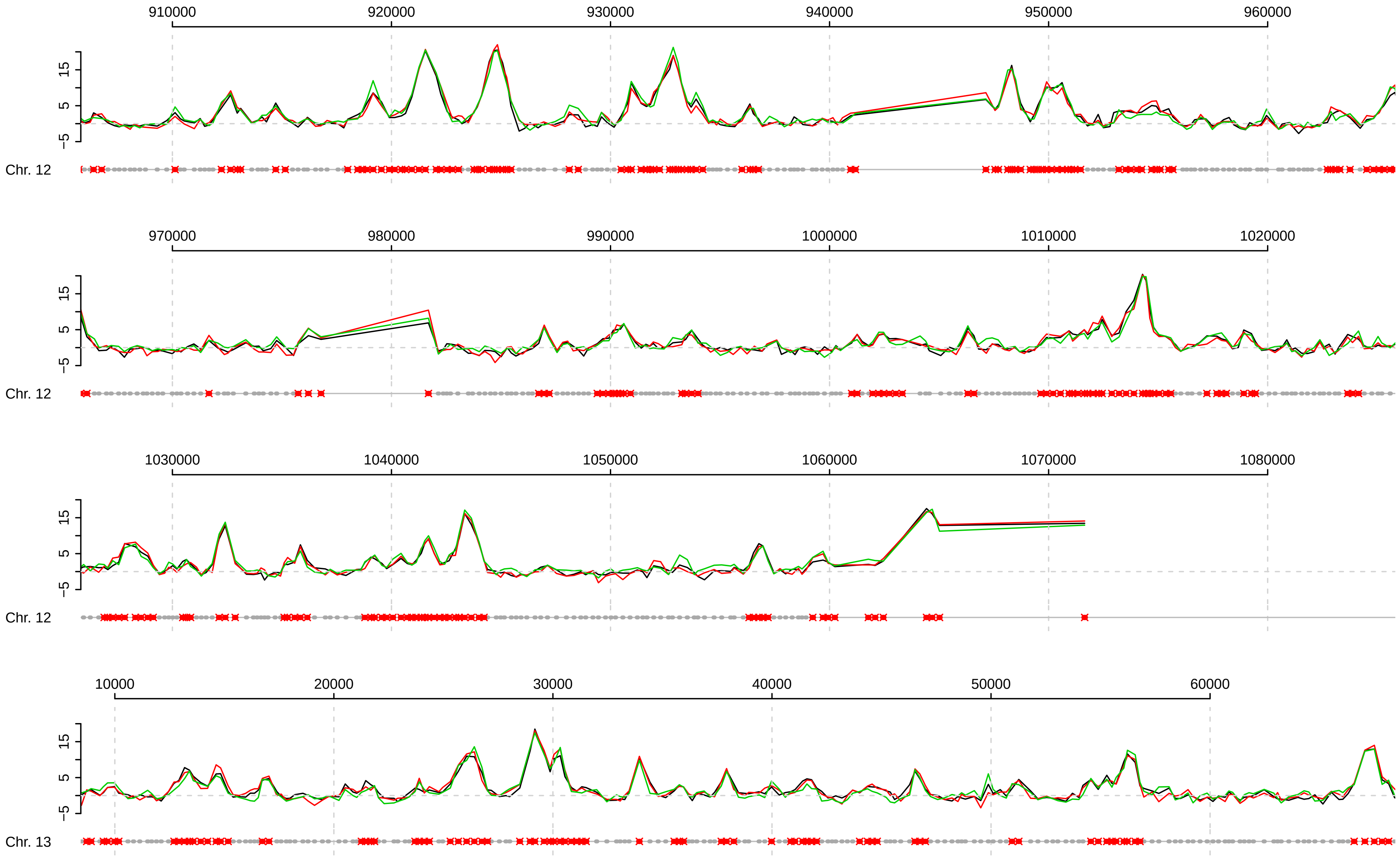

910000
920000
930000
940000
950000
960000
15
5
−5
Chr. 12
970000
980000
990000
1000000
1010000
1020000
15
5
−5
Chr. 12
1030000
1040000
1050000
1060000
1070000
1080000
15
5
−5
Chr. 12
10000
20000
30000
40000
50000
60000
15
5
−5
Chr. 13

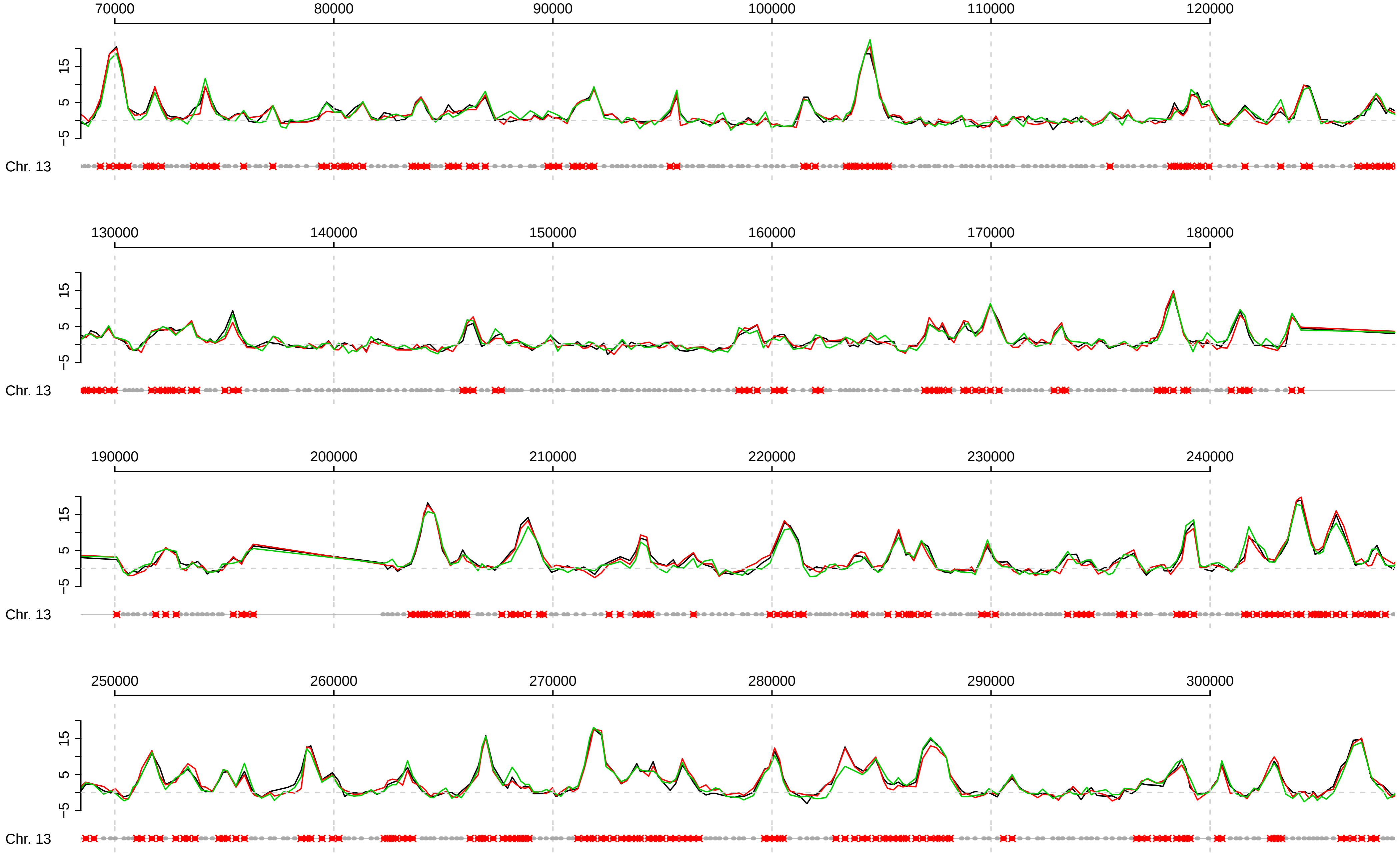

70000
80000
90000
100000
110000
120000
15
5
−5
Chr. 13
130000
140000
150000
160000
170000
180000
15
5
−5
Chr. 13
190000
200000
210000
220000
230000
240000
15
5
−5
Chr. 13
250000
260000
270000
280000
290000
300000
15
5
−5
Chr. 13

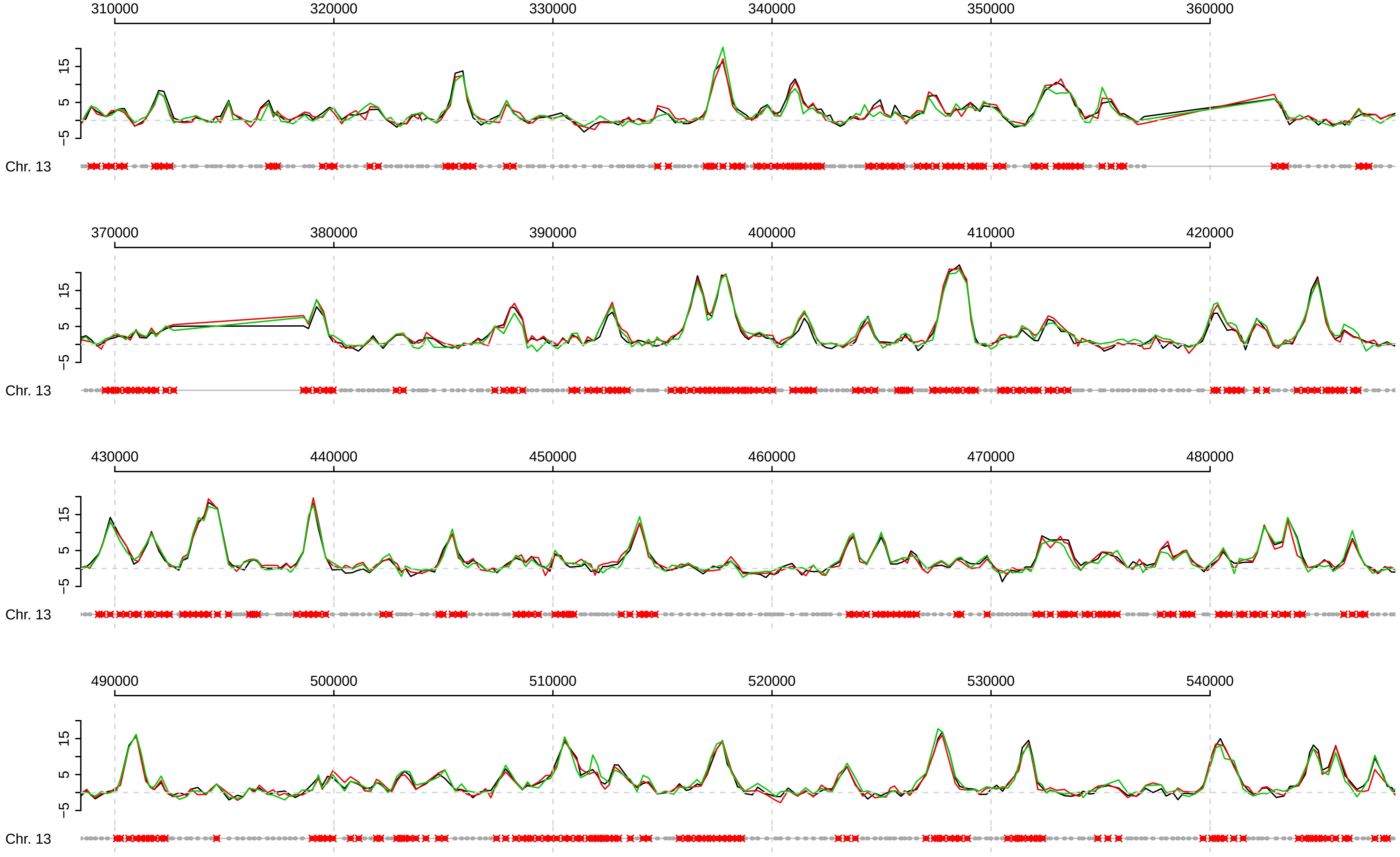
310000
320000
330000
340000
350000
360000
−5
5
15
Chr. 13
370000
380000
390000
400000
410000
420000
−5
5
15
Chr. 13
430000
440000
450000
460000
470000
480000
−5
5
15
Chr. 13
490000
500000
510000
520000
530000
540000
−5
5
15
Chr. 13

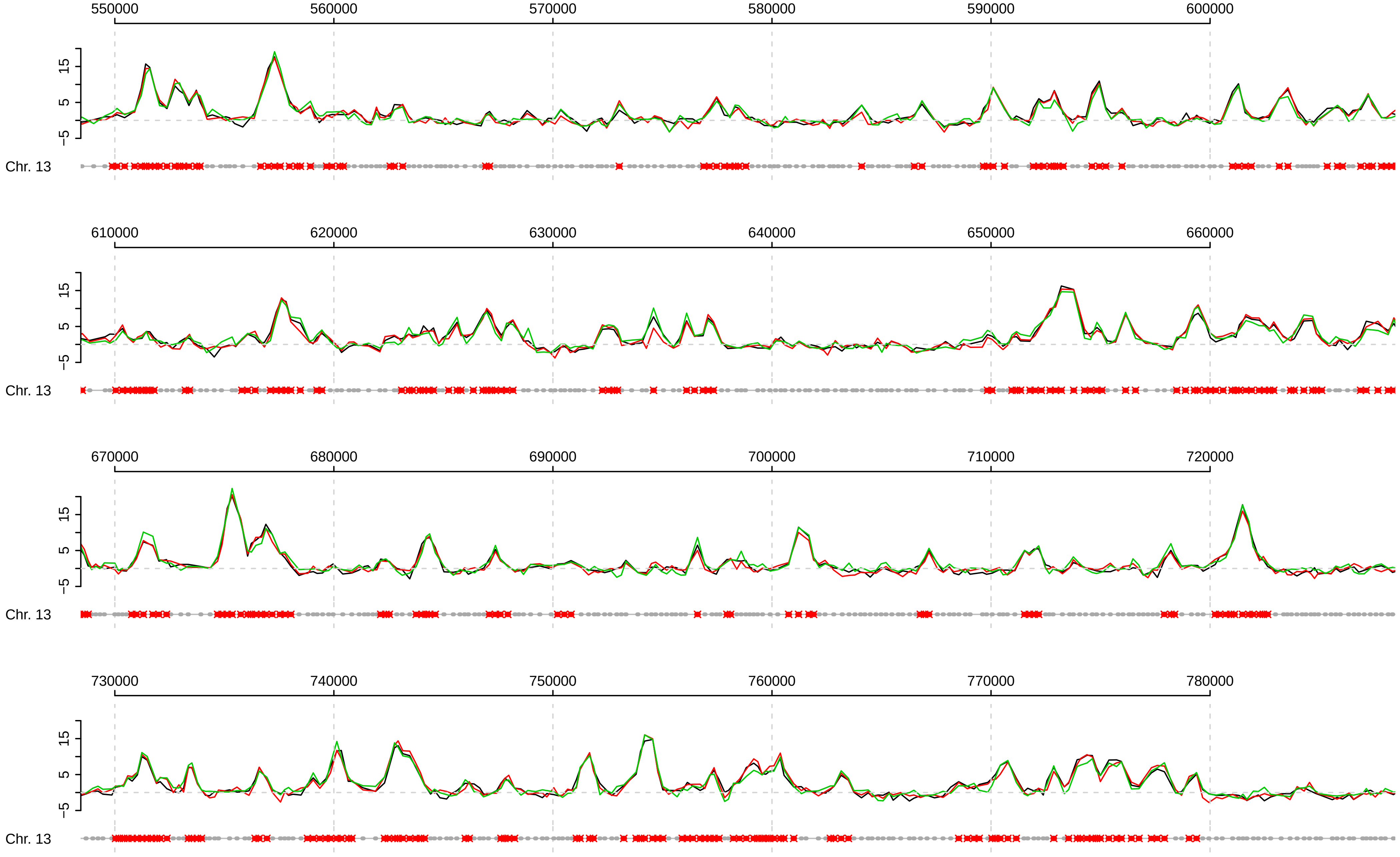

550000
560000
570000
580000
590000
600000
15
5
−5
Chr. 13
610000
620000
630000
640000
650000
660000
15
5
−5
Chr. 13
670000
680000
690000
700000
710000
720000
15
5
−5
Chr. 13
730000
740000
750000
760000
770000
780000
15
5
−5
Chr. 13

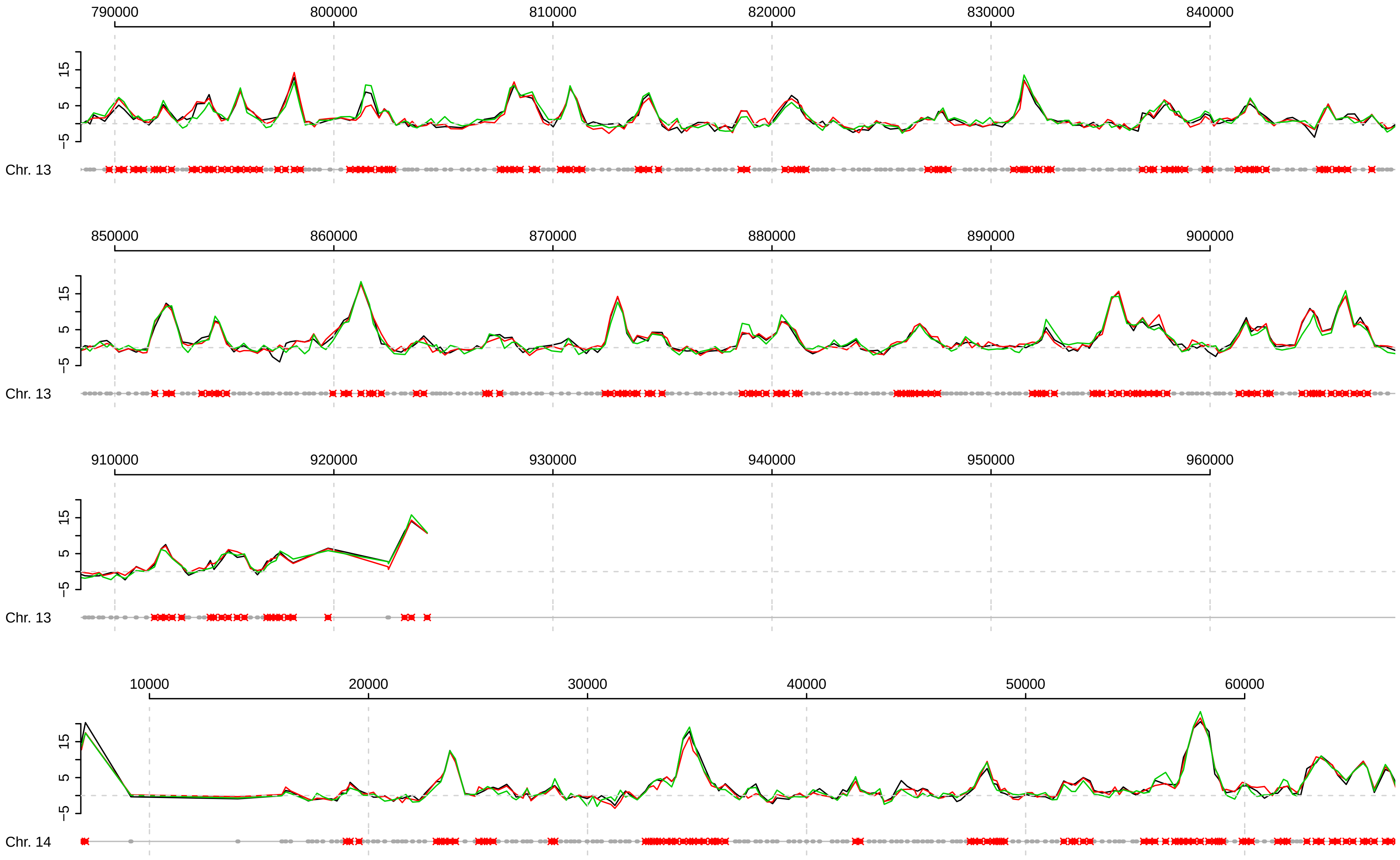

790000
800000
810000
820000
830000
840000
15
5
−5
Chr. 13
850000
860000
870000
880000
890000
900000
15
5
−5
Chr. 13
910000
920000
930000
940000
950000
960000
15
5
−5
Chr. 13
10000
20000
30000
40000
50000
60000
15
5
−5
Chr. 14

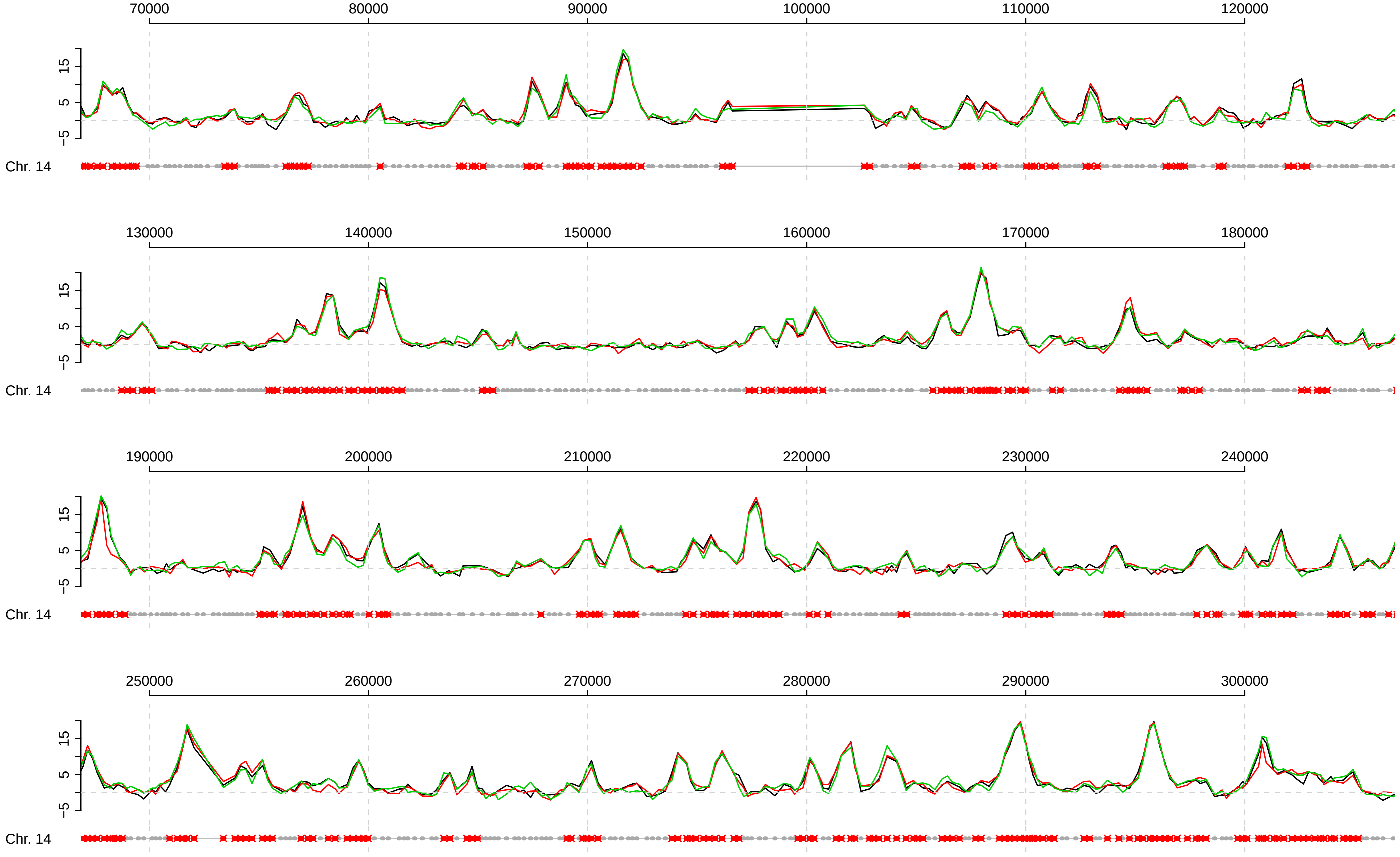

70000
80000
90000
100000
110000
120000
130000
140000
150000
160000
170000
180000
190000
200000
210000
220000
230000
240000
250000
260000
270000
280000
290000
300000
15
5
−5
Chr. 14

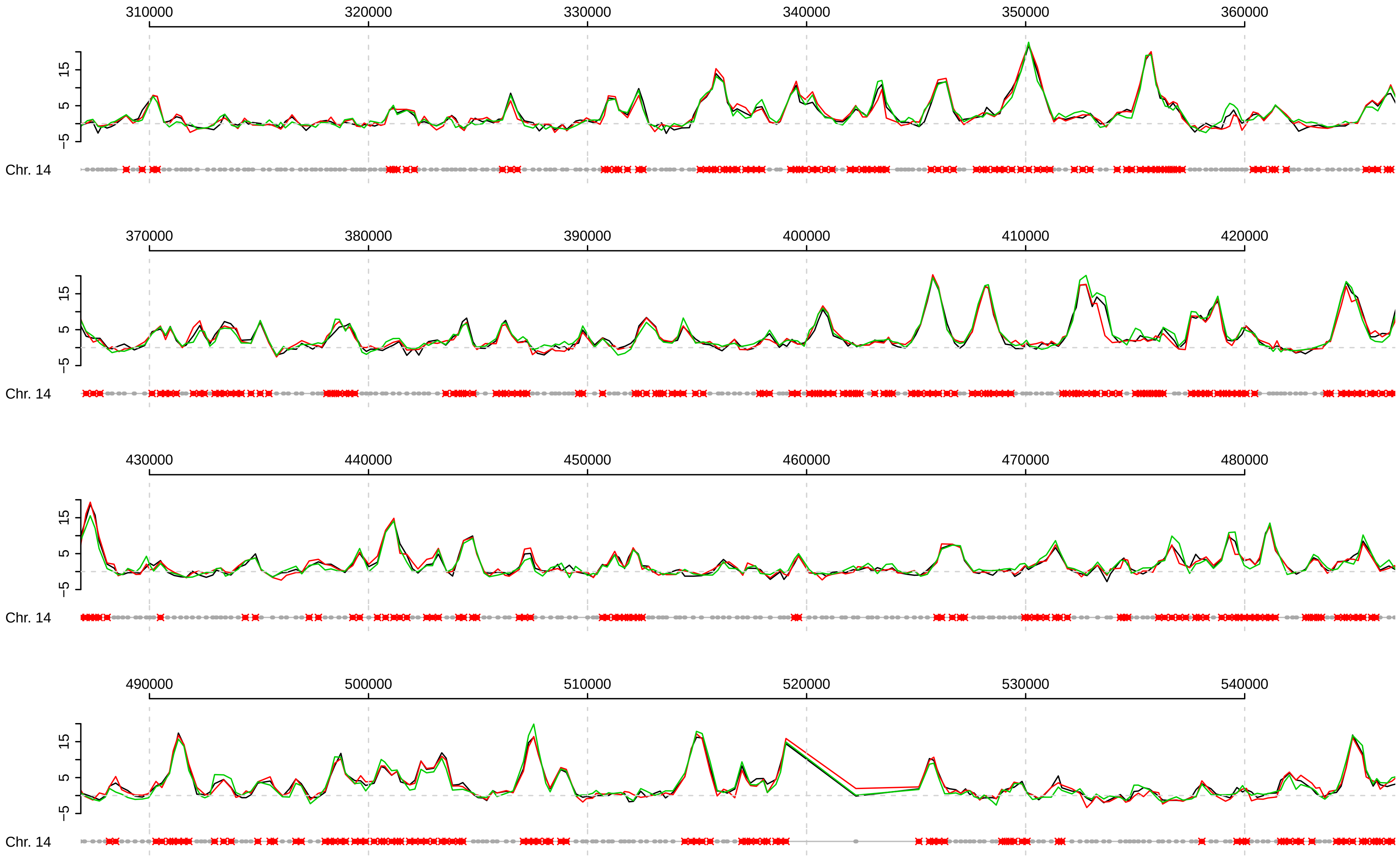

310000
320000
330000
340000
350000
360000
15
5
−5
Chr. 14
370000
380000
390000
400000
410000
420000
15
5
−5
Chr. 14
430000
440000
450000
460000
470000
480000
15
5
−5
Chr. 14
490000
500000
510000
520000
530000
540000
15
5
−5
Chr. 14

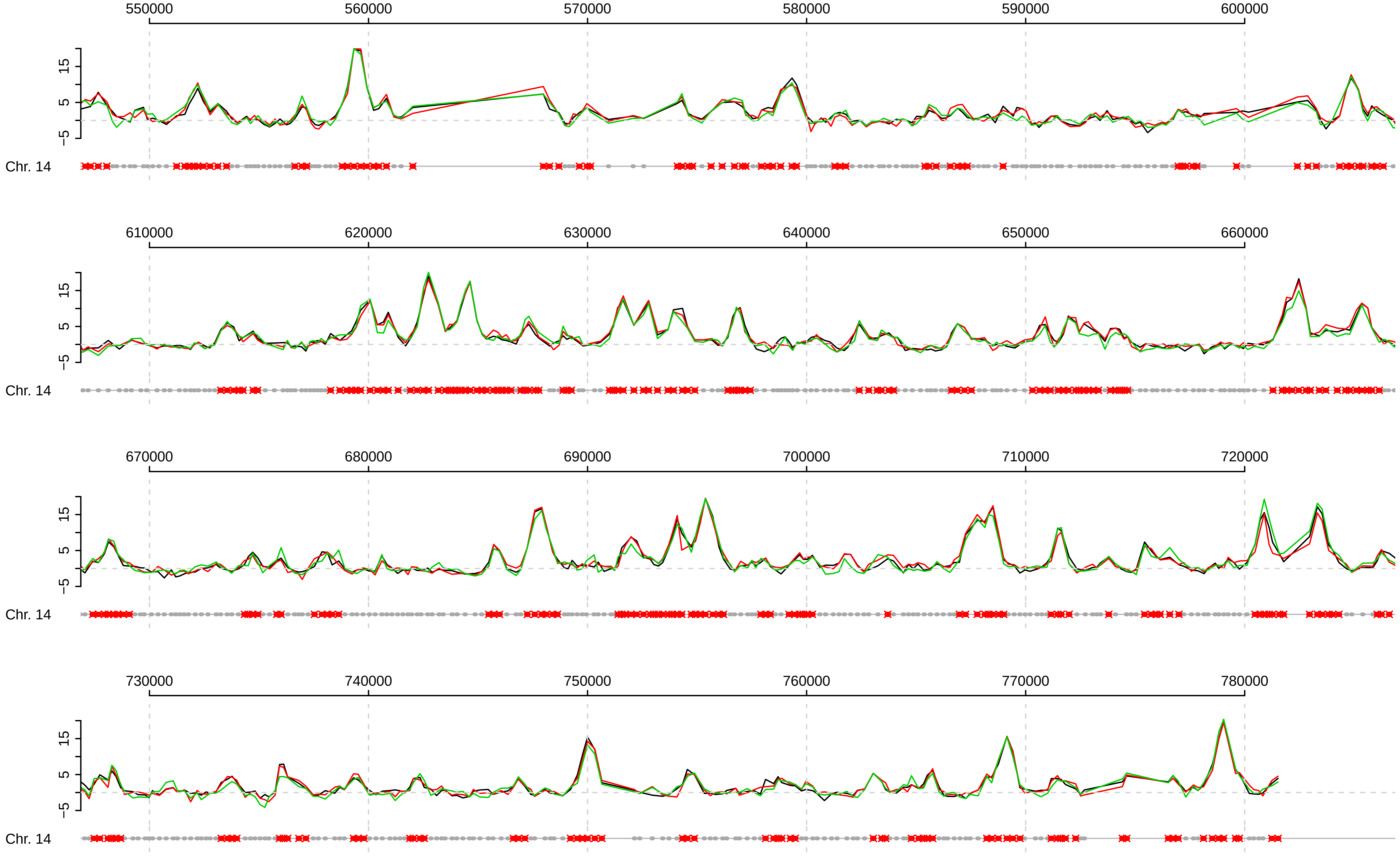

550000
560000
570000
580000
590000
600000
15
5
−5
Chr. 14
610000
620000
630000
640000
650000
660000
15
5
−5
Chr. 14
670000
680000
690000
700000
710000
720000
15
5
−5
Chr. 14
730000
740000
750000
760000
770000
780000
15
5
−5
Chr. 14

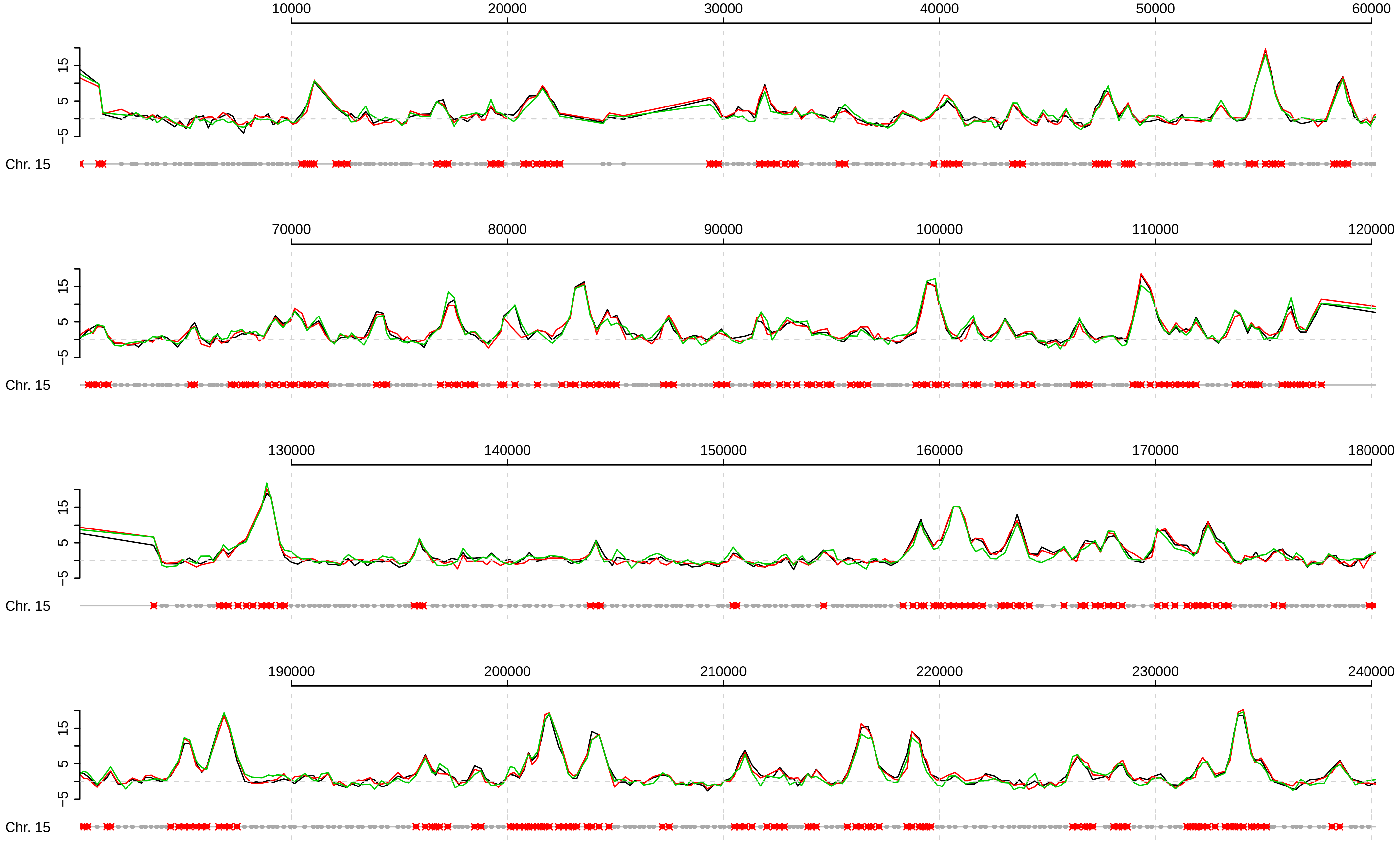
10000
20000
30000
40000
50000
60000
15
5
−5
Chr. 15
70000
80000
90000
100000
110000
120000
15
5
−5
Chr. 15
130000
140000
150000
160000
170000
180000
15
5
−5
Chr. 15
190000
200000
210000
220000
230000
240000
15
5
−5
Chr. 15

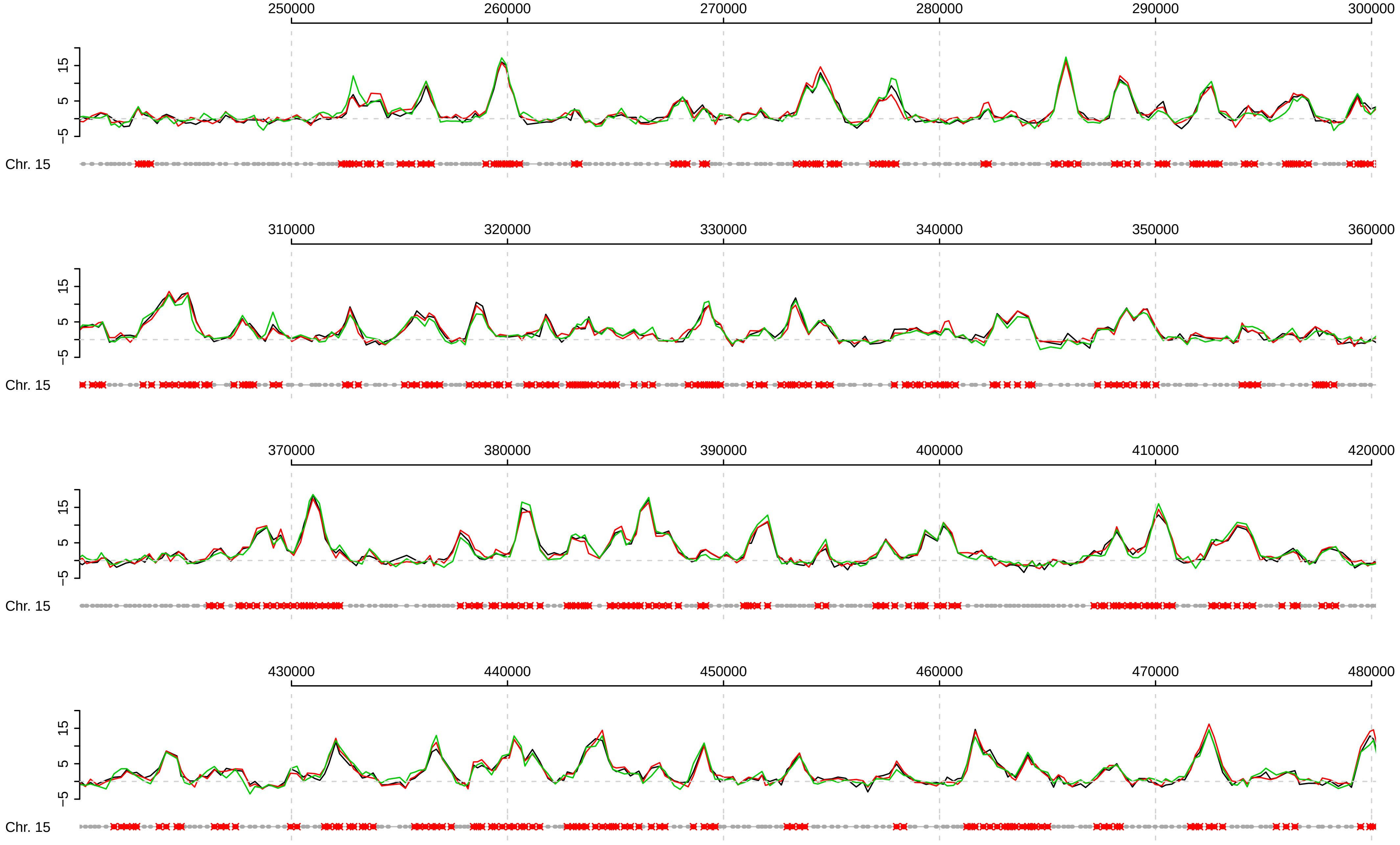

250000
260000
270000
280000
290000
300000
15
5
−5
Chr. 15
310000
320000
330000
340000
350000
360000
15
5
−5
Chr. 15
370000
380000
390000
400000
410000
420000
15
5
−5
Chr. 15
430000
440000
450000
460000
470000
480000
15
5
−5
Chr. 15

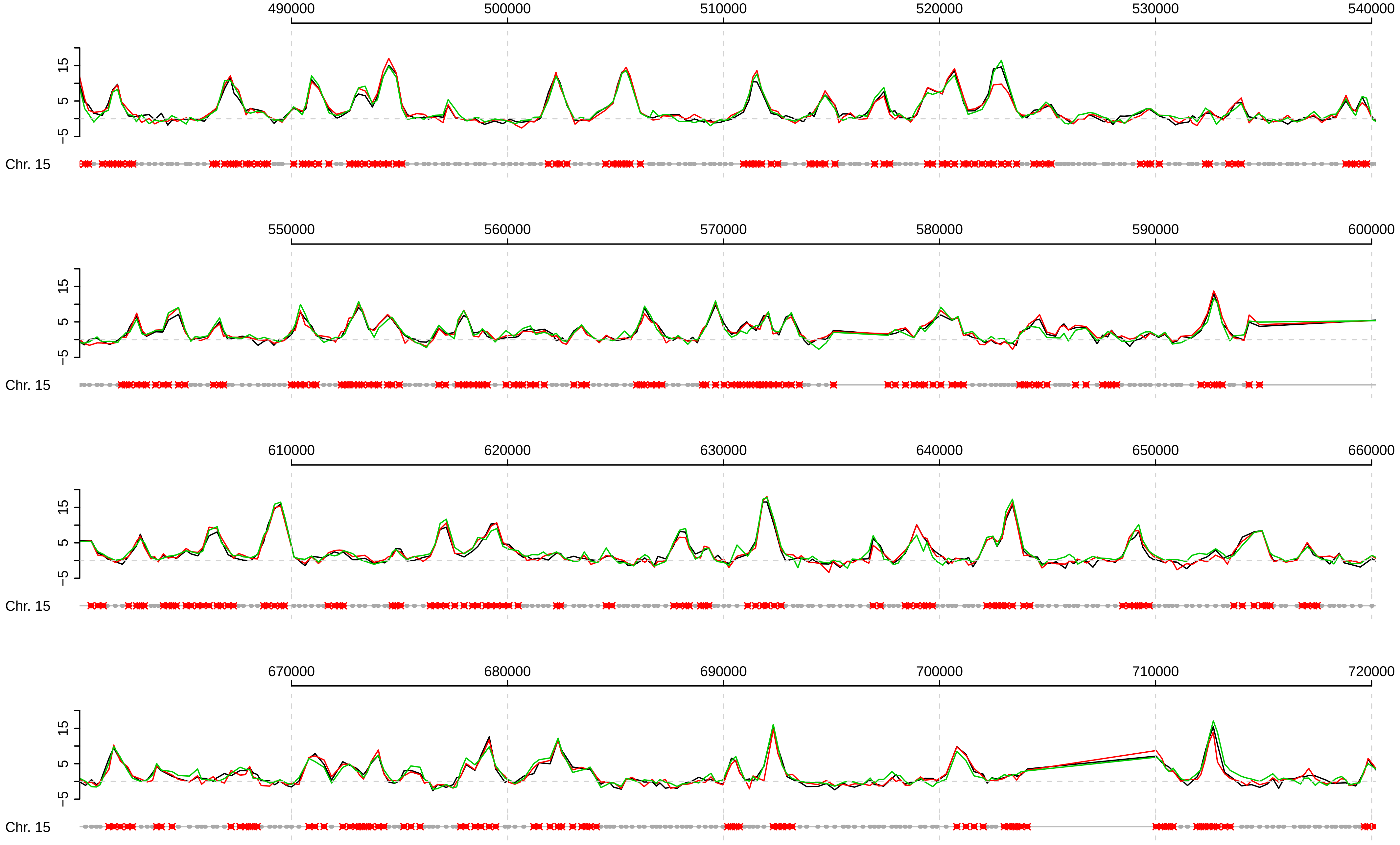

490000
500000
510000
520000
530000
540000
15
5
−5
Chr. 15
550000
560000
570000
580000
590000
600000
15
5
−5
Chr. 15
610000
620000
630000
640000
650000
660000
15
5
−5
Chr. 15
670000
680000
690000
700000
710000
720000
15
5
−5
Chr. 15

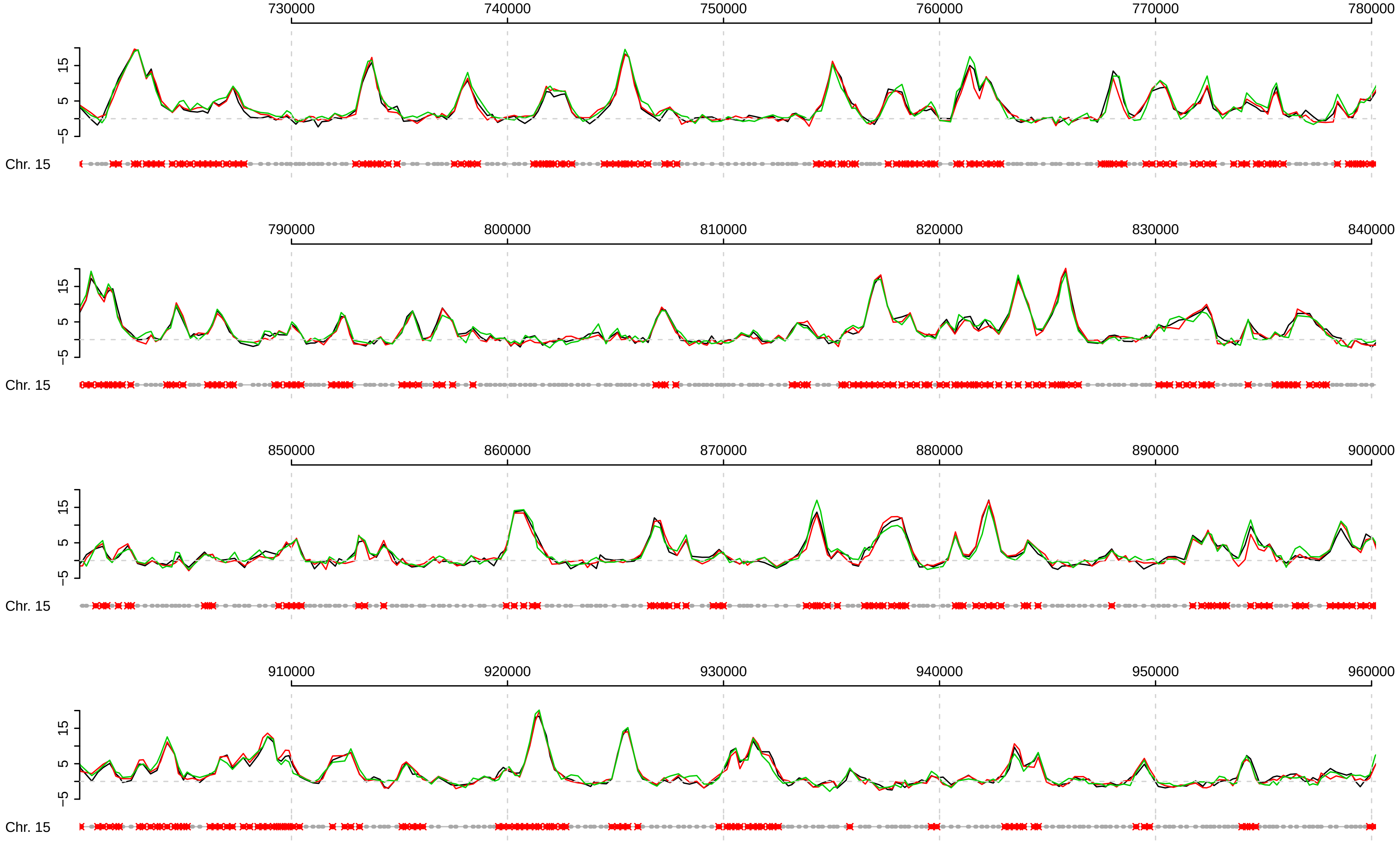
730000
740000
750000
760000
770000
780000
15
5
−5
Chr. 15
790000
800000
810000
820000
830000
840000
15
5
−5
Chr. 15
850000
860000
870000
880000
890000
900000
15
5
−5
Chr. 15
910000
920000
930000
940000
950000
960000
15
5
−5
Chr. 15

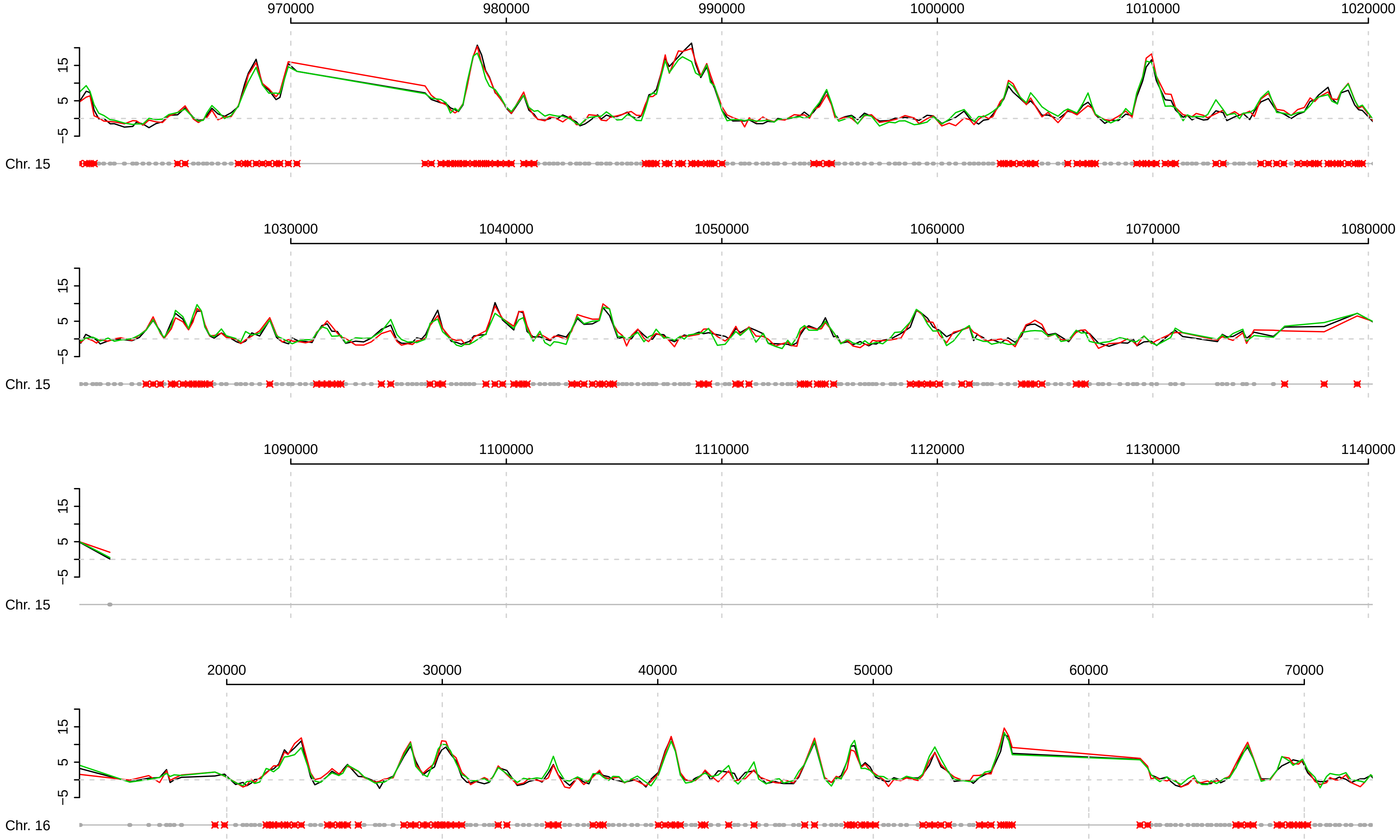

970000
980000
990000
1000000
1010000
1020000
15
5
−5
Chr. 15
1030000
1040000
1050000
1060000
1070000
1080000
15
5
−5
Chr. 15
1090000
1100000
1110000
1120000
1130000
1140000
15
5
−5
Chr. 15
20000
30000
40000
50000
60000
70000
15
5
−5
Chr. 16

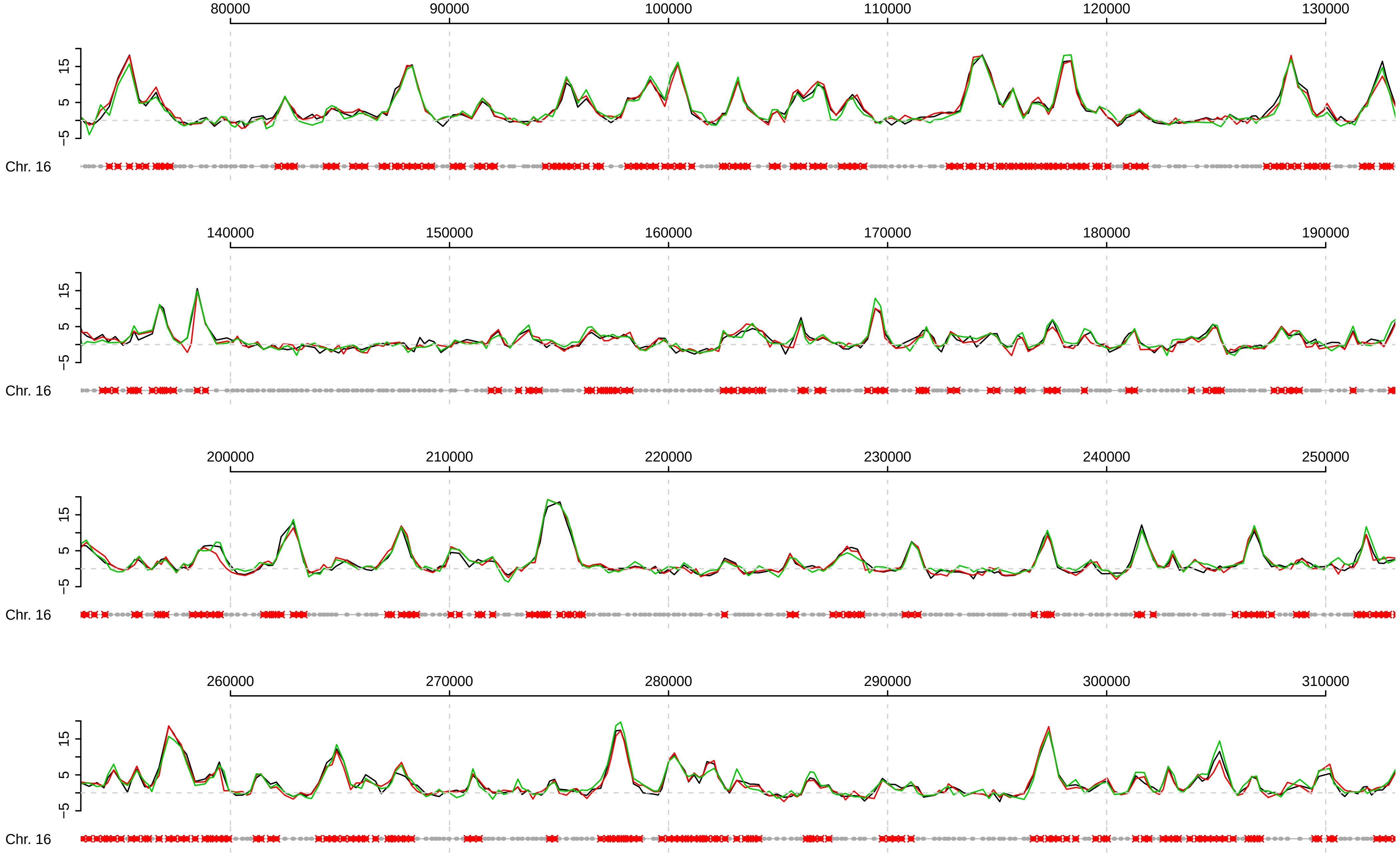

80000
90000
100000
110000
120000
130000
15
5
−5
Chr. 16
140000
150000
160000
170000
180000
190000
15
5
−5
Chr. 16
200000
210000
220000
230000
240000
250000
15
5
−5
Chr. 16
260000
270000
280000
290000
300000
310000
15
5
−5
Chr. 16

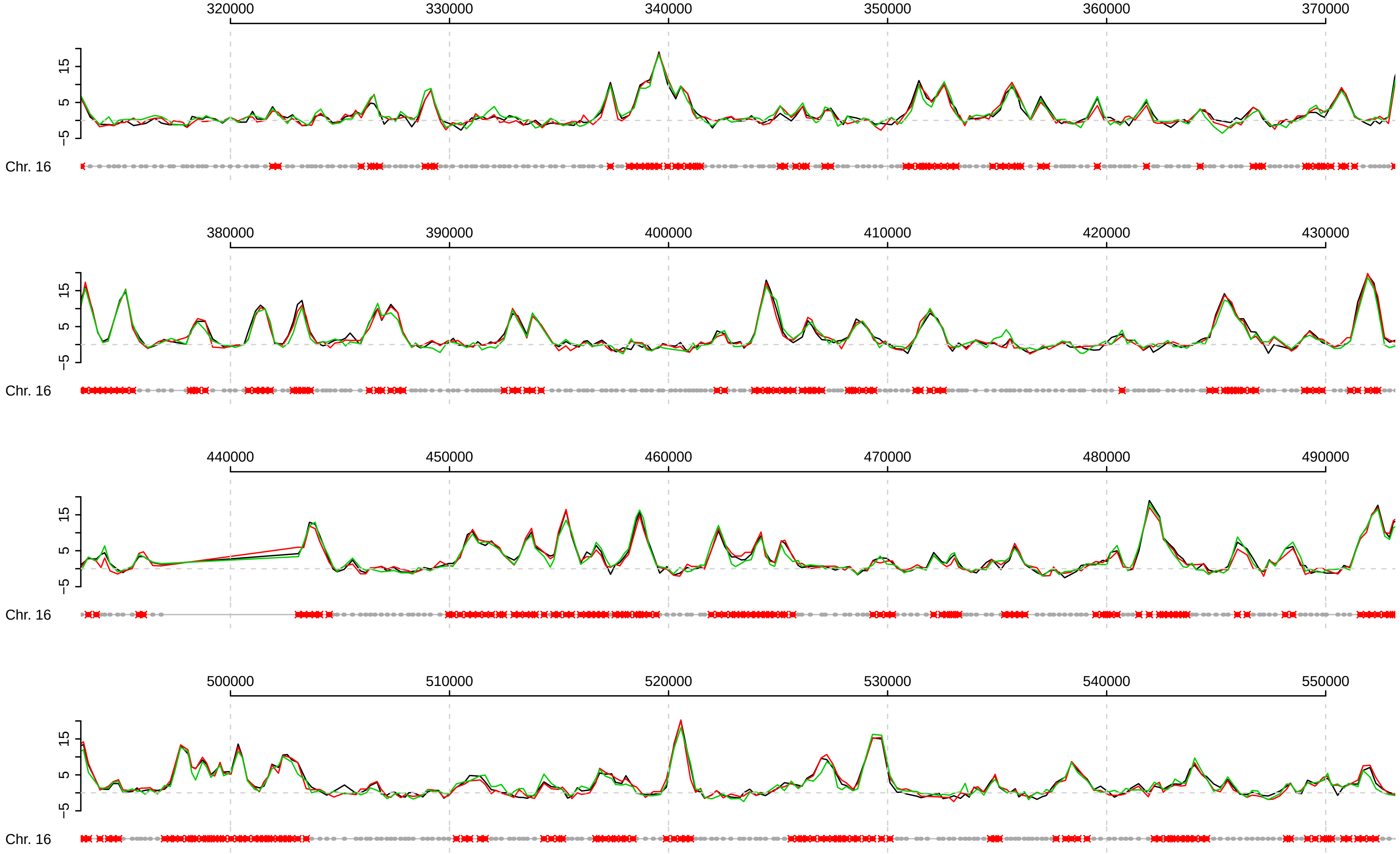
320000
330000
340000
350000
360000
370000
15
5
−5
Chr. 16
380000
390000
400000
410000
420000
430000
15
5
−5
Chr. 16
440000
450000
460000
470000
480000
490000
15
5
−5
Chr. 16
500000
510000
520000
530000
540000
550000
15
5
−5
Chr. 16

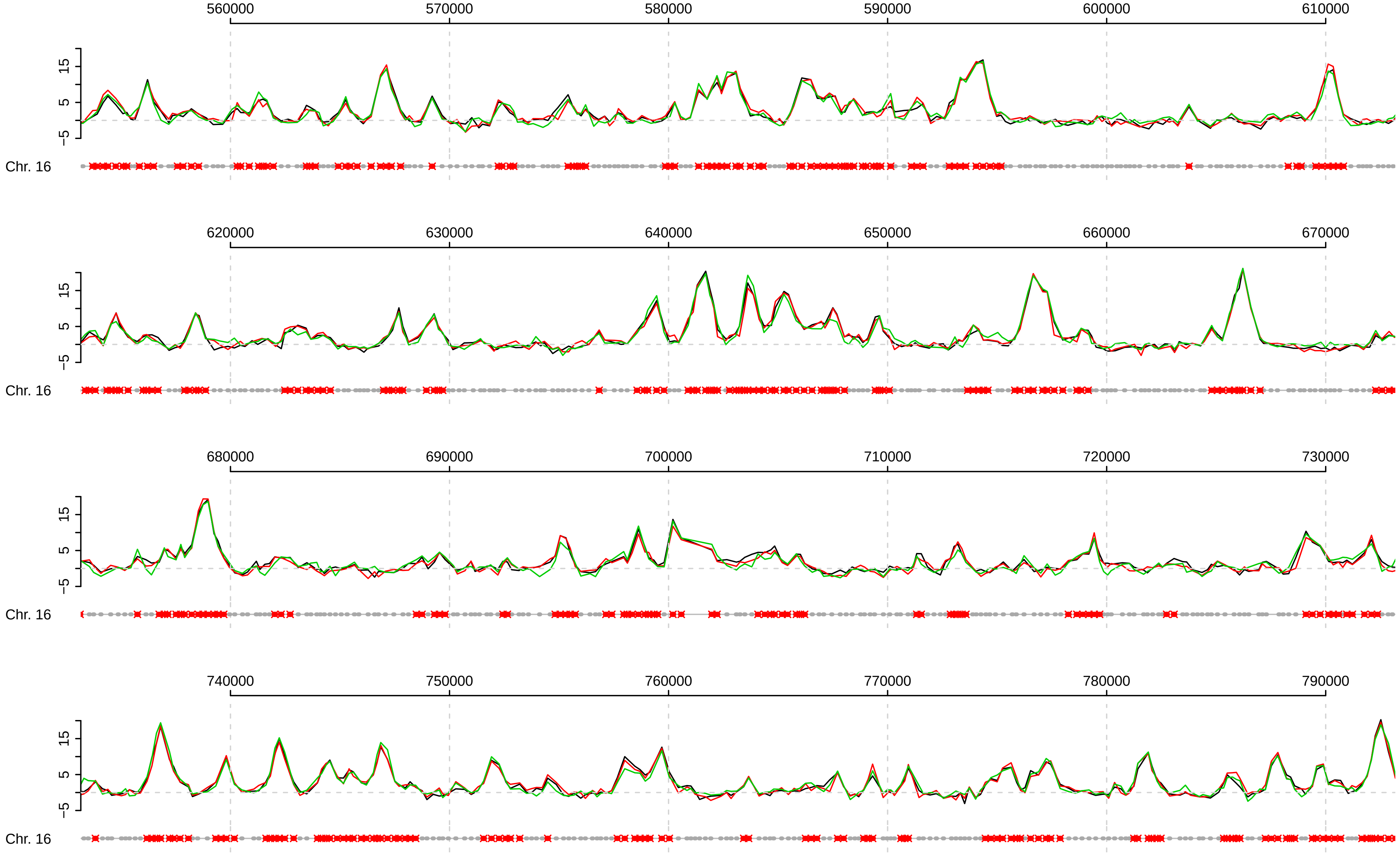

560000
570000
580000
590000
600000
610000
15
5
−5
Chr. 16
620000
630000
640000
650000
660000
670000
15
5
−5
Chr. 16
680000
690000
700000
710000
720000
730000
15
5
−5
Chr. 16
740000
750000
760000
770000
780000
790000
15
5
−5
Chr. 16

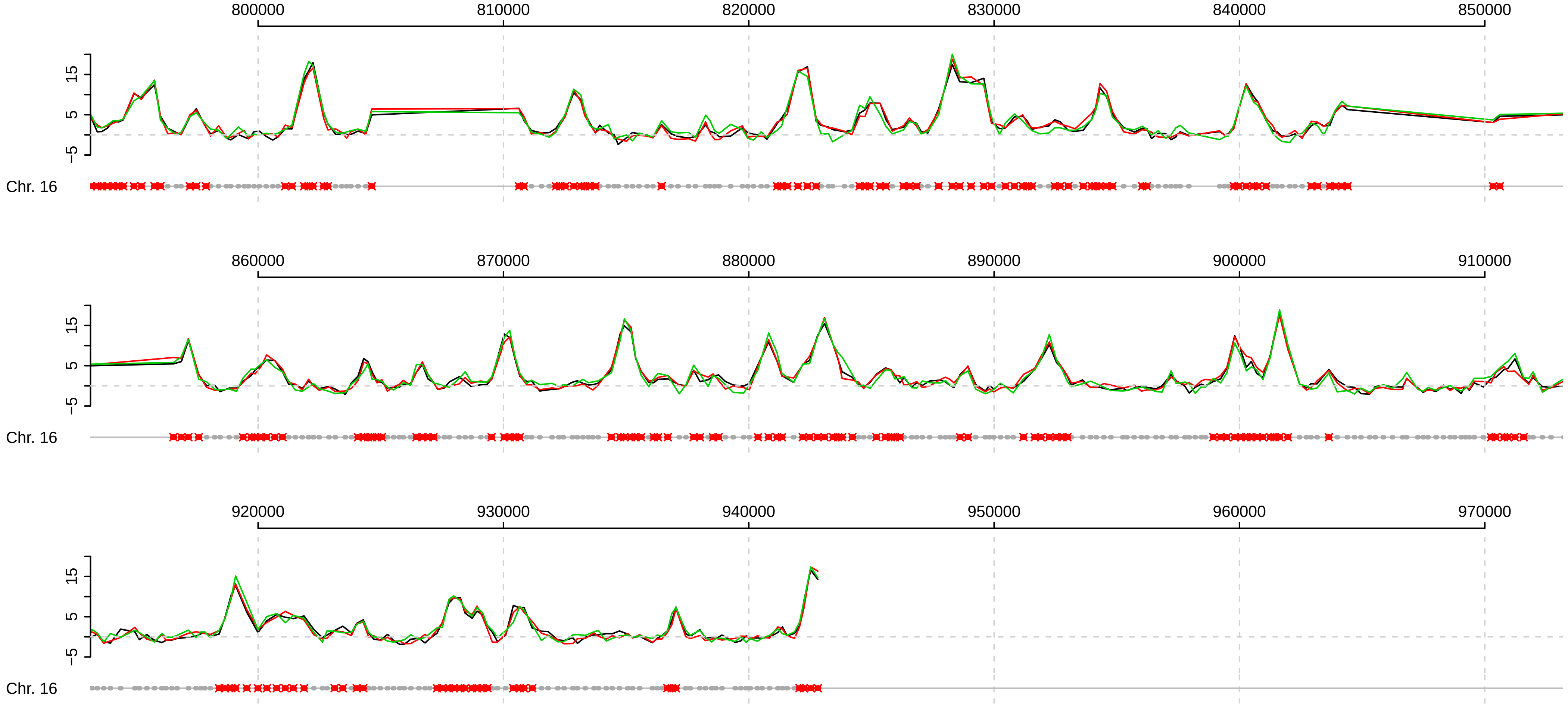

800000
810000
820000
830000
840000
850000
15
5
−5
Chr. 16
860000
870000
880000
890000
900000
910000
15
5
−5
Chr. 16
920000
930000
940000
950000
960000
970000
15
5
−5
Chr. 16
